# First Evidence for Ozonido‐TMC Complexes of Iron and Cobalt

**DOI:** 10.1002/anie.4716492

**Published:** 2026-07-09

**Authors:** Stefan Schaub, Dennis Gerbig, Raffael C. Wende, Heike Hausmann, Jonathan Becker, Christian Würtele, Laura Senft, Ina Kellner, Gunasekaran Velmurugan, Marion Kerscher, Ivana Ivanović‐Burmazović, Peter Comba, Joshua Telser, Peter R. Schreiner, Siegfried Schindler

**Affiliations:** ^1^ Institute of Inorganic Chemistry Justus Liebig University Giessen Germany; ^2^ Institute of Organic Chemistry Justus Liebig University Giessen Germany; ^3^ Department of Chemistry Ludwig‐Maximilians University München Germany; ^4^ Institute of Inorganic Chemistry Heidelberg University Heidelberg Germany; ^5^ Department of Chemistry National Institute of Technology Tiruchirappalli Tamil Nadu India; ^6^ Department of Biological Physical, and Health Sciences Roosevelt University Chicago Illinois USA

**Keywords:** cobalt complexes, iron complexes, oxido complexes, ozone, ozonido complexes, tetramethylcyclam

## Abstract

We present compelling evidence for the first Fe‐ and Co‐ozonido complexes: [(D_12_‐TMC)Fe^III^(O_3_)(OTf)](B_Ar_F), [(D_12_‐TMC)Fe^III^(O_3_)(I)](B_Ar_F), and {[(D_12_‐TMC)Co^III^(F)]_2_(O_3_)}(B_Ar_F)_2_ from the reactions of complexes [(D_12_‐TMC)Fe^II^(*X*)]B_Ar_F and [(D_12_‐TMC)Co^II^(*X*)]B_Ar_F (*X*  =  OTf, F, I) with ozone in 2‐Me‐THF/THF solutions using ultraviolet–visible spectroscopic and cryospray mass spectrometric techniques at cryogenic temperatures as low as −155°C (118 K). Further reactivity studies suggest the subsequent formation of Fe‐ and Co‐oxido complexes [(D_12_‐TMC)Fe^IV^(O)(OTf)]B_Ar_F, [(D_12_‐TMC)Co^IV^(O)(OTf)]B_Ar_F, and [(D_12_‐TMC)Co^IV^(O)(F)]B_Ar_F. Density functional theory computations with the *ω*B97X‐3c three‐component method and the *ω*B97X‐D4 hybrid functional corroborate our spectroscopic results. Further key results of our studies entail the synthesis of several hitherto unreported Fe and Co complexes, including complexes with strongly (F) and moderately stabilizing (OTf) anions without extraneous coordinated solvent molecules. The D_12_‐TMC ligand proves to be effective for preventing methyl group hydroxylation as observed, for example, in the case of [(TMCO)Co^III^(H_2_O)](OTf)_2_. With ozonide [N(CH_3_)_4_]O_3_, we instead obtained crystals of [(D_12_‐TMC‐propyl‐O)Co^III^(OTf)]B_Ar_F from [(D_12_‐TMC)Co^II^(OTf)]B_Ar_F. The generation of these transient Fe‐ and Co‐complexes demonstrates that temperatures below values attainable by typical stopped‐flow setups in conjunction with “ligand hardening” provide a promising strategy for studies of reactive complexes.

## Graphical abstract

Reaction of ozone with iron(II) complexes [(TMC)Fe(OTf)]B_Ar_F, [(D_12_‐TMC)Fe(OTf)]B_Ar_F, and cobalt(II) complexes [(TMC)Co(OTf)]B_Ar_F, [(D_12_‐TMC)Co(*X*)]B_Ar_F (*X*  =  OTf, F) gives ozonido and oxido complexes at temperatures as low as –155°C in a 4:1 mixture of 2‐Me‐THF and THF.

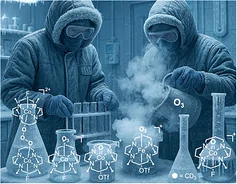

## 1 Introduction

**SCHEME 1:**
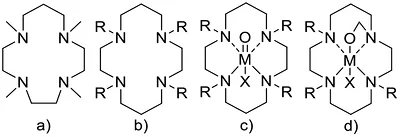
Macrocyclic ligands: (a) 13‐TMC; (b) TMC (= 14‐TMC, R = CH_3_; D_12_‐TMC, R = CD_3_; (CF_3_)_4_‐cyclam, R = CF_3_) (c) metal oxido complex [(TMC)*M* ^IV^(O)*X*]^n+^ (*M* = metal cation, *X*  =  ligand, for example, OTf, CH_3_CN, or H_2_O); (d) methyl hydroxylated ligand TMCO and its metal complex. For (c, d), charges are omitted. TMC is used throughout the manuscript as the abbreviation for 14‐TMC; different ring sizes are indicated as *x*‐TMC (*x*  =  13, 15).

Metal oxido complexes are important in nature as well as in the lab and industry for a plethora of oxygenation reactions. For example, cytochrome P450 reacts with dioxygen to form an iron(IV) oxido‐porphyrin radical complex (P450 Compound I) in the enzyme active site, causing the catalytic oxidation of organic substrates under ambient conditions [1, 2]. In contrast, practical oxygenation reactions often have to be performed at high temperatures and/or under high pressures while often using expensive and/or toxic catalysts. For example, despite their toxicity, chromium(VI) oxido complexes are still used to a large extent for oxygenation reactions [3, 4]. However, non‐heme high valent iron oxido complexes have provided powerful alternatives [5]. In contrast, due to the so‐called *oxo wall* [6, 7, 8, 9, 10], high valent oxido complexes of cobalt, nickel, or copper [11], while viable labile intermediates, have rarely been reported. This changed only recently when several high valent cobalt(IV) oxido complexes with non‐heme ligands were reported [12, 13, 14, 15, 16, 17, 18, 19, 20]. For our work presented herein, the spectroscopic evidence for a cobalt(IV) oxido complex, [(13‐TMC)Co^IV^(O)](OTf)_2_ (13‐TMC  =  1,4,7,10‐tetramethyl‐1,4,7,10‐tetraazacyclotridecane; OTf  =  triflate) by Nam and coworkers, is especially relevant (Scheme 1) [21, 22].

Due to our previous experience with ozone as a clean oxidant for the formation of the iron(IV) oxido complex [(TMC)Fe^IV^(O)(OTf)]OTf (**1a′‐OTf**; TMC  =  1,4,8,11‐tetramethyl‐1,4,8,11‐tetraazacyclotetradecane) from [(TMC)Fe^II^(OTf)]OTf (**1a‐OTf**), we anticipated the same procedure to be suitable for the preparation of the corresponding cobalt complex [23]. Modifying our experimental procedures by using lower temperatures and employing specifically altered precursor complexes, we envisioned the detection of iron‐ and cobalt‐ozonido complexes might even be feasible, which indeed we report in the following.

## 2 Results and Discussion

In the following, **a** and **b** denote protium and deuterium complex isotopologs, respectively. Prime ( **′** ) and double prime ( **″** ) denote oxido‐ and ozonido‐ complexes, respectively.

### 2.1 Iron

As described in the introduction, we previously synthesized the oxido complex **1a′‐OTf** by reacting the Fe(II) complex **1a‐OTf** with ozone in dichloromethane (DCM) [23]. In an effort to detect the most likely formed intermediate, the ozonido complex [(TMC)Fe^III^(O_3_)(OTf)]OTf (**1a″‐OTf**), it was necessary to change the solvent to a 2‐Me‐THF/THF (4:1) (THF  = tetrahydrofuran) mixture that allows investigations as low as −155°C in a custom low‐temperature setup (Scheme S‐I‐1,2). However, due to the low solubility of **1a‐OTf** under these conditions, it was required to change from one of the triflate anions to the B_Ar_F anion, tetrakis[3,5‐bis(trifluoromethyl)phenyl]borate, to yield [(TMC)Fe^II^(OTf)]B_Ar_F, **1a‐B_Ar_F** (Figure S‐II‐1). B_Ar_F was chosen because it is inert toward oxidation by ozone in contrast to tetraphenylborate. Furthermore, complexes with B_Ar_F are much more soluble at low temperatures.

To increase the lifetime of a potential ozonido intermediate, we opted to conduct the majority of our studies with complex [(D_12_‐TMC)Fe^II^(OTf)]B_Ar_F (**1b‐B_Ar_F**; Figure S‐II‐2) derived from [(D_12_‐TMC)Fe^II^(I)]B_Ar_F (**2b‐B_Ar_F**; Figure S‐II‐4) as well as with [(D_12_‐TMC)Fe^II^(F)]B_Ar_F (**3b‐B_Ar_F;** Figure S‐II‐5).

### 2.2 UV–vis Measurements

**FIGURE 1:**
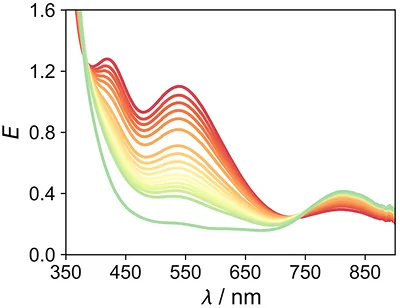
UV–vis spectra showing the decay of the signal obtained upon reaction of [(D_12_‐TMC)Fe^III^ (OTf)]B_Ar_F (**1b‐B_Ar_F**; 3 ×10^−3^ mol L^−1^ in 2‐Me‐THF/THF) and ozone at –130°C. The colors of the UV–vis trace reflect the color of the solution during the course of the reaction (from red to green). Baseline (green) after subsequent irradiation at 617 nm.

To elucidate the reactivity of the iron complexes with ozone, we conducted cryo‐UV–vis experiments in 2‐Me‐THF/THF solutions at temperatures as low as –155°C. None of the iron complexes discussed herein showed any reactivity toward dioxygen under the described conditions. Upon addition of a cooled solution of **1b‐B_Ar_F** to an ozonized sample of 2‐Me‐THF/THF at –155°C, followed by warming to –135°C, a crimson red colored solution formed over the course of several minutes. The color change was accompanied by the appearance of two prominent absorptions at 422 and 539 nm (Figure 1). The observed reactivity in conjunction with computed electronic transitions using time‐dependent density functional theory (TD‐DFT) indicates the formation of the iron‐ozonido complex [(D_12_‐TMC)Fe^III^(OTf)(O_3_)]B_Ar_F (**1b″‐B_Ar_F**): We computed values of 411, 422, and 623 nm for the overall high‐spin *S*  =  3 ground state, resulting from ferromagnetic coupling of d^5^ Fe^III^ with *S*_Fe_ = 5/2 and the ozonide radical anion with *S*_O_ = 1/2.

$$\begin{array}{cc} & \left[\left(D_{12}-\mathrm{TMC}\right){\mathrm{Fe}}^{\mathrm{II}}\left(\mathrm{OTf}\right)\right]B_{\mathrm{Ar}}F+O_{3}\\ & \;\to \left[\left(D_{12}-\mathrm{TMC}\right){\mathrm{Fe}}^{\mathrm{III}}\left(O_{3}\right)\left(\mathrm{OTf}\right)\right]B_{\mathrm{Ar}}F \end{array}$$

When increasing the temperature by only 5°C to –130°C, the solution turned amber and eventually green, which was reflected in the spectrum: The bands at 422 and 539 nm decay along with the formation of the expected band of an iron‐oxido species centered around 830 nm, here [(D_12_‐TMC)Fe^IV^(O)(OTf)]B_Ar_F (**1b′‐B_Ar_F**; Figure 1). This can be understood in light of the small reaction barrier of **1b″‐B_Ar_F** toward extrusion of dioxygen (as little as Δ*G* ^‡^  =  5 kcal mol^−1^ in the computed overall *S*  =  3 septet ground state; Scheme S‐VI‐1).

$$\begin{array}{cc} & \left[\left(D_{12}-\mathrm{TMC}\right){\mathrm{Fe}}^{\mathrm{III}}\left(O_{3}\right)\left(\mathrm{OTf}\right)\right]B_{\mathrm{Ar}}F\\ & \;\to \left[\left(D_{12}-\mathrm{TMC}\right){\mathrm{Fe}}^{\mathrm{IV}}\left(O\right)\left(\mathrm{OTf}\right)\right]B_{\mathrm{Ar}}F+O_{2} \end{array}$$

Strikingly, when a solution of **1b‐B_Ar_F** was ozonized directly for 5 s at –155°C, an amber mixture of **1b′‐B_Ar_F** and **1b″‐B_Ar_F** was obtained immediately. This again demonstrates the low thermal stability of **1b″‐B_Ar_F**: The warm gas stream already effects oxygen extrusion to a large degree.

We further tested the reactivity of potential **1b″‐B_Ar_F** toward excess amounts of water, *p*‐toluenesulfonic acid (*p*TSA), diisopropylethyl amine (DiPEA), and hydridic reducing agent 2,2,6,6‐tetramethyl‐piperidin‐1‐ol (i.e., TEMPOH) at –135°C. To our surprise, no spectral changes were observed under all these conditions. This also rules out the viability of a similarly absorbing hydroperoxido species [24] as an alternative explanation for the observed absorptions.

To solidify the experimental evidence for an iron ozonido complex, we set out to investigate the effect on reactivity of swapping the triflato for a fluorido ligand via the reaction of **2b‐B_Ar_F** with AgF to yield **3b‐B_Ar_F**. However, upon injection of a cooled **3b‐B_Ar_F** solution into an ozonized sample of 2‐Me‐THF/THF at –155°C, the quartz cuvette burst approximately 5 s after mixing due to a sudden gas release. This difference in reactivity is most probably due to the strong coordination of the fluorido ligand: The increased Gibbs free energy of reaction associated with the likely formation of [(D_12_‐TMC)Fe^III^(O_3_)(F)]B_Ar_F (**3b″‐B_Ar_F**) compared to **1b″‐B_Ar_F** probably turns out to be its undoing under our experimental conditions. Given the confines of the quartz cuvette, additional ΔΔ_r_ *G*  =  10 kcal mol^−1^ (Equation S‐VI‐1) cannot be dissipated in time to prevent immediate decomposition by dioxygen extrusion (Δ*G* ^‡^  =  8 kcal mol^−1^, *S*  =  3; Scheme S‐VI‐2).

**FIGURE 2:**
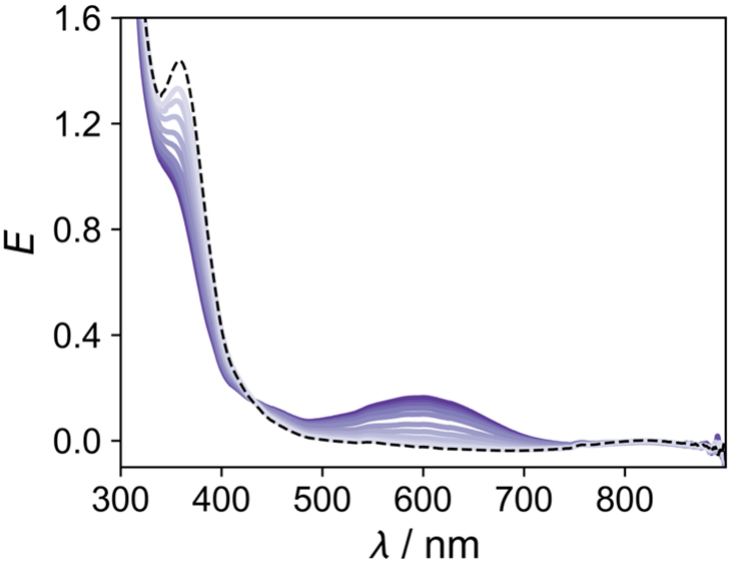
UV–vis spectra showing the decay of the signal obtained upon reaction of [(D_12_‐TMC)Fe^III^(O_3_)(I)]B_Ar_F (**2b″‐B_Ar_F**; 5 × 10^−3^ mol L^−1^ in 2‐Me‐THF/THF; lavender trace) and ozone over the temperature range from –100°C to –98°C.

We salvaged our previous idea by simply employing **2b‐B_Ar_F** instead. After injection of a **2b‐B_Ar_F** solution into the ozonized solvent mixture, we observed no spectral change up to a temperature of –115°C. Upon increasing the temperature further, a broad absorption centered on 597 nm appeared, and the sample turned to a pale cornflower‐blue color. A second, less pronounced signal was visible around 447 nm. Indeed, our computations place the absorption of an ozonido complex with the iodido ligand [(D_12_‐TMC)Fe^III^(O_3_)(I)]B_Ar_F (**2b″‐B_Ar_F**; Figures 2 and S‐III‐1) at 442 and 619 nm for the overall *S*  =  3 septet ground state.

$$\left[\left(D_{12}-\mathrm{TMC}\right){\mathrm{Fe}}^{\mathrm{II}}\left(I\right)\right]B_{\mathrm{Ar}}F+O_{3}\to \left[\left(D_{12}-\mathrm{TMC}\right){\mathrm{Fe}}^{\mathrm{III}}\left(O_{3}\right)\left(I\right)\right]B_{\mathrm{Ar}}F$$

The signals rapidly vanished when surpassing –100°C, with a concurrent increase of a pronounced feature at 359 nm and the sample turning bright yellow. At the same time, no pronounced absorption >800 nm was detected. The signal at 359 nm matches well the reported value of 363 nm for the hypoiodite anion, IO^− ^ [25], which is likely to be present as a hypoiodito‐*O* ligand after oxidation of the iodido ligand by a second equivalent of **2b″‐B_Ar_F**.

$$\left[\left(D_{12}-\mathrm{TMC}\right){\mathrm{Fe}}^{\mathrm{III}}\left(O_{3}\right)\left(I\right)\right]B_{\mathrm{Ar}}F+\left[\left(D_{12}-\mathrm{TMC}\right){\mathrm{Fe}}^{\mathrm{II}}\left(I\right)\right]B_{\mathrm{Ar}}F\to$$

$$\left[\left(D_{12}-\mathrm{TMC}\right){\mathrm{Fe}}^{\mathrm{II}}\left(I\right)\right]B_{\mathrm{Ar}}F+O_{2}+\left[\left(D_{12}-\mathrm{TMC}\right){\mathrm{Fe}}^{\mathrm{II}}\left(\mathrm{IO}\right)\right]B_{\mathrm{Ar}}F$$

During the course of our experiments, we furthermore synthesized and characterized related complexes [(D_12_‐TMC)Fe^IV^(O)(OTf)]OTf, **1b′‐OTf** (Figure S‐II‐3), and [(D_12_‐TMC)Fe^IV^(O)(H_2_O)]{N(OTf)_2_}B_Ar_F (Figure S‐II‐16) with the bis(trifluoromethane)sulfonimide anion.

### 2.3 Cryospray Ionization Mass Spectrometry

To gain further insight into the reaction of **1b‐B_Ar_F** with ozone (for **1a‐B_Ar_F**, see Supporting Information part IV), the product solution was analyzed by cryospray ionization mass spectrometry (CSI‐MS) or conventional electrospray ionization mass spectrometry (ESI‐MS) under modified conditions (see Supporting Information part IV) in positive ion mode. A summary of all relevant species together with their assignments is shown in Table 1.

**TABLE 1:** Summary of key species detected in the reaction mixture of **1b‐B_Ar_F** with ozone.

*m/z*		Detected ion	Species
Exp.	Calcd.		
340.2671	340.2673	[(D_12_‐TMC)Fe(O)]^+^	Fe^III^=O
369.2696	369.2701	[(D_12_‐TMC)Fe(HCOO)]^+^	Fe^II^
372.2566	372.2572	[(D_12_‐TMC)Fe(O_3_)]+	Fe^II^−O_3_
385.2650	385.2650	[(D_12_‐TMC)Fe(O)(HCOO)]^+^	Fe^IV^=O
489.2196	489.2193	[(D_12_‐TMC)Fe(O)(OTf)]^+^	Fe^IV^=O

We detected an Fe‐oxido complex as [(D_12_‐TMC)Fe^IV^(O)(OTf)]^+^ (*m/z*  489.2196), [(D_12_‐TMC)Fe^IV^(O)(HCOO)]^+^ (*m/z*   385.2650) as well as [(D_12_‐TMC)Fe^III^(O)]^+^ (*m/z*  340.2671) without an accompanying anion. In general, we observed that certain complex cations are able to capture an electron during the CSI process, thus providing the respective complex cations in a lower oxidation state [26, 27]. As such, an Fe‐ozonido complex ion was found as [(D_12_‐TMC)Fe^II^(O_3_)]^+^ (*m/z*  372.2566), which was then investigated in MS/MS mode. There, we observed the exchange of the ozonido for a formato ligand to give [(D_12_‐TMC)Fe^II^(HCOO)]^+^ (*m/z*  369.2696). As a consecutive decay product of THF oxidation [28, 29, 30, 31], formate is abundant in the reaction mixture and is likely present during measurements as formic acid under the conditions of MS/MS.

$$\begin{array}{cc} & {\left[\left(D_{12}-\mathrm{TMC}\right){\mathrm{Fe}}^{\mathrm{III}}\left(O_{3}\right)\left(\mathrm{OTf}\right)\right]}^{+}+H^{+}+e^{-}\\ & \;\overset{\mathrm{ESI}-\mathrm{MS}}{\to }{\left[\left(D_{12}-\mathrm{TMC}\right){\mathrm{Fe}}^{\mathrm{II}}\left(O_{3}\right)\right]}^{+}+\mathrm{HOTf} \end{array}$$

$$\begin{array}{cc} & {\left[\left(D_{12}-\mathrm{TMC}\right){\mathrm{Fe}}^{\mathrm{II}}\left(O_{3}\right)\right]}^{+}+\mathrm{HCOOH}\\ & \;\overset{\mathrm{MS}/\mathrm{MS}}{\to }{\left[\left(D_{12}-\mathrm{TMC}\right){\mathrm{Fe}}^{\mathrm{II}}\left(\mathrm{HCOO}\right)\right]}^{+}+\mathrm{HO}•+O_{2} \end{array}$$

Product ions of other oxygen‐containing intermediates, as well as such‐involving coordination by solvent‐derived ligands, are given in Supporting Information part IV.

We also recorded low‐temperature electron paramagnetic resonance (EPR) spectra of the reaction of **1a‐B_Ar_F** and **1b‐B_Ar_F** with ozone. While ozonization yielded strong signals typical for high‐spin Fe(III) species [32], we could not assign these to **1a″‐B_Ar_F** and **1b″‐B_Ar_F** exclusively. Fe(IV) species with (*S*  =  1), such as **1a′‐B_Ar_F** and **1b′‐B_Ar_F** formed here, are generally EPR‐silent using conventional spectrometers. Our EPR experiments are described in detail in Supporting Information part V.

### 2.4 Cobalt

Obtaining [(TMC)Co^II^(OTf)]OTf in the same way as the analogous iron complex **1a‐OTf** turned out to be extremely difficult. We successfully prepared [(TMC)Co^II^(CH_3_CN)](OTf)_2_, **4a‐(OTf)_2_**, which was structurally characterized (Figure S‐II‐6) and shows a similar molecular structure as the respective complex with perchlorate reported previously [33]. Furthermore, we crystallized [(TMC)Co^II^(CH_3_CN)](B_Ar_F)_2_, **4a‐(B_Ar_F)_2_** (Figure S‐II‐7). Contrary to the preparation of iron complex **1a‐OTf**, removal of MeCN by recrystallization of **4a‐(OTf)_2_** from DCM (azeotrope formation of MeCN and DCM) was unsuccessful [23].

**FIGURE 3:**
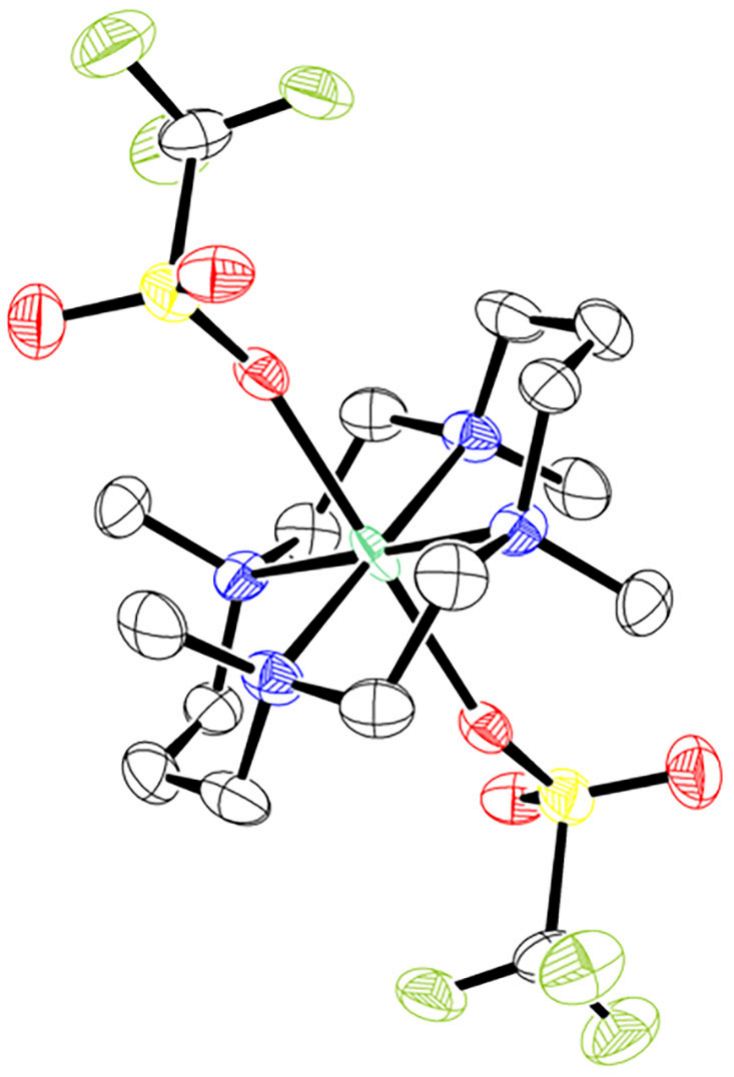
Molecular structure of [(TMC)Co^II^(OTf)_2_] (**5a**; color code: C, white; N, blue; O, red; S, yellow; Co, bright green; F, green; H omitted for clarity).

As it turned out, the synthesis of the acetonitrile‐free Co(II) complex requires pure Co(OTf)_2_ as the starting material. Although this compound has been reported, the published procedure did not lead to the product in the desired purity [34, 35]. However, a modified synthesis allowed us to obtain pure Co(OTf)_2_ in good yields (see Supporting Information part I). Applying powder diffraction analysis, we characterized the structure as an octahedral Co(II) complex, isostructural with the triflate salts of calcium, magnesium, and zinc (Figure S‐II‐26,27) [36]. With Co(OTf)_2_ in hand, we eventually succeeded in the synthesis of solvent‐free [(TMC)Co^II^(OTf)_2_] (**5a**) (Figures 3 and S‐II‐8), the molecular structure of which notably features a center of inversion. Unfortunately, **5a** is barely soluble in DCM at low temperatures. Complex [(D_12_‐TMC)Co^II^(OTf)_2_] (**5b**) (Figure S‐II‐10) also showed insufficient solubility in DCM at low temperatures [23]. Thus, we used [(D_12_‐TMC)Co^II^(OTf)]B_Ar_F (**5b‐B_Ar_F**; Figure S‐II‐11) for reactions with ozone, which has its triflato ligand situated *syn* to the methyl groups of the TMC ligand.

It is worthwhile comparing the structure of **5a** to the corresponding iron complex **1a‐OTf**. In **5a**, both triflato ligands coordinate with a Co─O distance of 2.3 Å, with Co(II) positioned in the ligand plane. Conversely, in **1a‐OTf**, only one triflato ligand coordinates with an Fe─O bond distance of 2.0 Å and iron(II) positioned slightly above the plane [8]. Furthermore, only one triflato ligand, positioned slightly above the *N*‐ligand plane, coordinates Co(II) in [(13‐TMC)Co^II^(OTf)](OTf) with a bond distance of 2.0 Å. The latter structural arrangement is akin to that of **1a‐OTf**, but different from that of **5a** [21].

**FIGURE 4:**
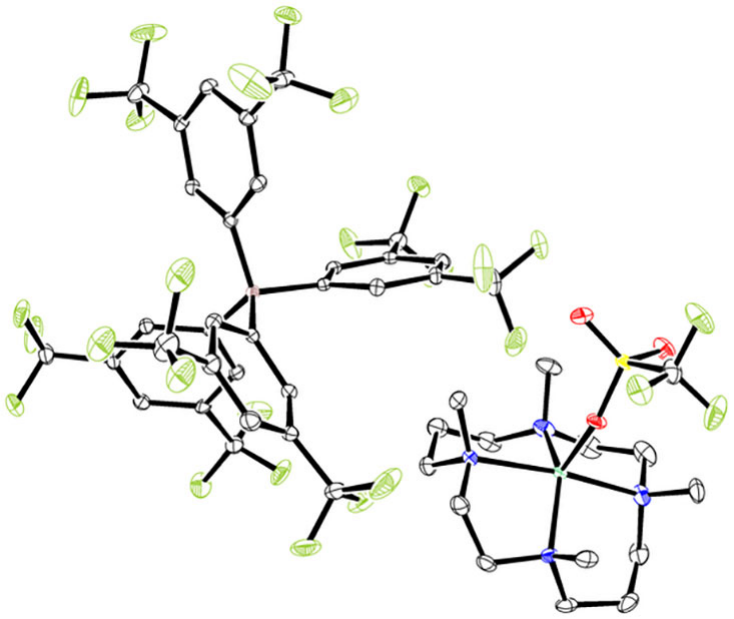
Molecular structure of [(TMC)Co^II^(OTf)]B_Ar_F (**5a‐B_Ar_F**; color code: C, white; N, blue; O, red; S, yellow; Co, bright green; F, green; B, purple; H omitted for clarity).

Obtaining Co(II)‐TMC complexes without additional ligands attached to the metal center, be it from coordinating anions, solvent, or other sources, is generally problematic. In addition to the B_Ar_F anion, we purposely chose triflate or perchlorate as inert, but supposedly weakly coordinating anions. Using pure silver salts AgB_Ar_F (as its stoichiometric ·MeCN ·THF complex salt), AgOTf, and AgClO_4_ in a procedure described in more detail in Supporting Information part I, we obtained the complexes [(TMC)Co^II^(OTf)]B_Ar_F (**5a‐B_Ar_F**; Figures 4 and S‐II‐9) and [(TMC)Co^II^(ClO_4_)]B_Ar_F (**6a‐B_Ar_F**; Figure S‐II‐12). Contrary to complex **5a** discussed above, **6a‐B_Ar_F** is structurally similar to both iron complex **1a‐B_Ar_F** and [(13‐TMC)Co^II^(OTf)](OTf): Only one triflato ligand, positioned slightly above the *N*‐ligand plane, coordinates to the metal center with a Co─O bond distance of 2.0 Å [21].

As an alternative to the B_Ar_F anion, we also used [Al{OC(CF_3_)_3_}_4_]^−^, leading to [(TMC)Co^II^(OTf)][Al{OC(CF_3_)_3_}_4_]. However, we obtained this compound only by crystal picking (Figure S‐II‐17), making it unsuitable for UV–vis spectrometric investigations.

During the syntheses of **5a‐B_Ar_F** and **5b‐B_Ar_F**, we obtained and characterized several precursor complexes: [(D_12_‐TMC)Co^II^(I)]I (Figure S‐II‐21), [(TMC)Co^II^(I)]B_Ar_F (Figure S‐II‐20), as well as [(D_12_‐TMC)Co^II^(I)]B_Ar_F (Figure S‐II‐22). When we attempted to accelerate the reaction of CoI_2_ with D_12_‐TMC by heating, we observed homolytic cleavage of the D_12_‐TMC ligand in the ethylene moiety. The molecular structure of the product is reported in the Supporting Information (Figure S‐II‐25).

### 2.5 UV–vis Measurements

**FIGURE 5:**
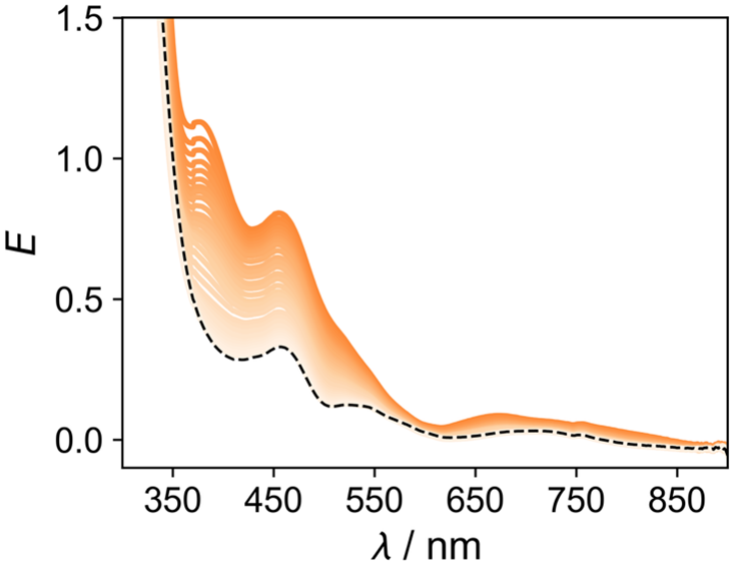
UV–vis spectra showing the decay of the signal obtained upon reaction of [(D_12_‐TMC)Co^II^(OTf)]B_Ar_F (**5b‐B_Ar_F**, 8 × 10^−4^ mol L^−1^ in 2‐Me‐THF/THF) and ozone over the temperature range from –65 to –50°C.

Analogous to the iron complexes, we also subjected the Co congeners to ozonization at low temperatures, none of which showed any reactivity toward dioxygen under the described conditions. Upon injection of **5b‐B_Ar_F** into an ozonized 2‐Me‐THF/THF solution at –150°C, no reaction was observed during warming to room temperature over the course of 1 h, and the pale pink sample was recovered. However, direct and repeated ozonization at –90°C afforded an orange solution with multiple partially overlapping absorptions appearing at 380, 459 (tailing to >550), and 660 nm (Figures 5 and S‐III‐2). These results agree favorably with the computed TD‐DFT values (369, 420, 464, 471, 557, 632 nm) of *S*  =  3/2 quartet Co‐oxido complex [(D_12_‐TMC)Co^IV^(O)(OTf)]B_Ar_F (**5b′‐B_Ar_F**):

$$\begin{array}{cc} & \left[\left(D_{12}-\mathrm{TMC}\right){\mathrm{Co}}^{\mathrm{II}}\left(\mathrm{OTf}\right)\right]B_{\mathrm{Ar}}F+O_{3}\\ & \;\to \left[\left(D_{12}-\mathrm{TMC}\right){\mathrm{Co}}^{\mathrm{III}}\left(O_{3}\right)\left(\mathrm{OTf}\right)\right]B_{\mathrm{Ar}}F \end{array}$$

$$\begin{array}{cc} & \left[\left(D_{12}-\mathrm{TMC}\right){\mathrm{Co}}^{\mathrm{III}}\left(O_{3}\right)\left(\mathrm{OTf}\right)\right]B_{\mathrm{Ar}}F\\ & \;\to \left[\left(D_{12}-\mathrm{TMC}\right){\mathrm{Co}}^{\mathrm{IV}}\left(O\right)\left(\mathrm{OTf}\right)\right]B_{\mathrm{Ar}}F+O_{2} \end{array}$$

Increasing the temperature resulted in diminishing signal intensity, albeit without the visible appearance of signals due to possible decomposition products. Irradiation with 623 nm light at –150°C also led to signal disappearance. In contrast to **1b″‐B_Ar_F**, the formation of the corresponding Co‐ozonido complex [(D_12_‐TMC)Co^III^(O_3_)(OTf)]B_Ar_F (**5b″‐B_Ar_F**) is apparently disfavored, resulting in the absence of its spontaneous formation in an ozonized cold solution. Still, prolonged ozonization probably generates some **5b″‐B_Ar_F**, which then should immediately extrude dioxygen (Δ*G* ^‡^  =  9 kcal mol^−1^; Scheme S‐VI‐5) under the influence of the warm gas stream to afford **5b′‐B_Ar_F**.

**FIGURE 6:**
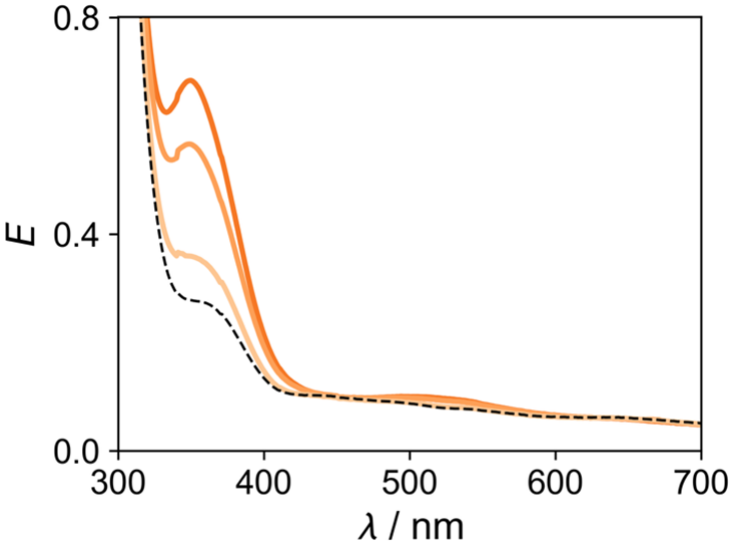
UV–vis spectra showing the decay of the signal obtained upon reaction of [(D_12_‐TMC)Co^II^(F)]B_Ar_F (**7b‐B_Ar_F**, 2 x10^−3^ mol/L in 2‐Me‐THF/THF; orange trace) and ozone over the temperature range of –90 to –50°C.

Similarly to the case of the iron‐ozonido complexes described above, we decided to study the reactivity of [(D_12_‐TMC)Co^II^(F)]B_Ar_F bearing the strongly coordinating fluorido ligand (**7b‐B_Ar_F**; Figure S‐II‐13) toward ozone. Somewhat unexpectedly, injection of **7b‐B_Ar_F** into an ozonized 2‐Me‐THF/THF solution at –150°C and warming to –110°C afforded a spectrum with a single pronounced feature at 352 nm (Figures 6 and S‐III‐3 and S‐III‐4). Upon further increasing the temperature, this signal strongly diminished in intensity but did not vanish entirely. Our computational results suggest that the strong absorption at 352 nm is due to mononuclear complex [(D_12_‐TMC)Co^III^(O_3_)(F)]B_Ar_F (**7b″‐B_Ar_F**), which subsequently forms dinuclear {[(D_12_‐TMC)(F)Co^III^]_2_(O_3_)}(B_Ar_F)_2_ (**di‐7b″‐B_Ar_F**): Indeed, we computed the formation of **di‐7b″‐B_Ar_F** from **7b″‐B_Ar_F** to be exothermic and only slightly endergonic. In turn, the formation of **7b″‐B_Ar_F** is favored over that of **5b″‐B_Ar_F** by ΔΔ_r_ *G*  =  9 kcal mol^−1^ (Equation S‐VI‐8).

(3) $$\begin{array}{cc} & \left[\left(D_{12}-\mathrm{TMC}\right){\mathrm{Co}}^{\mathrm{II}}\left(F\right)\right]B_{\mathrm{Ar}}F+O_{3}\\ & \;\to \left[\left(D_{12}-\mathrm{TMC}\right){\mathrm{Co}}^{\mathrm{III}}\left(O_{3}\right)\left(F\right)\right]B_{\mathrm{Ar}}F \end{array}$$

$$\begin{array}{ccc} & & \left[\left(D_{12}-\mathrm{TMC}\right){\mathrm{Co}}^{\mathrm{III}}\left(O_{3}\right)\left(F\right)\right]B_{\mathrm{Ar}}F+\left[\left(D_{12}-\mathrm{TMC}\right){\mathrm{Co}}^{\mathrm{II}}\left(F\right)\right]B_{\mathrm{Ar}}F\\ & & \to \left\{{\left[\left(D_{12}-\mathrm{TMC}\right)\left(F\right)Co^{\mathrm{III}}\right]}_{2}\left(O_{3}\right)\right\}{\left(B_{\mathrm{Ar}}F\right)}_{2} \end{array}$$

We computed a strong absorption at 358 nm for **7b″‐B_Ar_F** in the *S*  =  1 triplet state, whereas **di‐7b″‐B_Ar_F** shows a much weaker absorption at 352 nm. Hence, **di‐7b″‐B_Ar_F** absorbs similarly to **7b″‐B_Ar_F**, but at lower intensity, which explains the observed evolution of the spectral trace. The diminished absorbance of **di‐7b″‐B_Ar_F** is likely rooted in its low‐spin *S*  =  0 singlet ground state, resulting from antiferromagnetic coupling of two d^6^ Co^III^ with *S*_Co_  =  1 each via a formal O_3_^2–^ dianion. To the best of our knowledge, this would constitute the first example of a complex bearing the perozonido ligand.

**FIGURE 7:**
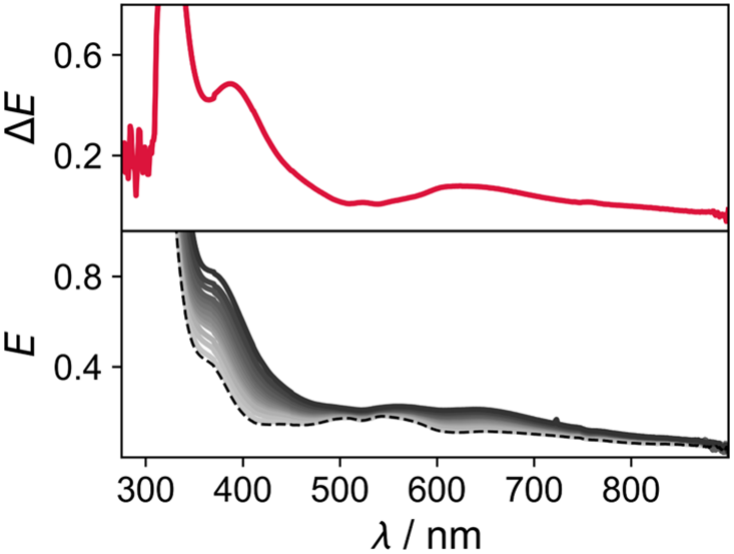
UV–vis spectral evolution over the temperature range from –35°C to 0°C after the reaction of [(D_12_‐TMC)Co^II^(F)]B_Ar_F (**7b‐B_Ar_F**; black trace; difference trace in red; 8 × 10^−4^ M in 2‐Me‐THF/THF) and ozone.

As the solution approached a temperature of –35°C, the spectrum again underwent change: Broad signals developed at 387 and 625 nm and persisted until approximately 15°C (Figures 7 and S‐III‐5).

These favorably match the computed absorptions of [(D_12_‐TMC)Co^IV^(O)(F)]B_Ar_F in the *S*  =  3/2 quartet state (**7b′‐B_Ar_F**; 377, 660 nm). The low‐energy absorption at 625 nm compares favorably to absorptions reported for similar macrocyclic Co‐oxido complexes [21]. We presume that above –35°C, entropy overrides the small preference of **di‐7b″‐B_Ar_F** over **7b″‐B_Ar_F**. However, upon dissociation of **di‐7b″‐B_Ar_F** to **7b″‐B_Ar_F**, the latter should immediately extrude dioxygen and generate **7b′‐B_Ar_F** due to the small reaction barrier of only Δ*G* ^‡^  =  9 [14] kcal mol^−1^ (*S*  =  5/2 [3/2]; Scheme S‐VI‐6).

**FIGURE 8:**
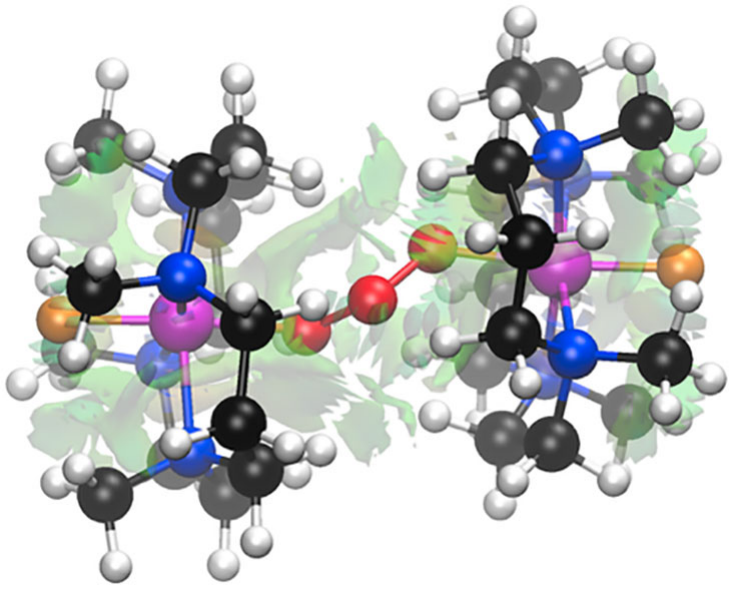
Non‐covalent interactions in dimeric {[(D_12_‐TMC)Co^III^(F)](O_3_)}_2_^2+^ (**7b″‐B_Ar_F**). Green (red) areas depict attractive (repulsive) interactions (plotted with cutoffs *r*_1_  = 0.05 a.u., *r*_2_  =  0.5 a.u. for the density and the reduced density gradient, respectively).

The structural arrangement of **di‐7b″‐B_Ar_F** is mainly held together by strong London dispersion (LD) [37, 38] interactions: While computed differences in covalent bonding and zero‐point vibrational energy for the formation of **di‐7b″‐B_Ar_F** are in sum close to relative zero, the attractive LD interactions overcompensate the entropic penalty of dimer formation by more than a factor of two (Schemes S‐VI‐7,8,9). LD interactions can thus be regarded as the thermodynamic driving force for dimerization of **7b″‐B_Ar_F**. The dimeric structure of **di‐7b″‐B_Ar_F** is also responsible for the increased thermal stability of the perozonido unit, delaying its decay up to a temperature of –35°C despite a similar tendency toward dioxygen extrusion as **1b″‐B_Ar_F**. A graphical representation of all attractive and repulsive non‐covalent interactions (NCI) is depicted in Figure 8 [39, 40].

### 2.6 Reactions of Cobalt Complexes With Ozone and [N(CH_3_)_4_]O_3_

Applying ozone to a solution of **4a‐(OTf)_2_** in DCM at low temperatures repeatedly resulted in unidentifiable polymeric material. Sample degradation certainly involves decomposition of the acetonitrile ligand, as observed previously [41, 42]. However, in one experiment, a few crystals formed that could be picked out and structurally characterized.

**FIGURE 9:**
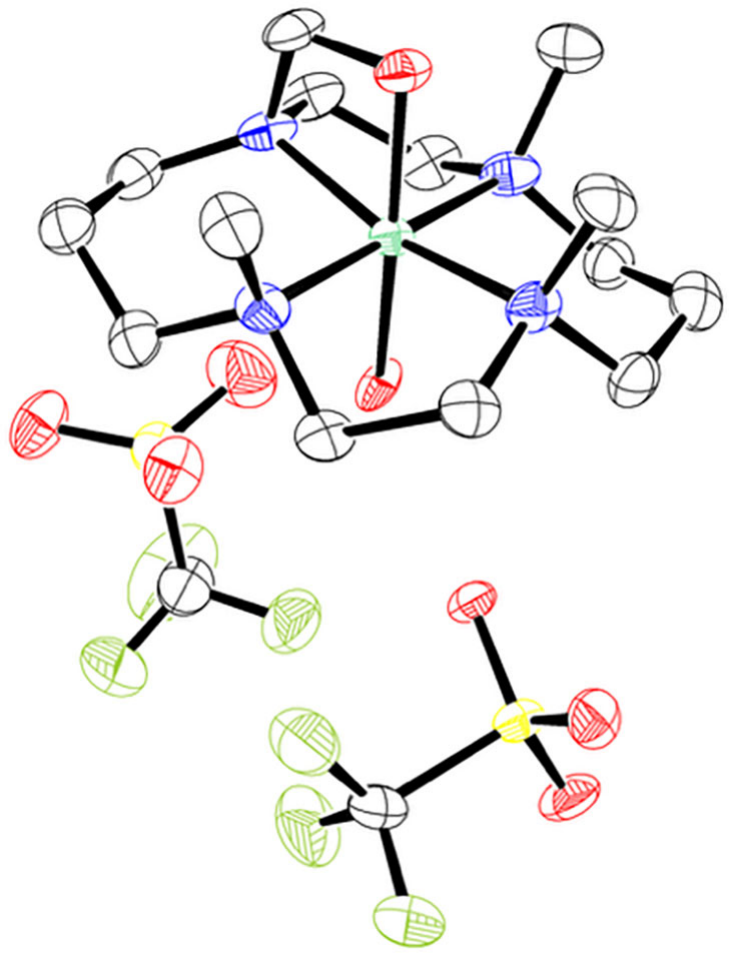
Molecular structure of [(TMCO)Co^III^(H_2_O)](OTf)_2_ (**8a‐(OTf)_2,_** color code: C, white; N, blue; O, red; S, yellow; Co, bright green; F, green; H omitted for clarity).

From the molecular structure, it is obvious that part of **4a‐(OTf)_2_** had undergone an intramolecular ligand hydroxylation to give [(TMCO)Co^III^(H_2_O)](OTf)_2_ (**8a‐(OTf)_2 _**; Figures 9 and S‐II‐14). Intramolecular hydroxylation of a methyl group has been reported before by Nam and coworkers: Reacting [(15‐TMC)Co^II^(CH_3_CN)_2_](ClO_4_)_2_ with hydrogen peroxide leads to Co(III) complex [(15‐TMC‐CH_2_‐O)Co^III^(OH)](ClO_4_), analogously to **8a‐(OTf)_2_** [43]. We envisioned that replacing the methyl groups in the TMC ligand with CF_3_‐groups would suppress their hydroxylation. However, (CF_3_)_4_‐cyclam (Scheme 1, b, R = CF_3_) turned out to be unsuitable for our purpose: The Lewis basicity of the amine nitrogen atoms decreased to such an extent that metal complex formation was not observed [44].

**FIGURE 10:**
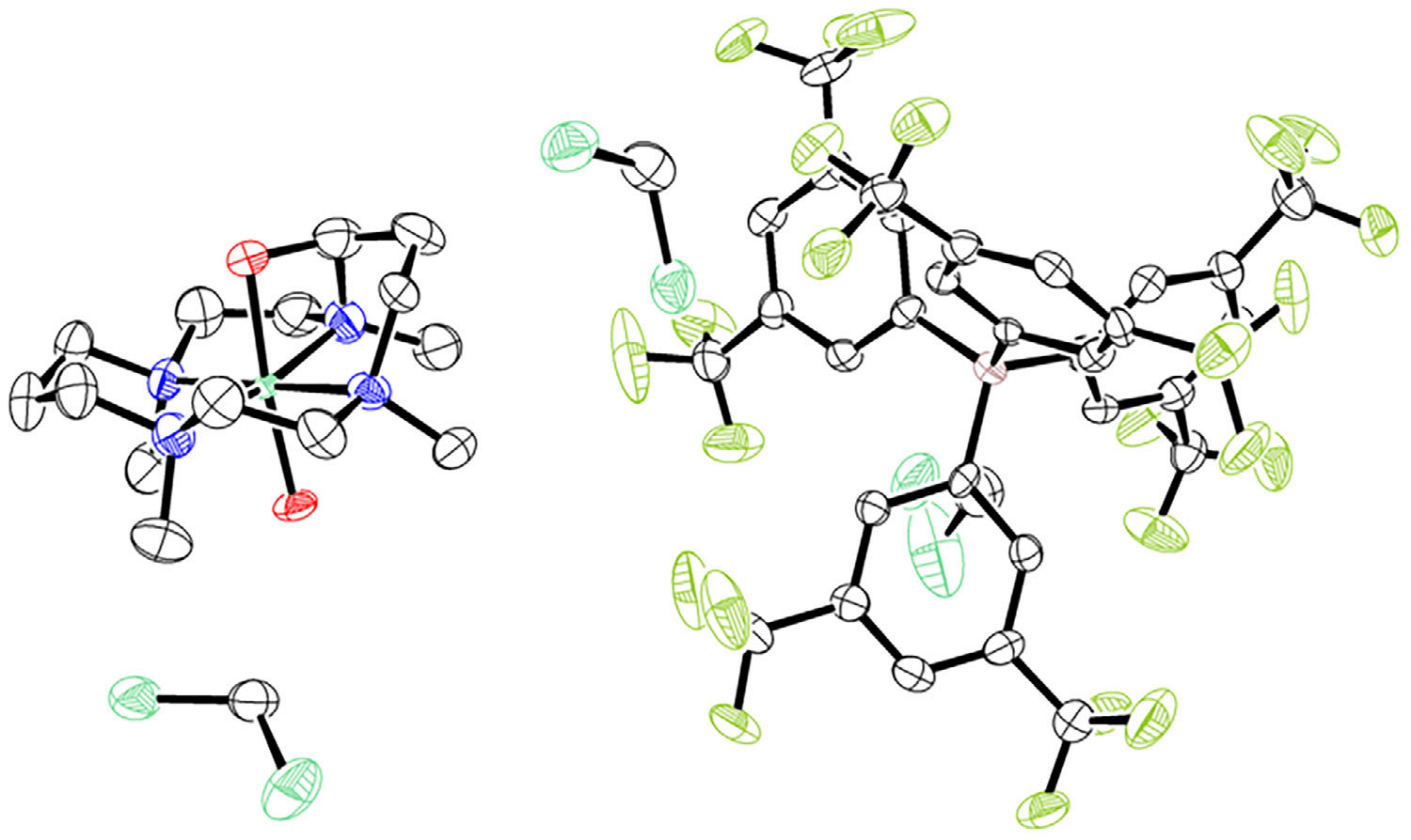
Molecular structure of **9b‐B_Ar_F**, [(D_12_‐TMC‐propyl‐O)Co^III^(OH)]B_Ar_F · 3 CH_2_Cl_2_ (color code: C, white; N, blue; O, red; Co, Cl, bright green; F, green; H omitted for clarity).

It is known that substitution of hydrogen by deuterium can slow oxidation or hydroxylation reactions [45, 46, 47]. In an attempt to probe the viability of a straightforward ligand exchange, we reacted **5b‐B_Ar_F** with [N(CH_3_)_4_]O_3_ in DCM at low temperatures. After several weeks at −80°C, we obtained [(D_12_‐TMC‐propyl‐O)Co^III^(OH)OTf]B_Ar_F · 3 CH_2_Cl_2_ (**9b‐B_Ar_F**), which we isolated by crystal picking (Figures 10 and S‐II‐15). Working with [N(CH_3_)_4_]O_3_ requires careful handling and a special experimental setup (see Supporting Information part I) [48, 49].

As anticipated, the molecular structure of **9b‐B_Ar_F** shows that all CD_3_ groups were still intact. Instead, hydroxylation had occurred at one of the propyl sites, forming a different hydroxylated ligand system compared to **8a‐(OTf)_2_**. Hydroxylation of the TMC ligand at positions other than the methyl groups has not been previously reported.

### 2.7 Cryospray Ionization Mass Spectrometry

Complex **5b‐B_Ar_F** (for **5a‐B_Ar_F**, see Supporting Information part IV) was reacted with ozone, and the product solution was analyzed by MS. The results are presented in Table 2. We observed largely similar products as in the case of the iron complexes **1a‐B_Ar_F** and **1b‐B_Ar_F**. A Co‐oxido complex could be observed in the form of [(D_12_‐TMC)Co^IV^(O)(OTf)]^+^ (*m/z*  492.2181). Despite its noteworthy absence in our UV–vis experiments, traces of a Co‐ozonido complex were detected as [(D_12_‐TMC)Co^II^(O_3_)]^+^ (*m/z*  375.2551). In MS/MS mode, we found a clean exchange of the ozonido for the formato ligand to give ion [(D_12_‐TMC)Co^II^(HCOO)]^+^ (*m/z*  372.2686).

$$\begin{array}{cc} & {\left[\left(D_{12}-\mathrm{TMC}\right){\mathrm{Co}}^{\mathrm{III}}\left(O_{3}\right)\left(\mathrm{OTf}\right)\right]}^{+}+H^{+}+e^{-}\\ & \;\overset{\mathrm{ESI}-\mathrm{MS}}{\to }{\left[\left(D_{12}-\mathrm{TMC}\right){\mathrm{Co}}^{\mathrm{II}}\left(O_{3}\right)\right]}^{+}+\mathrm{HOTf} \end{array}$$

$$\begin{array}{cc} & {\left[\left(D_{12}-\mathrm{TMC}\right){\mathrm{Co}}^{\mathrm{II}}\left(O_{3}\right)\right]}^{+}+\mathrm{HCOO}H\\ & \;\overset{\mathrm{MS}/\mathrm{MS}}{\to }{\left[\left(D_{12}-\mathrm{TMC}\right){\mathrm{Co}}^{\mathrm{II}}\left(\mathrm{HCOO}\right)\right]}^{+}+\mathrm{HO}•+O_{2} \end{array}$$

**TABLE 2:** Summary of key species detected in the reaction mixture of **5b‐B_Ar_F** with ozone.

*m/z*		Detected ion	Species
Exp.	Calcd.		
342.2578	342.2578	[(D_12_‐TMC‐propyl‐O)Co]^+^	Co^II^‐propyl‐O
359.2597	359.2605	[(D_12_‐TMC‐propyl‐O)Co(OH)]^+^ [(D_12_‐TMC)Co(OO)]^+^	Co^III^‐propyl‐O / Co^II^−O_2_
372.2686	372.2683	[(D_12_‐TMC)Co(HCOO)]^+^	Co^II^
375.2551	375.2554	[(D_12_‐TMC)Co(O_3_)]^+^	Co^II^−O_3_
388.2625	388.2623	[(D_12_‐TMC)Co(O)(HCOO)]^+^	Co^IV^=O
492.2181	492.2176	[(D_12_‐TMC)Co(O)(OTf)]^+^	Co^IV^=O

As exemplified by the molecular structure derived from single‐crystal x‐ray diffraction of **9b‐B_Ar_F**, we also found mass‐spectrometric evidence for oxygen insertion into ligand C─H bonds, notably in the form of species [(D_12_‐TMC‐propyl‐O)Co^II^]^+^ (*m/z*  342.2578). A second signal with *m/z*  359.2597 may indicate [(D_12_‐TMC‐propyl‐O)Co^III^(OH)]^+^, but also fits a potential Co‐superoxido complex [(D_12_‐TMC)Co^II^(OO)]^+^. For the latter, we found no evidence outside of CSI‐MS measurements.

Product ions of other oxygen‐containing intermediates, as well as such‐involving coordination by solvent‐derived ligands, are given in the Supporting Information part IV.

For the reaction of **7b‐B_Ar_F** with ozone, the CSI‐MS results are presented in Table 3. We observed a Co‐ozonido complex as [(D_12_‐TMC)Co^II^(O_3_)]^+^ (*m/z*  375.2551) and a Co‐oxido complex as [(D_12_‐TMC)Co^IV^(O)(F)]^+^ (*m/z*  362.2640). We continued to investigate both in MS/MS mode: For the Co‐ozonido complex ion, we again found exchange of the ozonido for the formato ligand to give [(D_12_‐TMC)Co^II^(HCOO)]^+^ (*m/z*  372.2686).

**TABLE 3:** Summary of key species detected in the reaction mixture of **7b‐B_Ar_F** with ozone.

*m/z*		Detected ion	Species
Exp.	Calcd.		
342.2573	342.2578	[(D_12_‐TMC‐propyl‐O)Co]^+^	Co^II^‐propyl‐O
359.2600	359.2605	[(D_12_‐TMC‐propyl‐O)Co(OH)]^+^ [(D_12_‐TMC)Co(OO)]^+^	Co^III^‐propyl‐O / Co^II^−O_2_
362.2640	362.2640	[(D_12_‐TMC)Co(O)(F)]^+^	Co^IV^=O
363.2732	362.2718	[(D_12_‐TMC)Co(OH)(F)]^+^	Co^III^−OH
365.2676	365.2675	[(D_12_‐TMC)Co(F)_2_]^+^	F−Co^III^−F
375.2550	375.2554	[(D_12_‐TMC)Co(O_3_)]^+^	Co^II^−O_3_
388.2628	388.2623	[(D_12_‐TMC)Co(O)(HCOO)]^+^	Co^IV^=O

Notably, the Co‐oxido complex ion yields a signal with increased *m/z* ratio, which we identified as [(D_12_‐TMC)Co^III^(OH)(F)]^+^ (*m/z*  363.2732) and envision to be the result of hydrogen capture by collision with an H‐donor, for example, formic acid or formate:

$$\begin{array}{cc} & {\left[\left(D_{12}\text{-TMC}\right){\text{Co}}^{\text{IV}}\left(O\right)\left(F\right)\right]}^{+}+\text{”H”}\\ & \;\overset{\text{MS/MS}}{\to }{\left[\left(D_{12}\text{-TMC}\right){\text{Co}}^{\text{III}}\left(\text{OH}\right)\left(F\right)\right]}^{+} \end{array}$$

Further support for dinuclear complex **di‐7b″‐B_Ar_F** comes from the ratio of signals for [(D_12_‐TMC)Co^IV^(O)(F)]^+^ (*m/z*  362.2640) and [(D_12_‐TMC)Co^II^(OO)]^+^ (*m/z*  359.2600): These neighboring signals show very similar intensity, thus indicating they originate from the same parent complex ion (see Figure S‐IV‐1). This parent ion is likely {[(D_12_‐TMC)(F)Co^III^](O_3_)[Co^II^(D_12_‐TMC)]}^2+^, which is formed from **di‐7b″‐B_Ar_F**, but cannot be observed directly:

$${\left\{{\left[\left(D_{12}-\mathrm{TMC}\right){\mathrm{Co}}^{\mathrm{III}}\left(F\right)\right]}_{2}\left(O_{3}\right)\right\}}^{2+}+H^{+}+e^{-}\overset{\mathrm{ESI}-\mathrm{MS}}{\to }$$

$${\left\{\left[\left(D_{12}-\mathrm{TMC}\right){\mathrm{Co}}^{\mathrm{III}}\left(F\right)\right]\left(O_{3}\right)\left[{\mathrm{Co}}^{\mathrm{II}}\left(D_{12}-\mathrm{TMC}\right)\right]\right\}}^{2+}+\mathrm{HF}$$

$${\left\{\left[\left(D_{12}-\mathrm{TMC}\right){\mathrm{Co}}^{\mathrm{III}}\left(F\right)\right]\left(O_{3}\right)\left[{\mathrm{Co}}^{\mathrm{II}}\left(D_{12}-\mathrm{TMC}\right)\right]\right\}}^{2+}\overset{\mathrm{ESI}-\mathrm{MS}}{\to }$$

$$\begin{array}{cc} & {\left[\left(D_{12}-\mathrm{TMC}\right){\mathrm{Co}}^{\mathrm{IV}}\left(O\right)\left(F\right)\right]}^{+}+{\left[\left(D_{12}-\mathrm{TMC}\right){\mathrm{Co}}^{\mathrm{II}}\left(\mathrm{OO}\right)\right]}^{+}\\ & \left(1:1\;\mathrm{ratio}\right) \end{array}$$

In an MS/MS study of the [(D_12_‐TMC)Co^II^(OO)]^+^ (*m/z*  359.2600) ion, we observed [(D_12_‐TMC)Co^III^(OH)(F)]^+^ (*m/z*  363.2732) as its product ion, the same as found previously for [(D_12_‐TMC)Co^IV^(O)(F)]^+^. A conceivable reaction pathway can be formulated as follows (with **di‐7b″‐B_Ar_F** likely serving as the source of fluoride; vide supra):

$$\begin{array}{cc} & {\left[\left(D_{12}-\mathrm{TMC}\right){\mathrm{Co}}^{\mathrm{II}}\left(\mathrm{OO}\right)\right]}^{+}+\mathrm{HF}\\ & \;\overset{\mathrm{MS}/\mathrm{MS}}{\to }{\left[\left(D_{12}-\mathrm{TMC}\right){\mathrm{Co}}^{\mathrm{IV}}\left(O\right)\left(F\right)\right]}^{+}+\mathrm{HO}• \end{array}$$

$$\begin{array}{cc} & {\left[\left(D_{12}\text{-TMC}\right){\text{Co}}^{\text{IV}}\left(O\right)\left(F\right)\right]}^{+}+\text{”H”}\\ & \;\overset{\text{MS/MS}}{\to }{\left[\left(D_{12}\text{-TMC}\right){\text{Co}}^{\text{III}}\left(\text{OH}\right)\left(F\right)\right]}^{+} \end{array}$$

Our efforts to obtain EPR spectra of the reaction of **5a‐B_Ar_F** and **5b‐B_Ar_F** with ozone are described in detail in Supporting Information part V.

## 3 Summary and Conclusion

Iron‐oxido complexes are catalytically important and have been widely studied, with numerous examples reported [50, 51, 52, 53, 54], including from our own group [23]. In contrast, the corresponding Co‐oxido species have been reported less frequently, with only a handful of examples [21]. We herein describe the use of ozone as an oxidant to produce Fe/Co‐ozonido and Fe/Co‐oxido complexes from Fe(II) and Co(II) precursors, respectively.

The synthesis of suitable precursor complexes turned out to be a major endeavor. Our supporting ligand, the TMC macrocycle, has been used to generate Fe‐oxido complexes [23, 50, 51, 52, 53, 54, 55]. During the course of our experiments, we synthesized [(TMC)Co^II^(OTf)_2_] without coordinating solvent from Co(OTf)_2_, which required its own synthetic procedure to yield a pure salt. In addition, we established access to TMC complexes featuring weakly coordinating anions via silver(I) salts. Consisting of different anions but no coordinating solvent, these complexes greatly improved the versatility of our Fe and Co compounds to investigate their reactivities. Furthermore, substitution of CH_3_ for CD_3_ groups in the cyclam backbone to give the D_12_‐TMC ligand effectively prevents their oxidation at low temperatures.

We provide evidence for the existence of an iron‐ozonido complex, the existence of which we surmised in 2018 [23], as well as of Co‐ozonido complexes. We observed spectral features in accordance with those computed for [(D_12_‐TMC)Fe^III^(O_3_)(OTf)]B_Ar_Fand {[(D_12_‐TMC)(F)Co^III^]_2_(O_3_)}(B_Ar_F)_2_ at −150°C. The dinuclear Co complex is held together by strong intermolecular LD interactions of the D_12_‐TMC ligands and would provide the first example of the perozonido dianion in a transition metal complex. However, attempted crystallization of the ozonido complexes failed due to their propensity toward oxygen extrusion. Thus, they react further to presumably yield the corresponding oxido complexes, [(D_12_‐TMC)Fe^IV^(O)(OTf)]B_Ar_F and [(D_12_‐TMC)Co^IV^(O)(F)]B_Ar_F, respectively, which can also form directly from the respective precursor complexes at higher temperatures. In addition, we found evidence for [(D_12_‐TMC)Co^IV^(O)(OTf)]B_Ar_F. Unfortunately, the required special reaction conditions did not allow Raman or EXAFS measurements to be performed in order to further characterize the postulated species.

The presented evidence for Fe‐ and Co‐ozonido complexes at temperatures as low as −135°C showcases that there is room for additional experimental setups that go beyond what is typically achievable by commercial stopped‐flow systems. Moreover, together with the modification of ligand systems and the use of appropriate strongly and weakly coordinating anions, our work suggests many opportunities for the investigation of reactive transition metal oxygen adducts relevant to catalytic applications.

## Author Contributions

**Stefan Schaub**: investigation, writing – original draft, methods, conceptualization, writing – review and editing. **Dennis Gerbig**: investigation, writing – review and editing, data curation, software, formal analysis. **Raffael C. Wende**: methods, validation, formal analysis. **Heike Hausmann**: methods, validation, formal analysis. **Jonathan Becker**: methods, validation, formal analysis. **Christian Würtele**: formal analysis. **Laura Senft**: formal analysis. **Ina Kellner**: formal analysis, validation. **Gunasekaran Velmurugan**: software, methods, validation, visualization. **Marion Kerscher**: formal analysis. **Ivana Ivanović‐Burmazović**: formal analysis, validation. **Peter Comba**: validation, formal analysis, visualization. **Joshua Telser**: methods, validation, formal analysis, visualization, writing – review and editing. **Peter R. Schreiner**: writing – review and editing, resources. **Siegfried Schindler**: conceptualization, investigation, funding acquisition, writing – original draft, writing – review and editing, project administration, supervision, resources.

## Conflicts of Interest

The authors declare no conflicts of interest.

## Supporting information



Experimental (Part I), X‐ray diffraction (Part II), UV–vis spectroscopy (Part III), CSI‐MS (Part IV), EPR spectroscopy (Part V), Computational (Part VI).
UV/Vis and MS measurement data is available in CSV format at https://doi.org/10.22029/jlupub‐20542.
The authors have cited additional references within the Supporting Information [56, 57, 58, 59, 60, 61, 62, 63, 64, 65, 66, 67, 68, 69, 70, 71, 72, 73, 74, 75, 76, 77, 78, 79, 80, 81, 82, 83, 84, 85, 86, 87, 88, 89, 90, 91, 92, 93].
**Supporting File 1**: anie73371‐sup‐0001‐SuppMat.pdf.

**Supporting File 2**: anie73371‐sup‐0002‐SuppMat.pdf.

**Supporting File 3**: anie73371‐sup‐0003‐SuppMat.pdf.

**Supporting File 4**: anie73371‐sup‐0004‐SuppMat.pdf.

**Supporting File 5**: anie73371‐sup‐0005‐SuppMat.pdf.

**Supporting File 6**: anie73371‐sup‐0006‐SuppMat.pdf.

## Data Availability

The data that support the findings of this study are openly available in Forschungsdaten JLU at https://doi.org/10.22029/jlupub‐20542.
