# Supplementary material for: First Evidence for Ozonido‐TMC Complexes of Iron and Cobalt
Source: Angew Chem Int Ed Engl. 2026 Jul 9;65(37):e4716492. doi: 10.1002/anie.4716492 (PMC13548893; doi:10.1002/anie.4716492)
Supplement: Supplementary file 1 [file ANIE-65-e4716492-s001.pdf]

Supporting Information Part VI — Computational investigation

**A.**	**General Procedures**	**2**
**B.**	**Potential Energy Surfaces of the $[(D_{12}\text{-TMC})(X)Fe^{II}]^+$ (X = OTf, F, I) + O_3 systems**	**3**
**C.**	**Potential Energy Surfaces of the $[(D_{12}\text{-TMC})(X)Co^{II}]^+$ (X = OTf, F) + O_3 systems**	**5**
**D.**	**Isodesmic Equations for the $[(D_{12}\text{-TMC})(X)Fe^{II}]^+$ (X = OTf, F) + O_3 system**	**7**
**E.**	**Isodesmic Equations for the $[(D_{12}\text{-TMC})(X)Co^{II}]^+$ (X = OTf, F) + O_3 system**	**9**
**F.**	**Cartesian coordinates and thermochemical data for the $[(D_{12}\text{-TMC})(X)Fe^{II}]^+$ (X = OTf, F, I) + O_3 systems**	**10**
**H.**	**Miscellaneous cartesian coordinates and thermochemical data**	**59**
**I.**	**Molecular orbitals and spin densities for $[(D_{12}\text{-TMC})(OTf)Fe^{III}(O_3)]^+$**	**60**
**J.**	**Molecular orbitals and spin densities for $[(D_{12}\text{-TMC})(X)Co^{III}(O_3)]^+$ (X = OTf, F)**	**61**

A. General Procedures

All computations were performed using the ORCA program in version 5.0.4.[1,2] We employed the recent member of the 3c family of functionals by the Grimme group, ωB97X-3c,[3] to optimize minima and transition structures and to compute vibrational frequencies for multiple spin states. The custom double-zeta basis set of the method generally provides triple-zeta accuracy and includes large-core effective core potentials as well as fourth-generation dispersion corrections (D4).[4] The method was implemented manually into ORCA as described in the original publication.[3] The effect of the solvent was implicitly accounted for by the CPCM model parametrized for THF. We computed the electronic absorptions of our so-obtained geometries with time-dependent DFT (TD-DFT), using the ωB97X-D4 functional in conjunction with the ma-def2-TZVPP basis set.[5] All of our TD-DFT results were obtained without employing the Tamm-Dancoff approximation.[6] Note that the combination of computational efficiency and accuracy of the ωB97X-3c method turned out to be paramount for the feasibility of our computational investigations: The computed structures enabled us to obtain theoretical absorption values that were helpful in guiding our UV-Vis measurements. Depictions of molecular orbitals and spin densities are drawn with an isocontour value of 0.03 a.u.

B. Potential Energy Surfaces of the **[(D12-TMC)(X)Fe^{II}]$^+$ (X = OTf, F, I) + O_3** systems[1]

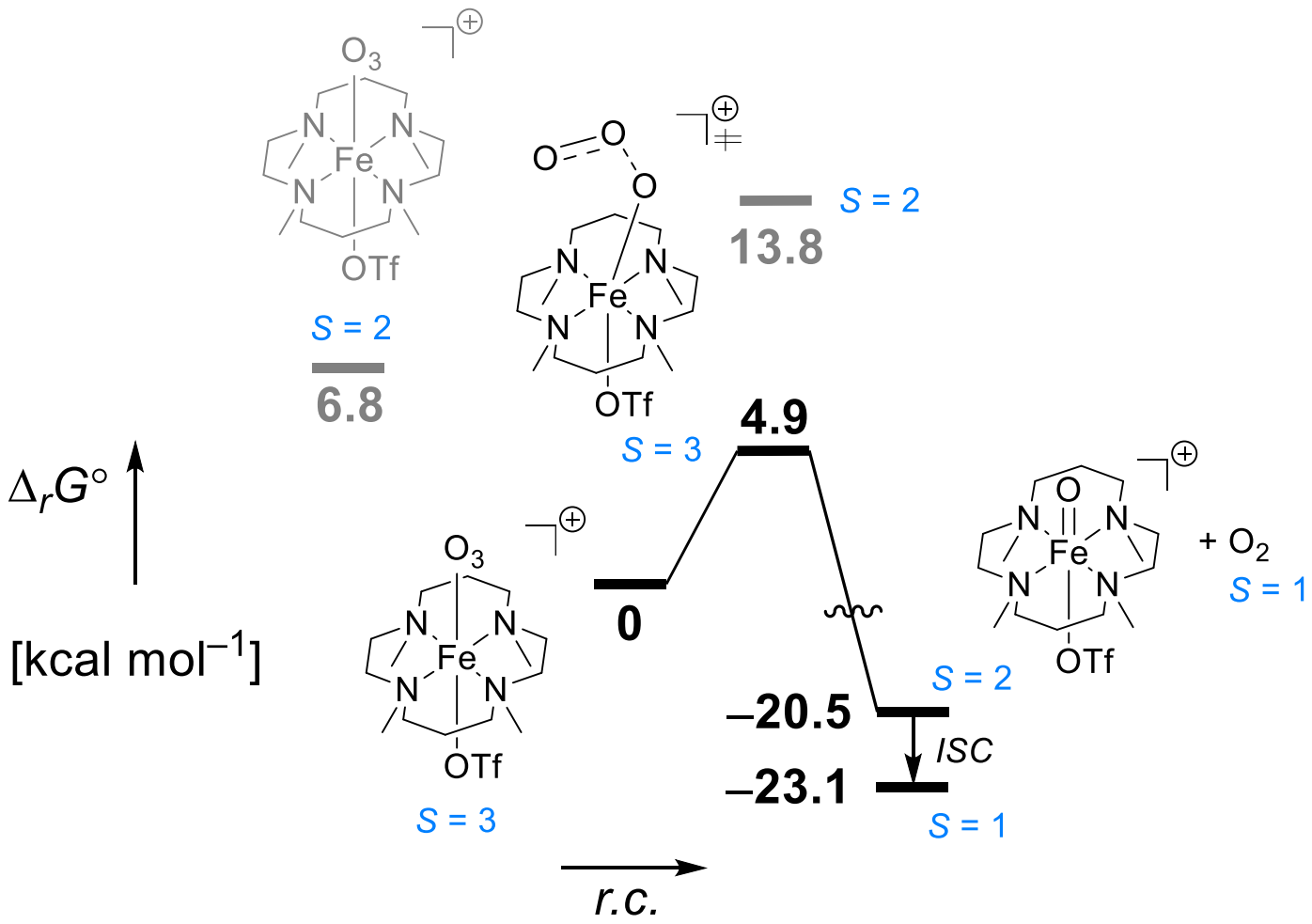

Scheme S1: Gibbs energy of reaction at T = 133 K for conversion of [(D12-TMC)(OTf)Fe^{III}(O_3)]$^+$ (methyl/O_3 *anti*) to [(D12-TMC)(OTf)Fe^{IV}(O)]$^+$ on the septet (S = 3) surface. Quintet (S = 2) [(D12-TMC)(OTf)Fe^{III}(O_3)]$^+$ given for comparison. Intersystem crossing (ISC) from quintet (S = 2) to triplet (S = 1) [(D12-TMC)(OTf)Fe^{IV}(O)]$^+$.

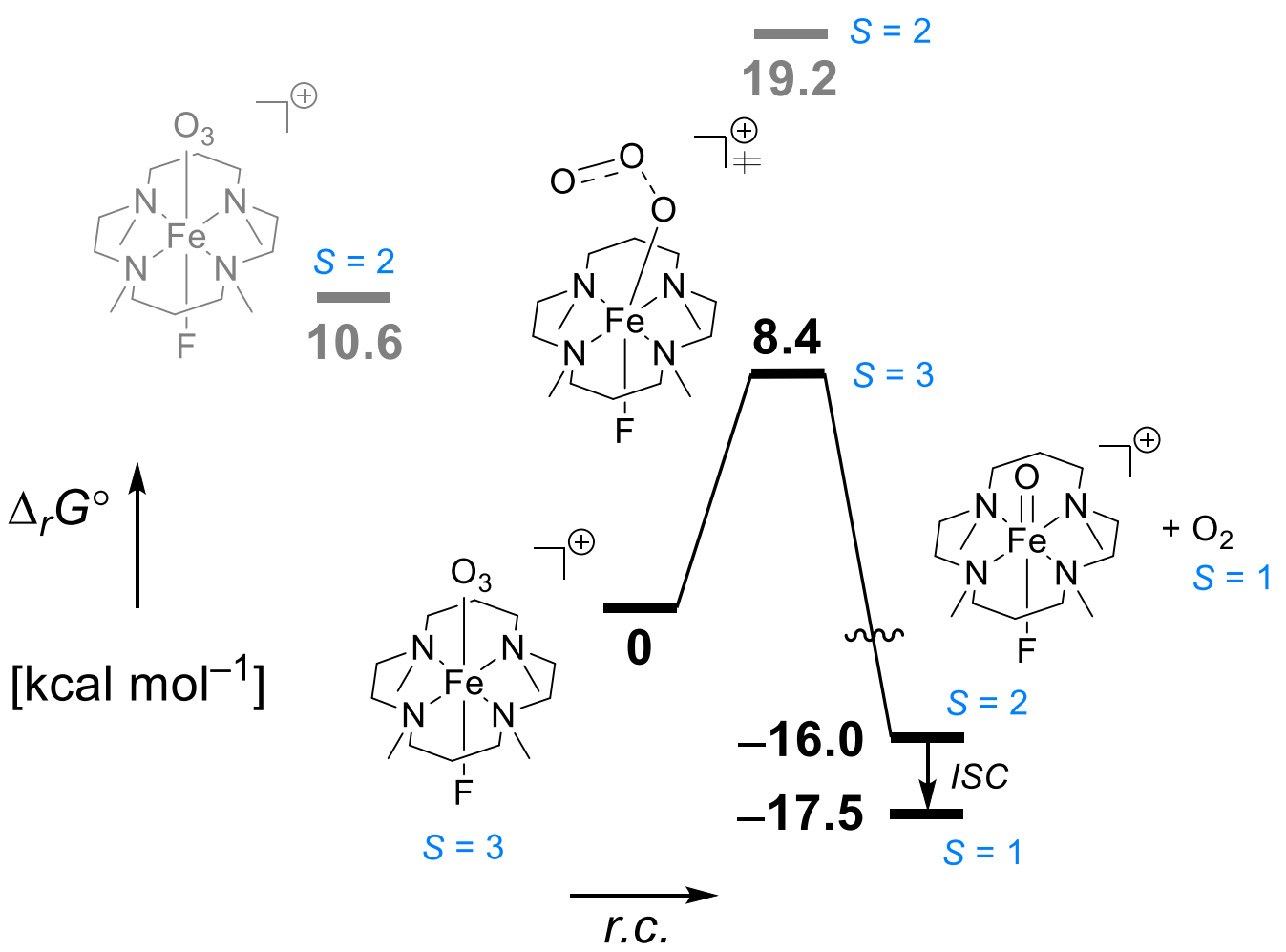

Scheme S2: Gibbs energy of reaction at T = 133 K for conversion of [(D12-TMC)(F)Fe^{III}(O_3)]$^+$ (methyl/O_3 *anti*) to [(D12-TMC)(F)Fe^{IV}(O)]$^+$ on the septet (S = 3) surface. Quintet (S = 2) [(D12-TMC)(F)Fe^{III}(O_3)]$^+$ given for comparison. Intersystem crossing (ISC) from quintet (S = 2) to triplet (S = 1) [(D12-TMC)(F)Fe^{IV}(O)]$^+$.

[1] CD_3 labels omitted for clarity

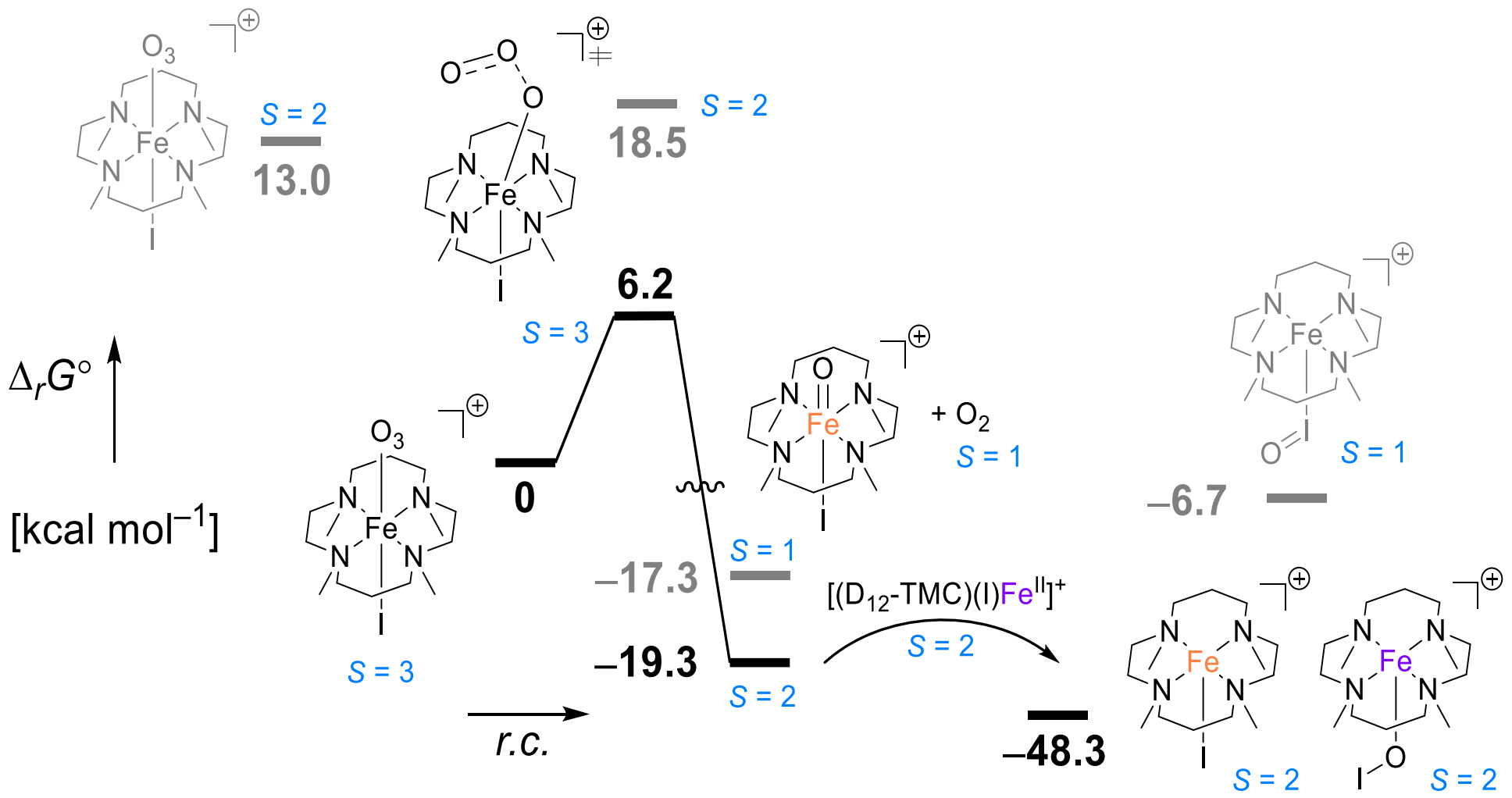

Scheme S3: Gibbs energy of reaction at T = 133 K for conversion of [(D$_{12}$-TMC)(I)Fe^{III}(O$_3$)]$^+$ (methyl/O$_3$ *anti*) to [(D$_{12}$-TMC)Fe^{II}(OI)]$^+$ *via* [(D$_{12}$-TMC)(I)Fe^{IV}(O)]$^+$ on the septet (S = 3) surface. Quintet (S = 2) [(D$_{12}$-TMC)(I)Fe^{III}(O$_3$)]$^+$ given for comparison. In contrast to [(D$_{12}$-TMC)(X)Fe^{IV}(O)]$^+$, X = OTf, F, the quintet state (S = 2) of [(D$_{12}$-TMC)(I)Fe^{IV}(O)]$^+$ is lower in energy than the triplet state (S = 1). Reaction to [(D$_{12}$-TMC)Fe^{II}(IO)]$^+$ (hypoiodito-*O*) occurs in a bimolecular fashion with a second equivalent of [(D$_{12}$-TMC)Fe^{II}(I)]$^+$ (color-coded). Isomeric [(D$_{12}$-TMC)Fe^{II}(IO)]$^+$ (hypoiodito-*I*) given for comparison.

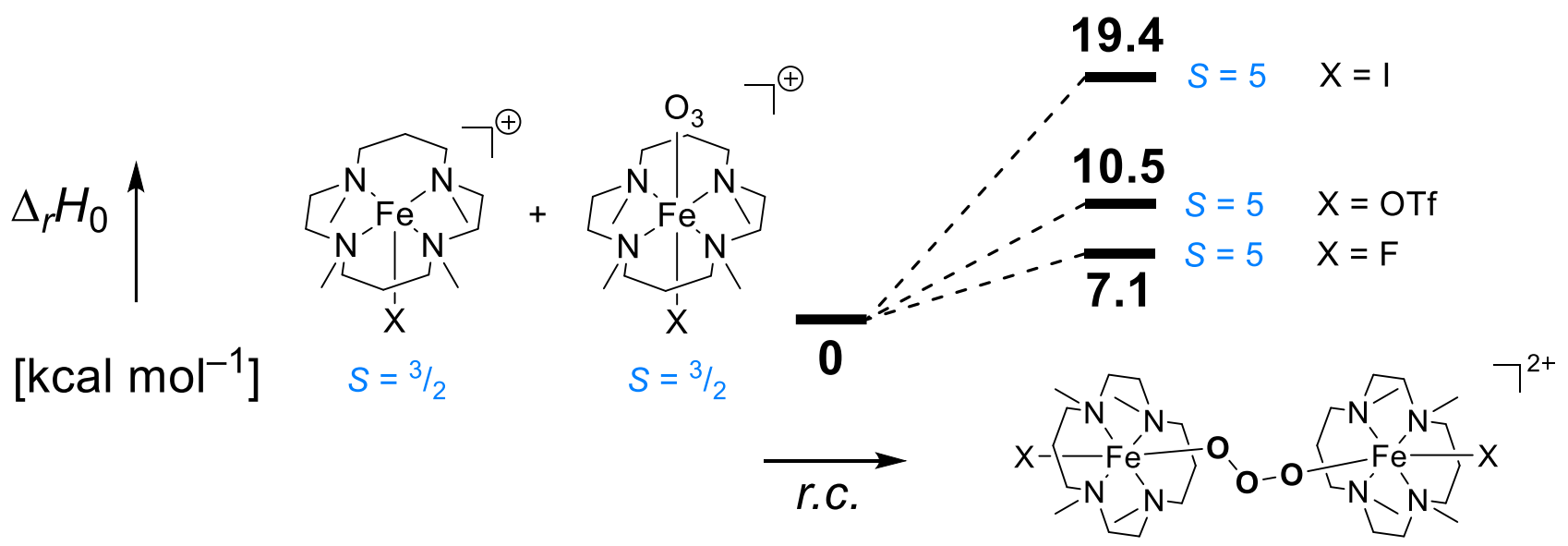

Scheme S4: Enthalpy of reaction at T = 0 K for generation of high spin {[(D$_{12}$-TMC)(X)Fe^{III}]$_2$(O$_3$)}$^{2+}$ from [(D$_{12}$-TMC)(X)Fe^{III}(O$_3$)]$^+$ (methyl/O$_3$ *anti*) and [(D$_{12}$-TMC)(X)Fe^{II}]$^+$ (X = OTf, F, I) on the undecet (S = 5) surface. Dimer formation is endothermic for all X.

C. Potential Energy Surfaces of the **$[(D_{12}\text{-TMC})(X)Co^{II}]^+$ (X = OTf, F) + O_3** systems[2]

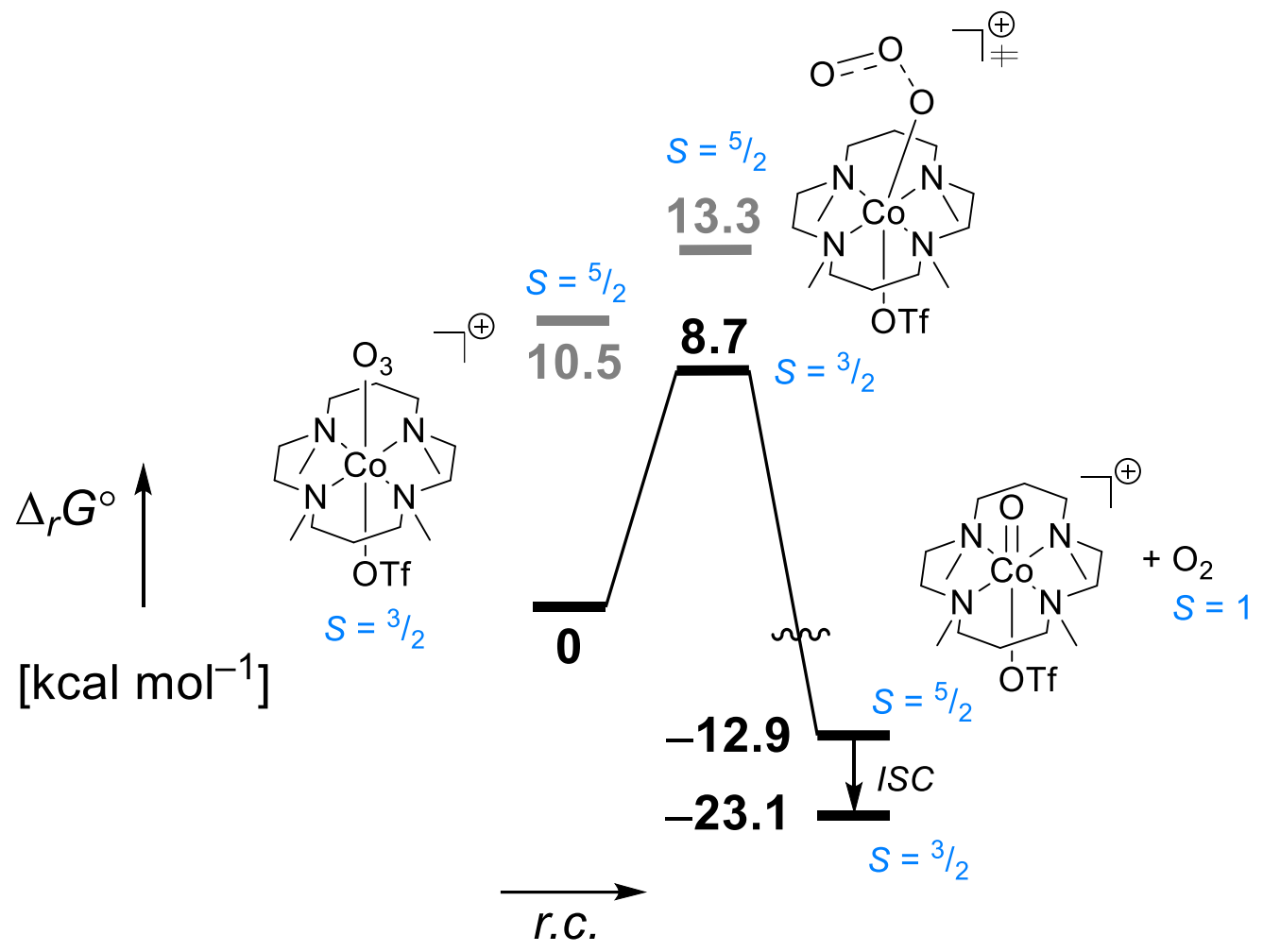

Scheme S5: Gibbs energy of reaction at $T = 133$ K for conversion of $[(D_{12}\text{-TMC})(OTf)Co^{III}(O_3)]^+$ (methyl/O_3 *anti*) to $[(D_{12}\text{-TMC})(OTf)Co^{IV}(O)]^+$ on the quartet ($S = ^3/_2$) surface. Sextet ($S = ^5/_2$) $[(D_{12}\text{-TMC})(OTf)Co^{III}(O_3)]^+$ given for comparison. Intersystem crossing (ISC) from sextet ($S = ^5/_2$) to quartet ($S = ^3/_2$) $[(D_{12}\text{-TMC})(OTf)Co^{IV}(O)]^+$.

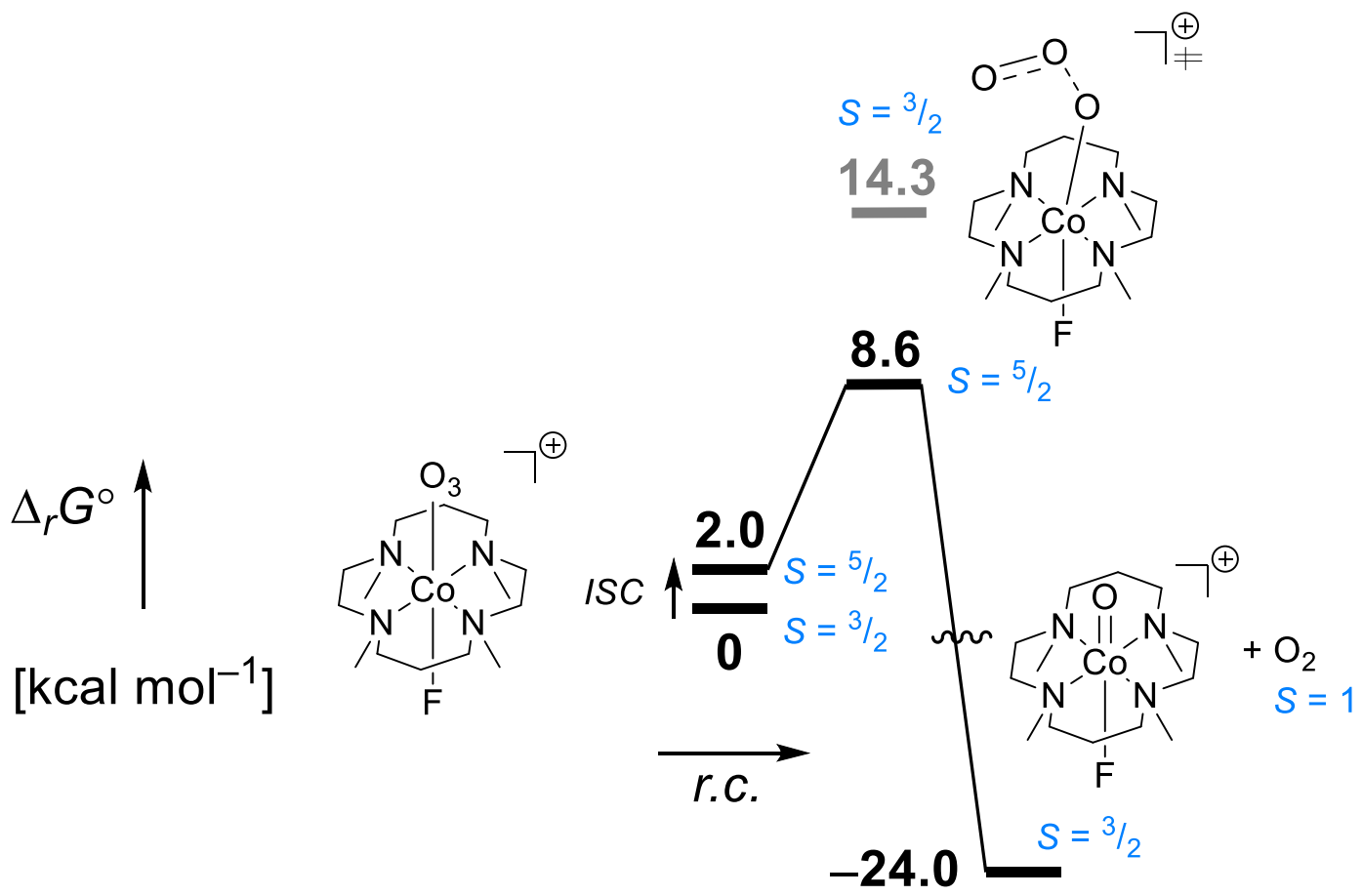

Scheme S6: Gibbs energy of reaction at $T = 133$ K for conversion of $[(D_{12}\text{-TMC})(F)Co^{III}(O_3)]^+$ (methyl/O_3 *anti*) to $[(D_{12}\text{-TMC})(F)Co^{IV}(O)]^+$ on the sextet ($S = ^5/_2$) surface. Intersystem crossing (ISC) from quartet ($S = ^3/_2$) to sextet ($S = ^5/_2$) $[(D_{12}\text{-TMC})(OTf)Co^{III}(O_3)]^+$. Quartet ($S = ^3/_2$) $[(D_{12}\text{-TMC})(OTf)Co^{III}(O_3)]^+$ given for comparison.

---

[2] CD_3 labels omitted for clarity

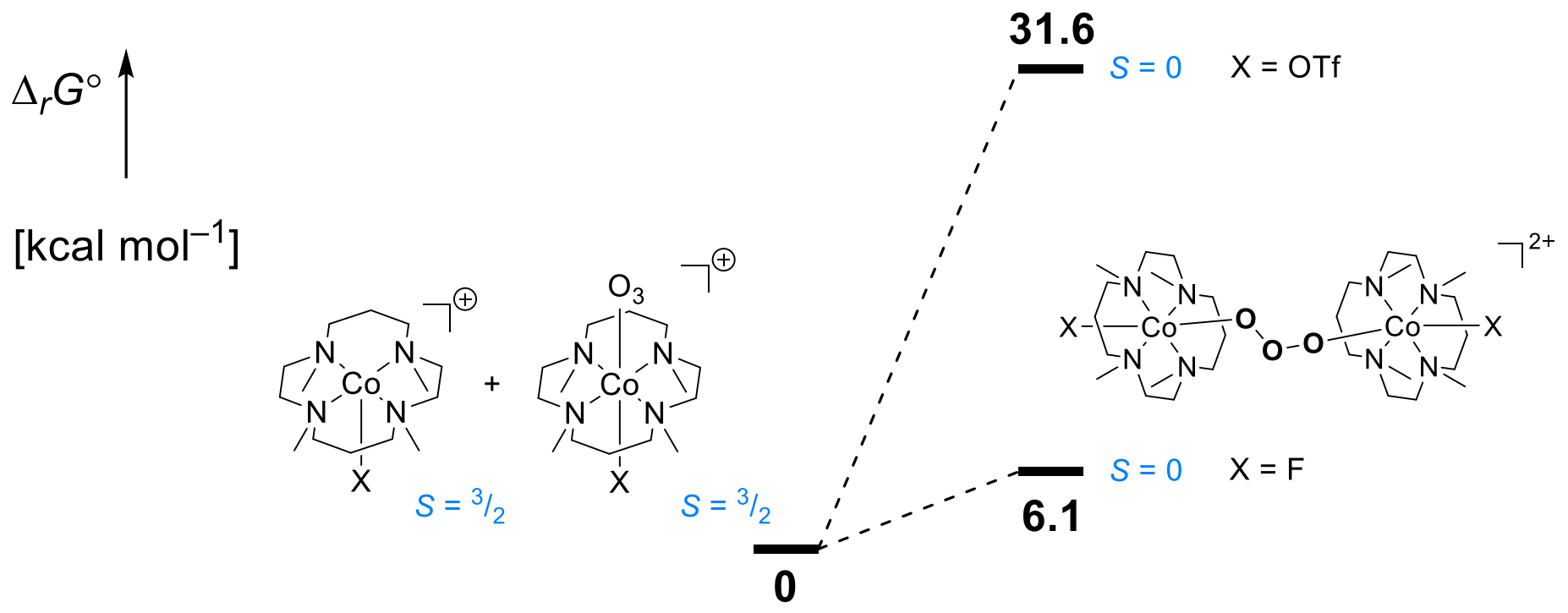

Scheme S7: Gibbs energy of reaction at $T = 133$ K for generation of low spin $\{[(D_{12}\text{-TMC})(X)Co^{III}]_2(O_3)\}^{2+}$ from $[(D_{12}\text{-TMC})(X)Co^{III}(O_3)]^+$ (methyl/O_3 *anti*) and $[(D_{12}\text{-TMC})(X)Co^{II}]^+$ (X = OTf, F) on the singlet ($S = 0$) surface. Dimer formation is slightly endergonic for X = F and strongly endergonic for X = OTf.

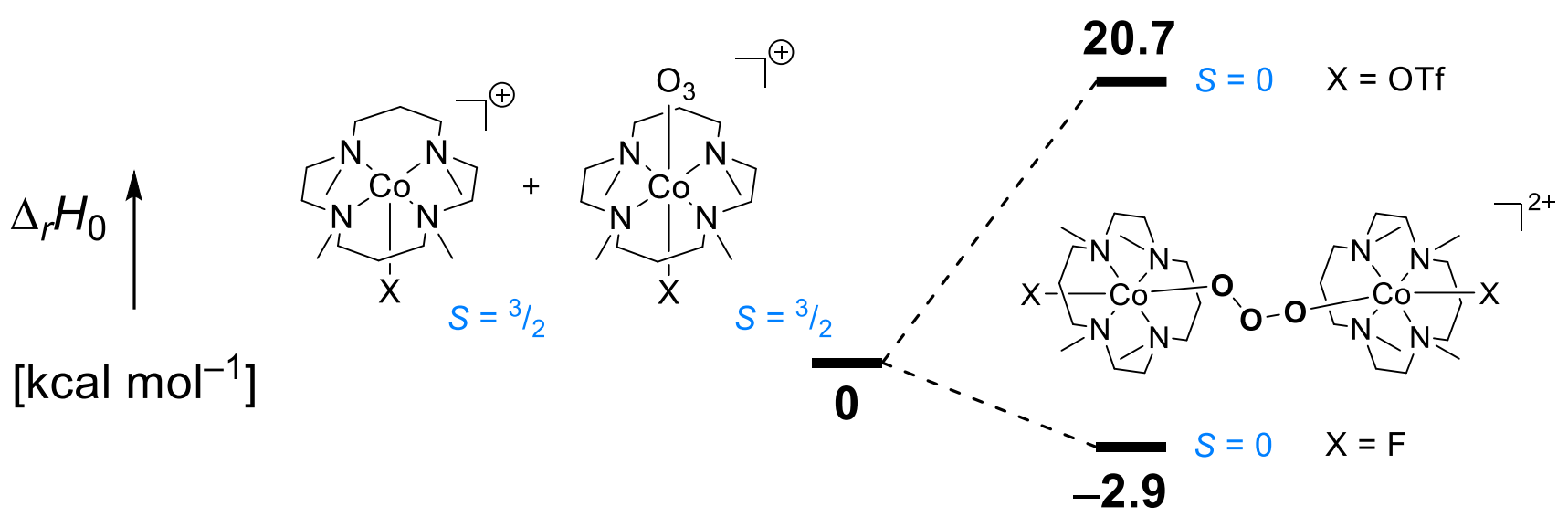

Scheme S8: Enthalpy of reaction at $T = 0$ K for generation of low spin $\{[(D_{12}\text{-TMC})(X)Co^{III}]_2(O_3)\}^{2+}$ from $[(D_{12}\text{-TMC})(X)Co^{III}(O_3)]^+$ (methyl/O_3 *anti*) and $[(D_{12}\text{-TMC})(X)Co^{II}]^+$ (X = OTf, F) on the singlet ($S = 0$) surface. Dimer formation is exothermic only for all X = F.

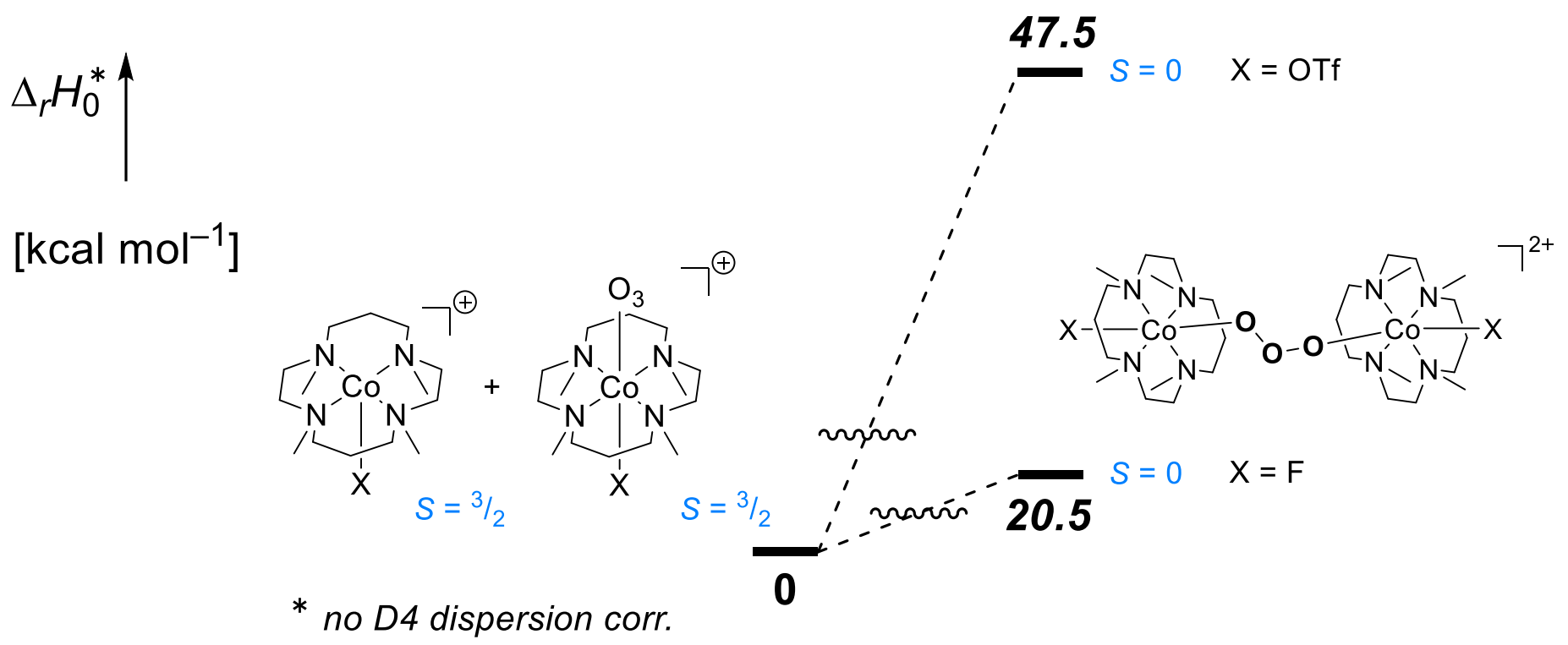

Scheme S9: Enthalpy of reaction at $T = 0$ K for generation of low spin $\{[(D_{12}\text{-TMC})(X)Co^{III}]_2(O_3)\}^{2+}$ from $[(D_{12}\text{-TMC})(X)Co^{III}(O_3)]^+$ (methyl/O_3 *anti*) and $[(D_{12}\text{-TMC})(X)Co^{II}]^+$ (X = OTf, F) on the singlet ($S = 0$) surface, but with dispersion contributions from D4 correction removed. Dimer formation is endothermic for X = OTf and X = F.

D. Isodesmic Equations for the **$[(D_{12}\text{-TMC})(X)Fe^{II}]^+$ (X = OTf, F, I) + O_3** system[3]

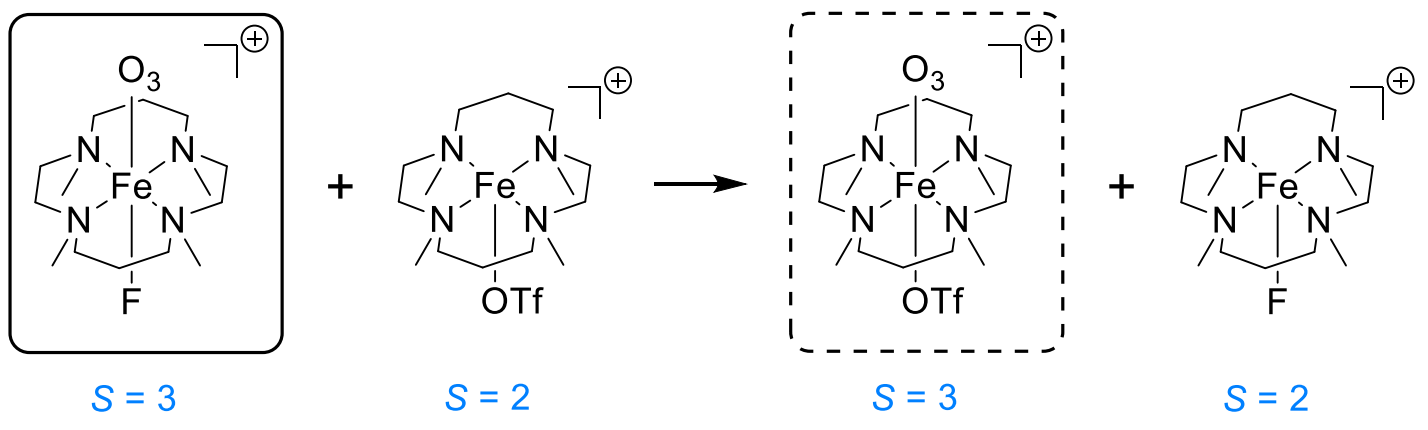

$\Delta_r G° = +10.2\ \text{kcal mol}^{-1}$

Equation S1: Isodesmic Gibbs energy of reaction at $T = 133$ K: $[(D_{12}\text{-TMC})(F)Fe^{III}(O_3)]^+$ is thermodynamically favored over $[(D_{12}\text{-TMC})(OTf)Fe^{III}(O_3)]^+$.

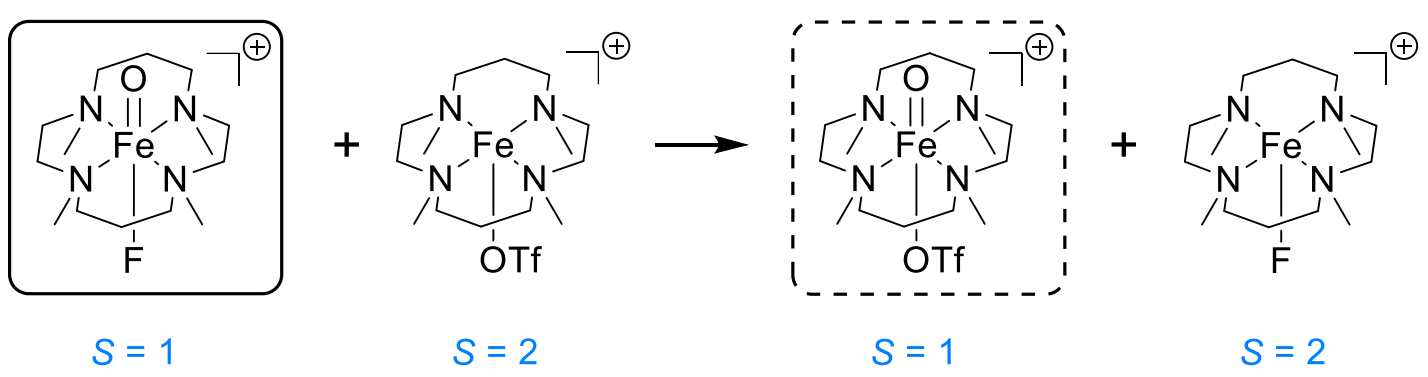

$\Delta_r G° = +4.7\ \text{kcal mol}^{-1}$

Equation S2: Isodesmic Gibbs energy of reaction at $T = 133$ K: $[(D_{12}\text{-TMC})(F)Fe^{IV}(O)]^+$ is thermodynamically favored over $[(D_{12}\text{-TMC})(OTf)Fe^{IV}(O)]^+$.

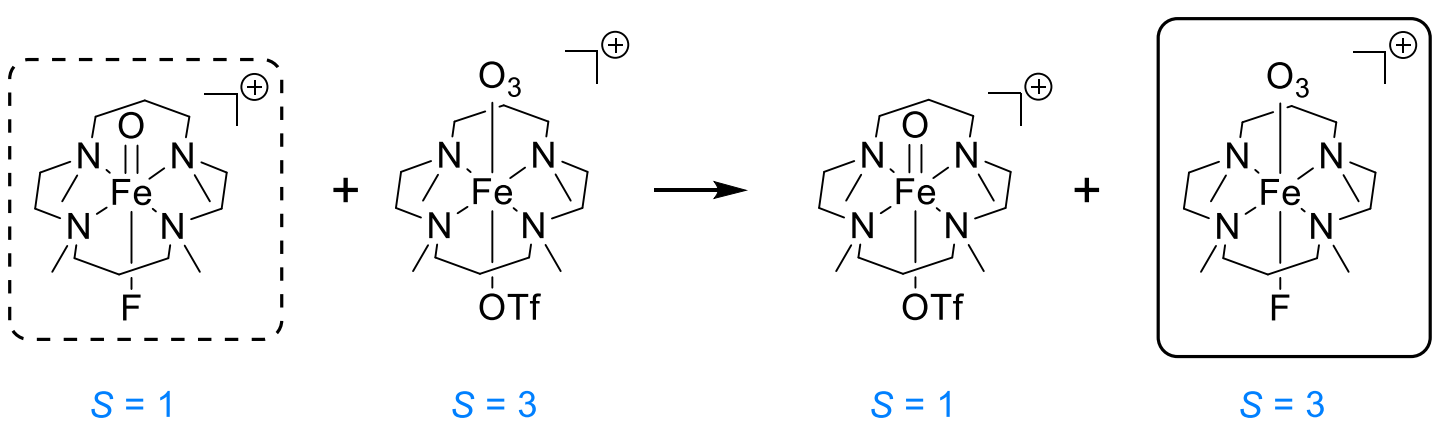

$\Delta_r G° = –5.6\ \text{kcal mol}^{-1}$

Equation S3: Isodesmic Gibbs energy of reaction at $T = 133$ K: $[(D_{12}\text{-TMC})(F)Fe^{III}(O_3)]^+$ is stabilized by the fluorido ligand to a larger extent than $[(D_{12}\text{-TMC})(F)Fe^{IV}(O)]^+$.

[3] CD_3 labels omitted for clarity

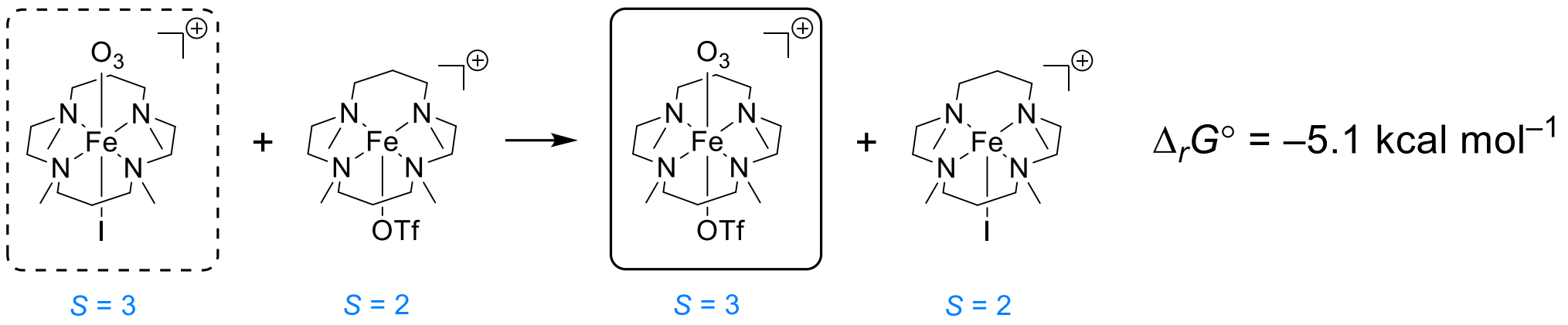

Equation S4: Isodesmic Gibbs energy of reaction at *T* = 133 K: $[(D_{12}\text{-TMC})(I)Fe^{III}(O_3)]^+$ is thermodynamically disfavored over $[(D_{12}\text{-TMC})(OTf)Fe^{III}(O_3)]^+$.

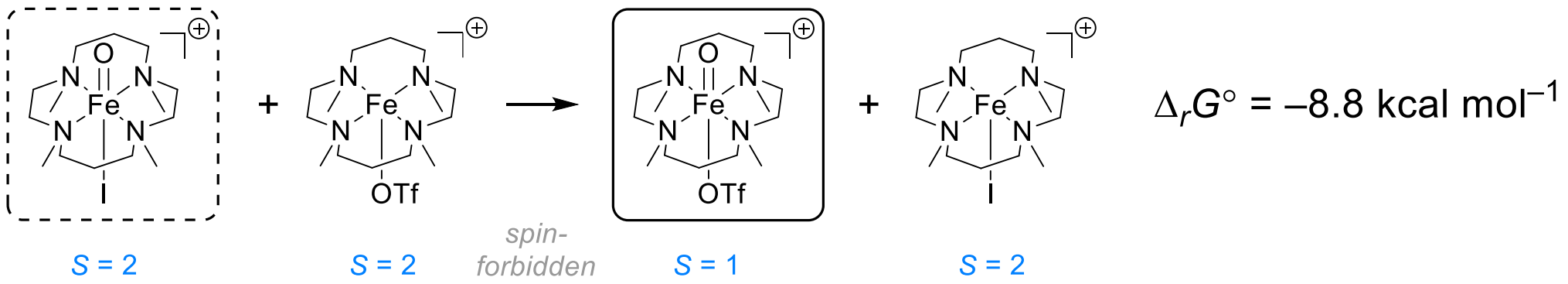

Equation S5: Isodesmic Gibbs energy of reaction at *T* = 133 K: $[(D_{12}\text{-TMC})(\mathbf{I})Fe^{IV}(O)]^+$ is thermodynamically disfavored over $[(D_{12}\text{-TMC})(OTf)Fe^{IV}(O)]^+$.

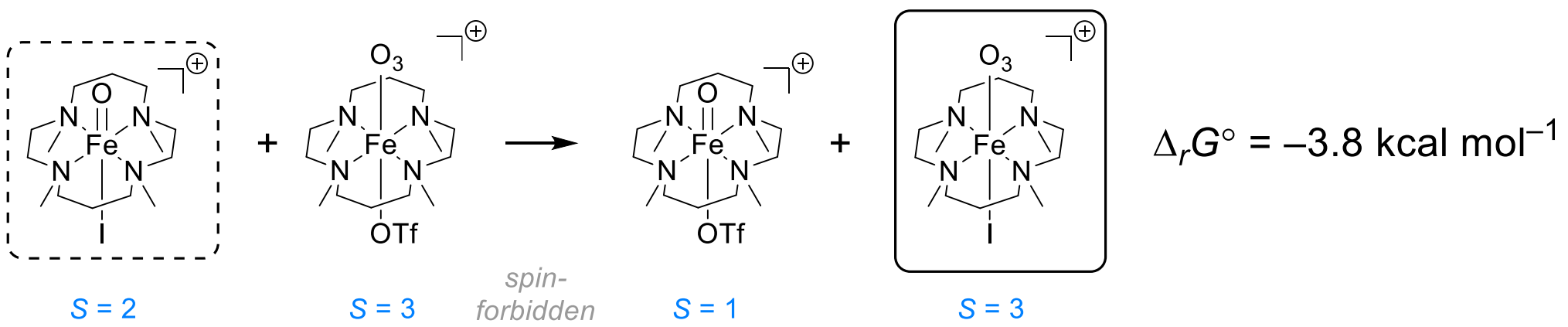

Equation S6: Isodesmic Gibbs energy of reaction at *T* = 133 K: $[(D_{12}\text{-TMC})(I)Fe^{III}(O_3)]^+$ is stabilized by the iodido ligand to a larger extent than $[(D_{12}\text{-TMC})(\mathbf{I})Fe^{IV}(O)]^+$.

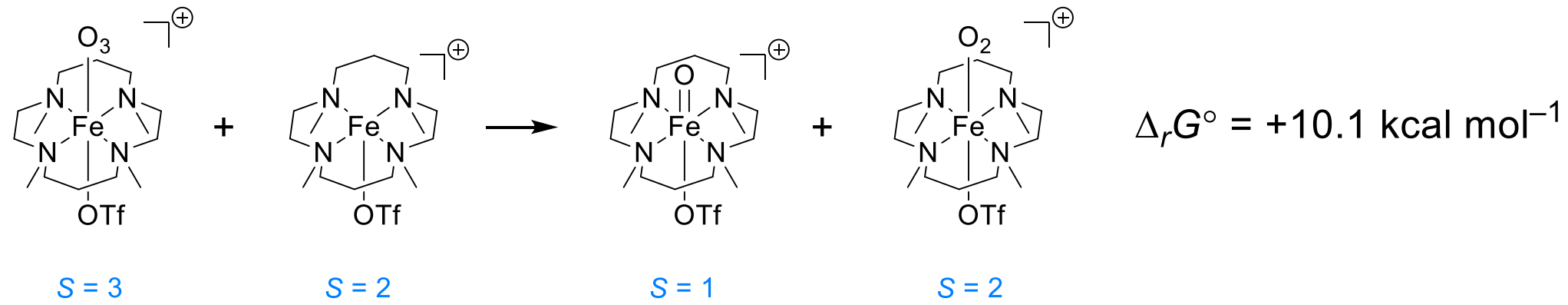

Equation S7: Isodesmic Gibbs energy of reaction at T = 133 K: Generation of $[(D_{12}\text{-TMC})(OTf)Fe^{IV}(O)]^+$ and $[(D_{12}\text{-TMC})(OTf)Fe^{III}(O_2)]^+$ from $[(D_{12}\text{-TMC})(OTf)Fe^{III}(O_3)]^+$ is endergonic.

E. Isodesmic Equations for the **$[(D_{12}\text{-TMC})(X)Co^{II}]^+$ (X = OTf, F) + O_3** system[4]

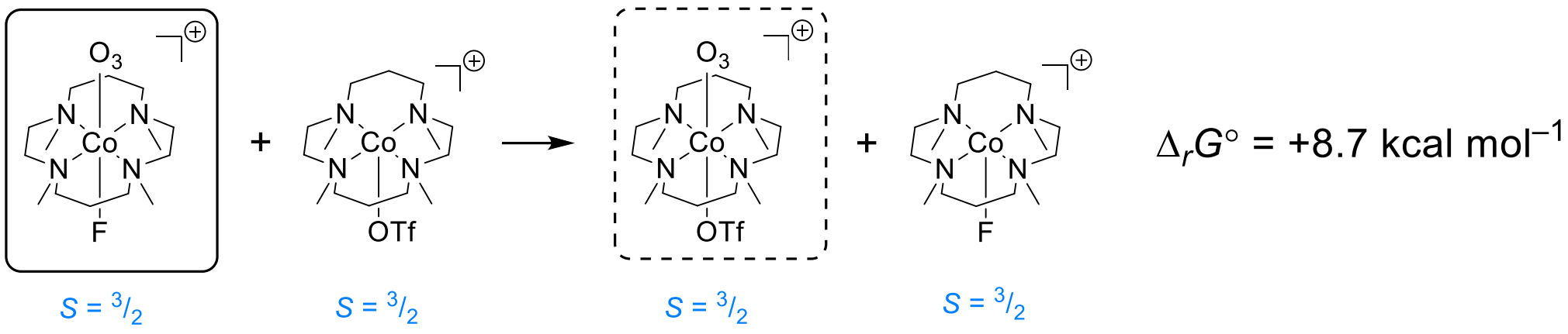

Equation S8: Isodesmic Gibbs energy of reaction at T = 133 K: $[(D_{12}\text{-TMC})(F)Co^{III}(O_3)]^+$ is thermodynamically favored over $[(D_{12}\text{-TMC})(OTf)Co^{III}(O_3)]^+$.

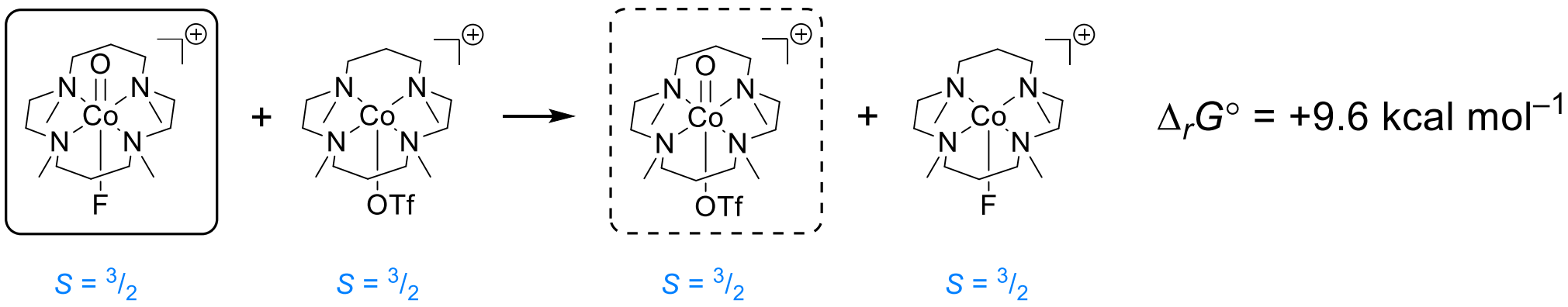

Equation S9: Isodesmic Gibbs energy of reaction at T = 133 K: $[(D_{12}\text{-TMC})(F)Co^{IV}(O)]^+$ is thermodynamically favored over $[(D_{12}\text{-TMC})(OTf)Co^{IV}(O)]^+$.

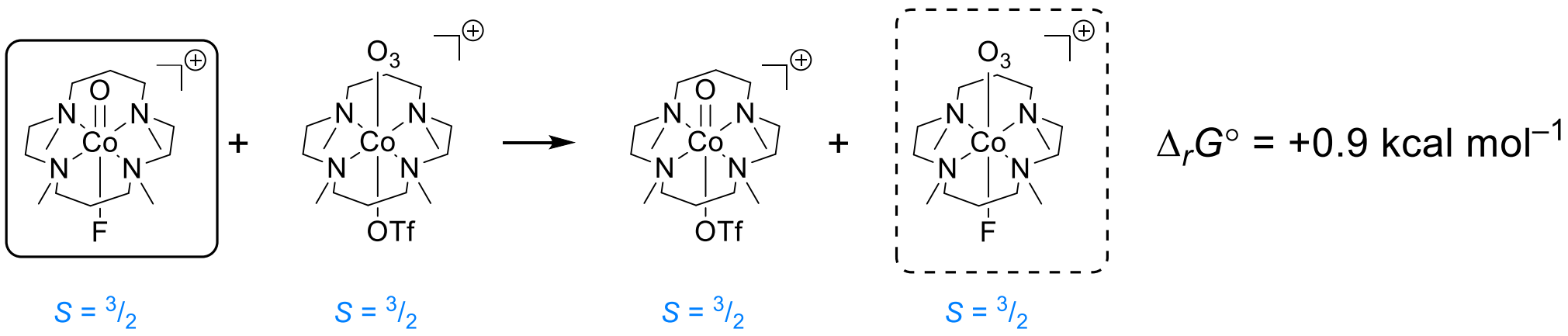

Equation S10: Isodesmic Gibbs energy of reaction at T = 133 K: $[(D_{12}\text{-TMC})(F)Co^{IV}(O)]^+$ is stabilized by the fluorido ligand to a slightly larger extent than $[(D_{12}\text{-TMC})(F)Co^{III}(O_3)]^+$.

[4] CD_3 labels omitted for clarity

F. Cartesian coordinates and thermochemical data for the **$[(D_{12}\text{-TMC})(X)Fe^{II}]^+$ (X = OTf, F, I) + O_3** systems

Electronic energy E_{el}, zero-point correction ZPE, thermal correction H_{133} to enthalpy at 133 K, entropy term S_{133} at 133 K; all quantities in units of hartree; total enthalpy at 0 K: $H_{tot} = E_{el}$ + ZPE ; total enthalpy at 133 K: $H_{tot} = E_{el} + H_{133}$; Gibbs energy at 133 K: $G = H_{tot} - S_{133}$.

$[(D_{12}\text{-TMC})(OTf)Fe^{III}(O_3)]^+$ (S = 3)

26	-0.256335000	-0.254828000	0.059040000
7	0.796439000	1.491874000	0.742599000
1	1.407275000	3.060571000	-2.241503000
1	-0.068467000	3.218694000	-1.359701000
7	-0.946328000	0.620595000	-1.838618000
6	1.531345000	2.163210000	-0.361524000
1	1.960406000	3.081799000	0.045797000
7	0.310868000	-1.102571000	1.999015000
6	0.039189000	-3.237143000	-0.484617000
1	2.224893000	1.735518000	2.280614000
7	-0.874789000	-2.121561000	-0.845515000
1	0.976584000	-3.063960000	-1.002109000
1	-0.393815000	-4.157861000	-0.882944000
6	0.195117000	1.360895000	-2.441300000
6	-1.353266000	-0.529266000	-2.695920000
6	1.778152000	0.925753000	1.699471000
1	2.358882000	1.523899000	-0.647115000
6	0.721803000	2.509401000	-1.596294000
1	-0.152563000	1.765304000	-3.395015000
1	0.988738000	0.650077000	-2.648485000
6	1.092503000	-2.336679000	1.716186000
1	-0.590242000	-3.685762000	1.536474000
6	-0.738225000	-1.850832000	-2.298677000
6	1.151301000	-0.061810000	2.655870000
1	0.940386000	-4.321465000	1.054489000
6	0.317109000	-3.428363000	0.994034000
1	1.425856000	-2.731124000	2.679140000
1	1.981971000	-2.068129000	1.156981000
1	1.939241000	-0.541153000	3.238409000
1	0.317567000	-1.867415000	-2.539457000
1	-1.227892000	-2.647936000	-2.862135000
1	2.576641000	0.462319000	1.135364000
1	-2.435431000	-0.599906000	-2.647622000
1	-1.096904000	-0.317028000	-3.734644000
1	0.515861000	0.468215000	3.358453000
8	1.384788000	-0.768466000	-0.893315000
8	-2.096367000	0.260758000	0.810057000
9	-4.041254000	2.505144000	0.551664000
16	-3.213231000	0.346765000	1.801861000
8	-2.881070000	1.176710000	2.937054000
8	-3.841975000	-0.928194000	2.050062000
6	-4.503198000	1.315274000	0.905106000
9	-4.908304000	0.668005000	-0.179442000
9	-5.539846000	1.481383000	1.710251000
6	-2.101819000	1.534828000	-1.771074000
1	-2.293945000	1.945731000	-2.763788000
1	-2.972215000	0.986682000	-1.444045000
1	-1.922135000	2.348296000	-1.081688000
6	-0.760418000	-1.457603000	2.951270000
1	-1.501738000	-2.097190000	2.492198000
1	-0.322166000	-1.978237000	3.804700000
1	-1.244715000	-0.560316000	3.311117000
6	-2.266005000	-2.488256000	-0.531019000
1	-2.542134000	-3.382535000	-1.091668000
1	-2.375695000	-2.684326000	0.528841000
1	-2.940186000	-1.679692000	-0.789190000
6	-0.065489000	2.466643000	1.434684000
1	-0.802268000	2.872842000	0.751578000
1	-0.594414000	1.995098000	2.254007000
1	0.549844000	3.280795000	1.820027000

8	2.752567000	-0.812504000	-0.594498000
8	3.419967000	-1.159558000	-1.613889000

E/h = -447.82439249
ZPE/h = 0.48613432
H_{133}/h = 0.49384507
S_{133}/h = 0.02653431

[(D12-TMC)(OTf)$Fe^{III}(O_3)$]$^+$ (*S* = 2)

26	-0.209457000	-0.270289000	0.035705000
7	0.765475000	1.423127000	0.693912000
1	1.436727000	3.024129000	-2.261652000
1	-0.043657000	3.220452000	-1.389643000
7	-0.917480000	0.602195000	-1.777310000
6	1.520182000	2.123145000	-0.381144000
1	1.936161000	3.028060000	0.065921000
7	0.303408000	-1.092541000	1.925277000
6	0.077811000	-3.201884000	-0.478510000
1	2.199016000	1.734422000	2.213570000
7	-0.827643000	-2.066716000	-0.803569000
1	1.012742000	-3.020470000	-0.996513000
1	-0.373168000	-4.100186000	-0.904583000
6	0.195681000	1.340861000	-2.433856000
6	-1.367552000	-0.526840000	-2.644579000
6	1.761354000	0.898676000	1.664928000
1	2.350558000	1.487968000	-0.665655000
6	0.734466000	2.495239000	-1.616453000
1	-0.195246000	1.729849000	-3.375995000
1	0.986292000	0.633414000	-2.659296000
6	1.106034000	-2.323692000	1.699002000
1	-0.543666000	-3.710012000	1.530492000
6	-0.719460000	-1.824629000	-2.267984000
6	1.123268000	-0.059464000	2.624433000
1	0.999721000	-4.310435000	1.031803000
6	0.356840000	-3.430824000	0.988090000
1	1.416105000	-2.684742000	2.681473000
1	2.005823000	-2.059921000	1.155331000
1	1.889042000	-0.546427000	3.228012000
1	0.333229000	-1.817149000	-2.522322000
1	-1.197965000	-2.647741000	-2.801002000
1	2.558323000	0.422191000	1.109312000
1	-2.446136000	-0.601656000	-2.554361000
1	-1.150411000	-0.293617000	-3.686809000
1	0.470169000	0.474206000	3.306735000
8	1.493868000	-0.755638000	-0.963455000
8	-2.158292000	0.257269000	0.826179000
9	-4.056565000	2.552668000	0.588125000
16	-3.269605000	0.359751000	1.806692000
8	-2.933862000	1.172239000	2.957866000
8	-3.935119000	-0.900951000	2.050977000
6	-4.541881000	1.365024000	0.925030000
9	-4.956341000	0.744456000	-0.173745000
9	-5.581750000	1.543130000	1.724795000
6	-2.066100000	1.528309000	-1.672787000
1	-2.262770000	1.950613000	-2.658823000
1	-2.936002000	0.983956000	-1.342429000
1	-1.869072000	2.331438000	-0.978460000
6	-0.799789000	-1.446115000	2.846101000
1	-1.538012000	-2.070914000	2.366399000
1	-0.381692000	-1.981485000	3.699529000
1	-1.276986000	-0.547393000	3.208284000
6	-2.217393000	-2.436186000	-0.466799000
1	-2.492368000	-3.324098000	-1.035768000
1	-2.308213000	-2.651205000	0.589390000
1	-2.897962000	-1.629004000	-0.703568000
6	-0.124585000	2.384723000	1.376846000
1	-0.852427000	2.784875000	0.683681000
1	-0.659202000	1.906915000	2.187003000
1	0.480655000	3.201898000	1.768689000
8	2.819183000	-0.918369000	-0.575142000
8	3.566142000	-1.153195000	-1.577772000

E/h = -447.81585329
ZPE/h = 0.48812829
H_{133}/h = 0.49560111
S_{133}/h = 0.02596354

[(D$_{12}$-TMC)(OTf)Fe^{III}(O$_3$)]$^+$ TS O$_2$ extrusion (S = 3)

26	-0.213198000	-0.264218000	0.043621000
7	0.790725000	1.509638000	0.757419000
1	1.397429000	3.081106000	-2.225666000
1	-0.085611000	3.220275000	-1.352745000
7	-0.932002000	0.617451000	-1.839422000
6	1.521758000	2.184512000	-0.344329000
1	1.948113000	3.106307000	0.059613000
7	0.311401000	-1.097943000	2.014445000
6	0.040592000	-3.237747000	-0.470704000
1	2.252893000	1.724802000	2.269521000
7	-0.867846000	-2.123863000	-0.845677000
1	0.984147000	-3.064254000	-0.977246000
1	-0.386978000	-4.161077000	-0.869729000
6	0.208953000	1.364283000	-2.430117000
6	-1.323931000	-0.532158000	-2.701322000
6	1.779564000	0.925965000	1.693500000
1	2.349447000	1.544511000	-0.629531000
6	0.715571000	2.521406000	-1.583795000
1	-0.126635000	1.761095000	-3.391782000
1	1.011835000	0.657937000	-2.619262000
6	1.085710000	-2.335250000	1.735126000
1	-0.610011000	-3.667833000	1.547607000
6	-0.709994000	-1.852227000	-2.294629000
6	1.160136000	-0.058479000	2.658244000
1	0.917419000	-4.323265000	1.082090000
6	0.304934000	-3.423288000	1.011783000
1	1.418411000	-2.732486000	2.697627000
1	1.971639000	-2.069216000	1.168258000
1	1.954465000	-0.536244000	3.234038000
1	0.350788000	-1.860361000	-2.515442000
1	-1.185082000	-2.650671000	-2.869283000
1	2.556261000	0.449328000	1.109139000
1	-2.406430000	-0.607412000	-2.667767000
1	-1.054857000	-0.321062000	-3.737266000
1	0.535082000	0.476179000	3.366916000
8	1.356189000	-0.739122000	-0.771569000
8	-2.103560000	0.261754000	0.810752000
9	-4.060142000	2.495562000	0.540493000
16	-3.220477000	0.340543000	1.791548000
8	-2.909030000	1.173161000	2.933433000
8	-3.851034000	-0.935482000	2.042404000
6	-4.514326000	1.300856000	0.892388000
9	-4.911652000	0.653733000	-0.196594000
9	-5.558932000	1.461894000	1.690095000
6	-2.091886000	1.523876000	-1.769743000
1	-2.279743000	1.948183000	-2.758031000
1	-2.961657000	0.967417000	-1.454878000
1	-1.921558000	2.327447000	-1.066314000
6	-0.758266000	-1.438754000	2.970688000
1	-1.502219000	-2.081170000	2.519495000
1	-0.323201000	-1.950262000	3.831848000
1	-1.241952000	-0.535916000	3.317906000
6	-2.262183000	-2.485369000	-0.546801000
1	-2.538384000	-3.376687000	-1.112870000
1	-2.382348000	-2.683089000	0.511685000
1	-2.929904000	-1.672148000	-0.807137000
6	-0.065528000	2.475382000	1.462853000
1	-0.798355000	2.895087000	0.782806000
1	-0.602610000	1.991072000	2.269620000
1	0.548702000	3.282176000	1.866522000
8	3.027163000	-0.844593000	-0.697436000
8	3.459975000	-1.290087000	-1.732730000

E/h = -447.81456165
ZPE/h = 0.48416571
H_{133}/h = 0.49193485
S_{133}/h = 0.02669445

[(D_{12}-TMC)(OTf)Fe^{III}(O_3)]$^+$ TS O_2 extrusion (S = 2)

26	-0.166683000	-0.277768000	0.014787000
7	0.756043000	1.434718000	0.702128000
1	1.431075000	3.040713000	-2.249219000
1	-0.057399000	3.221443000	-1.387771000
7	-0.902006000	0.596332000	-1.779354000
6	1.510850000	2.140672000	-0.366821000
1	1.921195000	3.048621000	0.079865000
7	0.303367000	-1.091638000	1.935361000
6	0.086702000	-3.201633000	-0.470847000
1	2.202760000	1.734003000	2.212958000
7	-0.814468000	-2.066609000	-0.805063000
1	1.025104000	-3.018282000	-0.981903000
1	-0.361498000	-4.101759000	-0.896560000
6	0.209191000	1.343215000	-2.427352000
6	-1.339388000	-0.531953000	-2.652298000
6	1.754360000	0.903297000	1.664831000
1	2.341589000	1.504183000	-0.648141000
6	0.730504000	2.504592000	-1.607942000
1	-0.173684000	1.726155000	-3.375643000
1	1.008443000	0.640104000	-2.637742000
6	1.108994000	-2.318219000	1.709618000
1	-0.542395000	-3.701960000	1.537671000
6	-0.693204000	-1.828863000	-2.267179000
6	1.121139000	-0.056089000	2.627672000
1	1.000685000	-4.308177000	1.045803000
6	0.360878000	-3.426346000	0.997755000
1	1.424666000	-2.680974000	2.690190000
1	2.001356000	-2.048968000	1.155868000
1	1.891637000	-0.536325000	3.231386000
1	0.363161000	-1.815333000	-2.507271000
1	-1.162700000	-2.653593000	-2.806227000
1	2.540543000	0.421431000	1.098315000
1	-2.418802000	-0.610247000	-2.574597000
1	-1.111417000	-0.298870000	-3.692348000
1	0.469865000	0.479040000	3.310944000
8	1.435666000	-0.725741000	-0.831651000
8	-2.170128000	0.258802000	0.834426000
9	-4.055898000	2.560452000	0.592396000
16	-3.281822000	0.359426000	1.806549000
8	-2.956325000	1.165709000	2.967191000
8	-3.958471000	-0.898274000	2.045888000
6	-4.547676000	1.373310000	0.925765000
9	-4.962295000	0.759299000	-0.177762000
9	-5.592008000	1.556975000	1.719915000
6	-2.056647000	1.513387000	-1.673316000
1	-2.253683000	1.941502000	-2.657096000
1	-2.923910000	0.961684000	-1.347500000
1	-1.867318000	2.312293000	-0.972136000
6	-0.799086000	-1.444141000	2.854367000
1	-1.537896000	-2.067417000	2.372848000
1	-0.386369000	-1.979582000	3.710969000
1	-1.278484000	-0.544962000	3.213188000
6	-2.206058000	-2.429660000	-0.474864000
1	-2.485027000	-3.315888000	-1.045030000
1	-2.301072000	-2.643722000	0.581297000
1	-2.881572000	-1.618242000	-0.711584000
6	-0.134702000	2.387952000	1.391534000
1	-0.854774000	2.801269000	0.697488000
1	-0.680289000	1.898705000	2.187459000
1	0.467197000	3.198377000	1.803101000
8	3.107925000	-0.941661000	-0.626554000
8	3.608038000	-1.313562000	-1.664096000

E/h = -447.80247538
ZPE/h = 0.48597172
H_{133}/h = 0.49353804
S_{133}/h = 0.02617912

[(D$_{12}$-TMC)(OTf)Fe^{IV}(O)]$^{+}$ (S = 2)

26	-3.051010000	0.829821000	1.707853000
7	-1.799833000	2.441388000	2.340625000
1	-1.219677000	3.936413000	-0.687159000
1	-2.589651000	4.270516000	0.311368000
7	-3.830035000	1.821976000	-0.104964000
6	-1.069728000	2.992433000	1.169151000
1	-0.459582000	3.824377000	1.528618000
7	-2.472106000	-0.117241000	3.614443000
6	-3.088583000	-2.115865000	1.131013000
1	-0.211001000	2.458853000	3.735272000
7	-3.944737000	-0.929087000	0.871600000
1	-2.172373000	-1.977379000	0.565111000
1	-3.603668000	-2.985256000	0.715427000
6	-2.659382000	2.411366000	-0.795149000
6	-4.430680000	0.732048000	-0.909173000
6	-0.827284000	1.729632000	3.204902000
1	-0.394481000	2.218104000	0.818016000
6	-1.919231000	3.469298000	0.007538000
1	-3.018192000	2.865274000	-1.723255000
1	-1.980262000	1.605154000	-1.061106000
6	-1.817559000	-1.399681000	3.268463000
1	-3.616674000	-2.599954000	3.172982000
6	-3.878794000	-0.631883000	-0.578849000
6	-1.505460000	0.833593000	4.214501000
1	-2.184261000	-3.342290000	2.555789000
6	-2.730054000	-2.397990000	2.575723000
1	-1.453048000	-1.845991000	4.197819000
1	-0.955616000	-1.185983000	2.641806000
1	-0.741142000	0.285408000	4.768789000
1	-2.839109000	-0.701829000	-0.881574000
1	-4.441548000	-1.388194000	-1.130064000
1	-0.176080000	1.149621000	2.559133000
1	-5.502677000	0.749385000	-0.736347000
1	-4.279277000	0.928408000	-1.972274000
1	-2.046980000	1.436593000	4.937317000
8	-1.736212000	0.314162000	0.957061000
8	-4.747934000	1.464282000	2.652806000
9	-7.658977000	1.178825000	2.310503000
16	-5.620743000	2.321254000	3.500448000
8	-6.002044000	3.565332000	2.874902000
8	-5.197647000	2.386629000	4.882176000
6	-7.171212000	1.323636000	3.536012000
9	-6.941180000	0.122128000	4.047639000
9	-8.073664000	1.940137000	4.282686000
6	-4.858769000	2.860944000	0.063436000
1	-5.105719000	3.287495000	-0.911381000
1	-5.749211000	2.426109000	0.497517000
1	-4.517447000	3.649849000	0.719090000
6	-3.522128000	-0.380137000	4.614573000
1	-4.332618000	-0.957275000	4.189552000
1	-3.093338000	-0.937313000	5.450621000
1	-3.926016000	0.553615000	4.978227000
6	-5.330878000	-1.201893000	1.273957000
1	-5.715750000	-2.050993000	0.707070000
1	-5.376715000	-1.434464000	2.331164000
1	-5.953732000	-0.335430000	1.088532000
6	-2.442802000	3.530447000	3.089932000
1	-3.200447000	4.013383000	2.483799000
1	-2.912948000	3.145950000	3.986696000
1	-1.691399000	4.269407000	3.372653000

E/h = -415.86724412
ZPE/h = 0.48086415
H_{133}/h = 0.48747342
S_{133}/h = 0.02395407

[(D_{12}-TMC)(OTf)Fe^{IV}(O)]$^+$ (S = 1)

26	-3.032212000	0.817993000	1.694105000
7	-1.845563000	2.412927000	2.303116000
1	-1.155218000	3.858130000	-0.727790000
1	-2.533112000	4.266606000	0.236593000
7	-3.765128000	1.780994000	-0.017553000
6	-1.070207000	2.972554000	1.163823000
1	-0.478412000	3.803893000	1.552051000
7	-2.498214000	-0.088229000	3.503580000
6	-3.078506000	-2.103272000	1.130820000
1	-0.279169000	2.496460000	3.726864000
7	-3.917841000	-0.894048000	0.915513000
1	-2.178579000	-1.970998000	0.539007000
1	-3.625311000	-2.954001000	0.719158000
6	-2.624269000	2.357476000	-0.772857000
6	-4.436018000	0.735811000	-0.835829000
6	-0.867682000	1.744568000	3.197817000
1	-0.382034000	2.199773000	0.836856000
6	-1.876094000	3.439738000	-0.024201000
1	-3.027497000	2.779541000	-1.695855000
1	-1.949848000	1.547908000	-1.038069000
6	-1.796884000	-1.364409000	3.214728000
1	-3.536707000	-2.646728000	3.173792000
6	-3.888286000	-0.624452000	-0.543114000
6	-1.571301000	0.858919000	4.179918000
1	-2.089794000	-3.321959000	2.508071000
6	-2.676445000	-2.403687000	2.554063000
1	-1.436263000	-1.759151000	4.166575000
1	-0.935192000	-1.146192000	2.590546000
1	-0.843483000	0.302468000	4.771216000
1	-2.856943000	-0.696688000	-0.871624000
1	-4.467568000	-1.385348000	-1.068909000
1	-0.193441000	1.164339000	2.576682000
1	-5.498091000	0.771764000	-0.614407000
1	-4.322527000	0.967399000	-1.895075000
1	-2.155353000	1.455401000	4.873455000
8	-1.714518000	0.303784000	0.949464000
8	-4.760714000	1.473954000	2.662357000
9	-7.677758000	1.183949000	2.330152000
16	-5.631889000	2.319198000	3.515373000
8	-6.021671000	3.569753000	2.904817000
8	-5.208303000	2.378137000	4.899162000
6	-7.184703000	1.326030000	3.554728000
9	-6.955919000	0.121782000	4.062446000
9	-8.085367000	1.940591000	4.305662000
6	-4.766632000	2.850470000	0.176775000
1	-4.998535000	3.290583000	-0.794205000
1	-5.667492000	2.434383000	0.602442000
1	-4.403214000	3.622779000	0.836532000
6	-3.576722000	-0.382676000	4.474071000
1	-4.373127000	-0.953513000	4.021275000
1	-3.153565000	-0.958616000	5.298496000
1	-3.988139000	0.537114000	4.859498000
6	-5.301933000	-1.170842000	1.333996000
1	-5.695330000	-2.003657000	0.750717000
1	-5.334620000	-1.435102000	2.382754000
1	-5.924289000	-0.299023000	1.180803000
6	-2.499708000	3.513288000	3.031381000
1	-3.241451000	3.996421000	2.408842000
1	-2.985995000	3.143005000	3.924456000
1	-1.748514000	4.249828000	3.317951000

E/h = -415.87450545
ZPE/h = 0.48372036
H_{133}/h = 0.48998377
S_{133}/h = 0.02341405

[(D$_{12}$-TMC)(F)Fe^{III}(O$_3$)]$^+$ (S = 3)

26	-0.263482000	-0.252624000	0.053223000
7	0.808464000	1.494095000	0.733553000
1	1.326880000	3.126628000	-2.229620000
1	-0.126664000	3.239723000	-1.305713000
7	-0.957888000	0.618919000	-1.835383000
6	1.514515000	2.183001000	-0.374835000
1	1.955544000	3.096972000	0.031909000
7	0.316659000	-1.097274000	1.986644000
6	0.066305000	-3.225398000	-0.481089000
1	2.283859000	1.707642000	2.233396000
7	-0.853349000	-2.125446000	-0.856186000
1	1.021244000	-3.021557000	-0.954796000
1	-0.325081000	-4.149663000	-0.914915000
6	0.133034000	1.417065000	-2.448134000
6	-1.343884000	-0.528724000	-2.701504000
6	1.802967000	0.910521000	1.660784000
1	2.328010000	1.541111000	-0.695427000
6	0.667193000	2.548050000	-1.581188000
1	-0.256968000	1.848361000	-3.374336000
1	0.938644000	0.736971000	-2.707323000
6	1.043401000	-2.372491000	1.777217000
1	-0.657102000	-3.684640000	1.506569000
6	-0.713557000	-1.847178000	-2.304985000
6	1.186515000	-0.075373000	2.630761000
1	0.881219000	-4.344404000	1.086198000
6	0.278043000	-3.438879000	1.007409000
1	1.292895000	-2.774094000	2.763414000
1	1.980459000	-2.151889000	1.277104000
1	1.985780000	-0.567662000	3.186483000
1	0.344846000	-1.844143000	-2.536549000
1	-1.187684000	-2.647644000	-2.878073000
1	2.572797000	0.430885000	1.069112000
1	-2.425939000	-0.613179000	-2.663980000
1	-1.081416000	-0.311260000	-3.737753000
1	0.581291000	0.457090000	3.358452000
8	1.460220000	-0.679647000	-0.965397000
9	-1.892940000	0.169862000	0.789434000
6	-2.149242000	1.473335000	-1.673143000
1	-2.452355000	1.860923000	-2.647775000
1	-2.955842000	0.893726000	-1.241729000
1	-1.944701000	2.305154000	-1.011681000
6	-0.831247000	-1.340317000	2.880572000
1	-1.525965000	-2.040717000	2.435265000
1	-0.471960000	-1.747847000	3.827672000
1	-1.359821000	-0.411514000	3.055804000
6	-2.242076000	-2.493921000	-0.544015000
1	-2.521059000	-3.386733000	-1.106754000
1	-2.350776000	-2.694318000	0.515399000
1	-2.915410000	-1.682700000	-0.795702000
6	-0.054946000	2.444740000	1.450663000
1	-0.778614000	2.880258000	0.771999000
1	-0.603514000	1.942183000	2.238883000
1	0.554672000	3.241375000	1.880691000
8	2.718508000	-1.071647000	-0.537833000
8	3.526492000	-1.186195000	-1.516495000

E/h = -335.12782443
ZPE/h = 0.45732826
H_{133}/h = 0.46337262
S_{133}/h = 0.02303520

$[(D_{12}\text{-TMC})(F)Fe^{III}(O_3)]^+$ (S = 2)

26	-0.256788000	-0.254067000	0.046906000
7	0.766725000	1.437571000	0.688891000
1	1.383278000	3.064135000	-2.260368000
1	-0.081519000	3.245766000	-1.360297000
7	-0.933730000	0.607486000	-1.760394000
6	1.505442000	2.147074000	-0.386118000
1	1.930460000	3.050782000	0.056730000
7	0.283155000	-1.080214000	1.915187000
6	0.087604000	-3.190433000	-0.480241000
1	2.242052000	1.707062000	2.179451000
7	-0.821079000	-2.066605000	-0.812240000
1	1.035072000	-2.988701000	-0.968043000
1	-0.333681000	-4.092909000	-0.929485000
6	0.145090000	1.378910000	-2.427902000
6	-1.377130000	-0.516599000	-2.634003000
6	1.765579000	0.888025000	1.636533000
1	2.326273000	1.508181000	-0.691419000
6	0.696220000	2.525959000	-1.606174000
1	-0.272768000	1.781179000	-3.353659000
1	0.940237000	0.686432000	-2.685747000
6	1.050978000	-2.337074000	1.743835000
1	-0.597314000	-3.714073000	1.503085000
6	-0.710056000	-1.810786000	-2.270496000
6	1.120746000	-0.058524000	2.606340000
1	0.959674000	-4.315028000	1.052935000
6	0.321650000	-3.432570000	0.993715000
1	1.297475000	-2.703293000	2.743248000
1	1.985598000	-2.099366000	1.247792000
1	1.885557000	-0.553556000	3.204881000
1	0.344730000	-1.778730000	-2.516555000
1	-1.171213000	-2.634236000	-2.819455000
1	2.533228000	0.385593000	1.061143000
1	-2.454411000	-0.604673000	-2.536080000
1	-1.170910000	-0.273421000	-3.676392000
1	0.481167000	0.484863000	3.294756000
8	1.559785000	-0.705814000	-0.997762000
9	-1.923764000	0.166671000	0.791129000
6	-2.105019000	1.492361000	-1.581007000
1	-2.400744000	1.880599000	-2.556671000
1	-2.918587000	0.931652000	-1.141401000
1	-1.875583000	2.320311000	-0.925958000
6	-0.873244000	-1.352206000	2.795845000
1	-1.541457000	-2.075466000	2.351119000
1	-0.503391000	-1.744578000	3.744391000
1	-1.428171000	-0.438474000	2.960099000
6	-2.209058000	-2.437211000	-0.480492000
1	-2.500547000	-3.301423000	-1.078345000
1	-2.291118000	-2.690496000	0.568144000
1	-2.881572000	-1.611606000	-0.673277000
6	-0.113795000	2.387940000	1.393950000
1	-0.794995000	2.856386000	0.696113000
1	-0.706138000	1.878104000	2.142614000
1	0.497125000	3.159117000	1.864237000
8	2.803555000	-1.095751000	-0.559304000
8	3.626301000	-1.254768000	-1.527486000

E/h = -335.11291378
ZPE/h = 0.45910772
H_{133}/h = 0.46497636
S_{133}/h = 0.02258257

$[(D_{12}\text{-TMC})(F)Fe^{III}(O_3)]^+$ TS O_2 extrusion (S = 3)

26	-0.211828000	-0.260719000	0.033618000
7	0.816730000	1.514687000	0.744222000
1	1.339228000	3.133076000	-2.224880000
1	-0.120507000	3.238382000	-1.309962000
7	-0.934760000	0.615713000	-1.840397000
6	1.523330000	2.196672000	-0.364734000
1	1.966735000	3.113838000	0.033542000
7	0.327401000	-1.092123000	1.996386000
6	0.067567000	-3.230372000	-0.477775000
1	2.306522000	1.706193000	2.233944000
7	-0.849273000	-2.133150000	-0.862211000
1	1.022508000	-3.026074000	-0.951157000
1	-0.324135000	-4.159818000	-0.901167000
6	0.159376000	1.413169000	-2.443794000
6	-1.316965000	-0.530144000	-2.708306000
6	1.810094000	0.917903000	1.661399000
1	2.332829000	1.546628000	-0.679795000
6	0.680465000	2.551755000	-1.577711000
1	-0.217587000	1.837733000	-3.378998000
1	0.970564000	0.731666000	-2.683599000
6	1.057995000	-2.359036000	1.771959000
1	-0.647003000	-3.668934000	1.518710000
6	-0.694467000	-1.851761000	-2.306770000
6	1.194625000	-0.067337000	2.633698000
1	0.886593000	-4.337193000	1.095779000
6	0.286257000	-3.429985000	1.012416000
1	1.331046000	-2.763855000	2.751242000
1	1.980818000	-2.128398000	1.249362000
1	1.996015000	-0.554126000	3.192277000
1	0.367589000	-1.845967000	-2.522764000
1	-1.161510000	-2.649169000	-2.890833000
1	2.565805000	0.430654000	1.057608000
1	-2.399601000	-0.612068000	-2.679173000
1	-1.046346000	-0.314833000	-3.743175000
1	0.589309000	0.467916000	3.359560000
8	1.407424000	-0.682431000	-0.846571000
9	-1.862440000	0.167875000	0.783816000
6	-2.124649000	1.467835000	-1.672900000
1	-2.425407000	1.868373000	-2.643516000
1	-2.932081000	0.883603000	-1.249642000
1	-1.921855000	2.289823000	-0.998811000
6	-0.816709000	-1.338323000	2.889099000
1	-1.512866000	-2.035895000	2.441402000
1	-0.460482000	-1.748446000	3.836991000
1	-1.345928000	-0.409768000	3.064119000
6	-2.238906000	-2.493716000	-0.557548000
1	-2.525908000	-3.379884000	-1.127734000
1	-2.350390000	-2.701742000	0.500334000
1	-2.905183000	-1.674320000	-0.801881000
6	-0.042767000	2.461788000	1.464233000
1	-0.756355000	2.911680000	0.783595000
1	-0.605166000	1.951744000	2.237996000
1	0.565862000	3.249915000	1.912539000
8	3.058895000	-1.111343000	-0.565215000
8	3.618418000	-1.328382000	-1.614233000

E/h = -335.11171672
ZPE/h = 0.45477671
H_{133}/h = 0.46092149
S_{133}/h = 0.02329047

[(D_{12}-TMC)(F)Fe^{III}(O_3)]$^+$ TS O_2 extrusion (S = 2)

26	-0.119986000	-0.279893000	-0.015884000
7	0.800074000	1.399266000	0.698683000
1	1.273620000	3.204917000	-2.178680000
1	-0.185259000	3.231367000	-1.255732000
7	-0.953712000	0.644692000	-1.916322000
6	1.478088000	2.161162000	-0.385080000
1	1.895519000	3.060847000	0.073359000
7	0.379226000	-1.124629000	2.045568000
6	0.086118000	-3.160786000	-0.456019000
1	2.311366000	1.697143000	2.142745000
7	-0.789003000	-2.023779000	-0.842169000
1	1.055139000	-2.982247000	-0.910284000
1	-0.330492000	-4.057830000	-0.921110000
6	0.108538000	1.482347000	-2.498018000
6	-1.288463000	-0.514935000	-2.769394000
6	1.835413000	0.866194000	1.616828000
1	2.303812000	1.552384000	-0.735609000
6	0.623346000	2.574254000	-1.570177000
1	-0.277463000	1.956877000	-3.407365000
1	0.930244000	0.832760000	-2.788193000
6	1.048893000	-2.420006000	1.852676000
1	-0.701058000	-3.642319000	1.498330000
6	-0.662237000	-1.815621000	-2.307124000
6	1.275613000	-0.108064000	2.633915000
1	0.811602000	-4.364090000	1.086760000
6	0.256900000	-3.426165000	1.029831000
1	1.250033000	-2.863772000	2.834370000
1	2.014524000	-2.243516000	1.390536000
1	2.112007000	-0.582477000	3.151868000
1	0.395045000	-1.830733000	-2.542930000
1	-1.140811000	-2.649072000	-2.826011000
1	2.595360000	0.391036000	1.008381000
1	-2.370212000	-0.616825000	-2.785094000
1	-0.982051000	-0.333747000	-3.801851000
1	0.717432000	0.437608000	3.389915000
8	1.424824000	-0.576377000	-0.985145000
9	-1.726447000	0.129110000	0.715516000
6	-2.168849000	1.445644000	-1.733929000
1	-2.514364000	1.829241000	-2.698484000
1	-2.945747000	0.836906000	-1.286704000
1	-1.988375000	2.283483000	-1.072302000
6	-0.771197000	-1.303667000	2.938255000
1	-1.475573000	-2.015197000	2.525156000
1	-0.435868000	-1.670380000	3.912838000
1	-1.292181000	-0.362088000	3.063656000
6	-2.188008000	-2.330705000	-0.503886000
1	-2.486605000	-3.245369000	-1.017390000
1	-2.296527000	-2.463397000	0.564512000
1	-2.834086000	-1.515133000	-0.802556000
6	-0.108796000	2.292315000	1.434953000
1	-0.855690000	2.698218000	0.765070000
1	-0.624062000	1.749075000	2.216094000
1	0.469172000	3.108057000	1.870771000
8	2.856155000	-1.343416000	-0.467638000
8	3.683986000	-1.249360000	-1.344908000

E/h = -335.09648911
ZPE/h = 0.45613419
H_{133}/h = 0.46189709
S_{133}/h = 0.02233896

[(D$_{12}$-TMC)(F)Fe^{IV}(O)]$^+$ (S = 2)

26	-0.180024000	-0.277842000	0.003679000
7	0.799931000	1.481415000	0.725117000
1	1.360500000	3.089478000	-2.244939000
1	-0.103072000	3.226519000	-1.338690000
7	-0.954808000	0.620597000	-1.839962000
6	1.515941000	2.155821000	-0.382446000
1	1.974930000	3.062541000	0.021371000
7	0.326770000	-1.105205000	1.970780000
6	0.058970000	-3.220833000	-0.484096000
1	2.298239000	1.688605000	2.202715000
7	-0.862587000	-2.118417000	-0.846365000
1	1.008221000	-3.012351000	-0.968605000
1	-0.339723000	-4.143197000	-0.915799000
6	0.150271000	1.386519000	-2.452518000
6	-1.354912000	-0.525655000	-2.689560000
6	1.799576000	0.894076000	1.642110000
1	2.316405000	1.491208000	-0.693958000
6	0.687043000	2.524755000	-1.598373000
1	-0.211250000	1.802672000	-3.397978000
1	0.952114000	0.687437000	-2.679000000
6	1.075762000	-2.358083000	1.742849000
1	-0.615673000	-3.690959000	1.518709000
6	-0.712329000	-1.835205000	-2.289365000
6	1.179179000	-0.080100000	2.617944000
1	0.917942000	-4.337138000	1.059373000
6	0.305039000	-3.436678000	0.997331000
1	1.372350000	-2.755800000	2.718327000
1	1.981194000	-2.107180000	1.194896000
1	1.975690000	-0.562497000	3.187188000
1	0.350392000	-1.813493000	-2.507525000
1	-1.163077000	-2.642251000	-2.872144000
1	2.543055000	0.390475000	1.032840000
1	-2.435801000	-0.615512000	-2.633208000
1	-1.110465000	-0.318977000	-3.733021000
1	0.564652000	0.458357000	3.333654000
8	1.251661000	-0.654745000	-0.650615000
9	-1.815793000	0.153141000	0.744864000
6	-2.130212000	1.486511000	-1.668155000
1	-2.439327000	1.880622000	-2.639237000
1	-2.938263000	0.914907000	-1.229052000
1	-1.912633000	2.313794000	-1.005726000
6	-0.824274000	-1.361029000	2.848622000
1	-1.514836000	-2.056821000	2.390797000
1	-0.476876000	-1.775806000	3.798046000
1	-1.358015000	-0.435178000	3.023878000
6	-2.249067000	-2.475442000	-0.529378000
1	-2.552720000	-3.340686000	-1.122131000
1	-2.341599000	-2.718010000	0.522572000
1	-2.910143000	-1.641553000	-0.733678000
6	-0.065893000	2.426731000	1.437764000
1	-0.768435000	2.881789000	0.750059000
1	-0.639302000	1.913465000	2.200630000
1	0.540526000	3.210150000	1.896535000

E/h = -303.16339478
ZPE/h = 0.45206368
H_{133}/h = 0.45697746
S_{133}/h = 0.02068514

[(D$_{12}$-TMC)(F)Fe^{IV}(O)]$^+$ (S = 1)

26	-0.164670000	-0.283045000	-0.002524000
7	0.759706000	1.439574000	0.682917000
1	1.413397000	3.034057000	-2.276594000
1	-0.063974000	3.229661000	-1.398502000
7	-0.904351000	0.589589000	-1.750433000
6	1.507434000	2.138272000	-0.389072000
1	1.943467000	3.040822000	0.046408000
7	0.303758000	-1.083151000	1.871079000
6	0.065765000	-3.203255000	-0.480118000
1	2.246937000	1.705848000	2.166235000
7	-0.836637000	-2.071876000	-0.801443000
1	1.007209000	-3.006870000	-0.983525000
1	-0.367752000	-4.105990000	-0.917825000
6	0.175603000	1.343717000	-2.425896000
6	-1.364639000	-0.523591000	-2.622632000
6	1.766564000	0.891631000	1.619538000
1	2.321878000	1.483140000	-0.682416000
6	0.717484000	2.506733000	-1.622647000
1	-0.225421000	1.723630000	-3.368896000
1	0.974895000	0.642595000	-2.652504000
6	1.085689000	-2.328171000	1.699624000
1	-0.560518000	-3.717553000	1.525246000
6	-0.711158000	-1.821453000	-2.254572000
6	1.125739000	-0.064976000	2.579013000
1	0.984693000	-4.317868000	1.032947000
6	0.342128000	-3.437670000	0.986305000
1	1.371713000	-2.678944000	2.694304000
1	1.991694000	-2.075881000	1.154557000
1	1.889878000	-0.558054000	3.180174000
1	0.347154000	-1.791805000	-2.490819000
1	-1.167138000	-2.641796000	-2.812834000
1	2.519455000	0.382905000	1.027126000
1	-2.442317000	-0.604094000	-2.520943000
1	-1.160074000	-0.285653000	-3.666685000
1	0.477702000	0.470438000	3.266066000
8	1.273371000	-0.660572000	-0.655369000
9	-1.814089000	0.146801000	0.742773000
6	-2.064739000	1.482596000	-1.562225000
1	-2.359095000	1.884980000	-2.533130000
1	-2.882812000	0.924708000	-1.127718000
1	-1.829746000	2.299316000	-0.896116000
6	-0.856961000	-1.364159000	2.739130000
1	-1.530048000	-2.071783000	2.278579000
1	-0.497773000	-1.774972000	3.684506000
1	-1.406769000	-0.449703000	2.915301000
6	-2.225462000	-2.428495000	-0.475053000
1	-2.548118000	-3.253784000	-1.111386000
1	-2.299765000	-2.737619000	0.559390000
1	-2.878670000	-1.576208000	-0.611246000
6	-0.114702000	2.388103000	1.389068000
1	-0.773358000	2.884383000	0.688379000
1	-0.732040000	1.869150000	2.111005000
1	0.494725000	3.141283000	1.890577000

E/h = -303.16897511
ZPE/h = 0.45477803
H_{133}/h = 0.45933651
S_{133}/h = 0.01982693

[(D_{12}-TMC)(I)Fe^{III}(O_3)]$^+$ (S = 3)

26	-0.358153000	-0.236363000	0.106553000
7	0.792700000	1.503340000	0.751608000
1	1.480628000	2.969428000	-2.271594000
1	0.003398000	3.193623000	-1.405576000
7	-0.981365000	0.646041000	-1.851267000
6	1.559069000	2.109108000	-0.372365000
1	2.019440000	3.023390000	0.009450000
7	0.285630000	-1.132645000	2.031298000
6	0.137963000	-3.206588000	-0.525521000
1	2.223216000	1.694701000	2.296391000
7	-0.853150000	-2.149205000	-0.861380000
1	1.063064000	-2.960927000	-1.033112000
1	-0.229885000	-4.144327000	-0.948847000
6	0.208965000	1.309070000	-2.450200000
6	-1.416084000	-0.524861000	-2.658615000
6	1.745997000	0.901269000	1.716507000
1	2.364016000	1.434649000	-0.633732000
6	0.771307000	2.454007000	-1.622294000
1	-0.099599000	1.705479000	-3.420793000
1	0.972104000	0.558860000	-2.622658000
6	1.131297000	-2.311896000	1.707866000
1	-0.482932000	-3.732316000	1.477534000
6	-0.733090000	-1.824239000	-2.306128000
6	1.078725000	-0.053541000	2.677822000
1	1.080511000	-4.284373000	0.994491000
6	0.416111000	-3.420275000	0.950093000
1	1.487278000	-2.720013000	2.657146000
1	2.004296000	-1.980367000	1.160006000
1	1.841204000	-0.497047000	3.321437000
1	0.320479000	-1.782926000	-2.553093000
1	-1.187980000	-2.624577000	-2.894009000
1	2.528993000	0.401928000	1.161283000
1	-2.487843000	-0.626465000	-2.513738000
1	-1.256572000	-0.314674000	-3.718244000
1	0.399185000	0.495130000	3.323382000
8	1.351727000	-0.727717000	-0.858989000
53	-2.765423000	0.386791000	1.182274000
6	-2.090802000	1.621393000	-1.897681000
1	-2.200146000	1.984959000	-2.921330000
1	-3.012836000	1.149792000	-1.585934000
1	-1.901733000	2.460746000	-1.240304000
6	-0.718566000	-1.573066000	3.022268000
1	-1.418104000	-2.273455000	2.584005000
1	-0.204868000	-2.052310000	3.857779000
1	-1.278186000	-0.722244000	3.386302000
6	-2.206355000	-2.663176000	-0.586423000
1	-2.369720000	-3.576474000	-1.161144000
1	-2.318341000	-2.880691000	0.469973000
1	-2.956426000	-1.929732000	-0.856152000
6	0.023144000	2.564854000	1.424489000
1	-0.718689000	2.978338000	0.750482000
1	-0.495953000	2.168951000	2.288748000
1	0.704564000	3.356125000	1.741050000
8	2.710130000	-0.845731000	-0.595371000
8	3.348090000	-1.201234000	-1.637303000

E/h = -322.20165946
ZPE/h = 0.45718046
H_{133}/h = 0.46286907
S_{133}/h = 0.02348214

[(D$_{12}$-TMC)(I)Fe^{III}(O$_3$)]$^+$ (S = 2)

26	-0.328035000	-0.248084000	0.098463000
7	0.806568000	1.558659000	0.743806000
1	1.498120000	2.950943000	-2.305992000
1	0.032474000	3.218449000	-1.432679000
7	-0.966004000	0.641660000	-1.781283000
6	1.580917000	2.145417000	-0.377082000
1	2.045145000	3.067333000	-0.015806000
7	0.266470000	-1.108655000	1.964918000
6	0.163562000	-3.221152000	-0.552148000
1	2.204568000	1.716421000	2.326872000
7	-0.858624000	-2.198051000	-0.870162000
1	1.080218000	-2.942010000	-1.059110000
1	-0.164174000	-4.174090000	-0.977143000
6	0.209744000	1.301849000	-2.420744000
6	-1.439407000	-0.515648000	-2.592982000
6	1.735108000	0.942450000	1.713393000
1	2.382935000	1.462120000	-0.627864000
6	0.790832000	2.464228000	-1.632487000
1	-0.136001000	1.680344000	-3.385158000
1	0.969347000	0.554739000	-2.609867000
6	1.128144000	-2.290145000	1.674701000
1	-0.445328000	-3.756121000	1.451656000
6	-0.759627000	-1.831573000	-2.299901000
6	1.039337000	-0.025566000	2.639698000
1	1.137138000	-4.265324000	0.980688000
6	0.446191000	-3.422964000	0.924154000
1	1.461816000	-2.674641000	2.640907000
1	2.012364000	-1.960548000	1.149451000
1	1.777017000	-0.479803000	3.303484000
1	0.288542000	-1.789915000	-2.568935000
1	-1.234622000	-2.601050000	-2.914165000
1	2.529644000	0.448169000	1.169657000
1	-2.505298000	-0.611279000	-2.408034000
1	-1.318395000	-0.276972000	-3.651311000
1	0.335988000	0.514290000	3.266472000
8	1.327525000	-0.651337000	-0.923751000
53	-2.699814000	0.339771000	1.139635000
6	-2.061975000	1.639397000	-1.812915000
1	-2.144204000	2.030995000	-2.827868000
1	-2.998741000	1.178360000	-1.535789000
1	-1.867853000	2.456986000	-1.131167000
6	-0.738700000	-1.568764000	2.953811000
1	-1.426923000	-2.276199000	2.510902000
1	-0.211654000	-2.048214000	3.779778000
1	-1.304945000	-0.730856000	3.333251000
6	-2.192931000	-2.747604000	-0.595881000
1	-2.341848000	-3.662479000	-1.174025000
1	-2.301124000	-2.974418000	0.459264000
1	-2.962863000	-2.031741000	-0.859413000
6	0.039182000	2.622975000	1.404871000
1	-0.691251000	3.043697000	0.722158000
1	-0.497101000	2.232425000	2.261994000
1	0.715959000	3.414321000	1.734782000
8	2.632908000	-0.913201000	-0.522210000
8	3.270108000	-1.529074000	-1.436453000

E/h = -322.18145974
ZPE/h = 0.45752467
H_{133}/h = 0.46353464
S_{133}/h = 0.02313749

[(D$_{12}$-TMC)(I)Fe^{III}(O$_3$)]$^+$ TS O_2 extrusion (S = 3)

26	-0.334080000	-0.223696000	0.100563000
7	0.785097000	1.535983000	0.781889000
1	1.475777000	2.986027000	-2.247308000
1	-0.006857000	3.202868000	-1.388279000
7	-0.983871000	0.660622000	-1.841116000
6	1.551323000	2.132081000	-0.343903000
1	2.018087000	3.047462000	0.028567000
7	0.266803000	-1.117496000	2.053600000
6	0.103972000	-3.196958000	-0.500498000
1	2.238932000	1.688011000	2.310337000
7	-0.877700000	-2.135565000	-0.845943000
1	1.032064000	-2.957444000	-1.005629000
1	-0.267737000	-4.135749000	-0.919069000
6	0.215393000	1.315762000	-2.425537000
6	-1.414119000	-0.509519000	-2.649468000
6	1.736728000	0.909579000	1.730492000
1	2.349970000	1.448546000	-0.602231000
6	0.767329000	2.468022000	-1.598980000
1	-0.073119000	1.703592000	-3.406170000
1	0.980032000	0.560997000	-2.575093000
6	1.106658000	-2.298365000	1.730113000
1	-0.519473000	-3.706502000	1.505506000
6	-0.740169000	-1.811490000	-2.287057000
6	1.065833000	-0.041380000	2.693983000
1	1.038338000	-4.275083000	1.024217000
6	0.382240000	-3.404632000	0.976475000
1	1.467565000	-2.708080000	2.677408000
1	1.971902000	-1.966668000	1.168481000
1	1.828293000	-0.485466000	3.337968000
1	0.317495000	-1.770386000	-2.518559000
1	-1.187901000	-2.610055000	-2.883475000
1	2.499077000	0.395612000	1.159145000
1	-2.487597000	-0.607665000	-2.515147000
1	-1.243927000	-0.303707000	-3.708561000
1	0.391359000	0.513493000	3.339828000
8	1.261337000	-0.699440000	-0.699683000
53	-2.792453000	0.434013000	1.203235000
6	-2.089163000	1.638023000	-1.893031000
1	-2.191159000	2.006776000	-2.916018000
1	-3.013717000	1.166971000	-1.587746000
1	-1.904241000	2.473201000	-1.229322000
6	-0.740714000	-1.547919000	3.042134000
1	-1.444797000	-2.243595000	2.603139000
1	-0.235779000	-2.028843000	3.882693000
1	-1.297263000	-0.692052000	3.399418000
6	-2.235809000	-2.633080000	-0.576411000
1	-2.411905000	-3.542604000	-1.154149000
1	-2.351123000	-2.851512000	0.479600000
1	-2.975530000	-1.887742000	-0.842310000
6	0.031773000	2.598954000	1.463779000
1	-0.698861000	3.032069000	0.789292000
1	-0.502358000	2.198544000	2.316944000
1	0.720872000	3.377160000	1.797442000
8	2.944306000	-0.824330000	-0.739821000
8	3.298891000	-1.358501000	-1.764403000

E/h = -322.18940532
ZPE/h = 0.45487529
H_{133}/h = 0.46107333
S_{133}/h = 0.02368529

$[(D_{12}\text{-TMC})(I)Fe^{III}(O_3)]^+$ TS O_2 extrusion (S = 2)

26	-0.279352000	-0.241507000	0.069174000
7	0.744422000	1.471007000	0.723553000
1	1.557214000	2.901818000	-2.287720000
1	0.067615000	3.210183000	-1.465842000
7	-0.941558000	0.642698000	-1.759016000
6	1.551309000	2.089669000	-0.364680000
1	1.995880000	2.997525000	0.047953000
7	0.246111000	-1.102298000	1.961703000
6	0.129914000	-3.162979000	-0.502664000
1	2.163923000	1.713069000	2.271888000
7	-0.845721000	-2.076746000	-0.795445000
1	1.044089000	-2.921909000	-1.029994000
1	-0.277092000	-4.080074000	-0.933201000
6	0.233010000	1.277246000	-2.418269000
6	-1.449689000	-0.493511000	-2.576315000
6	1.695755000	0.901031000	1.712217000
1	2.357167000	1.408129000	-0.601197000
6	0.814147000	2.438064000	-1.637629000
1	-0.108748000	1.644623000	-3.387972000
1	0.986091000	0.516026000	-2.584305000
6	1.127812000	-2.267942000	1.693857000
1	-0.430299000	-3.751062000	1.499955000
6	-0.763968000	-1.786105000	-2.251533000
6	1.000841000	-0.028036000	2.661294000
1	1.142024000	-4.240517000	0.972365000
6	0.444779000	-3.401902000	0.956190000
1	1.469992000	-2.638233000	2.662487000
1	1.996858000	-1.928070000	1.145470000
1	1.727858000	-0.474960000	3.341362000
1	0.284752000	-1.743461000	-2.520238000
1	-1.230969000	-2.600771000	-2.807955000
1	2.477464000	0.384375000	1.170085000
1	-2.515361000	-0.575342000	-2.386251000
1	-1.328830000	-0.262221000	-3.635625000
1	0.291017000	0.523035000	3.270355000
8	1.340996000	-0.704787000	-0.752244000
53	-2.928456000	0.453236000	1.251525000
6	-2.021104000	1.657776000	-1.750879000
1	-2.116832000	2.065455000	-2.758230000
1	-2.954198000	1.202749000	-1.454110000
1	-1.805029000	2.460771000	-1.060808000
6	-0.794948000	-1.560312000	2.910161000
1	-1.483488000	-2.247322000	2.439332000
1	-0.305475000	-2.059467000	3.747924000
1	-1.360914000	-0.715372000	3.273625000
6	-2.199816000	-2.565948000	-0.472209000
1	-2.389491000	-3.477957000	-1.039196000
1	-2.279253000	-2.781608000	0.585868000
1	-2.946493000	-1.822890000	-0.718031000
6	-0.055121000	2.526550000	1.373630000
1	-0.762028000	2.951596000	0.672136000
1	-0.614255000	2.123656000	2.207496000
1	0.616130000	3.310751000	1.724966000
8	3.027189000	-0.890638000	-0.696378000
8	3.434300000	-1.339885000	-1.745904000

E/h = -322.17126701
ZPE/h = 0.45628823
H_{133}/h = 0.46244350
S_{133}/h = 0.02352052

[(D$_{12}$-TMC)(I)Fe^{IV}(O)]$^+$ (S = 2)

26	-3.138900000	0.856082000	1.750622000
7	-1.809375000	2.469287000	2.358400000
1	-1.125439000	3.806709000	-0.726683000
1	-2.495104000	4.246194000	0.230654000
7	-3.854509000	1.866792000	-0.104202000
6	-1.047939000	2.942940000	1.170919000
1	-0.409547000	3.766377000	1.501129000
7	-2.500811000	-0.131406000	3.647089000
6	-3.046215000	-2.085412000	1.090606000
1	-0.240397000	2.446087000	3.775172000
7	-3.981509000	-0.950029000	0.868771000
1	-2.146096000	-1.879072000	0.520974000
1	-3.509486000	-2.976578000	0.659515000
6	-2.633100000	2.343946000	-0.794958000
6	-4.508443000	0.769856000	-0.852590000
6	-0.870700000	1.733138000	3.238813000
1	-0.397517000	2.132121000	0.859823000
6	-1.857670000	3.402629000	-0.025798000
1	-2.941119000	2.765396000	-1.756050000
1	-1.993506000	1.490168000	-0.997578000
6	-1.782277000	-1.360642000	3.235917000
1	-3.526985000	-2.640148000	3.124279000
6	-3.936361000	-0.597208000	-0.570213000
6	-1.579489000	0.861043000	4.245660000
1	-2.067248000	-3.299525000	2.476646000
6	-2.656158000	-2.382327000	2.525055000
1	-1.369976000	-1.820330000	4.138565000
1	-0.949925000	-1.078390000	2.598157000
1	-0.832559000	0.348035000	4.856502000
1	-2.901469000	-0.646622000	-0.892249000
1	-4.499250000	-1.337480000	-1.143101000
1	-0.225139000	1.131597000	2.607727000
1	-5.562843000	0.785959000	-0.590403000
1	-4.442177000	0.966261000	-1.925513000
1	-2.168311000	1.480557000	4.916656000
8	-1.810102000	0.338921000	0.995031000
53	-5.374802000	1.728844000	3.025121000
6	-4.810516000	2.988422000	-0.060115000
1	-4.936633000	3.391755000	-1.067506000
1	-5.766825000	2.644567000	0.310118000
1	-4.461269000	3.774768000	0.597066000
6	-3.467870000	-0.507423000	4.694920000
1	-4.265535000	-1.118037000	4.290772000
1	-2.948805000	-1.065637000	5.477665000
1	-3.912683000	0.380356000	5.123683000
6	-5.341482000	-1.357834000	1.246925000
1	-5.627115000	-2.244997000	0.678460000
1	-5.382439000	-1.583474000	2.306939000
1	-6.046030000	-0.559284000	1.047962000
6	-2.369753000	3.624509000	3.073537000
1	-3.095244000	4.135923000	2.450656000
1	-2.874530000	3.302245000	3.976271000
1	-1.566093000	4.316297000	3.333245000

E/h = -290.24168613
ZPE/h = 0.45132381
H_{133}/h = 0.45647095
S_{133}/h = 0.02148406

[(D$_{12}$-TMC)(I)Fe^{IV}(O)]$^{+}$ (S = 1)

26	-3.113644000	0.845958000	1.737334000
7	-1.858354000	2.435893000	2.317734000
1	-1.039433000	3.679311000	-0.777293000
1	-2.405915000	4.228841000	0.130516000
7	-3.796739000	1.831304000	-0.006945000
6	-1.042297000	2.906291000	1.162825000
1	-0.422943000	3.732270000	1.518908000
7	-2.534893000	-0.104989000	3.537382000
6	-3.018761000	-2.062596000	1.079397000
1	-0.335526000	2.495887000	3.788574000
7	-3.948989000	-0.910313000	0.918456000
1	-2.140358000	-1.852319000	0.479220000
1	-3.511032000	-2.937728000	0.649976000
6	-2.604258000	2.275415000	-0.774665000
6	-4.539005000	0.786654000	-0.754848000
6	-0.924048000	1.752628000	3.247566000
1	-0.378979000	2.094005000	0.886946000
6	-1.799413000	3.347874000	-0.068227000
1	-2.961295000	2.671144000	-1.727757000
1	-1.981436000	1.411469000	-0.981157000
6	-1.752240000	-1.317283000	3.181989000
1	-3.411990000	-2.700155000	3.106915000
6	-3.970297000	-0.579714000	-0.528293000
6	-1.669813000	0.890647000	4.218842000
1	-1.934724000	-3.261228000	2.402923000
6	-2.575701000	-2.382655000	2.487879000
1	-1.350812000	-1.728556000	4.110320000
1	-0.916673000	-1.021365000	2.556573000
1	-0.969090000	0.375572000	4.878535000
1	-2.952394000	-0.633916000	-0.899637000
1	-4.563245000	-1.322095000	-1.065427000
1	-0.239931000	1.153916000	2.655782000
1	-5.571951000	0.821045000	-0.421072000
1	-4.533816000	1.024993000	-1.820077000
1	-2.312384000	1.502974000	4.844542000
8	-1.787084000	0.330851000	0.988771000
53	-5.423656000	1.743611000	3.049451000
6	-4.704232000	3.000668000	0.067433000
1	-4.798253000	3.429314000	-0.931365000
1	-5.677980000	2.693021000	0.418335000
1	-4.327054000	3.753883000	0.743686000
6	-3.533388000	-0.532205000	4.545660000
1	-4.319935000	-1.123366000	4.099927000
1	-3.024577000	-1.125542000	5.306954000
1	-3.984894000	0.331968000	5.009553000
6	-5.296606000	-1.332236000	1.337498000
1	-5.590654000	-2.211856000	0.763550000
1	-5.303498000	-1.580913000	2.391430000
1	-6.013045000	-0.537451000	1.174966000
6	-2.440976000	3.607763000	2.993395000
1	-3.139382000	4.112767000	2.338234000
1	-2.976060000	3.307192000	3.884978000
1	-1.641617000	4.299281000	3.262875000

E/h = -290.24156831
ZPE/h = 0.45408082
H_{133}/h = 0.45893213
S_{133}/h = 0.02075961

[(D$_{12}$-TMC)(I)Fe^{II}]$^{+}$ (*S* = 2)

26	-0.733409000	-0.143426000	0.259247000
7	0.758653000	1.383352000	0.728778000
1	1.735592000	2.557105000	-2.327035000
1	0.344508000	3.099667000	-1.450698000
7	-1.106428000	0.746379000	-1.805665000
6	1.635995000	1.704283000	-0.417597000
1	2.369789000	2.444515000	-0.083461000
7	0.160407000	-1.168123000	2.084951000
6	0.381046000	-2.875151000	-0.631842000
1	2.065304000	1.649915000	2.368566000
7	-0.808343000	-2.022802000	-0.848928000
1	1.242620000	-2.327355000	-1.014904000
1	0.267677000	-3.773822000	-1.246265000
6	0.159603000	1.172536000	-2.429850000
6	-1.653686000	-0.412473000	-2.530458000
6	1.601109000	0.833404000	1.810561000
1	2.184194000	0.795209000	-0.667909000
6	0.942613000	2.215375000	-1.660642000
1	-0.060963000	1.549571000	-3.434266000
1	0.796766000	0.299060000	-2.551066000
6	1.168821000	-2.168515000	1.690238000
1	-0.193940000	-3.795001000	1.241757000
6	-0.867489000	-1.674414000	-2.283858000
6	0.815632000	-0.034696000	2.759266000
1	1.449837000	-4.027394000	0.751230000
6	0.656502000	-3.280425000	0.799292000
1	1.603641000	-2.599268000	2.598646000
1	1.974117000	-1.660780000	1.163208000
1	1.483853000	-0.398628000	3.546446000
1	0.150794000	-1.568639000	-2.646643000
1	-1.316954000	-2.497001000	-2.845108000
1	2.409670000	0.268976000	1.355062000
1	-2.685185000	-0.541562000	-2.207332000
1	-1.676680000	-0.209399000	-3.606014000
1	0.035492000	0.548212000	3.245818000
53	-3.150431000	0.493298000	1.365813000
6	-2.085014000	1.834597000	-1.908556000
1	-2.234438000	2.106126000	-2.957617000
1	-3.029136000	1.516965000	-1.480034000
1	-1.747897000	2.712529000	-1.369040000
6	-0.766846000	-1.773049000	3.048308000
1	-1.295132000	-2.610457000	2.606915000
1	-0.216227000	-2.130167000	3.923144000
1	-1.499884000	-1.037208000	3.359414000
6	-2.033052000	-2.756415000	-0.486903000
1	-2.046080000	-3.726331000	-0.989540000
1	-2.091635000	-2.907561000	0.584648000
1	-2.906275000	-2.187065000	-0.788093000
6	0.093387000	2.602843000	1.216523000
1	-0.637950000	2.958679000	0.500261000
1	-0.430264000	2.393068000	2.143544000
1	0.835790000	3.384888000	1.392369000

E/h = -274.29909771
ZPE/h = 0.44579677
H_{133}/h = 0.45139932
S_{133}/h = 0.02239061

[(D$_{12}$-TMC)(IO)Fe^{II}]$^{+}$, hyperiodito-*I* (*S* = 1)

26	-0.611300000	-0.191199000	0.148663000
7	0.676830000	1.394932000	0.723555000
1	1.752400000	2.546318000	-2.305750000
1	0.309232000	3.087494000	-1.514354000
7	-1.034667000	0.651464000	-1.741033000
6	1.605412000	1.801363000	-0.351976000
1	2.231228000	2.615029000	0.025458000
7	0.036498000	-1.094951000	1.934664000
6	0.324085000	-2.960265000	-0.633063000
1	1.893792000	1.711698000	2.427223000
7	-0.817659000	-2.047998000	-0.868863000
1	1.206827000	-2.486173000	-1.062371000
1	0.144234000	-3.881864000	-1.193896000
6	0.236662000	1.096106000	-2.363450000
6	-1.592073000	-0.456567000	-2.548540000
6	1.486734000	0.880880000	1.847280000
1	2.260814000	0.953233000	-0.551227000
6	0.949465000	2.217968000	-1.644953000
1	0.016023000	1.402344000	-3.389352000
1	0.908447000	0.241302000	-2.417783000
6	1.080151000	-2.098765000	1.622740000
1	-0.230531000	-3.792299000	1.285794000
6	-0.836696000	-1.728013000	-2.311807000
6	0.642936000	0.004869000	2.720300000
1	1.422433000	-4.006770000	0.810333000
6	0.606301000	-3.283497000	0.812576000
1	1.502400000	-2.447564000	2.568857000
1	1.882067000	-1.608304000	1.072902000
1	1.239312000	-0.408059000	3.537526000
1	0.189487000	-1.638496000	-2.655950000
1	-1.290924000	-2.546862000	-2.873551000
1	2.332182000	0.334806000	1.438424000
1	-2.635183000	-0.576859000	-2.265976000
1	-1.572579000	-0.193389000	-3.608810000
1	-0.179854000	0.572732000	3.150126000
53	-3.104077000	0.574455000	1.305809000
6	-2.007554000	1.755756000	-1.832375000
1	-2.058046000	2.110081000	-2.864049000
1	-2.989552000	1.404571000	-1.537303000
1	-1.735222000	2.582341000	-1.190887000
6	-0.963550000	-1.718530000	2.826551000
1	-1.531656000	-2.483287000	2.315600000
1	-0.448155000	-2.178191000	3.672894000
1	-1.637197000	-0.949078000	3.192861000
6	-2.080488000	-2.728116000	-0.530282000
1	-2.143254000	-3.675629000	-1.069317000
1	-2.144989000	-2.921267000	0.531744000
1	-2.925163000	-2.106792000	-0.808852000
6	-0.081214000	2.565949000	1.203711000
1	-0.709894000	2.964333000	0.418672000
1	-0.719121000	2.281042000	2.036673000
1	0.616219000	3.343406000	1.522719000
8	-2.499127000	1.079262000	3.105473000

E/h = -290.22014068
ZPE/h = 0.45024121
H_{133}/h = 0.45587139
S_{133}/h = 0.02223348

[(D$_{12}$-TMC)(IO)Fe^{II}]$^{+}$, hyperiodito-*O* (*S* = 2)

26	-0.784247000	-0.124788000	0.275972000
7	0.802525000	1.369442000	0.697640000
1	1.622616000	2.709124000	-2.330274000
1	0.235194000	3.135213000	-1.388168000
7	-1.091560000	0.703637000	-1.821313000
6	1.626654000	1.761070000	-0.459400000
1	2.350385000	2.514284000	-0.128302000
7	0.193003000	-1.149821000	2.067281000
6	0.421216000	-2.903464000	-0.594101000
1	2.230699000	1.573827000	2.244998000
7	-0.737588000	-2.031913000	-0.866041000
1	1.312895000	-2.371009000	-0.927264000
1	0.327534000	-3.799376000	-1.217732000
6	0.116722000	1.253914000	-2.457323000
6	-1.554913000	-0.461079000	-2.587636000
6	1.684951000	0.784313000	1.719750000
1	2.195211000	0.882075000	-0.765676000
6	0.870821000	2.294214000	-1.657314000
1	-0.169372000	1.679362000	-3.426087000
1	0.797986000	0.430615000	-2.663564000
6	1.136169000	-2.237175000	1.762066000
1	-0.286603000	-3.788562000	1.250531000
6	-0.725708000	-1.686781000	-2.298272000
6	0.916754000	-0.047168000	2.715692000
1	1.364038000	-4.109294000	0.840350000
6	0.609738000	-3.321224000	0.847774000
1	1.466120000	-2.685350000	2.706325000
1	2.017896000	-1.802367000	1.294865000
1	1.605098000	-0.434223000	3.474230000
1	0.307479000	-1.523062000	-2.594648000
1	-1.095069000	-2.528589000	-2.891006000
1	2.427391000	0.174316000	1.210763000
1	-2.596108000	-0.638301000	-2.325607000
1	-1.529766000	-0.250142000	-3.661865000
1	0.186539000	0.568191000	3.236166000
53	-4.165508000	-0.120384000	1.269728000
6	-2.153799000	1.713073000	-1.817423000
1	-2.392644000	2.021157000	-2.839955000
1	-3.043795000	1.303795000	-1.349296000
1	-1.849467000	2.588031000	-1.253747000
6	-0.841035000	-1.630183000	2.988414000
1	-1.386024000	-2.461752000	2.554122000
1	-0.391838000	-1.964643000	3.928648000
1	-1.545097000	-0.828225000	3.188516000
6	-1.983618000	-2.747252000	-0.558864000
1	-1.991970000	-3.722582000	-1.053697000
1	-2.095210000	-2.891974000	0.509809000
1	-2.837939000	-2.174825000	-0.904563000
6	0.132071000	2.549751000	1.259304000
1	-0.593989000	2.949478000	0.561149000
1	-0.404767000	2.280854000	2.162688000
1	0.868136000	3.324016000	1.493177000
8	-2.312972000	0.580319000	1.180784000

E/h = -290.28333324
ZPE/h = 0.44768173
H_{133}/h = 0.45370105
S_{133}/h = 0.02326259

[(D$_{12}$-TMC)(OTf)Fe^{II}]$^+$ (S = 2)

26	-0.639166000	-0.246112000	0.168412000
7	0.672613000	1.378971000	0.719185000
1	1.540382000	2.751634000	-2.285150000
1	0.087950000	3.111553000	-1.417848000
7	-1.086947000	0.614824000	-1.863420000
6	1.520256000	1.845653000	-0.398382000
1	2.150729000	2.660216000	-0.028775000
7	0.146172000	-1.194018000	2.046588000
6	0.460889000	-3.010894000	-0.608997000
1	2.006638000	1.655168000	2.336198000
7	-0.683406000	-2.121195000	-0.903229000
1	1.356097000	-2.516552000	-0.986565000
1	0.330770000	-3.931946000	-1.185304000
6	0.131008000	1.205475000	-2.450116000
6	-1.485995000	-0.564452000	-2.652012000
6	1.547587000	0.835698000	1.777585000
1	2.181779000	1.021941000	-0.667757000
6	0.784098000	2.305103000	-1.638346000
1	-0.120530000	1.592737000	-3.443151000
1	0.859619000	0.409122000	-2.594190000
6	1.160808000	-2.214453000	1.716884000
1	-0.197420000	-3.845304000	1.278826000
6	-0.643515000	-1.774929000	-2.338598000
6	0.796575000	-0.060057000	2.730943000
1	1.461004000	-4.110723000	0.863229000
6	0.673853000	-3.355885000	0.848459000
1	1.556555000	-2.617162000	2.655022000
1	1.988985000	-1.724578000	1.206857000
1	1.486822000	-0.426841000	3.496336000
1	0.394618000	-1.597817000	-2.608821000
1	-0.988811000	-2.623933000	-2.933912000
1	2.354411000	0.289704000	1.295248000
1	-2.532470000	-0.767031000	-2.437211000
1	-1.418271000	-0.346322000	-3.721995000
1	0.021779000	0.502117000	3.246444000
8	-2.531564000	0.262104000	0.837936000
9	-3.651812000	2.969773000	0.996159000
16	-3.460769000	0.540705000	1.958516000
8	-2.810781000	1.100738000	3.126581000
8	-4.422363000	-0.517490000	2.174981000
6	-4.444021000	1.940058000	1.270628000
9	-5.061771000	1.572964000	0.155093000
9	-5.354133000	2.327380000	2.152270000
6	-2.185381000	1.586092000	-1.916927000
1	-2.350322000	1.914246000	-2.947057000
1	-3.091499000	1.125653000	-1.539926000
1	-1.967544000	2.454839000	-1.306728000
6	-0.843753000	-1.771130000	2.966141000
1	-1.410029000	-2.560885000	2.485694000
1	-0.344421000	-2.186987000	3.845422000
1	-1.532462000	-1.001021000	3.292087000
6	-1.953711000	-2.801330000	-0.597220000
1	-2.011108000	-3.746439000	-1.141669000
1	-2.036471000	-2.999025000	0.465034000
1	-2.792351000	-2.174789000	-0.882864000
6	-0.114687000	2.497789000	1.263684000
1	-0.816626000	2.867571000	0.525821000
1	-0.685799000	2.171501000	2.126369000
1	0.552553000	3.310827000	1.558496000

E/h = -399.91361831
ZPE/h = 0.47470241
H_{133}/h = 0.48196921
S_{133}/h = 0.02561460

$[(D_{12}\text{-TMC})(F)Fe^{II}]^{+}$ (S = 2)

26	-0.767995000	-0.179870000	0.236112000
7	0.742933000	1.357129000	0.697857000
1	1.559708000	2.779159000	-2.296599000
1	0.134626000	3.138677000	-1.384909000
7	-1.076687000	0.660649000	-1.852056000
6	1.567861000	1.826652000	-0.430183000
1	2.229161000	2.620730000	-0.066025000
7	0.173305000	-1.154848000	2.064117000
6	0.420884000	-2.976485000	-0.579662000
1	2.163203000	1.602518000	2.246410000
7	-0.707430000	-2.079386000	-0.885872000
1	1.329757000	-2.482077000	-0.924388000
1	0.306119000	-3.890644000	-1.172782000
6	0.121450000	1.258871000	-2.461851000
6	-1.486841000	-0.515390000	-2.633225000
6	1.639351000	0.797310000	1.722656000
1	2.203204000	0.993753000	-0.733050000
6	0.812769000	2.327625000	-1.642095000
1	-0.159314000	1.676166000	-3.435763000
1	0.838087000	0.462056000	-2.654648000
6	1.117069000	-2.246160000	1.771094000
1	-0.309388000	-3.801724000	1.284159000
6	-0.649658000	-1.729294000	-2.314552000
6	0.894291000	-0.054408000	2.719147000
1	1.342121000	-4.142002000	0.899502000
6	0.592648000	-3.349528000	0.877028000
1	1.449110000	-2.679101000	2.721763000
1	1.997319000	-1.814298000	1.297073000
1	1.598457000	-0.443542000	3.461569000
1	0.391710000	-1.547863000	-2.571336000
1	-0.984984000	-2.573639000	-2.924452000
1	2.396357000	0.205455000	1.213134000
1	-2.533462000	-0.711872000	-2.412075000
1	-1.425459000	-0.303496000	-3.705573000
1	0.167548000	0.545295000	3.261938000
9	-2.474179000	0.154384000	0.917177000
6	-2.178610000	1.627002000	-1.857294000
1	-2.440558000	1.904535000	-2.882788000
1	-3.043577000	1.190539000	-1.367460000
1	-1.903699000	2.526704000	-1.317947000
6	-0.879269000	-1.627501000	2.968179000
1	-1.416382000	-2.461316000	2.529312000
1	-0.449647000	-1.953525000	3.920224000
1	-1.589189000	-0.825487000	3.146303000
6	-1.982121000	-2.751790000	-0.600315000
1	-2.039578000	-3.700514000	-1.141159000
1	-2.086376000	-2.942146000	0.461602000
1	-2.813728000	-2.122853000	-0.900627000
6	-0.017472000	2.475401000	1.271968000
1	-0.720005000	2.873257000	0.548827000
1	-0.589512000	2.140438000	2.131497000
1	0.660579000	3.274268000	1.584571000

E/h = -287.20107321
ZPE/h = 0.44598804
H_{133}/h = 0.45142322
S_{133}/h = 0.02171707

{[(D12-TMC)(OTf)Fe^{III}]$_2$(O_3)}$^{2+}$ (S = 5)

26	1.819232000	1.836240000	-2.094431000
7	3.697093000	2.455403000	-2.968994000
1	3.704388000	0.550596000	-5.820479000
1	2.935589000	2.092697000	-5.705851000
7	0.902236000	1.241555000	-3.998527000
6	4.145488000	1.497880000	-4.012247000
1	5.067143000	1.892420000	-4.447553000
7	2.645544000	2.694964000	-0.253063000
6	0.469392000	0.259392000	0.066384000
1	5.579456000	2.710016000	-2.036919000
7	0.074107000	0.909142000	-1.208201000
1	1.140478000	-0.556779000	-0.178386000
1	-0.428636000	-0.177635000	0.510353000
6	1.898046000	0.442593000	-4.757820000
6	-0.281762000	0.415424000	-3.624663000
6	4.591283000	2.309975000	-1.796926000
1	4.387321000	0.570903000	-3.505266000
6	3.163789000	1.199054000	-5.128831000
1	1.411770000	0.105881000	-5.676716000
1	2.147746000	-0.433638000	-4.170284000
6	2.543840000	1.651010000	0.799166000
1	0.481156000	1.985873000	1.370683000
6	-0.242561000	-0.134824000	-2.214258000
6	4.067228000	3.010836000	-0.565726000
1	1.218116000	0.555689000	1.997182000
6	1.133337000	1.160235000	1.093074000
1	2.957444000	2.076239000	1.717174000
1	3.181747000	0.821264000	0.516697000
1	4.698111000	2.747009000	0.284140000
1	0.508908000	-0.910314000	-2.133386000
1	-1.214392000	-0.578635000	-1.986687000
1	4.701615000	1.250972000	-1.601722000
1	-1.162265000	1.037886000	-3.749629000
1	-0.383167000	-0.416524000	-4.322770000
1	4.143995000	4.086583000	-0.690727000
8	2.588130000	0.075113000	-2.001785000
6	0.429075000	2.329693000	-4.878978000
1	0.103785000	1.903873000	-5.829960000
1	-0.398823000	2.846566000	-4.417102000
1	1.215799000	3.049056000	-5.064722000
6	2.031733000	3.930249000	0.270956000
1	0.975665000	3.795321000	0.455771000
1	2.529815000	4.203841000	1.202923000
1	2.150806000	4.732394000	-0.443919000
6	-1.104882000	1.760151000	-0.988434000
1	-1.917781000	1.158105000	-0.578847000
1	-0.873624000	2.560036000	-0.294557000
1	-1.430622000	2.202545000	-1.922588000
6	3.752060000	3.829214000	-3.487594000
1	3.094334000	3.934258000	-4.342569000
1	3.434233000	4.532359000	-2.727534000
1	4.773198000	4.065863000	-3.791764000
8	3.448341000	-0.553874000	-1.102554000
8	4.241730000	-1.429331000	-1.840886000
26	5.017022000	-3.154734000	-1.495121000
7	6.808808000	-2.032790000	-1.036284000
1	5.879666000	-0.735580000	1.993702000
1	6.594766000	-2.293485000	1.783117000
7	4.348759000	-3.440242000	0.574240000
6	6.509529000	-1.037228000	0.023124000
1	7.439815000	-0.515164000	0.260055000
7	5.780876000	-3.132137000	-3.553293000
6	2.542421000	-3.326731000	-3.236415000
1	8.029512000	-0.849786000	-2.297459000
7	3.082064000	-3.965647000	-2.008224000
1	2.327358000	-2.294097000	-2.986032000
1	1.594998000	-3.817440000	-3.473595000
6	4.499920000	-2.134305000	1.264999000
6	2.915795000	-3.833052000	0.475219000
6	7.045918000	-1.324748000	-2.318794000
1	5.826677000	-0.307605000	-0.398261000
6	5.918095000	-1.585056000	1.310057000
1	4.151159000	-2.268100000	2.292296000
1	3.832874000	-1.422954000	0.792767000
6	4.722539000	-2.554267000	-4.421883000

1	3.613735000	-4.377255000	-4.796487000
6	2.284316000	-3.491888000	-0.853450000
6	6.986787000	-2.255597000	-3.511362000
1	2.833548000	-2.909087000	-5.262033000
6	3.431155000	-3.356945000	-4.466111000
1	5.129420000	-2.497242000	-5.434587000
1	4.521983000	-1.544078000	-4.084284000
1	7.038970000	-1.661445000	-4.424839000
1	2.179626000	-2.418426000	-0.948787000
1	1.284278000	-3.930371000	-0.893089000
1	6.298652000	-0.546186000	-2.410257000
1	2.856789000	-4.902842000	0.650010000
1	2.346785000	-3.351061000	1.271451000
1	7.857228000	-2.904535000	-3.510544000
6	5.028280000	-4.469762000	1.383771000
1	4.612074000	-4.458399000	2.392922000
1	4.871732000	-5.444966000	0.944146000
1	6.092777000	-4.288587000	1.435441000
6	6.194845000	-4.422534000	-4.141651000
1	5.394726000	-5.148770000	-4.090688000
1	6.465188000	-4.266431000	-5.187702000
1	7.047520000	-4.815518000	-3.608038000
6	3.000963000	-5.428538000	-2.122296000
1	1.959559000	-5.729046000	-2.250240000
1	3.574449000	-5.769507000	-2.976482000
1	3.403653000	-5.900636000	-1.234314000
6	8.003173000	-2.808621000	-0.669058000
1	7.823464000	-3.382995000	0.232467000
1	8.265305000	-3.496298000	-1.464049000
1	8.840389000	-2.130119000	-0.496056000
8	0.887086000	3.702117000	-2.375925000
6	0.744234000	6.178320000	-3.128919000
9	1.814383000	6.499068000	-2.411274000
8	-0.469884000	5.234908000	-1.017325000
16	-0.152711000	4.769330000	-2.347020000
8	-1.265719000	4.508694000	-3.232897000
9	1.134634000	5.861950000	-4.355994000
9	-0.055949000	7.229583000	-3.192620000
8	5.939758000	-5.041969000	-1.306224000
6	6.079812000	-7.628894000	-1.498736000
9	5.048327000	-7.806145000	-0.682580000
8	7.346327000	-6.236943000	0.314195000
16	6.985095000	-6.078112000	-1.074679000
8	8.068042000	-6.019799000	-2.032040000
9	5.631522000	-7.581339000	-2.746144000
9	6.898758000	-8.661109000	-1.378497000

E/h = -847.725598295437
ZPE/h = 0.96512291
H_{133}/h = 0.97897109
S_{133}/h = 0.03819857

$\{[(D_{12}\text{-TMC})(F)Fe^{III}]_2(O_3)\}^{2+}$ (S = 5)

26	1.792031000	1.840645000	-2.105966000
7	3.706092000	2.394258000	-2.950945000
1	3.654862000	0.522223000	-5.817693000
1	2.926890000	2.080431000	-5.674257000
7	0.855033000	1.225128000	-3.995547000
6	4.122963000	1.425753000	-3.990934000
1	5.058703000	1.785642000	-4.429107000
7	2.619829000	2.662466000	-0.242520000
6	0.380615000	0.299408000	0.078746000
1	5.578216000	2.617461000	-1.989959000
7	0.028164000	0.936339000	-1.209964000
1	1.039059000	-0.533814000	-0.146324000
1	-0.535253000	-0.109233000	0.515703000
6	1.839629000	0.436348000	-4.771791000
6	-0.331752000	0.409009000	-3.620924000
6	4.577805000	2.235128000	-1.767964000
1	4.326466000	0.489955000	-3.482506000
6	3.129990000	1.170185000	-5.113177000
1	1.359416000	0.130992000	-5.706146000
1	2.068355000	-0.457278000	-4.201039000
6	2.478675000	1.650081000	0.828210000
1	0.416706000	2.038571000	1.368764000
6	-0.310630000	-0.113122000	-2.197227000
6	4.048312000	2.950099000	-0.541957000
1	1.109476000	0.597138000	2.017546000
6	1.052303000	1.195003000	1.106566000
1	2.886793000	2.079849000	1.747945000
1	3.096088000	0.797508000	0.564917000
1	4.668129000	2.678144000	0.314096000
1	0.430373000	-0.897529000	-2.099694000
1	-1.292771000	-0.534483000	-1.966613000
1	4.659076000	1.174035000	-1.568353000
1	-1.212018000	1.027624000	-3.769332000
1	-0.427333000	-0.438476000	-4.301456000
1	4.147494000	4.024266000	-0.668948000
8	2.510105000	0.009582000	-1.980703000
6	0.386914000	2.356540000	-4.815461000
1	-0.044191000	1.979508000	-5.745479000
1	-0.357428000	2.920851000	-4.267904000
1	1.202245000	3.028856000	-5.048032000
6	1.988753000	3.919224000	0.196521000
1	0.931094000	3.778189000	0.378702000
1	2.466537000	4.262404000	1.116762000
1	2.094938000	4.670321000	-0.575951000
6	-1.098763000	1.860097000	-1.038520000
1	-1.974121000	1.316101000	-0.677288000
1	-0.844875000	2.636811000	-0.326474000
1	-1.336216000	2.343225000	-1.979417000
6	3.773388000	3.766325000	-3.465235000
1	3.140567000	3.870174000	-4.339349000
1	3.419155000	4.468388000	-2.719371000
1	4.802621000	4.009339000	-3.738026000
8	3.435107000	-0.564434000	-1.096144000
8	4.293345000	-1.374267000	-1.855133000
26	5.047612000	-3.162945000	-1.507705000
7	6.842926000	-2.029868000	-1.044808000
1	5.963926000	-0.752042000	2.006670000
1	6.653507000	-2.317793000	1.775704000
7	4.371562000	-3.407920000	0.569047000
6	6.568304000	-1.045485000	0.026062000
1	7.505646000	-0.533026000	0.261157000
7	5.829891000	-3.114648000	-3.561688000
6	2.575360000	-3.296812000	-3.251909000
1	8.072026000	-0.838448000	-2.289829000
7	3.088373000	-3.931350000	-2.015722000
1	2.392499000	-2.254167000	-3.015813000
1	1.616602000	-3.764109000	-3.495964000
6	4.553765000	-2.124488000	1.283352000
6	2.935016000	-3.779956000	0.470605000
6	7.086805000	-1.311823000	-2.315869000
1	5.884662000	-0.309510000	-0.384266000
6	5.981520000	-1.596311000	1.315395000
1	4.218171000	-2.266601000	2.315182000
1	3.896563000	-1.393624000	0.825300000
6	4.775258000	-2.576259000	-4.452292000

1	3.657488000	-4.406366000	-4.765684000
6	2.301503000	-3.443243000	-0.863500000
6	7.022725000	-2.225112000	-3.524159000
1	2.890678000	-2.951631000	-5.290634000
6	3.477844000	-3.373649000	-4.472865000
1	5.179630000	-2.549431000	-5.468571000
1	4.571738000	-1.556913000	-4.142332000
1	7.054755000	-1.612548000	-4.426706000
1	2.210003000	-2.368932000	-0.967392000
1	1.296950000	-3.874757000	-0.891357000
1	6.335822000	-0.535496000	-2.397950000
1	2.858889000	-4.847296000	0.656891000
1	2.369407000	-3.282620000	1.260394000
1	7.903856000	-2.859684000	-3.548594000
6	5.067838000	-4.476092000	1.306994000
1	4.666226000	-4.538557000	2.320843000
1	4.930469000	-5.421165000	0.797225000
1	6.131069000	-4.279869000	1.359432000
6	6.265536000	-4.430009000	-4.063437000
1	5.448696000	-5.139928000	-4.051799000
1	6.634198000	-4.326043000	-5.086304000
1	7.051183000	-4.819322000	-3.427895000
6	3.013717000	-5.392874000	-2.117288000
1	1.971930000	-5.703984000	-2.220815000
1	3.572939000	-5.735815000	-2.980434000
1	3.447645000	-5.858324000	-1.239701000
6	8.002159000	-2.860295000	-0.697610000
1	7.814158000	-3.409836000	0.217310000
1	8.193236000	-3.586089000	-1.480096000
1	8.883637000	-2.229967000	-0.561055000
9	1.027705000	3.516784000	-2.378100000
9	5.831433000	-4.842371000	-1.335262000

E/h = -622.321160646865
ZPE/h = 0.90694091
H_{133}/h = 0.91733788
S_{133}/h = 0.03139255

{[(D_{12}-TMC)(I)Fe^{III}]$_2$(O_3)}$^{2+}$ (S = 5)

26	1.903046000	1.976084000	-2.211984000
7	3.738818000	2.632183000	-3.198274000
1	3.642018000	0.604936000	-5.963062000
1	2.820770000	2.120216000	-5.858168000
7	0.883905000	1.268719000	-4.070548000
6	4.153249000	1.638893000	-4.223823000
1	5.025256000	2.046872000	-4.741252000
7	2.839791000	2.759908000	-0.355173000
6	0.907570000	0.113198000	0.000280000
1	5.618669000	3.106283000	-2.351562000
7	0.343838000	0.815742000	-1.182168000
1	1.562070000	-0.663287000	-0.372936000
1	0.078420000	-0.368670000	0.524743000
6	1.894201000	0.468228000	-4.808609000
6	-0.233601000	0.421267000	-3.576141000
6	4.693364000	2.602039000	-2.062840000
1	4.476806000	0.746765000	-3.705710000
6	3.114507000	1.257098000	-5.264555000
1	1.401639000	0.056227000	-5.693449000
1	2.198062000	-0.358966000	-4.178687000
6	2.993018000	1.582302000	0.536013000
1	1.037322000	1.706490000	1.458205000
6	-0.012666000	-0.190504000	-2.211975000
6	4.151288000	3.276902000	-0.823703000
1	1.944563000	0.278485000	1.803673000
6	1.680152000	0.961622000	0.994408000
1	3.544081000	1.912589000	1.421039000
1	3.599959000	0.841179000	0.030394000
1	4.888086000	3.187334000	-0.022053000
1	0.787845000	-0.915918000	-2.250210000
1	-0.926634000	-0.710182000	-1.914616000
1	4.941128000	1.570984000	-1.850239000
1	-1.120332000	1.047947000	-3.552361000
1	-0.430947000	-0.378232000	-4.293835000
1	4.021073000	4.338050000	-1.014855000
8	2.748909000	0.216920000	-2.077244000
6	0.297568000	2.224213000	-5.032512000
1	-0.045651000	1.677025000	-5.912870000
1	-0.539651000	2.738969000	-4.581340000
1	1.022063000	2.969232000	-5.334636000
6	2.220731000	3.838912000	0.440978000
1	1.199224000	3.596485000	0.704292000
1	2.803460000	3.984147000	1.353079000
1	2.208064000	4.759036000	-0.126792000
6	-0.863626000	1.550208000	-0.771028000
1	-1.573651000	0.855949000	-0.317789000
1	-0.608829000	2.321041000	-0.052071000
1	-1.325780000	2.032349000	-1.623753000
6	3.751288000	3.972210000	-3.808571000
1	3.033041000	4.024532000	-4.619047000
1	3.485519000	4.727313000	-3.079357000
1	4.749726000	4.177910000	-4.198820000
8	4.016172000	-0.317875000	-1.853972000
8	3.851676000	-1.651811000	-1.483137000
26	4.936662000	-3.275332000	-1.400225000
7	6.600822000	-1.984819000	-0.819471000
1	5.372516000	-0.772125000	2.143754000
1	6.206032000	-2.276269000	1.996145000
7	4.169009000	-3.605081000	0.675632000
6	6.171952000	-1.026652000	0.233315000
1	7.057105000	-0.467327000	0.546850000
7	5.742873000	-3.054532000	-3.460264000
6	2.460613000	-3.352987000	-3.188783000
1	7.801017000	-0.711938000	-2.007998000
7	2.979390000	-4.085337000	-2.004357000
1	2.207827000	-2.353870000	-2.859583000
1	1.538978000	-3.847132000	-3.506539000
6	4.149103000	-2.266300000	1.318881000
6	2.794897000	-4.139227000	0.480090000
6	6.851192000	-1.247308000	-2.081729000
1	5.491744000	-0.319465000	-0.222432000
6	5.518277000	-1.618022000	1.469761000
1	3.715538000	-2.389318000	2.315012000
1	3.493300000	-1.623401000	0.743207000
6	4.652689000	-2.408063000	-4.233285000

1	3.627017000	-4.235714000	-4.782426000
6	2.141388000	-3.753064000	-0.826615000
6	6.912425000	-2.153066000	-3.290082000
1	2.804266000	-2.767128000	-5.163442000
6	3.392811000	-3.251550000	-4.382461000
1	5.044316000	-2.195147000	-5.231932000
1	4.415184000	-1.461015000	-3.764662000
1	7.033045000	-1.538166000	-4.184813000
1	1.947032000	-2.689713000	-0.850437000
1	1.183728000	-4.274027000	-0.900769000
1	6.073895000	-0.505797000	-2.205265000
1	2.864202000	-5.221180000	0.544815000
1	2.155803000	-3.814697000	1.304425000
1	7.791620000	-2.786987000	-3.222193000
6	4.849215000	-4.529800000	1.604289000
1	4.360210000	-4.478220000	2.579554000
1	4.792355000	-5.541199000	1.225619000
1	5.895135000	-4.274524000	1.715383000
6	6.215933000	-4.211108000	-4.247332000
1	5.456888000	-4.980075000	-4.308476000
1	6.471027000	-3.874129000	-5.254386000
1	7.090720000	-4.643293000	-3.781348000
6	2.902654000	-5.531777000	-2.266646000
1	1.870312000	-5.802552000	-2.496075000
1	3.537418000	-5.797776000	-3.104569000
1	3.239718000	-6.094355000	-1.404719000
6	7.844037000	-2.643852000	-0.385914000
1	7.669020000	-3.227128000	0.511075000
1	8.206027000	-3.316217000	-1.153749000
1	8.600289000	-1.883933000	-0.181226000
53	0.520015000	4.366565000	-2.430108000
53	6.282000000	-5.698733000	-1.224482000

E/h = -596.473313860975
ZPE/h = 0.90648989
H_{133}/h = 0.91735549
S_{133}/h = 0.03270487

$[(D_{12}\text{-TMC})(OTf)Fe^{III}(O_2)]^+$ (S = 2)

26	-0.267125000	-0.153983000	0.100072000
7	0.933094000	1.502158000	0.778478000
1	1.549905000	3.076736000	-2.199893000
1	0.124989000	3.317242000	-1.253571000
7	-0.956369000	0.796490000	-1.756567000
6	1.683776000	2.129010000	-0.342367000
1	2.199406000	3.003819000	0.059982000
7	0.277921000	-1.096543000	1.989476000
6	-0.142296000	-3.140257000	-0.534366000
1	2.441562000	1.560039000	2.262078000
7	-1.004275000	-1.968207000	-0.837941000
1	0.791499000	-3.005088000	-1.070333000
1	-0.629683000	-4.024009000	-0.952996000
6	0.208563000	1.475376000	-2.385981000
6	-1.461698000	-0.303119000	-2.624364000
6	1.885047000	0.815587000	1.688265000
1	2.447081000	1.430778000	-0.667042000
6	0.853566000	2.560808000	-1.537422000
1	-0.147310000	1.929655000	-3.314082000
1	0.940582000	0.717870000	-2.650602000
6	0.990608000	-2.360908000	1.671197000
1	-0.752777000	-3.630438000	1.481925000
6	-0.895171000	-1.661493000	-2.286147000
6	1.188509000	-0.120080000	2.649787000
1	0.739647000	-4.314878000	0.949057000
6	0.155223000	-3.394033000	0.931175000
1	1.313943000	-2.795467000	2.620327000
1	1.885905000	-2.118171000	1.106995000
1	1.942284000	-0.657278000	3.227695000
1	0.151974000	-1.716273000	-2.560391000
1	-1.432485000	-2.420469000	-2.858733000
1	2.595081000	0.267342000	1.079464000
1	-2.543028000	-0.320030000	-2.528500000
1	-1.242058000	-0.075430000	-3.668360000
1	0.593114000	0.447927000	3.358049000
8	1.348213000	-0.720079000	-0.895923000
8	-2.022369000	0.433106000	0.989083000
9	-4.885956000	0.001013000	0.440946000
16	-3.003457000	1.226018000	1.789727000
8	-3.400544000	2.456849000	1.150940000
8	-2.669235000	1.282926000	3.194329000
6	-4.494251000	0.143588000	1.700142000
9	-4.230191000	-1.053903000	2.203155000
9	-5.477041000	0.693219000	2.394069000
6	-2.050643000	1.776947000	-1.619193000
1	-2.276700000	2.201886000	-2.598842000
1	-2.933657000	1.286980000	-1.230625000
1	-1.780653000	2.573558000	-0.940007000
6	-0.808828000	-1.402344000	2.941368000
1	-1.571908000	-2.014201000	2.478923000
1	-0.392240000	-1.941149000	3.794541000
1	-1.267533000	-0.486746000	3.286284000
6	-2.403225000	-2.272720000	-0.496566000
1	-2.744419000	-3.128718000	-1.080707000
1	-2.491942000	-2.508490000	0.557425000
1	-3.037793000	-1.420158000	-0.707784000
6	0.201102000	2.545585000	1.516881000
1	-0.545260000	3.011131000	0.883469000
1	-0.296877000	2.123256000	2.381159000
1	0.904950000	3.308074000	1.853562000
8	2.586522000	-0.983676000	-0.663702000

E/h = -431.85491428
ZPE/h = 0.48279725
H_{133}/h = 0.48999501
S_{133}/h = 0.02531924

G. Cartesian coordinates and thermochemical data for the **[(D12-TMC)(X)Co]+ (X = OTf, F) + O3** systems

[(D12-TMC)(OTf)Co^{III}(O3)]+ (S = 5/2)

27	-0.244632000	-0.239143000	0.050587000
7	0.809382000	1.475957000	0.737504000
1	1.423101000	3.036889000	-2.247054000
1	-0.050530000	3.212297000	-1.364727000
7	-0.952204000	0.623112000	-1.818791000
6	1.542544000	2.155722000	-0.358843000
1	1.960772000	3.078320000	0.051228000
7	0.319934000	-1.099776000	1.975522000
6	0.037406000	-3.233123000	-0.482897000
1	2.198056000	1.750825000	2.303508000
7	-0.873180000	-2.112625000	-0.829976000
1	0.969908000	-3.067722000	-1.011497000
1	-0.405489000	-4.150927000	-0.877920000
6	0.196914000	1.344524000	-2.428265000
6	-1.376803000	-0.524084000	-2.667164000
6	1.781301000	0.932059000	1.712653000
1	2.380542000	1.529179000	-0.641100000
6	0.735011000	2.496175000	-1.595815000
1	-0.148587000	1.744573000	-3.384685000
1	0.982473000	0.624616000	-2.634352000
6	1.103802000	-2.332128000	1.705639000
1	-0.568423000	-3.693129000	1.540975000
6	-0.753080000	-1.842694000	-2.281306000
6	1.148130000	-0.065988000	2.652381000
1	0.961828000	-4.318063000	1.042901000
6	0.332009000	-3.428924000	0.990533000
1	1.435655000	-2.720368000	2.672023000
1	1.995318000	-2.068293000	1.147588000
1	1.930682000	-0.547183000	3.240895000
1	0.299222000	-1.857479000	-2.538172000
1	-1.245811000	-2.640167000	-2.842194000
1	2.602817000	0.485601000	1.169224000
1	-2.457596000	-0.595365000	-2.596308000
1	-1.142613000	-0.310380000	-3.711072000
1	0.504295000	0.457622000	3.351998000
8	1.337571000	-0.782633000	-0.858499000
8	-2.080485000	0.255348000	0.773694000
9	-4.041138000	2.502857000	0.534956000
16	-3.190941000	0.343213000	1.772572000
8	-2.855202000	1.177433000	2.903867000
8	-3.820397000	-0.929853000	2.028870000
6	-4.491537000	1.307781000	0.885508000
9	-4.900091000	0.662747000	-0.199203000
9	-5.524147000	1.462831000	1.698268000
6	-2.092793000	1.553732000	-1.756984000
1	-2.270568000	1.969983000	-2.750274000
1	-2.974070000	1.017748000	-1.441191000
1	-1.907856000	2.363031000	-1.064692000
6	-0.764097000	-1.459156000	2.910292000
1	-1.504966000	-2.087052000	2.436295000
1	-0.339549000	-1.993557000	3.762549000
1	-1.245405000	-0.563872000	3.277970000
6	-2.259444000	-2.480537000	-0.502228000
1	-2.539978000	-3.374013000	-1.062528000
1	-2.357542000	-2.680747000	0.557480000
1	-2.938476000	-1.673952000	-0.751380000
6	-0.077252000	2.438788000	1.414100000
1	-0.815047000	2.827759000	0.723139000
1	-0.602882000	1.964359000	2.233084000
1	0.521336000	3.266576000	1.797149000
8	2.723051000	-0.737609000	-0.581615000
8	3.370197000	-1.149704000	-1.584621000

E/h = -469.70750975
ZPE/h = 0.48654517
H_{133}/h = 0.49409900
S_{133}/h = 0.02614524

$[(D_{12}\text{-TMC})(OTf)Co^{III}(O_3)]^+$ (S = $^3/_2$)

27	-0.161233000	-0.291677000	0.015513000
7	0.749252000	1.395932000	0.684424000
1	1.448485000	3.005468000	-2.262880000
1	-0.038900000	3.210656000	-1.402960000
7	-0.902880000	0.586045000	-1.769367000
6	1.509993000	2.103674000	-0.383218000
1	1.916562000	3.006284000	0.076419000
7	0.311510000	-1.097135000	1.899313000
6	0.079371000	-3.206447000	-0.475163000
1	2.153878000	1.752209000	2.217432000
7	-0.813789000	-2.057474000	-0.794603000
1	1.009729000	-3.042853000	-1.006110000
1	-0.395790000	-4.095852000	-0.892857000
6	0.201724000	1.325347000	-2.436059000
6	-1.361382000	-0.538229000	-2.636914000
6	1.746168000	0.901130000	1.669925000
1	2.347389000	1.475995000	-0.661587000
6	0.737556000	2.481936000	-1.623022000
1	-0.195979000	1.710146000	-3.376942000
1	0.993880000	0.620046000	-2.663488000
6	1.122820000	-2.324135000	1.685416000
1	-0.517893000	-3.721529000	1.539319000
6	-0.707952000	-1.828516000	-2.261099000
6	1.112408000	-0.064031000	2.619033000
1	1.023227000	-4.313452000	1.021349000
6	0.374673000	-3.437735000	0.986618000
1	1.433070000	-2.674361000	2.671455000
1	2.020638000	-2.060046000	1.138345000
1	1.875695000	-0.550360000	3.225720000
1	0.343823000	-1.819130000	-2.517975000
1	-1.184387000	-2.659751000	-2.782678000
1	2.561043000	0.440713000	1.128646000
1	-2.438971000	-0.613705000	-2.539522000
1	-1.150029000	-0.301752000	-3.679368000
1	0.447284000	0.457942000	3.298155000
8	1.499225000	-0.802874000	-0.978328000
8	-2.188056000	0.248650000	0.832409000
9	-4.022285000	2.596756000	0.618666000
16	-3.282186000	0.376232000	1.818542000
8	-2.925100000	1.174033000	2.976745000
8	-3.984867000	-0.865538000	2.069224000
6	-4.536275000	1.419459000	0.955012000
9	-4.975061000	0.817493000	-0.146053000
9	-5.568157000	1.624192000	1.759913000
6	-2.054678000	1.508205000	-1.657218000
1	-2.263108000	1.917127000	-2.646489000
1	-2.917792000	0.964346000	-1.309731000
1	-1.854465000	2.322067000	-0.977603000
6	-0.807580000	-1.456118000	2.800164000
1	-1.543861000	-2.069684000	2.305189000
1	-0.401069000	-2.005125000	3.650383000
1	-1.282967000	-0.559782000	3.168635000
6	-2.206405000	-2.410470000	-0.451145000
1	-2.498377000	-3.279853000	-1.039370000
1	-2.289707000	-2.654673000	0.598685000
1	-2.875232000	-1.586937000	-0.658485000
6	-0.162887000	2.347404000	1.351866000
1	-0.900142000	2.720561000	0.655434000
1	-0.684318000	1.870741000	2.170248000
1	0.427221000	3.182744000	1.727487000
8	2.830753000	-0.826492000	-0.565925000
8	3.606828000	-1.046406000	-1.549929000

E/h = -469.72646168
ZPE/h = 0.48857273
H_{133}/h = 0.49602236
S_{133}/h = 0.02586102

[(D_{12}-TMC)(OTf)Co^{III}(O_3)]$^+$ TS O_2 extrusion (S = $^5/_2$)

27	-0.212141000	-0.260609000	0.040764000
7	0.788135000	1.495450000	0.751020000
1	1.411589000	3.054550000	-2.232207000
1	-0.072669000	3.210062000	-1.363745000
7	-0.934102000	0.610698000	-1.824865000
6	1.523696000	2.173312000	-0.342113000
1	1.942742000	3.098128000	0.063360000
7	0.305807000	-1.100134000	1.995984000
6	0.051200000	-3.233995000	-0.474489000
1	2.216531000	1.733704000	2.291058000
7	-0.854817000	-2.116757000	-0.837376000
1	0.990661000	-3.066635000	-0.990936000
1	-0.382479000	-4.155467000	-0.872078000
6	0.210400000	1.344028000	-2.421643000
6	-1.338635000	-0.533899000	-2.682778000
6	1.763893000	0.928764000	1.706589000
1	2.361198000	1.543552000	-0.620541000
6	0.724987000	2.504762000	-1.586777000
1	-0.122713000	1.736009000	-3.386421000
1	1.008600000	0.632128000	-2.610893000
6	1.095825000	-2.326890000	1.725444000
1	-0.576950000	-3.685240000	1.544512000
6	-0.716069000	-1.850760000	-2.285621000
6	1.132057000	-0.058823000	2.658604000
1	0.956926000	-4.315671000	1.065084000
6	0.330292000	-3.424730000	1.003413000
1	1.428940000	-2.718466000	2.690508000
1	1.983584000	-2.055076000	1.164126000
1	1.916807000	-0.530256000	3.252962000
1	0.341408000	-1.857961000	-2.523987000
1	-1.194845000	-2.651259000	-2.854822000
1	2.561186000	0.460135000	1.143891000
1	-2.420262000	-0.612133000	-2.632713000
1	-1.087100000	-0.320384000	-3.722916000
1	0.489013000	0.473533000	3.352709000
8	1.316405000	-0.751103000	-0.739802000
8	-2.075289000	0.261458000	0.788528000
9	-4.022369000	2.515443000	0.543701000
16	-3.188618000	0.344317000	1.775141000
8	-2.866114000	1.167503000	2.920581000
8	-3.831000000	-0.926624000	2.019691000
6	-4.481704000	1.322019000	0.892034000
9	-4.897129000	0.685077000	-0.195911000
9	-5.515696000	1.485604000	1.703027000
6	-2.083269000	1.528488000	-1.759046000
1	-2.262613000	1.956238000	-2.747702000
1	-2.960563000	0.980653000	-1.451030000
1	-1.909244000	2.329521000	-1.054340000
6	-0.770865000	-1.461451000	2.935403000
1	-1.511070000	-2.093706000	2.465167000
1	-0.343511000	-1.991268000	3.789628000
1	-1.256844000	-0.567419000	3.300544000
6	-2.244513000	-2.476416000	-0.520112000
1	-2.528118000	-3.369779000	-1.079743000
1	-2.351167000	-2.671403000	0.539947000
1	-2.916227000	-1.665049000	-0.774358000
6	-0.081208000	2.460351000	1.440333000
1	-0.809503000	2.872423000	0.751667000
1	-0.621531000	1.980167000	2.246505000
1	0.525802000	3.273534000	1.842767000
8	2.954962000	-0.744515000	-0.706531000
8	3.376078000	-1.276530000	-1.705529000

E/h = -469.70118464
ZPE/h = 0.48477517
H_{133}/h = 0.49236911
S_{133}/h = 0.02626714

[(D$_{12}$-TMC)(OTf)Co^{III}(O$_3$)]$^+$ TS O$_2$ extrusion (S = $^3/_2$)

27	-0.121924000	-0.296836000	-0.003777000
7	0.746634000	1.378499000	0.681102000
1	1.436465000	3.015230000	-2.251701000
1	-0.051278000	3.206997000	-1.390133000
7	-0.920703000	0.596156000	-1.788604000
6	1.503483000	2.101907000	-0.377531000
1	1.902491000	3.004869000	0.088025000
7	0.296709000	-1.109870000	1.935422000
6	0.092229000	-3.204334000	-0.473127000
1	2.143548000	1.739895000	2.220512000
7	-0.785126000	-2.045014000	-0.799736000
1	1.027267000	-3.053129000	-0.998925000
1	-0.391103000	-4.089829000	-0.889980000
6	0.193240000	1.331668000	-2.439025000
6	-1.352275000	-0.537555000	-2.651085000
6	1.733607000	0.886739000	1.677667000
1	2.344150000	1.483587000	-0.662299000
6	0.728084000	2.483018000	-1.615881000
1	-0.184532000	1.721595000	-3.386795000
1	0.985779000	0.621778000	-2.653892000
6	1.120182000	-2.323061000	1.704351000
1	-0.519173000	-3.718319000	1.536863000
6	-0.679281000	-1.818956000	-2.264965000
6	1.093474000	-0.069225000	2.637607000
1	1.023943000	-4.312639000	1.030005000
6	0.378420000	-3.434535000	0.992048000
1	1.444410000	-2.686273000	2.682053000
1	2.005438000	-2.039679000	1.144488000
1	1.858567000	-0.539957000	3.255785000
1	0.374396000	-1.793028000	-2.516894000
1	-1.138757000	-2.658975000	-2.788265000
1	2.548700000	0.415999000	1.147304000
1	-2.429094000	-0.633329000	-2.560334000
1	-1.140504000	-0.307558000	-3.695586000
1	0.428061000	0.464798000	3.307454000
8	1.437209000	-0.818210000	-0.871287000
8	-2.173358000	0.255698000	0.827026000
9	-3.984167000	2.617639000	0.638894000
16	-3.268762000	0.371794000	1.809202000
8	-2.914369000	1.145229000	2.985931000
8	-3.986858000	-0.866790000	2.035420000
6	-4.512638000	1.443409000	0.965215000
9	-4.969293000	0.862089000	-0.140047000
9	-5.537275000	1.655281000	1.778699000
6	-2.075735000	1.510631000	-1.695982000
1	-2.284546000	1.912888000	-2.688686000
1	-2.938291000	0.965648000	-1.346588000
1	-1.884285000	2.330403000	-1.019951000
6	-0.807503000	-1.480002000	2.844025000
1	-1.540297000	-2.102674000	2.353714000
1	-0.395873000	-2.022067000	3.697212000
1	-1.294936000	-0.588324000	3.209802000
6	-2.180619000	-2.382968000	-0.454825000
1	-2.482496000	-3.250909000	-1.040514000
1	-2.264041000	-2.621487000	0.596214000
1	-2.841409000	-1.553403000	-0.662308000
6	-0.175485000	2.322509000	1.344371000
1	-0.911773000	2.689056000	0.643362000
1	-0.697740000	1.842350000	2.159626000
1	0.407070000	3.161915000	1.723187000
8	3.141801000	-0.620946000	-0.635739000
8	3.707922000	-1.268190000	-1.484457000

E/h = -469.71064969
ZPE/h = 0.48664123
H_{133}/h = 0.49411598
S_{133}/h = 0.02592249

[(D$_{12}$-TMC)(OTf)Co^{IV}(O)]$^{+}$ (S = $^{5}/_{2}$)

27	-3.026510000	0.819395000	1.695137000
7	-1.780061000	2.446530000	2.325549000
1	-1.189673000	3.891235000	-0.720499000
1	-2.541361000	4.267555000	0.288999000
7	-3.818078000	1.809581000	-0.066898000
6	-1.039837000	2.973080000	1.152651000
1	-0.411646000	3.798875000	1.496696000
7	-2.499861000	-0.098482000	3.581953000
6	-3.052721000	-2.120943000	1.105521000
1	-0.214700000	2.456314000	3.752574000
7	-3.922875000	-0.946125000	0.853878000
1	-2.132893000	-1.970933000	0.544151000
1	-3.547966000	-2.999593000	0.684035000
6	-2.656370000	2.392896000	-0.784421000
6	-4.445782000	0.735246000	-0.877853000
6	-0.829867000	1.732857000	3.212124000
1	-0.373545000	2.185876000	0.805280000
6	-1.888841000	3.449591000	-0.009095000
1	-3.037770000	2.845737000	-1.703261000
1	-1.990116000	1.581956000	-1.069413000
6	-1.826146000	-1.381768000	3.260382000
1	-3.594097000	-2.621601000	3.138168000
6	-3.879100000	-0.630022000	-0.592633000
6	-1.545273000	0.854937000	4.208120000
1	-2.134792000	-3.329422000	2.542378000
6	-2.704065000	-2.399133000	2.553439000
1	-1.486241000	-1.814386000	4.204539000
1	-0.946159000	-1.164672000	2.660555000
1	-0.809968000	0.302211000	4.795246000
1	-2.844728000	-0.688051000	-0.918511000
1	-4.446281000	-1.378449000	-1.150963000
1	-0.167790000	1.139444000	2.588459000
1	-5.510228000	0.746909000	-0.664335000
1	-4.331796000	0.962113000	-1.939158000
1	-2.109757000	1.468234000	4.903600000
8	-1.562270000	0.239050000	0.867372000
8	-4.812627000	1.493564000	2.690155000
9	-7.715893000	1.162405000	2.375918000
16	-5.681088000	2.342921000	3.534276000
8	-6.089626000	3.582863000	2.910462000
8	-5.248964000	2.428387000	4.915668000
6	-7.222943000	1.334188000	3.597670000
9	-6.978143000	0.140360000	4.125044000
9	-8.134723000	1.946258000	4.339021000
6	-4.832500000	2.859771000	0.126208000
1	-5.079721000	3.299613000	-0.842073000
1	-5.722745000	2.429593000	0.563141000
1	-4.474556000	3.635632000	0.787861000
6	-3.571125000	-0.364838000	4.560144000
1	-4.370468000	-0.942579000	4.116558000
1	-3.153235000	-0.922768000	5.400493000
1	-3.982436000	0.567511000	4.918213000
6	-5.301282000	-1.234766000	1.268987000
1	-5.691452000	-2.072557000	0.688009000
1	-5.331885000	-1.492975000	2.320493000
1	-5.931056000	-0.366346000	1.118683000
6	-2.420584000	3.548794000	3.054105000
1	-3.160476000	4.037053000	2.431252000
1	-2.917239000	3.178008000	3.942552000
1	-1.665371000	4.280984000	3.346404000

E/h = -437.75197193
ZPE/h = 0.47897330
H_{133}/h = 0.48580704
S_{133}/h = 0.02474394

[(D12-TMC)(OTf)Co^{IV}(O)]$^+$ (S = $^3/_2$)

27	-3.020545000	0.817751000	1.690750000
7	-1.838662000	2.337320000	2.290647000
1	-1.156119000	3.861930000	-0.704147000
1	-2.508044000	4.263989000	0.296081000
7	-3.849620000	1.865487000	-0.098331000
6	-1.069998000	2.891141000	1.140630000
1	-0.439654000	3.689362000	1.537404000
7	-2.507612000	-0.138997000	3.639602000
6	-3.012780000	-2.043638000	1.115887000
1	-0.278551000	2.449679000	3.710656000
7	-3.861124000	-0.838735000	0.895327000
1	-2.089171000	-1.891519000	0.562998000
1	-3.532886000	-2.884464000	0.652829000
6	-2.682770000	2.417737000	-0.816611000
6	-4.471982000	0.775996000	-0.876423000
6	-0.871754000	1.681006000	3.212119000
1	-0.408810000	2.105393000	0.783283000
6	-1.884113000	3.429369000	-0.016489000
1	-3.045031000	2.898253000	-1.730373000
1	-2.039313000	1.593339000	-1.113466000
6	-1.816573000	-1.399818000	3.303330000
1	-3.579779000	-2.640879000	3.112466000
6	-3.869181000	-0.566079000	-0.566619000
6	-1.568259000	0.829819000	4.241354000
1	-2.115193000	-3.321676000	2.496493000
6	-2.682370000	-2.391526000	2.550236000
1	-1.481558000	-1.862491000	4.236361000
1	-0.931867000	-1.164589000	2.717495000
1	-0.816468000	0.309421000	4.839597000
1	-2.842665000	-0.616708000	-0.916928000
1	-4.434455000	-1.351666000	-1.070810000
1	-0.197961000	1.080201000	2.608803000
1	-5.534090000	0.769633000	-0.649433000
1	-4.380681000	0.972944000	-1.947138000
1	-2.132179000	1.457187000	4.925279000
8	-1.630214000	0.272790000	0.900931000
8	-4.757024000	1.457849000	2.653825000
9	-7.674828000	1.180054000	2.321292000
16	-5.623437000	2.310835000	3.502497000
8	-6.008883000	3.560895000	2.886928000
8	-5.202034000	2.375581000	4.887156000
6	-7.180109000	1.323413000	3.545395000
9	-6.955277000	0.118962000	4.055014000
9	-8.079459000	1.941450000	4.295661000
6	-4.859225000	2.921516000	0.053713000
1	-5.138836000	3.303998000	-0.931223000
1	-5.737042000	2.521405000	0.542744000
1	-4.487026000	3.742377000	0.651564000
6	-3.548619000	-0.427573000	4.637499000
1	-4.318011000	-1.068808000	4.228159000
1	-3.099671000	-0.931606000	5.497338000
1	-4.010158000	0.492647000	4.966312000
6	-5.233444000	-1.109485000	1.356576000
1	-5.613421000	-1.987255000	0.833825000
1	-5.242719000	-1.295448000	2.421716000
1	-5.872136000	-0.261456000	1.151734000
6	-2.519537000	3.439872000	2.992352000
1	-3.290094000	3.869879000	2.366939000
1	-2.972096000	3.083648000	3.907838000
1	-1.784181000	4.207406000	3.234366000

E/h = -437.77011167
ZPE/h = 0.48040892
H_{133}/h = 0.48698734
S_{133}/h = 0.02411404

[(D$_{12}$-TMC)(F)Co^{III}(O$_3$)]$^+$ (S = $^5/_2$)

27	-0.222835000	-0.267751000	0.033454000
7	0.820683000	1.466472000	0.716594000
1	1.341828000	3.109513000	-2.238085000
1	-0.112641000	3.222543000	-1.315497000
7	-0.934301000	0.597364000	-1.835803000
6	1.525614000	2.164518000	-0.383401000
1	1.961317000	3.078772000	0.029268000
7	0.343630000	-1.101560000	1.959229000
6	0.100485000	-3.233172000	-0.490505000
1	2.257442000	1.729302000	2.243559000
7	-0.816879000	-2.133160000	-0.861414000
1	1.055525000	-3.030219000	-0.964711000
1	-0.290732000	-4.157586000	-0.925150000
6	0.148178000	1.398715000	-2.453824000
6	-1.322434000	-0.545761000	-2.702612000
6	1.811991000	0.917071000	1.663312000
1	2.345768000	1.531036000	-0.704164000
6	0.681494000	2.530973000	-1.590189000
1	-0.246758000	1.828273000	-3.379099000
1	0.955747000	0.722792000	-2.717891000
6	1.072320000	-2.375841000	1.763195000
1	-0.619781000	-3.697594000	1.495975000
6	-0.690289000	-1.863399000	-2.309584000
6	1.201041000	-0.083543000	2.620171000
1	0.921947000	-4.350055000	1.075918000
6	0.313962000	-3.447689000	0.996376000
1	1.317113000	-2.771422000	2.753376000
1	2.012422000	-2.158387000	1.267511000
1	2.001765000	-0.575043000	3.174616000
1	0.365173000	-1.864470000	-2.554157000
1	-1.170915000	-2.664692000	-2.876616000
1	2.609064000	0.459806000	1.091213000
1	-2.404283000	-0.632767000	-2.662924000
1	-1.063973000	-0.326427000	-3.739669000
1	0.589763000	0.437442000	3.351205000
8	1.435962000	-0.694408000	-0.960168000
9	-1.826121000	0.115248000	0.736426000
6	-2.126002000	1.447785000	-1.667333000
1	-2.435241000	1.835525000	-2.640237000
1	-2.930601000	0.866505000	-1.234399000
1	-1.922436000	2.280561000	-1.007349000
6	-0.816055000	-1.347020000	2.836132000
1	-1.507517000	-2.041966000	2.378519000
1	-0.469854000	-1.761822000	3.785192000
1	-1.344554000	-0.418623000	3.013890000
6	-2.202561000	-2.500507000	-0.540128000
1	-2.484330000	-3.395558000	-1.098444000
1	-2.306166000	-2.698568000	0.519820000
1	-2.878408000	-1.691561000	-0.791300000
6	-0.072380000	2.401195000	1.417098000
1	-0.803256000	2.811657000	0.731679000
1	-0.610397000	1.893348000	2.208932000
1	0.514456000	3.217705000	1.841903000
8	2.719947000	-1.002417000	-0.516346000
8	3.517495000	-1.132611000	-1.497125000

E/h = -357.01587166
ZPE/h = 0.45787152
H_{133}/h = 0.46379465
S_{133}/h = 0.02273483

$[(D_{12}\text{-TMC})(F)Co^{III}(O_3)]^+$ (S = $^3/_2$)

27	-0.103653000	-0.309372000	-0.013390000
7	0.773003000	1.355411000	0.684298000
1	1.380970000	3.087827000	-2.217829000
1	-0.096767000	3.208951000	-1.330128000
7	-0.919897000	0.606296000	-1.870892000
6	1.495062000	2.094375000	-0.390052000
1	1.911119000	2.990320000	0.075615000
7	0.371380000	-1.137534000	1.974986000
6	0.063002000	-3.199764000	-0.461006000
1	2.151019000	1.759028000	2.227701000
7	-0.794564000	-2.033338000	-0.815451000
1	1.013854000	-3.059242000	-0.962598000
1	-0.414178000	-4.081364000	-0.894278000
6	0.156314000	1.399163000	-2.492122000
6	-1.317186000	-0.537131000	-2.722436000
6	1.780942000	0.894394000	1.673697000
1	2.330604000	1.482551000	-0.707573000
6	0.689488000	2.508876000	-1.603945000
1	-0.228643000	1.843657000	-3.415359000
1	0.963060000	0.721383000	-2.759413000
6	1.099133000	-2.403491000	1.773150000
1	-0.616202000	-3.698096000	1.531395000
6	-0.687361000	-1.834548000	-2.284955000
6	1.210822000	-0.114917000	2.639057000
1	0.900222000	-4.368384000	1.048506000
6	0.307863000	-3.453033000	1.012460000
1	1.365622000	-2.810316000	2.753758000
1	2.028113000	-2.186505000	1.251795000
1	2.031286000	-0.587739000	3.181419000
1	0.364654000	-1.854868000	-2.541449000
1	-1.175867000	-2.670232000	-2.789296000
1	2.619052000	0.478628000	1.133187000
1	-2.399440000	-0.620170000	-2.687744000
1	-1.051455000	-0.346449000	-3.763721000
1	0.598915000	0.391038000	3.379784000
8	1.472326000	-0.825342000	-0.984886000
9	-1.724408000	0.111523000	0.714575000
6	-2.109917000	1.450635000	-1.689367000
1	-2.452936000	1.812758000	-2.661900000
1	-2.895018000	0.875324000	-1.215014000
1	-1.897802000	2.303853000	-1.059874000
6	-0.795461000	-1.376454000	2.839579000
1	-1.483678000	-2.076563000	2.387959000
1	-0.461480000	-1.779429000	3.799098000
1	-1.328201000	-0.447246000	2.996671000
6	-2.194356000	-2.325780000	-0.462499000
1	-2.522564000	-3.199957000	-1.024773000
1	-2.280065000	-2.529876000	0.595894000
1	-2.827128000	-1.478780000	-0.690450000
6	-0.171820000	2.266020000	1.355686000
1	-0.932640000	2.599797000	0.664940000
1	-0.664448000	1.758731000	2.174250000
1	0.383144000	3.125751000	1.730765000
8	2.795943000	-0.790827000	-0.532505000
8	3.600651000	-0.891025000	-1.508881000

E/h = -357.02083462
ZPE/h = 0.45929612
H_{133}/h = 0.46495632
S_{133}/h = 0.02206507

[(D$_{12}$-TMC)(F)Co^{III}(O$_3$)]$^+$ TS O_2 extrusion (S = $^5/_2$)

27	-0.188149000	-0.278991000	0.018735000
7	0.802306000	1.491376000	0.735665000
1	1.343903000	3.108740000	-2.228287000
1	-0.123527000	3.215135000	-1.325706000
7	-0.920867000	0.585757000	-1.840711000
6	1.512834000	2.181466000	-0.361880000
1	1.944503000	3.102717000	0.040985000
7	0.331867000	-1.102954000	1.971839000
6	0.103519000	-3.244723000	-0.487262000
1	2.245753000	1.728552000	2.263038000
7	-0.808953000	-2.144875000	-0.867142000
1	1.057321000	-3.047894000	-0.966446000
1	-0.293749000	-4.173985000	-0.906848000
6	0.169027000	1.384138000	-2.444226000
6	-1.300088000	-0.556227000	-2.709802000
6	1.786557000	0.926620000	1.677960000
1	2.336041000	1.544888000	-0.669214000
6	0.681213000	2.529227000	-1.583403000
1	-0.204946000	1.802224000	-3.383976000
1	0.984881000	0.706248000	-2.679709000
6	1.083432000	-2.356941000	1.756476000
1	-0.596946000	-3.696393000	1.509818000
6	-0.667702000	-1.872660000	-2.311581000
6	1.170264000	-0.073385000	2.632766000
1	0.946251000	-4.339516000	1.082534000
6	0.330674000	-3.442287000	1.000699000
1	1.360577000	-2.754041000	2.738221000
1	2.004394000	-2.114541000	1.234751000
1	1.968613000	-0.551110000	3.203827000
1	0.391867000	-1.864799000	-2.540233000
1	-1.135450000	-2.674608000	-2.889380000
1	2.575361000	0.461342000	1.100813000
1	-2.381997000	-0.646962000	-2.677189000
1	-1.036076000	-0.337520000	-3.745961000
1	0.545009000	0.449693000	3.350626000
8	1.380396000	-0.714588000	-0.819571000
9	-1.809435000	0.126370000	0.740664000
6	-2.111754000	1.433937000	-1.674584000
1	-2.413530000	1.834438000	-2.645271000
1	-2.919502000	0.847950000	-1.254167000
1	-1.913727000	2.256625000	-1.000593000
6	-0.823909000	-1.369484000	2.841768000
1	-1.507998000	-2.066011000	2.375410000
1	-0.480775000	-1.788419000	3.791015000
1	-1.363288000	-0.447665000	3.021508000
6	-2.196338000	-2.502253000	-0.552102000
1	-2.491723000	-3.383336000	-1.126341000
1	-2.298666000	-2.719673000	0.504420000
1	-2.862931000	-1.679696000	-0.783351000
6	-0.087882000	2.424320000	1.434964000
1	-0.802364000	2.854257000	0.743356000
1	-0.647816000	1.908039000	2.206065000
1	0.497715000	3.229149000	1.884976000
8	3.054188000	-0.920474000	-0.584781000
8	3.565574000	-1.296976000	-1.611663000

E/h = -357.00291617
ZPE/h = 0.45548043
H_{133}/h = 0.46144920
S_{133}/h = 0.02283947

[(D$_{12}$-TMC)(F)Co^{III}(O$_3$)]$^+$ TS O$_2$ extrusion (S = $^3/_2$)

27	-0.191762000	-0.280906000	0.022034000
7	0.796020000	1.494060000	0.746565000
1	1.349734000	3.099937000	-2.221353000
1	-0.124889000	3.207376000	-1.330577000
7	-0.921365000	0.583573000	-1.840376000
6	1.507716000	2.185714000	-0.347727000
1	1.930676000	3.111768000	0.053730000
7	0.318139000	-1.105382000	1.980142000
6	0.105479000	-3.249953000	-0.480688000
1	2.252440000	1.712533000	2.265802000
7	-0.798670000	-2.143376000	-0.862542000
1	1.059396000	-3.063635000	-0.963767000
1	-0.302258000	-4.177090000	-0.894837000
6	0.181101000	1.369775000	-2.433551000
6	-1.294385000	-0.560317000	-2.707003000
6	1.777550000	0.917199000	1.684066000
1	2.339753000	1.557645000	-0.647179000
6	0.684067000	2.522604000	-1.577557000
1	-0.173452000	1.778840000	-3.384972000
1	0.997854000	0.685119000	-2.648160000
6	1.082788000	-2.349967000	1.759849000
1	-0.589369000	-3.700427000	1.518086000
6	-0.654419000	-1.872382000	-2.306450000
6	1.149926000	-0.072320000	2.641392000
1	0.956502000	-4.336218000	1.089886000
6	0.336316000	-3.442176000	1.007410000
1	1.370527000	-2.745479000	2.739330000
1	1.994843000	-2.092670000	1.227770000
1	1.941647000	-0.546657000	3.224789000
1	0.406116000	-1.858867000	-2.531485000
1	-1.115815000	-2.677811000	-2.884438000
1	2.553221000	0.433595000	1.102817000
1	-2.375713000	-0.657685000	-2.673473000
1	-1.032595000	-0.342768000	-3.744217000
1	0.518161000	0.457859000	3.348349000
8	1.353782000	-0.737155000	-0.730596000
9	-1.814997000	0.151009000	0.742633000
6	-2.108674000	1.437393000	-1.688454000
1	-2.399097000	1.839896000	-2.662040000
1	-2.923548000	0.855200000	-1.276350000
1	-1.914111000	2.258238000	-1.011112000
6	-0.835823000	-1.379591000	2.847980000
1	-1.513774000	-2.082720000	2.382379000
1	-0.493213000	-1.793401000	3.799899000
1	-1.382976000	-0.461295000	3.022831000
6	-2.188938000	-2.491404000	-0.547135000
1	-2.493069000	-3.363936000	-1.129660000
1	-2.288950000	-2.719601000	0.507257000
1	-2.848889000	-1.660449000	-0.766462000
6	-0.090131000	2.427669000	1.448453000
1	-0.794662000	2.870593000	0.754357000
1	-0.662248000	1.908830000	2.208641000
1	0.498400000	3.223195000	1.911481000
8	3.138159000	-0.733153000	-0.822848000
8	3.511432000	-1.420609000	-1.731399000

E/h = -356.99375546
ZPE/h = 0.45538687
H_{133}/h = 0.46137974
S_{133}/h = 0.02276106

[(D$_{12}$-TMC)(F)Co^{IV}(O)]$^+$ (S = $^5/_2$)

27	-3.157612000	0.860675000	1.756759000
7	-1.753749000	2.394948000	2.305021000
1	-1.228637000	3.921587000	-0.706852000
1	-2.556115000	4.276037000	0.338409000
7	-3.859486000	1.838900000	-0.074891000
6	-1.039089000	2.958914000	1.139967000
1	-0.413655000	3.783275000	1.495662000
7	-2.504912000	-0.090978000	3.627932000
6	-3.026479000	-2.112713000	1.107605000
1	-0.165322000	2.410589000	3.700559000
7	-3.886660000	-0.941500000	0.834662000
1	-2.088296000	-1.959162000	0.579942000
1	-3.507102000	-2.993799000	0.672025000
6	-2.704289000	2.431081000	-0.781839000
6	-4.451026000	0.749636000	-0.882979000
6	-0.801957000	1.686603000	3.184363000
1	-0.374941000	2.186076000	0.760578000
6	-1.914231000	3.461996000	0.006936000
1	-3.080694000	2.907790000	-1.692606000
1	-2.046082000	1.619554000	-1.080622000
6	-1.864761000	-1.385497000	3.312516000
1	-3.651797000	-2.592512000	3.121405000
6	-3.846172000	-0.607394000	-0.603620000
6	-1.513804000	0.839000000	4.212934000
1	-2.184931000	-3.328466000	2.587159000
6	-2.735522000	-2.386628000	2.571362000
1	-1.547263000	-1.838823000	4.256968000
1	-0.972719000	-1.179402000	2.726968000
1	-0.771175000	0.281234000	4.788224000
1	-2.808250000	-0.633861000	-0.921493000
1	-4.393082000	-1.362538000	-1.174760000
1	-0.166728000	1.069201000	2.556771000
1	-5.517264000	0.727647000	-0.675908000
1	-4.344341000	0.973832000	-1.946947000
1	-2.036466000	1.477989000	4.919134000
8	-1.604324000	0.257465000	0.884138000
9	-4.669071000	1.426482000	2.599240000
6	-4.893104000	2.861195000	0.136163000
1	-5.228999000	3.256638000	-0.825685000
1	-5.731547000	2.422886000	0.665289000
1	-4.511044000	3.679194000	0.734720000
6	-3.576241000	-0.290340000	4.612482000
1	-4.343163000	-0.948081000	4.222146000
1	-3.166903000	-0.728899000	5.526183000
1	-4.038782000	0.663348000	4.839326000
6	-5.267047000	-1.218603000	1.253758000
1	-5.665484000	-2.052555000	0.671724000
1	-5.297298000	-1.476295000	2.305494000
1	-5.891149000	-0.344479000	1.110889000
6	-2.436077000	3.461532000	3.049701000
1	-3.132377000	3.984554000	2.405743000
1	-2.999265000	3.046020000	3.877072000
1	-1.698791000	4.172574000	3.429340000

E/h = -325.04749570
ZPE/h = 0.45011015
H_{133}/h = 0.45520172
S_{133}/h = 0.02106840

[(D$_{12}$-TMC)(F)Co^{IV}(O)]$^+$ (S = $^3/_2$)

27	-2.984400000	0.792885000	1.662835000
7	-1.818391000	2.351200000	2.296602000
1	-1.249317000	3.993376000	-0.659100000
1	-2.620790000	4.284616000	0.350520000
7	-3.824800000	1.831707000	-0.136903000
6	-1.100307000	2.976927000	1.157529000
1	-0.507119000	3.801751000	1.560933000
7	-2.445736000	-0.129196000	3.633226000
6	-3.070663000	-2.089337000	1.147608000
1	-0.206229000	2.449801000	3.659307000
7	-3.872966000	-0.870448000	0.872636000
1	-2.132384000	-1.983461000	0.608802000
1	-3.604697000	-2.936182000	0.708936000
6	-2.691355000	2.470124000	-0.824300000
6	-4.398477000	0.739180000	-0.942690000
6	-0.813897000	1.690156000	3.161997000
1	-0.404587000	2.235753000	0.771888000
6	-1.949598000	3.496980000	0.014540000
1	-3.064757000	2.962237000	-1.729097000
1	-2.003063000	1.687446000	-1.136620000
6	-1.848992000	-1.441101000	3.337617000
1	-3.687573000	-2.569089000	3.164004000
6	-3.830959000	-0.613941000	-0.585853000
6	-1.453055000	0.800820000	4.202040000
1	-2.265526000	-3.363810000	2.590687000
6	-2.772072000	-2.397470000	2.601572000
1	-1.544630000	-1.907101000	4.281212000
1	-0.949930000	-1.276012000	2.747455000
1	-0.666685000	0.251099000	4.724662000
1	-2.793470000	-0.683136000	-0.899757000
1	-4.392828000	-1.392090000	-1.107496000
1	-0.159705000	1.109031000	2.518669000
1	-5.474284000	0.738115000	-0.791895000
1	-4.233849000	0.919978000	-2.007551000
1	-1.953062000	1.411042000	4.948871000
8	-1.616108000	0.263743000	0.890127000
9	-4.483084000	1.372006000	2.514753000
6	-4.879884000	2.815294000	0.122571000
1	-5.248135000	3.227827000	-0.821128000
1	-5.694324000	2.340717000	0.657525000
1	-4.512902000	3.628200000	0.735816000
6	-3.540561000	-0.275877000	4.596142000
1	-4.320397000	-0.917819000	4.207405000
1	-3.164102000	-0.706242000	5.528692000
1	-3.981161000	0.694255000	4.792400000
6	-5.260257000	-1.068872000	1.311956000
1	-5.701417000	-1.892808000	0.748895000
1	-5.287221000	-1.304086000	2.367926000
1	-5.837208000	-0.165647000	1.160943000
6	-2.548424000	3.372063000	3.058554000
1	-3.260857000	3.876680000	2.419189000
1	-3.099695000	2.913790000	3.869626000
1	-1.839382000	4.100849000	3.454790000

E/h = -325.06590260
ZPE/h = 0.45929612
H_{133}/h = 0.46495632
S_{133}/h = 0.02206507

[(D$_{12}$-TMC)(OTf)Co^{II}]$^{+}$ (S = $^{3}/_{2}$)

27	-0.626942000	-0.191232000	0.141632000
7	0.706610000	1.362627000	0.722906000
1	1.632909000	2.687581000	-2.287650000
1	0.190337000	3.109803000	-1.431609000
7	-1.058344000	0.638812000	-1.829334000
6	1.573858000	1.810558000	-0.387910000
1	2.211903000	2.617373000	-0.014760000
7	0.135151000	-1.145902000	2.019609000
6	0.412468000	-2.997451000	-0.613704000
1	1.999236000	1.684652000	2.364296000
7	-0.717902000	-2.086740000	-0.888720000
1	1.309321000	-2.528258000	-1.018413000
1	0.249341000	-3.923321000	-1.174180000
6	0.173430000	1.187960000	-2.432795000
6	-1.511419000	-0.521922000	-2.620074000
6	1.566092000	0.851508000	1.805630000
1	2.225900000	0.975369000	-0.645489000
6	0.859701000	2.277214000	-1.637019000
1	-0.077382000	1.569126000	-3.427626000
1	0.877574000	0.369266000	-2.572372000
6	1.149951000	-2.163148000	1.676881000
1	-0.209613000	-3.806486000	1.296613000
6	-0.696779000	-1.753265000	-2.325945000
6	0.790820000	-0.038744000	2.738707000
1	1.442700000	-4.074559000	0.855251000
6	0.652405000	-3.322954000	0.841986000
1	1.573684000	-2.546057000	2.611078000
1	1.960156000	-1.675122000	1.136920000
1	1.460410000	-0.429677000	3.510462000
1	0.339053000	-1.604454000	-2.620555000
1	-1.077906000	-2.593876000	-2.911262000
1	2.391844000	0.309810000	1.351918000
1	-2.558316000	-0.693967000	-2.382496000
1	-1.459074000	-0.293474000	-3.688152000
1	0.016020000	0.530220000	3.246271000
8	-2.516977000	0.183596000	0.810717000
9	-3.835742000	2.808870000	0.834818000
16	-3.410199000	0.480293000	1.959126000
8	-2.746571000	1.171243000	3.045656000
8	-4.276916000	-0.624899000	2.300973000
6	-4.530391000	1.748072000	1.227133000
9	-5.174187000	1.251958000	0.178185000
9	-5.420079000	2.132720000	2.129899000
6	-2.124283000	1.649023000	-1.873078000
1	-2.290530000	1.971373000	-2.904143000
1	-3.040006000	1.220915000	-1.482001000
1	-1.868326000	2.515957000	-1.276123000
6	-0.854905000	-1.741546000	2.927459000
1	-1.463754000	-2.479313000	2.419687000
1	-0.349670000	-2.224712000	3.768181000
1	-1.504638000	-0.968153000	3.318020000
6	-1.992551000	-2.747944000	-0.564719000
1	-2.076074000	-3.685997000	-1.118436000
1	-2.057398000	-2.956112000	0.495813000
1	-2.826382000	-2.104646000	-0.824909000
6	-0.094122000	2.489679000	1.229639000
1	-0.803575000	2.823702000	0.482206000
1	-0.657875000	2.185403000	2.104591000
1	0.562596000	3.322101000	1.492168000

E/h = -421.84007449
ZPE/h = 0.47514122
H_{133}/h = 0.48234999
S_{133}/h = 0.02542586

[(D12-TMC)(F)Co^II]^+ (S = $^3/_2$)

27	-0.653459000	-0.096284000	0.203484000
7	0.820257000	1.368484000	0.705718000
1	1.623366000	2.745647000	-2.314808000
1	0.246131000	3.176147000	-1.361218000
7	-1.086531000	0.740982000	-1.778420000
6	1.638221000	1.792078000	-0.448028000
1	2.347282000	2.552028000	-0.103509000
7	0.161793000	-1.110391000	2.061892000
6	0.280147000	-2.957733000	-0.555295000
1	2.266541000	1.560999000	2.236455000
7	-0.804817000	-1.996067000	-0.824961000
1	1.202313000	-2.519318000	-0.937975000
1	0.086770000	-3.865148000	-1.137676000
6	0.104451000	1.302870000	-2.439383000
6	-1.574353000	-0.412052000	-2.553407000
6	1.708620000	0.775892000	1.718318000
1	2.221846000	0.926954000	-0.765194000
6	0.874047000	2.331942000	-1.638496000
1	-0.207867000	1.744594000	-3.391955000
1	0.778204000	0.480883000	-2.677050000
6	1.061177000	-2.237619000	1.767799000
1	-0.420326000	-3.755914000	1.330048000
6	-0.781210000	-1.660217000	-2.259209000
6	0.932334000	-0.043768000	2.716007000
1	1.209625000	-4.144270000	0.900344000
6	0.482473000	-3.331020000	0.896209000
1	1.393604000	-2.672967000	2.717324000
1	1.948224000	-1.843062000	1.273910000
1	1.620566000	-0.465311000	3.455689000
1	0.257942000	-1.526128000	-2.551936000
1	-1.175773000	-2.493175000	-2.848158000
1	2.436244000	0.155744000	1.200056000
1	-2.621736000	-0.561897000	-2.303315000
1	-1.532301000	-0.195150000	-3.624870000
1	0.234439000	0.586948000	3.260800000
9	-2.283571000	0.359551000	1.045357000
6	-2.155515000	1.743399000	-1.717959000
1	-2.466337000	2.029640000	-2.726614000
1	-2.999851000	1.331313000	-1.174559000
1	-1.819298000	2.632405000	-1.196469000
6	-0.898833000	-1.534839000	2.978976000
1	-1.466056000	-2.358006000	2.559796000
1	-0.471783000	-1.860851000	3.932417000
1	-1.579702000	-0.706424000	3.143611000
6	-2.106330000	-2.585188000	-0.480968000
1	-2.254770000	-3.518290000	-1.030992000
1	-2.165254000	-2.785345000	0.581433000
1	-2.907471000	-1.896026000	-0.725447000
6	0.119490000	2.523164000	1.285180000
1	-0.591517000	2.935318000	0.579358000
1	-0.436614000	2.218499000	2.164748000
1	0.838889000	3.298571000	1.559986000

E/h = -309.12090852
ZPE/h = 0.44620811
H_{133}/h = 0.45161473
S_{133}/h = 0.02156459

$\{[(D_{12}\text{-TMC})(OTf)Co^{III}]_2(O_3)\}^{2+}$ (S = 0)

27	1.843832000	1.727467000	-2.096733000
7	3.568317000	2.273297000	-3.015504000
1	3.517279000	0.360223000	-5.867829000
1	2.785450000	1.925698000	-5.777093000
7	0.884830000	1.189934000	-3.871283000
6	4.003480000	1.337037000	-4.087647000
1	4.913138000	1.755660000	-4.522836000
7	2.716494000	2.482362000	-0.354662000
6	0.622273000	0.181760000	0.099271000
1	5.527858000	2.561047000	-2.278410000
7	0.234217000	0.895488000	-1.145038000
1	1.262766000	-0.642350000	-0.187173000
1	-0.289814000	-0.241008000	0.525053000
6	1.753863000	0.341525000	-4.724760000
6	-0.342271000	0.433772000	-3.482825000
6	4.578391000	2.143057000	-1.939297000
1	4.261813000	0.410587000	-3.594783000
6	3.011042000	1.042755000	-5.183502000
1	1.164921000	0.051131000	-5.595972000
1	2.003731000	-0.553905000	-4.173863000
6	2.701962000	1.496226000	0.754134000
1	0.697217000	1.831317000	1.494193000
6	-0.246752000	-0.135448000	-2.099644000
6	4.126105000	2.834531000	-0.693317000
1	1.457861000	0.364671000	2.008988000
6	1.323672000	1.014270000	1.143010000
1	3.167407000	1.974980000	1.617502000
1	3.330649000	0.667469000	0.465526000
1	4.781070000	2.570741000	0.136258000
1	0.445371000	-0.965525000	-2.073979000
1	-1.225965000	-0.494241000	-1.778534000
1	4.718986000	1.088818000	-1.754237000
1	-1.184699000	1.114104000	-3.550504000
1	-0.522016000	-0.365367000	-4.201173000
1	4.185497000	3.911532000	-0.811878000
8	2.477562000	-0.057526000	-2.094033000
6	0.421379000	2.305914000	-4.732522000
1	0.049541000	1.884373000	-5.666827000
1	-0.376479000	2.844861000	-4.249848000
1	1.221034000	2.994915000	-4.955318000
6	2.120575000	3.724123000	0.191786000
1	1.078954000	3.592330000	0.436129000
1	2.666823000	3.990191000	1.097263000
1	2.212400000	4.529334000	-0.519177000
6	-0.888024000	1.801539000	-0.836208000
1	-1.673146000	1.234527000	-0.335600000
1	-0.570346000	2.605288000	-0.186683000
1	-1.292615000	2.226988000	-1.744761000
6	3.601913000	3.651006000	-3.539418000
1	2.902001000	3.765753000	-4.354833000
1	3.346965000	4.359758000	-2.764472000
1	4.606471000	3.864019000	-3.905206000
8	3.442814000	-0.568351000	-1.203874000
8	4.356240000	-1.316207000	-1.969031000
27	4.991078000	-3.046589000	-1.523415000
7	6.670652000	-2.010333000	-0.969372000
1	5.682470000	-0.577767000	1.981540000
1	6.406453000	-2.145587000	1.860285000
7	4.262379000	-3.259027000	0.417518000
6	6.385774000	-0.965752000	0.048863000
1	7.332436000	-0.470471000	0.272879000
7	5.806111000	-3.046766000	-3.446457000
6	2.676430000	-3.182608000	-3.371619000
1	8.067913000	-0.881351000	-2.083209000
7	3.201486000	-3.793894000	-2.119433000
1	2.446767000	-2.153585000	-3.133716000
1	1.739770000	-3.693108000	-3.604272000
6	4.356226000	-1.996509000	1.191943000
6	2.832879000	-3.670654000	0.304735000
6	7.069198000	-1.301369000	-2.212563000
1	5.733288000	-0.234421000	-0.410444000
6	5.757932000	-1.450670000	1.331820000
1	3.953918000	-2.197921000	2.186325000
1	3.709011000	-1.272755000	0.720604000
6	4.870438000	-2.444281000	-4.429085000

1	3.753144000	-4.227557000	-4.930324000
6	2.278794000	-3.353784000	-1.047009000
6	7.060857000	-2.242190000	-3.378830000
1	3.018947000	-2.727362000	-5.380537000
6	3.578274000	-3.213256000	-4.579491000
1	5.385409000	-2.418478000	-5.390650000
1	4.669159000	-1.426543000	-4.127347000
1	7.188885000	-1.684886000	-4.306361000
1	2.143923000	-2.288502000	-1.155540000
1	1.309503000	-3.837394000	-1.180319000
1	6.374527000	-0.488385000	-2.372164000
1	2.771735000	-4.735739000	0.502336000
1	2.244077000	-3.171019000	1.073298000
1	7.893711000	-2.934024000	-3.309417000
6	4.911467000	-4.302515000	1.246842000
1	4.451614000	-4.282080000	2.235451000
1	4.759816000	-5.277182000	0.811245000
1	5.970827000	-4.130158000	1.347622000
6	6.200212000	-4.364003000	-4.003665000
1	5.377344000	-5.061514000	-3.991690000
1	6.517905000	-4.214864000	-5.036199000
1	7.020745000	-4.778608000	-3.442587000
6	3.134611000	-5.262338000	-2.235679000
1	2.105624000	-5.551507000	-2.449326000
1	3.770098000	-5.611224000	-3.037513000
1	3.452570000	-5.732753000	-1.316071000
6	7.817046000	-2.814502000	-0.505853000
1	7.559544000	-3.384370000	0.376531000
1	8.139768000	-3.497261000	-1.279722000
1	8.643071000	-2.145883000	-0.262605000
8	0.978621000	3.579861000	-2.330799000
6	0.794715000	6.048879000	-3.115287000
9	1.907276000	6.389438000	-2.475113000
8	-0.279992000	5.155556000	-0.917624000
16	-0.047492000	4.652140000	-2.251934000
8	-1.223387000	4.396194000	-3.056537000
9	1.106178000	5.715851000	-4.360712000
9	-0.014794000	7.094501000	-3.144051000
8	5.843385000	-4.910151000	-1.292644000
6	6.010445000	-7.502970000	-1.429341000
9	4.935522000	-7.671818000	-0.668870000
8	7.185380000	-6.077516000	0.409206000
16	6.879674000	-5.932995000	-0.995411000
8	8.009510000	-5.897415000	-1.900323000
9	5.634345000	-7.497834000	-2.701395000
9	6.832203000	-8.521226000	-1.233352000

E/h = -891.549741778288
ZPE/h = 0.97489739
H_{133}/h = 0.98741224
S_{133}/h = 0.03494909

{[(D_{12}-TMC)(F)Co^{III}]$_2$(O_3)}$^{2+}$ (S = 0)

27	1.813086000	1.715507000	-2.084219000
7	3.492170000	2.252073000	-3.074318000
1	3.260461000	0.383979000	-5.940232000
1	2.572189000	1.964107000	-5.781495000
7	0.744681000	1.209977000	-3.794486000
6	3.859509000	1.315087000	-4.165415000
1	4.760632000	1.710432000	-4.640979000
7	2.738646000	2.512814000	-0.401626000
6	0.610582000	0.286976000	0.197296000
1	5.482307000	2.534215000	-2.422853000
7	0.217460000	0.959265000	-1.063521000
1	1.231504000	-0.559749000	-0.075512000
1	-0.300421000	-0.099746000	0.660950000
6	1.552745000	0.382162000	-4.718069000
6	-0.457529000	0.443224000	-3.356983000
6	4.545517000	2.118901000	-2.044354000
1	4.106596000	0.375916000	-3.687201000
6	2.806541000	1.063710000	-5.217164000
1	0.920746000	0.127665000	-5.571840000
1	1.808002000	-0.534008000	-4.199646000
6	2.730750000	1.586408000	0.753841000
1	0.751144000	1.982243000	1.533074000
6	-0.303603000	-0.092841000	-1.965052000
6	4.147440000	2.821869000	-0.781352000
1	1.500387000	0.520465000	2.078555000
6	1.354363000	1.142443000	1.194048000
1	3.223084000	2.094679000	1.586279000
1	3.334416000	0.729349000	0.486586000
1	4.820311000	2.535668000	0.026331000
1	0.401989000	-0.913048000	-1.952804000
1	-1.266049000	-0.451080000	-1.593919000
1	4.688273000	1.063458000	-1.859677000
1	-1.315549000	1.105435000	-3.412272000
1	-0.645505000	-0.375647000	-4.050641000
1	4.237836000	3.897265000	-0.899212000
8	2.446177000	-0.096053000	-2.067626000
6	0.232492000	2.371657000	-4.555814000
1	-0.316271000	2.000178000	-5.422586000
1	-0.420269000	2.962800000	-3.929749000
1	1.036063000	3.007917000	-4.893614000
6	2.147847000	3.791550000	0.055157000
1	1.113212000	3.667707000	0.336980000
1	2.714781000	4.139728000	0.919710000
1	2.193286000	4.525581000	-0.736012000
6	-0.856427000	1.929210000	-0.791402000
1	-1.710083000	1.404898000	-0.359520000
1	-0.520901000	2.685754000	-0.096277000
1	-1.154548000	2.430149000	-1.702690000
6	3.484821000	3.631875000	-3.587383000
1	2.770605000	3.734762000	-4.392405000
1	3.202611000	4.326867000	-2.808193000
1	4.479276000	3.876764000	-3.962715000
8	3.450875000	-0.553951000	-1.188171000
8	4.396836000	-1.259240000	-1.958671000
27	5.026553000	-3.016584000	-1.504158000
7	6.692535000	-2.034597000	-0.855612000
1	5.634121000	-0.687541000	2.109557000
1	6.343782000	-2.259375000	1.962858000
7	4.234199000	-3.286668000	0.397659000
6	6.399168000	-1.024141000	0.188681000
1	7.345918000	-0.551536000	0.461720000
7	5.960208000	-3.030932000	-3.358287000
6	2.823475000	-3.185570000	-3.457190000
1	8.131749000	-0.863577000	-1.868358000
7	3.278207000	-3.779625000	-2.176064000
1	2.607136000	-2.145430000	-3.249675000
1	1.891418000	-3.684965000	-3.733556000
6	4.318624000	-2.066082000	1.231923000
6	2.804621000	-3.671757000	0.225210000
6	7.142787000	-1.287993000	-2.052502000
1	5.770110000	-0.268210000	-0.269558000
6	5.721064000	-1.540448000	1.434806000
1	3.884739000	-2.302165000	2.206535000
1	3.697557000	-1.312677000	0.766058000
6	5.078934000	-2.487670000	-4.417080000

1	3.991361000	-4.291727000	-4.903742000
6	2.310366000	-3.337993000	-1.149271000
6	7.187251000	-2.190643000	-3.249252000
1	3.287226000	-2.812696000	-5.463181000
6	3.795142000	-3.263082000	-4.608826000
1	5.643616000	-2.496199000	-5.352121000
1	4.856971000	-1.458520000	-4.162300000
1	7.309954000	-1.597049000	-4.154530000
1	2.190097000	-2.269306000	-1.256416000
1	1.344482000	-3.816418000	-1.326568000
1	6.440747000	-0.481204000	-2.215607000
1	2.716548000	-4.737145000	0.413604000
1	2.191879000	-3.166032000	0.970829000
1	8.044837000	-2.853154000	-3.189738000
6	4.867428000	-4.389285000	1.156410000
1	4.368473000	-4.469930000	2.123390000
1	4.771272000	-5.317129000	0.611739000
1	5.918698000	-4.203404000	1.314998000
6	6.418286000	-4.369721000	-3.791077000
1	5.593963000	-5.062183000	-3.865485000
1	6.893698000	-4.272443000	-4.768376000
1	7.123247000	-4.766846000	-3.075064000
6	3.246258000	-5.248097000	-2.273038000
1	2.220179000	-5.568613000	-2.457910000
1	3.871784000	-5.588519000	-3.086854000
1	3.616059000	-5.698245000	-1.361811000
6	7.783783000	-2.911792000	-0.400279000
1	7.502703000	-3.432857000	0.504016000
1	8.008384000	-3.656965000	-1.151787000
1	8.669519000	-2.307207000	-0.200058000
9	1.043692000	3.363117000	-2.267990000
9	5.780773000	-4.664808000	-1.266487000

E/h = -666.155273101938
ZPE/h = 0.91574874
H_{133}/h = 0.92507374
S_{133}/h = 0.02885465

H. Miscellaneous cartesian coordinates and thermochemical data

O_2 (S = 1)

```
8        0.000000000      0.000000000      0.599066000
8        0.000000000      0.000000000     -0.599066000
```

E/h = -31.98227097
ZPE/h = 0.00403164
H_{133}/h = 0.00550579
S_{133}/h = 0.00918504

I. Molecular orbitals and spin densities for $[(D_{12}\text{-TMC})(OTf)Fe^{III}(O_3)]^+$

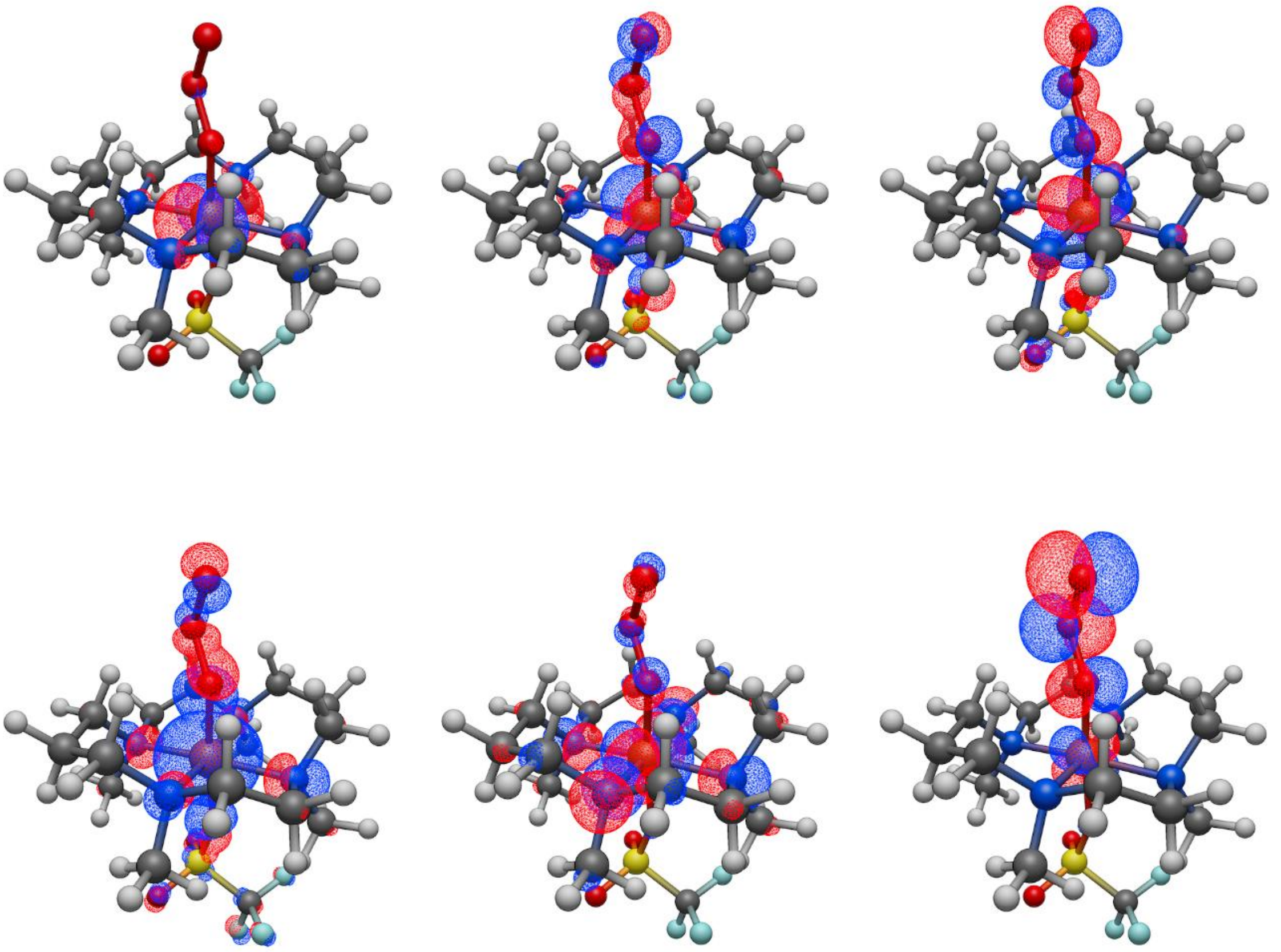

Figure S1: Quasi-restricted orbitals of $[(D_{12}\text{-TMC})(OTf)Fe^{III}(O_3)]^+$ in the septet (*S* = 3) state. From left to right and top to bottom: HOMO–5 to HOMO (spin-up, singly occupied).

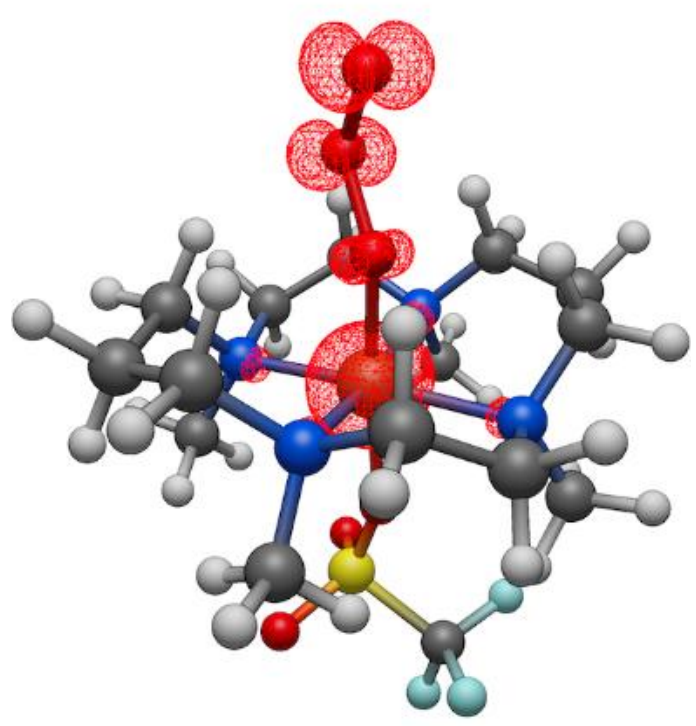

Figure S2: Spin density of $[(D_{12}\text{-TMC})(OTf)Fe^{III}(O_3)]^+$ in the septet (*S* = 3) state.

J. Molecular orbitals and spin densities for $[(D_{12}\text{-TMC})(X)Co^{III}(O_3)]^+$ (X = OTf, F)

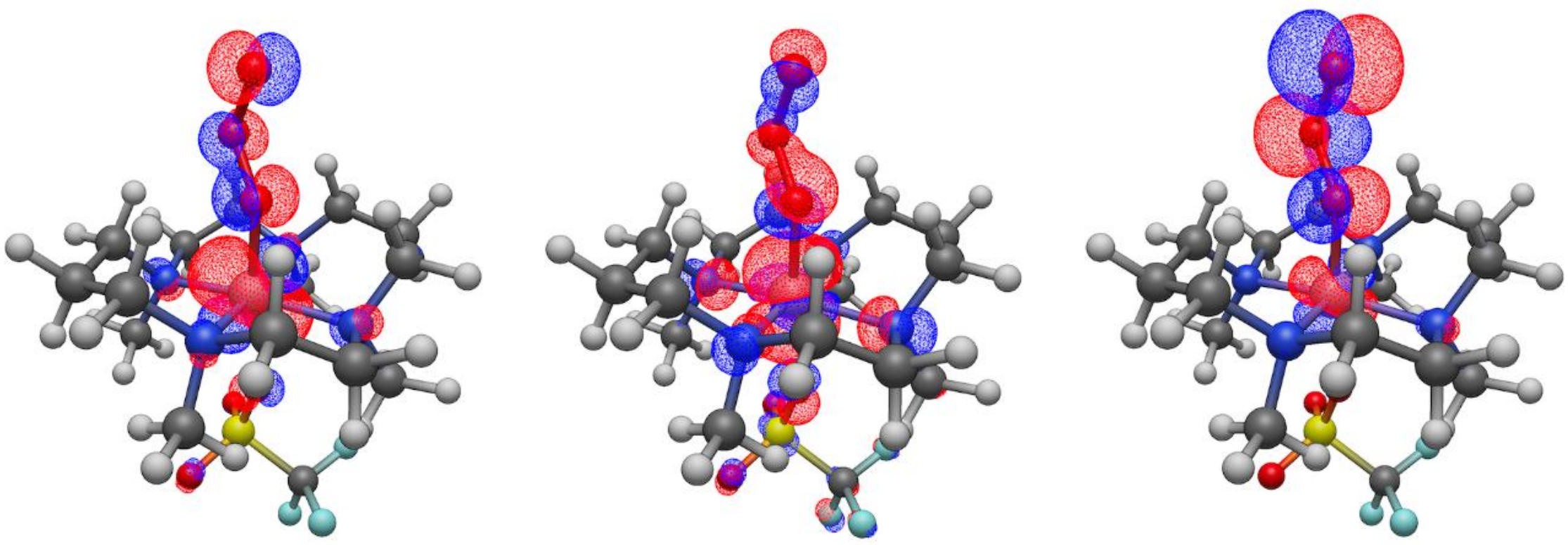

Figure S3: Quasi-restricted orbitals of $[(D_{12}\text{-TMC})(OTf)Co^{III}(O_3)]^+$ in the quartet (S = $^3/_2$) state. From left to right: HOMO–2 to HOMO (spin-up, singly occupied).

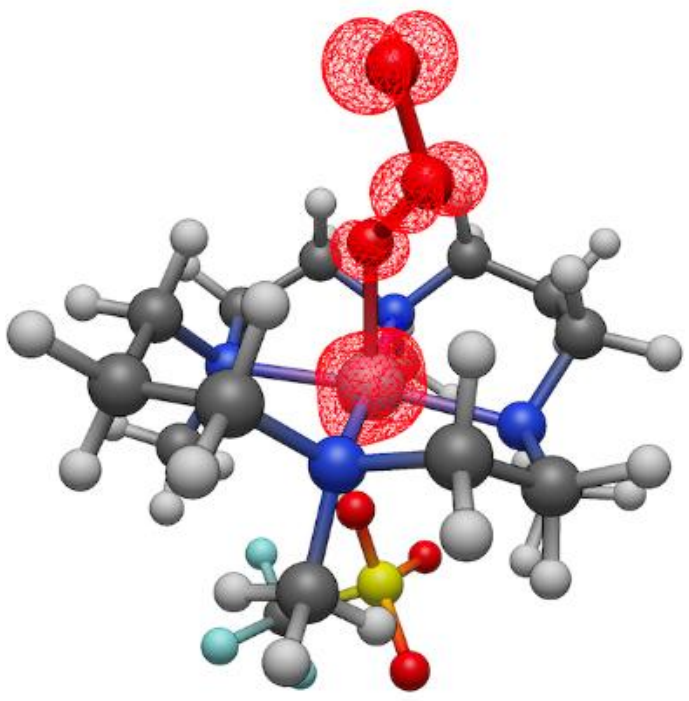

Figure S4: Spin density of $[(D_{12}\text{-TMC})(OTf)Co^{III}(O_3)]^+$ in the quartet (S = $^3/_2$) state.

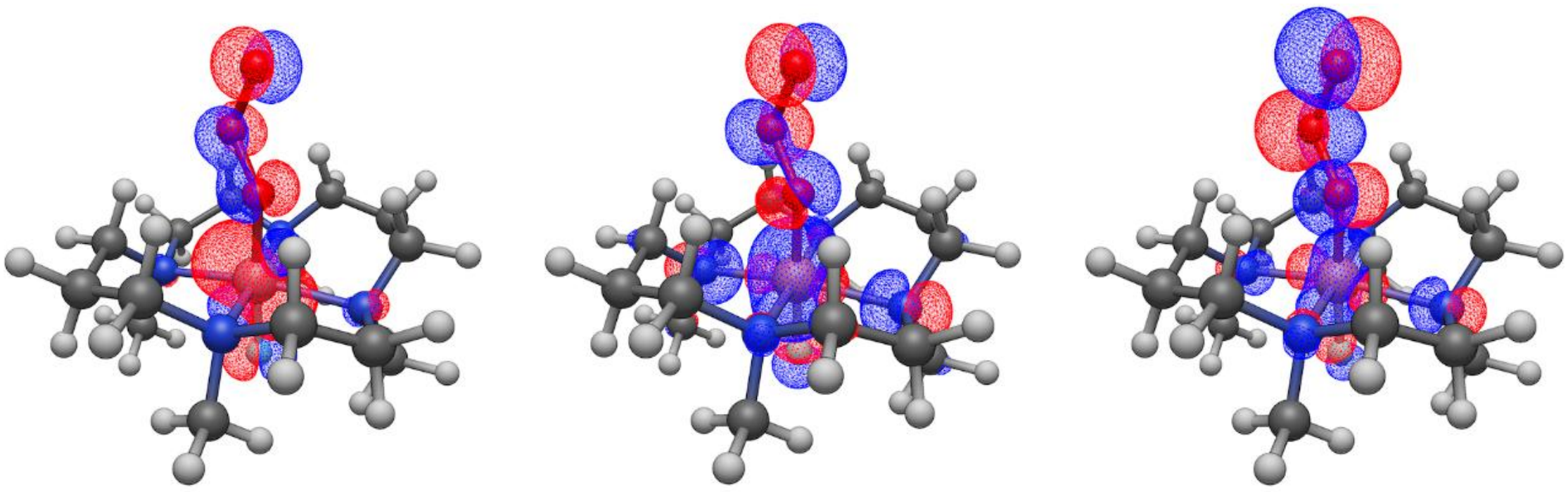

Figure S5: Quasi-restricted orbitals of $[(D_{12}\text{-TMC})(F)Co^{III}(O_3)]^+$ in the quartet ($S = {}^3/_2$) state. From left to right: HOMO–2 to HOMO (spin-up, singly occupied).

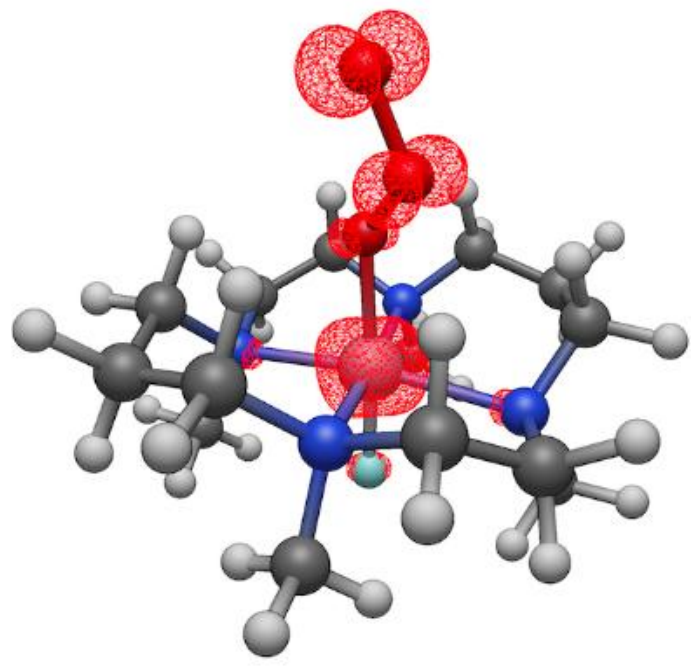

Figure S6: Spin density of $[(D_{12}\text{-TMC})(F)Co^{III}(O_3)]^+$ in the quartet ($S = {}^3/_2$) state.

References

[1] F. Neese, “Software Update: The ORCA Program System—Version 5.0” *WIREs Comput. Mol. Sci.* **2022**, *12*, e1606.

[2] F. Neese, F. Wennmohs, U. Becker, C. Riplinger, “The ORCA Quantum Chemistry Program Package” *J. Chem. Phys.* **2020**, *152*, 224108.

[3] M. Müller, A. Hansen, S. Grimme, “ωB97X-3c: A composite range-separated hybrid DFT method with a molecule-optimized polarized valence double-ζ basis set” *J. Chem. Phys.* **2023**, *158*, 014103.

[4] E. Caldeweyher, S. Ehlert, A. Hansen, H. Neugebauer, S. Spicher, C. Bannwarth, S. Grimme, “A generally applicable atomic-charge dependent London dispersion correction” *J. Chem. Phys.* **2019**, *150*, 154122.

[5] J. Zheng, X. Xu, D. G. Truhlar, “Minimally augmented Karlsruhe basis sets” *Theor. Chem. Acc.* **2011**, *128*, 295–305.

[6] S. Hirata, M. Head-Gordon, “Time-dependent density functional theory within the Tamm–Dancoff approximation” *Chem. Phys. Lett.* **1999**, *314*, 291–299.
