# Supplementary material for: First Evidence for Ozonido‐TMC Complexes of Iron and Cobalt
Source: Angew Chem Int Ed Engl. 2026 Jul 9;65(37):e4716492. doi: 10.1002/anie.4716492 (PMC13548893; doi:10.1002/anie.4716492)
Supplement: Supplementary file 2 [file ANIE-65-e4716492-s002.pdf]

Supporting Information Part III — UV-vis spectroscopy

A. General Procedures **2**

B. Additional UV-vis spectra for Fe complexes **3**

C. Additional UV-vis spectra for Co complexes **4**

D. Kinetics of the Fe ozonido to oxido complex conversion **6**

A. General Procedures

All UV-Vis spectra were measured on a Jasco V–760 spectrometer in a range of 190 to 900 nm, a resolution of 5 nm, and a scan speed of 2000 nm/min. The sample and lamp compartments were continuously purged with dry nitrogen during operation to prevent the condensation of moisture. Experiments were performed in a spectroscopic Dewar vessel equipped with three quartz windows. A four-sided quartz cuvette (10 mm optical path diameter) attached to a cylindrical glass tubing with silicon septa for Schlenk procedures was mounted inside the Dewar vessel. The experimental setup was mounted within the sample compartment of the spectrometer and aligned for optimal optical transmission. Black theater cloth was used to shield the assembly from ambient light.

During the experiments, the cuvette was cooled by a cold and dry nitrogen stream from a custom-made liquid nitrogen evaporator with adjustable flow. The temperature was measured by two Ni/Cr-Ni thermocouples: One was attached to the top of the cuvette's windows, the other at the bottom of the Dewar vessel. Two Teflon tubes were fed through one of the cuvette's silicone septa, one of which was used to maintain a permanent argon atmosphere above the sample solution. The second tube reached into the solution and was used to apply a dry ozone-oxygen mixture from a lab ozonizer supplied with oxygen of 5.0 quality. Both argon and oxygen-ozone gas streams were run through a cold trap held at –90 °C before entering the cuvette. Additional sample or reagent solutions could be transferred via syringe through a second silicone septum.

This setup was necessary to prevent contact between the metal cannula and ozone-containing solutions at all stages of the experiment. The argon atmosphere in the cuvette effectively suppressed the formation of combustible gas mixtures, which may even develop at the low temperatures attained during measurements.

B. Additional UV-vis spectra for Fe complexes

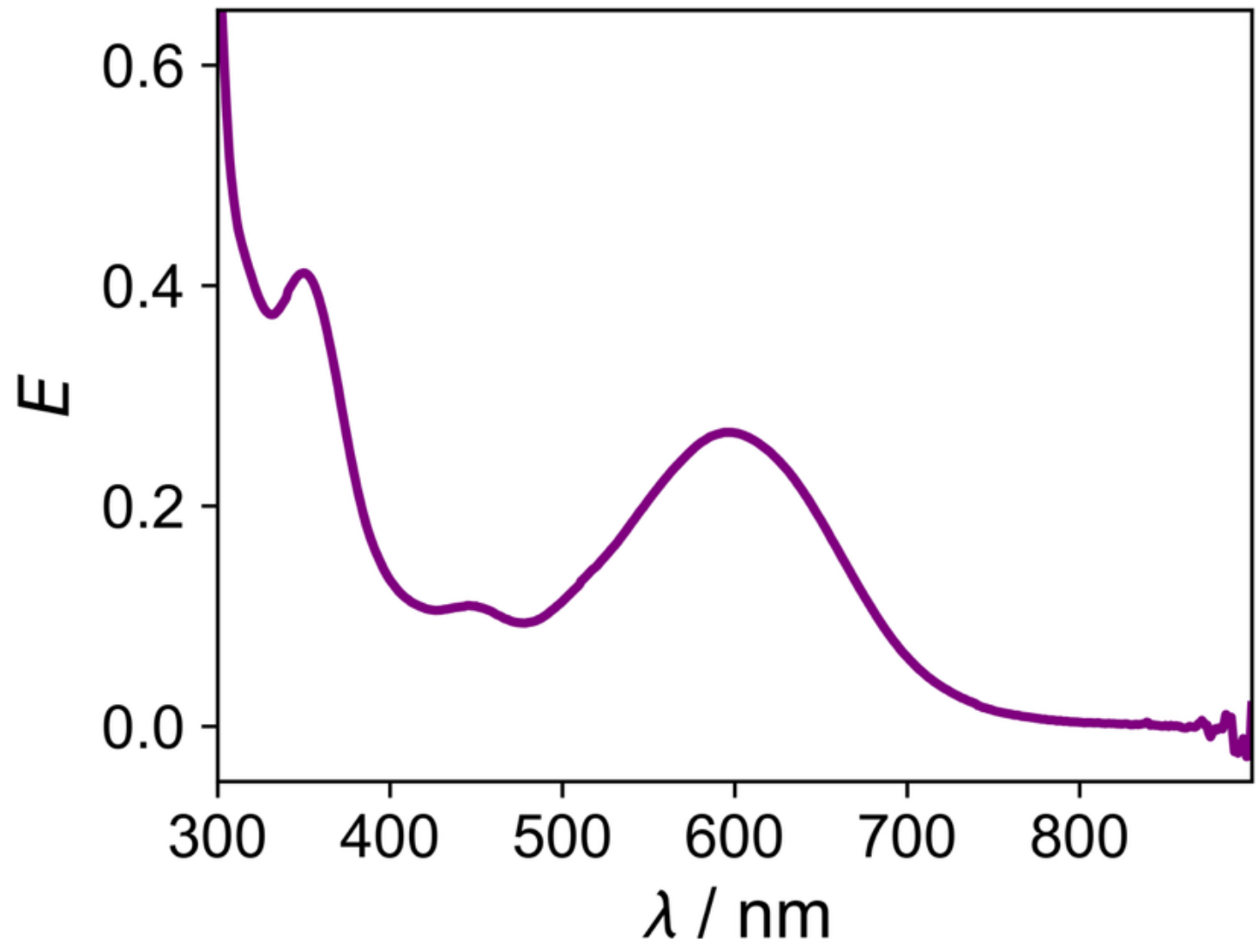

Figure S1: UV-vis difference spectrum from generation of [(D$_{12}$-TMC)Fe^{III}(O$_3$)(I)]B$_{Ar}$F (**2b″-B$_{Ar}$F**; 5·10^{-3} mol/L in 2-Me-THF/THF) at –115 °C.

C. Additional UV-vis spectra for Co complexes

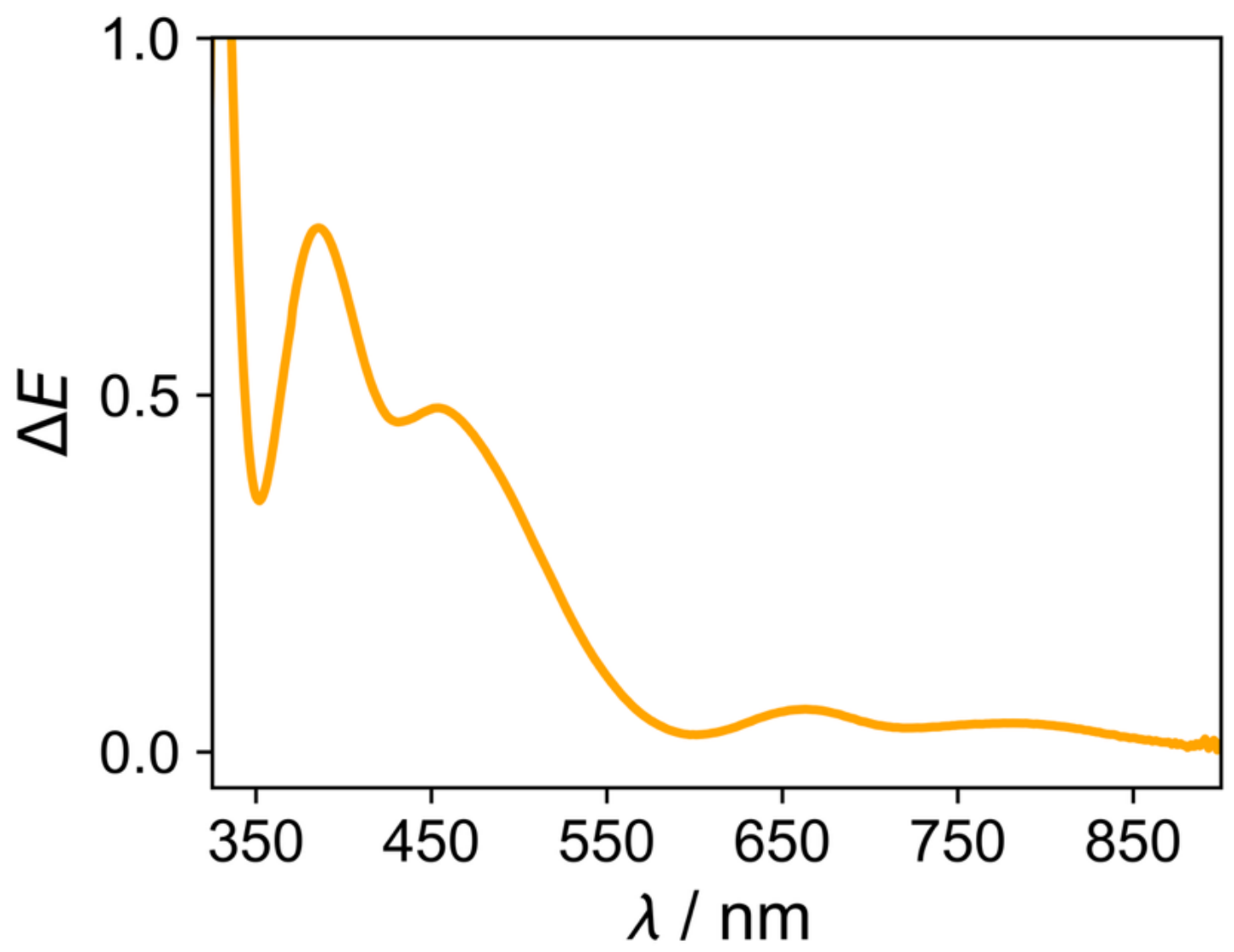

Figure S2: UV-vis difference spectrum from decay of [(D$_{12}$-TMC)Co^{IV}(O)(OTf)]B$_{Ar}$F (**5b′-B$_{Ar}$F**, 8·10^{-4} mol/L in 2-Me-THF/THF) over the temperature range from –65 to –50 °C (*cf.* Fig. 5 in main manuscript)

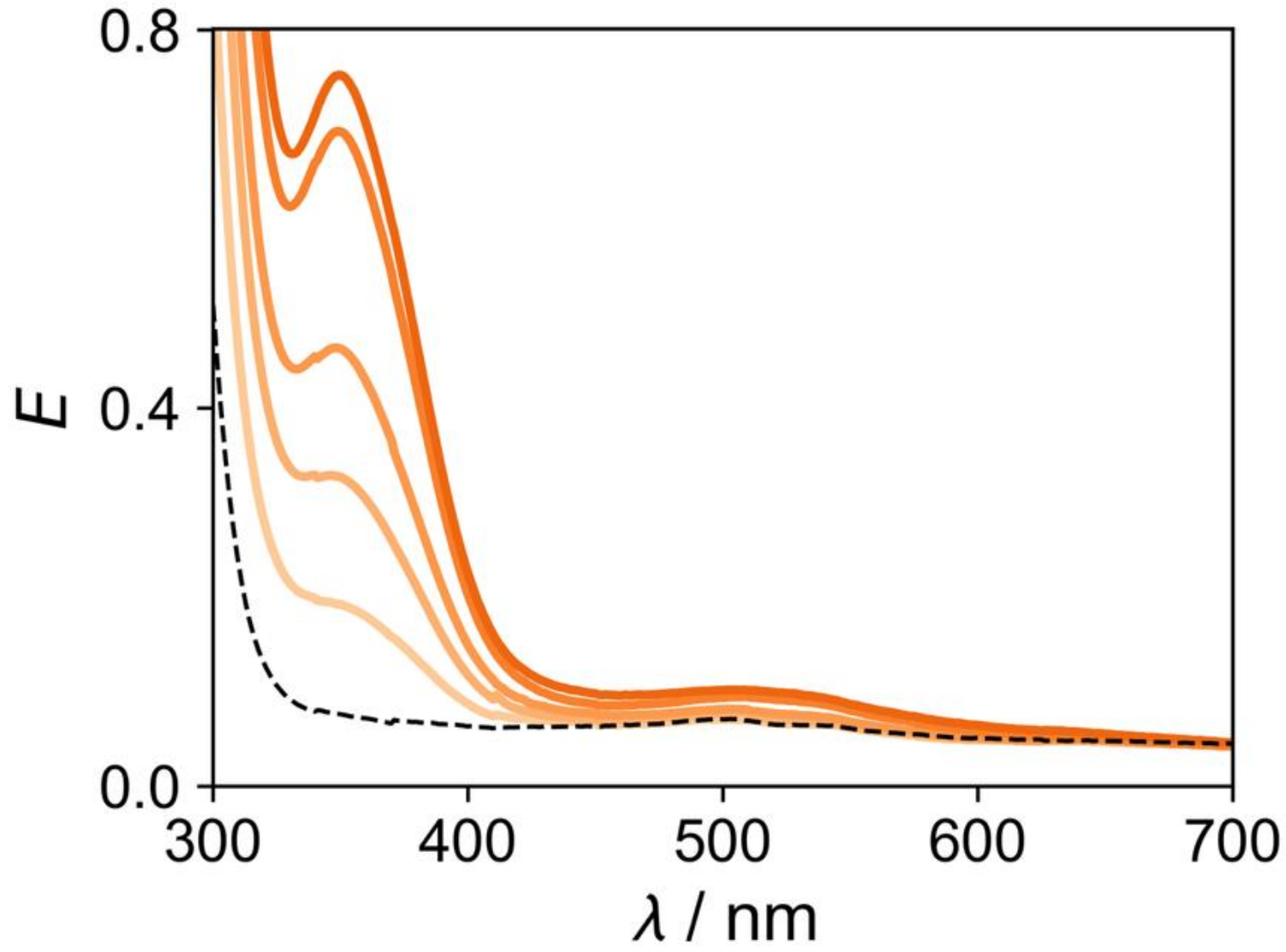

Figure S3: UV-vis spectra from generation of [(D$_{12}$-TMC)Co^{II}(O$_3$)(F)]B$_{Ar}$F (**7b″-B$_{Ar}$F**, 2·10^{-3} mol/L in 2-Me-THF/THF) over the temperature range of –123 to –92 °C.

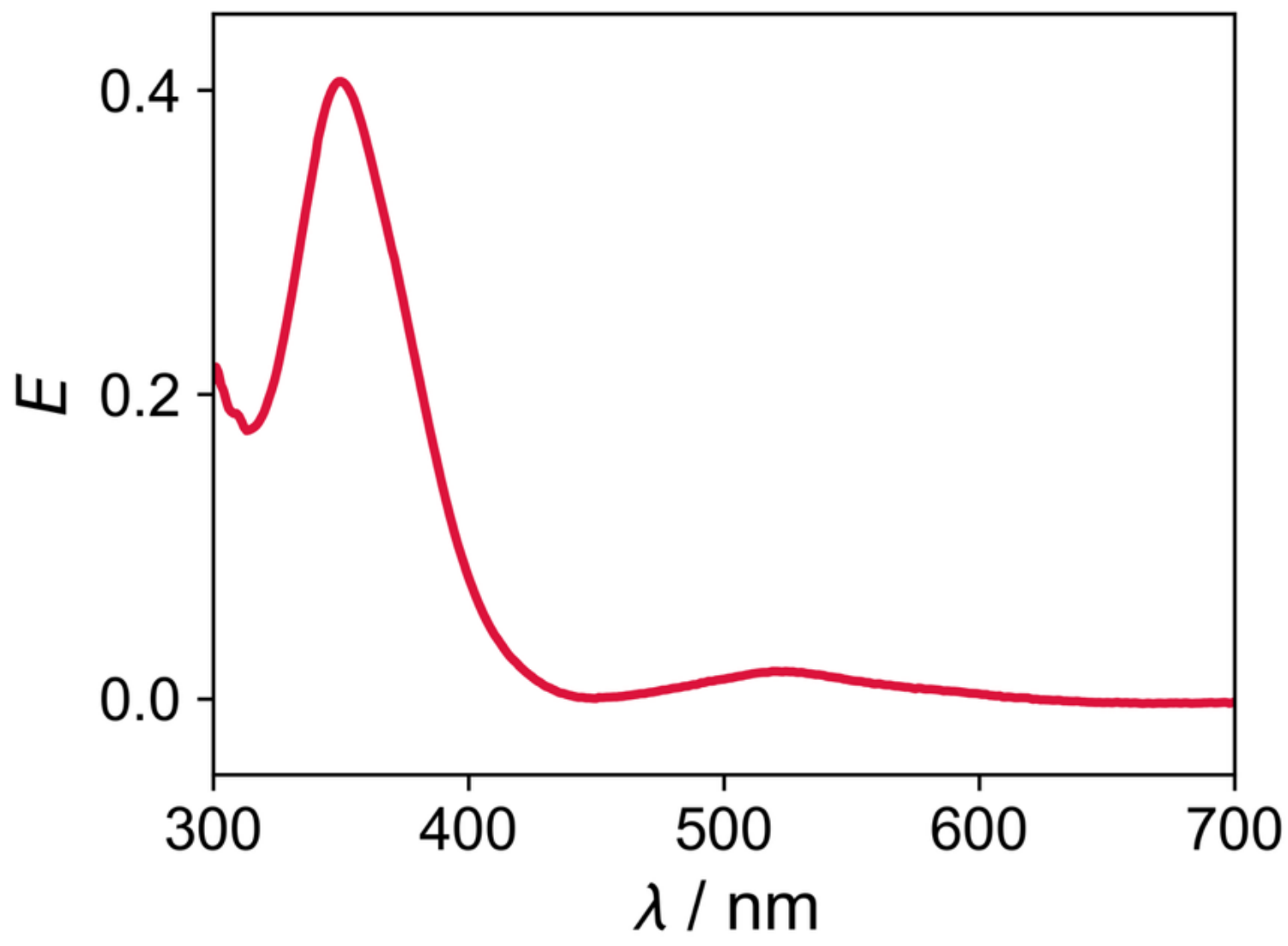

Figure S4: UV-vis difference spectrum from interconversion of [(D$_{12}$-TMC)Co^{II}(O$_3$)(F)]B$_{Ar}$F (**7b″-B$_{Ar}$F**, 2·10^{-3} mol/L in 2-Me-THF/THF) over the temperature range of –90 to –50 °C (*cf.* Fig. 6 in main manuscript).

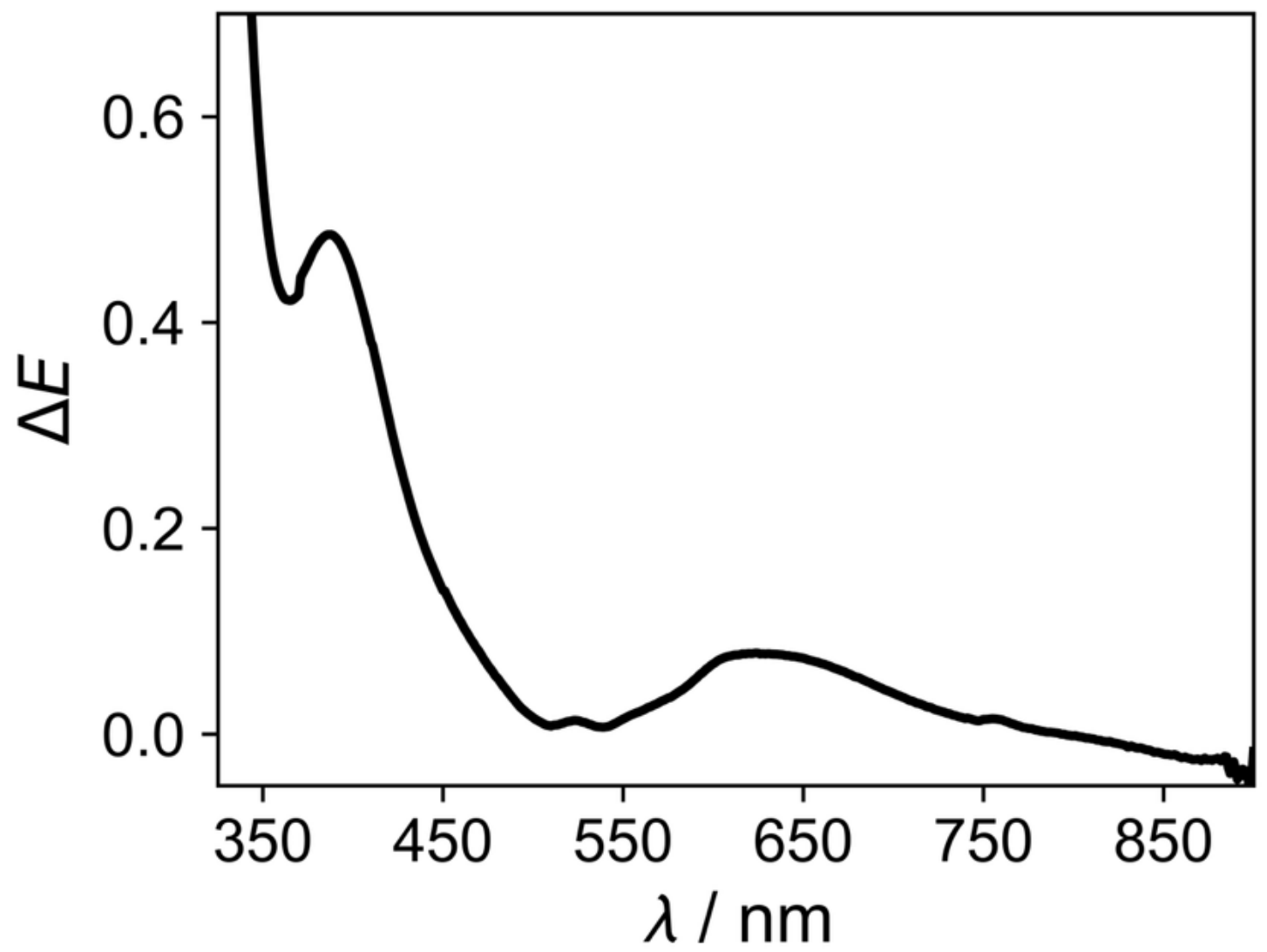

Figure S5: UV-vis difference spectrum from generation of [(D$_{12}$-TMC)Co^{IV}(O)(F)]B$_{Ar}$F (**7b′-B$_{Ar}$F** 8·10^{-4} M in 2-Me-THF/THF) over the temperature range from –35 °C to 0 °C (enlarged from Fig. 7 in main manuscript).

D. Kinetics of the Fe ozonido to oxido complex conversion

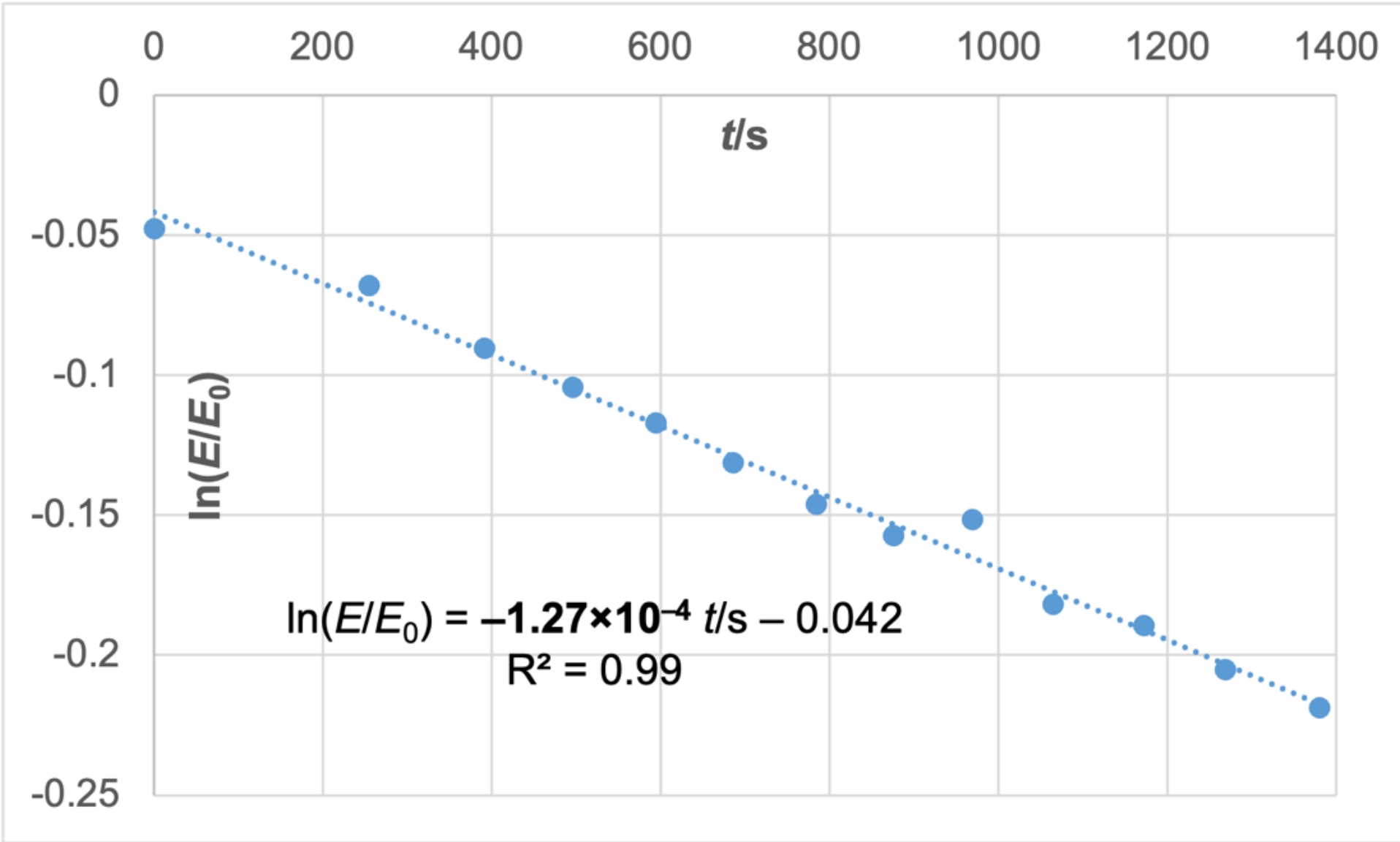

Figure S6: Kinetic trace ln(E/E_0) vs. t/s (538 nm) of the decay of [(D_{12}-TMC)Fe^{III}(O_3)(OTf)]$B_{Ar}F$ (**1b″-B_{Ar}F**, 3·10^{-3} M in 2-Me-THF/THF) at –131 °C.

t/s	E
0	0.597
254	0.585
391	0.572
496	0.564
594	0.557
686	0.549
784	0.541
875	0.535
969	0.538
1064	0.522
1172	0.518
1268	0.51
1380	0.503

Table S1: Time-dependent absorbance data E (538 nm) of the decay of [(D_{12}-TMC)Fe^{III}(O_3)(OTf)]$B_{Ar}F$ (**1b″-B_{Ar}F**; 3·10^{-3} M in 2-Me-THF/THF) at –131 °C. For kinetic evaluation, absorbance values were normalized with initial absorbance E_0 = 0.626.

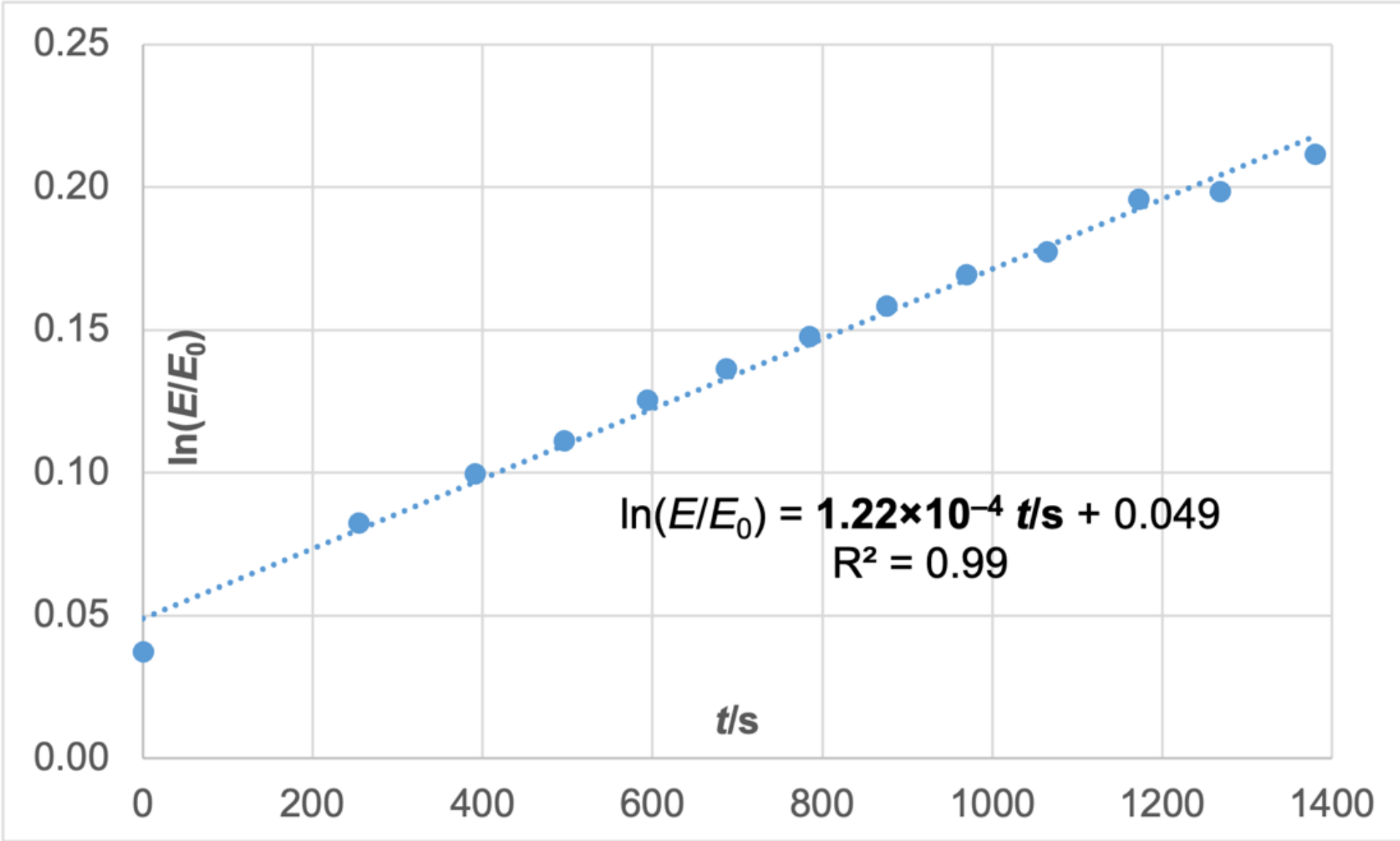

Figure S7: Kinetic trace ln(E/E_0) vs. *t*/s (813 nm) of the increase of [(D_{12}-TMC)Fe^{IV}(O)(OTf)]$B_{Ar}F$ (**1b′-B_{ArF}**; 3·10^{-3} M in 2-Me-THF/THF) at –131 °C.

t/s	*E*
0	0.326
254	0.341
391	0.347
496	0.351
594	0.356
686	0.36
784	0.364
875	0.368
969	0.372
1064	0.375
1172	0.382
1268	0.383
1380	0.388

Table S2: Time-dependent absorbance data *E* (813 nm) of the increase of [(D_{12}-TMC)Fe^{IV}(O)(OTf)]$B_{Ar}F$ (**1b′-$B_{Ar}F$**; 3·10^{-3} M in 2-Me-THF/THF) at –131 °C. For kinetic evaluation, absorbance values were normalized with initial absorbance E_0 = 0.314.
