# Supplementary material for: First Evidence for Ozonido‐TMC Complexes of Iron and Cobalt
Source: Angew Chem Int Ed Engl. 2026 Jul 9;65(37):e4716492. doi: 10.1002/anie.4716492 (PMC13548893; doi:10.1002/anie.4716492)
Supplement: Supplementary file 3 [file ANIE-65-e4716492-s003.pdf]

Supporting Information Part IV — Mass spectrometry

**A.**	**General Procedures**	**2**
**B.**	**Assignment of key species for the reaction of [(TMC)Fe^{II}(OTf)]B_{Ar}F and O_3**	**3**
**C.**	**Assignment of key species for the reaction of [(TMC)Co^{II}(OTf)]B_{Ar}F and O_3**	**4**
**D.**	**Detected complex ions for the reaction of [(D_{12}-TMC)Fe^{II}(OTf)]B_{Ar}F and O_3**	**5**
**E.**	**Detected complex ions for the reaction of [(D_{12}-TMC)Co^{II}(OTf)]B_{Ar}F and O_3**	**6**
**F.**	**Detected complex ions for the reaction of [(D_{12}-TMC)Co^{II}(F)]B_{Ar}F and O_3**	**8**
**G.**	**MS/MS studies for the reaction of [(D_{12}-TMC)Fe^{II}(OTf)]B_{Ar}F and O_3**	**10**
**H.**	**MS/MS studies for the reaction of [(D_{12}-TMC)Co^{II}(OTf)]B_{Ar}F and O_3**	**12**
**I.**	**MS/MS studies for the reaction of [(D_{12}-TMC)Co^{II}(F)]B_{Ar}F and O_3**	**14**

A. General Procedures

Cryospray-Ionization Mass Spectrometry (CSI-MS)

Cryospray-ionization mass spectrometry (CSI-MS) measurements were performed on a UHR-TOF Bruker Daltonik maXis plus, an ESI-quadrupole time-of-flight (qToF) mass spectrometer capable of a resolution of at least 60.000 (FWHM), which was coupled to a Bruker Daltonik Cryospray unit. Detection was performed in positive ion mode with a source voltage of 3.5 kV and a flow rate of 240 µL h^{-1}. The drying gas (nitrogen), required to achieve solvent removal, as well as the spray gas were held at −110 °C. The mass spectrometer was calibrated prior to every experiment via direct infusion of Agilent ESI-TOF low concentration tuning mixture, which provided an m/z range of peaks due to singly charged ions up to 2700 Da in both ion modes. Analysis and interpretation of the obtained mass spectra was performed with Bruker Compass DataAnalysis 4.2. The complex solutions were extracted from Teflon-capped vials with a pre-cooled syringe and manually transferred into the −110 °C cold cryospray unit within seconds.

Electrospray Ionisation Mass Spectrometry (ESI-MS; MS/MS)

Electrospray ionization mass spectrometry (ESI-MS) was performed on a Bruker Daltonics Impact II ultrahigh resolution quadrupole time-of-flight (UHR qTOF) mass spectrometer with a resolution of up to 50.000 (FWHM). Measurements were performed in positive ion mode with a source voltage of 4.5 kV. In order to prevent decomposition of the complexes, the source heater was switched off and the drying gas (nitrogen) flow rate was set to 8 L min^{-1} to achieve sufficient solvent removal. Collision induced dissociation (CID) was performed with nitrogen as collision gas using the Auto MS/MS tool implemented in the Bruker oTof control software. Analysis of the obtained mass spectra was performed with Bruker Compass DataAnalysis 5.1. The spectrometer was calibrated prior to measurements by direct infusion of sodium formate solution. The complex solutions were drawn from Teflon-capped vials with a pre-cooled syringe and transferred into the mass spectrometer by direct infusion (180 µL min^{-1} flow rate) using a syringe pump through thermally insulated peek tubing.

A typical sampling process is described below:

Initially, the Hamilton syringe and a home-built peek-cryo-tube were cooled by injecting a pre-cooled Me-THF/THF solution (–150 °C). This process was repeated three times. Immediately after, the pre-cooled sample solution (–140 °C or lower) was drawn up with the cold Hamilton syringe, which was then quickly placed into the syringe pump at the start of the measurement.

B. Assignment of key species for the reaction of [(TMC)Fe^{II}(OTf)]$B_{Ar}F$ and O_3

Table S1: Summary of key species detected in the reaction mixture of **1a-$B_{Ar}F$** with ozone.

m/z		detected as	species
exp.	calcd.		
328.1913	328.1920	[(TMC)Fe(O)]$^+$	Fe^{III}=O
357.1949	357.1947	[(TMC)Fe(HCOO)]$^+$	Fe^{II}
360.1816	360.1818	[(TMC)Fe(O_3)]$^+$	Fe^{II}–O_3
477.1431	477.1440	[(TMC)Fe(O)(OTf)]$^+$	Fe^{IV}=O

C. Assignment of key species for the reaction of [(TMC)Co^{II}(OTf)]$B_{Ar}F$ and O_3

Table S2: Summary of key species detected in the reaction mixture of **5a-B_{Ar}F** with ozone.

m/z		detected as	species
exp.	calcd.		
330.1823	330.1824	[(TMC-propyl-O)Co]$^+$	Co^{II}-propyl-O
347.1846	347.1852	[(TMC-propyl-O)Co(OH)]$^+$<br>[(TMC)Co(OO)]$^+$	Co^{III}-propyl-O /<br>Co^{II}–O_2
360.1920	360.1930	[(TMC)Co(HCOO)]$^+$	Co^{II}
363.1797	363.1801	[(TMC)Co(O_3)]$^+$	Co^{II}–O_3
376.1874	376.1879	[(TMC)Co(O)(HCOO)]$^+$	Co^{IV}=O
480.1418	480.1423	[(TMC)Co(O)(OTf)]$^+$	Co^{IV}=O

D. Detected complex ions for the reaction of $[(D_{12}\text{-TMC})Fe^{II}(OTf)]B_{Ar}F$ and O_3

Table S3: All ions detected in the reaction mixture of **1b-B$_{Ar}$F** with ozone.
Note that ChemDraw uses a mass of ^{56}Fe that is 0.0005 u too low (55.9344 vs. 55.9349).

m/z		ion
exp.	calcd.	
522.2916	522.2914	$C_{22}H_{30}D_{10}FeN_4O_6^+$
522.2916	514.3196	$C_{21}H_{30}D_{12}FeN_4O_6^+$
504.2059	504.2059	$C_{15}H_{19}D_{12}F_3FeN_4O_5S^+$
490.2262	490.2266	$C_{15}H_{21}D_{12}F_3FeN_4O_4S^+$
489.2189	489.2188	$C_{15}H_{20}D_{12}F_3FeN_4O_4S^+$
472.3089	472.3090	$C_{19}H_{28}D_{12}FeN_4O_5^+$
440.3191	440.3192	$C_{19}H_{28}D_{12}FeN_4O_3^+$
419.3051	419.3063	$C_{16}H_{27}D_{12}FeN_4O_4^+$
414.2673	414.2671	$C_{16}H_{22}D_{12}FeN_4O_4^+$
402.2548	402.2553	$C_{18}H_{30}D_6FeN_4O_2^+$
386.2709	386.2722	$C_{15}H_{22}D_{12}FeN_4O_3^+$
385.2635	385.2644	$C_{15}H_{21}D_{12}FeN_4O_3^+$
384.2567	384.2566	$C_{15}H_{20}D_{12}FeN_4O_3^+$
376.2434	376.2443	$C_{16}H_{34}D_3FeN_4O_2^+$
372.2566	372.2566	$C_{14}H_{20}D_{12}FeN_4O_3^+$
369.2696	369.2695	$C_{15}H_{21}D_{12}FeN_4O_2^+$
360.2730	360.2730	$C_{14}H_{21}D_{12}FFeN_4O^+$
359.2412	359.2416	$C_{16}H_{33}D_3FeN_4O^+$
356.2618	356.2617	$C_{14}H_{20}D_{12}FeN_4O_2^+$
354.2466	354.2460	$C_{14}H_{18}D_{12}FeN_4O_2^+$
341.1564	341.1558	$C_{12}H_{22}D_3F_3FeN_4^+$
340.2661	340.2667	$C_{14}H_{20}D_{12}FeN_4O^+$
339.2587	339.2589	$C_{14}H_{19}D_{12}FeN_4O^+$
338.2519	338.2511	$C_{14}H_{18}D_{12}FeN_4O^+$
324.2719	324.2718	$C_{14}H_{20}D_{12}FeN_4^+$
305.1718	305.1700	$C_{12}H_{18}D_6FFeN_4^+$
269.3452	269.3453	$C_{14}H_{21}D_{12}N_4^+$

E. Detected complex ions for the reaction of $[(D_{12}\text{-TMC})Co^{II}(OTf)]B_{Ar}F$ and O_3

Table S4: All ions detected in the reaction mixture of **5b-B$_{Ar}$F** with ozone.

m/z		ion
exp.	calcd.	
576.2753	576.2751	$C_{20}H_{28}D_{12}CoF_3N_4O_5S^+$
562.2594	562.2595	$C_{19}H_{26}D_{12}CoF_3N_4O_5S^+$
558.3573	558.3575	$C_{24}H_{35}D_{12}CoN_4O_6^+$
544.3418	544.3419	$C_{23}H_{33}D_{12}CoN_4O_6^+$
542.3624	542.3626	$C_{24}H_{35}D_{12}CoN_4O_5^+$
534.3211	534.3211	$C_{21}H_{31}D_{12}CoN_4O_7^+$
530.3266	530.3262	$C_{22}H_{31}D_{12}CoN_4O_6^+$
514.3313	514.3313	$C_{22}H_{31}D_{12}CoN_4O_5^+$
509.2198	509.2203	$C_{15}H_{21}D_{12}CoF_3N_4O_5S^+$
508.2131	508.2125	$C_{15}H_{20}D_{12}CoF_3N_4O_5S^+$
492.2183	492.2176	$C_{15}H_{20}D_{12}CoF_3N_4O_4S^+$
491.2101	491.2098	$C_{15}H_{19}D_{12}CoF_3N_4O_4S^+$
489.3106	489.3086	$C_{23}H_{34}D_8CoN_4O_3^+$
486.3360	486.3364	$C_{21}H_{31}D_{12}CoN_4O_4^+$
476.2223	476.2227	$C_{15}H_{20}D_{12}CoF_3N_4O_3S^+$
474.2991	474.3000	$C_{19}H_{27}D_{12}CoN_4O_5^+$
472.3212	472.3207	$C_{20}H_{29}D_{12}CoN_4O_4^+$
462.2915	462.2882	$C_{21}H_{35}D_6CoN_4O_3^+$
460.2834	460.2844	$C_{18}H_{25}D_{12}CoN_4O_5^+$
458.3049	458.3051	$C_{19}H_{27}D_{12}CoN_4O_4^+$
448.2763	448.2741	$C_{20}H_{35}D_5CoN_4O_3^+$
446.2679	446.2687	$C_{17}H_{23}D_{12}CoN_4O_5^+$
430.2730	430.2738	$C_{17}H_{23}D_{12}CoN_4O_4^+$
412.3051	412.3043	$C_{18}H_{31}D_9CoN_4O_2^+$
405.2529	405.2526	$C_{18}H_{28}D_7CoN_4O_2^+$
402.2780	402.2789	$C_{16}H_{23}D_{12}CoN_4O_3^+$
388.2625	388.2632	$C_{15}H_{21}D_{12}CoN_4O_3^+$
387.2552	387.2554	$C_{15}H_{20}D_{12}CoN_4O_3^+$
378.2338	378.2353	$C_{16}H_{33}D_3CoN_4O_2^+$

377.2281	377.2275	$C_{16}H_{32}D_3CoN_4O_2^+$
375.2546	375.2554	$C_{14}H_{20}D_{12}CoN_4O_3^+$
372.2677	372.2683	$C_{15}H_{21}D_{12}CoN_4O_2^+$
362.2390	362.2404	$C_{16}H_{33}D_3CoN_4O^+$
359.2597	359.2605	$C_{14}H_{20}D_{12}CoN_4O_2^+$
358.2519	358.2527	$C_{14}H_{19}D_{12}CoN_4O_2^+$
342.2569	342.2578	$C_{14}H_{19}D_{12}CoN_4O^+$

F. Detected complex ions for the reaction of $[(D_{12}\text{-TMC})Co^{II}(F)]B_{ArF}$ and O_3

Table S5: All ions detected in the reaction mixture of **7b-B_{ArF}** with ozone.

m/z		ion
exp.	calcd.	
514.2764	514.2769	$C_{24}H_{27}D_{10}CoN_4O_4^+$
463.3235	463.3231	$C_{22}H_{28}D_{12}CoN_4O_2^+$
416.2576	416.2581	$C_{16}H_{21}D_{12}CoN_4O_4^+$
412.2990	412.2996	$C_{18}H_{25}D_{12}CoN_4O_2^+$
409.2767	409.2761	$C_{18}H_{22}D_{12}CoN_4O_2^+$
405.2529	405.2526	$C_{18}H_{28}D_7CoN_4O_2^+$
402.2783	402.2789	$C_{16}H_{23}D_{12}CoN_4O_3^+$
400.2627	400.2632	$C_{16}H_{21}D_{12}CoN_4O_3^+$
388.2628	388.2632	$C_{15}H_{21}D_{12}CoN_4O_3^+$
386.2834	386.2840	$C_{16}H_{23}D_{12}CoN_4O_2^+$
379.2659	379.2667	$C_{14}H_{21}D_{12}CoFN_4O_2^+$
378.2557	378.2589	$C_{14}H_{20}D_{12}CoFN_4O_2^+$
375.2550	375.2554	$C_{14}H_{20}D_{12}CoN_4O_3^+$
372.2677	372.2683	$C_{15}H_{21}D_{12}CoN_4O_2^+$
370.2521	370.2527	$C_{15}H_{19}D_{12}CoN_4O_2^+$
365.2676	365.2675	$C_{14}H_{20}D_{12}CoF_2N_4^+$
363.2732	363.2718	$C_{14}H_{21}D_{12}CoFN_4O^+$
362.2640	362.2640	$C_{14}H_{20}D_{12}CoFN_4O^+$
361.2580	361.2562	$C_{14}H_{19}D_{12}CoFN_4O^+$
359.2600	359.2605	$C_{14}H_{20}D_{12}CoN_4O_2^+$
358.2523	358.2527	$C_{14}H_{19}D_{12}CoN_4O_2^+$
357.2031	357.2013	$C_{16}H_{28}D_3CoN_4O^+$
346.2686	346.2691	$C_{14}H_{20}D_{12}CoFN_4^+$
345.2619	345.2612	$C_{14}H_{19}D_{12}CoFN_4^+$
342.2573	342.2578	$C_{14}H_{19}D_{12}CoN_4O^+$
341.2507	341.2499	$C_{14}H_{18}D_{12}CoN_4O^+$
339.2355	339.2343	$C_{14}H_{16}D_{12}CoN_4O^+$

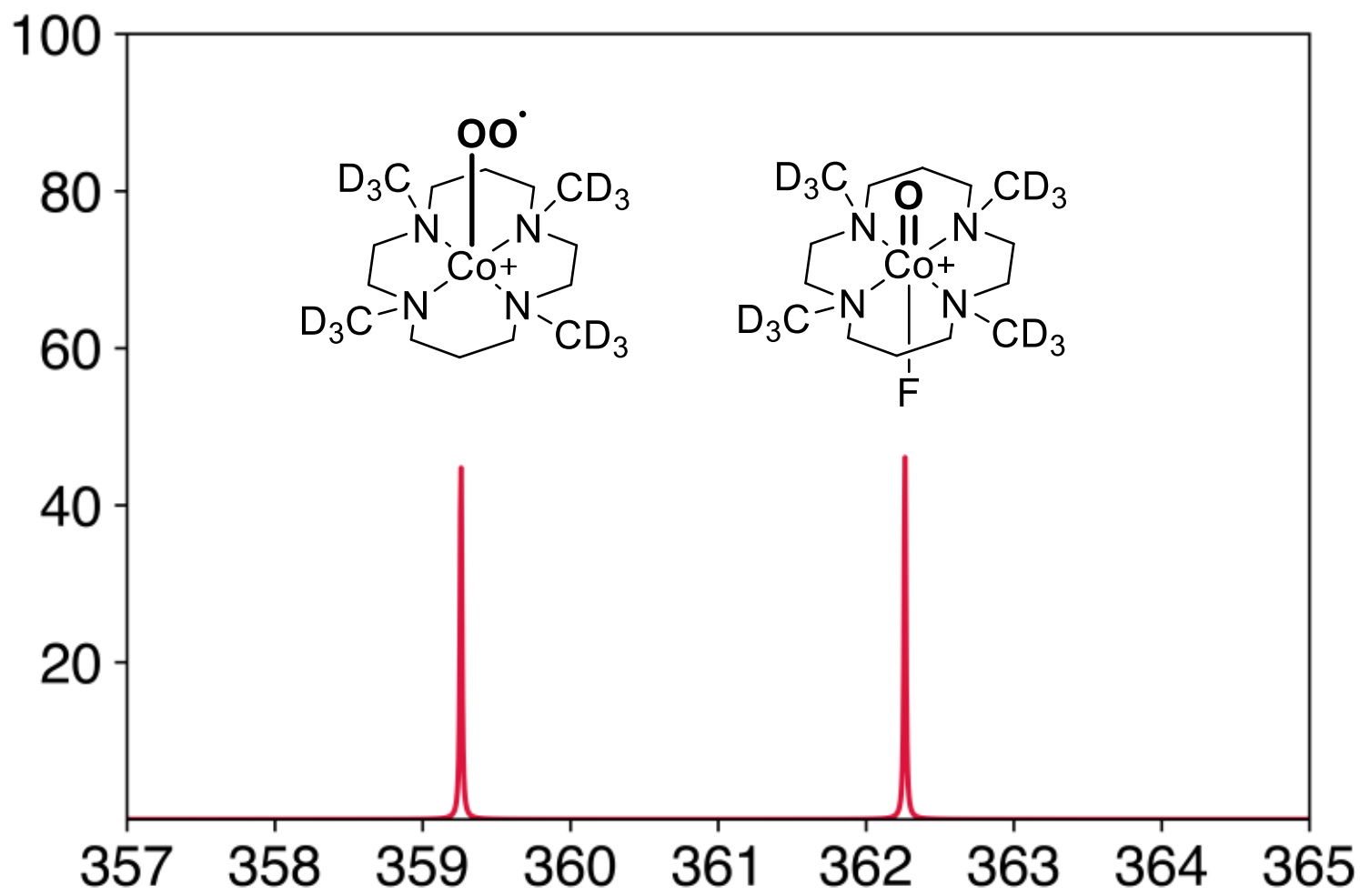

Figure 1: Ratio of signals m/z = 362.2390, 359.2597

G. MS/MS studies for the reaction of [(D$_{12}$-TMC)Fe^{II}(OTf)]B$_{ArF}$ and O$_3$

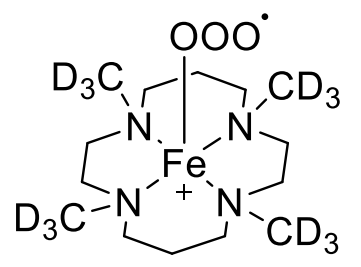

Chemical Formula:
$C_{14}H_{20}D_{12}FeN_4O_3^{\bullet+}$
Exact Mass: 372.26

376.2439

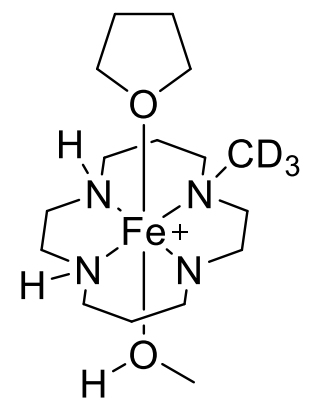

Chemical Formula:
$C_{16}H_{34}D_3FeN_4O_2^{+}$
Exact Mass: 376.2443

374.2483

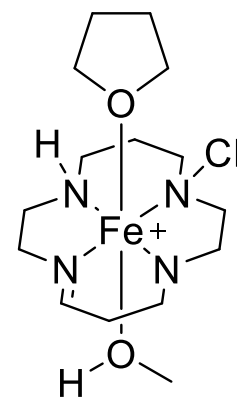

Chemical Formula:
$C_{16}H_{32}D_3FeN_4O_2^{+}$
Exact Mass: 374.2286

371.2306

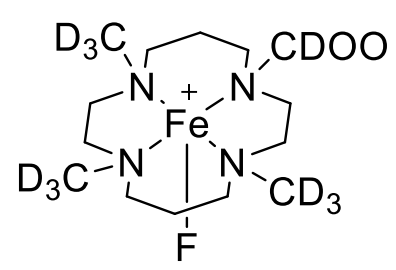

Chemical Formula:
$C_{13}H_{20}D_9FFeN_4^{+}$
Exact Mass: 325.23

369.2696

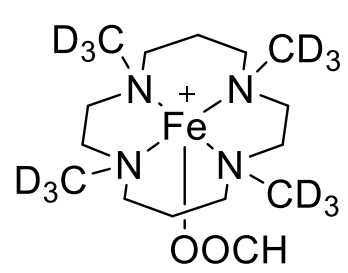

Chemical Formula:
$C_{15}H_{21}D_{12}FeN_4O_2^{+}$
Exact Mass: 369.2695

358.2333

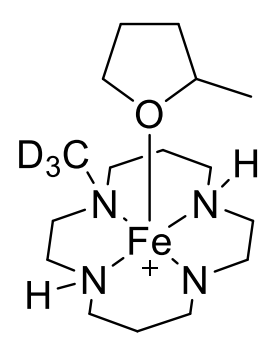

Chemical Formula:
$C_{16}H_{32}D_3FeN_4O^{+}$
Exact Mass: 358.2337

357.2268

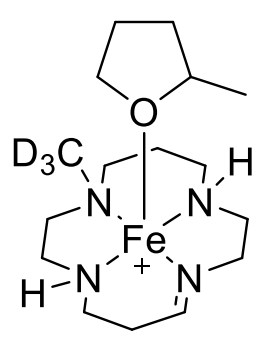

Chemical Formula:
$C_{16}H_{31}D_3FeN_4O^{+}$
Exact Mass: 357.23

354.2466

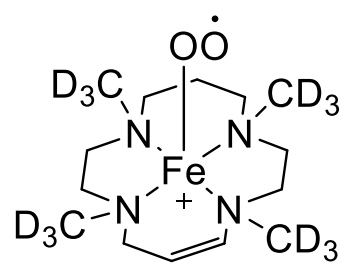

Chemical Formula:
$C_{14}H_{18}D_{12}FeN_4O_2^{\bullet+}$
Exact Mass: 354.2460

340.1911

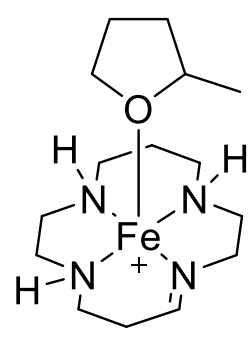

Chemical Formula:
$C_{15}H_{32}FeN_4O^{+}$
Exact Mass: 340.1914

323.2644

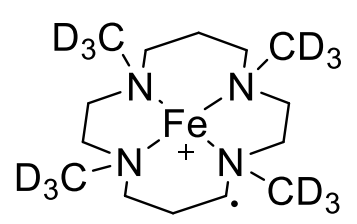

Chemical Formula:
$C_{14}H_{19}D_{12}FeN_4^{\bullet+}$
Exact Mass: 323.26

305.2222

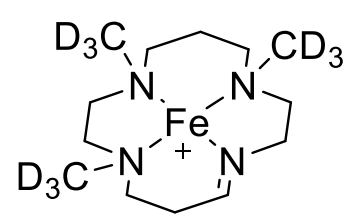

Chemical Formula:
$C_{13}H_{19}D_9FeN_4^{+}$
Exact Mass: 305.2217

298.2204

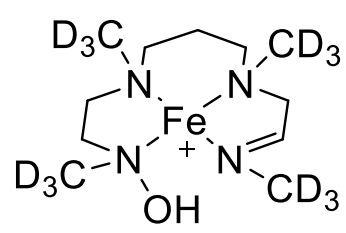

Chemical Formula:
$C_{11}H_{14}D_{12}FeN_4O^{+}$
Exact Mass: 298.2198

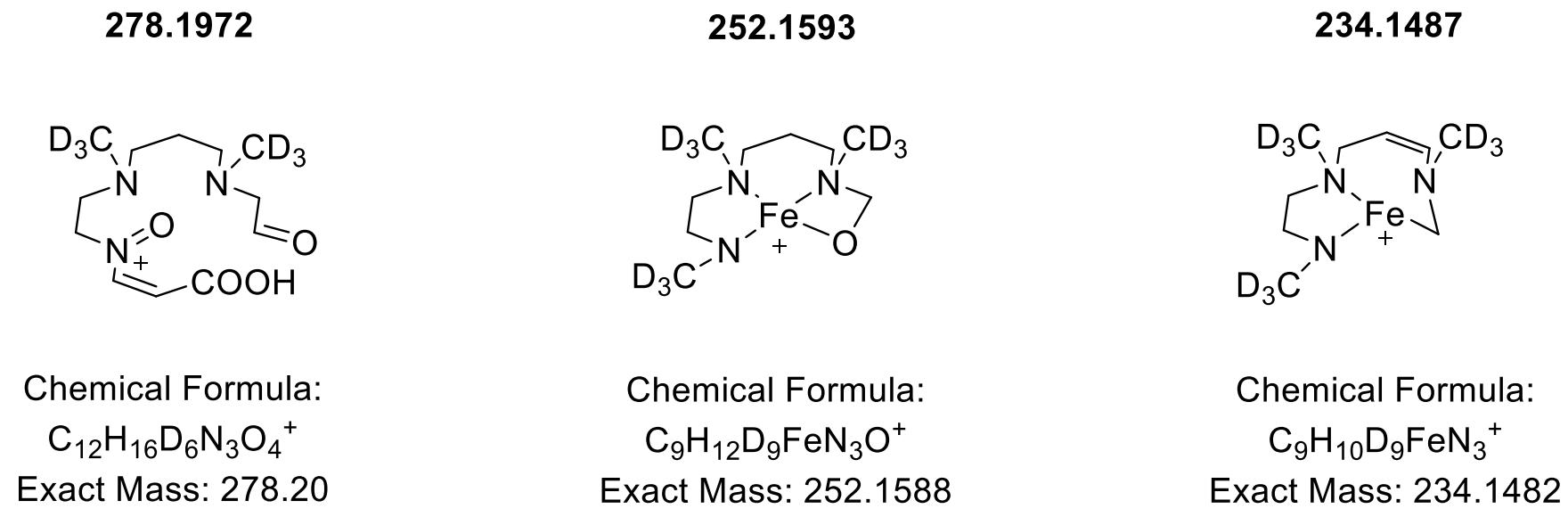

Scheme S1: MS/MS investigation of m/z = 372.2569.
Source of F in 371.2306 u is likely a preceding MS experiment with **5b-B$_{\mathbf{Ar}}$F**.
Note that ChemDraw uses a mass of ^{56}Fe that is 0.0005 u too low (55.9344 vs. 55.9349).

H. MS/MS studies for the reaction of [(D_{12}-TMC)Co^{II}(OTf)]$B_{Ar}F$ and O_3

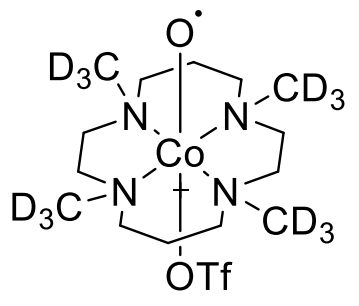

Chemical Formula:
$C_{15}H_{20}D_{12}CoF_3N_4O_4S^{\bullet+}$
Exact Mass: 492.2176

474.2073

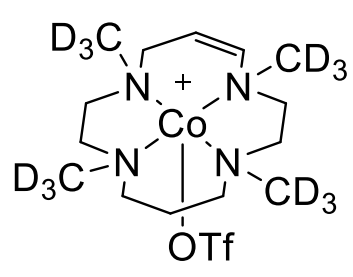

Chemical Formula:
$C_{15}H_{18}D_{12}CoF_3N_4O_3S^{+}$
Exact Mass: 474.2070

436.1917

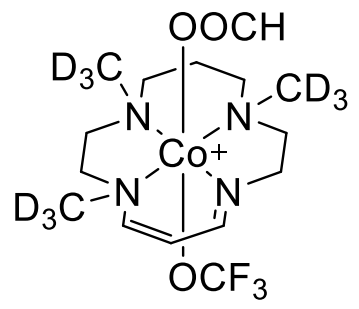

Chemical Formula:
$C_{15}H_{18}D_9CoF_3N_4O_3^{+}$
Exact Mass: 436.1927

387.2428

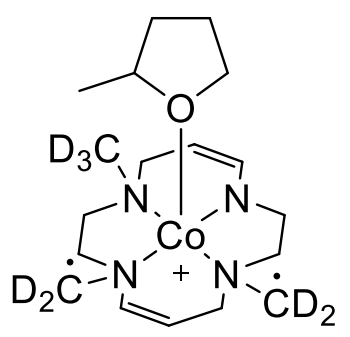

Chemical Formula:
$C_{18}H_{26}D_7CoN_4O^{2\bullet+}$
Exact Mass: 387.2420

342.2569

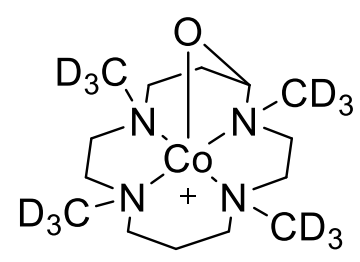

Chemical Formula:
$C_{14}H_{19}D_{12}CoN_4O^{+}$
Exact Mass: 342.2578

286.2312

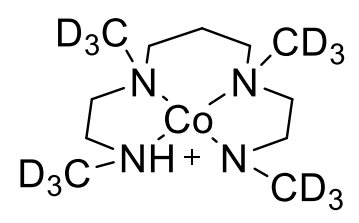

Chemical Formula:
$C_{11}H_{15}D_{12}CoN_4^{+}$
Exact Mass: 286.2315

Scheme S2: MS/MS investigation of m/z = 492.2183.

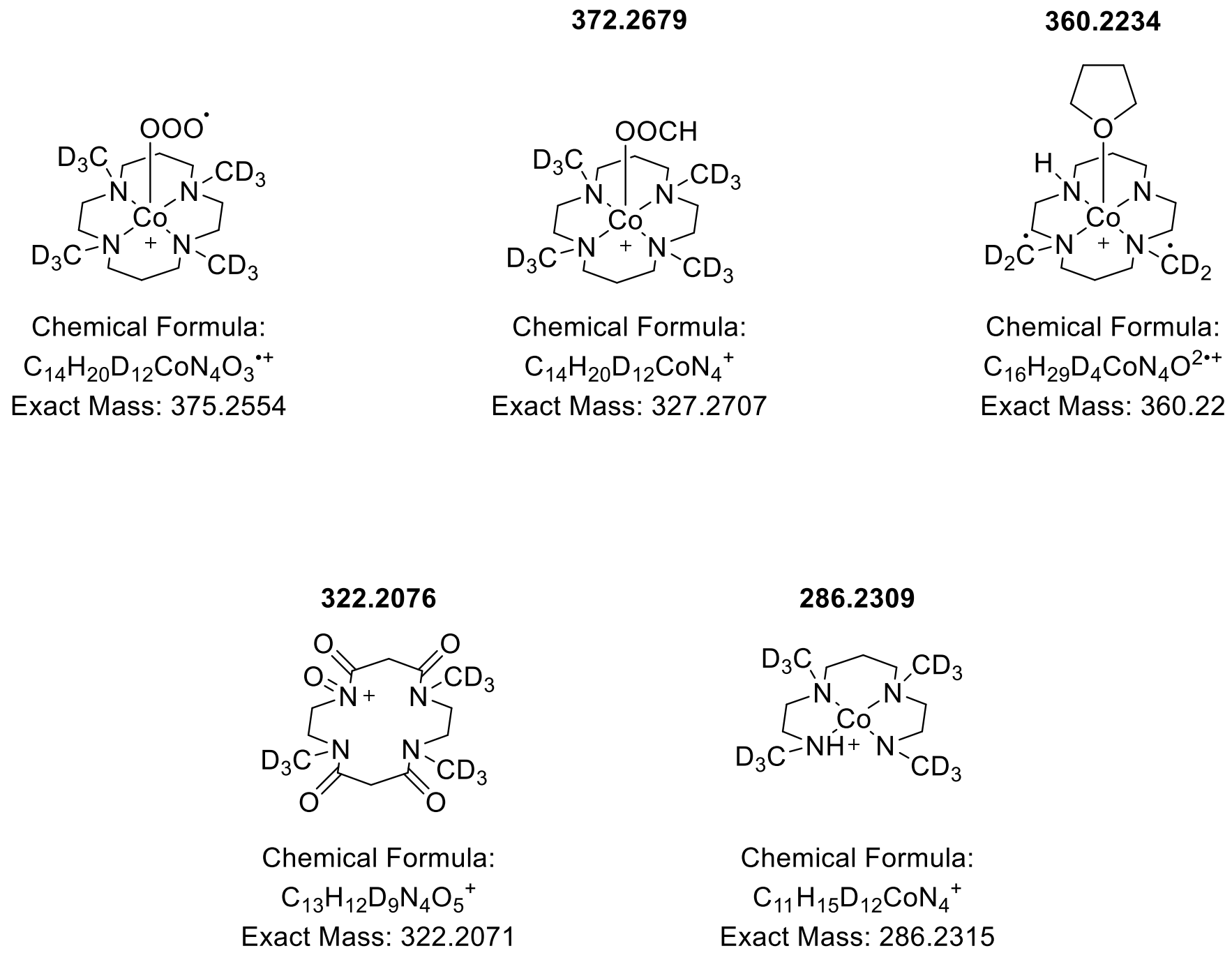

Scheme S3: MS/MS investigation of m/z = 375.2549.

I. MS/MS studies for the reaction of [(D_{12}-TMC)Co^{II}(F)]B_{ArF} and O_3

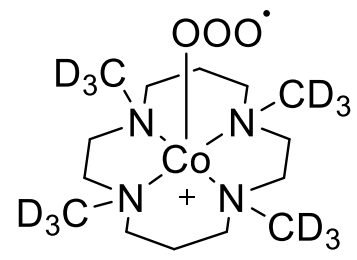

Chemical Formula: $C_{14}H_{20}D_{12}CoN_4O_3^{\bullet+}$
Exact Mass: 375.2554

372.2679

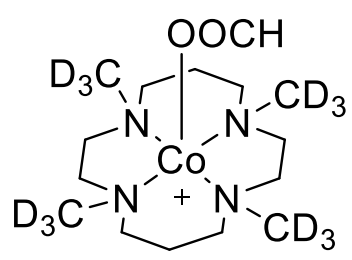

Chemical Formula: $C_{15}H_{21}D_{12}CoN_4O_2^{+}$
Exact Mass: 372.2683

357.2442

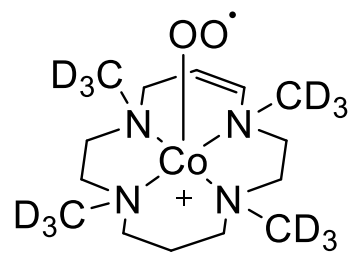

Chemical Formula: $C_{14}H_{18}D_{12}CoN_4O_2^{\bullet+}$
Exact Mass: 357.2448

341.2492

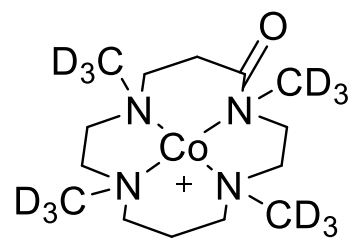

Chemical Formula: $C_{14}H_{18}D_{12}CoN_4O^{+}$
Exact Mass: 341.2499

325.2559

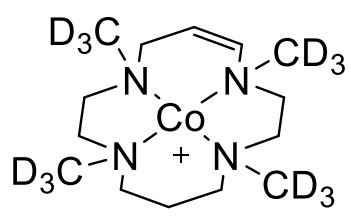

Chemical Formula: $C_{14}H_{18}D_{12}CoN_4^{+}$
Exact Mass: 325.2550

307.2126

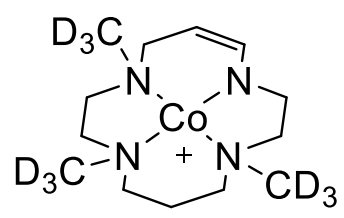

Chemical Formula: $C_{13}H_{18}D_9CoN_4^{+}$
Exact Mass: 307.2127

286.2315

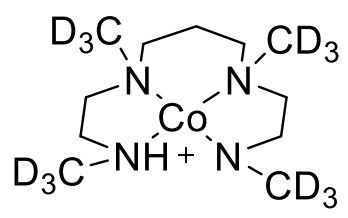

Chemical Formula: $C_{11}H_{15}D_{12}CoN_4^{+}$
Exact Mass: 286.2315

Scheme S4: MS/MS investigation of m/z = 375.2549.

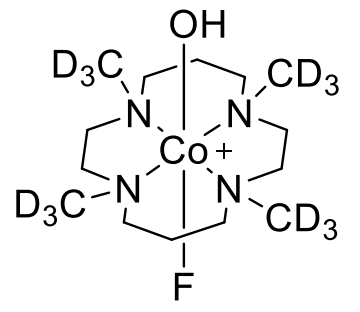

Chemical Formula:
$C_{14}H_{21}D_{12}CoFN_4O^+$
Exact Mass: 363.2718

345.2599

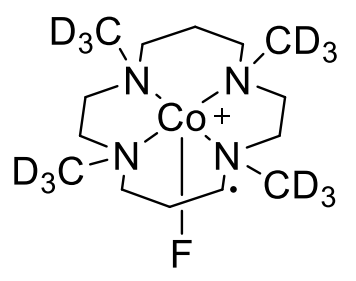

Chemical Formula:
$C_{14}H_{19}D_{12}CoFN_4^{\bullet+}$
Exact Mass: 345.2612

325.2540

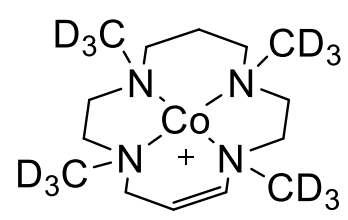

Chemical Formula:
$C_{14}H_{18}D_{12}CoN_4^+$
Exact Mass: 325.2550

307.2114

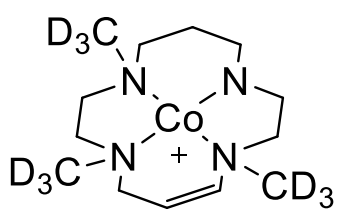

Chemical Formula:
$C_{13}H_{18}D_9CoN_4^+$
Exact Mass: 307.2127

286.2315

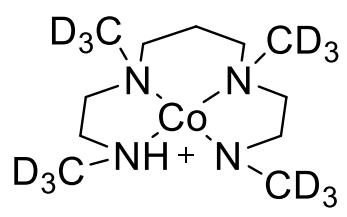

Chemical Formula:
$C_{11}H_{15}D_{12}CoN_4^+$
Exact Mass: 286.2315

Scheme S5: MS/MS investigation of m/z = 363.2725.

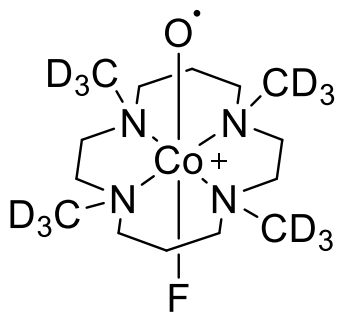

Chemical Formula:
$C_{14}H_{20}D_{12}CoFN_4O^{\bullet+}$
Exact Mass: 362.2640

363.2708

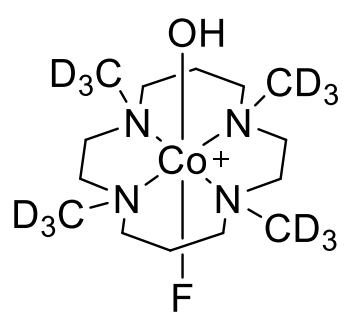

Chemical Formula:
$C_{14}H_{21}D_{12}CoFN_4O^+$
Exact Mass: 363.2718

345.2599

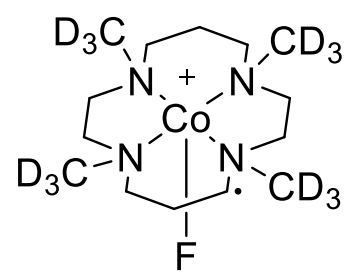

Chemical Formula:
$C_{14}H_{19}D_{12}CoFN_4^{\bullet+}$
Exact Mass: 345.2612

325.2540

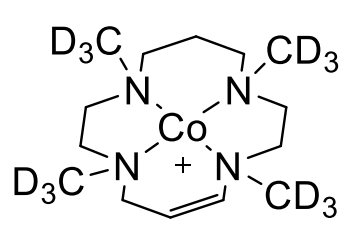

Chemical Formula:
$C_{14}H_{18}D_{12}CoN_4^+$
Exact Mass: 325.2550

307.2114

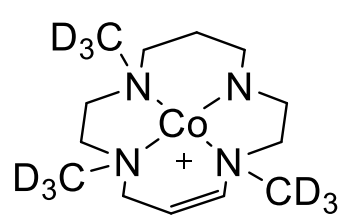

Chemical Formula:
$C_{13}H_{18}D_9CoN_4^+$
Exact Mass: 307.2127

286.2315

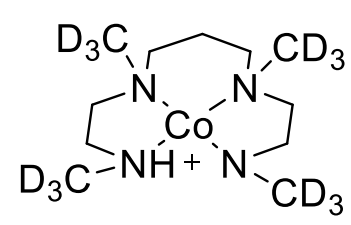

Chemical Formula:
$C_{11}H_{15}D_{12}CoN_4^+$
Exact Mass: 286.2315

Scheme S6: MS/MS investigation of m/z = 362.2631.

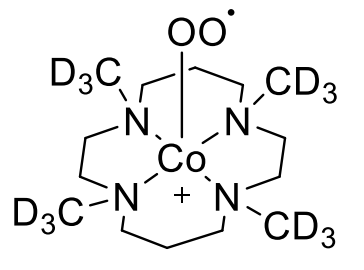

Chemical Formula:
$C_{14}H_{20}D_{12}CoN_4O_2^{\bullet+}$
Exact Mass: 359.2605

363.2708

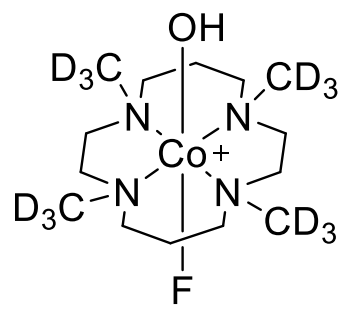

Chemical Formula:
$C_{14}H_{21}D_{12}CoFN_4O^+$
Exact Mass: 363.2718

345.2599

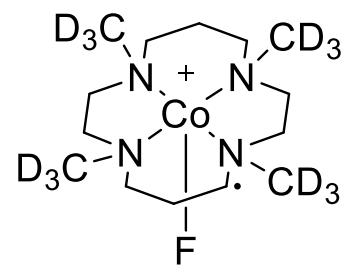

Chemical Formula:
$C_{14}H_{19}D_{12}CoFN_4^{\bullet+}$
Exact Mass: 345.2612

325.2540

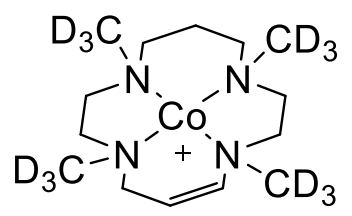

Chemical Formula:
$C_{14}H_{18}D_{12}CoN_4^+$
Exact Mass: 325.2550

307.2114

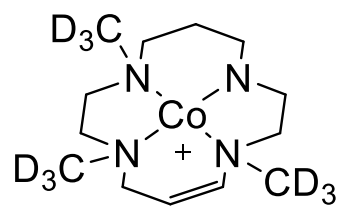

Chemical Formula:
$C_{13}H_{18}D_9CoN_4^+$
Exact Mass: 307.2127

286.2315

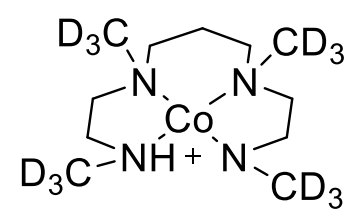

Chemical Formula:
$C_{11}H_{15}D_{12}CoN_4^+$
Exact Mass: 286.2315

Scheme S7: MS/MS investigation of m/z = 359.2601.

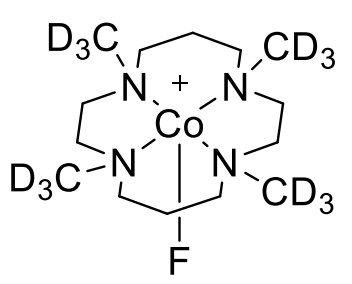

Chemical Formula:
$C_{14}H_{20}D_{12}CoFN_4^+$
Exact Mass: 346.2691

325.2540

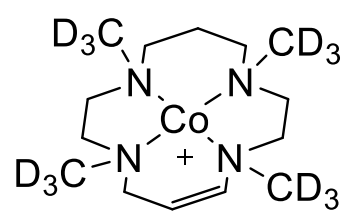

Chemical Formula:
$C_{14}H_{18}D_{12}CoN_4^+$
Exact Mass: 325.2550

286.2309

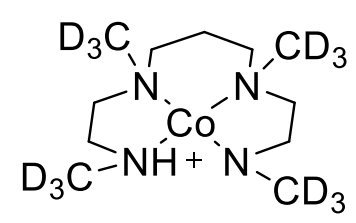

Chemical Formula:
$C_{11}H_{15}D_{12}CoN_4^+$
Exact Mass: 286.2315

Scheme S8: MS/MS investigation of m/z = 346.2683.
