# Supplementary material for: First Evidence for Ozonido‐TMC Complexes of Iron and Cobalt
Source: Angew Chem Int Ed Engl. 2026 Jul 9;65(37):e4716492. doi: 10.1002/anie.4716492 (PMC13548893; doi:10.1002/anie.4716492)
Supplement: Supplementary file 4 [file ANIE-65-e4716492-s004.pdf]

Supporting Information Part I — Experimental

A. Materials and Equipment 2

B. General Procedures 3

C. Syntheses 4

C.1. Synthesis of stoichiometric $AgB_{Ar}F$·MeCN·THF 4

C.2. Synthesis of $Co(OTf)_2$ 4

C.3. Synthesis of Iron Complexes 5

C.3.1. Synthesis of $[(TMC)Fe^{II}(OTf)]B_{Ar}F$ (1a-$B_{Ar}F$) 5

C.3.2. Synthesis of $[(D_{12}$-TMC)$Fe^{II}(I)]I$ 5

C.3.3. Synthesis of $[(D_{12}$-TMC)$Fe^{II}(I)]B_{Ar}F$ (2b-$B_{Ar}F$) 5

C.3.4. Synthesis of $[(D_{12}$-TMC)$Fe^{II}(OTf)]B_{Ar}F$ (1b-$B_{Ar}F$) 5

C.3.5. Synthesis of $[(D_{12}$-TMC)$Fe^{II}(F)]B_{Ar}F$ (3b-$B_{Ar}F$) 6

C.4. Synthesis of Cobalt Complexes 6

C.4.1. Synthesis of $[(TMC)Co^{II}(CH_3CN)](OTf)_2$ (4a-$(OTf)_2$) 6

C.4.2. Synthesis of $[(TMC)Co^{II}(OTf)_2]$ (5a) 6

C.4.3. Synthesis of $[(TMC)Co^{II}(I)]I$ and $[(D_{12}$-TMC)$Co^{II}(I)]I$ 6

C.4.4. Synthesis of $[(TMC)Co^{II}(I)]B_{Ar}F$ 7

C.4.5. Synthesis of $[(TMC)Co^{II}(OTf)]B_{Ar}F$ (5a-$B_{Ar}F$) and $[(D_{12}$-TMC)Co^{II} $(OTf)]B_{Ar}F$ (5b-$B_{Ar}F$) 7

C.4.6. Synthesis of $[(TMC)Co^{II}(ClO_4)]B_{Ar}F$ (6a-$B_{Ar}F$) 7

C.4.7. Synthesis of $[(TMC)Co^{II}(OTf)][Al\{OC(CF_3)_3\}_4]$ 8

C.4.8. Synthesis of $[(D_{12}$-TMC)$Co^{II}(F)]B_{Ar}F$ (7b-$B_{Ar}F$) 8

C.4.9. Synthesis of $[(TMC)Co^{II}(Br)]Br \cdot H_2O$ 8

D. NMR spectra of $AgB_{Ar}F$·MeCN·THF 9

E. Low temperature measurement setup 15

F. Glass septum cannula for ozonization on semi-preparative scale 16

A. Materials and Equipment

All glassware was dried in a special Schlenk tube under reduced pressure in a tube oven. Light sensitive substances were stored and used in brown glass vials. Commercially available compounds were of the highest available purity. Argon 5.0 was used for all Schlenk procedures. FeI_2 (ABCR), CoI_2 (Alfa Aesar) were received as 99.99% ultra-dry samples in glass ampules. AgOTf (99%) was received from Fluorochem. TMC (98%) was received from Sigma Aldrich (Merck) and was further dried by dissolving in diethyl ether and addition of activated molecular sieves under argon. D_{12}-TMC was synthesized according to literature procedures, purified by sublimation, and stored in a glove box to exclude moisture.[1] $NaB_{Ar}F$ was synthesized according to literature procedures.[2] $Li[Al\{OC(CF_3)_3\}_4]$ was provided by the group of Ingo Krossing (University of Freiburg, Germany). $NaClO_4$ (98%) was received from Sigma Aldrich (Merck) and used without further purification. $N(CH_3)_4O_3$ was prepared as described previously and stored at –80 °C in glass ampules.[3] A special glass tube cutter (VWR) was used to open the tetramethylammonium ozonide ampules. Ozone was generated from 5.0 oxygen with ozonizers of type OZONOSAN PM83K (Dr. J. Hänsler GmbH, Iffezheim, Germany) and S 1000 (E. Sander, Elektroapparatebau GmbH, Uetze-Eltze, Germany).

The crystallographic data have been deposited with the Cambridge Crystallographic Data Centre and the Leibniz-Institut für Informationsinfrastruktur as CCDC No. 2062705-2062715, 2062718-2062724, 2062764-2062766, 2062767, 2122182, 2122183, 2502781, and 2502782 can be obtained free of charge. See SI part II for depictions of molecular structures derived from X-ray diffraction.

Caution! The perchlorate salts used in this study are potentially explosive and should be handled with great care. The generation and handling of ozone gas and its application to organics is inherently dangerous. Safety precautions towards ozone regarding toxicity, explosion, and thus projectiles of broken glass must be applied.

B. General Procedures

Reactions with Ozone for Crystallization Attempts:

The complex solutions were prepared in dry glassware in a glove box under argon. For each reaction, 150 to 200 mg of complex were dissolved in either 2 mL of DCM for reactions at –80 °C or 2-Me-THF/THF (4:1) for reactions at –150 °C. For reactions with ozone, the glass vials containing the complex solutions were equipped with a special home-built glass septum (see SI part I), which allowed bubbling ozone through the solution without contact to the metal cannula: This procedure prevents decomposition of products by catalytically active transition metals. Ozone (up to approx. 15 vol-% in oxygen) was transferred into the vial by PE-tubing with a Hamilton valve and an attached cannula. Ozone was bubbled through the solution for 5 min. The product mixtures were subsequently stored in –80 °C or –145 °C freezers.

Reactions with $N(CH_3)_4O_3$ for Crystallization Attempts:

Complex solutions were prepared as described above. The addition of $N(CH_3)_4O_3$ was carried out in an argon-filled glove bag. The atmosphere in the bag was exchanged repeatedly by pumping for 2 h and subsequent flooding with argon (six cycles in total). A single vial of ozonide amounted to about 100 mg, which was divided equally into two portions as judged by visual inspection. Two capped vials with complex solutions in DCM or 2-Me-THF/THF were cooled in an isopentane bath to –80 °C or –145 °C, respectively, using liquid nitrogen within the glove bag. In the following, the vial caps were removed, the ozonide added, and the solutions mixed by gentle shaking in the coolant for at least 10 min. After recapping, the vials were quickly removed from the glove bag and stored in either an –80 °C or –145 °C freezer.

Reactions with Ozone for CSI- and ESI-MS Measurements:

The complex solutions were prepared in dry glassware in a glove box under argon. Complex solutions in 2-Me-THF/THF (4:1) were cooled to –120 °C for $[(D_{12}\text{-TMC})Co^{II}(OTf)]B_{Ar}F$ and $[(D_{12}\text{-TMC})Co^{II}(F)]B_{Ar}F$ (10-20 mM) as well as –135 °C for $[(D_{12}\text{-TMC})Fe^{II}(OTf)]B_{Ar}F$ (1-2 mM). For the reaction with ozone, the glass vials containing the complex solutions were equipped with a special home-built glass septum (vide supra). Ozone (up to approx. 15 vol-% in oxygen) was transferred into the vial by PE-tubing with a Hamilton valve and an attached cannula. Ozone was bubbled through the solution for two to three seconds only. Measurements of $[(TMC)Co^{II}(OTf)]B_{Ar}F$ and $[(TMC)Fe^{II}(OTf)]B_{Ar}F$ were conducted analogously.

C. Syntheses

C.1. Synthesis of stoichiometric $AgB_{Ar}F$·MeCN·THF

Although the synthesis of $AgB_{Ar}F$ has been described in the literature,[4] the as prepared product was obtained with varying amounts of coordinated solvent in its solid form. Therefore, the synthesis was modified: To ensure dryness of the product, all syntheses were performed in a glove box under argon. 4.43 g (26.1 mmol) $AgNO_3$ were transferred into a Schlenk flask and dissolved in 15 mL acetonitrile. Then, 7.0 g (7.9 mmol) $NaB_{Ar}F$ were added and the solution was stirred overnight in the dark. Afterwards, the solution was filtered and evaporated for 24 h under reduced pressure. The dry solid was dissolved in 12 mL THF. In the following, 5 mL DCM and 1 g dry molecular sieves (3 Å) were added. The solution was kept at rest for at least 8 h. Subsequent NMR measurements still showed a small residual sodium content. The molecular sieves were filtered off and the solution was evaporated overnight. The solid residue was then added to 6 mL DCM, followed by 6 mL diethyl ether. After 4 h, a minimal amount of solid had precipitated. The solution was filtered and kept under reduced pressure overnight to obtain an off-white solid. Yield: 6.86 g (80%). ^{1}H and ^{13}C NMR measurements confirmed the stoichiometry of the compound. ^{23}Na NMR measurements confirmed the absence of NMR-detectable amounts of $NaB_{Ar}F$ as an impurity.

C.2. Synthesis of $Co(OTf)_2$

The procedure was adapted from a known synthesis by Jansky and Yoke.[5,6] 10 g (83 mmol) $CoCO_3$ (99.5%) were kept in a beaker under argon. Then, 15 mL (167 mmol; a slight excess of 3 mmol) trifluoromethanesulfonic (98%) acid were added carefully. The mixture was heated to 60 °C for 7 d during which green $CoCO_3$ changed into orange $Co(OTf)_2$. The still hot wet solid was treated under reduced pressure to remove residual water. Subsequently, the compound was poured into a flask and quickly transferred into a glove box under argon to prevent exposure to ambient moisture. 10 g of the orange solid were put into a long quartz glass tube that was closed by a Schlenk cap with rubber sealing. Under Schlenk conditions, the solid was soaked with approximately 1 mL of trifluoromethanesulfonic acid (98%). The glass tube was evacuated and slowly heated to 400 °C for 6 h in a tube oven at an angle of about 30°. **This process is dangerous (see below) and proper precautions must be taken!** Acid and residual water condensed on the part of the glass above the rubber seal: We observed the monohydrate of trifluoromethanesulfonic acid as a colorless solid, which melted upon heating.[7] In one instance, the acid condensed in the glass tap, effectively sealing the glass tube, which ultimately **led to an explosion**.

After cooling, the evacuated tube was melted off to an ampule, which contained the dry solid. Finally, the closed ampule was transferred into a glove box under argon, in which it could be opened safely. Yield: 8.7 g (24.36 mmol; 87%). Pure $Co(OTf)_2$ is extremely hygroscopic, which complicates analytic procedures. The purity of the compound was confirmed by powder X-ray diffraction under inert conditions.

C.3. Synthesis of Iron Complexes

All complex syntheses were performed in an argon-filled glove box.

C.3.1. Synthesis of [(TMC)Fe^{II}(OTf)]$B_{Ar}F$ (**1a-B_{Ar}F**)

252.5 mg (0.985 mmol; corresponding to a slight excess of 2.5 mg) TMC were dissolved in 3 mL THF and 1 mL diethyl ether in a glass vial. Then, 301.9 mg (0.975 mmol) FeI_2 beads (99.99%) were added to the solution, which was stirred for 24 h at room temperature. A brown-yellow colored solid formed, which was thoroughly washed with diethyl ether and dried in vacuo for 1 h. Then, 2 mL THF were added and the suspension was stirred while adding 1057 mg (0.975 mmol) $AgB_{Ar}F$·MeCN·THF in 1mL THF. A yellow solid precipitated and was filtered off with a syringe PTFE filter (0.45 µm mesh). The solution was evaporated to dryness under reduced pressure overnight. In the following, the solid was dissolved in 2 mL THF and 250.5 mg (0.975 mmol) AgOTf in 1 mL THF were slowly added to the stirred solution. After 10 min, the yellow precipitate was filtered off and the solution was evaporated to dryness. Dissolving in THF and slow evaporation gave crystals suitable for X-ray analysis. The yield was almost quantitative, 1290.0 mg (0.974 mmol; > 99%). Anal. calc. for $C_{47}H_{45}F_{27}FeN_4O_3S$: C 42.6%, H 3.4%, N 4.2% found: C 42.4%, H 3.5%, N 4.1%.

C.3.2. Synthesis of [(D_{12}-TMC)Fe^{II}(I)]I

252.5 mg (0.940 mmol; corresponding to a slight excess of 2.5 mg) D_{12}-TMC were dissolved in 3 mL THF and 1 mL diethyl ether in a glass vial. Then, 304.88 mg (0.985 mmol) FeI_2 beads (99.99%) were added to the solution, which was stirred for 4 d at room temperature. A brown-yellow solid formed. The solid was thoroughly washed with diethyl ether and dried under reduced pressure for 1 h. After dissolving the solid in THF and slow evaporation, crystals were obtained, which unfortunately proved inadequate for X-ray analysis.

C.3.3. Synthesis of [(D_{12}-TMC)Fe^{II}(I)]$B_{Ar}F$ (**2b-B_{Ar}F**)

100.0 mg (0.173 mmol) [(D_{12}-TMC)Fe^{II}(I)]I were put into a glass vial and 2 mL THF were added. One equivalent 187.6 mg (0.173 mmol) $AgB_{Ar}F$·MeCN·THF in 2 mL THF were added slowly to the stirred suspension. The yellow precipitate of AgI was filtered off with a syringe PTFE filter (0.45 µm mesh). The solution was evaporated under reduced pressure overnight. Dissolving the solid in small amounts of THF followed by slow evaporation gave crystals suitable for X-ray analysis.

C.3.4. Synthesis of [(D_{12}-TMC)Fe^{II}(OTf)]$B_{Ar}F$ (**1b-B_{Ar}F**)

100.0 mg (0.0761 mmol) [(D_{12}-TMC)Fe^{II}(I)]$B_{Ar}F$ were put into a glass vial and 2 mL THF were added. 19.5 mg (0.076 mmol) AgOTf in 1 mL THF were added slowly to the stirred suspension. The yellow

precipitate of AgI was filtered off with a syringe PTFE filter (0.45 µm mesh). The solution was slowly evaporated and crystals suitable for X-ray analysis were obtained.

C.3.5. Synthesis of [(D_{12}-TMC)Fe^{II}(F)]$B_{Ar}F$ (**3b-B_{Ar}F**)

50.0 mg (0.380 mmol) [(D_{12}-TMC)Fe^{II}(I)]$B_{Ar}F$ were put into a brown glass vial and 3 mL of 4:1 Me-THF/THF were added. Then 4.82 mg (0.380 mmol) AgF were added at once and the suspension was stirred vigorously for 3-5 days due to the low solubility of AgF. A yellow precipitate formed that was filtered off with a syringe PTFE filter (0.45 µm mesh). The received solution was transferred to another vial and evaporated in vacuo. A pale yellow solid was obtained. Dissolving the solid in small amounts of THF followed by slow evaporation gave crystals suitable for X-ray analysis.

C.4. Synthesis of Cobalt Complexes

All complex syntheses were performed in an argon-filled glove box.

C.4.1. Synthesis of [(TMC)Co^{II}(CH_3CN)]$(OTf)_2$ (**4a-$(OTf)_2$**)

100.0 mg (0.389 mmol) TMC were added into a vial with 2 mL acetonitrile while stirring. Then, 139.2 mg (0.390 mmol) $Co(OTf)_2$ were added to the solution. After 24 h, a purple solution had formed. 2-3 mL diethyl ether were added over the course of a week while keeping the solution at –40°C. Crystals suitable for X-ray analysis formed after a few days.

C.4.2. Synthesis of [(TMC)$Co^{II}(OTf)_2$] (**5a**)

250.0 mg (0.975 mmol) TMC were added into a vial and dissolved in 3 mL THF. Then, 348.1 mg (0.975 mmol) $Co(OTf)_2$ were added to the solution, which was stirred for 24 h at room temperature. Slow evaporation of the solvent led to the formation of few crystals suitable for X-ray analysis. The high sensitivity of the complex towards moisture was observed during elemental analysis. Brief contact to air prior to the measurements immediately led to coordination of one equivalent of water. Anal. calc. for $C_{16}H_{32}CoF_6N_4O_6S_2 \cdot H_2O$: C 30.4%, H 5.4%, N 8.9% found: C 30.3%, H 4.9%, N 9.0%

C.4.3. Synthesis of [(TMC)Co^{II}(I)]I and [(D_{12}-TMC)Co^{II}(I)]I

In a brown glass vial, 252.5 mg (0.985 mmol; corresponding to a slight excess of 2.5 mg) TMC were dissolved in 3 mL THF and 1 mL diethyl ether. Then, 304.9 mg (0.974 mmol) CoI_2 beads (99.99%) were added to the solution, which was stirred for 48 h at room temperature. A green-grey solid formed, which was washed with diethyl ether to remove any residual ligand and dried under reduced pressure. The synthesis of [(D_{12}-TMC)Co(I)]I was performed analogously.

C.4.4. Synthesis of [(TMC)Co^{II}(I)]B_{Ar}F

100.0 mg (0.176 mmol) [(TMC)Co^{II}(I)]I were added to a brown glass vial and stirred in 2 mL THF. Then, *exactly* one equivalent, 190.5 mg (0.176 mmol) AgB_{Ar}F·MeCN·THF in 1 mL THF were added slowly to the solution, which was stirred for 15 min. Green [(TMC)Co^{II}(I)]B_{Ar}F formed in solution and yellow AgI precipitated. Then, the yellow solid was removed by a syringe PTFE filter (0.45 µm) and the filtrate was transferred to another brown glass vial. The solution was evaporated under reduced pressure overnight. During most experiments, no residual acetonitrile remained. In some cases, however, the remaining solid still appeared gel-like. The compound was then dissolved in DCM and the solution was evaporated to dryness. Dissolving the solid in THF followed by slow evaporation led to crystals suitable for X-ray analysis.

C.4.5. Synthesis of [(TMC)Co^{II}(OTf)]B_{Ar}F (**5a-B_{Ar}F**) and [(D_{12}-TMC)Co^{II} (OTf)]B_{Ar}F (**5b-B_{Ar}F**)

For this procedure, precisely weighed amounts of reagents are required. Even minor deviations can lead to side products, such as [(TMC)Co^{II}(OTf)$_2$] in the event of excess AgOTf. 100.0 mg (0.176 mmol) [(TMC)Co^{II}(I)]B_{Ar}F were added into a vial and suspended in 2 mL THF under stirring. Then, one equivalent, 190.1 mg (0.176 mmol), AgB_{Ar}F·MeCN·THF dissolved in 1 mL THF were added slowly to the suspension and stirring was continued for another 15 min. The solution turned green and yellow AgI precipitated. The yellow solid was removed by a syringe PTFE filter (0.45 µm) and the filtrate was transferred to another brown glass vial. The solution was evaporated to dryness under reduced pressure overnight. The dried green solid was weighed before dissolving in THF and one equivalent, 45.1 mg (0.176 mmol) AgOTf was added. Again, AgI precipitated while the product appeared pink in solution. The solid was removed with a syringe PTFE filter (0.45 µm mesh) and the solution was concentrated under reduced pressure to force agglomeration of any residual suspended AgI, which was filtered off. Dissolving the obtained solid in THF followed by evaporation lead to crystals suitable for X-ray analysis. The yield was almost quantitative, 233.2 mg (0.176 mmol; > 99%). Anal. calc. for $C_{47}H_{45}CoF_{27}N_4O_3S$: C 42.6%, H 3.4%, N 4.2% found: C 42.8%, H 3.5%, N 4.2%. The synthesis of [(D_{12}-TMC)Co^{II}(OTf)]B_{Ar}F (**5b-B_{Ar}F**) was performed analogously.

C.4.6. Synthesis of [(TMC)Co^{II}(ClO_4)]B_{Ar}F (**6a-B_{Ar}F**)

We devised two different methods:

Method A: 100.0 mg (0.390 mmol) TMC were put into a vial and dissolved in 3 mL MeOH. While stirring, 0.143 g (0.390 mmol) $Co(ClO_4)_2$· 6 H_2O were added and the solution turned pink. Then, 389.7 mg (0.3899 mmol) NaB_{Ar}F were added. After 24 h, the solution turned purple and a colorless solid precipitated. The solid was removed with a syringe PTFE filter (0.45 µm) and the solution was slowly evaporated. Some crystals suitable for X-ray analysis were obtained after crystal picking. The bulk of colorless crystals was NaB_{Ar}F.

Method B: 100.0 mg (0.077 mmol) [(TMC)Co^{II}(I)]B$_{Ar}$F were kept in a brown glass vial and dissolved in 3 mL THF. Then, 0.016 g (0.077 mmol) pre-dried $AgClO_4$ were added. AgI precipitated and was removed via a syringe PTFE filter (0.45 µm). After slow evaporation of the solvent, some crystals along with amorphous product were obtained. The yield was almost quantitative, 97.8 mg (0.077 mmol; > 99%).

C.4.7. Synthesis of [(TMC)Co^{II}(OTf)][Al{OC(CF_3)$_3$}$_4$]

100.0 mg (0.163 mmol) [(TMC)Co^{II}(OTf)$_2$] were put into a vial and dissolved in 1 mL 2-MeTHF and 1 mL diethyl ether. Then, 158.8 mg (0.163 mmol) Li[Al{OC(CF_3)$_3$}$_4$] were added. The solution was heated to 50 °C for 7 d. Afterwards, the solution was evaporated under reduced pressure overnight. The obtained solid was dissolved again in 3 mL 2-MeTHF and addition of a small amount of pentane precipitated LiOTf from solution as a colorless solid. This step was repeated at least three times. The solution was filtered again and slow evaporation of the solvent gave a colorless residue alongside some pink to purple crystals suitable for X-ray analysis.

C.4.8. Synthesis of [(D$_{12}$-TMC)Co^{II}(F)]B$_{Ar}$F (**7b-B$_{Ar}$F**)

50 mg (0.3794 mmol) [(D$_{12}$-TMC)Co^{II}(I)]B$_{Ar}$F were put into a brown glass vial and 3 mL of 4:1 Me-THF/THF were added. Then 4.81 mg (0.3794 mmol) AgF were added at once and the suspension was stirred vigorously for 3-5 d due to the low solubility of AgF. A yellow precipitate formed that was removed using a syringe PTFE filter (0.45 µm mesh). The solution was transferred to another vial and the solvent evaporated in vacuo. A pale lavender colored solid was obtained. Dissolving the solid in small amounts of THF followed by slow evaporation gave crystals suitable for X-ray analysis.

C.4.9. Synthesis of [(TMC)Co^{II}(Br)]Br · H_2O

50.00 mg (0.1949 mmol) of TMC were added into a vial and dissolved in 2 mL of acetone while stirring. Then 42.65 mg (0.1949 mmol) $CoBr_2$ were added. After a day of stirring, a violet colored solution had formed. Slow evaporation led to crystals suitable for x-ray analysis.

D. NMR spectra of AgB$_{Ar}$F·MeCN·THF

Standard NMR spectra were obtained using Bruker Avance III HD 400 MHz and Bruker Avance III HD 600 MHz spectrometers.

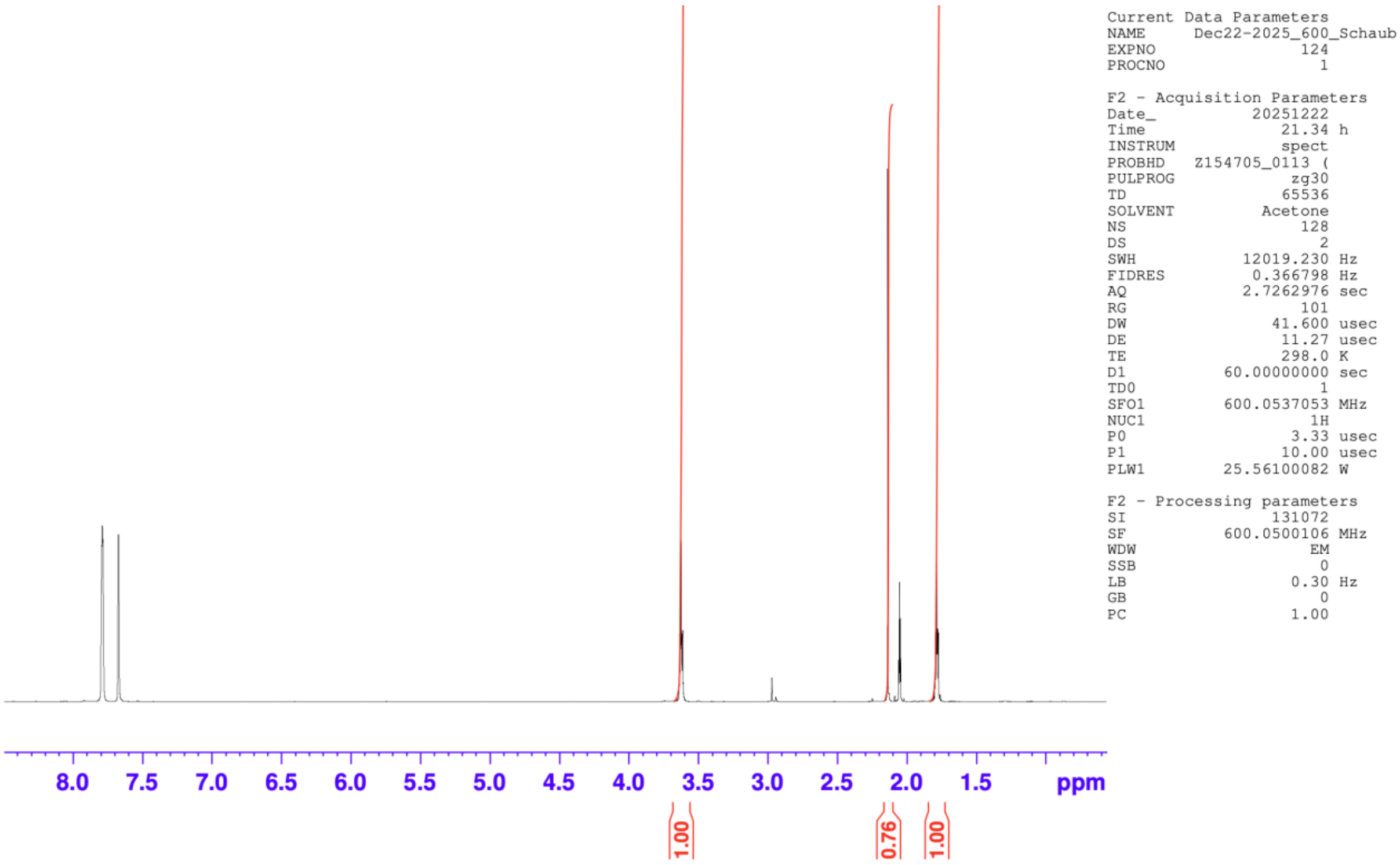

Figure S1: ^{1}H-NMR spectrum of AgB$_{Ar}$F·MeCN·THF in acetone-D$_6$.

^{1}H NMR (600 MHz, Acetone-D$_6$): δ 7.79 (m, 8H), 7.67 (br.s, 4H), 3.62 (m, 4H), 2.14 (s, 3H), 1.78 (m, 4H)

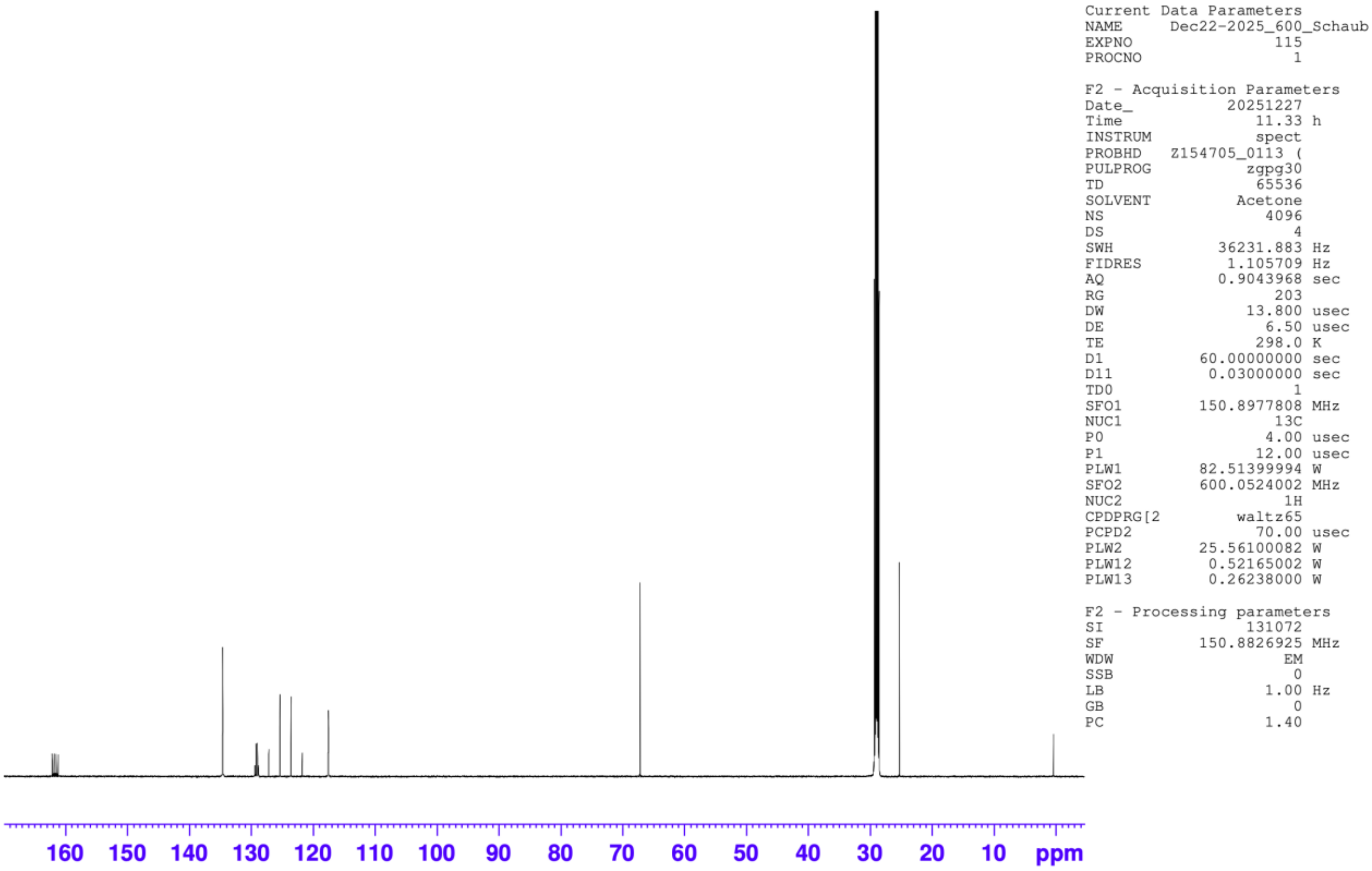

Figure S2: ^{13}C-NMR spectrum of AgB$_{ArF}$·MeCN·THF in acetone-D$_6$.

^{13}C{^{1}H} NMR (150 MHz, Acetone-D$_6$): δ 162.7, 135.6, 130.1, 125.4, 118.5 (CH,sp^2), 118.5 (Cq, sp), 68.2, 26.3, 0.33

The proton-decoupled ^{13}C spectrum of AgB$_{ArF}$·MeCN·THF in aceton-D$_6$ exhibits a well-resolved quartet (1:1:1:1) at 162.7 ppm arising from the spin coupling with the ^{11}B nucleus (I = 3/2), and a septet with lower intensity due to the spin coupling with the less abundant ^{10}B (I = 3), natural abundance 18.8 %).

In general, the coupling of ^{13}C with ^{11}B is not measureable because the ^{11}B nucleus possesses a quadrupole moment, and quadrupole relaxation is so rapid that the coupling is eliminated. Only in special cases, when the electric field gradient at the quadrupolar nucleus is zero or sufficiently small (i.e., salts, nucleus concentration = 4 at the ^{11}B, tetragonal environment), a coupling can be observed.

The ^1J (^{11}B,^{13}C) value in AgB$_{ArF}$·MeCN·THF, in which the B$_{ArF}$ anion is believed to exist as a separate ion pair, is approximately 50 Hz, which could also reflect the sp^2 character of the coupled carbon atom.

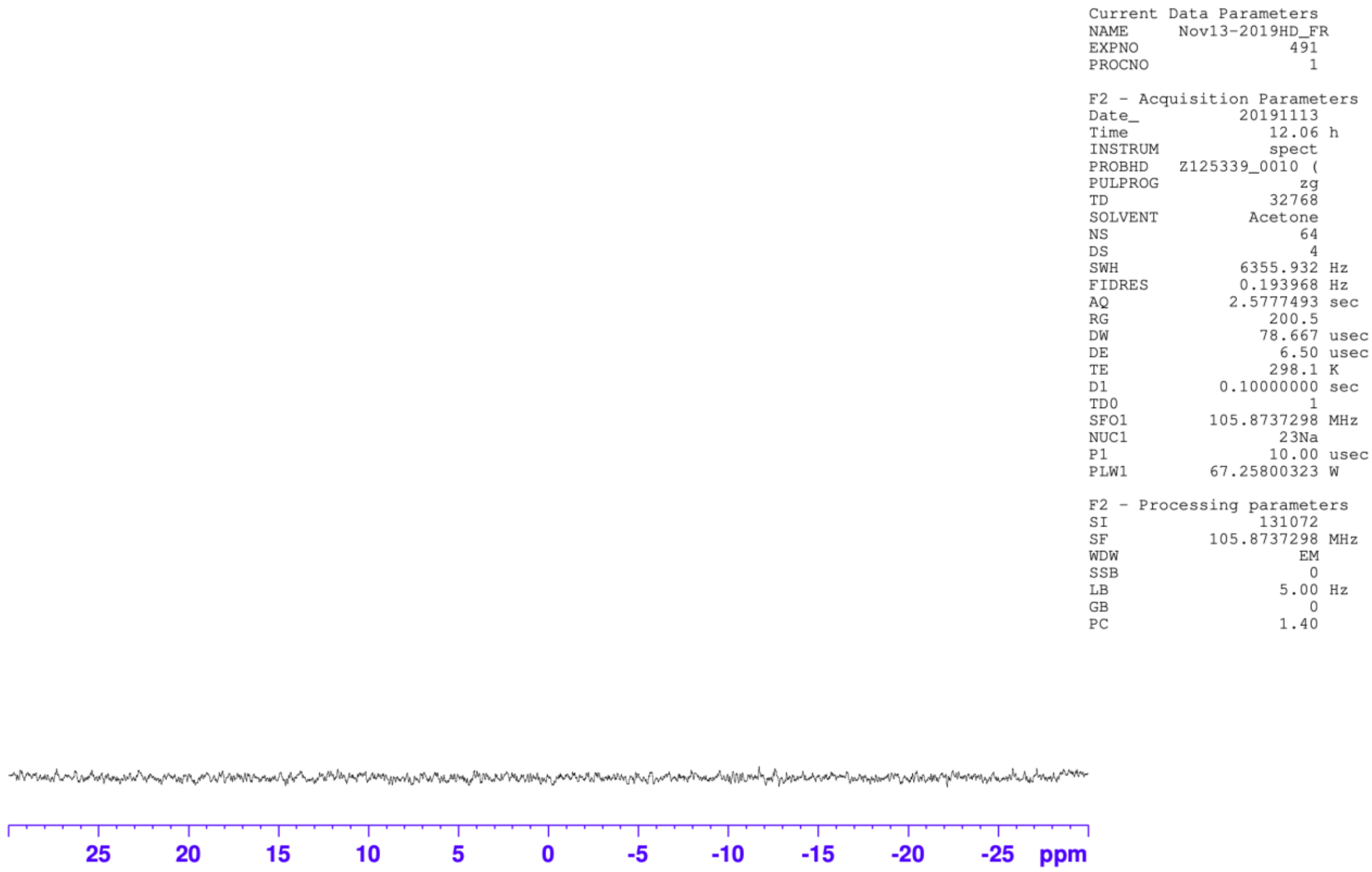

Figure S3: ^{23}Na-NMR spectrum of $AgB_{Ar}F \cdot MeCN \cdot THF$ in acetone-D_6.

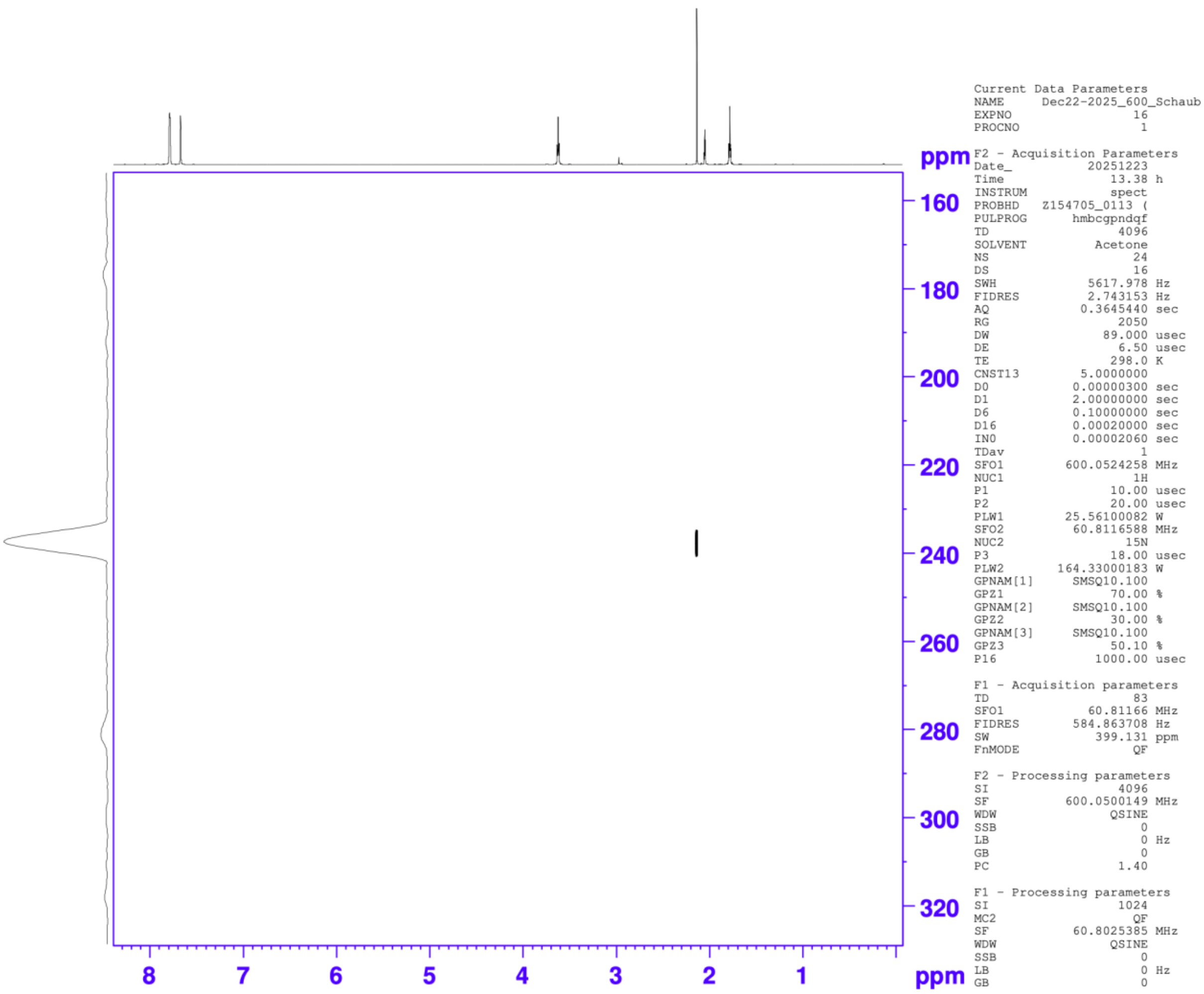

Figure S4: ^{1}H, ^{15}N-HMBC spectrum of $AgB_{Ar}F \cdot MeCN \cdot THF$ in acetone-D_6.

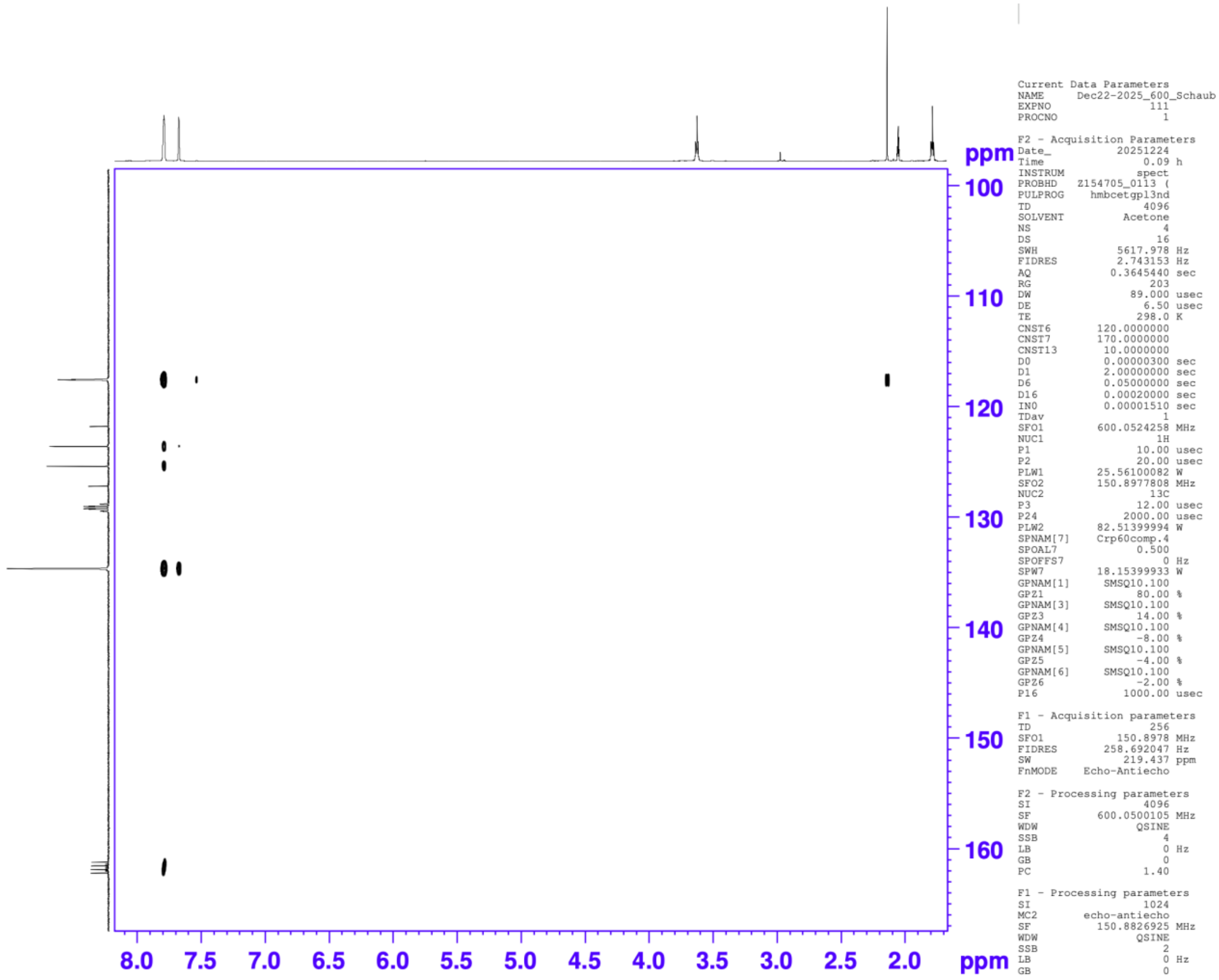

Figure S5: ^{1}H, ^{13}C-HMBC spectrum of $AgB_{Ar}F \cdot MeCN \cdot THF$ in acetone-D_6.

The ^{2}J (^{1}H,^{13}C) coupling of 9.9 Hz of the C(sp^3) protons in acetonitrile at 2.14 ppm with the sp hybridized nitrile carbon atom at 118.5 ppm can be seen in the HMBC as well as in the ^{13}C satellites of the ^{1}H signal of the methyl protons.

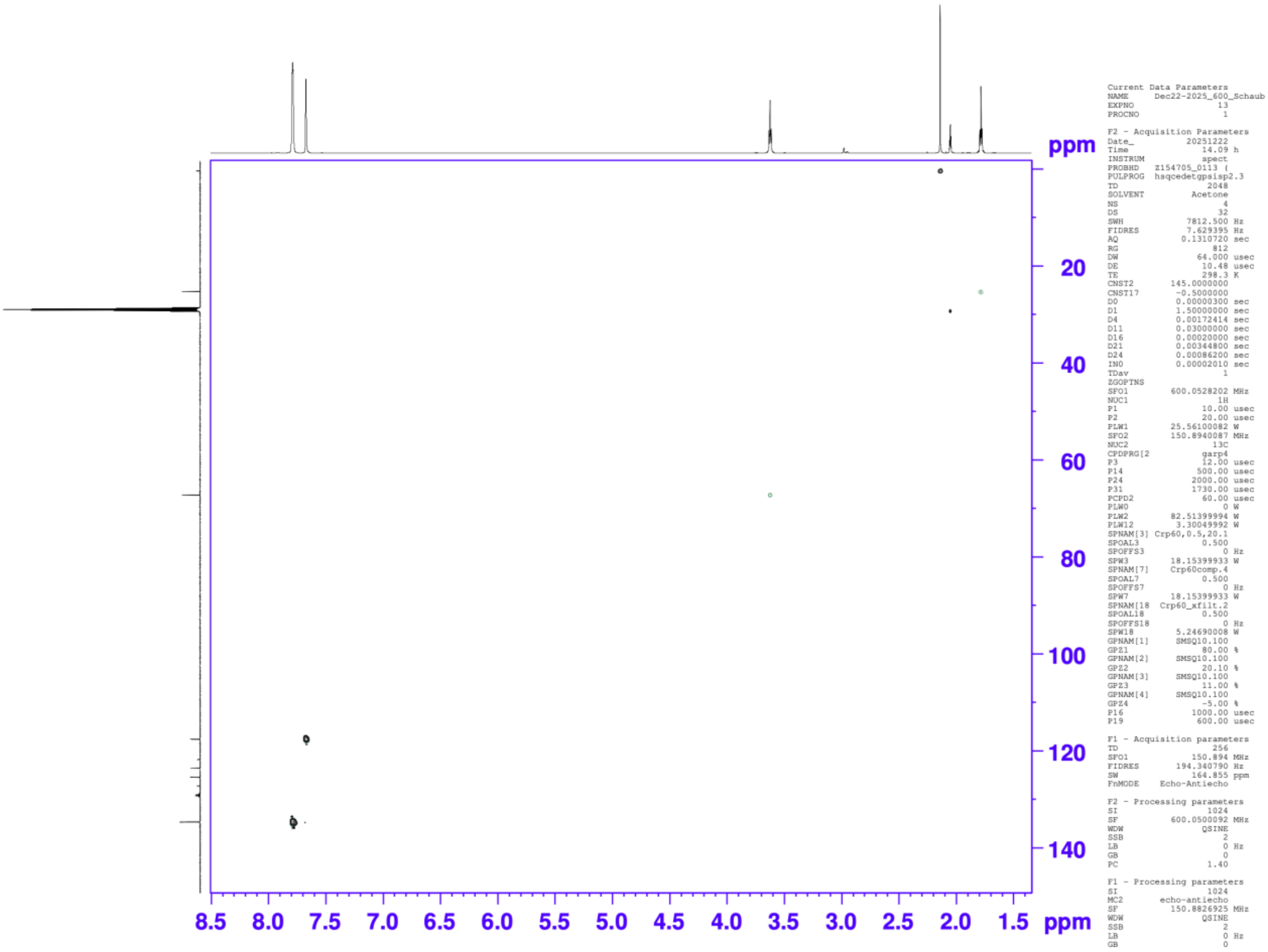

Figure S6: 135DEPT-edited HSQC spectrum of $AgB_{Ar}F$·MeCN·THF in acetone-D_6.

E. Low temperature measurement setup

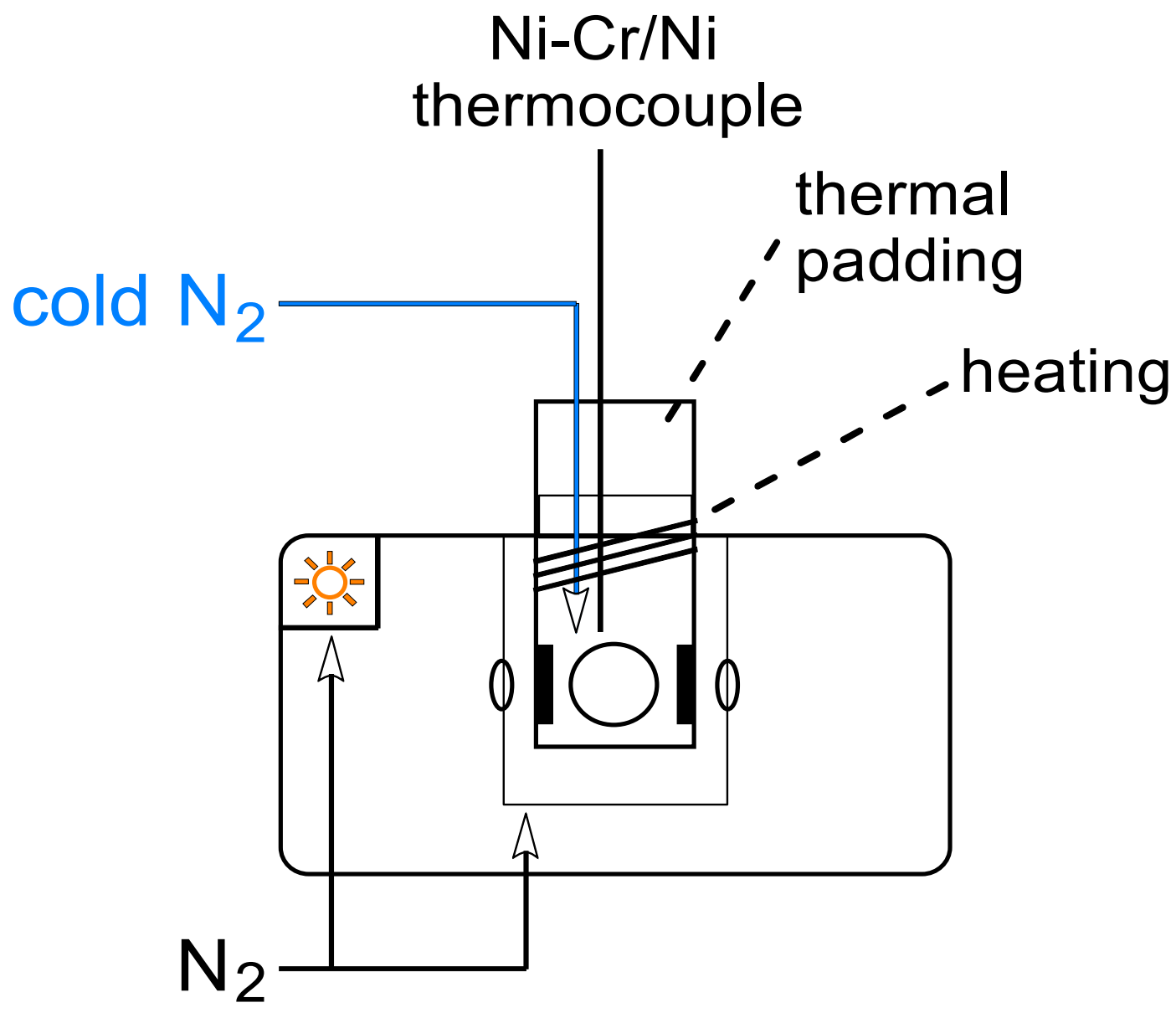

Scheme S1: Schematic assembly of nitrogen-cooled low-temperature UV-vis cell with thermal insulation, external window heating, and dry nitrogen purge gas inside the UV-vis spectrometer; optical quartz cuvette omitted

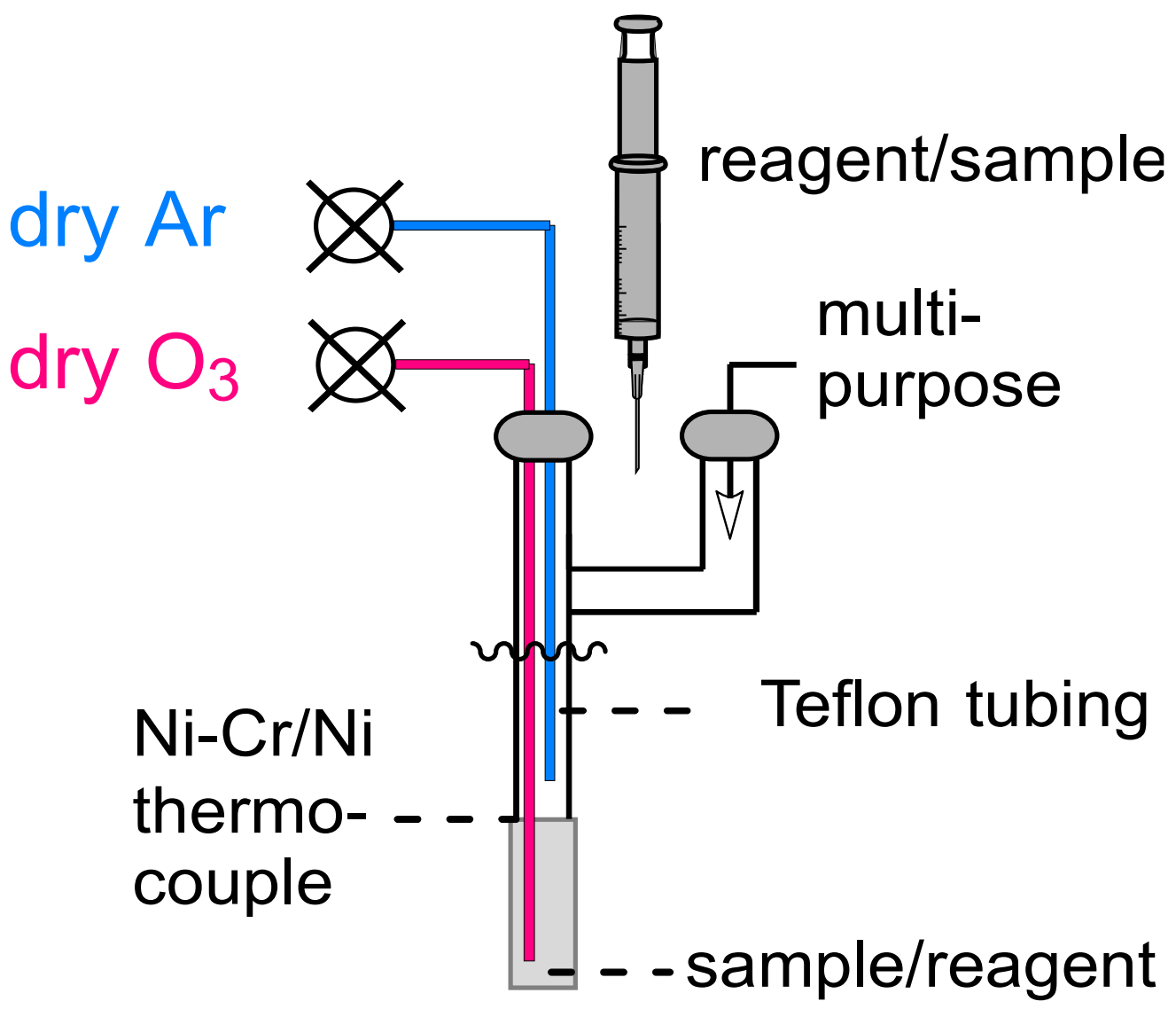

Scheme S2: Schematic depiction of the quartz Schlenk cuvette with Teflon tubing (sourced from HPLC supply) for the application of gases; dry Ar as purge gas is constantly applied above the liquid level while ozone is bubbled through the sample; samples or reagents are injected via syringe through silicone septa.

F. Glass septum cannula for ozonization on semi-preparative scale

To prevent any contact of complex solution with a metal surface, we used self-made glass septum cannulas and applied them in combination with scintillation vials with septum caps. The glass cannulas were made by cutting Pasteur pipettes into the desired length (a bit shorter than the height of the vial). The opening was further narrowed by careful melting with a Bunsen burner. We used regular Teflon tape as the septum in the glass cannula. The tape was squeezed into the cannula with a thin wood- or PVC rod to avoid any abrasion. The septum in the cannula must have a bowl or crater alike shape inside and must not be too thick: Otherwise, the septum will freeze and can no longer be pierced by the cannula.

After assembly, the glass cannula was dried under vacuum at 115 °C overnight in a special Schlenk-tube and was kept under argon in a glovebox to exclude any moisture. The used 20 mL screw neck vials were also dried under vacuum over night at 300 °C in a self-made (50 mm diameter and 65 cm length) Schlenk tube, fitting in a tube oven and in the big lock of standard glove boxes. After cooling, the Schlenk tube was transferred into a glove box while being under vacuum. The caps were just kept under vacuum overnight before also being transferred into the same glove box.

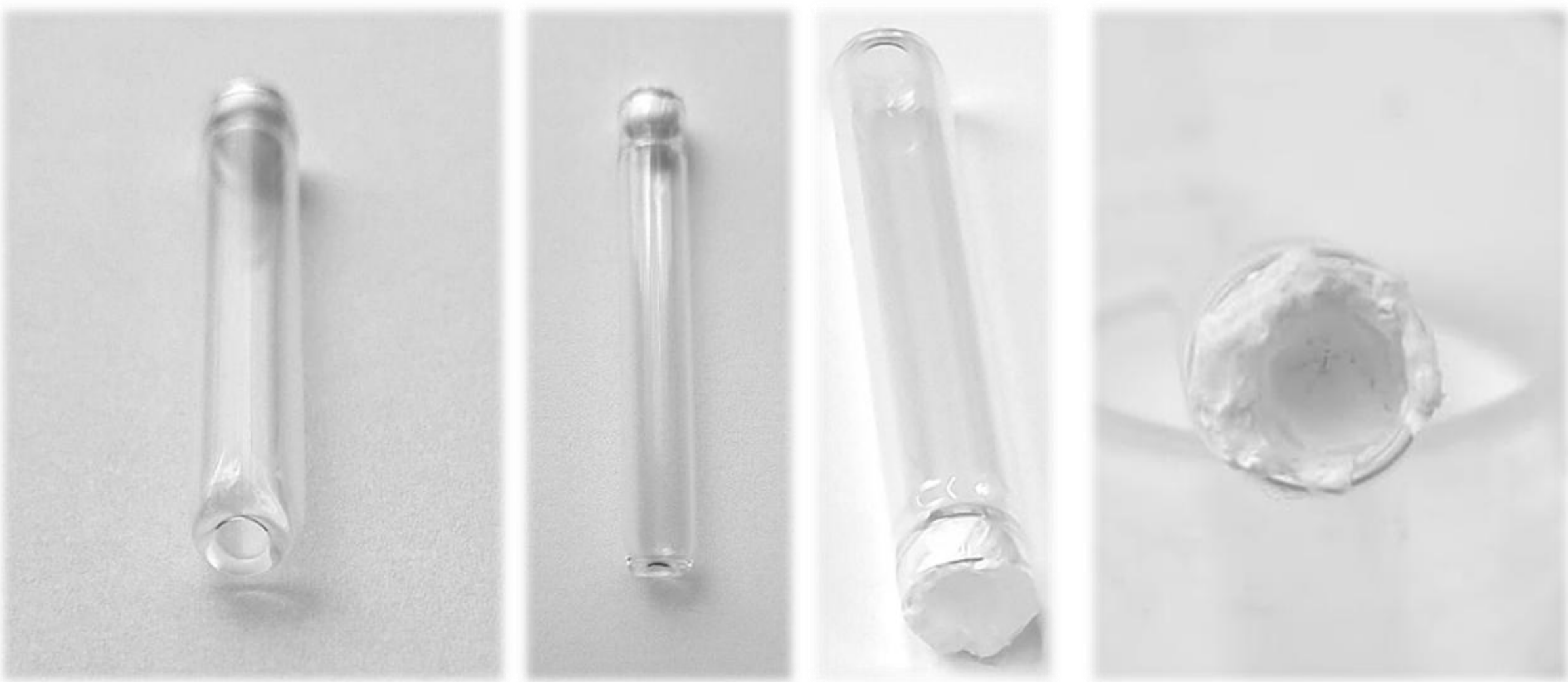

Figure S7: Glass cannula with Teflon septum.

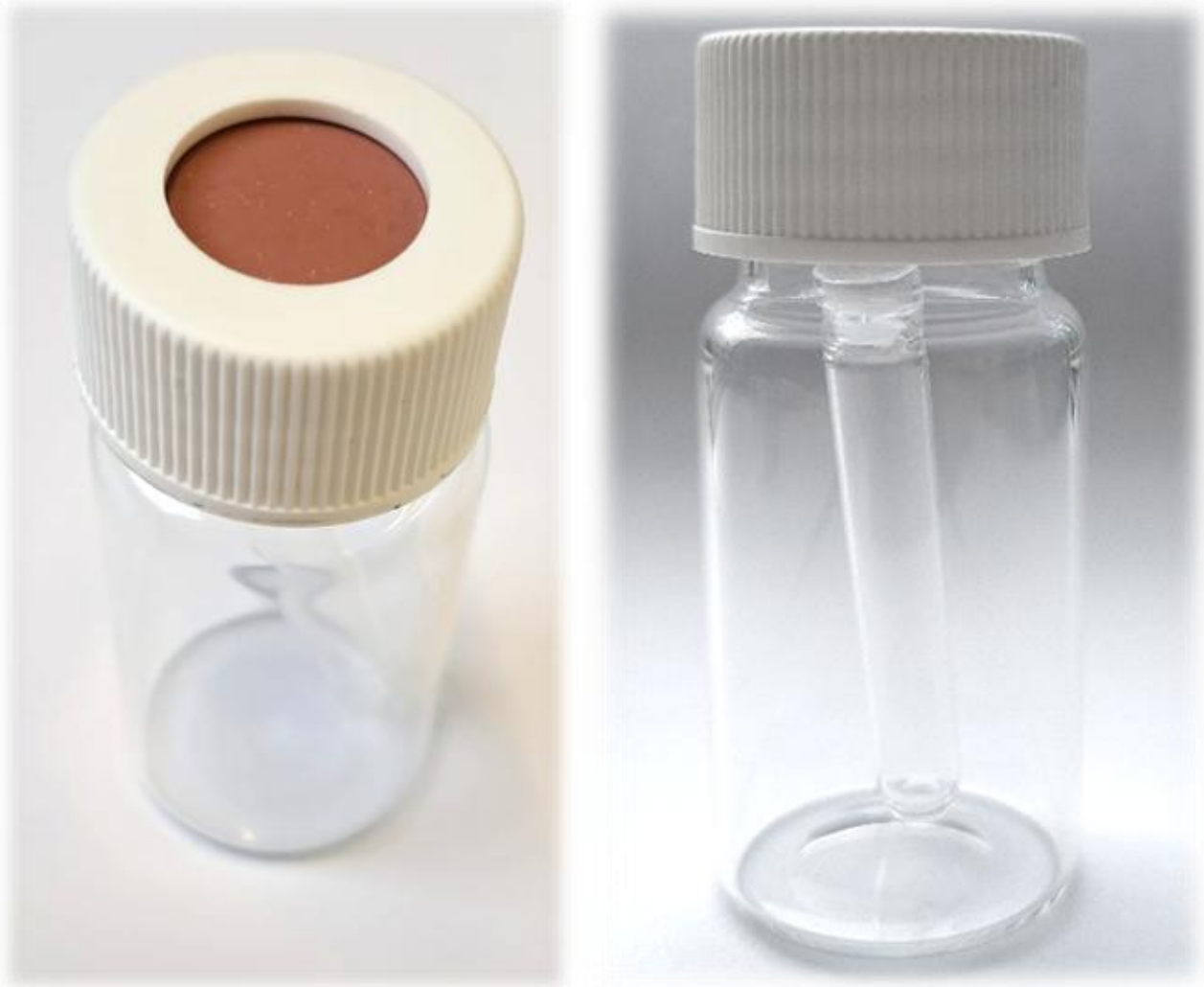

Figure S8: Glass cannula with Teflon septum in 20 mL scintillation vials (Screw neck vials 57 mm height, 27.5 mm diameter) with septa caps. Screw caps are made from polypropylene (PP); septa (22 mm diameter) are made from butyl-rubber (coated with PTFE towards the inside).

Upon use, both septa (i.e., of cap and cannula) have to be pierced with a regular metal cannula to apply ozone to the solution. To release any overpressure, the cap has to be pierced with a second metal cannula. When flammable solvents are used, *we highly recommend to inject a third cannula to apply a (moderate) continuous stream of inert gas into the scintillation vial to prevent the formation of potentially explosive solvent vapors*. In general, the height of the complex solution should not exceed 2-3 mL to prevent contact of the solution with the metal cannula due to hydrostatic pressure.

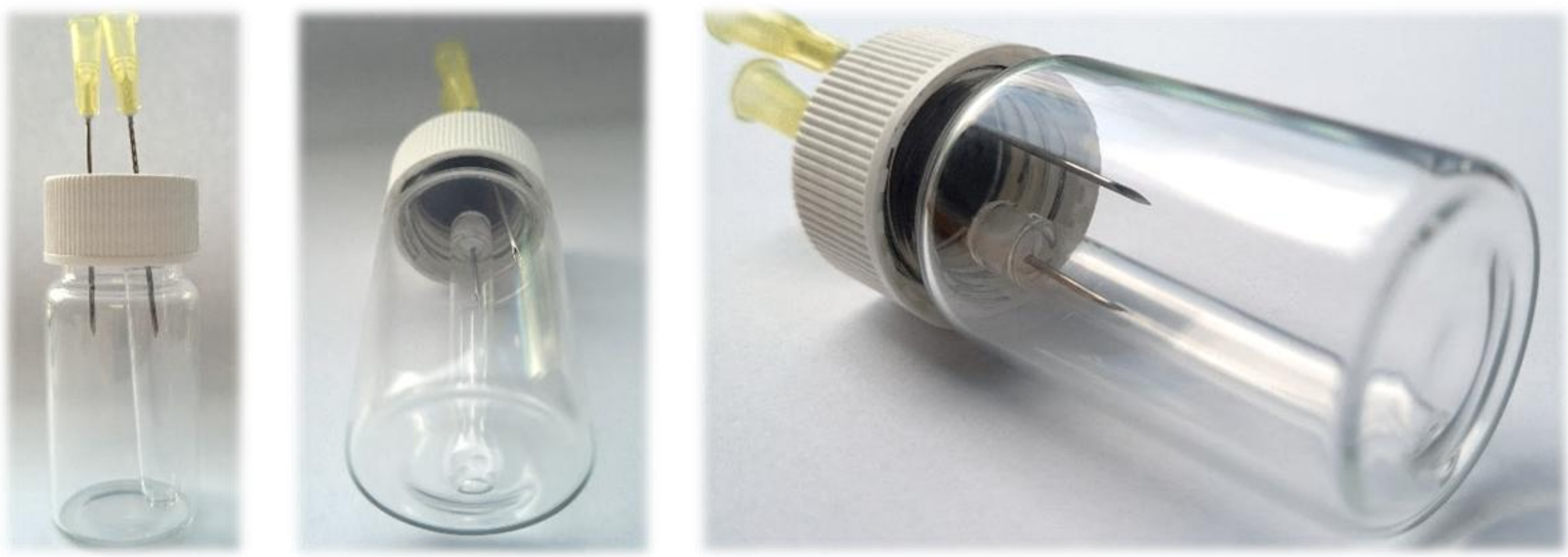

Figure S9: Glass cannula in scintillation vial with pierced septa for ozonization and pressure relief with "inert" solvents like DCM.

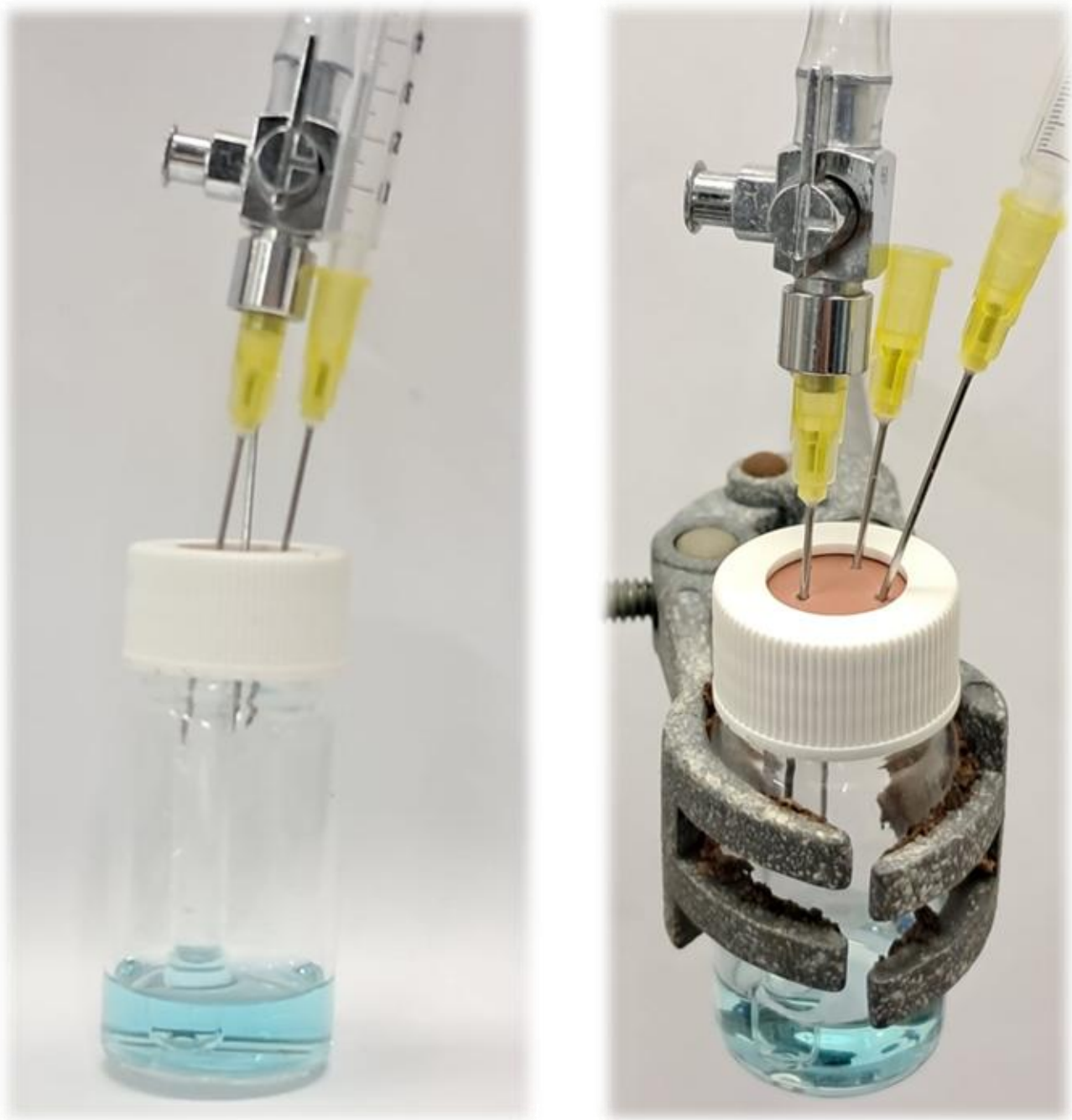

Figure S10: Glass cannula in scintillation vial with triple pierced septa for ozonization, pressure relief, and argon influx. The third cannula with argon is necessary to prevent spontaneous vapor combustion when using flammable solvents like Me-THF/THF. The blue liquid is exemplary and shows the level of solution that can be safely handled.

During ozonization, the vial was partially submerged in an isopentane bath cooled with liquid nitrogen to as low as –150 °C. For safety reasons, the argon purge was allowed to continue for at least ten seconds after ozonization to remove as much ozone above the complex solution as possible.

References

[1] S. Schaub, A. Miska, J. Becker, S. Zahn, D. Mollenhauer, S. Sakshath, V. Schünemann, S. Schindler, "Synthesis of an Iron(IV) Aqua–Oxido Complex Using Ozone as an Oxidant" *Angew. Chem. Int. Ed.* **2018**, *57*, 5355–5358.

[2] N. A. Yakelis, R. G. Bergman, "Safe Preparation and Purification of Sodium Tetrakis[(3,5-trifluoromethyl)phenyl]borate ($NaBArF_{24}$): Reliable and Sensitive Analysis of Water in Solutions of Fluorinated Tetraarylborates" *Organometallics* **2005**, *24*, 3579–3581.

[3] M. Jansen, H. Nuss, "Ionic Ozonides" *Z. Anorg. Allg. Chem.* **2007**, *633*, 1307–1315.

[4] D. Coucouvanis in *Inorganic Syntheses 33* (Ed.: P.P. Power), John Wiley & Sons, **2002**, p. 86.

[5] M. T. Jansky, Thesis, Oregon State University, **1978**.

[6] M. T. Jansky, J. T. Yoke, "Hydrates and tertiary amine complexes of cobalt(II) and nickel(II) trifluoromethanesulfonates" *J. Inorg. Nucl. Chem.* **1979**, *41*, 1707–1709.

[7] Ronald Corkum, John Milne, "The density, electrical conductivity, freezing point, and viscosity of mixtures of trifluoromethanesulfonic acid and water" *Can. J. Chem.* **1978**, *56*, 1832–1835.
