# Supplementary material for: First Evidence for Ozonido‐TMC Complexes of Iron and Cobalt
Source: Angew Chem Int Ed Engl. 2026 Jul 9;65(37):e4716492. doi: 10.1002/anie.4716492 (PMC13548893; doi:10.1002/anie.4716492)
Supplement: Supplementary file 5 [file ANIE-65-e4716492-s005.pdf]

Supporting Information Part V — EPR spectroscopy

A. General Procedures 2

B. EPR measurements of [(TMC)Fe^{II}(OTf)]$B_{Ar}F$ + O_3 3

C. EPR measurements of [(TMC)Co^{II}(OTf)]$B_{Ar}F$ / [(D_{12}-TMC)Co^{II}(OTf)]$B_{Ar}F$ + O_3 5

A. General Procedures

X-band EPR spectra were measured on a Bruker Elexsys 500 system, equipped with a RDK-408 Cryocooler as part of a Bruker CE-EPR4K-00100 In-Cavity Cryo-Free VT system and an ER 4116DM dual mode resonator in frozen solutions at 5-6.5 K, using the following main instrument settings: receiver gain: 60 dB; microwave power: 1.993 mW; modulation amplitude: 3 G; modulation frequency: 100 kHz; microwave frequencies are given in the captions of the figures for each spectrum. All spectra were baseline corrected using the Bruker Xepr 2.6b.86 software.

The EPR glass vials and Pasteur pipettes were dried in a Schlenk tube at 250 °C for 8 h under reduced pressure in a tube oven. To keep them dry, the Schlenk tube was afterwards transferred to a glove bag, where the atmosphere was removed by repeated pumping and flooding with argon. The process was performed six times. The silicone caps for the EPR vials were again dried under reduced pressure in a Schlenk tube for 8 h at room temperature. The samples were quickly transferred into the glove bag and cooled along with the EPR glass vials in an isopentane bath with liquid nitrogen at –145 °C. To prevent problems during the measurements, we used a custom setup to displace the evaporating nitrogen with a steady stream of argon. The samples were then quickly transferred from the reaction vials into the EPR glass vials via pre-cooled Pasteur pipettes. All samples were prepared 48 h prior to measurement and intermittently stored at –145 °C.

B. EPR measurements of $[(TMC)Fe^{II}(OTf)]B_{Ar}F + O_3$

The X-band EPR spectrum of the iron complex $[(TMC)Fe^{II}(OTf)]B_{Ar}F$ before and after its reaction with ozone is presented in Figure S1. In the first case, essentially no EPR signal is observed, as expected for high-spin Fe(II) ($3d^6$, $S_{Fe} = 2$). Although low-field EPR signals can be observed for Fe(II), particularly with rhombic symmetry and $D < 0$,[1,2] no such spectra were observed in the present case. Such signals are enhanced by parallel mode EPR detection, which was not available here. There is an extremely weak feature at ~160 mT that may be due to trace Fe(III) and a weak, but distinct signal at ~340 mT that is due to a very small amount of an unknown radical species (see Figure S1, inset). The first derivative presentation of conventional EPR exaggerates the amount of narrow/isotropic (e.g., radical) signals relative to broader/anisotropic (e.g., transition metals) signals.

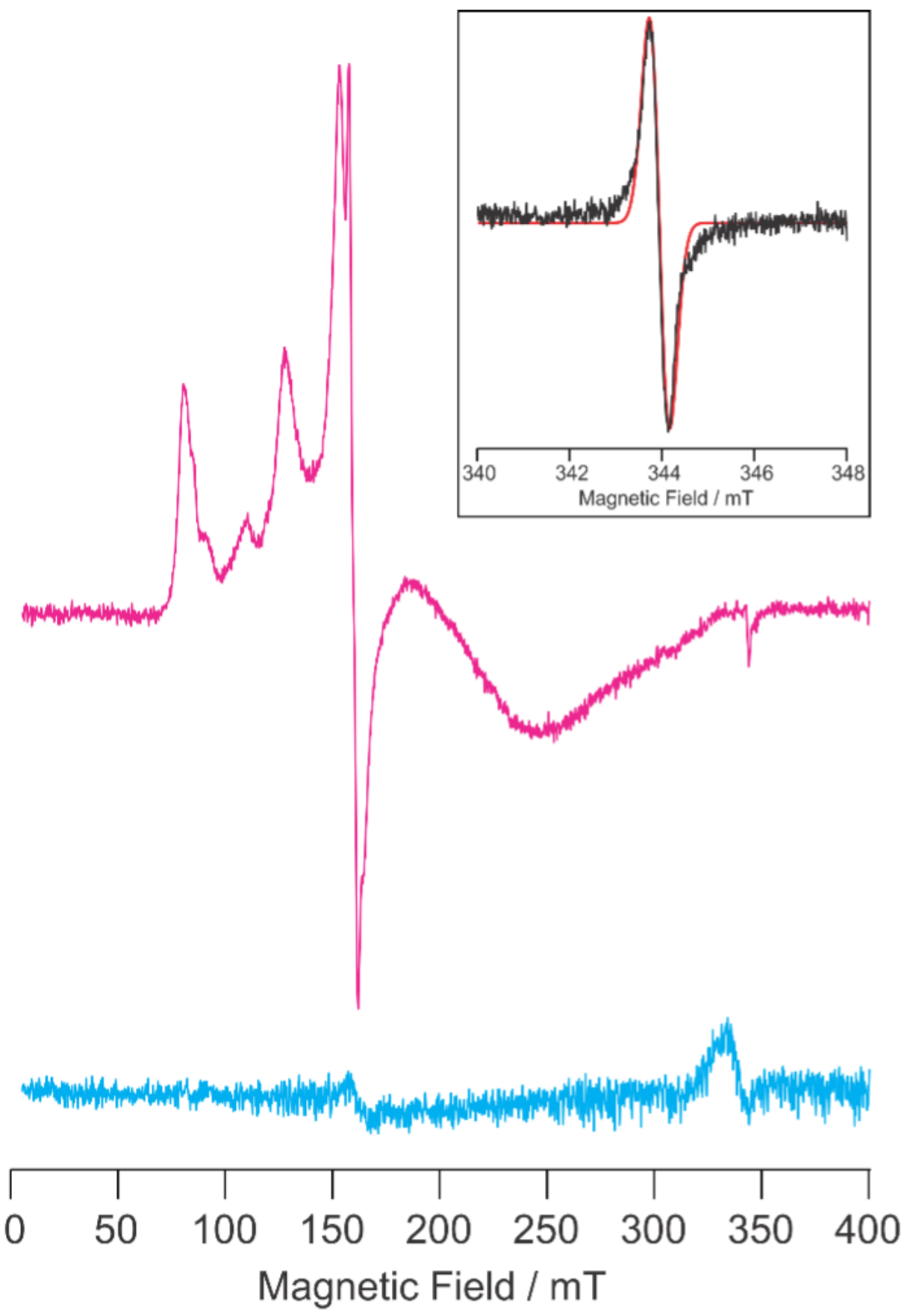

Figure S1: X-band EPR spectra of $[(TMC)Fe^{II}(OTf)]B_{Ar}F$ solutions: Cyan trace is before (9.6328 GHz, 7.3 K), magenta trace is after (9.6367 GHz, 6.3 K) reaction with ozone. Experimental conditions of microwave power, field modulation amplitude, time constant, scan time, gain, etc. were identical among the spectra. The inset shows the persistent radical present in low concentration (black trace, 9.6330 GHz, 6.5 K) together with a simulation ($g = 2.0012$, $W = 7$ MHz).

After the reaction with ozone, strong EPR signals result that are characteristic of high-spin Fe(III) ($3d^5$, $S_{Fe} = 5/2$) in a highly rhombic environment ($|E/D| \approx 1/3$),[3] as is commonly found in this coordination

sphere (i.e., N, O donors). HFEPR, or at least several microwave frequencies, would be needed to determine the zfs accurately.[4,5] Given that the spectra are utterly typical of rhombic Fe(III), it was not deemed worthwhile to perform EPR simulations to extract specific spin Hamiltonian parameters. Such efforts are labor intensive,[6–9] and would provide no further information relevant to the focus on reactivity of the present study.

Unfortunately, EPR measurements were unable to provide evidence for ozonation producing other paramagnetic species of interest: Fe(IV) oxido species (*S* = 1) such as produced here are "EPR-silent" using conventional spectrometers.

C. EPR measurements of $[(TMC)Co^{II}(OTf)]B_{Ar}F$ / $[(D_{12}\text{-}TMC)Co^{II}(OTf)]B_{Ar}F + O_3$

EPR spectra at X-band and low temperature (9.63 GHz; 6.4 K) were recorded for cobalt(II) complexes $[(TMC)Co^{II}(OTf)]B_{Ar}F$ and $[(D_{12}\text{-}TMC)Co^{II}(OTf)]B_{Ar}F$, for which we found no discernable spectral difference. As shown in Figure S2, $[(TMC)Co^{II}(OTf)]B_{Ar}F$ exhibits signals with effective g values of g'_{max} = 7.32, g'_{mid} = 1.93, and g'_{min} = 1.345 (each determined by simulation using an effective spin, S' = 1/2), with an additional feature at $g' \approx 2.65$. There is resolved ^{59}Co (I = 7/2, 100% abundance) hyperfine splitting at g'_{max} corresponding to $A'_{max}(^{59}Co)$ = 805 MHz. The g' values allow estimation of the rhombicity ($|E/D|$) of the zero-field splitting (zfs) interaction in the actual spin, S_{Co} = 3/2 (high-spin, $3d^7$), system by use of perturbation theory expressions.[10] With only X-band (i.e., a single, low microwave frequency) data, it is impossible to determine accurately the magnitude of zfs; for this, multiple, high-frequency and -field EPR (HFEPR) investigation would be required,[11] which is unfeasible due to the sensitivity of the complex. From the spectral signature, it is evident that $D < 0$ and $|D| \gg hc\tilde{\nu}$, where $\tilde{\nu}$ is the X-band energy (9.63 GHz; 0.321 cm^{-1}). A reasonable simulation could be obtained with use of the following S_{Co} = 3/2 spin Hamiltonian parameters: D = –4.674 cm^{-1} (5.0 T in equivalent field units), E = –1.1685 cm^{-1} (assigned the same sign as D by convention), $\boldsymbol{g}$ = [2.50, 2.24, 2.57], $\boldsymbol{A}(^{59}Co)$ = [260, 150, 280] MHz. Only $A_z(^{59}Co)$ = 280 MHz is well-determined, as shown in Figure 7 inset. This spectral region suggests that in solution there may be more than one conformation of $[(TMC)Co^{II}(OTf)]B_{Ar}F$, leading to slightly different $A_z(^{59}Co)$ values (and presumably the other parameters as well). The magnitude of D is not a unique solution, but is plausible based on values reported for high-spin Co(II) complexes[12–14] with four- and five-coordination.

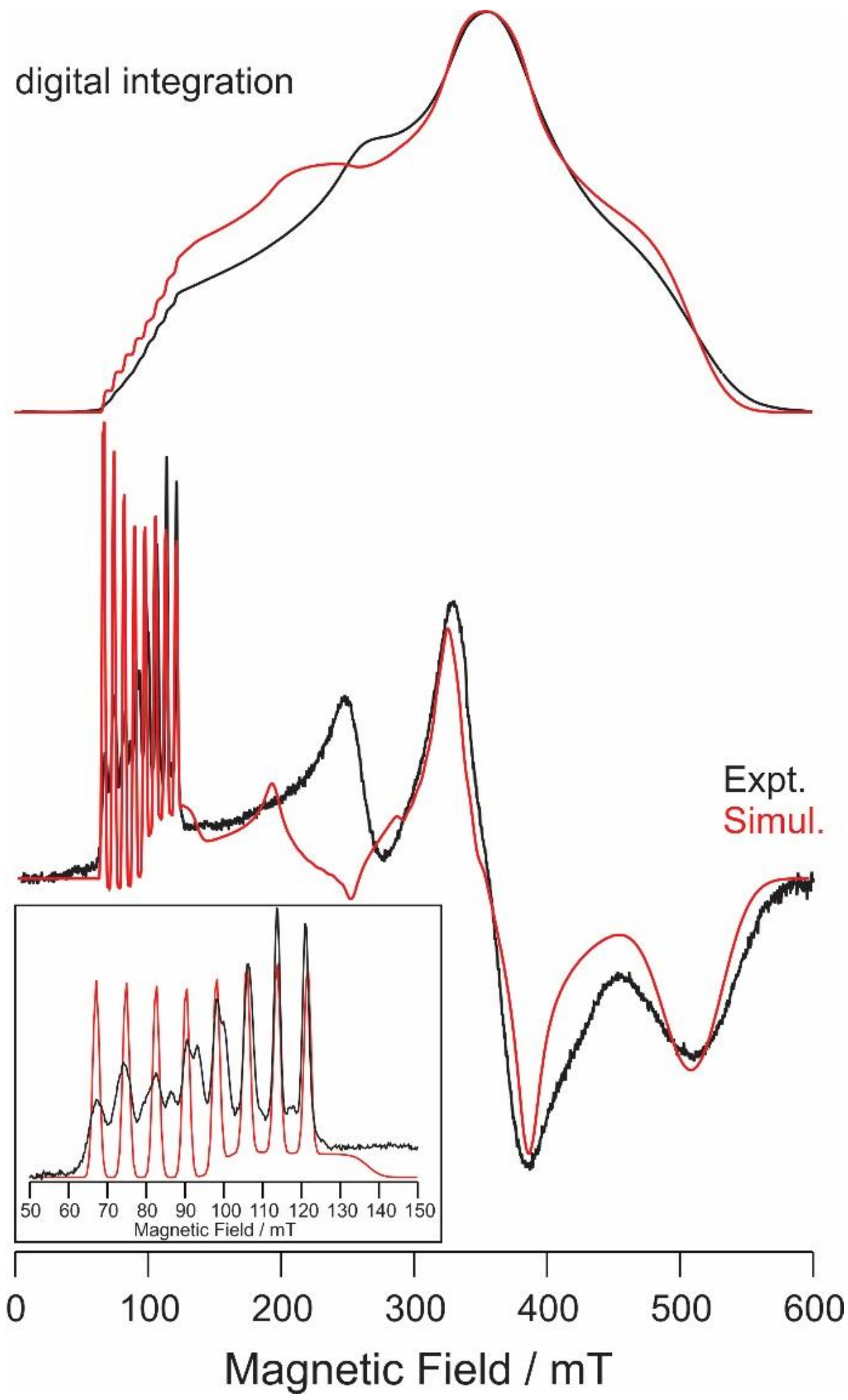

Figure S2: X-band EPR spectrum of [(TMC)Co^{II}(OTf)]$B_{Ar}F$ in Me-THF/THF (4:1) solution recorded at 9.6630 GHz and 6.4 K (black trace) with simulation (red trace) using S_{Co} = 3/2 spin Hamiltonian with: D = –4.674 cm^{-1}, E = –1.1685 cm^{-1}, $\boldsymbol{g}$ = [2.50, 2.24, 2.57], $\boldsymbol{A}$(^{59}Co) = [260, 150, 280] MHz, with Gaussian linewidths (hwhh) W_x = 200, W_y = 400, W_z = 100 MHz. The inset shows an expansion of the low field (g_z) region (50-150 mT), which suggests that there may be a minor conformation not reproduced in the simulation. Digital integrations of the experimental and simulated spectra are shown at top to provide another means of visualization. The match between experiment and simulation in the region ~200-300 mT (~3.4 < g > 2.3) is poor due to both the presence of a minor conformation and the inability to determine the magnitude of D absent multi-frequency EPR spectra. The inability to resolve A(^{59}Co) at other than the minimum resonant field (g_z) is an added complication.

Reaction of [(TMC)Co^{II}(OTf)]$B_{Ar}F$ with ozone led to qualitatively the same type of EPR spectrum, which is presented in Figure S3. However, the effective g values changed considerably: g'_{max} = 6.36, g'_{mid} = 2.69, and g'_{min} = 1.72 (again determined by simulation using S' = 1/2), with an additional feature at $g' \approx$ 3.87, analogous to that seen in [(TMC)Co^{II}(OTf)]$B_{Ar}F$, but also a weak derivative-shaped feature at $g \approx$ 2.04 (338 mT) that may be due to a radical species (S = 1/2) in low concentration. Notably, there appears to be a change in the ^{59}Co hyperfine coupling. There is still a resolved splitting at g'_{max}, but with

A'_{max}(^{59}Co) = 350 MHz, less than half of that in [(TMC)Co^{II}(OTf)]B$_{Ar}$F. At g'_{min}, the splitting is still unresolved, but appears larger than in [(TMC)Co^{II}(OTf)]B$_{Ar}$F. Qualitatively, the decrease in A'_{max}(^{59}Co) could be due to increasing spin delocalization from Co onto an axial oxido ligand.

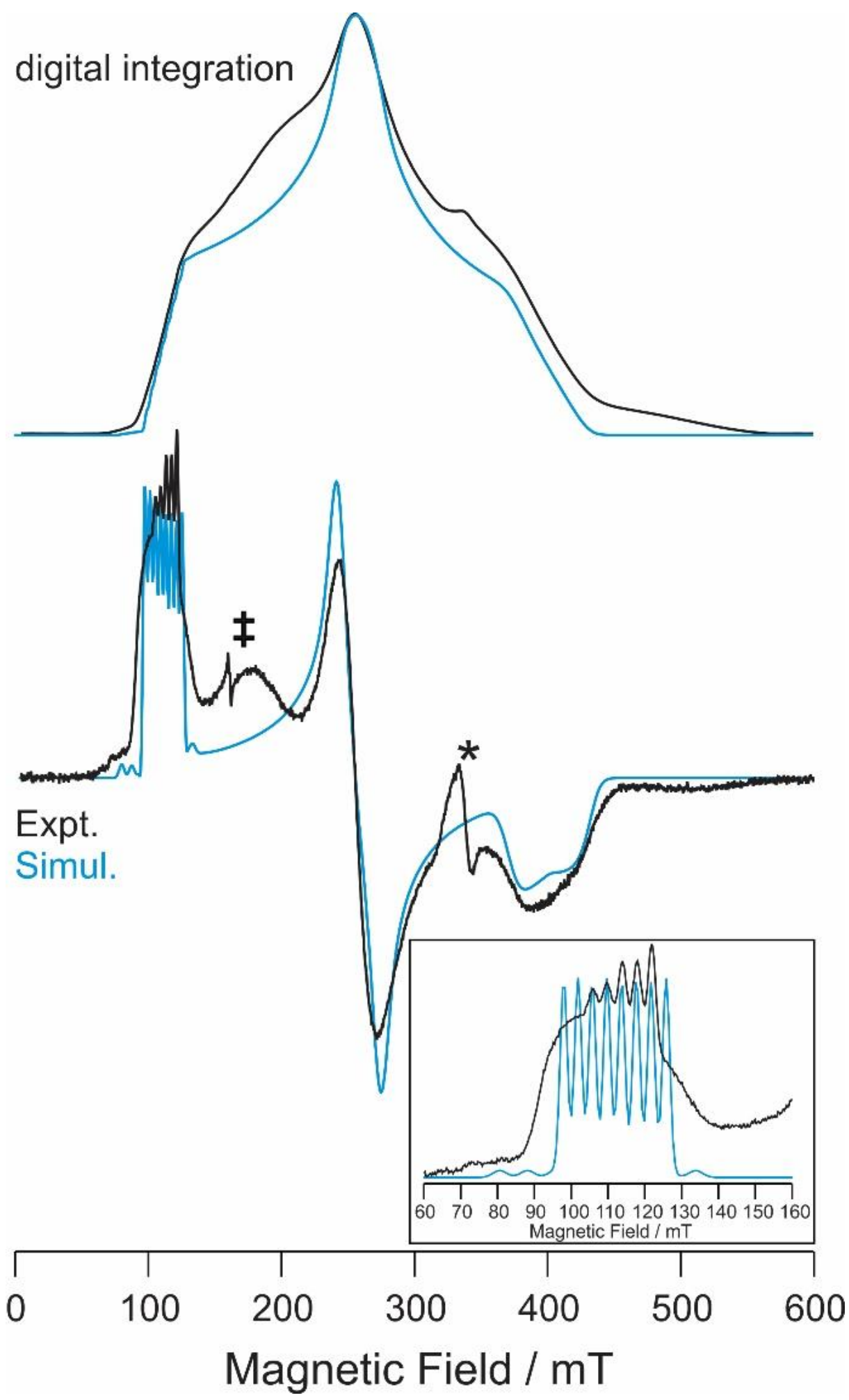

Figure S3: X-band EPR spectrum of [(TMC)Co^{II}(OTf)]B$_{Ar}$F after reaction with ozone (9.6404 GHz; 6.4 K; black trace) with simulation (blue trace) using S_{Co} = 3/2 spin Hamiltonian: D = –4.768 cm^{-1}, E = –1.589 cm^{-1}, $\boldsymbol{g}$ = [2.66, 2.35, 2.25], $\boldsymbol{A}$(^{59}Co) = [260, 150, 125] MHz, with Gaussian linewidths (hwhh) W_x = 200, W_y = 200, W_z = 100 MHz. The inset shows an expansion of the low field (g_z) region. The possible radical species (at 340 mT, $g \approx 2.0$) is indicated with an asterisk. The symbol ‡ indicates an additional species (at 150-200 mT; center is $g \approx 3.9$) of unknown provenance. Digital integrations of the experimental and simulated spectra are shown at top to provide another means of visualization.

Simulation of the spectrum was achieved with the following S_{Co} = 3/2 spin Hamiltonian parameters: D = –4.768 cm^{-1} (5.1 T in equivalent field units), E = –1.589 cm^{-1}, $\boldsymbol{g}$ = [2.66, 2.35, 2.25], $\boldsymbol{A}$(^{59}Co) = [260, 150, 125] MHz. That g_z is slightly closer to g_e (2.0023) in a possible Co-oxido complex is consistent with the decrease in A_z(^{59}Co), as both effects could arise from spin delocalization onto the oxido ligand. An oxyl radical would give g values close to g_e as with light atom radicals in general,[15] and as found for

the adsorbed O^- atomic radical, which has $\boldsymbol{g}$ = [2.036, 2.036, 1.994] (g_{avg} = 2.0022).[16] Similarly, adsorbed ozonide radical anion has $\boldsymbol{g}$ = [2.0060, 2.0130, 2.0104] (g_{avg} = 2.0098) and superoxide radical anion, the most anisotropic, has $\boldsymbol{g}$ = [2.0016, 2.0083, 2.0770] (g_{avg} = 2.029)[15] (also reported as $\boldsymbol{g}$ = [2.002, 2.008, 2.074] (g_{avg} = 2.028).[16]

Wang *et al*. have reported the EPR spectra of several cobalt complexes relevant to the present study. In particular, the X-band EPR spectra of [(13-TMC)Co^{II}(OTf)]OTf recorded in various solvents is qualitatively similar to that seen here for [(TMC)Co^{II}(OTf)]B_{Ar}F in terms of having a high g'_{max} (~6.5- 6.8; the spectrum is sensitive to solvent) with resolved $A(^{59}Co)$ and a low g'_{min} (~1.68).[17] Their putative Co(IV) complex, generated by oxidation of [(13-TMC)Co^{II}(OTf)]OTf by iodosylbenzene together with HOTf retained the S_{Co} = 3/2 ground state, with features at g' = 6.5 and 2.02, which was analyzed to arise from D = –1.1 ± 0.4 cm^{-1}, $|E/D|$ = 0.12, $\boldsymbol{g}$ = [2.68, 2.68, 2.085]. These spin Hamiltonian parameters derived from a single frequency measurement cannot be considered as definitive, but the key finding by Wang *et al*. is consistent with our EPR results: Namely, that a macrocyclic Co(IV)-oxido complex can exhibit an EPR spectrum that, in absence of other (i.e., chemical) information, could be assigned to high-spin Co(II), by virtue of having S_{Co} = 3/2 with moderate zfs (i.e., $|D|$ < 10 cm^{-1}). Other types of EPR spectra have been reported for Co(IV) complexes, including isotropic, i.e., more radical-like, spectra.[18–21]

References

[1] M. P. Hendrich, P. G. Debrunner, “Integer-spin electron paramagnetic resonance of iron proteins” *Biophys. J.* **1989**, *56*, 489–506.

[2] E. Münck, K. Ksurerus, M. P. Hendrich in *Metallobiochemistry Part D: Physical and Spectroscopic Methods for Probing Metal Ion Environment in Metalloproteins*, Academic Press, **1993**, pp. 463–479.

[3] J. T. Weisser, M. J. Nilges, M. J. Sever, J. J. Wilker, “EPR Investigation and Spectral Simulations of Iron−Catecholate Complexes and Iron−Peptide Models of Marine Adhesive Cross-Links” *Inorg. Chem.* **2006**, *45*, 7736–7747.

[4] F. Biaso, C. Duboc, B. Barbara, G. Serratrice, F. Thomas, D. Charapoff, C. Béguin, “High-Field EPR Study of Frozen Aqueous Solutions of Iron(III) Citrate Complexes” *Eur. J. Inorg. Chem.* **2005**, *2005*, 467–478.

[5] G. Mathies, H. Blok, J. A. J. M. Disselhorst, P. Gast, H. van der Meer, D. M. Miedema, R. M. Almeida, J. J. G. Moura, W. R. Hagen, E. J. J. Groenen, “Continuous-wave EPR at 275 GHz: Application to high-spin Fe^{3+} systems” *J. Magn. Reson.* **2011**, *210*, 126–132.

[6] R. Aasa, “Powder Line Shapes in the Electron Paramagnetic Resonance Spectra of High-Spin Ferric Complexes” *J. Chem. Phys.* **1970**, *52*, 3919–3930.

[7] M. J. Mombourquette, W. C. Tennant, J. A. Weil, “EPR study of Fe^{3+} in α-quartz: A reexamination of the so-called I center” *J. Chem. Phys.* **1986**, *85*, 68–79.

[8] D. Collison, A. K. Powell, “Electron spin resonance studies of ‘FeO_6’ tris chelate complexes: models for the effects of zero-field splitting in distorted S= 5/2 spin systems” *Inorg. Chem.* **1990**, *29*, 4735–4746.

[9] M. Azarkh, E. J. J. Groenen, “Simulation of multi-frequency EPR spectra for a distribution of the zero-field splitting” *J. Magn. Reson.* **2015**, *255*, 106–113.

[10] J. Telser in *eMagRes*, John Wiley & Sons, Ltd, **2017**, pp. 207–234.

[11] J. Krzystek, S. A. Zvyagin, A. Ozarowski, S. Trofimenko, J. Telser, “Tunable-frequency high-field electron paramagnetic resonance” *J. Magn. Reson.* **2006**, *178*, 174–183.

[12] J. Krzystek, A. Ozarowski, J. Telser, “Multi-frequency, high-field EPR as a powerful tool to accurately determine zero-field splitting in high-spin transition metal coordination complexes” *Coord. Chem. Rev.* **2006**, *250*, 2308–2324.

[13] D. Schweinfurth, M. G. Sommer, M. Atanasov, S. Demeshko, S. Hohloch, F. Meyer, F. Neese, B. Sarkar, “The Ligand Field of the Azido Ligand: Insights into Bonding Parameters and Magnetic Anisotropy in a Co(II)–Azido Complex” *J. Am. Chem. Soc.* **2015**, *137*, 1993–2005.

[14] A. N. Bone, C. N. Widener, D. H. Moseley, Z. Liu, Z. Lu, Y. Cheng, L. L. Daemen, M. Ozerov, J. Telser, K. Thirunavukkuarasu, D. Smirnov, S. M. Greer, S. Hill, J. Krzystek, K. Holldack, A.

Aliabadi, A. Schnegg, K. R. Dunbar, Z.-L. Xue, “Applying Unconventional Spectroscopies to the Single-Molecule Magnets, $Co(PPh_3)_2X_2$ (X=Cl, Br, I): Unveiling Magnetic Transitions and Spin-Phonon Coupling” *Chem. Eur. J.* **2021**, *27*, 11110–11125.

[15] M. Chiesa, E. Giamello, M. C. Paganini, Z. Sojka, D. M. Murphy, “Continuous wave electron paramagnetic resonance investigation of the hyperfine structure of $^{17}O_2^-$ adsorbed on the MgO surface” *J. Chem. Phys.* **2002**, *116*, 4266–4274.

[16] K. Hayashi, M. Hirano, S. Matsuishi, H. Hosono, “Microporous Crystal $12CaO\cdot7Al_2O_3$ Encaging Abundant O^- Radicals” *J. Am. Chem. Soc.* **2002**, *124*, 738–739.

[17] B. Wang, Y.-M. Lee, W.-Y. Tcho, S. Tussupbayev, S.-T. Kim, Y. Kim, M. S. Seo, K.-B. Cho, Y. Dede, B. C. Keegan, T. Ogura, S. H. Kim, T. Ohta, M.-H. Baik, K. Ray, J. Shearer, W. Nam, “Synthesis and reactivity of a mononuclear non-haem cobalt(IV)-oxo complex” *Nat. Commun.* **2017**, *8*, 14839.

[18] J. Topich, J. Halpern, “Organobis (dioximato) cobalt (IV) complexes: electron paramagnetic resonance spectra and electronic structures” *Inorg. Chem.* **1979**, *18*, 1339–1343.

[19] P. H. Rieger, “Electron paramagnetic resonance studies of low-spin d_5 transition metal complexes” *Coord. Chem. Rev.* **1994**, *135–136*, 203–286.

[20] S. Hong, F. F. Pfaff, E. Kwon, Y. Wang, M.-S. Seo, E. Bill, K. Ray, W. Nam, “Spectroscopic Capture and Reactivity of a Low-Spin Cobalt(IV)-Oxo Complex Stabilized by Binding Redox-Inactive Metal Ions” *Angew. Chem. Int. Ed.* **2014**, *53*, 10403–10407.

[21] S. Hong, F. F. Pfaff, E. Kwon, Y. Wang, M.-S. Seo, E. Bill, K. Ray, W. Nam, “Corrigendum: Spectroscopic Capture and Reactivity of a Low-Spin Cobalt(IV)-Oxo Complex Stabilized by Binding Redox-Inactive Metal Ions” *Angew. Chem. Int. Ed.* **2017**, *56*, 10630–10630.
