# Supplementary material for: First Evidence for Ozonido‐TMC Complexes of Iron and Cobalt
Source: Angew Chem Int Ed Engl. 2026 Jul 9;65(37):e4716492. doi: 10.1002/anie.4716492 (PMC13548893; doi:10.1002/anie.4716492)
Supplement: Supplementary file 6 [file ANIE-65-e4716492-s006.pdf]

Supporting Information Part II — X-ray diffraction

Table of Figures

Figure S1. Molecular structure of [(TMC)Fe^{II}(OTf)]$B_{Ar}F$ (**1a-$B_{Ar}F$**)

Figure S2. Molecular structure of [(D_{12}-TMC)Fe^{II}(OTf)]$B_{Ar}F$ (**1b-$B_{Ar}F$**)

Figure S3. Molecular structure of [(D_{12}-TMC)Fe^{IV}(O)(OTf)]OTf (**1b′-OTf**)

Figure S4. Molecular structure of [(D_{12}-TMC)Fe^{II}(I)]$B_{Ar}F$ (**2b-$B_{Ar}F$**)

Figure S5. Molecular structure of [(D_{12}-TMC)Fe^{II}(F)]$B_{Ar}F$ (**3b-$B_{Ar}F$**)

Figure S6. Molecular structure of [(TMC)Co^{II}(CH_3CN)]$(OTf)_2$ (**4a-$(OTf)_2$**)

Figure S7. Molecular structure of [(TMC)Co^{II}(MeCN)]$(B_{Ar}F)_2$ (**4a-$(B_{Ar}F)_2$**

Figure S8. Molecular structure of [(TMC)$Co^{II}$$(OTf)_2$] (**5a**)

Figure S9. Molecular structure of [(TMC)Co^{II}(OTf)]$B_{Ar}F$ (**5a-$B_{Ar}F$**)

Figure S10. Molecular structure of [(D_{12}-TMC)$Co^{II}$$(OTf)_2$] (**5b**)

Figure S11. Molecular structure of [(D_{12}-TMC)Co^{II}(OTf)]$B_{Ar}F$ (**5b-$B_{Ar}F$**)

Figure S12. Molecular structure of [(TMC)Co^{II}(ClO_4)]$B_{Ar}F$ (**6a-$B_{Ar}F$**)

Figure S13. Molecular structure of [(D_{12}-TMC)Co^{II}(F)]$B_{Ar}F$ (**7b-$B_{Ar}F$**)

Figure S14. Molecular structure of [(TMCO)Co^{III}(H_2O)]$(OTf)_2$ (**8a-$(OTf)_2$**)

Figure S15. Molecular structure of [(D_{12}-TMC-propyl-O)Co^{III}(OH)]$B_{Ar}F$ · 3 CH_2Cl_2 (**9b-$B_{Ar}F$**)

Figure S16. Molecular structure of [(D_{12}-TMC)Fe^{IV}(O)(H_2O)]{$N(OTf)_2$}$B_{Ar}F$

Figure S17. Molecular structure of [(TMC)Co^{II}(OTf)][Al{$OC(CF_3)_3$}$_4$]

Figure S18. Molecular structure of [(TMC)Co^{II}(Br)]Br

Figure S19. Molecular structure of [(TMC)Co^{II}(NO_3)]$B_{Ar}F$

Figure S20. Molecular structure of [(TMC)Co^{II}(I)]$B_{Ar}F$

Figure S21. Molecular structure of [(D_{12}-TMC)Co^{II}(I)]I

Figure S22. Molecular structure of [(D_{12}-TMC)Co^{II}(I)]$B_{Ar}F$

Figure S23. Molecular structure of [(D_{12}-TMC)Co^{II}(NO_3)]$B_{Ar}F$

Figure S24. Molecular structure of [(D_{12}-TMC)$Co^{II}$$(CH_3CN)_2$][$Co(NO_3)_4$]

Figure S25. Molecular structure of [(cleav.-D_{12}-TMC)$(Co^{II})_2$$(I)_4$]

Figure S26. Powder X-ray diffraction data for $Co(OTf)_2$ with calculated diffraction data from Rietveld refinement and difference plot

Figure S27. Excerpt of the crystal structure of $Co(OTf)_2$: Octahedral coordination of cobalt by triflate anions

Single crystal X-ray diffraction

Diffraction data were collected at low temperatures (100K) using φ- and ω-scans on a BRUKER D8 Venture system equipped with dual IμS microfocus sources, a PHOTON100 detector and an OXFORD CRYOSYSTEMS 700 low temperature system. Mo-Kα radiation with wavelength 0.71073 Å and a collimating Quazar multilayer mirror were used. Semi-empirical absorption correction from equivalents was applied using SADABS-2016/2[1] and the structures were solved by the dual space algorithm implemented in SHELXT2014/5.[2] Refinement was performed against F^2 on all data by full-matrix least squares using SHELXL2018/3.[3] All non-hydrogen atoms were refined anisotropically and C-H hydrogen atoms were positioned at geometrically calculated positions and refined using a riding model. O-H hydrogen atoms were located in the difference map and were set to ideal distances. The isotropic displacement parameters of all hydrogen atoms were fixed to 1.2x or 1.5x (CH_3 and OH hydrogens) the U_{eq} value of the atoms they are linked to.

The crystallographic data have been deposited with the Cambridge Crystallographic Data Centre and the Leibniz-Institut für Informationsinfrastruktur as CCDC No. 2062705-2062715, 2062718-2062724, 2062764-2062766, 2062767, 2122182, 2122183 2502781 and 2502782 can be obtained free of charge.[4]

[(TMC)Fe^{II}(OTf)]B_{ArF}, **1a-B_{ArF}**

The structure of **1a-B_{ArF}** was solved in the monoclinic space group $P2_1/c$ and the asymmetric unit contains one full formula unit. The ligand was found to be disordered over two positions and five of the CF_3 groups of the anion were also found to be disordered. Disorder was refined with the help of 1,2 and 1,3 same distance restraints, similarity restraints on anisotropic displacement parameters,[5] advanced rigid bond restraints[6] and setting some atoms close to each other to have the same anisotropic displacement parameters. The disorder ratios were refined and converged to 0.945(2) (ligand), 0.426(2):0.320(2):0.253(2), 0.70(2), 0.947(3), 0.863(4) and 0.899(3) (CF_3 groups).

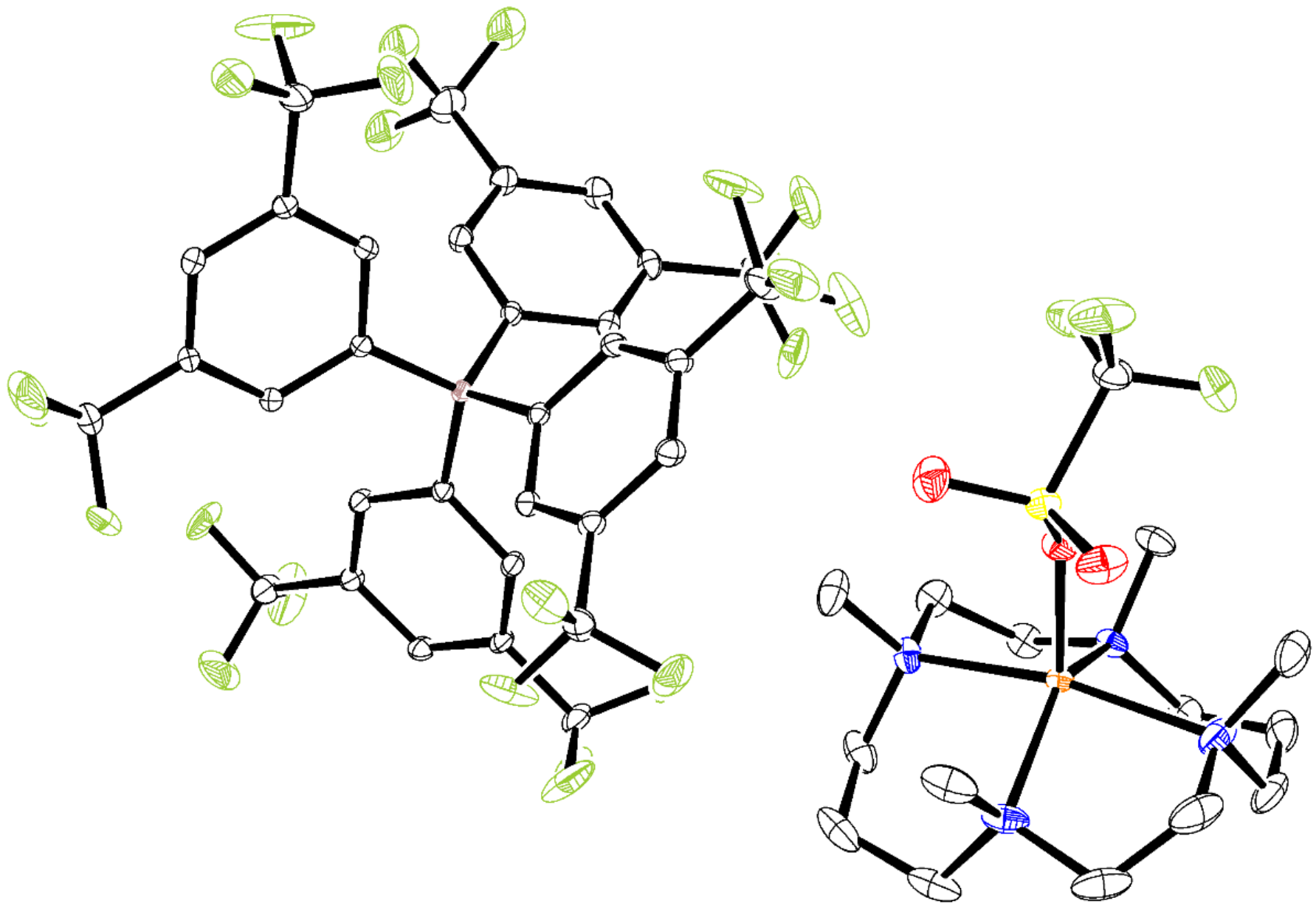

Figure S1. Molecular structure of (**1a-B_{ArF}**).

Table S1. Crystal data and structure refinement for **1a-B_{ArF}**.

CCDC No	2062764	
Empirical formula	$C_{47}H_{44}BF_{27}FeN_4O_3S$	
Formula weight	1324.58	
Temperature	100(2) K	
Wavelength	0.71073 Å	
Crystal system	Monoclinic	
Space group	$P2_1/c$	
Unit cell dimensions	a = 12.2455(6) Å	α = 90°
	b = 25.1684(13) Å	β = 96.7707(18)°
	c = 17.4272(9) Å	γ = 90°
Volume	5333.6(5) Å^3	
Z	4	
Density (calculated)	1.650 Mg/m^3	
Absorption coefficient	0.462 mm^{-1}	
F(000)	2672	
Crystal size	0.246 x 0.203 x 0.097 mm^3	
Theta range for data collection	2.304 to 32.576°.	
Index ranges	-18 ≤ h ≤ 18, -38 ≤ k ≤ 38, -26 ≤ l ≤ 26	
Reflections collected	1051377	
Independent reflections	19427 [R(int) = 0.0768]	
Completeness to theta = 25.242°	99.9 %	
Absorption correction	Semi-empirical from equivalents	
Refinement method	Full-matrix least-squares on F^2	
Data / restraints / parameters	19427 / 3996 / 1009	
Goodness-of-fit on F^2	0.899	
Final R indices [I>2sigma(I)]	R1 = 0.0557, wR2 = 0.1368	
R indices (all data)	R1 = 0.0677, wR2 = 0.1456	
Largest diff. peak and hole	1.960 and -1.825 eÅ^{-3}	

Table S2. Atomic coordinates (x 10^4) and equivalent isotropic displacement parameters ($Å^2$ x 10^3) for **1a-B$_{ArF}$**. U(eq) is defined as one third of the trace of the orthogonalized U^{ij} tensor.

	x	y	z	U(eq)
Fe(1)	287(1)	1813(1)	2589(1)	11(1)
N(1)	1122(2)	1261(1)	1909(1)	23(1)
N(2)	1844(1)	2255(1)	2976(1)	17(1)
N(3)	52(1)	1914(1)	3787(1)	16(1)
N(4)	-1077(2)	1231(1)	2379(1)	24(1)
C(1)	2240(2)	1098(1)	2257(2)	30(1)
C(2)	3046(2)	1549(1)	2449(1)	31(1)
C(3)	2812(2)	1894(1)	3128(1)	24(1)
C(4)	2100(2)	2687(1)	2448(1)	27(1)
C(5)	1636(2)	2509(1)	3711(1)	22(1)
C(6)	1084(2)	2125(1)	4211(1)	20(1)
C(7)	-826(2)	2320(1)	3812(1)	22(1)
C(8)	-253(2)	1425(1)	4194(1)	22(1)
C(9)	-1254(2)	1134(1)	3811(2)	30(1)
C(10)	-1118(2)	867(1)	3048(2)	30(1)
C(11)	-2192(2)	1461(1)	2171(2)	32(1)
C(12)	-789(3)	919(1)	1709(2)	35(1)
C(13)	422(3)	777(1)	1788(2)	36(1)
C(14)	1193(2)	1518(1)	1144(1)	30(1)
N(1A)	-530(20)	1079(9)	2279(14)	31(3)
N(2A)	1858(19)	1514(9)	2109(13)	27(3)
N(3A)	1420(20)	2432(9)	3018(13)	28(3)
N(4A)	-455(18)	1748(8)	3656(13)	25(3)
C(1A)	140(20)	577(12)	2420(20)	33(4)
C(2A)	1170(20)	565(12)	2020(20)	34(4)
C(3A)	2070(30)	952(11)	2320(30)	31(4)
C(4A)	1650(40)	1615(18)	1282(15)	31(7)
C(5A)	2770(20)	1842(11)	2480(20)	30(4)
C(6A)	2400(20)	2406(11)	2583(19)	30(4)
C(7A)	970(30)	2988(12)	2870(20)	31(6)
C(8A)	1880(30)	2438(18)	3858(15)	28(4)
C(9A)	1000(30)	2352(13)	4393(17)	29(4)
C(10A)	420(20)	1824(11)	4293(17)	27(4)
C(11A)	-1300(30)	2163(13)	3630(20)	22(1)
C(12A)	-860(30)	1198(10)	3642(16)	30(1)
C(13A)	-1380(20)	1068(14)	2823(16)	30(4)
C(14A)	-1150(40)	1039(19)	1483(17)	42(7)
S(1)	-787(1)	2599(1)	1227(1)	16(1)
F(1)	-2258(1)	3284(1)	682(1)	42(1)
F(2)	-1596(1)	3429(1)	1864(1)	34(1)
F(3)	-2748(1)	2793(1)	1596(1)	44(1)
O(1)	-491(1)	2420(1)	2035(1)	19(1)
O(2)	-1261(1)	2190(1)	723(1)	28(1)
O(3)	56(1)	2917(1)	955(1)	27(1)
C(15)	-1918(2)	3054(1)	1353(1)	24(1)
F(21)	6102(2)	2381(1)	3340(1)	46(1)
F(22)	5019(1)	2699(1)	4101(1)	33(1)
F(23)	6665(1)	2488(1)	4541(1)	38(1)
F(24)	9200(6)	4574(2)	3791(4)	32(1)
F(25)	9692(4)	3760(2)	3763(6)	44(1)
F(26)	9235(5)	4094(3)	4802(2)	46(1)
F(24A)	9314(15)	4478(8)	3611(13)	54(4)
F(25A)	9690(9)	3716(4)	4074(12)	39(3)
F(26A)	9055(10)	4286(9)	4767(8)	55(3)
F(31)	8274(1)	4450(1)	778(1)	30(1)
F(32)	8764(1)	5241(1)	1128(1)	49(1)
F(33)	7987(1)	5096(1)	-15(1)	44(1)
F(31A)	8530(20)	4580(10)	1129(15)	37(6)
F(32A)	8543(18)	5367(7)	576(16)	44(1)
F(33A)	7711(19)	4749(11)	-1(9)	44(1)
F(34)	4599(2)	6079(1)	-122(1)	28(1)
F(35)	3411(1)	5835(1)	593(1)	48(1)
F(36)	4605(2)	6430(1)	994(1)	51(1)
F(34A)	4082(16)	5983(7)	-45(7)	45(4)
F(35A)	3812(10)	6169(5)	1134(6)	28(1)
F(36A)	5180(10)	6466(4)	659(8)	32(3)
F(41)	4916(2)	2805(2)	841(2)	33(1)
F(42)	3224(2)	2539(1)	776(1)	36(1)
F(43)	3710(2)	3069(1)	-70(1)	26(1)
F(41A)	3786(11)	2499(3)	1189(7)	36(1)
F(42A)	3409(9)	2840(5)	80(7)	36(3)
F(43A)	4996(10)	2916(9)	727(16)	23(3)
F(44)	543(1)	3982(1)	2006(1)	49(1)
F(45)	482(1)	4084(1)	788(1)	34(1)
F(46)	982(1)	4731(1)	1535(1)	58(1)
F(51)	2363(1)	4120(1)	4555(1)	36(1)
F(52)	1518(1)	4859(1)	4482(1)	55(1)
F(53)	2686(1)	4690(1)	5463(1)	36(1)
F(54)	5791(3)	6268(1)	4511(3)	37(1)
F(55)	3929(4)	6481(2)	4431(3)	37(1)
F(56)	4721(4)	6495(1)	3500(2)	37(1)
F(54A)	5505(4)	6215(2)	4908(3)	37(1)
F(55A)	4167(5)	6501(2)	4583(4)	37(1)
F(56A)	5212(5)	6440(2)	3683(3)	37(1)
F(54B)	5942(5)	6271(2)	4097(4)	37(1)
F(55B)	4959(6)	6294(2)	5001(3)	37(1)
F(56B)	4301(5)	6559(2)	3881(4)	37(1)
C(21)	6004(1)	4092(1)	3074(1)	11(1)
C(22)	5709(1)	3584(1)	3299(1)	13(1)
C(23)	6429(1)	3258(1)	3763(1)	13(1)

C(24)	7489(1)	3423(1)	4020(1)	14(1)
C(25)	7811(1)	3924(1)	3792(1)	12(1)
C(26)	7084(1)	4254(1)	3338(1)	12(1)
C(27)	6059(2)	2709(1)	3945(1)	19(1)
C(28)	8970(1)	4095(1)	4040(1)	19(1)
C(31)	5597(1)	4859(1)	1947(1)	10(1)
C(32)	6577(1)	4760(1)	1630(1)	11(1)
C(33)	6877(1)	5055(1)	1009(1)	12(1)
C(34)	6212(1)	5456(1)	667(1)	14(1)
C(35)	5230(1)	5556(1)	967(1)	14(1)
C(36)	4938(1)	5270(1)	1595(1)	13(1)
C(37)	7964(2)	4954(1)	723(1)	21(1)
C(38)	4477(2)	5983(1)	620(1)	23(1)
C(41)	4137(1)	4143(1)	2078(1)	10(1)
C(42)	4395(1)	3700(1)	1643(1)	12(1)
C(43)	3595(1)	3426(1)	1165(1)	12(1)
C(44)	2497(1)	3586(1)	1094(1)	14(1)
C(45)	2232(1)	4027(1)	1506(1)	14(1)
C(46)	3035(1)	4299(1)	1988(1)	12(1)
C(47)	3888(2)	2957(1)	703(1)	19(1)
C(48)	1061(2)	4205(1)	1460(1)	23(1)
C(51)	4586(1)	4837(1)	3258(1)	10(1)
C(52)	3824(1)	4607(1)	3704(1)	13(1)
C(53)	3381(1)	4884(1)	4283(1)	15(1)
C(54)	3699(2)	5404(1)	4464(1)	18(1)
C(55)	4463(1)	5639(1)	4043(1)	16(1)
C(56)	4891(1)	5360(1)	3449(1)	14(1)
C(57)	2498(2)	4638(1)	4694(1)	21(1)
C(58)	4817(2)	6199(1)	4216(1)	29(1)
B(21)	5104(1)	4486(1)	2596(1)	10(1)

Table S3. Bond lengths [Å] and angles [°] for **1a-B_{ArF}**.

Fe(1)-O(1)	1.9890(13)	C(9A)-C(10A)	1.508(19)
Fe(1)-N(1A)	2.14(2)	C(9A)-H(9AA)	0.9900
Fe(1)-N(3)	2.1560(15)	C(9A)-H(9AC)	0.9900
Fe(1)-N(3A)	2.16(2)	C(10A)-H(10C)	0.9900
Fe(1)-N(1)	2.1600(17)	C(10A)-H(10D)	0.9900
Fe(1)-N(4A)	2.17(2)	C(11A)-H(11D)	0.9800
Fe(1)-N(4)	2.2191(18)	C(11A)-H(11E)	0.9800
Fe(1)-N(2)	2.2417(16)	C(11A)-H(11F)	0.9800
Fe(1)-N(2A)	2.31(2)	C(12A)-C(13A)	1.527(19)
N(1)-C(1)	1.488(3)	C(12A)-H(12C)	0.9900
N(1)-C(13)	1.491(3)	C(12A)-H(12D)	0.9900
N(1)-C(14)	1.493(3)	C(13A)-H(13C)	0.9900
N(2)-C(5)	1.480(3)	C(13A)-H(13D)	0.9900
N(2)-C(4)	1.481(3)	C(14A)-H(14D)	0.9800
N(2)-C(3)	1.491(3)	C(14A)-H(14E)	0.9800
N(3)-C(6)	1.485(3)	C(14A)-H(14F)	0.9800
N(3)-C(7)	1.488(2)	S(1)-O(3)	1.4295(15)
N(3)-C(8)	1.489(3)	S(1)-O(2)	1.4322(15)
N(4)-C(12)	1.484(3)	S(1)-O(1)	1.4821(13)
N(4)-C(10)	1.488(3)	S(1)-C(15)	1.830(2)
N(4)-C(11)	1.489(3)	F(1)-C(15)	1.327(2)
C(1)-C(2)	1.517(4)	F(2)-C(15)	1.324(3)
C(1)-H(1A)	0.9900	F(3)-C(15)	1.321(3)
C(1)-H(1AB)	0.9900	F(21)-C(27)	1.345(2)
C(2)-C(3)	1.522(3)	F(22)-C(27)	1.334(2)
C(2)-H(2A)	0.9900	F(23)-C(27)	1.327(2)
C(2)-H(2AB)	0.9900	F(24)-C(28)	1.323(4)
C(3)-H(3A)	0.9900	F(25)-C(28)	1.352(4)
C(3)-H(3AB)	0.9900	F(26)-C(28)	1.331(4)
C(4)-H(4A)	0.9800	F(24A)-C(28)	1.318(10)
C(4)-H(4B)	0.9800	F(25A)-C(28)	1.295(8)
C(4)-H(4C)	0.9800	F(26A)-C(28)	1.349(9)
C(5)-C(6)	1.514(3)	F(31)-C(37)	1.325(2)
C(5)-H(5A)	0.9900	F(32)-C(37)	1.348(3)
C(5)-H(5AB)	0.9900	F(33)-C(37)	1.338(2)
C(6)-H(6A)	0.9900	F(31A)-C(37)	1.325(12)
C(6)-H(6AB)	0.9900	F(32A)-C(37)	1.300(12)
C(7)-H(7A)	0.9800	F(33A)-C(37)	1.365(12)
C(7)-H(7B)	0.9800	F(34)-C(38)	1.342(2)
C(7)-H(7C)	0.9800	F(35)-C(38)	1.353(3)
C(8)-C(9)	1.515(3)	F(36)-C(38)	1.302(3)
C(8)-H(8A)	0.9900	F(34A)-C(38)	1.201(10)
C(8)-H(8AB)	0.9900	F(35A)-C(38)	1.363(9)
C(9)-C(10)	1.516(4)	F(36A)-C(38)	1.487(9)
C(9)-H(9A)	0.9900	F(41)-C(47)	1.311(3)
C(9)-H(9AB)	0.9900	F(42)-C(47)	1.344(2)
C(10)-H(10A)	0.9900	F(43)-C(47)	1.368(2)
C(10)-H(10B)	0.9900	F(41A)-C(47)	1.444(9)
C(11)-H(11A)	0.9800	F(42A)-C(47)	1.208(8)
C(11)-H(11B)	0.9800	F(43A)-C(47)	1.357(11)
C(11)-H(11C)	0.9800	F(44)-C(48)	1.328(3)
C(12)-C(13)	1.515(4)	F(45)-C(48)	1.332(2)
C(12)-H(12A)	0.9900	F(46)-C(48)	1.333(2)
C(12)-H(12B)	0.9900	F(51)-C(57)	1.332(2)
C(13)-H(13A)	0.9900	F(52)-C(57)	1.336(2)
C(13)-H(13B)	0.9900	F(53)-C(57)	1.340(2)
C(14)-H(14A)	0.9800	F(54)-C(58)	1.255(4)
C(14)-H(14B)	0.9800	F(55)-C(58)	1.387(5)
C(14)-H(14C)	0.9800	F(56)-C(58)	1.446(5)
N(1A)-C(13A)	1.490(18)	F(54A)-C(58)	1.389(5)
N(1A)-C(1A)	1.505(19)	F(55A)-C(58)	1.320(6)
N(1A)-C(14A)	1.506(19)	F(56A)-C(58)	1.254(5)
N(2A)-C(4A)	1.456(18)	F(54B)-C(58)	1.429(6)
N(2A)-C(5A)	1.475(18)	F(55B)-C(58)	1.380(6)
N(2A)-C(3A)	1.478(19)	F(56B)-C(58)	1.214(6)
N(3A)-C(6A)	1.491(18)	C(21)-C(22)	1.398(2)
N(3A)-C(8A)	1.506(19)	C(21)-C(26)	1.408(2)
N(3A)-C(7A)	1.517(18)	C(21)-B(21)	1.635(2)
N(4A)-C(10A)	1.465(18)	C(22)-C(23)	1.391(2)
N(4A)-C(11A)	1.468(18)	C(22)-H(22)	0.9500
N(4A)-C(12A)	1.471(18)	C(23)-C(24)	1.386(2)
C(1A)-C(2A)	1.519(19)	C(23)-C(27)	1.499(2)
C(1A)-H(1AA)	0.9900	C(24)-C(25)	1.393(2)
C(1A)-H(1AC)	0.9900	C(24)-H(24)	0.9500
C(2A)-C(3A)	1.515(19)	C(25)-C(26)	1.393(2)
C(2A)-H(2AA)	0.9900	C(25)-C(28)	1.497(2)
C(2A)-H(2AC)	0.9900	C(26)-H(26)	0.9500
C(3A)-H(3AA)	0.9900	C(31)-C(32)	1.402(2)
C(3A)-H(3AC)	0.9900	C(31)-C(36)	1.407(2)
C(4A)-H(4AA)	0.9800	C(31)-B(21)	1.638(2)
C(4A)-H(4AB)	0.9800	C(32)-C(33)	1.397(2)
C(4A)-H(4AC)	0.9800	C(32)-H(32)	0.9500
C(5A)-C(6A)	1.507(19)	C(33)-C(34)	1.387(2)
C(5A)-H(5AA)	0.9900	C(33)-C(37)	1.496(2)
C(5A)-H(5AC)	0.9900	C(34)-C(35)	1.389(2)
C(6A)-H(6AA)	0.9900	C(34)-H(34)	0.9500
C(6A)-H(6AC)	0.9900	C(35)-C(36)	1.392(2)
C(7A)-H(7AA)	0.9800	C(35)-C(38)	1.495(2)
C(7A)-H(7AB)	0.9800	C(36)-H(36)	0.9500
C(7A)-H(7AC)	0.9800	C(41)-C(46)	1.397(2)
C(8A)-C(9A)	1.516(19)	C(41)-C(42)	1.406(2)
C(8A)-H(8AA)	0.9900	C(41)-B(21)	1.646(2)
C(8A)-H(8AC)	0.9900	C(42)-C(43)	1.390(2)

C(42)-H(42)	0.9500
C(43)-C(44)	1.394(2)
C(43)-C(47)	1.498(2)
C(44)-C(45)	1.383(2)
C(44)-H(44)	0.9500
C(45)-C(46)	1.395(2)
C(45)-C(48)	1.495(2)
C(46)-H(46)	0.9500
C(51)-C(56)	1.398(2)
C(51)-C(52)	1.407(2)
C(51)-B(21)	1.639(2)
C(52)-C(53)	1.389(2)
C(52)-H(52)	0.9500
C(53)-C(54)	1.392(2)
C(53)-C(57)	1.499(2)
C(54)-C(55)	1.387(2)
C(54)-H(54)	0.9500
C(55)-C(56)	1.401(2)
C(55)-C(58)	1.495(3)
C(56)-H(56)	0.9500
O(1)-Fe(1)-N(1A)	111.1(7)
O(1)-Fe(1)-N(3)	105.59(6)
O(1)-Fe(1)-N(3A)	82.3(7)
N(1A)-Fe(1)-N(3A)	166.3(10)
O(1)-Fe(1)-N(1)	117.28(6)
N(3)-Fe(1)-N(1)	137.12(6)
O(1)-Fe(1)-N(4A)	104.4(6)
N(1A)-Fe(1)-N(4A)	85.5(8)
N(3A)-Fe(1)-N(4A)	94.3(8)
O(1)-Fe(1)-N(4)	96.84(6)
N(3)-Fe(1)-N(4)	93.07(7)
N(1)-Fe(1)-N(4)	83.15(8)
O(1)-Fe(1)-N(2)	96.13(6)
N(3)-Fe(1)-N(2)	81.75(6)
N(1)-Fe(1)-N(2)	92.45(7)
N(4)-Fe(1)-N(2)	166.91(6)
O(1)-Fe(1)-N(2A)	116.5(6)
N(1A)-Fe(1)-N(2A)	90.6(8)
N(3A)-Fe(1)-N(2A)	80.3(8)
N(4A)-Fe(1)-N(2A)	137.4(8)
C(1)-N(1)-C(13)	108.54(19)
C(1)-N(1)-C(14)	109.59(19)
C(13)-N(1)-C(14)	108.73(19)
C(1)-N(1)-Fe(1)	115.26(14)
C(13)-N(1)-Fe(1)	107.56(15)
C(14)-N(1)-Fe(1)	106.98(13)
C(5)-N(2)-C(4)	106.95(18)
C(5)-N(2)-C(3)	108.82(16)
C(4)-N(2)-C(3)	109.54(17)
C(5)-N(2)-Fe(1)	104.31(12)
C(4)-N(2)-Fe(1)	114.36(12)
C(3)-N(2)-Fe(1)	112.46(13)
C(6)-N(3)-C(7)	108.40(16)
C(6)-N(3)-C(8)	107.72(15)
C(7)-N(3)-C(8)	109.38(15)
C(6)-N(3)-Fe(1)	108.74(11)
C(7)-N(3)-Fe(1)	106.77(11)
C(8)-N(3)-Fe(1)	115.65(13)
C(12)-N(4)-C(10)	108.85(19)
C(12)-N(4)-C(11)	108.10(19)
C(10)-N(4)-C(11)	108.38(19)
C(12)-N(4)-Fe(1)	103.61(15)
C(10)-N(4)-Fe(1)	111.87(13)
C(11)-N(4)-Fe(1)	115.73(13)
N(1)-C(1)-C(2)	115.3(2)
N(1)-C(1)-H(1A)	108.5
C(2)-C(1)-H(1A)	108.5
N(1)-C(1)-H(1AB)	108.5
C(2)-C(1)-H(1AB)	108.5
H(1A)-C(1)-H(1AB)	107.5
C(1)-C(2)-C(3)	114.97(19)
C(1)-C(2)-H(2A)	108.5
C(3)-C(2)-H(2A)	108.5
C(1)-C(2)-H(2AB)	108.5
C(3)-C(2)-H(2AB)	108.5
H(2A)-C(2)-H(2AB)	107.5
N(2)-C(3)-C(2)	115.33(17)
N(2)-C(3)-H(3A)	108.4
C(2)-C(3)-H(3A)	108.4
N(2)-C(3)-H(3AB)	108.4
C(2)-C(3)-H(3AB)	108.4
H(3A)-C(3)-H(3AB)	107.5
N(2)-C(4)-H(4A)	109.5
N(2)-C(4)-H(4B)	109.5
H(4A)-C(4)-H(4B)	109.5
N(2)-C(4)-H(4C)	109.5
H(4A)-C(4)-H(4C)	109.5
H(4B)-C(4)-H(4C)	109.5
N(2)-C(5)-C(6)	110.94(18)
N(2)-C(5)-H(5A)	109.5
C(6)-C(5)-H(5A)	109.5
N(2)-C(5)-H(5AB)	109.5
C(6)-C(5)-H(5AB)	109.5
H(5A)-C(5)-H(5AB)	108.0
N(3)-C(6)-C(5)	110.66(16)
N(3)-C(6)-H(6A)	109.5
C(5)-C(6)-H(6A)	109.5
N(3)-C(6)-H(6AB)	109.5
C(5)-C(6)-H(6AB)	109.5
H(6A)-C(6)-H(6AB)	108.1
N(3)-C(7)-H(7A)	109.5
N(3)-C(7)-H(7B)	109.5
H(7A)-C(7)-H(7B)	109.5
N(3)-C(7)-H(7C)	109.5
H(7A)-C(7)-H(7C)	109.5
H(7B)-C(7)-H(7C)	109.5
N(3)-C(8)-C(9)	115.21(17)
N(3)-C(8)-H(8A)	108.5
C(9)-C(8)-H(8A)	108.5
N(3)-C(8)-H(8AB)	108.5
C(9)-C(8)-H(8AB)	108.5
H(8A)-C(8)-H(8AB)	107.5
C(8)-C(9)-C(10)	115.74(19)
C(8)-C(9)-H(9A)	108.3
C(10)-C(9)-H(9A)	108.3
C(8)-C(9)-H(9AB)	108.3
C(10)-C(9)-H(9AB)	108.3
H(9A)-C(9)-H(9AB)	107.4
N(4)-C(10)-C(9)	115.54(19)
N(4)-C(10)-H(10A)	108.4
C(9)-C(10)-H(10A)	108.4
N(4)-C(10)-H(10B)	108.4
C(9)-C(10)-H(10B)	108.4
H(10A)-C(10)-H(10B)	107.5
N(4)-C(11)-H(11A)	109.5
N(4)-C(11)-H(11B)	109.5
H(11A)-C(11)-H(11B)	109.5
N(4)-C(11)-H(11C)	109.5
H(11A)-C(11)-H(11C)	109.5
H(11B)-C(11)-H(11C)	109.5
N(4)-C(12)-C(13)	112.0(2)
N(4)-C(12)-H(12A)	109.2
C(13)-C(12)-H(12A)	109.2
N(4)-C(12)-H(12B)	109.2
C(13)-C(12)-H(12B)	109.2
H(12A)-C(12)-H(12B)	107.9
N(1)-C(13)-C(12)	111.12(18)
N(1)-C(13)-H(13A)	109.4
C(12)-C(13)-H(13A)	109.4
N(1)-C(13)-H(13B)	109.4
C(12)-C(13)-H(13B)	109.4
H(13A)-C(13)-H(13B)	108.0
N(1)-C(14)-H(14A)	109.5
N(1)-C(14)-H(14B)	109.5
H(14A)-C(14)-H(14B)	109.5
N(1)-C(14)-H(14C)	109.5
H(14A)-C(14)-H(14C)	109.5
H(14B)-C(14)-H(14C)	109.5
C(13A)-N(1A)-C(1A)	106(2)
C(13A)-N(1A)-C(14A)	106(2)
C(1A)-N(1A)-C(14A)	107(2)
C(13A)-N(1A)-Fe(1)	101.3(15)
C(1A)-N(1A)-Fe(1)	117.4(18)
C(14A)-N(1A)-Fe(1)	117(2)
C(4A)-N(2A)-C(5A)	112(2)
C(4A)-N(2A)-C(3A)	115(2)
C(5A)-N(2A)-C(3A)	109.0(19)
C(4A)-N(2A)-Fe(1)	104(2)
C(5A)-N(2A)-Fe(1)	105.9(15)
C(3A)-N(2A)-Fe(1)	110(2)
C(6A)-N(3A)-C(8A)	105(2)
C(6A)-N(3A)-C(7A)	104.7(19)
C(8A)-N(3A)-C(7A)	104(2)
C(6A)-N(3A)-Fe(1)	108.3(15)
C(8A)-N(3A)-Fe(1)	119.6(19)
C(7A)-N(3A)-Fe(1)	114(2)
C(10A)-N(4A)-C(11A)	113(2)
C(10A)-N(4A)-C(12A)	110.6(18)
C(11A)-N(4A)-C(12A)	116(2)
C(10A)-N(4A)-Fe(1)	107.2(17)
C(11A)-N(4A)-Fe(1)	106.7(18)
C(12A)-N(4A)-Fe(1)	103.4(16)
N(1A)-C(1A)-C(2A)	114(2)
N(1A)-C(1A)-H(1AA)	108.8
C(2A)-C(1A)-H(1AA)	108.8
N(1A)-C(1A)-H(1AC)	108.8
C(2A)-C(1A)-H(1AC)	108.8
H(1AA)-C(1A)-H(1AC)	107.7
C(3A)-C(2A)-C(1A)	116(2)
C(3A)-C(2A)-H(2AA)	108.3
C(1A)-C(2A)-H(2AA)	108.3
C(3A)-C(2A)-H(2AC)	108.3
C(1A)-C(2A)-H(2AC)	108.3
H(2AA)-C(2A)-H(2AC)	107.4
N(2A)-C(3A)-C(2A)	116(2)
N(2A)-C(3A)-H(3AA)	108.4
C(2A)-C(3A)-H(3AA)	108.4
N(2A)-C(3A)-H(3AC)	108.4
C(2A)-C(3A)-H(3AC)	108.4

H(3AA)-C(3A)-H(3AC)	107.4	C(25)-C(26)-C(21)	121.73(14)
N(2A)-C(4A)-H(4AA)	109.5	C(25)-C(26)-H(26)	119.1
N(2A)-C(4A)-H(4AB)	109.5	C(21)-C(26)-H(26)	119.1
H(4AA)-C(4A)-H(4AB)	109.5	F(23)-C(27)-F(22)	106.93(16)
N(2A)-C(4A)-H(4AC)	109.5	F(23)-C(27)-F(21)	106.63(17)
H(4AA)-C(4A)-H(4AC)	109.5	F(22)-C(27)-F(21)	105.88(17)
H(4AB)-C(4A)-H(4AC)	109.5	F(23)-C(27)-C(23)	113.48(15)
N(2A)-C(5A)-C(6A)	111(2)	F(22)-C(27)-C(23)	112.41(15)
N(2A)-C(5A)-H(5AA)	109.5	F(21)-C(27)-C(23)	111.03(15)
C(6A)-C(5A)-H(5AA)	109.5	F(25A)-C(28)-F(24A)	107.6(9)
N(2A)-C(5A)-H(5AC)	109.5	F(24)-C(28)-F(26)	107.0(3)
C(6A)-C(5A)-H(5AC)	109.5	F(25A)-C(28)-F(26A)	104.0(6)
H(5AA)-C(5A)-H(5AC)	108.1	F(24A)-C(28)-F(26A)	106.1(8)
N(3A)-C(6A)-C(5A)	112(2)	F(24)-C(28)-F(25)	106.1(4)
N(3A)-C(6A)-H(6AA)	109.3	F(26)-C(28)-F(25)	105.2(3)
C(5A)-C(6A)-H(6AA)	109.3	F(25A)-C(28)-C(25)	114.8(6)
N(3A)-C(6A)-H(6AC)	109.3	F(24A)-C(28)-C(25)	113.7(8)
C(5A)-C(6A)-H(6AC)	109.3	F(24)-C(28)-C(25)	113.5(3)
H(6AA)-C(6A)-H(6AC)	107.9	F(26)-C(28)-C(25)	113.5(3)
N(3A)-C(7A)-H(7AA)	109.5	F(26A)-C(28)-C(25)	109.8(5)
N(3A)-C(7A)-H(7AB)	109.5	F(25)-C(28)-C(25)	110.9(3)
H(7AA)-C(7A)-H(7AB)	109.5	C(32)-C(31)-C(36)	115.57(14)
N(3A)-C(7A)-H(7AC)	109.5	C(32)-C(31)-B(21)	124.94(13)
H(7AA)-C(7A)-H(7AC)	109.5	C(36)-C(31)-B(21)	118.90(13)
H(7AB)-C(7A)-H(7AC)	109.5	C(33)-C(32)-C(31)	121.81(14)
N(3A)-C(8A)-C(9A)	113(2)	C(33)-C(32)-H(32)	119.1
N(3A)-C(8A)-H(8AA)	109.0	C(31)-C(32)-H(32)	119.1
C(9A)-C(8A)-H(8AA)	109.0	C(34)-C(33)-C(32)	121.63(14)
N(3A)-C(8A)-H(8AC)	109.0	C(34)-C(33)-C(37)	118.80(14)
C(9A)-C(8A)-H(8AC)	109.0	C(32)-C(33)-C(37)	119.53(14)
H(8AA)-C(8A)-H(8AC)	107.8	C(33)-C(34)-C(35)	117.50(14)
C(10A)-C(9A)-C(8A)	114(2)	C(33)-C(34)-H(34)	121.2
C(10A)-C(9A)-H(9AA)	108.7	C(35)-C(34)-H(34)	121.2
C(8A)-C(9A)-H(9AA)	108.7	C(34)-C(35)-C(36)	121.03(15)
C(10A)-C(9A)-H(9AC)	108.7	C(34)-C(35)-C(38)	119.87(15)
C(8A)-C(9A)-H(9AC)	108.7	C(36)-C(35)-C(38)	119.10(15)
H(9AA)-C(9A)-H(9AC)	107.6	C(35)-C(36)-C(31)	122.44(14)
N(4A)-C(10A)-C(9A)	120(2)	C(35)-C(36)-H(36)	118.8
N(4A)-C(10A)-H(10C)	107.4	C(31)-C(36)-H(36)	118.8
C(9A)-C(10A)-H(10C)	107.4	F(32A)-C(37)-F(31A)	114.2(15)
N(4A)-C(10A)-H(10D)	107.4	F(31)-C(37)-F(33)	106.59(18)
C(9A)-C(10A)-H(10D)	107.4	F(31)-C(37)-F(32)	106.90(18)
H(10C)-C(10A)-H(10D)	106.9	F(33)-C(37)-F(32)	105.32(18)
N(4A)-C(11A)-H(11D)	109.5	F(32A)-C(37)-F(33A)	100.9(13)
N(4A)-C(11A)-H(11E)	109.5	F(31A)-C(37)-F(33A)	105.7(15)
H(11D)-C(11A)-H(11E)	109.5	F(32A)-C(37)-C(33)	117.2(10)
N(4A)-C(11A)-H(11F)	109.5	F(31)-C(37)-C(33)	113.41(15)
H(11D)-C(11A)-H(11F)	109.5	F(31A)-C(37)-C(33)	112.0(10)
H(11E)-C(11A)-H(11F)	109.5	F(33)-C(37)-C(33)	113.29(16)
N(4A)-C(12A)-C(13A)	109(2)	F(32)-C(37)-C(33)	110.79(16)
N(4A)-C(12A)-H(12C)	110.0	F(33A)-C(37)-C(33)	105.0(10)
C(13A)-C(12A)-H(12C)	110.0	F(36)-C(38)-F(34)	107.77(18)
N(4A)-C(12A)-H(12D)	110.0	F(36)-C(38)-F(35)	108.3(2)
C(13A)-C(12A)-H(12D)	110.0	F(34)-C(38)-F(35)	103.48(18)
H(12C)-C(12A)-H(12D)	108.3	F(34A)-C(38)-F(35A)	115.4(10)
N(1A)-C(13A)-C(12A)	110(2)	F(34A)-C(38)-F(36A)	102.0(10)
N(1A)-C(13A)-H(13C)	109.6	F(35A)-C(38)-F(36A)	94.3(8)
C(12A)-C(13A)-H(13C)	109.6	F(34A)-C(38)-C(35)	123.4(8)
N(1A)-C(13A)-H(13D)	109.6	F(36)-C(38)-C(35)	112.72(18)
C(12A)-C(13A)-H(13D)	109.6	F(34)-C(38)-C(35)	112.76(16)
H(13C)-C(13A)-H(13D)	108.1	F(35)-C(38)-C(35)	111.30(16)
N(1A)-C(14A)-H(14D)	109.5	F(35A)-C(38)-C(35)	111.7(5)
N(1A)-C(14A)-H(14E)	109.5	F(36A)-C(38)-C(35)	104.0(5)
H(14D)-C(14A)-H(14E)	109.5	C(46)-C(41)-C(42)	115.96(14)
N(1A)-C(14A)-H(14F)	109.5	C(46)-C(41)-B(21)	122.51(13)
H(14D)-C(14A)-H(14F)	109.5	C(42)-C(41)-B(21)	121.29(13)
H(14E)-C(14A)-H(14F)	109.5	C(43)-C(42)-C(41)	121.96(14)
O(3)-S(1)-O(2)	117.08(10)	C(43)-C(42)-H(42)	119.0
O(3)-S(1)-O(1)	112.39(9)	C(41)-C(42)-H(42)	119.0
O(2)-S(1)-O(1)	113.50(9)	C(42)-C(43)-C(44)	120.97(14)
O(3)-S(1)-C(15)	106.01(10)	C(42)-C(43)-C(47)	121.14(15)
O(2)-S(1)-C(15)	105.45(10)	C(44)-C(43)-C(47)	117.88(14)
O(1)-S(1)-C(15)	100.37(9)	C(45)-C(44)-C(43)	117.88(14)
S(1)-O(1)-Fe(1)	138.12(9)	C(45)-C(44)-H(44)	121.1
F(3)-C(15)-F(2)	108.5(2)	C(43)-C(44)-H(44)	121.1
F(3)-C(15)-F(1)	108.95(18)	C(44)-C(45)-C(46)	121.03(15)
F(2)-C(15)-F(1)	108.69(17)	C(44)-C(45)-C(48)	119.71(15)
F(3)-C(15)-S(1)	110.44(14)	C(46)-C(45)-C(48)	119.22(15)
F(2)-C(15)-S(1)	110.93(13)	C(45)-C(46)-C(41)	122.17(14)
F(1)-C(15)-S(1)	109.25(16)	C(45)-C(46)-H(46)	118.9
C(22)-C(21)-C(26)	115.88(14)	C(41)-C(46)-H(46)	118.9
C(22)-C(21)-B(21)	121.09(13)	F(41)-C(47)-F(42)	109.5(2)
C(26)-C(21)-B(21)	122.83(13)	F(42A)-C(47)-F(43A)	112.8(13)
C(23)-C(22)-C(21)	122.36(14)	F(41)-C(47)-F(43)	106.2(2)
C(23)-C(22)-H(22)	118.8	F(42)-C(47)-F(43)	103.10(17)
C(21)-C(22)-H(22)	118.8	F(42A)-C(47)-F(41A)	105.5(8)
C(24)-C(23)-C(22)	121.10(14)	F(43A)-C(47)-F(41A)	94.4(10)
C(24)-C(23)-C(27)	120.16(14)	F(42A)-C(47)-C(43)	123.6(5)
C(22)-C(23)-C(27)	118.64(15)	F(41)-C(47)-C(43)	114.6(2)
C(23)-C(24)-C(25)	117.70(14)	F(42)-C(47)-C(43)	112.39(15)
C(23)-C(24)-H(24)	121.1	F(43A)-C(47)-C(43)	110.1(12)
C(25)-C(24)-H(24)	121.1	F(43)-C(47)-C(43)	110.26(16)
C(26)-C(25)-C(24)	121.20(14)	F(41A)-C(47)-C(43)	105.7(4)
C(26)-C(25)-C(28)	120.86(14)	F(44)-C(48)-F(45)	106.47(17)
C(24)-C(25)-C(28)	117.94(14)	F(44)-C(48)-F(46)	107.6(2)

F(45)-C(48)-F(46)	106.01(18)	F(52)-C(57)-C(53)	111.75(16)
F(44)-C(48)-C(45)	111.82(17)	F(53)-C(57)-C(53)	112.88(16)
F(45)-C(48)-C(45)	112.72(16)	F(56A)-C(58)-F(55A)	112.8(4)
F(46)-C(48)-C(45)	111.84(15)	F(56B)-C(58)-F(55B)	110.1(4)
C(56)-C(51)-C(52)	115.54(14)	F(54)-C(58)-F(55)	123.7(3)
C(56)-C(51)-B(21)	124.15(13)	F(56A)-C(58)-F(54A)	112.4(4)
C(52)-C(51)-B(21)	120.24(13)	F(55A)-C(58)-F(54A)	84.3(4)
C(53)-C(52)-C(51)	122.45(15)	F(56B)-C(58)-F(54B)	106.9(4)
C(53)-C(52)-H(52)	118.8	F(55B)-C(58)-F(54B)	96.3(4)
C(51)-C(52)-H(52)	118.8	F(54)-C(58)-F(56)	105.0(3)
C(52)-C(53)-C(54)	120.91(15)	F(55)-C(58)-F(56)	88.9(3)
C(52)-C(53)-C(57)	120.33(16)	F(56B)-C(58)-C(55)	119.0(3)
C(54)-C(53)-C(57)	118.66(16)	F(56A)-C(58)-C(55)	115.8(3)
C(55)-C(54)-C(53)	117.91(15)	F(54)-C(58)-C(55)	116.9(2)
C(55)-C(54)-H(54)	121.0	F(55A)-C(58)-C(55)	117.7(3)
C(53)-C(54)-H(54)	121.0	F(55B)-C(58)-C(55)	111.4(3)
C(54)-C(55)-C(56)	120.86(16)	F(55)-C(58)-C(55)	108.7(2)
C(54)-C(55)-C(58)	119.42(16)	F(54A)-C(58)-C(55)	109.6(3)
C(56)-C(55)-C(58)	119.71(16)	F(54B)-C(58)-C(55)	110.6(3)
C(51)-C(56)-C(55)	122.32(15)	F(56)-C(58)-C(55)	108.7(2)
C(51)-C(56)-H(56)	118.8	C(21)-B(21)-C(31)	114.68(12)
C(55)-C(56)-H(56)	118.8	C(21)-B(21)-C(51)	105.19(12)
F(51)-C(57)-F(52)	105.57(18)	C(31)-B(21)-C(51)	112.40(12)
F(51)-C(57)-F(53)	106.36(16)	C(21)-B(21)-C(41)	111.08(12)
F(52)-C(57)-F(53)	106.18(18)	C(31)-B(21)-C(41)	102.82(12)
F(51)-C(57)-C(53)	113.52(16)	C(51)-B(21)-C(41)	110.82(12)

Table S4. Anisotropic displacement parameters ($Å^2$ x 10^3) for **1a-B$_{ArF}$**.
The anisotropic displacement factor exponent takes the form: $-2p^2[h^2 a^{*2}U^{11} + ... + 2 h k a^* b^* U^{12}]$

	U^{11}	U^{22}	U^{33}	U^{23}	U^{13}	U^{12}
Fe(1)	13(1)	11(1)	9(1)	0(1)	1(1)	2(1)
N(1)	32(1)	21(1)	15(1)	-3(1)	3(1)	10(1)
N(2)	14(1)	23(1)	14(1)	3(1)	1(1)	-2(1)
N(3)	17(1)	18(1)	12(1)	2(1)	4(1)	5(1)
N(4)	25(1)	16(1)	30(1)	4(1)	-8(1)	-5(1)
C(1)	38(1)	34(1)	21(1)	2(1)	7(1)	22(1)
C(2)	21(1)	50(1)	22(1)	6(1)	8(1)	16(1)
C(3)	14(1)	38(1)	19(1)	5(1)	0(1)	4(1)
C(4)	24(1)	31(1)	25(1)	11(1)	-1(1)	-10(1)
C(5)	25(1)	24(1)	17(1)	-4(1)	-2(1)	-4(1)
C(6)	24(1)	26(1)	11(1)	-1(1)	1(1)	3(1)
C(7)	24(1)	25(1)	18(1)	3(1)	9(1)	11(1)
C(8)	24(1)	26(1)	18(1)	10(1)	8(1)	5(1)
C(9)	23(1)	31(1)	38(1)	20(1)	7(1)	-2(1)
C(10)	28(1)	18(1)	42(1)	13(1)	-7(1)	-9(1)
C(11)	22(1)	28(1)	42(1)	11(1)	-12(1)	-9(1)
C(12)	48(2)	18(1)	35(1)	-8(1)	-12(1)	-6(1)
C(13)	56(2)	18(1)	33(1)	-10(1)	-2(1)	7(1)
C(14)	40(1)	36(1)	13(1)	-3(1)	4(1)	17(1)
N(1A)	37(6)	19(6)	36(6)	3(6)	5(6)	-2(6)
N(2A)	28(7)	35(6)	18(6)	0(6)	6(6)	10(5)
N(3A)	27(7)	32(7)	24(6)	-2(6)	0(5)	0(6)
N(4A)	23(6)	24(5)	28(6)	5(5)	9(5)	6(4)
C(1A)	46(8)	23(8)	31(9)	-4(7)	9(7)	3(6)
C(2A)	45(7)	30(7)	29(8)	-4(8)	8(7)	11(6)
C(3A)	36(8)	34(7)	23(8)	1(7)	6(8)	13(6)
C(4A)	38(16)	38(16)	18(8)	5(9)	10(9)	7(14)
C(5A)	27(7)	39(7)	25(8)	-1(7)	5(7)	5(6)
C(6A)	28(8)	35(7)	27(8)	1(7)	2(7)	2(7)
C(7A)	35(14)	30(11)	28(13)	0(10)	7(11)	0(9)
C(8A)	28(8)	33(9)	22(6)	-5(7)	1(6)	2(7)
C(9A)	32(8)	31(7)	25(7)	-4(7)	5(6)	5(6)
C(10A)	26(7)	27(7)	26(7)	2(7)	4(6)	10(6)
C(11A)	24(1)	25(1)	18(1)	3(1)	9(1)	11(1)
C(12A)	23(1)	31(1)	38(1)	20(1)	7(1)	-2(1)
C(13A)	28(7)	22(8)	41(6)	11(6)	3(5)	-1(7)
C(14A)	54(16)	32(16)	37(8)	8(12)	1(9)	-17(13)
S(1)	18(1)	17(1)	13(1)	4(1)	-1(1)	2(1)
F(1)	51(1)	29(1)	39(1)	6(1)	-21(1)	15(1)
F(2)	34(1)	29(1)	36(1)	-9(1)	-2(1)	9(1)
F(3)	17(1)	40(1)	77(1)	7(1)	8(1)	3(1)
O(1)	21(1)	20(1)	15(1)	6(1)	1(1)	4(1)
O(2)	36(1)	22(1)	24(1)	-4(1)	-8(1)	2(1)
O(3)	28(1)	31(1)	23(1)	7(1)	8(1)	-3(1)
C(15)	19(1)	20(1)	31(1)	3(1)	-5(1)	4(1)
F(21)	88(1)	14(1)	40(1)	-4(1)	21(1)	-14(1)
F(22)	20(1)	24(1)	54(1)	18(1)	6(1)	-7(1)
F(23)	31(1)	28(1)	50(1)	28(1)	-14(1)	-8(1)
F(24)	18(2)	20(1)	54(2)	16(1)	-13(1)	-11(1)
F(25)	12(1)	38(2)	82(4)	-17(2)	3(2)	1(1)
F(26)	37(2)	70(3)	24(1)	14(1)	-18(1)	-26(2)
F(24A)	21(4)	58(7)	78(8)	37(6)	-11(5)	-17(5)
F(25A)	16(2)	23(3)	75(7)	1(4)	-12(4)	4(2)
F(26A)	34(4)	77(8)	49(4)	-36(4)	-19(3)	-4(5)
F(31)	25(1)	24(1)	46(1)	6(1)	20(1)	12(1)
F(32)	14(1)	52(1)	82(2)	-20(1)	13(1)	-11(1)
F(33)	42(1)	58(1)	40(1)	25(1)	31(1)	23(1)
F(31A)	33(11)	39(11)	46(10)	18(9)	26(9)	23(9)
F(32A)	42(1)	58(1)	40(1)	25(1)	31(1)	23(1)
F(33A)	42(1)	58(1)	40(1)	25(1)	31(1)	23(1)
F(34)	30(1)	34(1)	21(1)	14(1)	5(1)	10(1)
F(35)	16(1)	63(1)	65(1)	43(1)	10(1)	13(1)
F(36)	91(2)	23(1)	35(1)	-6(1)	-10(1)	32(1)
F(34A)	49(9)	43(8)	40(5)	8(6)	-7(5)	14(7)
F(35A)	30(1)	34(1)	21(1)	14(1)	5(1)	10(1)
F(36A)	41(6)	17(4)	42(7)	15(5)	24(5)	10(4)
F(41)	28(1)	31(2)	36(2)	-18(1)	-12(1)	18(1)
F(42)	50(1)	17(1)	47(1)	-14(1)	28(1)	-12(1)
F(43)	29(1)	34(1)	14(1)	-8(1)	2(1)	4(1)
F(41A)	50(1)	17(1)	47(1)	-14(1)	28(1)	-12(1)
F(42A)	18(4)	40(6)	47(5)	-35(4)	-7(4)	5(4)
F(43A)	19(4)	19(7)	31(7)	-3(5)	2(4)	11(4)
F(44)	17(1)	84(1)	47(1)	-5(1)	12(1)	3(1)
F(45)	18(1)	36(1)	45(1)	-14(1)	-16(1)	7(1)
F(46)	20(1)	24(1)	122(2)	-35(1)	-18(1)	11(1)
F(51)	44(1)	34(1)	34(1)	-4(1)	22(1)	-21(1)
F(52)	18(1)	76(1)	74(1)	45(1)	17(1)	10(1)
F(53)	48(1)	48(1)	15(1)	-1(1)	15(1)	-16(1)
F(54)	40(1)	23(1)	50(1)	-16(1)	11(1)	-12(1)
F(55)	40(1)	23(1)	50(1)	-16(1)	11(1)	-12(1)
F(56)	40(1)	23(1)	50(1)	-16(1)	11(1)	-12(1)
F(54A)	40(1)	23(1)	50(1)	-16(1)	11(1)	-12(1)
F(55A)	40(1)	23(1)	50(1)	-16(1)	11(1)	-12(1)
F(56A)	40(1)	23(1)	50(1)	-16(1)	11(1)	-12(1)
F(54B)	40(1)	23(1)	50(1)	-16(1)	11(1)	-12(1)
F(55B)	40(1)	23(1)	50(1)	-16(1)	11(1)	-12(1)
F(56B)	40(1)	23(1)	50(1)	-16(1)	11(1)	-12(1)
C(21)	10(1)	11(1)	11(1)	2(1)	1(1)	-1(1)
C(22)	12(1)	11(1)	15(1)	3(1)	1(1)	-2(1)
C(23)	14(1)	10(1)	15(1)	4(1)	2(1)	-1(1)

C(24)	14(1)	12(1)	15(1)	2(1)	-1(1)	2(1)
C(25)	11(1)	12(1)	14(1)	1(1)	-2(1)	-1(1)
C(26)	12(1)	10(1)	13(1)	2(1)	0(1)	-1(1)
C(27)	20(1)	13(1)	24(1)	8(1)	0(1)	-2(1)
C(28)	14(1)	17(1)	24(1)	4(1)	-5(1)	-2(1)
C(31)	10(1)	9(1)	12(1)	1(1)	1(1)	-1(1)
C(32)	10(1)	10(1)	15(1)	1(1)	2(1)	0(1)
C(33)	10(1)	12(1)	15(1)	1(1)	5(1)	0(1)
C(34)	14(1)	12(1)	16(1)	3(1)	5(1)	-1(1)
C(35)	13(1)	13(1)	17(1)	5(1)	4(1)	2(1)
C(36)	12(1)	13(1)	15(1)	4(1)	4(1)	3(1)
C(37)	16(1)	21(1)	29(1)	6(1)	11(1)	3(1)
C(38)	24(1)	22(1)	25(1)	12(1)	10(1)	10(1)
C(41)	10(1)	10(1)	10(1)	2(1)	1(1)	-1(1)
C(42)	12(1)	11(1)	12(1)	1(1)	2(1)	0(1)
C(43)	15(1)	10(1)	12(1)	-1(1)	3(1)	0(1)
C(44)	14(1)	12(1)	15(1)	-2(1)	-1(1)	-1(1)
C(45)	11(1)	11(1)	18(1)	-1(1)	-1(1)	0(1)
C(46)	12(1)	10(1)	15(1)	-1(1)	0(1)	0(1)
C(47)	18(1)	19(1)	20(1)	-7(1)	5(1)	-1(1)
C(48)	13(1)	20(1)	35(1)	-12(1)	-4(1)	2(1)
C(51)	9(1)	11(1)	11(1)	1(1)	0(1)	-1(1)
C(52)	13(1)	14(1)	12(1)	2(1)	2(1)	-2(1)
C(53)	13(1)	20(1)	11(1)	3(1)	3(1)	-1(1)
C(54)	20(1)	21(1)	13(1)	-3(1)	4(1)	0(1)
C(55)	18(1)	15(1)	16(1)	-4(1)	3(1)	-3(1)
C(56)	13(1)	13(1)	14(1)	-1(1)	3(1)	-2(1)
C(57)	20(1)	28(1)	16(1)	4(1)	6(1)	-2(1)
C(58)	34(1)	19(1)	36(1)	-11(1)	10(1)	-7(1)
B(21)	9(1)	9(1)	12(1)	2(1)	1(1)	-1(1)

Table S5. Hydrogen coordinates (x 10^4) and isotropic displacement parameters (Å^{2}x 10^3) for **1a-B$_{ArF}$**.

	x	y	z	U(eq)
H(1A)	2551	853	1894	36
H(1AB)	2169	896	2737	36
H(2A)	3045	1778	1987	37
H(2AB)	3794	1398	2564	37
H(3A)	2695	1660	3566	29
H(3AB)	3471	2113	3289	29
H(4A)	2748	2882	2680	41
H(4B)	1472	2929	2361	41
H(4C)	2251	2534	1954	41
H(5A)	1161	2824	3599	27
H(5AB)	2342	2629	3993	27
H(6A)	1590	1827	4367	24
H(6AB)	916	2308	4686	24
H(7A)	-980	2368	4347	33
H(7B)	-1495	2202	3494	33
H(7C)	-580	2658	3612	33
H(8A)	-388	1523	4724	27
H(8AB)	381	1178	4237	27
H(9A)	-1870	1389	3724	36
H(9AB)	-1465	859	4173	36
H(10A)	-432	655	3114	37
H(10B)	-1737	616	2923	37
H(11A)	-2731	1173	2076	47
H(11B)	-2196	1678	1704	47
H(11C)	-2385	1683	2597	47
H(12A)	-977	1127	1230	42
H(12B)	-1231	588	1664	42
H(13A)	593	532	2232	43
H(13B)	589	590	1316	43
H(14A)	1621	1292	833	44
H(14B)	1553	1865	1223	44
H(14C)	451	1566	875	44
H(1AA)	-333	270	2241	39
H(1AC)	345	535	2984	39
H(2AA)	1478	201	2062	41
H(2AC)	961	638	1463	41
H(3AA)	2178	925	2892	37
H(3AC)	2760	841	2128	37
H(4AA)	1046	1391	1055	46
H(4AB)	1460	1990	1194	46
H(4AC)	2317	1533	1041	46
H(5AA)	3385	1838	2167	36
H(5AC)	3032	1690	2997	36
H(6AA)	3006	2612	2864	36
H(6AC)	2211	2570	2068	36
H(7AA)	315	3036	3141	46
H(7AB)	1528	3249	3062	46
H(7AC)	764	3040	2315	46
H(8AA)	2243	2783	3981	33
H(8AC)	2441	2156	3952	33
H(9AA)	1349	2382	4934	35
H(9AC)	449	2639	4304	35
H(10C)	990	1550	4240	32
H(10D)	109	1747	4779	32
H(11D)	-1869	2095	3196	33
H(11E)	-1636	2159	4115	33
H(11F)	-965	2511	3565	33
H(12C)	-1416	1158	4008	36
H(12D)	-247	951	3799	36
H(13C)	-1719	711	2815	36
H(13D)	-1961	1330	2656	36
H(14D)	-628	1044	1096	63
H(14E)	-1566	706	1438	63
H(14F)	-1654	1340	1396	63
H(22)	4991	3456	3129	15
H(24)	7979	3202	4341	17
H(26)	7323	4597	3204	14
H(32)	7051	4485	1843	14
H(34)	6419	5655	244	16
H(36)	4269	5355	1793	16
H(42)	5137	3584	1676	14
H(44)	1949	3397	772	17
H(46)	2825	4601	2265	15
H(52)	3603	4250	3604	16
H(54)	3403	5593	4864	21
H(56)	5406	5533	3167	16

[(D_{12}-TMC)Fe^{II}(OTf)]$B_{Ar}F$, **1b-B_{ArF}**

The crystal structure of **1b-B_{ArF}** was solved in the monoclinic space group $P2_1/c$ and the asymmetric unit contains one full formula unit. The ligand was found to be disordered over two positions and five of the CF_3 groups of the anion were also found to be disordered. Disorder was refined with the help of 1,2 and 1,3 same distance restraints, similarity restraints on anisotropic displacement parameters, advanced rigid bond restraints and setting some atoms close to each other to have the same anisotropic displacement parameters. The disorder ratios were refined and converged to 0.976(2) (ligand), 0.422(2):0.347(2):0.231(2), 0.67(4), 0.976(2), 0.886(5) and 0.939(3) (CF_3 groups).

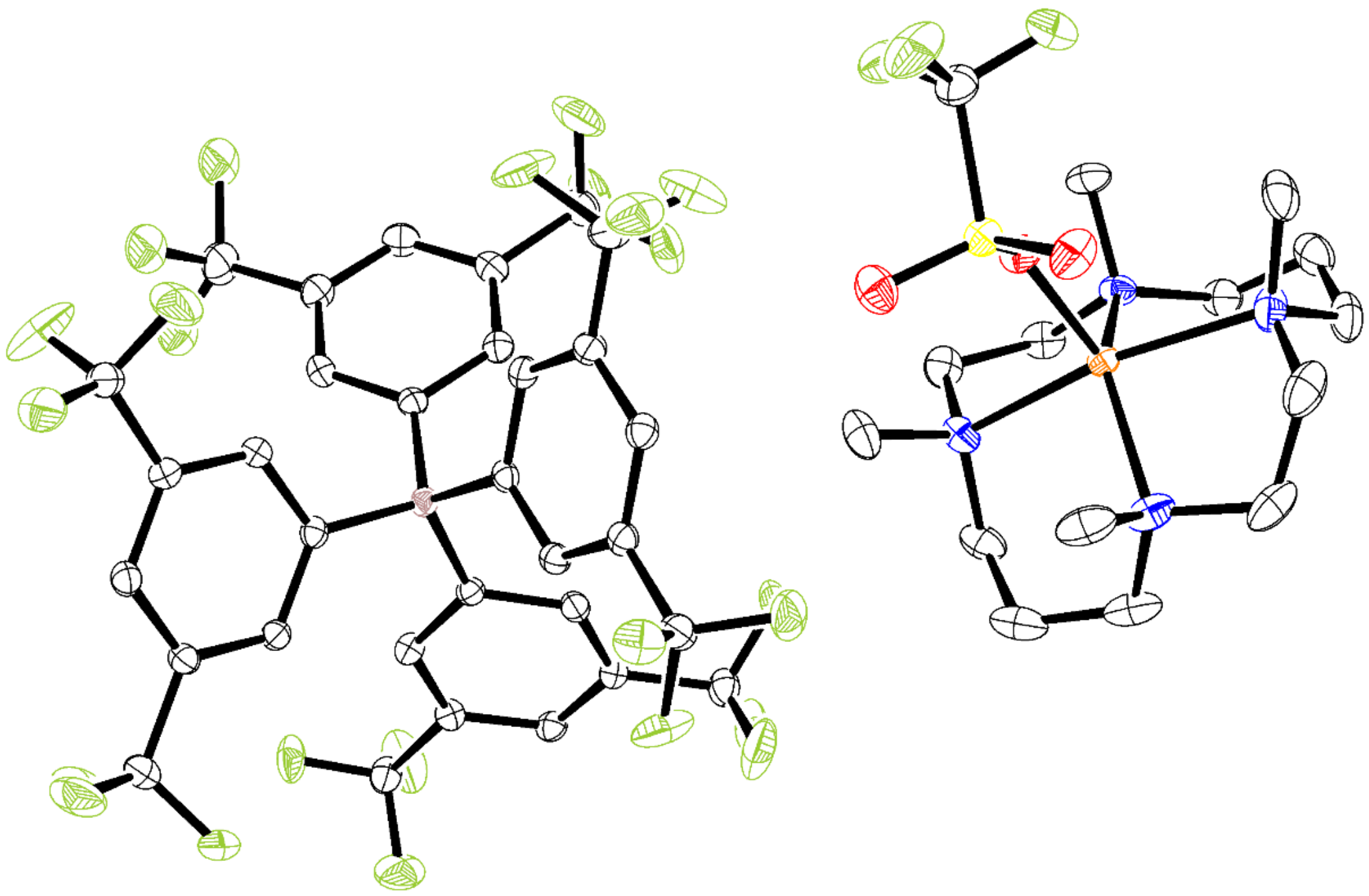

Figure S2. Molecular structure of **1b-B_{ArF}**.

Table S6. Crystal data and structure refinement for **1b-B_{ArF}**

CCDC No	2062765	
Empirical formula	$C_{47}H_{32}BD_{12}F_{27}FeN_4O_3S$	
Formula weight	1336.65	
Temperature	100(2) K	
Wavelength	0.71073 Å	
Crystal system	Monoclinic	
Space group	$P2_1/c$	
Unit cell dimensions	a = 12.2588(4) Å	α = 90°
	b = 25.1709(9) Å	β = 96.7556(14)°
	c = 17.4126(6) Å	γ = 90°
Volume	5335.6(3) $Å^3$	
Z	4	
Density (calculated)	1.664 Mg/m^3	
Absorption coefficient	0.462 mm^{-1}	
F(000)	2672	
Crystal size	0.734 x 0.554 x 0.247 mm^3	
Theta range for data collection	2.001 to 28.699°.	
Index ranges	-16 ≤ h ≤ 16, -34 ≤ k ≤ 34, -23 ≤ l ≤ 23	
Reflections collected	202360	
Independent reflections	13801 [R(int) = 0.0645]	
Completeness to theta = 25.242°	100.0 %	
Absorption correction	Semi-empirical from equivalents	
Refinement method	Full-matrix least-squares on F^2	
Data / restraints / parameters	13801 / 4046 / 997	
Goodness-of-fit on F^2	1.030	
Final R indices [I>2σ(I)]	R1 = 0.0497, wR2 = 0.1152	
R indices (all data)	R1 = 0.0702, wR2 = 0.1275	
Largest diff. peak and hole	1.548 and -1.261 e$Å^{-3}$	

Table S7. Atomic coordinates (x 10^4) and equivalent isotropic displacement parameters ($Å^2$x 10^3) for **1b-B$_{Ar}$F**.
U(eq) is defined as one third of the trace of the orthogonalized U^{ij} tensor.

	x	y	z	U(eq)
Fe(1)	287(1)	1813(1)	2594(1)	15(1)
N(1)	1110(2)	1258(1)	1913(1)	27(1)
N(2)	1850(2)	2251(1)	2973(1)	22(1)
N(3)	58(2)	1914(1)	3792(1)	20(1)
N(4)	-1082(2)	1232(1)	2386(1)	26(1)
C(1)	2230(2)	1090(1)	2254(2)	33(1)
C(2)	3037(2)	1539(1)	2445(2)	34(1)
C(3)	2816(2)	1888(1)	3123(2)	28(1)
C(4)	2106(2)	2681(1)	2443(2)	31(1)
D(4A)	2762	2873	2669	46
D(4B)	1484	2927	2362	46
D(4C)	2242	2528	1945	46
C(5)	1649(2)	2505(1)	3711(2)	26(1)
C(6)	1094(2)	2125(1)	4215(1)	25(1)
C(7)	-818(2)	2323(1)	3821(1)	25(1)
D(7A)	-958	2376	4359	38
D(7B)	-1493	2204	3513	38
D(7C)	-577	2659	3612	38
C(8)	-247(2)	1425(1)	4201(1)	26(1)
C(9)	-1256(2)	1138(1)	3820(2)	33(1)
C(10)	-1126(2)	869(1)	3056(2)	32(1)
C(11)	-2192(2)	1466(1)	2183(2)	34(1)
D(11A)	-2735	1180	2095	50
D(11B)	-2200	1679	1711	50
D(11C)	-2375	1692	2607	50
C(12)	-805(3)	920(1)	1715(2)	36(1)
C(13)	403(3)	775(1)	1792(2)	37(1)
C(14)	1180(2)	1513(1)	1146(1)	33(1)
D(14A)	1589	1281	830	50
D(14B)	1560	1855	1222	50
D(14C)	438	1570	883	50
N(1A)	-460(40)	1114(18)	2300(30)	30(3)
N(2A)	1970(40)	1487(18)	2060(30)	30(4)
N(3A)	1470(40)	2396(17)	3020(30)	24(3)
N(4A)	-450(40)	1773(18)	3670(20)	24(3)
C(1A)	160(50)	600(20)	2440(40)	31(5)
C(2A)	1130(50)	560(20)	1990(40)	33(5)
C(3A)	2090(50)	920(20)	2300(50)	32(4)
C(4A)	2260(80)	1530(30)	1260(30)	34(9)
D(4AA)	1715	1335	906	51
D(4AB)	2254	1905	1105	51
D(4AC)	2988	1379	1233	51
C(5A)	2820(40)	1790(20)	2560(40)	30(4)
C(6A)	2460(50)	2370(20)	2590(40)	26(4)
C(7A)	990(60)	2940(30)	2840(50)	31(1)
D(7AA)	336	2988	3101	46
D(7AB)	1537	3213	3016	46
D(7AC)	794	2976	2278	46
C(8A)	1890(40)	2410(30)	3860(30)	24(4)
C(9A)	1030(50)	2350(30)	4410(30)	25(5)
C(10A)	390(50)	1830(20)	4340(30)	23(4)
C(11A)	-1320(60)	2180(30)	3640(50)	25(1)
D(11D)	-985	2534	3663	38
D(11E)	-1821	2143	3166	38
D(11F)	-1731	2134	4090	38
C(12A)	-920(60)	1230(20)	3640(30)	33(1)
C(13A)	-1370(40)	1080(30)	2810(30)	32(4)
C(14A)	-1020(70)	1090(40)	1490(30)	36(1)
D(14D)	-465	1104	1123	55
D(14E)	-1426	752	1411	55
D(14F)	-1528	1386	1392	55
S(1)	-783(1)	2602(1)	1235(1)	20(1)
F(1)	-2253(2)	3285(1)	688(1)	44(1)
F(2)	-1590(1)	3433(1)	1869(1)	36(1)
F(3)	-2741(1)	2797(1)	1604(1)	47(1)
O(1)	-487(1)	2422(1)	2042(1)	22(1)
O(2)	-1259(2)	2191(1)	734(1)	30(1)
O(3)	56(2)	2918(1)	957(1)	30(1)
C(15)	-1910(2)	3057(1)	1359(2)	28(1)
F(21)	6130(2)	2377(1)	3342(1)	49(1)
F(22)	5031(1)	2692(1)	4094(1)	36(1)
F(23)	6675(1)	2485(1)	4545(1)	41(1)
F(24)	9194(9)	4578(3)	3807(6)	34(2)
F(25)	9691(6)	3763(4)	3761(9)	46(2)
F(26)	9238(9)	4089(5)	4812(3)	48(2)
F(24A)	9310(20)	4500(9)	3647(16)	49(4)
F(25A)	9718(12)	3718(6)	4043(17)	44(3)
F(26A)	9076(15)	4256(12)	4793(8)	53(4)
F(31)	8285(1)	4451(1)	780(1)	34(1)
F(32)	8775(1)	5237(1)	1143(1)	51(1)
F(33)	8005(2)	5101(1)	-6(1)	46(1)
F(31A)	8640(20)	4658(17)	1220(20)	41(7)
F(32A)	8500(30)	5385(10)	540(30)	46(1)
F(33A)	7830(30)	4669(16)	69(15)	46(1)
F(34)	4605(2)	6076(1)	-122(1)	32(1)
F(35)	3416(1)	5824(1)	587(1)	48(1)
F(36)	4595(2)	6424(1)	997(1)	54(1)
F(34A)	3980(20)	5931(12)	-72(9)	55(6)
F(35A)	3745(16)	6126(9)	1096(11)	32(1)
F(36A)	5115(16)	6436(6)	606(16)	39(5)

F(41)	4921(2)	2806(2)	841(3)	37(1)
F(42)	3235(2)	2536(1)	776(2)	39(1)
F(43)	3718(2)	3069(1)	-69(1)	29(1)
F(41A)	3740(17)	2503(4)	1134(10)	39(1)
F(42A)	3433(14)	2879(8)	24(8)	37(4)
F(43A)	4993(11)	2906(13)	731(19)	22(4)
F(44)	555(1)	3969(1)	2022(1)	51(1)
F(45)	488(1)	4084(1)	805(1)	38(1)
F(46)	985(1)	4723(1)	1567(1)	61(1)
F(51)	2366(1)	4121(1)	4556(1)	38(1)
F(52)	1522(1)	4861(1)	4479(1)	57(1)
F(53)	2684(2)	4692(1)	5462(1)	41(1)
F(54)	5865(3)	6269(2)	4415(3)	38(1)
F(55)	3919(4)	6482(2)	4421(3)	38(1)
F(56)	4682(4)	6494(2)	3487(3)	38(1)
F(54A)	5562(4)	6232(2)	4833(3)	38(1)
F(55A)	4196(5)	6495(2)	4611(4)	38(1)
F(56A)	5085(4)	6464(2)	3631(3)	38(1)
F(54B)	5936(6)	6268(3)	4043(5)	38(1)
F(55B)	5018(7)	6281(3)	5012(4)	38(1)
F(56B)	4313(6)	6563(2)	3885(4)	38(1)
C(21)	6014(2)	4089(1)	3077(1)	15(1)
C(22)	5724(2)	3580(1)	3300(1)	17(1)
C(23)	6444(2)	3253(1)	3765(1)	18(1)
C(24)	7496(2)	3423(1)	4026(1)	18(1)
C(25)	7814(2)	3924(1)	3798(1)	17(1)
C(26)	7089(2)	4252(1)	3344(1)	17(1)
C(27)	6075(2)	2706(1)	3946(1)	23(1)
C(28)	8972(2)	4096(1)	4048(1)	23(1)
C(31)	5607(2)	4856(1)	1948(1)	15(1)
C(32)	6585(2)	4760(1)	1634(1)	16(1)
C(33)	6886(2)	5054(1)	1013(1)	17(1)
C(34)	6216(2)	5452(1)	668(1)	18(1)
C(35)	5235(2)	5550(1)	966(1)	18(1)
C(36)	4945(2)	5264(1)	1594(1)	17(1)
C(37)	7975(2)	4954(1)	731(1)	25(1)
C(38)	4475(2)	5974(1)	613(1)	25(1)
C(41)	4148(2)	4140(1)	2084(1)	15(1)
C(42)	4400(2)	3698(1)	1643(1)	16(1)
C(43)	3600(2)	3426(1)	1167(1)	17(1)
C(44)	2505(2)	3584(1)	1100(1)	18(1)
C(45)	2240(2)	4025(1)	1517(1)	19(1)
C(46)	3044(2)	4295(1)	1997(1)	17(1)
C(47)	3892(2)	2958(1)	701(1)	22(1)
C(48)	1071(2)	4199(1)	1476(2)	27(1)
C(51)	4595(2)	4836(1)	3262(1)	15(1)
C(52)	3833(2)	4607(1)	3708(1)	17(1)
C(53)	3385(2)	4884(1)	4283(1)	19(1)
C(54)	3703(2)	5406(1)	4462(1)	22(1)
C(55)	4464(2)	5638(1)	4040(1)	21(1)
C(56)	4895(2)	5360(1)	3450(1)	17(1)
C(57)	2501(2)	4641(1)	4693(1)	25(1)
C(58)	4815(2)	6200(1)	4211(2)	32(1)
B(21)	5115(2)	4484(1)	2598(1)	15(1)

Table S8. Bond lengths [Å] and angles [°] for **1b-B$_{ArF}$**.

Fe(1)-O(1)	1.9897(15)	C(9A)-C(10A)	1.51(2)
Fe(1)-N(1A)	2.02(4)	C(9A)-H(9AA)	0.9900
Fe(1)-N(4A)	2.17(4)	C(9A)-H(9AC)	0.9900
Fe(1)-N(3)	2.1523(19)	C(10A)-H(10C)	0.9900
Fe(1)-N(1)	2.159(2)	C(10A)-H(10D)	0.9900
Fe(1)-N(3A)	2.14(4)	C(11A)-D(11D)	0.9800
Fe(1)-N(2A)	2.49(4)	C(11A)-D(11E)	0.9800
Fe(1)-N(4)	2.222(2)	C(11A)-D(11F)	0.9800
Fe(1)-N(2)	2.241(2)	C(12A)-C(13A)	1.52(2)
N(1)-C(1)	1.491(3)	C(12A)-H(12C)	0.9900
N(1)-C(14)	1.493(3)	C(12A)-H(12D)	0.9900
N(1)-C(13)	1.494(4)	C(13A)-H(13C)	0.9900
N(2)-C(4)	1.480(3)	C(13A)-H(13D)	0.9900
N(2)-C(5)	1.483(3)	C(14A)-D(14D)	0.9800
N(2)-C(3)	1.493(3)	C(14A)-D(14E)	0.9800
N(3)-C(6)	1.489(3)	C(14A)-D(14F)	0.9800
N(3)-C(8)	1.491(3)	S(1)-O(3)	1.4281(18)
N(3)-C(7)	1.493(3)	S(1)-O(2)	1.4314(18)
N(4)-C(12)	1.481(4)	S(1)-O(1)	1.4813(16)
N(4)-C(11)	1.487(3)	S(1)-C(15)	1.827(3)
N(4)-C(10)	1.489(3)	F(1)-C(15)	1.325(3)
C(1)-C(2)	1.512(4)	F(2)-C(15)	1.325(3)
C(1)-H(1A)	0.9900	F(3)-C(15)	1.324(3)
C(1)-H(1AB)	0.9900	F(21)-C(27)	1.346(3)
C(2)-C(3)	1.521(4)	F(22)-C(27)	1.334(3)
C(2)-H(2A)	0.9900	F(23)-C(27)	1.326(3)
C(2)-H(2AB)	0.9900	F(24)-C(28)	1.323(6)
C(3)-H(3A)	0.9900	F(25)-C(28)	1.352(6)
C(3)-H(3AB)	0.9900	F(26)-C(28)	1.331(6)
C(4)-D(4A)	0.9800	F(24A)-C(28)	1.327(11)
C(4)-D(4B)	0.9800	F(25A)-C(28)	1.319(10)
C(4)-D(4C)	0.9800	F(26A)-C(28)	1.351(10)
C(5)-C(6)	1.512(4)	F(31)-C(37)	1.322(3)
C(5)-H(5A)	0.9900	F(32)-C(37)	1.348(3)
C(5)-H(5AB)	0.9900	F(33)-C(37)	1.339(3)
C(6)-H(6A)	0.9900	F(31A)-C(37)	1.335(13)
C(6)-H(6AB)	0.9900	F(32A)-C(37)	1.322(13)
C(7)-D(7A)	0.9800	F(33A)-C(37)	1.353(13)
C(7)-D(7B)	0.9800	F(34)-C(38)	1.334(3)
C(7)-D(7C)	0.9800	F(35)-C(38)	1.347(3)
C(8)-C(9)	1.515(4)	F(36)-C(38)	1.315(3)
C(8)-H(8A)	0.9900	F(34A)-C(38)	1.277(12)
C(8)-H(8AB)	0.9900	F(35A)-C(38)	1.354(11)
C(9)-C(10)	1.518(4)	F(36A)-C(38)	1.404(12)
C(9)-H(9A)	0.9900	F(41)-C(47)	1.313(3)
C(9)-H(9AB)	0.9900	F(42)-C(47)	1.348(3)
C(10)-H(10A)	0.9900	F(43)-C(47)	1.361(3)
C(10)-H(10B)	0.9900	F(41A)-C(47)	1.396(10)
C(11)-D(11A)	0.9800	F(42A)-C(47)	1.260(10)
C(11)-D(11B)	0.9800	F(43A)-C(47)	1.351(12)
C(11)-D(11C)	0.9800	F(44)-C(48)	1.335(3)
C(12)-C(13)	1.515(4)	F(45)-C(48)	1.328(3)
C(12)-H(12A)	0.9900	F(46)-C(48)	1.334(3)
C(12)-H(12B)	0.9900	F(51)-C(57)	1.336(3)
C(13)-H(13A)	0.9900	F(52)-C(57)	1.335(3)
C(13)-H(13B)	0.9900	F(53)-C(57)	1.338(3)
C(14)-D(14A)	0.9800	F(54)-C(58)	1.307(5)
C(14)-D(14B)	0.9800	F(55)-C(58)	1.392(5)
C(14)-D(14C)	0.9800	F(56)-C(58)	1.456(6)
N(1A)-C(13A)	1.51(2)	F(54A)-C(58)	1.336(5)
N(1A)-C(14A)	1.51(2)	F(55A)-C(58)	1.318(6)
N(1A)-C(1A)	1.51(2)	F(56A)-C(58)	1.285(6)
N(2A)-C(4A)	1.49(2)	F(54B)-C(58)	1.449(7)
N(2A)-C(5A)	1.49(2)	F(55B)-C(58)	1.402(7)
N(2A)-C(3A)	1.50(2)	F(56B)-C(58)	1.206(7)
N(3A)-C(6A)	1.50(2)	C(21)-C(22)	1.398(3)
N(3A)-C(8A)	1.50(2)	C(21)-C(26)	1.407(3)
N(3A)-C(7A)	1.51(2)	C(21)-B(21)	1.637(3)
N(4A)-C(12A)	1.47(2)	C(22)-C(23)	1.394(3)
N(4A)-C(10A)	1.48(2)	C(22)-H(22)	0.9500
N(4A)-C(11A)	1.47(2)	C(23)-C(24)	1.383(3)
C(1A)-C(2A)	1.51(2)	C(23)-C(27)	1.495(3)
C(1A)-H(1AA)	0.9900	C(24)-C(25)	1.392(3)
C(1A)-H(1AC)	0.9900	C(24)-H(24)	0.9500
C(2A)-C(3A)	1.52(2)	C(25)-C(26)	1.389(3)
C(2A)-H(2AA)	0.9900	C(25)-C(28)	1.499(3)
C(2A)-H(2AC)	0.9900	C(26)-H(26)	0.9500
C(3A)-H(3AA)	0.9900	C(31)-C(32)	1.396(3)
C(3A)-H(3AC)	0.9900	C(31)-C(36)	1.406(3)
C(4A)-D(4AA)	0.9800	C(31)-B(21)	1.638(3)
C(4A)-D(4AB)	0.9800	C(32)-C(33)	1.396(3)
C(4A)-D(4AC)	0.9800	C(32)-H(32)	0.9500
C(5A)-C(6A)	1.52(2)	C(33)-C(34)	1.385(3)
C(5A)-H(5AA)	0.9900	C(33)-C(37)	1.497(3)
C(5A)-H(5AC)	0.9900	C(34)-C(35)	1.387(3)
C(6A)-H(6AA)	0.9900	C(34)-H(34)	0.9500
C(6A)-H(6AC)	0.9900	C(35)-C(36)	1.389(3)
C(7A)-D(7AA)	0.9800	C(35)-C(38)	1.499(3)
C(7A)-D(7AB)	0.9800	C(36)-H(36)	0.9500
C(7A)-D(7AC)	0.9800	C(41)-C(46)	1.399(3)
C(8A)-C(9A)	1.51(2)	C(41)-C(42)	1.406(3)
C(8A)-H(8AA)	0.9900	C(41)-B(21)	1.646(3)
C(8A)-H(8AC)	0.9900	C(42)-C(43)	1.389(3)

C(42)-H(42)	0.9500
C(43)-C(44)	1.393(3)
C(43)-C(47)	1.497(3)
C(44)-C(45)	1.385(3)
C(44)-H(44)	0.9500
C(45)-C(46)	1.393(3)
C(45)-C(48)	1.492(3)
C(46)-H(46)	0.9500
C(51)-C(56)	1.397(3)
C(51)-C(52)	1.406(3)
C(51)-B(21)	1.645(3)
C(52)-C(53)	1.385(3)
C(52)-H(52)	0.9500
C(53)-C(54)	1.396(3)
C(53)-C(57)	1.497(3)
C(54)-C(55)	1.382(3)
C(54)-H(54)	0.9500
C(55)-C(56)	1.397(3)
C(55)-C(58)	1.498(3)
C(56)-H(56)	0.9500
O(1)-Fe(1)-N(1A)	112.0(15)
O(1)-Fe(1)-N(4A)	103.1(13)
N(1A)-Fe(1)-N(4A)	87.3(15)
O(1)-Fe(1)-N(3)	105.55(7)
O(1)-Fe(1)-N(1)	117.41(7)
N(3)-Fe(1)-N(1)	137.04(8)
O(1)-Fe(1)-N(3A)	84.5(14)
N(1A)-Fe(1)-N(3A)	163(2)
N(4A)-Fe(1)-N(3A)	94.1(14)
O(1)-Fe(1)-N(2A)	116.2(12)
N(1A)-Fe(1)-N(2A)	89.3(14)
N(4A)-Fe(1)-N(2A)	138.6(18)
N(3A)-Fe(1)-N(2A)	78.2(13)
O(1)-Fe(1)-N(4)	96.90(7)
N(3)-Fe(1)-N(4)	93.09(8)
N(1)-Fe(1)-N(4)	82.95(9)
O(1)-Fe(1)-N(2)	96.06(7)
N(3)-Fe(1)-N(2)	82.05(7)
N(1)-Fe(1)-N(2)	92.35(8)
N(4)-Fe(1)-N(2)	166.96(7)
C(1)-N(1)-C(14)	109.3(2)
C(1)-N(1)-C(13)	108.5(2)
C(14)-N(1)-C(13)	108.5(2)
C(1)-N(1)-Fe(1)	115.58(16)
C(14)-N(1)-Fe(1)	107.05(15)
C(13)-N(1)-Fe(1)	107.66(16)
C(4)-N(2)-C(5)	107.1(2)
C(4)-N(2)-C(3)	109.59(19)
C(5)-N(2)-C(3)	108.70(19)
C(4)-N(2)-Fe(1)	114.39(14)
C(5)-N(2)-Fe(1)	104.06(14)
C(3)-N(2)-Fe(1)	112.55(15)
C(6)-N(3)-C(8)	107.79(18)
C(6)-N(3)-C(7)	108.21(19)
C(8)-N(3)-C(7)	109.35(18)
C(6)-N(3)-Fe(1)	108.68(14)
C(8)-N(3)-Fe(1)	115.70(15)
C(7)-N(3)-Fe(1)	106.90(14)
C(12)-N(4)-C(11)	108.1(2)
C(12)-N(4)-C(10)	109.0(2)
C(11)-N(4)-C(10)	108.2(2)
C(12)-N(4)-Fe(1)	103.84(17)
C(11)-N(4)-Fe(1)	115.60(15)
C(10)-N(4)-Fe(1)	111.84(15)
N(1)-C(1)-C(2)	115.1(2)
N(1)-C(1)-H(1A)	108.5
C(2)-C(1)-H(1A)	108.5
N(1)-C(1)-H(1AB)	108.5
C(2)-C(1)-H(1AB)	108.5
H(1A)-C(1)-H(1AB)	107.5
C(1)-C(2)-C(3)	115.5(2)
C(1)-C(2)-H(2A)	108.4
C(3)-C(2)-H(2A)	108.4
C(1)-C(2)-H(2AB)	108.4
C(3)-C(2)-H(2AB)	108.4
H(2A)-C(2)-H(2AB)	107.5
N(2)-C(3)-C(2)	115.3(2)
N(2)-C(3)-H(3A)	108.5
C(2)-C(3)-H(3A)	108.5
N(2)-C(3)-H(3AB)	108.5
C(2)-C(3)-H(3AB)	108.5
H(3A)-C(3)-H(3AB)	107.5
N(2)-C(4)-D(4A)	109.5
N(2)-C(4)-D(4B)	109.5
D(4A)-C(4)-D(4B)	109.5
N(2)-C(4)-D(4C)	109.5
D(4A)-C(4)-D(4C)	109.5
D(4B)-C(4)-D(4C)	109.5
N(2)-C(5)-C(6)	111.3(2)
N(2)-C(5)-H(5A)	109.4
C(6)-C(5)-H(5A)	109.4
N(2)-C(5)-H(5AB)	109.4
C(6)-C(5)-H(5AB)	109.4
H(5A)-C(5)-H(5AB)	108.0
N(3)-C(6)-C(5)	110.74(19)
N(3)-C(6)-H(6A)	109.5
C(5)-C(6)-H(6A)	109.5
N(3)-C(6)-H(6AB)	109.5
C(5)-C(6)-H(6AB)	109.5
H(6A)-C(6)-H(6AB)	108.1
N(3)-C(7)-D(7A)	109.5
N(3)-C(7)-D(7B)	109.5
D(7A)-C(7)-D(7B)	109.5
N(3)-C(7)-D(7C)	109.5
D(7A)-C(7)-D(7C)	109.5
D(7B)-C(7)-D(7C)	109.5
N(3)-C(8)-C(9)	115.0(2)
N(3)-C(8)-H(8A)	108.5
C(9)-C(8)-H(8A)	108.5
N(3)-C(8)-H(8AB)	108.5
C(9)-C(8)-H(8AB)	108.5
H(8A)-C(8)-H(8AB)	107.5
C(8)-C(9)-C(10)	115.6(2)
C(8)-C(9)-H(9A)	108.4
C(10)-C(9)-H(9A)	108.4
C(8)-C(9)-H(9AB)	108.4
C(10)-C(9)-H(9AB)	108.4
H(9A)-C(9)-H(9AB)	107.5
N(4)-C(10)-C(9)	115.5(2)
N(4)-C(10)-H(10A)	108.4
C(9)-C(10)-H(10A)	108.4
N(4)-C(10)-H(10B)	108.4
C(9)-C(10)-H(10B)	108.4
H(10A)-C(10)-H(10B)	107.5
N(4)-C(11)-D(11A)	109.5
N(4)-C(11)-D(11B)	109.5
D(11A)-C(11)-D(11B)	109.5
N(4)-C(11)-D(11C)	109.5
D(11A)-C(11)-D(11C)	109.5
D(11B)-C(11)-D(11C)	109.5
N(4)-C(12)-C(13)	111.7(2)
N(4)-C(12)-H(12A)	109.3
C(13)-C(12)-H(12A)	109.3
N(4)-C(12)-H(12B)	109.3
C(13)-C(12)-H(12B)	109.3
H(12A)-C(12)-H(12B)	107.9
N(1)-C(13)-C(12)	111.1(2)
N(1)-C(13)-H(13A)	109.4
C(12)-C(13)-H(13A)	109.4
N(1)-C(13)-H(13B)	109.4
C(12)-C(13)-H(13B)	109.4
H(13A)-C(13)-H(13B)	108.0
N(1)-C(14)-D(14A)	109.5
N(1)-C(14)-D(14B)	109.5
D(14A)-C(14)-D(14B)	109.5
N(1)-C(14)-D(14C)	109.5
D(14A)-C(14)-D(14C)	109.5
D(14B)-C(14)-D(14C)	109.5
C(13A)-N(1A)-C(14A)	106(3)
C(13A)-N(1A)-C(1A)	104(3)
C(14A)-N(1A)-C(1A)	106(3)
C(13A)-N(1A)-Fe(1)	104(2)
C(14A)-N(1A)-Fe(1)	115(5)
C(1A)-N(1A)-Fe(1)	121(3)
C(4A)-N(2A)-C(5A)	106(3)
C(4A)-N(2A)-C(3A)	108(3)
C(5A)-N(2A)-C(3A)	107(3)
C(4A)-N(2A)-Fe(1)	129(4)
C(5A)-N(2A)-Fe(1)	100(2)
C(3A)-N(2A)-Fe(1)	106(3)
C(6A)-N(3A)-C(8A)	107(3)
C(6A)-N(3A)-C(7A)	105(3)
C(8A)-N(3A)-C(7A)	105(3)
C(6A)-N(3A)-Fe(1)	111(2)
C(8A)-N(3A)-Fe(1)	120(3)
C(7A)-N(3A)-Fe(1)	109(4)
C(12A)-N(4A)-C(10A)	110(3)
C(12A)-N(4A)-C(11A)	111(3)
C(10A)-N(4A)-C(11A)	113(3)
C(12A)-N(4A)-Fe(1)	102(2)
C(10A)-N(4A)-Fe(1)	111(3)
C(11A)-N(4A)-Fe(1)	109(4)
C(2A)-C(1A)-N(1A)	112(3)
C(2A)-C(1A)-H(1AA)	109.2
N(1A)-C(1A)-H(1AA)	109.2
C(2A)-C(1A)-H(1AC)	109.2
N(1A)-C(1A)-H(1AC)	109.2
H(1AA)-C(1A)-H(1AC)	107.9
C(1A)-C(2A)-C(3A)	114(3)
C(1A)-C(2A)-H(2AA)	108.8
C(3A)-C(2A)-H(2AA)	108.8
C(1A)-C(2A)-H(2AC)	108.8
C(3A)-C(2A)-H(2AC)	108.8
H(2AA)-C(2A)-H(2AC)	107.7
N(2A)-C(3A)-C(2A)	115(3)
N(2A)-C(3A)-H(3AA)	108.6
C(2A)-C(3A)-H(3AA)	108.6
N(2A)-C(3A)-H(3AC)	108.6
C(2A)-C(3A)-H(3AC)	108.6

H(3AA)-C(3A)-H(3AC)	107.5
N(2A)-C(4A)-D(4AA)	109.5
N(2A)-C(4A)-D(4AB)	109.5
D(4AA)-C(4A)-D(4AB)	109.5
N(2A)-C(4A)-D(4AC)	109.5
D(4AA)-C(4A)-D(4AC)	109.5
D(4AB)-C(4A)-D(4AC)	109.5
N(2A)-C(5A)-C(6A)	109(3)
N(2A)-C(5A)-H(5AA)	109.8
C(6A)-C(5A)-H(5AA)	109.8
N(2A)-C(5A)-H(5AC)	109.8
C(6A)-C(5A)-H(5AC)	109.8
H(5AA)-C(5A)-H(5AC)	108.3
N(3A)-C(6A)-C(5A)	109(3)
N(3A)-C(6A)-H(6AA)	110.0
C(5A)-C(6A)-H(6AA)	110.0
N(3A)-C(6A)-H(6AC)	110.0
C(5A)-C(6A)-H(6AC)	110.0
H(6AA)-C(6A)-H(6AC)	108.4
N(3A)-C(7A)-D(7AA)	109.5
N(3A)-C(7A)-D(7AB)	109.5
D(7AA)-C(7A)-D(7AB)	109.5
N(3A)-C(7A)-D(7AC)	109.5
D(7AA)-C(7A)-D(7AC)	109.5
D(7AB)-C(7A)-D(7AC)	109.5
N(3A)-C(8A)-C(9A)	115(3)
N(3A)-C(8A)-H(8AA)	108.4
C(9A)-C(8A)-H(8AA)	108.4
N(3A)-C(8A)-H(8AC)	108.4
C(9A)-C(8A)-H(8AC)	108.4
H(8AA)-C(8A)-H(8AC)	107.5
C(10A)-C(9A)-C(8A)	116(3)
C(10A)-C(9A)-H(9AA)	108.2
C(8A)-C(9A)-H(9AA)	108.2
C(10A)-C(9A)-H(9AC)	108.2
C(8A)-C(9A)-H(9AC)	108.2
H(9AA)-C(9A)-H(9AC)	107.3
N(4A)-C(10A)-C(9A)	117(3)
N(4A)-C(10A)-H(10C)	108.0
C(9A)-C(10A)-H(10C)	108.0
N(4A)-C(10A)-H(10D)	108.0
C(9A)-C(10A)-H(10D)	108.0
H(10C)-C(10A)-H(10D)	107.3
N(4A)-C(11A)-D(11D)	109.5
N(4A)-C(11A)-D(11E)	109.5
D(11D)-C(11A)-D(11E)	109.5
N(4A)-C(11A)-D(11F)	109.5
D(11D)-C(11A)-D(11F)	109.5
D(11E)-C(11A)-D(11F)	109.5
N(4A)-C(12A)-C(13A)	111(3)
N(4A)-C(12A)-H(12C)	109.5
C(13A)-C(12A)-H(12C)	109.5
N(4A)-C(12A)-H(12D)	109.5
C(13A)-C(12A)-H(12D)	109.5
H(12C)-C(12A)-H(12D)	108.1
N(1A)-C(13A)-C(12A)	110(3)
N(1A)-C(13A)-H(13C)	109.7
C(12A)-C(13A)-H(13C)	109.7
N(1A)-C(13A)-H(13D)	109.7
C(12A)-C(13A)-H(13D)	109.7
H(13C)-C(13A)-H(13D)	108.2
N(1A)-C(14A)-D(14D)	109.5
N(1A)-C(14A)-D(14E)	109.5
D(14D)-C(14A)-D(14E)	109.5
N(1A)-C(14A)-D(14F)	109.5
D(14D)-C(14A)-D(14F)	109.5
D(14E)-C(14A)-D(14F)	109.5
O(3)-S(1)-O(2)	117.02(11)
O(3)-S(1)-O(1)	112.68(10)
O(2)-S(1)-O(1)	113.22(10)
O(3)-S(1)-C(15)	105.99(11)
O(2)-S(1)-C(15)	105.36(11)
O(1)-S(1)-C(15)	100.56(11)
S(1)-O(1)-Fe(1)	138.02(10)
F(3)-C(15)-F(2)	108.4(2)
F(3)-C(15)-F(1)	108.8(2)
F(2)-C(15)-F(1)	108.6(2)
F(3)-C(15)-S(1)	110.51(17)
F(2)-C(15)-S(1)	111.04(16)
F(1)-C(15)-S(1)	109.49(18)
C(22)-C(21)-C(26)	115.77(18)
C(22)-C(21)-B(21)	121.34(18)
C(26)-C(21)-B(21)	122.69(18)
C(23)-C(22)-C(21)	122.53(19)
C(23)-C(22)-H(22)	118.7
C(21)-C(22)-H(22)	118.7
C(24)-C(23)-C(22)	120.72(19)
C(24)-C(23)-C(27)	120.61(19)
C(22)-C(23)-C(27)	118.61(19)
C(23)-C(24)-C(25)	117.92(19)
C(23)-C(24)-H(24)	121.0
C(25)-C(24)-H(24)	121.0
C(26)-C(25)-C(24)	121.26(19)
C(26)-C(25)-C(28)	120.99(19)
C(24)-C(25)-C(28)	117.75(18)
C(25)-C(26)-C(21)	121.78(19)
C(25)-C(26)-H(26)	119.1
C(21)-C(26)-H(26)	119.1
F(23)-C(27)-F(22)	107.08(19)
F(23)-C(27)-F(21)	106.3(2)
F(22)-C(27)-F(21)	105.7(2)
F(23)-C(27)-C(23)	113.50(19)
F(22)-C(27)-C(23)	112.76(19)
F(21)-C(27)-C(23)	110.94(19)
F(25A)-C(28)-F(24A)	106.8(11)
F(24)-C(28)-F(26)	107.0(5)
F(25A)-C(28)-F(26A)	103.6(8)
F(24A)-C(28)-F(26A)	106.0(10)
F(24)-C(28)-F(25)	106.4(5)
F(26)-C(28)-F(25)	105.6(4)
F(25A)-C(28)-C(25)	115.1(8)
F(24)-C(28)-C(25)	113.2(5)
F(24A)-C(28)-C(25)	114.2(11)
F(26)-C(28)-C(25)	113.5(4)
F(26A)-C(28)-C(25)	110.1(8)
F(25)-C(28)-C(25)	110.5(4)
C(32)-C(31)-C(36)	115.69(18)
C(32)-C(31)-B(21)	125.06(18)
C(36)-C(31)-B(21)	118.69(18)
C(33)-C(32)-C(31)	121.86(19)
C(33)-C(32)-H(32)	119.1
C(31)-C(32)-H(32)	119.1
C(34)-C(33)-C(32)	121.47(19)
C(34)-C(33)-C(37)	119.04(19)
C(32)-C(33)-C(37)	119.44(19)
C(33)-C(34)-C(35)	117.59(19)
C(33)-C(34)-H(34)	121.2
C(35)-C(34)-H(34)	121.2
C(34)-C(35)-C(36)	120.99(19)
C(34)-C(35)-C(38)	119.89(19)
C(36)-C(35)-C(38)	119.12(19)
C(35)-C(36)-C(31)	122.39(19)
C(35)-C(36)-H(36)	118.8
C(31)-C(36)-H(36)	118.8
F(32A)-C(37)-F(31A)	110.2(19)
F(31)-C(37)-F(33)	106.6(2)
F(31)-C(37)-F(32)	106.6(2)
F(33)-C(37)-F(32)	105.4(2)
F(32A)-C(37)-F(33A)	103.7(17)
F(31A)-C(37)-F(33A)	104.9(18)
F(32A)-C(37)-C(33)	115.1(15)
F(31)-C(37)-C(33)	113.59(19)
F(31A)-C(37)-C(33)	112.6(14)
F(33)-C(37)-C(33)	113.18(19)
F(32)-C(37)-C(33)	111.0(2)
F(33A)-C(37)-C(33)	109.4(15)
F(36)-C(38)-F(34)	107.6(2)
F(36)-C(38)-F(35)	107.9(2)
F(34)-C(38)-F(35)	104.1(2)
F(34A)-C(38)-F(35A)	109.2(15)
F(34A)-C(38)-F(36A)	105.3(15)
F(35A)-C(38)-F(36A)	100.5(13)
F(34A)-C(38)-C(35)	121.8(12)
F(36)-C(38)-C(35)	112.2(2)
F(34)-C(38)-C(35)	113.08(19)
F(35)-C(38)-C(35)	111.43(19)
F(35A)-C(38)-C(35)	111.8(9)
F(36A)-C(38)-C(35)	105.8(9)
C(46)-C(41)-C(42)	115.67(18)
C(46)-C(41)-B(21)	122.52(18)
C(42)-C(41)-B(21)	121.53(18)
C(43)-C(42)-C(41)	122.14(19)
C(43)-C(42)-H(42)	118.9
C(41)-C(42)-H(42)	118.9
C(42)-C(43)-C(44)	120.93(19)
C(42)-C(43)-C(47)	121.03(19)
C(44)-C(43)-C(47)	118.04(19)
C(45)-C(44)-C(43)	117.97(19)
C(45)-C(44)-H(44)	121.0
C(43)-C(44)-H(44)	121.0
C(44)-C(45)-C(46)	120.84(19)
C(44)-C(45)-C(48)	119.56(19)
C(46)-C(45)-C(48)	119.55(19)
C(45)-C(46)-C(41)	122.43(19)
C(45)-C(46)-H(46)	118.8
C(41)-C(46)-H(46)	118.8
F(41)-C(47)-F(42)	108.9(3)
F(42A)-C(47)-F(43A)	110.8(15)
F(41)-C(47)-F(43)	106.2(3)
F(42)-C(47)-F(43)	103.41(19)
F(42A)-C(47)-F(41A)	107.6(10)
F(43A)-C(47)-F(41A)	95.5(13)
F(42A)-C(47)-C(43)	121.5(9)
F(41)-C(47)-C(43)	114.5(3)
F(42)-C(47)-C(43)	112.47(18)
F(43A)-C(47)-C(43)	110.9(14)
F(43)-C(47)-C(43)	110.6(2)
F(41A)-C(47)-C(43)	107.2(6)
F(45)-C(48)-F(46)	106.2(2)
F(45)-C(48)-F(44)	106.3(2)

F(46)-C(48)-F(44)	107.1(2)	F(51)-C(57)-C(53)	113.4(2)
F(45)-C(48)-C(45)	113.1(2)	F(53)-C(57)-C(53)	113.1(2)
F(46)-C(48)-C(45)	111.99(19)	F(56A)-C(58)-F(55A)	110.0(4)
F(44)-C(48)-C(45)	111.8(2)	F(56A)-C(58)-F(54A)	112.9(4)
C(56)-C(51)-C(52)	115.62(19)	F(55A)-C(58)-F(54A)	85.4(4)
C(56)-C(51)-B(21)	124.02(18)	F(54)-C(58)-F(55)	130.0(3)
C(52)-C(51)-B(21)	120.30(18)	F(56B)-C(58)-F(55B)	112.3(5)
C(53)-C(52)-C(51)	122.6(2)	F(56B)-C(58)-F(54B)	104.9(5)
C(53)-C(52)-H(52)	118.7	F(55B)-C(58)-F(54B)	97.0(5)
C(51)-C(52)-H(52)	118.7	F(54)-C(58)-F(56)	100.0(3)
C(52)-C(53)-C(54)	120.7(2)	F(55)-C(58)-F(56)	87.4(3)
C(52)-C(53)-C(57)	120.7(2)	F(56B)-C(58)-C(55)	120.2(4)
C(54)-C(53)-C(57)	118.5(2)	F(56A)-C(58)-C(55)	115.3(3)
C(55)-C(54)-C(53)	117.8(2)	F(54)-C(58)-C(55)	115.5(3)
C(55)-C(54)-H(54)	121.1	F(55A)-C(58)-C(55)	117.9(3)
C(53)-C(54)-H(54)	121.1	F(54A)-C(58)-C(55)	111.8(3)
C(54)-C(55)-C(56)	121.2(2)	F(55)-C(58)-C(55)	108.5(3)
C(54)-C(55)-C(58)	119.2(2)	F(55B)-C(58)-C(55)	110.4(3)
C(56)-C(55)-C(58)	119.6(2)	F(54B)-C(58)-C(55)	109.2(3)
C(51)-C(56)-C(55)	122.1(2)	F(56)-C(58)-C(55)	108.0(2)
C(51)-C(56)-H(56)	118.9	C(21)-B(21)-C(31)	114.71(17)
C(55)-C(56)-H(56)	118.9	C(21)-B(21)-C(51)	105.25(16)
F(52)-C(57)-F(51)	105.6(2)	C(31)-B(21)-C(51)	112.54(17)
F(52)-C(57)-F(53)	106.1(2)	C(21)-B(21)-C(41)	110.82(17)
F(51)-C(57)-F(53)	106.06(19)	C(31)-B(21)-C(41)	103.03(16)
F(52)-C(57)-C(53)	111.95(19)	C(51)-B(21)-C(41)	110.62(16)

Table S9. Anisotropic displacement parameters (Å^{2}x 10^3) for **1b-B$_{ArF}$F**. The anisotropic displacement factor exponent takes the form: -2p^2[h^2 a*^{2}U^{11} + ... + 2 h k a* b* U^{12}]

	U^{11}	U^{22}	U^{33}	U^{23}	U^{13}	U^{12}
Fe(1)	16(1)	15(1)	14(1)	0(1)	0(1)	2(1)
N(1)	33(1)	25(1)	22(1)	-3(1)	2(1)	10(1)
N(2)	19(1)	28(1)	18(1)	4(1)	1(1)	-1(1)
N(3)	19(1)	23(1)	17(1)	3(1)	5(1)	4(1)
N(4)	26(1)	19(1)	31(1)	3(1)	-6(1)	-5(1)
C(1)	38(1)	39(2)	24(1)	1(1)	7(1)	21(1)
C(2)	24(1)	53(2)	26(1)	5(1)	7(1)	14(1)
C(3)	17(1)	42(1)	25(1)	6(1)	1(1)	3(1)
C(4)	26(1)	37(1)	29(1)	10(1)	1(1)	-9(1)
C(5)	26(1)	28(1)	23(1)	-2(1)	-3(1)	-3(1)
C(6)	27(1)	32(1)	15(1)	0(1)	1(1)	4(1)
C(7)	28(1)	28(1)	22(1)	2(1)	9(1)	10(1)
C(8)	28(1)	27(1)	24(1)	10(1)	8(1)	5(1)
C(9)	26(1)	33(1)	41(2)	18(1)	7(1)	-1(1)
C(10)	29(1)	22(1)	42(2)	12(1)	-7(1)	-7(1)
C(11)	22(1)	30(1)	45(2)	10(1)	-11(1)	-9(1)
C(12)	47(2)	23(1)	36(2)	-6(1)	-9(1)	-3(1)
C(13)	53(2)	21(1)	35(1)	-10(1)	-2(1)	7(1)
C(14)	45(2)	38(1)	18(1)	-1(1)	6(1)	19(1)
N(1A)	35(7)	20(6)	34(5)	1(6)	-1(5)	-3(6)
N(2A)	30(7)	36(7)	25(8)	0(7)	5(6)	9(6)
N(3A)	21(7)	33(6)	19(7)	0(6)	3(6)	-7(5)
N(4A)	20(6)	28(5)	24(6)	8(6)	8(5)	4(4)
C(1A)	43(8)	20(8)	31(10)	-4(8)	1(8)	1(7)
C(2A)	42(8)	30(8)	27(9)	-1(9)	0(8)	9(7)
C(3A)	33(8)	35(7)	26(9)	0(8)	3(8)	11(6)
C(4A)	40(20)	40(20)	26(10)	-1(11)	8(11)	10(19)
C(5A)	25(7)	40(7)	24(8)	4(7)	6(7)	4(6)
C(6A)	22(8)	36(7)	21(9)	7(8)	4(8)	-4(6)
C(7A)	26(1)	37(1)	29(1)	10(1)	1(1)	-9(1)
C(8A)	24(8)	28(9)	19(7)	-3(7)	1(6)	2(7)
C(9A)	26(8)	29(8)	20(8)	3(8)	2(7)	6(7)
C(10A)	24(7)	26(8)	20(7)	4(8)	8(7)	9(7)
C(11A)	28(1)	28(1)	22(1)	2(1)	9(1)	10(1)
C(12A)	26(1)	33(1)	41(2)	18(1)	7(1)	-1(1)
C(13A)	29(7)	23(8)	43(6)	12(7)	0(6)	-4(7)
C(14A)	47(2)	23(1)	36(2)	-6(1)	-9(1)	-3(1)
S(1)	21(1)	20(1)	17(1)	3(1)	-1(1)	2(1)
F(1)	51(1)	33(1)	42(1)	7(1)	-19(1)	14(1)
F(2)	36(1)	32(1)	39(1)	-9(1)	-2(1)	8(1)
F(3)	21(1)	43(1)	78(1)	6(1)	9(1)	3(1)
O(1)	25(1)	23(1)	18(1)	6(1)	1(1)	4(1)
O(2)	38(1)	25(1)	25(1)	-2(1)	-5(1)	2(1)
O(3)	30(1)	34(1)	25(1)	6(1)	7(1)	-4(1)
C(15)	24(1)	25(1)	33(1)	2(1)	-5(1)	3(1)
F(21)	87(1)	20(1)	45(1)	-4(1)	22(1)	-13(1)
F(22)	23(1)	29(1)	56(1)	16(1)	6(1)	-7(1)
F(23)	35(1)	32(1)	53(1)	27(1)	-13(1)	-8(1)
F(24)	21(2)	25(2)	52(3)	15(2)	-9(2)	-9(1)
F(25)	16(2)	42(2)	79(5)	-16(3)	1(2)	2(1)
F(26)	40(3)	69(4)	28(2)	15(2)	-17(2)	-22(3)
F(24A)	24(5)	49(6)	70(8)	27(6)	-8(5)	-13(5)
F(25A)	18(3)	28(3)	82(9)	-2(5)	-13(5)	4(2)
F(26A)	40(5)	74(9)	40(4)	-22(4)	-19(3)	-7(5)
F(31)	27(1)	27(1)	52(1)	7(1)	19(1)	10(1)
F(32)	18(1)	57(1)	79(2)	-21(1)	12(1)	-12(1)
F(33)	41(1)	62(1)	40(1)	22(1)	27(1)	20(1)
F(31A)	19(12)	63(16)	46(11)	28(12)	23(10)	17(12)
F(32A)	41(1)	62(1)	40(1)	22(1)	27(1)	20(1)
F(33A)	41(1)	62(1)	40(1)	22(1)	27(1)	20(1)
F(34)	33(1)	38(1)	25(1)	14(1)	4(1)	10(1)
F(35)	20(1)	62(1)	63(1)	36(1)	8(1)	12(1)
F(36)	90(2)	28(1)	40(1)	-7(1)	-12(1)	31(1)
F(34A)	61(14)	66(14)	36(6)	13(9)	-2(7)	16(11)
F(35A)	33(1)	38(1)	25(1)	14(1)	4(1)	10(1)
F(36A)	42(10)	21(7)	61(14)	11(8)	36(9)	16(6)
F(41)	31(1)	36(2)	40(2)	-18(2)	-12(1)	19(1)
F(42)	51(1)	22(1)	48(1)	-14(1)	26(1)	-12(1)
F(43)	31(1)	37(1)	18(1)	-7(1)	1(1)	2(1)
F(41A)	51(1)	22(1)	48(1)	-14(1)	26(1)	-12(1)
F(42A)	17(7)	47(10)	46(6)	-38(6)	-8(6)	5(7)
F(43A)	21(5)	33(11)	11(7)	-2(7)	4(5)	4(5)
F(44)	22(1)	82(1)	49(1)	-5(1)	12(1)	3(1)
F(45)	23(1)	39(1)	47(1)	-12(1)	-14(1)	6(1)
F(46)	24(1)	28(1)	124(2)	-34(1)	-17(1)	10(1)
F(51)	44(1)	36(1)	36(1)	-4(1)	20(1)	-17(1)
F(52)	21(1)	75(1)	77(1)	43(1)	17(1)	11(1)
F(53)	52(1)	53(1)	21(1)	-2(1)	16(1)	-16(1)
F(54)	43(1)	27(1)	45(1)	-16(1)	9(1)	-12(1)
F(55)	43(1)	27(1)	45(1)	-16(1)	9(1)	-12(1)
F(56)	43(1)	27(1)	45(1)	-16(1)	9(1)	-12(1)
F(54A)	43(1)	27(1)	45(1)	-16(1)	9(1)	-12(1)
F(55A)	43(1)	27(1)	45(1)	-16(1)	9(1)	-12(1)
F(56A)	43(1)	27(1)	45(1)	-16(1)	9(1)	-12(1)
F(54B)	43(1)	27(1)	45(1)	-16(1)	9(1)	-12(1)
F(55B)	43(1)	27(1)	45(1)	-16(1)	9(1)	-12(1)
F(56B)	43(1)	27(1)	45(1)	-16(1)	9(1)	-12(1)
C(21)	15(1)	15(1)	15(1)	0(1)	2(1)	1(1)
C(22)	16(1)	18(1)	18(1)	1(1)	3(1)	-2(1)
C(23)	18(1)	16(1)	20(1)	2(1)	3(1)	0(1)

C(24)	18(1)	18(1)	17(1)	3(1)	1(1)	3(1)
C(25)	17(1)	19(1)	16(1)	0(1)	0(1)	-2(1)
C(26)	19(1)	15(1)	17(1)	0(1)	0(1)	0(1)
C(27)	22(1)	20(1)	26(1)	6(1)	-1(1)	-1(1)
C(28)	19(1)	20(1)	28(1)	3(1)	-5(1)	-2(1)
C(31)	15(1)	14(1)	16(1)	-1(1)	0(1)	-2(1)
C(32)	14(1)	14(1)	18(1)	0(1)	0(1)	0(1)
C(33)	15(1)	17(1)	20(1)	-1(1)	4(1)	-1(1)
C(34)	19(1)	17(1)	18(1)	2(1)	4(1)	-2(1)
C(35)	17(1)	16(1)	22(1)	1(1)	3(1)	2(1)
C(36)	15(1)	16(1)	19(1)	1(1)	4(1)	0(1)
C(37)	19(1)	26(1)	32(1)	5(1)	9(1)	2(1)
C(38)	26(1)	25(1)	27(1)	9(1)	11(1)	7(1)
C(41)	15(1)	15(1)	15(1)	3(1)	2(1)	-2(1)
C(42)	16(1)	18(1)	15(1)	2(1)	2(1)	1(1)
C(43)	20(1)	17(1)	14(1)	1(1)	4(1)	-1(1)
C(44)	18(1)	17(1)	19(1)	1(1)	0(1)	-1(1)
C(45)	15(1)	18(1)	23(1)	0(1)	-1(1)	0(1)
C(46)	18(1)	14(1)	19(1)	-1(1)	2(1)	-1(1)
C(47)	21(1)	22(1)	24(1)	-5(1)	6(1)	-3(1)
C(48)	18(1)	24(1)	38(1)	-12(1)	-4(1)	1(1)
C(51)	14(1)	17(1)	15(1)	1(1)	0(1)	1(1)
C(52)	18(1)	18(1)	16(1)	2(1)	-1(1)	0(1)
C(53)	16(1)	25(1)	16(1)	3(1)	1(1)	0(1)
C(54)	24(1)	26(1)	17(1)	-3(1)	4(1)	2(1)
C(55)	23(1)	20(1)	21(1)	-4(1)	1(1)	-2(1)
C(56)	16(1)	19(1)	17(1)	1(1)	2(1)	-3(1)
C(57)	24(1)	32(1)	21(1)	5(1)	6(1)	0(1)
C(58)	39(1)	24(1)	36(1)	-10(1)	10(1)	-6(1)
B(21)	14(1)	14(1)	16(1)	2(1)	1(1)	0(1)

Table S10. Hydrogen coordinates (x 10^4) and isotropic displacement parameters (Å^{2}x 10^3) for **1b-B$_{Ar}$F**.

	x	y	z	U(eq)
H(1A)	2535	847	1887	40
H(1AB)	2162	888	2734	40
H(2A)	3037	1766	1982	41
H(2AB)	3781	1385	2559	41
H(3A)	2702	1656	3565	34
H(3AB)	3477	2106	3279	34
H(5A)	1179	2822	3601	31
H(5AB)	2357	2623	3992	31
H(6A)	1597	1826	4373	30
H(6AB)	928	2311	4688	30
H(8A)	-375	1523	4734	31
H(8AB)	382	1176	4240	31
H(9A)	-1866	1397	3734	40
H(9AB)	-1471	866	4184	40
H(10A)	-443	656	3122	38
H(10B)	-1747	620	2933	38
H(12A)	-991	1130	1236	44
H(12B)	-1251	592	1669	44
H(13A)	571	530	2235	44
H(13B)	567	588	1319	44
H(1AA)	411	562	3003	38
H(1AC)	-351	297	2296	38
H(2AA)	897	655	1447	40
H(2AC)	1393	186	2000	40
H(3AA)	2177	897	2874	38
H(3AC)	2770	774	2124	38
H(5AA)	3529	1768	2343	35
H(5AC)	2916	1639	3087	35
H(6AA)	3066	2583	2860	31
H(6AC)	2283	2508	2061	31
H(8AA)	2268	2758	3977	28
H(8AC)	2450	2130	3975	28
H(9AA)	1391	2377	4946	30
H(9AC)	501	2644	4322	30
H(10C)	915	1535	4335	28
H(10D)	17	1791	4816	28
H(12C)	-1519	1216	3973	40
H(12D)	-345	976	3841	40
H(13C)	-1669	720	2804	38
H(13D)	-1964	1331	2620	38
H(22)	5008	3451	3128	21
H(24)	7986	3204	4349	21
H(26)	7327	4596	3211	21
H(32)	7060	4486	1849	19
H(34)	6421	5650	243	22
H(36)	4275	5347	1791	20
H(42)	5141	3583	1672	19
H(44)	1956	3395	779	22
H(46)	2836	4595	2277	20
H(52)	3617	4249	3610	21
H(54)	3406	5596	4861	26
H(56)	5410	5533	3167	21

[(D_{12}-TMC)Fe^{IV}(O)(OTf)]OTf, **1b′-OTf**

The structure of **1b′-OTf** was solved in the orthorhombic space group $P2_12_12$ as pseudo-merohedral twin with additional inversion twinning (twin law 0 1 0 1 0 0 0 0 -1) . The twin ratios were refined and converged to 0.14(2), 0.39(5) and 0.15(2). The asymmetric unit contains four complexes, four non-coordinated anions and four solvent molecules, two of them were found to be disordered over two positions each. The overall structure and the disorder was refined with the help of similarity restraints on anisotropic displacement parameters, same distance restraints, advanced rigid bond restraints and by setting some atoms to have the same anisotropic displacement parameters. The disorder ratio was refined and converged to 0.870(7) and 0.85(1).

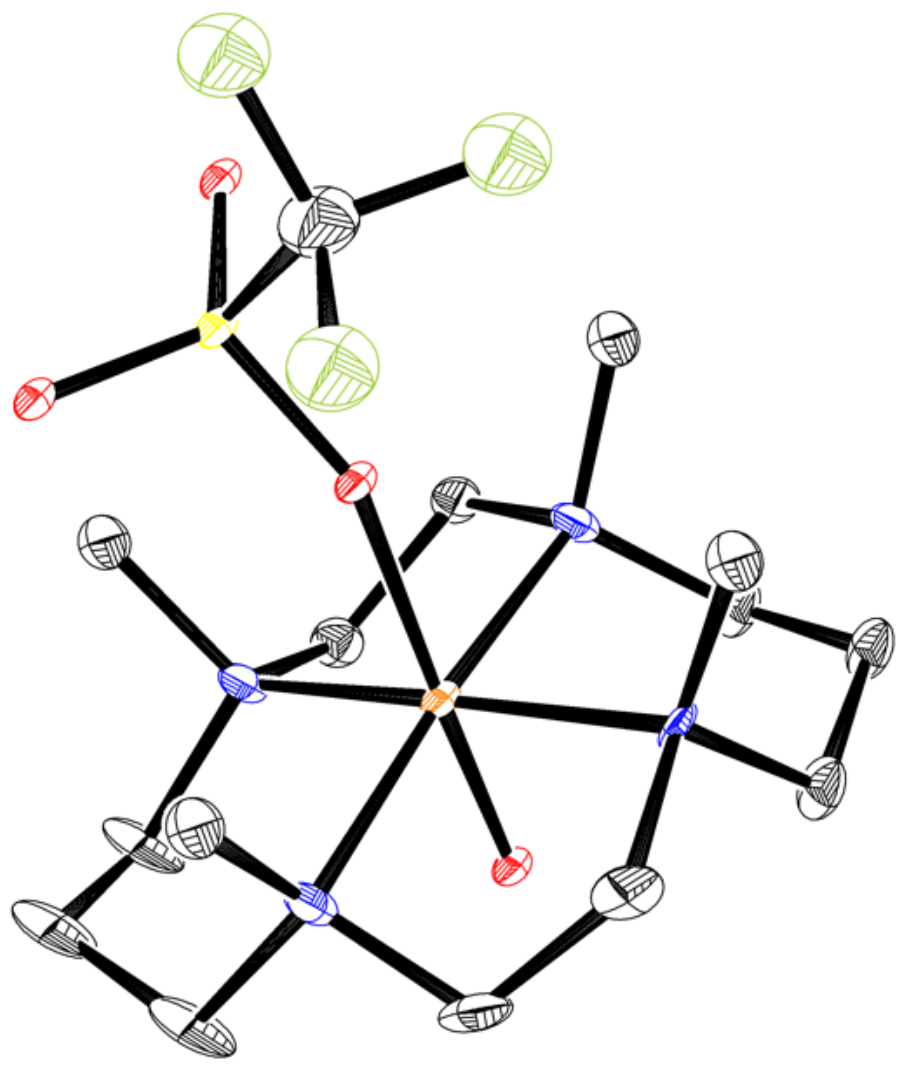

Figure S3. Molecular Structure of **1b′-OTf**.

Table S11. Crystal data and structure refinement for **1b′-OTf**.

CCDC No	2122182	
Empirical formula	$C_{17}H_{22}Cl_2D_{12}F_6FeN_4O_7S_2$	
Formula weight	723.42	
Temperature	100(2) K	
Wavelength	0.71073 Å	
Crystal system	Orthorhombic	
Space group	$P2_12_12$	
Unit cell dimensions	a = 34.6756(11) Å	α = 90°
	b = 34.7151(11) Å	β = 90°
	c = 9.0861(3) Å	γ = 90°
Volume	10937.5(6) $Å^3$	
Z	16	
Density (calculated)	1.757 Mg/m^3	
Absorption coefficient	0.986 mm^{-1}	
F(000)	5856	
Crystal size	0.341 x 0.301 x 0.250 mm^3	
Theta range for data collection	2.241 to 27.103°.	
Index ranges	-44 ≤ h ≤ 44, -44 ≤ k ≤ 44, -11 ≤ l ≤ 10	
Reflections collected	139766	
Independent reflections	24145 [R(int) = 0.1101]	
Completeness to theta = 25.242°	99.9 %	
Absorption correction	Semi-empirical from equivalents	
Refinement method	Full-matrix least-squares on F^2	
Data / restraints / parameters	24145 / 3008 / 1027	
Goodness-of-fit on F^2	1.009	
Final R indices [I>2σ(I)]	R1 = 0.0496, wR2 = 0.0903	
R indices (all data)	R1 = 0.0822, wR2 = 0.1023	
Largest diff. peak and hole	0.669 and -0.614 e$Å^{-3}$	

Table S12. Atomic coordinates (x 10^4) and equivalent isotropic displacement parameters (Å^2 x 10^3) for **1b′-OTf**.
U(eq) is defined as one third of the trace of the orthogonalized U^{ij} tensor.

	x	y	z	U(eq)
Fe11	8727(1)	6357(1)	5917(3)	12(1)
S11	8385(1)	7065(1)	8235(6)	12(1)
F11	7866(3)	6674(3)	9729(15)	34(1)
F21	7653(3)	6963(3)	7746(12)	34(1)
F31	7783(3)	7270(3)	9824(12)	34(1)
O11	8939(3)	6065(3)	4749(15)	12(1)
O21	8441(4)	6701(3)	7347(15)	12(1)
O31	8614(3)	7084(3)	9523(15)	12(1)
O41	8350(4)	7396(3)	7335(14)	12(1)
N11	8996(5)	6074(4)	7715(18)	15(1)
N21	8232(4)	5982(4)	6007(18)	10(2)
N31	8467(4)	6597(4)	4052(16)	15(1)
N41	9224(4)	6682(4)	5801(17)	15(1)
C11	9026(5)	5653(4)	7360(20)	18(2)
C21	8641(5)	5464(5)	7120(20)	18(2)
C31	8381(4)	5600(4)	5834(18)	18(2)
C41	7963(5)	6021(4)	7241(18)	19(2)
D4A1	7887	6292	7345	28
D4B1	8089	5935	8149	28
D4C1	7734	5864	7057	28
C51	7995(4)	6086(5)	4626(18)	18(3)
C61	8252(5)	6266(5)	3500(19)	20(3)
C71	8202(4)	6919(4)	4400(20)	19(2)
D7A1	8343	7119	4942	28
D7B1	7989	6823	5012	28
D7C1	8099	7027	3489	28
C81	8731(5)	6728(6)	2900(20)	32(2)
C91	9027(5)	7009(5)	3430(20)	32(2)
C101	9345(6)	6800(6)	4281(18)	32(2)
C111	9227(5)	7056(4)	6690(20)	19(2)
D11A1	9487	7166	6683	28
D11B1	9152	7001	7711	28
D11C1	9045	7239	6260	28
C121	9529(5)	6425(5)	6416(18)	17(3)
C131	9383(4)	6256(5)	7835(18)	16(3)
C141	8816(5)	6126(5)	9210(20)	19(2)
D14A1	8829	6398	9488	28
D14B1	8955	5970	9930	28
D14C1	8546	6044	9169	28
C151	7921(5)	6996(6)	8920(20)	29(2)
Fe12	8676(1)	3754(1)	4801(3)	7(1)
S12	7974(1)	3424(1)	2430(6)	11(1)
F12	8395(3)	2969(3)	827(13)	23(1)
F22	8106(3)	2674(3)	2569(10)	23(1)
F32	7790(2)	2864(2)	622(9)	23(1)
O12	8966(3)	3966(3)	5952(14)	7(1)
O22	8317(3)	3480(4)	3327(16)	13(1)
O32	7931(2)	3669(3)	1207(13)	13(1)
O42	7628(3)	3342(2)	3299(11)	13(1)
N12	8951(4)	4027(4)	3013(17)	10(1)
N22	9045(4)	3275(4)	4710(20)	10(1)
N32	8433(4)	3498(4)	6600(18)	10(1)
N42	8337(4)	4251(4)	4909(19)	10(1)
C12	9369(4)	4066(5)	3380(20)	13(2)
C22	9572(4)	3697(5)	3511(19)	13(2)
C32	9452(4)	3424(5)	4840(20)	13(2)
C42	9035(5)	3001(5)	3455(19)	15(2)
D4A2	9099	3136	2543	22
D4B2	8776	2890	3372	22
D4C2	9222	2794	3625	22
C52	8963(4)	3043(5)	6049(18)	13(2)
C62	8765(4)	3268(5)	7235(19)	13(2)
C72	8117(4)	3214(4)	6220(20)	10(2)
D7A2	7896	3352	5805	15
D7B2	8036	3076	7109	15
D7C2	8213	3028	5493	15
C82	8289(4)	3759(4)	7800(19)	17(2)
C92	8031(4)	4083(4)	7264(18)	17(2)
C102	8230(5)	4370(5)	6497(16)	17(2)
C112	7970(4)	4250(5)	4093(17)	15(2)
D11A2	8020	4201	3049	22
D11B2	7843	4501	4202	22
D11C2	7801	4048	4486	22
C122	8605(4)	4555(5)	4329(18)	15(3)
C132	8769(5)	4424(4)	2898(19)	15(3)
C142	8917(4)	3873(3)	1489(16)	10(2)
D14A2	9052	4045	805	15
D14B2	8644	3857	1215	15
D14C2	9033	3616	1445	15
C152	8063(5)	2962(5)	1600(20)	19(2)
Fe13	6328(1)	3757(1)	9802(3)	7(1)
S13	7035(1)	3425(1)	7438(6)	12(1)
F13	6621(3)	2946(3)	5796(13)	23(1)
F23	7244(2)	2847(2)	5858(9)	23(1)
F33	6899(3)	2694(3)	7733(10)	23(1)
O13	6034(4)	3979(4)	10965(16)	14(1)
O23	6678(3)	3479(3)	8338(16)	12(1)
O33	7032(2)	3695(3)	6183(13)	12(1)
O43	7378(3)	3401(2)	8251(11)	12(1)
N13	6068(4)	4022(4)	7968(18)	12(1)
N23	5954(4)	3276(4)	9720(20)	11(2)

N33	6560(4)	3475(4)	11590(18)	11(2)
N43	6651(4)	4257(4)	9920(20)	12(1)
C13	5644(4)	4066(5)	8320(20)	15(2)
C23	5423(4)	3678(5)	8620(20)	15(2)
C33	5547(4)	3429(4)	9820(20)	11(2)
C43	5984(5)	3013(5)	8443(19)	14(2)
D4A3	6245	2905	8399	21
D4B3	5931	3157	7537	21
D4C3	5797	2804	8549	21
C53	6046(5)	3025(4)	11051(17)	10(2)
C63	6226(4)	3257(4)	12240(18)	10(2)
C73	6887(4)	3220(5)	11230(20)	14(2)
D7A3	6812	3044	10431	21
D7B3	6960	3070	12096	21
D7C3	7107	3376	10906	21
C83	6687(4)	3732(4)	12797(18)	11(2)
C93	6968(4)	4047(3)	12364(18)	11(2)
C103	6751(4)	4372(4)	11409(16)	11(2)
C113	7012(4)	4258(5)	8980(17)	14(2)
D11A3	6948	4172	7982	21
D11B3	7203	4083	9408	21
D11C3	7118	4519	8940	21
C123	6385(4)	4546(5)	9294(18)	16(3)
C133	6234(5)	4412(4)	7837(19)	16(3)
C143	6100(4)	3832(4)	6499(18)	14(2)
D14A3	5959	3587	6514	21
D14B3	6372	3783	6276	21
D14C3	5990	4000	5743	21
C153	6936(6)	2952(5)	6620(30)	20(2)
Fe14	6272(1)	6354(1)	10912(3)	8(1)
S14	6616(1)	7057(1)	13233(6)	14(1)
F14	7204(3)	7300(3)	14682(11)	24(1)
F24	7357(3)	6933(3)	12899(10)	24(1)
F34	7116(3)	6675(3)	14802(13)	24(1)
O14	6048(4)	6063(4)	9756(17)	21(2)
O24	6555(3)	6717(3)	12385(15)	11(1)
O34	6635(4)	7406(4)	12391(17)	27(2)
O44	6383(4)	7076(4)	14563(17)	25(2)
N14	6747(4)	5990(4)	10957(18)	13(1)
N24	6009(4)	6093(4)	12692(18)	10(2)
N34	5767(4)	6689(4)	10852(18)	13(1)
N44	6540(4)	6589(4)	9154(16)	13(1)
C14	6599(4)	5562(3)	10928(17)	14(1)
C24	6347(5)	5450(5)	12180(20)	14(1)
C34	5966(5)	5677(4)	12370(20)	14(1)
C44	6186(5)	6130(5)	14180(20)	16(2)
D4A4	6445	6017	14173	24
D4B4	6026	5994	14905	24
D4C4	6204	6403	14450	24
C54	5599(4)	6249(5)	12840(20)	19(3)
C64	5478(4)	6404(5)	11391(19)	19(3)
C74	5765(5)	7032(4)	11764(19)	15(2)
D7A4	5964	7210	11421	22
D7B4	5816	6960	12789	22
D7C4	5512	7157	11699	22
C84	5652(4)	6787(4)	9318(16)	14(1)
C94	5946(4)	6996(4)	8433(19)	14(1)
C104	6276(4)	6732(4)	7930(20)	14(1)
C114	6818(4)	6909(4)	9490(19)	15(2)
D11A4	6970	6968	8609	22
D11B4	6991	6829	10287	22
D11C4	6674	7139	9793	22
C124	6771(5)	6263(4)	8432(19)	17(2)
C134	6986(5)	6060(5)	9630(18)	17(2)
C144	7033(5)	5984(4)	12213(17)	15(2)
D14A4	7137	6244	12362	22
D14B4	7244	5807	11978	22
D14C4	6904	5898	13113	22
C154	7119(4)	6986(5)	13960(20)	14(1)
S15	8166(1)	5329(1)	11275(6)	13(1)
F15	7926(3)	4633(3)	10606(14)	32(3)
F25	7886(3)	5038(3)	8846(14)	26(3)
F35	7478(2)	5069(4)	10586(14)	30(3)
O15	8040(2)	5249(3)	12794(13)	23(1)
O25	8539(3)	5224(3)	10962(9)	23(1)
O35	8032(4)	5702(4)	10800(15)	23(1)
C15	7848(6)	4997(6)	10290(20)	16(3)
S16	6840(1)	5328(1)	6271(6)	20(1)
F16	7083(3)	4633(3)	5634(14)	28(3)
F26	7518(2)	5061(4)	5600(15)	36(3)
F36	7111(3)	5026(4)	3808(14)	27(3)
O16	6895(2)	5243(3)	7779(13)	23(1)
O26	6452(3)	5209(3)	5652(9)	23(1)
O36	6958(4)	5695(4)	5713(15)	23(1)
C16	7147(5)	4997(6)	5270(20)	16(3)
S17	4700(1)	6860(2)	4415(6)	17(1)
F17	5008(4)	7073(4)	6970(13)	28(3)
F27	5041(4)	7499(2)	5203(17)	34(3)
F37	5417(3)	6984(3)	5184(16)	34(3)
O17	4818(2)	6463(3)	4726(12)	24(1)
O27	4347(3)	7036(3)	4978(14)	24(1)
O37	4794(3)	7000(3)	2939(14)	24(1)
C17	5071(5)	7132(5)	5510(20)	14(3)
S18	10298(1)	6857(2)	-595(6)	16(1)
F18	10002(4)	7074(3)	1898(13)	21(2)

F28	9578(3)	7017(3)	223(15)	28(3)
F38	9936(3)	7490(2)	215(16)	28(3)
O18	10256(2)	6468(3)	-178(11)	22(1)
O28	10208(3)	6937(3)	-2091(13)	22(1)
O38	10659(3)	6980(3)	-44(14)	22(1)
C18	9948(5)	7111(5)	460(20)	13(2)
Cl19	4243(2)	5304(2)	13993(8)	42(2)
Cl29	4843(2)	5512(2)	11845(8)	32(1)
C19	4540(4)	5667(5)	13265(18)	23(3)
Cl110	5340(2)	5793(2)	6611(8)	41(2)
Cl210	5491(2)	5195(2)	8871(8)	44(2)
C110	5690(5)	5472(5)	7410(30)	27(3)
Cl111	9656(2)	5801(2)	1675(9)	34(2)
Cl211	9492(2)	5210(2)	3891(8)	35(2)
C111	9330(6)	5488(7)	2440(30)	34(2)
Cl1A11	9707(7)	5654(8)	1290(30)	34(2)
Cl2A11	9618(8)	5167(8)	3500(30)	35(2)
C1A11	9444(17)	5276(18)	1820(60)	34(2)
Cl112	9232(2)	4693(2)	8952(8)	32(1)
Cl212	9836(2)	4485(2)	6808(9)	35(2)
Cl1A12	9473(7)	4752(5)	9440(20)	32(1)
Cl2A12	9905(9)	4550(11)	7150(30)	35(2)
C112	9557(5)	4335(5)	8340(19)	36(4)

Table S13. Bond lengths [Å] and angles [°] for **1b′-OTf**.

Fe11-O11	1.640(12)	C22-H2AB2	0.9900
Fe11-O21	2.025(12)	C32-H3A2	0.9900
Fe11-N41	2.064(15)	C32-H3AB2	0.9900
Fe11-N31	2.093(14)	C42-D4A2	0.9800
Fe11-N11	2.123(16)	C42-D4B2	0.9800
Fe11-N21	2.156(13)	C42-D4C2	0.9800
S11-O31	1.417(13)	C52-C62	1.496(13)
S11-O41	1.417(12)	C52-H5A2	0.9900
S11-O21	1.512(12)	C52-H5AB2	0.9900
S11-C151	1.742(16)	C62-H6A2	0.9900
F11-C151	1.35(2)	C62-H6AB2	0.9900
F21-C151	1.42(2)	C72-D7A2	0.9800
F31-C151	1.35(2)	C72-D7B2	0.9800
N11-C131	1.48(2)	C72-D7C2	0.9800
N11-C11	1.50(2)	C82-C92	1.517(13)
N11-C141	1.50(2)	C82-H8A2	0.9900
N21-C31	1.434(17)	C82-H8AB2	0.9900
N21-C41	1.46(2)	C92-C102	1.40(2)
N21-C51	1.54(2)	C92-H9A2	0.9900
N31-C61	1.46(2)	C92-H9AB2	0.9900
N31-C81	1.46(2)	C102-H10A2	0.9900
N31-C71	1.48(2)	C102-H10B2	0.9900
N41-C121	1.49(2)	C112-D11A2	0.9800
N41-C101	1.50(2)	C112-D11B2	0.9800
N41-C111	1.53(2)	C112-D11C2	0.9800
C11-C21	1.51(2)	C122-C132	1.489(13)
C11-H1A1	0.9900	C122-H12A2	0.9900
C11-H1AB1	0.9900	C122-H12B2	0.9900
C21-C31	1.55(2)	C132-H13A2	0.9900
C21-H2A1	0.9900	C132-H13B2	0.9900
C21-H2AB1	0.9900	C142-D14A2	0.9800
C31-H3A1	0.9900	C142-D14B2	0.9800
C31-H3AB1	0.9900	C142-D14C2	0.9800
C41-D4A1	0.9800	Fe13-O13	1.660(13)
C41-D4B1	0.9800	Fe13-O23	2.041(13)
C41-D4C1	0.9800	Fe13-N33	2.060(16)
C51-C61	1.492(14)	Fe13-N43	2.068(14)
C51-H5A1	0.9900	Fe13-N13	2.108(16)
C51-H5AB1	0.9900	Fe13-N23	2.116(14)
C61-H6A1	0.9900	S13-O43	1.404(11)
C61-H6AB1	0.9900	S13-O33	1.474(11)
C71-D7A1	0.9800	S13-O23	1.496(13)
C71-D7B1	0.9800	S13-C153	1.833(19)
C71-D7C1	0.9800	F13-C153	1.33(2)
C81-C91	1.493(17)	F23-C153	1.33(2)
C81-H8A1	0.9900	F33-C153	1.35(2)
C81-H8AB1	0.9900	N13-C133	1.48(2)
C91-C101	1.53(2)	N13-C143	1.49(2)
C91-H9A1	0.9900	N13-C13	1.512(19)
C91-H9AB1	0.9900	N23-C43	1.48(2)
C101-H10A1	0.9900	N23-C33	1.510(18)
C101-H10B1	0.9900	N23-C53	1.52(2)
C111-D11A1	0.9800	N33-C73	1.48(2)
C111-D11B1	0.9800	N33-C83	1.48(2)
C111-D11C1	0.9800	N33-C63	1.503(19)
C121-C131	1.504(13)	N43-C103	1.45(2)
C121-H12A1	0.9900	N43-C123	1.48(2)
C121-H12B1	0.9900	N43-C113	1.51(2)
C131-H13A1	0.9900	C13-C23	1.57(2)
C131-H13B1	0.9900	C13-H1A3	0.9900
C141-D14A1	0.9800	C13-H1AB3	0.9900
C141-D14B1	0.9800	C23-C33	1.46(2)
C141-D14C1	0.9800	C23-H2A3	0.9900
Fe12-O12	1.626(12)	C23-H2AB3	0.9900
Fe12-N32	2.045(16)	C33-H3A3	0.9900
Fe12-O22	2.061(13)	C33-H3AB3	0.9900
Fe12-N42	2.089(14)	C43-D4A3	0.9800
Fe12-N22	2.101(14)	C43-D4B3	0.9800
Fe12-N12	2.109(15)	C43-D4C3	0.9800
S12-O32	1.408(12)	C53-C63	1.485(13)
S12-O22	1.455(13)	C53-H5A3	0.9900
S12-O42	1.465(10)	C53-H5AB3	0.9900
S12-C152	1.80(2)	C63-H6A3	0.9900
F12-C152	1.35(2)	C63-H6AB3	0.9900
F22-C152	1.34(2)	C73-D7A3	0.9800
F32-C152	1.34(2)	C73-D7B3	0.9800
N12-C142	1.49(2)	C73-D7C3	0.9800
N12-C12	1.493(18)	C83-C93	1.517(12)
N12-C132	1.52(2)	C83-H8A3	0.9900
N22-C42	1.48(2)	C83-H8AB3	0.9900
N22-C52	1.49(2)	C93-C103	1.61(2)
N22-C32	1.508(18)	C93-H9A3	0.9900
N32-C82	1.50(2)	C93-H9AB3	0.9900
N32-C72	1.52(2)	C103-H10A3	0.9900
N32-C62	1.52(2)	C103-H10B3	0.9900
N42-C112	1.475(19)	C113-D11A3	0.9800
N42-C122	1.50(2)	C113-D11B3	0.9800
N42-C102	1.55(2)	C113-D11C3	0.9800
C12-C22	1.47(2)	C123-C133	1.498(13)
C12-H1A2	0.9900	C123-H12A3	0.9900
C12-H1AB2	0.9900	C123-H12B3	0.9900
C22-C32	1.59(2)	C133-H13A3	0.9900
C22-H2A2	0.9900	C133-H13B3	0.9900

C143-D14A3	0.9800	Cl29-C19	1.751(16)
C143-D14B3	0.9800	C19-H1A9	0.9900
C143-D14C3	0.9800	C19-H1AB9	0.9900
Fe14-O14	1.650(14)	Cl110-C110	1.799(19)
Fe14-N44	2.020(14)	Cl210-C110	1.78(2)
Fe14-N24	2.065(15)	C110-H1A10	0.9900
Fe14-N14	2.079(14)	C110-H1AB10	0.9900
Fe14-O24	2.085(12)	Cl111-C111	1.71(2)
Fe14-N34	2.103(14)	Cl211-C111	1.73(3)
S14-O24	1.423(12)	C111-H1A11	0.9900
S14-O34	1.435(14)	C111-H1AB11	0.9900
S14-O44	1.454(15)	Cl1A11-C1A11	1.67(3)
S14-C154	1.882(14)	Cl2A11-C1A11	1.69(3)
F14-C154	1.305(19)	C1A11-H1AA11	0.9900
F24-C154	1.285(19)	C1A11-H1AC11	0.9900
F34-C154	1.32(2)	Cl112-C112	1.769(14)
N14-C134	1.48(2)	Cl212-C112	1.775(15)
N14-C144	1.51(2)	Cl1A12-C112	1.786(19)
N14-C14	1.570(18)	Cl2A12-C112	1.78(2)
N24-C34	1.482(19)	C112-H1A12	0.9900
N24-C44	1.49(2)	C112-H1AB12	0.9900
N24-C54	1.527(19)	C112-HA1A12	0.9900
N34-C74	1.45(2)	C112-HA1B12	0.9900
N34-C84	1.49(2)		
N34-C64	1.49(2)	O11-Fe11-O21	177.0(6)
N44-C114	1.504(19)	O11-Fe11-N41	86.1(6)
N44-C104	1.52(2)	O21-Fe11-N41	96.9(6)
N44-C124	1.53(2)	O11-Fe11-N31	85.2(6)
C14-C24	1.49(2)	O21-Fe11-N31	94.2(5)
C14-H1A4	0.9900	N41-Fe11-N31	95.8(6)
C14-H1AB4	0.9900	O11-Fe11-N11	90.9(6)
C24-C34	1.55(2)	O21-Fe11-N11	89.7(6)
C24-H2A4	0.9900	N41-Fe11-N11	85.7(6)
C24-H2AB4	0.9900	N31-Fe11-N11	175.6(6)
C34-H3A4	0.9900	O11-Fe11-N21	90.5(6)
C34-H3AB4	0.9900	O21-Fe11-N21	86.6(5)
C44-D4A4	0.9800	N41-Fe11-N21	176.1(6)
C44-D4B4	0.9800	N31-Fe11-N21	85.8(6)
C44-D4C4	0.9800	N11-Fe11-N21	92.4(6)
C54-C64	1.485(14)	O31-S11-O41	119.1(7)
C54-H5A4	0.9900	O31-S11-O21	114.1(7)
C54-H5AB4	0.9900	O41-S11-O21	112.4(7)
C64-H6A4	0.9900	O31-S11-C151	103.3(9)
C64-H6AB4	0.9900	O41-S11-C151	103.9(9)
C74-D7A4	0.9800	O21-S11-C151	101.2(9)
C74-D7B4	0.9800	S11-O21-Fe11	154.0(8)
C74-D7C4	0.9800	C131-N11-C11	111.6(13)
C84-C94	1.487(15)	C131-N11-C141	105.0(14)
C84-H8A4	0.9900	C11-N11-C141	109.9(14)
C84-H8AB4	0.9900	C131-N11-Fe11	104.8(10)
C94-C104	1.536(16)	C11-N11-Fe11	108.4(12)
C94-H9A4	0.9900	C141-N11-Fe11	117.0(11)
C94-H9AB4	0.9900	C31-N21-C41	113.5(13)
C104-H10A4	0.9900	C31-N21-C51	108.6(13)
C104-H10B4	0.9900	C41-N21-C51	105.3(11)
C114-D11A4	0.9800	C31-N21-Fe11	105.4(9)
C114-D11B4	0.9800	C41-N21-Fe11	118.7(10)
C114-D11C4	0.9800	C51-N21-Fe11	104.6(9)
C124-C134	1.495(14)	C61-N31-C81	108.7(13)
C124-H12A4	0.9900	C61-N31-C71	110.6(13)
C124-H12B4	0.9900	C81-N31-C71	107.9(13)
C134-H13A4	0.9900	C61-N31-Fe11	100.7(10)
C134-H13B4	0.9900	C81-N31-Fe11	115.5(12)
C144-D14A4	0.9800	C71-N31-Fe11	113.2(11)
C144-D14B4	0.9800	C121-N41-C101	108.1(13)
C144-D14C4	0.9800	C121-N41-C111	107.7(13)
S15-O25	1.377(10)	C101-N41-C111	104.9(13)
S15-O35	1.442(13)	C121-N41-Fe11	104.1(10)
S15-O15	1.473(12)	C101-N41-Fe11	115.4(12)
S15-C15	1.83(2)	C111-N41-Fe11	116.3(11)
F15-C15	1.32(2)	N11-C11-C21	113.2(14)
F25-C15	1.33(2)	N11-C11-H1A1	108.9
F35-C15	1.33(2)	C21-C11-H1A1	108.9
S16-O16	1.415(13)	N11-C11-H1AB1	108.9
S16-O36	1.431(14)	C21-C11-H1AB1	108.9
S16-O26	1.518(10)	H1A1-C11-H1AB1	107.7
S16-C16	1.81(2)	C11-C21-C31	119.6(15)
F16-C16	1.33(2)	C11-C21-H2A1	107.4
F26-C16	1.34(2)	C31-C21-H2A1	107.4
F36-C16	1.34(2)	C11-C21-H2AB1	107.4
S17-O27	1.461(12)	C31-C21-H2AB1	107.4
S17-O37	1.463(13)	H2A1-C21-H2AB1	106.9
S17-O17	1.464(11)	N21-C31-C21	114.2(13)
S17-C17	1.881(18)	N21-C31-H3A1	108.7
F17-C17	1.36(2)	C21-C31-H3A1	108.7
F27-C17	1.31(2)	N21-C31-H3AB1	108.7
F37-C17	1.34(2)	C21-C31-H3AB1	108.7
S18-O18	1.411(11)	H3A1-C31-H3AB1	107.6
S18-O38	1.413(12)	N21-C41-D4A1	109.5
S18-O28	1.422(13)	N21-C41-D4B1	109.5
S18-C18	1.781(18)	D4A1-C41-D4B1	109.5
F18-C18	1.32(2)	N21-C41-D4C1	109.5
F28-C18	1.34(2)	D4A1-C41-D4C1	109.5
F38-C18	1.336(19)	D4B1-C41-D4C1	109.5
Cl19-C19	1.756(15)	C61-C51-N21	109.8(13)

C61-C51-H5A1	109.7	C52-N22-C32	107.5(14)
N21-C51-H5A1	109.7	C42-N22-Fe12	121.7(11)
C61-C51-H5AB1	109.7	C52-N22-Fe12	106.1(10)
N21-C51-H5AB1	109.7	C32-N22-Fe12	107.2(10)
H5A1-C51-H5AB1	108.2	C82-N32-C72	108.6(12)
N31-C61-C51	113.5(14)	C82-N32-C62	107.1(13)
N31-C61-H6A1	108.9	C72-N32-C62	107.2(12)
C51-C61-H6A1	108.9	C82-N32-Fe12	117.0(10)
N31-C61-H6AB1	108.9	C72-N32-Fe12	113.5(12)
C51-C61-H6AB1	108.9	C62-N32-Fe12	102.7(10)
H6A1-C61-H6AB1	107.7	C112-N42-C122	111.1(13)
N31-C71-D7A1	109.5	C112-N42-C102	105.2(12)
N31-C71-D7B1	109.5	C122-N42-C102	106.7(12)
D7A1-C71-D7B1	109.5	C112-N42-Fe12	117.5(10)
N31-C71-D7C1	109.5	C122-N42-Fe12	102.4(9)
D7A1-C71-D7C1	109.5	C102-N42-Fe12	113.5(10)
D7B1-C71-D7C1	109.5	C22-C12-N12	113.9(14)
N31-C81-C91	114.0(16)	C22-C12-H1A2	108.8
N31-C81-H8A1	108.7	N12-C12-H1A2	108.8
C91-C81-H8A1	108.7	C22-C12-H1AB2	108.8
N31-C81-H8AB1	108.7	N12-C12-H1AB2	108.8
C91-C81-H8AB1	108.7	H1A2-C12-H1AB2	107.7
H8A1-C81-H8AB1	107.6	C12-C22-C32	117.3(15)
C81-C91-C101	110.3(17)	C12-C22-H2A2	108.0
C81-C91-H9A1	109.6	C32-C22-H2A2	108.0
C101-C91-H9A1	109.6	C12-C22-H2AB2	108.0
C81-C91-H9AB1	109.6	C32-C22-H2AB2	108.0
C101-C91-H9AB1	109.6	H2A2-C22-H2AB2	107.2
H9A1-C91-H9AB1	108.1	N22-C32-C22	112.9(15)
N41-C101-C91	113.3(16)	N22-C32-H3A2	109.0
N41-C101-H10A1	108.9	C22-C32-H3A2	109.0
C91-C101-H10A1	108.9	N22-C32-H3AB2	109.0
N41-C101-H10B1	108.9	C22-C32-H3AB2	109.0
C91-C101-H10B1	108.9	H3A2-C32-H3AB2	107.8
H10A1-C101-H10B1	107.7	N22-C42-D4A2	109.5
N41-C111-D11A1	109.5	N22-C42-D4B2	109.5
N41-C111-D11B1	109.5	D4A2-C42-D4B2	109.5
D11A1-C111-D11B1	109.5	N22-C42-D4C2	109.5
N41-C111-D11C1	109.5	D4A2-C42-D4C2	109.5
D11A1-C111-D11C1	109.5	D4B2-C42-D4C2	109.5
D11B1-C111-D11C1	109.5	N22-C52-C62	113.3(14)
N41-C121-C131	108.3(14)	N22-C52-H5A2	108.9
N41-C121-H12A1	110.0	C62-C52-H5A2	108.9
C131-C121-H12A1	110.0	N22-C52-H5AB2	108.9
N41-C121-H12B1	110.0	C62-C52-H5AB2	108.9
C131-C121-H12B1	110.0	H5A2-C52-H5AB2	107.7
H12A1-C121-H12B1	108.4	C52-C62-N32	110.4(14)
N11-C131-C121	114.1(14)	C52-C62-H6A2	109.6
N11-C131-H13A1	108.7	N32-C62-H6A2	109.6
C121-C131-H13A1	108.7	C52-C62-H6AB2	109.6
N11-C131-H13B1	108.7	N32-C62-H6AB2	109.6
C121-C131-H13B1	108.7	H6A2-C62-H6AB2	108.1
H13A1-C131-H13B1	107.6	N32-C72-D7A2	109.5
N11-C141-D14A1	109.5	N32-C72-D7B2	109.5
N11-C141-D14B1	109.5	D7A2-C72-D7B2	109.5
D14A1-C141-D14B1	109.5	N32-C72-D7C2	109.5
N11-C141-D14C1	109.5	D7A2-C72-D7C2	109.5
D14A1-C141-D14C1	109.5	D7B2-C72-D7C2	109.5
D14B1-C141-D14C1	109.5	N32-C82-C92	114.2(14)
F31-C151-F11	101.7(15)	N32-C82-H8A2	108.7
F31-C151-F21	106.3(13)	C92-C82-H8A2	108.7
F11-C151-F21	104.5(13)	N32-C82-H8AB2	108.7
F31-C151-S11	116.7(13)	C92-C82-H8AB2	108.7
F11-C151-S11	116.0(13)	H8A2-C82-H8AB2	107.6
F21-C151-S11	110.4(13)	C102-C92-C82	113.4(14)
O12-Fe12-N32	86.4(6)	C102-C92-H9A2	108.9
O12-Fe12-O22	178.9(6)	C82-C92-H9A2	108.9
N32-Fe12-O22	94.0(5)	C102-C92-H9AB2	108.9
O12-Fe12-N42	86.9(6)	C82-C92-H9AB2	108.9
N32-Fe12-N42	95.1(6)	H9A2-C92-H9AB2	107.7
O22-Fe12-N42	94.1(5)	C92-C102-N42	113.1(14)
O12-Fe12-N22	90.4(6)	C92-C102-H10A2	109.0
N32-Fe12-N22	86.5(6)	N42-C102-H10A2	109.0
O22-Fe12-N22	88.6(6)	C92-C102-H10B2	109.0
N42-Fe12-N22	176.7(5)	N42-C102-H10B2	109.0
O12-Fe12-N12	90.7(5)	H10A2-C102-H10B2	107.8
N32-Fe12-N12	177.1(6)	N42-C112-D11A2	109.5
O22-Fe12-N12	88.9(5)	N42-C112-D11B2	109.5
N42-Fe12-N12	85.4(6)	D11A2-C112-D11B2	109.5
N22-Fe12-N12	92.8(6)	N42-C112-D11C2	109.5
O32-S12-O22	116.6(7)	D11A2-C112-D11C2	109.5
O32-S12-O42	117.0(6)	D11B2-C112-D11C2	109.5
O22-S12-O42	113.3(7)	C132-C122-N42	109.1(14)
O32-S12-C152	103.0(9)	C132-C122-H12A2	109.9
O22-S12-C152	102.5(8)	N42-C122-H12A2	109.9
O42-S12-C152	101.2(8)	C132-C122-H12B2	109.9
S12-O22-Fe12	156.9(8)	N42-C122-H12B2	109.9
C142-N12-C12	108.4(13)	H12A2-C122-H12B2	108.3
C142-N12-C132	103.2(12)	C122-C132-N12	112.0(14)
C12-N12-C132	109.8(12)	C122-C132-H13A2	109.2
C142-N12-Fe12	121.2(9)	N12-C132-H13A2	109.2
C12-N12-Fe12	108.1(11)	C122-C132-H13B2	109.2
C132-N12-Fe12	105.7(10)	N12-C132-H13B2	109.2
C42-N22-C52	105.9(11)	H13A2-C132-H13B2	107.9
C42-N22-C32	107.7(13)	N12-C142-D14A2	109.5

N12-C142-D14B2	109.5
D14A2-C142-D14B2	109.5
N12-C142-D14C2	109.5
D14A2-C142-D14C2	109.5
D14B2-C142-D14C2	109.5
F22-C152-F32	108.9(14)
F22-C152-F12	105.1(14)
F32-C152-F12	105.1(16)
F22-C152-S12	114.1(14)
F32-C152-S12	112.6(12)
F12-C152-S12	110.5(13)
O13-Fe13-O23	178.4(6)
O13-Fe13-N33	87.7(6)
O23-Fe13-N33	93.4(5)
O13-Fe13-N43	84.9(6)
O23-Fe13-N43	96.2(6)
N33-Fe13-N43	98.5(6)
O13-Fe13-N13	92.1(6)
O23-Fe13-N13	86.8(6)
N33-Fe13-N13	176.8(6)
N43-Fe13-N13	84.6(6)
O13-Fe13-N23	90.6(6)
O23-Fe13-N23	88.3(6)
N33-Fe13-N23	83.8(6)
N43-Fe13-N23	174.8(6)
N13-Fe13-N23	93.1(6)
O43-S13-O33	116.8(6)
O43-S13-O23	114.9(7)
O33-S13-O23	109.8(7)
O43-S13-C153	108.4(7)
O33-S13-C153	104.8(9)
O23-S13-C153	100.3(8)
S13-O23-Fe13	155.1(8)
C133-N13-C143	107.7(13)
C133-N13-C13	107.6(12)
C143-N13-C13	107.8(13)
C133-N13-Fe13	107.3(11)
C143-N13-Fe13	118.8(9)
C13-N13-Fe13	107.2(11)
C43-N23-C33	109.4(13)
C43-N23-C53	104.7(11)
C33-N23-C53	110.5(14)
C43-N23-Fe13	118.0(11)
C33-N23-Fe13	107.1(9)
C53-N23-Fe13	107.1(10)
C73-N33-C83	107.4(13)
C73-N33-C63	112.3(13)
C83-N33-C63	104.0(12)
C73-N33-Fe13	114.0(12)
C83-N33-Fe13	114.4(10)
C63-N33-Fe13	104.4(10)
C103-N43-C123	108.6(13)
C103-N43-C113	109.1(12)
C123-N43-C113	107.3(13)
C103-N43-Fe13	114.2(11)
C123-N43-Fe13	102.2(10)
C113-N43-Fe13	114.9(11)
N13-C13-C23	115.0(14)
N13-C13-H1A3	108.5
C23-C13-H1A3	108.5
N13-C13-H1AB3	108.5
C23-C13-H1AB3	108.5
H1A3-C13-H1AB3	107.5
C33-C23-C13	119.6(15)
C33-C23-H2A3	107.4
C13-C23-H2A3	107.4
C33-C23-H2AB3	107.4
C13-C23-H2AB3	107.4
H2A3-C23-H2AB3	106.9
C23-C33-N23	116.0(15)
C23-C33-H3A3	108.3
N23-C33-H3A3	108.3
C23-C33-H3AB3	108.3
N23-C33-H3AB3	108.3
H3A3-C33-H3AB3	107.4
N23-C43-D4A3	109.5
N23-C43-D4B3	109.5
D4A3-C43-D4B3	109.5
N23-C43-D4C3	109.5
D4A3-C43-D4C3	109.5
D4B3-C43-D4C3	109.5
C63-C53-N23	110.8(13)
C63-C53-H5A3	109.5
N23-C53-H5A3	109.5
C63-C53-H5AB3	109.5
N23-C53-H5AB3	109.5
H5A3-C53-H5AB3	108.1
C53-C63-N33	108.1(13)
C53-C63-H6A3	110.1
N33-C63-H6A3	110.1
C53-C63-H6AB3	110.1
N33-C63-H6AB3	110.1
H6A3-C63-H6AB3	108.4
N33-C73-D7A3	109.5
N33-C73-D7B3	109.5
D7A3-C73-D7B3	109.5
N33-C73-D7C3	109.5
D7A3-C73-D7C3	109.5
D7B3-C73-D7C3	109.5
N33-C83-C93	115.7(13)
N33-C83-H8A3	108.4
C93-C83-H8A3	108.4
N33-C83-H8AB3	108.4
C93-C83-H8AB3	108.4
H8A3-C83-H8AB3	107.4
C83-C93-C103	110.3(13)
C83-C93-H9A3	109.6
C103-C93-H9A3	109.6
C83-C93-H9AB3	109.6
C103-C93-H9AB3	109.6
H9A3-C93-H9AB3	108.1
N43-C103-C93	114.8(13)
N43-C103-H10A3	108.6
C93-C103-H10A3	108.6
N43-C103-H10B3	108.6
C93-C103-H10B3	108.6
H10A3-C103-H10B3	107.5
N43-C113-D11A3	109.5
N43-C113-D11B3	109.5
D11A3-C113-D11B3	109.5
N43-C113-D11C3	109.5
D11A3-C113-D11C3	109.5
D11B3-C113-D11C3	109.5
N43-C123-C133	110.3(14)
N43-C123-H12A3	109.6
C133-C123-H12A3	109.6
N43-C123-H12B3	109.6
C133-C123-H12B3	109.6
H12A3-C123-H12B3	108.1
N13-C133-C123	110.5(14)
N13-C133-H13A3	109.5
C123-C133-H13A3	109.5
N13-C133-H13B3	109.5
C123-C133-H13B3	109.5
H13A3-C133-H13B3	108.1
N13-C143-D14A3	109.5
N13-C143-D14B3	109.5
D14A3-C143-D14B3	109.5
N13-C143-D14C3	109.5
D14A3-C143-D14C3	109.5
D14B3-C143-D14C3	109.5
F13-C153-F23	111.1(17)
F13-C153-F33	109.5(15)
F23-C153-F33	106.4(14)
F13-C153-S13	113.4(13)
F23-C153-S13	108.0(12)
F33-C153-S13	108.1(14)
O14-Fe14-N44	87.7(7)
O14-Fe14-N24	91.3(6)
N44-Fe14-N24	177.7(6)
O14-Fe14-N14	90.7(6)
N44-Fe14-N14	84.0(6)
N24-Fe14-N14	93.9(6)
O14-Fe14-O24	179.5(6)
N44-Fe14-O24	92.7(5)
N24-Fe14-O24	88.3(6)
N14-Fe14-O24	88.9(5)
O14-Fe14-N34	86.0(6)
N44-Fe14-N34	98.0(6)
N24-Fe14-N34	84.0(6)
N14-Fe14-N34	176.1(6)
O24-Fe14-N34	94.3(5)
O24-S14-O34	114.7(8)
O24-S14-O44	113.9(8)
O34-S14-O44	115.5(8)
O24-S14-C154	102.6(8)
O34-S14-C154	104.7(8)
O44-S14-C154	103.1(8)
S14-O24-Fe14	156.6(8)
C134-N14-C144	104.5(11)
C134-N14-C14	108.9(13)
C144-N14-C14	102.4(11)
C134-N14-Fe14	109.1(10)
C144-N14-Fe14	122.9(11)
C14-N14-Fe14	108.3(9)
C34-N24-C44	107.8(13)
C34-N24-C54	105.6(12)
C44-N24-C54	105.7(14)
C34-N24-Fe14	108.4(12)
C44-N24-Fe14	119.5(11)
C54-N24-Fe14	108.9(10)
C74-N34-C84	110.3(13)
C74-N34-C64	110.8(13)
C84-N34-C64	106.3(13)
C74-N34-Fe14	116.3(11)
C84-N34-Fe14	111.9(10)
C64-N34-Fe14	100.6(10)
C114-N44-C104	107.0(12)
C114-N44-C124	107.2(11)
C104-N44-C124	104.0(12)

C114-N44-Fe14	115.7(11)	F14-C154-F34	113.1(15)
C104-N44-Fe14	115.7(10)	F24-C154-S14	110.6(12)
C124-N44-Fe14	106.4(10)	F14-C154-S14	106.1(10)
C24-C14-N14	115.2(13)	F34-C154-S14	107.7(10)
C24-C14-H1A4	108.5	O25-S15-O35	118.7(7)
N14-C14-H1A4	108.5	O25-S15-O15	115.0(6)
C24-C14-H1AB4	108.5	O35-S15-O15	110.7(8)
N14-C14-H1AB4	108.5	O25-S15-C15	107.4(8)
H1A4-C14-H1AB4	107.5	O35-S15-C15	103.1(9)
C14-C24-C34	116.8(14)	O15-S15-C15	99.2(8)
C14-C24-H2A4	108.1	F15-C15-F25	107.3(17)
C34-C24-H2A4	108.1	F15-C15-F35	109.5(17)
C14-C24-H2AB4	108.1	F25-C15-F35	106.0(17)
C34-C24-H2AB4	108.1	F15-C15-S15	111.9(14)
H2A4-C24-H2AB4	107.3	F25-C15-S15	110.7(14)
N24-C34-C24	115.5(14)	F35-C15-S15	111.2(14)
N24-C34-H3A4	108.4	O16-S16-O36	119.4(8)
C24-C34-H3A4	108.4	O16-S16-O26	114.9(6)
N24-C34-H3AB4	108.4	O36-S16-O26	111.3(7)
C24-C34-H3AB4	108.4	O16-S16-C16	105.9(8)
H3A4-C34-H3AB4	107.5	O36-S16-C16	102.7(9)
N24-C44-D4A4	109.5	O26-S16-C16	99.4(7)
N24-C44-D4B4	109.5	F16-C16-F26	105.2(15)
D4A4-C44-D4B4	109.5	F16-C16-F36	107.6(17)
N24-C44-D4C4	109.5	F26-C16-F36	107.3(17)
D4A4-C44-D4C4	109.5	F16-C16-S16	112.5(14)
D4B4-C44-D4C4	109.5	F26-C16-S16	110.4(14)
C64-C54-N24	108.3(15)	F36-C16-S16	113.5(14)
C64-C54-H5A4	110.0	O27-S17-O37	111.6(8)
N24-C54-H5A4	110.0	O27-S17-O17	124.0(7)
C64-C54-H5AB4	110.0	O37-S17-O17	115.3(6)
N24-C54-H5AB4	110.0	O27-S17-C17	100.1(7)
H5A4-C54-H5AB4	108.4	O37-S17-C17	99.6(8)
C54-C64-N34	109.9(14)	O17-S17-C17	100.4(7)
C54-C64-H6A4	109.7	F27-C17-F37	113.4(16)
N34-C64-H6A4	109.7	F27-C17-F17	110.1(17)
C54-C64-H6AB4	109.7	F37-C17-F17	107.5(16)
N34-C64-H6AB4	109.7	F27-C17-S17	108.8(13)
H6A4-C64-H6AB4	108.2	F37-C17-S17	107.5(12)
N34-C74-D7A4	109.5	F17-C17-S17	109.4(13)
N34-C74-D7B4	109.5	O18-S18-O38	106.5(6)
D7A4-C74-D7B4	109.5	O18-S18-O28	114.9(6)
N34-C74-D7C4	109.5	O38-S18-O28	118.4(8)
D7A4-C74-D7C4	109.5	O18-S18-C18	105.1(8)
D7B4-C74-D7C4	109.5	O38-S18-C18	105.2(8)
C94-C84-N34	115.8(14)	O28-S18-C18	105.6(8)
C94-C84-H8A4	108.3	F18-C18-F38	105.4(16)
N34-C84-H8A4	108.3	F18-C18-F28	105.6(16)
C94-C84-H8AB4	108.3	F38-C18-F28	100.6(14)
N34-C84-H8AB4	108.3	F18-C18-S18	112.8(13)
H8A4-C84-H8AB4	107.4	F38-C18-S18	114.7(13)
C84-C94-C104	112.4(13)	F28-C18-S18	116.3(13)
C84-C94-H9A4	109.1	Cl29-C19-Cl19	114.1(11)
C104-C94-H9A4	109.1	Cl29-C19-H1A9	108.7
C84-C94-H9AB4	109.1	Cl19-C19-H1A9	108.7
C104-C94-H9AB4	109.1	Cl29-C19-H1AB9	108.7
H9A4-C94-H9AB4	107.8	Cl19-C19-H1AB9	108.7
N44-C104-C94	115.0(14)	H1A9-C19-H1AB9	107.6
N44-C104-H10A4	108.5	Cl210-C110-Cl110	111.9(10)
C94-C104-H10A4	108.5	Cl210-C110-H1A10	109.2
N44-C104-H10B4	108.5	Cl110-C110-H1A10	109.2
C94-C104-H10B4	108.5	Cl210-C110-H1AB10	109.2
H10A4-C104-H10B4	107.5	Cl110-C110-H1AB10	109.2
N44-C114-D11A4	109.5	H1A10-C110-H1AB10	107.9
N44-C114-D11B4	109.5	Cl111-C111-Cl211	116.7(13)
D11A4-C114-D11B4	109.5	Cl111-C111-H1A11	108.1
N44-C114-D11C4	109.5	Cl211-C111-H1A11	108.1
D11A4-C114-D11C4	109.5	Cl111-C111-H1AB11	108.1
D11B4-C114-D11C4	109.5	Cl211-C111-H1AB11	108.1
C134-C124-N44	107.3(14)	H1A11-C111-H1AB11	107.3
C134-C124-H12A4	110.3	Cl1A11-C1A11-Cl2A11	104(2)
N44-C124-H12A4	110.3	Cl1A11-C1A11-H1AA11	110.9
C134-C124-H12B4	110.3	Cl2A11-C1A11-H1AA11	110.9
N44-C124-H12B4	110.3	Cl1A11-C1A11-H1AC11	110.9
H12A4-C124-H12B4	108.5	Cl2A11-C1A11-H1AC11	110.9
N14-C134-C124	113.1(14)	H1AA11-C1A11-H1AC11	109.0
N14-C134-H13A4	109.0	Cl112-C112-Cl212	112.8(10)
C124-C134-H13A4	109.0	Cl2A12-C112-Cl1A12	96.3(16)
N14-C134-H13B4	109.0	Cl112-C112-H1A12	109.0
C124-C134-H13B4	109.0	Cl212-C112-H1A12	109.0
H13A4-C134-H13B4	107.8	Cl112-C112-H1AB12	109.0
N14-C144-D14A4	109.5	Cl212-C112-H1AB12	109.0
N14-C144-D14B4	109.5	H1A12-C112-H1AB12	107.8
D14A4-C144-D14B4	109.5	Cl2A12-C112-HA1A12	112.5
N14-C144-D14C4	109.5	Cl1A12-C112-HA1A12	112.5
D14A4-C144-D14C4	109.5	Cl2A12-C112-HA1B12	112.5
D14B4-C144-D14C4	109.5	Cl1A12-C112-HA1B12	112.5
F24-C154-F14	110.5(13)	HA1A12-C112-HA1B12	110.0
F24-C154-F34	108.8(12)		

Table S14. Anisotropic displacement parameters (Å^{2}x 10^3) for **1b′-OTf**.
The anisotropic displacement factor exponent takes the form: -2p^2[h^2 a*^{2}U^{11} + ... + 2 h k a* b* U^{12}]

	U^{11}	U^{22}	U^{33}	U^{23}	U^{13}	U^{12}
Fe11	19(1)	7(1)	10(1)	-2(1)	-1(1)	0(1)
S11	19(1)	7(1)	10(1)	-2(1)	-1(1)	0(1)
F11	28(3)	37(3)	38(3)	-6(2)	13(2)	-1(2)
F21	28(3)	37(3)	38(3)	-6(2)	13(2)	-1(2)
F31	28(3)	37(3)	38(3)	-6(2)	13(2)	-1(2)
O11	19(1)	7(1)	10(1)	-2(1)	-1(1)	0(1)
O21	19(1)	7(1)	10(1)	-2(1)	-1(1)	0(1)
O31	19(1)	7(1)	10(1)	-2(1)	-1(1)	0(1)
O41	19(1)	7(1)	10(1)	-2(1)	-1(1)	0(1)
N11	22(3)	16(3)	8(3)	3(2)	2(2)	1(2)
N21	12(4)	6(3)	13(4)	-3(3)	-7(3)	-2(3)
N31	22(3)	16(3)	8(3)	3(2)	2(2)	1(2)
N41	22(3)	16(3)	8(3)	3(2)	2(2)	1(2)
C11	22(4)	9(3)	23(4)	0(3)	-7(3)	2(2)
C21	22(4)	9(3)	23(4)	0(3)	-7(3)	2(2)
C31	22(4)	9(3)	23(4)	0(3)	-7(3)	2(2)
C41	19(4)	19(3)	18(4)	1(3)	2(3)	-4(3)
C51	15(5)	22(6)	16(5)	-3(5)	-12(4)	4(4)
C61	27(6)	24(5)	10(6)	-7(5)	-6(5)	3(4)
C71	19(4)	19(3)	18(4)	1(3)	2(3)	-4(3)
C81	34(5)	49(5)	12(4)	10(4)	14(3)	2(4)
C91	34(5)	49(5)	12(4)	10(4)	14(3)	2(4)
C101	34(5)	49(5)	12(4)	10(4)	14(3)	2(4)
C111	19(4)	19(3)	18(4)	1(3)	2(3)	-4(3)
C121	17(5)	19(6)	16(7)	0(5)	-2(5)	0(4)
C131	21(5)	15(5)	13(7)	1(5)	-7(4)	1(4)
C141	19(4)	19(3)	18(4)	1(3)	2(3)	-4(3)
C151	28(4)	30(4)	30(5)	-7(3)	11(3)	0(4)
Fe12	8(1)	9(1)	6(1)	0(1)	-1(1)	0(1)
S12	6(2)	18(2)	8(2)	-3(2)	1(2)	1(2)
F12	29(3)	25(3)	16(3)	-6(2)	-3(2)	3(2)
F22	29(3)	25(3)	16(3)	-6(2)	-3(2)	3(2)
F32	29(3)	25(3)	16(3)	-6(2)	-3(2)	3(2)
O12	8(1)	9(1)	6(1)	0(1)	-1(1)	0(1)
O22	10(2)	19(3)	9(3)	0(2)	1(2)	0(2)
O32	10(2)	19(3)	9(3)	0(2)	1(2)	0(2)
O42	10(2)	19(3)	9(3)	0(2)	1(2)	0(2)
N12	7(2)	13(2)	9(3)	-2(2)	1(2)	1(2)
N22	7(2)	13(2)	9(3)	-2(2)	1(2)	1(2)
N32	7(2)	13(2)	9(3)	-2(2)	1(2)	1(2)
N42	7(2)	13(2)	9(3)	-2(2)	1(2)	1(2)
C12	6(3)	20(4)	14(4)	4(3)	-1(3)	0(2)
C22	6(3)	20(4)	14(4)	4(3)	-1(3)	0(2)
C32	6(3)	20(4)	14(4)	4(3)	-1(3)	0(2)
C42	16(4)	13(4)	15(4)	-5(3)	-4(3)	6(3)
C52	6(3)	20(4)	14(4)	4(3)	-1(3)	0(2)
C62	6(3)	20(4)	14(4)	4(3)	-1(3)	0(2)
C72	13(4)	11(4)	6(4)	2(3)	0(3)	-3(3)
C82	15(4)	25(5)	10(4)	-11(3)	4(3)	1(3)
C92	15(4)	25(5)	10(4)	-11(3)	4(3)	1(3)
C102	15(4)	25(5)	10(4)	-11(3)	4(3)	1(3)
C112	16(4)	13(4)	15(4)	-5(3)	-4(3)	6(3)
C122	13(4)	12(4)	18(7)	3(4)	4(5)	2(3)
C132	13(4)	12(4)	18(7)	3(4)	4(5)	2(3)
C142	13(4)	11(4)	6(4)	2(3)	0(3)	-3(3)
C152	20(4)	22(4)	16(5)	-5(3)	-4(3)	3(4)
Fe13	6(1)	10(1)	6(1)	1(1)	-1(1)	-1(1)
S13	15(2)	12(2)	9(3)	-2(2)	5(2)	-1(2)
F13	28(3)	17(3)	22(3)	-9(2)	4(2)	1(2)
F23	28(3)	17(3)	22(3)	-9(2)	4(2)	1(2)
F33	28(3)	17(3)	22(3)	-9(2)	4(2)	1(2)
O13	16(3)	11(3)	16(4)	-2(3)	3(3)	5(2)
O23	12(2)	13(3)	12(3)	1(2)	7(2)	0(2)
O33	12(2)	13(3)	12(3)	1(2)	7(2)	0(2)
O43	12(2)	13(3)	12(3)	1(2)	7(2)	0(2)
N13	12(3)	12(3)	13(3)	2(2)	0(2)	0(2)
N23	8(3)	12(3)	12(4)	6(3)	-1(3)	-2(3)
N33	15(4)	8(4)	9(4)	0(3)	2(3)	0(3)
N43	12(3)	12(3)	13(3)	2(2)	0(2)	0(2)
C13	8(3)	20(5)	17(5)	4(4)	-4(3)	0(3)
C23	8(3)	20(5)	17(5)	4(4)	-4(3)	0(3)
C33	8(4)	6(4)	19(5)	-1(4)	1(4)	-4(3)
C43	11(3)	17(4)	14(3)	0(3)	-1(3)	-1(3)
C53	14(3)	7(4)	9(4)	5(3)	3(3)	0(3)
C63	14(3)	7(4)	9(4)	5(3)	3(3)	0(3)
C73	11(3)	17(4)	14(3)	0(3)	-1(3)	-1(3)
C83	17(4)	7(3)	10(3)	3(3)	-2(3)	-2(3)
C93	17(4)	7(3)	10(3)	3(3)	-2(3)	-2(3)
C103	17(4)	7(3)	10(3)	3(3)	-2(3)	-2(3)
C113	11(3)	17(4)	14(3)	0(3)	-1(3)	-1(3)
C123	20(5)	9(4)	18(7)	4(4)	1(5)	3(3)
C133	20(5)	9(4)	18(7)	4(4)	1(5)	3(3)
C143	11(3)	17(4)	14(3)	0(3)	-1(3)	-1(3)
C153	22(4)	15(4)	22(5)	-6(3)	4(3)	3(4)
Fe14	4(1)	14(1)	6(1)	2(1)	0(1)	1(1)
S14	7(1)	20(1)	16(2)	-2(1)	-4(1)	0(1)
F14	17(2)	33(3)	24(3)	-4(2)	-6(2)	0(2)
F24	17(2)	33(3)	24(3)	-4(2)	-6(2)	0(2)
F34	17(2)	33(3)	24(3)	-4(2)	-6(2)	0(2)
O14	17(3)	25(3)	21(4)	-1(3)	-5(3)	-4(3)

O24	5(3)	16(3)	13(3)	-2(2)	-2(2)	0(2)
O34	33(4)	13(3)	34(5)	0(3)	2(3)	-1(3)
O44	24(3)	34(4)	18(4)	-4(3)	3(3)	5(3)
N14	11(2)	15(3)	13(3)	-1(2)	0(2)	1(2)
N24	8(3)	5(3)	16(4)	4(3)	3(3)	1(3)
N34	11(2)	15(3)	13(3)	-1(2)	0(2)	1(2)
N44	11(2)	15(3)	13(3)	-1(2)	0(2)	1(2)
C14	20(3)	8(2)	16(3)	1(2)	-2(3)	1(2)
C24	20(3)	8(2)	16(3)	1(2)	-2(3)	1(2)
C34	20(3)	8(2)	16(3)	1(2)	-2(3)	1(2)
C44	20(5)	17(5)	12(5)	4(4)	7(4)	1(4)
C54	4(3)	22(5)	32(8)	0(5)	3(4)	-1(3)
C64	4(3)	22(5)	32(8)	0(5)	3(4)	-1(3)
C74	14(4)	14(3)	16(4)	1(3)	-1(3)	0(3)
C84	20(3)	8(2)	16(3)	1(2)	-2(3)	1(2)
C94	20(3)	8(2)	16(3)	1(2)	-2(3)	1(2)
C104	20(3)	8(2)	16(3)	1(2)	-2(3)	1(2)
C114	14(4)	14(3)	16(4)	1(3)	-1(3)	0(3)
C124	21(5)	12(4)	18(5)	-3(4)	9(4)	0(3)
C134	21(5)	12(4)	18(5)	-3(4)	9(4)	0(3)
C144	14(4)	14(3)	16(4)	1(3)	-1(3)	0(3)
C154	7(1)	20(1)	16(2)	-2(1)	-4(1)	0(1)
S15	19(2)	9(2)	11(2)	-3(2)	-6(2)	-4(2)
F15	63(7)	7(4)	25(7)	-5(4)	-19(6)	5(4)
F25	40(6)	24(6)	13(5)	-6(4)	-10(4)	-13(5)
F35	20(5)	27(6)	44(8)	-20(6)	-5(5)	-7(4)
O15	30(3)	24(3)	15(3)	0(2)	-2(2)	1(2)
O25	30(3)	24(3)	15(3)	0(2)	-2(2)	1(2)
O35	30(3)	24(3)	15(3)	0(2)	-2(2)	1(2)
C15	24(5)	9(4)	16(5)	-2(4)	-6(4)	1(4)
S16	20(2)	24(2)	14(3)	2(2)	4(2)	4(2)
F16	33(5)	18(4)	33(7)	2(5)	6(6)	8(4)
F26	14(5)	51(8)	44(8)	7(6)	-11(5)	8(4)
F36	32(6)	36(6)	14(5)	0(5)	-1(4)	0(5)
O16	26(3)	29(3)	14(3)	-3(2)	6(2)	4(2)
O26	26(3)	29(3)	14(3)	-3(2)	6(2)	4(2)
O36	26(3)	29(3)	14(3)	-3(2)	6(2)	4(2)
C16	16(5)	18(5)	13(5)	0(5)	-4(4)	3(4)
S17	14(2)	26(2)	12(3)	-4(2)	0(2)	-5(2)
F17	34(6)	41(6)	9(5)	-8(4)	-2(4)	-4(6)
F27	52(7)	14(4)	36(8)	-9(4)	-2(6)	-5(4)
F37	9(4)	61(7)	31(7)	-31(6)	-9(4)	6(4)
O17	21(3)	31(3)	20(3)	-5(2)	2(2)	-1(2)
O27	21(3)	31(3)	20(3)	-5(2)	2(2)	-1(2)
O37	21(3)	31(3)	20(3)	-5(2)	2(2)	-1(2)
C17	12(5)	21(5)	9(5)	-5(4)	0(4)	3(4)
S18	13(2)	23(2)	12(3)	-6(2)	1(2)	4(2)
F18	27(5)	30(6)	6(4)	-1(4)	4(4)	7(5)
F28	19(4)	39(5)	27(7)	-8(5)	-2(4)	-2(4)
F38	29(5)	18(5)	37(8)	12(4)	8(5)	11(3)
O18	15(2)	35(3)	18(3)	-6(2)	-1(2)	6(2)
O28	15(2)	35(3)	18(3)	-6(2)	-1(2)	6(2)
O38	15(2)	35(3)	18(3)	-6(2)	-1(2)	6(2)
C18	16(4)	15(5)	7(5)	1(4)	0(4)	5(4)
Cl19	64(4)	26(3)	37(4)	-9(3)	13(3)	-15(3)
Cl29	28(3)	32(3)	36(4)	-2(3)	-1(3)	6(2)
C19	16(6)	33(7)	22(7)	11(6)	-9(5)	6(5)
Cl110	30(3)	58(4)	36(4)	6(3)	6(3)	17(3)
Cl210	43(3)	30(3)	58(4)	10(3)	20(3)	-5(2)
C110	30(6)	17(5)	34(7)	0(4)	8(5)	4(4)
Cl111	19(3)	48(4)	36(4)	-6(3)	-9(3)	-3(3)
Cl211	33(3)	50(4)	23(3)	-9(3)	-2(3)	3(3)
C111	19(3)	48(4)	36(4)	-6(3)	-9(3)	-3(3)
Cl1A11	19(3)	48(4)	36(4)	-6(3)	-9(3)	-3(3)
Cl2A11	33(3)	50(4)	23(3)	-9(3)	-2(3)	3(3)
C1A11	19(3)	48(4)	36(4)	-6(3)	-9(3)	-3(3)
Cl112	35(3)	24(3)	36(4)	1(3)	-2(3)	10(2)
Cl212	27(3)	46(4)	31(4)	11(3)	-2(3)	-6(3)
Cl1A12	35(3)	24(3)	36(4)	1(3)	-2(3)	10(2)
Cl2A12	27(3)	46(4)	31(4)	11(3)	-2(3)	-6(3)
C112	56(10)	12(6)	39(9)	15(5)	17(8)	11(5)

Table S15. Hydrogen coordinates (x 10^4) and isotropic displacement parameters ($Å^2$x 10^3) for **1b'-OTf**.

	x	y	z	U(eq)
H1A1	9161	5521	8177	21
H1AB1	9185	5621	6462	21
H2A1	8490	5494	8038	21
H2AB1	8687	5185	6983	21
H3A1	8532	5588	4909	21
H3AB1	8162	5419	5736	21
H5A1	7875	5851	4215	21
H5AB1	7787	6268	4894	21
H6A1	8437	6070	3142	24
H6AB1	8093	6349	2652	24
H8A1	8579	6849	2107	38
H8AB1	8864	6501	2482	38
H9A1	8903	7203	4071	38
H9AB1	9141	7144	2574	38
H10A1	9422	6567	3725	38
H10B1	9573	6970	4353	38
H12A1	9768	6573	6597	21
H12B1	9589	6216	5708	21
H13A1	9569	6061	8186	20
H13B1	9370	6463	8583	20
H1A2	9496	4221	2599	16
H1AB2	9395	4209	4315	16
H2A2	9852	3752	3599	16
H2AB2	9534	3552	2583	16
H3A2	9632	3203	4877	16
H3AB2	9478	3569	5772	16
H5A2	9208	2939	6440	16
H5AB2	8798	2821	5774	16
H6A2	8952	3445	7703	16
H6AB2	8668	3090	7999	16
H8A2	8514	3872	8313	20
H8AB2	8145	3603	8526	20
H9A2	7830	3974	6614	20
H9AB2	7900	4201	8122	20
H10A2	8470	4434	7040	20
H10B2	8069	4605	6461	20
H12A2	8463	4800	4190	18
H12B2	8816	4601	5043	18
H13A2	8965	4612	2565	18
H13B2	8561	4418	2151	18
H1A3	5517	4199	7483	18
H1AB3	5618	4234	9193	18
H2A3	5149	3744	8796	18
H2AB3	5432	3524	7705	18
H3A3	5368	3207	9879	13
H3AB3	5522	3575	10756	13
H5A3	5806	2905	11422	12
H5AB3	6225	2816	10753	12
H6A3	6317	3086	13040	12
H6AB3	6035	3439	12655	12
H8A3	6808	3573	13572	13
H8AB3	6456	3854	13235	13
H9A3	7181	3935	11781	13
H9AB3	7079	4165	13260	13
H10A3	6512	4448	11930	13
H10B3	6919	4603	11356	13
H12A3	6522	4794	9166	19
H12B3	6167	4588	9979	19
H13A3	6033	4593	7484	19
H13B3	6446	4409	7108	19
H1A4	6455	5521	10002	17
H1AB4	6826	5389	10913	17
H2A4	6498	5478	13104	17
H2AB4	6283	5174	12077	17
H3A4	5814	5649	11450	17
H3AB4	5815	5557	13171	17
H5A4	5423	6041	13155	23
H5AB4	5592	6456	13593	23
H6A4	5222	6529	11484	23
H6AB4	5454	6190	10674	23
H8A4	5586	6545	8799	17
H8AB4	5416	6946	9358	17
H9A4	6054	7209	9029	17
H9AB4	5820	7110	7558	17
H10A4	6164	6506	7420	17
H10B4	6435	6874	7199	17
H12A4	6596	6082	7922	20
H12B4	6954	6370	7702	20
H13A4	7080	5810	9250	20
H13B4	7213	6216	9910	20
H1A9	4374	5877	12889	28
H1AB9	4700	5774	14068	28
H1A10	5788	5297	6634	33
H1AB10	5910	5625	7781	33
H1A11	9238	5312	1655	41
H1AB11	9105	5638	2780	41
H1AA11	9477	5057	1132	41
H1AC11	9167	5344	1871	41
H1A12	9409	4101	8067	43
H1AB12	9732	4265	9159	43
HA1A12	9665	4118	8917	43
HA1B12	9323	4249	7808	43

[(D$_{12}$-TMC)Fe^{II}(I)]B$_{Ar}$F, **2b-B$_{Ar}$F**

The structure of [(D$_{12}$-TMC)Fe(I)]B$_{Ar}$F (**2b-B$_{Ar}$F**) was solved in the orthorhombic space group *Pbca* and the asymmetric unit contains one full molecule. The cationic complex was found to be fully disordered and two of the CF_3 groups of the anion were also found to be disordered, all over two positions each. All disorder was refined with the help of similarity restraints on distances, similarity restraints on anisotropic displacement parameters and advanced rigid bond restraints. Some atoms were set to the same anisotropic displacement parameters. The disorder ratios were refined and converged to 0.708(3) (complex) and 0.731(8) and 0.52(3) (CF_3 groups).

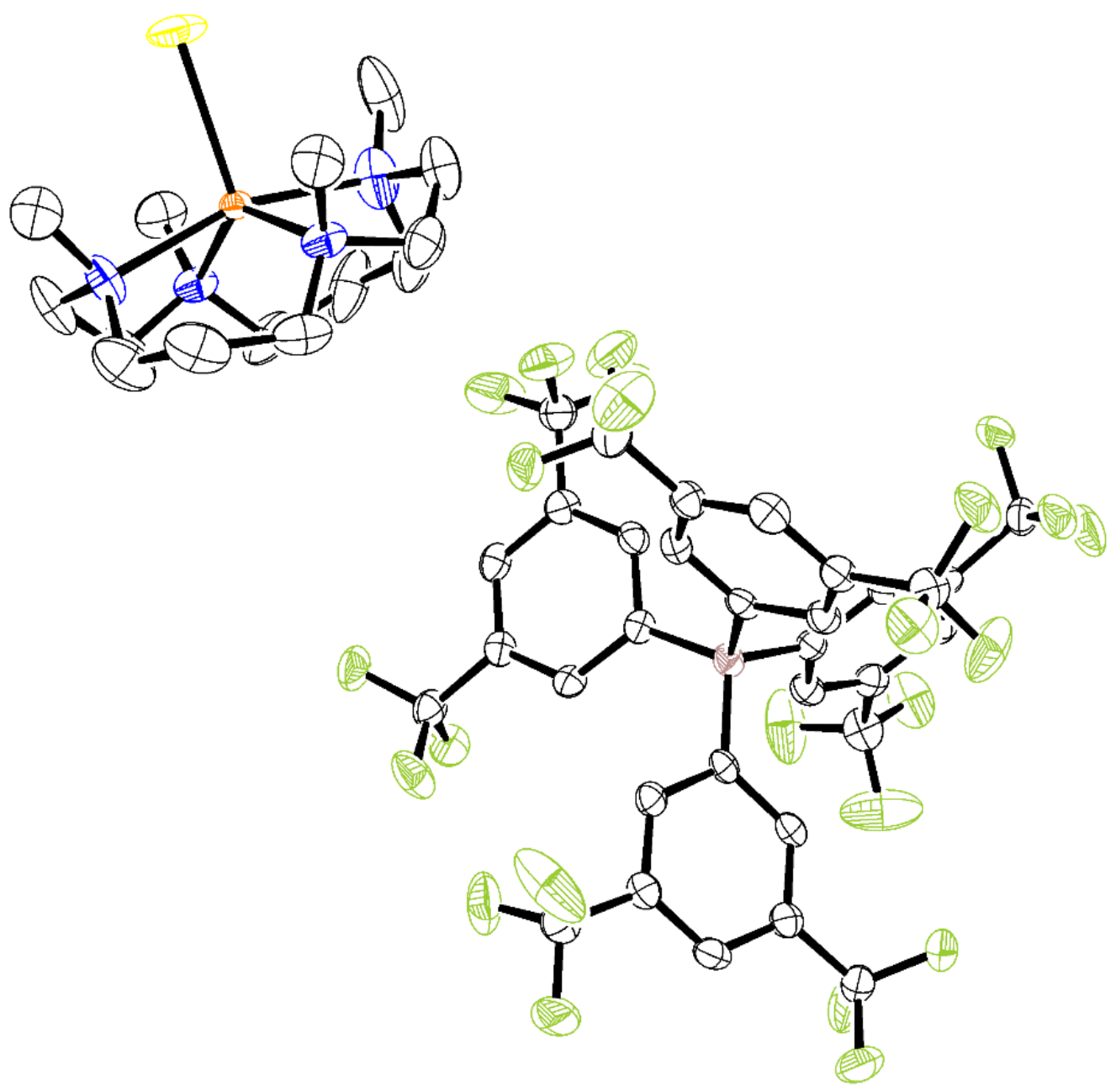

Figure S4. Molecular structure of [(D$_{12}$-TMC)Fe^{II}(I)]B$_{Ar}$F (**2b-B$_{Ar}$F**).

Table S16. Crystal data and structure refinement for **2b-B$_{Ar}$F**.

CCDC No	2062766	
Empirical formula	$C_{46}H_{32}BD_{12}F_{24}FeIN_4$	
Formula weight	1314.48	
Temperature	100(2) K	
Wavelength	0.71073 Å	
Crystal system	Orthorhombic	
Space group	*Pbca*	
Unit cell dimensions	a = 18.2383(7) Å	α = 90°
	b = 19.7877(8) Å	β = 90°
	c = 28.2676(11) Å	γ = 90°
Volume	10201.6(7) Å^3	
Z	8	
Density (calculated)	1.712 Mg/m^3	
Absorption coefficient	1.027 mm^{-1}	
F(000)	5184	
Crystal size	0.741 x 0.214 x 0.191 mm^3	
Theta range for data collection	2.058 to 26.022°.	
Index ranges	$-22 \le h \le 22$, $-24 \le k \le 24$, $-34 \le l \le 34$	
Reflections collected	80415	
Independent reflections	10054 [R(int) = 0.0691]	
Completeness to theta = 25.242°	100.0 %	
Absorption correction	Semi-empirical from equivalents	
Refinement method	Full-matrix least-squares on F^2	
Data / restraints / parameters	10054 / 2255 / 887	
Goodness-of-fit on F^2	1.019	
Final R indices [I>2sigma(I)]	R1 = 0.0455, wR2 = 0.0996	
R indices (all data)	R1 = 0.0808, wR2 = 0.1168	
Largest diff. peak and hole	0.614 and -0.638 eÅ^{-3}	

Table S17. Atomic coordinates (x 10^4) and equivalent isotropic displacement parameters ($Å^2$x 10^3) for **2b-B$_{Ar}$F**.
U(eq) is defined as one third of the trace of the orthogonalized U^{ij} tensor.

	x	y	z	U(eq)
I(1)	2714(1)	3427(1)	10157(1)	53(1)
Fe(1)	2907(3)	3796(2)	9283(1)	21(1)
N(1)	2129(2)	3455(2)	8748(2)	31(1)
N(2)	2325(4)	4818(4)	9310(2)	53(2)
N(3)	3872(3)	4422(3)	9241(2)	31(1)
N(4)	3523(3)	2859(3)	9018(2)	39(2)
C(1)	1876(4)	3970(4)	8403(2)	56(2)
C(2)	1520(4)	4589(3)	8608(3)	70(2)
C(3)	2053(4)	5073(3)	8847(3)	66(2)
C(4)	1760(4)	4879(4)	9669(3)	77(2)
D(4A)	1578	5345	9676	115
D(4B)	1966	4762	9979	115
D(4C)	1355	4572	9595	115
C(5)	2961(3)	5275(3)	9447(3)	52(2)
C(6)	3635(3)	5127(3)	9174(3)	44(2)
C(7)	4299(7)	4399(7)	9700(4)	49(1)
D(7A)	4440	3931	9769	74
D(7B)	3993	4573	9958	74
D(7C)	4741	4678	9671	74
C(8)	4385(4)	4242(4)	8856(2)	43(2)
C(9)	4664(3)	3524(4)	8856(3)	57(2)
C(10)	4116(3)	2988(4)	8691(3)	61(2)
C(11)	3817(3)	2422(3)	9417(2)	49(1)
D(11A)	4023	2006	9285	74
D(11B)	3417	2310	9635	74
D(11C)	4200	2670	9588	74
C(12)	2928(3)	2451(3)	8798(3)	54(2)
C(13)	2468(4)	2907(4)	8472(2)	49(2)
C(14)	1506(9)	3201(8)	9027(7)	48(1)
D(14A)	1104	3076	8814	72
D(14B)	1339	3554	9245	72
D(14C)	1660	2804	9208	72
I(1A)	2920(2)	3243(2)	10132(1)	38(1)
Fe(1A)	2941(6)	3851(6)	9327(4)	21(1)
N(1A)	2384(8)	4830(7)	9480(5)	46(3)
N(2A)	4027(6)	4365(7)	9253(6)	31(1)
N(3A)	3537(8)	3078(7)	8915(5)	36(3)
N(4A)	1985(5)	3716(5)	8853(3)	31(1)
C(1A)	2690(8)	5431(7)	9244(6)	56(3)
C(2A)	3505(8)	5506(8)	9312(6)	54(3)
C(3A)	3979(9)	5034(7)	9035(5)	44(3)
C(4A)	4369(16)	4346(16)	9706(8)	49(1)
D(4AA)	4145	4686	9912	74
D(4AB)	4894	4442	9671	74
D(4AC)	4305	3896	9845	74
C(5A)	4469(10)	3940(8)	8914(7)	44(3)
C(6A)	4325(7)	3217(7)	9006(5)	42(3)
C(7A)	3466(10)	2357(7)	9087(5)	55(4)
D(7AA)	3116	2113	8887	83
D(7AB)	3292	2355	9415	83
D(7AC)	3946	2134	9070	83
C(8A)	3427(7)	3062(7)	8397(4)	40(3)
C(9A)	2645(8)	2976(8)	8254(5)	45(3)
C(10A)	2147(7)	3573(8)	8346(4)	43(3)
C(11A)	1500(20)	3155(19)	9025(16)	48(1)
D(11D)	1129	3054	8786	72
D(11E)	1261	3293	9320	72
D(11F)	1798	2750	9083	72
C(12A)	1554(9)	4370(8)	8872(6)	62(3)
C(13A)	1592(8)	4722(9)	9359(6)	65(3)
C(14A)	2346(8)	4990(8)	10008(5)	49(1)
D(14D)	2124	4609	10176	74
D(14E)	2047	5396	10057	74
D(14F)	2842	5069	10130	74
F(21)	443(2)	7983(1)	5768(1)	79(1)
F(22)	1113(2)	8686(2)	5406(1)	88(1)
F(23)	246(1)	9028(1)	5849(1)	57(1)
F(24)	1518(2)	10249(1)	7094(1)	58(1)
F(25)	2506(1)	9854(1)	7386(1)	45(1)
F(26)	1485(1)	9524(1)	7659(1)	50(1)
F(31)	2136(1)	4940(1)	5986(1)	50(1)
F(32)	1023(1)	5247(1)	5877(1)	42(1)
F(33)	1274(1)	4516(1)	6411(1)	45(1)
F(34)	539(2)	6767(1)	7837(1)	65(1)
F(35)	1302(1)	6076(1)	8117(1)	58(1)
F(36)	342(2)	5708(2)	7773(1)	79(1)
F(41)	4815(1)	9014(1)	7187(1)	64(1)
F(42)	4871(1)	8921(1)	7944(1)	48(1)
F(43)	5617(1)	8370(1)	7509(1)	51(1)
F(44)	4630(8)	6462(10)	8505(6)	61(4)
F(45)	4046(11)	5787(4)	8075(4)	65(3)
F(46)	3476(7)	6450(6)	8539(5)	60(3)
F(44A)	4466(10)	6561(9)	8585(5)	59(4)
F(45A)	3394(5)	6229(11)	8372(8)	85(4)
F(46A)	4327(10)	5844(6)	8037(6)	74(4)
F(51)	5087(4)	5846(3)	6095(2)	90(2)
F(52)	4958(3)	5870(2)	5347(2)	66(2)
F(53)	4163(2)	5399(2)	5764(2)	66(2)
F(51A)	5341(6)	6035(6)	5623(6)	74(4)
F(52A)	4374(9)	5481(6)	5482(6)	76(4)

F(53A)	4743(8)	5585(6)	6170(4)	51(3)
F(54)	4449(2)	8133(1)	4851(1)	56(1)
F(55)	3277(1)	8179(1)	4859(1)	55(1)
F(56)	3900(1)	8812(1)	5319(1)	41(1)
C(21)	2272(2)	8008(2)	6667(1)	24(1)
C(22)	1759(2)	8031(2)	6296(1)	27(1)
C(23)	1303(2)	8582(2)	6226(1)	28(1)
C(24)	1330(2)	9138(2)	6525(1)	28(1)
C(25)	1813(2)	9119(2)	6900(1)	25(1)
C(26)	2274(2)	8565(2)	6970(1)	24(1)
C(27)	785(2)	8574(2)	5813(1)	40(1)
C(28)	1826(2)	9686(2)	7252(1)	31(1)
C(31)	2222(2)	6699(2)	6798(1)	23(1)
C(32)	2118(2)	6162(2)	6480(1)	24(1)
C(33)	1609(2)	5652(2)	6566(1)	25(1)
C(34)	1187(2)	5646(2)	6971(1)	28(1)
C(35)	1275(2)	6175(2)	7292(1)	28(1)
C(36)	1777(2)	6690(2)	7203(1)	26(1)
C(37)	1517(2)	5094(2)	6214(1)	32(1)
C(38)	861(2)	6175(2)	7747(1)	39(1)
C(41)	3374(2)	7388(2)	7159(1)	23(1)
C(42)	3867(2)	7937(2)	7179(1)	25(1)
C(43)	4401(2)	7989(2)	7525(1)	27(1)
C(44)	4479(2)	7490(2)	7871(1)	31(1)
C(45)	4003(2)	6949(2)	7859(1)	28(1)
C(46)	3467(2)	6899(2)	7507(1)	25(1)
C(47)	4917(2)	8570(2)	7538(1)	34(1)
C(48)	4044(2)	6411(2)	8231(1)	39(1)
C(51)	3308(2)	7245(2)	6251(1)	22(1)
C(52)	3746(2)	6666(2)	6194(1)	25(1)
C(53)	4202(2)	6574(2)	5807(1)	28(1)
C(54)	4255(2)	7066(2)	5456(1)	28(1)
C(55)	3848(2)	7655(2)	5512(1)	26(1)
C(56)	3387(2)	7738(2)	5899(1)	25(1)
C(57)	4626(2)	5933(2)	5759(1)	38(1)
C(58)	3869(2)	8187(2)	5141(1)	32(1)
B(21)	2790(2)	7332(2)	6719(1)	24(1)

Table S18. Bond lengths [Å] and angles [°] for **2b-B$_{ArF}$**.

I(1)-Fe(1)	2.600(4)	C(8A)-H(8AC)	0.9900
Fe(1)-N(3)	2.155(6)	C(9A)-C(10A)	1.513(16)
Fe(1)-N(1)	2.182(6)	C(9A)-H(9AA)	0.9900
Fe(1)-N(2)	2.285(7)	C(9A)-H(9AC)	0.9900
Fe(1)-N(4)	2.293(7)	C(10A)-H(10C)	0.9900
N(1)-C(14)	1.472(12)	C(10A)-H(10D)	0.9900
N(1)-C(13)	1.474(8)	C(11A)-D(11D)	0.9800
N(1)-C(1)	1.483(7)	C(11A)-D(11E)	0.9800
N(2)-C(4)	1.453(10)	C(11A)-D(11F)	0.9800
N(2)-C(3)	1.486(9)	C(12A)-C(13A)	1.544(17)
N(2)-C(5)	1.520(9)	C(12A)-H(12C)	0.9900
N(3)-C(6)	1.474(9)	C(12A)-H(12D)	0.9900
N(3)-C(8)	1.479(8)	C(13A)-H(13C)	0.9900
N(3)-C(7)	1.513(8)	C(13A)-H(13D)	0.9900
N(4)-C(10)	1.446(8)	C(14A)-D(14D)	0.9800
N(4)-C(12)	1.489(8)	C(14A)-D(14E)	0.9800
N(4)-C(11)	1.518(9)	C(14A)-D(14F)	0.9800
C(1)-C(2)	1.503(10)	F(21)-C(27)	1.332(5)
C(1)-H(1A)	0.9900	F(22)-C(27)	1.316(5)
C(1)-H(1AB)	0.9900	F(23)-C(27)	1.335(4)
C(2)-C(3)	1.523(9)	F(24)-C(28)	1.325(4)
C(2)-H(2A)	0.9900	F(25)-C(28)	1.339(4)
C(2)-H(2AB)	0.9900	F(26)-C(28)	1.346(4)
C(3)-H(3A)	0.9900	F(31)-C(37)	1.336(4)
C(3)-H(3AB)	0.9900	F(32)-C(37)	1.346(4)
C(4)-D(4A)	0.9800	F(33)-C(37)	1.347(4)
C(4)-D(4B)	0.9800	F(34)-C(38)	1.333(5)
C(4)-D(4C)	0.9800	F(35)-C(38)	1.334(4)
C(5)-C(6)	1.481(9)	F(36)-C(38)	1.324(4)
C(5)-H(5A)	0.9900	F(41)-C(47)	1.338(4)
C(5)-H(5AB)	0.9900	F(42)-C(47)	1.344(4)
C(6)-H(6A)	0.9900	F(43)-C(47)	1.339(4)
C(6)-H(6AB)	0.9900	F(44)-C(48)	1.324(9)
C(7)-D(7A)	0.9800	F(45)-C(48)	1.310(8)
C(7)-D(7B)	0.9800	F(46)-C(48)	1.355(8)
C(7)-D(7C)	0.9800	F(44A)-C(48)	1.297(10)
C(8)-C(9)	1.510(9)	F(45A)-C(48)	1.303(9)
C(8)-H(8A)	0.9900	F(46A)-C(48)	1.350(9)
C(8)-H(8AB)	0.9900	F(51)-C(57)	1.280(5)
C(9)-C(10)	1.529(9)	F(52)-C(57)	1.320(5)
C(9)-H(9A)	0.9900	F(53)-C(57)	1.352(5)
C(9)-H(9AB)	0.9900	F(51A)-C(57)	1.375(9)
C(10)-H(10A)	0.9900	F(52A)-C(57)	1.274(9)
C(10)-H(10B)	0.9900	F(53A)-C(57)	1.366(9)
C(11)-D(11A)	0.9800	F(54)-C(58)	1.343(4)
C(11)-D(11B)	0.9800	F(55)-C(58)	1.341(4)
C(11)-D(11C)	0.9800	F(56)-C(58)	1.337(4)
C(12)-C(13)	1.538(9)	C(21)-C(26)	1.395(5)
C(12)-H(12A)	0.9900	C(21)-C(22)	1.404(5)
C(12)-H(12B)	0.9900	C(21)-B(21)	1.644(5)
C(13)-H(13A)	0.9900	C(22)-C(23)	1.386(5)
C(13)-H(13B)	0.9900	C(22)-H(22)	0.9500
C(14)-D(14A)	0.9800	C(23)-C(24)	1.389(5)
C(14)-D(14B)	0.9800	C(23)-C(27)	1.500(5)
C(14)-D(14C)	0.9800	C(24)-C(25)	1.377(5)
I(1A)-Fe(1A)	2.574(11)	C(24)-H(24)	0.9500
Fe(1A)-N(3A)	2.208(14)	C(25)-C(26)	1.396(5)
Fe(1A)-N(4A)	2.213(13)	C(25)-C(28)	1.499(5)
Fe(1A)-N(1A)	2.231(15)	C(26)-H(26)	0.9500
Fe(1A)-N(2A)	2.237(14)	C(31)-C(36)	1.402(5)
N(1A)-C(1A)	1.475(16)	C(31)-C(32)	1.405(5)
N(1A)-C(13A)	1.500(17)	C(31)-B(21)	1.640(5)
N(1A)-C(14A)	1.527(16)	C(32)-C(33)	1.392(4)
N(2A)-C(4A)	1.423(17)	C(32)-H(32)	0.9500
N(2A)-C(3A)	1.462(16)	C(33)-C(34)	1.379(5)
N(2A)-C(5A)	1.509(16)	C(33)-C(37)	1.496(5)
N(3A)-C(8A)	1.477(14)	C(34)-C(35)	1.394(5)
N(3A)-C(6A)	1.487(16)	C(34)-H(34)	0.9500
N(3A)-C(7A)	1.513(14)	C(35)-C(36)	1.394(5)
N(4A)-C(10A)	1.490(13)	C(35)-C(38)	1.493(5)
N(4A)-C(11A)	1.500(19)	C(36)-H(36)	0.9500
N(4A)-C(12A)	1.515(15)	C(41)-C(46)	1.392(5)
C(1A)-C(2A)	1.505(17)	C(41)-C(42)	1.412(5)
C(1A)-H(1AA)	0.9900	C(41)-B(21)	1.640(5)
C(1A)-H(1AC)	0.9900	C(42)-C(43)	1.383(5)
C(2A)-C(3A)	1.493(15)	C(42)-H(42)	0.9500
C(2A)-H(2AA)	0.9900	C(43)-C(44)	1.397(5)
C(2A)-H(2AC)	0.9900	C(43)-C(47)	1.487(5)
C(3A)-H(3AA)	0.9900	C(44)-C(45)	1.379(5)
C(3A)-H(3AC)	0.9900	C(44)-H(44)	0.9500
C(4A)-D(4AA)	0.9800	C(45)-C(46)	1.397(5)
C(4A)-D(4AB)	0.9800	C(45)-C(48)	1.498(5)
C(4A)-D(4AC)	0.9800	C(46)-H(46)	0.9500
C(5A)-C(6A)	1.477(16)	C(51)-C(56)	1.400(5)
C(5A)-H(5AA)	0.9900	C(51)-C(52)	1.406(4)
C(5A)-H(5AC)	0.9900	C(51)-B(21)	1.635(5)
C(6A)-H(6AA)	0.9900	C(52)-C(53)	1.386(5)
C(6A)-H(6AC)	0.9900	C(52)-H(52)	0.9500
C(7A)-D(7AA)	0.9800	C(53)-C(54)	1.395(5)
C(7A)-D(7AB)	0.9800	C(53)-C(57)	1.491(5)
C(7A)-D(7AC)	0.9800	C(54)-C(55)	1.391(5)
C(8A)-C(9A)	1.493(15)	C(54)-H(54)	0.9500
C(8A)-H(8AA)	0.9900	C(55)-C(56)	1.389(5)

C(55)-C(58)	1.488(5)
C(56)-H(56)	0.9500
N(3)-Fe(1)-N(1)	132.1(3)
N(3)-Fe(1)-N(2)	82.7(3)
N(1)-Fe(1)-N(2)	89.6(3)
N(3)-Fe(1)-N(4)	92.7(3)
N(1)-Fe(1)-N(4)	80.9(2)
N(2)-Fe(1)-N(4)	162.4(3)
N(3)-Fe(1)-I(1)	108.9(2)
N(1)-Fe(1)-I(1)	119.0(2)
N(2)-Fe(1)-I(1)	98.9(2)
N(4)-Fe(1)-I(1)	98.6(2)
C(14)-N(1)-C(13)	110.9(8)
C(14)-N(1)-C(1)	110.3(9)
C(13)-N(1)-C(1)	106.7(5)
C(14)-N(1)-Fe(1)	103.7(8)
C(13)-N(1)-Fe(1)	108.8(4)
C(1)-N(1)-Fe(1)	116.5(4)
C(4)-N(2)-C(3)	110.5(7)
C(4)-N(2)-C(5)	108.2(6)
C(3)-N(2)-C(5)	106.1(6)
C(4)-N(2)-Fe(1)	115.2(5)
C(3)-N(2)-Fe(1)	115.3(5)
C(5)-N(2)-Fe(1)	100.4(4)
C(6)-N(3)-C(8)	108.5(6)
C(6)-N(3)-C(7)	106.9(7)
C(8)-N(3)-C(7)	107.3(6)
C(6)-N(3)-Fe(1)	108.1(4)
C(8)-N(3)-Fe(1)	114.8(5)
C(7)-N(3)-Fe(1)	110.9(6)
C(10)-N(4)-C(12)	112.0(6)
C(10)-N(4)-C(11)	108.1(5)
C(12)-N(4)-C(11)	105.0(6)
C(10)-N(4)-Fe(1)	115.7(5)
C(12)-N(4)-Fe(1)	102.6(4)
C(11)-N(4)-Fe(1)	113.0(4)
N(1)-C(1)-C(2)	116.1(6)
N(1)-C(1)-H(1A)	108.3
C(2)-C(1)-H(1A)	108.3
N(1)-C(1)-H(1AB)	108.3
C(2)-C(1)-H(1AB)	108.3
H(1A)-C(1)-H(1AB)	107.4
C(1)-C(2)-C(3)	114.0(6)
C(1)-C(2)-H(2A)	108.7
C(3)-C(2)-H(2A)	108.7
C(1)-C(2)-H(2AB)	108.7
C(3)-C(2)-H(2AB)	108.7
H(2A)-C(2)-H(2AB)	107.6
N(2)-C(3)-C(2)	113.0(6)
N(2)-C(3)-H(3A)	109.0
C(2)-C(3)-H(3A)	109.0
N(2)-C(3)-H(3AB)	109.0
C(2)-C(3)-H(3AB)	109.0
H(3A)-C(3)-H(3AB)	107.8
N(2)-C(4)-D(4A)	109.5
N(2)-C(4)-D(4B)	109.5
D(4A)-C(4)-D(4B)	109.5
N(2)-C(4)-D(4C)	109.5
D(4A)-C(4)-D(4C)	109.5
D(4B)-C(4)-D(4C)	109.5
C(6)-C(5)-N(2)	112.4(6)
C(6)-C(5)-H(5A)	109.1
N(2)-C(5)-H(5A)	109.1
C(6)-C(5)-H(5AB)	109.1
N(2)-C(5)-H(5AB)	109.1
H(5A)-C(5)-H(5AB)	107.8
N(3)-C(6)-C(5)	111.3(5)
N(3)-C(6)-H(6A)	109.4
C(5)-C(6)-H(6A)	109.4
N(3)-C(6)-H(6AB)	109.4
C(5)-C(6)-H(6AB)	109.4
H(6A)-C(6)-H(6AB)	108.0
N(3)-C(7)-D(7A)	109.5
N(3)-C(7)-D(7B)	109.5
D(7A)-C(7)-D(7B)	109.5
N(3)-C(7)-D(7C)	109.5
D(7A)-C(7)-D(7C)	109.5
D(7B)-C(7)-D(7C)	109.5
N(3)-C(8)-C(9)	116.0(6)
N(3)-C(8)-H(8A)	108.3
C(9)-C(8)-H(8A)	108.3
N(3)-C(8)-H(8AB)	108.3
C(9)-C(8)-H(8AB)	108.3
H(8A)-C(8)-H(8AB)	107.4
C(8)-C(9)-C(10)	115.7(6)
C(8)-C(9)-H(9A)	108.4
C(10)-C(9)-H(9A)	108.4
C(8)-C(9)-H(9AB)	108.4
C(10)-C(9)-H(9AB)	108.4
H(9A)-C(9)-H(9AB)	107.4
N(4)-C(10)-C(9)	114.6(6)
N(4)-C(10)-H(10A)	108.6
C(9)-C(10)-H(10A)	108.6
N(4)-C(10)-H(10B)	108.6

C(9)-C(10)-H(10B)	108.6
H(10A)-C(10)-H(10B)	107.6
N(4)-C(11)-D(11A)	109.5
N(4)-C(11)-D(11B)	109.5
D(11A)-C(11)-D(11B)	109.5
N(4)-C(11)-D(11C)	109.5
D(11A)-C(11)-D(11C)	109.5
D(11B)-C(11)-D(11C)	109.5
N(4)-C(12)-C(13)	109.3(5)
N(4)-C(12)-H(12A)	109.8
C(13)-C(12)-H(12A)	109.8
N(4)-C(12)-H(12B)	109.8
C(13)-C(12)-H(12B)	109.8
H(12A)-C(12)-H(12B)	108.3
N(1)-C(13)-C(12)	110.1(5)
N(1)-C(13)-H(13A)	109.6
C(12)-C(13)-H(13A)	109.6
N(1)-C(13)-H(13B)	109.6
C(12)-C(13)-H(13B)	109.6
H(13A)-C(13)-H(13B)	108.2
N(1)-C(14)-D(14A)	109.5
N(1)-C(14)-D(14B)	109.5
D(14A)-C(14)-D(14B)	109.5
N(1)-C(14)-D(14C)	109.5
D(14A)-C(14)-D(14C)	109.5
D(14B)-C(14)-D(14C)	109.5
N(3A)-Fe(1A)-N(4A)	89.2(6)
N(3A)-Fe(1A)-N(1A)	158.2(7)
N(4A)-Fe(1A)-N(1A)	82.2(6)
N(3A)-Fe(1A)-N(2A)	80.2(7)
N(4A)-Fe(1A)-N(2A)	134.1(7)
N(1A)-Fe(1A)-N(2A)	91.5(7)
N(3A)-Fe(1A)-I(1A)	98.6(5)
N(4A)-Fe(1A)-I(1A)	117.8(5)
N(1A)-Fe(1A)-I(1A)	103.1(5)
N(2A)-Fe(1A)-I(1A)	107.9(6)
C(1A)-N(1A)-C(13A)	112.1(13)
C(1A)-N(1A)-C(14A)	107.1(12)
C(13A)-N(1A)-C(14A)	102.1(12)
C(1A)-N(1A)-Fe(1A)	116.1(10)
C(13A)-N(1A)-Fe(1A)	105.6(10)
C(14A)-N(1A)-Fe(1A)	113.0(9)
C(4A)-N(2A)-C(3A)	115.5(16)
C(4A)-N(2A)-C(5A)	108.7(15)
C(3A)-N(2A)-C(5A)	105.6(12)
C(4A)-N(2A)-Fe(1A)	107.1(15)
C(3A)-N(2A)-Fe(1A)	113.4(9)
C(5A)-N(2A)-Fe(1A)	106.2(10)
C(8A)-N(3A)-C(6A)	107.9(11)
C(8A)-N(3A)-C(7A)	106.6(11)
C(6A)-N(3A)-C(7A)	101.6(11)
C(8A)-N(3A)-Fe(1A)	118.1(9)
C(6A)-N(3A)-Fe(1A)	104.9(9)
C(7A)-N(3A)-Fe(1A)	116.3(10)
C(10A)-N(4A)-C(11A)	106.7(19)
C(10A)-N(4A)-C(12A)	107.3(10)
C(11A)-N(4A)-C(12A)	108(2)
C(10A)-N(4A)-Fe(1A)	116.7(8)
C(11A)-N(4A)-Fe(1A)	110.9(18)
C(12A)-N(4A)-Fe(1A)	106.5(8)
N(1A)-C(1A)-C(2A)	113.2(13)
N(1A)-C(1A)-H(1AA)	108.9
C(2A)-C(1A)-H(1AA)	108.9
N(1A)-C(1A)-H(1AC)	108.9
C(2A)-C(1A)-H(1AC)	108.9
H(1AA)-C(1A)-H(1AC)	107.7
C(3A)-C(2A)-C(1A)	116.3(13)
C(3A)-C(2A)-H(2AA)	108.2
C(1A)-C(2A)-H(2AA)	108.2
C(3A)-C(2A)-H(2AC)	108.2
C(1A)-C(2A)-H(2AC)	108.2
H(2AA)-C(2A)-H(2AC)	107.4
N(2A)-C(3A)-C(2A)	112.3(13)
N(2A)-C(3A)-H(3AA)	109.1
C(2A)-C(3A)-H(3AA)	109.1
N(2A)-C(3A)-H(3AC)	109.1
C(2A)-C(3A)-H(3AC)	109.1
H(3AA)-C(3A)-H(3AC)	107.9
N(2A)-C(4A)-D(4AA)	109.5
N(2A)-C(4A)-D(4AB)	109.5
D(4AA)-C(4A)-D(4AB)	109.5
N(2A)-C(4A)-D(4AC)	109.5
D(4AA)-C(4A)-D(4AC)	109.5
D(4AB)-C(4A)-D(4AC)	109.5
C(6A)-C(5A)-N(2A)	109.5(12)
C(6A)-C(5A)-H(5AA)	109.8
N(2A)-C(5A)-H(5AA)	109.8
C(6A)-C(5A)-H(5AC)	109.8
N(2A)-C(5A)-H(5AC)	109.8
H(5AA)-C(5A)-H(5AC)	108.2
C(5A)-C(6A)-N(3A)	108.7(12)
C(5A)-C(6A)-H(6AA)	110.0
N(3A)-C(6A)-H(6AA)	110.0
C(5A)-C(6A)-H(6AC)	110.0
N(3A)-C(6A)-H(6AC)	110.0

H(6AA)-C(6A)-H(6AC)	108.3
N(3A)-C(7A)-D(7AA)	109.5
N(3A)-C(7A)-D(7AB)	109.5
D(7AA)-C(7A)-D(7AB)	109.5
N(3A)-C(7A)-D(7AC)	109.5
D(7AA)-C(7A)-D(7AC)	109.5
D(7AB)-C(7A)-D(7AC)	109.5
N(3A)-C(8A)-C(9A)	113.6(11)
N(3A)-C(8A)-H(8AA)	108.8
C(9A)-C(8A)-H(8AA)	108.8
N(3A)-C(8A)-H(8AC)	108.8
C(9A)-C(8A)-H(8AC)	108.8
H(8AA)-C(8A)-H(8AC)	107.7
C(8A)-C(9A)-C(10A)	115.9(12)
C(8A)-C(9A)-H(9AA)	108.3
C(10A)-C(9A)-H(9AA)	108.3
C(8A)-C(9A)-H(9AC)	108.3
C(10A)-C(9A)-H(9AC)	108.3
H(9AA)-C(9A)-H(9AC)	107.4
N(4A)-C(10A)-C(9A)	115.7(11)
N(4A)-C(10A)-H(10C)	108.4
C(9A)-C(10A)-H(10C)	108.4
N(4A)-C(10A)-H(10D)	108.4
C(9A)-C(10A)-H(10D)	108.4
H(10C)-C(10A)-H(10D)	107.4
N(4A)-C(11A)-D(11D)	109.5
N(4A)-C(11A)-D(11E)	109.5
D(11D)-C(11A)-D(11E)	109.5
N(4A)-C(11A)-D(11F)	109.5
D(11D)-C(11A)-D(11F)	109.5
D(11E)-C(11A)-D(11F)	109.5
N(4A)-C(12A)-C(13A)	113.1(11)
N(4A)-C(12A)-H(12C)	109.0
C(13A)-C(12A)-H(12C)	109.0
N(4A)-C(12A)-H(12D)	109.0
C(13A)-C(12A)-H(12D)	109.0
H(12C)-C(12A)-H(12D)	107.8
N(1A)-C(13A)-C(12A)	108.2(12)
N(1A)-C(13A)-H(13C)	110.1
C(12A)-C(13A)-H(13C)	110.1
N(1A)-C(13A)-H(13D)	110.1
C(12A)-C(13A)-H(13D)	110.1
H(13C)-C(13A)-H(13D)	108.4
N(1A)-C(14A)-D(14D)	109.5
N(1A)-C(14A)-D(14E)	109.5
D(14D)-C(14A)-D(14E)	109.5
N(1A)-C(14A)-D(14F)	109.5
D(14D)-C(14A)-D(14F)	109.5
D(14E)-C(14A)-D(14F)	109.5
C(26)-C(21)-C(22)	115.7(3)
C(26)-C(21)-B(21)	125.8(3)
C(22)-C(21)-B(21)	118.4(3)
C(23)-C(22)-C(21)	122.1(3)
C(23)-C(22)-H(22)	118.9
C(21)-C(22)-H(22)	118.9
C(22)-C(23)-C(24)	121.0(3)
C(22)-C(23)-C(27)	118.8(3)
C(24)-C(23)-C(27)	120.2(3)
C(25)-C(24)-C(23)	118.0(3)
C(25)-C(24)-H(24)	121.0
C(23)-C(24)-H(24)	121.0
C(24)-C(25)-C(26)	121.0(3)
C(24)-C(25)-C(28)	120.0(3)
C(26)-C(25)-C(28)	118.9(3)
C(21)-C(26)-C(25)	122.1(3)
C(21)-C(26)-H(26)	118.9
C(25)-C(26)-H(26)	119.0
F(22)-C(27)-F(21)	106.0(4)
F(22)-C(27)-F(23)	106.7(3)
F(21)-C(27)-F(23)	104.6(3)
F(22)-C(27)-C(23)	113.1(3)
F(21)-C(27)-C(23)	112.3(3)
F(23)-C(27)-C(23)	113.4(3)
F(24)-C(28)-F(25)	106.1(3)
F(24)-C(28)-F(26)	107.0(3)
F(25)-C(28)-F(26)	104.1(3)
F(24)-C(28)-C(25)	113.5(3)
F(25)-C(28)-C(25)	112.9(3)
F(26)-C(28)-C(25)	112.5(3)
C(36)-C(31)-C(32)	115.6(3)
C(36)-C(31)-B(21)	119.0(3)
C(32)-C(31)-B(21)	125.3(3)
C(33)-C(32)-C(31)	121.9(3)
C(33)-C(32)-H(32)	119.1
C(31)-C(32)-H(32)	119.1
C(34)-C(33)-C(32)	121.5(3)
C(34)-C(33)-C(37)	118.8(3)
C(32)-C(33)-C(37)	119.6(3)
C(33)-C(34)-C(35)	118.0(3)
C(33)-C(34)-H(34)	121.0
C(35)-C(34)-H(34)	121.0
C(36)-C(35)-C(34)	120.5(3)
C(36)-C(35)-C(38)	119.2(3)
C(34)-C(35)-C(38)	120.2(3)

C(35)-C(36)-C(31)	122.5(3)
C(35)-C(36)-H(36)	118.7
C(31)-C(36)-H(36)	118.7
F(31)-C(37)-F(32)	105.9(3)
F(31)-C(37)-F(33)	106.5(3)
F(32)-C(37)-F(33)	105.2(3)
F(31)-C(37)-C(33)	113.3(3)
F(32)-C(37)-C(33)	112.4(3)
F(33)-C(37)-C(33)	112.9(3)
F(36)-C(38)-F(34)	106.7(3)
F(36)-C(38)-F(35)	106.5(3)
F(34)-C(38)-F(35)	104.2(3)
F(36)-C(38)-C(35)	114.2(3)
F(34)-C(38)-C(35)	112.9(3)
F(35)-C(38)-C(35)	111.7(3)
C(46)-C(41)-C(42)	115.4(3)
C(46)-C(41)-B(21)	124.7(3)
C(42)-C(41)-B(21)	119.8(3)
C(43)-C(42)-C(41)	122.3(3)
C(43)-C(42)-H(42)	118.9
C(41)-C(42)-H(42)	118.9
C(42)-C(43)-C(44)	121.0(3)
C(42)-C(43)-C(47)	121.3(3)
C(44)-C(43)-C(47)	117.7(3)
C(45)-C(44)-C(43)	117.8(3)
C(45)-C(44)-H(44)	121.1
C(43)-C(44)-H(44)	121.1
C(44)-C(45)-C(46)	120.9(3)
C(44)-C(45)-C(48)	120.1(3)
C(46)-C(45)-C(48)	119.0(3)
C(41)-C(46)-C(45)	122.7(3)
C(41)-C(46)-H(46)	118.7
C(45)-C(46)-H(46)	118.7
F(41)-C(47)-F(43)	106.5(3)
F(41)-C(47)-F(42)	106.4(3)
F(43)-C(47)-F(42)	105.3(3)
F(41)-C(47)-C(43)	113.8(3)
F(43)-C(47)-C(43)	112.0(3)
F(42)-C(47)-C(43)	112.3(3)
F(44A)-C(48)-F(45A)	111.5(10)
F(45)-C(48)-F(44)	105.5(9)
F(44A)-C(48)-F(46A)	106.1(9)
F(45A)-C(48)-F(46A)	104.0(8)
F(45)-C(48)-F(46)	105.7(7)
F(44)-C(48)-F(46)	103.7(8)
F(44A)-C(48)-C(45)	114.2(8)
F(45A)-C(48)-C(45)	111.4(6)
F(45)-C(48)-C(45)	115.7(6)
F(44)-C(48)-C(45)	113.4(8)
F(46A)-C(48)-C(45)	109.0(8)
F(46)-C(48)-C(45)	111.8(6)
C(56)-C(51)-C(52)	115.2(3)
C(56)-C(51)-B(21)	124.1(3)
C(52)-C(51)-B(21)	120.5(3)
C(53)-C(52)-C(51)	122.7(3)
C(53)-C(52)-H(52)	118.7
C(51)-C(52)-H(52)	118.7
C(52)-C(53)-C(54)	120.8(3)
C(52)-C(53)-C(57)	119.7(3)
C(54)-C(53)-C(57)	119.5(3)
C(55)-C(54)-C(53)	117.7(3)
C(55)-C(54)-H(54)	121.1
C(53)-C(54)-H(54)	121.1
C(56)-C(55)-C(54)	120.9(3)
C(56)-C(55)-C(58)	119.2(3)
C(54)-C(55)-C(58)	119.8(3)
C(55)-C(56)-C(51)	122.6(3)
C(55)-C(56)-H(56)	118.7
C(51)-C(56)-H(56)	118.7
F(51)-C(57)-F(52)	109.9(4)
F(51)-C(57)-F(53)	107.3(5)
F(52)-C(57)-F(53)	102.9(4)
F(52A)-C(57)-F(53A)	103.0(9)
F(52A)-C(57)-F(51A)	105.8(9)
F(53A)-C(57)-F(51A)	99.4(7)
F(52A)-C(57)-C(53)	117.8(6)
F(51)-C(57)-C(53)	112.8(4)
F(52)-C(57)-C(53)	113.5(3)
F(53)-C(57)-C(53)	109.8(3)
F(53A)-C(57)-C(53)	115.6(6)
F(51A)-C(57)-C(53)	113.1(5)
F(56)-C(58)-F(55)	105.5(3)
F(56)-C(58)-F(54)	105.7(3)
F(55)-C(58)-F(54)	105.7(3)
F(56)-C(58)-C(55)	113.0(3)
F(55)-C(58)-C(55)	113.0(3)
F(54)-C(58)-C(55)	113.3(3)
C(51)-B(21)-C(41)	104.1(3)
C(51)-B(21)-C(31)	113.2(3)
C(41)-B(21)-C(31)	111.1(3)
C(51)-B(21)-C(21)	110.1(3)
C(41)-B(21)-C(21)	112.8(3)
C(31)-B(21)-C(21)	105.7(3)

Table S19. Anisotropic displacement parameters ($Å^2$x 10^3) for **2b-B$_{ArF}$**.
The anisotropic displacement factor exponent takes the form: $-2p^2[h^2 a^{*2}U^{11} + ... + 2 h k a^* b^* U^{12}]$

	U^{11}	U^{22}	U^{33}	U^{23}	U^{13}	U^{12}
I(1)	63(1)	74(1)	21(1)	4(1)	5(1)	-34(1)
Fe(1)	23(1)	24(1)	16(1)	1(1)	0(1)	-2(1)
N(1)	26(2)	43(2)	26(1)	3(1)	-4(1)	-7(1)
N(2)	39(3)	41(3)	78(4)	-6(3)	-17(3)	4(2)
N(3)	26(2)	43(2)	26(1)	3(1)	-4(1)	-7(1)
N(4)	32(2)	32(3)	53(3)	-17(3)	-2(2)	6(2)
C(1)	60(4)	60(4)	47(3)	25(3)	-30(3)	-20(3)
C(2)	62(4)	45(4)	103(5)	33(4)	-43(4)	-9(3)
C(3)	55(4)	40(3)	102(4)	20(3)	-35(3)	0(3)
C(4)	46(4)	45(4)	140(7)	-39(4)	18(4)	1(3)
C(5)	43(3)	33(3)	80(5)	-13(3)	-15(3)	1(3)
C(6)	40(3)	36(3)	55(4)	7(3)	-6(3)	-13(3)
C(7)	43(2)	53(2)	51(2)	0(2)	-12(2)	-3(2)
C(8)	26(3)	71(4)	33(3)	2(3)	0(2)	-13(3)
C(9)	29(3)	83(5)	57(4)	-13(4)	8(3)	-1(3)
C(10)	40(3)	77(4)	66(4)	-25(3)	7(3)	3(3)
C(11)	43(2)	53(2)	51(2)	0(2)	-12(2)	-3(2)
C(12)	47(3)	40(3)	76(4)	-35(3)	-2(3)	2(3)
C(13)	42(3)	59(4)	47(4)	-24(3)	4(3)	-16(3)
C(14)	34(2)	59(3)	51(3)	3(2)	1(2)	-20(2)
I(1A)	50(1)	41(1)	22(1)	8(1)	7(1)	-7(1)
Fe(1A)	23(1)	24(1)	16(1)	1(1)	0(1)	-2(1)
N(1A)	53(6)	34(5)	52(5)	1(5)	-5(5)	22(4)
N(2A)	26(2)	43(2)	26(1)	3(1)	-4(1)	-7(1)
N(3A)	36(5)	39(6)	34(5)	-1(5)	3(4)	5(4)
N(4A)	26(2)	43(2)	26(1)	3(1)	-4(1)	-7(1)
C(1A)	70(6)	42(6)	57(6)	1(5)	4(6)	19(5)
C(2A)	73(7)	46(7)	42(7)	-6(6)	-1(6)	2(6)
C(3A)	49(7)	37(5)	46(6)	-3(5)	-1(6)	-12(5)
C(4A)	43(2)	53(2)	51(2)	0(2)	-12(2)	-3(2)
C(5A)	25(6)	57(6)	49(6)	-11(6)	-2(5)	2(6)
C(6A)	33(5)	59(6)	35(6)	-7(6)	-1(5)	12(5)
C(7A)	98(11)	37(7)	30(7)	-9(6)	12(8)	9(7)
C(8A)	49(5)	41(6)	30(5)	-12(4)	3(4)	2(5)
C(9A)	59(6)	52(6)	24(6)	2(6)	-6(5)	-17(5)
C(10A)	36(6)	68(7)	23(4)	10(5)	-17(4)	-14(5)
C(11A)	34(2)	59(3)	51(3)	3(2)	1(2)	-20(2)
C(12A)	45(6)	71(6)	70(7)	13(6)	-16(6)	11(5)
C(13A)	56(6)	69(7)	72(7)	3(6)	-9(6)	25(6)
C(14A)	43(2)	53(2)	51(2)	0(2)	-12(2)	-3(2)
F(21)	82(2)	44(2)	111(2)	-7(2)	-65(2)	-4(1)
F(22)	51(2)	179(3)	34(1)	9(2)	-10(1)	10(2)
F(23)	45(1)	54(2)	73(2)	-7(1)	-24(1)	15(1)
F(24)	92(2)	25(1)	57(2)	-10(1)	-14(1)	17(1)
F(25)	42(1)	40(1)	51(1)	-16(1)	7(1)	-16(1)
F(26)	66(2)	41(1)	42(1)	-16(1)	28(1)	-19(1)
F(31)	40(1)	44(1)	67(2)	-28(1)	10(1)	-12(1)
F(32)	52(1)	42(1)	31(1)	-2(1)	-17(1)	-12(1)
F(33)	66(2)	27(1)	42(1)	2(1)	-8(1)	-16(1)
F(34)	76(2)	62(2)	57(2)	4(1)	35(1)	15(1)
F(35)	68(2)	75(2)	31(1)	12(1)	2(1)	-5(1)
F(36)	79(2)	97(2)	61(2)	-26(2)	34(2)	-61(2)
F(41)	69(2)	56(2)	66(2)	24(1)	-30(1)	-40(1)
F(42)	44(1)	47(1)	54(1)	-23(1)	1(1)	-15(1)
F(43)	26(1)	57(2)	69(2)	-15(1)	5(1)	-14(1)
F(44)	50(4)	77(7)	57(6)	22(5)	-25(4)	-2(4)
F(45)	137(10)	27(3)	32(4)	3(2)	-12(5)	0(4)
F(46)	68(5)	50(5)	61(5)	26(4)	34(4)	21(4)
F(44A)	91(8)	48(5)	39(4)	11(3)	-22(5)	-28(6)
F(45A)	40(4)	94(10)	121(10)	79(7)	-16(4)	-13(4)
F(46A)	88(8)	39(4)	95(7)	2(4)	-28(5)	22(5)
F(51)	98(4)	95(5)	76(3)	-44(3)	-56(3)	75(3)
F(52)	92(4)	47(2)	57(3)	8(2)	41(3)	36(2)
F(53)	58(2)	25(2)	116(4)	1(2)	29(3)	5(2)
F(51A)	60(5)	55(6)	107(9)	23(6)	43(6)	32(5)
F(52A)	92(8)	55(6)	82(8)	-33(6)	-43(7)	36(6)
F(53A)	62(7)	40(6)	50(5)	11(4)	8(5)	23(5)
F(54)	74(2)	48(1)	45(1)	16(1)	29(1)	12(1)
F(55)	70(2)	42(1)	54(2)	16(1)	-28(1)	-14(1)
F(56)	58(1)	25(1)	40(1)	0(1)	4(1)	-7(1)
C(21)	21(2)	24(2)	26(2)	-2(1)	5(1)	-6(1)
C(22)	24(2)	30(2)	28(2)	-5(2)	2(1)	0(1)
C(23)	22(2)	32(2)	31(2)	0(2)	2(1)	-6(1)
C(24)	27(2)	25(2)	33(2)	4(2)	8(1)	-1(2)
C(25)	27(2)	23(2)	25(2)	-1(1)	10(1)	-5(1)
C(26)	24(2)	25(2)	23(2)	-1(1)	1(1)	-5(1)
C(27)	31(2)	41(2)	47(2)	-2(2)	-6(2)	5(2)
C(28)	33(2)	23(2)	37(2)	-1(2)	4(2)	-4(2)
C(31)	21(2)	23(2)	25(2)	1(1)	-5(1)	-2(1)
C(32)	24(2)	27(2)	21(2)	2(1)	-6(1)	-1(1)
C(33)	25(2)	25(2)	26(2)	1(1)	-9(1)	-2(1)
C(34)	26(2)	25(2)	33(2)	4(2)	-7(1)	-5(1)
C(35)	25(2)	32(2)	28(2)	0(2)	-2(1)	-5(2)
C(36)	29(2)	24(2)	24(2)	-4(1)	-3(1)	-1(1)
C(37)	35(2)	29(2)	33(2)	1(2)	-4(2)	-10(2)
C(38)	46(2)	36(2)	35(2)	-3(2)	3(2)	-11(2)
C(41)	21(2)	25(2)	24(2)	-7(1)	4(1)	2(1)
C(42)	24(2)	26(2)	26(2)	-4(1)	2(1)	-3(1)
C(43)	22(2)	30(2)	30(2)	-5(2)	2(1)	-4(1)
C(44)	26(2)	36(2)	32(2)	-5(2)	-7(2)	0(2)

C(45)	28(2)	26(2)	31(2)	-2(1)	-2(1)	3(1)
C(46)	23(2)	22(2)	30(2)	-4(1)	1(1)	-1(1)
C(47)	30(2)	39(2)	34(2)	-3(2)	-6(2)	-9(2)
C(48)	38(2)	34(2)	43(2)	4(2)	-9(2)	-1(2)
C(51)	22(2)	21(2)	25(2)	-7(1)	-4(1)	-4(1)
C(52)	27(2)	22(2)	25(2)	-3(1)	-3(1)	-4(1)
C(53)	29(2)	26(2)	28(2)	-4(1)	-2(1)	0(1)
C(54)	28(2)	33(2)	24(2)	-7(1)	4(1)	-2(2)
C(55)	26(2)	27(2)	26(2)	-4(1)	-3(1)	-6(1)
C(56)	25(2)	22(2)	26(2)	-7(1)	-1(1)	-1(1)
C(57)	45(2)	33(2)	36(2)	-1(2)	6(2)	7(2)
C(58)	38(2)	26(2)	31(2)	1(2)	0(2)	-3(2)
B(21)	23(2)	23(2)	26(2)	-3(2)	-2(2)	-3(2)

Table S20. Hydrogen coordinates (x 10^4) and isotropic displacement parameters ($Å^2$x 10^3) for **2b-B$_{Ar}$F**.

	x	y	z	U(eq)
H(1A)	1523	3752	8185	67
H(1AB)	2303	4115	8212	67
H(2A)	1147	4446	8842	84
H(2AB)	1262	4833	8352	84
H(3A)	2476	5149	8635	79
H(3AB)	1806	5513	8897	79
H(5A)	2818	5751	9394	62
H(5AB)	3064	5218	9789	62
H(6A)	3542	5209	8834	53
H(6AB)	4031	5436	9277	53
H(8A)	4813	4550	8874	52
H(8AB)	4138	4328	8550	52
H(9A)	4823	3409	9181	68
H(9AB)	5102	3501	8650	68
H(10A)	3905	3133	8385	73
H(10B)	4384	2560	8636	73
H(12A)	2612	2255	9048	65
H(12B)	3143	2075	8613	65
H(13A)	2785	3101	8222	59
H(13B)	2081	2635	8316	59
H(1AA)	2443	5840	9368	68
H(1AC)	2584	5403	8901	68
H(2AA)	3643	5974	9227	64
H(2AC)	3615	5443	9652	64
H(3AA)	3778	4987	8711	53
H(3AC)	4477	5228	9009	53
H(5AA)	4333	4052	8585	52
H(5AC)	4998	4036	8956	52
H(6AA)	4447	3106	9339	51
H(6AC)	4634	2934	8797	51
H(8AA)	3719	2687	8263	48
H(8AC)	3615	3489	8260	48
H(9AA)	2444	2578	8422	54
H(9AC)	2631	2873	7911	54
H(10C)	2373	3980	8205	51
H(10D)	1676	3494	8180	51
H(12C)	1035	4274	8794	74
H(12D)	1747	4682	8627	74
H(13C)	1332	5161	9347	79
H(13D)	1355	4436	9602	79
H(22)	1724	7656	6088	33
H(24)	1023	9519	6474	34
H(26)	2600	8568	7232	29
H(32)	2402	6146	6199	29
H(34)	846	5292	7029	34
H(36)	1820	7049	7424	31
H(42)	3831	8283	6947	30
H(44)	4847	7522	8108	37
H(46)	3153	6516	7507	30
H(52)	3730	6325	6430	30
H(54)	4559	7001	5186	34
H(56)	3114	8145	5926	30

[(D$_{12}$-TMC)Fe^{II}(F)]B$_{Ar}$F, **3b-B$_{Ar}$F**

The asymmetric unit contains one complex molecule and one disordered B$_{Ar}$F anion. The disordered CF_3 group was refined in four different positions with population of 0.203(3), 0.385(3), 0.166(3) and 0.244(3) using PART/SUMP commands and some restraints (SADI, SIMU, RIGU). The reflection -1 0 1 was removed from the refinement using OMIT command.

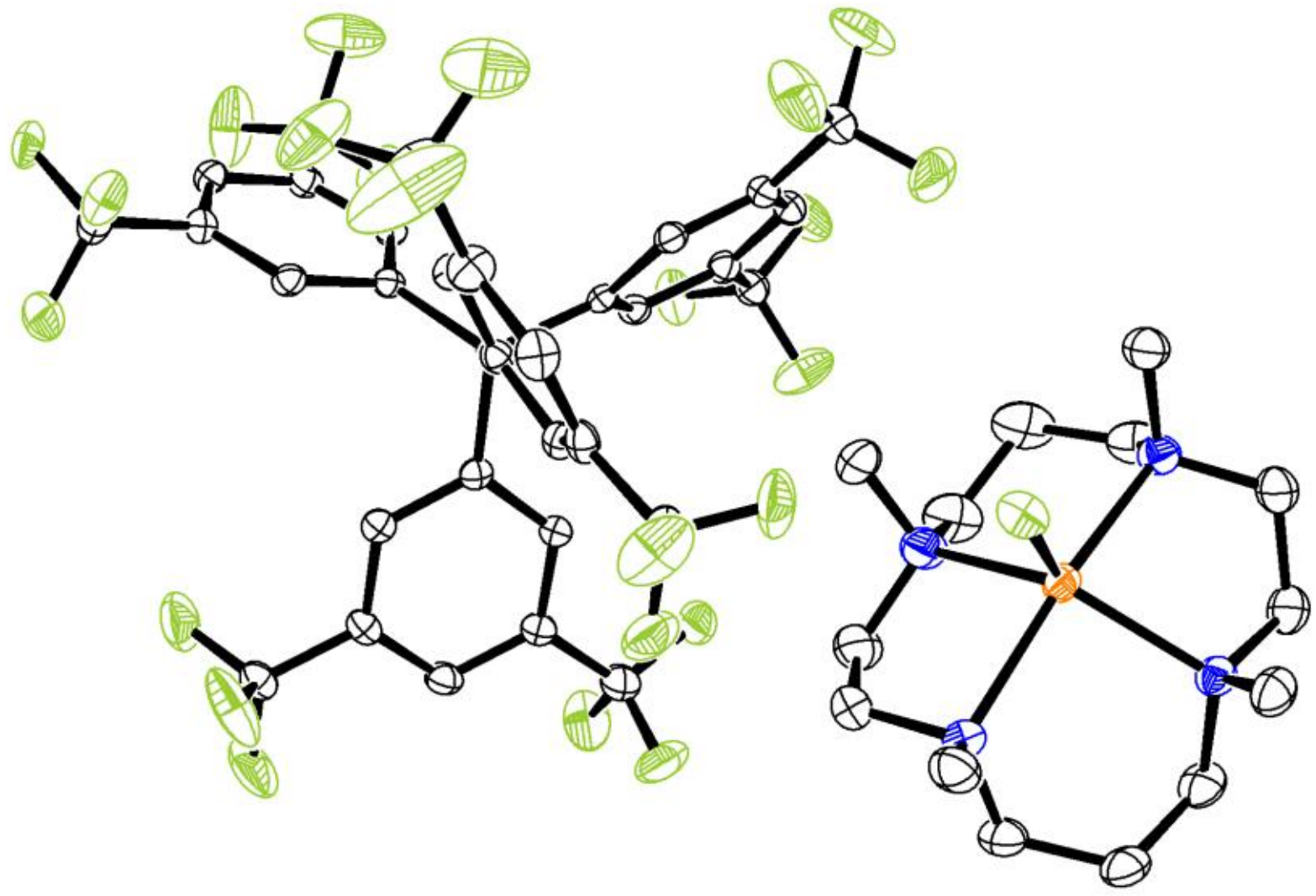

Figure S5. Molecular structure of **3b-B$_{Ar}$F**.

Table 21. Crystal data and structure refinement for **3b-B$_{Ar}$F**.

CCDC No	2502781	
Empirical formula	$C_{46}H_{32}BD_{12}F_{25}FeN_4$	
Formula weight	1206.58	
Temperature	100(2) K	
Wavelength	0.71073 Å	
Crystal system	Triclinic	
Space group	$P\bar{1}$	
Unit cell dimensions	a = 12.9545(9) Å	α = 96.213(4)°
	b = 14.0406(10) Å	β = 106.540(3)°
	c = 14.2649(11) Å	γ = 92.923(3)°
Volume	2463.5(3) Å^3	
Z	2	
Density (calculated)	1.627 Mg/m^3	
Absorption coefficient	0.440 mm^{-1}	
F(000)	1208	
Crystal size	0.596 x 0.316 x 0.279 mm^3	
Theta range for data collection	1.958 to 28.282°.	
Index ranges	$-17 \le h \le 17$, $-18 \le k \le 18$, $-19 \le l \le 19$	
Reflections collected	244712	
Independent reflections	12219 [R(int) = 0.1305]	
Completeness to theta = 25.242°	100.0 %	
Absorption correction	Semi-empirical from equivalents	
Max. and min. transmission	0.7375 and 0.6769	
Refinement method	Full-matrix least-squares on F^2	
Data / restraints / parameters	12219 / 1100 / 783	
Goodness-of-fit onF^2	1.063	
Final R indices [I>2sigma(I)]	R1 = 0.0625, wR2 = 0.1480	
R indices (all data)	R1 = 0.0911, wR2 = 0.1686	
Extinction coefficient	n/a	
Largest diff. peak and hole	1.503 and -1.554 eÅ^{-3}	

Table 22. Atomic coordinates (x 10^4) and equivalent isotropic displacement parameters ($Å^2$ x 10^3) for **3b-B$_{ArF}$**.
U(eq) is defined as one third of the trace of the orthogonalized U^{ij} tensor.

	x	y	z	U(eq)
Fe(1)	10306(1)	6615(1)	9350(1)	25(1)
F(1)	9388(2)	5512(1)	9100(2)	34(1)
F(004)	6516(2)	10501(2)	10645(1)	41(1)
F(5)	1299(3)	9819(2)	7836(2)	66(1)
F(6)	885(2)	10479(2)	6531(2)	63(1)
F(7)	2492(2)	10697(2)	7504(2)	43(1)
F(8)	7241(2)	5313(2)	5885(2)	43(1)
F(9)	7390(2)	4937(2)	7313(2)	48(1)
F(10)	6649(2)	3906(2)	6051(2)	52(1)
F(11)	2957(3)	3356(2)	6048(3)	97(1)
F(12)	2839(3)	4212(2)	7345(3)	83(1)
F(13)	1967(2)	4523(2)	5946(2)	62(1)
F(14)	8149(2)	9906(2)	7219(1)	35(1)
F(15)	8627(2)	9220(2)	6017(2)	54(1)
F(16)	7913(2)	10561(2)	5898(2)	54(1)
F(17)	5254(2)	9040(2)	2861(2)	63(1)
F(18)	4655(3)	7623(2)	2920(2)	58(1)
F(19)	3733(2)	8781(2)	3093(2)	63(1)
F(20)	5347(2)	6025(2)	9869(2)	58(1)
F(21)	6913(2)	6686(2)	10633(2)	66(1)
F(22)	5542(2)	7120(2)	11051(2)	56(1)
F(23)	5919(2)	10890(1)	9196(2)	37(1)
F(24)	7498(2)	10399(2)	9664(2)	45(1)
N(1)	9223(2)	7714(2)	8843(2)	33(1)
N(2)	10419(2)	6516(2)	7797(2)	28(1)
N(3)	12040(2)	6571(2)	9946(2)	25(1)
N(4)	10444(2)	7127(2)	10914(2)	27(1)
C(1)	8118(3)	7308(3)	8725(3)	41(1)
D(1A)	7601	7759	8439	62
D(1B)	8045	7196	9370	62
D(1C)	7971	6698	8287	62
C(2)	10291(3)	5510(3)	7328(3)	35(1)
D(2A)	10209	5500	6622	52
D(2B)	9648	5178	7416	52
D(2C)	10931	5186	7634	52
C(3)	12278(3)	5548(2)	9940(3)	32(1)
D(3A)	12067	5230	9259	48
D(3B)	11871	5227	10317	48
D(3C)	13054	5512	10240	48
C(4)	9605(3)	6593(3)	11211(3)	31(1)
D(4A)	8890	6759	10842	47
D(4B)	9712	6765	11919	47
D(4C)	9654	5901	11071	47
C(5)	9319(3)	7893(3)	7855(3)	40(1)
C(6)	9455(3)	6979(3)	7271(3)	36(1)
C(7)	11408(3)	7015(3)	7682(3)	33(1)
C(8)	12463(3)	6737(3)	8333(3)	33(1)
C(9)	12701(3)	7077(3)	9418(3)	32(1)
C(10)	12362(3)	7044(3)	10983(3)	33(1)
C(11)	11512(3)	6817(3)	11475(2)	33(1)
C(12)	10413(3)	8169(3)	11168(3)	38(1)
C(13)	9453(4)	8601(3)	10545(3)	45(1)
C(14)	9448(4)	8657(3)	9490(3)	45(1)
C(15)	3312(2)	8060(2)	6405(2)	16(1)
C(16)	2506(2)	7567(2)	5596(2)	18(1)
C(17)	1461(2)	7854(2)	5290(2)	20(1)
C(18)	1175(2)	8667(2)	5763(2)	21(1)
C(19)	1960(2)	9178(2)	6553(2)	19(1)
C(20)	2995(2)	8877(2)	6873(2)	17(1)
C(21)	639(2)	7259(2)	4451(2)	29(1)
C(22)	1665(3)	10049(2)	7087(3)	29(1)
C(23)	4587(2)	6566(2)	6498(2)	17(1)
C(24)	5536(2)	6178(2)	6423(2)	18(1)
C(25)	5646(2)	5194(2)	6372(2)	21(1)
C(26)	4799(3)	4550(2)	6363(2)	25(1)
C(27)	3846(3)	4910(2)	6421(2)	27(1)
C(28)	3749(2)	5896(2)	6504(2)	22(1)
C(29)	6717(3)	4835(2)	6392(2)	29(1)
C(30)	2913(3)	4244(3)	6431(4)	45(1)
C(31)	5209(2)	8260(2)	6046(2)	16(1)
C(32)	6249(2)	8739(2)	6414(2)	19(1)
C(33)	6761(2)	9179(2)	5815(2)	22(1)
C(34)	6276(2)	9123(2)	4808(2)	23(1)
C(35)	5264(2)	8630(2)	4423(2)	19(1)
C(36)	4737(2)	8224(2)	5028(2)	18(1)
C(37)	7850(3)	9717(3)	6236(2)	32(1)
C(38)	4730(3)	8518(2)	3333(2)	24(1)
C(39)	5137(2)	8037(2)	7909(2)	16(1)
C(40)	5278(2)	7359(2)	8582(2)	18(1)
C(41)	5757(2)	7612(2)	9589(2)	20(1)
C(42)	6144(2)	8551(2)	9976(2)	21(1)
C(43)	6024(2)	9236(2)	9325(2)	19(1)
C(44)	5525(2)	8984(2)	8322(2)	17(1)
C(45)	5890(3)	6866(2)	10282(2)	27(1)
C(46)	6477(2)	10243(2)	9703(2)	24(1)
B(1)	4552(2)	7733(2)	6718(2)	15(1)
F(2)	657(12)	6328(7)	4512(13)	33(3)
F(3)	-382(10)	7487(13)	4404(14)	27(3)
F(4)	799(14)	7424(16)	3593(8)	39(3)
F(2A)	1039(8)	6975(10)	3706(7)	38(2)
F(3A)	365(9)	6394(6)	4754(7)	35(2)

F(4A)	-283(7)	7630(8)	4115(9)	40(3)
F(2B)	908(14)	6438(10)	4123(17)	35(3)
F(3B)	422(19)	7795(12)	3652(10)	34(3)
F(4B)	-323(11)	7155(18)	4621(13)	36(3)
F(2C)	1072(12)	6675(10)	3851(11)	25(3)
F(3C)	-20(13)	7783(9)	3848(11)	38(3)
F(4C)	25(12)	6658(12)	4784(9)	39(3)

Table 23. Bond lengths [Å] and angles [°] for **3b-B$_{Ar}$F**.

Fe(1)-F(1)	1.840(2)	C(21)-F(4)	1.340(9)
Fe(1)-N(3)	2.171(3)	C(21)-F(2A)	1.342(7)
Fe(1)-N(1)	2.181(3)	C(21)-F(4C)	1.342(9)
Fe(1)-N(4)	2.222(3)	C(21)-F(3)	1.361(10)
Fe(1)-N(2)	2.252(3)	C(21)-F(2C)	1.374(9)
F(004)-C(46)	1.340(3)	C(21)-F(3A)	1.394(7)
F(5)-C(22)	1.347(4)	C(21)-F(3B)	1.404(10)
F(6)-C(22)	1.312(4)	C(23)-C(24)	1.397(4)
F(7)-C(22)	1.322(4)	C(23)-C(28)	1.401(4)
F(8)-C(29)	1.332(4)	C(23)-B(1)	1.640(4)
F(9)-C(29)	1.342(4)	C(24)-C(25)	1.392(4)
F(10)-C(29)	1.331(4)	C(24)-H(24)	0.9500
F(11)-C(30)	1.317(5)	C(25)-C(26)	1.382(4)
F(12)-C(30)	1.340(6)	C(25)-C(29)	1.493(4)
F(13)-C(30)	1.326(5)	C(26)-C(27)	1.379(4)
F(14)-C(37)	1.336(4)	C(26)-H(26)	0.9500
F(15)-C(37)	1.342(4)	C(27)-C(28)	1.392(4)
F(16)-C(37)	1.334(4)	C(27)-C(30)	1.495(5)
F(17)-C(38)	1.329(4)	C(28)-H(28)	0.9500
F(18)-C(38)	1.315(4)	C(31)-C(36)	1.400(4)
F(19)-C(38)	1.322(4)	C(31)-C(32)	1.404(4)
F(20)-C(45)	1.328(4)	C(31)-B(1)	1.656(4)
F(21)-C(45)	1.325(4)	C(32)-C(33)	1.396(4)
F(22)-C(45)	1.322(4)	C(32)-H(32)	0.9500
F(23)-C(46)	1.334(4)	C(33)-C(34)	1.386(4)
F(24)-C(46)	1.347(4)	C(33)-C(37)	1.494(4)
N(1)-C(1)	1.470(5)	C(34)-C(35)	1.384(4)
N(1)-C(14)	1.495(5)	C(34)-H(34)	0.9500
N(1)-C(5)	1.496(5)	C(35)-C(36)	1.392(4)
N(2)-C(2)	1.475(4)	C(35)-C(38)	1.497(4)
N(2)-C(6)	1.484(4)	C(36)-H(36)	0.9500
N(2)-C(7)	1.487(4)	C(39)-C(44)	1.400(4)
N(3)-C(3)	1.483(4)	C(39)-C(40)	1.406(4)
N(3)-C(10)	1.488(4)	C(39)-B(1)	1.649(4)
N(3)-C(9)	1.492(4)	C(40)-C(41)	1.390(4)
N(4)-C(4)	1.474(4)	C(40)-H(40)	0.9500
N(4)-C(12)	1.474(4)	C(41)-C(42)	1.382(4)
N(4)-C(11)	1.500(4)	C(41)-C(45)	1.500(4)
C(1)-D(1A)	0.9800	C(42)-C(43)	1.391(4)
C(1)-D(1B)	0.9800	C(42)-H(42)	0.9500
C(1)-D(1C)	0.9800	C(43)-C(44)	1.389(4)
C(2)-D(2A)	0.9800	C(43)-C(46)	1.486(4)
C(2)-D(2B)	0.9800	C(44)-H(44)	0.9500
C(2)-D(2C)	0.9800		
C(3)-D(3A)	0.9800	F(1)-Fe(1)-N(3)	120.87(10)
C(3)-D(3B)	0.9800	F(1)-Fe(1)-N(1)	103.38(11)
C(3)-D(3C)	0.9800	N(3)-Fe(1)-N(1)	135.67(11)
C(4)-D(4A)	0.9800	F(1)-Fe(1)-N(4)	102.57(9)
C(4)-D(4B)	0.9800	N(3)-Fe(1)-N(4)	82.25(10)
C(4)-D(4C)	0.9800	N(1)-Fe(1)-N(4)	91.26(11)
C(5)-C(6)	1.500(6)	F(1)-Fe(1)-N(2)	93.84(9)
C(5)-H(5A)	0.9900	N(3)-Fe(1)-N(2)	91.71(10)
C(5)-H(5AB)	0.9900	N(1)-Fe(1)-N(2)	82.22(10)
C(6)-H(6A)	0.9900	N(4)-Fe(1)-N(2)	163.37(10)
C(6)-H(6AB)	0.9900	C(1)-N(1)-C(14)	110.0(3)
C(7)-C(8)	1.515(5)	C(1)-N(1)-C(5)	108.9(3)
C(7)-H(7A)	0.9900	C(14)-N(1)-C(5)	107.7(3)
C(7)-H(7AB)	0.9900	C(1)-N(1)-Fe(1)	107.1(2)
C(8)-C(9)	1.508(5)	C(14)-N(1)-Fe(1)	115.8(2)
C(8)-H(8A)	0.9900	C(5)-N(1)-Fe(1)	107.1(2)
C(8)-H(8AB)	0.9900	C(2)-N(2)-C(6)	106.8(3)
C(9)-H(9A)	0.9900	C(2)-N(2)-C(7)	109.5(3)
C(9)-H(9AB)	0.9900	C(6)-N(2)-C(7)	109.4(3)
C(10)-C(11)	1.503(5)	C(2)-N(2)-Fe(1)	111.9(2)
C(10)-H(10A)	0.9900	C(6)-N(2)-Fe(1)	103.19(19)
C(10)-H(10B)	0.9900	C(7)-N(2)-Fe(1)	115.5(2)
C(11)-H(11A)	0.9900	C(3)-N(3)-C(10)	108.9(3)
C(11)-H(11B)	0.9900	C(3)-N(3)-C(9)	109.8(3)
C(12)-C(13)	1.506(6)	C(10)-N(3)-C(9)	107.3(2)
C(12)-H(12A)	0.9900	C(3)-N(3)-Fe(1)	108.12(19)
C(12)-H(12B)	0.9900	C(10)-N(3)-Fe(1)	107.65(19)
C(13)-C(14)	1.513(6)	C(9)-N(3)-Fe(1)	114.9(2)
C(13)-H(13A)	0.9900	C(4)-N(4)-C(12)	109.9(3)
C(13)-H(13B)	0.9900	C(4)-N(4)-C(11)	107.0(3)
C(14)-H(14A)	0.9900	C(12)-N(4)-C(11)	109.8(3)
C(14)-H(14B)	0.9900	C(4)-N(4)-Fe(1)	109.19(19)
C(15)-C(20)	1.403(4)	C(12)-N(4)-Fe(1)	116.6(2)
C(15)-C(16)	1.404(4)	C(11)-N(4)-Fe(1)	103.85(19)
C(15)-B(1)	1.644(4)	N(1)-C(1)-D(1A)	109.5
C(16)-C(17)	1.394(4)	N(1)-C(1)-D(1B)	109.5
C(16)-H(16)	0.9500	D(1A)-C(1)-D(1B)	109.5
C(17)-C(18)	1.383(4)	N(1)-C(1)-D(1C)	109.5
C(17)-C(21)	1.494(4)	D(1A)-C(1)-D(1C)	109.5
C(18)-C(19)	1.385(4)	D(1B)-C(1)-D(1C)	109.5
C(18)-H(18)	0.9500	N(2)-C(2)-D(2A)	109.5
C(19)-C(20)	1.391(4)	N(2)-C(2)-D(2B)	109.5
C(19)-C(22)	1.496(4)	D(2A)-C(2)-D(2B)	109.5
C(20)-H(20)	0.9500	N(2)-C(2)-D(2C)	109.5
C(21)-F(2B)	1.298(10)	D(2A)-C(2)-D(2C)	109.5
C(21)-F(4A)	1.311(7)	D(2B)-C(2)-D(2C)	109.5
C(21)-F(2)	1.320(10)	N(3)-C(3)-D(3A)	109.5
C(21)-F(4B)	1.339(10)	N(3)-C(3)-D(3B)	109.5
C(21)-F(3C)	1.340(9)	D(3A)-C(3)-D(3B)	109.5

N(3)-C(3)-D(3C)	109.5
D(3A)-C(3)-D(3C)	109.5
D(3B)-C(3)-D(3C)	109.5
N(4)-C(4)-D(4A)	109.5
N(4)-C(4)-D(4B)	109.5
D(4A)-C(4)-D(4B)	109.5
N(4)-C(4)-D(4C)	109.5
D(4A)-C(4)-D(4C)	109.5
D(4B)-C(4)-D(4C)	109.5
N(1)-C(5)-C(6)	111.1(3)
N(1)-C(5)-H(5A)	109.4
C(6)-C(5)-H(5A)	109.4
N(1)-C(5)-H(5AB)	109.4
C(6)-C(5)-H(5AB)	109.4
H(5A)-C(5)-H(5AB)	108.0
N(2)-C(6)-C(5)	111.7(3)
N(2)-C(6)-H(6A)	109.3
C(5)-C(6)-H(6A)	109.3
N(2)-C(6)-H(6AB)	109.3
C(5)-C(6)-H(6AB)	109.3
H(6A)-C(6)-H(6AB)	107.9
N(2)-C(7)-C(8)	114.9(3)
N(2)-C(7)-H(7A)	108.5
C(8)-C(7)-H(7A)	108.5
N(2)-C(7)-H(7AB)	108.5
C(8)-C(7)-H(7AB)	108.5
H(7A)-C(7)-H(7AB)	107.5
C(9)-C(8)-C(7)	115.3(3)
C(9)-C(8)-H(8A)	108.4
C(7)-C(8)-H(8A)	108.4
C(9)-C(8)-H(8AB)	108.4
C(7)-C(8)-H(8AB)	108.4
H(8A)-C(8)-H(8AB)	107.5
N(3)-C(9)-C(8)	116.0(3)
N(3)-C(9)-H(9A)	108.3
C(8)-C(9)-H(9A)	108.3
N(3)-C(9)-H(9AB)	108.3
C(8)-C(9)-H(9AB)	108.3
H(9A)-C(9)-H(9AB)	107.4
N(3)-C(10)-C(11)	110.8(3)
N(3)-C(10)-H(10A)	109.5
C(11)-C(10)-H(10A)	109.5
N(3)-C(10)-H(10B)	109.5
C(11)-C(10)-H(10B)	109.5
H(10A)-C(10)-H(10B)	108.1
N(4)-C(11)-C(10)	110.5(3)
N(4)-C(11)-H(11A)	109.5
C(10)-C(11)-H(11A)	109.5
N(4)-C(11)-H(11B)	109.5
C(10)-C(11)-H(11B)	109.5
H(11A)-C(11)-H(11B)	108.1
N(4)-C(12)-C(13)	115.2(3)
N(4)-C(12)-H(12A)	108.5
C(13)-C(12)-H(12A)	108.5
N(4)-C(12)-H(12B)	108.5
C(13)-C(12)-H(12B)	108.5
H(12A)-C(12)-H(12B)	107.5
C(12)-C(13)-C(14)	115.2(3)
C(12)-C(13)-H(13A)	108.5
C(14)-C(13)-H(13A)	108.5
C(12)-C(13)-H(13B)	108.5
C(14)-C(13)-H(13B)	108.5
H(13A)-C(13)-H(13B)	107.5
N(1)-C(14)-C(13)	114.9(3)
N(1)-C(14)-H(14A)	108.5
C(13)-C(14)-H(14A)	108.5
N(1)-C(14)-H(14B)	108.5
C(13)-C(14)-H(14B)	108.5
H(14A)-C(14)-H(14B)	107.5
C(20)-C(15)-C(16)	115.0(2)
C(20)-C(15)-B(1)	123.3(2)
C(16)-C(15)-B(1)	121.5(2)
C(17)-C(16)-C(15)	122.6(3)
C(17)-C(16)-H(16)	118.7
C(15)-C(16)-H(16)	118.7
C(18)-C(17)-C(16)	121.0(3)
C(18)-C(17)-C(21)	120.0(3)
C(16)-C(17)-C(21)	119.0(3)
C(17)-C(18)-C(19)	117.6(3)
C(17)-C(18)-H(18)	121.2
C(19)-C(18)-H(18)	121.2
C(18)-C(19)-C(20)	121.3(3)
C(18)-C(19)-C(22)	118.6(3)
C(20)-C(19)-C(22)	120.0(3)
C(19)-C(20)-C(15)	122.4(3)
C(19)-C(20)-H(20)	118.8
C(15)-C(20)-H(20)	118.8
F(2B)-C(21)-F(4B)	110.8(9)
F(2)-C(21)-F(4)	108.6(7)
F(4A)-C(21)-F(2A)	110.2(6)
F(3C)-C(21)-F(4C)	108.0(7)
F(2)-C(21)-F(3)	106.9(8)
F(4)-C(21)-F(3)	106.5(8)
F(3C)-C(21)-F(2C)	105.2(7)
F(4C)-C(21)-F(2C)	105.0(7)
F(4A)-C(21)-F(3A)	104.9(5)
F(2A)-C(21)-F(3A)	103.2(5)
F(2B)-C(21)-F(3B)	105.6(9)
F(4B)-C(21)-F(3B)	102.7(8)
F(6)-C(22)-F(7)	108.1(3)
F(6)-C(22)-F(5)	105.2(3)
F(7)-C(22)-F(5)	104.8(3)
F(6)-C(22)-C(19)	113.5(3)
F(7)-C(22)-C(19)	113.4(3)
F(5)-C(22)-C(19)	111.1(3)
C(24)-C(23)-C(28)	115.4(3)
C(24)-C(23)-B(1)	120.4(2)
C(28)-C(23)-B(1)	123.7(2)
C(25)-C(24)-C(23)	122.2(3)
C(25)-C(24)-H(24)	118.9
C(23)-C(24)-H(24)	118.9
C(26)-C(25)-C(24)	121.0(3)
C(26)-C(25)-C(29)	119.4(3)
C(24)-C(25)-C(29)	119.4(3)
C(27)-C(26)-C(25)	118.1(3)
C(27)-C(26)-H(26)	120.9
C(25)-C(26)-H(26)	120.9
C(26)-C(27)-C(28)	120.8(3)
C(26)-C(27)-C(30)	120.1(3)
C(28)-C(27)-C(30)	119.1(3)
C(27)-C(28)-C(23)	122.5(3)
C(27)-C(28)-H(28)	118.8
C(23)-C(28)-H(28)	118.8
F(10)-C(29)-F(8)	107.3(3)
F(10)-C(29)-F(9)	106.2(3)
F(8)-C(29)-F(9)	104.7(3)
F(10)-C(29)-C(25)	113.1(3)
F(8)-C(29)-C(25)	113.4(3)
F(9)-C(29)-C(25)	111.5(3)
F(11)-C(30)-F(13)	107.0(4)
F(11)-C(30)-F(12)	107.5(4)
F(13)-C(30)-F(12)	103.9(3)
F(11)-C(30)-C(27)	113.2(3)
F(13)-C(30)-C(27)	112.8(3)
F(12)-C(30)-C(27)	111.9(4)
C(36)-C(31)-C(32)	115.2(2)
C(36)-C(31)-B(1)	119.9(2)
C(32)-C(31)-B(1)	124.8(2)
C(33)-C(32)-C(31)	122.4(3)
C(33)-C(32)-H(32)	118.8
C(31)-C(32)-H(32)	118.8
C(34)-C(33)-C(32)	120.9(3)
C(34)-C(33)-C(37)	118.0(3)
C(32)-C(33)-C(37)	121.1(3)
C(35)-C(34)-C(33)	117.8(3)
C(35)-C(34)-H(34)	121.1
C(33)-C(34)-H(34)	121.1
C(34)-C(35)-C(36)	121.2(3)
C(34)-C(35)-C(38)	119.5(3)
C(36)-C(35)-C(38)	119.3(3)
C(35)-C(36)-C(31)	122.4(3)
C(35)-C(36)-H(36)	118.8
C(31)-C(36)-H(36)	118.8
F(16)-C(37)-F(14)	106.3(3)
F(16)-C(37)-F(15)	106.3(3)
F(14)-C(37)-F(15)	105.7(3)
F(16)-C(37)-C(33)	112.9(3)
F(14)-C(37)-C(33)	113.5(3)
F(15)-C(37)-C(33)	111.7(3)
F(18)-C(38)-F(19)	105.6(3)
F(18)-C(38)-F(17)	106.4(3)
F(19)-C(38)-F(17)	105.4(3)
F(18)-C(38)-C(35)	112.5(2)
F(19)-C(38)-C(35)	113.4(3)
F(17)-C(38)-C(35)	112.9(3)
C(44)-C(39)-C(40)	115.3(2)
C(44)-C(39)-B(1)	122.6(2)
C(40)-C(39)-B(1)	122.1(2)
C(41)-C(40)-C(39)	122.4(3)
C(41)-C(40)-H(40)	118.8
C(39)-C(40)-H(40)	118.8
C(42)-C(41)-C(40)	121.1(3)
C(42)-C(41)-C(45)	118.2(3)
C(40)-C(41)-C(45)	120.7(3)
C(41)-C(42)-C(43)	117.7(3)
C(41)-C(42)-H(42)	121.1
C(43)-C(42)-H(42)	121.1
C(44)-C(43)-C(42)	121.0(3)
C(44)-C(43)-C(46)	119.5(3)
C(42)-C(43)-C(46)	119.5(3)
C(43)-C(44)-C(39)	122.4(3)
C(43)-C(44)-H(44)	118.8
C(39)-C(44)-H(44)	118.8
F(22)-C(45)-F(21)	106.9(3)
F(22)-C(45)-F(20)	105.1(3)
F(21)-C(45)-F(20)	106.1(3)
F(22)-C(45)-C(41)	112.7(3)
F(21)-C(45)-C(41)	112.1(3)
F(20)-C(45)-C(41)	113.4(3)
F(23)-C(46)-F(004)	105.8(2)

F(23)-C(46)-F(24)	105.7(3)	C(23)-B(1)-C(31)	108.5(2)
F(004)-C(46)-F(24)	105.7(2)	C(15)-B(1)-C(31)	107.2(2)
F(23)-C(46)-C(43)	113.4(2)	C(39)-B(1)-C(31)	111.7(2)
F(004)-C(46)-C(43)	113.5(3)		
F(24)-C(46)-C(43)	112.2(2)		
C(23)-B(1)-C(15)	112.2(2)		
C(23)-B(1)-C(39)	106.6(2)		
C(15)-B(1)-C(39)	110.6(2)		

Table 24. Anisotropic displacement parameters ($Å^2 \times 10^3$) for **3b-B$_{ArF}$**.
The anisotropic displacement factor exponent takes the form: $-2p^2 [h^2 a^{*2} U^{11} + ... + 2 h k a^* b^* U^{12}]$

	U^{11}	U^{22}	U^{33}	U^{23}	U^{13}	U^{12}
Fe(1)	20(1)	31(1)	23(1)	0(1)	6(1)	5(1)
F(1)	41(1)	33(1)	30(1)	4(1)	13(1)	2(1)
F(004)	63(1)	33(1)	21(1)	-8(1)	11(1)	-4(1)
F(5)	95(2)	51(2)	75(2)	-6(1)	67(2)	12(1)
F(6)	55(2)	43(1)	68(2)	-16(1)	-16(1)	35(1)
F(7)	36(1)	29(1)	57(1)	-17(1)	8(1)	5(1)
F(8)	28(1)	52(1)	55(1)	7(1)	19(1)	12(1)
F(9)	38(1)	61(2)	37(1)	1(1)	-6(1)	28(1)
F(10)	44(1)	27(1)	84(2)	-20(1)	24(1)	10(1)
F(11)	67(2)	21(1)	213(4)	-16(2)	71(2)	-11(1)
F(12)	82(2)	86(2)	95(2)	44(2)	42(2)	-19(2)
F(13)	32(1)	45(1)	109(2)	15(1)	19(1)	-9(1)
F(14)	27(1)	43(1)	28(1)	4(1)	2(1)	-10(1)
F(15)	24(1)	83(2)	55(2)	-5(1)	18(1)	0(1)
F(16)	49(1)	54(1)	52(1)	27(1)	0(1)	-25(1)
F(17)	69(2)	88(2)	25(1)	23(1)	4(1)	-31(2)
F(18)	107(2)	30(1)	21(1)	-2(1)	-4(1)	15(1)
F(19)	47(1)	109(2)	28(1)	5(1)	-1(1)	42(2)
F(20)	86(2)	36(1)	41(1)	20(1)	2(1)	-11(1)
F(21)	31(1)	98(2)	86(2)	72(2)	17(1)	23(1)
F(22)	85(2)	61(2)	41(1)	28(1)	39(1)	25(1)
F(23)	47(1)	20(1)	33(1)	-2(1)	-2(1)	4(1)
F(24)	31(1)	30(1)	74(2)	-11(1)	24(1)	-8(1)
N(1)	38(2)	34(2)	34(2)	11(1)	18(1)	15(1)
N(2)	24(1)	34(1)	28(1)	5(1)	10(1)	4(1)
N(3)	21(1)	27(1)	26(1)	2(1)	6(1)	-2(1)
N(4)	26(1)	29(1)	25(1)	-2(1)	8(1)	-1(1)
C(1)	28(2)	57(2)	44(2)	19(2)	15(2)	18(2)
C(2)	38(2)	39(2)	27(2)	-2(1)	12(1)	1(2)
C(3)	28(2)	30(2)	40(2)	9(1)	9(1)	6(1)
C(4)	31(2)	34(2)	29(2)	3(1)	10(1)	-1(1)
C(5)	43(2)	43(2)	41(2)	20(2)	18(2)	15(2)
C(6)	32(2)	51(2)	27(2)	12(2)	8(1)	8(2)
C(7)	32(2)	39(2)	34(2)	10(2)	18(1)	2(1)
C(8)	25(2)	38(2)	41(2)	7(2)	16(1)	-1(1)
C(9)	22(2)	32(2)	41(2)	5(1)	9(1)	-7(1)
C(10)	25(2)	39(2)	29(2)	0(1)	1(1)	-3(1)
C(11)	29(2)	42(2)	24(2)	0(1)	3(1)	-2(1)
C(12)	48(2)	29(2)	40(2)	-9(1)	24(2)	-6(2)
C(13)	66(3)	26(2)	54(2)	1(2)	35(2)	14(2)
C(14)	61(3)	29(2)	56(2)	10(2)	31(2)	18(2)
C(15)	16(1)	16(1)	16(1)	2(1)	6(1)	2(1)
C(16)	17(1)	18(1)	19(1)	0(1)	7(1)	2(1)
C(17)	18(1)	22(1)	18(1)	2(1)	4(1)	0(1)
C(18)	17(1)	25(1)	21(1)	5(1)	6(1)	5(1)
C(19)	21(1)	19(1)	20(1)	2(1)	9(1)	5(1)
C(20)	17(1)	19(1)	17(1)	1(1)	6(1)	2(1)
C(21)	20(1)	35(2)	27(2)	-5(1)	2(1)	2(1)
C(22)	25(2)	26(2)	34(2)	-3(1)	7(1)	10(1)
C(23)	19(1)	18(1)	15(1)	2(1)	5(1)	3(1)
C(24)	19(1)	17(1)	16(1)	-2(1)	3(1)	2(1)
C(25)	24(1)	20(1)	15(1)	-3(1)	2(1)	6(1)
C(26)	30(2)	16(1)	28(2)	2(1)	5(1)	5(1)
C(27)	29(2)	19(1)	34(2)	6(1)	10(1)	0(1)
C(28)	22(1)	19(1)	27(2)	3(1)	9(1)	2(1)
C(29)	30(2)	23(2)	29(2)	-6(1)	3(1)	10(1)
C(30)	39(2)	23(2)	79(3)	10(2)	26(2)	-1(1)
C(31)	17(1)	14(1)	20(1)	3(1)	7(1)	4(1)
C(32)	18(1)	23(1)	17(1)	4(1)	6(1)	3(1)
C(33)	22(1)	25(1)	21(1)	6(1)	8(1)	1(1)
C(34)	26(1)	25(2)	22(1)	7(1)	11(1)	3(1)
C(35)	25(1)	17(1)	16(1)	2(1)	7(1)	5(1)
C(36)	19(1)	16(1)	19(1)	1(1)	6(1)	3(1)
C(37)	28(2)	42(2)	26(2)	10(1)	9(1)	-3(1)
C(38)	29(2)	23(1)	19(1)	5(1)	7(1)	5(1)
C(39)	12(1)	18(1)	18(1)	2(1)	6(1)	3(1)
C(40)	15(1)	19(1)	20(1)	3(1)	6(1)	1(1)
C(41)	18(1)	27(1)	19(1)	8(1)	7(1)	5(1)
C(42)	20(1)	27(1)	15(1)	1(1)	5(1)	3(1)
C(43)	18(1)	21(1)	19(1)	0(1)	6(1)	2(1)
C(44)	18(1)	18(1)	17(1)	2(1)	7(1)	3(1)
C(45)	26(2)	33(2)	23(2)	11(1)	7(1)	5(1)
C(46)	25(1)	26(2)	19(1)	-2(1)	5(1)	1(1)
B(1)	14(1)	17(1)	15(1)	2(1)	5(1)	1(1)
F(2)	21(6)	30(4)	38(6)	-21(4)	5(5)	4(4)
F(3)	18(4)	28(6)	30(7)	10(5)	-3(4)	1(4)
F(4)	35(6)	54(7)	23(4)	-10(5)	5(4)	-1(5)
F(2A)	36(4)	48(6)	24(4)	-9(4)	4(3)	4(4)
F(3A)	29(5)	26(3)	45(4)	-10(3)	9(3)	-3(3)
F(4A)	22(4)	47(5)	38(6)	-3(4)	-8(3)	6(3)
F(2B)	31(6)	26(5)	36(7)	-11(5)	-2(5)	-3(5)
F(3B)	30(7)	40(6)	22(5)	0(4)	-6(5)	5(5)
F(4B)	18(4)	42(7)	41(6)	-4(6)	2(4)	-8(5)
F(2C)	29(4)	19(5)	23(5)	-1(4)	2(4)	4(4)
F(3C)	20(5)	46(5)	35(6)	-5(4)	-11(4)	15(5)
F(4C)	25(5)	42(6)	46(5)	-9(5)	10(4)	-12(4)

Table 25. Hydrogen coordinates (x 10^4) and isotropic displacement parameters ($Å^2$ x 10^3) for **3b-B$_{ArF}$**.

	x	y	z	U(eq)
H(5A)	9948	8361	7944	47
H(5AB)	8663	8176	7488	47
H(6A)	8803	6529	7143	43
H(6AB)	9528	7121	6627	43
H(7A)	11378	7717	7827	40
H(7AB)	11404	6878	6985	40
H(8A)	12456	6027	8243	40
H(8AB)	13061	6998	8105	40
H(9A)	13473	7009	9743	39
H(9AB)	12592	7771	9497	39
H(10A)	12469	7749	10993	40
H(10B)	13056	6819	11352	40
H(11A)	11442	6117	11508	40
H(11B)	11731	7153	12157	40
H(12A)	11079	8503	11110	46
H(12B)	10419	8293	11866	46
H(13A)	9426	9259	10862	54
H(13B)	8788	8217	10538	54
H(14A)	8897	9089	9199	54
H(14B)	10160	8953	9494	54
H(16)	2679	7015	5242	21
H(18)	466	8868	5552	25
H(20)	3505	9237	7429	21
H(24)	6126	6599	6405	22
H(26)	4871	3878	6318	30
H(28)	3090	6124	6565	27
H(32)	6618	8765	7096	23
H(34)	6627	9413	4396	28
H(36)	4032	7912	4740	21
H(40)	5036	6705	8340	21
H(42)	6480	8722	10662	25
H(44)	5444	9472	7901	21

Table 26. Torsion angles [°] for **3b-B_{Ar}F**.

C(1)-N(1)-C(5)-C(6)	-78.7(4)
C(14)-N(1)-C(5)-C(6)	162.0(3)
Fe(1)-N(1)-C(5)-C(6)	36.8(4)
C(2)-N(2)-C(6)-C(5)	162.8(3)
C(7)-N(2)-C(6)-C(5)	-78.7(4)
Fe(1)-N(2)-C(6)-C(5)	44.7(3)
N(1)-C(5)-C(6)-N(2)	-58.1(4)
C(2)-N(2)-C(7)-C(8)	-73.9(4)
C(6)-N(2)-C(7)-C(8)	169.3(3)
Fe(1)-N(2)-C(7)-C(8)	53.5(4)
N(2)-C(7)-C(8)-C(9)	-69.9(4)
C(3)-N(3)-C(9)-C(8)	64.5(4)
C(10)-N(3)-C(9)-C(8)	-177.3(3)
Fe(1)-N(3)-C(9)-C(8)	-57.6(3)
C(7)-C(8)-C(9)-N(3)	72.7(4)
C(3)-N(3)-C(10)-C(11)	-80.2(3)
C(9)-N(3)-C(10)-C(11)	161.1(3)
Fe(1)-N(3)-C(10)-C(11)	36.9(3)
C(4)-N(4)-C(11)-C(10)	160.4(3)
C(12)-N(4)-C(11)-C(10)	-80.4(3)
Fe(1)-N(4)-C(11)-C(10)	45.0(3)
N(3)-C(10)-C(11)-N(4)	-57.6(4)
C(4)-N(4)-C(12)-C(13)	-71.4(4)
C(11)-N(4)-C(12)-C(13)	171.2(3)
Fe(1)-N(4)-C(12)-C(13)	53.5(4)
N(4)-C(12)-C(13)-C(14)	-70.3(4)
C(1)-N(1)-C(14)-C(13)	65.0(4)
C(5)-N(1)-C(14)-C(13)	-176.4(3)
Fe(1)-N(1)-C(14)-C(13)	-56.6(4)
C(12)-C(13)-C(14)-N(1)	72.1(5)
C(20)-C(15)-C(16)-C(17)	-1.2(4)
B(1)-C(15)-C(16)-C(17)	-177.2(3)
C(15)-C(16)-C(17)-C(18)	1.7(4)
C(15)-C(16)-C(17)-C(21)	-177.0(3)
C(16)-C(17)-C(18)-C(19)	-0.6(4)
C(21)-C(17)-C(18)-C(19)	178.2(3)
C(17)-C(18)-C(19)-C(20)	-1.0(4)
C(17)-C(18)-C(19)-C(22)	-178.9(3)
C(18)-C(19)-C(20)-C(15)	1.6(4)
C(22)-C(19)-C(20)-C(15)	179.4(3)
C(16)-C(15)-C(20)-C(19)	-0.5(4)
B(1)-C(15)-C(20)-C(19)	175.5(3)
C(18)-C(19)-C(22)-F(6)	-28.2(4)
C(20)-C(19)-C(22)-F(6)	153.9(3)
C(18)-C(19)-C(22)-F(7)	-152.1(3)
C(20)-C(19)-C(22)-F(7)	30.0(4)
C(18)-C(19)-C(22)-F(5)	90.1(4)
C(20)-C(19)-C(22)-F(5)	-87.8(4)
C(28)-C(23)-C(24)-C(25)	-1.0(4)
B(1)-C(23)-C(24)-C(25)	171.0(2)
C(23)-C(24)-C(25)-C(26)	2.0(4)
C(23)-C(24)-C(25)-C(29)	-174.0(3)
C(24)-C(25)-C(26)-C(27)	-0.9(4)
C(29)-C(25)-C(26)-C(27)	175.1(3)
C(25)-C(26)-C(27)-C(28)	-1.1(5)
C(25)-C(26)-C(27)-C(30)	-179.1(3)
C(26)-C(27)-C(28)-C(23)	2.2(5)
C(30)-C(27)-C(28)-C(23)	-179.8(3)
C(24)-C(23)-C(28)-C(27)	-1.1(4)
B(1)-C(23)-C(28)-C(27)	-172.8(3)
C(26)-C(25)-C(29)-F(10)	22.8(4)
C(24)-C(25)-C(29)-F(10)	-161.2(3)
C(26)-C(25)-C(29)-F(8)	145.2(3)
C(24)-C(25)-C(29)-F(8)	-38.7(4)
C(26)-C(25)-C(29)-F(9)	-96.8(3)
C(24)-C(25)-C(29)-F(9)	79.2(4)
C(26)-C(27)-C(30)-F(11)	-22.1(6)
C(28)-C(27)-C(30)-F(11)	159.9(4)
C(26)-C(27)-C(30)-F(13)	-143.7(4)
C(28)-C(27)-C(30)-F(13)	38.3(5)
C(26)-C(27)-C(30)-F(12)	99.6(4)
C(28)-C(27)-C(30)-F(12)	-78.4(4)
C(36)-C(31)-C(32)-C(33)	2.2(4)
B(1)-C(31)-C(32)-C(33)	-179.4(3)
C(31)-C(32)-C(33)-C(34)	-2.9(5)
C(31)-C(32)-C(33)-C(37)	177.5(3)
C(32)-C(33)-C(34)-C(35)	0.9(4)
C(37)-C(33)-C(34)-C(35)	-179.5(3)
C(33)-C(34)-C(35)-C(36)	1.6(4)
C(33)-C(34)-C(35)-C(38)	-177.5(3)
C(34)-C(35)-C(36)-C(31)	-2.3(4)
C(38)-C(35)-C(36)-C(31)	176.8(3)
C(32)-C(31)-C(36)-C(35)	0.3(4)
B(1)-C(31)-C(36)-C(35)	-178.1(2)
C(34)-C(33)-C(37)-F(16)	45.6(4)
C(32)-C(33)-C(37)-F(16)	-134.8(3)
C(34)-C(33)-C(37)-F(14)	166.6(3)
C(32)-C(33)-C(37)-F(14)	-13.8(5)
C(34)-C(33)-C(37)-F(15)	-74.0(4)
C(32)-C(33)-C(37)-F(15)	105.5(3)
C(34)-C(35)-C(38)-F(18)	111.5(3)
C(36)-C(35)-C(38)-F(18)	-67.7(4)
C(34)-C(35)-C(38)-F(19)	-128.7(3)

C(36)-C(35)-C(38)-F(19)	52.1(4)
C(34)-C(35)-C(38)-F(17)	-8.9(4)
C(36)-C(35)-C(38)-F(17)	171.9(3)
C(44)-C(39)-C(40)-C(41)	0.6(4)
B(1)-C(39)-C(40)-C(41)	-179.1(2)
C(39)-C(40)-C(41)-C(42)	-1.4(4)
C(39)-C(40)-C(41)-C(45)	179.9(3)
C(40)-C(41)-C(42)-C(43)	0.8(4)
C(45)-C(41)-C(42)-C(43)	179.5(3)
C(41)-C(42)-C(43)-C(44)	0.4(4)
C(41)-C(42)-C(43)-C(46)	-177.0(3)
C(42)-C(43)-C(44)-C(39)	-1.2(4)
C(46)-C(43)-C(44)-C(39)	176.2(3)
C(40)-C(39)-C(44)-C(43)	0.7(4)
B(1)-C(39)-C(44)-C(43)	-179.6(2)
C(42)-C(41)-C(45)-F(22)	50.7(4)
C(40)-C(41)-C(45)-F(22)	-130.6(3)
C(42)-C(41)-C(45)-F(21)	-69.9(4)
C(40)-C(41)-C(45)-F(21)	108.8(3)
C(42)-C(41)-C(45)-F(20)	170.0(3)
C(40)-C(41)-C(45)-F(20)	-11.3(4)
C(44)-C(43)-C(46)-F(23)	32.9(4)
C(42)-C(43)-C(46)-F(23)	-149.6(3)
C(44)-C(43)-C(46)-F(004)	153.6(3)
C(42)-C(43)-C(46)-F(004)	-28.9(4)
C(44)-C(43)-C(46)-F(24)	-86.7(3)
C(42)-C(43)-C(46)-F(24)	90.7(3)
C(24)-C(23)-B(1)-C(15)	160.2(2)
C(28)-C(23)-B(1)-C(15)	-28.5(4)
C(24)-C(23)-B(1)-C(39)	-78.6(3)
C(28)-C(23)-B(1)-C(39)	92.8(3)
C(24)-C(23)-B(1)-C(31)	41.9(3)
C(28)-C(23)-B(1)-C(31)	-146.7(3)
C(20)-C(15)-B(1)-C(23)	150.8(2)
C(16)-C(15)-B(1)-C(23)	-33.6(3)
C(20)-C(15)-B(1)-C(39)	31.8(3)
C(16)-C(15)-B(1)-C(39)	-152.5(2)
C(20)-C(15)-B(1)-C(31)	-90.2(3)
C(16)-C(15)-B(1)-C(31)	85.5(3)
C(44)-C(39)-B(1)-C(23)	161.8(2)
C(40)-C(39)-B(1)-C(23)	-18.4(3)
C(44)-C(39)-B(1)-C(15)	-75.9(3)
C(40)-C(39)-B(1)-C(15)	103.9(3)
C(44)-C(39)-B(1)-C(31)	43.5(3)
C(40)-C(39)-B(1)-C(31)	-136.8(2)
C(36)-C(31)-B(1)-C(23)	73.8(3)
C(32)-C(31)-B(1)-C(23)	-104.5(3)
C(36)-C(31)-B(1)-C(15)	-47.6(3)
C(32)-C(31)-B(1)-C(15)	134.1(3)
C(36)-C(31)-B(1)-C(39)	-169.0(2)
C(32)-C(31)-B(1)-C(39)	12.8(4)

[(TMC)Co^{II}(CH$_3$CN)](OTf)$_2$, **4a-(OTf)$_2$**

The crystal structure of **4a-(OTf)$_2$** was solved in the tetragonal space group *P*4$_3$ as four-component inversion twin. The twin law 0 -1 0 -1 0 0 0 0 -1 and the two inversion laws were applied and their ratio was refined to 0.16(2), 0.08(2) and 0.08(2). The asymmetric unit contains four molecules and the corresponding anions. One complex molecule and one of the anions were found to be disordered over two positions. The disorder was refined with the help of same distance restraints, similarity restraints for anisotropic displacement parameters and advanced rigid bond restraints. Some atoms with very similar positions were set to the same anisotropic displacement parameters. The two disorder rations were allowed to refine freely and their ratios converged to 0.973(6) and 0.634(12).

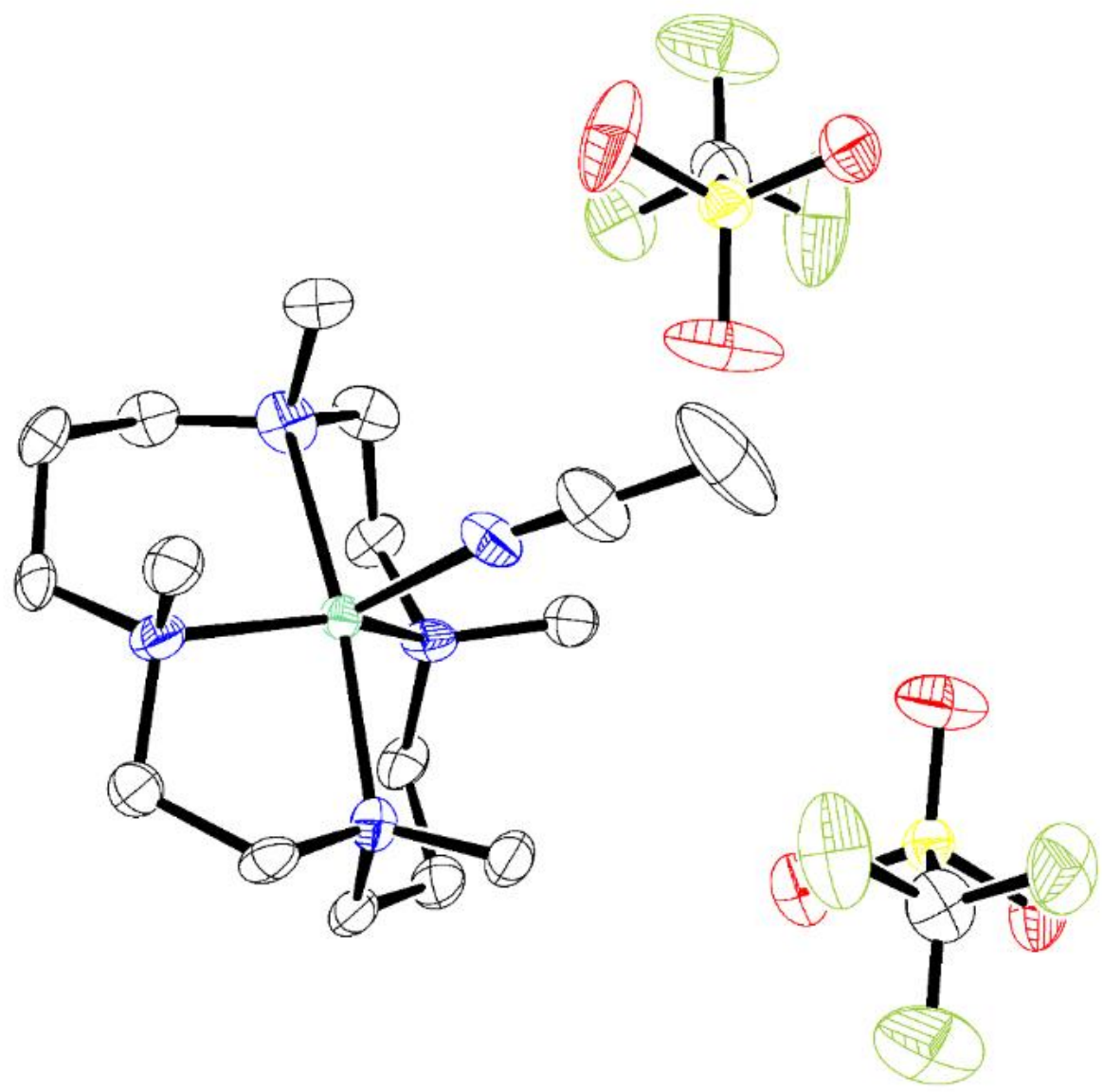

Figure S6. Molecular structure of [(TMC)Co^{II}(CH$_3$CN)](OTf)$_2$ (**4a-(OTf)$_2$**).

Table S27. Crystal data and structure refinement for **4a-(OTf)$_2$**.

CCDC No	2062705	
Empirical formula	$C_{72}H_{140}Co_4F_{24}N_{20}O_{24}S_8$	
Formula weight	2618.23	
Temperature	100(2) K	
Wavelength	0.71073 Å	
Crystal system	Tetragonal	
Space group	*P*4$_3$	
Unit cell dimensions	a = 13.8313(15) Å	α = 90°
	b = 13.8313(15) Å	β = 90°
	c = 58.030(9) Å	γ = 90°
Volume	11101(3) Å^3	
Z	4	
Density (calculated)	1.567 Mg/m^3	
Absorption coefficient	0.851 mm^{-1}	
F(000)	5424	
Crystal size	0.488 x 0.237 x 0.096 mm^3	
Theta range for data collection	1.472 to 26.021°.	
Index ranges	-17 ≤ h ≤ 17, -17 ≤ k ≤ 17, -71 ≤ l ≤ 71	
Reflections collected	135844	
Independent reflections	21857 [R(int) = 0.0961]	
Completeness to theta = 25.242°	100.0 %	
Absorption correction	Semi-empirical from equivalents	
Refinement method	Full-matrix least-squares on *F*^{2}	
Data / restraints / parameters	21857 / 3972 / 1531	
Goodness-of-fit on *F*^{2}	1.023	
Final R indices [I>2σ(I)]	R1 = 0.0577, wR2 = 0.1340	
R indices (all data)	R1 = 0.0726, wR2 = 0.1431	
Extinction coefficient	0.00055(7)	
Largest diff. peak and hole	0.657 and -0.569 eÅ^{-3}	

Table S28. Atomic coordinates (x 10^4) and equivalent isotropic displacement parameters ($Å^2$x 10^3) for **4a-(OTf)$_2$**. U(eq) is defined as one third of the trace of the orthogonalized U^{ij} tensor.

	x	y	z	U(eq)
Co11	14684(1)	-370(1)	4299(1)	21(1)
N11	14198(6)	-1791(6)	4236(2)	31(2)
N21	13127(6)	-41(6)	4307(2)	31(2)
N31	15031(6)	979(6)	4152(2)	26(2)
N41	16206(6)	-704(6)	4235(2)	28(2)
C11	13155(7)	-1719(8)	4166(2)	33(2)
C21	12652(8)	-1015(7)	4319(2)	34(3)
C31	12805(8)	468(8)	4099(2)	34(3)
C41	13306(8)	1433(8)	4054(2)	36(3)
C51	14338(8)	1386(8)	3976(2)	32(2)
C61	15961(7)	847(8)	4027(2)	29(2)
C71	16660(8)	249(8)	4171(2)	31(2)
C81	16332(8)	-1373(7)	4038(2)	33(2)
C91	15797(8)	-2362(8)	4067(2)	34(3)
C101	14710(7)	-2317(8)	4046(2)	31(2)
C111	14283(9)	-2377(7)	4452(2)	33(3)
C121	12812(9)	530(8)	4515(2)	37(2)
C131	15159(9)	1699(8)	4342(2)	37(2)
C141	16750(7)	-1053(8)	4440(2)	31(2)
N51	14848(7)	-307(6)	4647(2)	28(2)
C151	15102(11)	-683(10)	5084(2)	46(3)
C161	14961(9)	-461(7)	4838(2)	29(2)
Co12	9330(1)	4622(1)	4300(1)	21(1)
N12	9796(6)	3203(6)	4224(2)	26(2)
N22	10877(5)	4945(5)	4321(2)	23(2)
N32	8998(6)	5981(6)	4159(1)	25(2)
N42	7798(6)	4312(6)	4228(2)	25(2)
C12	10851(7)	3279(7)	4159(2)	27(2)
C22	11345(7)	3980(7)	4322(2)	28(2)
C32	11232(8)	5464(8)	4115(2)	30(3)
C42	10751(8)	6452(7)	4074(2)	30(2)
C52	9723(7)	6387(8)	3991(2)	30(2)
C62	8065(7)	5878(7)	4032(2)	24(2)
C72	7359(8)	5270(7)	4170(2)	29(2)
C82	7687(7)	3658(7)	4028(2)	28(2)
C92	8197(7)	2674(7)	4049(2)	28(2)
C102	9290(7)	2701(8)	4030(2)	27(2)
C112	9695(8)	2581(7)	4435(2)	29(2)
C122	11163(8)	5465(8)	4533(2)	31(3)
C132	8863(8)	6672(7)	4354(2)	31(2)
C142	7234(7)	3912(7)	4427(2)	24(2)
N52	9142(7)	4618(6)	4647(1)	27(2)
C152	8832(11)	4226(12)	5083(2)	51(4)
C162	9000(8)	4457(8)	4840(2)	29(2)
Co13	4362(1)	5411(1)	5677(1)	20(1)
N13	3993(6)	4056(6)	5822(2)	28(2)
N23	2857(6)	5745(6)	5741(2)	27(2)
N33	4851(6)	6834(6)	5745(2)	26(2)
N43	5922(6)	5065(6)	5666(2)	32(2)
C13	3047(7)	4208(8)	5944(2)	30(2)
C23	2373(7)	4805(7)	5798(2)	29(2)
C33	2749(8)	6439(7)	5939(2)	31(2)
C43	3260(8)	7395(8)	5914(2)	35(3)
C53	4354(8)	7351(8)	5938(2)	30(2)
C63	5889(7)	6749(8)	5813(2)	33(2)
C73	6401(8)	6014(8)	5656(2)	35(3)
C83	6237(8)	4554(8)	5876(2)	33(3)
C93	5740(8)	3566(7)	5917(2)	31(2)
C103	4693(7)	3656(7)	5995(2)	27(2)
C113	3878(9)	3350(8)	5630(2)	35(3)
C123	2307(8)	6151(8)	5542(2)	30(2)
C133	4771(8)	7421(8)	5528(2)	32(2)
C143	6243(9)	4514(8)	5458(2)	38(3)
N53	4215(7)	5348(7)	5329(1)	33(2)
C153	3992(14)	5644(17)	4888(2)	95(8)
C163	4116(11)	5476(10)	5135(2)	45(3)
Co14	9641(1)	10430(1)	5688(1)	20(1)
N14	11153(7)	10737(6)	5758(2)	31(2)
N24	10014(6)	9028(6)	5815(2)	30(2)
N34	8087(7)	10084(6)	5680(2)	35(2)
N44	9154(6)	11844(6)	5768(2)	32(2)
C14	11636(8)	9781(8)	5812(2)	37(3)
C24	10956(8)	9151(8)	5941(2)	39(3)
C34	9335(8)	8603(9)	5988(2)	36(3)
C44	8295(8)	8547(8)	5916(2)	35(3)
C54	7790(9)	9523(8)	5888(2)	40(3)
C64	7591(9)	11052(8)	5676(3)	45(3)
C74	8123(7)	11725(8)	5835(2)	38(3)
C84	9650(8)	12299(9)	5966(2)	35(3)
C94	10749(9)	12364(8)	5939(2)	36(3)
C104	11268(9)	11408(8)	5959(2)	34(3)
C114	11700(8)	11156(9)	5559(2)	32(3)
C124	10116(9)	8377(8)	5617(2)	39(3)
C134	7760(9)	9563(9)	5467(2)	40(3)
C144	9236(10)	12460(8)	5556(2)	36(3)
N1A4	10210(90)	9170(80)	5560(30)	37(7)
N2A4	8400(80)	10180(100)	5570(30)	36(8)
N3A4	9570(110)	11890(90)	5790(30)	33(8)
N4A4	11350(110)	11030(110)	5660(40)	33(10)
C1A4	9310(100)	8680(80)	5640(30)	38(10)

C2A4	8450(100)	9110(100)	5520(30)	37(12)
C3A4	7950(90)	10300(110)	5800(30)	38(9)
C4A4	7860(100)	11350(120)	5880(40)	39(13)
C5A4	8780(120)	11810(150)	5970(30)	38(12)
C6A4	10480(110)	12130(160)	5920(40)	34(11)
C7A4	11180(150)	11300(200)	5900(30)	35(11)
C8A4	12220(90)	10420(110)	5640(50)	37(3)
C9A4	12000(100)	9320(110)	5650(50)	37(3)
C10A4	11000(120)	9070(150)	5730(30)	39(3)
C11A4	10530(150)	8690(180)	5340(30)	39(3)
C12A4	7750(140)	10620(150)	5400(30)	40(3)
C13A4	9350(190)	12700(160)	5630(40)	36(3)
C14A4	11500(200)	11950(150)	5530(40)	32(3)
N54	9760(7)	10456(7)	5337(2)	31(2)
C154	10097(12)	10862(12)	4905(2)	57(4)
C164	9881(10)	10620(9)	5148(2)	37(3)
S15	14296(2)	4355(2)	4008(1)	25(1)
F15	14382(6)	3941(5)	4448(1)	41(2)
F25	15107(4)	5267(5)	4356(1)	35(2)
F35	13555(4)	5230(5)	4366(1)	34(2)
O15	15171(5)	3807(6)	3981(1)	33(2)
O25	14294(7)	5263(5)	3883(1)	37(2)
O35	13412(5)	3800(6)	3993(1)	32(2)
C15	14345(7)	4712(7)	4311(2)	26(2)
S16	6691(2)	6515(2)	4896(1)	31(1)
F16	7513(10)	8204(6)	4941(2)	106(4)
F26	8498(6)	7032(10)	4908(2)	101(4)
F36	7758(6)	7268(6)	5222(1)	60(2)
O16	5855(7)	6884(10)	5015(2)	83(4)
O26	7015(10)	5565(6)	4962(2)	78(4)
O36	6644(6)	6673(6)	4652(1)	41(2)
C16	7653(8)	7287(8)	4995(2)	44(3)
S17	9657(2)	9330(2)	4034(1)	30(1)
F17	9508(6)	8846(5)	4472(1)	46(2)
F27	8880(4)	10212(5)	4388(1)	40(2)
F37	10418(5)	10088(6)	4406(1)	43(2)
O17	8741(6)	8827(6)	3994(2)	42(2)
O27	9733(8)	10263(6)	3925(2)	56(3)
O37	10514(6)	8738(6)	4016(2)	37(2)
C17	9608(7)	9635(7)	4340(2)	30(2)
S18	11703(2)	3559(2)	5083(1)	29(1)
F18	12818(7)	2836(6)	4761(1)	60(2)
F28	13470(6)	2862(9)	5098(2)	87(3)
F38	12401(8)	1810(6)	5016(2)	75(3)
O18	11580(7)	3337(6)	5322(1)	43(2)
O28	10899(6)	3308(7)	4938(2)	50(2)
O38	12140(8)	4490(6)	5033(2)	55(3)
C18	12646(8)	2730(8)	4985(2)	43(3)
S19	17274(2)	1526(2)	4907(1)	31(1)
F19	15455(6)	2010(9)	4894(2)	85(3)
F29	16432(8)	3212(6)	4960(2)	79(3)
F39	16115(7)	2192(7)	5224(1)	65(2)
O19	17367(7)	1701(6)	4663(1)	46(2)
O29	18057(7)	1852(9)	5047(2)	70(3)
O39	16946(9)	563(6)	4961(2)	67(3)
C19	16270(8)	2283(8)	5000(2)	41(3)
S110	2269(11)	8584(9)	5074(3)	28(1)
F110	478(8)	7964(12)	5081(2)	76(4)
F210	1174(16)	7841(16)	4748(2)	60(6)
F310	1539(12)	6848(8)	5019(3)	67(4)
O110	1910(13)	9532(8)	5016(3)	51(4)
O210	2318(12)	8383(15)	5317(2)	38(4)
O310	3091(11)	8266(14)	4942(4)	60(6)
C110	1310(11)	7776(10)	4975(3)	41(3)
S1A10	2310(20)	8594(16)	5096(6)	28(1)
F1A10	972(16)	7250(20)	5121(4)	76(4)
F2A10	1280(30)	7740(20)	4773(4)	65(10)
F3A10	2296(19)	6807(13)	4946(4)	67(7)
O1A10	1520(20)	9330(16)	5092(5)	51(4)
O2A10	2640(20)	8370(30)	5317(5)	38(4)
O3A10	3013(19)	8770(20)	4920(5)	56(9)
C1A10	1670(20)	7530(20)	4998(6)	41(3)
S111	14532(2)	700(2)	5965(1)	27(1)
F111	15361(5)	-189(5)	5618(1)	39(2)
F211	13803(5)	-266(5)	5611(1)	40(2)
F311	14532(6)	1052(5)	5521(1)	49(2)
O111	14506(6)	-195(6)	6092(2)	39(2)
O211	13651(5)	1268(6)	5980(1)	30(2)
O311	15410(5)	1255(6)	5983(1)	30(2)
C111	14553(7)	306(7)	5663(2)	26(2)
S112	9416(2)	5715(2)	5958(1)	31(1)
F112	10126(5)	4918(6)	5585(1)	46(2)
F212	8580(5)	4873(5)	5604(1)	36(2)
F312	9285(7)	6197(5)	5522(1)	56(2)
O112	8521(6)	6225(6)	5997(2)	40(2)
O212	9504(8)	4785(6)	6062(2)	54(3)
O312	10279(6)	6320(6)	5973(2)	32(2)
C112	9357(7)	5435(7)	5652(2)	29(2)

Table S29. Bond lengths [Å] and angles [°] for **4a-(OTf)₂**.

Co11-N51	2.030(9)	C72-H7A2	0.9900
Co11-N31	2.107(8)	C72-H7B2	0.9900
Co11-N11	2.111(8)	C82-C92	1.538(13)
Co11-N41	2.188(8)	C82-H8A2	0.9900
Co11-N21	2.201(8)	C82-H8B2	0.9900
N11-C101	1.496(13)	C92-C102	1.516(12)
N11-C111	1.498(12)	C92-H9A2	0.9900
N11-C11	1.502(12)	C92-H9B2	0.9900
N21-C31	1.469(12)	C102-H10A2	0.9900
N21-C21	1.501(12)	C102-H10B2	0.9900
N21-C121	1.504(13)	C112-H11A2	0.9800
N31-C61	1.489(12)	C112-H11B2	0.9800
N31-C131	1.495(12)	C112-H11C2	0.9800
N31-C51	1.511(13)	C122-H12A2	0.9800
N41-C81	1.482(12)	C122-H12B2	0.9800
N41-C141	1.490(12)	C122-H12C2	0.9800
N41-C71	1.507(12)	C132-H13A2	0.9800
C11-C21	1.493(14)	C132-H13B2	0.9800
C11-H1A1	0.9900	C132-H13C2	0.9800
C11-H1B1	0.9900	C142-H14A2	0.9800
C21-H2A1	0.9900	C142-H14B2	0.9800
C21-H2B1	0.9900	C142-H14C2	0.9800
C31-C41	1.525(14)	N52-C162	1.158(11)
C31-H3A1	0.9900	C152-C162	1.470(13)
C31-H3B1	0.9900	C152-H15A2	0.9800
C41-C51	1.501(14)	C152-H15B2	0.9800
C41-H4A1	0.9900	C152-H15C2	0.9800
C41-H4B1	0.9900	Co13-N53	2.033(8)
C51-H5A1	0.9900	Co13-N33	2.117(8)
C51-H5B1	0.9900	Co13-N13	2.117(8)
C61-C71	1.523(13)	Co13-N23	2.164(9)
C61-H6A1	0.9900	Co13-N43	2.211(9)
C61-H6B1	0.9900	N13-C113	1.492(12)
C71-H7A1	0.9900	N13-C103	1.498(12)
C71-H7B1	0.9900	N13-C13	1.502(12)
C81-C91	1.564(14)	N23-C123	1.489(12)
C81-H8A1	0.9900	N23-C23	1.501(12)
C81-H8B1	0.9900	N23-C33	1.505(12)
C91-C101	1.510(13)	N33-C63	1.493(12)
C91-H9A1	0.9900	N33-C53	1.499(12)
C91-H9B1	0.9900	N33-C133	1.503(12)
C101-H10A1	0.9900	N43-C73	1.472(12)
C101-H10B1	0.9900	N43-C83	1.476(13)
C111-H11A1	0.9800	N43-C143	1.496(13)
C111-H11B1	0.9800	C13-C23	1.506(13)
C111-H11C1	0.9800	C13-H1A3	0.9900
C121-H12A1	0.9800	C13-H1B3	0.9900
C121-H12B1	0.9800	C23-H2A3	0.9900
C121-H12C1	0.9800	C23-H2B3	0.9900
C131-H13A1	0.9800	C33-C43	1.506(14)
C131-H13B1	0.9800	C33-H3A3	0.9900
C131-H13C1	0.9800	C33-H3B3	0.9900
C141-H14A1	0.9800	C43-C53	1.520(14)
C141-H14B1	0.9800	C43-H4A3	0.9900
C141-H14C1	0.9800	C43-H4B3	0.9900
N51-C161	1.143(11)	C53-H5A3	0.9900
C151-C161	1.473(13)	C53-H5B3	0.9900
C151-H15A1	0.9800	C63-C73	1.537(14)
C151-H15B1	0.9800	C63-H6A3	0.9900
C151-H15C1	0.9800	C63-H6B3	0.9900
Co12-N52	2.027(8)	C73-H7A3	0.9900
Co12-N32	2.102(8)	C73-H7B3	0.9900
Co12-N12	2.112(8)	C83-C93	1.547(13)
Co12-N22	2.188(8)	C83-H8A3	0.9900
Co12-N42	2.202(8)	C83-H8B3	0.9900
N12-C102	1.500(12)	C93-C103	1.523(13)
N12-C112	1.500(12)	C93-H9A3	0.9900
N12-C12	1.513(12)	C93-H9B3	0.9900
N22-C32	1.478(12)	C103-H10A3	0.9900
N22-C122	1.479(12)	C103-H10B3	0.9900
N22-C22	1.482(11)	C113-H11A3	0.9800
N32-C62	1.493(12)	C113-H11B3	0.9800
N32-C132	1.496(12)	C113-H11C3	0.9800
N32-C52	1.505(12)	C123-H12A3	0.9800
N42-C82	1.483(12)	C123-H12B3	0.9800
N42-C72	1.496(12)	C123-H12C3	0.9800
N42-C142	1.496(11)	C133-H13A3	0.9800
C12-C22	1.517(13)	C133-H13B3	0.9800
C12-H1A2	0.9900	C133-H13C3	0.9800
C12-H1B2	0.9900	C143-H14A3	0.9800
C22-H2A2	0.9900	C143-H14B3	0.9800
C22-H2B2	0.9900	C143-H14C3	0.9800
C32-C42	1.539(12)	N53-C163	1.146(12)
C32-H3A2	0.9900	C153-C163	1.465(14)
C32-H3B2	0.9900	C153-H15A3	0.9800
C42-C52	1.504(13)	C153-H15B3	0.9800
C42-H4A2	0.9900	C153-H15C3	0.9800
C42-H4B2	0.9900	Co14-N2A4	1.88(11)
C52-H5A2	0.9900	Co14-N54	2.040(10)
C52-H5B2	0.9900	Co14-N1A4	2.05(11)
C62-C72	1.518(13)	Co14-N3A4	2.12(11)
C62-H6A2	0.9900	Co14-N44	2.120(9)
C62-H6B2	0.9900	Co14-N24	2.137(9)

Co14-N14	2.171(9)
Co14-N34	2.203(10)
Co14-C3A4	2.44(11)
Co14-C1A4	2.48(12)
N14-C114	1.494(13)
N14-C104	1.503(13)
N14-C14	1.516(13)
N24-C124	1.468(13)
N24-C34	1.496(13)
N24-C24	1.504(13)
N34-C54	1.495(14)
N34-C134	1.502(13)
N34-C64	1.504(14)
N44-C84	1.480(13)
N44-C74	1.486(13)
N44-C144	1.498(13)
C14-C24	1.484(14)
C14-H1A4	0.9900
C14-H1B4	0.9900
C24-H2A4	0.9900
C24-H2B4	0.9900
C34-C44	1.500(15)
C34-H3A4	0.9900
C34-H3B4	0.9900
C44-C54	1.527(15)
C44-H4A4	0.9900
C44-H4B4	0.9900
C54-H5A4	0.9900
C54-H5B4	0.9900
C64-C74	1.500(15)
C64-H6A4	0.9900
C64-H6B4	0.9900
C74-H7A4	0.9900
C74-H7B4	0.9900
C84-C94	1.531(14)
C84-H8A4	0.9900
C84-H8B4	0.9900
C94-C104	1.509(14)
C94-H9A4	0.9900
C94-H9B4	0.9900
C104-H10A4	0.9900
C104-H10B4	0.9900
C114-H11A4	0.9800
C114-H11B4	0.9800
C114-H11C4	0.9800
C124-H12A4	0.9800
C124-H12B4	0.9800
C124-H12C4	0.9800
C134-H13A4	0.9800
C134-H13B4	0.9800
C134-H13C4	0.9800
C144-H14A4	0.9800
C144-H14B4	0.9800
C144-H14C4	0.9800
N1A4-C11A4	1.50(2)
N1A4-C10A4	1.50(3)
N1A4-C1A4	1.50(2)
N2A4-C12A4	1.49(2)
N2A4-C3A4	1.49(2)
N2A4-C2A4	1.50(2)
N3A4-C6A4	1.50(3)
N3A4-C13A4	1.50(3)
N3A4-C5A4	1.50(3)
N4A4-C8A4	1.48(3)
N4A4-C7A4	1.49(2)
N4A4-C14A4	1.49(3)
C1A4-C2A4	1.50(2)
C1A4-H1AA4	0.9900
C1A4-H1AB4	0.9900
C2A4-H2AA4	0.9900
C2A4-H2AB4	0.9900
C3A4-C4A4	1.52(3)
C3A4-H3AA4	0.9900
C3A4-H3AB4	0.9900
C4A4-C5A4	1.51(3)
C4A4-H4AA4	0.9900
C4A4-H4AB4	0.9900
C5A4-H5AA4	0.9900
C5A4-H5AB4	0.9900
C6A4-C7A4	1.52(3)
C6A4-H6AA4	0.9900
C6A4-H6AB4	0.9900
C7A4-H7AA4	0.9900
C7A4-H7AB4	0.9900
C8A4-C9A4	1.55(3)
C8A4-H8AA4	0.9900
C8A4-H8AB4	0.9900
C9A4-C10A4	1.52(3)
C9A4-H9AA4	0.9900
C9A4-H9AB4	0.9900
C10A4-H10C4	0.9900
C10A4-H10D4	0.9900
C11A4-H11D4	0.9800
C11A4-H11E4	0.9800
C11A4-H11F4	0.9800

C12A4-H12D4	0.9800
C12A4-H12E4	0.9800
C12A4-H12F4	0.9800
C13A4-H13D4	0.9800
C13A4-H13E4	0.9800
C13A4-H13F4	0.9800
C14A4-H14D4	0.9800
C14A4-H14E4	0.9800
C14A4-H14F4	0.9800
N54-C164	1.134(12)
C154-C164	1.481(14)
C154-H15A4	0.9800
C154-H15B4	0.9800
C154-H15C4	0.9800
S15-O15	1.436(7)
S15-O35	1.446(8)
S15-O25	1.450(8)
S15-C15	1.828(10)
F15-C15	1.331(11)
F25-C15	1.329(11)
F35-C15	1.346(10)
S16-O36	1.434(8)
S16-O26	1.438(9)
S16-O16	1.440(9)
S16-C16	1.800(11)
F16-C16	1.320(13)
F26-C16	1.321(13)
F36-C16	1.330(13)
S17-O27	1.440(9)
S17-O37	1.445(8)
S17-O17	1.464(8)
S17-C17	1.821(10)
F17-C17	1.341(12)
F27-C17	1.316(11)
F37-C17	1.342(11)
S18-O18	1.432(8)
S18-O28	1.437(8)
S18-O38	1.452(8)
S18-C18	1.826(11)
F18-C18	1.329(13)
F28-C18	1.327(13)
F38-C18	1.330(13)
S19-O29	1.428(9)
S19-O19	1.439(8)
S19-O39	1.443(9)
S19-C19	1.821(10)
F19-C19	1.338(13)
F29-C19	1.324(13)
F39-C19	1.327(13)
S110-O310	1.438(16)
S110-O110	1.441(16)
S110-O210	1.442(18)
S110-C110	1.826(15)
F110-C110	1.334(16)
F210-C110	1.331(16)
F310-C110	1.347(16)
S1A10-O2A10	1.40(4)
S1A10-O3A10	1.43(3)
S1A10-O1A10	1.49(3)
S1A10-C1A10	1.81(3)
F1A10-C1A10	1.26(4)
F2A10-C1A10	1.44(4)
F3A10-C1A10	1.35(4)
S111-O311	1.440(7)
S111-O111	1.443(8)
S111-O211	1.453(8)
S111-C111	1.833(10)
F111-C111	1.336(11)
F211-C111	1.339(11)
F311-C111	1.321(11)
S112-O212	1.426(9)
S112-O112	1.443(8)
S112-O312	1.460(8)
S112-C112	1.819(10)
F112-C112	1.339(11)
F212-C112	1.355(11)
F312-C112	1.300(11)
N51-Co11-N31	109.9(3)
N51-Co11-N11	104.4(4)
N31-Co11-N11	145.7(3)
N51-Co11-N41	94.1(4)
N31-Co11-N41	84.2(3)
N11-Co11-N41	94.5(3)
N51-Co11-N21	94.6(4)
N31-Co11-N21	92.8(3)
N11-Co11-N21	83.4(3)
N41-Co11-N21	171.3(3)
C101-N11-C111	108.4(8)
C101-N11-C11	106.7(8)
C111-N11-C11	109.8(9)
C101-N11-Co11	115.5(7)
C111-N11-Co11	109.4(6)
C11-N11-Co11	106.9(6)
C31-N21-C21	109.6(9)

C31-N21-C121	108.7(8)
C21-N21-C121	107.9(8)
C31-N21-Co11	112.2(7)
C21-N21-Co11	104.1(6)
C121-N21-Co11	114.2(7)
C61-N31-C131	109.8(8)
C61-N31-C51	105.2(8)
C131-N31-C51	109.0(8)
C61-N31-Co11	106.7(6)
C131-N31-Co11	108.5(6)
C51-N31-Co11	117.4(6)
C81-N41-C141	110.8(8)
C81-N41-C71	107.9(8)
C141-N41-C71	105.6(8)
C81-N41-Co11	112.2(6)
C141-N41-Co11	114.7(6)
C71-N41-Co11	105.0(6)
C21-C11-N11	109.2(9)
C21-C11-H1A1	109.8
N11-C11-H1A1	109.8
C21-C11-H1B1	109.8
N11-C11-H1B1	109.8
H1A1-C11-H1B1	108.3
C11-C21-N21	110.8(9)
C11-C21-H2A1	109.5
N21-C21-H2A1	109.5
C11-C21-H2B1	109.5
N21-C21-H2B1	109.5
H2A1-C21-H2B1	108.1
N21-C31-C41	114.9(9)
N21-C31-H3A1	108.5
C41-C31-H3A1	108.5
N21-C31-H3B1	108.5
C41-C31-H3B1	108.5
H3A1-C31-H3B1	107.5
C51-C41-C31	116.5(10)
C51-C41-H4A1	108.2
C31-C41-H4A1	108.2
C51-C41-H4B1	108.2
C31-C41-H4B1	108.2
H4A1-C41-H4B1	107.3
C41-C51-N31	114.5(9)
C41-C51-H5A1	108.6
N31-C51-H5A1	108.6
C41-C51-H5B1	108.6
N31-C51-H5B1	108.6
H5A1-C51-H5B1	107.6
N31-C61-C71	110.3(8)
N31-C61-H6A1	109.6
C71-C61-H6A1	109.6
N31-C61-H6B1	109.6
C71-C61-H6B1	109.6
H6A1-C61-H6B1	108.1
N41-C71-C61	110.2(8)
N41-C71-H7A1	109.6
C61-C71-H7A1	109.6
N41-C71-H7B1	109.6
C61-C71-H7B1	109.6
H7A1-C71-H7B1	108.1
N41-C81-C91	113.9(9)
N41-C81-H8A1	108.8
C91-C81-H8A1	108.8
N41-C81-H8B1	108.8
C91-C81-H8B1	108.8
H8A1-C81-H8B1	107.7
C101-C91-C81	115.2(9)
C101-C91-H9A1	108.5
C81-C91-H9A1	108.5
C101-C91-H9B1	108.5
C81-C91-H9B1	108.5
H9A1-C91-H9B1	107.5
N11-C101-C91	115.6(9)
N11-C101-H10A1	108.4
C91-C101-H10A1	108.4
N11-C101-H10B1	108.4
C91-C101-H10B1	108.4
H10A1-C101-H10B1	107.4
N11-C111-H11A1	109.5
N11-C111-H11B1	109.5
H11A1-C111-H11B1	109.5
N11-C111-H11C1	109.5
H11A1-C111-H11C1	109.5
H11B1-C111-H11C1	109.5
N21-C121-H12A1	109.5
N21-C121-H12B1	109.5
H12A1-C121-H12B1	109.5
N21-C121-H12C1	109.5
H12A1-C121-H12C1	109.5
H12B1-C121-H12C1	109.5
N31-C131-H13A1	109.5
N31-C131-H13B1	109.5
H13A1-C131-H13B1	109.5
N31-C131-H13C1	109.5
H13A1-C131-H13C1	109.5
H13B1-C131-H13C1	109.5
N41-C141-H14A1	109.5
N41-C141-H14B1	109.5
H14A1-C141-H14B1	109.5
N41-C141-H14C1	109.5
H14A1-C141-H14C1	109.5
H14B1-C141-H14C1	109.5
C161-N51-Co11	166.8(9)
C161-C151-H15A1	109.5
C161-C151-H15B1	109.5
H15A1-C151-H15B1	109.5
C161-C151-H15C1	109.5
H15A1-C151-H15C1	109.5
H15B1-C151-H15C1	109.5
N51-C161-C151	178.7(12)
N52-Co12-N32	111.2(3)
N52-Co12-N12	104.1(4)
N32-Co12-N12	144.7(3)
N52-Co12-N22	94.2(4)
N32-Co12-N22	93.0(3)
N12-Co12-N22	84.4(3)
N52-Co12-N42	93.7(4)
N32-Co12-N42	83.7(3)
N12-Co12-N42	94.2(3)
N22-Co12-N42	172.2(3)
C102-N12-C112	107.7(8)
C102-N12-C12	107.0(8)
C112-N12-C12	109.6(8)
C102-N12-Co12	116.5(6)
C112-N12-Co12	109.5(6)
C12-N12-Co12	106.4(6)
C32-N22-C122	110.3(8)
C32-N22-C22	107.1(8)
C122-N22-C22	108.5(8)
C32-N22-Co12	112.4(6)
C122-N22-Co12	113.9(6)
C22-N22-Co12	104.1(6)
C62-N32-C132	109.1(8)
C62-N32-C52	107.1(7)
C132-N32-C52	109.6(8)
C62-N32-Co12	107.2(6)
C132-N32-Co12	107.6(6)
C52-N32-Co12	116.1(6)
C82-N42-C72	108.8(8)
C82-N42-C142	108.9(8)
C72-N42-C142	106.8(7)
C82-N42-Co12	111.6(6)
C72-N42-Co12	105.1(6)
C142-N42-Co12	115.4(6)
N12-C12-C22	108.8(8)
N12-C12-H1A2	109.9
C22-C12-H1A2	109.9
N12-C12-H1B2	109.9
C22-C12-H1B2	109.9
H1A2-C12-H1B2	108.3
N22-C22-C12	112.1(8)
N22-C22-H2A2	109.2
C12-C22-H2A2	109.2
N22-C22-H2B2	109.2
C12-C22-H2B2	109.2
H2A2-C22-H2B2	107.9
N22-C32-C42	114.3(9)
N22-C32-H3A2	108.7
C42-C32-H3A2	108.7
N22-C32-H3B2	108.7
C42-C32-H3B2	108.7
H3A2-C32-H3B2	107.6
C52-C42-C32	113.8(9)
C52-C42-H4A2	108.8
C32-C42-H4A2	108.8
C52-C42-H4B2	108.8
C32-C42-H4B2	108.8
H4A2-C42-H4B2	107.7
C42-C52-N32	116.5(9)
C42-C52-H5A2	108.2
N32-C52-H5A2	108.2
C42-C52-H5B2	108.2
N32-C52-H5B2	108.2
H5A2-C52-H5B2	107.3
N32-C62-C72	110.4(8)
N32-C62-H6A2	109.6
C72-C62-H6A2	109.6
N32-C62-H6B2	109.6
C72-C62-H6B2	109.6
H6A2-C62-H6B2	108.1
N42-C72-C62	110.4(8)
N42-C72-H7A2	109.6
C62-C72-H7A2	109.6
N42-C72-H7B2	109.6
C62-C72-H7B2	109.6
H7A2-C72-H7B2	108.1
N42-C82-C92	115.3(9)
N42-C82-H8A2	108.4
C92-C82-H8A2	108.4
N42-C82-H8B2	108.4
C92-C82-H8B2	108.4

H8A2-C82-H8B2	107.5
C102-C92-C82	115.4(9)
C102-C92-H9A2	108.4
C82-C92-H9A2	108.4
C102-C92-H9B2	108.4
C82-C92-H9B2	108.4
H9A2-C92-H9B2	107.5
N12-C102-C92	114.8(9)
N12-C102-H10A2	108.6
C92-C102-H10A2	108.6
N12-C102-H10B2	108.6
C92-C102-H10B2	108.6
H10A2-C102-H10B2	107.5
N12-C112-H11A2	109.5
N12-C112-H11B2	109.5
H11A2-C112-H11B2	109.5
N12-C112-H11C2	109.5
H11A2-C112-H11C2	109.5
H11B2-C112-H11C2	109.5
N22-C122-H12A2	109.5
N22-C122-H12B2	109.5
H12A2-C122-H12B2	109.5
N22-C122-H12C2	109.5
H12A2-C122-H12C2	109.5
H12B2-C122-H12C2	109.5
N32-C132-H13A2	109.5
N32-C132-H13B2	109.5
H13A2-C132-H13B2	109.5
N32-C132-H13C2	109.5
H13A2-C132-H13C2	109.5
H13B2-C132-H13C2	109.5
N42-C142-H14A2	109.5
N42-C142-H14B2	109.5
H14A2-C142-H14B2	109.5
N42-C142-H14C2	109.5
H14A2-C142-H14C2	109.5
H14B2-C142-H14C2	109.5
C162-N52-Co12	168.8(9)
C162-C152-H15A2	109.5
C162-C152-H15B2	109.5
H15A2-C152-H15B2	109.5
C162-C152-H15C2	109.5
H15A2-C152-H15C2	109.5
H15B2-C152-H15C2	109.5
N52-C162-C152	178.5(13)
N53-Co13-N33	104.8(4)
N53-Co13-N13	109.5(4)
N33-Co13-N13	145.7(3)
N53-Co13-N23	94.7(4)
N33-Co13-N23	94.4(3)
N13-Co13-N23	83.6(3)
N53-Co13-N43	93.4(4)
N33-Co13-N43	84.0(3)
N13-Co13-N43	93.2(3)
N23-Co13-N43	171.9(3)
C113-N13-C103	109.0(8)
C113-N13-C13	110.6(8)
C103-N13-C13	107.5(8)
C113-N13-Co13	107.9(6)
C103-N13-Co13	115.9(6)
C13-N13-Co13	105.9(6)
C123-N23-C23	105.7(8)
C123-N23-C33	107.4(8)
C23-N23-C33	109.7(8)
C123-N23-Co13	116.1(6)
C23-N23-Co13	106.4(6)
C33-N23-Co13	111.2(6)
C63-N33-C53	106.3(8)
C63-N33-C133	109.6(8)
C53-N33-C133	109.6(8)
C63-N33-Co13	106.4(6)
C53-N33-Co13	115.9(6)
C133-N33-Co13	108.9(6)
C73-N43-C83	109.0(8)
C73-N43-C143	106.8(8)
C83-N43-C143	109.7(9)
C73-N43-Co13	104.3(7)
C83-N43-Co13	111.5(7)
C143-N43-Co13	115.0(7)
N13-C13-C23	110.5(8)
N13-C13-H1A3	109.6
C23-C13-H1A3	109.6
N13-C13-H1B3	109.6
C23-C13-H1B3	109.6
H1A3-C13-H1B3	108.1
N23-C23-C13	109.0(8)
N23-C23-H2A3	109.9
C13-C23-H2A3	109.9
N23-C23-H2B3	109.9
C13-C23-H2B3	109.9
H2A3-C23-H2B3	108.3
N23-C33-C43	116.1(9)
N23-C33-H3A3	108.3
C43-C33-H3A3	108.3
N23-C33-H3B3	108.3
C43-C33-H3B3	108.3
H3A3-C33-H3B3	107.4
C33-C43-C53	115.1(9)
C33-C43-H4A3	108.5
C53-C43-H4A3	108.5
C33-C43-H4B3	108.5
C53-C43-H4B3	108.5
H4A3-C43-H4B3	107.5
N33-C53-C43	114.0(9)
N33-C53-H5A3	108.7
C43-C53-H5A3	108.7
N33-C53-H5B3	108.7
C43-C53-H5B3	108.7
H5A3-C53-H5B3	107.6
N33-C63-C73	109.8(8)
N33-C63-H6A3	109.7
C73-C63-H6A3	109.7
N33-C63-H6B3	109.7
C73-C63-H6B3	109.7
H6A3-C63-H6B3	108.2
N43-C73-C63	111.1(9)
N43-C73-H7A3	109.4
C63-C73-H7A3	109.4
N43-C73-H7B3	109.4
C63-C73-H7B3	109.4
H7A3-C73-H7B3	108.0
N43-C83-C93	114.6(9)
N43-C83-H8A3	108.6
C93-C83-H8A3	108.6
N43-C83-H8B3	108.6
C93-C83-H8B3	108.6
H8A3-C83-H8B3	107.6
C103-C93-C83	113.3(9)
C103-C93-H9A3	108.9
C83-C93-H9A3	108.9
C103-C93-H9B3	108.9
C83-C93-H9B3	108.9
H9A3-C93-H9B3	107.7
N13-C103-C93	116.5(9)
N13-C103-H10A3	108.2
C93-C103-H10A3	108.2
N13-C103-H10B3	108.2
C93-C103-H10B3	108.2
H10A3-C103-H10B3	107.3
N13-C113-H11A3	109.5
N13-C113-H11B3	109.5
H11A3-C113-H11B3	109.5
N13-C113-H11C3	109.5
H11A3-C113-H11C3	109.5
H11B3-C113-H11C3	109.5
N23-C123-H12A3	109.5
N23-C123-H12B3	109.5
H12A3-C123-H12B3	109.5
N23-C123-H12C3	109.5
H12A3-C123-H12C3	109.5
H12B3-C123-H12C3	109.5
N33-C133-H13A3	109.5
N33-C133-H13B3	109.5
H13A3-C133-H13B3	109.5
N33-C133-H13C3	109.5
H13A3-C133-H13C3	109.5
H13B3-C133-H13C3	109.5
N43-C143-H14A3	109.5
N43-C143-H14B3	109.5
H14A3-C143-H14B3	109.5
N43-C143-H14C3	109.5
H14A3-C143-H14C3	109.5
H14B3-C143-H14C3	109.5
C163-N53-Co13	168.6(11)
C163-C153-H15A3	109.5
C163-C153-H15B3	109.5
H15A3-C153-H15B3	109.5
C163-C153-H15C3	109.5
H15A3-C153-H15C3	109.5
H15B3-C153-H15C3	109.5
N53-C163-C153	179.7(17)
N2A4-Co14-N54	74(5)
N2A4-Co14-N1A4	94(5)
N54-Co14-N1A4	67(4)
N2A4-Co14-N3A4	104(5)
N54-Co14-N3A4	106(5)
N1A4-Co14-N3A4	159(6)
N54-Co14-N44	103.2(4)
N54-Co14-N24	109.9(4)
N44-Co14-N24	146.9(4)
N54-Co14-N14	96.0(4)
N44-Co14-N14	94.9(3)
N24-Co14-N14	83.2(3)
N54-Co14-N34	93.5(4)
N44-Co14-N34	83.9(3)
N24-Co14-N34	92.6(3)
N14-Co14-N34	170.4(4)
N2A4-Co14-C3A4	38(2)
N54-Co14-C3A4	111(4)
N1A4-Co14-C3A4	114(5)

N3A4-Co14-C3A4	87(4)
N2A4-Co14-C1A4	67(3)
N54-Co14-C1A4	86(5)
N1A4-Co14-C1A4	37(2)
N3A4-Co14-C1A4	163(5)
C3A4-Co14-C1A4	77(3)
C114-N14-C104	107.9(9)
C114-N14-C14	106.0(9)
C104-N14-C14	109.2(9)
C114-N14-Co14	114.8(7)
C104-N14-Co14	111.6(7)
C14-N14-Co14	107.0(6)
C124-N24-C34	110.2(9)
C124-N24-C24	111.6(9)
C34-N24-C24	105.2(9)
C124-N24-Co14	108.0(7)
C34-N24-Co14	115.9(7)
C24-N24-Co14	105.9(6)
C54-N34-C134	109.5(9)
C54-N34-C64	110.4(10)
C134-N34-C64	106.2(9)
C54-N34-Co14	111.4(7)
C134-N34-Co14	114.5(8)
C64-N34-Co14	104.6(7)
C84-N44-C74	106.8(9)
C84-N44-C144	111.1(9)
C74-N44-C144	110.4(10)
C84-N44-Co14	114.6(7)
C74-N44-Co14	105.0(6)
C144-N44-Co14	108.7(7)
C24-C14-N14	109.8(10)
C24-C14-H1A4	109.7
N14-C14-H1A4	109.7
C24-C14-H1B4	109.7
N14-C14-H1B4	109.7
H1A4-C14-H1B4	108.2
C14-C24-N24	111.6(10)
C14-C24-H2A4	109.3
N24-C24-H2A4	109.3
C14-C24-H2B4	109.3
N24-C24-H2B4	109.3
H2A4-C24-H2B4	108.0
N24-C34-C44	115.8(10)
N24-C34-H3A4	108.3
C44-C34-H3A4	108.3
N24-C34-H3B4	108.3
C44-C34-H3B4	108.3
H3A4-C34-H3B4	107.4
C34-C44-C54	115.0(10)
C34-C44-H4A4	108.5
C54-C44-H4A4	108.5
C34-C44-H4B4	108.5
C54-C44-H4B4	108.5
H4A4-C44-H4B4	107.5
N34-C54-C44	114.7(10)
N34-C54-H5A4	108.6
C44-C54-H5A4	108.6
N34-C54-H5B4	108.6
C44-C54-H5B4	108.6
H5A4-C54-H5B4	107.6
C74-C64-N34	108.6(10)
C74-C64-H6A4	110.0
N34-C64-H6A4	110.0
C74-C64-H6B4	110.0
N34-C64-H6B4	110.0
H6A4-C64-H6B4	108.3
N44-C74-C64	112.4(10)
N44-C74-H7A4	109.1
C64-C74-H7A4	109.1
N44-C74-H7B4	109.1
C64-C74-H7B4	109.1
H7A4-C74-H7B4	107.9
N44-C84-C94	113.9(10)
N44-C84-H8A4	108.8
C94-C84-H8A4	108.8
N44-C84-H8B4	108.8
C94-C84-H8B4	108.8
H8A4-C84-H8B4	107.7
C104-C94-C84	114.4(10)
C104-C94-H9A4	108.7
C84-C94-H9A4	108.7
C104-C94-H9B4	108.7
C84-C94-H9B4	108.7
H9A4-C94-H9B4	107.6
N14-C104-C94	115.4(9)
N14-C104-H10A4	108.4
C94-C104-H10A4	108.4
N14-C104-H10B4	108.4
C94-C104-H10B4	108.4
H10A4-C104-H10B4	107.5
N14-C114-H11A4	109.5
N14-C114-H11B4	109.5
H11A4-C114-H11B4	109.5
N14-C114-H11C4	109.5
H11A4-C114-H11C4	109.5
H11B4-C114-H11C4	109.5
N24-C124-H12A4	109.5
N24-C124-H12B4	109.5
H12A4-C124-H12B4	109.5
N24-C124-H12C4	109.5
H12A4-C124-H12C4	109.5
H12B4-C124-H12C4	109.5
N34-C134-H13A4	109.5
N34-C134-H13B4	109.5
H13A4-C134-H13B4	109.5
N34-C134-H13C4	109.5
H13A4-C134-H13C4	109.5
H13B4-C134-H13C4	109.5
N44-C144-H14A4	109.5
N44-C144-H14B4	109.5
H14A4-C144-H14B4	109.5
N44-C144-H14C4	109.5
H14A4-C144-H14C4	109.5
H14B4-C144-H14C4	109.5
C11A4-N1A4-C10A4	108(4)
C11A4-N1A4-C1A4	109(4)
C10A4-N1A4-C1A4	109(4)
C11A4-N1A4-Co14	144(10)
C10A4-N1A4-Co14	96(10)
C1A4-N1A4-Co14	87(4)
C12A4-N2A4-C3A4	109(4)
C12A4-N2A4-C2A4	108(4)
C3A4-N2A4-C2A4	107(3)
C12A4-N2A4-Co14	136(7)
C3A4-N2A4-Co14	92(5)
C2A4-N2A4-Co14	102(4)
C6A4-N3A4-C13A4	109(4)
C6A4-N3A4-C5A4	107(4)
C13A4-N3A4-C5A4	110(4)
C6A4-N3A4-Co14	109(9)
C13A4-N3A4-Co14	123(10)
C5A4-N3A4-Co14	99(6)
C8A4-N4A4-C7A4	109(4)
C8A4-N4A4-C14A4	111(4)
C7A4-N4A4-C14A4	108(4)
C2A4-C1A4-N1A4	109(3)
C2A4-C1A4-Co14	79(3)
N1A4-C1A4-Co14	56(3)
C2A4-C1A4-H1AA4	109.9
N1A4-C1A4-H1AA4	109.9
Co14-C1A4-H1AA4	165.7
C2A4-C1A4-H1AB4	109.9
N1A4-C1A4-H1AB4	109.9
Co14-C1A4-H1AB4	78.1
H1AA4-C1A4-H1AB4	108.3
N2A4-C2A4-C1A4	110(3)
N2A4-C2A4-H2AA4	109.7
C1A4-C2A4-H2AA4	109.7
N2A4-C2A4-H2AB4	109.7
C1A4-C2A4-H2AB4	109.7
H2AA4-C2A4-H2AB4	108.2
N2A4-C3A4-C4A4	114(4)
N2A4-C3A4-Co14	50(3)
C4A4-C3A4-Co14	95(4)
N2A4-C3A4-H3AA4	108.8
C4A4-C3A4-H3AA4	108.8
Co14-C3A4-H3AA4	72.1
N2A4-C3A4-H3AB4	108.8
C4A4-C3A4-H3AB4	108.8
Co14-C3A4-H3AB4	154.1
H3AA4-C3A4-H3AB4	107.7
C5A4-C4A4-C3A4	115(4)
C5A4-C4A4-H4AA4	108.4
C3A4-C4A4-H4AA4	108.4
C5A4-C4A4-H4AB4	108.4
C3A4-C4A4-H4AB4	108.4
H4AA4-C4A4-H4AB4	107.5
N3A4-C5A4-C4A4	115(4)
N3A4-C5A4-H5AA4	108.6
C4A4-C5A4-H5AA4	108.6
N3A4-C5A4-H5AB4	108.6
C4A4-C5A4-H5AB4	108.6
H5AA4-C5A4-H5AB4	107.6
N3A4-C6A4-C7A4	109(4)
N3A4-C6A4-H6AA4	109.8
C7A4-C6A4-H6AA4	109.8
N3A4-C6A4-H6AB4	109.8
C7A4-C6A4-H6AB4	109.8
H6AA4-C6A4-H6AB4	108.3
N4A4-C7A4-C6A4	111(4)
N4A4-C7A4-H7AA4	109.5
C6A4-C7A4-H7AA4	109.5
N4A4-C7A4-H7AB4	109.5
C6A4-C7A4-H7AB4	109.5
H7AA4-C7A4-H7AB4	108.1
N4A4-C8A4-C9A4	114(4)
N4A4-C8A4-H8AA4	108.8
C9A4-C8A4-H8AA4	108.8
N4A4-C8A4-H8AB4	108.8
C9A4-C8A4-H8AB4	108.8

H8AA4-C8A4-H8AB4	107.7	F17-C17-F37	107.5(9)
C10A4-C9A4-C8A4	114(4)	F27-C17-S17	112.2(7)
C10A4-C9A4-H9AA4	108.7	F17-C17-S17	111.9(7)
C8A4-C9A4-H9AA4	108.7	F37-C17-S17	110.9(7)
C10A4-C9A4-H9AB4	108.7	O18-S18-O28	115.1(6)
C8A4-C9A4-H9AB4	108.7	O18-S18-O38	115.6(6)
H9AA4-C9A4-H9AB4	107.6	O28-S18-O38	114.8(6)
N1A4-C10A4-C9A4	115(4)	O18-S18-C18	104.6(5)
N1A4-C10A4-H10C4	108.5	O28-S18-C18	102.7(6)
C9A4-C10A4-H10C4	108.5	O38-S18-C18	101.4(6)
N1A4-C10A4-H10D4	108.5	F28-C18-F18	108.3(11)
C9A4-C10A4-H10D4	108.5	F28-C18-F38	106.4(11)
H10C4-C10A4-H10D4	107.5	F18-C18-F38	106.6(9)
N1A4-C11A4-H11D4	109.5	F28-C18-S18	111.9(8)
N1A4-C11A4-H11E4	109.5	F18-C18-S18	111.3(8)
H11D4-C11A4-H11E4	109.5	F38-C18-S18	112.1(9)
N1A4-C11A4-H11F4	109.5	O29-S19-O19	116.1(7)
H11D4-C11A4-H11F4	109.5	O29-S19-O39	113.8(7)
H11E4-C11A4-H11F4	109.5	O19-S19-O39	113.5(6)
N2A4-C12A4-H12D4	109.5	O29-S19-C19	103.1(6)
N2A4-C12A4-H12E4	109.5	O19-S19-C19	105.2(5)
H12D4-C12A4-H12E4	109.5	O39-S19-C19	103.1(6)
N2A4-C12A4-H12F4	109.5	F29-C19-F39	106.8(10)
H12D4-C12A4-H12F4	109.5	F29-C19-F19	109.7(11)
H12E4-C12A4-H12F4	109.5	F39-C19-F19	106.6(10)
N3A4-C13A4-H13D4	109.5	F29-C19-S19	112.2(8)
N3A4-C13A4-H13E4	109.5	F39-C19-S19	111.1(8)
H13D4-C13A4-H13E4	109.5	F19-C19-S19	110.2(8)
N3A4-C13A4-H13F4	109.5	O310-S110-O110	115.3(13)
H13D4-C13A4-H13F4	109.5	O310-S110-O210	115.1(14)
H13E4-C13A4-H13F4	109.5	O110-S110-O210	114.7(13)
N4A4-C14A4-H14D4	109.5	O310-S110-C110	102.7(11)
N4A4-C14A4-H14E4	109.5	O110-S110-C110	103.6(11)
H14D4-C14A4-H14E4	109.5	O210-S110-C110	102.9(10)
N4A4-C14A4-H14F4	109.5	F210-C110-F110	108.9(14)
H14D4-C14A4-H14F4	109.5	F210-C110-F310	106.8(13)
H14E4-C14A4-H14F4	109.5	F110-C110-F310	107.4(13)
C164-N54-Co14	168.7(10)	F210-C110-S110	111.8(12)
C164-C154-H15A4	109.5	F110-C110-S110	111.2(11)
C164-C154-H15B4	109.5	F310-C110-S110	110.6(11)
H15A4-C154-H15B4	109.5	O2A10-S1A10-O3A10	118(2)
C164-C154-H15C4	109.5	O2A10-S1A10-O1A10	114(2)
H15A4-C154-H15C4	109.5	O3A10-S1A10-O1A10	112(2)
H15B4-C154-H15C4	109.5	O2A10-S1A10-C1A10	105(2)
N54-C164-C154	176.5(15)	O3A10-S1A10-C1A10	104.5(19)
O15-S15-O35	115.3(5)	O1A10-S1A10-C1A10	101.4(18)
O15-S15-O25	113.9(5)	F1A10-C1A10-F3A10	113(3)
O35-S15-O25	115.4(5)	F1A10-C1A10-F2A10	107(3)
O15-S15-C15	102.4(5)	F3A10-C1A10-F2A10	101(3)
O35-S15-C15	103.4(5)	F1A10-C1A10-S1A10	116(2)
O25-S15-C15	104.2(5)	F3A10-C1A10-S1A10	111(2)
F25-C15-F15	108.4(8)	F2A10-C1A10-S1A10	107(2)
F25-C15-F35	106.8(8)	O311-S111-O111	116.1(5)
F15-C15-F35	108.3(8)	O311-S111-O211	114.4(5)
F25-C15-S15	112.0(7)	O111-S111-O211	114.4(5)
F15-C15-S15	111.0(7)	O311-S111-C111	102.5(5)
F35-C15-S15	110.1(7)	O111-S111-C111	103.6(5)
O36-S16-O26	114.4(6)	O211-S111-C111	103.3(5)
O36-S16-O16	112.5(7)	F311-C111-F111	107.3(9)
O26-S16-O16	116.7(9)	F311-C111-F211	107.6(9)
O36-S16-C16	104.8(5)	F111-C111-F211	107.5(8)
O26-S16-C16	103.2(6)	F311-C111-S111	111.4(7)
O16-S16-C16	103.3(6)	F111-C111-S111	110.5(7)
F16-C16-F26	107.2(12)	F211-C111-S111	112.2(7)
F16-C16-F36	105.6(10)	O212-S112-O112	116.5(6)
F26-C16-F36	106.1(11)	O212-S112-O312	115.0(6)
F16-C16-S16	112.8(9)	O112-S112-O312	114.4(5)
F26-C16-S16	112.0(8)	O212-S112-C112	102.9(6)
F36-C16-S16	112.6(8)	O112-S112-C112	102.6(5)
O27-S17-O37	114.5(6)	O312-S112-C112	102.6(5)
O27-S17-O17	114.8(6)	F312-C112-F112	109.0(9)
O37-S17-O17	115.3(5)	F312-C112-F212	106.6(8)
O27-S17-C17	102.9(5)	F112-C112-F212	105.4(8)
O37-S17-C17	103.6(5)	F312-C112-S112	113.4(7)
O17-S17-C17	103.4(5)	F112-C112-S112	111.1(7)
F27-C17-F17	107.0(8)	F212-C112-S112	110.9(7)
F27-C17-F37	107.1(8)		

Table S30. Anisotropic displacement parameters ($Å^2$x 10^3) for **4a-(OTf)₂**.
The anisotropic displacement factor exponent takes the form: $-2p^2[h^2 a^{*2}U^{11} + ... + 2 h k a^* b^* U^{12}]$

	U^{11}	U^{22}	U^{33}	U^{23}	U^{13}	U^{12}
Co11	19(1)	18(1)	24(1)	0(1)	0(1)	-2(1)
N11	32(5)	20(4)	42(5)	0(4)	-5(4)	7(4)
N21	29(5)	19(4)	44(5)	4(4)	-5(4)	6(3)
N31	19(4)	22(4)	35(5)	0(3)	2(4)	-5(3)
N41	20(4)	19(4)	44(5)	-2(4)	2(4)	-8(3)
C11	26(5)	23(5)	51(7)	-3(5)	-4(5)	1(4)
C21	28(6)	25(5)	50(7)	-1(5)	5(5)	1(4)
C31	21(6)	31(6)	49(7)	7(5)	-8(5)	11(4)
C41	31(5)	35(6)	42(7)	4(5)	-7(5)	6(5)
C51	42(6)	24(6)	31(6)	-2(5)	1(5)	7(5)
C61	28(5)	26(6)	34(6)	6(5)	5(4)	-7(4)
C71	23(5)	30(5)	41(6)	4(5)	-1(5)	-3(4)
C81	35(6)	30(5)	34(6)	2(4)	6(5)	4(5)
C91	28(5)	26(5)	48(7)	-13(5)	-4(5)	10(4)
C101	30(5)	25(6)	37(6)	-6(5)	-7(5)	-1(4)
C111	43(7)	14(5)	43(6)	4(5)	0(5)	-1(5)
C121	39(5)	25(4)	47(5)	-3(3)	5(4)	-3(3)
C131	39(5)	25(4)	47(5)	-3(3)	5(4)	-3(3)
C141	18(5)	28(6)	47(6)	1(5)	4(5)	3(4)
N51	30(5)	18(4)	36(5)	7(4)	5(4)	-5(4)
C151	52(8)	54(8)	33(6)	4(6)	-2(6)	-6(7)
C161	27(6)	25(6)	35(5)	-5(4)	2(4)	-10(5)
Co12	18(1)	20(1)	24(1)	-1(1)	0(1)	2(1)
N12	20(4)	22(4)	34(5)	5(3)	-4(4)	-3(3)
N22	12(4)	20(4)	38(5)	3(3)	-1(3)	5(3)
N32	28(4)	23(4)	24(4)	-3(3)	1(3)	-1(3)
N42	17(4)	27(4)	32(5)	5(3)	1(3)	-4(3)
C12	29(5)	16(5)	35(6)	0(4)	0(4)	2(4)
C22	18(5)	19(5)	46(7)	0(4)	-4(5)	-4(4)
C32	24(6)	30(6)	37(6)	6(5)	-2(5)	2(4)
C42	38(5)	10(4)	41(6)	2(4)	3(5)	-2(4)
C52	31(5)	28(6)	30(6)	0(5)	1(4)	-4(5)
C62	22(5)	21(5)	30(5)	2(4)	-1(4)	7(4)
C72	27(5)	23(5)	37(6)	2(4)	2(5)	2(4)
C82	7(4)	37(5)	39(6)	-4(5)	-5(4)	-4(4)
C92	16(5)	29(5)	40(6)	-4(5)	-2(4)	-4(4)
C102	22(5)	21(5)	39(6)	-5(5)	0(4)	-4(4)
C112	27(5)	23(5)	36(6)	6(4)	-1(5)	4(4)
C122	18(5)	35(6)	39(6)	-12(5)	-5(5)	6(4)
C132	32(6)	14(5)	47(6)	-8(4)	7(5)	-9(4)
C142	17(5)	14(5)	42(6)	4(4)	6(4)	-2(4)
N52	27(5)	23(5)	31(4)	-1(4)	-1(4)	-1(4)
C152	44(8)	77(10)	33(6)	14(7)	-1(6)	-4(7)
C162	18(5)	33(6)	35(5)	-1(4)	-4(4)	-6(4)
Co13	22(1)	17(1)	22(1)	1(1)	1(1)	-1(1)
N13	24(4)	23(4)	35(5)	-2(4)	3(4)	3(3)
N23	30(5)	24(4)	28(5)	4(4)	-1(4)	1(4)
N33	18(4)	22(4)	37(5)	-4(4)	5(4)	-6(3)
N43	23(4)	30(5)	42(5)	-2(4)	-4(4)	-20(4)
C13	27(5)	27(6)	34(6)	1(5)	1(4)	-4(4)
C23	15(5)	25(5)	46(7)	0(5)	0(4)	0(4)
C33	31(6)	33(5)	27(5)	1(4)	-1(5)	14(4)
C43	46(6)	27(5)	34(6)	-3(5)	5(5)	11(5)
C53	41(6)	18(5)	32(6)	-8(4)	-5(5)	7(4)
C63	21(5)	23(5)	54(7)	-1(5)	-5(5)	-6(4)
C73	23(5)	39(6)	42(7)	-8(5)	1(5)	-17(4)
C83	29(6)	27(6)	44(6)	-7(5)	-1(5)	-5(5)
C93	35(5)	17(5)	39(6)	-1(4)	-8(5)	3(4)
C103	36(5)	14(5)	31(6)	6(4)	0(4)	-1(4)
C113	39(7)	23(5)	42(6)	0(4)	4(5)	3(5)
C123	27(6)	27(6)	36(6)	7(5)	-3(5)	4(4)
C133	34(6)	21(5)	41(6)	1(5)	7(5)	-2(5)
C143	31(6)	40(7)	44(7)	-9(5)	4(5)	3(5)
N53	36(5)	34(5)	30(4)	-2(4)	11(4)	-8(4)
C153	77(12)	180(20)	25(6)	1(9)	-5(7)	-66(13)
C163	42(8)	58(9)	35(5)	-3(5)	2(5)	-24(6)
Co14	22(1)	16(1)	24(1)	-2(1)	0(1)	-2(1)
N14	33(5)	18(4)	43(6)	10(4)	-9(4)	-6(4)
N24	15(4)	18(4)	57(6)	1(4)	-2(4)	-4(3)
N34	31(5)	27(5)	48(6)	3(4)	-2(4)	-4(4)
N44	25(5)	23(5)	49(5)	-6(4)	4(4)	-1(4)
C14	32(5)	24(5)	54(7)	5(5)	-10(5)	-3(4)
C24	38(6)	23(6)	56(7)	9(5)	-4(5)	2(5)
C34	44(6)	21(6)	43(7)	4(5)	-2(5)	-5(5)
C44	36(6)	33(6)	36(7)	1(5)	5(5)	-11(5)
C54	30(7)	38(7)	52(7)	1(5)	5(6)	-12(5)
C64	33(6)	41(6)	61(8)	-9(6)	-4(6)	0(5)
C74	18(5)	24(6)	72(8)	-6(5)	6(5)	6(4)
C84	33(5)	30(6)	41(6)	-7(5)	1(5)	-4(5)
C94	45(6)	29(6)	33(6)	-5(5)	4(5)	-13(5)
C104	31(6)	40(6)	31(6)	6(5)	-12(5)	-14(5)
C114	17(5)	37(6)	42(6)	-1(5)	0(5)	-2(5)
C124	33(6)	24(6)	59(7)	0(5)	8(5)	4(5)
C134	23(6)	40(7)	59(7)	-6(5)	-12(5)	1(5)
C144	38(7)	21(6)	50(7)	-4(5)	-4(5)	3(5)
N1A4	29(12)	29(18)	54(13)	-1(12)	1(11)	5(14)
N2A4	24(15)	31(17)	52(15)	-4(14)	-4(11)	3(14)
N3A4	32(16)	28(15)	40(17)	-3(10)	-3(14)	0(13)
N4A4	27(18)	30(14)	42(18)	-3(14)	-12(16)	-5(14)
C1A4	30(14)	30(19)	60(30)	-1(18)	3(15)	4(14)

C2A4	28(17)	31(17)	50(30)	-5(18)	0(20)	0(17)
C3A4	30(20)	29(19)	56(14)	-3(17)	3(15)	1(15)
C4A4	30(20)	30(20)	60(30)	0(20)	0(20)	2(19)
C5A4	33(19)	30(20)	50(20)	-2(17)	1(18)	-1(19)
C6A4	35(17)	30(20)	40(20)	0(19)	-3(15)	-8(15)
C7A4	30(20)	30(20)	41(17)	1(16)	-9(17)	-7(19)
C8A4	32(5)	24(5)	54(7)	5(5)	-10(5)	-3(4)
C9A4	32(5)	24(5)	54(7)	5(5)	-10(5)	-3(4)
C10A4	38(5)	23(6)	56(7)	9(5)	-4(5)	2(5)
C11A4	33(6)	24(6)	59(7)	0(5)	8(5)	4(5)
C12A4	23(6)	40(7)	59(7)	-6(5)	-12(5)	1(5)
C13A4	38(7)	21(6)	50(7)	-4(5)	-4(5)	3(5)
C14A4	17(5)	37(6)	42(6)	-1(5)	0(5)	-2(5)
N54	29(5)	32(5)	33(5)	-6(4)	-8(4)	0(4)
C154	70(10)	68(10)	35(6)	12(7)	4(7)	22(8)
C164	36(7)	41(7)	35(5)	-7(5)	-7(5)	10(5)
S15	28(1)	21(1)	25(1)	-1(1)	3(1)	3(1)
F15	56(5)	36(4)	31(4)	0(3)	5(3)	5(3)
F25	19(3)	38(4)	48(4)	-18(3)	-3(3)	-1(3)
F35	22(3)	38(4)	42(4)	-15(3)	5(3)	6(3)
O15	29(4)	33(4)	37(5)	-3(4)	12(4)	9(3)
O25	60(5)	23(4)	29(4)	10(3)	0(4)	0(4)
O35	28(4)	29(4)	38(5)	-6(3)	-4(3)	-1(3)
C15	17(4)	34(5)	28(5)	-1(4)	-2(4)	1(4)
S16	30(2)	29(1)	34(2)	2(1)	5(1)	0(1)
F16	173(11)	43(5)	102(8)	24(5)	-70(8)	-44(6)
F26	41(5)	171(11)	92(7)	-57(8)	5(5)	-13(6)
F36	70(6)	69(6)	41(4)	-9(4)	-13(4)	-13(5)
O16	42(6)	125(10)	82(8)	-57(8)	23(6)	-8(6)
O26	124(11)	29(5)	80(8)	6(5)	-46(8)	9(5)
O36	46(5)	37(5)	40(5)	2(4)	2(4)	-8(4)
C16	56(7)	37(6)	40(6)	2(6)	9(6)	-4(5)
S17	34(2)	26(1)	29(2)	0(1)	-1(1)	3(1)
F17	64(5)	41(4)	32(4)	7(3)	1(4)	1(4)
F27	19(3)	46(4)	54(5)	-24(3)	3(3)	1(3)
F37	26(3)	51(4)	52(4)	-19(4)	-2(3)	-3(3)
O17	34(5)	41(5)	51(6)	-9(4)	-10(4)	1(4)
O27	94(8)	27(5)	46(6)	5(4)	15(5)	12(5)
O37	35(4)	37(5)	40(5)	-3(4)	4(4)	8(4)
C17	22(5)	30(5)	39(6)	0(4)	0(4)	-2(4)
S18	30(1)	25(1)	34(2)	2(1)	-4(1)	-2(1)
F18	78(6)	56(5)	46(4)	-7(4)	14(4)	9(4)
F28	48(5)	127(9)	86(7)	-44(6)	-16(5)	34(5)
F38	111(8)	34(4)	79(6)	-2(4)	28(6)	15(5)
O18	57(6)	42(5)	31(4)	1(4)	5(4)	7(4)
O28	28(5)	68(6)	55(6)	-5(5)	-15(4)	-7(4)
O38	63(7)	32(5)	70(7)	1(4)	17(5)	-17(4)
C18	44(6)	38(6)	45(6)	-3(6)	-6(5)	11(5)
S19	31(2)	26(1)	36(2)	2(1)	-5(1)	0(1)
F19	29(4)	140(9)	86(6)	-44(6)	-8(4)	22(5)
F29	109(8)	37(4)	93(7)	6(4)	27(6)	24(5)
F39	69(6)	81(6)	44(4)	-15(4)	9(4)	9(5)
O19	55(6)	42(5)	43(5)	7(4)	7(4)	17(4)
O29	36(5)	91(8)	85(8)	-39(7)	-28(5)	5(5)
O39	102(9)	36(5)	62(7)	8(5)	22(6)	-6(5)
C19	40(6)	40(6)	44(6)	-7(6)	0(5)	7(5)
S110	30(2)	22(1)	31(4)	2(2)	1(2)	0(1)
F110	34(6)	114(12)	80(8)	-23(8)	-2(6)	-34(6)
F210	73(13)	73(14)	34(6)	-8(8)	-21(7)	4(10)
F310	106(12)	33(6)	62(9)	8(6)	-19(9)	-38(7)
O110	71(11)	19(6)	63(11)	16(5)	-1(7)	0(6)
O210	38(12)	42(5)	34(5)	4(4)	-9(6)	-23(8)
O310	43(9)	79(14)	58(11)	-23(12)	16(8)	1(9)
C110	43(8)	43(7)	38(6)	-7(6)	-8(6)	-11(6)
S1A10	30(2)	22(1)	31(4)	2(2)	1(2)	0(1)
F1A10	34(6)	114(12)	80(8)	-23(8)	-2(6)	-34(6)
F2A10	100(20)	33(16)	64(12)	-2(13)	-39(13)	-21(15)
F3A10	100(17)	20(9)	80(17)	6(9)	17(13)	6(10)
O1A10	71(11)	19(6)	63(11)	16(5)	-1(7)	0(6)
O2A10	38(12)	42(5)	34(5)	4(4)	-9(6)	-23(8)
O3A10	37(13)	90(20)	37(12)	-7(16)	12(11)	-40(14)
C1A10	43(8)	43(7)	38(6)	-7(6)	-8(6)	-11(6)
S111	31(2)	25(1)	26(1)	-1(1)	-3(1)	-4(1)
F111	28(3)	41(4)	49(4)	-13(3)	0(3)	6(3)
F211	38(4)	41(4)	43(4)	-12(3)	-8(3)	-3(3)
F311	82(6)	33(4)	31(4)	3(3)	1(4)	4(4)
O111	49(5)	19(4)	48(5)	7(4)	-9(4)	-1(3)
O211	27(4)	35(4)	28(4)	-1(3)	0(3)	1(3)
O311	26(4)	30(4)	34(4)	-11(3)	-1(3)	-11(3)
C111	27(5)	21(5)	30(5)	-4(4)	1(4)	3(4)
S112	33(2)	30(2)	30(2)	0(1)	-3(1)	3(1)
F112	31(4)	55(5)	54(5)	-21(4)	7(3)	-1(3)
F212	27(3)	27(3)	53(4)	-10(3)	-6(3)	0(3)
F312	92(6)	32(4)	43(4)	7(3)	-20(4)	-14(4)
O112	38(5)	30(4)	52(6)	-9(4)	10(4)	3(4)
O212	86(8)	30(5)	46(6)	6(4)	-12(5)	-11(5)
O312	29(4)	28(4)	41(5)	-4(4)	-10(4)	-7(3)
C112	21(5)	27(5)	38(6)	3(4)	-3(4)	0(4)

Table S31. Hydrogen coordinates (x 10^4) and isotropic displacement parameters ($Å^2$x 10^3) for **4a-(OTf)$_2$**.

	x	y	z	U(eq)
H1A1	13109	-1501	4003	40
H1B1	12843	-2361	4178	40
H2A1	12668	-1254	4480	41
H2B1	11967	-957	4272	41
H3A1	12101	583	4111	40
H3B1	12915	41	3964	40
H4A1	12929	1787	3936	43
H4B1	13281	1818	4198	43
H5A1	14553	2046	3934	39
H5B1	14372	983	3835	39
H6A1	16251	1486	3993	35
H6B1	15840	517	3878	35
H7A1	17261	133	4082	37
H7B1	16832	609	4313	37
H8A1	17031	-1499	4016	40
H8B1	16090	-1054	3896	40
H9A1	16046	-2819	3950	41
H9B1	15961	-2629	4221	41
H10A1	14547	-2000	3898	37
H10B1	14458	-2986	4040	37
H11A1	14962	-2401	4500	50
H11B1	14050	-3035	4423	50
H11C1	13894	-2079	4574	50
H12A1	13338	958	4564	56
H12B1	12650	86	4640	56
H12C1	12243	918	4475	56
H13A1	15356	2322	4276	56
H13B1	15657	1469	4449	56
H13C1	14546	1778	4425	56
H14A1	16350	-1511	4526	47
H14B1	16914	-502	4539	47
H14C1	17345	-1375	4390	47
H15A1	14484	-622	5166	70
H15B1	15571	-230	5150	70
H15C1	15345	-1346	5101	70
H1A2	10911	3513	3998	32
H1B2	11162	2636	4169	32
H2A2	11328	3713	4480	33
H2B2	12031	4051	4276	33
H3A2	11122	5053	3978	37
H3B2	11939	5561	4130	37
H4A2	10765	6824	4220	36
H4B2	11136	6815	3959	36
H5A2	9713	5982	3851	36
H5B2	9509	7043	3946	36
H6A2	8183	5567	3881	29
H6B2	7783	6525	4004	29
H7A2	7187	5615	4314	35
H7B2	6760	5170	4080	35
H8A2	7940	3991	3889	33
H8B2	6989	3542	4002	33
H9A2	8025	2388	4200	34
H9B2	7942	2239	3928	34
H10A2	9532	2028	4020	33
H10B2	9464	3029	3884	33
H11A2	9011	2426	4459	43
H11B2	9945	2930	4569	43
H11C2	10062	1982	4414	43
H12A2	10941	6136	4524	46
H12B2	11869	5453	4548	46
H12C2	10870	5150	4667	46
H13A2	8615	7288	4295	47
H13B2	9484	6781	4431	47
H13C2	8400	6401	4465	47
H14A2	7082	4434	4535	37
H14B2	7617	3416	4505	37
H14C2	6631	3626	4370	37
H15A2	8305	4630	5143	77
H15B2	9422	4352	5172	77
H15C2	8655	3543	5098	77
H1A3	3162	4540	6093	35
H1B3	2745	3573	5977	35
H2A3	2214	4451	5655	34
H2B3	1765	4928	5883	34
H3A3	2990	6118	6080	37
H3B3	2051	6567	5962	37
H4A3	3005	7844	6033	43
H4B3	3102	7669	5761	43
H5A3	4608	8020	5947	36
H5B3	4516	7024	6085	36
H6A3	5935	6537	5975	39
H6B3	6207	7388	5799	39
H7A3	6396	6254	5495	42
H7B3	7083	5946	5705	42
H8A3	6108	4974	6011	40
H8B3	6945	4451	5868	40
H9A3	5762	3189	5772	37
H9B3	6108	3203	6035	37
H10A3	4465	3007	6042	32
H10B3	4677	4073	6133	32
H11A3	3331	3544	5533	52
H11B3	3757	2705	5693	52

H11C3	4470	3339	5537	52
H12A3	2574	6784	5501	45
H12B3	1626	6223	5585	45
H12C3	2360	5712	5410	45
H13A3	5258	7202	5417	48
H13B3	4878	8105	5563	48
H13C3	4124	7338	5461	48
H14A3	6244	4945	5323	57
H14B3	5797	3975	5430	57
H14C3	6896	4262	5483	57
H15A3	4576	5439	4806	143
H15B3	3879	6334	4860	143
H15C3	3437	5271	4832	143
H1A4	11831	9457	5667	44
H1B4	12224	9895	5905	44
H2A4	10830	9438	6095	47
H2B4	11257	8509	5965	47
H3A4	9374	8991	6131	43
H3B4	9559	7942	6026	43
H4A4	7937	8162	6032	42
H4B4	8258	8197	5768	42
H5A4	7083	9412	5881	48
H5B4	7922	9919	6027	48
H6A4	7590	11316	5518	54
H6B4	6912	10983	5728	54
H7A4	7803	12364	5833	45
H7B4	8090	11470	5994	45
H8A4	9501	11923	6107	42
H8B4	9389	12959	5988	42
H9A4	10897	12648	5786	43
H9B4	11005	12809	6058	43
H10A4	11966	11536	5982	41
H10B4	11030	11074	6099	41
H11A4	12392	11160	5595	48
H11B4	11480	11820	5531	48
H11C4	11586	10764	5421	48
H12A4	9479	8263	5548	58
H12B4	10392	7761	5668	58
H12C4	10544	8673	5502	58
H13A4	7974	9919	5330	61
H13B4	7053	9516	5466	61
H13C4	8039	8911	5465	61
H14A4	9918	12523	5513	54
H14B4	8967	13102	5588	54
H14C4	8877	12158	5430	54
H1AA4	9346	7978	5609	46
H1AB4	9253	8764	5811	46
H2AA4	8495	9004	5356	45
H2AB4	7850	8795	5580	45
H3AA4	8343	9950	5918	45
H3AB4	7298	10006	5801	45
H4AA4	7622	11736	5746	47
H4AB4	7358	11389	6001	47
H5AA4	8621	12464	6027	46
H5AB4	9014	11423	6100	46
H6AA4	10325	12255	6087	41
H6AB4	10777	12724	5859	41
H7AA4	11807	11470	5977	42
H7AB4	10922	10724	5988	42
H8AA4	12663	10577	5770	44
H8AB4	12560	10568	5496	44
H9AA4	12088	9050	5491	44
H9AB4	12483	8999	5750	44
H10C4	11000	8393	5789	47
H10D4	10843	9491	5865	47
H11D4	10643	8002	5368	58
H11E4	11137	8989	5286	58
H11F4	10035	8767	5221	58
H12D4	7550	11263	5448	61
H12E4	7178	10210	5376	61
H12F4	8097	10673	5249	61
H13D4	9306	12447	5474	54
H13E4	9872	13185	5638	54
H13F4	8739	13005	5674	54
H14D4	11718	11797	5372	48
H14E4	11944	12361	5606	48
H14F4	10855	12280	5515	48
H15A4	9610	10566	4804	86
H15B4	10739	10614	4864	86
H15C4	10086	11565	4885	86

[(TMC)Co^{II}(MeCN)]($B_{Ar}F$)$_2$, **4a-(B_{Ar}F)$_2$**

The structure of **4a-(B_{Ar}F)$_2$** was solved in the monoclinic space group *C*2/*c* and the asymmetric unit contains half of the molecule and one additional acetonitrile molecule. Three of the CF_3 groups were found to be disordered. The disorder was modelled over two positions each and refined with the help of same distance restraints (1,2 and 1,3), similarity restraints on the anisotropic displacement parameters of fluorine atoms and with setting some of the disordered atoms to the same anisotropic displacement parameters. The disorder ratios were refined and converged to 0.76(1), 0.872(7) and 0.72(4).

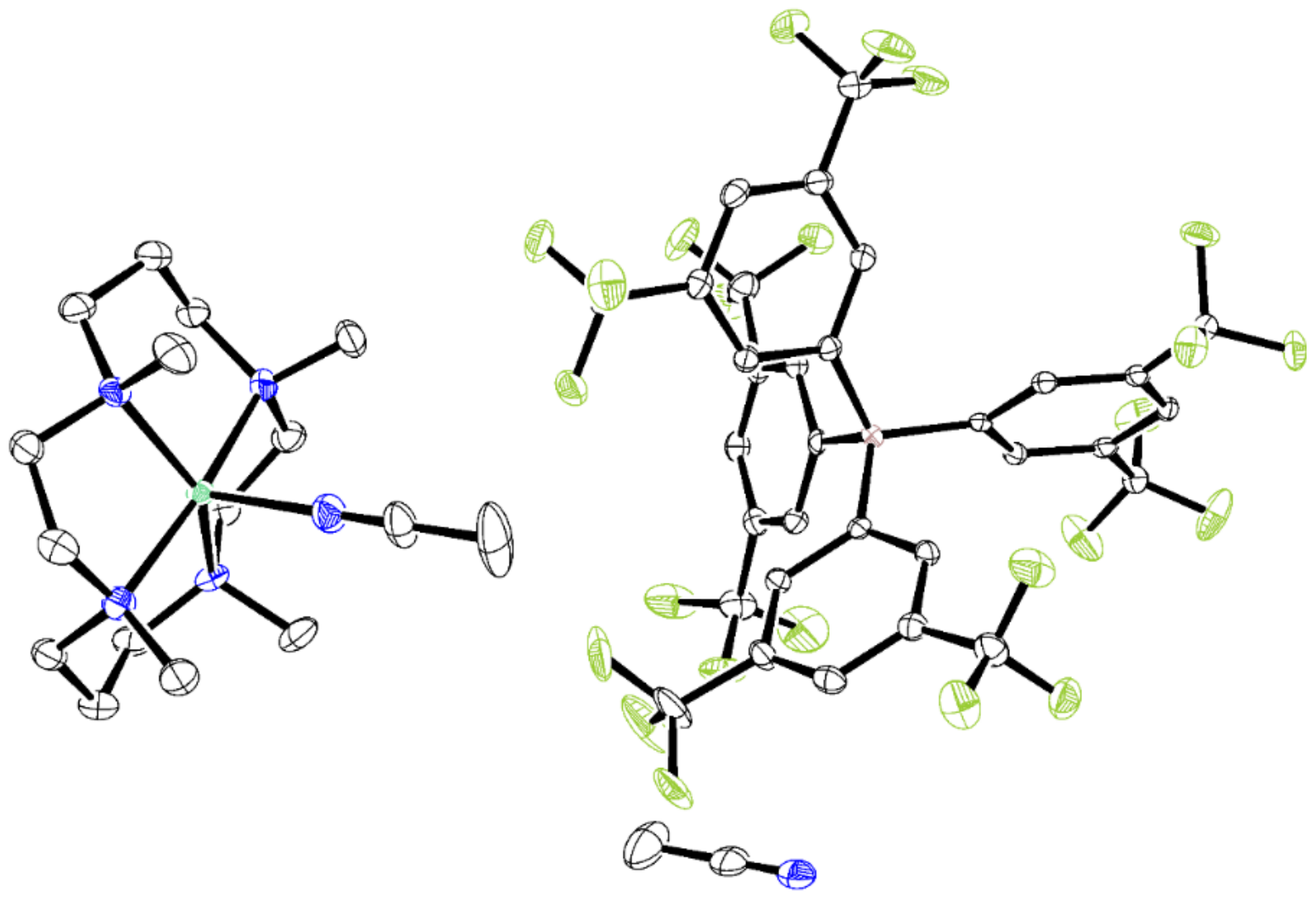

Figure S7. Molecular structure of **4a-(B_{Ar}F)$_2$** (only one anion is shown together with one solvent molecule (acetonitrile).

Table S32. Crystal data and structure refinement for **4a-(B_{Ar}F)$_2$**.

CCDC No	2062710	
Empirical formula	$C_{82}H_{62}B_2CoF_{48}N_6$	
Formula weight	2123.92	
Temperature	101(2) K	
Wavelength	0.71073 Å	
Crystal system	Monoclinic	
Space group	*C*2/*c*	
Unit cell dimensions	a = 21.4576(8) Å	α = 90°
	b = 18.7507(7) Å	β = 95.4007(13)°
	c = 21.3042(8) Å	γ = 90°
Volume	8533.6(6) $Å^3$	
Z	4	
Density (calculated)	1.653 Mg/m^3	
Absorption coefficient	0.356 mm^{-1}	
F(000)	4260	
Crystal size	0.519 x 0.104 x 0.061 mm^3	
Theta range for data collection	2.172 to 26.732°.	
Index ranges	-27 ≤ h ≤ 27, -23 ≤ k ≤ 23, -26 ≤ l ≤ 26	
Reflections collected	246045	
Independent reflections	9052 [R(int) = 0.0655]	
Completeness to theta = 25.242°	99.9 %	
Absorption correction	Semi-empirical from equivalents	
Refinement method	Full-matrix least-squares on F^2	
Data / restraints / parameters	9052 / 561 / 683	
Goodness-of-fit on F^2	1.027	
Final R indices [I>2σ(I)]	R1 = 0.0430, wR2 = 0.0971	
R indices (all data)	R1 = 0.0551, wR2 = 0.1030	
Extinction coefficient	0.00030(4)	
Largest diff. peak and hole	0.546 and -0.490 e$Å^{-3}$	

Table S33. Atomic coordinates (x 10^4) and equivalent isotropic displacement parameters ($Å^2$x 10^3) for **4a-($B_{Ar}F$)$_2$**.
U(eq) is defined as one third of the trace of the orthogonalized U^{ij} tensor.

	x	y	z	U(eq)
Co(1)	5000	1000(1)	2500	15(1)
N(1)	4054(1)	685(1)	2312(1)	19(1)
N(2)	4802(1)	934(1)	3491(1)	21(1)
C(1)	3672(1)	1332(1)	2164(1)	30(1)
C(2)	3876(1)	358(1)	2906(1)	24(1)
C(3)	4107(1)	807(1)	3469(1)	25(1)
C(4)	4940(1)	1580(1)	3877(1)	27(1)
C(5)	5125(1)	312(1)	3808(1)	23(1)
C(6)	5832(1)	340(1)	3825(1)	26(1)
C(7)	6088(1)	157(1)	3203(1)	25(1)
N(11)	5000	2108(2)	2500	28(1)
C(11)	5000	2710(2)	2500	34(1)
C(12)	5000	3489(2)	2500	67(2)
N(21)	0	4071(2)	2500	34(1)
C(21)	0	3457(2)	2500	35(1)
C(22)	0	2684(2)	2500	68(2)
F(31)	3564(1)	7165(1)	6374(1)	31(1)
F(32)	3648(1)	7436(1)	5412(1)	24(1)
F(33)	2934(1)	7930(1)	5900(1)	24(1)
F(34)	862(1)	6882(1)	5606(1)	38(1)
F(35)	1018(1)	6050(1)	6272(1)	38(1)
F(36)	736(1)	5797(1)	5314(1)	43(1)
F(41)	4561(1)	3273(1)	3936(1)	30(1)
F(42)	5087(1)	2853(1)	4758(1)	31(1)
F(43)	5418(1)	3772(1)	4291(1)	32(1)
F(44)	4972(2)	4041(1)	6827(1)	56(1)
F(45)	5256(1)	5074(2)	6541(1)	42(1)
F(46)	4366(2)	4949(2)	6897(2)	38(1)
F(44A)	4621(11)	4081(7)	6919(6)	56(1)
F(45A)	5331(5)	4752(12)	6615(8)	42(1)
F(46A)	4412(14)	5136(13)	6804(14)	53(9)
F(51)	2318(5)	3368(4)	6717(3)	27(1)
F(52)	1750(3)	2470(5)	6392(3)	37(1)
F(53)	2747(4)	2463(6)	6311(5)	41(2)
F(51A)	2199(10)	3271(10)	6738(7)	27(1)
F(52A)	1884(15)	2285(14)	6297(13)	60(5)
F(53A)	2828(9)	2584(19)	6331(11)	39(5)
F(54)	471(1)	3569(1)	4302(1)	58(1)
F(55)	828(1)	2514(1)	4194(1)	50(1)
F(56)	1073(1)	3351(1)	3586(1)	35(1)
F(61)	3391(3)	3650(2)	2528(1)	56(1)
F(62)	2403(3)	3672(3)	2488(2)	62(1)
F(63)	2866(2)	4345(2)	1858(1)	42(1)
F(61A)	3073(7)	4241(5)	1876(5)	56(1)
F(62A)	3144(7)	3492(4)	2581(5)	62(1)
F(63A)	2253(5)	3926(7)	2338(8)	51(3)
F(64)	3617(1)	6656(1)	2504(1)	33(1)
F(65)	2874(1)	7070(1)	3007(1)	30(1)
F(66)	3780(1)	6899(1)	3494(1)	34(1)
C(31)	2635(1)	5531(1)	5151(1)	11(1)
C(32)	3017(1)	6114(1)	5334(1)	13(1)
C(33)	2788(1)	6716(1)	5618(1)	13(1)
C(34)	2164(1)	6765(1)	5731(1)	13(1)
C(35)	1776(1)	6196(1)	5553(1)	13(1)
C(36)	2007(1)	5592(1)	5275(1)	13(1)
C(37)	3227(1)	7307(1)	5823(1)	16(1)
C(38)	1103(1)	6234(1)	5681(1)	17(1)
C(41)	3610(1)	4610(1)	5050(1)	13(1)
C(42)	3970(1)	4195(1)	4674(1)	14(1)
C(43)	4553(1)	3929(1)	4889(1)	16(1)
C(44)	4815(1)	4076(1)	5495(1)	18(1)
C(45)	4472(1)	4486(1)	5877(1)	18(1)
C(46)	3880(1)	4744(1)	5663(1)	16(1)
C(47)	4904(1)	3464(1)	4475(1)	21(1)
C(48)	4752(1)	4633(1)	6534(1)	27(1)
C(51)	2442(1)	4152(1)	4907(1)	13(1)
C(52)	2527(1)	3768(1)	5472(1)	16(1)
C(53)	2128(1)	3222(1)	5621(1)	18(1)
C(54)	1627(1)	3016(1)	5200(1)	18(1)
C(55)	1532(1)	3384(1)	4637(1)	17(1)
C(56)	1926(1)	3940(1)	4497(1)	16(1)
C(57)	2243(1)	2864(1)	6250(1)	25(1)
C(58)	981(1)	3201(1)	4183(1)	26(1)
C(61)	2913(1)	5031(1)	4028(1)	12(1)
C(62)	2854(1)	4516(1)	3546(1)	15(1)
C(63)	2946(1)	4680(1)	2923(1)	19(1)
C(64)	3111(1)	5358(1)	2750(1)	19(1)
C(65)	3176(1)	5874(1)	3215(1)	17(1)
C(66)	3070(1)	5716(1)	3834(1)	15(1)
C(67)	2884(2)	4095(1)	2442(1)	42(1)
C(68)	3362(1)	6619(1)	3051(1)	22(1)
B(31)	2893(1)	4835(1)	4782(1)	12(1)

Table S34. Bond lengths [Å] and angles [°] for **4a-(B_{ArF})$_2$**.

Co(1)-N(11)	2.078(3)	C(41)-B(31)	1.645(3)
Co(1)-N(1)	2.1159(17)	C(42)-C(43)	1.383(3)
Co(1)-N(1)#1	2.1159(17)	C(42)-H(42)	0.9500
Co(1)-N(2)#1	2.1956(18)	C(43)-C(44)	1.385(3)
Co(1)-N(2)	2.1956(18)	C(43)-C(47)	1.493(3)
N(1)-C(1)	1.482(3)	C(44)-C(45)	1.382(3)
N(1)-C(2)	1.487(3)	C(44)-H(44)	0.9500
N(1)-C(7)#1	1.488(3)	C(45)-C(46)	1.396(3)
N(2)-C(4)	1.479(3)	C(45)-C(48)	1.495(3)
N(2)-C(5)	1.487(3)	C(46)-H(46)	0.9500
N(2)-C(3)	1.505(3)	C(51)-C(52)	1.399(3)
C(1)-H(1A)	0.9800	C(51)-C(56)	1.402(3)
C(1)-H(1B)	0.9800	C(51)-B(31)	1.642(3)
C(1)-H(1C)	0.9800	C(52)-C(53)	1.391(3)
C(2)-C(3)	1.509(3)	C(52)-H(52)	0.9500
C(2)-H(2A)	0.9900	C(53)-C(54)	1.387(3)
C(2)-H(2AB)	0.9900	C(53)-C(57)	1.499(3)
C(3)-H(3A)	0.9900	C(54)-C(55)	1.384(3)
C(3)-H(3AB)	0.9900	C(54)-H(54)	0.9500
C(4)-H(4A)	0.9800	C(55)-C(56)	1.392(3)
C(4)-H(4B)	0.9800	C(55)-C(58)	1.496(3)
C(4)-H(4C)	0.9800	C(56)-H(56)	0.9500
C(5)-C(6)	1.516(3)	C(61)-C(66)	1.400(3)
C(5)-H(5A)	0.9900	C(61)-C(62)	1.406(3)
C(5)-H(5AB)	0.9900	C(61)-B(31)	1.653(3)
C(6)-C(7)	1.519(3)	C(62)-C(63)	1.394(3)
C(6)-H(6A)	0.9900	C(62)-H(62)	0.9500
C(6)-H(6AB)	0.9900	C(63)-C(64)	1.380(3)
C(7)-H(7A)	0.9900	C(63)-C(67)	1.499(3)
C(7)-H(7AB)	0.9900	C(64)-C(65)	1.383(3)
N(11)-C(11)	1.128(4)	C(64)-H(64)	0.9500
C(11)-C(12)	1.461(5)	C(65)-C(66)	1.392(3)
C(12)-H(12A)	0.9800	C(65)-C(68)	1.501(3)
C(12)-H(12B)	0.9800	C(66)-H(66)	0.9500
C(12)-H(12C)	0.9800		
C(12)-H(12A)#1	0.9800	N(11)-Co(1)-N(1)	106.20(5)
C(12)-H(12B)#1	0.9800	N(11)-Co(1)-N(1)#1	106.20(5)
C(12)-H(12C)#1	0.9800	N(1)-Co(1)-N(1)#1	147.61(10)
N(21)-C(21)	1.150(5)	N(11)-Co(1)-N(2)#1	93.24(5)
C(21)-C(22)	1.450(6)	N(1)-Co(1)-N(2)#1	94.12(7)
C(22)-H(22A)	0.9800	N(1)#1-Co(1)-N(2)#1	84.06(7)
C(22)-H(22B)	0.9800	N(11)-Co(1)-N(2)	93.24(5)
C(22)-H(22C)	0.9800	N(1)-Co(1)-N(2)	84.06(7)
C(22)-H(22A)#2	0.9800	N(1)#1-Co(1)-N(2)	94.13(7)
C(22)-H(22B)#2	0.9800	N(2)#1-Co(1)-N(2)	173.52(9)
C(22)-H(22C)#2	0.9799	C(1)-N(1)-C(2)	109.70(18)
F(31)-C(37)	1.347(2)	C(1)-N(1)-C(7)#1	108.51(17)
F(32)-C(37)	1.339(2)	C(2)-N(1)-C(7)#1	107.61(17)
F(33)-C(37)	1.343(2)	C(1)-N(1)-Co(1)	108.28(14)
F(34)-C(38)	1.324(2)	C(2)-N(1)-Co(1)	105.78(12)
F(35)-C(38)	1.333(2)	C(7)#1-N(1)-Co(1)	116.81(13)
F(36)-C(38)	1.337(2)	C(4)-N(2)-C(5)	109.22(17)
F(41)-C(47)	1.353(2)	C(4)-N(2)-C(3)	107.04(17)
F(42)-C(47)	1.336(3)	C(5)-N(2)-C(3)	107.93(17)
F(43)-C(47)	1.337(3)	C(4)-N(2)-Co(1)	116.18(14)
F(44)-C(48)	1.337(3)	C(5)-N(2)-Co(1)	110.93(13)
F(45)-C(48)	1.360(3)	C(3)-N(2)-Co(1)	105.10(13)
F(46)-C(48)	1.324(4)	N(1)-C(1)-H(1A)	109.5
F(44A)-C(48)	1.366(11)	N(1)-C(1)-H(1B)	109.5
F(45A)-C(48)	1.260(10)	H(1A)-C(1)-H(1B)	109.5
F(46A)-C(48)	1.354(12)	N(1)-C(1)-H(1C)	109.5
F(51)-C(57)	1.371(6)	H(1A)-C(1)-H(1C)	109.5
F(52)-C(57)	1.348(5)	H(1B)-C(1)-H(1C)	109.5
F(53)-C(57)	1.314(6)	N(1)-C(2)-C(3)	110.83(18)
F(51A)-C(57)	1.300(11)	N(1)-C(2)-H(2A)	109.5
F(52A)-C(57)	1.341(11)	C(3)-C(2)-H(2A)	109.5
F(53A)-C(57)	1.357(12)	N(1)-C(2)-H(2AB)	109.5
F(54)-C(58)	1.339(3)	C(3)-C(2)-H(2AB)	109.5
F(55)-C(58)	1.330(3)	H(2A)-C(2)-H(2AB)	108.1
F(56)-C(58)	1.336(3)	N(2)-C(3)-C(2)	111.24(18)
F(61)-C(67)	1.370(4)	N(2)-C(3)-H(3A)	109.4
F(62)-C(67)	1.314(5)	C(2)-C(3)-H(3A)	109.4
F(63)-C(67)	1.325(3)	N(2)-C(3)-H(3AB)	109.4
F(61A)-C(67)	1.334(9)	C(2)-C(3)-H(3AB)	109.4
F(62A)-C(67)	1.284(9)	H(3A)-C(3)-H(3AB)	108.0
F(63A)-C(67)	1.388(9)	N(2)-C(4)-H(4A)	109.5
F(64)-C(68)	1.335(3)	N(2)-C(4)-H(4B)	109.5
F(65)-C(68)	1.342(3)	H(4A)-C(4)-H(4B)	109.5
F(66)-C(68)	1.346(3)	N(2)-C(4)-H(4C)	109.5
C(31)-C(32)	1.399(3)	H(4A)-C(4)-H(4C)	109.5
C(31)-C(36)	1.402(3)	H(4B)-C(4)-H(4C)	109.5
C(31)-B(31)	1.646(3)	N(2)-C(5)-C(6)	113.98(19)
C(32)-C(33)	1.392(3)	N(2)-C(5)-H(5A)	108.8
C(32)-H(32)	0.9500	C(6)-C(5)-H(5A)	108.8
C(33)-C(34)	1.384(3)	N(2)-C(5)-H(5AB)	108.8
C(33)-C(37)	1.494(3)	C(6)-C(5)-H(5AB)	108.8
C(34)-C(35)	1.386(3)	H(5A)-C(5)-H(5AB)	107.7
C(34)-H(34)	0.9500	C(5)-C(6)-C(7)	114.50(18)
C(35)-C(36)	1.390(3)	C(5)-C(6)-H(6A)	108.6
C(35)-C(38)	1.496(3)	C(7)-C(6)-H(6A)	108.6
C(36)-H(36)	0.9500	C(5)-C(6)-H(6AB)	108.6
C(41)-C(46)	1.399(3)	C(7)-C(6)-H(6AB)	108.6
C(41)-C(42)	1.401(3)	H(6A)-C(6)-H(6AB)	107.6

N(1)#1-C(7)-C(6)	115.30(19)	C(46)-C(45)-C(48)	121.05(19)
N(1)#1-C(7)-H(7A)	108.5	C(45)-C(46)-C(41)	121.87(19)
C(6)-C(7)-H(7A)	108.5	C(45)-C(46)-H(46)	119.1
N(1)#1-C(7)-H(7AB)	108.5	C(41)-C(46)-H(46)	119.1
C(6)-C(7)-H(7AB)	108.5	F(42)-C(47)-F(43)	106.66(17)
H(7A)-C(7)-H(7AB)	107.5	F(42)-C(47)-F(41)	105.62(17)
C(11)-N(11)-Co(1)	180.0	F(43)-C(47)-F(41)	105.24(18)
N(11)-C(11)-C(12)	180.0	F(42)-C(47)-C(43)	112.17(18)
C(11)-C(12)-H(12A)	109.5	F(43)-C(47)-C(43)	113.46(18)
C(11)-C(12)-H(12B)	109.5	F(41)-C(47)-C(43)	113.06(17)
H(12A)-C(12)-H(12B)	109.5	F(46)-C(48)-F(44)	108.1(3)
C(11)-C(12)-H(12C)	109.5	F(45A)-C(48)-F(46A)	112.5(15)
H(12A)-C(12)-H(12C)	109.5	F(46)-C(48)-F(45)	105.1(3)
H(12B)-C(12)-H(12C)	109.5	F(44)-C(48)-F(45)	104.7(2)
C(11)-C(12)-H(12A)#1	109.474(2)	F(45A)-C(48)-F(44A)	108.0(9)
H(12A)-C(12)-H(12A)#1	141.1	F(46A)-C(48)-F(44A)	97.1(13)
H(12B)-C(12)-H(12A)#1	56.3	F(45A)-C(48)-C(45)	117.8(8)
H(12C)-C(12)-H(12A)#1	56.2	F(46)-C(48)-C(45)	114.3(3)
C(11)-C(12)-H(12B)#1	109.475(1)	F(44)-C(48)-C(45)	112.1(2)
H(12A)-C(12)-H(12B)#1	56.3	F(46A)-C(48)-C(45)	110.0(14)
H(12B)-C(12)-H(12B)#1	141.1	F(45)-C(48)-C(45)	111.8(2)
H(12C)-C(12)-H(12B)#1	56.2	F(44A)-C(48)-C(45)	109.5(7)
H(12A)#1-C(12)-H(12B)#1	109.5	C(52)-C(51)-C(56)	115.02(18)
C(11)-C(12)-H(12C)#1	109.474(19)	C(52)-C(51)-B(31)	120.59(17)
H(12A)-C(12)-H(12C)#1	56.2	C(56)-C(51)-B(31)	124.23(17)
H(12B)-C(12)-H(12C)#1	56.3	C(53)-C(52)-C(51)	122.66(19)
H(12C)-C(12)-H(12C)#1	141.1	C(53)-C(52)-H(52)	118.7
H(12A)#1-C(12)-H(12C)#1	109.5	C(51)-C(52)-H(52)	118.7
H(12B)#1-C(12)-H(12C)#1	109.5	C(54)-C(53)-C(52)	121.00(19)
N(21)-C(21)-C(22)	180.0	C(54)-C(53)-C(57)	120.35(19)
C(21)-C(22)-H(22A)	109.5	C(52)-C(53)-C(57)	118.64(19)
C(21)-C(22)-H(22B)	109.5	C(55)-C(54)-C(53)	117.60(19)
H(22A)-C(22)-H(22B)	109.5	C(55)-C(54)-H(54)	121.2
C(21)-C(22)-H(22C)	109.5	C(53)-C(54)-H(54)	121.2
H(22A)-C(22)-H(22C)	109.5	C(54)-C(55)-C(56)	121.09(19)
H(22B)-C(22)-H(22C)	109.5	C(54)-C(55)-C(58)	119.30(19)
C(21)-C(22)-H(22A)#2	109.469(15)	C(56)-C(55)-C(58)	119.56(19)
H(22A)-C(22)-H(22A)#2	141.1	C(55)-C(56)-C(51)	122.60(19)
H(22B)-C(22)-H(22A)#2	56.2	C(55)-C(56)-H(56)	118.7
H(22C)-C(22)-H(22A)#2	56.3	C(51)-C(56)-H(56)	118.7
C(21)-C(22)-H(22B)#2	109.47(2)	F(51A)-C(57)-F(52A)	109.2(14)
H(22A)-C(22)-H(22B)#2	56.3	F(53)-C(57)-F(52)	108.7(6)
H(22B)-C(22)-H(22B)#2	141.1	F(51A)-C(57)-F(53A)	105.2(13)
H(22C)-C(22)-H(22B)#2	56.3	F(52A)-C(57)-F(53A)	102.0(14)
H(22A)#2-C(22)-H(22B)#2	109.5	F(53)-C(57)-F(51)	106.4(6)
C(21)-C(22)-H(22C)#2	109.469(2)	F(52)-C(57)-F(51)	104.9(4)
H(22A)-C(22)-H(22C)#2	56.3	F(51A)-C(57)-C(53)	115.7(10)
H(22B)-C(22)-H(22C)#2	56.2	F(53)-C(57)-C(53)	114.2(5)
H(22C)-C(22)-H(22C)#2	141.1	F(52A)-C(57)-C(53)	112.5(9)
H(22A)#2-C(22)-H(22C)#2	109.5	F(52)-C(57)-C(53)	112.3(4)
H(22B)#2-C(22)-H(22C)#2	109.5	F(53A)-C(57)-C(53)	111.1(11)
C(32)-C(31)-C(36)	115.57(17)	F(51)-C(57)-C(53)	109.8(4)
C(32)-C(31)-B(31)	122.56(17)	F(55)-C(58)-F(56)	106.36(19)
C(36)-C(31)-B(31)	121.81(17)	F(55)-C(58)-F(54)	106.8(2)
C(33)-C(32)-C(31)	122.22(18)	F(56)-C(58)-F(54)	105.6(2)
C(33)-C(32)-H(32)	118.9	F(55)-C(58)-C(55)	113.13(19)
C(31)-C(32)-H(32)	118.9	F(56)-C(58)-C(55)	112.82(18)
C(34)-C(33)-C(32)	121.07(18)	F(54)-C(58)-C(55)	111.6(2)
C(34)-C(33)-C(37)	119.54(17)	C(66)-C(61)-C(62)	115.00(17)
C(32)-C(33)-C(37)	119.36(18)	C(66)-C(61)-B(31)	121.31(17)
C(33)-C(34)-C(35)	117.89(18)	C(62)-C(61)-B(31)	123.23(17)
C(33)-C(34)-H(34)	121.1	C(63)-C(62)-C(61)	122.20(19)
C(35)-C(34)-H(34)	121.1	C(63)-C(62)-H(62)	118.9
C(34)-C(35)-C(36)	120.94(18)	C(61)-C(62)-H(62)	118.9
C(34)-C(35)-C(38)	118.63(17)	C(64)-C(63)-C(62)	121.37(19)
C(36)-C(35)-C(38)	120.41(18)	C(64)-C(63)-C(67)	119.89(19)
C(35)-C(36)-C(31)	122.31(18)	C(62)-C(63)-C(67)	118.70(19)
C(35)-C(36)-H(36)	118.8	C(63)-C(64)-C(65)	117.71(19)
C(31)-C(36)-H(36)	118.8	C(63)-C(64)-H(64)	121.1
F(32)-C(37)-F(33)	106.02(16)	C(65)-C(64)-H(64)	121.1
F(32)-C(37)-F(31)	105.35(16)	C(64)-C(65)-C(66)	120.99(19)
F(33)-C(37)-F(31)	106.50(16)	C(64)-C(65)-C(68)	119.78(19)
F(32)-C(37)-C(33)	112.91(16)	C(66)-C(65)-C(68)	119.23(18)
F(33)-C(37)-C(33)	113.11(17)	C(65)-C(66)-C(61)	122.70(18)
F(31)-C(37)-C(33)	112.36(16)	C(65)-C(66)-H(66)	118.6
F(34)-C(38)-F(35)	105.39(17)	C(61)-C(66)-H(66)	118.6
F(34)-C(38)-F(36)	106.82(18)	F(62)-C(67)-F(63)	109.2(3)
F(35)-C(38)-F(36)	105.54(17)	F(62A)-C(67)-F(61A)	103.2(6)
F(34)-C(38)-C(35)	113.29(17)	F(62)-C(67)-F(61)	103.9(3)
F(35)-C(38)-C(35)	112.34(17)	F(63)-C(67)-F(61)	107.2(3)
F(36)-C(38)-C(35)	112.83(17)	F(62A)-C(67)-F(63A)	103.3(6)
C(46)-C(41)-C(42)	115.51(18)	F(61A)-C(67)-F(63A)	106.1(8)
C(46)-C(41)-B(31)	124.74(17)	F(62A)-C(67)-C(63)	118.6(5)
C(42)-C(41)-B(31)	119.46(17)	F(62)-C(67)-C(63)	114.2(3)
C(43)-C(42)-C(41)	122.69(18)	F(63)-C(67)-C(63)	112.2(2)
C(43)-C(42)-H(42)	118.7	F(61A)-C(67)-C(63)	117.0(5)
C(41)-C(42)-H(42)	118.7	F(61)-C(67)-C(63)	109.7(3)
C(42)-C(43)-C(44)	120.86(19)	F(63A)-C(67)-C(63)	107.3(6)
C(42)-C(43)-C(47)	120.31(18)	F(64)-C(68)-F(65)	106.59(17)
C(44)-C(43)-C(47)	118.82(18)	F(64)-C(68)-F(66)	106.86(18)
C(45)-C(44)-C(43)	117.85(19)	F(65)-C(68)-F(66)	105.53(17)
C(45)-C(44)-H(44)	121.1	F(64)-C(68)-C(65)	113.01(18)
C(43)-C(44)-H(44)	121.1	F(65)-C(68)-C(65)	112.35(18)
C(44)-C(45)-C(46)	121.21(19)	F(66)-C(68)-C(65)	112.00(18)
C(44)-C(45)-C(48)	117.73(19)	C(51)-B(31)-C(41)	106.87(15)

C(51)-B(31)-C(31)	108.09(15)	C(41)-B(31)-C(61)	106.59(15)
C(41)-B(31)-C(31)	112.49(15)	C(31)-B(31)-C(61)	109.20(15)
C(51)-B(31)-C(61)	113.67(15)		

Symmetry transformations used to generate equivalent atoms: #1 -x+1,y,-z+1/2 #2 -x,y,-z+1/2

Table S35. Anisotropic displacement parameters ($Å^2$x 10^3) for **4a-(B$_{Ar}$F)$_2$**.
The anisotropic displacement factor exponent takes the form: $-2p^2[h^2 a^{*2}U^{11} + ... + 2 h k a^* b^* U^{12}]$

	U^{11}	U^{22}	U^{33}	U^{23}	U^{13}	U^{12}
Co(1)	13(1)	15(1)	16(1)	0	0(1)	0
N(1)	15(1)	22(1)	20(1)	5(1)	-2(1)	2(1)
N(2)	26(1)	16(1)	21(1)	-2(1)	1(1)	2(1)
C(1)	24(1)	34(1)	33(1)	9(1)	0(1)	12(1)
C(2)	18(1)	30(1)	23(1)	6(1)	1(1)	-2(1)
C(3)	22(1)	32(1)	22(1)	2(1)	3(1)	6(1)
C(4)	36(1)	22(1)	23(1)	-6(1)	4(1)	1(1)
C(5)	27(1)	23(1)	19(1)	2(1)	-1(1)	2(1)
C(6)	24(1)	32(1)	21(1)	-1(1)	-4(1)	2(1)
C(7)	19(1)	32(1)	23(1)	-1(1)	-2(1)	4(1)
N(11)	39(2)	21(2)	26(1)	0	3(1)	0
C(11)	58(2)	20(2)	24(2)	0	14(2)	0
C(12)	134(5)	18(2)	57(3)	0	49(3)	0
N(21)	36(2)	36(2)	28(2)	0	-11(1)	0
C(21)	28(2)	38(2)	35(2)	0	-14(2)	0
C(22)	61(3)	34(2)	100(4)	0	-38(3)	0
F(31)	40(1)	23(1)	24(1)	3(1)	-17(1)	-10(1)
F(32)	19(1)	20(1)	35(1)	0(1)	8(1)	-4(1)
F(33)	23(1)	13(1)	36(1)	-7(1)	4(1)	0(1)
F(34)	20(1)	25(1)	72(1)	15(1)	20(1)	10(1)
F(35)	24(1)	62(1)	28(1)	17(1)	13(1)	3(1)
F(36)	13(1)	63(1)	53(1)	-34(1)	5(1)	-6(1)
F(41)	25(1)	37(1)	28(1)	-15(1)	-3(1)	9(1)
F(42)	39(1)	22(1)	33(1)	2(1)	8(1)	14(1)
F(43)	23(1)	31(1)	44(1)	-6(1)	15(1)	-3(1)
F(44)	96(3)	41(1)	24(1)	1(1)	-21(1)	33(1)
F(45)	32(1)	60(2)	31(1)	-10(1)	-12(1)	-10(1)
F(46)	33(1)	63(2)	17(1)	-12(1)	-4(1)	9(1)
F(44A)	96(3)	41(1)	24(1)	1(1)	-21(1)	33(1)
F(45A)	32(1)	60(2)	31(1)	-10(1)	-12(1)	-10(1)
F(46A)	54(12)	77(16)	26(12)	-17(11)	-9(9)	-8(11)
F(51)	35(3)	31(2)	16(1)	4(1)	6(1)	1(2)
F(52)	42(2)	41(2)	29(2)	16(2)	5(1)	-17(2)
F(53)	52(2)	36(3)	36(3)	14(2)	14(2)	28(2)
F(51A)	35(3)	31(2)	16(1)	4(1)	6(1)	1(2)
F(52A)	78(10)	44(7)	55(9)	31(6)	-14(7)	-20(7)
F(53A)	55(7)	41(10)	18(5)	6(5)	-10(5)	29(6)
F(54)	15(1)	87(1)	71(1)	-18(1)	-4(1)	3(1)
F(55)	62(1)	40(1)	44(1)	10(1)	-21(1)	-34(1)
F(56)	30(1)	45(1)	26(1)	8(1)	-12(1)	-14(1)
F(61)	96(3)	30(2)	45(1)	-19(1)	12(2)	22(2)
F(62)	108(3)	44(2)	36(2)	-21(2)	20(2)	-46(2)
F(63)	88(2)	26(1)	12(1)	-6(1)	8(1)	-9(1)
F(61A)	96(3)	30(2)	45(1)	-19(1)	12(2)	22(2)
F(62A)	108(3)	44(2)	36(2)	-21(2)	20(2)	-46(2)
F(63A)	56(5)	33(6)	61(7)	-21(5)	-8(4)	-25(4)
F(64)	48(1)	29(1)	26(1)	2(1)	20(1)	-9(1)
F(65)	41(1)	19(1)	31(1)	3(1)	6(1)	4(1)
F(66)	42(1)	29(1)	31(1)	2(1)	-4(1)	-18(1)
C(31)	14(1)	13(1)	7(1)	1(1)	-1(1)	1(1)
C(32)	12(1)	16(1)	10(1)	1(1)	1(1)	2(1)
C(33)	14(1)	13(1)	10(1)	2(1)	-1(1)	0(1)
C(34)	16(1)	12(1)	12(1)	0(1)	2(1)	3(1)
C(35)	13(1)	17(1)	9(1)	2(1)	1(1)	2(1)
C(36)	13(1)	13(1)	12(1)	0(1)	0(1)	-1(1)
C(37)	18(1)	14(1)	17(1)	0(1)	0(1)	2(1)
C(38)	15(1)	17(1)	18(1)	-1(1)	4(1)	0(1)
C(41)	14(1)	11(1)	15(1)	1(1)	1(1)	-1(1)
C(42)	14(1)	14(1)	16(1)	-1(1)	1(1)	0(1)
C(43)	14(1)	13(1)	21(1)	1(1)	2(1)	-1(1)
C(44)	13(1)	17(1)	24(1)	2(1)	-1(1)	2(1)
C(45)	20(1)	16(1)	18(1)	2(1)	-4(1)	-2(1)
C(46)	18(1)	14(1)	16(1)	0(1)	1(1)	2(1)
C(47)	14(1)	21(1)	27(1)	-1(1)	1(1)	3(1)
C(48)	28(1)	28(1)	22(1)	-1(1)	-6(1)	7(1)
C(51)	14(1)	11(1)	14(1)	-3(1)	3(1)	4(1)
C(52)	18(1)	15(1)	15(1)	-2(1)	1(1)	3(1)
C(53)	23(1)	14(1)	18(1)	0(1)	5(1)	4(1)
C(54)	18(1)	14(1)	23(1)	0(1)	6(1)	-2(1)
C(55)	16(1)	17(1)	20(1)	-2(1)	2(1)	-1(1)
C(56)	15(1)	16(1)	16(1)	0(1)	1(1)	1(1)
C(57)	29(1)	21(1)	24(1)	6(1)	3(1)	1(1)
C(58)	21(1)	30(1)	28(1)	3(1)	-1(1)	-9(1)
C(61)	8(1)	16(1)	14(1)	-1(1)	-1(1)	3(1)
C(62)	14(1)	15(1)	15(1)	-2(1)	0(1)	1(1)
C(63)	22(1)	20(1)	15(1)	-6(1)	2(1)	0(1)
C(64)	21(1)	24(1)	12(1)	0(1)	3(1)	0(1)
C(65)	14(1)	18(1)	17(1)	0(1)	1(1)	0(1)
C(66)	14(1)	16(1)	14(1)	-3(1)	0(1)	2(1)
C(67)	88(2)	26(1)	12(1)	-6(1)	8(1)	-9(1)
C(68)	28(1)	22(1)	17(1)	0(1)	5(1)	-3(1)
B(31)	14(1)	12(1)	11(1)	-2(1)	1(1)	0(1)

Table S36. Hydrogen coordinates (x 10^4) and isotropic displacement parameters ($Å^2$x 10^3) for **4a-($B_{Ar}F$)$_2$**.

	x	y	z	U(eq)
H(1A)	3785	1542	1769	46
H(1B)	3750	1680	2507	46
H(1C)	3227	1204	2119	46
H(2A)	4059	-126	2953	28
H(2AB)	3415	314	2886	28
H(3A)	3886	1270	3447	30
H(3AB)	4011	562	3860	30
H(4A)	5386	1694	3885	40
H(4B)	4833	1494	4307	40
H(4C)	4692	1980	3693	40
H(5A)	4974	-129	3588	28
H(5AB)	5008	284	4246	28
H(6A)	6013	4	4151	31
H(6AB)	5974	825	3952	31
H(7A)	6548	105	3279	30
H(7AB)	5916	-311	3059	30
H(12A)	5123	3663	2096	100
H(12B)	5298	3663	2843	100
H(12C)	4579	3663	2561	100
H(22A)	-253	2510	2124	102
H(22B)	-177	2510	2879	102
H(22C)	430	2510	2497	102
H(32)	3447	6098	5263	15
H(34)	2008	7176	5924	16
H(36)	1729	5207	5165	15
H(42)	3808	4092	4253	17
H(44)	5218	3900	5642	22
H(46)	3654	5017	5940	19
H(52)	2872	3886	5767	19
H(54)	1359	2635	5296	22
H(56)	1841	4186	4109	19
H(62)	2748	4041	3649	18
H(64)	3178	5466	2326	23
H(66)	3106	6088	4138	17

[Co^{II}(TMC)($OTf)_2$] (**5a**)

5a was solved in the monoclinic space group $P2_1/n$. The asymmetric unit contains half of a molecule. The structure was found to be heavily disordered over three positions (ligand) and two positions (coordinated anion). The disorder was refined using same distance restraints on 1,2 and 1,3-distances, similarity restraints on the anisotropic displacement parameters and advanced rigid bond restraints. The disorder ratios were refined freely and converged to 0.542(3):0.301(3):0.157(3) and 0.536(4).

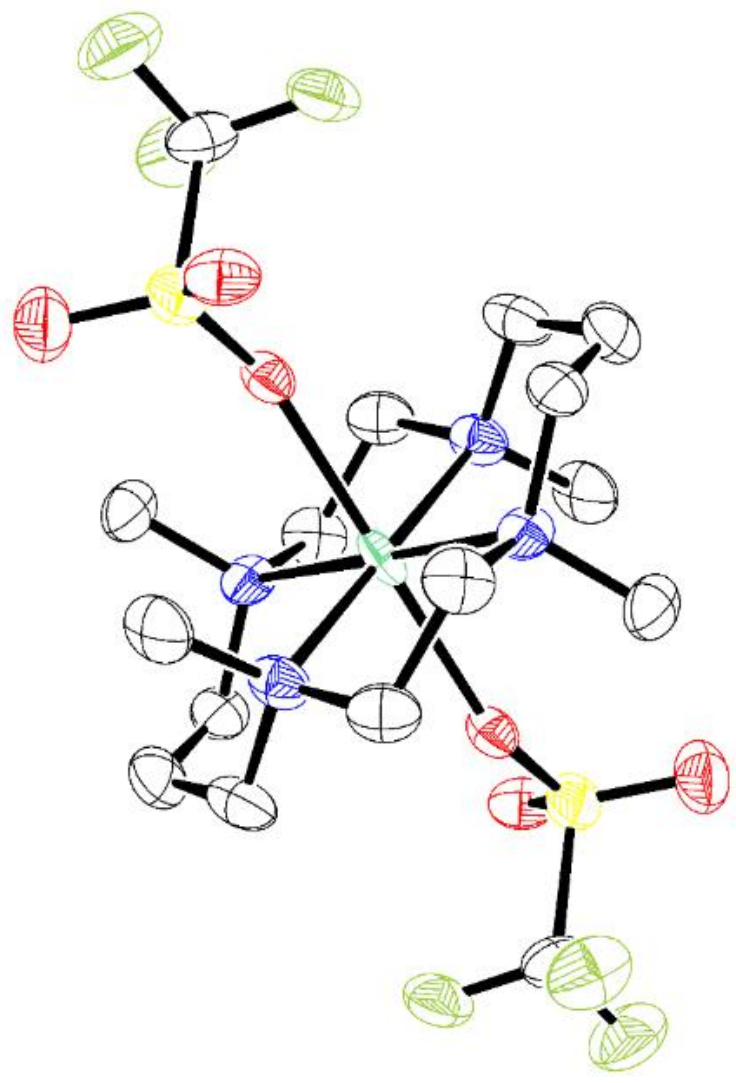

Figure S8. Molecular structure of **5a**.

Table S37. Crystal data and structure refinement for **5a**.

CCDC No	2062707	
Empirical formula	$C_{16}H_{32}CoF_6N_4O_6S_2$	
Formula weight	613.50	
Temperature	100(2) K	
Wavelength	0.71073 Å	
Crystal system	Monoclinic	
Space group	$P2_1/n$	
Unit cell dimensions	a = 8.7340(2) Å	α = 90°
	b = 10.2095(3) Å	β = 101.9440(13)°
	c = 13.8900(3) Å	γ = 90°
Volume	1211.75(5) $Å^3$	
Z	2	
Density (calculated)	1.681 Mg/m^3	
Absorption coefficient	0.967 mm^{-1}	
F(000)	634	
Crystal size	0.316 x 0.141 x 0.125 mm^3	
Theta range for data collection	2.495 to 28.279°.	
Index ranges	$-11 \le h \le 11$, $-13 \le k \le 13$, $-18 \le l \le 18$	
Reflections collected	200179	
Independent reflections	3002 [R(int) = 0.0438]	
Completeness to theta = 25.242°	100.0 %	
Absorption correction	Semi-empirical from equivalents	
Refinement method	Full-matrix least-squares on F^2	
Data / restraints / parameters	3002 / 1604 / 404	
Goodness-of-fit on F^2	1.097	
Final R indices [I>2σ(I)]	R1 = 0.0556, wR2 = 0.1394	
R indices (all data)	R1 = 0.0586, wR2 = 0.1420	
Largest diff. peak and hole	1.299 and -0.646 $eÅ^{-3}$	

Table S38. Atomic coordinates (x 10^4) and equivalent isotropic displacement parameters ($Å^2$x 10^3) for **5a**.
U(eq) is defined as one third of the trace of the orthogonalized U^{ij} tensor.

	x	y	z	U(eq)
Co(1)	5000	5000	5000	29(1)
N(1)	3046(5)	3751(4)	4990(4)	32(1)
N(2)	5781(5)	4388(5)	6514(3)	35(1)
C(1)	3090(7)	3499(8)	6054(5)	42(1)
C(2)	4795(7)	3208(6)	6560(5)	42(1)
C(3)	5553(9)	5297(7)	7315(4)	50(2)
C(4)	7445(6)	4008(7)	6697(4)	44(1)
C(12)	1462(7)	4881(7)	3497(5)	48(1)
C(13)	1584(6)	4467(6)	4555(5)	40(1)
C(14)	3021(7)	2458(6)	4484(5)	45(1)
N(1A)	2762(9)	4620(8)	4109(6)	37(2)
N(2A)	4376(10)	3600(9)	6027(6)	40(2)
C(1A)	2268(12)	3398(10)	4519(8)	44(2)
C(2A)	2634(13)	3516(15)	5655(9)	49(2)
C(3A)	4997(18)	2245(11)	6049(11)	60(3)
C(4A)	4693(15)	4133(14)	7036(7)	47(2)
C(5A)	6465(15)	4435(14)	7399(9)	55(3)
C(6A)	7123(13)	5614(12)	6934(7)	45(2)
C(14A)	1524(11)	5621(12)	4090(9)	47(3)
N(1B)	3078(18)	5184(16)	3782(11)	45(3)
N(2B)	3599(18)	3753(14)	5640(11)	37(2)
C(1B)	2020(20)	4120(20)	3978(13)	52(3)
C(2B)	2018(19)	4040(20)	5090(13)	48(3)
C(3B)	3910(30)	2327(15)	5635(18)	49(4)
C(4B)	3670(20)	4170(20)	6681(12)	45(3)
C(12B)	4680(20)	5960(30)	2677(17)	64(6)
C(13B)	3440(30)	4930(20)	2802(14)	53(5)
C(14B)	2180(30)	6440(20)	3720(20)	71(6)
S(1)	3044(6)	7624(5)	6125(3)	39(1)
F(1)	841(4)	8252(4)	4611(3)	51(1)
F(2)	1339(7)	9725(5)	5746(5)	69(1)
F(3)	2936(7)	9404(7)	4769(5)	61(2)
O(1)	3705(9)	6783(10)	5465(5)	32(1)
O(2)	1863(9)	6943(11)	6515(8)	40(2)
O(3)	4170(6)	8385(6)	6771(4)	58(2)
C(21)	1973(8)	8819(8)	5261(6)	43(2)
S(1A)	3216(6)	7455(5)	6383(3)	36(1)
F(1A)	2337(12)	9163(8)	4959(6)	81(3)
F(2A)	2314(9)	9938(5)	6384(6)	72(2)
F(3A)	4515(7)	9632(5)	5968(4)	66(2)
O(1A)	4045(10)	6776(12)	5740(6)	34(2)
O(2A)	1589(12)	7143(15)	6310(11)	63(4)
O(3A)	4086(7)	7630(6)	7370(4)	53(1)
C(21A)	3080(9)	9141(7)	5904(6)	52(2)

Table S39. Bond lengths [Å] and angles [°] for **5a**.

Co(1)-N(2B)	2.087(16)	F(3)-C(21)	1.330(8)
Co(1)-N(2B)#1	2.087(16)	S(1A)-O(3A)	1.434(7)
Co(1)-N(1A)	2.121(8)	S(1A)-O(2A)	1.439(10)
Co(1)-N(1A)#1	2.121(8)	S(1A)-O(1A)	1.439(8)
Co(1)-N(1)#1	2.128(4)	S(1A)-C(21A)	1.840(9)
Co(1)-N(1)	2.128(4)	F(1A)-C(21A)	1.338(9)
Co(1)-N(1B)	2.130(15)	F(2A)-C(21A)	1.319(7)
Co(1)-N(1B)#1	2.130(15)	F(3A)-C(21A)	1.335(8)
Co(1)-N(2)	2.165(4)		
Co(1)-N(2)#1	2.165(4)	N(1)#1-Co(1)-N(1)	180.0
Co(1)-N(2A)	2.167(9)	N(2B)-Co(1)-N(1B)	87.5(6)
Co(1)-N(2A)#1	2.167(9)	N(2B)#1-Co(1)-N(1B)	92.5(6)
N(1)-C(13)	1.486(6)	N(1)#1-Co(1)-N(1B)#1	63.2(4)
N(1)-C(1)	1.493(7)	N(1)-Co(1)-N(1B)#1	116.8(4)
N(1)-C(14)	1.493(7)	N(1)#1-Co(1)-N(2)	94.33(18)
N(2)-C(4)	1.474(6)	N(1)-Co(1)-N(2)	85.67(18)
N(2)-C(2)	1.490(7)	N(1)#1-Co(1)-N(2)#1	85.67(18)
N(2)-C(3)	1.493(7)	N(1)-Co(1)-N(2)#1	94.33(18)
C(1)-C(2)	1.538(8)	N(2)-Co(1)-N(2)#1	180.0
C(1)-H(1A)	0.9900	N(1A)-Co(1)-N(2A)	86.4(3)
C(1)-H(1AB)	0.9900	N(1A)#1-Co(1)-N(2A)	93.6(3)
C(2)-H(2A)	0.9900	N(1)#1-Co(1)-N(2A)#1	45.5(3)
C(2)-H(2AB)	0.9900	N(1)-Co(1)-N(2A)#1	134.5(3)
C(3)-H(3A)	0.9800	N(2)-Co(1)-N(2A)#1	139.3(3)
C(3)-H(3B)	0.9800	N(2)#1-Co(1)-N(2A)#1	40.7(3)
C(3)-H(3C)	0.9800	C(13)-N(1)-C(1)	109.3(4)
C(4)-C(12)#1	1.541(8)	C(13)-N(1)-C(14)	108.4(5)
C(4)-H(4A)	0.9900	C(1)-N(1)-C(14)	107.9(5)
C(4)-H(4AB)	0.9900	C(13)-N(1)-Co(1)	109.2(3)
C(12)-C(13)	1.512(8)	C(1)-N(1)-Co(1)	103.9(4)
C(12)-H(12A)	0.9900	C(14)-N(1)-Co(1)	117.8(3)
C(12)-H(12B)	0.9900	C(4)-N(2)-C(2)	109.8(5)
C(13)-H(13A)	0.9900	C(4)-N(2)-C(3)	108.3(5)
C(13)-H(13B)	0.9900	C(2)-N(2)-C(3)	107.6(5)
C(14)-H(14A)	0.9800	C(4)-N(2)-Co(1)	110.2(3)
C(14)-H(14B)	0.9800	C(2)-N(2)-Co(1)	101.9(3)
C(14)-H(14C)	0.9800	C(3)-N(2)-Co(1)	118.7(4)
N(1A)-C(1A)	1.473(10)	N(1)-C(1)-C(2)	108.1(5)
N(1A)-C(14A)	1.484(10)	N(1)-C(1)-H(1A)	110.1
N(1A)-C(6A)#1	1.492(10)	C(2)-C(1)-H(1A)	110.1
N(2A)-C(4A)	1.475(11)	N(1)-C(1)-H(1AB)	110.1
N(2A)-C(3A)	1.484(11)	C(2)-C(1)-H(1AB)	110.1
N(2A)-C(2A)	1.504(11)	H(1A)-C(1)-H(1AB)	108.4
C(1A)-C(2A)	1.548(12)	N(2)-C(2)-C(1)	109.8(5)
C(1A)-H(1AA)	0.9900	N(2)-C(2)-H(2A)	109.7
C(1A)-H(1AC)	0.9900	C(1)-C(2)-H(2A)	109.7
C(2A)-H(2AA)	0.9900	N(2)-C(2)-H(2AB)	109.7
C(2A)-H(2AC)	0.9900	C(1)-C(2)-H(2AB)	109.7
C(3A)-H(3AA)	0.9800	H(2A)-C(2)-H(2AB)	108.2
C(3A)-H(3AB)	0.9800	N(2)-C(3)-H(3A)	109.5
C(3A)-H(3AC)	0.9800	N(2)-C(3)-H(3B)	109.5
C(4A)-C(5A)	1.556(12)	H(3A)-C(3)-H(3B)	109.5
C(4A)-H(4AA)	0.9900	N(2)-C(3)-H(3C)	109.5
C(4A)-H(4AC)	0.9900	H(3A)-C(3)-H(3C)	109.5
C(5A)-C(6A)	1.533(13)	H(3B)-C(3)-H(3C)	109.5
C(5A)-H(5AA)	0.9900	N(2)-C(4)-C(12)#1	113.6(5)
C(5A)-H(5AB)	0.9900	N(2)-C(4)-H(4A)	108.8
C(6A)-H(6AA)	0.9900	C(12)#1-C(4)-H(4A)	108.8
C(6A)-H(6AB)	0.9900	N(2)-C(4)-H(4AB)	108.8
C(14A)-H(14D)	0.9800	C(12)#1-C(4)-H(4AB)	108.8
C(14A)-H(14E)	0.9800	H(4A)-C(4)-H(4AB)	107.7
C(14A)-H(14F)	0.9800	C(13)-C(12)-C(4)#1	117.0(5)
N(1B)-C(13B)	1.482(14)	C(13)-C(12)-H(12A)	108.1
N(1B)-C(1B)	1.489(13)	C(4)#1-C(12)-H(12A)	108.1
N(1B)-C(14B)	1.493(14)	C(13)-C(12)-H(12B)	108.1
N(2B)-C(2B)	1.464(13)	C(4)#1-C(12)-H(12B)	108.1
N(2B)-C(3B)	1.482(13)	H(12A)-C(12)-H(12B)	107.3
N(2B)-C(4B)	1.496(13)	N(1)-C(13)-C(12)	114.4(5)
C(1B)-C(2B)	1.547(14)	N(1)-C(13)-H(13A)	108.7
C(1B)-H(1BA)	0.9900	C(12)-C(13)-H(13A)	108.7
C(1B)-H(1BB)	0.9900	N(1)-C(13)-H(13B)	108.7
C(2B)-H(2BA)	0.9900	C(12)-C(13)-H(13B)	108.7
C(2B)-H(2BB)	0.9900	H(13A)-C(13)-H(13B)	107.6
C(3B)-H(3BA)	0.9800	N(1)-C(14)-H(14A)	109.5
C(3B)-H(3BB)	0.9800	N(1)-C(14)-H(14B)	109.5
C(3B)-H(3BC)	0.9800	H(14A)-C(14)-H(14B)	109.5
C(4B)-C(12B)#1	1.529(15)	N(1)-C(14)-H(14C)	109.5
C(4B)-H(4BA)	0.9900	H(14A)-C(14)-H(14C)	109.5
C(4B)-H(4BB)	0.9900	H(14B)-C(14)-H(14C)	109.5
C(12B)-C(13B)	1.547(15)	C(1A)-N(1A)-C(14A)	108.6(8)
C(12B)-H(12C)	0.9900	C(1A)-N(1A)-Co(1)	103.8(6)
C(12B)-H(12D)	0.9900	C(14A)-N(1A)-Co(1)	117.7(6)
C(13B)-H(13C)	0.9900	C(6A)#1-N(1A)-Co(1)	110.9(6)
C(13B)-H(13D)	0.9900	C(4A)-N(2A)-C(3A)	109.1(10)
C(14B)-H(14G)	0.9800	C(4A)-N(2A)-C(2A)	109.0(8)
C(14B)-H(14H)	0.9800	C(3A)-N(2A)-C(2A)	107.1(10)
C(14B)-H(14I)	0.9800	C(4A)-N(2A)-Co(1)	111.0(7)
S(1)-O(3)	1.418(6)	C(3A)-N(2A)-Co(1)	119.2(7)
S(1)-O(2)	1.439(8)	C(2A)-N(2A)-Co(1)	100.7(7)
S(1)-O(1)	1.460(8)	N(1A)-C(1A)-C(2A)	108.2(8)
S(1)-C(21)	1.826(9)	N(1A)-C(1A)-H(1AA)	110.1
F(1)-C(21)	1.327(9)	C(2A)-C(1A)-H(1AA)	110.1
F(2)-C(21)	1.330(9)	N(1A)-C(1A)-H(1AC)	110.1

C(2A)-C(1A)-H(1AC)	110.1
H(1AA)-C(1A)-H(1AC)	108.4
N(2A)-C(2A)-C(1A)	109.6(9)
N(2A)-C(2A)-H(2AA)	109.8
C(1A)-C(2A)-H(2AA)	109.8
N(2A)-C(2A)-H(2AC)	109.8
C(1A)-C(2A)-H(2AC)	109.8
H(2AA)-C(2A)-H(2AC)	108.2
N(2A)-C(3A)-H(3AA)	109.5
N(2A)-C(3A)-H(3AB)	109.5
H(3AA)-C(3A)-H(3AB)	109.5
N(2A)-C(3A)-H(3AC)	109.5
H(3AA)-C(3A)-H(3AC)	109.5
H(3AB)-C(3A)-H(3AC)	109.5
N(2A)-C(4A)-C(5A)	111.1(9)
N(2A)-C(4A)-H(4AA)	109.4
C(5A)-C(4A)-H(4AA)	109.4
N(2A)-C(4A)-H(4AC)	109.4
C(5A)-C(4A)-H(4AC)	109.4
H(4AA)-C(4A)-H(4AC)	108.0
C(6A)-C(5A)-C(4A)	117.0(11)
C(6A)-C(5A)-H(5AA)	108.1
C(4A)-C(5A)-H(5AA)	108.1
C(6A)-C(5A)-H(5AB)	108.1
C(4A)-C(5A)-H(5AB)	108.1
H(5AA)-C(5A)-H(5AB)	107.3
N(1A)#1-C(6A)-C(5A)	113.0(9)
N(1A)#1-C(6A)-H(6AA)	109.0
C(5A)-C(6A)-H(6AA)	109.0
N(1A)#1-C(6A)-H(6AB)	109.0
C(5A)-C(6A)-H(6AB)	109.0
H(6AA)-C(6A)-H(6AB)	107.8
N(1A)-C(14A)-H(14D)	109.5
N(1A)-C(14A)-H(14E)	109.5
H(14D)-C(14A)-H(14E)	109.5
N(1A)-C(14A)-H(14F)	109.5
H(14D)-C(14A)-H(14F)	109.5
H(14E)-C(14A)-H(14F)	109.5
C(13B)-N(1B)-C(1B)	107.3(13)
C(13B)-N(1B)-C(14B)	108.1(15)
C(1B)-N(1B)-C(14B)	107.2(15)
C(13B)-N(1B)-Co(1)	115.5(12)
C(1B)-N(1B)-Co(1)	102.0(10)
C(14B)-N(1B)-Co(1)	115.9(13)
C(2B)-N(2B)-C(3B)	110.5(15)
C(2B)-N(2B)-C(4B)	107.2(12)
C(3B)-N(2B)-C(4B)	108.1(14)
C(2B)-N(2B)-Co(1)	103.1(11)
C(3B)-N(2B)-Co(1)	118.3(11)
C(4B)-N(2B)-Co(1)	109.1(11)
N(1B)-C(1B)-C(2B)	110.3(13)
N(1B)-C(1B)-H(1BA)	109.6
C(2B)-C(1B)-H(1BA)	109.6
N(1B)-C(1B)-H(1BB)	109.6
C(2B)-C(1B)-H(1BB)	109.6
H(1BA)-C(1B)-H(1BB)	108.1
N(2B)-C(2B)-C(1B)	109.6(13)
N(2B)-C(2B)-H(2BA)	109.7
C(1B)-C(2B)-H(2BA)	109.7
N(2B)-C(2B)-H(2BB)	109.7
C(1B)-C(2B)-H(2BB)	109.7
H(2BA)-C(2B)-H(2BB)	108.2
N(2B)-C(3B)-H(3BA)	109.5
N(2B)-C(3B)-H(3BB)	109.5
H(3BA)-C(3B)-H(3BB)	109.5
N(2B)-C(3B)-H(3BC)	109.5
H(3BA)-C(3B)-H(3BC)	109.5
H(3BB)-C(3B)-H(3BC)	109.5
N(2B)-C(4B)-H(4BA)	109.0
C(12B)#1-C(4B)-H(4BA)	109.0
N(2B)-C(4B)-H(4BB)	109.0
C(12B)#1-C(4B)-H(4BB)	109.0
H(4BA)-C(4B)-H(4BB)	107.8
C(4B)#1-C(12B)-C(13B)	118(2)
C(4B)#1-C(12B)-H(12C)	107.7
C(13B)-C(12B)-H(12C)	107.7
C(4B)#1-C(12B)-H(12D)	107.7
C(13B)-C(12B)-H(12D)	107.7
H(12C)-C(12B)-H(12D)	107.1
N(1B)-C(13B)-C(12B)	105.8(15)
N(1B)-C(13B)-H(13C)	110.6
C(12B)-C(13B)-H(13C)	110.6
N(1B)-C(13B)-H(13D)	110.6
C(12B)-C(13B)-H(13D)	110.6
H(13C)-C(13B)-H(13D)	108.7
N(1B)-C(14B)-H(14G)	109.5
N(1B)-C(14B)-H(14H)	109.5
H(14G)-C(14B)-H(14H)	109.5
N(1B)-C(14B)-H(14I)	109.5
H(14G)-C(14B)-H(14I)	109.5
H(14H)-C(14B)-H(14I)	109.5
O(3)-S(1)-O(2)	118.9(6)
O(3)-S(1)-O(1)	114.0(5)
O(2)-S(1)-O(1)	111.1(7)
O(3)-S(1)-C(21)	104.1(5)
O(2)-S(1)-C(21)	105.1(5)
O(1)-S(1)-C(21)	101.3(4)
S(1)-O(1)-Co(1)	157.2(6)
F(1)-C(21)-F(3)	107.9(7)
F(1)-C(21)-F(2)	108.5(6)
F(3)-C(21)-F(2)	108.7(8)
F(1)-C(21)-S(1)	111.3(5)
F(3)-C(21)-S(1)	110.2(6)
F(2)-C(21)-S(1)	110.0(6)
O(3A)-S(1A)-O(2A)	114.5(7)
O(3A)-S(1A)-O(1A)	114.7(5)
O(2A)-S(1A)-O(1A)	117.8(8)
O(3A)-S(1A)-C(21A)	102.1(5)
O(2A)-S(1A)-C(21A)	101.1(7)
O(1A)-S(1A)-C(21A)	103.3(6)
S(1A)-O(1A)-Co(1)	157.5(8)
F(2A)-C(21A)-F(3A)	107.6(6)
F(2A)-C(21A)-F(1A)	106.9(7)
F(3A)-C(21A)-F(1A)	108.5(8)
F(2A)-C(21A)-S(1A)	113.3(6)
F(3A)-C(21A)-S(1A)	109.7(5)
F(1A)-C(21A)-S(1A)	110.6(6)

Symmetry transformations used to generate equivalent atoms: #1 -x+1,-y+1,-z+1

Table S40. Anisotropic displacement parameters (Å^{2}x 10^3) for **5a**.
The anisotropic displacement factor exponent takes the form: -2p^2[h^2 a*^{2}U^{11} + ... + 2 h k a* b* U^{12}]

	U^{11}	U^{22}	U^{33}	U^{23}	U^{13}	U^{12}
Co(1)	19(1)	43(1)	21(1)	7(1)	-3(1)	-12(1)
N(1)	19(2)	32(2)	49(2)	-5(2)	15(2)	0(2)
N(2)	35(2)	46(2)	24(2)	4(2)	6(2)	7(2)
C(1)	34(3)	47(3)	49(3)	9(3)	18(3)	-4(3)
C(2)	44(3)	45(3)	38(3)	12(2)	13(2)	7(2)
C(3)	57(4)	71(4)	23(2)	-3(3)	6(2)	14(3)
C(4)	37(3)	64(4)	30(3)	6(3)	2(2)	17(3)
C(12)	28(3)	57(4)	55(3)	-11(3)	-1(2)	0(2)
C(13)	18(2)	41(3)	60(3)	-4(2)	8(2)	-1(2)
C(14)	30(3)	37(3)	68(4)	-13(3)	13(3)	-8(2)
N(1A)	19(3)	37(4)	52(4)	-22(3)	1(3)	-4(3)
N(2A)	34(4)	55(4)	34(4)	7(3)	16(3)	3(3)
C(1A)	23(4)	41(4)	64(5)	-13(4)	3(4)	-9(3)
C(2A)	39(5)	49(5)	61(5)	0(5)	16(4)	-13(4)
C(3A)	79(8)	45(5)	68(8)	13(5)	41(7)	3(6)
C(4A)	55(5)	59(5)	31(4)	8(4)	16(4)	1(5)
C(5A)	57(6)	64(6)	39(5)	0(5)	-1(4)	8(5)
C(6A)	40(5)	59(6)	32(5)	-12(4)	-4(4)	5(4)
C(14A)	27(4)	56(6)	51(6)	-14(5)	-11(4)	9(4)
N(1B)	30(6)	51(7)	44(6)	-6(5)	-15(5)	-10(5)
N(2B)	27(5)	39(5)	50(5)	-2(5)	19(5)	-4(5)
C(1B)	30(6)	63(7)	57(6)	-6(6)	-2(5)	-11(6)
C(2B)	28(5)	54(7)	59(6)	-8(6)	2(5)	-8(5)
C(3B)	53(9)	39(6)	69(10)	8(7)	45(8)	-5(7)
C(4B)	42(6)	54(7)	44(5)	5(5)	19(5)	-2(6)
C(12B)	47(12)	80(15)	65(14)	10(13)	16(10)	8(11)
C(13B)	34(9)	71(12)	50(8)	14(9)	-4(7)	9(9)
C(14B)	75(13)	75(10)	48(12)	-6(10)	-22(10)	22(9)
S(1)	28(1)	44(2)	45(2)	-3(1)	6(1)	0(1)
F(1)	32(2)	58(2)	57(2)	4(2)	1(2)	13(2)
F(2)	68(3)	51(2)	92(4)	-11(2)	27(3)	18(2)
F(3)	57(3)	48(3)	88(4)	13(2)	36(3)	-4(2)
O(1)	23(3)	42(2)	31(3)	-1(3)	3(2)	1(2)
O(2)	33(3)	50(3)	44(3)	6(2)	20(3)	11(3)
O(3)	44(2)	68(3)	61(3)	-29(3)	5(2)	0(2)
C(21)	35(3)	37(3)	60(4)	6(3)	20(3)	8(2)
S(1A)	27(2)	33(1)	51(2)	-6(1)	14(2)	0(1)
F(1A)	104(8)	67(6)	66(4)	4(4)	-1(5)	48(6)
F(2A)	81(4)	54(3)	93(4)	-16(3)	46(4)	18(3)
F(3A)	82(3)	43(2)	86(4)	-1(2)	50(3)	-7(2)
O(1A)	22(4)	41(3)	41(4)	5(4)	6(3)	5(3)
O(2A)	34(4)	62(7)	97(9)	-16(6)	22(4)	-7(4)
O(3A)	66(4)	53(3)	43(3)	-12(2)	15(2)	-7(3)
C(21A)	59(4)	40(3)	60(4)	-4(3)	22(3)	15(3)

Table S41. Hydrogen coordinates (x 10^4) and isotropic displacement parameters ($Å^2$x 10^3) for **5a**.

	x	y	z	U(eq)
H(1A)	2415	2742	6128	50
H(1AB)	2703	4275	6358	50
H(2A)	4844	2964	7256	50
H(2AB)	5195	2463	6229	50
H(3A)	6110	6118	7264	76
H(3B)	5963	4891	7956	76
H(3C)	4434	5477	7250	76
H(4A)	7564	3254	6271	53
H(4AB)	7774	3720	7390	53
H(12A)	370	5156	3231	57
H(12B)	1669	4103	3117	57
H(13A)	1516	5258	4957	48
H(13B)	679	3902	4595	48
H(14A)	3123	2595	3802	67
H(14B)	2030	2012	4491	67
H(14C)	3893	1919	4827	67
H(1AA)	2839	2644	4314	52
H(1AC)	1133	3256	4273	52
H(2AA)	2126	4310	5853	59
H(2AC)	2215	2744	5946	59
H(3AA)	4819	1896	5378	90
H(3AB)	4461	1692	6453	90
H(3AC)	6123	2253	6333	90
H(4AA)	4356	3491	7485	56
H(4AC)	4081	4946	7050	56
H(5AA)	6662	4576	8119	66
H(5AB)	7064	3649	7282	66
H(6AA)	6443	6383	6961	54
H(6AB)	8177	5823	7324	54
H(14D)	592	5376	3599	71
H(14E)	1906	6475	3918	71
H(14F)	1257	5674	4740	71
H(1BA)	2372	3271	3754	62
H(1BB)	943	4287	3604	62
H(2BA)	1659	4888	5315	57
H(2BB)	1288	3349	5210	57
H(3BA)	2916	1854	5439	74
H(3BB)	4434	2047	6296	74
H(3BC)	4580	2135	5168	74
H(4BA)	2941	3625	6967	54
H(4BB)	3328	5092	6687	54
H(12C)	4787	5937	1980	76
H(12D)	4273	6831	2799	76
H(13C)	2488	5025	2278	64
H(13D)	3855	4030	2772	64
H(14G)	1828	6574	4343	106
H(14H)	1271	6390	3178	106
H(14I)	2855	7165	3616	106

[(TMC)Co^{II}(OTf)]B$_{Ar}$F, **5a-B$_{Ar}$F**

The structure of **5a-B$_{Ar}$F** was solved in the monoclinic space group $P2_1/c$. The asymmetric unit contains one full complex and anion. The ligand was found to be fully disordered and six of the CF_3 groups were also found to be disordered. The disorder was refined with the help of same distance restraints on 1,2 and 1,3 distances, similarity restraints on anisotropic displacement parameters and advanced rigid bond restraints. Some atoms had to be set to the same anisotropic displacement parameters to allow for anisotropic refinement. The disorder ratio for the ligand converged to 0.921(3) and for the CF_3 groups to 0.378(2):0.353(3):0.269(3), 0.78(2), 0.81(1), 0.905(8), 0.74(1) and 0.803(6).

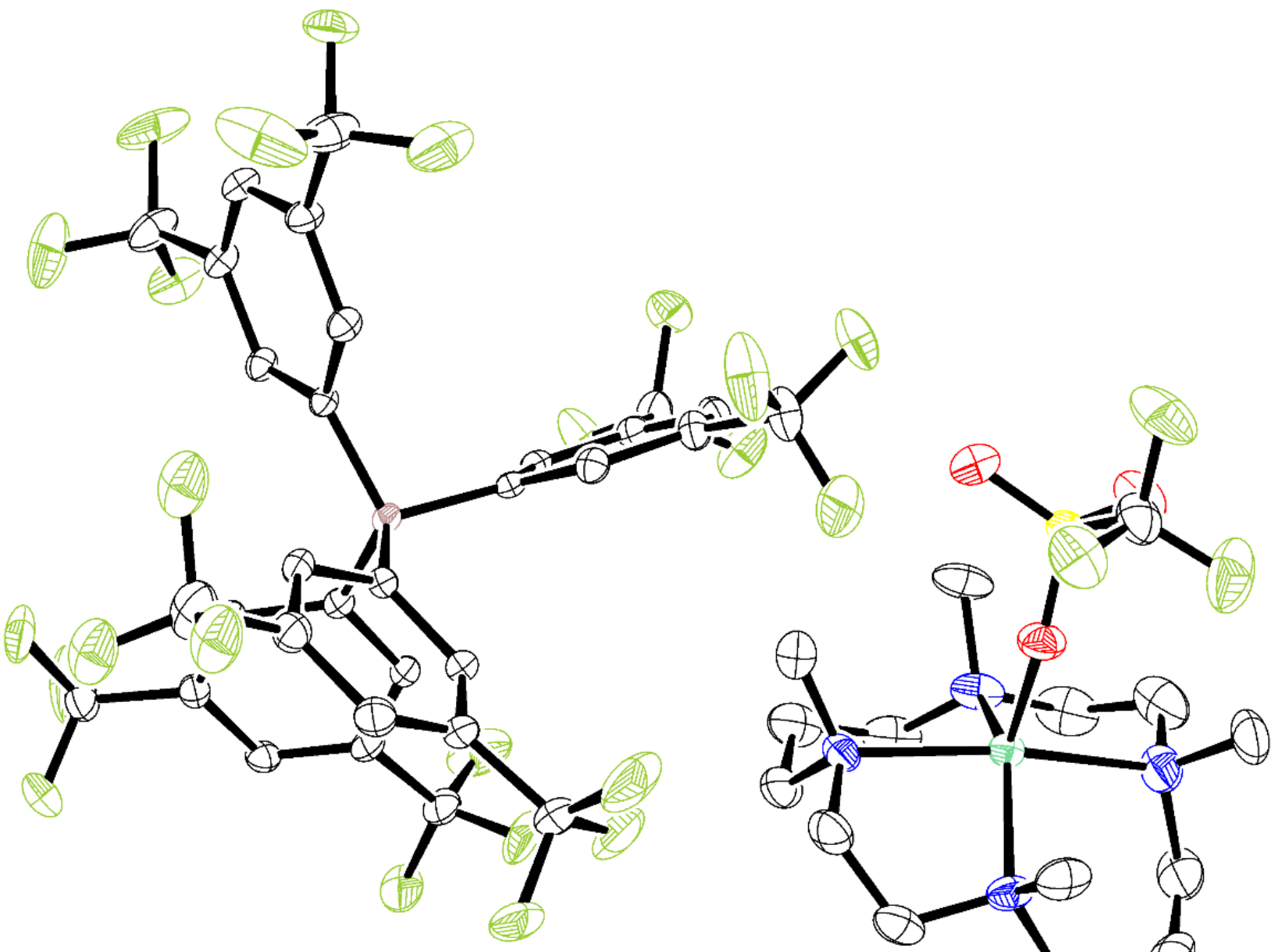

Figure S9. Molecular structure of **5a-B$_{Ar}$F**.

Table S42. Crystal data and structure refinement for **5a-B$_{Ar}$F**.

CCDC No	2062714	
Empirical formula	$C_{47}H_{44}BCoF_{27}N_4O_3S$	
Formula weight	1327.66	
Temperature	100(2) K	
Wavelength	0.71073 Å	
Crystal system	Monoclinic	
Space group	$P2_1/c$	
Unit cell dimensions	a = 12.2496(4) Å	α = 90°
	b = 25.1588(8) Å	β = 97.3036(12)°
	c = 17.3931(6) Å	γ = 90°
Volume	5316.8(3) Å^3	
Z	4	
Density (calculated)	1.659 Mg/m^3	
Absorption coefficient	0.503 mm^{-1}	
F(000)	2676	
Crystal size	0.413 x 0.109 x 0.091 mm^3	
Theta range for data collection	2.316 to 27.102°.	
Index ranges	-15 ≤ h ≤ 15, -32 ≤ k ≤ 32, -22 ≤ l ≤ 22	
Reflections collected	1249509	
Independent reflections	11724 [R(int) = 0.0672]	
Completeness to theta = 25.242°	99.9 %	
Absorption correction	Semi-empirical from equivalents	
Refinement method	Full-matrix least-squares on F^2	
Data / restraints / parameters	11724 / 4716 / 1043	
Goodness-of-fit on F^2	1.095	
Final R indices [I>2σ(I)]	R1 = 0.0688, wR2 = 0.1797	
R indices (all data)	R1 = 0.0738, wR2 = 0.1838	
Largest diff. peak and hole	1.537 and -1.194 eÅ^{-3}	

Table S43. Atomic coordinates (x 10^4) and equivalent isotropic displacement parameters (Å^{2}x 10^3) for **5a-B$_{ArF}$**. U(eq) is defined as one third of the trace of the orthogonalized U^{ij} tensor.

	x	y	z	U(eq)
Co(1)	9684(1)	8213(1)	7409(1)	21(1)
N(1)	11034(3)	8771(2)	7643(2)	40(1)
N(2)	9980(3)	8086(1)	6249(2)	29(1)
N(3)	8196(3)	7734(2)	7035(2)	30(1)
N(4)	8843(3)	8725(1)	8098(2)	34(1)
C(1)	11084(5)	9140(2)	6982(3)	50(1)
C(2)	11243(4)	8881(2)	6226(3)	49(1)
C(3)	10264(4)	8574(2)	5834(2)	37(1)
C(4)	10873(4)	7690(2)	6245(3)	36(1)
C(5)	8960(4)	7860(2)	5814(2)	35(1)
C(6)	8416(4)	7479(2)	6303(2)	35(1)
C(7)	7939(4)	7311(2)	7567(3)	41(1)
C(8)	7204(3)	8085(2)	6861(3)	38(1)
C(9)	6939(4)	8429(2)	7530(3)	46(1)
C(10)	7724(4)	8883(2)	7732(3)	43(1)
C(11)	8759(5)	8460(2)	8864(2)	42(1)
C(12)	9538(5)	9210(2)	8237(3)	52(1)
C(13)	10750(5)	9072(2)	8317(3)	53(1)
C(14)	12161(4)	8544(2)	7862(3)	49(1)
N(1A)	10520(20)	8253(11)	6372(17)	41(4)
N(2A)	8680(30)	7597(11)	7025(19)	39(4)
N(3A)	8110(20)	8511(12)	7841(16)	44(4)
N(4A)	10500(30)	8922(12)	7749(18)	48(4)
C(1A)	9660(30)	8158(14)	5710(20)	41(5)
C(2A)	9170(40)	7605(16)	5660(19)	43(4)
C(3A)	9250(40)	7329(15)	6440(20)	43(5)
C(4A)	8220(50)	7213(19)	7540(30)	41(1)
C(5A)	7750(40)	7943(18)	6710(20)	45(4)
C(6A)	7260(30)	8183(16)	7380(20)	46(4)
C(7A)	8110(40)	8420(20)	8664(18)	58(9)
C(8A)	7930(40)	9080(13)	7620(30)	44(5)
C(9A)	8820(30)	9452(15)	7970(30)	51(5)
C(10A)	9880(30)	9436(15)	7620(30)	49(5)
C(11A)	11090(50)	8940(20)	8580(20)	56(9)
C(12A)	11390(30)	8920(20)	7240(20)	49(4)
C(13A)	10890(40)	8804(13)	6410(20)	45(5)
C(14A)	11460(30)	7880(19)	6390(30)	50(9)
S(1)	10763(1)	7421(1)	8796(1)	28(1)
F(1)	11569(2)	6601(1)	8136(2)	50(1)
F(2)	12236(3)	6729(1)	9327(2)	58(1)
F(3)	12723(2)	7231(1)	8431(2)	64(1)
O(1)	10454(2)	7607(1)	7997(2)	33(1)
O(2)	11236(3)	7821(1)	9315(2)	45(1)
O(3)	9937(2)	7096(1)	9067(2)	40(1)
C(15)	11892(3)	6968(2)	8662(3)	38(1)
F(21)	1777(6)	5545(2)	9223(4)	38(1)
F(22)	1282(3)	4743(2)	8897(4)	58(1)
F(23)	2070(4)	4908(2)	10040(2)	54(1)
F(21A)	1620(30)	5490(9)	9057(18)	47(6)
F(22A)	1469(13)	4642(6)	9300(16)	58(1)
F(23A)	2392(15)	5125(9)	10100(6)	54(1)
F(24)	5447(3)	3899(2)	10128(2)	34(1)
F(25)	6621(2)	4120(2)	9398(2)	61(1)
F(26)	5373(5)	3542(1)	9011(2)	63(1)
F(24A)	5881(15)	3959(8)	10091(7)	52(4)
F(25A)	6234(11)	3811(6)	8908(8)	61(1)
F(26A)	4826(10)	3514(4)	9305(9)	40(3)
F(31)	807(4)	5421(2)	6237(4)	46(1)
F(32)	771(5)	5890(4)	5221(2)	64(2)
F(33)	327(4)	6236(2)	6245(6)	59(2)
F(31A)	956(14)	5601(13)	5347(14)	70(6)
F(32A)	337(13)	6275(6)	5836(16)	50(5)
F(33A)	671(16)	5585(12)	6517(14)	60(5)
F(34)	3348(2)	7498(1)	5461(2)	46(1)
F(35)	3939(3)	7608(1)	6662(2)	59(1)
F(36)	4992(2)	7286(1)	5898(2)	42(1)
F(41)	5134(5)	7217(4)	9130(6)	46(2)
F(42)	6854(4)	7456(2)	9257(3)	42(1)
F(43)	6286(4)	6936(2)	10068(2)	33(1)
F(41A)	5046(10)	7114(10)	9251(16)	31(4)
F(42A)	6613(13)	7159(8)	9948(9)	61(5)
F(43A)	6315(19)	7510(4)	8836(12)	73(5)
F(44)	9496(2)	5993(2)	8046(2)	53(1)
F(45)	9547(2)	5911(1)	9270(2)	40(1)
F(46)	9045(3)	5258(1)	8537(3)	55(1)
F(44A)	9340(20)	5687(16)	7905(10)	53(1)
F(45A)	9729(16)	6119(11)	8930(20)	54(7)
F(46A)	9190(20)	5319(9)	8940(20)	47(6)
F(51)	7643(2)	5880(1)	5456(2)	48(1)
F(52)	7300(2)	5312(1)	4543(1)	43(1)
F(53)	8489(2)	5145(1)	5523(2)	67(1)
F(54)	4241(5)	3741(2)	5394(4)	42(1)
F(55)	5234(6)	3508(2)	6477(4)	42(1)
F(56)	5897(7)	3499(4)	5451(5)	42(1)
F(54A)	4630(7)	3585(3)	6243(4)	42(1)
F(55A)	6061(7)	3508(4)	5636(5)	42(1)
F(56A)	4568(7)	3797(3)	5028(4)	42(1)
F(54B)	4082(7)	3720(3)	5894(6)	42(1)
F(55B)	5741(7)	3436(3)	6115(5)	42(1)
F(56B)	5089(9)	3685(4)	5008(5)	42(1)

C(21)	4449(2)	5131(1)	8074(2)	15(1)
C(22)	3481(2)	5236(1)	8393(2)	17(1)
C(23)	3180(2)	4937(1)	9014(2)	19(1)
C(24)	3833(3)	4529(1)	9342(2)	20(1)
C(25)	4802(3)	4423(1)	9040(2)	20(1)
C(26)	5093(2)	4712(1)	8416(2)	18(1)
C(27)	2101(3)	5043(1)	9303(2)	34(1)
C(28)	5536(3)	3983(2)	9379(2)	34(1)
C(31)	4027(2)	5897(1)	6943(2)	16(1)
C(32)	2941(2)	5737(1)	6684(2)	18(1)
C(33)	2209(2)	6064(1)	6227(2)	20(1)
C(34)	2527(3)	6565(1)	5995(2)	20(1)
C(35)	3593(3)	6730(1)	6244(2)	20(1)
C(36)	4317(2)	6404(1)	6708(2)	18(1)
C(37)	1048(3)	5896(1)	5987(2)	28(1)
C(38)	3961(3)	7278(1)	6059(2)	27(1)
C(41)	5903(2)	5852(1)	7944(2)	16(1)
C(42)	5646(2)	6300(1)	8369(2)	16(1)
C(43)	6443(3)	6578(1)	8846(2)	18(1)
C(44)	7538(3)	6416(1)	8928(2)	21(1)
C(45)	7803(2)	5970(1)	8530(2)	21(1)
C(46)	7001(2)	5693(1)	8047(2)	18(1)
C(47)	6144(3)	7057(1)	9284(2)	29(1)
C(48)	8973(3)	5785(1)	8591(2)	30(1)
C(51)	5449(2)	5156(1)	6760(2)	16(1)
C(52)	6209(2)	5385(1)	6316(2)	19(1)
C(53)	6631(3)	5113(1)	5725(2)	22(1)
C(54)	6297(3)	4598(1)	5537(2)	24(1)
C(55)	5539(3)	4362(1)	5956(2)	23(1)
C(56)	5131(2)	4634(1)	6557(2)	19(1)
C(57)	7506(3)	5362(2)	5314(2)	29(1)
C(58)	5177(3)	3804(2)	5773(2)	37(1)
B(21)	4935(3)	5504(1)	7424(2)	15(1)

Table S44. Bond lengths [Å] and angles [°] for **5a-B$_{Ar}$F**.

Co(1)-O(1)	2.004(3)	C(9A)-C(10A)	1.50(2)
Co(1)-N(2A)	2.04(3)	C(9A)-H(9AA)	0.9900
Co(1)-N(4A)	2.09(3)	C(9A)-H(9AC)	0.9900
Co(1)-N(4)	2.115(3)	C(10A)-H(10C)	0.9900
Co(1)-N(2)	2.118(3)	C(10A)-H(10D)	0.9900
Co(1)-N(1)	2.167(4)	C(11A)-H(11D)	0.9800
Co(1)-N(1A)	2.18(3)	C(11A)-H(11E)	0.9800
Co(1)-N(3)	2.213(3)	C(11A)-H(11F)	0.9800
Co(1)-N(3A)	2.29(3)	C(12A)-C(13A)	1.52(2)
N(1)-C(13)	1.475(7)	C(12A)-H(12C)	0.9900
N(1)-C(1)	1.484(6)	C(12A)-H(12D)	0.9900
N(1)-C(14)	1.498(6)	C(13A)-H(13C)	0.9900
N(2)-C(4)	1.481(5)	C(13A)-H(13D)	0.9900
N(2)-C(5)	1.488(6)	C(14A)-H(14D)	0.9800
N(2)-C(3)	1.489(5)	C(14A)-H(14E)	0.9800
N(3)-C(7)	1.471(6)	C(14A)-H(14F)	0.9800
N(3)-C(6)	1.481(5)	S(1)-O(2)	1.424(3)
N(3)-C(8)	1.501(5)	S(1)-O(3)	1.426(3)
N(4)-C(10)	1.489(6)	S(1)-O(1)	1.469(3)
N(4)-C(12)	1.490(6)	S(1)-C(15)	1.829(4)
N(4)-C(11)	1.506(5)	F(1)-C(15)	1.327(5)
C(1)-C(2)	1.502(8)	F(2)-C(15)	1.324(5)
C(1)-H(1A)	0.9900	F(3)-C(15)	1.319(5)
C(1)-H(1AB)	0.9900	F(21)-C(27)	1.324(5)
C(2)-C(3)	1.514(7)	F(22)-C(27)	1.377(6)
C(2)-H(2A)	0.9900	F(23)-C(27)	1.332(5)
C(2)-H(2AB)	0.9900	F(21A)-C(27)	1.314(12)
C(3)-H(3A)	0.9900	F(22A)-C(27)	1.272(11)
C(3)-H(3AB)	0.9900	F(23A)-C(27)	1.401(12)
C(4)-H(4A)	0.9800	F(24)-C(28)	1.337(5)
C(4)-H(4B)	0.9800	F(25)-C(28)	1.371(5)
C(4)-H(4C)	0.9800	F(26)-C(28)	1.283(5)
C(5)-C(6)	1.495(6)	F(24A)-C(28)	1.258(11)
C(5)-H(5A)	0.9900	F(25A)-C(28)	1.330(10)
C(5)-H(5AB)	0.9900	F(26A)-C(28)	1.462(9)
C(6)-H(6A)	0.9900	F(31)-C(37)	1.315(5)
C(6)-H(6AB)	0.9900	F(32)-C(37)	1.333(5)
C(7)-H(7A)	0.9800	F(33)-C(37)	1.347(5)
C(7)-H(7B)	0.9800	F(31A)-C(37)	1.331(10)
C(7)-H(7C)	0.9800	F(32A)-C(37)	1.296(10)
C(8)-C(9)	1.518(7)	F(33A)-C(37)	1.335(11)
C(8)-H(8A)	0.9900	F(34)-C(38)	1.324(4)
C(8)-H(8AB)	0.9900	F(35)-C(38)	1.340(4)
C(9)-C(10)	1.505(8)	F(36)-C(38)	1.328(4)
C(9)-H(9A)	0.9900	F(41)-C(47)	1.297(6)
C(9)-H(9AB)	0.9900	F(42)-C(47)	1.333(5)
C(10)-H(10A)	0.9900	F(43)-C(47)	1.386(5)
C(10)-H(10B)	0.9900	F(41A)-C(47)	1.346(11)
C(11)-H(11A)	0.9800	F(42A)-C(47)	1.250(9)
C(11)-H(11B)	0.9800	F(43A)-C(47)	1.411(10)
C(11)-H(11C)	0.9800	F(44)-C(48)	1.318(5)
C(12)-C(13)	1.513(9)	F(45)-C(48)	1.333(4)
C(12)-H(12A)	0.9900	F(46)-C(48)	1.335(4)
C(12)-H(12B)	0.9900	F(44A)-C(48)	1.353(12)
C(13)-H(13A)	0.9900	F(45A)-C(48)	1.331(12)
C(13)-H(13B)	0.9900	F(46A)-C(48)	1.333(12)
C(14)-H(14A)	0.9800	F(51)-C(57)	1.332(5)
C(14)-H(14B)	0.9800	F(52)-C(57)	1.339(4)
C(14)-H(14C)	0.9800	F(53)-C(57)	1.331(5)
N(1A)-C(13A)	1.46(2)	F(54)-C(58)	1.258(7)
N(1A)-C(14A)	1.48(2)	F(55)-C(58)	1.426(7)
N(1A)-C(1A)	1.48(2)	F(56)-C(58)	1.344(8)
N(2A)-C(3A)	1.471(19)	F(54A)-C(58)	1.248(7)
N(2A)-C(4A)	1.477(19)	F(55A)-C(58)	1.361(9)
N(2A)-C(5A)	1.48(2)	F(56A)-C(58)	1.411(7)
N(3A)-C(7A)	1.449(19)	F(54B)-C(58)	1.399(8)
N(3A)-C(6A)	1.48(2)	F(55B)-C(58)	1.258(8)
N(3A)-C(8A)	1.49(2)	F(56B)-C(58)	1.356(8)
N(4A)-C(12A)	1.49(2)	C(21)-C(22)	1.397(4)
N(4A)-C(10A)	1.50(2)	C(21)-C(26)	1.403(4)
N(4A)-C(11A)	1.53(2)	C(21)-B(21)	1.640(4)
C(1A)-C(2A)	1.51(2)	C(22)-C(23)	1.403(4)
C(1A)-H(1AA)	0.9900	C(22)-H(22)	0.9500
C(1A)-H(1AC)	0.9900	C(23)-C(24)	1.380(4)
C(2A)-C(3A)	1.52(2)	C(23)-C(27)	1.497(4)
C(2A)-H(2AA)	0.9900	C(24)-C(25)	1.383(4)
C(2A)-H(2AC)	0.9900	C(24)-H(24)	0.9500
C(3A)-H(3AA)	0.9900	C(25)-C(26)	1.390(4)
C(3A)-H(3AC)	0.9900	C(25)-C(28)	1.500(4)
C(4A)-H(4AA)	0.9800	C(26)-H(26)	0.9500
C(4A)-H(4AB)	0.9800	C(31)-C(36)	1.399(4)
C(4A)-H(4AC)	0.9800	C(31)-C(32)	1.408(4)
C(5A)-C(6A)	1.51(2)	C(31)-B(21)	1.636(4)
C(5A)-H(5AA)	0.9900	C(32)-C(33)	1.390(4)
C(5A)-H(5AC)	0.9900	C(32)-H(32)	0.9500
C(6A)-H(6AA)	0.9900	C(33)-C(34)	1.394(4)
C(6A)-H(6AC)	0.9900	C(33)-C(37)	1.492(4)
C(7A)-H(7AA)	0.9800	C(34)-C(35)	1.386(4)
C(7A)-H(7AB)	0.9800	C(34)-H(34)	0.9500
C(7A)-H(7AC)	0.9800	C(35)-C(36)	1.390(4)
C(8A)-C(9A)	1.51(2)	C(35)-C(38)	1.499(4)
C(8A)-H(8AA)	0.9900	C(36)-H(36)	0.9500
C(8A)-H(8AC)	0.9900	C(41)-C(46)	1.393(4)

C(41)-C(42)	1.406(4)
C(41)-B(21)	1.648(4)
C(42)-C(43)	1.387(4)
C(42)-H(42)	0.9500
C(43)-C(44)	1.393(4)
C(43)-C(47)	1.495(4)
C(44)-C(45)	1.378(4)
C(44)-H(44)	0.9500
C(45)-C(46)	1.395(4)
C(45)-C(48)	1.498(4)
C(46)-H(46)	0.9500
C(51)-C(56)	1.401(4)
C(51)-C(52)	1.407(4)
C(51)-B(21)	1.638(4)
C(52)-C(53)	1.389(4)
C(52)-H(52)	0.9500
C(53)-C(54)	1.386(5)
C(53)-C(57)	1.499(4)
C(54)-C(55)	1.385(5)
C(54)-H(54)	0.9500
C(55)-C(56)	1.393(4)
C(55)-C(58)	1.495(5)
C(56)-H(56)	0.9500

O(1)-Co(1)-N(2A)	79.3(10)
O(1)-Co(1)-N(4A)	109.2(10)
N(2A)-Co(1)-N(4A)	170.8(14)
O(1)-Co(1)-N(4)	113.92(12)
O(1)-Co(1)-N(2)	104.21(12)
N(4)-Co(1)-N(2)	141.82(13)
O(1)-Co(1)-N(1)	95.65(13)
N(4)-Co(1)-N(1)	85.08(16)
N(2)-Co(1)-N(1)	93.35(16)
O(1)-Co(1)-N(1A)	102.6(8)
N(2A)-Co(1)-N(1A)	95.2(10)
N(4A)-Co(1)-N(1A)	86.5(10)
O(1)-Co(1)-N(3)	93.00(13)
N(4)-Co(1)-N(3)	93.16(15)
N(2)-Co(1)-N(3)	82.65(13)
N(1)-Co(1)-N(3)	171.14(14)
O(1)-Co(1)-N(3A)	116.3(8)
N(2A)-Co(1)-N(3A)	81.7(10)
N(4A)-Co(1)-N(3A)	91.1(10)
N(1A)-Co(1)-N(3A)	139.5(11)
C(13)-N(1)-C(1)	109.3(4)
C(13)-N(1)-C(14)	107.3(4)
C(1)-N(1)-C(14)	107.9(4)
C(13)-N(1)-Co(1)	103.3(3)
C(1)-N(1)-Co(1)	111.4(3)
C(14)-N(1)-Co(1)	117.3(3)
C(4)-N(2)-C(5)	108.3(4)
C(4)-N(2)-C(3)	109.6(3)
C(5)-N(2)-C(3)	107.6(3)
C(4)-N(2)-Co(1)	108.8(2)
C(5)-N(2)-Co(1)	108.0(2)
C(3)-N(2)-Co(1)	114.5(3)
C(7)-N(3)-C(6)	107.6(4)
C(7)-N(3)-C(8)	108.7(3)
C(6)-N(3)-C(8)	108.2(3)
C(7)-N(3)-Co(1)	116.3(3)
C(6)-N(3)-Co(1)	104.8(2)
C(8)-N(3)-Co(1)	110.8(3)
C(10)-N(4)-C(12)	109.1(4)
C(10)-N(4)-C(11)	109.4(4)
C(12)-N(4)-C(11)	108.7(4)
C(10)-N(4)-Co(1)	114.1(3)
C(12)-N(4)-Co(1)	106.4(3)
C(11)-N(4)-Co(1)	109.0(2)
N(1)-C(1)-C(2)	115.5(4)
N(1)-C(1)-H(1A)	108.4
C(2)-C(1)-H(1A)	108.4
N(1)-C(1)-H(1AB)	108.4
C(2)-C(1)-H(1AB)	108.4
H(1A)-C(1)-H(1AB)	107.5
C(1)-C(2)-C(3)	115.5(4)
C(1)-C(2)-H(2A)	108.4
C(3)-C(2)-H(2A)	108.4
C(1)-C(2)-H(2AB)	108.4
C(3)-C(2)-H(2AB)	108.4
H(2A)-C(2)-H(2AB)	107.5
N(2)-C(3)-C(2)	115.1(4)
N(2)-C(3)-H(3A)	108.5
C(2)-C(3)-H(3A)	108.5
N(2)-C(3)-H(3AB)	108.5
C(2)-C(3)-H(3AB)	108.5
H(3A)-C(3)-H(3AB)	107.5
N(2)-C(4)-H(4A)	109.5
N(2)-C(4)-H(4B)	109.5
H(4A)-C(4)-H(4B)	109.5
N(2)-C(4)-H(4C)	109.5
H(4A)-C(4)-H(4C)	109.5
H(4B)-C(4)-H(4C)	109.5
N(2)-C(5)-C(6)	111.2(3)
N(2)-C(5)-H(5A)	109.4
C(6)-C(5)-H(5A)	109.4
N(2)-C(5)-H(5AB)	109.4
C(6)-C(5)-H(5AB)	109.4
H(5A)-C(5)-H(5AB)	108.0
N(3)-C(6)-C(5)	110.7(3)
N(3)-C(6)-H(6A)	109.5
C(5)-C(6)-H(6A)	109.5
N(3)-C(6)-H(6AB)	109.5
C(5)-C(6)-H(6AB)	109.5
H(6A)-C(6)-H(6AB)	108.1
N(3)-C(7)-H(7A)	109.5
N(3)-C(7)-H(7B)	109.5
H(7A)-C(7)-H(7B)	109.5
N(3)-C(7)-H(7C)	109.5
H(7A)-C(7)-H(7C)	109.5
H(7B)-C(7)-H(7C)	109.5
N(3)-C(8)-C(9)	115.3(3)
N(3)-C(8)-H(8A)	108.5
C(9)-C(8)-H(8A)	108.5
N(3)-C(8)-H(8AB)	108.5
C(9)-C(8)-H(8AB)	108.5
H(8A)-C(8)-H(8AB)	107.5
C(10)-C(9)-C(8)	114.8(4)
C(10)-C(9)-H(9A)	108.6
C(8)-C(9)-H(9A)	108.6
C(10)-C(9)-H(9AB)	108.6
C(8)-C(9)-H(9AB)	108.6
H(9A)-C(9)-H(9AB)	107.6
N(4)-C(10)-C(9)	115.1(4)
N(4)-C(10)-H(10A)	108.5
C(9)-C(10)-H(10A)	108.5
N(4)-C(10)-H(10B)	108.5
C(9)-C(10)-H(10B)	108.5
H(10A)-C(10)-H(10B)	107.5
N(4)-C(11)-H(11A)	109.5
N(4)-C(11)-H(11B)	109.5
H(11A)-C(11)-H(11B)	109.5
N(4)-C(11)-H(11C)	109.5
H(11A)-C(11)-H(11C)	109.5
H(11B)-C(11)-H(11C)	109.5
N(4)-C(12)-C(13)	111.2(4)
N(4)-C(12)-H(12A)	109.4
C(13)-C(12)-H(12A)	109.4
N(4)-C(12)-H(12B)	109.4
C(13)-C(12)-H(12B)	109.4
H(12A)-C(12)-H(12B)	108.0
N(1)-C(13)-C(12)	111.9(4)
N(1)-C(13)-H(13A)	109.2
C(12)-C(13)-H(13A)	109.2
N(1)-C(13)-H(13B)	109.2
C(12)-C(13)-H(13B)	109.2
H(13A)-C(13)-H(13B)	107.9
N(1)-C(14)-H(14A)	109.5
N(1)-C(14)-H(14B)	109.5
H(14A)-C(14)-H(14B)	109.5
N(1)-C(14)-H(14C)	109.5
H(14A)-C(14)-H(14C)	109.5
H(14B)-C(14)-H(14C)	109.5
C(13A)-N(1A)-C(14A)	111(2)
C(13A)-N(1A)-C(1A)	112(2)
C(14A)-N(1A)-C(1A)	113(2)
C(13A)-N(1A)-Co(1)	100.6(19)
C(14A)-N(1A)-Co(1)	113(2)
C(1A)-N(1A)-Co(1)	106(2)
C(3A)-N(2A)-C(4A)	112(3)
C(3A)-N(2A)-C(5A)	115(2)
C(4A)-N(2A)-C(5A)	106(2)
C(3A)-N(2A)-Co(1)	105(2)
C(4A)-N(2A)-Co(1)	124(3)
C(5A)-N(2A)-Co(1)	95(2)
C(7A)-N(3A)-C(6A)	111(3)
C(7A)-N(3A)-C(8A)	113(3)
C(6A)-N(3A)-C(8A)	109(2)
C(7A)-N(3A)-Co(1)	112(2)
C(6A)-N(3A)-Co(1)	101.8(16)
C(8A)-N(3A)-Co(1)	109(2)
C(12A)-N(4A)-C(10A)	108(2)
C(12A)-N(4A)-C(11A)	106(2)
C(10A)-N(4A)-C(11A)	106(2)
C(12A)-N(4A)-Co(1)	100.7(19)
C(10A)-N(4A)-Co(1)	119(2)
C(11A)-N(4A)-Co(1)	116(2)
N(1A)-C(1A)-C(2A)	115(2)
N(1A)-C(1A)-H(1AA)	108.4
C(2A)-C(1A)-H(1AA)	108.4
N(1A)-C(1A)-H(1AC)	108.4
C(2A)-C(1A)-H(1AC)	108.4
H(1AA)-C(1A)-H(1AC)	107.5
C(1A)-C(2A)-C(3A)	113(2)
C(1A)-C(2A)-H(2AA)	109.0
C(3A)-C(2A)-H(2AA)	109.0
C(1A)-C(2A)-H(2AC)	109.0
C(3A)-C(2A)-H(2AC)	109.0
H(2AA)-C(2A)-H(2AC)	107.8
N(2A)-C(3A)-C(2A)	115(2)
N(2A)-C(3A)-H(3AA)	108.5

C(2A)-C(3A)-H(3AA)	108.5	C(24)-C(25)-C(26)	120.9(3)
N(2A)-C(3A)-H(3AC)	108.5	C(24)-C(25)-C(28)	119.5(3)
C(2A)-C(3A)-H(3AC)	108.5	C(26)-C(25)-C(28)	119.5(3)
H(3AA)-C(3A)-H(3AC)	107.5	C(25)-C(26)-C(21)	122.5(3)
N(2A)-C(4A)-H(4AA)	109.5	C(25)-C(26)-H(26)	118.7
N(2A)-C(4A)-H(4AB)	109.5	C(21)-C(26)-H(26)	118.7
H(4AA)-C(4A)-H(4AB)	109.5	F(22A)-C(27)-F(21A)	115.6(16)
N(2A)-C(4A)-H(4AC)	109.5	F(21)-C(27)-F(23)	107.5(4)
H(4AA)-C(4A)-H(4AC)	109.5	F(21)-C(27)-F(22)	106.2(4)
H(4AB)-C(4A)-H(4AC)	109.5	F(23)-C(27)-F(22)	104.2(4)
N(2A)-C(5A)-C(6A)	108(2)	F(22A)-C(27)-F(23A)	101.4(11)
N(2A)-C(5A)-H(5AA)	110.1	F(21A)-C(27)-F(23A)	104.2(14)
C(6A)-C(5A)-H(5AA)	110.1	F(22A)-C(27)-C(23)	114.8(9)
N(2A)-C(5A)-H(5AC)	110.1	F(21A)-C(27)-C(23)	114.8(15)
C(6A)-C(5A)-H(5AC)	110.1	F(21)-C(27)-C(23)	113.6(4)
H(5AA)-C(5A)-H(5AC)	108.4	F(23)-C(27)-C(23)	114.4(3)
N(3A)-C(6A)-C(5A)	109(2)	F(22)-C(27)-C(23)	110.2(3)
N(3A)-C(6A)-H(6AA)	110.0	F(23A)-C(27)-C(23)	103.6(8)
C(5A)-C(6A)-H(6AA)	110.0	F(24A)-C(28)-F(25A)	115.4(10)
N(3A)-C(6A)-H(6AC)	110.0	F(26)-C(28)-F(24)	108.8(4)
C(5A)-C(6A)-H(6AC)	110.0	F(26)-C(28)-F(25)	108.6(4)
H(6AA)-C(6A)-H(6AC)	108.4	F(24)-C(28)-F(25)	102.5(4)
N(3A)-C(7A)-H(7AA)	109.5	F(24A)-C(28)-F(26A)	99.9(10)
N(3A)-C(7A)-H(7AB)	109.5	F(25A)-C(28)-F(26A)	96.0(9)
H(7AA)-C(7A)-H(7AB)	109.5	F(24A)-C(28)-C(25)	122.1(10)
N(3A)-C(7A)-H(7AC)	109.5	F(26)-C(28)-C(25)	113.2(3)
H(7AA)-C(7A)-H(7AC)	109.5	F(25A)-C(28)-C(25)	113.4(6)
H(7AB)-C(7A)-H(7AC)	109.5	F(24)-C(28)-C(25)	112.4(3)
N(3A)-C(8A)-C(9A)	115(3)	F(25)-C(28)-C(25)	110.7(3)
N(3A)-C(8A)-H(8AA)	108.6	F(26A)-C(28)-C(25)	104.0(5)
C(9A)-C(8A)-H(8AA)	108.6	C(36)-C(31)-C(32)	115.6(3)
N(3A)-C(8A)-H(8AC)	108.6	C(36)-C(31)-B(21)	121.2(3)
C(9A)-C(8A)-H(8AC)	108.6	C(32)-C(31)-B(21)	123.0(3)
H(8AA)-C(8A)-H(8AC)	107.6	C(33)-C(32)-C(31)	121.9(3)
C(10A)-C(9A)-C(8A)	116(3)	C(33)-C(32)-H(32)	119.0
C(10A)-C(9A)-H(9AA)	108.3	C(31)-C(32)-H(32)	119.0
C(8A)-C(9A)-H(9AA)	108.3	C(32)-C(33)-C(34)	121.1(3)
C(10A)-C(9A)-H(9AC)	108.3	C(32)-C(33)-C(37)	121.0(3)
C(8A)-C(9A)-H(9AC)	108.3	C(34)-C(33)-C(37)	117.9(3)
H(9AA)-C(9A)-H(9AC)	107.4	C(35)-C(34)-C(33)	117.8(3)
N(4A)-C(10A)-C(9A)	114(3)	C(35)-C(34)-H(34)	121.1
N(4A)-C(10A)-H(10C)	108.7	C(33)-C(34)-H(34)	121.1
C(9A)-C(10A)-H(10C)	108.7	C(34)-C(35)-C(36)	120.8(3)
N(4A)-C(10A)-H(10D)	108.7	C(34)-C(35)-C(38)	120.4(3)
C(9A)-C(10A)-H(10D)	108.7	C(36)-C(35)-C(38)	118.7(3)
H(10C)-C(10A)-H(10D)	107.6	C(35)-C(36)-C(31)	122.7(3)
N(4A)-C(11A)-H(11D)	109.5	C(35)-C(36)-H(36)	118.6
N(4A)-C(11A)-H(11E)	109.5	C(31)-C(36)-H(36)	118.6
H(11D)-C(11A)-H(11E)	109.5	F(32A)-C(37)-F(31A)	104.9(10)
N(4A)-C(11A)-H(11F)	109.5	F(31)-C(37)-F(32)	106.4(4)
H(11D)-C(11A)-H(11F)	109.5	F(32A)-C(37)-F(33A)	106.8(11)
H(11E)-C(11A)-H(11F)	109.5	F(31A)-C(37)-F(33A)	104.6(12)
N(4A)-C(12A)-C(13A)	110(2)	F(31)-C(37)-F(33)	106.4(4)
N(4A)-C(12A)-H(12C)	109.8	F(32)-C(37)-F(33)	104.2(4)
C(13A)-C(12A)-H(12C)	109.8	F(32A)-C(37)-C(33)	116.1(8)
N(4A)-C(12A)-H(12D)	109.8	F(31)-C(37)-C(33)	114.3(3)
C(13A)-C(12A)-H(12D)	109.8	F(31A)-C(37)-C(33)	111.7(8)
H(12C)-C(12A)-H(12D)	108.2	F(32)-C(37)-C(33)	113.3(3)
N(1A)-C(13A)-C(12A)	108(2)	F(33A)-C(37)-C(33)	112.0(9)
N(1A)-C(13A)-H(13C)	110.1	F(33)-C(37)-C(33)	111.6(3)
C(12A)-C(13A)-H(13C)	110.1	F(34)-C(38)-F(36)	106.6(3)
N(1A)-C(13A)-H(13D)	110.1	F(34)-C(38)-F(35)	106.9(3)
C(12A)-C(13A)-H(13D)	110.1	F(36)-C(38)-F(35)	105.7(3)
H(13C)-C(13A)-H(13D)	108.4	F(34)-C(38)-C(35)	113.4(3)
N(1A)-C(14A)-H(14D)	109.5	F(36)-C(38)-C(35)	112.3(3)
N(1A)-C(14A)-H(14E)	109.5	F(35)-C(38)-C(35)	111.4(3)
H(14D)-C(14A)-H(14E)	109.5	C(46)-C(41)-C(42)	116.1(3)
N(1A)-C(14A)-H(14F)	109.5	C(46)-C(41)-B(21)	122.3(3)
H(14D)-C(14A)-H(14F)	109.5	C(42)-C(41)-B(21)	121.4(3)
H(14E)-C(14A)-H(14F)	109.5	C(43)-C(42)-C(41)	122.1(3)
O(2)-S(1)-O(3)	116.4(2)	C(43)-C(42)-H(42)	119.0
O(2)-S(1)-O(1)	114.10(18)	C(41)-C(42)-H(42)	119.0
O(3)-S(1)-O(1)	112.74(17)	C(42)-C(43)-C(44)	120.6(3)
O(2)-S(1)-C(15)	105.47(19)	C(42)-C(43)-C(47)	120.8(3)
O(3)-S(1)-C(15)	105.39(19)	C(44)-C(43)-C(47)	118.7(3)
O(1)-S(1)-C(15)	100.75(18)	C(45)-C(44)-C(43)	118.2(3)
S(1)-O(1)-Co(1)	140.69(18)	C(45)-C(44)-H(44)	120.9
F(3)-C(15)-F(2)	108.8(3)	C(43)-C(44)-H(44)	120.9
F(3)-C(15)-F(1)	108.2(4)	C(44)-C(45)-C(46)	121.1(3)
F(2)-C(15)-F(1)	108.6(3)	C(44)-C(45)-C(48)	120.0(3)
F(3)-C(15)-S(1)	110.7(3)	C(46)-C(45)-C(48)	118.8(3)
F(2)-C(15)-S(1)	109.6(3)	C(41)-C(46)-C(45)	121.9(3)
F(1)-C(15)-S(1)	111.0(3)	C(41)-C(46)-H(46)	119.1
C(22)-C(21)-C(26)	115.7(3)	C(45)-C(46)-H(46)	119.1
C(22)-C(21)-B(21)	124.5(3)	F(41)-C(47)-F(42)	111.6(5)
C(26)-C(21)-B(21)	119.3(3)	F(42A)-C(47)-F(41A)	110.9(13)
C(21)-C(22)-C(23)	121.6(3)	F(41)-C(47)-F(43)	105.6(5)
C(21)-C(22)-H(22)	119.2	F(42)-C(47)-F(43)	101.4(3)
C(23)-C(22)-H(22)	119.2	F(42A)-C(47)-F(43A)	105.1(10)
C(24)-C(23)-C(22)	121.4(3)	F(41A)-C(47)-F(43A)	96.2(12)
C(24)-C(23)-C(27)	119.0(3)	F(42A)-C(47)-C(43)	121.5(7)
C(22)-C(23)-C(27)	119.5(3)	F(41)-C(47)-C(43)	115.8(5)
C(23)-C(24)-C(25)	117.8(3)	F(42)-C(47)-C(43)	112.7(3)
C(23)-C(24)-H(24)	121.1	F(41A)-C(47)-C(43)	111.9(12)
C(25)-C(24)-H(24)	121.1	F(43)-C(47)-C(43)	108.4(3)

F(43A)-C(47)-C(43)	107.9(6)	F(53)-C(57)-F(51)	105.3(3)
F(44)-C(48)-F(45)	107.1(3)	F(53)-C(57)-F(52)	106.5(3)
F(45A)-C(48)-F(46A)	105.4(15)	F(51)-C(57)-F(52)	106.3(3)
F(44)-C(48)-F(46)	107.5(4)	F(53)-C(57)-C(53)	112.0(3)
F(45)-C(48)-F(46)	105.5(3)	F(51)-C(57)-C(53)	113.7(3)
F(45A)-C(48)-F(44A)	102.3(15)	F(52)-C(57)-C(53)	112.5(3)
F(46A)-C(48)-F(44A)	100.2(15)	F(54)-C(58)-F(56)	108.2(5)
F(44)-C(48)-C(45)	112.0(3)	F(55B)-C(58)-F(56B)	105.8(6)
F(45A)-C(48)-C(45)	116.2(11)	F(54A)-C(58)-F(55A)	112.3(6)
F(45)-C(48)-C(45)	112.4(3)	F(55B)-C(58)-F(54B)	107.3(6)
F(46A)-C(48)-C(45)	115.8(12)	F(56B)-C(58)-F(54B)	99.0(6)
F(46)-C(48)-C(45)	112.0(3)	F(54A)-C(58)-F(56A)	109.4(6)
F(44A)-C(48)-C(45)	114.7(11)	F(55A)-C(58)-F(56A)	99.9(5)
C(56)-C(51)-C(52)	115.4(3)	F(54)-C(58)-F(55)	109.1(5)
C(56)-C(51)-B(21)	124.0(3)	F(56)-C(58)-F(55)	95.6(6)
C(52)-C(51)-B(21)	120.5(3)	F(54A)-C(58)-C(55)	116.4(4)
C(53)-C(52)-C(51)	122.6(3)	F(54)-C(58)-C(55)	117.1(4)
C(53)-C(52)-H(52)	118.7	F(55B)-C(58)-C(55)	117.4(5)
C(51)-C(52)-H(52)	118.7	F(56)-C(58)-C(55)	115.6(5)
C(54)-C(53)-C(52)	120.7(3)	F(56B)-C(58)-C(55)	113.6(5)
C(54)-C(53)-C(57)	119.1(3)	F(55A)-C(58)-C(55)	109.3(5)
C(52)-C(53)-C(57)	120.2(3)	F(54B)-C(58)-C(55)	111.9(4)
C(55)-C(54)-C(53)	118.2(3)	F(56A)-C(58)-C(55)	108.3(4)
C(55)-C(54)-H(54)	120.9	F(55)-C(58)-C(55)	109.0(4)
C(53)-C(54)-H(54)	120.9	C(31)-B(21)-C(51)	105.1(2)
C(54)-C(55)-C(56)	120.9(3)	C(31)-B(21)-C(21)	114.5(2)
C(54)-C(55)-C(58)	119.4(3)	C(51)-B(21)-C(21)	112.6(2)
C(56)-C(55)-C(58)	119.7(3)	C(31)-B(21)-C(41)	110.9(2)
C(55)-C(56)-C(51)	122.3(3)	C(51)-B(21)-C(41)	110.9(2)
C(55)-C(56)-H(56)	118.9	C(21)-B(21)-C(41)	103.0(2)
C(51)-C(56)-H(56)	118.9		

Table S45. Anisotropic displacement parameters ($Å^2$x 10^3) for **5a-B$_{Ar}$F**.
The anisotropic displacement factor exponent takes the form: $-2p^2[h^2 a^{*2}U^{11} + ... + 2 h k a^* b^* U^{12}]$

	U^{11}	U^{22}	U^{33}	U^{23}	U^{13}	U^{12}
Co(1)	21(1)	22(1)	21(1)	0(1)	3(1)	4(1)
N(1)	39(2)	28(2)	49(2)	10(2)	-6(2)	-9(2)
N(2)	31(2)	33(2)	24(2)	5(1)	10(1)	8(1)
N(3)	23(2)	43(2)	22(2)	1(1)	-1(1)	0(1)
N(4)	50(2)	28(2)	24(2)	-1(1)	7(1)	14(1)
C(1)	49(3)	33(2)	63(3)	19(2)	-8(2)	-13(2)
C(2)	37(2)	49(3)	61(3)	30(2)	11(2)	-2(2)
C(3)	42(2)	41(2)	32(2)	13(2)	14(2)	9(2)
C(4)	39(2)	41(2)	32(2)	5(2)	16(2)	15(2)
C(5)	40(2)	42(2)	22(2)	-4(2)	2(2)	8(2)
C(6)	40(2)	37(2)	27(2)	-8(2)	-3(2)	-5(2)
C(7)	33(3)	47(3)	42(2)	4(2)	1(2)	-10(2)
C(8)	23(2)	58(3)	31(2)	6(2)	2(2)	6(2)
C(9)	34(2)	67(3)	37(2)	7(2)	8(2)	22(2)
C(10)	54(3)	45(3)	32(2)	4(2)	14(2)	27(2)
C(11)	60(3)	45(2)	19(2)	-2(2)	7(2)	22(2)
C(12)	84(3)	26(2)	43(2)	-8(2)	2(2)	7(2)
C(13)	72(3)	28(2)	53(3)	-1(2)	-16(2)	-11(2)
C(14)	35(2)	45(3)	61(3)	15(2)	-16(2)	-17(2)
N(1A)	38(8)	42(7)	43(7)	12(6)	7(7)	0(6)
N(2A)	36(8)	46(8)	35(7)	-3(6)	3(6)	-3(6)
N(3A)	48(7)	47(7)	35(7)	3(7)	1(7)	17(6)
N(4A)	59(7)	33(7)	50(7)	1(7)	0(7)	-2(6)
C(1A)	42(8)	44(8)	37(8)	7(8)	5(7)	6(7)
C(2A)	47(9)	45(8)	37(8)	-2(8)	6(8)	4(8)
C(3A)	48(10)	44(9)	37(8)	-3(7)	4(7)	1(8)
C(4A)	33(3)	47(3)	42(2)	4(2)	1(2)	-10(2)
C(5A)	39(8)	56(8)	39(8)	-1(6)	4(7)	3(7)
C(6A)	42(8)	55(8)	39(8)	-1(7)	4(7)	11(7)
C(7A)	70(20)	64(19)	35(9)	4(11)	3(11)	8(19)
C(8A)	54(9)	43(8)	37(10)	-1(8)	13(9)	19(7)
C(9A)	69(8)	41(8)	43(9)	0(9)	7(8)	12(7)
C(10A)	68(8)	33(9)	45(10)	-6(9)	5(8)	0(7)
C(11A)	75(19)	32(18)	55(10)	-1(14)	-9(11)	-20(16)
C(12A)	49(8)	38(9)	58(8)	14(8)	0(7)	-2(8)
C(13A)	42(9)	41(7)	53(8)	17(8)	8(8)	-1(7)
C(14A)	44(14)	48(14)	60(20)	2(16)	-2(14)	7(13)
S(1)	29(1)	30(1)	25(1)	6(1)	-2(1)	2(1)
F(1)	47(2)	47(1)	54(2)	-8(1)	0(1)	10(1)
F(2)	66(2)	43(2)	57(2)	7(1)	-24(1)	18(1)
F(3)	28(1)	58(2)	107(3)	15(2)	12(1)	4(1)
O(1)	34(1)	37(1)	29(1)	10(1)	2(1)	8(1)
O(2)	49(2)	38(2)	44(2)	-3(1)	-12(1)	2(1)
O(3)	43(2)	46(2)	33(1)	8(1)	10(1)	-5(1)
C(15)	30(2)	33(2)	47(2)	6(2)	-5(2)	4(2)
F(21)	34(3)	34(2)	49(3)	8(2)	23(2)	16(2)
F(22)	21(2)	55(2)	101(4)	-6(2)	19(2)	-9(1)
F(23)	55(2)	61(3)	55(2)	31(2)	44(2)	22(2)
F(21A)	26(9)	48(8)	71(13)	23(9)	24(9)	13(7)
F(22A)	21(2)	55(2)	101(4)	-6(2)	19(2)	-9(1)
F(23A)	55(2)	61(3)	55(2)	31(2)	44(2)	22(2)
F(24)	35(2)	43(2)	25(1)	15(1)	2(1)	9(2)
F(25)	26(2)	80(3)	79(3)	54(2)	14(2)	20(2)
F(26)	108(4)	35(2)	40(2)	-10(2)	-10(2)	45(2)
F(24A)	42(10)	43(8)	62(5)	0(6)	-25(6)	13(8)
F(25A)	26(2)	80(3)	79(3)	54(2)	14(2)	20(2)
F(26A)	46(6)	18(4)	57(8)	0(5)	5(5)	11(4)
F(31)	26(2)	30(2)	74(3)	21(2)	-17(2)	-14(2)
F(32)	54(3)	96(5)	34(2)	17(2)	-23(2)	-39(3)
F(33)	18(2)	48(2)	109(5)	-21(3)	0(2)	-1(1)
F(31A)	41(7)	92(13)	72(9)	-51(9)	-14(7)	-8(8)
F(32A)	29(6)	38(6)	74(11)	8(6)	-26(7)	3(4)
F(33A)	26(7)	72(12)	77(10)	31(9)	-4(7)	-20(8)
F(34)	41(1)	36(1)	58(2)	30(1)	-12(1)	-8(1)
F(35)	112(3)	20(1)	49(2)	-3(1)	26(2)	-19(1)
F(36)	29(1)	31(1)	67(2)	22(1)	6(1)	-9(1)
F(41)	42(2)	37(4)	53(4)	-21(3)	-20(3)	23(2)
F(42)	57(3)	22(2)	53(3)	-15(2)	27(2)	-13(2)
F(43)	39(2)	37(2)	22(1)	-10(1)	4(1)	2(2)
F(41A)	27(4)	23(7)	42(7)	-8(5)	6(4)	10(4)
F(42A)	39(7)	66(10)	72(6)	-52(7)	-18(6)	19(7)
F(43A)	111(12)	18(4)	108(11)	-4(5)	83(10)	4(6)
F(44)	22(1)	82(3)	57(2)	2(2)	15(1)	2(1)
F(45)	25(1)	43(2)	48(2)	-12(1)	-12(1)	8(1)
F(46)	25(1)	31(1)	104(3)	-27(2)	-10(2)	11(1)
F(44A)	22(1)	82(3)	57(2)	2(2)	15(1)	2(1)
F(45A)	15(8)	47(11)	96(15)	-25(12)	-12(11)	1(8)
F(46A)	30(12)	44(9)	68(13)	12(10)	8(12)	11(8)
F(51)	60(2)	49(1)	42(1)	-4(1)	27(1)	-29(1)
F(52)	52(1)	57(2)	22(1)	0(1)	17(1)	-17(1)
F(53)	23(1)	99(2)	83(2)	53(2)	19(1)	6(1)
F(54)	47(2)	29(1)	51(2)	-17(1)	14(1)	-11(1)
F(55)	47(2)	29(1)	51(2)	-17(1)	14(1)	-11(1)
F(56)	47(2)	29(1)	51(2)	-17(1)	14(1)	-11(1)
F(54A)	47(2)	29(1)	51(2)	-17(1)	14(1)	-11(1)
F(55A)	47(2)	29(1)	51(2)	-17(1)	14(1)	-11(1)
F(56A)	47(2)	29(1)	51(2)	-17(1)	14(1)	-11(1)
F(54B)	47(2)	29(1)	51(2)	-17(1)	14(1)	-11(1)
F(55B)	47(2)	29(1)	51(2)	-17(1)	14(1)	-11(1)
F(56B)	47(2)	29(1)	51(2)	-17(1)	14(1)	-11(1)

C(21)	16(1)	14(1)	15(1)	1(1)	2(1)	-3(1)
C(22)	17(1)	14(1)	21(1)	2(1)	3(1)	-1(1)
C(23)	19(1)	16(1)	22(1)	1(1)	8(1)	-1(1)
C(24)	23(1)	18(1)	21(1)	3(1)	8(1)	-2(1)
C(25)	22(1)	17(1)	21(1)	4(1)	4(1)	1(1)
C(26)	16(1)	16(1)	21(1)	1(1)	5(1)	1(1)
C(27)	29(2)	32(2)	45(2)	11(2)	22(2)	6(1)
C(28)	34(2)	32(2)	38(2)	16(2)	14(2)	13(1)
C(31)	17(1)	16(1)	17(1)	2(1)	3(1)	-1(1)
C(32)	19(1)	16(1)	19(1)	2(1)	1(1)	-3(1)
C(33)	19(1)	19(1)	20(1)	1(1)	0(1)	-2(1)
C(34)	21(1)	18(1)	20(1)	3(1)	0(1)	2(1)
C(35)	23(1)	15(1)	22(1)	4(1)	4(1)	-2(1)
C(36)	17(1)	19(1)	19(1)	3(1)	1(1)	-3(1)
C(37)	23(2)	26(2)	34(2)	4(1)	-6(1)	-2(1)
C(38)	29(2)	20(2)	31(2)	9(1)	2(1)	-3(1)
C(41)	18(1)	14(1)	15(1)	5(1)	2(1)	-2(1)
C(42)	17(1)	17(1)	16(1)	3(1)	4(1)	0(1)
C(43)	21(1)	17(1)	18(1)	1(1)	4(1)	-1(1)
C(44)	21(1)	20(1)	20(1)	-1(1)	-2(1)	-2(1)
C(45)	17(1)	20(1)	25(2)	0(1)	0(1)	0(1)
C(46)	20(1)	14(1)	21(1)	0(1)	3(1)	0(1)
C(47)	26(2)	27(2)	33(2)	-9(1)	8(1)	-4(1)
C(48)	19(2)	30(2)	40(2)	-8(1)	-3(1)	1(1)
C(51)	14(1)	19(1)	15(1)	2(1)	0(1)	-2(1)
C(52)	18(1)	21(1)	17(1)	3(1)	1(1)	-4(1)
C(53)	19(1)	31(2)	17(1)	5(1)	3(1)	-4(1)
C(54)	24(2)	32(2)	18(1)	-3(1)	6(1)	-2(1)
C(55)	25(2)	24(2)	21(1)	-4(1)	4(1)	-4(1)
C(56)	17(1)	21(1)	18(1)	0(1)	4(1)	-5(1)
C(57)	26(2)	40(2)	23(2)	5(1)	9(1)	-5(1)
C(58)	43(2)	28(2)	41(2)	-13(1)	13(2)	-11(1)
B(21)	16(1)	14(1)	17(1)	3(1)	2(1)	-2(1)

Table S46. Hydrogen coordinates (x 10^4) and isotropic displacement parameters ($Å^2$x 10^3) for **5a-B$_{Ar}$F**.

	x	y	z	U(eq)
H(1A)	11696	9393	7123	60
H(1AB)	10393	9348	6906	60
H(2A)	11878	8636	6318	59
H(2AB)	11436	9160	5866	59
H(3A)	9616	8813	5775	45
H(3AB)	10415	8473	5308	45
H(4A)	11027	7633	5712	55
H(4B)	10647	7354	6461	55
H(4C)	11539	7821	6561	55
H(5A)	9147	7675	5346	42
H(5AB)	8444	8152	5644	42
H(6A)	7714	7355	6013	42
H(6AB)	8896	7165	6423	42
H(7A)	7289	7114	7335	62
H(7B)	7791	7468	8059	62
H(7C)	8567	7067	7662	62
H(8A)	6558	7858	6691	45
H(8AB)	7319	8320	6421	45
H(9A)	6189	8576	7399	55
H(9AB)	6934	8201	7993	55
H(10A)	7799	9085	7253	51
H(10B)	7399	9125	8090	51
H(11A)	8415	8110	8775	62
H(11B)	8310	8680	9168	62
H(11C)	9496	8418	9149	62
H(12A)	9370	9388	8716	62
H(12B)	9361	9461	7801	62
H(13A)	11188	9404	8371	64
H(13B)	10944	8859	8793	64
H(14A)	12378	8340	7426	74
H(14B)	12157	8310	8312	74
H(14C)	12687	8834	7992	74
H(1AA)	9970	8232	5225	49
H(1AC)	9051	8415	5743	49
H(2AA)	9564	7389	5306	51
H(2AC)	8390	7626	5436	51
H(3AA)	8949	6966	6361	52
H(3AC)	10040	7294	6649	52
H(4AA)	7848	7406	7922	62
H(4AB)	7696	6980	7235	62
H(4AC)	8821	7000	7813	62
H(5AA)	7189	7731	6378	54
H(5AC)	8016	8227	6383	54
H(6AA)	6614	8407	7186	55
H(6AC)	7003	7898	7707	55
H(7AA)	8676	8640	8956	87
H(7AB)	7386	8513	8812	87
H(7AC)	8262	8044	8781	87
H(8AA)	7221	9199	7768	53
H(8AC)	7880	9107	7044	53
H(9AA)	8535	9819	7924	61
H(9AC)	8986	9369	8528	61
H(10C)	9725	9502	7060	59
H(10D)	10364	9728	7851	59
H(11D)	10539	8942	8943	83
H(11E)	11559	8627	8672	83
H(11F)	11535	9263	8648	83
H(12C)	11940	8647	7417	59
H(12D)	11757	9271	7263	59
H(13C)	10267	9046	6253	54
H(13D)	11453	8859	6054	54
H(14D)	11180	7514	6363	75
H(14E)	11852	7949	5944	75
H(14F)	11954	7930	6870	75
H(22)	3016	5517	8186	21
H(24)	3625	4328	9762	24
H(26)	5754	4623	8214	21
H(32)	2701	5396	6825	22
H(34)	2029	6786	5676	24
H(36)	5040	6530	6872	22
H(42)	4903	6417	8328	20
H(44)	8088	6607	9248	25
H(46)	7210	5387	7781	22
H(52)	6442	5741	6425	23
H(54)	6582	4411	5131	29
H(56)	4619	4460	6838	22

[(D$_{12}$-TMC)Co^{II}(OTf)$_2$], 5b

5b was solved in the monoclinic space group *P*2$_1$/*n*. The asymmetric unit contains half of a molecule. The structure was found to be heavily disordered over three positions (ligand) and two positions (coordinated anion). The disorder was refined using same distance restraints on 1,2 and 1,3-distances, similarity restraints on the anisotropic displacement parameters and advanced rigid bond restraints. The disorder ratios were refined freely and converged to 0.531(3):0.328(3):0.141(3) and 0.535(4).

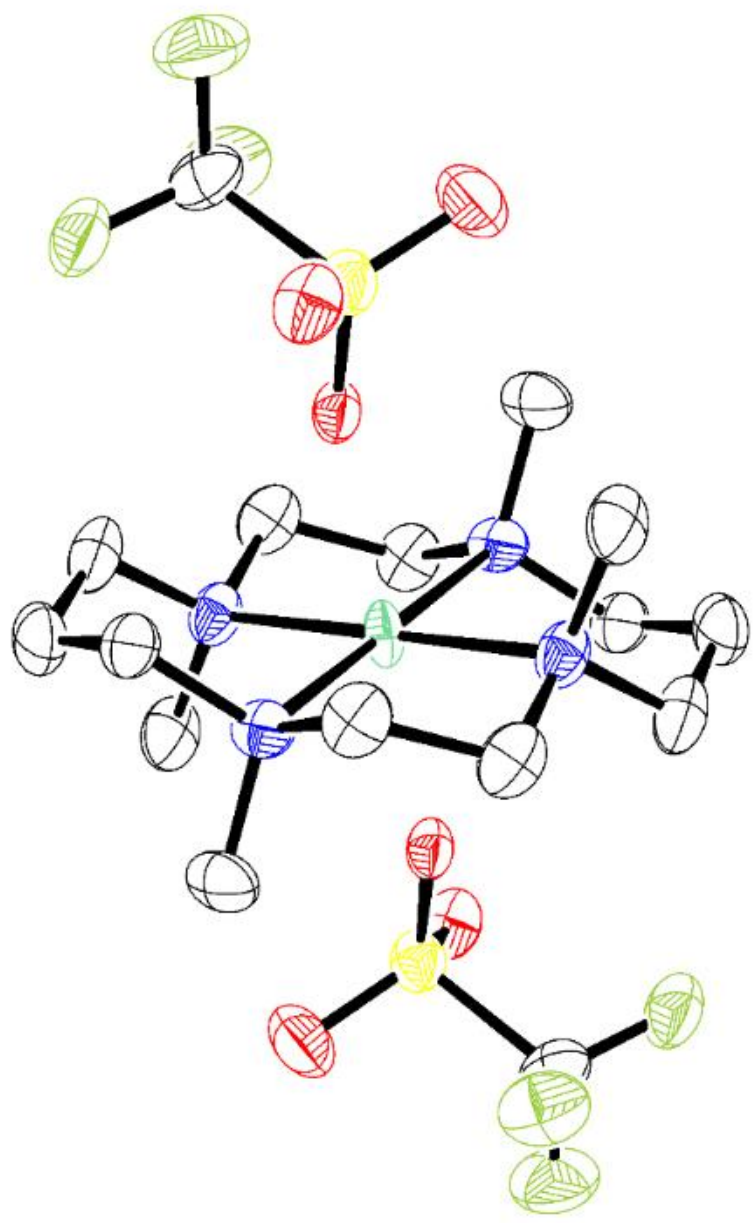

Figure S10. Molecular structure of **5b**.

Table S47. Crystal data and structure refinement for **5b**.

CCDC No	2062708	
Empirical formula	$C_{16}H_{20}CoD_{12}F_6N_4O_6S_2$	
Formula weight	625.58	
Temperature	100(2) K	
Wavelength	0.71073 Å	
Crystal system	Monoclinic	
Space group	*P*2$_1$/*n*	
Unit cell dimensions	a = 8.7388(3) Å	α = 90°
	b = 10.1713(4) Å	β = 101.885(2)°
	c = 13.8799(5) Å	γ = 90°
Volume	1207.27(8) Å^3	
Z	2	
Density (calculated)	1.721 Mg/m^3	
Absorption coefficient	0.971 mm^{-1}	
F(000)	634	
Crystal size	0.196 x 0.184 x 0.147 mm^3	
Theta range for data collection	2.502 to 30.032°.	
Index ranges	-12 ≤ h ≤ 12, -14 ≤ k ≤ 14, -19 ≤ l ≤ 19	
Reflections collected	87808	
Independent reflections	3535 [R(int) = 0.0640]	
Completeness to theta = 25.242°	100.0 %	
Absorption correction	Semi-empirical from equivalents	
Refinement method	Full-matrix least-squares on *F*^{2}	
Data / restraints / parameters	3535 / 1579 / 404	
Goodness-of-fit on *F*^{2}	1.152	
Final R indices [I>2σ(I)]	R1 = 0.0579, wR2 = 0.1469	
R indices (all data)	R1 = 0.0658, wR2 = 0.1521	
Largest diff. peak and hole	1.046 and -0.749 eÅ^{-3}	

Table S48. Atomic coordinates (x 10^4) and equivalent isotropic displacement parameters ($Å^2$x 10^3) for **5b**. U(eq) is defined as one third of the trace of the orthogonalized U^{ij} tensor.

	x	y	z	U(eq)
Co(1)	5000	5000	5000	28(1)
N(1)	3050(5)	3747(5)	4998(4)	33(1)
N(2)	5780(5)	4401(5)	6514(3)	34(1)
C(1)	3108(7)	3492(8)	6063(5)	40(1)
C(2)	4805(7)	3216(6)	6572(5)	41(1)
C(3)	5559(9)	5319(8)	7311(4)	47(2)
D(3A)	6171	6120	7281	71
D(3B)	5910	4895	7952	71
D(3C)	4449	5548	7224	71
C(4)	7456(6)	4029(7)	6695(5)	44(1)
C(12)	1461(7)	4868(7)	3505(5)	47(1)
C(13)	1588(6)	4451(6)	4565(5)	39(1)
C(14)	3024(7)	2445(6)	4494(6)	45(2)
D(14A)	3145	2578	3814	68
D(14B)	2026	2005	4492	68
D(14C)	3884	1899	4845	68
N(1A)	2748(8)	4624(8)	4124(6)	35(2)
N(2A)	4388(9)	3586(8)	6036(6)	37(2)
C(1A)	2264(11)	3389(9)	4540(7)	44(2)
C(2A)	2647(11)	3501(13)	5675(8)	44(2)
C(3A)	5004(15)	2232(10)	6032(9)	53(3)
D(3AA)	4815	1901	5355	79
D(3AB)	4474	1664	6431	79
D(3AC)	6130	2234	6309	79
C(4A)	4749(13)	4112(13)	7046(7)	46(2)
C(5A)	6511(13)	4422(13)	7397(8)	52(2)
C(6A)	7148(12)	5608(10)	6920(6)	41(2)
C(14A)	1510(10)	5620(11)	4127(8)	44(2)
D(14D)	583	5391	3627	66
D(14E)	1893	6487	3979	66
D(14F)	1236	5639	4777	66
N(1B)	3075(19)	5169(18)	3796(12)	44(3)
N(2B)	3610(20)	3746(17)	5672(12)	40(3)
C(1B)	2050(20)	4080(20)	3994(13)	49(3)
C(2B)	2020(20)	4020(20)	5105(13)	45(3)
C(3B)	3930(30)	2317(17)	5652(19)	47(4)
D(3BA)	2947	1835	5463	71
D(3BB)	4482	2030	6306	71
D(3BC)	4590	2142	5172	71
C(4B)	3710(20)	4160(20)	6713(13)	45(3)
C(12B)	4640(30)	5970(30)	2660(19)	64(7)
C(13B)	3460(30)	4910(30)	2816(15)	52(5)
C(14B)	2140(30)	6400(20)	3710(20)	64(6)
D(14G)	1810	6571	4335	95
D(14H)	1217	6315	3180	95
D(14I)	2782	7141	3568	95
S(1)	3052(4)	7643(4)	6117(2)	37(1)
F(1)	841(4)	8268(4)	4611(3)	52(1)
F(2)	1340(7)	9746(5)	5748(5)	69(2)
F(3)	2922(7)	9429(6)	4759(5)	60(2)
O(1)	3709(8)	6793(9)	5459(5)	31(1)
O(2)	1876(11)	6994(15)	6541(9)	43(2)
O(3)	4174(6)	8413(6)	6767(4)	59(2)
C(21)	1982(9)	8835(9)	5260(6)	44(2)
S(1A)	3231(5)	7430(4)	6403(3)	34(1)
F(1A)	2300(12)	9128(9)	4979(6)	80(3)
F(2A)	2307(8)	9912(5)	6404(6)	72(2)
F(3A)	4494(7)	9631(5)	5965(4)	64(2)
O(1A)	4056(9)	6763(10)	5744(5)	32(2)
O(2A)	1625(13)	7051(18)	6309(11)	59(4)
O(3A)	4116(7)	7618(6)	7379(4)	51(1)
C(21A)	3071(9)	9102(8)	5916(6)	53(2)

Table S49. Bond lengths [Å] and angles [°] for **5b**.

Co(1)-N(2B)	2.108(19)	F(3)-C(21)	1.326(8)
Co(1)-N(2B)#1	2.108(19)	S(1A)-O(3A)	1.427(6)
Co(1)-N(1B)	2.120(16)	S(1A)-O(2A)	1.435(9)
Co(1)-N(1B)#1	2.120(17)	S(1A)-O(1A)	1.446(8)
Co(1)-N(1A)#1	2.124(7)	S(1A)-C(21A)	1.825(9)
Co(1)-N(1A)	2.124(7)	F(1A)-C(21A)	1.337(9)
Co(1)-N(1)	2.128(4)	F(2A)-C(21A)	1.330(7)
Co(1)-N(1)#1	2.128(4)	F(3A)-C(21A)	1.343(9)
Co(1)-N(2)	2.160(4)		
Co(1)-N(2)#1	2.160(4)	N(2B)-Co(1)-N(1B)	87.8(6)
Co(1)-N(2A)#1	2.177(8)	N(2B)#1-Co(1)-N(1B)	92.2(6)
Co(1)-N(2A)	2.177(8)	N(1A)#1-Co(1)-N(1A)	180.0
N(1)-C(13)	1.480(6)	N(1)-Co(1)-N(1)#1	180.0
N(1)-C(1)	1.491(7)	N(1)-Co(1)-N(2)	85.67(19)
N(1)-C(14)	1.495(7)	N(1)#1-Co(1)-N(2)	94.33(19)
N(2)-C(4)	1.483(7)	N(1)-Co(1)-N(2)#1	94.33(19)
N(2)-C(2)	1.487(7)	N(1)#1-Co(1)-N(2)#1	85.67(19)
N(2)-C(3)	1.490(7)	N(2)-Co(1)-N(2)#1	180.0
C(1)-C(2)	1.532(8)	N(1)-Co(1)-N(2A)#1	134.5(2)
C(1)-H(1A)	0.9900	N(1)#1-Co(1)-N(2A)#1	45.5(2)
C(1)-H(1AB)	0.9900	N(2)-Co(1)-N(2A)#1	139.3(2)
C(2)-H(2A)	0.9900	N(2)#1-Co(1)-N(2A)#1	40.7(2)
C(2)-H(2AB)	0.9900	N(1A)#1-Co(1)-N(2A)	93.7(3)
C(3)-D(3A)	0.9800	N(1A)-Co(1)-N(2A)	86.3(3)
C(3)-D(3B)	0.9800	N(2A)#1-Co(1)-N(2A)	180.0
C(3)-D(3C)	0.9800	C(13)-N(1)-C(1)	109.7(4)
C(4)-C(12)#1	1.530(9)	C(13)-N(1)-C(14)	108.1(5)
C(4)-H(4A)	0.9900	C(1)-N(1)-C(14)	107.7(5)
C(4)-H(4AB)	0.9900	C(13)-N(1)-Co(1)	109.6(3)
C(12)-C(13)	1.513(9)	C(1)-N(1)-Co(1)	103.8(4)
C(12)-H(12A)	0.9900	C(14)-N(1)-Co(1)	117.8(3)
C(12)-H(12B)	0.9900	C(4)-N(2)-C(2)	109.9(5)
C(13)-H(13A)	0.9900	C(4)-N(2)-C(3)	108.0(5)
C(13)-H(13B)	0.9900	C(2)-N(2)-C(3)	107.8(5)
C(14)-D(14A)	0.9800	C(4)-N(2)-Co(1)	109.8(3)
C(14)-D(14B)	0.9800	C(2)-N(2)-Co(1)	102.2(3)
C(14)-D(14C)	0.9800	C(3)-N(2)-Co(1)	118.8(4)
N(1A)-C(1A)	1.481(10)	N(1)-C(1)-C(2)	108.7(5)
N(1A)-C(14A)	1.483(9)	N(1)-C(1)-H(1A)	109.9
N(1A)-C(6A)#1	1.489(9)	C(2)-C(1)-H(1A)	109.9
N(2A)-C(4A)	1.473(10)	N(1)-C(1)-H(1AB)	109.9
N(2A)-C(3A)	1.479(10)	C(2)-C(1)-H(1AB)	109.9
N(2A)-C(2A)	1.503(10)	H(1A)-C(1)-H(1AB)	108.3
C(1A)-C(2A)	1.545(12)	N(2)-C(2)-C(1)	109.7(5)
C(1A)-H(1AA)	0.9900	N(2)-C(2)-H(2A)	109.7
C(1A)-H(1AC)	0.9900	C(1)-C(2)-H(2A)	109.7
C(2A)-H(2AA)	0.9900	N(2)-C(2)-H(2AB)	109.7
C(2A)-H(2AC)	0.9900	C(1)-C(2)-H(2AB)	109.7
C(3A)-D(3AA)	0.9800	H(2A)-C(2)-H(2AB)	108.2
C(3A)-D(3AB)	0.9800	N(2)-C(3)-D(3A)	109.5
C(3A)-D(3AC)	0.9800	N(2)-C(3)-D(3B)	109.5
C(4A)-C(5A)	1.549(12)	D(3A)-C(3)-D(3B)	109.5
C(4A)-H(4AA)	0.9900	N(2)-C(3)-D(3C)	109.5
C(4A)-H(4AC)	0.9900	D(3A)-C(3)-D(3C)	109.5
C(5A)-C(6A)	1.535(12)	D(3B)-C(3)-D(3C)	109.5
C(5A)-H(5AA)	0.9900	N(2)-C(4)-C(12)#1	114.1(5)
C(5A)-H(5AB)	0.9900	N(2)-C(4)-H(4A)	108.7
C(6A)-H(6AA)	0.9900	C(12)#1-C(4)-H(4A)	108.7
C(6A)-H(6AB)	0.9900	N(2)-C(4)-H(4AB)	108.7
C(14A)-D(14D)	0.9800	C(12)#1-C(4)-H(4AB)	108.7
C(14A)-D(14E)	0.9800	H(4A)-C(4)-H(4AB)	107.6
C(14A)-D(14F)	0.9800	C(13)-C(12)-C(4)#1	117.3(5)
N(1B)-C(1B)	1.486(13)	C(13)-C(12)-H(12A)	108.0
N(1B)-C(13B)	1.489(14)	C(4)#1-C(12)-H(12A)	108.0
N(1B)-C(14B)	1.491(14)	C(13)-C(12)-H(12B)	108.0
N(2B)-C(2B)	1.471(13)	C(4)#1-C(12)-H(12B)	108.0
N(2B)-C(3B)	1.483(14)	H(12A)-C(12)-H(12B)	107.2
N(2B)-C(4B)	1.489(13)	N(1)-C(13)-C(12)	114.5(5)
C(1B)-C(2B)	1.549(14)	N(1)-C(13)-H(13A)	108.6
C(1B)-H(1BA)	0.9900	C(12)-C(13)-H(13A)	108.6
C(1B)-H(1BB)	0.9900	N(1)-C(13)-H(13B)	108.6
C(2B)-H(2BA)	0.9900	C(12)-C(13)-H(13B)	108.6
C(2B)-H(2BB)	0.9900	H(13A)-C(13)-H(13B)	107.6
C(3B)-D(3BA)	0.9800	N(1)-C(14)-D(14A)	109.5
C(3B)-D(3BB)	0.9800	N(1)-C(14)-D(14B)	109.5
C(3B)-D(3BC)	0.9800	D(14A)-C(14)-D(14B)	109.5
C(4B)-C(12B)#1	1.533(15)	N(1)-C(14)-D(14C)	109.5
C(4B)-H(4BA)	0.9900	D(14A)-C(14)-D(14C)	109.5
C(4B)-H(4BB)	0.9900	D(14B)-C(14)-D(14C)	109.5
C(12B)-C(13B)	1.543(15)	C(1A)-N(1A)-C(14A)	108.1(7)
C(12B)-H(12C)	0.9900	C(1A)-N(1A)-Co(1)	103.6(5)
C(12B)-H(12D)	0.9900	C(14A)-N(1A)-Co(1)	117.8(6)
C(13B)-H(13C)	0.9900	C(6A)#1-N(1A)-Co(1)	110.5(5)
C(13B)-H(13D)	0.9900	C(4A)-N(2A)-C(3A)	109.6(9)
C(14B)-D(14G)	0.9800	C(4A)-N(2A)-C(2A)	110.0(7)
C(14B)-D(14H)	0.9800	C(3A)-N(2A)-C(2A)	106.8(9)
C(14B)-D(14I)	0.9800	C(4A)-N(2A)-Co(1)	110.8(6)
S(1)-O(3)	1.423(6)	C(3A)-N(2A)-Co(1)	118.3(6)
S(1)-O(2)	1.445(9)	C(2A)-N(2A)-Co(1)	100.8(6)
S(1)-O(1)	1.458(7)	N(1A)-C(1A)-C(2A)	108.4(8)
S(1)-C(21)	1.817(10)	N(1A)-C(1A)-H(1AA)	110.0
F(1)-C(21)	1.329(9)	C(2A)-C(1A)-H(1AA)	110.0
F(2)-C(21)	1.336(9)	N(1A)-C(1A)-H(1AC)	110.0

C(2A)-C(1A)-H(1AC)	110.0
H(1AA)-C(1A)-H(1AC)	108.4
N(2A)-C(2A)-C(1A)	109.6(8)
N(2A)-C(2A)-H(2AA)	109.7
C(1A)-C(2A)-H(2AA)	109.7
N(2A)-C(2A)-H(2AC)	109.7
C(1A)-C(2A)-H(2AC)	109.7
H(2AA)-C(2A)-H(2AC)	108.2
N(2A)-C(3A)-D(3AA)	109.5
N(2A)-C(3A)-D(3AB)	109.5
D(3AA)-C(3A)-D(3AB)	109.5
N(2A)-C(3A)-D(3AC)	109.5
D(3AA)-C(3A)-D(3AC)	109.5
D(3AB)-C(3A)-D(3AC)	109.5
N(2A)-C(4A)-C(5A)	112.2(9)
N(2A)-C(4A)-H(4AA)	109.2
C(5A)-C(4A)-H(4AA)	109.2
N(2A)-C(4A)-H(4AC)	109.2
C(5A)-C(4A)-H(4AC)	109.2
H(4AA)-C(4A)-H(4AC)	107.9
C(6A)-C(5A)-C(4A)	116.7(9)
C(6A)-C(5A)-H(5AA)	108.1
C(4A)-C(5A)-H(5AA)	108.1
C(6A)-C(5A)-H(5AB)	108.1
C(4A)-C(5A)-H(5AB)	108.1
H(5AA)-C(5A)-H(5AB)	107.3
N(1A)#1-C(6A)-C(5A)	113.3(8)
N(1A)#1-C(6A)-H(6AA)	108.9
C(5A)-C(6A)-H(6AA)	108.9
N(1A)#1-C(6A)-H(6AB)	108.9
C(5A)-C(6A)-H(6AB)	108.9
H(6AA)-C(6A)-H(6AB)	107.7
N(1A)-C(14A)-D(14D)	109.5
N(1A)-C(14A)-D(14E)	109.5
D(14D)-C(14A)-D(14E)	109.5
N(1A)-C(14A)-D(14F)	109.5
D(14D)-C(14A)-D(14F)	109.5
D(14E)-C(14A)-D(14F)	109.5
C(1B)-N(1B)-C(13B)	107.1(14)
C(1B)-N(1B)-C(14B)	107.4(16)
C(13B)-N(1B)-C(14B)	107.4(15)
C(1B)-N(1B)-Co(1)	101.9(11)
C(13B)-N(1B)-Co(1)	114.6(13)
C(14B)-N(1B)-Co(1)	117.6(14)
C(2B)-N(2B)-C(3B)	109.9(15)
C(2B)-N(2B)-C(4B)	108.9(13)
C(3B)-N(2B)-C(4B)	108.8(15)
C(2B)-N(2B)-Co(1)	102.3(11)
C(3B)-N(2B)-Co(1)	117.0(13)
C(4B)-N(2B)-Co(1)	109.6(13)
N(1B)-C(1B)-C(2B)	110.2(14)
N(1B)-C(1B)-H(1BA)	109.6
C(2B)-C(1B)-H(1BA)	109.6
N(1B)-C(1B)-H(1BB)	109.6
C(2B)-C(1B)-H(1BB)	109.6
H(1BA)-C(1B)-H(1BB)	108.1
N(2B)-C(2B)-C(1B)	109.7(14)
N(2B)-C(2B)-H(2BA)	109.7
C(1B)-C(2B)-H(2BA)	109.7
N(2B)-C(2B)-H(2BB)	109.7
C(1B)-C(2B)-H(2BB)	109.7
H(2BA)-C(2B)-H(2BB)	108.2
N(2B)-C(3B)-D(3BA)	109.5
N(2B)-C(3B)-D(3BB)	109.5
D(3BA)-C(3B)-D(3BB)	109.5
N(2B)-C(3B)-D(3BC)	109.5
D(3BA)-C(3B)-D(3BC)	109.5
D(3BB)-C(3B)-D(3BC)	109.5
N(2B)-C(4B)-H(4BA)	109.1
C(12B)#1-C(4B)-H(4BA)	109.1
N(2B)-C(4B)-H(4BB)	109.1
C(12B)#1-C(4B)-H(4BB)	109.1
H(4BA)-C(4B)-H(4BB)	107.8
C(4B)#1-C(12B)-C(13B)	116(2)
C(4B)#1-C(12B)-H(12C)	108.3
C(13B)-C(12B)-H(12C)	108.3
C(4B)#1-C(12B)-H(12D)	108.3
C(13B)-C(12B)-H(12D)	108.3
H(12C)-C(12B)-H(12D)	107.4
N(1B)-C(13B)-C(12B)	106.7(16)
N(1B)-C(13B)-H(13C)	110.4
C(12B)-C(13B)-H(13C)	110.4
N(1B)-C(13B)-H(13D)	110.4
C(12B)-C(13B)-H(13D)	110.4
H(13C)-C(13B)-H(13D)	108.6
N(1B)-C(14B)-D(14G)	109.5
N(1B)-C(14B)-D(14H)	109.5
D(14G)-C(14B)-D(14H)	109.5
N(1B)-C(14B)-D(14I)	109.5
D(14G)-C(14B)-D(14I)	109.5
D(14H)-C(14B)-D(14I)	109.5
O(3)-S(1)-O(2)	116.6(6)
O(3)-S(1)-O(1)	114.5(4)
O(2)-S(1)-O(1)	112.9(8)
O(3)-S(1)-C(21)	104.0(4)
O(2)-S(1)-C(21)	105.0(5)
O(1)-S(1)-C(21)	101.7(4)
S(1)-O(1)-Co(1)	157.1(5)
F(3)-C(21)-F(1)	107.5(7)
F(3)-C(21)-F(2)	108.7(8)
F(1)-C(21)-F(2)	108.1(7)
F(3)-C(21)-S(1)	110.8(7)
F(1)-C(21)-S(1)	111.5(6)
F(2)-C(21)-S(1)	110.1(6)
O(3A)-S(1A)-O(2A)	116.8(7)
O(3A)-S(1A)-O(1A)	114.8(4)
O(2A)-S(1A)-O(1A)	115.0(8)
O(3A)-S(1A)-C(21A)	102.2(4)
O(2A)-S(1A)-C(21A)	102.6(8)
O(1A)-S(1A)-C(21A)	102.3(5)
S(1A)-O(1A)-Co(1)	156.9(7)
F(2A)-C(21A)-F(1A)	106.1(7)
F(2A)-C(21A)-F(3A)	106.4(7)
F(1A)-C(21A)-F(3A)	108.5(8)
F(2A)-C(21A)-S(1A)	113.4(6)
F(1A)-C(21A)-S(1A)	111.3(6)
F(3A)-C(21A)-S(1A)	110.8(5)

Symmetry transformations used to generate equivalent atoms: #1 -x+1,-y+1,-z+1

Table S50. Anisotropic displacement parameters (Å^{2}x 10^3) for **5b**.
The anisotropic displacement factor exponent takes the form: -2p^2[h^2 a*^{2}U^{11} + ... + 2 h k a* b* U^{12}]

	U^{11}	U^{22}	U^{33}	U^{23}	U^{13}	U^{12}
Co(1)	19(1)	40(1)	21(1)	6(1)	-1(1)	-9(1)
N(1)	21(2)	34(2)	47(2)	-5(2)	15(2)	-2(2)
N(2)	34(2)	44(2)	23(2)	4(2)	5(2)	8(2)
C(1)	32(3)	45(3)	47(3)	9(3)	16(3)	-4(3)
C(2)	42(3)	49(3)	35(3)	13(3)	14(2)	5(2)
C(3)	49(4)	68(4)	23(2)	-2(2)	5(2)	14(3)
C(4)	32(3)	66(4)	31(3)	4(3)	4(2)	15(3)
C(12)	26(2)	57(4)	52(3)	-9(3)	-1(2)	1(2)
C(13)	19(2)	41(3)	57(3)	-5(2)	7(2)	0(2)
C(14)	29(3)	38(3)	71(4)	-14(3)	14(3)	-8(2)
N(1A)	17(3)	39(4)	47(4)	-17(3)	1(3)	-3(3)
N(2A)	32(3)	51(4)	32(3)	7(3)	16(3)	6(3)
C(1A)	25(4)	42(4)	61(5)	-9(4)	-1(4)	-8(3)
C(2A)	34(4)	46(5)	55(5)	-1(4)	17(4)	-7(4)
C(3A)	65(7)	45(5)	56(6)	12(5)	32(6)	10(5)
C(4A)	53(5)	58(5)	30(4)	8(4)	14(4)	-2(5)
C(5A)	54(5)	63(6)	34(5)	4(4)	2(4)	9(5)
C(6A)	35(4)	55(5)	29(4)	-14(4)	-1(3)	4(4)
C(14A)	25(4)	56(5)	46(5)	-5(4)	-8(4)	7(4)
N(1B)	26(6)	54(7)	45(6)	-6(6)	-13(5)	-6(5)
N(2B)	30(5)	47(5)	46(5)	-4(5)	17(5)	-5(5)
C(1B)	28(6)	61(7)	53(6)	-8(6)	-3(5)	-8(6)
C(2B)	27(5)	52(7)	55(6)	-8(6)	4(5)	-8(5)
C(3B)	44(9)	43(7)	65(10)	7(7)	35(8)	-6(7)
C(4B)	43(7)	54(7)	41(6)	7(6)	17(5)	-1(6)
C(12B)	53(14)	69(15)	72(16)	1(13)	20(11)	6(12)
C(13B)	34(10)	63(12)	54(9)	12(9)	0(8)	11(9)
C(14B)	61(13)	71(10)	45(12)	-8(10)	-23(10)	18(9)
S(1)	29(1)	43(1)	38(1)	-2(1)	6(1)	1(1)
F(1)	34(2)	60(3)	57(2)	4(2)	0(2)	11(2)
F(2)	68(3)	53(3)	91(4)	-13(2)	25(3)	19(2)
F(3)	57(3)	49(3)	81(4)	11(2)	32(2)	-4(2)
O(1)	22(3)	41(2)	29(3)	-1(3)	1(2)	3(2)
O(2)	35(3)	58(4)	38(4)	5(3)	14(3)	6(3)
O(3)	44(3)	65(3)	63(3)	-30(3)	2(2)	-3(2)
C(21)	34(3)	41(4)	59(4)	4(3)	19(3)	8(2)
S(1A)	27(1)	34(1)	43(2)	-7(1)	13(1)	0(1)
F(1A)	102(7)	70(6)	63(4)	3(4)	3(4)	48(5)
F(2A)	77(4)	55(3)	96(5)	-21(3)	45(4)	18(3)
F(3A)	81(3)	42(3)	82(4)	-2(2)	45(3)	-5(2)
O(1A)	21(3)	40(3)	34(4)	0(3)	6(3)	5(3)
O(2A)	35(4)	70(8)	77(9)	-36(6)	24(5)	-17(5)
O(3A)	61(3)	52(3)	42(3)	-15(2)	16(2)	-8(3)
C(21A)	54(4)	49(4)	59(4)	-11(3)	22(3)	16(3)

Table S51. Hydrogen coordinates (x 10^4) and isotropic displacement parameters ($Å^2$x 10^3) for **5b**.

	x	y	z	U(eq)
H(1A)	2442	2727	6138	48
H(1AB)	2710	4267	6366	48
H(2A)	4854	2982	7270	49
H(2AB)	5214	2464	6248	49
H(4A)	7576	3267	6273	52
H(4AB)	7789	3746	7390	52
H(12A)	369	5148	3244	56
H(12B)	1659	4087	3124	56
H(13A)	1509	5242	4967	47
H(13B)	690	3875	4604	47
H(1AA)	2830	2634	4329	53
H(1AC)	1128	3246	4302	53
H(2AA)	2142	4295	5880	53
H(2AC)	2235	2723	5967	53
H(4AA)	4428	3463	7498	56
H(4AC)	4136	4925	7073	56
H(5AA)	6717	4568	8118	62
H(5AB)	7113	3635	7278	62
H(6AA)	6460	6374	6950	49
H(6AB)	8201	5828	7305	49
H(1BA)	2431	3236	3782	59
H(1BB)	970	4224	3611	59
H(2BA)	1650	4869	5320	54
H(2BB)	1299	3322	5227	54
H(4BA)	2980	3614	7005	54
H(4BB)	3365	5086	6724	54
H(12C)	4713	5965	1958	76
H(12D)	4219	6843	2796	76
H(13C)	2499	4962	2292	62
H(13D)	3917	4024	2801	62

[(D$_{12}$-TMC)Co^{II}(OTf)]B$_{Ar}$F (**5b-B$_{Ar}$F**)

5b-B$_{Ar}$F was solved in the monoclinic space group *P*2$_1$/*c*. The asymmetric unit contains one full complex and anion. The ligand was found to be fully disordered and six of the CF_3 groups were also found to be disordered. The disorder was refined with the help of same distance restraints on 1,2 and 1,3 distances, similarity restraints on anisotropic displacement parameters and advanced rigid bond restraints. Some atoms had to be set to the same anisotropic displacement parameters to allow for anisotropic refinement. The disorder ratio for the ligand converged to 0.974(2) and for the CF_3 groups to 0.396(2):0.386(2):0.217(2), 0.65(4), 0.964(3)(1), 0.78(3), 0.879(5) and 0.915(3).

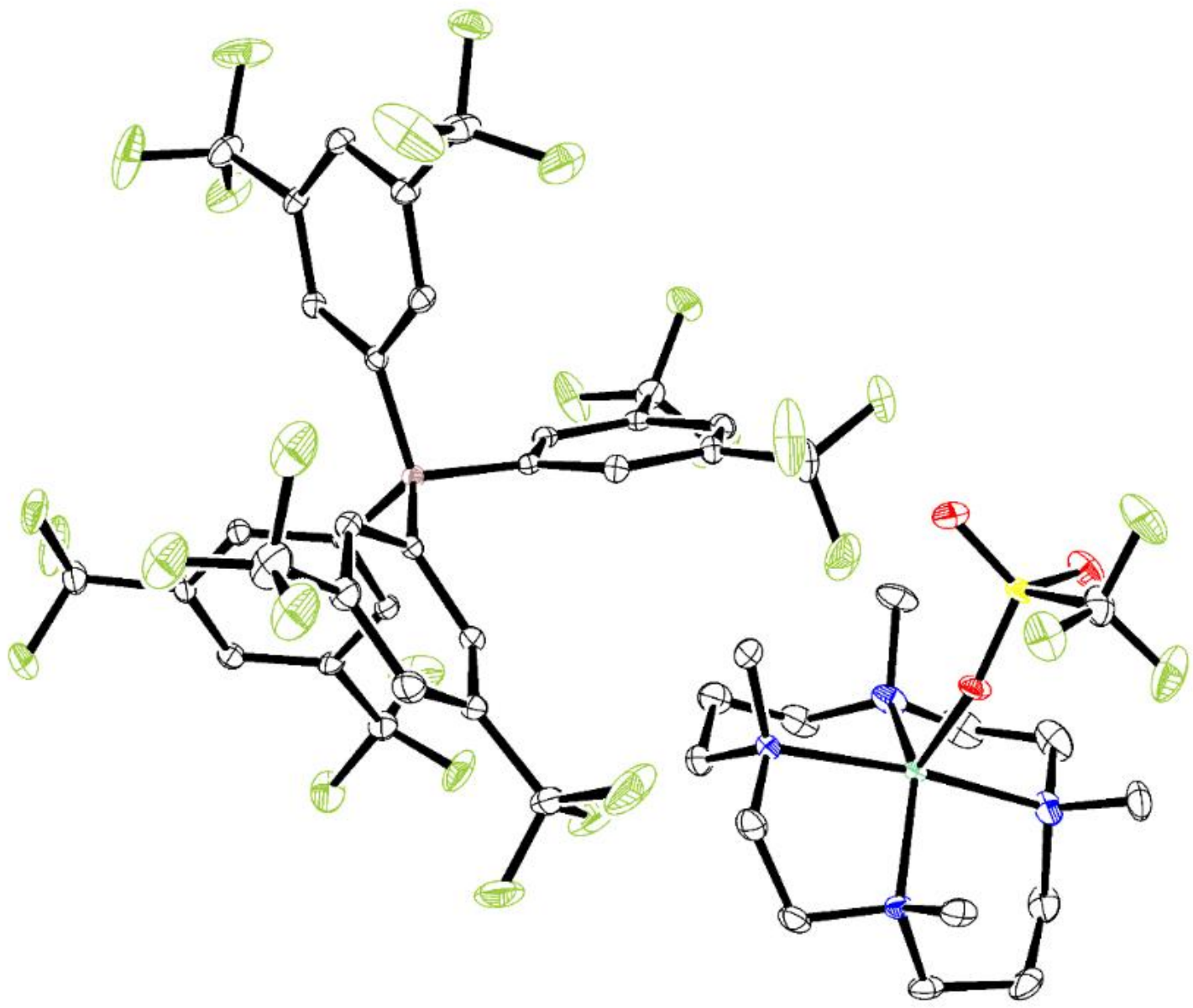

Figure S11. Molecular structure of [(D$_{12}$-TMC)Co^{II}(OTf)]B$_{Ar}$F (**5b-B$_{Ar}$F**).

Table S52. Crystal data and structure refinement for **5b-B$_{Ar}$F**.

CCDC No	2062715	
Empirical formula	$C_{47}H_{32}BCoD_{12}F_{27}N_4O_3S$	
Formula weight	1339.73	
Temperature	100(2) K	
Wavelength	0.71073 Å	
Crystal system	Monoclinic	
Space group	*P*2$_1$/*c*	
Unit cell dimensions	a = 12.2574(4) Å	α = 90°
	b = 25.1110(8) Å	β = 97.0219(13)°
	c = 17.3721(6) Å	γ = 90°
Volume	5306.9(3) Å^3	
Z	4	
Density (calculated)	1.677 Mg/m^3	
Absorption coefficient	0.504 mm^{-1}	
F(000)	2676	
Crystal size	0.170 x 0.127 x 0.123 mm^3	
Theta range for data collection	2.311 to 31.505°.	
Index ranges	-18 ≤ h ≤ 18, -36 ≤ k ≤ 36, -25 ≤ l ≤ 25	
Reflections collected	219025	
Independent reflections	17649 [R(int) = 0.0779]	
Completeness to theta = 25.242°	99.9 %	
Absorption correction	Semi-empirical from equivalents	
Refinement method	Full-matrix least-squares on *F*^{2}	
Data / restraints / parameters	17649 / 4716 / 1025	
Goodness-of-fit on *F*^{2}	1.031	
Final R indices [I>2σ(I)]	R1 = 0.0531, wR2 = 0.1122	
R indices (all data)	R1 = 0.0815, wR2 = 0.1237	
Largest diff. peak and hole	1.704 and -1.308 eÅ^{-3}	

Table S53. Atomic coordinates (x 10^4) and equivalent isotropic displacement parameters (Å^{2}x 10^3) for **5b-B$_{ArF}$**. U(eq) is defined as one third of the trace of the orthogonalized U^{ij} tensor.

	x	y	z	U(eq)
Co(1)	9681(1)	8208(1)	7392(1)	10(1)
N(1)	11060(2)	8754(1)	7635(1)	20(1)
N(2)	9958(1)	8086(1)	6234(1)	15(1)
N(3)	8171(2)	7745(1)	7037(1)	16(1)
N(4)	8878(2)	8725(1)	8099(1)	20(1)
C(1)	11117(2)	9129(1)	6976(2)	26(1)
C(2)	11253(2)	8869(1)	6208(2)	26(1)
C(3)	10257(2)	8576(1)	5824(1)	20(1)
C(4)	10843(2)	7680(1)	6219(1)	19(1)
D(4A)	10989	7624	5682	28
D(4B)	10608	7344	6433	28
D(4C)	11514	7806	6530	28
C(5)	8933(2)	7867(1)	5799(1)	19(1)
C(6)	8379(2)	7488(1)	6302(1)	20(1)
C(7)	7916(2)	7316(1)	7570(1)	24(1)
D(7A)	7272	7117	7335	35
D(7B)	7761	7472	8062	35
D(7C)	8547	7075	7665	35
C(8)	7189(2)	8100(1)	6873(1)	21(1)
C(9)	6947(2)	8449(1)	7546(1)	27(1)
C(10)	7761(2)	8897(1)	7743(1)	26(1)
C(11)	8790(2)	8467(1)	8864(1)	25(1)
D(11A)	8435	8119	8780	38
D(11B)	8351	8693	9169	38
D(11C)	9527	8421	9145	38
C(12)	9581(2)	9210(1)	8238(2)	30(1)
C(13)	10787(2)	9059(1)	8321(2)	29(1)
C(14)	12172(2)	8520(1)	7844(2)	26(1)
D(14A)	12361	8299	7415	39
D(14B)	12175	8301	8311	39
D(14C)	12713	8807	7944	39
N(1A)	10470(30)	8229(14)	6340(20)	17(3)
N(2A)	8630(40)	7605(14)	7040(20)	18(3)
N(3A)	8180(30)	8512(16)	7940(20)	23(4)
N(4A)	10540(30)	8892(16)	7710(20)	24(4)
C(1A)	9550(40)	8147(19)	5730(30)	19(4)
C(2A)	8990(50)	7620(20)	5620(20)	19(4)
C(3A)	8730(60)	7308(18)	6310(30)	21(4)
C(4A)	8510(60)	7190(20)	7660(30)	24(1)
D(4AA)	9202	6997	7772	35
D(4AB)	8329	7370	8126	35
D(4AC)	7920	6944	7468	35
C(5A)	7560(40)	7890(20)	6890(30)	20(4)
C(6A)	7250(30)	8200(20)	7550(30)	22(4)
C(7A)	8400(50)	8330(30)	8750(20)	26(10)
D(7AA)	9017	8534	9015	39
D(7AB)	7746	8388	9011	39
D(7AC)	8585	7952	8758	39
C(8A)	8000(40)	9092(17)	7850(50)	27(5)
C(9A)	8980(40)	9448(19)	8110(40)	27(5)
C(10A)	9910(40)	9410(20)	7620(40)	25(5)
C(11A)	11140(50)	8880(20)	8520(20)	22(9)
D(11D)	10607	8856	8894	33
D(11E)	11627	8568	8576	33
D(11F)	11579	9204	8614	33
C(12A)	11400(40)	8930(20)	7170(30)	24(4)
C(13A)	10890(50)	8779(17)	6350(20)	22(4)
C(14A)	11340(40)	7820(20)	6390(30)	19(1)
D(14D)	11910	7913	6813	28
D(14E)	11023	7475	6489	28
D(14F)	11655	7812	5899	28
S(1)	10747(1)	7404(1)	8773(1)	14(1)
F(1)	11549(1)	6577(1)	8119(1)	31(1)
F(2)	12225(1)	6715(1)	9305(1)	38(1)
F(3)	12702(1)	7213(1)	8394(1)	41(1)
O(1)	10431(1)	7587(1)	7971(1)	17(1)
O(2)	11233(1)	7811(1)	9282(1)	24(1)
O(3)	9923(1)	7082(1)	9059(1)	23(1)
C(15)	11872(2)	6950(1)	8637(1)	22(1)
F(21)	1711(1)	5543(1)	9206(1)	29(1)
F(22)	1236(1)	4749(1)	8855(1)	44(1)
F(23)	2004(1)	4898(1)	10007(1)	40(1)
F(21A)	1440(20)	5406(13)	8859(19)	29(1)
F(22A)	1460(20)	4612(9)	9400(30)	44(1)
F(23A)	2250(30)	5240(16)	10002(11)	40(1)
F(24)	5395(2)	3914(1)	10130(1)	26(1)
F(25)	6595(1)	4167(1)	9424(1)	41(1)
F(26)	5417(2)	3568(1)	9008(1)	47(1)
F(24A)	5838(19)	4003(10)	10117(7)	40(5)
F(25A)	6309(12)	3879(8)	8950(10)	41(1)
F(26A)	4874(12)	3542(5)	9300(12)	32(4)
F(31)	803(9)	5413(3)	6195(6)	29(1)
F(32)	724(8)	5911(5)	5197(3)	40(2)
F(33)	301(6)	6227(4)	6262(8)	39(2)
F(31A)	900(14)	5750(11)	5208(7)	49(3)
F(32A)	283(10)	6280(5)	5979(16)	42(3)
F(33A)	693(18)	5490(8)	6355(14)	42(4)
F(34)	3309(1)	7513(1)	5448(1)	35(1)
F(35)	3859(2)	7622(1)	6656(1)	42(1)
F(36)	4958(1)	7308(1)	5903(1)	30(1)

F(41)	5081(2)	7201(2)	9155(3)	31(1)
F(42)	6768(2)	7469(1)	9224(1)	34(1)
F(43)	6286(2)	6939(1)	10074(1)	24(1)
F(41A)	5005(10)	7096(11)	9261(17)	17(4)
F(42A)	6574(13)	7126(8)	9972(7)	35(4)
F(43A)	6276(14)	7502(4)	8858(9)	34(1)
F(44)	9458(3)	6035(4)	8017(3)	32(1)
F(45)	9496(6)	5900(4)	9242(3)	25(1)
F(46)	9019(7)	5267(2)	8442(6)	43(2)
F(44A)	9420(13)	5889(9)	7921(8)	32(1)
F(45A)	9590(20)	5976(13)	9153(13)	28(4)
F(46A)	8990(20)	5253(5)	8591(17)	26(3)
F(51)	7647(1)	5878(1)	5455(1)	33(1)
F(52)	7306(1)	5313(1)	4535(1)	35(1)
F(53)	8476(1)	5133(1)	5513(1)	52(1)
F(54)	4148(3)	3728(2)	5560(3)	35(1)
F(55)	5326(4)	3498(2)	6509(3)	35(1)
F(56)	5846(4)	3497(2)	5422(3)	35(1)
F(54A)	4914(4)	3535(2)	6372(3)	35(1)
F(55A)	6095(4)	3516(2)	5577(3)	35(1)
F(56A)	4445(4)	3765(2)	5153(3)	35(1)
F(54B)	4075(6)	3722(3)	5950(5)	35(1)
F(55B)	5716(6)	3430(2)	6100(4)	35(1)
F(56B)	4980(7)	3714(3)	4979(4)	35(1)
C(21)	4401(2)	5140(1)	8054(1)	10(1)
C(22)	3421(2)	5235(1)	8368(1)	11(1)
C(23)	3123(2)	4941(1)	8992(1)	12(1)
C(24)	3793(2)	4542(1)	9337(1)	13(1)
C(25)	4774(2)	4444(1)	9039(1)	13(1)
C(26)	5062(2)	4730(1)	8412(1)	12(1)
C(27)	2033(2)	5038(1)	9268(1)	20(1)
C(28)	5534(2)	4019(1)	9394(1)	20(1)
C(31)	3986(2)	5905(1)	6923(1)	10(1)
C(32)	2908(2)	5743(1)	6658(1)	11(1)
C(33)	2176(2)	6071(1)	6204(1)	12(1)
C(34)	2493(2)	6574(1)	5974(1)	14(1)
C(35)	3550(2)	6741(1)	6230(1)	12(1)
C(36)	4275(2)	6415(1)	6695(1)	12(1)
C(37)	1016(2)	5900(1)	5960(1)	18(1)
C(38)	3915(2)	7292(1)	6051(1)	18(1)
C(41)	5858(2)	5858(1)	7921(1)	10(1)
C(42)	5603(2)	6302(1)	8358(1)	11(1)
C(43)	6401(2)	6576(1)	8839(1)	11(1)
C(44)	7499(2)	6415(1)	8915(1)	13(1)
C(45)	7761(2)	5971(1)	8503(1)	12(1)
C(46)	6958(2)	5700(1)	8018(1)	12(1)
C(47)	6111(2)	7047(1)	9300(1)	17(1)
C(48)	8930(2)	5792(1)	8552(1)	19(1)
C(51)	5410(1)	5160(1)	6739(1)	10(1)
C(52)	6173(2)	5392(1)	6295(1)	12(1)
C(53)	6618(2)	5113(1)	5717(1)	14(1)
C(54)	6300(2)	4592(1)	5536(1)	17(1)
C(55)	5538(2)	4358(1)	5957(1)	16(1)
C(56)	5110(2)	4636(1)	6550(1)	13(1)
C(57)	7502(2)	5360(1)	5308(1)	19(1)
C(58)	5188(2)	3795(1)	5785(1)	27(1)
B(21)	4892(2)	5513(1)	7403(1)	10(1)

Table S54. Bond lengths [Å] and angles [°] for **5b-B$_{ArF}$**.

Co(1)-O(1)	2.0142(14)	C(9A)-C(10A)	1.51(2)
Co(1)-N(2A)	2.03(3)	C(9A)-H(9AA)	0.9900
Co(1)-N(4A)	2.06(3)	C(9A)-H(9AC)	0.9900
Co(1)-N(2)	2.1023(17)	C(10A)-H(10C)	0.9900
Co(1)-N(4)	2.1130(18)	C(10A)-H(10D)	0.9900
Co(1)-N(1A)	2.17(3)	C(11A)-D(11D)	0.9800
Co(1)-N(1)	2.1777(19)	C(11A)-D(11E)	0.9800
Co(1)-N(3)	2.2076(18)	C(11A)-D(11F)	0.9800
Co(1)-N(3A)	2.31(3)	C(12A)-C(13A)	1.53(2)
N(1)-C(14)	1.488(3)	C(12A)-H(12C)	0.9900
N(1)-C(13)	1.488(3)	C(12A)-H(12D)	0.9900
N(1)-C(1)	1.491(3)	C(13A)-H(13C)	0.9900
N(2)-C(5)	1.490(3)	C(13A)-H(13D)	0.9900
N(2)-C(4)	1.490(3)	C(14A)-D(14D)	0.9800
N(2)-C(3)	1.492(3)	C(14A)-D(14E)	0.9800
N(3)-C(7)	1.478(3)	C(14A)-D(14F)	0.9800
N(3)-C(6)	1.480(3)	S(1)-O(3)	1.4303(16)
N(3)-C(8)	1.497(3)	S(1)-O(2)	1.4332(16)
N(4)-C(11)	1.494(3)	S(1)-O(1)	1.4733(14)
N(4)-C(12)	1.494(3)	S(1)-C(15)	1.826(2)
N(4)-C(10)	1.495(3)	F(1)-C(15)	1.326(3)
C(1)-C(2)	1.513(4)	F(2)-C(15)	1.326(3)
C(1)-H(1A)	0.9900	F(3)-C(15)	1.323(3)
C(1)-H(1AB)	0.9900	F(21)-C(27)	1.329(3)
C(2)-C(3)	1.509(3)	F(22)-C(27)	1.351(3)
C(2)-H(2A)	0.9900	F(23)-C(27)	1.336(3)
C(2)-H(2AB)	0.9900	F(21A)-C(27)	1.327(12)
C(3)-H(3A)	0.9900	F(22A)-C(27)	1.317(12)
C(3)-H(3AB)	0.9900	F(23A)-C(27)	1.368(13)
C(4)-D(4A)	0.9800	F(24)-C(28)	1.336(3)
C(4)-D(4B)	0.9800	F(25)-C(28)	1.348(3)
C(4)-D(4C)	0.9800	F(26)-C(28)	1.314(3)
C(5)-C(6)	1.509(3)	F(24A)-C(28)	1.267(11)
C(5)-H(5A)	0.9900	F(25A)-C(28)	1.341(11)
C(5)-H(5AB)	0.9900	F(26A)-C(28)	1.443(11)
C(6)-H(6A)	0.9900	F(31)-C(37)	1.325(6)
C(6)-H(6AB)	0.9900	F(32)-C(37)	1.330(5)
C(7)-D(7A)	0.9800	F(33)-C(37)	1.354(5)
C(7)-D(7B)	0.9800	F(31A)-C(37)	1.350(9)
C(7)-D(7C)	0.9800	F(32A)-C(37)	1.315(9)
C(8)-C(9)	1.519(3)	F(33A)-C(37)	1.323(10)
C(8)-H(8A)	0.9900	F(34)-C(38)	1.329(2)
C(8)-H(8AB)	0.9900	F(35)-C(38)	1.346(3)
C(9)-C(10)	1.514(4)	F(36)-C(38)	1.334(2)
C(9)-H(9A)	0.9900	F(41)-C(47)	1.315(3)
C(9)-H(9AB)	0.9900	F(42)-C(47)	1.346(3)
C(10)-H(10A)	0.9900	F(43)-C(47)	1.362(3)
C(10)-H(10B)	0.9900	F(41A)-C(47)	1.355(12)
C(11)-D(11A)	0.9800	F(42A)-C(47)	1.250(10)
C(11)-D(11B)	0.9800	F(43A)-C(47)	1.405(10)
C(11)-D(11C)	0.9800	F(44)-C(48)	1.342(4)
C(12)-C(13)	1.516(4)	F(45)-C(48)	1.337(4)
C(12)-H(12A)	0.9900	F(46)-C(48)	1.337(4)
C(12)-H(12B)	0.9900	F(44A)-C(48)	1.335(10)
C(13)-H(13A)	0.9900	F(45A)-C(48)	1.322(11)
C(13)-H(13B)	0.9900	F(46A)-C(48)	1.355(12)
C(14)-D(14A)	0.9800	F(51)-C(57)	1.332(3)
C(14)-D(14B)	0.9800	F(52)-C(57)	1.340(2)
C(14)-D(14C)	0.9800	F(53)-C(57)	1.333(3)
N(1A)-C(1A)	1.464(19)	F(54)-C(58)	1.298(4)
N(1A)-C(14A)	1.465(19)	F(55)-C(58)	1.453(5)
N(1A)-C(13A)	1.475(19)	F(56)-C(58)	1.317(6)
N(2A)-C(5A)	1.49(2)	F(54A)-C(58)	1.289(5)
N(2A)-C(3A)	1.495(19)	F(55A)-C(58)	1.399(6)
N(2A)-C(4A)	1.508(19)	F(56A)-C(58)	1.341(5)
N(3A)-C(7A)	1.465(19)	F(54B)-C(58)	1.440(7)
N(3A)-C(6A)	1.471(19)	F(55B)-C(58)	1.213(7)
N(3A)-C(8A)	1.48(2)	F(56B)-C(58)	1.407(7)
N(4A)-C(12A)	1.50(2)	C(21)-C(22)	1.399(2)
N(4A)-C(11A)	1.502(19)	C(21)-C(26)	1.406(3)
N(4A)-C(10A)	1.51(2)	C(21)-B(21)	1.639(3)
C(1A)-C(2A)	1.50(2)	C(22)-C(23)	1.396(3)
C(1A)-H(1AA)	0.9900	C(22)-H(22)	0.9500
C(1A)-H(1AC)	0.9900	C(23)-C(24)	1.384(3)
C(2A)-C(3A)	1.494(19)	C(23)-C(27)	1.494(3)
C(2A)-H(2AA)	0.9900	C(24)-C(25)	1.389(3)
C(2A)-H(2AC)	0.9900	C(24)-H(24)	0.9500
C(3A)-H(3AA)	0.9900	C(25)-C(26)	1.388(3)
C(3A)-H(3AC)	0.9900	C(25)-C(28)	1.498(3)
C(4A)-D(4AA)	0.9800	C(26)-H(26)	0.9500
C(4A)-D(4AB)	0.9800	C(31)-C(36)	1.398(3)
C(4A)-D(4AC)	0.9800	C(31)-C(32)	1.406(2)
C(5A)-C(6A)	1.49(2)	C(31)-B(21)	1.635(3)
C(5A)-H(5AA)	0.9900	C(32)-C(33)	1.390(3)
C(5A)-H(5AC)	0.9900	C(32)-H(32)	0.9500
C(6A)-H(6AA)	0.9900	C(33)-C(34)	1.394(3)
C(6A)-H(6AC)	0.9900	C(33)-C(37)	1.498(3)
C(7A)-D(7AA)	0.9800	C(34)-C(35)	1.383(3)
C(7A)-D(7AB)	0.9800	C(34)-H(34)	0.9500
C(7A)-D(7AC)	0.9800	C(35)-C(36)	1.391(3)
C(8A)-C(9A)	1.52(2)	C(35)-C(38)	1.498(3)
C(8A)-H(8AA)	0.9900	C(36)-H(36)	0.9500
C(8A)-H(8AC)	0.9900	C(41)-C(46)	1.396(2)

C(41)-C(42)	1.407(3)
C(41)-B(21)	1.644(3)
C(42)-C(43)	1.388(3)
C(42)-H(42)	0.9500
C(43)-C(44)	1.396(3)
C(43)-C(47)	1.496(3)
C(44)-C(45)	1.383(3)
C(44)-H(44)	0.9500
C(45)-C(46)	1.392(3)
C(45)-C(48)	1.496(3)
C(46)-H(46)	0.9500
C(51)-C(56)	1.395(3)
C(51)-C(52)	1.408(3)
C(51)-B(21)	1.642(3)
C(52)-C(53)	1.390(3)
C(52)-H(52)	0.9500
C(53)-C(54)	1.391(3)
C(53)-C(57)	1.500(3)
C(54)-C(55)	1.385(3)
C(54)-H(54)	0.9500
C(55)-C(56)	1.398(3)
C(55)-C(58)	1.496(3)
C(56)-H(56)	0.9500
O(1)-Co(1)-N(2A)	78.9(13)
O(1)-Co(1)-N(4A)	108.9(12)
N(2A)-Co(1)-N(4A)	171.3(18)
O(1)-Co(1)-N(2)	104.33(6)
O(1)-Co(1)-N(4)	113.74(7)
N(2)-Co(1)-N(4)	141.89(7)
O(1)-Co(1)-N(1A)	102.5(11)
N(2A)-Co(1)-N(1A)	95.5(12)
N(4A)-Co(1)-N(1A)	86.5(13)
O(1)-Co(1)-N(1)	95.43(7)
N(2)-Co(1)-N(1)	93.72(7)
N(4)-Co(1)-N(1)	84.57(8)
O(1)-Co(1)-N(3)	92.77(7)
N(2)-Co(1)-N(3)	83.35(7)
N(4)-Co(1)-N(3)	92.95(8)
N(1)-Co(1)-N(3)	171.75(7)
O(1)-Co(1)-N(3A)	112.9(11)
N(2A)-Co(1)-N(3A)	82.1(12)
N(4A)-Co(1)-N(3A)	91.2(12)
N(1A)-Co(1)-N(3A)	143.3(16)
C(14)-N(1)-C(13)	107.24(19)
C(14)-N(1)-C(1)	107.97(19)
C(13)-N(1)-C(1)	109.06(18)
C(14)-N(1)-Co(1)	117.72(14)
C(13)-N(1)-Co(1)	103.56(15)
C(1)-N(1)-Co(1)	110.91(13)
C(5)-N(2)-C(4)	108.00(17)
C(5)-N(2)-C(3)	107.63(16)
C(4)-N(2)-C(3)	109.51(16)
C(5)-N(2)-Co(1)	108.30(12)
C(4)-N(2)-Co(1)	108.66(12)
C(3)-N(2)-Co(1)	114.54(13)
C(7)-N(3)-C(6)	107.05(18)
C(7)-N(3)-C(8)	109.03(17)
C(6)-N(3)-C(8)	108.36(16)
C(7)-N(3)-Co(1)	116.27(13)
C(6)-N(3)-Co(1)	104.12(13)
C(8)-N(3)-Co(1)	111.56(14)
C(11)-N(4)-C(12)	108.20(19)
C(11)-N(4)-C(10)	109.36(19)
C(12)-N(4)-C(10)	108.26(19)
C(11)-N(4)-Co(1)	109.87(14)
C(12)-N(4)-Co(1)	107.09(15)
C(10)-N(4)-Co(1)	113.89(14)
N(1)-C(1)-C(2)	115.25(19)
N(1)-C(1)-H(1A)	108.5
C(2)-C(1)-H(1A)	108.5
N(1)-C(1)-H(1AB)	108.5
C(2)-C(1)-H(1AB)	108.5
H(1A)-C(1)-H(1AB)	107.5
C(3)-C(2)-C(1)	115.4(2)
C(3)-C(2)-H(2A)	108.4
C(1)-C(2)-H(2A)	108.4
C(3)-C(2)-H(2AB)	108.4
C(1)-C(2)-H(2AB)	108.4
H(2A)-C(2)-H(2AB)	107.5
N(2)-C(3)-C(2)	115.05(18)
N(2)-C(3)-H(3A)	108.5
C(2)-C(3)-H(3A)	108.5
N(2)-C(3)-H(3AB)	108.5
C(2)-C(3)-H(3AB)	108.5
H(3A)-C(3)-H(3AB)	107.5
N(2)-C(4)-D(4A)	109.5
N(2)-C(4)-D(4B)	109.5
D(4A)-C(4)-D(4B)	109.5
N(2)-C(4)-D(4C)	109.5
D(4A)-C(4)-D(4C)	109.5
D(4B)-C(4)-D(4C)	109.5
N(2)-C(5)-C(6)	110.49(17)
N(2)-C(5)-H(5A)	109.6
C(6)-C(5)-H(5A)	109.6
N(2)-C(5)-H(5AB)	109.6
C(6)-C(5)-H(5AB)	109.6
H(5A)-C(5)-H(5AB)	108.1
N(3)-C(6)-C(5)	111.15(18)
N(3)-C(6)-H(6A)	109.4
C(5)-C(6)-H(6A)	109.4
N(3)-C(6)-H(6AB)	109.4
C(5)-C(6)-H(6AB)	109.4
H(6A)-C(6)-H(6AB)	108.0
N(3)-C(7)-D(7A)	109.5
N(3)-C(7)-D(7B)	109.5
D(7A)-C(7)-D(7B)	109.5
N(3)-C(7)-D(7C)	109.5
D(7A)-C(7)-D(7C)	109.5
D(7B)-C(7)-D(7C)	109.5
N(3)-C(8)-C(9)	115.15(18)
N(3)-C(8)-H(8A)	108.5
C(9)-C(8)-H(8A)	108.5
N(3)-C(8)-H(8AB)	108.5
C(9)-C(8)-H(8AB)	108.5
H(8A)-C(8)-H(8AB)	107.5
C(10)-C(9)-C(8)	114.68(19)
C(10)-C(9)-H(9A)	108.6
C(8)-C(9)-H(9A)	108.6
C(10)-C(9)-H(9AB)	108.6
C(8)-C(9)-H(9AB)	108.6
H(9A)-C(9)-H(9AB)	107.6
N(4)-C(10)-C(9)	115.1(2)
N(4)-C(10)-H(10A)	108.5
C(9)-C(10)-H(10A)	108.5
N(4)-C(10)-H(10B)	108.5
C(9)-C(10)-H(10B)	108.5
H(10A)-C(10)-H(10B)	107.5
N(4)-C(11)-D(11A)	109.5
N(4)-C(11)-D(11B)	109.5
D(11A)-C(11)-D(11B)	109.5
N(4)-C(11)-D(11C)	109.5
D(11A)-C(11)-D(11C)	109.5
D(11B)-C(11)-D(11C)	109.5
N(4)-C(12)-C(13)	110.38(19)
N(4)-C(12)-H(12A)	109.6
C(13)-C(12)-H(12A)	109.6
N(4)-C(12)-H(12B)	109.6
C(13)-C(12)-H(12B)	109.6
H(12A)-C(12)-H(12B)	108.1
N(1)-C(13)-C(12)	111.37(19)
N(1)-C(13)-H(13A)	109.4
C(12)-C(13)-H(13A)	109.4
N(1)-C(13)-H(13B)	109.4
C(12)-C(13)-H(13B)	109.4
H(13A)-C(13)-H(13B)	108.0
N(1)-C(14)-D(14A)	109.5
N(1)-C(14)-D(14B)	109.5
D(14A)-C(14)-D(14B)	109.5
N(1)-C(14)-D(14C)	109.5
D(14A)-C(14)-D(14C)	109.5
D(14B)-C(14)-D(14C)	109.5
C(1A)-N(1A)-C(14A)	116(3)
C(1A)-N(1A)-C(13A)	112(2)
C(14A)-N(1A)-C(13A)	114(3)
C(1A)-N(1A)-Co(1)	103(3)
C(14A)-N(1A)-Co(1)	109(3)
C(13A)-N(1A)-Co(1)	102(2)
C(5A)-N(2A)-C(3A)	104(3)
C(5A)-N(2A)-C(4A)	107(3)
C(3A)-N(2A)-C(4A)	107(2)
C(5A)-N(2A)-Co(1)	102(3)
C(3A)-N(2A)-Co(1)	121(3)
C(4A)-N(2A)-Co(1)	114(3)
C(7A)-N(3A)-C(6A)	109(3)
C(7A)-N(3A)-C(8A)	114(3)
C(6A)-N(3A)-C(8A)	112(2)
C(7A)-N(3A)-Co(1)	103(3)
C(6A)-N(3A)-Co(1)	104.2(18)
C(8A)-N(3A)-Co(1)	114(3)
C(12A)-N(4A)-C(11A)	107(3)
C(12A)-N(4A)-C(10A)	106(2)
C(11A)-N(4A)-C(10A)	108(3)
C(12A)-N(4A)-Co(1)	105(2)
C(11A)-N(4A)-Co(1)	114(3)
C(10A)-N(4A)-Co(1)	117(3)
N(1A)-C(1A)-C(2A)	121(3)
N(1A)-C(1A)-H(1AA)	107.0
C(2A)-C(1A)-H(1AA)	107.0
N(1A)-C(1A)-H(1AC)	107.0
C(2A)-C(1A)-H(1AC)	107.0
H(1AA)-C(1A)-H(1AC)	106.7
C(3A)-C(2A)-C(1A)	120(3)
C(3A)-C(2A)-H(2AA)	107.4
C(1A)-C(2A)-H(2AA)	107.4
C(3A)-C(2A)-H(2AC)	107.4
C(1A)-C(2A)-H(2AC)	107.4
H(2AA)-C(2A)-H(2AC)	106.9
C(2A)-C(3A)-N(2A)	118(3)
C(2A)-C(3A)-H(3AA)	107.8

N(2A)-C(3A)-H(3AA)	107.8	C(26)-C(25)-C(24)	120.97(17)
C(2A)-C(3A)-H(3AC)	107.8	C(26)-C(25)-C(28)	119.21(17)
N(2A)-C(3A)-H(3AC)	107.8	C(24)-C(25)-C(28)	119.82(17)
H(3AA)-C(3A)-H(3AC)	107.1	C(25)-C(26)-C(21)	122.60(17)
N(2A)-C(4A)-D(4AA)	109.5	C(25)-C(26)-H(26)	118.7
N(2A)-C(4A)-D(4AB)	109.5	C(21)-C(26)-H(26)	118.7
D(4AA)-C(4A)-D(4AB)	109.5	F(22A)-C(27)-F(21A)	112.8(17)
N(2A)-C(4A)-D(4AC)	109.5	F(21)-C(27)-F(23)	106.63(18)
D(4AA)-C(4A)-D(4AC)	109.5	F(21)-C(27)-F(22)	106.44(18)
D(4AB)-C(4A)-D(4AC)	109.5	F(23)-C(27)-F(22)	105.48(19)
N(2A)-C(5A)-C(6A)	115(3)	F(22A)-C(27)-F(23A)	101.1(17)
N(2A)-C(5A)-H(5AA)	108.6	F(21A)-C(27)-F(23A)	105.9(17)
C(6A)-C(5A)-H(5AA)	108.6	F(22A)-C(27)-C(23)	116.2(14)
N(2A)-C(5A)-H(5AC)	108.6	F(21A)-C(27)-C(23)	113.2(13)
C(6A)-C(5A)-H(5AC)	108.6	F(21)-C(27)-C(23)	113.45(17)
H(5AA)-C(5A)-H(5AC)	107.6	F(23)-C(27)-C(23)	113.29(18)
N(3A)-C(6A)-C(5A)	112(3)	F(22)-C(27)-C(23)	110.99(18)
N(3A)-C(6A)-H(6AA)	109.1	F(23A)-C(27)-C(23)	106.1(13)
C(5A)-C(6A)-H(6AA)	109.1	F(26)-C(28)-F(24)	107.4(2)
N(3A)-C(6A)-H(6AC)	109.1	F(24A)-C(28)-F(25A)	114.8(12)
C(5A)-C(6A)-H(6AC)	109.1	F(26)-C(28)-F(25)	107.6(2)
H(6AA)-C(6A)-H(6AC)	107.8	F(24)-C(28)-F(25)	104.62(19)
N(3A)-C(7A)-D(7AA)	109.5	F(24A)-C(28)-F(26A)	100.4(12)
N(3A)-C(7A)-D(7AB)	109.5	F(25A)-C(28)-F(26A)	98.3(11)
D(7AA)-C(7A)-D(7AB)	109.5	F(24A)-C(28)-C(25)	121.5(11)
N(3A)-C(7A)-D(7AC)	109.5	F(26)-C(28)-C(25)	112.31(19)
D(7AA)-C(7A)-D(7AC)	109.5	F(24)-C(28)-C(25)	112.84(18)
D(7AB)-C(7A)-D(7AC)	109.5	F(25A)-C(28)-C(25)	113.5(7)
N(3A)-C(8A)-C(9A)	117(3)	F(25)-C(28)-C(25)	111.56(17)
N(3A)-C(8A)-H(8AA)	108.2	F(26A)-C(28)-C(25)	103.4(7)
C(9A)-C(8A)-H(8AA)	108.2	C(36)-C(31)-C(32)	115.71(16)
N(3A)-C(8A)-H(8AC)	108.2	C(36)-C(31)-B(21)	121.14(16)
C(9A)-C(8A)-H(8AC)	108.2	C(32)-C(31)-B(21)	122.95(16)
H(8AA)-C(8A)-H(8AC)	107.3	C(33)-C(32)-C(31)	121.91(17)
C(10A)-C(9A)-C(8A)	115(3)	C(33)-C(32)-H(32)	119.0
C(10A)-C(9A)-H(9AA)	108.6	C(31)-C(32)-H(32)	119.0
C(8A)-C(9A)-H(9AA)	108.6	C(32)-C(33)-C(34)	121.13(17)
C(10A)-C(9A)-H(9AC)	108.6	C(32)-C(33)-C(37)	120.98(17)
C(8A)-C(9A)-H(9AC)	108.6	C(34)-C(33)-C(37)	117.89(17)
H(9AA)-C(9A)-H(9AC)	107.5	C(35)-C(34)-C(33)	117.76(17)
N(4A)-C(10A)-C(9A)	114(3)	C(35)-C(34)-H(34)	121.1
N(4A)-C(10A)-H(10C)	108.6	C(33)-C(34)-H(34)	121.1
C(9A)-C(10A)-H(10C)	108.6	C(34)-C(35)-C(36)	121.00(17)
N(4A)-C(10A)-H(10D)	108.6	C(34)-C(35)-C(38)	120.34(17)
C(9A)-C(10A)-H(10D)	108.6	C(36)-C(35)-C(38)	118.55(17)
H(10C)-C(10A)-H(10D)	107.6	C(35)-C(36)-C(31)	122.47(17)
N(4A)-C(11A)-D(11D)	109.5	C(35)-C(36)-H(36)	118.8
N(4A)-C(11A)-D(11E)	109.5	C(31)-C(36)-H(36)	118.8
D(11D)-C(11A)-D(11E)	109.5	F(32A)-C(37)-F(33A)	107.6(9)
N(4A)-C(11A)-D(11F)	109.5	F(31)-C(37)-F(32)	106.8(5)
D(11D)-C(11A)-D(11F)	109.5	F(32A)-C(37)-F(31A)	103.7(7)
D(11E)-C(11A)-D(11F)	109.5	F(33A)-C(37)-F(31A)	106.5(9)
N(4A)-C(12A)-C(13A)	110(3)	F(31)-C(37)-F(33)	106.2(5)
N(4A)-C(12A)-H(12C)	109.7	F(32)-C(37)-F(33)	105.4(4)
C(13A)-C(12A)-H(12C)	109.7	F(32A)-C(37)-C(33)	114.3(7)
N(4A)-C(12A)-H(12D)	109.7	F(33A)-C(37)-C(33)	114.2(10)
C(13A)-C(12A)-H(12D)	109.7	F(31)-C(37)-C(33)	113.2(5)
H(12C)-C(12A)-H(12D)	108.2	F(32)-C(37)-C(33)	114.0(4)
N(1A)-C(13A)-C(12A)	110(2)	F(31A)-C(37)-C(33)	109.8(7)
N(1A)-C(13A)-H(13C)	109.7	F(33)-C(37)-C(33)	110.6(4)
C(12A)-C(13A)-H(13C)	109.7	F(34)-C(38)-F(36)	107.03(17)
N(1A)-C(13A)-H(13D)	109.7	F(34)-C(38)-F(35)	106.29(18)
C(12A)-C(13A)-H(13D)	109.7	F(36)-C(38)-F(35)	105.91(18)
H(13C)-C(13A)-H(13D)	108.2	F(34)-C(38)-C(35)	113.36(17)
N(1A)-C(14A)-D(14D)	109.5	F(36)-C(38)-C(35)	112.59(17)
N(1A)-C(14A)-D(14E)	109.5	F(35)-C(38)-C(35)	111.17(17)
D(14D)-C(14A)-D(14E)	109.5	C(46)-C(41)-C(42)	115.78(16)
N(1A)-C(14A)-D(14F)	109.5	C(46)-C(41)-B(21)	122.52(16)
D(14D)-C(14A)-D(14F)	109.5	C(42)-C(41)-B(21)	121.41(16)
D(14E)-C(14A)-D(14F)	109.5	C(43)-C(42)-C(41)	122.07(17)
O(3)-S(1)-O(2)	116.49(10)	C(43)-C(42)-H(42)	119.0
O(3)-S(1)-O(1)	112.97(9)	C(41)-C(42)-H(42)	119.0
O(2)-S(1)-O(1)	113.80(9)	C(42)-C(43)-C(44)	120.95(17)
O(3)-S(1)-C(15)	105.57(10)	C(42)-C(43)-C(47)	121.18(17)
O(2)-S(1)-C(15)	105.17(10)	C(44)-C(43)-C(47)	117.86(17)
O(1)-S(1)-C(15)	100.81(10)	C(45)-C(44)-C(43)	117.78(17)
S(1)-O(1)-Co(1)	139.82(9)	C(45)-C(44)-H(44)	121.1
F(3)-C(15)-F(1)	108.2(2)	C(43)-C(44)-H(44)	121.1
F(3)-C(15)-F(2)	108.62(19)	C(44)-C(45)-C(46)	121.04(17)
F(1)-C(15)-F(2)	108.50(18)	C(44)-C(45)-C(48)	119.69(17)
F(3)-C(15)-S(1)	110.65(15)	C(46)-C(45)-C(48)	119.21(17)
F(1)-C(15)-S(1)	111.18(15)	C(45)-C(46)-C(41)	122.36(17)
F(2)-C(15)-S(1)	109.63(17)	C(45)-C(46)-H(46)	118.8
C(22)-C(21)-C(26)	115.41(16)	C(41)-C(46)-H(46)	118.8
C(22)-C(21)-B(21)	125.17(16)	F(41)-C(47)-F(42)	108.8(2)
C(26)-C(21)-B(21)	118.87(16)	F(42A)-C(47)-F(41A)	111.7(14)
C(23)-C(22)-C(21)	122.00(17)	F(41)-C(47)-F(43)	106.2(2)
C(23)-C(22)-H(22)	119.0	F(42)-C(47)-F(43)	103.34(18)
C(21)-C(22)-H(22)	119.0	F(42A)-C(47)-F(43A)	107.6(9)
C(24)-C(23)-C(22)	121.44(17)	F(41A)-C(47)-F(43A)	96.3(12)
C(24)-C(23)-C(27)	119.03(17)	F(42A)-C(47)-C(43)	121.0(8)
C(22)-C(23)-C(27)	119.45(17)	F(41)-C(47)-C(43)	114.5(3)
C(23)-C(24)-C(25)	117.56(17)	F(42)-C(47)-C(43)	112.67(17)
C(23)-C(24)-H(24)	121.2	F(41A)-C(47)-C(43)	110.2(13)
C(25)-C(24)-H(24)	121.2	F(43)-C(47)-C(43)	110.54(18)

F(43A)-C(47)-C(43)	106.9(5)
F(45A)-C(48)-F(44A)	106.9(13)
F(46)-C(48)-F(45)	106.7(5)
F(46)-C(48)-F(44)	107.1(4)
F(45)-C(48)-F(44)	106.7(4)
F(45A)-C(48)-F(46A)	106.6(15)
F(44A)-C(48)-F(46A)	101.3(12)
F(45A)-C(48)-C(45)	115.4(12)
F(44A)-C(48)-C(45)	115.0(8)
F(46)-C(48)-C(45)	112.5(4)
F(45)-C(48)-C(45)	112.2(4)
F(44)-C(48)-C(45)	111.3(3)
F(46A)-C(48)-C(45)	110.5(12)
C(56)-C(51)-C(52)	115.76(17)
C(56)-C(51)-B(21)	124.10(16)
C(52)-C(51)-B(21)	120.08(16)
C(53)-C(52)-C(51)	122.22(18)
C(53)-C(52)-H(52)	118.9
C(51)-C(52)-H(52)	118.9
C(52)-C(53)-C(54)	120.85(18)
C(52)-C(53)-C(57)	120.25(18)
C(54)-C(53)-C(57)	118.80(18)
C(55)-C(54)-C(53)	117.96(18)
C(55)-C(54)-H(54)	121.0
C(53)-C(54)-H(54)	121.0
C(54)-C(55)-C(56)	120.97(18)
C(54)-C(55)-C(58)	119.38(18)
C(56)-C(55)-C(58)	119.64(18)
C(51)-C(56)-C(55)	122.20(18)
C(51)-C(56)-H(56)	118.9
C(55)-C(56)-H(56)	118.9
F(51)-C(57)-F(53)	105.86(19)
F(51)-C(57)-F(52)	106.31(17)
F(53)-C(57)-F(52)	105.92(19)
F(51)-C(57)-C(53)	113.62(17)
F(53)-C(57)-C(53)	111.82(17)
F(52)-C(57)-C(53)	112.73(18)
F(54)-C(58)-F(56)	114.7(3)
F(54A)-C(58)-F(56A)	113.8(3)
F(54A)-C(58)-F(55A)	104.0(4)
F(56A)-C(58)-F(55A)	104.0(3)
F(55B)-C(58)-F(56B)	111.2(5)
F(55B)-C(58)-F(54B)	106.2(5)
F(56B)-C(58)-F(54B)	96.8(5)
F(54)-C(58)-F(55)	101.6(3)
F(56)-C(58)-F(55)	96.2(4)
F(55B)-C(58)-C(55)	120.0(4)
F(54A)-C(58)-C(55)	114.6(3)
F(54)-C(58)-C(55)	115.4(3)
F(56)-C(58)-C(55)	117.1(3)
F(56A)-C(58)-C(55)	111.5(2)
F(55A)-C(58)-C(55)	107.9(3)
F(56B)-C(58)-C(55)	110.4(3)
F(54B)-C(58)-C(55)	109.6(3)
F(55)-C(58)-C(55)	108.2(2)
C(31)-B(21)-C(21)	114.43(15)
C(31)-B(21)-C(51)	105.30(14)
C(21)-B(21)-C(51)	112.51(15)
C(31)-B(21)-C(41)	111.10(15)
C(21)-B(21)-C(41)	102.92(14)
C(51)-B(21)-C(41)	110.72(14)

Table S55. Anisotropic displacement parameters (Å^{2}x 10^3) for **5b-B$_{Ar}$F**.
The anisotropic displacement factor exponent takes the form: -2p^2[h^2 a*^{2}U^{11} + ... + 2 h k a* b* U^{12}]

	U11	U22	U33	U23	U13	U12
Co(1)	12(1)	10(1)	8(1)	0(1)	1(1)	2(1)
N(1)	21(1)	13(1)	24(1)	4(1)	-5(1)	-3(1)
N(2)	15(1)	17(1)	13(1)	3(1)	4(1)	4(1)
N(3)	13(1)	21(1)	12(1)	2(1)	0(1)	-1(1)
N(4)	27(1)	18(1)	15(1)	-2(1)	2(1)	7(1)
C(1)	24(1)	15(1)	36(1)	10(1)	-4(1)	-6(1)
C(2)	20(1)	26(1)	34(1)	16(1)	8(1)	-1(1)
C(3)	23(1)	22(1)	17(1)	9(1)	7(1)	6(1)
C(4)	22(1)	20(1)	16(1)	2(1)	8(1)	10(1)
C(5)	21(1)	25(1)	10(1)	-2(1)	1(1)	3(1)
C(6)	23(1)	21(1)	16(1)	-4(1)	-1(1)	-4(1)
C(7)	19(1)	29(1)	22(1)	7(1)	0(1)	-9(1)
C(8)	13(1)	32(1)	18(1)	3(1)	0(1)	3(1)
C(9)	17(1)	42(1)	22(1)	5(1)	6(1)	13(1)
C(10)	29(1)	30(1)	18(1)	0(1)	4(1)	18(1)
C(11)	35(1)	30(1)	12(1)	-1(1)	6(1)	12(1)
C(12)	48(2)	15(1)	27(1)	-7(1)	-1(1)	6(1)
C(13)	40(1)	15(1)	28(1)	-6(1)	-9(1)	-6(1)
C(14)	18(1)	24(1)	34(1)	9(1)	-9(1)	-7(1)
N(1A)	16(6)	17(6)	20(7)	7(6)	7(6)	3(5)
N(2A)	17(7)	24(7)	15(6)	1(5)	4(6)	-1(6)
N(3A)	23(7)	29(7)	17(8)	-1(6)	3(7)	7(6)
N(4A)	30(7)	15(7)	26(7)	4(7)	0(7)	0(6)
C(1A)	19(7)	21(8)	17(8)	1(7)	7(7)	6(7)
C(2A)	20(9)	21(8)	16(7)	-3(7)	4(7)	6(7)
C(3A)	21(9)	24(8)	16(7)	-3(6)	-1(7)	-1(8)
C(4A)	19(1)	29(1)	22(1)	7(1)	0(1)	-9(1)
C(5A)	17(8)	27(8)	16(8)	2(6)	4(7)	-1(7)
C(6A)	19(8)	30(7)	18(7)	1(7)	4(7)	7(6)
C(7A)	30(20)	34(19)	14(10)	-3(12)	5(11)	11(17)
C(8A)	33(8)	27(7)	21(9)	-4(8)	2(8)	8(7)
C(9A)	37(9)	21(9)	23(10)	3(9)	-2(8)	7(7)
C(10A)	37(8)	13(9)	23(10)	-2(9)	-1(8)	1(7)
C(11A)	30(18)	8(19)	27(9)	3(13)	-1(10)	-7(16)
C(12A)	25(8)	15(8)	30(7)	11(7)	-1(6)	0(7)
C(13A)	21(9)	18(6)	27(7)	14(7)	4(7)	2(7)
C(14A)	22(1)	20(1)	16(1)	2(1)	8(1)	10(1)
S(1)	16(1)	14(1)	12(1)	4(1)	-1(1)	1(1)
F(1)	31(1)	27(1)	35(1)	-9(1)	-1(1)	8(1)
F(2)	46(1)	27(1)	37(1)	8(1)	-18(1)	13(1)
F(3)	17(1)	35(1)	72(1)	7(1)	10(1)	3(1)
O(1)	20(1)	17(1)	12(1)	6(1)	1(1)	4(1)
O(2)	31(1)	19(1)	21(1)	-3(1)	-7(1)	1(1)
O(3)	26(1)	26(1)	19(1)	6(1)	6(1)	-4(1)
C(15)	18(1)	18(1)	30(1)	3(1)	-3(1)	4(1)
F(21)	23(1)	23(1)	45(1)	7(1)	18(1)	10(1)
F(22)	14(1)	48(1)	72(1)	-18(1)	11(1)	-10(1)
F(23)	37(1)	54(1)	36(1)	23(1)	27(1)	20(1)
F(21A)	23(1)	23(1)	45(1)	7(1)	18(1)	10(1)
F(22A)	14(1)	48(1)	72(1)	-18(1)	11(1)	-10(1)
F(23A)	37(1)	54(1)	36(1)	23(1)	27(1)	20(1)
F(24)	24(1)	34(1)	21(1)	14(1)	4(1)	9(1)
F(25)	16(1)	53(1)	56(1)	34(1)	9(1)	11(1)
F(26)	80(2)	23(1)	34(1)	-7(1)	-7(1)	30(1)
F(24A)	37(12)	39(11)	41(5)	5(7)	-14(6)	7(9)
F(25A)	16(1)	53(1)	56(1)	34(1)	9(1)	11(1)
F(26A)	30(7)	16(5)	52(10)	-1(6)	7(7)	14(5)
F(31)	18(2)	19(2)	46(3)	13(2)	-9(2)	-9(1)
F(32)	31(3)	60(4)	23(2)	14(2)	-14(1)	-20(2)
F(33)	10(2)	37(2)	68(4)	-15(2)	4(2)	0(1)
F(31A)	34(4)	74(8)	34(3)	-22(4)	-17(3)	-10(5)
F(32A)	16(3)	24(3)	81(8)	-2(4)	-11(4)	5(2)
F(33A)	18(4)	43(6)	61(7)	27(5)	-6(4)	-12(4)
F(34)	30(1)	27(1)	45(1)	26(1)	-12(1)	-8(1)
F(35)	76(1)	14(1)	38(1)	-4(1)	20(1)	-11(1)
F(36)	18(1)	23(1)	49(1)	15(1)	5(1)	-6(1)
F(41)	27(1)	30(2)	34(2)	-16(1)	-12(1)	17(1)
F(42)	46(1)	16(1)	44(1)	-14(1)	27(1)	-13(1)
F(43)	27(1)	31(1)	14(1)	-7(1)	2(1)	4(1)
F(41A)	16(4)	19(9)	18(7)	-3(6)	8(4)	9(4)
F(42A)	21(7)	44(10)	37(5)	-33(5)	-7(5)	6(6)
F(43A)	46(1)	16(1)	44(1)	-14(1)	27(1)	-13(1)
F(44)	16(1)	46(3)	36(1)	-9(2)	11(1)	0(1)
F(45)	16(2)	26(2)	29(1)	-6(1)	-9(1)	2(1)
F(46)	19(2)	21(1)	82(5)	-28(2)	-14(2)	8(1)
F(44A)	16(1)	46(3)	36(1)	-9(2)	11(1)	0(1)
F(45A)	13(5)	19(7)	46(7)	-17(7)	-13(5)	7(5)
F(46A)	19(6)	19(4)	41(7)	-4(4)	6(5)	7(4)
F(51)	40(1)	31(1)	32(1)	-4(1)	20(1)	-18(1)
F(52)	46(1)	45(1)	15(1)	-2(1)	14(1)	-16(1)
F(53)	18(1)	67(1)	74(1)	44(1)	18(1)	11(1)
F(54)	38(1)	21(1)	46(1)	-15(1)	12(1)	-12(1)
F(55)	38(1)	21(1)	46(1)	-15(1)	12(1)	-12(1)
F(56)	38(1)	21(1)	46(1)	-15(1)	12(1)	-12(1)
F(54A)	38(1)	21(1)	46(1)	-15(1)	12(1)	-12(1)
F(55A)	38(1)	21(1)	46(1)	-15(1)	12(1)	-12(1)
F(56A)	38(1)	21(1)	46(1)	-15(1)	12(1)	-12(1)
F(54B)	38(1)	21(1)	46(1)	-15(1)	12(1)	-12(1)
F(55B)	38(1)	21(1)	46(1)	-15(1)	12(1)	-12(1)
F(56B)	38(1)	21(1)	46(1)	-15(1)	12(1)	-12(1)

C(21)	10(1)	9(1)	11(1)	-1(1)	0(1)	-1(1)
C(22)	10(1)	9(1)	13(1)	0(1)	0(1)	0(1)
C(23)	10(1)	12(1)	15(1)	0(1)	4(1)	-1(1)
C(24)	14(1)	11(1)	15(1)	2(1)	3(1)	-1(1)
C(25)	13(1)	11(1)	15(1)	3(1)	2(1)	2(1)
C(26)	10(1)	12(1)	15(1)	1(1)	4(1)	1(1)
C(27)	15(1)	21(1)	27(1)	4(1)	10(1)	2(1)
C(28)	21(1)	18(1)	24(1)	10(1)	9(1)	7(1)
C(31)	11(1)	11(1)	9(1)	1(1)	2(1)	0(1)
C(32)	11(1)	9(1)	13(1)	1(1)	0(1)	-1(1)
C(33)	11(1)	12(1)	12(1)	-1(1)	-1(1)	-1(1)
C(34)	14(1)	12(1)	14(1)	3(1)	0(1)	2(1)
C(35)	14(1)	9(1)	14(1)	3(1)	2(1)	-1(1)
C(36)	11(1)	13(1)	13(1)	2(1)	1(1)	0(1)
C(37)	15(1)	16(1)	21(1)	3(1)	-5(1)	-2(1)
C(38)	18(1)	13(1)	21(1)	6(1)	0(1)	-2(1)
C(41)	11(1)	10(1)	9(1)	2(1)	2(1)	-1(1)
C(42)	12(1)	12(1)	11(1)	2(1)	3(1)	0(1)
C(43)	16(1)	9(1)	9(1)	1(1)	3(1)	0(1)
C(44)	15(1)	11(1)	12(1)	0(1)	0(1)	-1(1)
C(45)	11(1)	11(1)	14(1)	0(1)	0(1)	0(1)
C(46)	12(1)	10(1)	14(1)	-1(1)	1(1)	1(1)
C(47)	17(1)	16(1)	18(1)	-4(1)	5(1)	-1(1)
C(48)	12(1)	17(1)	27(1)	-9(1)	-3(1)	1(1)
C(51)	9(1)	13(1)	9(1)	2(1)	0(1)	0(1)
C(52)	12(1)	14(1)	11(1)	2(1)	-1(1)	-1(1)
C(53)	12(1)	19(1)	11(1)	3(1)	1(1)	0(1)
C(54)	18(1)	20(1)	14(1)	-2(1)	3(1)	1(1)
C(55)	18(1)	15(1)	16(1)	-4(1)	2(1)	-2(1)
C(56)	12(1)	15(1)	13(1)	0(1)	2(1)	-2(1)
C(57)	18(1)	25(1)	16(1)	4(1)	6(1)	0(1)
C(58)	32(1)	18(1)	33(1)	-9(1)	8(1)	-7(1)
B(21)	9(1)	10(1)	11(1)	2(1)	1(1)	0(1)

Table S56. Hydrogen coordinates (x 10^4) and isotropic displacement parameters ($Å^2$x 10^3) for **5b-B$_{Ar}$F**.

	x	y	z	U(eq)
H(1A)	11741	9375	7114	31
H(1AB)	10436	9345	6910	31
H(2A)	11455	9148	5848	31
H(2AB)	11874	8615	6290	31
H(3A)	9621	8823	5778	24
H(3AB)	10391	8477	5292	24
H(5A)	9113	7678	5331	23
H(5AB)	8425	8163	5630	23
H(6A)	7673	7367	6018	24
H(6AB)	8851	7171	6419	24
H(8A)	6537	7875	6714	26
H(8AB)	7299	8333	6429	26
H(9A)	6205	8604	7420	32
H(9AB)	6935	8222	8010	32
H(10A)	7839	9098	7263	31
H(10B)	7453	9143	8106	31
H(12A)	9414	9391	8716	36
H(12B)	9418	9460	7799	36
H(13A)	11241	9385	8380	34
H(13B)	10965	8840	8794	34
H(1AA)	9819	8240	5232	22
H(1AC)	8980	8411	5816	22
H(2AA)	8292	7675	5278	22
H(2AC)	9456	7389	5326	22
H(3AA)	9312	7035	6421	25
H(3AC)	8032	7116	6156	25
H(5AA)	6976	7623	6734	24
H(5AC)	7592	8131	6439	24
H(6AA)	6647	8451	7364	27
H(6AC)	6974	7960	7931	27
H(8AA)	7390	9194	8149	32
H(8AC)	7753	9167	7300	32
H(9AA)	8721	9822	8107	33
H(9AC)	9259	9357	8654	33
H(10C)	9611	9451	7070	30
H(10D)	10428	9706	7760	30
H(12C)	12010	8681	7338	28
H(12D)	11693	9294	7168	28
H(13C)	10283	9027	6173	26
H(13D)	11452	8810	5986	26
H(22)	2944	5509	8150	13
H(24)	3588	4343	9762	16
H(26)	5732	4647	8216	15
H(32)	2672	5399	6793	13
H(34)	1999	6795	5653	16
H(36)	4993	6544	6865	15
H(42)	4861	6419	8323	13
H(44)	8048	6605	9238	15
H(46)	7168	5398	7743	14
H(52)	6390	5751	6395	15
H(54)	6596	4402	5137	21
H(56)	4597	4462	6832	16

[(TMC)Co^{II}(ClO$_4$)]B$_{Ar}$F, **6a-B$_{Ar}$F**

The crystal structure of **6a-B$_{Ar}$F** was solved in the monoclinic space group *P*2$_1$/*n*. The asymmetric unit contains two complexes, two non-coordinated anions and four diethyl ether molecules. Both ligands and the coordinates perchlorate anions were found to be fully disordered, the B$_{Ar}$F anions were found to have 11 disordered CF_3 groups in total. All of the disorders were refined with the help of 1,2 and 1,3 same distance restraints, similarity restraints on anisotropic displacement parameters, advanced rigid bond restraints and with setting some anisotropic displacement parameters to the same values. All disorder ratios were refined freely and converged to 0.714(3) and 0.651(6) (ligands and perchlorate anions) and 0.52(1), 0.49(3), 0.61(1), 0.674(5), 0.77(6), 0.57(4), 0.75(2), 0.416(6), 0.65(2), 0.58(1) and 0.73(1).

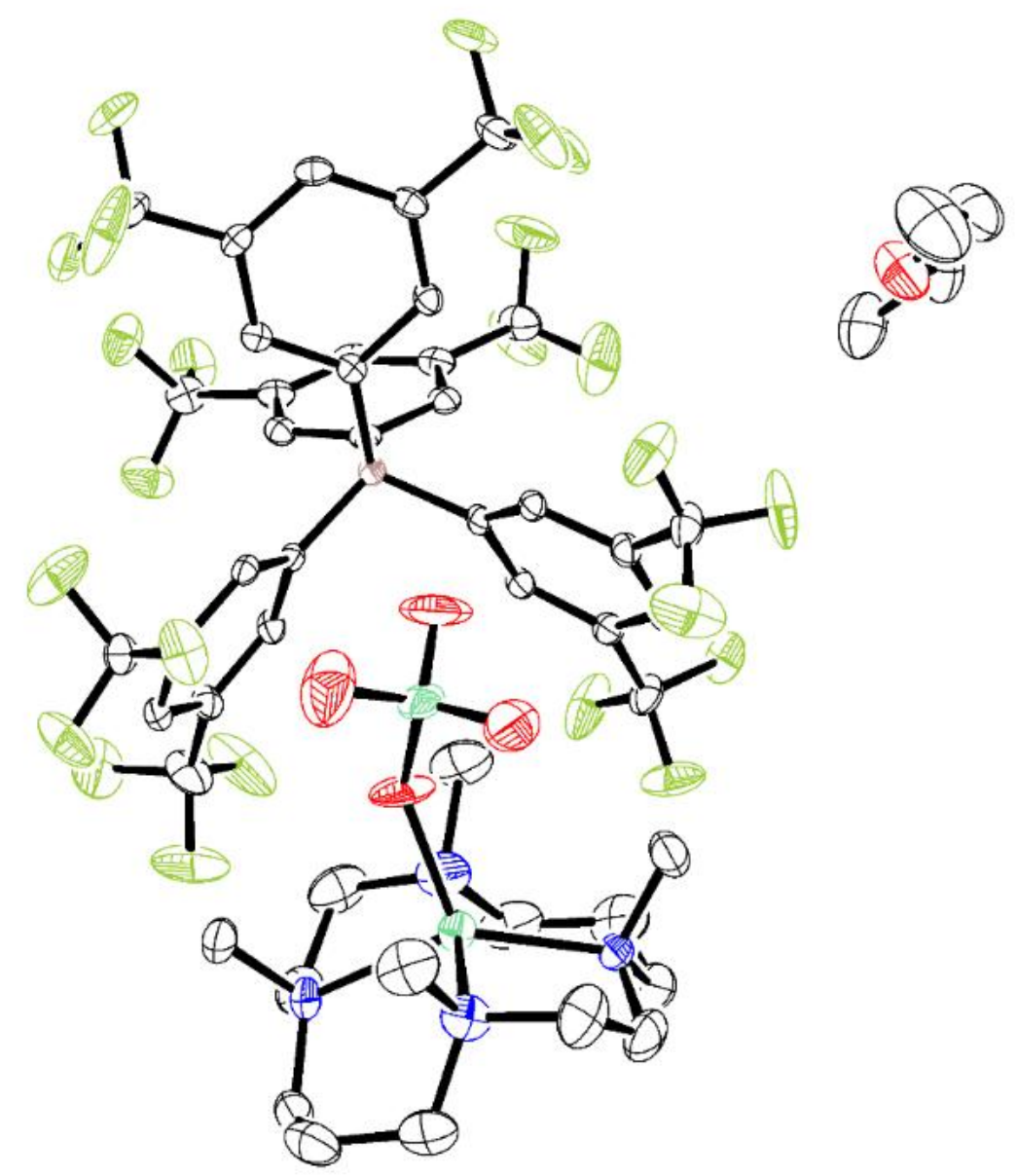

Figure S12. Molecular structure of **6a-B$_{Ar}$F**.

Table S57. Crystal data and structure refinement for **6a-B$_{Ar}$F**.

CCDC No	2062718	
Empirical formula	$C_{108}H_{128}B_2Cl_2Co_2F_{48}N_8O_{12}$	
Formula weight	2852.56	
Temperature	100(2) K	
Wavelength	0.71073 Å	
Crystal system	Monoclinic	
Space group	*P*2$_1$/*n*	
Unit cell dimensions	a = 16.6665(9) Å	α = 90°
	b = 27.0554(14) Å	β = 90.255(2)°
	c = 27.6034(14) Å	γ = 90°
Volume	12446.8(11) Å^3	
Z	4	
Density (calculated)	1.522 Mg/m^3	
Absorption coefficient	0.441 mm^{-1}	
F(000)	5832	
Crystal size	0.990 x 0.116 x 0.115 mm^3	
Theta range for data collection	2.108 to 26.732°.	
Index ranges	-21 ≤ h ≤ 21, -34 ≤ k ≤ 34, -34 ≤ l ≤ 34	
Reflections collected	1291059	
Independent reflections	26410 [R(int) = 0.1111]	
Completeness to theta = 25.242°	99.9 %	
Absorption correction	Semi-empirical from equivalents	
Refinement method	Full-matrix least-squares on F^2	
Data / restraints / parameters	26410 / 12135 / 2279	
Goodness-of-fit on F^2	1.087	
Final R indices [I>2σ(I)]	R1 = 0.0950, wR2 = 0.2227	
R indices (all data)	R1 = 0.1117, wR2 = 0.2358	
Largest diff. peak and hole	3.623 and -1.856 eÅ^{-3}	

Table S58. Atomic coordinates (x 10^4) and equivalent isotropic displacement parameters ($Å^2$x 10^3) for **6a-B$_{ArF}$**. U(eq) is defined as one third of the trace of the orthogonalized U^{ij} tensor.

	x	y	z	U(eq)
Co(1)	286(1)	7008(1)	-201(1)	24(1)
Cl(1)	2282(6)	6954(4)	-189(4)	37(1)
O(1)	1451(5)	6797(5)	-128(5)	47(3)
O(2)	2288(12)	7426(6)	-415(7)	56(4)
O(3)	2674(8)	6953(7)	262(5)	73(4)
O(4)	2657(10)	6605(7)	-498(6)	73(5)
N(1)	78(6)	6775(3)	536(3)	39(2)
N(2)	-3(4)	7737(2)	-66(2)	30(1)
N(3)	283(6)	7202(3)	-950(3)	34(2)
N(4)	-194(6)	6272(3)	-359(3)	30(2)
C(1)	-611(7)	7032(4)	770(4)	49(2)
C(2)	-549(7)	7584(5)	768(3)	54(3)
C(3)	-669(5)	7828(3)	281(3)	44(2)
C(4)	722(5)	8001(3)	95(4)	50(3)
C(5)	-252(5)	7947(3)	-550(3)	42(2)
C(6)	309(7)	7749(3)	-940(4)	47(2)
C(7)	992(7)	7034(5)	-1230(5)	49(3)
C(8)	-455(6)	7037(4)	-1221(3)	39(2)
C(9)	-586(7)	6480(4)	-1218(4)	44(2)
C(10)	-832(7)	6260(5)	-740(4)	36(2)
C(11)	448(9)	5926(5)	-495(6)	43(3)
C(12)	-591(5)	6103(3)	102(3)	41(2)
C(13)	-72(6)	6234(3)	524(3)	47(2)
C(14)	802(8)	6845(6)	853(5)	49(3)
Cl(1A)	2314(11)	6923(9)	-129(8)	37(1)
O(1A)	1486(9)	6751(7)	-142(8)	26(3)
O(2A)	2340(20)	7419(10)	-307(14)	53(6)
O(3A)	2555(13)	6923(11)	364(7)	41(4)
O(4A)	2793(14)	6608(12)	-406(8)	38(4)
N(1A)	-81(10)	6862(5)	523(6)	33(3)
N(2A)	496(9)	7811(5)	-19(4)	45(3)
N(3A)	117(10)	7293(5)	-891(6)	37(3)
N(4A)	-58(11)	6302(6)	-355(5)	30(3)
C(1A)	-632(11)	7243(6)	736(7)	37(3)
C(2A)	-313(13)	7760(6)	736(6)	45(4)
C(3A)	-215(10)	7996(6)	243(5)	47(3)
C(4A)	1221(10)	7972(6)	264(6)	50(4)
C(5A)	561(10)	8067(5)	-502(5)	48(3)
C(6A)	21(12)	7837(6)	-860(7)	48(4)
C(7A)	755(12)	7174(8)	-1228(8)	43(4)
C(8A)	-687(10)	7111(6)	-1081(7)	39(3)
C(9A)	-760(13)	6549(7)	-1108(7)	40(4)
C(10A)	-827(14)	6282(10)	-624(7)	33(3)
C(11A)	547(17)	6016(11)	-637(9)	44(6)
C(12A)	-112(10)	6060(5)	134(5)	34(3)
C(13A)	-560(8)	6390(5)	473(5)	33(2)
C(14A)	585(12)	6748(10)	867(11)	41(5)
Co(21)	9704(1)	1903(1)	5239(1)	19(1)
Cl(21)	7716(3)	1967(2)	5147(2)	44(1)
O(21)	8510(3)	1986(3)	5327(3)	58(2)
O(22)	7806(5)	2268(4)	4691(3)	91(3)
O(23)	7541(6)	1488(3)	4979(3)	56(2)
O(24)	7143(3)	2176(3)	5418(3)	61(2)
N(21)	9946(3)	2608(2)	4922(2)	27(1)
N(22)	10147(3)	2105(2)	5926(2)	25(1)
N(23)	9670(3)	1159(2)	5564(2)	31(1)
N(24)	10000(3)	1618(2)	4556(2)	26(1)
C(21)	10747(4)	2801(2)	5073(3)	32(1)
C(22)	10847(5)	2875(3)	5604(3)	35(2)
C(23)	10891(4)	2406(2)	5908(3)	33(1)
C(24)	9509(4)	2383(3)	6181(2)	38(2)
C(25)	10353(5)	1644(3)	6198(2)	36(2)
C(26)	9737(7)	1258(3)	6101(3)	40(2)
C(27)	8939(5)	860(3)	5510(3)	46(2)
C(28)	10376(5)	861(3)	5407(3)	36(2)
C(29)	10390(4)	764(2)	4867(3)	35(1)
C(30)	10612(4)	1202(3)	4555(3)	31(1)
C(31)	9266(4)	1450(3)	4302(2)	38(2)
C(32)	10384(5)	2034(3)	4286(3)	32(2)
C(33)	9960(6)	2500(3)	4381(3)	36(2)
C(34)	9351(5)	3011(3)	4994(3)	42(2)
Cl(2A)	7728(8)	1904(6)	5233(5)	47(3)
O(21A)	8516(7)	2107(7)	5116(6)	58(2)
O(22A)	7698(7)	1766(5)	5725(4)	46(3)
O(23A)	7209(8)	2331(6)	5169(7)	61(2)
O(24A)	7459(16)	1627(9)	4844(8)	75(6)
N(21A)	10132(7)	2643(4)	5325(5)	37(2)
N(22A)	9762(8)	1735(5)	5978(5)	44(3)
N(23A)	9939(8)	1171(5)	5120(5)	45(3)
N(24A)	9805(8)	2086(5)	4457(5)	44(3)
C(21A)	10785(11)	2687(8)	5699(6)	39(3)
C(22A)	10601(12)	2466(6)	6184(6)	44(3)
C(23A)	10518(10)	1912(6)	6202(7)	45(3)
C(24A)	9082(11)	1896(9)	6292(7)	57(5)
C(25A)	9791(18)	1179(7)	5998(8)	51(4)
C(26A)	10252(14)	977(8)	5582(6)	53(4)
C(27A)	9276(11)	868(9)	4932(10)	69(6)
C(28A)	10639(12)	1100(8)	4765(7)	46(3)
C(29A)	10532(12)	1338(6)	4275(6)	47(3)
C(30A)	10532(12)	1882(7)	4223(10)	48(4)

C(31A)	9094(10)	1933(10)	4172(7)	63(6)
C(32A)	9874(16)	2636(6)	4454(8)	46(4)
C(33A)	10413(12)	2814(7)	4848(6)	49(3)
C(34A)	9453(11)	2966(8)	5478(9)	54(5)
F(41)	2223(14)	7102(5)	3717(5)	71(4)
F(42)	3219(5)	6708(8)	3996(7)	72(5)
F(43)	2061(9)	6547(5)	4242(4)	59(3)
F(41A)	1956(7)	7019(5)	3787(7)	56(3)
F(42A)	3196(5)	6896(5)	3778(5)	54(3)
F(43A)	2468(16)	6509(5)	4287(3)	81(5)
F(44)	2099(2)	4805(1)	3812(1)	49(1)
F(45)	1553(2)	4690(1)	3116(1)	50(1)
F(46)	2818(2)	4631(1)	3196(1)	43(1)
F(51)	-308(3)	5357(3)	2018(2)	76(2)
F(52)	-18(3)	4690(2)	1644(2)	63(1)
F(53)	-572(2)	5251(2)	1264(2)	69(2)
F(51A)	-36(11)	5087(8)	2106(4)	76(2)
F(52A)	-335(10)	4821(6)	1417(7)	63(1)
F(53A)	-558(8)	5569(5)	1639(8)	63(1)
F(54)	2389(2)	5726(1)	156(1)	45(1)
F(55)	1642(2)	5096(1)	114(1)	51(1)
F(56)	2803(2)	5054(2)	446(1)	57(1)
F(61)	4592(2)	4874(1)	1756(2)	54(1)
F(62)	5196(3)	5284(1)	1202(1)	77(2)
F(63)	5732(2)	5173(1)	1889(1)	43(1)
F(64)	5945(2)	6855(1)	2518(1)	53(1)
F(65)	5437(2)	7289(1)	1954(1)	58(1)
F(66)	4874(2)	7257(1)	2651(1)	49(1)
F(71)	-135(4)	7135(2)	2503(4)	60(3)
F(72)	-317(5)	7673(4)	1939(3)	51(2)
F(73)	262(4)	7870(3)	2596(2)	36(1)
F(71A)	-287(8)	7116(5)	2198(12)	52(5)
F(72A)	-182(14)	7840(9)	1982(9)	43(5)
F(73A)	217(10)	7656(12)	2691(5)	46(5)
F(74)	2200(4)	8043(3)	831(2)	68(3)
F(75)	3296(3)	7854(2)	1155(3)	56(2)
F(76)	2572(6)	8417(2)	1465(3)	57(2)
F(74A)	2852(8)	7845(3)	870(3)	58(4)
F(75A)	3289(6)	8088(4)	1531(4)	68(4)
F(76A)	2260(8)	8419(4)	1252(6)	60(4)
C(41)	2345(2)	6069(2)	2599(1)	18(1)
C(42)	2394(2)	6415(2)	2976(2)	20(1)
C(43)	2393(2)	6271(2)	3461(1)	23(1)
C(44)	2327(2)	5781(2)	3590(2)	25(1)
C(45)	2270(2)	5433(2)	3224(2)	22(1)
C(46)	2289(2)	5576(2)	2742(1)	19(1)
C(47)	2485(3)	6660(2)	3840(2)	33(1)
C(48)	2182(3)	4896(2)	3340(2)	28(1)
C(51)	1970(2)	5874(1)	1663(1)	14(1)
C(52)	1216(2)	5679(1)	1792(2)	19(1)
C(53)	774(2)	5388(2)	1475(2)	22(1)
C(54)	1067(2)	5270(2)	1020(2)	21(1)
C(55)	1798(2)	5461(1)	883(1)	18(1)
C(56)	2236(2)	5764(1)	1197(1)	16(1)
C(57)	-28(3)	5191(2)	1621(2)	40(1)
C(58)	2149(3)	5330(2)	404(2)	25(1)
C(61)	3436(2)	6210(1)	1970(1)	15(1)
C(62)	3829(2)	5780(1)	1827(1)	16(1)
C(63)	4662(2)	5736(2)	1843(1)	18(1)
C(64)	5141(2)	6124(2)	2003(1)	20(1)
C(65)	4761(2)	6554(2)	2147(1)	19(1)
C(66)	3933(2)	6598(2)	2132(1)	18(1)
C(67)	5048(3)	5274(2)	1667(2)	26(1)
C(68)	5251(3)	6987(2)	2314(2)	31(1)
C(71)	2096(2)	6792(1)	1937(1)	16(1)
C(72)	1351(2)	6925(2)	2137(1)	18(1)
C(73)	1003(2)	7382(2)	2046(2)	21(1)
C(74)	1395(2)	7735(2)	1771(2)	22(1)
C(75)	2125(2)	7610(1)	1568(1)	20(1)
C(76)	2458(2)	7143(1)	1643(1)	17(1)
C(77)	201(3)	7506(2)	2257(2)	28(1)
C(78)	2572(3)	7983(2)	1277(2)	31(1)
B(41)	2459(2)	6238(2)	2034(2)	15(1)
F(81)	5095(9)	3941(5)	2315(4)	33(2)
F(82)	4923(8)	3753(3)	3057(3)	56(2)
F(83)	4030(4)	4173(5)	2683(6)	77(3)
F(81A)	5184(15)	3864(9)	2391(8)	33(4)
F(82A)	4563(14)	3841(6)	3066(5)	56(2)
F(83A)	4114(8)	4272(8)	2494(7)	55(4)
F(84)	4119(6)	5899(6)	3180(8)	58(4)
F(85)	5236(8)	6263(4)	3258(4)	30(2)
F(86)	4775(16)	5843(7)	3842(3)	69(4)
F(84A)	4199(8)	5953(8)	3097(5)	36(3)
F(85A)	5276(11)	6249(6)	3392(14)	70(5)
F(86A)	4534(16)	5758(11)	3805(5)	64(5)
F(91)	7659(11)	5378(4)	4897(6)	77(5)
F(92)	7075(5)	5991(4)	4600(3)	57(1)
F(93)	8201(6)	6086(4)	4924(3)	57(1)
F(91A)	7377(7)	5410(4)	4807(4)	68(3)
F(92A)	7422(4)	6177(3)	4668(2)	57(1)
F(93A)	8424(4)	5815(3)	4983(2)	57(1)
F(94)	10439(6)	6122(4)	3797(3)	59(3)
F(95)	9874(3)	6456(2)	3212(3)	71(2)
F(96)	10373(3)	5757(3)	3104(3)	62(2)
F(94A)	10368(12)	6269(7)	3724(7)	65(5)

F(95A)	10042(7)	6129(6)	2982(4)	71(2)
F(96A)	10487(6)	5571(4)	3406(5)	62(2)
F(101)	7110(2)	6632(2)	1816(2)	72(1)
F(102)	7810(3)	6459(2)	1197(1)	76(1)
F(103)	8367(2)	6571(2)	1881(2)	66(1)
F(104)	6856(5)	4262(3)	1238(4)	58(1)
F(105)	6965(9)	4851(7)	746(4)	65(3)
F(106)	8023(4)	4476(3)	948(3)	58(1)
F(04A)	6691(6)	4345(4)	1272(5)	58(1)
F(05A)	7161(14)	4792(10)	705(5)	65(3)
F(06A)	7932(5)	4279(4)	1133(4)	58(1)
F(111)	9596(5)	3525(4)	2206(3)	93(3)
F(112)	10249(6)	4097(4)	2561(5)	60(3)
F(113)	10186(3)	3364(2)	2865(3)	60(2)
F(11B)	9575(15)	3779(10)	2123(4)	93(3)
F(12B)	10329(15)	4062(10)	2665(13)	47(5)
F(13B)	10028(10)	3290(4)	2624(8)	60(2)
F(114)	7034(6)	3071(5)	3608(4)	93(4)
F(115)	8146(7)	2933(4)	3912(5)	86(4)
F(116)	7387(11)	3489(4)	4207(4)	102(5)
F(14B)	7751(11)	2858(3)	3774(6)	102(5)
F(15B)	8030(9)	3413(6)	4282(3)	102(4)
F(16B)	6926(5)	3421(5)	3966(5)	85(5)
C(81)	6542(2)	4978(1)	3030(1)	14(1)
C(82)	6081(2)	4570(1)	2883(1)	17(1)
C(83)	5247(2)	4580(2)	2881(1)	19(1)
C(84)	4833(2)	4994(2)	3034(1)	19(1)
C(85)	5276(2)	5399(1)	3185(1)	17(1)
C(86)	6106(2)	5391(1)	3179(1)	15(1)
C(87)	4805(3)	4127(2)	2721(2)	33(1)
C(88)	4842(2)	5844(2)	3367(2)	29(1)
C(91)	7984(2)	5338(1)	3344(1)	16(1)
C(92)	7712(2)	5414(1)	3815(1)	17(1)
C(93)	8165(2)	5673(1)	4158(1)	20(1)
C(94)	8916(2)	5858(2)	4042(2)	23(1)
C(95)	9194(2)	5789(2)	3575(2)	22(1)
C(96)	8738(2)	5535(2)	3235(2)	19(1)
C(97)	7823(3)	5770(2)	4650(2)	32(1)
C(98)	9986(3)	5994(2)	3429(2)	40(1)
C(101)	7603(2)	5180(2)	2395(2)	20(1)
C(102)	7672(2)	5676(2)	2263(2)	22(1)
C(103)	7654(3)	5834(2)	1783(2)	29(1)
C(104)	7551(3)	5496(2)	1411(2)	35(1)
C(105)	7474(3)	5005(2)	1530(2)	35(1)
C(106)	7508(2)	4846(2)	2010(2)	27(1)
C(107)	7736(3)	6369(2)	1666(2)	40(1)
C(108)	7340(3)	4626(2)	1138(2)	50(1)
C(111)	7925(2)	4444(2)	3036(2)	19(1)
C(112)	8628(2)	4303(2)	2797(2)	25(1)
C(113)	9009(3)	3856(2)	2895(2)	33(1)
C(114)	8704(3)	3527(2)	3225(2)	36(1)
C(115)	8016(3)	3663(2)	3472(2)	34(1)
C(116)	7643(3)	4110(2)	3382(2)	25(1)
C(117)	9749(3)	3729(2)	2619(2)	51(1)
C(118)	7674(4)	3318(2)	3830(2)	55(1)
B(81)	7515(3)	4988(2)	2952(2)	17(1)
O(1S)	7591(2)	7531(1)	3143(1)	34(1)
C(1S)	7713(4)	8351(2)	2865(3)	64(2)
C(2S)	7847(4)	7819(2)	2755(3)	56(2)
C(3S)	7736(3)	7024(2)	3077(2)	39(1)
C(4S)	7350(4)	6742(2)	3470(2)	45(1)
O(11S)	5336(2)	4273(2)	4621(1)	46(1)
C(11S)	4135(5)	4243(4)	4181(3)	104(4)
C(12S)	4902(5)	4508(3)	4249(3)	78(2)
C(13S)	6076(4)	4513(3)	4714(2)	65(2)
C(14S)	6510(5)	4254(4)	5100(3)	80(3)
O(21S)	5304(2)	9212(2)	465(1)	46(1)
C(21S)	4430(4)	8929(2)	1073(2)	49(1)
C(22S)	4988(4)	9329(2)	922(2)	47(1)
C(23S)	5862(4)	9567(2)	301(2)	49(1)
C(24S)	6113(5)	9447(3)	-198(3)	71(2)
O(31S)	7445(3)	1347(2)	6862(2)	64(1)
C(31S)	7561(4)	552(2)	6579(3)	57(2)
C(32S)	7294(5)	851(2)	6969(2)	64(2)
C(33S)	7254(7)	1639(3)	7251(3)	88(3)
C(34S)	7240(6)	2148(3)	7156(3)	90(3)

Table S59. Bond lengths [Å] and angles [°] for **6a-B$_{Ar}$F**.

Co(1)-O(1)	2.032(9)	C(5A)-H(5AC)	0.9900
Co(1)-N(4A)	2.039(16)	C(6A)-H(6AA)	0.9900
Co(1)-N(2)	2.064(6)	C(6A)-H(6AC)	0.9900
Co(1)-N(3A)	2.073(15)	C(7A)-H(7AA)	0.9800
Co(1)-O(1A)	2.122(15)	C(7A)-H(7AB)	0.9800
Co(1)-N(1A)	2.129(15)	C(7A)-H(7AC)	0.9800
Co(1)-N(3)	2.133(9)	C(8A)-C(9A)	1.528(15)
Co(1)-N(1)	2.158(9)	C(8A)-H(8AA)	0.9900
Co(1)-N(4)	2.190(9)	C(8A)-H(8AC)	0.9900
Co(1)-N(2A)	2.257(13)	C(9A)-C(10A)	1.525(15)
Cl(1)-O(3)	1.403(8)	C(9A)-H(9AA)	0.9900
Cl(1)-O(4)	1.418(9)	C(9A)-H(9AC)	0.9900
Cl(1)-O(2)	1.422(8)	C(10A)-H(10C)	0.9900
Cl(1)-O(1)	1.459(8)	C(10A)-H(10D)	0.9900
N(1)-C(13)	1.485(10)	C(11A)-H(11D)	0.9800
N(1)-C(1)	1.492(10)	C(11A)-H(11E)	0.9800
N(1)-C(14)	1.499(10)	C(11A)-H(11F)	0.9800
N(2)-C(4)	1.473(9)	C(12A)-C(13A)	1.497(14)
N(2)-C(3)	1.489(9)	C(12A)-H(12C)	0.9900
N(2)-C(5)	1.508(9)	C(12A)-H(12D)	0.9900
N(3)-C(6)	1.482(10)	C(13A)-H(13C)	0.9900
N(3)-C(7)	1.486(10)	C(13A)-H(13D)	0.9900
N(3)-C(8)	1.505(10)	C(14A)-H(14D)	0.9800
N(4)-C(11)	1.471(10)	C(14A)-H(14E)	0.9800
N(4)-C(10)	1.493(9)	C(14A)-H(14F)	0.9800
N(4)-C(12)	1.509(10)	Co(21)-O(21)	2.018(5)
C(1)-C(2)	1.499(13)	Co(21)-N(23A)	2.047(12)
C(1)-H(1A)	0.9900	Co(21)-O(21A)	2.081(13)
C(1)-H(1AB)	0.9900	Co(21)-N(22A)	2.091(13)
C(2)-C(3)	1.511(12)	Co(21)-N(24)	2.100(5)
C(2)-H(2A)	0.9900	Co(21)-N(22)	2.104(5)
C(2)-H(2AB)	0.9900	Co(21)-N(21A)	2.138(12)
C(3)-H(3A)	0.9900	Co(21)-N(21)	2.139(5)
C(3)-H(3AB)	0.9900	Co(21)-N(23)	2.205(5)
C(4)-H(4A)	0.9800	Co(21)-N(24A)	2.221(13)
C(4)-H(4B)	0.9800	Cl(21)-O(24)	1.341(8)
C(4)-H(4C)	0.9800	Cl(21)-O(23)	1.407(9)
C(5)-C(6)	1.525(11)	Cl(21)-O(21)	1.414(7)
C(5)-H(5A)	0.9900	Cl(21)-O(22)	1.506(9)
C(5)-H(5AB)	0.9900	N(21)-C(34)	1.488(7)
C(6)-H(6A)	0.9900	N(21)-C(21)	1.491(8)
C(6)-H(6AB)	0.9900	N(21)-C(33)	1.520(9)
C(7)-H(7A)	0.9800	N(22)-C(24)	1.484(8)
C(7)-H(7B)	0.9800	N(22)-C(23)	1.485(8)
C(7)-H(7C)	0.9800	N(22)-C(25)	1.495(8)
C(8)-C(9)	1.521(11)	N(23)-C(27)	1.470(8)
C(8)-H(8A)	0.9900	N(23)-C(28)	1.493(8)
C(8)-H(8AB)	0.9900	N(23)-C(26)	1.508(9)
C(9)-C(10)	1.506(11)	N(24)-C(31)	1.479(8)
C(9)-H(9A)	0.9900	N(24)-C(32)	1.494(8)
C(9)-H(9AB)	0.9900	N(24)-C(30)	1.519(8)
C(10)-H(10A)	0.9900	C(21)-C(22)	1.487(10)
C(10)-H(10B)	0.9900	C(21)-H(21A)	0.9900
C(11)-H(11A)	0.9800	C(21)-H(21B)	0.9900
C(11)-H(11B)	0.9800	C(22)-C(23)	1.523(9)
C(11)-H(11C)	0.9800	C(22)-H(22A)	0.9900
C(12)-C(13)	1.491(11)	C(22)-H(22B)	0.9900
C(12)-H(12A)	0.9900	C(23)-H(23A)	0.9900
C(12)-H(12B)	0.9900	C(23)-H(23B)	0.9900
C(13)-H(13A)	0.9900	C(24)-H(24A)	0.9800
C(13)-H(13B)	0.9900	C(24)-H(24B)	0.9800
C(14)-H(14A)	0.9800	C(24)-H(24C)	0.9800
C(14)-H(14B)	0.9800	C(25)-C(26)	1.488(11)
C(14)-H(14C)	0.9800	C(25)-H(25A)	0.9900
Cl(1A)-O(4A)	1.400(13)	C(25)-H(25B)	0.9900
Cl(1A)-O(3A)	1.417(13)	C(26)-H(26A)	0.9900
Cl(1A)-O(2A)	1.429(13)	C(26)-H(26B)	0.9900
Cl(1A)-O(1A)	1.457(13)	C(27)-H(27A)	0.9800
N(1A)-C(14A)	1.490(15)	C(27)-H(27B)	0.9800
N(1A)-C(1A)	1.503(14)	C(27)-H(27C)	0.9800
N(1A)-C(13A)	1.510(13)	C(28)-C(29)	1.513(9)
N(2A)-C(3A)	1.479(14)	C(28)-H(28A)	0.9900
N(2A)-C(4A)	1.502(15)	C(28)-H(28B)	0.9900
N(2A)-C(5A)	1.506(14)	C(29)-C(30)	1.513(9)
N(3A)-C(7A)	1.453(15)	C(29)-H(29A)	0.9900
N(3A)-C(6A)	1.485(14)	C(29)-H(29B)	0.9900
N(3A)-C(8A)	1.519(13)	C(30)-H(30A)	0.9900
N(4A)-C(10A)	1.479(14)	C(30)-H(30B)	0.9900
N(4A)-C(11A)	1.493(14)	C(31)-H(31A)	0.9800
N(4A)-C(12A)	1.503(13)	C(31)-H(31B)	0.9800
C(1A)-C(2A)	1.496(15)	C(31)-H(31C)	0.9800
C(1A)-H(1AA)	0.9900	C(32)-C(33)	1.471(10)
C(1A)-H(1AC)	0.9900	C(32)-H(32A)	0.9900
C(2A)-C(3A)	1.514(15)	C(32)-H(32B)	0.9900
C(2A)-H(2AA)	0.9900	C(33)-H(33A)	0.9900
C(2A)-H(2AC)	0.9900	C(33)-H(33B)	0.9900
C(3A)-H(3AA)	0.9900	C(34)-H(34A)	0.9800
C(3A)-H(3AC)	0.9900	C(34)-H(34B)	0.9800
C(4A)-H(4AA)	0.9800	C(34)-H(34C)	0.9800
C(4A)-H(4AB)	0.9800	Cl(2A)-O(24A)	1.381(15)
C(4A)-H(4AC)	0.9800	Cl(2A)-O(22A)	1.411(14)
C(5A)-C(6A)	1.472(15)	Cl(2A)-O(23A)	1.453(15)
C(5A)-H(5AA)	0.9900	Cl(2A)-O(21A)	1.461(13)

N(21A)-C(33A)	1.475(15)
N(21A)-C(34A)	1.491(14)
N(21A)-C(21A)	1.501(15)
N(22A)-C(23A)	1.482(15)
N(22A)-C(24A)	1.495(15)
N(22A)-C(25A)	1.506(16)
N(23A)-C(27A)	1.469(15)
N(23A)-C(26A)	1.474(15)
N(23A)-C(28A)	1.537(15)
N(24A)-C(31A)	1.479(14)
N(24A)-C(30A)	1.484(15)
N(24A)-C(32A)	1.493(15)
C(21A)-C(22A)	1.498(16)
C(21A)-H(21C)	0.9900
C(21A)-H(21D)	0.9900
C(22A)-C(23A)	1.506(16)
C(22A)-H(22C)	0.9900
C(22A)-H(22D)	0.9900
C(23A)-H(23C)	0.9900
C(23A)-H(23D)	0.9900
C(24A)-H(24D)	0.9800
C(24A)-H(24E)	0.9800
C(24A)-H(24F)	0.9800
C(25A)-C(26A)	1.488(16)
C(25A)-H(25C)	0.9900
C(25A)-H(25D)	0.9900
C(26A)-H(26C)	0.9900
C(26A)-H(26D)	0.9900
C(27A)-H(27D)	0.9800
C(27A)-H(27E)	0.9800
C(27A)-H(27F)	0.9800
C(28A)-C(29A)	1.508(16)
C(28A)-H(28C)	0.9900
C(28A)-H(28D)	0.9900
C(29A)-C(30A)	1.479(16)
C(29A)-H(29C)	0.9900
C(29A)-H(29D)	0.9900
C(30A)-H(30C)	0.9900
C(30A)-H(30D)	0.9900
C(31A)-H(31D)	0.9800
C(31A)-H(31E)	0.9800
C(31A)-H(31F)	0.9800
C(32A)-C(33A)	1.488(16)
C(32A)-H(32C)	0.9900
C(32A)-H(32D)	0.9900
C(33A)-H(33C)	0.9900
C(33A)-H(33D)	0.9900
C(34A)-H(34D)	0.9800
C(34A)-H(34E)	0.9800
C(34A)-H(34F)	0.9800
F(41)-C(47)	1.317(10)
F(42)-C(47)	1.302(8)
F(43)-C(47)	1.352(8)
F(41A)-C(47)	1.321(9)
F(42A)-C(47)	1.358(8)
F(43A)-C(47)	1.298(9)
F(44)-C(48)	1.335(5)
F(45)-C(48)	1.335(6)
F(46)-C(48)	1.341(5)
F(51)-C(57)	1.274(6)
F(52)-C(57)	1.358(7)
F(53)-C(57)	1.345(7)
F(51A)-C(57)	1.369(11)
F(52A)-C(57)	1.257(11)
F(53A)-C(57)	1.351(11)
F(54)-C(58)	1.335(5)
F(55)-C(58)	1.321(5)
F(56)-C(58)	1.325(5)
F(61)-C(67)	1.345(6)
F(62)-C(67)	1.310(6)
F(63)-C(67)	1.321(5)
F(64)-C(68)	1.332(6)
F(65)-C(68)	1.324(6)
F(66)-C(68)	1.341(6)
F(71)-C(77)	1.334(6)
F(72)-C(77)	1.309(7)
F(73)-C(77)	1.363(6)
F(71A)-C(77)	1.340(10)
F(72A)-C(77)	1.341(11)
F(73A)-C(77)	1.264(10)
F(74)-C(78)	1.386(7)
F(75)-C(78)	1.302(6)
F(76)-C(78)	1.284(7)
F(74A)-C(78)	1.273(8)
F(75A)-C(78)	1.412(8)
F(76A)-C(78)	1.293(9)
C(41)-C(46)	1.393(6)
C(41)-C(42)	1.403(6)
C(41)-B(41)	1.636(6)
C(42)-C(43)	1.394(6)
C(42)-H(42)	0.9500
C(43)-C(44)	1.377(6)
C(43)-C(47)	1.491(6)
C(44)-C(45)	1.383(6)
C(44)-H(44)	0.9500
C(45)-C(46)	1.387(6)
C(45)-C(48)	1.494(6)
C(46)-H(46)	0.9500
C(51)-C(56)	1.396(5)
C(51)-C(52)	1.409(5)
C(51)-B(41)	1.636(6)
C(52)-C(53)	1.387(6)
C(52)-H(52)	0.9500
C(53)-C(54)	1.386(6)
C(53)-C(57)	1.496(6)
C(54)-C(55)	1.378(6)
C(54)-H(54)	0.9500
C(55)-C(56)	1.396(5)
C(55)-C(58)	1.492(6)
C(56)-H(56)	0.9500
C(61)-C(62)	1.392(5)
C(61)-C(66)	1.409(5)
C(61)-B(41)	1.640(5)
C(62)-C(63)	1.394(5)
C(62)-H(62)	0.9500
C(63)-C(64)	1.390(6)
C(63)-C(67)	1.487(6)
C(64)-C(65)	1.386(6)
C(64)-H(64)	0.9500
C(65)-C(66)	1.385(6)
C(65)-C(68)	1.498(6)
C(66)-H(66)	0.9500
C(71)-C(76)	1.391(5)
C(71)-C(72)	1.409(5)
C(71)-B(41)	1.638(6)
C(72)-C(73)	1.387(6)
C(72)-H(72)	0.9500
C(73)-C(74)	1.388(6)
C(73)-C(77)	1.498(5)
C(74)-C(75)	1.384(6)
C(74)-H(74)	0.9500
C(75)-C(76)	1.394(5)
C(75)-C(78)	1.491(6)
C(76)-H(76)	0.9500
F(81)-C(87)	1.324(8)
F(82)-C(87)	1.386(8)
F(83)-C(87)	1.301(8)
F(81A)-C(87)	1.319(11)
F(82A)-C(87)	1.292(10)
F(83A)-C(87)	1.367(11)
F(84)-C(88)	1.316(9)
F(85)-C(88)	1.345(8)
F(86)-C(88)	1.317(9)
F(84A)-C(88)	1.335(10)
F(85A)-C(88)	1.313(11)
F(86A)-C(88)	1.335(11)
F(91)-C(97)	1.292(10)
F(92)-C(97)	1.390(9)
F(93)-C(97)	1.302(8)
F(91A)-C(97)	1.300(8)
F(92A)-C(97)	1.292(7)
F(93A)-C(97)	1.361(7)
F(94)-C(98)	1.308(7)
F(95)-C(98)	1.399(7)
F(96)-C(98)	1.281(6)
F(94A)-C(98)	1.271(11)
F(95A)-C(98)	1.292(10)
F(96A)-C(98)	1.418(10)
F(101)-C(107)	1.328(7)
F(102)-C(107)	1.324(6)
F(103)-C(107)	1.323(6)
F(104)-C(108)	1.305(9)
F(105)-C(108)	1.389(9)
F(106)-C(108)	1.319(7)
F(04A)-C(108)	1.375(10)
F(05A)-C(108)	1.311(11)
F(06A)-C(108)	1.363(9)
F(111)-C(117)	1.290(8)
F(112)-C(117)	1.308(8)
F(113)-C(117)	1.401(8)
F(11B)-C(117)	1.404(12)
F(12B)-C(117)	1.325(12)
F(13B)-C(117)	1.278(10)
F(114)-C(118)	1.398(9)
F(115)-C(118)	1.324(9)
F(116)-C(118)	1.237(9)
F(14B)-C(118)	1.260(10)
F(15B)-C(118)	1.403(10)
F(16B)-C(118)	1.334(9)
C(81)-C(86)	1.397(5)
C(81)-C(82)	1.402(5)
C(81)-B(81)	1.637(5)
C(82)-C(83)	1.389(5)
C(82)-H(82)	0.9500
C(83)-C(84)	1.382(6)
C(83)-C(87)	1.496(5)
C(84)-C(85)	1.384(5)
C(84)-H(84)	0.9500
C(85)-C(86)	1.384(5)
C(85)-C(88)	1.494(5)

C(86)-H(86)	0.9500
C(91)-C(92)	1.395(5)
C(91)-C(96)	1.399(5)
C(91)-B(81)	1.634(5)
C(92)-C(93)	1.396(5)
C(92)-H(92)	0.9500
C(93)-C(94)	1.386(6)
C(93)-C(97)	1.497(6)
C(94)-C(95)	1.387(6)
C(94)-H(94)	0.9500
C(95)-C(96)	1.388(6)
C(95)-C(98)	1.488(6)
C(96)-H(96)	0.9500
C(101)-C(102)	1.397(6)
C(101)-C(106)	1.403(6)
C(101)-B(81)	1.631(6)
C(102)-C(103)	1.393(6)
C(102)-H(102)	0.9500
C(103)-C(104)	1.384(7)
C(103)-C(107)	1.491(8)
C(104)-C(105)	1.376(8)
C(104)-H(104)	0.9500
C(105)-C(106)	1.395(7)
C(105)-C(108)	1.504(7)
C(106)-H(106)	0.9500
C(111)-C(116)	1.397(6)
C(111)-C(112)	1.401(5)
C(111)-B(81)	1.638(6)
C(112)-C(113)	1.392(6)
C(112)-H(112)	0.9500
C(113)-C(114)	1.373(8)
C(113)-C(117)	1.493(6)
C(114)-C(115)	1.386(7)
C(114)-H(114)	0.9500
C(115)-C(116)	1.383(6)
C(115)-C(118)	1.474(8)
C(116)-H(116)	0.9500
O(1S)-C(2S)	1.394(7)
O(1S)-C(3S)	1.405(6)
C(1S)-C(2S)	1.488(10)
C(1S)-H(1SA)	0.9800
C(1S)-H(1SB)	0.9800
C(1S)-H(1SC)	0.9800
C(2S)-H(2SA)	0.9900
C(2S)-H(2SB)	0.9900
C(3S)-C(4S)	1.476(8)
C(3S)-H(3SA)	0.9900
C(3S)-H(3SB)	0.9900
C(4S)-H(4SA)	0.9800
C(4S)-H(4SB)	0.9800
C(4S)-H(4SC)	0.9800
O(11S)-C(12S)	1.405(7)
O(11S)-C(13S)	1.415(7)
C(11S)-C(12S)	1.478(10)
C(11S)-H(11G)	0.9800
C(11S)-H(11H)	0.9800
C(11S)-H(11I)	0.9800
C(12S)-H(12E)	0.9900
C(12S)-H(12F)	0.9900
C(13S)-C(14S)	1.464(9)
C(13S)-H(13E)	0.9900
C(13S)-H(13F)	0.9900
C(14S)-H(14G)	0.9800
C(14S)-H(14H)	0.9800
C(14S)-H(14I)	0.9800
O(21S)-C(22S)	1.403(6)
O(21S)-C(23S)	1.414(6)
C(21S)-C(22S)	1.486(8)
C(21S)-H(21E)	0.9800
C(21S)-H(21F)	0.9800
C(21S)-H(21G)	0.9800
C(22S)-H(22E)	0.9900
C(22S)-H(22F)	0.9900
C(23S)-C(24S)	1.478(8)
C(23S)-H(23E)	0.9900
C(23S)-H(23F)	0.9900
C(24S)-H(24G)	0.9800
C(24S)-H(24H)	0.9800
C(24S)-H(24I)	0.9800
O(31S)-C(33S)	1.372(8)
O(31S)-C(32S)	1.397(7)
C(31S)-C(32S)	1.420(8)
C(31S)-H(31G)	0.9800
C(31S)-H(31H)	0.9800
C(31S)-H(31I)	0.9800
C(32S)-H(32S)	0.9900
C(32S)-H(32T)	0.9900
C(33S)-C(34S)	1.400(9)
C(33S)-H(33S)	0.9900
C(33S)-H(33T)	0.9900
C(34S)-H(34G)	0.9800
C(34S)-H(34H)	0.9800
C(34S)-H(34I)	0.9800
O(1)-Co(1)-N(2)	118.3(4)

N(4A)-Co(1)-N(3A)	96.8(5)
N(4A)-Co(1)-O(1A)	88.5(7)
N(3A)-Co(1)-O(1A)	108.5(8)
N(4A)-Co(1)-N(1A)	86.6(5)
N(3A)-Co(1)-N(1A)	153.2(7)
O(1A)-Co(1)-N(1A)	98.2(7)
O(1)-Co(1)-N(3)	99.4(5)
N(2)-Co(1)-N(3)	86.6(3)
O(1)-Co(1)-N(1)	89.0(5)
N(2)-Co(1)-N(1)	94.0(3)
N(3)-Co(1)-N(1)	170.2(4)
O(1)-Co(1)-N(4)	96.5(4)
N(2)-Co(1)-N(4)	145.0(3)
N(3)-Co(1)-N(4)	91.8(3)
N(1)-Co(1)-N(4)	82.1(3)
N(4A)-Co(1)-N(2A)	172.5(7)
N(3A)-Co(1)-N(2A)	82.3(5)
O(1A)-Co(1)-N(2A)	98.8(6)
N(1A)-Co(1)-N(2A)	90.9(4)
O(3)-Cl(1)-O(4)	109.2(9)
O(3)-Cl(1)-O(2)	112.6(9)
O(4)-Cl(1)-O(2)	109.3(10)
O(3)-Cl(1)-O(1)	109.6(9)
O(4)-Cl(1)-O(1)	107.3(9)
O(2)-Cl(1)-O(1)	108.6(9)
Cl(1)-O(1)-Co(1)	144.5(9)
C(13)-N(1)-C(1)	109.8(8)
C(13)-N(1)-C(14)	105.8(8)
C(1)-N(1)-C(14)	107.9(8)
C(13)-N(1)-Co(1)	107.1(6)
C(1)-N(1)-Co(1)	113.6(6)
C(14)-N(1)-Co(1)	112.4(8)
C(4)-N(2)-C(3)	109.8(6)
C(4)-N(2)-C(5)	107.8(7)
C(3)-N(2)-C(5)	107.7(6)
C(4)-N(2)-Co(1)	109.1(5)
C(3)-N(2)-Co(1)	116.7(5)
C(5)-N(2)-Co(1)	105.4(4)
C(6)-N(3)-C(7)	107.0(8)
C(6)-N(3)-C(8)	109.2(8)
C(7)-N(3)-C(8)	107.5(8)
C(6)-N(3)-Co(1)	103.2(6)
C(7)-N(3)-Co(1)	115.5(7)
C(8)-N(3)-Co(1)	114.0(6)
C(11)-N(4)-C(10)	108.8(9)
C(11)-N(4)-C(12)	110.2(8)
C(10)-N(4)-C(12)	106.0(7)
C(11)-N(4)-Co(1)	111.3(8)
C(10)-N(4)-Co(1)	114.8(7)
C(12)-N(4)-Co(1)	105.6(5)
N(1)-C(1)-C(2)	114.2(9)
N(1)-C(1)-H(1A)	108.7
C(2)-C(1)-H(1A)	108.7
N(1)-C(1)-H(1AB)	108.7
C(2)-C(1)-H(1AB)	108.7
H(1A)-C(1)-H(1AB)	107.6
C(1)-C(2)-C(3)	115.4(8)
C(1)-C(2)-H(2A)	108.4
C(3)-C(2)-H(2A)	108.4
C(1)-C(2)-H(2AB)	108.4
C(3)-C(2)-H(2AB)	108.4
H(2A)-C(2)-H(2AB)	107.5
N(2)-C(3)-C(2)	113.8(7)
N(2)-C(3)-H(3A)	108.8
C(2)-C(3)-H(3A)	108.8
N(2)-C(3)-H(3AB)	108.8
C(2)-C(3)-H(3AB)	108.8
H(3A)-C(3)-H(3AB)	107.7
N(2)-C(4)-H(4A)	109.5
N(2)-C(4)-H(4B)	109.5
H(4A)-C(4)-H(4B)	109.5
N(2)-C(4)-H(4C)	109.5
H(4A)-C(4)-H(4C)	109.5
H(4B)-C(4)-H(4C)	109.5
N(2)-C(5)-C(6)	109.0(7)
N(2)-C(5)-H(5A)	109.9
C(6)-C(5)-H(5A)	109.9
N(2)-C(5)-H(5AB)	109.9
C(6)-C(5)-H(5AB)	109.9
H(5A)-C(5)-H(5AB)	108.3
N(3)-C(6)-C(5)	110.3(7)
N(3)-C(6)-H(6A)	109.6
C(5)-C(6)-H(6A)	109.6
N(3)-C(6)-H(6AB)	109.6
C(5)-C(6)-H(6AB)	109.6
H(6A)-C(6)-H(6AB)	108.1
N(3)-C(7)-H(7A)	109.5
N(3)-C(7)-H(7B)	109.5
H(7A)-C(7)-H(7B)	109.5
N(3)-C(7)-H(7C)	109.5
H(7A)-C(7)-H(7C)	109.5
H(7B)-C(7)-H(7C)	109.5
N(3)-C(8)-C(9)	114.1(8)
N(3)-C(8)-H(8A)	108.7
C(9)-C(8)-H(8A)	108.7

N(3)-C(8)-H(8AB)	108.7
C(9)-C(8)-H(8AB)	108.7
H(8A)-C(8)-H(8AB)	107.6
C(10)-C(9)-C(8)	115.8(9)
C(10)-C(9)-H(9A)	108.3
C(8)-C(9)-H(9A)	108.3
C(10)-C(9)-H(9AB)	108.3
C(8)-C(9)-H(9AB)	108.3
H(9A)-C(9)-H(9AB)	107.4
N(4)-C(10)-C(9)	114.4(9)
N(4)-C(10)-H(10A)	108.7
C(9)-C(10)-H(10A)	108.7
N(4)-C(10)-H(10B)	108.7
C(9)-C(10)-H(10B)	108.7
H(10A)-C(10)-H(10B)	107.6
N(4)-C(11)-H(11A)	109.5
N(4)-C(11)-H(11B)	109.5
H(11A)-C(11)-H(11B)	109.5
N(4)-C(11)-H(11C)	109.5
H(11A)-C(11)-H(11C)	109.5
H(11B)-C(11)-H(11C)	109.5
C(13)-C(12)-N(4)	109.4(7)
C(13)-C(12)-H(12A)	109.8
N(4)-C(12)-H(12A)	109.8
C(13)-C(12)-H(12B)	109.8
N(4)-C(12)-H(12B)	109.8
H(12A)-C(12)-H(12B)	108.2
N(1)-C(13)-C(12)	110.4(7)
N(1)-C(13)-H(13A)	109.6
C(12)-C(13)-H(13A)	109.6
N(1)-C(13)-H(13B)	109.6
C(12)-C(13)-H(13B)	109.6
H(13A)-C(13)-H(13B)	108.1
N(1)-C(14)-H(14A)	109.5
N(1)-C(14)-H(14B)	109.5
H(14A)-C(14)-H(14B)	109.5
N(1)-C(14)-H(14C)	109.5
H(14A)-C(14)-H(14C)	109.5
H(14B)-C(14)-H(14C)	109.5
O(4A)-Cl(1A)-O(3A)	111.4(14)
O(4A)-Cl(1A)-O(2A)	111.6(16)
O(3A)-Cl(1A)-O(2A)	108.8(16)
O(4A)-Cl(1A)-O(1A)	109.5(14)
O(3A)-Cl(1A)-O(1A)	106.8(15)
O(2A)-Cl(1A)-O(1A)	108.6(15)
Cl(1A)-O(1A)-Co(1)	142.1(13)
C(14A)-N(1A)-C(1A)	110.3(15)
C(14A)-N(1A)-C(13A)	106.0(13)
C(1A)-N(1A)-C(13A)	107.1(12)
C(14A)-N(1A)-Co(1)	114.8(15)
C(1A)-N(1A)-Co(1)	114.8(11)
C(13A)-N(1A)-Co(1)	103.0(9)
C(3A)-N(2A)-C(4A)	107.0(12)
C(3A)-N(2A)-C(5A)	109.7(12)
C(4A)-N(2A)-C(5A)	105.5(12)
C(3A)-N(2A)-Co(1)	108.1(10)
C(4A)-N(2A)-Co(1)	121.2(9)
C(5A)-N(2A)-Co(1)	105.0(8)
C(7A)-N(3A)-C(6A)	109.6(14)
C(7A)-N(3A)-C(8A)	110.7(13)
C(6A)-N(3A)-C(8A)	104.2(12)
C(7A)-N(3A)-Co(1)	114.2(13)
C(6A)-N(3A)-Co(1)	109.3(11)
C(8A)-N(3A)-Co(1)	108.2(11)
C(10A)-N(4A)-C(11A)	107.8(15)
C(10A)-N(4A)-C(12A)	112.3(13)
C(11A)-N(4A)-C(12A)	106.5(13)
C(10A)-N(4A)-Co(1)	112.5(14)
C(11A)-N(4A)-Co(1)	113.9(16)
C(12A)-N(4A)-Co(1)	103.8(9)
C(2A)-C(1A)-N(1A)	115.2(13)
C(2A)-C(1A)-H(1AA)	108.5
N(1A)-C(1A)-H(1AA)	108.5
C(2A)-C(1A)-H(1AC)	108.5
N(1A)-C(1A)-H(1AC)	108.5
H(1AA)-C(1A)-H(1AC)	107.5
C(1A)-C(2A)-C(3A)	115.7(14)
C(1A)-C(2A)-H(2AA)	108.4
C(3A)-C(2A)-H(2AA)	108.4
C(1A)-C(2A)-H(2AC)	108.4
C(3A)-C(2A)-H(2AC)	108.4
H(2AA)-C(2A)-H(2AC)	107.4
N(2A)-C(3A)-C(2A)	112.8(12)
N(2A)-C(3A)-H(3AA)	109.0
C(2A)-C(3A)-H(3AA)	109.0
N(2A)-C(3A)-H(3AC)	109.0
C(2A)-C(3A)-H(3AC)	109.0
H(3AA)-C(3A)-H(3AC)	107.8
N(2A)-C(4A)-H(4AA)	109.5
N(2A)-C(4A)-H(4AB)	109.5
H(4AA)-C(4A)-H(4AB)	109.5
N(2A)-C(4A)-H(4AC)	109.5
H(4AA)-C(4A)-H(4AC)	109.5
H(4AB)-C(4A)-H(4AC)	109.5
C(6A)-C(5A)-N(2A)	110.7(13)
C(6A)-C(5A)-H(5AA)	109.5
N(2A)-C(5A)-H(5AA)	109.5
C(6A)-C(5A)-H(5AC)	109.5
N(2A)-C(5A)-H(5AC)	109.5
H(5AA)-C(5A)-H(5AC)	108.1
C(5A)-C(6A)-N(3A)	113.1(13)
C(5A)-C(6A)-H(6AA)	109.0
N(3A)-C(6A)-H(6AA)	109.0
C(5A)-C(6A)-H(6AC)	109.0
N(3A)-C(6A)-H(6AC)	109.0
H(6AA)-C(6A)-H(6AC)	107.8
N(3A)-C(7A)-H(7AA)	109.5
N(3A)-C(7A)-H(7AB)	109.5
H(7AA)-C(7A)-H(7AB)	109.5
N(3A)-C(7A)-H(7AC)	109.5
H(7AA)-C(7A)-H(7AC)	109.5
H(7AB)-C(7A)-H(7AC)	109.5
N(3A)-C(8A)-C(9A)	114.2(13)
N(3A)-C(8A)-H(8AA)	108.7
C(9A)-C(8A)-H(8AA)	108.7
N(3A)-C(8A)-H(8AC)	108.7
C(9A)-C(8A)-H(8AC)	108.7
H(8AA)-C(8A)-H(8AC)	107.6
C(10A)-C(9A)-C(8A)	115.7(15)
C(10A)-C(9A)-H(9AA)	108.4
C(8A)-C(9A)-H(9AA)	108.4
C(10A)-C(9A)-H(9AC)	108.4
C(8A)-C(9A)-H(9AC)	108.4
H(9AA)-C(9A)-H(9AC)	107.4
N(4A)-C(10A)-C(9A)	110.8(15)
N(4A)-C(10A)-H(10C)	109.5
C(9A)-C(10A)-H(10C)	109.5
N(4A)-C(10A)-H(10D)	109.5
C(9A)-C(10A)-H(10D)	109.5
H(10C)-C(10A)-H(10D)	108.1
N(4A)-C(11A)-H(11D)	109.5
N(4A)-C(11A)-H(11E)	109.5
H(11D)-C(11A)-H(11E)	109.5
N(4A)-C(11A)-H(11F)	109.5
H(11D)-C(11A)-H(11F)	109.5
H(11E)-C(11A)-H(11F)	109.5
C(13A)-C(12A)-N(4A)	109.5(11)
C(13A)-C(12A)-H(12C)	109.8
N(4A)-C(12A)-H(12C)	109.8
C(13A)-C(12A)-H(12D)	109.8
N(4A)-C(12A)-H(12D)	109.8
H(12C)-C(12A)-H(12D)	108.2
C(12A)-C(13A)-N(1A)	107.3(11)
C(12A)-C(13A)-H(13C)	110.3
N(1A)-C(13A)-H(13C)	110.3
C(12A)-C(13A)-H(13D)	110.3
N(1A)-C(13A)-H(13D)	110.3
H(13C)-C(13A)-H(13D)	108.5
N(1A)-C(14A)-H(14D)	109.5
N(1A)-C(14A)-H(14E)	109.5
H(14D)-C(14A)-H(14E)	109.5
N(1A)-C(14A)-H(14F)	109.5
H(14D)-C(14A)-H(14F)	109.5
H(14E)-C(14A)-H(14F)	109.5
N(23A)-Co(21)-O(21A)	114.4(6)
N(23A)-Co(21)-N(22A)	86.5(5)
O(21A)-Co(21)-N(22A)	104.8(6)
O(21)-Co(21)-N(24)	112.6(2)
O(21)-Co(21)-N(22)	101.8(2)
N(24)-Co(21)-N(22)	145.3(2)
N(23A)-Co(21)-N(21A)	149.3(5)
O(21A)-Co(21)-N(21A)	95.0(6)
N(22A)-Co(21)-N(21A)	94.7(5)
O(21)-Co(21)-N(21)	98.0(3)
N(24)-Co(21)-N(21)	85.0(2)
N(22)-Co(21)-N(21)	94.1(2)
O(21)-Co(21)-N(23)	91.4(3)
N(24)-Co(21)-N(23)	92.1(2)
N(22)-Co(21)-N(23)	83.07(19)
N(21)-Co(21)-N(23)	170.6(2)
N(23A)-Co(21)-N(24A)	92.5(5)
O(21A)-Co(21)-N(24A)	81.9(5)
N(22A)-Co(21)-N(24A)	173.0(5)
N(21A)-Co(21)-N(24A)	82.7(5)
O(24)-Cl(21)-O(23)	115.2(6)
O(24)-Cl(21)-O(21)	117.1(6)
O(23)-Cl(21)-O(21)	110.0(6)
O(24)-Cl(21)-O(22)	108.1(6)
O(23)-Cl(21)-O(22)	104.2(7)
O(21)-Cl(21)-O(22)	100.3(6)
Cl(21)-O(21)-Co(21)	151.1(6)
C(34)-N(21)-C(21)	107.6(5)
C(34)-N(21)-C(33)	106.6(5)
C(21)-N(21)-C(33)	109.0(6)
C(34)-N(21)-Co(21)	118.1(4)
C(21)-N(21)-Co(21)	111.6(4)
C(33)-N(21)-Co(21)	103.6(4)
C(24)-N(22)-C(23)	109.7(5)
C(24)-N(22)-C(25)	110.4(5)
C(23)-N(22)-C(25)	106.6(5)

C(24)-N(22)-Co(21)	108.0(4)	H(34A)-C(34)-H(34B)	109.5
C(23)-N(22)-Co(21)	113.7(4)	N(21)-C(34)-H(34C)	109.5
C(25)-N(22)-Co(21)	108.4(4)	H(34A)-C(34)-H(34C)	109.5
C(27)-N(23)-C(28)	109.1(6)	H(34B)-C(34)-H(34C)	109.5
C(27)-N(23)-C(26)	104.8(6)	O(24A)-Cl(2A)-O(22A)	126.2(16)
C(28)-N(23)-C(26)	109.0(6)	O(24A)-Cl(2A)-O(23A)	98.4(14)
C(27)-N(23)-Co(21)	119.0(4)	O(22A)-Cl(2A)-O(23A)	107.7(13)
C(28)-N(23)-Co(21)	110.7(4)	O(24A)-Cl(2A)-O(21A)	108.8(15)
C(26)-N(23)-Co(21)	103.7(4)	O(22A)-Cl(2A)-O(21A)	110.3(12)
C(31)-N(24)-C(32)	110.6(6)	O(23A)-Cl(2A)-O(21A)	102.2(13)
C(31)-N(24)-C(30)	109.0(5)	Cl(2A)-O(21A)-Co(21)	136.1(12)
C(32)-N(24)-C(30)	105.5(5)	C(33A)-N(21A)-C(34A)	108.2(13)
C(31)-N(24)-Co(21)	110.0(4)	C(33A)-N(21A)-C(21A)	111.0(12)
C(32)-N(24)-Co(21)	105.9(4)	C(34A)-N(21A)-C(21A)	108.0(13)
C(30)-N(24)-Co(21)	115.7(4)	C(33A)-N(21A)-Co(21)	107.6(9)
C(22)-C(21)-N(21)	114.8(5)	C(34A)-N(21A)-Co(21)	109.1(11)
C(22)-C(21)-H(21A)	108.6	C(21A)-N(21A)-Co(21)	113.0(10)
N(21)-C(21)-H(21A)	108.6	C(23A)-N(22A)-C(24A)	108.1(13)
C(22)-C(21)-H(21B)	108.6	C(23A)-N(22A)-C(25A)	106.3(15)
N(21)-C(21)-H(21B)	108.6	C(24A)-N(22A)-C(25A)	107.1(14)
H(21A)-C(21)-H(21B)	107.5	C(23A)-N(22A)-Co(21)	111.9(11)
C(21)-C(22)-C(23)	115.9(6)	C(24A)-N(22A)-Co(21)	118.0(11)
C(21)-C(22)-H(22A)	108.3	C(25A)-N(22A)-Co(21)	104.7(11)
C(23)-C(22)-H(22A)	108.3	C(27A)-N(23A)-C(26A)	111.7(15)
C(21)-C(22)-H(22B)	108.3	C(27A)-N(23A)-C(28A)	106.2(14)
C(23)-C(22)-H(22B)	108.3	C(26A)-N(23A)-C(28A)	103.8(12)
H(22A)-C(22)-H(22B)	107.4	C(27A)-N(23A)-Co(21)	116.9(13)
N(22)-C(23)-C(22)	116.0(6)	C(26A)-N(23A)-Co(21)	105.8(10)
N(22)-C(23)-H(23A)	108.3	C(28A)-N(23A)-Co(21)	111.7(11)
C(22)-C(23)-H(23A)	108.3	C(31A)-N(24A)-C(30A)	108.5(14)
N(22)-C(23)-H(23B)	108.3	C(31A)-N(24A)-C(32A)	109.7(15)
C(22)-C(23)-H(23B)	108.3	C(30A)-N(24A)-C(32A)	107.8(14)
H(23A)-C(23)-H(23B)	107.4	C(31A)-N(24A)-Co(21)	113.0(11)
N(22)-C(24)-H(24A)	109.5	C(30A)-N(24A)-Co(21)	114.0(12)
N(22)-C(24)-H(24B)	109.5	C(32A)-N(24A)-Co(21)	103.6(11)
H(24A)-C(24)-H(24B)	109.5	C(22A)-C(21A)-N(21A)	115.7(13)
N(22)-C(24)-H(24C)	109.5	C(22A)-C(21A)-H(21C)	108.4
H(24A)-C(24)-H(24C)	109.5	N(21A)-C(21A)-H(21C)	108.4
H(24B)-C(24)-H(24C)	109.5	C(22A)-C(21A)-H(21D)	108.4
C(26)-C(25)-N(22)	109.8(6)	N(21A)-C(21A)-H(21D)	108.4
C(26)-C(25)-H(25A)	109.7	H(21C)-C(21A)-H(21D)	107.4
N(22)-C(25)-H(25A)	109.7	C(21A)-C(22A)-C(23A)	116.4(15)
C(26)-C(25)-H(25B)	109.7	C(21A)-C(22A)-H(22C)	108.2
N(22)-C(25)-H(25B)	109.7	C(23A)-C(22A)-H(22C)	108.2
H(25A)-C(25)-H(25B)	108.2	C(21A)-C(22A)-H(22D)	108.2
C(25)-C(26)-N(23)	110.5(6)	C(23A)-C(22A)-H(22D)	108.2
C(25)-C(26)-H(26A)	109.6	H(22C)-C(22A)-H(22D)	107.3
N(23)-C(26)-H(26A)	109.6	N(22A)-C(23A)-C(22A)	112.7(13)
C(25)-C(26)-H(26B)	109.6	N(22A)-C(23A)-H(23C)	109.1
N(23)-C(26)-H(26B)	109.6	C(22A)-C(23A)-H(23C)	109.1
H(26A)-C(26)-H(26B)	108.1	N(22A)-C(23A)-H(23D)	109.1
N(23)-C(27)-H(27A)	109.5	C(22A)-C(23A)-H(23D)	109.1
N(23)-C(27)-H(27B)	109.5	H(23C)-C(23A)-H(23D)	107.8
H(27A)-C(27)-H(27B)	109.5	N(22A)-C(24A)-H(24D)	109.5
N(23)-C(27)-H(27C)	109.5	N(22A)-C(24A)-H(24E)	109.5
H(27A)-C(27)-H(27C)	109.5	H(24D)-C(24A)-H(24E)	109.5
H(27B)-C(27)-H(27C)	109.5	N(22A)-C(24A)-H(24F)	109.5
N(23)-C(28)-C(29)	113.3(6)	H(24D)-C(24A)-H(24F)	109.5
N(23)-C(28)-H(28A)	108.9	H(24E)-C(24A)-H(24F)	109.5
C(29)-C(28)-H(28A)	108.9	C(26A)-C(25A)-N(22A)	110.8(16)
N(23)-C(28)-H(28B)	108.9	C(26A)-C(25A)-H(25C)	109.5
C(29)-C(28)-H(28B)	108.9	N(22A)-C(25A)-H(25C)	109.5
H(28A)-C(28)-H(28B)	107.7	C(26A)-C(25A)-H(25D)	109.5
C(30)-C(29)-C(28)	115.6(6)	N(22A)-C(25A)-H(25D)	109.5
C(30)-C(29)-H(29A)	108.4	H(25C)-C(25A)-H(25D)	108.1
C(28)-C(29)-H(29A)	108.4	N(23A)-C(26A)-C(25A)	110.8(17)
C(30)-C(29)-H(29B)	108.4	N(23A)-C(26A)-H(26C)	109.5
C(28)-C(29)-H(29B)	108.4	C(25A)-C(26A)-H(26C)	109.5
H(29A)-C(29)-H(29B)	107.5	N(23A)-C(26A)-H(26D)	109.5
C(29)-C(30)-N(24)	114.5(5)	C(25A)-C(26A)-H(26D)	109.5
C(29)-C(30)-H(30A)	108.6	H(26C)-C(26A)-H(26D)	108.1
N(24)-C(30)-H(30A)	108.6	N(23A)-C(27A)-H(27D)	109.5
C(29)-C(30)-H(30B)	108.6	N(23A)-C(27A)-H(27E)	109.5
N(24)-C(30)-H(30B)	108.6	H(27D)-C(27A)-H(27E)	109.5
H(30A)-C(30)-H(30B)	107.6	N(23A)-C(27A)-H(27F)	109.5
N(24)-C(31)-H(31A)	109.5	H(27D)-C(27A)-H(27F)	109.5
N(24)-C(31)-H(31B)	109.5	H(27E)-C(27A)-H(27F)	109.5
H(31A)-C(31)-H(31B)	109.5	C(29A)-C(28A)-N(23A)	115.5(14)
N(24)-C(31)-H(31C)	109.5	C(29A)-C(28A)-H(28C)	108.4
H(31A)-C(31)-H(31C)	109.5	N(23A)-C(28A)-H(28C)	108.4
H(31B)-C(31)-H(31C)	109.5	C(29A)-C(28A)-H(28D)	108.4
C(33)-C(32)-N(24)	110.5(6)	N(23A)-C(28A)-H(28D)	108.4
C(33)-C(32)-H(32A)	109.6	H(28C)-C(28A)-H(28D)	107.5
N(24)-C(32)-H(32A)	109.6	C(30A)-C(29A)-C(28A)	120.9(17)
C(33)-C(32)-H(32B)	109.6	C(30A)-C(29A)-H(29C)	107.1
N(24)-C(32)-H(32B)	109.6	C(28A)-C(29A)-H(29C)	107.1
H(32A)-C(32)-H(32B)	108.1	C(30A)-C(29A)-H(29D)	107.1
C(32)-C(33)-N(21)	110.4(6)	C(28A)-C(29A)-H(29D)	107.1
C(32)-C(33)-H(33A)	109.6	H(29C)-C(29A)-H(29D)	106.8
N(21)-C(33)-H(33A)	109.6	C(29A)-C(30A)-N(24A)	109.1(14)
C(32)-C(33)-H(33B)	109.6	C(29A)-C(30A)-H(30C)	109.9
N(21)-C(33)-H(33B)	109.6	N(24A)-C(30A)-H(30C)	109.9
H(33A)-C(33)-H(33B)	108.1	C(29A)-C(30A)-H(30D)	109.9
N(21)-C(34)-H(34A)	109.5	N(24A)-C(30A)-H(30D)	109.9
N(21)-C(34)-H(34B)	109.5	H(30C)-C(30A)-H(30D)	108.3

N(24A)-C(31A)-H(31D)	109.5
N(24A)-C(31A)-H(31E)	109.5
H(31D)-C(31A)-H(31E)	109.5
N(24A)-C(31A)-H(31F)	109.5
H(31D)-C(31A)-H(31F)	109.5
H(31E)-C(31A)-H(31F)	109.5
C(33A)-C(32A)-N(24A)	111.4(15)
C(33A)-C(32A)-H(32C)	109.4
N(24A)-C(32A)-H(32C)	109.4
C(33A)-C(32A)-H(32D)	109.4
N(24A)-C(32A)-H(32D)	109.4
H(32C)-C(32A)-H(32D)	108.0
N(21A)-C(33A)-C(32A)	111.0(15)
N(21A)-C(33A)-H(33C)	109.4
C(32A)-C(33A)-H(33C)	109.4
N(21A)-C(33A)-H(33D)	109.4
C(32A)-C(33A)-H(33D)	109.4
H(33C)-C(33A)-H(33D)	108.0
N(21A)-C(34A)-H(34D)	109.5
N(21A)-C(34A)-H(34E)	109.5
H(34D)-C(34A)-H(34E)	109.5
N(21A)-C(34A)-H(34F)	109.5
H(34D)-C(34A)-H(34F)	109.5
H(34E)-C(34A)-H(34F)	109.5
C(46)-C(41)-C(42)	115.7(4)
C(46)-C(41)-B(41)	123.0(3)
C(42)-C(41)-B(41)	120.9(4)
C(43)-C(42)-C(41)	121.7(4)
C(43)-C(42)-H(42)	119.2
C(41)-C(42)-H(42)	119.2
C(44)-C(43)-C(42)	121.2(4)
C(44)-C(43)-C(47)	120.4(4)
C(42)-C(43)-C(47)	118.4(4)
C(43)-C(44)-C(45)	118.2(4)
C(43)-C(44)-H(44)	120.9
C(45)-C(44)-H(44)	120.9
C(44)-C(45)-C(46)	120.6(4)
C(44)-C(45)-C(48)	120.8(4)
C(46)-C(45)-C(48)	118.6(4)
C(45)-C(46)-C(41)	122.7(4)
C(45)-C(46)-H(46)	118.7
C(41)-C(46)-H(46)	118.7
F(42)-C(47)-F(41)	107.7(9)
F(43A)-C(47)-F(41A)	108.6(9)
F(42)-C(47)-F(43)	104.2(8)
F(41)-C(47)-F(43)	104.0(9)
F(43A)-C(47)-F(42A)	106.9(8)
F(41A)-C(47)-F(42A)	102.8(8)
F(43A)-C(47)-C(43)	116.3(7)
F(42)-C(47)-C(43)	113.4(6)
F(41)-C(47)-C(43)	115.3(8)
F(41A)-C(47)-C(43)	112.1(8)
F(43)-C(47)-C(43)	111.3(6)
F(42A)-C(47)-C(43)	109.3(5)
F(45)-C(48)-F(44)	106.8(4)
F(45)-C(48)-F(46)	105.1(4)
F(44)-C(48)-F(46)	105.9(4)
F(45)-C(48)-C(45)	112.6(4)
F(44)-C(48)-C(45)	113.5(4)
F(46)-C(48)-C(45)	112.2(4)
C(56)-C(51)-C(52)	116.1(3)
C(56)-C(51)-B(41)	123.1(3)
C(52)-C(51)-B(41)	120.6(3)
C(53)-C(52)-C(51)	121.7(4)
C(53)-C(52)-H(52)	119.2
C(51)-C(52)-H(52)	119.2
C(54)-C(53)-C(52)	121.0(4)
C(54)-C(53)-C(57)	118.7(4)
C(52)-C(53)-C(57)	120.3(4)
C(55)-C(54)-C(53)	118.5(4)
C(55)-C(54)-H(54)	120.8
C(53)-C(54)-H(54)	120.8
C(54)-C(55)-C(56)	120.7(4)
C(54)-C(55)-C(58)	120.3(4)
C(56)-C(55)-C(58)	118.9(4)
C(51)-C(56)-C(55)	122.0(3)
C(51)-C(56)-H(56)	119.0
C(55)-C(56)-H(56)	119.0
F(51)-C(57)-F(53)	109.8(5)
F(52A)-C(57)-F(53A)	110.8(11)
F(51)-C(57)-F(52)	108.3(5)
F(53)-C(57)-F(52)	99.3(5)
F(52A)-C(57)-F(51A)	105.5(11)
F(53A)-C(57)-F(51A)	96.4(11)
F(52A)-C(57)-C(53)	121.7(8)
F(51)-C(57)-C(53)	116.0(4)
F(53)-C(57)-C(53)	111.2(4)
F(53A)-C(57)-C(53)	109.1(7)
F(52)-C(57)-C(53)	110.9(4)
F(51A)-C(57)-C(53)	110.4(8)
F(55)-C(58)-F(56)	107.9(4)
F(55)-C(58)-F(54)	105.5(4)
F(56)-C(58)-F(54)	104.5(4)
F(55)-C(58)-C(55)	113.4(4)
F(56)-C(58)-C(55)	112.5(4)
F(54)-C(58)-C(55)	112.5(3)
C(62)-C(61)-C(66)	115.8(3)
C(62)-C(61)-B(41)	122.5(3)
C(66)-C(61)-B(41)	120.9(3)
C(61)-C(62)-C(63)	122.2(4)
C(61)-C(62)-H(62)	118.9
C(63)-C(62)-H(62)	118.9
C(64)-C(63)-C(62)	121.0(4)
C(64)-C(63)-C(67)	119.3(4)
C(62)-C(63)-C(67)	119.6(4)
C(65)-C(64)-C(63)	117.6(4)
C(65)-C(64)-H(64)	121.2
C(63)-C(64)-H(64)	121.2
C(66)-C(65)-C(64)	121.3(4)
C(66)-C(65)-C(68)	119.0(4)
C(64)-C(65)-C(68)	119.7(4)
C(65)-C(66)-C(61)	122.0(4)
C(65)-C(66)-H(66)	119.0
C(61)-C(66)-H(66)	119.0
F(62)-C(67)-F(63)	107.0(4)
F(62)-C(67)-F(61)	107.7(4)
F(63)-C(67)-F(61)	103.7(4)
F(62)-C(67)-C(63)	112.7(4)
F(63)-C(67)-C(63)	113.3(4)
F(61)-C(67)-C(63)	111.8(4)
F(65)-C(68)-F(64)	106.0(4)
F(65)-C(68)-F(66)	107.3(4)
F(64)-C(68)-F(66)	105.2(4)
F(65)-C(68)-C(65)	112.3(4)
F(64)-C(68)-C(65)	113.1(4)
F(66)-C(68)-C(65)	112.5(4)
C(76)-C(71)-C(72)	116.0(4)
C(76)-C(71)-B(41)	124.1(3)
C(72)-C(71)-B(41)	119.8(3)
C(73)-C(72)-C(71)	121.8(4)
C(73)-C(72)-H(72)	119.1
C(71)-C(72)-H(72)	119.1
C(72)-C(73)-C(74)	121.0(4)
C(72)-C(73)-C(77)	120.1(4)
C(74)-C(73)-C(77)	118.9(4)
C(75)-C(74)-C(73)	118.0(4)
C(75)-C(74)-H(74)	121.0
C(73)-C(74)-H(74)	121.0
C(74)-C(75)-C(76)	120.9(4)
C(74)-C(75)-C(78)	119.6(4)
C(76)-C(75)-C(78)	119.5(4)
C(71)-C(76)-C(75)	122.2(4)
C(71)-C(76)-H(76)	118.9
C(75)-C(76)-H(76)	118.9
F(72)-C(77)-F(71)	108.9(6)
F(73A)-C(77)-F(71A)	112.3(11)
F(73A)-C(77)-F(72A)	109.1(13)
F(71A)-C(77)-F(72A)	100.0(12)
F(72)-C(77)-F(73)	104.9(5)
F(71)-C(77)-F(73)	103.0(5)
F(73A)-C(77)-C(73)	115.1(8)
F(72)-C(77)-C(73)	113.8(6)
F(71)-C(77)-C(73)	114.0(4)
F(71A)-C(77)-C(73)	108.6(7)
F(72A)-C(77)-C(73)	110.7(12)
F(73)-C(77)-C(73)	111.3(4)
F(74A)-C(78)-F(76A)	111.7(9)
F(76)-C(78)-F(75)	110.6(6)
F(76)-C(78)-F(74)	104.5(6)
F(75)-C(78)-F(74)	102.3(6)
F(74A)-C(78)-F(75A)	100.6(8)
F(76A)-C(78)-F(75A)	100.4(8)
F(74A)-C(78)-C(75)	117.6(5)
F(76)-C(78)-C(75)	113.6(5)
F(76A)-C(78)-C(75)	116.4(7)
F(75)-C(78)-C(75)	115.1(4)
F(74)-C(78)-C(75)	109.6(4)
F(75A)-C(78)-C(75)	107.0(5)
C(41)-B(41)-C(51)	111.7(3)
C(41)-B(41)-C(71)	111.6(3)
C(51)-B(41)-C(71)	105.3(3)
C(41)-B(41)-C(61)	102.2(3)
C(51)-B(41)-C(61)	113.4(3)
C(71)-B(41)-C(61)	113.0(3)
C(86)-C(81)-C(82)	115.5(3)
C(86)-C(81)-B(81)	122.8(3)
C(82)-C(81)-B(81)	121.2(3)
C(83)-C(82)-C(81)	122.2(3)
C(83)-C(82)-H(82)	118.9
C(81)-C(82)-H(82)	118.9
C(84)-C(83)-C(82)	121.0(4)
C(84)-C(83)-C(87)	120.5(4)
C(82)-C(83)-C(87)	118.5(4)
C(83)-C(84)-C(85)	117.8(4)
C(83)-C(84)-H(84)	121.1
C(85)-C(84)-H(84)	121.1
C(84)-C(85)-C(86)	121.1(3)
C(84)-C(85)-C(88)	118.8(3)
C(86)-C(85)-C(88)	120.0(3)
C(85)-C(86)-C(81)	122.4(3)

C(85)-C(86)-H(86)	118.8
C(81)-C(86)-H(86)	118.8
F(82A)-C(87)-F(81A)	109.7(13)
F(83)-C(87)-F(81)	109.4(9)
F(82A)-C(87)-F(83A)	104.2(10)
F(81A)-C(87)-F(83A)	104.0(13)
F(83)-C(87)-F(82)	105.2(6)
F(81)-C(87)-F(82)	103.7(7)
F(82A)-C(87)-C(83)	115.4(9)
F(83)-C(87)-C(83)	115.7(6)
F(81A)-C(87)-C(83)	114.1(12)
F(81)-C(87)-C(83)	112.4(8)
F(83A)-C(87)-C(83)	108.3(10)
F(82)-C(87)-C(83)	109.5(5)
F(84)-C(88)-F(86)	108.0(10)
F(85A)-C(88)-F(86A)	108.1(12)
F(85A)-C(88)-F(84A)	106.7(12)
F(86A)-C(88)-F(84A)	103.4(11)
F(84)-C(88)-F(85)	105.3(9)
F(86)-C(88)-F(85)	105.6(9)
F(85A)-C(88)-C(85)	115.0(10)
F(84)-C(88)-C(85)	113.7(8)
F(86)-C(88)-C(85)	112.1(9)
F(86A)-C(88)-C(85)	110.6(12)
F(84A)-C(88)-C(85)	112.2(9)
F(85)-C(88)-C(85)	111.6(7)
C(92)-C(91)-C(96)	116.2(4)
C(92)-C(91)-B(81)	123.0(3)
C(96)-C(91)-B(81)	120.4(3)
C(91)-C(92)-C(93)	121.9(4)
C(91)-C(92)-H(92)	119.1
C(93)-C(92)-H(92)	119.1
C(94)-C(93)-C(92)	120.8(4)
C(94)-C(93)-C(97)	119.5(4)
C(92)-C(93)-C(97)	119.7(4)
C(93)-C(94)-C(95)	118.2(4)
C(93)-C(94)-H(94)	120.9
C(95)-C(94)-H(94)	120.9
C(94)-C(95)-C(96)	120.7(4)
C(94)-C(95)-C(98)	120.2(4)
C(96)-C(95)-C(98)	119.1(4)
C(95)-C(96)-C(91)	122.2(4)
C(95)-C(96)-H(96)	118.9
C(91)-C(96)-H(96)	118.9
F(92A)-C(97)-F(91A)	109.2(7)
F(91)-C(97)-F(93)	109.6(9)
F(92A)-C(97)-F(93A)	106.0(5)
F(91A)-C(97)-F(93A)	105.2(6)
F(91)-C(97)-F(92)	102.4(9)
F(93)-C(97)-F(92)	101.8(6)
F(92A)-C(97)-C(93)	112.6(4)
F(91)-C(97)-C(93)	114.8(8)
F(91A)-C(97)-C(93)	113.1(6)
F(93)-C(97)-C(93)	117.2(5)
F(93A)-C(97)-C(93)	110.3(4)
F(92)-C(97)-C(93)	109.3(5)
F(94A)-C(98)-F(95A)	114.1(12)
F(96)-C(98)-F(94)	112.7(7)
F(96)-C(98)-F(95)	102.3(6)
F(94)-C(98)-F(95)	99.9(6)
F(94A)-C(98)-F(96A)	102.0(11)
F(95A)-C(98)-F(96A)	98.1(9)
F(94A)-C(98)-C(95)	119.2(11)
F(96)-C(98)-C(95)	116.9(4)
F(95A)-C(98)-C(95)	115.5(6)
F(94)-C(98)-C(95)	113.5(6)
F(95)-C(98)-C(95)	109.3(4)
F(96A)-C(98)-C(95)	103.6(6)
C(102)-C(101)-C(106)	115.5(4)
C(102)-C(101)-B(81)	124.0(3)
C(106)-C(101)-B(81)	120.0(4)
C(103)-C(102)-C(101)	122.7(4)
C(103)-C(102)-H(102)	118.7
C(101)-C(102)-H(102)	118.7
C(104)-C(103)-C(102)	120.5(5)
C(104)-C(103)-C(107)	119.5(4)
C(102)-C(103)-C(107)	120.0(4)
C(105)-C(104)-C(103)	118.1(4)
C(105)-C(104)-H(104)	120.9
C(103)-C(104)-H(104)	120.9
C(104)-C(105)-C(106)	121.4(4)
C(104)-C(105)-C(108)	120.0(5)
C(106)-C(105)-C(108)	118.6(5)
C(105)-C(106)-C(101)	121.7(5)
C(105)-C(106)-H(106)	119.1
C(101)-C(106)-H(106)	119.1
F(103)-C(107)-F(102)	106.5(4)
F(103)-C(107)-F(101)	105.4(5)
F(102)-C(107)-F(101)	106.4(5)
F(103)-C(107)-C(103)	112.2(4)
F(102)-C(107)-C(103)	113.5(5)
F(101)-C(107)-C(103)	112.3(4)
F(104)-C(108)-F(106)	112.8(6)
F(05A)-C(108)-F(06A)	112.9(10)
F(05A)-C(108)-F(04A)	105.0(12)
F(06A)-C(108)-F(04A)	101.1(8)
F(104)-C(108)-F(105)	102.7(9)
F(106)-C(108)-F(105)	102.2(7)
F(104)-C(108)-C(105)	116.9(5)
F(05A)-C(108)-C(105)	117.0(13)
F(106)-C(108)-C(105)	111.7(5)
F(06A)-C(108)-C(105)	111.8(5)
F(04A)-C(108)-C(105)	107.3(6)
F(105)-C(108)-C(105)	109.1(9)
C(116)-C(111)-C(112)	115.4(4)
C(116)-C(111)-B(81)	122.4(3)
C(112)-C(111)-B(81)	121.9(4)
C(113)-C(112)-C(111)	121.9(4)
C(113)-C(112)-H(112)	119.1
C(111)-C(112)-H(112)	119.1
C(114)-C(113)-C(112)	121.4(4)
C(114)-C(113)-C(117)	120.0(4)
C(112)-C(113)-C(117)	118.5(5)
C(113)-C(114)-C(115)	117.7(4)
C(113)-C(114)-H(114)	121.1
C(115)-C(114)-H(114)	121.1
C(116)-C(115)-C(114)	121.0(5)
C(116)-C(115)-C(118)	120.0(4)
C(114)-C(115)-C(118)	119.1(4)
C(115)-C(116)-C(111)	122.5(4)
C(115)-C(116)-H(116)	118.8
C(111)-C(116)-H(116)	118.8
F(111)-C(117)-F(112)	110.0(8)
F(13B)-C(117)-F(12B)	111.5(15)
F(111)-C(117)-F(113)	103.1(5)
F(112)-C(117)-F(113)	105.4(7)
F(13B)-C(117)-F(11B)	100.0(11)
F(12B)-C(117)-F(11B)	100.2(15)
F(13B)-C(117)-C(113)	120.6(9)
F(111)-C(117)-C(113)	112.8(6)
F(112)-C(117)-C(113)	114.6(7)
F(12B)-C(117)-C(113)	113.5(14)
F(113)-C(117)-C(113)	110.0(5)
F(11B)-C(117)-C(113)	107.9(10)
F(116)-C(118)-F(115)	112.4(10)
F(14B)-C(118)-F(16B)	109.7(10)
F(116)-C(118)-F(114)	104.5(9)
F(115)-C(118)-F(114)	98.6(8)
F(14B)-C(118)-F(15B)	104.2(10)
F(16B)-C(118)-F(15B)	95.9(9)
F(116)-C(118)-C(115)	118.7(7)
F(14B)-C(118)-C(115)	120.2(8)
F(115)-C(118)-C(115)	112.3(7)
F(16B)-C(118)-C(115)	115.0(6)
F(114)-C(118)-C(115)	107.8(6)
F(15B)-C(118)-C(115)	108.4(6)
C(101)-B(81)-C(91)	113.3(3)
C(101)-B(81)-C(111)	112.3(3)
C(91)-B(81)-C(111)	103.2(3)
C(101)-B(81)-C(81)	102.8(3)
C(91)-B(81)-C(81)	113.3(3)
C(111)-B(81)-C(81)	112.3(3)
C(2S)-O(1S)-C(3S)	113.2(4)
C(2S)-C(1S)-H(1SA)	109.5
C(2S)-C(1S)-H(1SB)	109.5
H(1SA)-C(1S)-H(1SB)	109.5
C(2S)-C(1S)-H(1SC)	109.5
H(1SA)-C(1S)-H(1SC)	109.5
H(1SB)-C(1S)-H(1SC)	109.5
O(1S)-C(2S)-C(1S)	109.9(6)
O(1S)-C(2S)-H(2SA)	109.7
C(1S)-C(2S)-H(2SA)	109.7
O(1S)-C(2S)-H(2SB)	109.7
C(1S)-C(2S)-H(2SB)	109.7
H(2SA)-C(2S)-H(2SB)	108.2
O(1S)-C(3S)-C(4S)	109.3(4)
O(1S)-C(3S)-H(3SA)	109.8
C(4S)-C(3S)-H(3SA)	109.8
O(1S)-C(3S)-H(3SB)	109.8
C(4S)-C(3S)-H(3SB)	109.8
H(3SA)-C(3S)-H(3SB)	108.3
C(3S)-C(4S)-H(4SA)	109.5
C(3S)-C(4S)-H(4SB)	109.5
H(4SA)-C(4S)-H(4SB)	109.5
C(3S)-C(4S)-H(4SC)	109.5
H(4SA)-C(4S)-H(4SC)	109.5
H(4SB)-C(4S)-H(4SC)	109.5
C(12S)-O(11S)-C(13S)	111.8(5)
C(12S)-C(11S)-H(11G)	109.5
C(12S)-C(11S)-H(11H)	109.5
H(11G)-C(11S)-H(11H)	109.5
C(12S)-C(11S)-H(11I)	109.5
H(11G)-C(11S)-H(11I)	109.5
H(11H)-C(11S)-H(11I)	109.5
O(11S)-C(12S)-C(11S)	108.4(6)
O(11S)-C(12S)-H(12E)	110.0
C(11S)-C(12S)-H(12E)	110.0
O(11S)-C(12S)-H(12F)	110.0
C(11S)-C(12S)-H(12F)	110.0
H(12E)-C(12S)-H(12F)	108.4

O(11S)-C(13S)-C(14S)	109.9(5)	C(23S)-C(24S)-H(24G)	109.5
O(11S)-C(13S)-H(13E)	109.7	C(23S)-C(24S)-H(24H)	109.5
C(14S)-C(13S)-H(13E)	109.7	H(24G)-C(24S)-H(24H)	109.5
O(11S)-C(13S)-H(13F)	109.7	C(23S)-C(24S)-H(24I)	109.5
C(14S)-C(13S)-H(13F)	109.7	H(24G)-C(24S)-H(24I)	109.5
H(13E)-C(13S)-H(13F)	108.2	H(24H)-C(24S)-H(24I)	109.5
C(13S)-C(14S)-H(14G)	109.5	C(33S)-O(31S)-C(32S)	110.2(6)
C(13S)-C(14S)-H(14H)	109.5	C(32S)-C(31S)-H(31G)	109.5
H(14G)-C(14S)-H(14H)	109.5	C(32S)-C(31S)-H(31H)	109.5
C(13S)-C(14S)-H(14I)	109.5	H(31G)-C(31S)-H(31H)	109.5
H(14G)-C(14S)-H(14I)	109.5	C(32S)-C(31S)-H(31I)	109.5
H(14H)-C(14S)-H(14I)	109.5	H(31G)-C(31S)-H(31I)	109.5
C(22S)-O(21S)-C(23S)	112.7(4)	H(31H)-C(31S)-H(31I)	109.5
C(22S)-C(21S)-H(21E)	109.5	O(31S)-C(32S)-C(31S)	109.2(6)
C(22S)-C(21S)-H(21F)	109.5	O(31S)-C(32S)-H(32S)	109.8
H(21E)-C(21S)-H(21F)	109.5	C(31S)-C(32S)-H(32S)	109.8
C(22S)-C(21S)-H(21G)	109.5	O(31S)-C(32S)-H(32T)	109.8
H(21E)-C(21S)-H(21G)	109.5	C(31S)-C(32S)-H(32T)	109.8
H(21F)-C(21S)-H(21G)	109.5	H(32S)-C(32S)-H(32T)	108.3
O(21S)-C(22S)-C(21S)	109.0(4)	O(31S)-C(33S)-C(34S)	115.0(7)
O(21S)-C(22S)-H(22E)	109.9	O(31S)-C(33S)-H(33S)	108.5
C(21S)-C(22S)-H(22E)	109.9	C(34S)-C(33S)-H(33S)	108.5
O(21S)-C(22S)-H(22F)	109.9	O(31S)-C(33S)-H(33T)	108.5
C(21S)-C(22S)-H(22F)	109.9	C(34S)-C(33S)-H(33T)	108.5
H(22E)-C(22S)-H(22F)	108.3	H(33S)-C(33S)-H(33T)	107.5
O(21S)-C(23S)-C(24S)	109.8(5)	C(33S)-C(34S)-H(34G)	109.5
O(21S)-C(23S)-H(23E)	109.7	C(33S)-C(34S)-H(34H)	109.5
C(24S)-C(23S)-H(23E)	109.7	H(34G)-C(34S)-H(34H)	109.5
O(21S)-C(23S)-H(23F)	109.7	C(33S)-C(34S)-H(34I)	109.5
C(24S)-C(23S)-H(23F)	109.7	H(34G)-C(34S)-H(34I)	109.5
H(23E)-C(23S)-H(23F)	108.2	H(34H)-C(34S)-H(34I)	109.5

Table S60. Anisotropic displacement parameters ($Å^2$x 10^3) for **6a-B$_{ArF}$**.
The anisotropic displacement factor exponent takes the form: $-2p^2[h^2 a^{*2}U^{11} + ... + 2 h k a^* b^* U^{12}]$

	U^{11}	U^{22}	U^{33}	U^{23}	U^{13}	U^{12}
Co(1)	30(1)	25(1)	18(1)	3(1)	2(1)	1(1)
Cl(1)	13(1)	33(2)	65(3)	4(2)	-4(1)	0(1)
O(1)	10(3)	67(6)	63(6)	16(5)	-6(3)	-1(3)
O(2)	36(6)	40(4)	90(9)	18(4)	-7(5)	-9(4)
O(3)	38(6)	75(7)	104(8)	24(6)	-46(6)	-16(5)
O(4)	56(9)	50(5)	115(10)	-8(7)	30(7)	6(6)
N(1)	41(4)	57(4)	20(3)	1(3)	3(3)	2(3)
N(2)	18(3)	25(3)	48(3)	-13(2)	-3(2)	7(2)
N(3)	35(4)	38(4)	29(3)	7(3)	2(3)	-6(3)
N(4)	34(4)	15(3)	42(3)	-2(3)	-4(3)	1(3)
C(1)	44(4)	81(6)	21(4)	5(5)	6(3)	15(5)
C(2)	45(5)	77(6)	41(4)	-18(5)	9(4)	20(5)
C(3)	36(4)	46(4)	49(4)	-20(3)	2(3)	16(4)
C(4)	29(4)	34(5)	87(7)	-27(5)	-8(4)	1(4)
C(5)	44(4)	27(3)	53(4)	13(3)	-1(3)	5(3)
C(6)	56(6)	35(4)	51(4)	20(3)	12(4)	-1(4)
C(7)	46(6)	63(7)	37(5)	1(5)	7(5)	6(5)
C(8)	38(5)	60(4)	19(4)	1(3)	0(3)	4(4)
C(9)	35(5)	59(5)	37(5)	-15(4)	-5(4)	-4(4)
C(10)	27(4)	30(4)	50(5)	-12(4)	-2(4)	-1(3)
C(11)	34(5)	20(4)	73(9)	-5(5)	-11(5)	3(4)
C(12)	34(4)	29(4)	60(4)	20(3)	3(3)	-6(3)
C(13)	45(4)	55(4)	42(4)	24(3)	1(3)	5(4)
C(14)	49(7)	78(8)	21(4)	0(5)	-5(5)	14(6)
Cl(1A)	13(1)	33(2)	65(3)	4(2)	-4(1)	0(1)
O(1A)	17(4)	16(5)	46(7)	-11(5)	3(5)	5(4)
O(2A)	27(9)	40(6)	92(15)	12(8)	13(10)	9(6)
O(3A)	20(6)	57(10)	45(5)	-18(6)	8(4)	-9(6)
O(4A)	12(5)	46(8)	55(7)	13(6)	9(5)	11(5)
N(1A)	39(6)	41(5)	19(5)	-4(4)	4(4)	-8(5)
N(2A)	53(7)	33(5)	48(5)	2(5)	2(5)	5(5)
N(3A)	38(7)	42(6)	30(5)	16(5)	-7(5)	-7(5)
N(4A)	37(6)	18(5)	35(5)	-1(4)	-6(4)	1(5)
C(1A)	43(6)	43(6)	24(6)	-4(6)	10(5)	-4(6)
C(2A)	48(8)	39(7)	46(6)	-13(6)	-3(6)	1(6)
C(3A)	48(6)	41(6)	53(6)	-6(5)	-2(5)	6(6)
C(4A)	57(8)	29(7)	63(9)	-4(7)	-4(7)	4(7)
C(5A)	53(7)	33(5)	60(6)	8(5)	2(5)	-3(5)
C(6A)	46(8)	40(6)	59(7)	21(6)	-4(6)	-2(6)
C(7A)	41(9)	56(11)	30(7)	18(8)	-7(7)	-4(7)
C(8A)	34(7)	58(6)	26(7)	5(6)	-8(5)	3(6)
C(9A)	27(7)	57(6)	36(7)	-12(6)	-8(6)	0(6)
C(10A)	30(6)	32(6)	38(7)	-13(6)	0(5)	-2(5)
C(11A)	37(9)	46(13)	49(10)	-17(9)	-9(7)	6(9)
C(12A)	32(6)	24(5)	44(5)	14(4)	-6(5)	-2(5)
C(13A)	34(6)	37(5)	29(5)	15(4)	-5(4)	-5(4)
C(14A)	27(8)	64(11)	33(8)	8(8)	2(7)	-12(7)
Co(21)	20(1)	20(1)	15(1)	1(1)	0(1)	-3(1)
Cl(21)	14(1)	61(2)	58(2)	-10(2)	-2(1)	-1(1)
O(21)	14(2)	75(4)	87(5)	-22(3)	-5(2)	3(2)
O(22)	77(5)	121(6)	76(5)	37(5)	1(4)	11(5)
O(23)	27(3)	67(4)	74(5)	-11(4)	-1(4)	-4(3)
O(24)	16(2)	66(4)	103(6)	4(3)	18(3)	4(2)
N(21)	20(2)	26(2)	35(3)	10(2)	10(2)	2(2)
N(22)	39(3)	20(2)	16(2)	-4(2)	0(2)	2(2)
N(23)	39(3)	21(2)	33(3)	0(2)	-4(2)	4(2)
N(24)	27(2)	31(2)	21(2)	-6(2)	5(2)	-2(2)
C(21)	25(3)	18(3)	53(4)	6(3)	10(3)	-6(2)
C(22)	27(3)	21(3)	58(4)	-5(3)	5(3)	-5(3)
C(23)	35(3)	30(3)	33(3)	-12(3)	-4(3)	-3(3)
C(24)	47(4)	41(4)	25(3)	-7(3)	14(3)	3(3)
C(25)	60(4)	30(3)	17(3)	3(3)	-7(3)	2(3)
C(26)	55(4)	37(4)	28(4)	12(3)	8(3)	-1(3)
C(27)	48(4)	32(4)	58(5)	7(3)	-2(4)	-9(3)
C(28)	42(4)	22(3)	44(4)	1(3)	1(3)	4(3)
C(29)	33(3)	21(3)	52(4)	-7(3)	-1(3)	0(3)
C(30)	28(3)	38(4)	27(4)	-6(3)	5(3)	3(3)
C(31)	29(3)	61(5)	24(3)	-13(3)	0(3)	4(3)
C(32)	29(3)	49(4)	20(3)	5(3)	8(3)	3(3)
C(33)	38(4)	40(4)	29(3)	16(3)	1(3)	5(3)
C(34)	39(4)	33(4)	52(4)	12(3)	10(3)	18(3)
Cl(2A)	10(3)	80(6)	53(4)	-17(4)	0(3)	8(3)
O(21A)	14(2)	75(4)	87(5)	-22(3)	-5(2)	3(2)
O(22A)	19(6)	72(8)	46(5)	-2(6)	9(5)	-6(5)
O(23A)	16(2)	66(4)	103(6)	4(3)	18(3)	4(2)
O(24A)	43(11)	115(12)	66(10)	-45(10)	6(8)	-33(10)
N(21A)	34(5)	22(5)	55(5)	-6(5)	1(5)	2(4)
N(22A)	49(6)	44(5)	38(5)	0(5)	-2(5)	2(5)
N(23A)	48(6)	30(5)	57(5)	-3(5)	7(5)	1(4)
N(24A)	36(5)	56(6)	39(5)	2(5)	7(4)	-2(5)
C(21A)	30(6)	34(8)	54(6)	-7(6)	1(5)	-11(6)
C(22A)	37(7)	52(6)	44(6)	-10(6)	3(6)	-1(6)
C(23A)	49(6)	50(6)	35(6)	-3(6)	-3(6)	7(6)
C(24A)	56(9)	88(13)	29(8)	-7(9)	0(7)	8(9)
C(25A)	65(8)	38(6)	49(7)	12(6)	-2(7)	1(6)
C(26A)	63(8)	35(7)	62(6)	11(6)	7(6)	0(6)
C(27A)	42(9)	55(10)	110(15)	-9(11)	2(9)	-8(8)
C(28A)	41(6)	42(7)	56(7)	-11(6)	8(6)	7(6)
C(29A)	34(7)	62(6)	43(7)	-12(6)	4(6)	-1(6)
C(30A)	39(7)	66(7)	41(7)	-5(7)	6(6)	0(6)

C(31A)	44(8)	121(16)	25(8)	-5(10)	7(7)	-16(10)
C(32A)	39(7)	51(6)	49(7)	19(6)	5(6)	5(6)
C(33A)	45(7)	39(6)	62(6)	11(6)	3(5)	1(6)
C(34A)	42(8)	40(9)	80(12)	-17(9)	6(8)	5(7)
F(41)	132(12)	51(5)	29(4)	-16(4)	3(7)	30(7)
F(42)	31(3)	114(10)	72(9)	-62(7)	-11(4)	3(4)
F(43)	68(7)	85(6)	25(4)	-32(4)	19(4)	-27(5)
F(41A)	44(5)	57(5)	67(8)	-38(4)	-6(4)	22(4)
F(42A)	39(4)	75(6)	49(6)	-37(5)	-4(4)	-17(4)
F(43A)	158(13)	65(6)	21(3)	-8(3)	-3(6)	-18(8)
F(44)	75(2)	42(2)	29(2)	14(1)	13(2)	-6(2)
F(45)	46(2)	43(2)	62(2)	22(2)	-14(2)	-22(2)
F(46)	45(2)	30(2)	53(2)	8(1)	18(1)	4(1)
F(51)	41(3)	116(5)	71(3)	-51(3)	40(2)	-48(3)
F(52)	42(3)	47(2)	101(4)	4(2)	24(2)	-22(2)
F(53)	18(2)	114(5)	76(3)	18(3)	-5(2)	-27(2)
F(51A)	41(3)	116(5)	71(3)	-51(3)	40(2)	-48(3)
F(52A)	42(3)	47(2)	101(4)	4(2)	24(2)	-22(2)
F(53A)	42(3)	47(2)	101(4)	4(2)	24(2)	-22(2)
F(54)	71(2)	38(2)	27(2)	1(1)	15(1)	-14(2)
F(55)	52(2)	67(2)	34(2)	-25(2)	6(1)	-23(2)
F(56)	59(2)	81(3)	31(2)	-2(2)	12(1)	44(2)
F(61)	35(2)	24(1)	102(3)	-10(2)	-2(2)	6(1)
F(62)	147(4)	58(2)	26(2)	0(2)	15(2)	68(3)
F(63)	29(2)	48(2)	52(2)	2(1)	-1(1)	22(1)
F(64)	25(2)	61(2)	75(2)	-23(2)	-17(2)	-8(1)
F(65)	75(2)	44(2)	55(2)	0(2)	3(2)	-40(2)
F(66)	37(2)	50(2)	60(2)	-36(2)	6(1)	-12(1)
F(71)	41(3)	38(3)	102(7)	17(3)	48(4)	15(2)
F(72)	23(3)	94(5)	36(3)	-14(3)	-3(2)	22(4)
F(73)	48(3)	38(3)	23(2)	-4(2)	15(2)	15(2)
F(71A)	19(6)	44(6)	93(14)	-17(7)	32(7)	-5(4)
F(72A)	27(9)	58(10)	42(9)	10(8)	12(6)	22(7)
F(73A)	26(6)	84(14)	27(5)	-2(6)	10(5)	6(8)
F(74)	68(5)	84(5)	50(3)	35(3)	-16(3)	-36(4)
F(75)	36(3)	37(3)	95(7)	31(4)	31(3)	7(2)
F(76)	90(7)	17(3)	66(5)	-8(3)	36(4)	-18(3)
F(74A)	92(10)	33(4)	49(5)	4(4)	41(5)	-3(5)
F(75A)	53(5)	77(8)	73(7)	42(6)	-9(5)	-41(5)
F(76A)	68(8)	22(4)	91(10)	19(5)	36(6)	15(5)
C(41)	10(2)	23(2)	20(2)	-2(1)	3(1)	-1(1)
C(42)	12(2)	24(2)	23(2)	-3(2)	2(1)	-1(2)
C(43)	15(2)	36(2)	18(2)	-8(2)	4(1)	1(2)
C(44)	18(2)	40(2)	17(2)	2(2)	4(2)	-1(2)
C(45)	14(2)	30(2)	23(2)	3(2)	4(2)	-3(2)
C(46)	15(2)	23(2)	20(2)	-2(2)	2(1)	-2(2)
C(47)	29(2)	46(3)	24(2)	-12(2)	0(2)	2(2)
C(48)	27(2)	32(2)	24(2)	6(2)	7(2)	-3(2)
C(51)	12(2)	11(2)	19(2)	1(1)	2(1)	-1(1)
C(52)	17(2)	17(2)	22(2)	-4(2)	7(2)	-1(1)
C(53)	18(2)	20(2)	29(2)	-3(2)	4(2)	-4(2)
C(54)	20(2)	15(2)	29(2)	-3(2)	-2(2)	-2(2)
C(55)	19(2)	16(2)	21(2)	-2(1)	1(1)	2(1)
C(56)	14(2)	13(2)	21(2)	2(1)	2(1)	1(1)
C(57)	24(2)	48(3)	50(3)	-10(2)	12(2)	-17(2)
C(58)	28(2)	23(2)	23(2)	-3(2)	3(2)	2(2)
C(61)	16(2)	16(2)	13(2)	2(1)	2(1)	-2(1)
C(62)	17(2)	17(2)	14(2)	3(1)	1(1)	-2(1)
C(63)	19(2)	19(2)	17(2)	2(1)	1(1)	5(1)
C(64)	14(2)	29(2)	16(2)	1(2)	1(1)	0(2)
C(65)	17(2)	24(2)	16(2)	-2(2)	2(1)	-5(2)
C(66)	18(2)	18(2)	19(2)	-4(2)	4(1)	-2(1)
C(67)	23(2)	24(2)	32(2)	1(2)	-2(2)	10(2)
C(68)	22(2)	35(2)	36(2)	-10(2)	-1(2)	-9(2)
C(71)	16(2)	15(2)	16(2)	-6(1)	2(1)	1(1)
C(72)	16(2)	19(2)	21(2)	-5(2)	3(1)	-2(1)
C(73)	16(2)	23(2)	25(2)	-8(2)	3(2)	2(1)
C(74)	23(2)	17(2)	26(2)	-6(2)	2(2)	5(2)
C(75)	20(2)	18(2)	22(2)	-4(2)	2(2)	-1(1)
C(76)	15(2)	16(2)	21(2)	-4(1)	2(1)	0(1)
C(77)	24(2)	26(2)	36(2)	-2(2)	9(2)	7(2)
C(78)	34(2)	18(2)	40(2)	4(2)	7(2)	0(2)
B(41)	12(2)	16(2)	16(2)	-2(2)	4(2)	0(1)
F(81)	43(5)	24(4)	31(3)	-11(3)	0(3)	-4(3)
F(82)	83(6)	35(3)	51(2)	-6(2)	20(4)	-39(4)
F(83)	20(3)	60(5)	153(10)	-60(6)	13(4)	-16(3)
F(81A)	27(6)	22(7)	50(8)	-20(7)	4(6)	-9(5)
F(82A)	83(6)	35(3)	51(2)	-6(2)	20(4)	-39(4)
F(83A)	18(5)	67(9)	79(9)	-37(6)	-13(5)	-8(5)
F(84)	19(3)	36(5)	119(10)	-23(6)	-2(4)	6(3)
F(85)	38(4)	15(3)	37(5)	-6(3)	1(3)	2(2)
F(86)	127(11)	42(5)	38(4)	-2(4)	36(5)	42(7)
F(84A)	36(5)	43(7)	29(5)	14(4)	10(3)	27(4)
F(85A)	38(6)	32(5)	140(14)	-46(7)	10(8)	5(4)
F(86A)	96(10)	67(11)	27(4)	-5(5)	22(5)	51(7)
F(91)	138(12)	44(5)	50(8)	16(5)	59(8)	28(5)
F(92)	60(2)	77(3)	34(2)	-28(2)	8(2)	9(2)
F(93)	60(2)	77(3)	34(2)	-28(2)	8(2)	9(2)
F(91A)	95(7)	85(6)	24(4)	-21(4)	28(4)	-63(6)
F(92A)	60(2)	77(3)	34(2)	-28(2)	8(2)	9(2)
F(93A)	60(2)	77(3)	34(2)	-28(2)	8(2)	9(2)
F(94)	27(3)	95(7)	55(3)	-1(4)	-8(3)	-35(4)
F(95)	38(3)	76(4)	98(4)	33(3)	10(3)	-26(3)
F(96)	27(2)	80(4)	79(4)	-37(3)	30(3)	-23(2)
F(94A)	39(8)	61(9)	96(10)	-57(9)	29(7)	-32(7)

F(95A)	38(3)	76(4)	98(4)	33(3)	10(3)	-26(3)
F(96A)	27(2)	80(4)	79(4)	-37(3)	30(3)	-23(2)
F(101)	65(2)	53(2)	98(3)	26(2)	32(2)	9(2)
F(102)	117(4)	79(3)	34(2)	23(2)	12(2)	-22(3)
F(103)	67(2)	58(2)	74(3)	27(2)	-19(2)	-30(2)
F(104)	50(2)	75(3)	48(2)	-40(2)	11(2)	-9(2)
F(105)	65(7)	109(5)	22(2)	-25(3)	5(3)	-9(4)
F(106)	50(2)	75(3)	48(2)	-40(2)	11(2)	-9(2)
F(04A)	50(2)	75(3)	48(2)	-40(2)	11(2)	-9(2)
F(05A)	65(7)	109(5)	22(2)	-25(3)	5(3)	-9(4)
F(06A)	50(2)	75(3)	48(2)	-40(2)	11(2)	-9(2)
F(111)	50(3)	150(8)	79(3)	-76(4)	10(3)	28(5)
F(112)	29(3)	57(4)	95(8)	0(4)	28(4)	14(2)
F(113)	41(3)	50(2)	88(5)	-24(3)	10(3)	22(2)
F(11B)	50(3)	150(8)	79(3)	-76(4)	10(3)	28(5)
F(12B)	35(8)	52(8)	55(10)	-9(8)	10(6)	2(7)
F(13B)	41(3)	50(2)	88(5)	-24(3)	10(3)	22(2)
F(114)	88(7)	101(8)	89(7)	52(6)	-8(5)	-44(6)
F(115)	73(7)	69(7)	115(9)	59(6)	11(6)	31(5)
F(116)	167(14)	64(6)	76(7)	11(5)	62(8)	7(8)
F(14B)	152(14)	33(4)	122(11)	16(5)	52(10)	-1(6)
F(15B)	107(9)	131(10)	67(5)	41(6)	-2(6)	-40(8)
F(16B)	53(5)	78(7)	124(11)	72(7)	37(5)	14(5)
C(81)	15(2)	16(2)	9(2)	-3(1)	3(1)	0(1)
C(82)	19(2)	13(2)	20(2)	-4(1)	4(1)	0(1)
C(83)	15(2)	21(2)	21(2)	-5(2)	4(1)	-5(1)
C(84)	14(2)	23(2)	19(2)	-1(2)	4(1)	-2(1)
C(85)	14(2)	16(2)	20(2)	-1(1)	4(1)	3(1)
C(86)	16(2)	12(2)	18(2)	-3(1)	4(1)	-2(1)
C(87)	25(2)	31(2)	43(2)	-15(2)	11(2)	-14(2)
C(88)	23(2)	22(2)	42(2)	-7(2)	6(2)	6(2)
C(91)	14(2)	12(2)	21(2)	-4(1)	2(1)	1(1)
C(92)	18(2)	13(2)	19(2)	-2(1)	2(1)	-1(1)
C(93)	22(2)	17(2)	20(2)	-3(1)	0(2)	-1(2)
C(94)	20(2)	18(2)	31(2)	-7(2)	-7(2)	0(2)
C(95)	13(2)	21(2)	33(2)	-4(2)	0(2)	-2(1)
C(96)	14(2)	20(2)	23(2)	-5(2)	3(1)	1(1)
C(97)	38(2)	36(2)	23(2)	-9(2)	3(2)	-7(2)
C(98)	20(2)	54(3)	46(3)	-13(2)	5(2)	-15(2)
C(101)	8(2)	31(2)	21(2)	-7(2)	3(1)	-3(2)
C(102)	15(2)	32(2)	18(2)	-4(2)	4(1)	-3(2)
C(103)	17(2)	48(3)	23(2)	5(2)	6(2)	-5(2)
C(104)	19(2)	71(3)	15(2)	-2(2)	5(2)	-8(2)
C(105)	20(2)	64(3)	21(2)	-14(2)	6(2)	-9(2)
C(106)	15(2)	40(2)	26(2)	-12(2)	6(2)	-5(2)
C(107)	35(3)	54(3)	30(2)	13(2)	5(2)	-6(2)
C(108)	41(3)	83(4)	25(2)	-29(2)	8(2)	-10(2)
C(111)	15(2)	20(2)	22(2)	-12(2)	1(1)	1(1)
C(112)	20(2)	24(2)	30(2)	-15(2)	6(2)	-1(2)
C(113)	21(2)	28(2)	49(3)	-19(2)	0(2)	5(2)
C(114)	27(2)	23(2)	58(3)	-10(2)	-6(2)	9(2)
C(115)	29(2)	29(2)	43(3)	-1(2)	-5(2)	6(2)
C(116)	19(2)	24(2)	31(2)	-6(2)	2(2)	4(2)
C(117)	33(3)	45(3)	74(3)	-31(3)	11(2)	13(2)
C(118)	49(3)	44(3)	73(4)	21(3)	6(3)	15(2)
B(81)	14(2)	18(2)	19(2)	-8(2)	4(2)	-2(2)
O(1S)	39(2)	24(2)	37(2)	3(1)	-1(2)	2(1)
C(1S)	58(4)	40(3)	94(5)	34(3)	-17(4)	-7(3)
C(2S)	62(4)	51(3)	57(4)	18(3)	13(3)	-4(3)
C(3S)	38(3)	32(2)	49(3)	-10(2)	-2(2)	4(2)
C(4S)	49(3)	24(2)	61(4)	4(2)	-12(3)	2(2)
O(11S)	55(2)	51(2)	31(2)	16(2)	2(2)	-5(2)
C(11S)	62(5)	184(10)	66(5)	61(6)	-15(4)	17(5)
C(12S)	84(5)	98(6)	52(4)	40(4)	3(3)	19(4)
C(13S)	79(4)	71(4)	44(3)	4(3)	9(3)	-32(4)
C(14S)	65(5)	122(7)	53(4)	13(4)	-6(3)	-40(5)
O(21S)	40(2)	48(2)	51(2)	-17(2)	14(2)	-11(2)
C(21S)	59(4)	41(3)	47(3)	-5(3)	20(3)	10(3)
C(22S)	67(4)	33(3)	41(3)	-7(2)	11(3)	4(2)
C(23S)	42(3)	50(3)	56(3)	-11(3)	7(3)	-12(3)
C(24S)	61(4)	80(5)	71(4)	-21(4)	27(4)	-26(4)
O(31S)	106(4)	37(2)	50(2)	-10(2)	-18(2)	8(2)
C(31S)	66(4)	32(3)	72(4)	-5(3)	-6(3)	-2(3)
C(32S)	90(5)	52(3)	48(3)	9(3)	-1(3)	12(3)
C(33S)	144(8)	54(4)	66(5)	-17(3)	-18(5)	19(5)
C(34S)	146(8)	46(4)	78(5)	-31(4)	-50(5)	26(4)

Table S61. Hydrogen coordinates (x 10^4) and isotropic displacement parameters ($Å^2$x 10^3) for **6a-B$_{Ar}$F**.

	x	y	z	U(eq)
H(1A)	-652	6917	1110	58
H(1AB)	-1112	6935	601	58
H(2A)	-13	7678	894	65
H(2AB)	-953	7718	995	65
H(3A)	-1176	7705	136	52
H(3AB)	-726	8189	329	52
H(4A)	1162	7934	-130	75
H(4B)	873	7889	421	75
H(4C)	614	8357	102	75
H(5A)	-223	8313	-540	50
H(5AB)	-812	7851	-625	50
H(6A)	144	7881	-1260	56
H(6AB)	864	7861	-873	56
H(7A)	941	7144	-1566	73
H(7B)	1023	6672	-1220	73
H(7C)	1481	7174	-1086	73
H(8A)	-415	7150	-1561	47
H(8AB)	-930	7198	-1076	47
H(9A)	-1003	6400	-1462	52
H(9AB)	-82	6319	-1323	52
H(10A)	-1307	6441	-620	43
H(10B)	-994	5912	-794	43
H(11A)	243	5587	-485	64
H(11B)	898	5959	-267	64
H(11C)	632	6002	-823	64
H(12A)	-1121	6264	135	49
H(12B)	-676	5741	92	49
H(13A)	-337	6131	828	57
H(13B)	445	6056	500	57
H(14A)	1241	6637	735	74
H(14B)	671	6750	1186	74
H(14C)	966	7192	846	74
H(1AA)	-1143	7239	553	44
H(1AC)	-752	7148	1074	44
H(2AA)	215	7760	901	53
H(2AC)	-679	7969	930	53
H(3AA)	-701	7930	46	57
H(3AC)	-168	8359	283	57
H(4AA)	1707	7855	102	74
H(4AB)	1198	7832	591	74
H(4AC)	1232	8333	285	74
H(5AA)	420	8421	-464	58
H(5AC)	1121	8048	-618	58
H(6AA)	126	7983	-1183	58
H(6AC)	-541	7914	-773	58
H(7AA)	890	6823	-1201	64
H(7AB)	1230	7374	-1152	64
H(7AC)	576	7247	-1559	64
H(8AA)	-776	7250	-1408	47
H(8AC)	-1116	7239	-868	47
H(9AA)	-1239	6466	-1306	48
H(9AC)	-285	6418	-1281	48
H(10C)	-1254	6438	-428	40
H(10D)	-978	5932	-680	40
H(11D)	354	5677	-686	66
H(11E)	1056	6008	-458	66
H(11F)	629	6174	-952	66
H(12C)	-393	5739	104	40
H(12D)	434	5997	262	40
H(13C)	-619	6229	793	40
H(13D)	-1101	6463	342	40
H(14D)	944	6503	721	62
H(14E)	363	6614	1168	62
H(14F)	885	7050	938	62
H(21A)	11165	2568	4961	38
H(21B)	10840	3121	4909	38
H(22A)	10392	3077	5721	42
H(22B)	11344	3068	5660	42
H(23A)	11329	2197	5781	39
H(23B)	11038	2500	6244	39
H(24A)	9396	2691	6005	56
H(24B)	9688	2462	6511	56
H(24C)	9021	2182	6195	56
H(25A)	10887	1523	6096	43
H(25B)	10376	1715	6550	43
H(26A)	9211	1369	6226	48
H(26B)	9886	949	6271	48
H(27A)	8806	826	5165	69
H(27B)	8494	1022	5678	69
H(27C)	9029	532	5650	69
H(28A)	10370	540	5580	43
H(28B)	10873	1037	5501	43
H(29A)	10777	494	4804	42
H(29B)	9853	646	4766	42
H(30A)	10684	1087	4217	37
H(30B)	11133	1335	4668	37
H(31A)	9406	1333	3977	57
H(31B)	8886	1725	4275	57
H(31C)	9020	1179	4484	57
H(32A)	10952	2065	4388	39
H(32B)	10372	1962	3934	39
H(33A)	9403	2478	4256	43

H(33B)	10232	2774	4210	43
H(34A)	9525	3307	4819	62
H(34B)	9309	3086	5340	62
H(34C)	8826	2905	4870	62
H(21C)	11273	2526	5571	47
H(21D)	10909	3041	5745	47
H(22C)	10096	2614	6303	53
H(22D)	11033	2564	6412	53
H(23C)	10978	1760	6032	54
H(23D)	10538	1803	6544	54
H(24D)	9026	2257	6274	86
H(24E)	9189	1798	6627	86
H(24F)	8584	1740	6179	86
H(25C)	10046	1073	6305	61
H(25D)	9238	1046	5989	61
H(26C)	10825	1069	5617	64
H(26D)	10215	612	5582	64
H(27D)	9013	1041	4663	104
H(27E)	8886	810	5191	104
H(27F)	9485	550	4818	104
H(28C)	11132	1235	4917	56
H(28D)	10723	741	4718	56
H(29C)	10962	1208	4064	56
H(29D)	10018	1216	4140	56
H(30C)	10536	1972	3875	58
H(30D)	11019	2022	4377	58
H(31D)	9212	1963	3826	95
H(31E)	8638	2145	4254	95
H(31F)	8962	1588	4248	95
H(32C)	10087	2745	4137	56
H(32D)	9335	2784	4494	56
H(33C)	10964	2691	4792	59
H(33D)	10429	3180	4844	59
H(34D)	9056	2985	5215	81
H(34E)	9656	3298	5550	81
H(34F)	9201	2827	5767	81
H(42)	2430	6757	2900	24
H(44)	2321	5684	3921	30
H(46)	2262	5328	2498	23
H(52)	1006	5749	2104	23
H(54)	769	5063	807	26
H(56)	2730	5900	1089	19
H(62)	3519	5509	1714	19
H(64)	5709	6095	2013	23
H(66)	3693	6899	2233	22
H(72)	1079	6697	2339	22
H(74)	1170	8054	1722	26
H(76)	2949	7063	1487	21
H(82)	6348	4278	2782	21
H(84)	4263	5000	3036	23
H(86)	6390	5678	3280	18
H(92)	7204	5285	3906	20
H(94)	9231	6027	4277	27
H(96)	8944	5494	2917	23
H(102)	7733	5917	2512	26
H(104)	7533	5601	1082	42
H(106)	7465	4503	2078	33
H(112)	8851	4518	2561	30
H(114)	8957	3218	3283	44
H(116)	7177	4193	3561	30
H(1SA)	7844	8550	2579	96
H(1SB)	8058	8451	3136	96
H(1SC)	7150	8404	2951	96
H(2SA)	7546	7727	2459	68
H(2SB)	8424	7760	2696	68
H(3SA)	8321	6960	3079	47
H(3SB)	7518	6916	2759	47
H(4SA)	6767	6785	3450	67
H(4SB)	7544	6864	3783	67
H(4SC)	7482	6391	3437	67
H(11G)	3794	4427	3955	155
H(11H)	3862	4214	4494	155
H(11I)	4239	3912	4051	155
H(12E)	5214	4502	3944	93
H(12F)	4799	4857	4336	93
H(13E)	5977	4859	4812	78
H(13F)	6403	4516	4416	78
H(14G)	6664	3924	4987	120
H(14H)	6164	4223	5384	120
H(14I)	6992	4442	5187	120
H(21E)	4007	8890	828	74
H(21F)	4188	9015	1385	74
H(21G)	4728	8618	1106	74
H(22E)	4697	9647	905	56
H(22F)	5428	9362	1162	56
H(23E)	6337	9571	518	59
H(23F)	5615	9900	307	59
H(24G)	5643	9447	-412	106
H(24H)	6364	9119	-203	106
H(24I)	6499	9694	-311	106
H(31G)	7351	685	6273	85
H(31H)	7366	214	6624	85
H(31I)	8148	552	6571	85
H(32S)	6712	801	7020	76
H(32T)	7580	757	7271	76

H(33S)	7647	1576	7513	106
H(33T)	6719	1539	7371	106
H(34G)	7772	2255	7049	135
H(34H)	7095	2327	7452	135
H(34I)	6844	2217	6902	135

[(D$_{12}$-TMC)Co^{II}(F)]B$_{Ar}$F, **7b-B$_{Ar}$F**

The asymmetric unit contains one complex molecule and one disordered B$_{Ar}$F anion. The disordered CF_3 group was refined in four different positions with population of 0.259(3), 0.397(3), 0.145(3) and 0.198(3) using PART/SUMP commands and some restraints (SADI, SIMU, RIGU).

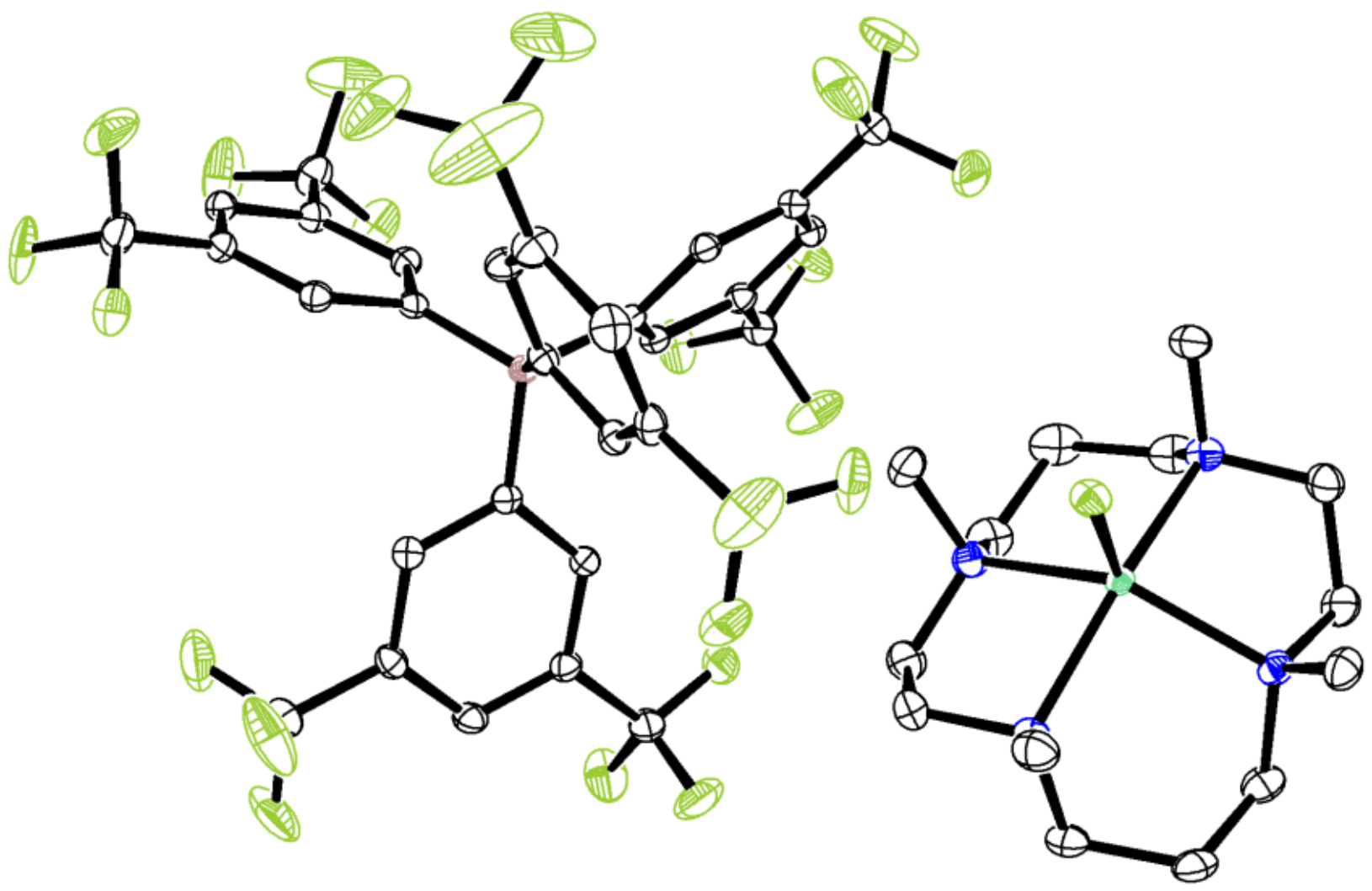

Fig S13. Molecular structure of **7b-B$_{Ar}$F**.

Table S62. Crystal data and structure refinement for **7b-B$_{Ar}$F**.

CCDC No	2502782	
Empirical formula	$C_{46}H_{32}BCoD_{12}F_{25}N_4$	
Formula weight	1209.66	
Temperature	100(2) K	
Wavelength	0.71073 Å	
Crystal system	Triclinic	
Space group	$P\bar{1}$	
Unit cell dimensions	a = 12.9349(9) Å	α = 96.314(3)°
	b = 14.0562(10) Å	β = 106.791(3)°
	c = 14.2775(10) Å	γ = 93.058(3)°
Volume	2460.2(3) Å^3	
Z	2	
Density (calculated)	1.633 Mg/m^3	
Absorption coefficient	0.483 mm^{-1}	
F(000)	1210	
Crystal size	0.262 x 0.259 x 0.192 mm^3	
Theta range for data collection	1.955 to 28.282°.	
Index ranges	-17 ≤ h ≤ 17, -18 ≤ k ≤ 18, -19 ≤ l ≤ 19	
Reflections collected	269350	
Independent reflections	12203 [R(int) = 0.1059]	
Completeness to theta = 25.242°	99.9 %	
Absorption correction	Semi-empirical from equivalents	
Max. and min. transmission	0.7474 and 0.6651	
Refinement method	Full-matrix least-squares on F^2	
Data / restraints / parameters	12203 / 1100 / 783	
Goodness-of-fit on F^2	1.072	
Final R indices [I>2sigma(I)]	R1 = 0.0382, wR2 = 0.0962	
R indices (all data)	R1 = 0.0471, wR2 = 0.1041	
Extinction coefficient	n/a	
Largest diff. peak and hole	0.611 and -0.569 eÅ^{-3}	

Table S63. Atomic coordinates (x 10^4) and equivalent isotropic displacement parameters ($Å^2$ x 10^3) for **7b-B$_{ArF}$F**.
U(eq) is defined as one third of the trace of the orthogonalized U^{ij} tensor.

	x	y	z	U(eq)
Co(1)	10342(1)	6666(1)	9363(1)	13(1)
F(1)	9411(1)	5546(1)	9098(1)	20(1)
N(1)	9264(1)	7726(1)	8878(1)	22(1)
B(1)	4559(1)	7737(1)	6714(1)	12(1)
C(1)	8145(1)	7315(1)	8766(1)	27(1)
D(1A)	7629	7781	8518	41
D(1B)	8092	7172	9408	41
D(1C)	7976	6723	8299	41
N(2)	10411(1)	6512(1)	7809(1)	20(1)
F(11)	5345(1)	6030(1)	9867(1)	48(1)
C(2)	10272(2)	5502(1)	7342(1)	26(1)
D(2A)	9644	5167	7457	38
D(2B)	10924	5185	7629	38
D(2C)	10154	5486	6631	38
F(12)	6923(1)	6699(1)	10652(1)	56(1)
N(3)	12032(1)	6559(1)	9929(1)	18(1)
C(3)	12242(1)	5528(1)	9898(1)	23(1)
D(3A)	11820	5204	10260	34
D(3B)	13016	5475	10204	34
D(3C)	12029	5225	9211	34
F(13)	5538(1)	7130(1)	11051(1)	45(1)
N(4)	10454(1)	7093(1)	10915(1)	19(1)
C(4)	9600(1)	6562(1)	11198(1)	22(1)
D(4A)	8889	6747	10836	33
D(4B)	9715	6717	11909	33
D(4C)	9629	5870	11036	33
F(5)	6515(1)	10508(1)	10655(1)	35(1)
C(5)	9334(2)	7902(1)	7889(1)	28(1)
F(6)	5935(1)	10891(1)	9200(1)	33(1)
C(6)	9442(2)	6979(1)	7290(1)	26(1)
C(7)	11390(1)	7006(1)	7676(1)	24(1)
F(7)	7522(1)	10407(1)	9698(1)	39(1)
F(8)	2504(1)	10703(1)	7496(1)	36(1)
C(8)	12461(1)	6735(1)	8322(1)	24(1)
C(9)	12699(1)	7072(1)	9415(1)	24(1)
F(9)	1315(1)	9823(1)	7844(1)	55(1)
C(10)	12371(1)	7006(1)	10980(1)	24(1)
F(10)	882(1)	10477(1)	6525(1)	54(1)
C(11)	11512(1)	6762(1)	11462(1)	23(1)
C(12)	10456(2)	8138(1)	11206(1)	27(1)
C(13)	9504(2)	8603(1)	10587(2)	31(1)
C(14)	9495(2)	8663(1)	9531(2)	29(1)
F(14)	3709(1)	8760(1)	3085(1)	56(1)
C(15)	5150(1)	8038(1)	7909(1)	13(1)
F(15)	4660(1)	7616(1)	2914(1)	51(1)
C(16)	5539(1)	8987(1)	8327(1)	14(1)
F(16)	5231(1)	9044(1)	2857(1)	56(1)
C(17)	6038(1)	9238(1)	9335(1)	16(1)
F(17)	7936(1)	10553(1)	5900(1)	44(1)
C(19)	5765(1)	7616(1)	9591(1)	16(1)
F(19)	8161(1)	9916(1)	7232(1)	28(1)
C(18)	6156(1)	8557(1)	9984(1)	18(1)
F(18)	8644(1)	9206(1)	6041(1)	42(1)
C(20)	5286(1)	7361(1)	8582(1)	14(1)
F(20)	7248(1)	5316(1)	5882(1)	36(1)
C(30)	6491(1)	10253(1)	9719(1)	20(1)
F(21)	7407(1)	4961(1)	7325(1)	38(1)
C(24)	3003(1)	8878(1)	6873(1)	15(1)
F(24)	1956(1)	4530(1)	5948(1)	56(1)
C(25)	1962(1)	9177(1)	6550(1)	16(1)
F(25)	2939(1)	3361(1)	6032(2)	82(1)
C(26)	1168(1)	8666(1)	5758(1)	17(1)
C(27)	1454(1)	7851(1)	5283(1)	16(1)
C(28)	2498(1)	7564(1)	5587(1)	15(1)
C(29)	1669(1)	10046(1)	7089(1)	24(1)
C(21)	623(1)	7253(1)	4443(1)	25(1)
C(31)	5212(1)	8263(1)	6047(1)	13(1)
C(32)	4737(1)	8219(1)	5024(1)	15(1)
C(33)	5257(1)	8632(1)	4420(1)	17(1)
C(34)	6272(1)	9132(1)	4812(1)	18(1)
C(35)	6763(1)	9184(1)	5819(1)	17(1)
C(36)	6256(1)	8746(1)	6418(1)	15(1)
C(37)	4720(1)	8515(1)	3328(1)	21(1)
C(38)	7865(1)	9716(1)	6245(1)	23(1)
C(39)	4590(1)	6569(1)	6495(1)	14(1)
C(40)	3748(1)	5899(1)	6494(1)	18(1)
C(41)	3842(1)	4914(1)	6415(1)	22(1)
C(42)	4802(1)	4551(1)	6359(1)	21(1)
C(43)	5651(1)	5199(1)	6371(1)	18(1)
C(44)	5542(1)	6181(1)	6421(1)	15(1)
C(45)	2903(2)	4244(1)	6423(2)	36(1)
C(46)	6726(1)	4845(1)	6399(1)	23(1)
C(23)	3313(1)	8062(1)	6399(1)	13(1)
F(23)	2849(2)	4207(1)	7344(1)	72(1)
C(22)	5897(1)	6870(1)	10285(1)	22(1)
F(22)	6660(1)	3910(1)	6067(1)	44(1)
F(2)	-51(9)	7770(7)	3845(8)	39(2)
F(3)	1060(8)	6681(7)	3838(8)	22(2)
F(4)	29(9)	6645(9)	4772(6)	40(2)
F(2A)	1007(6)	6989(7)	3685(4)	37(2)
F(3A)	-305(6)	7623(5)	4119(6)	39(2)

F(4A)	362(6)	6383(4)	4733(5)	33(2)
F(2B)	361(15)	7790(9)	3651(8)	28(2)
F(3B)	-328(10)	7121(17)	4638(11)	39(3)
F(4B)	888(12)	6436(9)	4104(14)	33(3)
F(2C)	773(10)	7456(12)	3596(5)	38(2)
F(3C)	-398(8)	7469(10)	4416(10)	28(2)
F(4C)	655(10)	6330(6)	4484(10)	33(2)

Table S64. Bond lengths [Å] and angles [°] for **7b-B$_{Ar}$F**.

Co(1)-F(1)	1.8650(10)	F(1)-Co(1)-N(3)	118.28(5)
Co(1)-N(3)	2.1200(13)	F(1)-Co(1)-N(1)	102.76(5)
Co(1)-N(1)	2.1290(14)	N(3)-Co(1)-N(1)	138.84(6)
Co(1)-N(4)	2.1913(14)	F(1)-Co(1)-N(4)	100.01(5)
Co(1)-N(2)	2.2328(14)	N(3)-Co(1)-N(4)	83.83(5)
N(1)-C(5)	1.487(2)	N(1)-Co(1)-N(4)	92.53(6)
N(1)-C(1)	1.488(2)	F(1)-Co(1)-N(2)	91.22(5)
N(1)-C(14)	1.490(2)	N(3)-Co(1)-N(2)	92.16(5)
B(1)-C(39)	1.643(2)	N(1)-Co(1)-N(2)	83.51(5)
B(1)-C(23)	1.648(2)	N(4)-Co(1)-N(2)	168.68(5)
B(1)-C(31)	1.650(2)	C(5)-N(1)-C(1)	108.38(14)
B(1)-C(15)	1.650(2)	C(5)-N(1)-C(14)	108.38(14)
N(2)-C(2)	1.479(2)	C(1)-N(1)-C(14)	109.61(14)
N(2)-C(7)	1.482(2)	C(5)-N(1)-Co(1)	107.11(10)
N(2)-C(6)	1.486(2)	C(1)-N(1)-Co(1)	107.83(10)
F(11)-C(22)	1.329(2)	C(14)-N(1)-Co(1)	115.32(11)
F(12)-C(22)	1.325(2)	C(39)-B(1)-C(23)	112.17(12)
N(3)-C(3)	1.487(2)	C(39)-B(1)-C(31)	108.68(12)
N(3)-C(10)	1.490(2)	C(23)-B(1)-C(31)	107.18(11)
N(3)-C(9)	1.490(2)	C(39)-B(1)-C(15)	106.28(11)
F(13)-C(22)	1.330(2)	C(23)-B(1)-C(15)	110.66(12)
N(4)-C(4)	1.476(2)	C(31)-B(1)-C(15)	111.93(12)
N(4)-C(12)	1.481(2)	C(2)-N(2)-C(7)	109.39(14)
N(4)-C(11)	1.492(2)	C(2)-N(2)-C(6)	107.11(14)
F(5)-C(30)	1.3366(19)	C(7)-N(2)-C(6)	108.96(13)
C(5)-C(6)	1.510(3)	C(2)-N(2)-Co(1)	113.50(10)
F(6)-C(30)	1.333(2)	C(7)-N(2)-Co(1)	114.59(11)
C(7)-C(8)	1.520(2)	C(6)-N(2)-Co(1)	102.76(10)
F(7)-C(30)	1.349(2)	C(3)-N(3)-C(10)	108.50(13)
F(8)-C(29)	1.330(2)	C(3)-N(3)-C(9)	110.29(13)
C(8)-C(9)	1.516(3)	C(10)-N(3)-C(9)	107.42(13)
F(9)-C(29)	1.346(2)	C(3)-N(3)-Co(1)	109.28(10)
C(10)-C(11)	1.510(2)	C(10)-N(3)-Co(1)	107.03(10)
F(10)-C(29)	1.324(2)	C(9)-N(3)-Co(1)	114.12(10)
C(12)-C(13)	1.520(3)	C(4)-N(4)-C(12)	109.87(13)
C(13)-C(14)	1.516(3)	C(4)-N(4)-C(11)	107.09(13)
F(14)-C(37)	1.328(2)	C(12)-N(4)-C(11)	109.33(13)
C(15)-C(16)	1.403(2)	C(4)-N(4)-Co(1)	111.28(10)
C(15)-C(20)	1.408(2)	C(12)-N(4)-Co(1)	115.28(11)
F(15)-C(37)	1.323(2)	C(11)-N(4)-Co(1)	103.51(10)
C(16)-C(17)	1.393(2)	N(1)-C(5)-C(6)	111.02(14)
F(16)-C(37)	1.326(2)	N(2)-C(6)-C(5)	111.08(15)
C(17)-C(18)	1.388(2)	N(2)-C(7)-C(8)	114.90(14)
C(17)-C(30)	1.495(2)	C(9)-C(8)-C(7)	114.85(15)
F(17)-C(38)	1.333(2)	N(3)-C(9)-C(8)	115.70(14)
C(19)-C(18)	1.386(2)	N(3)-C(10)-C(11)	110.28(13)
C(19)-C(20)	1.391(2)	N(4)-C(11)-C(10)	110.31(14)
C(19)-C(22)	1.503(2)	N(4)-C(12)-C(13)	114.92(15)
F(19)-C(38)	1.342(2)	C(14)-C(13)-C(12)	115.27(16)
F(18)-C(38)	1.348(2)	N(1)-C(14)-C(13)	115.06(15)
F(20)-C(46)	1.337(2)	C(16)-C(15)-C(20)	115.22(13)
F(21)-C(46)	1.347(2)	C(16)-C(15)-B(1)	122.47(13)
C(24)-C(25)	1.395(2)	C(20)-C(15)-B(1)	122.30(13)
C(24)-C(23)	1.403(2)	C(17)-C(16)-C(15)	122.31(14)
F(24)-C(45)	1.325(3)	C(18)-C(17)-C(16)	121.33(14)
C(25)-C(26)	1.386(2)	C(18)-C(17)-C(30)	119.40(14)
C(25)-C(29)	1.497(2)	C(16)-C(17)-C(30)	119.24(14)
F(25)-C(45)	1.314(2)	C(18)-C(19)-C(20)	121.24(14)
C(26)-C(27)	1.388(2)	C(18)-C(19)-C(22)	118.09(14)
C(27)-C(28)	1.391(2)	C(20)-C(19)-C(22)	120.66(15)
C(27)-C(21)	1.495(2)	C(19)-C(18)-C(17)	117.50(14)
C(28)-C(23)	1.408(2)	C(19)-C(20)-C(15)	122.37(14)
C(21)-F(4B)	1.299(9)	F(6)-C(30)-F(5)	106.24(14)
C(21)-F(4C)	1.308(8)	F(6)-C(30)-F(7)	106.12(14)
C(21)-F(3A)	1.313(6)	F(5)-C(30)-F(7)	105.80(14)
C(21)-F(4)	1.331(7)	F(6)-C(30)-C(17)	113.07(13)
C(21)-F(2C)	1.336(7)	F(5)-C(30)-C(17)	113.18(14)
C(21)-F(2)	1.338(7)	F(7)-C(30)-C(17)	111.86(14)
C(21)-F(2A)	1.342(5)	C(25)-C(24)-C(23)	122.04(14)
C(21)-F(3B)	1.347(9)	C(26)-C(25)-C(24)	121.55(14)
C(21)-F(3C)	1.362(8)	C(26)-C(25)-C(29)	118.50(14)
C(21)-F(3)	1.375(7)	C(24)-C(25)-C(29)	119.90(14)
C(21)-F(4A)	1.389(5)	C(25)-C(26)-C(27)	117.42(14)
C(21)-F(2B)	1.399(8)	C(26)-C(27)-C(28)	121.20(14)
C(31)-C(32)	1.404(2)	C(26)-C(27)-C(21)	119.86(14)
C(31)-C(36)	1.405(2)	C(28)-C(27)-C(21)	118.93(14)
C(32)-C(33)	1.392(2)	C(27)-C(28)-C(23)	122.40(14)
C(33)-C(34)	1.386(2)	F(10)-C(29)-F(8)	107.44(15)
C(33)-C(37)	1.499(2)	F(10)-C(29)-F(9)	105.78(17)
C(34)-C(35)	1.386(2)	F(8)-C(29)-F(9)	105.11(16)
C(35)-C(36)	1.393(2)	F(10)-C(29)-C(25)	113.17(15)
C(35)-C(38)	1.498(2)	F(8)-C(29)-C(25)	113.09(14)
C(39)-C(44)	1.400(2)	F(9)-C(29)-C(25)	111.66(15)
C(39)-C(40)	1.401(2)	F(4C)-C(21)-F(2C)	109.5(6)
C(40)-C(41)	1.392(2)	F(4)-C(21)-F(2)	108.0(5)
C(41)-C(42)	1.387(2)	F(3A)-C(21)-F(2A)	109.6(4)
C(41)-C(45)	1.499(2)	F(4B)-C(21)-F(3B)	109.7(7)
C(42)-C(43)	1.384(2)	F(4C)-C(21)-F(3C)	107.2(6)
C(43)-C(44)	1.390(2)	F(2C)-C(21)-F(3C)	106.7(6)
C(43)-C(46)	1.494(2)	F(4)-C(21)-F(3)	104.8(5)
C(45)-F(23)	1.343(3)	F(2)-C(21)-F(3)	105.3(5)
C(46)-F(22)	1.337(2)	F(3A)-C(21)-F(4A)	105.2(4)
		F(2A)-C(21)-F(4A)	103.3(4)

F(4B)-C(21)-F(2B)	106.2(7)	C(42)-C(41)-C(40)	120.76(15)
F(3B)-C(21)-F(2B)	102.7(7)	C(42)-C(41)-C(45)	119.96(15)
C(32)-C(31)-C(36)	115.30(13)	C(40)-C(41)-C(45)	119.25(16)
C(32)-C(31)-B(1)	119.85(13)	C(43)-C(42)-C(41)	117.78(15)
C(36)-C(31)-B(1)	124.82(13)	C(42)-C(43)-C(44)	121.28(15)
C(33)-C(32)-C(31)	122.43(14)	C(42)-C(43)-C(46)	119.35(15)
C(34)-C(33)-C(32)	121.02(14)	C(44)-C(43)-C(46)	119.24(15)
C(34)-C(33)-C(37)	119.63(14)	C(43)-C(44)-C(39)	122.16(14)
C(32)-C(33)-C(37)	119.33(14)	F(25)-C(45)-F(24)	107.2(2)
C(35)-C(34)-C(33)	117.78(14)	F(25)-C(45)-F(23)	107.5(2)
C(34)-C(35)-C(36)	121.17(14)	F(24)-C(45)-F(23)	104.28(19)
C(34)-C(35)-C(38)	118.05(14)	F(25)-C(45)-C(41)	113.18(17)
C(36)-C(35)-C(38)	120.78(14)	F(24)-C(45)-C(41)	112.52(16)
C(35)-C(36)-C(31)	122.23(14)	F(23)-C(45)-C(41)	111.59(19)
F(15)-C(37)-F(16)	106.26(16)	F(22)-C(46)-F(20)	107.14(15)
F(15)-C(37)-F(14)	105.57(16)	F(22)-C(46)-F(21)	106.16(15)
F(16)-C(37)-F(14)	105.47(16)	F(20)-C(46)-F(21)	104.90(15)
F(15)-C(37)-C(33)	112.41(14)	F(22)-C(46)-C(43)	113.12(15)
F(16)-C(37)-C(33)	113.11(14)	F(20)-C(46)-C(43)	113.40(14)
F(14)-C(37)-C(33)	113.36(14)	F(21)-C(46)-C(43)	111.51(14)
F(17)-C(38)-F(19)	106.37(15)	C(24)-C(23)-C(28)	115.35(13)
F(17)-C(38)-F(18)	106.69(15)	C(24)-C(23)-B(1)	123.03(13)
F(19)-C(38)-F(18)	105.46(14)	C(28)-C(23)-B(1)	121.50(13)
F(17)-C(38)-C(35)	112.59(14)	F(12)-C(22)-F(11)	106.90(16)
F(19)-C(38)-C(35)	113.28(14)	F(12)-C(22)-F(13)	106.58(15)
F(18)-C(38)-C(35)	111.92(15)	F(11)-C(22)-F(13)	105.03(15)
C(44)-C(39)-C(40)	115.44(14)	F(12)-C(22)-C(19)	112.29(14)
C(44)-C(39)-B(1)	120.32(13)	F(11)-C(22)-C(19)	113.27(14)
C(40)-C(39)-B(1)	123.75(13)	F(13)-C(22)-C(19)	112.24(14)
C(41)-C(40)-C(39)	122.54(15)		

Table S65. Anisotropic displacement parameters (Å^2 x 10^3) for **7b-B$_{ArF}$**.
The anisotropic displacement factor exponent takes the form: -2p^2[h^2 a*^{2} U^{11} + ... + 2 h k a* b* U^{12}]

	U^{11}	U^{22}	U^{33}	U^{23}	U^{13}	U^{12}
Co(1)	12(1)	12(1)	15(1)	3(1)	4(1)	0(1)
F(1)	22(1)	17(1)	21(1)	3(1)	6(1)	-4(1)
N(1)	22(1)	19(1)	28(1)	9(1)	11(1)	7(1)
B(1)	13(1)	11(1)	13(1)	2(1)	4(1)	0(1)
C(1)	19(1)	33(1)	33(1)	11(1)	9(1)	8(1)
N(2)	18(1)	23(1)	19(1)	6(1)	8(1)	2(1)
F(11)	72(1)	28(1)	36(1)	17(1)	3(1)	-10(1)
C(2)	28(1)	28(1)	21(1)	0(1)	9(1)	-1(1)
F(12)	24(1)	83(1)	73(1)	62(1)	14(1)	19(1)
N(3)	14(1)	18(1)	19(1)	0(1)	4(1)	-1(1)
C(3)	19(1)	20(1)	29(1)	6(1)	6(1)	4(1)
F(13)	70(1)	48(1)	33(1)	22(1)	33(1)	19(1)
N(4)	19(1)	18(1)	19(1)	-1(1)	7(1)	-2(1)
C(4)	23(1)	25(1)	20(1)	2(1)	9(1)	-1(1)
F(5)	54(1)	28(1)	19(1)	-7(1)	11(1)	-7(1)
C(5)	29(1)	29(1)	32(1)	17(1)	12(1)	10(1)
F(6)	42(1)	16(1)	31(1)	0(1)	-2(1)	1(1)
C(6)	23(1)	36(1)	20(1)	11(1)	4(1)	5(1)
C(7)	24(1)	28(1)	26(1)	9(1)	13(1)	0(1)
F(7)	25(1)	27(1)	63(1)	-10(1)	20(1)	-10(1)
F(8)	30(1)	22(1)	49(1)	-12(1)	8(1)	1(1)
C(8)	20(1)	27(1)	30(1)	6(1)	14(1)	-1(1)
C(9)	17(1)	24(1)	31(1)	2(1)	8(1)	-5(1)
F(9)	80(1)	41(1)	65(1)	-4(1)	58(1)	8(1)
C(10)	18(1)	28(1)	21(1)	-1(1)	1(1)	-4(1)
F(10)	45(1)	36(1)	61(1)	-12(1)	-12(1)	28(1)
C(11)	21(1)	26(1)	18(1)	2(1)	3(1)	-2(1)
C(12)	34(1)	19(1)	29(1)	-6(1)	15(1)	-4(1)
C(13)	43(1)	16(1)	42(1)	0(1)	24(1)	6(1)
C(14)	35(1)	16(1)	42(1)	7(1)	18(1)	8(1)
F(14)	39(1)	99(1)	23(1)	2(1)	-2(1)	34(1)
C(15)	10(1)	15(1)	14(1)	3(1)	4(1)	1(1)
F(15)	93(1)	26(1)	20(1)	-2(1)	-4(1)	13(1)
C(16)	13(1)	15(1)	15(1)	2(1)	5(1)	1(1)
F(16)	64(1)	75(1)	20(1)	20(1)	2(1)	-31(1)
C(17)	15(1)	16(1)	16(1)	0(1)	5(1)	-1(1)
F(17)	37(1)	43(1)	45(1)	26(1)	-1(1)	-20(1)
C(19)	14(1)	20(1)	16(1)	6(1)	6(1)	2(1)
F(19)	23(1)	32(1)	23(1)	4(1)	1(1)	-9(1)
C(18)	16(1)	22(1)	14(1)	2(1)	3(1)	0(1)
F(18)	18(1)	65(1)	44(1)	-2(1)	14(1)	1(1)
C(20)	13(1)	15(1)	17(1)	3(1)	5(1)	1(1)
F(20)	24(1)	45(1)	44(1)	7(1)	15(1)	9(1)
C(30)	20(1)	19(1)	18(1)	-1(1)	5(1)	-2(1)
F(21)	29(1)	47(1)	31(1)	1(1)	-5(1)	18(1)
C(24)	15(1)	14(1)	15(1)	2(1)	4(1)	0(1)
F(24)	25(1)	41(1)	101(1)	17(1)	16(1)	-9(1)
C(25)	18(1)	15(1)	18(1)	3(1)	7(1)	3(1)
F(25)	59(1)	17(1)	180(2)	-16(1)	63(1)	-13(1)
C(26)	15(1)	20(1)	18(1)	6(1)	5(1)	3(1)
C(27)	14(1)	18(1)	15(1)	3(1)	3(1)	-2(1)
C(28)	16(1)	14(1)	16(1)	2(1)	6(1)	0(1)
C(29)	20(1)	22(1)	30(1)	-1(1)	7(1)	6(1)
C(21)	17(1)	31(1)	23(1)	-2(1)	1(1)	1(1)
C(31)	14(1)	12(1)	15(1)	2(1)	5(1)	3(1)
C(32)	16(1)	14(1)	16(1)	1(1)	4(1)	1(1)
C(33)	20(1)	16(1)	15(1)	4(1)	5(1)	4(1)
C(34)	20(1)	19(1)	20(1)	7(1)	8(1)	2(1)
C(35)	14(1)	18(1)	19(1)	5(1)	5(1)	0(1)
C(36)	15(1)	16(1)	15(1)	4(1)	5(1)	2(1)
C(37)	26(1)	20(1)	16(1)	5(1)	5(1)	2(1)
C(38)	19(1)	29(1)	23(1)	8(1)	6(1)	-4(1)
C(39)	16(1)	13(1)	12(1)	1(1)	3(1)	1(1)
C(40)	18(1)	15(1)	22(1)	3(1)	8(1)	1(1)
C(41)	23(1)	14(1)	28(1)	4(1)	8(1)	-1(1)
C(42)	25(1)	13(1)	23(1)	2(1)	4(1)	2(1)
C(43)	19(1)	18(1)	14(1)	-1(1)	2(1)	5(1)
C(44)	16(1)	15(1)	13(1)	0(1)	3(1)	0(1)
C(45)	33(1)	18(1)	64(1)	8(1)	23(1)	-1(1)
C(46)	22(1)	19(1)	26(1)	-4(1)	3(1)	6(1)
C(23)	14(1)	13(1)	14(1)	4(1)	5(1)	0(1)
F(23)	74(1)	77(1)	79(1)	36(1)	38(1)	-20(1)
C(22)	21(1)	26(1)	20(1)	9(1)	6(1)	4(1)
F(22)	34(1)	22(1)	72(1)	-16(1)	18(1)	7(1)
F(2)	20(4)	45(4)	36(5)	-5(3)	-13(3)	16(4)
F(3)	22(3)	16(4)	24(4)	-3(3)	0(2)	2(3)
F(4)	24(4)	44(5)	45(3)	-10(4)	11(3)	-18(3)
F(2A)	36(3)	51(5)	17(2)	-10(3)	4(2)	0(3)
F(3A)	22(3)	45(4)	35(5)	0(3)	-11(2)	7(3)
F(4A)	28(4)	24(2)	39(3)	-7(2)	5(2)	-6(2)
F(2B)	25(6)	30(4)	20(4)	1(3)	-8(4)	6(4)
F(3B)	18(4)	49(7)	43(5)	-6(5)	6(4)	-13(4)
F(4B)	32(5)	23(4)	34(6)	-8(4)	-3(4)	-2(4)
F(2C)	36(5)	55(6)	16(3)	-8(4)	3(3)	-2(4)
F(3C)	12(3)	31(5)	33(6)	5(4)	-5(3)	1(3)
F(4C)	25(5)	25(3)	40(6)	-18(3)	4(3)	3(3)

Table S66. Hydrogen coordinates (x 10^4) and isotropic displacement parameters ($Å^2$ x 10^3) for **7b-B$_{ArF}$**.

	x	y	z	U(eq)
H(5A)	9968	8363	7968	34
H(5AB)	8675	8190	7534	34
H(6A)	8783	6534	7170	31
H(6AB)	9503	7117	6642	31
H(7A)	11362	7708	7814	29
H(7AB)	11370	6859	6976	29
H(8A)	12462	6026	8228	29
H(8AB)	13054	7007	8096	29
H(9A)	13472	7005	9745	28
H(9AB)	12591	7765	9499	28
H(10A)	12488	7712	11013	28
H(10B)	13063	6768	11338	28
H(11A)	11429	6059	11468	27
H(11B)	11736	7077	12154	27
H(12A)	11136	8465	11164	32
H(12B)	10460	8245	11904	32
H(13A)	8825	8236	10577	38
H(13B)	9504	9262	10914	38
H(14A)	8943	9095	9242	35
H(14B)	10210	8959	9538	35
H(16)	5458	9475	7908	17
H(18)	6492	8730	10672	21
H(20)	5041	6706	8339	17
H(24)	3518	9239	7429	18
H(26)	457	8865	5548	21
H(28)	2669	7011	5233	18
H(32)	4033	7896	4734	18
H(34)	6620	9429	4403	22
H(36)	6628	8775	7101	18
H(40)	3087	6126	6550	21
H(42)	4874	3880	6313	26
H(44)	6134	6602	6405	18

Table S67. Torsion angles [°] for **7b-B$_{Ar}$F**.

C(1)-N(1)-C(5)-C(6)	-78.01(18)
C(14)-N(1)-C(5)-C(6)	163.11(15)
Co(1)-N(1)-C(5)-C(6)	38.09(17)
C(2)-N(2)-C(6)-C(5)	162.89(14)
C(7)-N(2)-C(6)-C(5)	-78.88(17)
Co(1)-N(2)-C(6)-C(5)	43.05(15)
N(1)-C(5)-C(6)-N(2)	-57.8(2)
C(2)-N(2)-C(7)-C(8)	-74.28(19)
C(6)-N(2)-C(7)-C(8)	168.93(15)
Co(1)-N(2)-C(7)-C(8)	54.47(18)
N(2)-C(7)-C(8)-C(9)	-68.4(2)
C(3)-N(3)-C(9)-C(8)	63.07(19)
C(10)-N(3)-C(9)-C(8)	-178.86(15)
Co(1)-N(3)-C(9)-C(8)	-60.38(17)
C(7)-C(8)-C(9)-N(3)	71.8(2)
C(3)-N(3)-C(10)-C(11)	-79.36(17)
C(9)-N(3)-C(10)-C(11)	161.42(14)
Co(1)-N(3)-C(10)-C(11)	38.46(16)
C(4)-N(4)-C(11)-C(10)	161.23(14)
C(12)-N(4)-C(11)-C(10)	-79.78(17)
Co(1)-N(4)-C(11)-C(10)	43.56(15)
N(3)-C(10)-C(11)-N(4)	-57.78(18)
C(4)-N(4)-C(12)-C(13)	-72.11(19)
C(11)-N(4)-C(12)-C(13)	170.63(15)
Co(1)-N(4)-C(12)-C(13)	54.56(18)
N(4)-C(12)-C(13)-C(14)	-68.9(2)
C(5)-N(1)-C(14)-C(13)	-177.49(16)
C(1)-N(1)-C(14)-C(13)	64.4(2)
Co(1)-N(1)-C(14)-C(13)	-57.48(19)
C(12)-C(13)-C(14)-N(1)	70.3(2)
C(39)-B(1)-C(15)-C(16)	162.23(13)
C(23)-B(1)-C(15)-C(16)	-75.75(17)
C(31)-B(1)-C(15)-C(16)	43.72(18)
C(39)-B(1)-C(15)-C(20)	-18.67(18)
C(23)-B(1)-C(15)-C(20)	103.34(15)
C(31)-B(1)-C(15)-C(20)	-137.19(14)
C(20)-C(15)-C(16)-C(17)	0.9(2)
B(1)-C(15)-C(16)-C(17)	-179.92(14)
C(15)-C(16)-C(17)-C(18)	-1.4(2)
C(15)-C(16)-C(17)-C(30)	176.38(14)
C(20)-C(19)-C(18)-C(17)	0.8(2)
C(22)-C(19)-C(18)-C(17)	179.42(14)
C(16)-C(17)-C(18)-C(19)	0.5(2)
C(30)-C(17)-C(18)-C(19)	-177.31(14)
C(18)-C(19)-C(20)-C(15)	-1.3(2)
C(22)-C(19)-C(20)-C(15)	-179.85(14)
C(16)-C(15)-C(20)-C(19)	0.4(2)
B(1)-C(15)-C(20)-C(19)	-178.76(14)
C(18)-C(17)-C(30)-F(6)	-150.14(15)
C(16)-C(17)-C(30)-F(6)	32.0(2)
C(18)-C(17)-C(30)-F(5)	-29.3(2)
C(16)-C(17)-C(30)-F(5)	152.91(15)
C(18)-C(17)-C(30)-F(7)	90.13(18)
C(16)-C(17)-C(30)-F(7)	-87.69(18)
C(23)-C(24)-C(25)-C(26)	1.7(2)
C(23)-C(24)-C(25)-C(29)	179.21(15)
C(24)-C(25)-C(26)-C(27)	-1.1(2)
C(29)-C(25)-C(26)-C(27)	-178.67(15)
C(25)-C(26)-C(27)-C(28)	-0.5(2)
C(25)-C(26)-C(27)-C(21)	178.16(15)
C(26)-C(27)-C(28)-C(23)	1.6(2)
C(21)-C(27)-C(28)-C(23)	-177.06(15)
C(26)-C(25)-C(29)-F(10)	-28.7(2)
C(24)-C(25)-C(29)-F(10)	153.64(16)
C(26)-C(25)-C(29)-F(8)	-151.21(15)
C(24)-C(25)-C(29)-F(8)	31.2(2)
C(26)-C(25)-C(29)-F(9)	90.48(19)
C(24)-C(25)-C(29)-F(9)	-87.1(2)
C(39)-B(1)-C(31)-C(32)	73.35(16)
C(23)-B(1)-C(31)-C(32)	-48.07(17)
C(15)-B(1)-C(31)-C(32)	-169.57(13)
C(39)-B(1)-C(31)-C(36)	-104.68(16)
C(23)-B(1)-C(31)-C(36)	133.90(14)
C(15)-B(1)-C(31)-C(36)	12.4(2)
C(36)-C(31)-C(32)-C(33)	-0.5(2)
B(1)-C(31)-C(32)-C(33)	-178.69(14)
C(31)-C(32)-C(33)-C(34)	-1.6(2)
C(31)-C(32)-C(33)-C(37)	177.05(14)
C(32)-C(33)-C(34)-C(35)	1.7(2)
C(37)-C(33)-C(34)-C(35)	-176.96(15)
C(33)-C(34)-C(35)-C(36)	0.3(2)
C(33)-C(34)-C(35)-C(38)	179.45(15)
C(34)-C(35)-C(36)-C(31)	-2.5(2)
C(38)-C(35)-C(36)-C(31)	178.39(15)
C(32)-C(31)-C(36)-C(35)	2.5(2)
B(1)-C(31)-C(36)-C(35)	-179.41(14)
C(34)-C(33)-C(37)-F(15)	110.76(18)
C(32)-C(33)-C(37)-F(15)	-67.9(2)
C(34)-C(33)-C(37)-F(16)	-9.6(2)
C(32)-C(33)-C(37)-F(16)	171.73(16)
C(34)-C(33)-C(37)-F(14)	-129.61(18)
C(32)-C(33)-C(37)-F(14)	51.7(2)
C(34)-C(35)-C(38)-F(17)	44.9(2)

C(36)-C(35)-C(38)-F(17)	-135.92(17)
C(34)-C(35)-C(38)-F(19)	165.64(15)
C(36)-C(35)-C(38)-F(19)	-15.2(2)
C(34)-C(35)-C(38)-F(18)	-75.28(19)
C(36)-C(35)-C(38)-F(18)	103.90(18)
C(23)-B(1)-C(39)-C(44)	160.29(13)
C(31)-B(1)-C(39)-C(44)	41.98(17)
C(15)-B(1)-C(39)-C(44)	-78.65(16)
C(23)-B(1)-C(39)-C(40)	-28.10(19)
C(31)-B(1)-C(39)-C(40)	-146.42(14)
C(15)-B(1)-C(39)-C(40)	92.95(16)
C(44)-C(39)-C(40)-C(41)	-0.7(2)
B(1)-C(39)-C(40)-C(41)	-172.67(15)
C(39)-C(40)-C(41)-C(42)	1.8(3)
C(39)-C(40)-C(41)-C(45)	179.82(17)
C(40)-C(41)-C(42)-C(43)	-0.8(3)
C(45)-C(41)-C(42)-C(43)	-178.88(17)
C(41)-C(42)-C(43)-C(44)	-1.1(2)
C(41)-C(42)-C(43)-C(46)	174.80(15)
C(42)-C(43)-C(44)-C(39)	2.2(2)
C(46)-C(43)-C(44)-C(39)	-173.70(14)
C(40)-C(39)-C(44)-C(43)	-1.2(2)
B(1)-C(39)-C(44)-C(43)	171.03(13)
C(42)-C(41)-C(45)-F(25)	-22.9(3)
C(40)-C(41)-C(45)-F(25)	159.1(2)
C(42)-C(41)-C(45)-F(24)	-144.65(19)
C(40)-C(41)-C(45)-F(24)	37.3(3)
C(42)-C(41)-C(45)-F(23)	98.5(2)
C(40)-C(41)-C(45)-F(23)	-79.5(2)
C(42)-C(43)-C(46)-F(22)	22.2(2)
C(44)-C(43)-C(46)-F(22)	-161.89(15)
C(42)-C(43)-C(46)-F(20)	144.43(15)
C(44)-C(43)-C(46)-F(20)	-39.6(2)
C(42)-C(43)-C(46)-F(21)	-97.43(18)
C(44)-C(43)-C(46)-F(21)	78.5(2)
C(25)-C(24)-C(23)-C(28)	-0.6(2)
C(25)-C(24)-C(23)-B(1)	175.53(14)
C(27)-C(28)-C(23)-C(24)	-1.0(2)
C(27)-C(28)-C(23)-B(1)	-177.20(14)
C(39)-B(1)-C(23)-C(24)	150.57(14)
C(31)-B(1)-C(23)-C(24)	-90.23(16)
C(15)-B(1)-C(23)-C(24)	32.07(19)
C(39)-B(1)-C(23)-C(28)	-33.57(18)
C(31)-B(1)-C(23)-C(28)	85.63(16)
C(15)-B(1)-C(23)-C(28)	-152.07(13)
C(18)-C(19)-C(22)-F(12)	-69.1(2)
C(20)-C(19)-C(22)-F(12)	109.49(19)
C(18)-C(19)-C(22)-F(11)	169.68(15)
C(20)-C(19)-C(22)-F(11)	-11.7(2)
C(18)-C(19)-C(22)-F(13)	50.9(2)
C(20)-C(19)-C(22)-F(13)	-130.46(17)

[(TMCO)Co^{III}(H_2O)](OTf)$_2$, **8a-(OTf)₂**

The structure of **8a-(OTf)$_2$** was solved in the triclinic space group $P\bar{1}$ and the asymmetric unit contains one molecule with the anions and heavily disordered solvent. The main molecule was found to be disordered over three, the anions each over two positions. The disorder was refined with the help of same distance restraints, similarity restraints on anisotropic displacement parameters and advanced rigid bond restraints. Some atoms with very similar positions were set to the same anisotropic displacement parameters. The disorder ratios were allowed to refine freely and converged to 0.375(3):0.316(3):0.310(3), 0.638(4) and 0.49(1). The disordered solvent was modelled as bulk solvent model using the program SQUEEZE as implemented in PLATON and included into the refinement as .fab file. SQUEEZE identified one solvent accessible void at 0.0 0.5 0.5 with a volume of 245 Å^3 and an equivalent of 125 electrons.

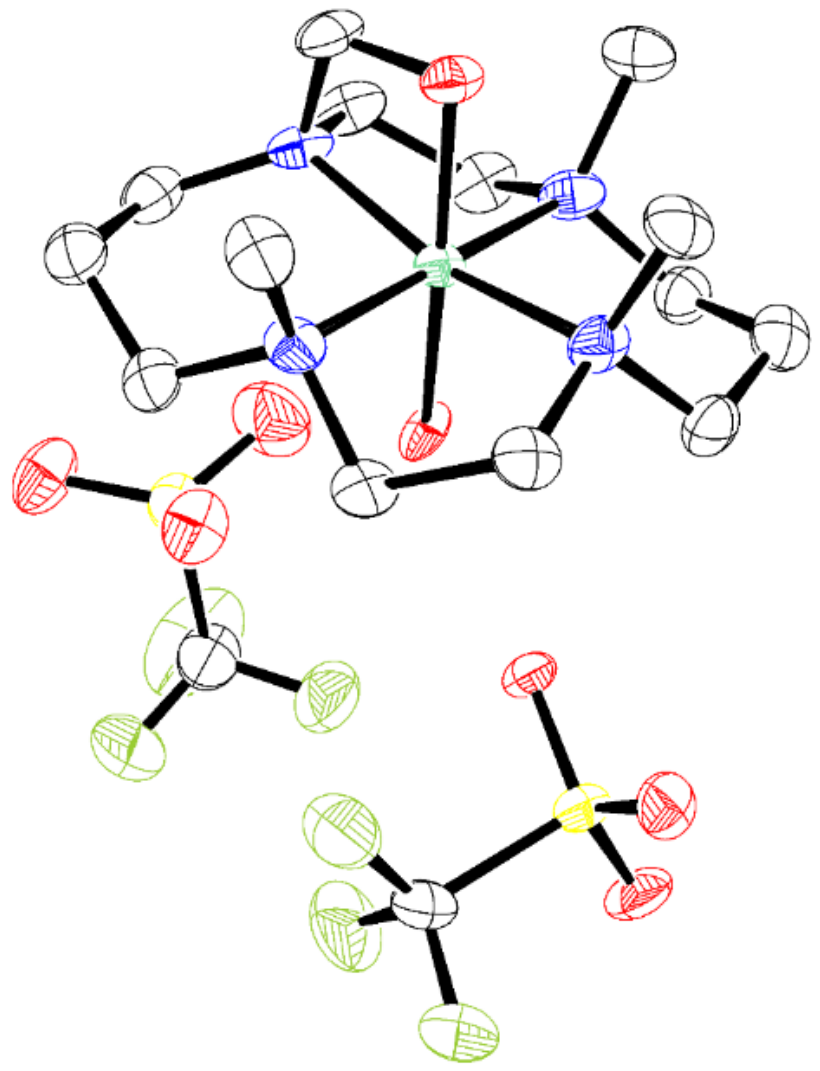

Figure S14. Molecular structure of **8a-(OTf)$_2$**.

Table S68. Crystal data and structure refinement for **8a-(OTf)$_2$**.

CCDC No	2062706	
Empirical formula	$C_{16}H_{33}CoF_6N_4O_8S_2$	
Formula weight	646.51	
Temperature	100(2) K	
Wavelength	0.71073 Å	
Crystal system	Triclinic	
Space group	$P\bar{1}$	
Unit cell dimensions	a = 11.2820(8) Å	α = 106.690(2)°
	b = 12.3403(9) Å	β= 105.913(2)°
	c = 12.7716(9) Å	γ = 106.239(2)°
Volume	1508.88(19) Å^3	
Z	2	
Density (calculated)	1.423 Mg/m^3	
Absorption coefficient	0.785 mm^{-1}	
F(000)	668	
Crystal size	0.170 x 0.074 x 0.068 mm^3	
Theta range for data collection	2.787 to 27.103°.	
Index ranges	-14 ≤ h ≤ 14, -15 ≤ k ≤ 15, -16 ≤ l ≤ 16	
Reflections collected	86108	
Independent reflections	6655 [R(int) = 0.0638]	
Completeness to theta = 25.242°	99.8 %	
Absorption correction	Semi-empirical from equivalents	
Refinement method	Full-matrix least-squares on F^2	
Data / restraints / parameters	6655 / 3463 / 858	
Goodness-of-fit on F^2	1.039	
Final R indices [I>2σ(I)]	R1 = 0.0453, wR2 = 0.1125	
R indices (all data)	R1 = 0.0606, wR2 = 0.1221	
Largest diff. peak and hole	0.608 and -0.588 eÅ^{-3}	

Table S69. Atomic coordinates (x 10^4) and equivalent isotropic displacement parameters ($Å^2$x 10^3) for **8a-(OTf)₂**. U(eq) is defined as one third of the trace of the orthogonalized U^{ij} tensor.

	x	y	z	U(eq)
Co(1)	8106(7)	844(6)	8116(6)	20(1)
O(1)	6980(14)	1700(13)	7509(9)	26(2)
O(2)	9226(17)	11(17)	8380(20)	29(3)
N(1)	9618(11)	1573(9)	7677(9)	28(2)
N(2)	7279(15)	-346(15)	6400(9)	30(2)
N(3)	6828(15)	-164(13)	8655(13)	30(2)
N(4)	8920(13)	2080(11)	9805(9)	28(2)
C(1)	9600(13)	3358(12)	9911(12)	31(2)
C(2)	10756(11)	3567(11)	9511(10)	37(2)
C(3)	10384(12)	2931(10)	8214(11)	35(2)
C(4)	10227(18)	822(19)	8210(20)	33(3)
C(5)	9262(11)	1159(13)	6386(9)	37(2)
C(6)	7755(14)	400(20)	5743(16)	37(3)
C(7)	7690(30)	-1420(20)	6140(20)	35(4)
C(8)	5779(17)	-830(30)	5870(20)	32(3)
C(9)	5130(20)	-1476(16)	6546(15)	36(3)
C(10)	5401(18)	-660(30)	7796(15)	36(3)
C(11)	7140(40)	-1210(30)	8870(30)	36(3)
C(12)	6911(18)	695(12)	9828(13)	32(3)
C(13)	7741(14)	2020(14)	10137(13)	28(2)
C(14)	9870(20)	1860(20)	10700(20)	36(4)
Co(1A)	8105(8)	761(6)	7952(6)	20(1)
O(1A)	6826(15)	1429(13)	7239(11)	26(2)
O(2A)	9231(19)	-20(20)	8420(20)	28(3)
N(1A)	9593(12)	1303(10)	7440(10)	28(2)
N(2A)	7275(12)	-706(10)	6337(10)	28(2)
N(3A)	6804(17)	-111(15)	8572(15)	29(2)
N(4A)	8869(14)	2137(10)	9535(9)	24(2)
C(1A)	9476(14)	3331(13)	9454(12)	28(2)
C(2A)	10633(13)	3448(14)	9051(12)	31(2)
C(3A)	10255(14)	2639(11)	7783(12)	28(2)
C(4A)	10204(18)	654(19)	8120(20)	30(3)
C(5A)	9184(11)	646(11)	6142(10)	28(2)
C(6A)	7671(12)	-117(11)	5530(11)	32(2)
C(7A)	7690(20)	-1787(16)	6180(20)	34(3)
C(8A)	5772(14)	-1232(13)	5807(15)	31(3)
C(9A)	5120(20)	-1729(19)	6555(19)	35(4)
C(10A)	5360(20)	-770(30)	7723(17)	34(4)
C(11A)	7260(40)	-1020(30)	8970(40)	36(3)
C(12A)	6870(20)	858(15)	9671(15)	30(3)
C(13A)	7663(17)	2126(15)	9814(16)	26(3)
C(14A)	9810(20)	2030(20)	10520(20)	28(3)
Co(1B)	7970(6)	570(5)	7941(6)	20(1)
O(1B)	7090(20)	1743(18)	7705(16)	26(2)
O(2B)	9459(16)	215(18)	8520(20)	26(3)
N(1B)	8561(13)	1822(12)	9637(10)	31(2)
N(2B)	9710(10)	1748(10)	8074(8)	33(2)
N(3B)	7252(19)	-398(18)	6195(10)	30(3)
N(4B)	6416(8)	-635(7)	8014(7)	28(2)
C(1B)	5634(8)	-99(8)	8618(8)	27(2)
C(2B)	6383(11)	837(10)	9857(10)	32(2)
C(3B)	7421(16)	1980(17)	9960(20)	29(3)
C(4B)	9435(15)	1529(15)	10579(19)	35(4)
C(5B)	9367(18)	3039(14)	9703(18)	38(3)
C(6B)	10400(10)	2889(11)	9169(11)	42(2)
C(7B)	10430(20)	910(20)	8230(20)	30(3)
C(8B)	9802(10)	2107(11)	7070(10)	39(2)
C(9B)	9068(12)	1063(14)	5873(10)	41(2)
C(10B)	7579(17)	410(20)	5539(19)	38(3)
C(11B)	7680(40)	-1440(30)	5860(20)	40(5)
C(12B)	5770(20)	-890(40)	5920(30)	36(4)
C(13B)	5507(14)	-1484(17)	6765(12)	36(3)
C(14B)	6920(30)	-1370(20)	8598(18)	34(4)
S(21)	3750(10)	1843(8)	7992(8)	30(1)
F(21)	3691(7)	3463(6)	9754(6)	57(2)
F(22)	5686(6)	3598(6)	9890(6)	62(2)
F(23)	4615(6)	4218(5)	8715(7)	65(2)
O(21)	2527(9)	1824(9)	7209(10)	40(2)
O(22)	3541(16)	1061(13)	8609(13)	37(2)
O(23)	4739(9)	1819(9)	7466(8)	26(2)
C(21)	4468(7)	3364(6)	9140(8)	38(2)
S(21A)	3617(10)	1689(8)	7764(8)	31(1)
F(21A)	5580(7)	3756(7)	9228(10)	99(3)
F(22A)	3663(8)	3669(6)	9231(9)	79(2)
F(23A)	4171(12)	3897(7)	7823(12)	114(3)
O(21A)	4472(9)	1495(9)	7118(8)	29(2)
O(22A)	2278(10)	1371(10)	7008(11)	54(3)
O(23A)	3841(17)	1273(16)	8711(15)	57(4)
C(21A)	4291(10)	3334(8)	8538(11)	63(2)
S(31)	7915(5)	4345(5)	6743(7)	34(1)
F(31)	5386(5)	3942(6)	6107(5)	63(2)
F(32)	6681(6)	5784(6)	7219(7)	90(2)
F(33)	6453(4)	5102(5)	5439(5)	105(2)
O(31)	7883(4)	4052(3)	7758(3)	43(1)
O(32)	7567(5)	3289(4)	5716(4)	66(1)
O(33)	9044(4)	5386(4)	7003(4)	49(1)
C(31)	6524(5)	4811(5)	6373(5)	50(1)
S(31A)	7921(11)	4340(10)	6627(12)	36(2)
F(31A)	7005(6)	4521(6)	8259(5)	57(2)
F(32A)	5562(9)	4223(11)	6619(9)	62(3)

F(33A)	7152(9)	5988(8)	7712(9)	64(3)
O(31A)	7738(9)	4696(8)	5629(7)	70(2)
O(32A)	7437(5)	3023(4)	6273(5)	24(1)
O(33A)	9247(6)	5004(7)	7515(7)	53(2)
C(31A)	6849(8)	4798(7)	7298(8)	43(2)

Table S70. Bond lengths [Å] and angles [°] for **8a-(OTf)$_2$**.

Co(1)-O(2)	1.860(11)	C(5A)-H(5AC)	0.9900
Co(1)-N(1)	1.998(9)	C(6A)-H(6AA)	0.9900
Co(1)-O(1)	2.003(9)	C(6A)-H(6AC)	0.9900
Co(1)-N(4)	2.015(9)	C(7A)-H(7AA)	0.9800
Co(1)-N(2)	2.024(10)	C(7A)-H(7AB)	0.9800
Co(1)-N(3)	2.033(9)	C(7A)-H(7AC)	0.9800
Co(1)-C(4)	2.371(18)	C(8A)-C(9A)	1.515(15)
O(1)-H(1A)	0.83(2)	C(8A)-H(8AA)	0.9900
O(1)-H(1B)	0.84(2)	C(8A)-H(8AC)	0.9900
O(2)-C(4)	1.395(12)	C(9A)-C(10A)	1.510(14)
N(1)-C(5)	1.476(11)	C(9A)-H(9AA)	0.9900
N(1)-C(3)	1.493(11)	C(9A)-H(9AC)	0.9900
N(1)-C(4)	1.504(14)	C(10A)-H(10C)	0.9900
N(2)-C(6)	1.499(13)	C(10A)-H(10D)	0.9900
N(2)-C(8)	1.501(13)	C(11A)-H(11D)	0.9800
N(2)-C(7)	1.504(15)	C(11A)-H(11E)	0.9800
N(3)-C(11)	1.501(14)	C(11A)-H(11F)	0.9800
N(3)-C(10)	1.504(11)	C(12A)-C(13A)	1.499(14)
N(3)-C(12)	1.527(11)	C(12A)-H(12C)	0.9900
N(4)-C(14)	1.483(13)	C(12A)-H(12D)	0.9900
N(4)-C(13)	1.492(12)	C(13A)-H(13C)	0.9900
N(4)-C(1)	1.495(12)	C(13A)-H(13D)	0.9900
C(1)-C(2)	1.510(13)	C(14A)-H(14D)	0.9800
C(1)-H(1AA)	0.9900	C(14A)-H(14E)	0.9800
C(1)-H(1AB)	0.9900	C(14A)-H(14F)	0.9800
C(2)-C(3)	1.491(13)	Co(1B)-O(2B)	1.865(12)
C(2)-H(2A)	0.9900	Co(1B)-N(3B)	1.999(11)
C(2)-H(2AB)	0.9900	Co(1B)-N(4B)	2.001(8)
C(3)-H(3A)	0.9900	Co(1B)-O(1B)	2.014(12)
C(3)-H(3AB)	0.9900	Co(1B)-N(2B)	2.019(9)
C(4)-H(4A)	0.9900	Co(1B)-N(1B)	2.058(10)
C(4)-H(4AB)	0.9900	O(1B)-H(1E)	0.84(2)
C(5)-C(6)	1.532(13)	O(1B)-H(1F)	0.83(2)
C(5)-H(5A)	0.9900	O(2B)-C(7B)	1.400(13)
C(5)-H(5AB)	0.9900	N(1B)-C(5B)	1.486(13)
C(6)-H(6A)	0.9900	N(1B)-C(3B)	1.497(15)
C(6)-H(6AB)	0.9900	N(1B)-C(4B)	1.533(16)
C(7)-H(7A)	0.9800	N(2B)-C(6B)	1.484(13)
C(7)-H(7B)	0.9800	N(2B)-C(8B)	1.491(11)
C(7)-H(7C)	0.9800	N(2B)-C(7B)	1.503(18)
C(8)-C(9)	1.525(13)	N(3B)-C(11B)	1.489(17)
C(8)-H(8A)	0.9900	N(3B)-C(10B)	1.506(15)
C(8)-H(8AB)	0.9900	N(3B)-C(12B)	1.513(17)
C(9)-C(10)	1.514(13)	N(4B)-C(14B)	1.467(16)
C(9)-H(9A)	0.9900	N(4B)-C(1B)	1.490(10)
C(9)-H(9AB)	0.9900	N(4B)-C(13B)	1.495(13)
C(10)-H(10A)	0.9900	C(1B)-C(2B)	1.491(12)
C(10)-H(10B)	0.9900	C(1B)-H(1BA)	0.9900
C(11)-H(11A)	0.9800	C(1B)-H(1BB)	0.9900
C(11)-H(11B)	0.9800	C(2B)-C(3B)	1.506(16)
C(11)-H(11C)	0.9800	C(2B)-H(2BA)	0.9900
C(12)-C(13)	1.509(14)	C(2B)-H(2BB)	0.9900
C(12)-H(12A)	0.9900	C(3B)-H(3BA)	0.9900
C(12)-H(12B)	0.9900	C(3B)-H(3BB)	0.9900
C(13)-H(13A)	0.9900	C(4B)-H(4BA)	0.9800
C(13)-H(13B)	0.9900	C(4B)-H(4BB)	0.9800
C(14)-H(14A)	0.9800	C(4B)-H(4BC)	0.9800
C(14)-H(14B)	0.9800	C(5B)-C(6B)	1.531(15)
C(14)-H(14C)	0.9800	C(5B)-H(5BA)	0.9900
Co(1A)-O(2A)	1.867(12)	C(5B)-H(5BB)	0.9900
Co(1A)-N(1A)	1.980(10)	C(6B)-H(6BA)	0.9900
Co(1A)-N(4A)	1.992(10)	C(6B)-H(6BB)	0.9900
Co(1A)-O(1A)	1.993(11)	C(7B)-H(7BA)	0.9900
Co(1A)-N(3A)	2.045(11)	C(7B)-H(7BB)	0.9900
Co(1A)-N(2A)	2.064(10)	C(8B)-C(9B)	1.503(15)
Co(1A)-C(4A)	2.363(19)	C(8B)-H(8BA)	0.9900
O(1A)-H(1C)	0.84(2)	C(8B)-H(8BB)	0.9900
O(1A)-H(1D)	0.85(2)	C(9B)-C(10B)	1.519(16)
O(2A)-C(4A)	1.393(13)	C(9B)-H(9BA)	0.9900
N(1A)-C(3A)	1.477(12)	C(9B)-H(9BB)	0.9900
N(1A)-C(5A)	1.489(12)	C(10B)-H(10E)	0.9900
N(1A)-C(4A)	1.504(14)	C(10B)-H(10F)	0.9900
N(2A)-C(8A)	1.502(13)	C(11B)-H(11G)	0.9800
N(2A)-C(7A)	1.510(13)	C(11B)-H(11H)	0.9800
N(2A)-C(6A)	1.514(12)	C(11B)-H(11I)	0.9800
N(3A)-C(10A)	1.507(13)	C(12B)-C(13B)	1.510(18)
N(3A)-C(11A)	1.509(15)	C(12B)-H(12E)	0.9900
N(3A)-C(12A)	1.529(13)	C(12B)-H(12F)	0.9900
N(4A)-C(14A)	1.474(14)	C(13B)-H(13E)	0.9900
N(4A)-C(1A)	1.488(12)	C(13B)-H(13F)	0.9900
N(4A)-C(13A)	1.495(12)	C(14B)-H(14G)	0.9800
C(1A)-C(2A)	1.514(12)	C(14B)-H(14H)	0.9800
C(1A)-H(1AC)	0.9900	C(14B)-H(14I)	0.9800
C(1A)-H(1AD)	0.9900	S(21)-O(22)	1.421(11)
C(2A)-C(3A)	1.503(14)	S(21)-O(23)	1.454(11)
C(2A)-H(2AA)	0.9900	S(21)-O(21)	1.455(11)
C(2A)-H(2AC)	0.9900	S(21)-C(21)	1.803(8)
C(3A)-H(3AA)	0.9900	F(21)-C(21)	1.334(8)
C(3A)-H(3AC)	0.9900	F(22)-C(21)	1.337(8)
C(4A)-H(4AA)	0.9900	F(23)-C(21)	1.308(8)
C(4A)-H(4AC)	0.9900	S(21A)-O(22A)	1.421(11)
C(5A)-C(6A)	1.536(13)	S(21A)-O(23A)	1.433(12)
C(5A)-H(5AA)	0.9900	S(21A)-O(21A)	1.456(11)

S(21A)-C(21A)	1.802(10)
F(21A)-C(21A)	1.332(10)
F(22A)-C(21A)	1.323(9)
F(23A)-C(21A)	1.298(11)
S(31)-O(33)	1.410(7)
S(31)-O(32)	1.423(7)
S(31)-O(31)	1.449(9)
S(31)-C(31)	1.808(7)
F(31)-C(31)	1.302(7)
F(32)-C(31)	1.293(8)
F(33)-C(31)	1.330(7)
S(31A)-O(33A)	1.431(11)
S(31A)-O(31A)	1.446(15)
S(31A)-O(32A)	1.446(12)
S(31A)-C(31A)	1.785(11)
F(31A)-C(31A)	1.345(10)
F(32A)-C(31A)	1.320(11)
F(33A)-C(31A)	1.314(11)
O(2)-Co(1)-N(1)	74.4(7)
O(2)-Co(1)-O(1)	167.4(9)
N(1)-Co(1)-O(1)	96.1(7)
O(2)-Co(1)-N(4)	94.6(8)
N(1)-Co(1)-N(4)	95.8(5)
O(1)-Co(1)-N(4)	94.6(6)
O(2)-Co(1)-N(2)	87.2(9)
N(1)-Co(1)-N(2)	83.4(5)
O(1)-Co(1)-N(2)	83.4(7)
N(4)-Co(1)-N(2)	177.8(8)
O(2)-Co(1)-N(3)	91.0(7)
N(1)-Co(1)-N(3)	165.4(8)
O(1)-Co(1)-N(3)	98.3(8)
N(4)-Co(1)-N(3)	85.2(5)
N(2)-Co(1)-N(3)	96.1(6)
O(2)-Co(1)-C(4)	36.0(4)
N(1)-Co(1)-C(4)	39.1(4)
O(1)-Co(1)-C(4)	135.2(7)
N(4)-Co(1)-C(4)	90.9(7)
N(2)-Co(1)-C(4)	89.8(7)
N(3)-Co(1)-C(4)	126.5(7)
Co(1)-O(1)-H(1A)	126(3)
Co(1)-O(1)-H(1B)	124(3)
H(1A)-O(1)-H(1B)	106(3)
C(4)-O(2)-Co(1)	92.4(10)
C(5)-N(1)-C(3)	107.5(9)
C(5)-N(1)-C(4)	113.5(12)
C(3)-N(1)-C(4)	117.2(11)
C(5)-N(1)-Co(1)	113.5(7)
C(3)-N(1)-Co(1)	119.9(8)
C(4)-N(1)-Co(1)	84.0(8)
C(6)-N(2)-C(8)	106.0(11)
C(6)-N(2)-C(7)	107.7(13)
C(8)-N(2)-C(7)	108.2(14)
C(6)-N(2)-Co(1)	105.0(10)
C(8)-N(2)-Co(1)	113.9(14)
C(7)-N(2)-Co(1)	115.4(13)
C(11)-N(3)-C(10)	108.6(14)
C(11)-N(3)-C(12)	107.1(14)
C(10)-N(3)-C(12)	106.9(11)
C(11)-N(3)-Co(1)	115(2)
C(10)-N(3)-Co(1)	111.5(13)
C(12)-N(3)-Co(1)	107.6(8)
C(14)-N(4)-C(13)	107.2(12)
C(14)-N(4)-C(1)	106.9(11)
C(13)-N(4)-C(1)	108.5(10)
C(14)-N(4)-Co(1)	117.9(13)
C(13)-N(4)-Co(1)	103.6(8)
C(1)-N(4)-Co(1)	112.4(8)
N(4)-C(1)-C(2)	116.0(10)
N(4)-C(1)-H(1AA)	108.3
C(2)-C(1)-H(1AA)	108.3
N(4)-C(1)-H(1AB)	108.3
C(2)-C(1)-H(1AB)	108.3
H(1AA)-C(1)-H(1AB)	107.4
C(3)-C(2)-C(1)	115.1(9)
C(3)-C(2)-H(2A)	108.5
C(1)-C(2)-H(2A)	108.5
C(3)-C(2)-H(2AB)	108.5
C(1)-C(2)-H(2AB)	108.5
H(2A)-C(2)-H(2AB)	107.5
C(2)-C(3)-N(1)	116.9(9)
C(2)-C(3)-H(3A)	108.1
N(1)-C(3)-H(3A)	108.1
C(2)-C(3)-H(3AB)	108.1
N(1)-C(3)-H(3AB)	108.1
H(3A)-C(3)-H(3AB)	107.3
O(2)-C(4)-N(1)	107.2(11)
O(2)-C(4)-Co(1)	51.6(7)
N(1)-C(4)-Co(1)	56.9(6)
O(2)-C(4)-H(4A)	110.3
N(1)-C(4)-H(4A)	110.3
Co(1)-C(4)-H(4A)	135.7
O(2)-C(4)-H(4AB)	110.3
N(1)-C(4)-H(4AB)	110.3
Co(1)-C(4)-H(4AB)	115.7
H(4A)-C(4)-H(4AB)	108.5
N(1)-C(5)-C(6)	109.2(10)
N(1)-C(5)-H(5A)	109.8
C(6)-C(5)-H(5A)	109.8
N(1)-C(5)-H(5AB)	109.8
C(6)-C(5)-H(5AB)	109.8
H(5A)-C(5)-H(5AB)	108.3
N(2)-C(6)-C(5)	110.1(10)
N(2)-C(6)-H(6A)	109.6
C(5)-C(6)-H(6A)	109.6
N(2)-C(6)-H(6AB)	109.6
C(5)-C(6)-H(6AB)	109.6
H(6A)-C(6)-H(6AB)	108.2
N(2)-C(7)-H(7A)	109.5
N(2)-C(7)-H(7B)	109.5
H(7A)-C(7)-H(7B)	109.5
N(2)-C(7)-H(7C)	109.5
H(7A)-C(7)-H(7C)	109.5
H(7B)-C(7)-H(7C)	109.5
N(2)-C(8)-C(9)	112.7(14)
N(2)-C(8)-H(8A)	109.0
C(9)-C(8)-H(8A)	109.0
N(2)-C(8)-H(8AB)	109.0
C(9)-C(8)-H(8AB)	109.0
H(8A)-C(8)-H(8AB)	107.8
C(10)-C(9)-C(8)	115.4(16)
C(10)-C(9)-H(9A)	108.4
C(8)-C(9)-H(9A)	108.4
C(10)-C(9)-H(9AB)	108.4
C(8)-C(9)-H(9AB)	108.4
H(9A)-C(9)-H(9AB)	107.5
N(3)-C(10)-C(9)	115.0(13)
N(3)-C(10)-H(10A)	108.5
C(9)-C(10)-H(10A)	108.5
N(3)-C(10)-H(10B)	108.5
C(9)-C(10)-H(10B)	108.5
H(10A)-C(10)-H(10B)	107.5
N(3)-C(11)-H(11A)	109.5
N(3)-C(11)-H(11B)	109.5
H(11A)-C(11)-H(11B)	109.5
N(3)-C(11)-H(11C)	109.5
H(11A)-C(11)-H(11C)	109.5
H(11B)-C(11)-H(11C)	109.5
C(13)-C(12)-N(3)	112.3(11)
C(13)-C(12)-H(12A)	109.2
N(3)-C(12)-H(12A)	109.2
C(13)-C(12)-H(12B)	109.2
N(3)-C(12)-H(12B)	109.2
H(12A)-C(12)-H(12B)	107.9
N(4)-C(13)-C(12)	108.9(11)
N(4)-C(13)-H(13A)	109.9
C(12)-C(13)-H(13A)	109.9
N(4)-C(13)-H(13B)	109.9
C(12)-C(13)-H(13B)	109.9
H(13A)-C(13)-H(13B)	108.3
N(4)-C(14)-H(14A)	109.5
N(4)-C(14)-H(14B)	109.5
H(14A)-C(14)-H(14B)	109.5
N(4)-C(14)-H(14C)	109.5
H(14A)-C(14)-H(14C)	109.5
H(14B)-C(14)-H(14C)	109.5
O(2A)-Co(1A)-N(1A)	74.9(7)
O(2A)-Co(1A)-N(4A)	94.2(9)
N(1A)-Co(1A)-N(4A)	97.9(5)
O(2A)-Co(1A)-O(1A)	172.6(9)
N(1A)-Co(1A)-O(1A)	103.2(8)
N(4A)-Co(1A)-O(1A)	93.2(6)
O(2A)-Co(1A)-N(3A)	90.0(8)
N(1A)-Co(1A)-N(3A)	164.7(8)
N(4A)-Co(1A)-N(3A)	85.4(6)
O(1A)-Co(1A)-N(3A)	91.5(9)
O(2A)-Co(1A)-N(2A)	85.3(9)
N(1A)-Co(1A)-N(2A)	84.0(5)
N(4A)-Co(1A)-N(2A)	177.9(6)
O(1A)-Co(1A)-N(2A)	87.4(6)
N(3A)-Co(1A)-N(2A)	92.5(6)
O(2A)-Co(1A)-C(4A)	36.1(5)
N(1A)-Co(1A)-C(4A)	39.3(4)
N(4A)-Co(1A)-C(4A)	92.8(7)
O(1A)-Co(1A)-C(4A)	142.5(8)
N(3A)-Co(1A)-C(4A)	125.9(8)
N(2A)-Co(1A)-C(4A)	88.0(7)
Co(1A)-O(1A)-H(1C)	123(3)
Co(1A)-O(1A)-H(1D)	122(4)
H(1C)-O(1A)-H(1D)	102(3)
C(4A)-O(2A)-Co(1A)	91.7(10)
C(3A)-N(1A)-C(5A)	109.7(9)
C(3A)-N(1A)-C(4A)	121.1(11)
C(5A)-N(1A)-C(4A)	111.4(11)
C(3A)-N(1A)-Co(1A)	116.7(8)
C(5A)-N(1A)-Co(1A)	111.5(7)
C(4A)-N(1A)-Co(1A)	84.2(8)
C(8A)-N(2A)-C(7A)	106.4(11)
C(8A)-N(2A)-C(6A)	104.1(10)
C(7A)-N(2A)-C(6A)	107.4(10)

C(8A)-N(2A)-Co(1A)	114.0(9)	C(12A)-C(13A)-H(13D)	109.7
C(7A)-N(2A)-Co(1A)	120.7(12)	H(13C)-C(13A)-H(13D)	108.2
C(6A)-N(2A)-Co(1A)	102.8(7)	N(4A)-C(14A)-H(14D)	109.5
C(10A)-N(3A)-C(11A)	108.2(15)	N(4A)-C(14A)-H(14E)	109.5
C(10A)-N(3A)-C(12A)	107.8(12)	H(14D)-C(14A)-H(14E)	109.5
C(11A)-N(3A)-C(12A)	107.0(15)	N(4A)-C(14A)-H(14F)	109.5
C(10A)-N(3A)-Co(1A)	116.0(15)	H(14D)-C(14A)-H(14F)	109.5
C(11A)-N(3A)-Co(1A)	110(2)	H(14E)-C(14A)-H(14F)	109.5
C(12A)-N(3A)-Co(1A)	107.5(9)	O(2B)-Co(1B)-N(3B)	103.4(12)
C(14A)-N(4A)-C(1A)	110.2(11)	O(2B)-Co(1B)-N(4B)	107.1(8)
C(14A)-N(4A)-C(13A)	107.4(11)	N(3B)-Co(1B)-N(4B)	88.6(6)
C(1A)-N(4A)-C(13A)	107.9(10)	O(2B)-Co(1B)-O(1B)	152.4(10)
C(14A)-N(4A)-Co(1A)	117.0(13)	N(3B)-Co(1B)-O(1B)	91.5(10)
C(1A)-N(4A)-Co(1A)	110.6(8)	N(4B)-Co(1B)-O(1B)	96.2(9)
C(13A)-N(4A)-Co(1A)	103.1(9)	O(2B)-Co(1B)-N(2B)	65.2(8)
N(4A)-C(1A)-C(2A)	115.0(10)	N(3B)-Co(1B)-N(2B)	97.0(6)
N(4A)-C(1A)-H(1AC)	108.5	N(4B)-Co(1B)-N(2B)	171.3(6)
C(2A)-C(1A)-H(1AC)	108.5	O(1B)-Co(1B)-N(2B)	90.3(9)
N(4A)-C(1A)-H(1AD)	108.5	O(2B)-Co(1B)-N(1B)	86.8(10)
C(2A)-C(1A)-H(1AD)	108.5	N(3B)-Co(1B)-N(1B)	168.0(10)
H(1AC)-C(1A)-H(1AD)	107.5	N(4B)-Co(1B)-N(1B)	94.5(5)
C(3A)-C(2A)-C(1A)	114.8(10)	O(1B)-Co(1B)-N(1B)	76.6(7)
C(3A)-C(2A)-H(2AA)	108.6	N(2B)-Co(1B)-N(1B)	81.3(5)
C(1A)-C(2A)-H(2AA)	108.6	Co(1B)-O(1B)-H(1E)	123(4)
C(3A)-C(2A)-H(2AC)	108.6	Co(1B)-O(1B)-H(1F)	127(4)
C(1A)-C(2A)-H(2AC)	108.6	H(1E)-O(1B)-H(1F)	106(3)
H(2AA)-C(2A)-H(2AC)	107.5	C(7B)-O(2B)-Co(1B)	104.1(14)
N(1A)-C(3A)-C(2A)	116.6(9)	C(5B)-N(1B)-C(3B)	105.8(11)
N(1A)-C(3A)-H(3AA)	108.2	C(5B)-N(1B)-C(4B)	107.9(11)
C(2A)-C(3A)-H(3AA)	108.2	C(3B)-N(1B)-C(4B)	108.9(12)
N(1A)-C(3A)-H(3AC)	108.2	C(5B)-N(1B)-Co(1B)	107.3(9)
C(2A)-C(3A)-H(3AC)	108.2	C(3B)-N(1B)-Co(1B)	113.6(10)
H(3AA)-C(3A)-H(3AC)	107.3	C(4B)-N(1B)-Co(1B)	113.0(12)
O(2A)-C(4A)-N(1A)	107.7(12)	C(6B)-N(2B)-C(8B)	107.1(9)
O(2A)-C(4A)-Co(1A)	52.2(7)	C(6B)-N(2B)-C(7B)	107.1(12)
N(1A)-C(4A)-Co(1A)	56.5(6)	C(8B)-N(2B)-C(7B)	111.6(13)
O(2A)-C(4A)-H(4AA)	110.2	C(6B)-N(2B)-Co(1B)	115.3(7)
N(1A)-C(4A)-H(4AA)	110.2	C(8B)-N(2B)-Co(1B)	120.8(7)
Co(1A)-C(4A)-H(4AA)	134.3	C(7B)-N(2B)-Co(1B)	93.5(11)
O(2A)-C(4A)-H(4AC)	110.2	C(11B)-N(3B)-C(10B)	110.4(15)
N(1A)-C(4A)-H(4AC)	110.2	C(11B)-N(3B)-C(12B)	109.5(18)
Co(1A)-C(4A)-H(4AC)	117.2	C(10B)-N(3B)-C(12B)	110.6(15)
H(4AA)-C(4A)-H(4AC)	108.5	C(11B)-N(3B)-Co(1B)	113.8(14)
N(1A)-C(5A)-C(6A)	111.4(9)	C(10B)-N(3B)-Co(1B)	111.8(13)
N(1A)-C(5A)-H(5AA)	109.3	C(12B)-N(3B)-Co(1B)	100.4(15)
C(6A)-C(5A)-H(5AA)	109.3	C(14B)-N(4B)-C(1B)	109.6(12)
N(1A)-C(5A)-H(5AC)	109.3	C(14B)-N(4B)-C(13B)	107.1(12)
C(6A)-C(5A)-H(5AC)	109.3	C(1B)-N(4B)-C(13B)	108.9(9)
H(5AA)-C(5A)-H(5AC)	108.0	C(14B)-N(4B)-Co(1B)	108.1(12)
N(2A)-C(6A)-C(5A)	107.0(9)	C(1B)-N(4B)-Co(1B)	115.8(6)
N(2A)-C(6A)-H(6AA)	110.3	C(13B)-N(4B)-Co(1B)	107.0(8)
C(5A)-C(6A)-H(6AA)	110.3	N(4B)-C(1B)-C(2B)	117.2(7)
N(2A)-C(6A)-H(6AC)	110.3	N(4B)-C(1B)-H(1BA)	108.0
C(5A)-C(6A)-H(6AC)	110.3	C(2B)-C(1B)-H(1BA)	108.0
H(6AA)-C(6A)-H(6AC)	108.6	N(4B)-C(1B)-H(1BB)	108.0
N(2A)-C(7A)-H(7AA)	109.5	C(2B)-C(1B)-H(1BB)	108.0
N(2A)-C(7A)-H(7AB)	109.5	H(1BA)-C(1B)-H(1BB)	107.2
H(7AA)-C(7A)-H(7AB)	109.5	C(1B)-C(2B)-C(3B)	113.3(12)
N(2A)-C(7A)-H(7AC)	109.5	C(1B)-C(2B)-H(2BA)	108.9
H(7AA)-C(7A)-H(7AC)	109.5	C(3B)-C(2B)-H(2BA)	108.9
H(7AB)-C(7A)-H(7AC)	109.5	C(1B)-C(2B)-H(2BB)	108.9
N(2A)-C(8A)-C(9A)	114.3(13)	C(3B)-C(2B)-H(2BB)	108.9
N(2A)-C(8A)-H(8AA)	108.7	H(2BA)-C(2B)-H(2BB)	107.7
C(9A)-C(8A)-H(8AA)	108.7	N(1B)-C(3B)-C(2B)	116.2(13)
N(2A)-C(8A)-H(8AC)	108.7	N(1B)-C(3B)-H(3BA)	108.2
C(9A)-C(8A)-H(8AC)	108.7	C(2B)-C(3B)-H(3BA)	108.2
H(8AA)-C(8A)-H(8AC)	107.6	N(1B)-C(3B)-H(3BB)	108.2
C(10A)-C(9A)-C(8A)	114.8(16)	C(2B)-C(3B)-H(3BB)	108.2
C(10A)-C(9A)-H(9AA)	108.6	H(3BA)-C(3B)-H(3BB)	107.4
C(8A)-C(9A)-H(9AA)	108.6	N(1B)-C(4B)-H(4BA)	109.5
C(10A)-C(9A)-H(9AC)	108.6	N(1B)-C(4B)-H(4BB)	109.5
C(8A)-C(9A)-H(9AC)	108.6	H(4BA)-C(4B)-H(4BB)	109.5
H(9AA)-C(9A)-H(9AC)	107.5	N(1B)-C(4B)-H(4BC)	109.5
N(3A)-C(10A)-C(9A)	114.9(15)	H(4BA)-C(4B)-H(4BC)	109.5
N(3A)-C(10A)-H(10C)	108.5	H(4BB)-C(4B)-H(4BC)	109.5
C(9A)-C(10A)-H(10C)	108.5	N(1B)-C(5B)-C(6B)	108.5(11)
N(3A)-C(10A)-H(10D)	108.5	N(1B)-C(5B)-H(5BA)	110.0
C(9A)-C(10A)-H(10D)	108.5	C(6B)-C(5B)-H(5BA)	110.0
H(10C)-C(10A)-H(10D)	107.5	N(1B)-C(5B)-H(5BB)	110.0
N(3A)-C(11A)-H(11D)	109.5	C(6B)-C(5B)-H(5BB)	110.0
N(3A)-C(11A)-H(11E)	109.5	H(5BA)-C(5B)-H(5BB)	108.4
H(11D)-C(11A)-H(11E)	109.5	N(2B)-C(6B)-C(5B)	107.4(10)
N(3A)-C(11A)-H(11F)	109.5	N(2B)-C(6B)-H(6BA)	110.2
H(11D)-C(11A)-H(11F)	109.5	C(5B)-C(6B)-H(6BA)	110.2
H(11E)-C(11A)-H(11F)	109.5	N(2B)-C(6B)-H(6BB)	110.2
C(13A)-C(12A)-N(3A)	110.9(12)	C(5B)-C(6B)-H(6BB)	110.2
C(13A)-C(12A)-H(12C)	109.5	H(6BA)-C(6B)-H(6BB)	108.5
N(3A)-C(12A)-H(12C)	109.5	O(2B)-C(7B)-N(2B)	92.4(15)
C(13A)-C(12A)-H(12D)	109.5	O(2B)-C(7B)-H(7BA)	113.2
N(3A)-C(12A)-H(12D)	109.5	N(2B)-C(7B)-H(7BA)	113.2
H(12C)-C(12A)-H(12D)	108.0	O(2B)-C(7B)-H(7BB)	113.2
N(4A)-C(13A)-C(12A)	109.8(12)	N(2B)-C(7B)-H(7BB)	113.2
N(4A)-C(13A)-H(13C)	109.7	H(7BA)-C(7B)-H(7BB)	110.6
C(12A)-C(13A)-H(13C)	109.7	N(2B)-C(8B)-C(9B)	114.1(9)
N(4A)-C(13A)-H(13D)	109.7	N(2B)-C(8B)-H(8BA)	108.7

C(9B)-C(8B)-H(8BA)	108.7	O(23)-S(21)-C(21)	104.2(6)
N(2B)-C(8B)-H(8BB)	108.7	O(21)-S(21)-C(21)	102.5(7)
C(9B)-C(8B)-H(8BB)	108.7	F(23)-C(21)-F(21)	109.3(6)
H(8BA)-C(8B)-H(8BB)	107.6	F(23)-C(21)-F(22)	106.3(6)
C(8B)-C(9B)-C(10B)	115.4(13)	F(21)-C(21)-F(22)	108.2(6)
C(8B)-C(9B)-H(9BA)	108.4	F(23)-C(21)-S(21)	112.3(6)
C(10B)-C(9B)-H(9BA)	108.4	F(21)-C(21)-S(21)	109.6(6)
C(8B)-C(9B)-H(9BB)	108.4	F(22)-C(21)-S(21)	111.1(6)
C(10B)-C(9B)-H(9BB)	108.4	O(22A)-S(21A)-O(23A)	118.6(10)
H(9BA)-C(9B)-H(9BB)	107.5	O(22A)-S(21A)-O(21A)	113.0(8)
N(3B)-C(10B)-C(9B)	114.0(13)	O(23A)-S(21A)-O(21A)	111.9(11)
N(3B)-C(10B)-H(10E)	108.7	O(22A)-S(21A)-C(21A)	105.3(7)
C(9B)-C(10B)-H(10E)	108.7	O(23A)-S(21A)-C(21A)	102.7(9)
N(3B)-C(10B)-H(10F)	108.7	O(21A)-S(21A)-C(21A)	103.4(7)
C(9B)-C(10B)-H(10F)	108.7	F(23A)-C(21A)-F(22A)	106.3(8)
H(10E)-C(10B)-H(10F)	107.6	F(23A)-C(21A)-F(21A)	108.3(9)
N(3B)-C(11B)-H(11G)	109.5	F(22A)-C(21A)-F(21A)	107.1(8)
N(3B)-C(11B)-H(11H)	109.5	F(23A)-C(21A)-S(21A)	112.5(8)
H(11G)-C(11B)-H(11H)	109.5	F(22A)-C(21A)-S(21A)	111.3(7)
N(3B)-C(11B)-H(11I)	109.5	F(21A)-C(21A)-S(21A)	111.0(7)
H(11G)-C(11B)-H(11I)	109.5	O(33)-S(31)-O(32)	116.8(6)
H(11H)-C(11B)-H(11I)	109.5	O(33)-S(31)-O(31)	114.0(5)
C(13B)-C(12B)-N(3B)	109.2(15)	O(32)-S(31)-O(31)	112.6(5)
C(13B)-C(12B)-H(12E)	109.8	O(33)-S(31)-C(31)	104.3(4)
N(3B)-C(12B)-H(12E)	109.8	O(32)-S(31)-C(31)	103.4(5)
C(13B)-C(12B)-H(12F)	109.8	O(31)-S(31)-C(31)	103.8(4)
N(3B)-C(12B)-H(12F)	109.8	F(32)-C(31)-F(31)	109.1(6)
H(12E)-C(12B)-H(12F)	108.3	F(32)-C(31)-F(33)	106.3(6)
N(4B)-C(13B)-C(12B)	110.2(17)	F(31)-C(31)-F(33)	107.9(5)
N(4B)-C(13B)-H(13E)	109.6	F(32)-C(31)-S(31)	111.9(5)
C(12B)-C(13B)-H(13E)	109.6	F(31)-C(31)-S(31)	112.3(5)
N(4B)-C(13B)-H(13F)	109.6	F(33)-C(31)-S(31)	109.2(5)
C(12B)-C(13B)-H(13F)	109.6	O(33A)-S(31A)-O(31A)	112.3(10)
H(13E)-C(13B)-H(13F)	108.1	O(33A)-S(31A)-O(32A)	114.7(9)
N(4B)-C(14B)-H(14G)	109.5	O(31A)-S(31A)-O(32A)	112.7(9)
N(4B)-C(14B)-H(14H)	109.5	O(33A)-S(31A)-C(31A)	105.8(8)
H(14G)-C(14B)-H(14H)	109.5	O(31A)-S(31A)-C(31A)	105.5(8)
N(4B)-C(14B)-H(14I)	109.5	O(32A)-S(31A)-C(31A)	104.8(8)
H(14G)-C(14B)-H(14I)	109.5	F(33A)-C(31A)-F(32A)	109.1(8)
H(14H)-C(14B)-H(14I)	109.5	F(33A)-C(31A)-F(31A)	104.4(8)
O(22)-S(21)-O(23)	116.7(10)	F(32A)-C(31A)-F(31A)	105.8(8)
O(22)-S(21)-O(21)	114.4(9)	F(33A)-C(31A)-S(31A)	114.1(8)
O(23)-S(21)-O(21)	113.1(8)	F(32A)-C(31A)-S(31A)	114.3(8)
O(22)-S(21)-C(21)	103.6(8)	F(31A)-C(31A)-S(31A)	108.3(7)

Table S71. Anisotropic displacement parameters ($Å^2$x 10^3) for **8a-(OTf)₂**.
The anisotropic displacement factor exponent takes the form: $-2p^2[h^2 a^{*2}U^{11} + ... + 2 h k a^* b^* U^{12}]$

	U^{11}	U^{22}	U^{33}	U^{23}	U^{13}	U^{12}
Co(1)	22(1)	22(1)	23(1)	13(1)	12(1)	12(1)
O(1)	12(3)	36(3)	29(5)	21(3)	0(3)	7(3)
O(2)	26(5)	34(5)	35(5)	18(4)	15(4)	18(4)
N(1)	27(3)	42(4)	31(4)	20(3)	18(3)	22(3)
N(2)	32(3)	42(4)	29(4)	19(4)	15(3)	23(3)
N(3)	31(4)	34(4)	39(4)	23(3)	22(3)	15(3)
N(4)	29(4)	30(4)	25(4)	12(3)	4(3)	16(3)
C(1)	27(4)	33(5)	27(5)	12(4)	1(4)	11(3)
C(2)	30(4)	37(5)	41(5)	15(4)	7(4)	14(4)
C(3)	27(4)	41(4)	42(5)	23(4)	14(4)	15(3)
C(4)	23(5)	43(5)	43(5)	23(4)	14(4)	21(4)
C(5)	32(4)	57(5)	33(4)	22(4)	17(4)	24(4)
C(6)	36(4)	49(5)	33(5)	24(4)	15(4)	20(4)
C(7)	44(6)	45(8)	21(9)	9(7)	15(6)	26(6)
C(8)	31(4)	32(5)	33(4)	14(4)	13(4)	12(4)
C(9)	37(5)	31(5)	42(4)	21(4)	10(4)	14(4)
C(10)	30(4)	35(6)	50(4)	22(4)	22(4)	10(4)
C(11)	48(6)	31(6)	45(6)	24(5)	24(4)	22(6)
C(12)	40(5)	34(4)	36(4)	23(3)	19(4)	20(4)
C(13)	31(4)	34(4)	29(5)	22(3)	8(4)	20(3)
C(14)	39(7)	35(9)	28(6)	15(6)	-2(6)	17(6)
Co(1A)	22(1)	22(1)	23(1)	13(1)	12(1)	12(1)
O(1A)	12(3)	36(3)	29(5)	21(3)	0(3)	7(3)
O(2A)	26(5)	35(6)	34(5)	20(4)	15(4)	17(5)
N(1A)	22(3)	43(4)	35(4)	22(3)	18(3)	22(3)
N(2A)	28(3)	39(5)	31(4)	23(3)	15(3)	22(3)
N(3A)	31(4)	33(4)	37(4)	22(3)	15(4)	19(3)
N(4A)	24(4)	28(4)	30(4)	19(3)	11(3)	16(3)
C(1A)	34(4)	33(5)	29(5)	20(4)	15(4)	19(4)
C(2A)	29(4)	38(5)	37(5)	22(4)	16(4)	17(4)
C(3A)	25(4)	42(5)	33(5)	24(4)	19(4)	18(4)
C(4A)	23(5)	44(6)	39(5)	25(4)	15(4)	22(4)
C(5A)	33(4)	43(5)	34(4)	28(4)	26(3)	27(4)
C(6A)	36(4)	44(5)	33(4)	23(4)	20(3)	26(4)
C(7A)	43(6)	35(8)	36(7)	13(6)	21(5)	26(6)
C(8A)	30(4)	31(6)	34(4)	10(5)	11(3)	20(4)
C(9A)	31(5)	32(6)	41(5)	11(4)	16(4)	12(5)
C(10A)	31(5)	32(6)	46(5)	19(4)	18(4)	14(4)
C(11A)	48(6)	31(6)	45(6)	24(5)	24(4)	22(6)
C(12A)	33(5)	41(5)	31(5)	26(4)	16(4)	18(4)
C(13A)	28(5)	30(4)	29(5)	15(4)	16(4)	13(4)
C(14A)	40(7)	31(7)	27(7)	15(5)	14(5)	27(6)
Co(1B)	22(1)	22(1)	23(1)	13(1)	12(1)	12(1)
O(1B)	12(3)	36(3)	29(5)	21(3)	0(3)	7(3)
O(2B)	19(4)	27(5)	35(5)	20(4)	10(4)	8(4)
N(1B)	23(5)	29(5)	34(4)	15(3)	2(3)	8(3)
N(2B)	24(3)	48(4)	40(4)	31(3)	13(3)	19(3)
N(3B)	37(4)	44(5)	17(4)	13(4)	11(3)	23(4)
N(4B)	26(4)	29(4)	35(4)	18(3)	11(3)	14(3)
C(1B)	22(3)	34(4)	37(4)	22(3)	18(3)	12(3)
C(2B)	32(4)	35(4)	38(4)	21(3)	16(4)	18(4)
C(3B)	28(5)	34(5)	31(6)	19(4)	8(4)	19(4)
C(4B)	34(8)	30(8)	28(6)	8(6)	-5(6)	12(6)
C(5B)	30(5)	34(6)	44(6)	18(5)	10(4)	8(4)
C(6B)	29(4)	41(4)	51(4)	22(4)	12(4)	7(4)
C(7B)	26(6)	40(5)	39(5)	27(4)	21(4)	15(4)
C(8B)	28(4)	56(5)	48(5)	35(4)	19(4)	18(4)
C(9B)	37(4)	66(6)	37(5)	30(5)	21(4)	27(4)
C(10B)	38(5)	53(5)	31(6)	24(5)	17(4)	18(5)
C(11B)	57(7)	50(8)	23(10)	8(7)	22(8)	32(6)
C(12B)	34(5)	37(7)	33(5)	10(5)	7(4)	14(5)
C(13B)	30(6)	37(6)	38(4)	11(4)	8(4)	16(5)
C(14B)	38(8)	39(8)	39(9)	27(7)	19(6)	20(6)
S(21)	24(2)	34(2)	35(3)	12(2)	16(2)	12(2)
F(21)	60(3)	51(3)	63(4)	7(3)	38(3)	25(2)
F(22)	46(3)	51(3)	60(4)	0(3)	-2(2)	25(2)
F(23)	77(4)	34(3)	82(4)	30(3)	23(3)	21(2)
O(21)	20(3)	55(5)	42(4)	14(4)	10(3)	18(3)
O(22)	43(5)	32(4)	41(4)	19(3)	25(3)	10(4)
O(23)	19(3)	33(5)	28(4)	11(3)	11(3)	11(3)
C(21)	36(3)	36(3)	48(4)	11(3)	18(3)	22(3)
S(21A)	31(2)	29(2)	35(3)	9(2)	18(2)	13(1)
F(21A)	57(3)	53(4)	125(7)	-32(4)	41(4)	-4(3)
F(22A)	84(4)	49(3)	110(6)	5(4)	70(4)	28(3)
F(23A)	180(8)	72(4)	172(9)	76(5)	120(7)	84(5)
O(21A)	21(3)	36(5)	26(4)	5(3)	8(3)	14(3)
O(22A)	29(4)	71(7)	55(5)	10(5)	22(3)	18(4)
O(23A)	63(8)	60(7)	58(5)	24(5)	39(5)	22(6)
C(21A)	64(4)	47(4)	83(6)	12(4)	50(4)	22(3)
S(31)	30(2)	32(2)	41(2)	20(1)	10(1)	12(1)
F(31)	35(2)	54(3)	92(4)	29(3)	14(3)	17(2)
F(32)	67(4)	44(3)	143(6)	12(3)	34(4)	32(3)
F(33)	70(3)	144(4)	134(4)	115(4)	19(3)	43(3)
O(31)	44(2)	47(2)	42(2)	29(2)	14(2)	14(2)
O(32)	80(3)	61(3)	51(3)	9(2)	32(2)	27(2)
O(33)	37(2)	57(2)	57(2)	37(2)	18(2)	10(2)
C(31)	41(3)	41(3)	76(4)	35(3)	17(3)	21(2)
S(31A)	38(3)	41(3)	39(3)	21(2)	22(2)	17(2)
F(31A)	56(3)	65(4)	57(3)	18(3)	36(3)	25(3)
F(32A)	28(3)	56(5)	77(6)	-1(4)	12(4)	18(3)

F(33A)	45(5)	31(4)	93(6)	3(4)	9(4)	22(3)
O(31A)	106(6)	72(5)	64(4)	50(4)	45(4)	42(5)
O(32A)	30(3)	30(3)	22(3)	15(2)	15(2)	15(2)
O(33A)	25(3)	48(4)	60(4)	-2(4)	13(3)	6(3)
C(31A)	29(3)	35(4)	60(4)	13(3)	21(3)	11(3)

Table S72. Hydrogen coordinates (x 10^4) and isotropic displacement parameters ($Å^2$x 10^3) for **8a-(OTf)$_2$**.

	x	y	z	U(eq)
H(1A)	6180(50)	1530(110)	7410(130)	39
H(1B)	7140(60)	2100(70)	7100(70)	39
H(1AA)	9932	3923	10751	37
H(1AB)	8926	3585	9444	37
H(2A)	11213	4460	9741	45
H(2AB)	11405	3291	9939	45
H(3A)	11216	3099	8060	41
H(3AB)	9847	3303	7797	41
H(4A)	10565	361	7675	40
H(4AB)	10984	1362	8979	40
H(5A)	9762	656	6143	44
H(5AB)	9507	1878	6174	44
H(6A)	7273	949	5686	44
H(6AB)	7562	-158	4929	44
H(7A)	7263	-1929	5289	53
H(7B)	7410	-1913	6576	53
H(7C)	8664	-1122	6388	53
H(8A)	5451	-1408	5040	39
H(8AB)	5500	-134	5870	39
H(9A)	4154	-1868	6092	43
H(9AB)	5456	-2139	6580	43
H(10A)	5144	35	7770	43
H(10B)	4819	-1140	8098	43
H(11A)	6515	-1611	9171	55
H(11B)	8057	-888	9450	55
H(11C)	7053	-1799	8123	55
H(12A)	5996	613	9778	39
H(12B)	7307	448	10468	39
H(13A)	7197	2376	9703	33
H(13B)	8046	2500	10997	33
H(14A)	10112	2474	11491	54
H(14B)	10682	1935	10532	54
H(14C)	9455	1038	10671	54
H(1C)	6260(70)	1070(60)	6530(30)	39
H(1D)	6390(80)	1740(100)	7610(40)	39
H(1AC)	9793	3998	10241	33
H(1AD)	8772	3453	8896	33
H(2AA)	11067	4313	9166	38
H(2AC)	11300	3251	9565	38
H(3AA)	9653	2892	7271	34
H(3AC)	11077	2792	7611	34
H(4AA)	10506	97	7633	36
H(4AC)	10987	1258	8848	36
H(5AA)	9670	99	6005	33
H(5AC)	9432	1252	5792	33
H(6AA)	7182	416	5403	38
H(6AC)	7458	-755	4751	38
H(7AA)	7331	-2315	5339	51
H(7AB)	7335	-2257	6608	51
H(7AC)	8667	-1489	6503	51
H(8AA)	5461	-1903	5023	37
H(8AC)	5468	-585	5679	37
H(9AA)	4146	-2154	6092	42
H(9AC)	5461	-2347	6714	42
H(10C)	5016	-158	7563	41
H(10D)	4839	-1176	8111	41
H(11D)	6716	-1341	9373	55
H(11E)	8202	-610	9506	55
H(11F)	7154	-1699	8272	55
H(12C)	5949	783	9599	36
H(12D)	7284	716	10382	36
H(13C)	7103	2387	9274	32
H(13D)	7944	2710	10637	32
H(14D)	10003	2684	11268	42
H(14E)	10643	2096	10394	42
H(14F)	9411	1228	10550	42
H(1E)	6260(30)	1530(80)	7410(170)	39
H(1F)	7420(60)	2380(60)	7610(100)	39
H(1BA)	4920	-780	8622	33
H(1BB)	5188	280	8134	33
H(2BA)	6828	468	10353	38
H(2BB)	5739	1067	10173	38
H(3BA)	6969	2328	9440	34
H(3BB)	7792	2590	10782	34
H(4BA)	9693	2157	11366	53
H(4BB)	10239	1520	10430	53
H(4BC)	8929	722	10545	53
H(5BA)	9825	3634	10539	45
H(5BB)	8777	3353	9258	45
H(6BA)	10762	3604	8980	50
H(6BB)	11150	2827	9737	50
H(7BA)	10493	438	7492	36
H(7BB)	11324	1342	8885	36
H(8BA)	10757	2468	7196	47
H(8BB)	9438	2751	7076	47
H(9BA)	9485	454	5848	50
H(9BB)	9189	1385	5267	50
H(10E)	7183	1028	5696	46
H(10F)	7155	-104	4678	46
H(11G)	7363	-1827	4995	60
H(11H)	7296	-2048	6156	60
H(11I)	8657	-1138	6207	60

H(12E)	5449	-210	6017	44
H(12F)	5275	-1500	5097	44
H(13E)	4564	-1687	6682	43
H(13F)	5659	-2258	6565	43
H(14G)	7481	-855	9433	51
H(14H)	7440	-1716	8207	51
H(14I)	6157	-2044	8545	51

Table S73. Hydrogen bonds for **8a-(OTf)$_2$** [Å and °].

D-H...A	d(D-H)	d(H...A)	d(D...A)	<(DHA)
O(1)-H(1A)...O(23)	0.83(2)	1.77(5)	2.560(17)	157(12)
O(1)-H(1B)...S(31)	0.84(2)	2.87(4)	3.633(17)	152(8)
O(1)-H(1B)...O(31)	0.84(2)	2.13(7)	2.683(16)	123(8)
O(1)-H(1B)...O(32)	0.84(2)	2.66(3)	3.495(16)	178(8)
O(1A)-H(1D)...O(21A)	0.85(2)	1.99(6)	2.643(18)	132(7)

[(D12-TMC-propyl-O)Co^{III}(OH)]B$_{ArF}$ · 3 CH_2Cl_2 , **9b-B$_{ArF}$**

The structure of **9b-B$_{ArF}$** was solved in the orthorhombic space group *Pbca*. The asymmetric contains the heavily disordered complex molecule, anion and three solvent molecules (CH_2Cl_2). The disorder of the complex was modelled across three positions, two of them were interpreted as hydroxylated ligand. The third positions shows coordination of free OH^- instead. The anion also exhibits full molecule disorder, as do the solvent molecules. The disorder was refined with the help of same distance restraints on 1,2 and 1,3-distances, similarity restraints on the anisotropic displacement parameters, restraints to a common plane and advanced rigid bond restraints. Additionally, disordered atoms with very close positions were set to the same anisotropic displacement parameters. All disorder ratios were allowed to refine freely and converged to 0.483(3):0.281(3):0.237(3) (complex), 0.522(6) (anion) and 0.838(3):0.057(3):0.105(2), 0.71(1) and 0.73(2) (solvent molecules).

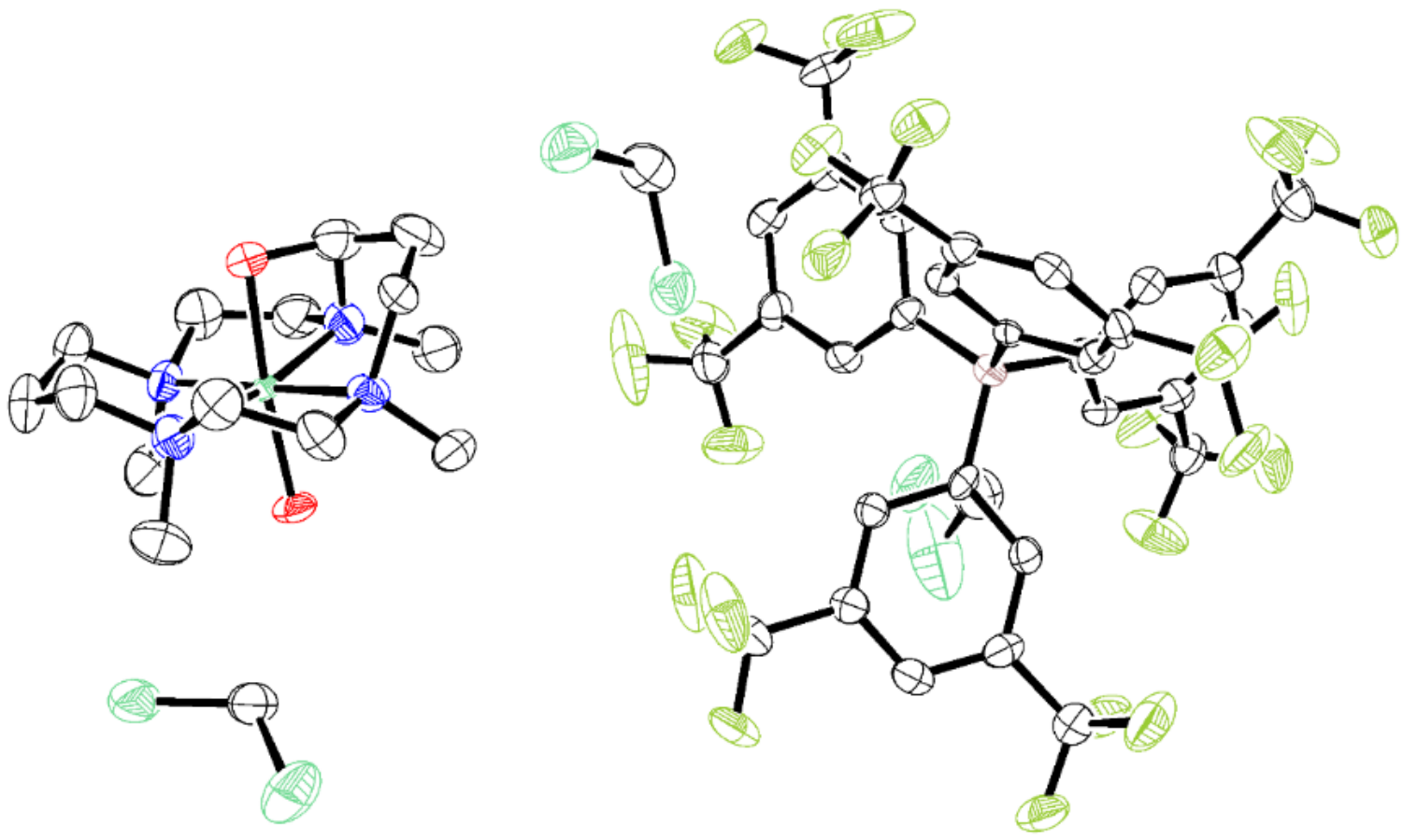

Figure S15. Molecular structure of (**9b-B$_{ArF}$**).

Table S74. Crystal data and structure refinement for **9b-B$_{ArF}$**.

CCDC No	2062724	
Empirical formula	$C_{49}H_{38.19}BCl_6CoD_{12}F_{24}N_4O_2$	
Formula weight	1477.63	
Temperature	100(2) K	
Wavelength	0.71073 Å	
Crystal system	Orthorhombic	
Space group	*Pbca*	
Unit cell dimensions	a = 24.4906(18) Å	α = 90°
	b = 19.1183(14) Å	β = 90°
	c = 25.3616(19) Å	γ = 90°
Volume	11874.8(15) Å^3	
Z	8	
Density (calculated)	1.653 Mg/m^3	
Absorption coefficient	0.678 mm^{-1}	
F(000)	5906	
Crystal size	0.248 x 0.137 x 0.060 mm^3	
Theta range for data collection	1.572 to 25.350°.	
Index ranges	-29 ≤ h ≤ 29, -23 ≤ k ≤ 23, -30 ≤ l ≤ 30	
Reflections collected	125729	
Independent reflections	10882 [R(int) = 0.0574]	
Completeness to theta = 25.242°	100.0 %	
Absorption correction	Semi-empirical from equivalents	
Refinement method	Full-matrix least-squares on F^2	
Data / restraints / parameters	10882 / 7301 / 1765	
Goodness-of-fit on F^2	1.026	
Final R indices [I>2σ(I)]	R1 = 0.0508, wR2 = 0.1257	
R indices (all data)	R1 = 0.0770, wR2 = 0.1448	
Extinction coefficient	0.00058(8)	
Largest diff. peak and hole	0.551 and -0.414 eÅ^{-3}	

Table S75. Atomic coordinates (x 10^4) and equivalent isotropic displacement parameters (Å^{2}x 10^3) for **9b-B$_{ArF}$**. U(eq) is defined as one third of the trace of the orthogonalized U^{ij} tensor.

	x	y	z	U(eq)
O(2)	6515(16)	4215(13)	4930(14)	37(3)
Co(1)	6700(2)	3391(4)	4575(2)	17(1)
O(1)	6770(4)	2550(7)	4190(6)	42(2)
N(1)	5956(5)	3082(6)	4381(6)	44(2)
N(2)	6706(5)	2842(6)	5285(5)	35(2)
N(3)	7519(6)	3564(10)	4714(7)	39(2)
N(4)	6714(6)	3920(10)	3863(6)	36(2)
C(1)	6194(4)	2360(4)	4306(3)	51(2)
C(2)	6153(6)	1921(7)	4813(5)	54(2)
C(3)	6623(3)	2074(4)	5183(3)	39(2)
C(4)	6326(7)	3066(10)	5713(7)	39(3)
D(4A)	5948	3017	5591	58
D(4B)	6383	2772	6025	58
D(4C)	6397	3556	5803	58
C(5)	7269(6)	2932(10)	5501(5)	45(2)
C(6)	7670(6)	2956(11)	5059(6)	47(3)
C(7)	7667(17)	4217(13)	5004(12)	60(6)
D(7A)	7439	4260	5320	90
D(7B)	8052	4197	5108	90
D(7C)	7608	4623	4775	90
C(8)	7873(8)	3510(10)	4234(7)	51(3)
C(9)	7738(6)	3990(10)	3795(7)	51(3)
C(10)	7211(6)	3785(14)	3531(7)	46(3)
C(11)	6657(12)	4693(10)	3926(11)	57(5)
D(11A)	6967	4874	4128	85
D(11B)	6650	4915	3577	85
D(11C)	6317	4798	4113	85
C(12)	6250(6)	3623(9)	3557(6)	54(3)
C(13)	5782(6)	3446(9)	3892(6)	51(3)
C(14)	5456(6)	3131(6)	4716(6)	53(3)
D(14A)	5525	2903	5056	80
D(14B)	5365	3624	4775	80
D(14C)	5152	2897	4538	80
O(2A)	6418(16)	4312(16)	4970(16)	51(5)
Co(1A)	6663(7)	3509(9)	4616(7)	40(3)
O(1A)	6920(8)	2541(10)	4388(5)	56(3)
N(1A)	5900(10)	3177(11)	4357(10)	42(3)
N(2A)	6681(9)	2804(10)	5215(8)	40(3)
N(3A)	7511(10)	3506(18)	4738(11)	44(3)
N(4A)	6786(11)	3860(20)	3844(11)	40(3)
C(1A)	5842(9)	2399(10)	4276(7)	46(3)
C(2A)	6109(8)	1957(11)	4680(9)	53(3)
C(3A)	6673(6)	2192(7)	4848(6)	51(2)
C(4A)	6255(10)	2811(16)	5635(10)	41(5)
D(4AA)	6297	3232	5852	61
D(4AB)	5892	2811	5472	61
D(4AC)	6295	2395	5857	61
C(5A)	7209(9)	2848(19)	5511(9)	44(3)
C(6A)	7670(11)	2970(20)	5146(11)	44(3)
C(7A)	7640(20)	4214(19)	4954(19)	47(6)
D(7AA)	7398	4313	5254	71
D(7AB)	8020	4226	5071	71
D(7AC)	7582	4567	4679	71
C(8A)	7884(15)	3364(18)	4279(11)	50(4)
C(9A)	7815(11)	3870(20)	3840(12)	50(4)
C(10A)	7302(10)	3770(30)	3535(12)	46(4)
C(11A)	6650(20)	4620(20)	3870(30)	40(4)
D(11D)	6314	4688	4071	60
D(11E)	6950	4873	4043	60
D(11F)	6602	4804	3511	60
C(12A)	6338(11)	3510(20)	3531(12)	40(3)
C(13A)	5829(10)	3573(14)	3847(10)	45(3)
C(14A)	5437(14)	3413(12)	4701(11)	41(5)
D(14D)	5465	3918	4760	62
D(14E)	5090	3307	4527	62
D(14F)	5455	3168	5040	62
O(2B)	6490(30)	4160(30)	4870(30)	37(3)
Co(1B)	6732(7)	3327(10)	4558(8)	50(4)
O(1B)	6943(13)	2520(20)	4153(15)	72(6)
N(1B)	5878(12)	3158(13)	4324(10)	43(3)
N(2B)	6626(6)	2661(9)	5185(9)	40(3)
N(3B)	7471(11)	3590(12)	4835(8)	44(3)
N(4B)	6800(11)	3820(20)	3834(14)	40(3)
C(1B)	5752(11)	2388(12)	4319(8)	49(3)
C(2B)	5802(6)	2031(8)	4835(6)	46(3)
C(3B)	6362(6)	1983(8)	5038(8)	42(3)
C(4B)	6401(13)	2920(20)	5700(14)	38(6)
D(4BA)	6576	3362	5793	57
D(4BB)	6006	2989	5666	57
D(4BC)	6474	2572	5976	57
C(5B)	7214(5)	2474(8)	5290(7)	48(3)
C(6B)	7541(7)	3127(9)	5306(7)	49(3)
C(7B)	7550(20)	4337(12)	5000(17)	52(6)
D(7BA)	7278	4460	5268	78
D(7BB)	7918	4395	5147	78
D(7BC)	7507	4644	4693	78
C(8B)	7888(11)	3421(16)	4430(7)	45(3)
C(9B)	7827(11)	3812(19)	3927(9)	48(4)
C(10B)	7343(10)	3621(16)	3609(10)	46(4)
C(11B)	6721(19)	4590(20)	3810(30)	40(4)

D(11G)	6998	4822	4032	60
D(11H)	6756	4755	3449	60
D(11I)	6357	4711	3947	60
C(12B)	6378(12)	3470(30)	3501(12)	43(3)
C(13B)	5846(11)	3412(16)	3763(9)	48(4)
C(14B)	5442(18)	3560(16)	4608(12)	51(6)
D(14G)	5533	4059	4606	77
D(14H)	5091	3488	4431	77
D(14I)	5417	3395	4973	77
F(21)	3669(5)	3375(10)	8834(7)	57(3)
F(22)	4188(6)	2801(8)	9352(7)	75(4)
F(23)	4426(5)	3807(4)	9100(4)	52(2)
F(24)	5746(5)	3864(5)	7781(4)	83(4)
F(25)	5580(3)	3322(5)	7083(3)	81(3)
F(26)	6095(4)	2878(5)	7639(4)	76(3)
F(31)	5892(10)	-415(14)	6931(6)	46(4)
F(32)	6415(5)	-116(5)	7557(5)	53(2)
F(33)	6065(5)	-1131(5)	7565(6)	47(2)
F(34)	5327(6)	-609(9)	9343(8)	46(2)
F(35)	4489(4)	-774(7)	9173(6)	61(2)
F(36)	4762(7)	227(6)	9429(6)	64(3)
F(41)	3171(4)	-1020(8)	8060(7)	61(3)
F(42)	2629(4)	-637(6)	8618(4)	79(3)
F(43)	2362(4)	-804(5)	7835(3)	63(2)
F(44)	1920(2)	1845(3)	8321(3)	67(2)
F(45)	2032(3)	2085(4)	7520(2)	76(2)
F(46)	2515(2)	2575(2)	8092(3)	68(2)
F(51)	4612(7)	-34(11)	5368(6)	74(3)
F(52)	3760(4)	-133(10)	5534(6)	55(3)
F(53)	4361(7)	-602(8)	6037(6)	69(4)
F(54)	4193(5)	3092(6)	6113(4)	82(3)
F(55)	3931(4)	2595(3)	5442(2)	79(2)
F(56)	4761(3)	2632(4)	5562(4)	119(3)
C(21)	4494(6)	1930(6)	7813(5)	22(2)
C(22)	4265(7)	2264(8)	8247(6)	28(3)
C(23)	4465(9)	2884(10)	8466(8)	32(2)
C(24)	4908(9)	3214(11)	8241(8)	32(3)
C(25)	5165(7)	2877(10)	7825(8)	29(2)
C(26)	4960(9)	2268(11)	7602(9)	25(3)
C(27)	4193(6)	3211(8)	8931(6)	43(2)
C(28)	5646(5)	3239(7)	7587(5)	37(2)
C(31)	4691(5)	601(6)	7798(6)	21(2)
C(32)	5126(6)	299(9)	7525(7)	23(2)
C(33)	5472(7)	-192(11)	7757(7)	27(3)
C(34)	5393(9)	-409(13)	8270(7)	25(3)
C(35)	4974(8)	-103(12)	8559(6)	25(2)
C(36)	4628(9)	380(13)	8323(7)	24(3)
C(37)	5953(5)	-463(7)	7457(6)	33(2)
C(38)	4892(6)	-314(8)	9119(5)	37(2)
C(41)	3639(5)	1022(8)	7734(4)	26(2)
C(42)	3459(6)	340(8)	7837(7)	25(2)
C(43)	2923(7)	183(7)	7990(12)	29(2)
C(44)	2542(7)	718(7)	8030(12)	29(3)
C(45)	2708(6)	1392(7)	7922(9)	29(2)
C(46)	3245(6)	1550(9)	7785(10)	25(3)
C(47)	2772(6)	-553(7)	8113(6)	39(2)
C(48)	2301(4)	1980(6)	7968(4)	35(2)
C(51)	4272(5)	1209(8)	6913(5)	24(2)
C(52)	4262(7)	596(9)	6610(6)	27(3)
C(53)	4272(10)	618(7)	6063(6)	29(2)
C(54)	4292(13)	1246(8)	5792(7)	30(3)
C(55)	4287(10)	1852(7)	6085(6)	30(2)
C(56)	4266(11)	1836(9)	6631(6)	25(3)
C(57)	4250(6)	-43(7)	5751(6)	38(2)
C(58)	4303(5)	2552(6)	5814(4)	43(2)
B(21)	4269(5)	1190(7)	7558(5)	25(2)
F(21A)	3706(7)	3509(11)	8867(7)	65(4)
F(22A)	4023(6)	2688(8)	9338(7)	54(3)
F(23A)	4475(6)	3629(7)	9252(5)	98(4)
F(24A)	5645(5)	3890(5)	7579(5)	68(3)
F(25A)	5764(4)	2997(5)	7121(3)	76(3)
F(26A)	6093(4)	3043(7)	7877(3)	71(3)
F(31A)	5837(11)	-489(16)	6947(7)	40(3)
F(32A)	6379(6)	116(6)	7434(6)	55(3)
F(33A)	6192(6)	-956(6)	7627(6)	62(3)
F(34A)	5382(7)	-471(11)	9392(9)	59(4)
F(35A)	4647(5)	-946(7)	9132(7)	77(4)
F(36A)	4629(7)	82(7)	9429(6)	68(4)
F(41A)	2898(5)	-672(8)	8670(4)	89(3)
F(42A)	2293(4)	-729(6)	8107(5)	93(4)
F(43A)	3117(6)	-1057(10)	7927(7)	69(4)
F(44A)	1758(2)	1648(3)	7781(3)	76(2)
F(45A)	2335(3)	2383(3)	7550(3)	65(2)
F(46A)	2224(4)	2170(6)	8360(3)	109(4)
F(51A)	4689(8)	-129(13)	5500(7)	92(5)
F(52A)	3850(6)	-63(11)	5475(6)	73(4)
F(53A)	4224(8)	-604(8)	6099(7)	57(3)
F(54A)	4057(4)	3055(7)	6045(5)	61(3)
F(55A)	4332(3)	2575(3)	5347(2)	58(2)
F(56A)	4887(2)	2848(3)	5948(2)	45(1)
C(21A)	4488(6)	1944(7)	7887(6)	25(3)
C(22A)	4250(8)	2257(9)	8325(7)	26(3)
C(23A)	4460(10)	2878(12)	8527(9)	32(3)
C(24A)	4910(10)	3202(13)	8313(9)	34(3)
C(25A)	5147(8)	2913(11)	7860(9)	30(3)

C(26A)	4954(10)	2272(12)	7677(9)	26(3)
C(27A)	4167(7)	3172(8)	8994(6)	43(3)
C(28A)	5649(6)	3202(7)	7605(5)	36(2)
C(31A)	4710(6)	631(7)	7858(7)	25(2)
C(32A)	5150(7)	372(10)	7569(8)	23(3)
C(33A)	5512(8)	-112(12)	7782(8)	24(3)
C(34A)	5459(9)	-349(14)	8292(8)	25(3)
C(35A)	5027(9)	-90(13)	8588(7)	27(3)
C(36A)	4667(10)	394(14)	8378(8)	24(3)
C(37A)	5981(6)	-358(8)	7451(7)	34(3)
C(38A)	4925(7)	-349(9)	9133(6)	39(3)
C(41A)	3647(5)	999(9)	7765(5)	26(2)
C(42A)	3499(7)	308(9)	7887(7)	28(3)
C(43A)	2955(7)	141(7)	7994(12)	29(3)
C(44A)	2545(9)	635(8)	7999(14)	32(3)
C(45A)	2681(6)	1313(7)	7873(9)	29(3)
C(46A)	3219(6)	1482(10)	7736(11)	28(3)
C(47A)	2821(6)	-587(8)	8161(7)	42(3)
C(48A)	2256(5)	1876(6)	7900(5)	40(3)
C(51A)	4295(5)	1249(9)	6976(5)	23(2)
C(52A)	4273(8)	638(10)	6671(6)	24(3)
C(53A)	4289(12)	639(8)	6124(7)	30(3)
C(54A)	4335(14)	1275(8)	5856(7)	30(3)
C(55A)	4333(10)	1889(7)	6139(6)	28(3)
C(56A)	4320(12)	1874(10)	6690(6)	25(3)
C(57A)	4268(7)	-19(8)	5815(6)	40(3)
C(58A)	4399(4)	2585(7)	5863(4)	37(2)
B(21A)	4277(6)	1208(7)	7621(5)	22(2)
Cl(61)	7001(1)	6133(1)	4680(1)	73(1)
Cl(62)	6737(1)	6046(1)	5788(1)	82(1)
C(61)	6526(2)	5860(2)	5146(2)	50(1)
Cl(1C)	7301(8)	5923(9)	4899(9)	52(5)
Cl(2C)	6645(15)	5775(19)	5810(16)	80(3)
C(61A)	7240(20)	6140(40)	5577(13)	50(1)
Cl(1D)	6228(5)	6289(6)	5443(6)	80(3)
Cl(2D)	7334(5)	5852(7)	5482(6)	82(4)
C(61B)	6688(9)	5670(20)	5715(18)	50(1)
Cl(71)	6520(2)	326(2)	5850(1)	59(1)
Cl(72)	5706(2)	1281(3)	6278(2)	51(1)
C(71)	5818(4)	539(4)	5901(4)	52(2)
Cl(1A)	6751(4)	621(6)	5732(3)	68(3)
Cl(2A)	5770(5)	1090(8)	6237(6)	84(4)
C(71A)	6059(8)	415(11)	5874(9)	55(5)
Cl(81)	2941(1)	3237(2)	6672(1)	72(1)
Cl(82)	2049(3)	4246(4)	6518(4)	146(2)
C(81)	2236(4)	3436(5)	6721(9)	78(3)
Cl(1B)	2953(6)	3297(10)	6525(14)	170(6)
Cl(2B)	2125(5)	4314(4)	6607(3)	66(3)
C(81A)	2244(10)	3436(10)	6620(30)	78(3)

Table S76. Bond lengths [Å] and angles [°] for **9b-B$_{Ar}$F**.

O(2)-Co(1)	1.871(10)	C(7A)-D(7AA)	0.9800
O(2)-H(2)	0.84(2)	C(7A)-D(7AB)	0.9800
Co(1)-O(1)	1.888(15)	C(7A)-D(7AC)	0.9800
Co(1)-N(1)	1.976(14)	C(8A)-C(9A)	1.486(14)
Co(1)-N(3)	2.064(16)	C(8A)-H(8AA)	0.9900
Co(1)-N(4)	2.069(15)	C(8A)-H(8AC)	0.9900
Co(1)-N(2)	2.085(13)	C(9A)-C(10A)	1.488(14)
Co(1)-C(1)	2.424(10)	C(9A)-H(9AA)	0.9500
O(1)-C(1)	1.484(12)	C(10A)-H(10C)	0.9900
N(1)-C(13)	1.485(11)	C(10A)-H(10D)	0.9900
N(1)-C(14)	1.493(10)	C(11A)-D(11D)	0.9800
N(1)-C(1)	1.511(10)	C(11A)-D(11E)	0.9800
N(2)-C(4)	1.492(10)	C(11A)-D(11F)	0.9800
N(2)-C(5)	1.493(10)	C(12A)-C(13A)	1.486(14)
N(2)-C(3)	1.504(10)	C(12A)-H(12C)	0.9900
N(3)-C(7)	1.495(11)	C(12A)-H(12D)	0.9900
N(3)-C(8)	1.497(10)	C(13A)-H(13C)	0.9900
N(3)-C(6)	1.502(11)	C(13A)-H(13D)	0.9900
N(4)-C(12)	1.488(10)	C(14A)-D(14D)	0.9800
N(4)-C(11)	1.494(10)	C(14A)-D(14E)	0.9800
N(4)-C(10)	1.501(10)	C(14A)-D(14F)	0.9800
C(1)-C(2)	1.539(11)	O(2B)-Co(1B)	1.880(15)
C(1)-H(1)	1.0000	O(2B)-H(2CA)	0.84(2)
C(2)-C(3)	1.513(10)	Co(1B)-O(1B)	1.93(4)
C(2)-H(2A)	0.9900	Co(1B)-N(3B)	2.01(3)
C(2)-H(2AB)	0.9900	Co(1B)-N(2B)	2.05(3)
C(3)-H(3A)	0.9900	Co(1B)-N(4B)	2.07(4)
C(3)-H(3AB)	0.9900	Co(1B)-N(1B)	2.20(3)
C(4)-D(4A)	0.9800	O(1B)-H(1B)	0.85(2)
C(4)-D(4B)	0.9800	N(1B)-C(14B)	1.499(14)
C(4)-D(4C)	0.9800	N(1B)-C(1B)	1.504(14)
C(5)-C(6)	1.491(11)	N(1B)-C(13B)	1.506(14)
C(5)-H(5A)	0.9900	N(2B)-C(3B)	1.496(13)
C(5)-H(5AB)	0.9900	N(2B)-C(4B)	1.501(14)
C(6)-H(6A)	0.9900	N(2B)-C(5B)	1.508(13)
C(6)-H(6AB)	0.9900	N(3B)-C(8B)	1.484(13)
C(7)-D(7A)	0.9800	N(3B)-C(6B)	1.496(13)
C(7)-D(7B)	0.9800	N(3B)-C(7B)	1.502(14)
C(7)-D(7C)	0.9800	N(4B)-C(11B)	1.488(14)
C(8)-C(9)	1.480(10)	N(4B)-C(12B)	1.497(14)
C(8)-H(8A)	0.9900	N(4B)-C(10B)	1.497(14)
C(8)-H(8AB)	0.9900	C(1B)-C(2B)	1.481(14)
C(9)-C(10)	1.507(11)	C(1B)-H(1BA)	0.9900
C(9)-H(9A)	0.9900	C(1B)-H(1BB)	0.9900
C(9)-H(9AB)	0.9900	C(2B)-C(3B)	1.469(13)
C(10)-H(10A)	0.9900	C(2B)-H(2CB)	0.9900
C(10)-H(10B)	0.9900	C(2B)-H(2CC)	0.9900
C(11)-D(11A)	0.9800	C(3B)-H(3BA)	0.9900
C(11)-D(11B)	0.9800	C(3B)-H(3BB)	0.9900
C(11)-D(11C)	0.9800	C(4B)-D(4BA)	0.9800
C(12)-C(13)	1.467(11)	C(4B)-D(4BB)	0.9800
C(12)-H(12A)	0.9900	C(4B)-D(4BC)	0.9800
C(12)-H(12B)	0.9900	C(5B)-C(6B)	1.485(13)
C(13)-H(13A)	0.9900	C(5B)-H(5BA)	0.9900
C(13)-H(13B)	0.9900	C(5B)-H(5BB)	0.9900
C(14)-D(14A)	0.9800	C(6B)-H(6BA)	0.9900
C(14)-D(14B)	0.9800	C(6B)-H(6BB)	0.9900
C(14)-D(14C)	0.9800	C(7B)-D(7BA)	0.9800
O(2A)-Co(1A)	1.875(14)	C(7B)-D(7BB)	0.9800
O(2A)-H(2BA)	0.84(2)	C(7B)-D(7BC)	0.9800
Co(1A)-N(2A)	2.03(2)	C(8B)-C(9B)	1.485(14)
Co(1A)-O(1A)	2.04(2)	C(8B)-H(8BA)	0.9900
Co(1A)-N(1A)	2.08(2)	C(8B)-H(8BB)	0.9900
Co(1A)-N(4A)	2.09(3)	C(9B)-C(10B)	1.480(14)
Co(1A)-N(3A)	2.10(2)	C(9B)-H(9BA)	0.9900
O(1A)-C(3A)	1.475(17)	C(9B)-H(9BB)	0.9900
N(1A)-C(14A)	1.500(13)	C(10B)-H(10E)	0.9900
N(1A)-C(1A)	1.507(13)	C(10B)-H(10F)	0.9900
N(1A)-C(13A)	1.511(13)	C(11B)-D(11G)	0.9800
N(2A)-C(4A)	1.491(13)	C(11B)-D(11H)	0.9800
N(2A)-C(3A)	1.497(13)	C(11B)-D(11I)	0.9800
N(2A)-C(5A)	1.498(13)	C(12B)-C(13B)	1.467(14)
N(3A)-C(7A)	1.492(14)	C(12B)-H(12E)	0.9900
N(3A)-C(8A)	1.503(14)	C(12B)-H(12F)	0.9900
N(3A)-C(6A)	1.506(14)	C(13B)-H(13E)	0.9900
N(4A)-C(11A)	1.497(14)	C(13B)-H(13F)	0.9900
N(4A)-C(10A)	1.497(14)	C(14B)-D(14G)	0.9800
N(4A)-C(12A)	1.509(13)	C(14B)-D(14H)	0.9800
C(1A)-C(2A)	1.481(13)	C(14B)-D(14I)	0.9800
C(1A)-H(1AA)	0.9900	F(21)-C(27)	1.343(11)
C(1A)-H(1AB)	0.9900	F(22)-C(27)	1.325(11)
C(2A)-C(3A)	1.511(13)	F(23)-C(27)	1.344(11)
C(2A)-H(2AA)	0.9900	F(24)-C(28)	1.316(11)
C(2A)-H(2AC)	0.9900	F(25)-C(28)	1.297(11)
C(3A)-H(3AA)	1.0000	F(26)-C(28)	1.304(11)
C(4A)-D(4AA)	0.9800	F(31)-C(37)	1.345(11)
C(4A)-D(4AB)	0.9800	F(32)-C(37)	1.337(10)
C(4A)-D(4AC)	0.9800	F(33)-C(37)	1.334(10)
C(5A)-C(6A)	1.479(13)	F(34)-C(38)	1.332(10)
C(5A)-H(5AA)	0.9900	F(35)-C(38)	1.328(11)
C(5A)-H(5AC)	0.9900	F(36)-C(38)	1.337(11)
C(6A)-H(6AA)	0.9900	F(41)-C(47)	1.330(14)
C(6A)-H(6AC)	0.9900	F(42)-C(47)	1.337(13)

F(43)-C(47)	1.317(14)
F(44)-C(48)	1.319(11)
F(45)-C(48)	1.330(11)
F(46)-C(48)	1.292(11)
F(51)-C(57)	1.313(11)
F(52)-C(57)	1.331(11)
F(53)-C(57)	1.319(11)
F(54)-C(58)	1.309(11)
F(55)-C(58)	1.314(11)
F(56)-C(58)	1.299(11)
C(21)-C(22)	1.391(10)
C(21)-C(26)	1.415(10)
C(21)-B(21)	1.651(9)
C(22)-C(23)	1.398(10)
C(22)-H(22)	0.9500
C(23)-C(24)	1.378(10)
C(23)-C(27)	1.490(10)
C(24)-C(25)	1.387(10)
C(24)-H(24)	0.9500
C(25)-C(26)	1.388(10)
C(25)-C(28)	1.495(10)
C(26)-H(26)	0.9500
C(31)-C(32)	1.397(10)
C(31)-C(36)	1.405(10)
C(31)-B(21)	1.644(9)
C(32)-C(33)	1.395(10)
C(32)-H(32)	0.9500
C(33)-C(34)	1.379(10)
C(33)-C(37)	1.496(10)
C(34)-C(35)	1.391(10)
C(34)-H(34)	0.9500
C(35)-C(36)	1.388(10)
C(35)-C(38)	1.491(10)
C(36)-H(36)	0.9500
C(41)-C(42)	1.400(10)
C(41)-C(46)	1.401(10)
C(41)-B(21)	1.638(9)
C(42)-C(43)	1.400(10)
C(42)-H(42)	0.9500
C(43)-C(44)	1.387(10)
C(43)-C(47)	1.489(10)
C(44)-C(45)	1.379(10)
C(44)-H(44)	0.9500
C(45)-C(46)	1.394(10)
C(45)-C(48)	1.506(10)
C(46)-H(46)	0.9500
C(51)-C(56)	1.396(10)
C(51)-C(52)	1.403(10)
C(51)-B(21)	1.635(10)
C(52)-C(53)	1.388(10)
C(52)-H(52)	0.9500
C(53)-C(54)	1.383(10)
C(53)-C(57)	1.492(10)
C(54)-C(55)	1.376(10)
C(54)-H(54)	0.9500
C(55)-C(56)	1.387(10)
C(55)-C(58)	1.504(10)
C(56)-H(56)	0.9500
F(21A)-C(27A)	1.340(12)
F(22A)-C(27A)	1.319(12)
F(23A)-C(27A)	1.327(12)
F(24A)-C(28A)	1.318(12)
F(25A)-C(28A)	1.320(12)
F(26A)-C(28A)	1.323(12)
F(31A)-C(37A)	1.349(12)
F(32A)-C(37A)	1.331(11)
F(33A)-C(37A)	1.333(11)
F(34A)-C(38A)	1.319(12)
F(35A)-C(38A)	1.328(12)
F(36A)-C(38A)	1.330(12)
F(41A)-C(47A)	1.316(15)
F(42A)-C(47A)	1.329(15)
F(43A)-C(47A)	1.298(16)
F(44A)-C(48A)	1.330(12)
F(45A)-C(48A)	1.329(12)
F(46A)-C(48A)	1.297(13)
F(51A)-C(57A)	1.319(12)
F(52A)-C(57A)	1.340(12)
F(53A)-C(57A)	1.335(12)
F(54A)-C(58A)	1.312(11)
F(55A)-C(58A)	1.319(11)
F(56A)-C(58A)	1.314(11)
C(21A)-C(22A)	1.388(11)
C(21A)-C(26A)	1.408(11)
C(21A)-B(21A)	1.643(10)
C(22A)-C(23A)	1.393(11)
C(22A)-H(22A)	0.9500
C(23A)-C(24A)	1.376(11)
C(23A)-C(27A)	1.494(11)
C(24A)-C(25A)	1.402(11)
C(24A)-H(24A)	0.9500
C(25A)-C(26A)	1.393(11)
C(25A)-C(28A)	1.493(11)
C(26A)-H(26A)	0.9500
C(31A)-C(32A)	1.395(11)
C(31A)-C(36A)	1.398(11)
C(31A)-B(21A)	1.645(10)
C(32A)-C(33A)	1.389(11)
C(32A)-H(32A)	0.9500
C(33A)-C(34A)	1.378(11)
C(33A)-C(37A)	1.499(11)
C(34A)-C(35A)	1.389(11)
C(34A)-H(34A)	0.9500
C(35A)-C(36A)	1.385(11)
C(35A)-C(38A)	1.489(11)
C(36A)-H(36A)	0.9500
C(41A)-C(46A)	1.399(11)
C(41A)-C(42A)	1.404(11)
C(41A)-B(21A)	1.635(10)
C(42A)-C(43A)	1.396(11)
C(42A)-H(42A)	0.9500
C(43A)-C(44A)	1.377(11)
C(43A)-C(47A)	1.492(11)
C(44A)-C(45A)	1.376(11)
C(44A)-H(44A)	0.9500
C(45A)-C(46A)	1.399(11)
C(45A)-C(48A)	1.501(11)
C(46A)-H(46A)	0.9500
C(51A)-C(56A)	1.399(11)
C(51A)-C(52A)	1.401(11)
C(51A)-B(21A)	1.638(10)
C(52A)-C(53A)	1.389(11)
C(52A)-H(52A)	0.9500
C(53A)-C(54A)	1.396(11)
C(53A)-C(57A)	1.483(11)
C(54A)-C(55A)	1.375(11)
C(54A)-H(54A)	0.9500
C(55A)-C(56A)	1.398(11)
C(55A)-C(58A)	1.513(10)
C(56A)-H(56A)	0.9500
Cl(61)-C(61)	1.739(5)
Cl(62)-C(61)	1.746(5)
C(61)-H(61A)	0.9900
C(61)-H(61B)	0.9900
Cl(1C)-C(61A)	1.774(17)
Cl(2C)-C(61A)	1.716(17)
C(61A)-H(61C)	0.9900
C(61A)-H(61D)	0.9900
Cl(1D)-C(61B)	1.771(17)
Cl(2D)-C(61B)	1.722(17)
C(61B)-H(61E)	0.9900
C(61B)-H(61F)	0.9900
Cl(71)-C(71)	1.772(7)
Cl(72)-C(71)	1.734(8)
C(71)-H(71A)	0.9900
C(71)-H(71B)	0.9900
Cl(1A)-C(71A)	1.777(14)
Cl(2A)-C(71A)	1.736(14)
C(71A)-H(71C)	0.9900
C(71A)-H(71D)	0.9900
Cl(81)-C(81)	1.772(8)
Cl(82)-C(81)	1.695(9)
C(81)-H(81A)	0.9900
C(81)-H(81B)	0.9900
Cl(1B)-C(81A)	1.772(16)
Cl(2B)-C(81A)	1.705(16)
C(81A)-H(81C)	0.9900
C(81A)-H(81D)	0.9900
Co(1)-O(2)-H(2)	109.5
O(2)-Co(1)-O(1)	171.1(13)
O(2)-Co(1)-N(1)	98.5(13)
O(1)-Co(1)-N(1)	72.6(5)
O(2)-Co(1)-N(3)	91.0(14)
O(1)-Co(1)-N(3)	97.8(7)
N(1)-Co(1)-N(3)	170.5(8)
O(2)-Co(1)-N(4)	90.7(14)
O(1)-Co(1)-N(4)	88.0(7)
N(1)-Co(1)-N(4)	86.9(6)
N(3)-Co(1)-N(4)	93.1(6)
O(2)-Co(1)-N(2)	90.5(13)
O(1)-Co(1)-N(2)	91.0(6)
N(1)-Co(1)-N(2)	94.1(5)
N(3)-Co(1)-N(2)	85.7(6)
N(4)-Co(1)-N(2)	178.3(7)
O(2)-Co(1)-C(1)	134.2(13)
O(1)-Co(1)-C(1)	37.7(3)
N(1)-Co(1)-C(1)	38.5(3)
N(3)-Co(1)-C(1)	132.4(7)
N(4)-Co(1)-C(1)	99.3(6)
N(2)-Co(1)-C(1)	80.7(4)
C(1)-O(1)-Co(1)	91.1(6)
C(13)-N(1)-C(14)	102.2(9)
C(13)-N(1)-C(1)	115.7(11)
C(14)-N(1)-C(1)	116.5(9)
C(13)-N(1)-Co(1)	109.4(9)
C(14)-N(1)-Co(1)	126.6(10)
C(1)-N(1)-Co(1)	87.0(7)
C(4)-N(2)-C(5)	106.1(10)
C(4)-N(2)-C(3)	108.8(9)

C(5)-N(2)-C(3)	107.5(10)
C(4)-N(2)-Co(1)	118.6(11)
C(5)-N(2)-Co(1)	105.4(9)
C(3)-N(2)-Co(1)	109.9(7)
C(7)-N(3)-C(8)	108.4(13)
C(7)-N(3)-C(6)	107.4(13)
C(8)-N(3)-C(6)	106.2(10)
C(7)-N(3)-Co(1)	117.0(19)
C(8)-N(3)-Co(1)	114.4(12)
C(6)-N(3)-Co(1)	102.4(10)
C(12)-N(4)-C(11)	111.2(11)
C(12)-N(4)-C(10)	105.1(10)
C(11)-N(4)-C(10)	107.8(11)
C(12)-N(4)-Co(1)	104.8(9)
C(11)-N(4)-Co(1)	112.9(14)
C(10)-N(4)-Co(1)	114.8(11)
O(1)-C(1)-N(1)	99.7(8)
O(1)-C(1)-C(2)	111.2(9)
N(1)-C(1)-C(2)	111.6(9)
O(1)-C(1)-Co(1)	51.2(6)
N(1)-C(1)-Co(1)	54.5(6)
C(2)-C(1)-Co(1)	104.0(6)
O(1)-C(1)-H(1)	111.3
N(1)-C(1)-H(1)	111.3
C(2)-C(1)-H(1)	111.3
Co(1)-C(1)-H(1)	144.6
C(3)-C(2)-C(1)	111.2(8)
C(3)-C(2)-H(2A)	109.4
C(1)-C(2)-H(2A)	109.4
C(3)-C(2)-H(2AB)	109.4
C(1)-C(2)-H(2AB)	109.4
H(2A)-C(2)-H(2AB)	108.0
N(2)-C(3)-C(2)	113.5(8)
N(2)-C(3)-H(3A)	108.9
C(2)-C(3)-H(3A)	108.9
N(2)-C(3)-H(3AB)	108.9
C(2)-C(3)-H(3AB)	108.9
H(3A)-C(3)-H(3AB)	107.7
N(2)-C(4)-D(4A)	109.5
N(2)-C(4)-D(4B)	109.5
D(4A)-C(4)-D(4B)	109.5
N(2)-C(4)-D(4C)	109.5
D(4A)-C(4)-D(4C)	109.5
D(4B)-C(4)-D(4C)	109.5
C(6)-C(5)-N(2)	109.6(10)
C(6)-C(5)-H(5A)	109.7
N(2)-C(5)-H(5A)	109.7
C(6)-C(5)-H(5AB)	109.7
N(2)-C(5)-H(5AB)	109.7
H(5A)-C(5)-H(5AB)	108.2
C(5)-C(6)-N(3)	107.4(10)
C(5)-C(6)-H(6A)	110.2
N(3)-C(6)-H(6A)	110.2
C(5)-C(6)-H(6AB)	110.2
N(3)-C(6)-H(6AB)	110.2
H(6A)-C(6)-H(6AB)	108.5
N(3)-C(7)-D(7A)	109.5
N(3)-C(7)-D(7B)	109.5
D(7A)-C(7)-D(7B)	109.5
N(3)-C(7)-D(7C)	109.5
D(7A)-C(7)-D(7C)	109.5
D(7B)-C(7)-D(7C)	109.5
C(9)-C(8)-N(3)	116.0(11)
C(9)-C(8)-H(8A)	108.3
N(3)-C(8)-H(8A)	108.3
C(9)-C(8)-H(8AB)	108.3
N(3)-C(8)-H(8AB)	108.3
H(8A)-C(8)-H(8AB)	107.4
C(8)-C(9)-C(10)	111.4(11)
C(8)-C(9)-H(9A)	109.4
C(10)-C(9)-H(9A)	109.4
C(8)-C(9)-H(9AB)	109.4
C(10)-C(9)-H(9AB)	109.4
H(9A)-C(9)-H(9AB)	108.0
N(4)-C(10)-C(9)	113.5(11)
N(4)-C(10)-H(10A)	108.9
C(9)-C(10)-H(10A)	108.9
N(4)-C(10)-H(10B)	108.9
C(9)-C(10)-H(10B)	108.9
H(10A)-C(10)-H(10B)	107.7
N(4)-C(11)-D(11A)	109.5
N(4)-C(11)-D(11B)	109.5
D(11A)-C(11)-D(11B)	109.5
N(4)-C(11)-D(11C)	109.5
D(11A)-C(11)-D(11C)	109.5
D(11B)-C(11)-D(11C)	109.5
C(13)-C(12)-N(4)	112.5(11)
C(13)-C(12)-H(12A)	109.1
N(4)-C(12)-H(12A)	109.1
C(13)-C(12)-H(12B)	109.1
N(4)-C(12)-H(12B)	109.1
H(12A)-C(12)-H(12B)	107.8
C(12)-C(13)-N(1)	111.6(11)
C(12)-C(13)-H(13A)	109.3
N(1)-C(13)-H(13A)	109.3
C(12)-C(13)-H(13B)	109.3
N(1)-C(13)-H(13B)	109.3
H(13A)-C(13)-H(13B)	108.0
N(1)-C(14)-D(14A)	109.5
N(1)-C(14)-D(14B)	109.5
D(14A)-C(14)-D(14B)	109.5
N(1)-C(14)-D(14C)	109.5
D(14A)-C(14)-D(14C)	109.5
D(14B)-C(14)-D(14C)	109.5
Co(1A)-O(2A)-H(2BA)	109.5
O(2A)-Co(1A)-N(2A)	101.1(16)
O(2A)-Co(1A)-O(1A)	167.8(17)
N(2A)-Co(1A)-O(1A)	66.7(8)
O(2A)-Co(1A)-N(1A)	96.6(16)
N(2A)-Co(1A)-N(1A)	93.1(11)
O(1A)-Co(1A)-N(1A)	84.9(10)
O(2A)-Co(1A)-N(4A)	103.4(19)
N(2A)-Co(1A)-N(4A)	155.4(15)
O(1A)-Co(1A)-N(4A)	88.8(13)
N(1A)-Co(1A)-N(4A)	86.1(12)
O(2A)-Co(1A)-N(3A)	104.3(17)
N(2A)-Co(1A)-N(3A)	82.3(12)
O(1A)-Co(1A)-N(3A)	74.6(13)
N(1A)-Co(1A)-N(3A)	159.1(15)
N(4A)-Co(1A)-N(3A)	89.8(13)
C(3A)-O(1A)-Co(1A)	93.3(11)
C(14A)-N(1A)-C(1A)	107.7(15)
C(14A)-N(1A)-C(13A)	105.1(15)
C(1A)-N(1A)-C(13A)	111.5(16)
C(14A)-N(1A)-Co(1A)	114(2)
C(1A)-N(1A)-Co(1A)	115.5(15)
C(13A)-N(1A)-Co(1A)	102.8(16)
C(4A)-N(2A)-C(3A)	116.2(15)
C(4A)-N(2A)-C(5A)	104.3(14)
C(3A)-N(2A)-C(5A)	111.6(16)
C(4A)-N(2A)-Co(1A)	120.8(19)
C(3A)-N(2A)-Co(1A)	93.0(12)
C(5A)-N(2A)-Co(1A)	110.9(17)
C(7A)-N(3A)-C(8A)	108.7(18)
C(7A)-N(3A)-C(6A)	108.0(18)
C(8A)-N(3A)-C(6A)	104.6(16)
C(7A)-N(3A)-Co(1A)	105(3)
C(8A)-N(3A)-Co(1A)	119(2)
C(6A)-N(3A)-Co(1A)	111.0(17)
C(11A)-N(4A)-C(10A)	108.8(18)
C(11A)-N(4A)-C(12A)	107.2(17)
C(10A)-N(4A)-C(12A)	106.7(16)
C(11A)-N(4A)-Co(1A)	104(3)
C(10A)-N(4A)-Co(1A)	125(2)
C(12A)-N(4A)-Co(1A)	104.2(19)
C(2A)-C(1A)-N(1A)	115.2(15)
C(2A)-C(1A)-H(1AA)	108.5
N(1A)-C(1A)-H(1AA)	108.5
C(2A)-C(1A)-H(1AB)	108.5
N(1A)-C(1A)-H(1AB)	108.5
H(1AA)-C(1A)-H(1AB)	107.5
C(1A)-C(2A)-C(3A)	115.4(14)
C(1A)-C(2A)-H(2AA)	108.4
C(3A)-C(2A)-H(2AA)	108.4
C(1A)-C(2A)-H(2AC)	108.4
C(3A)-C(2A)-H(2AC)	108.4
H(2AA)-C(2A)-H(2AC)	107.5
O(1A)-C(3A)-N(2A)	97.6(13)
O(1A)-C(3A)-C(2A)	106.7(14)
N(2A)-C(3A)-C(2A)	114.8(14)
O(1A)-C(3A)-H(3AA)	112.2
N(2A)-C(3A)-H(3AA)	112.2
C(2A)-C(3A)-H(3AA)	112.2
N(2A)-C(4A)-D(4AA)	109.5
N(2A)-C(4A)-D(4AB)	109.5
D(4AA)-C(4A)-D(4AB)	109.5
N(2A)-C(4A)-D(4AC)	109.5
D(4AA)-C(4A)-D(4AC)	109.5
D(4AB)-C(4A)-D(4AC)	109.5
C(6A)-C(5A)-N(2A)	110.7(16)
C(6A)-C(5A)-H(5AA)	109.5
N(2A)-C(5A)-H(5AA)	109.5
C(6A)-C(5A)-H(5AC)	109.5
N(2A)-C(5A)-H(5AC)	109.5
H(5AA)-C(5A)-H(5AC)	108.1
C(5A)-C(6A)-N(3A)	109.9(16)
C(5A)-C(6A)-H(6AA)	109.7
N(3A)-C(6A)-H(6AA)	109.7
C(5A)-C(6A)-H(6AC)	109.7
N(3A)-C(6A)-H(6AC)	109.7
H(6AA)-C(6A)-H(6AC)	108.2
N(3A)-C(7A)-D(7AA)	109.5
N(3A)-C(7A)-D(7AB)	109.5
D(7AA)-C(7A)-D(7AB)	109.5
N(3A)-C(7A)-D(7AC)	109.5
D(7AA)-C(7A)-D(7AC)	109.5
D(7AB)-C(7A)-D(7AC)	109.5
C(9A)-C(8A)-N(3A)	113.2(18)
C(9A)-C(8A)-H(8AA)	108.9
N(3A)-C(8A)-H(8AA)	108.9

C(9A)-C(8A)-H(8AC)	108.9	N(2B)-C(4B)-D(4BA)	109.5
N(3A)-C(8A)-H(8AC)	108.9	N(2B)-C(4B)-D(4BB)	109.5
H(8AA)-C(8A)-H(8AC)	107.7	D(4BA)-C(4B)-D(4BB)	109.5
C(8A)-C(9A)-C(10A)	113.5(19)	N(2B)-C(4B)-D(4BC)	109.5
C(8A)-C(9A)-H(9AA)	123.2	D(4BA)-C(4B)-D(4BC)	109.5
C(10A)-C(9A)-H(9AA)	123.2	D(4BB)-C(4B)-D(4BC)	109.5
C(9A)-C(10A)-N(4A)	115.2(19)	C(6B)-C(5B)-N(2B)	108.7(12)
C(9A)-C(10A)-H(10C)	108.5	C(6B)-C(5B)-H(5BA)	110.0
N(4A)-C(10A)-H(10C)	108.5	N(2B)-C(5B)-H(5BA)	110.0
C(9A)-C(10A)-H(10D)	108.5	C(6B)-C(5B)-H(5BB)	110.0
N(4A)-C(10A)-H(10D)	108.5	N(2B)-C(5B)-H(5BB)	110.0
H(10C)-C(10A)-H(10D)	107.5	H(5BA)-C(5B)-H(5BB)	108.3
N(4A)-C(11A)-D(11D)	109.5	C(5B)-C(6B)-N(3B)	114.4(14)
N(4A)-C(11A)-D(11E)	109.5	C(5B)-C(6B)-H(6BA)	108.6
D(11D)-C(11A)-D(11E)	109.5	N(3B)-C(6B)-H(6BA)	108.6
N(4A)-C(11A)-D(11F)	109.5	C(5B)-C(6B)-H(6BB)	108.6
D(11D)-C(11A)-D(11F)	109.5	N(3B)-C(6B)-H(6BB)	108.6
D(11E)-C(11A)-D(11F)	109.5	H(6BA)-C(6B)-H(6BB)	107.6
C(13A)-C(12A)-N(4A)	106.8(16)	N(3B)-C(7B)-D(7BA)	109.5
C(13A)-C(12A)-H(12C)	110.4	N(3B)-C(7B)-D(7BB)	109.5
N(4A)-C(12A)-H(12C)	110.4	D(7BA)-C(7B)-D(7BB)	109.5
C(13A)-C(12A)-H(12D)	110.4	N(3B)-C(7B)-D(7BC)	109.5
N(4A)-C(12A)-H(12D)	110.4	D(7BA)-C(7B)-D(7BC)	109.5
H(12C)-C(12A)-H(12D)	108.6	D(7BB)-C(7B)-D(7BC)	109.5
C(12A)-C(13A)-N(1A)	108.9(16)	N(3B)-C(8B)-C(9B)	114.5(16)
C(12A)-C(13A)-H(13C)	109.9	N(3B)-C(8B)-H(8BA)	108.6
N(1A)-C(13A)-H(13C)	109.9	C(9B)-C(8B)-H(8BA)	108.6
C(12A)-C(13A)-H(13D)	109.9	N(3B)-C(8B)-H(8BB)	108.6
N(1A)-C(13A)-H(13D)	109.9	C(9B)-C(8B)-H(8BB)	108.6
H(13C)-C(13A)-H(13D)	108.3	H(8BA)-C(8B)-H(8BB)	107.6
N(1A)-C(14A)-D(14D)	109.5	C(10B)-C(9B)-C(8B)	115.3(17)
N(1A)-C(14A)-D(14E)	109.5	C(10B)-C(9B)-H(9BA)	108.5
D(14D)-C(14A)-D(14E)	109.5	C(8B)-C(9B)-H(9BA)	108.5
N(1A)-C(14A)-D(14F)	109.5	C(10B)-C(9B)-H(9BB)	108.5
D(14D)-C(14A)-D(14F)	109.5	C(8B)-C(9B)-H(9BB)	108.5
D(14E)-C(14A)-D(14F)	109.5	H(9BA)-C(9B)-H(9BB)	107.5
Co(1B)-O(2B)-H(2CA)	109.5	C(9B)-C(10B)-N(4B)	116.1(18)
O(2B)-Co(1B)-O(1B)	172(3)	C(9B)-C(10B)-H(10E)	108.3
O(2B)-Co(1B)-N(3B)	86(3)	N(4B)-C(10B)-H(10E)	108.3
O(1B)-Co(1B)-N(3B)	98.4(15)	C(9B)-C(10B)-H(10F)	108.3
O(2B)-Co(1B)-N(2B)	99(3)	N(4B)-C(10B)-H(10F)	108.3
O(1B)-Co(1B)-N(2B)	87.0(14)	H(10E)-C(10B)-H(10F)	107.4
N(3B)-Co(1B)-N(2B)	89.9(10)	N(4B)-C(11B)-D(11G)	109.5
O(2B)-Co(1B)-N(4B)	91(3)	N(4B)-C(11B)-D(11H)	109.5
O(1B)-Co(1B)-N(4B)	82.7(18)	D(11G)-C(11B)-D(11H)	109.5
N(3B)-Co(1B)-N(4B)	97.1(12)	N(4B)-C(11B)-D(11I)	109.5
N(2B)-Co(1B)-N(4B)	168.3(16)	D(11G)-C(11B)-D(11I)	109.5
O(2B)-Co(1B)-N(1B)	86(3)	D(11H)-C(11B)-D(11I)	109.5
O(1B)-Co(1B)-N(1B)	89.6(15)	C(13B)-C(12B)-N(4B)	113.0(17)
N(3B)-Co(1B)-N(1B)	172.0(15)	C(13B)-C(12B)-H(12E)	109.0
N(2B)-Co(1B)-N(1B)	89.9(9)	N(4B)-C(12B)-H(12E)	109.0
N(4B)-Co(1B)-N(1B)	84.5(12)	C(13B)-C(12B)-H(12F)	109.0
Co(1B)-O(1B)-H(1B)	109.5	N(4B)-C(12B)-H(12F)	109.0
C(14B)-N(1B)-C(1B)	111.1(17)	H(12E)-C(12B)-H(12F)	107.8
C(14B)-N(1B)-C(13B)	104.6(16)	C(12B)-C(13B)-N(1B)	113.9(18)
C(1B)-N(1B)-C(13B)	107.3(18)	C(12B)-C(13B)-H(13E)	108.8
C(14B)-N(1B)-Co(1B)	118(2)	N(1B)-C(13B)-H(13E)	108.8
C(1B)-N(1B)-Co(1B)	109.9(17)	C(12B)-C(13B)-H(13F)	108.8
C(13B)-N(1B)-Co(1B)	104.9(16)	N(1B)-C(13B)-H(13F)	108.8
C(3B)-N(2B)-C(4B)	110.0(17)	H(13E)-C(13B)-H(13F)	107.7
C(3B)-N(2B)-C(5B)	104.6(13)	N(1B)-C(14B)-D(14G)	109.5
C(4B)-N(2B)-C(5B)	106.0(16)	N(1B)-C(14B)-D(14H)	109.5
C(3B)-N(2B)-Co(1B)	113.5(14)	D(14G)-C(14B)-D(14H)	109.5
C(4B)-N(2B)-Co(1B)	121(2)	N(1B)-C(14B)-D(14I)	109.5
C(5B)-N(2B)-Co(1B)	99.4(12)	D(14G)-C(14B)-D(14I)	109.5
C(8B)-N(3B)-C(6B)	110.2(16)	D(14H)-C(14B)-D(14I)	109.5
C(8B)-N(3B)-C(7B)	108.1(17)	C(22)-C(21)-C(26)	114.4(10)
C(6B)-N(3B)-C(7B)	108.9(18)	C(22)-C(21)-B(21)	124.6(10)
C(8B)-N(3B)-Co(1B)	109.0(18)	C(26)-C(21)-B(21)	120.9(9)
C(6B)-N(3B)-Co(1B)	103.6(15)	C(21)-C(22)-C(23)	124.2(11)
C(7B)-N(3B)-Co(1B)	117(2)	C(21)-C(22)-H(22)	117.9
C(11B)-N(4B)-C(12B)	110.0(19)	C(23)-C(22)-H(22)	117.9
C(11B)-N(4B)-C(10B)	110.9(18)	C(24)-C(23)-C(22)	119.9(10)
C(12B)-N(4B)-C(10B)	106.3(17)	C(24)-C(23)-C(27)	119.1(10)
C(11B)-N(4B)-Co(1B)	118(3)	C(22)-C(23)-C(27)	120.9(10)
C(12B)-N(4B)-Co(1B)	104(2)	C(23)-C(24)-C(25)	117.3(11)
C(10B)-N(4B)-Co(1B)	107(2)	C(23)-C(24)-H(24)	121.3
C(2B)-C(1B)-N(1B)	115.3(16)	C(25)-C(24)-H(24)	121.3
C(2B)-C(1B)-H(1BA)	108.4	C(24)-C(25)-C(26)	122.3(10)
N(1B)-C(1B)-H(1BA)	108.4	C(24)-C(25)-C(28)	116.7(10)
C(2B)-C(1B)-H(1BB)	108.4	C(26)-C(25)-C(28)	120.6(10)
N(1B)-C(1B)-H(1BB)	108.4	C(25)-C(26)-C(21)	121.4(11)
H(1BA)-C(1B)-H(1BB)	107.5	C(25)-C(26)-H(26)	119.3
C(3B)-C(2B)-C(1B)	114.5(15)	C(21)-C(26)-H(26)	119.3
C(3B)-C(2B)-H(2CB)	108.6	F(22)-C(27)-F(21)	106.0(12)
C(1B)-C(2B)-H(2CB)	108.6	F(22)-C(27)-F(23)	104.3(11)
C(3B)-C(2B)-H(2CC)	108.6	F(21)-C(27)-F(23)	105.5(11)
C(1B)-C(2B)-H(2CC)	108.6	F(22)-C(27)-C(23)	113.1(12)
H(2CB)-C(2B)-H(2CC)	107.6	F(21)-C(27)-C(23)	112.3(12)
C(2B)-C(3B)-N(2B)	115.9(12)	F(23)-C(27)-C(23)	114.8(10)
C(2B)-C(3B)-H(3BA)	108.3	F(25)-C(28)-F(26)	105.6(9)
N(2B)-C(3B)-H(3BA)	108.3	F(25)-C(28)-F(24)	106.3(10)
C(2B)-C(3B)-H(3BB)	108.3	F(26)-C(28)-F(24)	106.7(10)
N(2B)-C(3B)-H(3BB)	108.3	F(25)-C(28)-C(25)	110.9(11)
H(3BA)-C(3B)-H(3BB)	107.4	F(26)-C(28)-C(25)	112.2(12)

F(24)-C(28)-C(25)	114.6(10)
C(32)-C(31)-C(36)	115.3(10)
C(32)-C(31)-B(21)	125.4(10)
C(36)-C(31)-B(21)	119.2(9)
C(33)-C(32)-C(31)	122.2(11)
C(33)-C(32)-H(32)	118.9
C(31)-C(32)-H(32)	118.9
C(34)-C(33)-C(32)	121.1(11)
C(34)-C(33)-C(37)	119.1(10)
C(32)-C(33)-C(37)	119.7(10)
C(33)-C(34)-C(35)	118.2(11)
C(33)-C(34)-H(34)	120.9
C(35)-C(34)-H(34)	120.9
C(36)-C(35)-C(34)	120.2(10)
C(36)-C(35)-C(38)	120.6(10)
C(34)-C(35)-C(38)	119.2(10)
C(35)-C(36)-C(31)	122.9(11)
C(35)-C(36)-H(36)	118.6
C(31)-C(36)-H(36)	118.6
F(33)-C(37)-F(32)	105.2(9)
F(33)-C(37)-F(31)	107.1(13)
F(32)-C(37)-F(31)	104.5(13)
F(33)-C(37)-C(33)	112.8(11)
F(32)-C(37)-C(33)	113.4(11)
F(31)-C(37)-C(33)	113.1(13)
F(35)-C(38)-F(34)	105.7(11)
F(35)-C(38)-F(36)	106.0(11)
F(34)-C(38)-F(36)	105.5(12)
F(35)-C(38)-C(35)	112.2(12)
F(34)-C(38)-C(35)	114.4(12)
F(36)-C(38)-C(35)	112.5(12)
C(42)-C(41)-C(46)	115.8(10)
C(42)-C(41)-B(21)	122.0(10)
C(46)-C(41)-B(21)	122.1(10)
C(43)-C(42)-C(41)	123.1(11)
C(43)-C(42)-H(42)	118.4
C(41)-C(42)-H(42)	118.4
C(44)-C(43)-C(42)	119.5(10)
C(44)-C(43)-C(47)	120.9(10)
C(42)-C(43)-C(47)	119.6(11)
C(45)-C(44)-C(43)	118.5(11)
C(45)-C(44)-H(44)	120.8
C(43)-C(44)-H(44)	120.8
C(44)-C(45)-C(46)	121.9(10)
C(44)-C(45)-C(48)	119.2(10)
C(46)-C(45)-C(48)	118.9(10)
C(45)-C(46)-C(41)	121.1(11)
C(45)-C(46)-H(46)	119.4
C(41)-C(46)-H(46)	119.4
F(43)-C(47)-F(41)	105.2(11)
F(43)-C(47)-F(42)	105.6(10)
F(41)-C(47)-F(42)	102.1(11)
F(43)-C(47)-C(43)	114.9(13)
F(41)-C(47)-C(43)	115.5(12)
F(42)-C(47)-C(43)	112.3(14)
F(46)-C(48)-F(44)	107.1(9)
F(46)-C(48)-F(45)	106.0(9)
F(44)-C(48)-F(45)	105.0(9)
F(46)-C(48)-C(45)	114.0(9)
F(44)-C(48)-C(45)	112.0(10)
F(45)-C(48)-C(45)	112.1(11)
C(56)-C(51)-C(52)	115.8(10)
C(56)-C(51)-B(21)	122.2(10)
C(52)-C(51)-B(21)	122.0(10)
C(53)-C(52)-C(51)	121.6(11)
C(53)-C(52)-H(52)	119.2
C(51)-C(52)-H(52)	119.2
C(54)-C(53)-C(52)	121.4(11)
C(54)-C(53)-C(57)	118.3(10)
C(52)-C(53)-C(57)	120.3(10)
C(55)-C(54)-C(53)	117.6(11)
C(55)-C(54)-H(54)	121.2
C(53)-C(54)-H(54)	121.2
C(54)-C(55)-C(56)	121.4(10)
C(54)-C(55)-C(58)	120.2(10)
C(56)-C(55)-C(58)	118.4(10)
C(55)-C(56)-C(51)	122.0(11)
C(55)-C(56)-H(56)	119.0
C(51)-C(56)-H(56)	119.0
F(51)-C(57)-F(53)	106.2(11)
F(51)-C(57)-F(52)	107.8(12)
F(53)-C(57)-F(52)	107.8(13)
F(51)-C(57)-C(53)	110.9(12)
F(53)-C(57)-C(53)	112.8(11)
F(52)-C(57)-C(53)	111.0(12)
F(56)-C(58)-F(54)	111.7(11)
F(56)-C(58)-F(55)	103.7(10)
F(54)-C(58)-F(55)	102.9(10)
F(56)-C(58)-C(55)	110.6(11)
F(54)-C(58)-C(55)	115.6(10)
F(55)-C(58)-C(55)	111.4(10)
C(51)-B(21)-C(41)	106.3(5)
C(51)-B(21)-C(31)	112.5(8)
C(41)-B(21)-C(31)	110.9(7)
C(51)-B(21)-C(21)	111.7(8)
C(41)-B(21)-C(21)	112.1(8)
C(31)-B(21)-C(21)	103.4(5)
C(22A)-C(21A)-C(26A)	116.8(11)
C(22A)-C(21A)-B(21A)	124.4(11)
C(26A)-C(21A)-B(21A)	118.8(10)
C(21A)-C(22A)-C(23A)	120.4(12)
C(21A)-C(22A)-H(22A)	119.8
C(23A)-C(22A)-H(22A)	119.8
C(24A)-C(23A)-C(22A)	122.4(11)
C(24A)-C(23A)-C(27A)	121.8(11)
C(22A)-C(23A)-C(27A)	115.8(11)
C(23A)-C(24A)-C(25A)	118.5(12)
C(23A)-C(24A)-H(24A)	120.7
C(25A)-C(24A)-H(24A)	120.7
C(26A)-C(25A)-C(24A)	118.6(11)
C(26A)-C(25A)-C(28A)	117.5(11)
C(24A)-C(25A)-C(28A)	123.4(12)
C(25A)-C(26A)-C(21A)	122.9(12)
C(25A)-C(26A)-H(26A)	118.6
C(21A)-C(26A)-H(26A)	118.6
F(22A)-C(27A)-F(23A)	106.8(11)
F(22A)-C(27A)-F(21A)	105.7(13)
F(23A)-C(27A)-F(21A)	106.2(13)
F(22A)-C(27A)-C(23A)	112.9(13)
F(23A)-C(27A)-C(23A)	111.5(12)
F(21A)-C(27A)-C(23A)	113.2(13)
F(24A)-C(28A)-F(25A)	104.5(10)
F(24A)-C(28A)-F(26A)	105.1(11)
F(25A)-C(28A)-F(26A)	104.0(10)
F(24A)-C(28A)-C(25A)	112.6(12)
F(25A)-C(28A)-C(25A)	118.0(11)
F(26A)-C(28A)-C(25A)	111.5(13)
C(32A)-C(31A)-C(36A)	116.0(11)
C(32A)-C(31A)-B(21A)	123.0(11)
C(36A)-C(31A)-B(21A)	120.9(11)
C(33A)-C(32A)-C(31A)	121.7(12)
C(33A)-C(32A)-H(32A)	119.2
C(31A)-C(32A)-H(32A)	119.2
C(34A)-C(33A)-C(32A)	121.6(12)
C(34A)-C(33A)-C(37A)	119.6(11)
C(32A)-C(33A)-C(37A)	118.7(12)
C(33A)-C(34A)-C(35A)	117.5(12)
C(33A)-C(34A)-H(34A)	121.2
C(35A)-C(34A)-H(34A)	121.2
C(36A)-C(35A)-C(34A)	121.0(12)
C(36A)-C(35A)-C(38A)	118.2(11)
C(34A)-C(35A)-C(38A)	120.7(12)
C(35A)-C(36A)-C(31A)	122.1(12)
C(35A)-C(36A)-H(36A)	118.9
C(31A)-C(36A)-H(36A)	118.9
F(32A)-C(37A)-F(33A)	108.1(11)
F(32A)-C(37A)-F(31A)	106.7(14)
F(33A)-C(37A)-F(31A)	105.0(14)
F(32A)-C(37A)-C(33A)	111.5(12)
F(33A)-C(37A)-C(33A)	112.2(12)
F(31A)-C(37A)-C(33A)	112.8(14)
F(34A)-C(38A)-F(35A)	106.5(13)
F(34A)-C(38A)-F(36A)	106.9(14)
F(35A)-C(38A)-F(36A)	104.7(12)
F(34A)-C(38A)-C(35A)	112.2(14)
F(35A)-C(38A)-C(35A)	111.7(14)
F(36A)-C(38A)-C(35A)	114.2(12)
C(46A)-C(41A)-C(42A)	116.0(11)
C(46A)-C(41A)-B(21A)	122.2(11)
C(42A)-C(41A)-B(21A)	121.6(11)
C(43A)-C(42A)-C(41A)	120.3(12)
C(43A)-C(42A)-H(42A)	119.8
C(41A)-C(42A)-H(42A)	119.8
C(44A)-C(43A)-C(42A)	122.7(12)
C(44A)-C(43A)-C(47A)	118.5(11)
C(42A)-C(43A)-C(47A)	118.6(12)
C(45A)-C(44A)-C(43A)	117.8(12)
C(45A)-C(44A)-H(44A)	121.1
C(43A)-C(44A)-H(44A)	121.1
C(44A)-C(45A)-C(46A)	120.2(11)
C(44A)-C(45A)-C(48A)	119.8(11)
C(46A)-C(45A)-C(48A)	119.9(11)
C(45A)-C(46A)-C(41A)	122.6(12)
C(45A)-C(46A)-H(46A)	118.7
C(41A)-C(46A)-H(46A)	118.7
F(43A)-C(47A)-F(41A)	106.5(14)
F(43A)-C(47A)-F(42A)	110.8(13)
F(41A)-C(47A)-F(42A)	102.5(11)
F(43A)-C(47A)-C(43A)	113.1(15)
F(41A)-C(47A)-C(43A)	111.2(16)
F(42A)-C(47A)-C(43A)	112.0(13)
F(46A)-C(48A)-F(45A)	107.1(10)
F(46A)-C(48A)-F(44A)	106.9(11)
F(45A)-C(48A)-F(44A)	102.8(10)
F(46A)-C(48A)-C(45A)	113.3(12)
F(45A)-C(48A)-C(45A)	113.0(11)
F(44A)-C(48A)-C(45A)	113.0(10)
C(56A)-C(51A)-C(52A)	115.3(11)
C(56A)-C(51A)-B(21A)	124.0(10)
C(52A)-C(51A)-B(21A)	120.7(10)

C(53A)-C(52A)-C(51A)	123.3(12)
C(53A)-C(52A)-H(52A)	118.4
C(51A)-C(52A)-H(52A)	118.4
C(52A)-C(53A)-C(54A)	119.3(11)
C(52A)-C(53A)-C(57A)	121.7(11)
C(54A)-C(53A)-C(57A)	119.0(11)
C(55A)-C(54A)-C(53A)	119.3(12)
C(55A)-C(54A)-H(54A)	120.4
C(53A)-C(54A)-H(54A)	120.4
C(54A)-C(55A)-C(56A)	120.2(11)
C(54A)-C(55A)-C(58A)	120.7(11)
C(56A)-C(55A)-C(58A)	118.8(11)
C(55A)-C(56A)-C(51A)	122.4(12)
C(55A)-C(56A)-H(56A)	118.8
C(51A)-C(56A)-H(56A)	118.8
F(51A)-C(57A)-F(53A)	104.9(13)
F(51A)-C(57A)-F(52A)	101.4(13)
F(53A)-C(57A)-F(52A)	103.5(13)
F(51A)-C(57A)-C(53A)	115.3(14)
F(53A)-C(57A)-C(53A)	115.3(12)
F(52A)-C(57A)-C(53A)	114.8(13)
F(54A)-C(58A)-F(56A)	105.2(10)
F(54A)-C(58A)-F(55A)	106.2(10)
F(56A)-C(58A)-F(55A)	106.5(8)
F(54A)-C(58A)-C(55A)	111.8(11)
F(56A)-C(58A)-C(55A)	111.0(11)
F(55A)-C(58A)-C(55A)	115.5(10)
C(41A)-B(21A)-C(51A)	105.2(6)
C(41A)-B(21A)-C(21A)	114.4(8)
C(51A)-B(21A)-C(21A)	111.2(8)
C(41A)-B(21A)-C(31A)	111.2(8)
C(51A)-B(21A)-C(31A)	112.3(8)
C(21A)-B(21A)-C(31A)	102.8(6)
Cl(61)-C(61)-Cl(62)	112.1(3)
Cl(61)-C(61)-H(61A)	109.2
Cl(62)-C(61)-H(61A)	109.2
Cl(61)-C(61)-H(61B)	109.2
Cl(62)-C(61)-H(61B)	109.2
H(61A)-C(61)-H(61B)	107.9
Cl(2C)-C(61A)-Cl(1C)	108.3(19)
Cl(2C)-C(61A)-H(61C)	110.0
Cl(1C)-C(61A)-H(61C)	110.0
Cl(2C)-C(61A)-H(61D)	110.0
Cl(1C)-C(61A)-H(61D)	110.0
H(61C)-C(61A)-H(61D)	108.4
Cl(2D)-C(61B)-Cl(1D)	108.6(14)
Cl(2D)-C(61B)-H(61E)	110.0
Cl(1D)-C(61B)-H(61E)	110.0
Cl(2D)-C(61B)-H(61F)	110.0
Cl(1D)-C(61B)-H(61F)	110.0
H(61E)-C(61B)-H(61F)	108.4
Cl(72)-C(71)-Cl(71)	112.5(5)
Cl(72)-C(71)-H(71A)	109.1
Cl(71)-C(71)-H(71A)	109.1
Cl(72)-C(71)-H(71B)	109.1
Cl(71)-C(71)-H(71B)	109.1
H(71A)-C(71)-H(71B)	107.8
Cl(2A)-C(71A)-Cl(1A)	109.4(10)
Cl(2A)-C(71A)-H(71C)	109.8
Cl(1A)-C(71A)-H(71C)	109.8
Cl(2A)-C(71A)-H(71D)	109.8
Cl(1A)-C(71A)-H(71D)	109.8
H(71C)-C(71A)-H(71D)	108.2
Cl(82)-C(81)-Cl(81)	116.0(6)
Cl(82)-C(81)-H(81A)	108.3
Cl(81)-C(81)-H(81A)	108.3
Cl(82)-C(81)-H(81B)	108.3
Cl(81)-C(81)-H(81B)	108.3
H(81A)-C(81)-H(81B)	107.4
Cl(2B)-C(81A)-Cl(1B)	108.2(14)
Cl(2B)-C(81A)-H(81C)	110.1
Cl(1B)-C(81A)-H(81C)	110.1
Cl(2B)-C(81A)-H(81D)	110.1
Cl(1B)-C(81A)-H(81D)	110.1
H(81C)-C(81A)-H(81D)	108.4

Table S77. Anisotropic displacement parameters ($Å^2$x 10^3) for **9b-B$_{Ar}$F**.
The anisotropic displacement factor exponent takes the form: $-2p^2[h^2 a^{*2}U^{11} + ... + 2 h k a^* b^* U^{12}]$

	U^{11}	U^{22}	U^{33}	U^{23}	U^{13}	U^{12}
O(2)	48(5)	25(4)	37(8)	-11(3)	6(5)	5(4)
Co(1)	15(1)	15(2)	21(1)	-2(1)	4(1)	4(1)
O(1)	50(4)	37(3)	39(4)	-12(3)	10(4)	-6(3)
N(1)	44(4)	45(4)	42(4)	0(3)	1(3)	-8(3)
N(2)	37(3)	26(4)	42(4)	-2(3)	0(3)	2(3)
N(3)	29(4)	45(4)	44(4)	-2(3)	-1(3)	-3(3)
N(4)	38(4)	41(5)	29(4)	1(3)	7(3)	0(3)
C(1)	55(4)	50(4)	49(4)	-11(3)	-3(3)	-5(3)
C(2)	66(5)	33(4)	63(6)	-1(4)	-6(4)	-14(4)
C(3)	48(4)	26(4)	43(4)	2(3)	8(3)	-5(3)
C(4)	45(6)	39(8)	32(5)	-3(5)	4(5)	6(6)
C(5)	42(4)	46(6)	46(4)	8(4)	-11(4)	-7(4)
C(6)	33(4)	59(4)	48(6)	-1(5)	-10(4)	5(4)
C(7)	60(13)	56(7)	63(9)	-12(7)	-18(8)	-13(7)
C(8)	36(4)	60(7)	58(5)	6(5)	9(4)	2(5)
C(9)	40(5)	65(6)	50(5)	13(4)	16(4)	0(5)
C(10)	52(5)	51(5)	34(4)	2(4)	13(4)	-2(5)
C(11)	83(9)	40(6)	48(11)	17(6)	10(7)	16(6)
C(12)	53(5)	65(6)	43(5)	5(5)	-7(4)	-2(5)
C(13)	48(5)	60(6)	45(5)	-9(4)	-9(4)	-4(4)
C(14)	34(4)	62(8)	64(6)	-13(7)	-1(4)	-3(6)
O(2A)	67(13)	33(9)	54(10)	-11(7)	13(9)	-1(7)
Co(1A)	34(3)	40(6)	45(4)	5(3)	-2(2)	0(3)
O(1A)	46(6)	71(6)	50(6)	-6(5)	0(6)	3(5)
N(1A)	44(5)	46(5)	37(5)	-10(5)	-2(5)	-5(5)
N(2A)	40(5)	36(5)	45(5)	-2(5)	0(4)	0(5)
N(3A)	36(5)	51(6)	45(6)	-1(5)	-1(5)	-2(5)
N(4A)	41(5)	43(6)	37(6)	-6(5)	-3(5)	-1(5)
C(1A)	40(6)	50(5)	48(6)	-21(5)	2(5)	-20(5)
C(2A)	62(6)	42(6)	56(7)	-12(5)	7(5)	-15(5)
C(3A)	57(5)	41(5)	55(5)	-8(4)	-1(4)	-2(4)
C(4A)	51(9)	37(12)	34(9)	3(7)	3(7)	-3(8)
C(5A)	42(5)	41(7)	48(6)	0(5)	-6(5)	-7(5)
C(6A)	38(6)	53(6)	42(7)	-6(6)	-8(5)	7(6)
C(7A)	31(14)	54(8)	56(12)	2(8)	0(11)	-9(10)
C(8A)	38(6)	61(8)	51(6)	8(6)	5(6)	0(6)
C(9A)	39(6)	61(8)	49(7)	6(6)	4(6)	1(6)
C(10A)	44(6)	53(7)	40(6)	1(6)	7(5)	3(6)
C(11A)	55(8)	42(5)	23(9)	-10(5)	-7(7)	1(5)
C(12A)	40(6)	48(7)	34(6)	-14(6)	-3(5)	7(6)
C(13A)	38(5)	58(7)	37(6)	-10(5)	-8(5)	7(6)
C(14A)	32(8)	28(11)	64(11)	21(9)	10(8)	-6(9)
O(2B)	48(5)	25(4)	37(8)	-11(3)	6(5)	5(4)
Co(1B)	52(5)	56(6)	43(4)	-1(3)	-9(3)	0(3)
O(1B)	87(13)	69(8)	61(11)	-14(9)	1(11)	8(11)
N(1B)	46(6)	48(5)	34(5)	-7(5)	-5(5)	-10(5)
N(2B)	41(5)	33(6)	45(6)	6(5)	6(5)	1(4)
N(3B)	31(5)	56(6)	44(6)	-2(5)	1(5)	1(5)
N(4B)	40(5)	44(6)	36(6)	-8(5)	-4(5)	-2(5)
C(1B)	50(7)	49(5)	49(6)	-14(5)	3(6)	-20(5)
C(2B)	51(6)	34(6)	53(6)	-13(5)	13(6)	-11(6)
C(3B)	56(6)	22(5)	49(7)	8(5)	10(5)	-2(5)
C(4B)	46(11)	21(13)	48(9)	0(8)	0(9)	5(10)
C(5B)	45(5)	57(5)	43(6)	4(5)	0(5)	12(5)
C(6B)	36(6)	67(6)	44(6)	0(6)	-5(6)	7(5)
C(7B)	22(11)	64(8)	69(13)	-23(9)	-8(10)	-2(9)
C(8B)	33(6)	55(7)	48(7)	3(6)	0(5)	1(5)
C(9B)	35(6)	62(8)	46(7)	6(6)	5(6)	3(6)
C(10B)	42(6)	55(8)	40(6)	0(6)	7(5)	3(6)
C(11B)	55(8)	42(5)	23(9)	-10(5)	-7(7)	1(5)
C(12B)	44(6)	55(7)	30(6)	-9(6)	-6(5)	-3(6)
C(13B)	45(6)	65(7)	35(6)	-8(6)	-9(5)	-11(6)
C(14B)	43(10)	65(13)	46(10)	7(11)	4(8)	-1(11)
F(21)	47(4)	66(7)	58(5)	-29(4)	7(3)	8(3)
F(22)	125(11)	62(6)	38(4)	6(4)	25(6)	24(6)
F(23)	71(4)	42(3)	43(4)	-18(2)	7(3)	4(2)
F(24)	94(7)	48(5)	107(7)	-36(5)	45(5)	-45(4)
F(25)	52(4)	137(8)	54(3)	37(4)	-6(3)	-41(4)
F(26)	37(3)	56(4)	136(8)	40(5)	33(5)	15(3)
F(31)	53(8)	55(7)	31(4)	-4(4)	4(4)	18(6)
F(32)	33(3)	72(6)	55(6)	-24(4)	15(3)	-12(4)
F(33)	50(5)	44(4)	46(3)	-5(3)	-2(3)	18(3)
F(34)	54(5)	55(4)	28(4)	4(3)	-8(3)	17(4)
F(35)	49(4)	86(6)	50(4)	31(4)	12(3)	-16(3)
F(36)	117(9)	49(4)	27(3)	-1(3)	24(4)	20(4)
F(41)	24(3)	33(4)	125(10)	17(5)	3(4)	-1(3)
F(42)	131(8)	43(3)	63(4)	9(4)	43(5)	-17(5)
F(43)	55(4)	42(3)	94(5)	2(4)	-33(4)	-16(3)
F(44)	63(4)	47(3)	91(5)	16(3)	49(3)	18(2)
F(45)	89(5)	98(6)	41(3)	-17(3)	-24(3)	66(4)
F(46)	40(2)	28(2)	134(5)	-17(3)	5(3)	8(2)
F(51)	95(6)	79(6)	48(5)	-36(4)	28(4)	-13(5)
F(52)	39(3)	50(6)	74(5)	-18(4)	-16(3)	3(3)
F(53)	111(9)	49(5)	48(6)	-20(3)	-36(6)	47(5)
F(54)	176(10)	37(4)	32(3)	1(3)	-4(5)	-10(5)
F(55)	124(5)	52(3)	62(3)	24(3)	-35(4)	-8(4)
F(56)	74(4)	108(6)	174(8)	86(6)	67(5)	16(4)
C(21)	27(4)	28(4)	11(4)	3(3)	-2(3)	5(3)
C(22)	32(5)	26(4)	25(5)	4(4)	2(4)	-1(4)
C(23)	40(4)	30(4)	25(5)	-5(4)	-1(4)	5(4)

C(24)	36(4)	25(4)	35(6)	1(4)	-7(4)	-4(4)
C(25)	26(4)	28(5)	34(5)	-2(4)	-1(4)	1(4)
C(26)	27(4)	25(4)	23(6)	1(4)	0(4)	-1(3)
C(27)	53(5)	41(5)	35(5)	-2(4)	7(4)	-4(4)
C(28)	31(4)	29(5)	50(5)	2(4)	-3(4)	-5(4)
C(31)	25(4)	22(4)	16(4)	-4(3)	-2(3)	-11(3)
C(32)	23(4)	25(5)	23(5)	-3(4)	-1(3)	-6(3)
C(33)	25(4)	28(5)	27(4)	-6(4)	-3(3)	-3(4)
C(34)	23(5)	21(5)	30(4)	3(4)	-5(3)	-4(4)
C(35)	24(4)	27(4)	26(4)	5(3)	1(3)	-8(4)
C(36)	20(4)	29(5)	22(4)	-4(4)	-2(4)	2(4)
C(37)	30(4)	37(5)	33(4)	-8(4)	-3(3)	1(3)
C(38)	39(5)	40(5)	31(4)	9(4)	3(4)	9(4)
C(41)	32(4)	26(4)	22(5)	3(4)	0(4)	2(3)
C(42)	24(4)	27(4)	25(5)	3(4)	-2(4)	8(3)
C(43)	26(4)	31(4)	31(5)	4(4)	-2(4)	-1(3)
C(44)	22(4)	37(4)	29(6)	3(5)	1(4)	3(3)
C(45)	31(4)	29(4)	27(5)	-2(4)	-1(3)	4(3)
C(46)	29(3)	21(4)	24(6)	-4(4)	-4(3)	1(3)
C(47)	27(5)	37(4)	52(5)	6(4)	-1(4)	-2(3)
C(48)	34(4)	39(4)	33(5)	3(4)	5(3)	7(3)
C(51)	22(4)	29(4)	21(4)	-3(3)	-2(3)	1(4)
C(52)	28(5)	26(4)	27(5)	-3(3)	-4(4)	3(4)
C(53)	22(4)	42(4)	24(4)	-7(3)	-4(4)	8(4)
C(54)	27(6)	43(4)	19(5)	-3(3)	-8(4)	3(4)
C(55)	30(5)	39(4)	20(4)	2(3)	-4(4)	-3(4)
C(56)	22(5)	28(4)	24(4)	-3(3)	-7(4)	-3(4)
C(57)	45(5)	42(4)	29(5)	-15(3)	-8(4)	13(4)
C(58)	53(5)	44(4)	34(5)	4(3)	4(3)	-12(4)
B(21)	28(4)	21(4)	26(4)	0(3)	2(3)	-2(3)
F(21A)	82(7)	62(7)	52(7)	6(6)	29(5)	31(5)
F(22A)	79(6)	51(5)	32(3)	0(3)	20(4)	5(4)
F(23A)	90(7)	128(9)	75(8)	-72(6)	34(5)	-53(7)
F(24A)	47(4)	30(4)	126(9)	24(4)	25(5)	7(3)
F(25A)	82(7)	89(6)	58(4)	-33(4)	34(4)	-56(4)
F(26A)	29(3)	107(8)	78(5)	47(5)	-2(4)	-1(4)
F(31A)	36(4)	50(6)	33(5)	-11(4)	10(3)	2(4)
F(32A)	34(4)	73(6)	59(6)	-17(4)	23(4)	-21(5)
F(33A)	52(6)	73(7)	60(7)	19(6)	21(5)	40(5)
F(34A)	42(4)	106(12)	29(5)	10(7)	-5(3)	10(5)
F(35A)	99(9)	73(6)	58(5)	29(5)	-1(7)	-42(6)
F(36A)	79(6)	90(8)	35(4)	30(5)	22(4)	48(6)
F(41A)	135(9)	67(5)	65(4)	35(3)	-22(5)	-31(7)
F(42A)	29(3)	67(6)	183(11)	50(8)	-16(6)	-19(3)
F(43A)	91(8)	29(4)	87(7)	-6(4)	27(6)	-5(5)
F(44A)	33(2)	72(4)	122(6)	10(4)	-5(3)	23(2)
F(45A)	51(3)	50(3)	95(5)	28(3)	12(3)	22(3)
F(46A)	148(9)	136(9)	42(4)	-37(5)	-19(5)	103(7)
F(51A)	85(6)	95(8)	96(11)	-51(8)	36(7)	1(5)
F(52A)	115(8)	39(5)	66(6)	-12(4)	-63(7)	5(7)
F(53A)	95(8)	35(4)	40(4)	-12(3)	-4(4)	6(5)
F(54A)	55(3)	47(5)	82(7)	30(4)	30(4)	22(3)
F(55A)	88(5)	60(3)	26(2)	13(2)	-1(3)	-11(4)
F(56A)	44(2)	43(3)	48(3)	21(2)	-10(2)	-16(2)
C(21A)	32(4)	22(4)	21(5)	-4(3)	1(4)	-1(3)
C(22A)	35(5)	29(5)	13(4)	-2(4)	4(4)	4(4)
C(23A)	39(5)	31(5)	27(5)	-6(4)	0(4)	2(4)
C(24A)	41(5)	32(5)	29(6)	-9(5)	-3(4)	2(4)
C(25A)	31(5)	25(5)	34(5)	2(4)	-2(4)	0(4)
C(26A)	31(5)	27(5)	20(6)	0(4)	2(4)	6(4)
C(27A)	56(5)	41(5)	32(5)	-13(4)	11(4)	-3(4)
C(28A)	37(5)	28(5)	43(5)	0(4)	-2(4)	-1(4)
C(31A)	28(4)	24(4)	24(5)	-3(4)	-1(3)	1(4)
C(32A)	27(4)	22(5)	20(5)	-4(4)	-2(3)	-8(3)
C(33A)	20(4)	25(5)	28(4)	-7(4)	-1(3)	-8(3)
C(34A)	19(5)	25(6)	30(4)	-2(4)	-3(4)	-6(4)
C(35A)	28(5)	27(5)	26(4)	3(4)	-2(4)	-4(4)
C(36A)	26(5)	25(5)	21(5)	1(4)	2(4)	-7(4)
C(37A)	31(5)	39(5)	33(5)	-1(4)	1(4)	2(4)
C(38A)	38(5)	47(5)	32(5)	14(4)	6(4)	2(4)
C(41A)	28(4)	29(5)	20(5)	-3(4)	1(4)	0(3)
C(42A)	25(4)	33(5)	27(6)	-5(4)	-2(4)	-4(4)
C(43A)	26(4)	32(4)	29(5)	-2(4)	-2(4)	-1(3)
C(44A)	28(5)	38(5)	31(6)	4(5)	4(5)	-2(4)
C(45A)	27(4)	37(5)	23(5)	3(4)	1(4)	8(4)
C(46A)	34(4)	31(6)	19(6)	3(5)	6(4)	-1(4)
C(47A)	29(5)	39(5)	56(5)	11(4)	-4(4)	-8(4)
C(48A)	41(4)	42(5)	38(5)	-2(4)	4(4)	9(4)
C(51A)	22(5)	20(4)	26(4)	-2(3)	-3(3)	-3(4)
C(52A)	22(5)	31(5)	19(4)	0(4)	-1(4)	-2(4)
C(53A)	32(5)	32(4)	26(5)	-9(3)	-7(4)	5(4)
C(54A)	27(6)	44(4)	19(5)	1(4)	-3(5)	4(4)
C(55A)	28(5)	31(4)	26(5)	2(3)	-3(4)	-3(4)
C(56A)	26(7)	26(4)	24(4)	1(4)	-2(4)	-7(4)
C(57A)	47(5)	47(5)	25(5)	-5(4)	-9(4)	4(5)
C(58A)	39(4)	45(5)	28(4)	14(4)	3(4)	1(3)
B(21A)	29(4)	24(4)	13(4)	-1(3)	3(3)	0(3)
Cl(61)	123(2)	45(1)	50(1)	-4(1)	18(1)	-24(1)
Cl(62)	98(1)	110(2)	37(1)	-4(1)	1(1)	5(1)
C(61)	65(3)	41(2)	44(2)	-2(2)	-3(2)	-3(2)
Cl(1C)	53(10)	31(9)	73(11)	3(8)	10(8)	-23(7)
Cl(2C)	70(6)	59(6)	112(8)	21(6)	19(5)	7(5)
C(61A)	65(3)	41(2)	44(2)	-2(2)	-3(2)	-3(2)
Cl(1D)	70(6)	59(6)	112(8)	21(6)	19(5)	7(5)
Cl(2D)	70(6)	54(6)	123(10)	-9(7)	-1(7)	1(5)

C(61B)	65(3)	41(2)	44(2)	-2(2)	-3(2)	-3(2)
Cl(71)	78(2)	48(1)	52(1)	-3(1)	6(1)	11(1)
Cl(72)	37(1)	56(2)	59(1)	3(1)	10(1)	1(1)
C(71)	55(5)	52(5)	48(4)	2(3)	-15(4)	-8(4)
Cl(1A)	71(4)	72(4)	61(3)	-6(3)	-5(3)	7(4)
Cl(2A)	52(4)	94(9)	105(7)	50(6)	27(4)	7(5)
C(71A)	66(12)	46(9)	52(8)	-5(7)	-17(10)	-8(10)
Cl(81)	44(2)	119(2)	54(2)	-17(2)	5(1)	-17(1)
Cl(82)	89(2)	150(5)	198(5)	88(4)	31(3)	7(3)
C(81)	63(3)	78(3)	93(9)	-12(3)	14(3)	-11(3)
Cl(1B)	148(10)	137(9)	225(13)	-124(9)	-7(8)	-11(6)
Cl(2B)	105(6)	41(4)	51(5)	19(3)	18(3)	-18(3)
C(81A)	63(3)	78(3)	93(9)	-12(3)	14(3)	-11(3)

Table 78. Hydrogen coordinates (x 10^4) and isotropic displacement parameters (Å^{2}x 10^3) for **9b-B$_{ArF}$F**.

	x	y	z	U(eq)
H(2)	6190(20)	4310(30)	4870(40)	55
H(1)	6021	2114	4001	62
H(2A)	5804	2026	4994	65
H(2AB)	6155	1418	4721	65
H(3A)	6556	1835	5523	47
H(3AB)	6963	1878	5031	47
H(5A)	7289	3371	5707	53
H(5AB)	7358	2538	5740	53
H(6A)	7657	2515	4855	56
H(6AB)	8045	3017	5199	56
H(8A)	8256	3598	4341	61
H(8AB)	7853	3024	4100	61
H(9A)	8037	3984	3533	62
H(9AB)	7705	4473	3933	62
H(10A)	7176	4048	3196	55
H(10B)	7225	3281	3443	55
H(12A)	6134	3966	3287	64
H(12B)	6375	3197	3371	64
H(13A)	5528	3141	3693	61
H(13B)	5584	3880	3986	61
H(2BA)	6230(70)	4194(16)	5230(40)	77
H(1AA)	5448	2283	4269	55
H(1AB)	5996	2278	3927	55
H(2AA)	6136	1474	4542	64
H(2AC)	5872	1943	4996	64
H(3AA)	6900	1794	4980	61
H(5AA)	7190	3235	5771	52
H(5AC)	7270	2407	5707	52
H(6AA)	7771	2528	4970	53
H(6AC)	7991	3140	5347	53
H(8AA)	8267	3381	4403	60
H(8AC)	7812	2886	4146	60
H(9AA)	8074	4225	3760	60
H(10C)	7305	3289	3384	55
H(10D)	7299	4101	3237	55
H(12C)	6294	3741	3185	49
H(12D)	6427	3010	3471	49
H(13C)	5517	3379	3646	53
H(13D)	5753	4072	3922	53
H(2CA)	6430(80)	4100(40)	5190(30)	55
H(1B)	6660(16)	2320(90)	4040(100)	109
H(1BA)	5375	2323	4188	59
H(1BB)	6001	2157	4066	59
H(2CB)	5575	2284	5096	55
H(2CC)	5652	1552	4802	55
H(3BA)	6591	1752	4767	51
H(3BB)	6360	1676	5353	51
H(5BA)	7245	2224	5631	58
H(5BB)	7353	2162	5008	58
H(6BA)	7932	3002	5338	59
H(6BB)	7439	3395	5625	59
H(8BA)	7871	2914	4353	54
H(8BB)	8254	3521	4578	54
H(9BA)	7807	4317	4009	57
H(9BB)	8158	3734	3711	57
H(10E)	7346	3109	3555	55
H(10F)	7379	3843	3258	55
H(12E)	6334	3730	3168	51
H(12F)	6509	2991	3411	51
H(13E)	5612	3087	3559	58
H(13F)	5669	3877	3759	58
H(22)	3952	2058	8404	33
H(24)	5032	3655	8366	39
H(26)	5136	2074	7302	30
H(32)	5189	432	7169	28
H(34)	5620	-759	8422	30
H(36)	4337	569	8525	28
H(42)	3712	-33	7801	30
H(44)	2176	621	8128	35
H(46)	3347	2023	7726	30
H(52)	4249	156	6783	32
H(54)	4309	1258	5418	35
H(56)	4246	2265	6819	30
H(22A)	3941	2045	8487	31
H(24A)	5057	3613	8469	41
H(26A)	5145	2047	7399	31
H(32A)	5205	531	7218	28
H(34A)	5709	-678	8436	30
H(36A)	4382	572	8594	29
H(42A)	3770	-47	7897	34
H(44A)	2181	511	8087	38
H(46A)	3297	1944	7620	33
H(52A)	4246	201	6848	29
H(54A)	4368	1283	5483	36
H(56A)	4328	2304	6877	30
H(61A)	6469	5349	5110	60
H(61B)	6173	6094	5078	60
H(61C)	7556	5950	5775	60
H(61D)	7231	6651	5622	60
H(61E)	6683	5704	6105	60
H(61F)	6579	5193	5612	60
H(71A)	5668	614	5543	62

H(71B)	5621	140	6061	62
H(71C)	5854	349	5542	66
H(71D)	6040	-26	6078	66
H(81A)	2031	3088	6512	93
H(81B)	2125	3380	7094	93
H(81C)	2033	3199	6343	93
H(81D)	2131	3241	6968	93

$[(D_{12}\text{-TMC})Fe^{IV}(O)(H_2O)]\{N(OTf)_2\}B_{Ar}F$

The crystal structure of $[(D_{12}\text{-TMC})Fe^{IV}(O)(H_2O)]\{N(OTf)_2\}B_{Ar}F$ was solved in the monoclinic space group $P2_1/c$. The asymmetric unit contains one full anion, cation and a solvent molecule. Additional, heavily disordered solvent was identified in the unit cell. The disordered solvent was modelled as bulk solvent model using the program SQUEEZE[7] as implemented in PLATON[8-11] and included into the refinement as .fab file. SQUEEZE identified two solvent accessible voids at 0.5 0.5 0.0 and 0.5 0.0 0.5, each with a volume of 354 $Å^3$ and an equivalent of 916 electrons. All anions were found to be disordered over two positions each. The disorder ratios were refined using similarity restraints on anisotropic displacement parameters, same distance restraints, advanced rigid bond restraints and by setting some anisotropic displacement parameters to have the same values. All disorder ratios were allowed to refine freely and converged to 0.619(7) and 0.70(1).

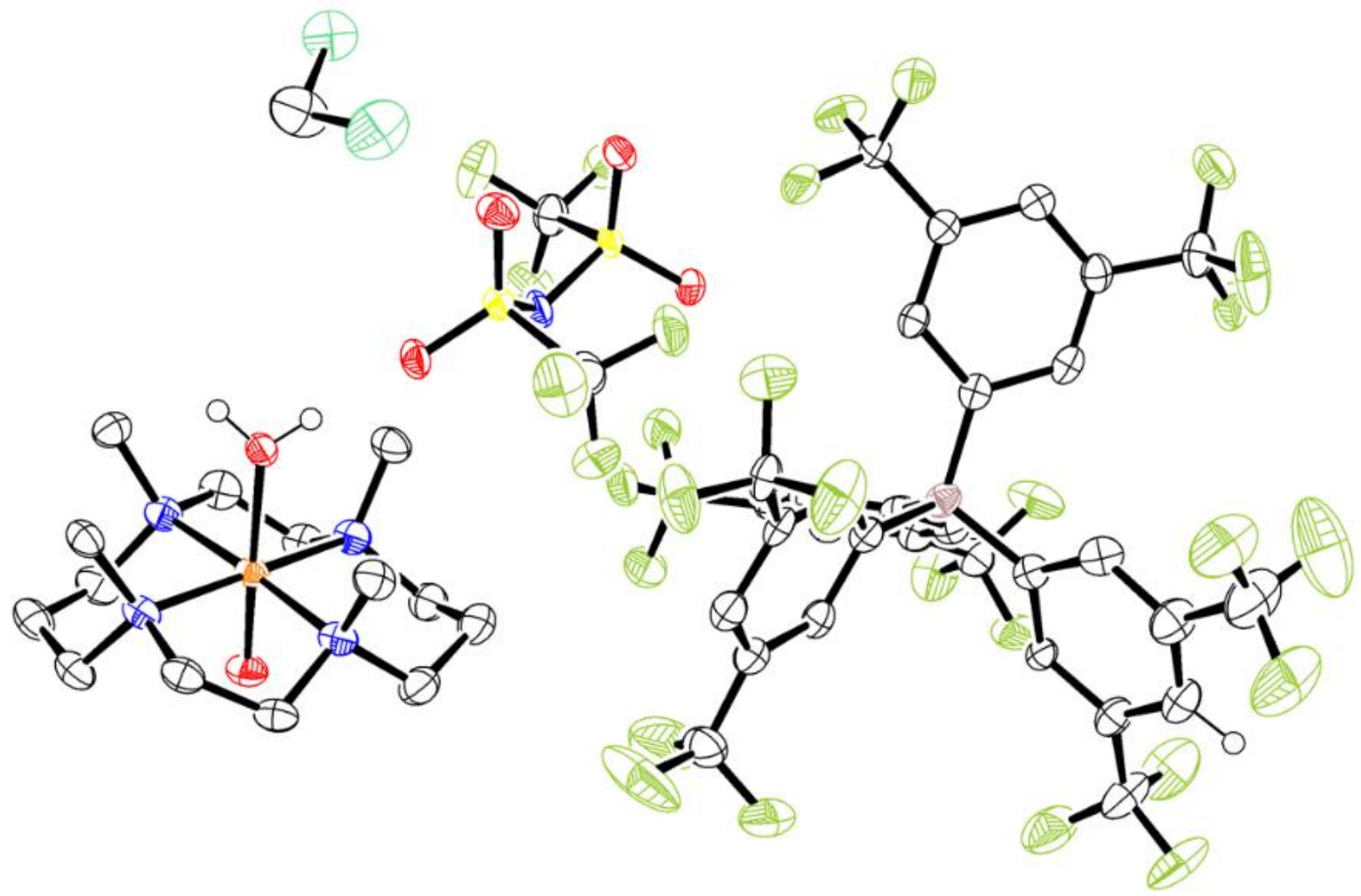

Figure S16. Molecular structure of $[(D_{12}\text{-TMC})Fe^{IV}(O)(H_2O)]\{N(OTf)_2\}B_{Ar}F$.

Table S79. Crystal data and structure refinement for **$[(D_{12}\text{-TMC})Fe^{IV}(O)(H_2O)]\{N(OTf)_2\}B_{Ar}F$**.

CCDC No	2122183	
Empirical formula	$C_{49}H_{36}BCl_2D_{12}F_{30}FeN_5O_6S_2$	
Formula weight	1586.67	
Temperature	100(2) K	
Wavelength	0.71073 Å	
Crystal system	Monoclinic	
Space group	$P2_1/c$	
Unit cell dimensions	a = 13.0646(6) Å	α = 90°
	b = 20.0733(10) Å	β = 91.2820(10)°
	c = 28.8686(13) Å	γ = 90°
Volume	7568.9(6) $Å^3$	
Z	4	
Density (calculated)	1.392 Mg/m^3	
Absorption coefficient	0.441 mm^{-1}	
F(000)	3168	
Crystal size	0.249 x 0.243 x 0.141 mm^3	
Theta range for data collection	1.977 to 27.102°.	
Index ranges	$-16 \le h \le 16$, $-25 \le k \le 25$, $-37 \le l \le 37$	
Reflections collected	170305	
Independent reflections	16700 [R(int) = 0.0441]	
Completeness to theta = 25.242°	99.9 %	
Absorption correction	Semi-empirical from equivalents	
Refinement method	Full-matrix least-squares on F^2	
Data / restraints / parameters	16700 / 4092 / 1501	
Goodness-of-fit on F^2	1.040	
Final R indices [I>2σ(I)]	R1 = 0.0606, wR2 = 0.1617	
R indices (all data)	R1 = 0.0742, wR2 = 0.1716	
Largest diff. peak and hole	0.696 and -1.017 $eÅ^{-3}$	

Table S80. Atomic coordinates (x 10^4) and equivalent isotropic displacement parameters ($Å^2$x 10^3) for **[(D$_{12}$-TMC)Fe^{IV}(O)(H$_2$O)]{N(OTf)$_2$}B$_{ArF}$**. U(eq) is defined as one third of the trace of the orthogonalized U^{ij} tensor.

	x	y	z	U(eq)
Fe(1)	2471(1)	3274(1)	5804(1)	23(1)
O(1)	2095(2)	2608(1)	6086(1)	30(1)
O(2)	2949(2)	4114(1)	5474(1)	25(1)
N(1)	981(2)	3676(1)	5812(1)	26(1)
N(2)	2212(2)	2794(1)	5163(1)	28(1)
N(3)	3908(2)	2833(1)	5785(1)	29(1)
N(4)	2659(2)	3723(1)	6448(1)	29(1)
C(1)	228(2)	3107(2)	5764(1)	32(1)
C(2)	303(3)	2719(2)	5313(1)	37(1)
C(3)	1286(3)	2352(2)	5216(1)	36(1)
C(4)	2065(3)	3199(2)	4731(1)	32(1)
D(4A)	1968	2901	4465	48
D(4B)	2671	3477	4685	48
D(4C)	1461	3484	4760	48
C(5)	3136(3)	2366(2)	5077(1)	34(1)
C(6)	3700(3)	2202(2)	5521(1)	34(1)
C(7)	4713(2)	3229(2)	5545(1)	33(1)
D(7A)	4442	3382	5244	50
D(7B)	5316	2948	5499	50
D(7C)	4906	3615	5736	50
C(8)	4336(3)	2620(2)	6248(1)	36(1)
C(9)	4376(3)	3169(2)	6611(1)	39(1)
C(10)	3322(3)	3338(2)	6785(1)	37(1)
C(11)	3025(3)	4428(2)	6444(1)	33(1)
D(11A)	3727	4443	6332	50
D(11B)	3010	4610	6759	50
D(11C)	2578	4693	6239	50
C(12)	1596(3)	3706(2)	6634(1)	32(1)
C(13)	848(2)	3993(2)	6286(1)	31(1)
C(14)	677(2)	4208(2)	5472(1)	29(1)
D(14A)	695	4028	5157	43
D(14B)	1155	4583	5501	43
D(14C)	-17	4361	5537	43
S(21)	5620(6)	5151(4)	5675(2)	24(1)
S(22)	7511(5)	4561(4)	5498(3)	24(1)
F(21)	5682(4)	4866(4)	6562(2)	59(2)
F(22)	6799(4)	5592(4)	6351(2)	50(1)
F(23)	5214(4)	5869(4)	6396(2)	62(2)
F(24)	8488(7)	3852(7)	4902(4)	42(2)
F(25)	6964(7)	4149(6)	4674(3)	51(2)
F(26)	7170(13)	3352(5)	5168(5)	50(2)
O(21)	4583(7)	4919(7)	5660(6)	27(2)
O(22)	5866(13)	5730(5)	5410(3)	33(2)
O(23)	7834(15)	5163(6)	5269(6)	30(3)
O(24)	8170(9)	4300(9)	5857(5)	31(1)
N(21)	6357(5)	4535(4)	5632(3)	26(2)
C(21)	5837(4)	5388(5)	6283(2)	40(1)
C(22)	7523(8)	3940(5)	5035(3)	34(2)
S(21A)	5667(13)	5175(9)	5667(5)	22(2)
S(22A)	7615(12)	4615(9)	5546(6)	21(2)
F(21A)	5585(8)	5275(11)	6561(3)	58(4)
F(22A)	6790(9)	5876(9)	6232(6)	55(4)
F(23A)	5212(9)	6179(8)	6198(6)	60(4)
F(24A)	8344(18)	3954(18)	4852(10)	40(4)
F(25A)	6742(15)	4142(15)	4785(6)	42(4)
F(26A)	7290(30)	3396(11)	5268(10)	40(4)
O(21A)	4677(16)	4872(18)	5717(14)	28(5)
O(22A)	5790(30)	5649(12)	5304(7)	27(4)
O(23A)	7920(30)	5213(14)	5314(14)	21(3)
O(24A)	8300(20)	4320(20)	5881(12)	31(1)
N(21A)	6490(12)	4614(9)	5733(7)	22(3)
C(21A)	5819(10)	5663(9)	6203(5)	40(3)
C(22A)	7483(18)	3996(11)	5090(7)	31(3)
F(31)	6772(10)	3220(8)	6351(7)	37(2)
F(32)	7947(10)	2618(6)	6046(4)	42(2)
F(33)	7016(14)	2215(8)	6580(6)	48(2)
F(34)	10404(6)	1628(3)	7309(3)	54(2)
F(35)	10477(12)	2116(6)	7917(3)	57(3)
F(36)	11485(10)	2436(6)	7400(6)	56(3)
C(31)	8964(15)	3837(7)	7350(7)	25(2)
C(32)	8271(16)	3637(7)	6995(7)	24(2)
C(33)	8273(14)	2999(7)	6808(7)	26(2)
C(34)	9009(17)	2533(9)	6954(9)	29(2)
C(35)	9732(14)	2724(7)	7288(7)	27(2)
C(36)	9700(20)	3361(11)	7482(14)	27(2)
C(37)	7507(10)	2774(7)	6448(5)	31(2)
C(38)	10520(9)	2242(5)	7466(4)	35(2)
F(41)	8684(7)	6138(6)	6151(4)	45(2)
F(42)	9930(12)	5686(7)	5794(5)	42(2)
F(43)	10117(8)	6657(5)	6098(5)	41(2)
F(44)	13112(12)	5880(8)	7059(6)	45(3)
F(45)	12428(11)	6152(6)	7703(5)	77(3)
F(46)	12843(11)	5140(5)	7582(4)	40(2)
C(41)	9707(10)	5051(11)	7279(5)	22(2)
C(42)	9401(14)	5347(15)	6860(8)	22(2)
C(43)	10069(11)	5743(11)	6608(6)	24(2)
C(44)	11072(11)	5870(12)	6762(6)	24(2)
C(45)	11389(10)	5563(10)	7170(4)	25(2)
C(46)	10732(12)	5167(14)	7422(6)	26(2)
C(47)	9701(9)	6060(6)	6163(4)	26(2)
C(48)	12445(9)	5688(6)	7378(4)	33(2)

F(51)	5197(10)	3561(6)	7738(5)	93(3)
F(52)	5457(6)	3943(5)	8404(3)	62(2)
F(53)	4293(6)	4352(4)	7985(4)	105(3)
F(54)	6419(11)	6811(7)	7784(5)	66(3)
F(55)	5164(6)	6519(5)	7342(3)	55(2)
F(56)	6652(7)	6682(7)	7077(4)	44(2)
C(51)	7761(8)	4859(7)	7598(9)	26(2)
C(52)	6997(8)	4426(7)	7758(7)	27(2)
C(53)	5984(9)	4618(6)	7788(7)	31(2)
C(54)	5672(11)	5263(6)	7666(9)	29(2)
C(55)	6421(10)	5705(5)	7524(6)	25(2)
C(56)	7445(12)	5511(8)	7494(14)	25(2)
C(57)	5226(7)	4133(5)	7971(4)	42(2)
C(58)	6144(8)	6421(5)	7437(4)	27(2)
F(61)	9410(13)	3521(8)	9634(3)	65(3)
F(62)	10348(5)	3062(2)	9135(2)	72(2)
F(63)	8749(5)	2976(2)	9059(2)	66(2)
F(64)	9970(10)	5899(7)	9489(3)	87(3)
F(65)	11303(6)	5869(5)	9023(4)	126(3)
F(66)	9948(8)	6389(3)	8853(2)	81(2)
C(61)	9304(15)	4576(6)	8139(4)	25(2)
C(62)	9220(11)	4015(6)	8427(4)	22(2)
C(63)	9461(10)	4039(4)	8902(4)	29(2)
C(64)	9809(15)	4623(6)	9106(5)	34(3)
C(65)	9862(17)	5198(5)	8837(4)	39(2)
C(66)	9668(10)	5159(6)	8361(4)	31(2)
C(67)	9458(8)	3405(4)	9181(3)	48(2)
C(68)	10298(9)	5827(5)	9051(4)	64(2)
B(31)	8948(8)	4584(5)	7585(4)	26(2)
F(31A)	6980(17)	3256(13)	6332(12)	39(4)
F(32A)	7950(20)	2470(11)	6117(7)	59(5)
F(33A)	6900(20)	2303(13)	6664(9)	54(5)
F(34A)	10759(12)	1756(7)	7208(6)	88(5)
F(35A)	10565(18)	2187(9)	7947(5)	40(3)
F(36A)	11569(17)	2597(10)	7468(10)	46(3)
C(31A)	8970(20)	3814(12)	7389(11)	26(3)
C(32A)	8270(30)	3586(13)	7050(12)	24(2)
C(33A)	8350(20)	2961(12)	6845(11)	27(3)
C(34A)	9130(30)	2520(15)	6983(14)	26(3)
C(35A)	9820(20)	2737(11)	7316(11)	27(3)
C(36A)	9780(40)	3378(16)	7500(20)	24(3)
C(37A)	7562(16)	2752(11)	6495(7)	34(3)
C(38A)	10667(15)	2277(8)	7488(6)	35(3)
F(41A)	8805(12)	6312(10)	6224(8)	47(4)
F(42A)	9730(20)	5630(12)	5853(8)	45(4)
F(43A)	10349(14)	6549(9)	6069(10)	47(4)
F(44A)	13100(20)	5865(16)	7131(9)	51(4)
F(45A)	12394(16)	6201(7)	7761(6)	45(3)
F(46A)	12800(20)	5195(11)	7688(8)	62(5)
C(41A)	9678(17)	5038(19)	7329(9)	25(3)
C(42A)	9430(20)	5320(30)	6900(13)	25(3)
C(43A)	10082(19)	5737(19)	6661(9)	25(3)
C(44A)	11070(20)	5850(20)	6839(10)	28(3)
C(45A)	11363(16)	5558(16)	7251(8)	27(3)
C(46A)	10700(20)	5150(20)	7489(11)	26(2)
C(47A)	9752(15)	6053(10)	6216(7)	29(3)
C(48A)	12414(15)	5717(11)	7455(7)	39(3)
F(51A)	5311(10)	3473(6)	7732(6)	43(3)
F(52A)	5073(13)	4018(10)	8352(5)	91(5)
F(53A)	4199(10)	4209(7)	7753(5)	76(4)
F(54A)	6527(12)	6835(10)	7847(6)	40(3)
F(55A)	5120(10)	6542(8)	7555(5)	67(4)
F(56A)	6389(15)	6666(12)	7126(7)	69(4)
C(51A)	7685(14)	4827(11)	7585(15)	26(3)
C(52A)	6892(14)	4382(12)	7692(12)	28(3)
C(53A)	5894(15)	4588(10)	7758(13)	34(3)
C(54A)	5617(19)	5260(10)	7722(16)	31(3)
C(55A)	6383(16)	5705(8)	7603(10)	27(3)
C(56A)	7389(19)	5497(13)	7530(20)	25(2)
C(57A)	5124(12)	4082(8)	7895(6)	44(3)
C(58A)	6115(13)	6424(9)	7538(7)	34(3)
F(61A)	9230(20)	3613(13)	9679(5)	68(5)
F(62A)	9537(9)	2962(4)	9106(3)	60(2)
F(63A)	8110(5)	3348(4)	9181(3)	66(2)
F(64A)	10386(17)	5868(12)	9475(5)	95(5)
F(65A)	10969(9)	6093(5)	8820(3)	66(2)
F(66A)	9381(9)	6339(5)	8907(5)	74(3)
C(61A)	9180(30)	4578(11)	8166(6)	28(3)
C(62A)	9040(20)	4046(11)	8468(7)	31(4)
C(63A)	9299(19)	4108(7)	8940(6)	37(3)
C(64A)	9670(30)	4686(9)	9138(8)	36(4)
C(65A)	9790(30)	5219(9)	8838(7)	42(3)
C(66A)	9442(18)	5184(10)	8377(7)	32(3)
C(67A)	9046(9)	3518(6)	9232(4)	40(2)
C(68A)	10110(12)	5895(7)	9020(6)	57(3)
B(31A)	8880(13)	4566(9)	7607(6)	27(2)
Cl(71)	6862(1)	3135(1)	3889(1)	82(1)
Cl(72)	7476(1)	2479(1)	3040(1)	60(1)
C(71)	6475(5)	2636(3)	3420(2)	70(1)

Table S81. Bond lengths [Å] and angles [°] for **[(D$_{12}$-TMC)Fe^{IV}(O)(H$_2$O)]{N(OTf)$_2$}B$_{Ar}$F**.

Fe(1)-O(1)	1.648(2)	C(31)-C(32)	1.411(8)
Fe(1)-O(2)	2.042(2)	C(31)-B(31)	1.646(8)
Fe(1)-N(4)	2.076(3)	C(32)-C(33)	1.390(8)
Fe(1)-N(3)	2.078(3)	C(32)-H(32)	0.9500
Fe(1)-N(1)	2.107(3)	C(33)-C(34)	1.399(9)
Fe(1)-N(2)	2.107(3)	C(33)-C(37)	1.497(8)
O(2)-H(2A)	0.844(18)	C(34)-C(35)	1.387(9)
O(2)-H(2B)	0.839(18)	C(34)-H(34)	0.9500
N(1)-C(14)	1.498(4)	C(35)-C(36)	1.397(8)
N(1)-C(1)	1.511(4)	C(35)-C(38)	1.495(8)
N(1)-C(13)	1.523(4)	C(36)-H(36)	0.9500
N(2)-C(4)	1.498(4)	F(41)-C(47)	1.338(8)
N(2)-C(5)	1.508(4)	F(42)-C(47)	1.342(9)
N(2)-C(3)	1.510(4)	F(43)-C(47)	1.331(8)
N(3)-C(8)	1.498(4)	F(44)-C(48)	1.339(9)
N(3)-C(7)	1.499(4)	F(45)-C(48)	1.323(9)
N(3)-C(6)	1.501(4)	F(46)-C(48)	1.347(8)
N(4)-C(11)	1.494(4)	C(41)-C(42)	1.400(8)
N(4)-C(12)	1.500(4)	C(41)-C(46)	1.412(8)
N(4)-C(10)	1.502(4)	C(41)-B(31)	1.638(8)
C(1)-C(2)	1.523(5)	C(42)-C(43)	1.395(8)
C(1)-H(1A)	0.9900	C(42)-H(42)	0.9500
C(1)-H(1AB)	0.9900	C(43)-C(44)	1.398(8)
C(2)-C(3)	1.512(5)	C(43)-C(47)	1.503(8)
C(2)-H(2AA)	0.9900	C(44)-C(45)	1.384(9)
C(2)-H(2AB)	0.9900	C(44)-H(44)	0.9500
C(3)-H(3A)	0.9900	C(45)-C(46)	1.388(8)
C(3)-H(3AB)	0.9900	C(45)-C(48)	1.513(8)
C(4)-D(4A)	0.9800	C(46)-H(46)	0.9500
C(4)-D(4B)	0.9800	F(51)-C(57)	1.330(10)
C(4)-D(4C)	0.9800	F(52)-C(57)	1.336(9)
C(5)-C(6)	1.499(5)	F(53)-C(57)	1.298(9)
C(5)-H(5A)	0.9900	F(54)-C(58)	1.316(8)
C(5)-H(5AB)	0.9900	F(55)-C(58)	1.318(8)
C(6)-H(6A)	0.9900	F(56)-C(58)	1.350(9)
C(6)-H(6AB)	0.9900	C(51)-C(56)	1.402(9)
C(7)-D(7A)	0.9800	C(51)-C(52)	1.409(9)
C(7)-D(7B)	0.9800	C(51)-B(31)	1.647(8)
C(7)-D(7C)	0.9800	C(52)-C(53)	1.384(8)
C(8)-C(9)	1.521(5)	C(52)-H(52)	0.9500
C(8)-H(8A)	0.9900	C(53)-C(54)	1.400(9)
C(8)-H(8AB)	0.9900	C(53)-C(57)	1.493(9)
C(9)-C(10)	1.515(5)	C(54)-C(55)	1.389(9)
C(9)-H(9A)	0.9900	C(54)-H(54)	0.9500
C(9)-H(9AB)	0.9900	C(55)-C(56)	1.398(9)
C(10)-H(10A)	0.9900	C(55)-C(58)	1.502(8)
C(10)-H(10B)	0.9900	C(56)-H(56)	0.9500
C(11)-D(11A)	0.9800	F(61)-C(67)	1.331(9)
C(11)-D(11B)	0.9800	F(62)-C(67)	1.360(11)
C(11)-D(11C)	0.9800	F(63)-C(67)	1.307(10)
C(12)-C(13)	1.502(5)	F(64)-C(68)	1.353(11)
C(12)-H(12A)	0.9900	F(65)-C(68)	1.320(11)
C(12)-H(12B)	0.9900	F(66)-C(68)	1.340(10)
C(13)-H(13A)	0.9900	C(61)-C(62)	1.407(8)
C(13)-H(13B)	0.9900	C(61)-C(66)	1.412(8)
C(14)-D(14A)	0.9800	C(61)-B(31)	1.655(8)
C(14)-D(14B)	0.9800	C(62)-C(63)	1.399(8)
C(14)-D(14C)	0.9800	C(62)-H(62)	0.9500
S(21)-O(22)	1.432(7)	C(63)-C(64)	1.386(9)
S(21)-O(21)	1.432(6)	C(63)-C(67)	1.506(9)
S(21)-N(21)	1.573(6)	C(64)-C(65)	1.394(9)
S(21)-C(21)	1.835(7)	C(64)-H(64)	0.9500
S(22)-O(24)	1.433(6)	C(65)-C(66)	1.395(9)
S(22)-O(23)	1.443(6)	C(65)-C(68)	1.512(9)
S(22)-N(21)	1.566(6)	C(66)-H(66)	0.9500
S(22)-C(22)	1.827(7)	F(31A)-C(37A)	1.344(13)
F(21)-C(21)	1.341(8)	F(32A)-C(37A)	1.337(13)
F(22)-C(21)	1.333(6)	F(33A)-C(37A)	1.349(13)
F(23)-C(21)	1.308(8)	F(34A)-C(38A)	1.331(13)
F(24)-C(22)	1.339(7)	F(35A)-C(38A)	1.345(13)
F(25)-C(22)	1.327(7)	F(36A)-C(38A)	1.344(14)
F(26)-C(22)	1.327(8)	C(31A)-C(36A)	1.402(13)
S(21A)-O(22A)	1.426(13)	C(31A)-C(32A)	1.407(12)
S(21A)-O(21A)	1.439(14)	C(31A)-B(31A)	1.642(12)
S(21A)-N(21A)	1.565(13)	C(32A)-C(33A)	1.393(13)
S(21A)-C(21A)	1.840(13)	C(32A)-H(32A)	0.9500
S(22A)-O(24A)	1.429(13)	C(33A)-C(34A)	1.396(13)
S(22A)-O(23A)	1.437(13)	C(33A)-C(37A)	1.489(12)
S(22A)-N(21A)	1.577(13)	C(34A)-C(35A)	1.381(13)
S(22A)-C(22A)	1.814(14)	C(34A)-H(34A)	0.9500
F(21A)-C(21A)	1.334(15)	C(35A)-C(36A)	1.398(12)
F(22A)-C(21A)	1.339(13)	C(35A)-C(38A)	1.513(12)
F(23A)-C(21A)	1.305(15)	C(36A)-H(36A)	0.9500
F(24A)-C(22A)	1.334(15)	F(41A)-C(47A)	1.342(13)
F(25A)-C(22A)	1.328(14)	F(42A)-C(47A)	1.349(13)
F(26A)-C(22A)	1.335(14)	F(43A)-C(47A)	1.338(13)
F(31)-C(37)	1.338(9)	F(44A)-C(48A)	1.342(13)
F(32)-C(37)	1.342(9)	F(45A)-C(48A)	1.314(13)
F(33)-C(37)	1.352(9)	F(46A)-C(48A)	1.339(13)
F(34)-C(38)	1.319(9)	C(41A)-C(42A)	1.392(13)
F(35)-C(38)	1.329(9)	C(41A)-C(46A)	1.416(12)
F(36)-C(38)	1.337(9)	C(41A)-B(31A)	1.633(12)
C(31)-C(36)	1.402(9)	C(42A)-C(43A)	1.396(13)

C(42A)-H(42A)	0.9500
C(43A)-C(44A)	1.399(12)
C(43A)-C(47A)	1.488(12)
C(44A)-C(45A)	1.373(13)
C(44A)-H(44A)	0.9500
C(45A)-C(46A)	1.386(13)
C(45A)-C(48A)	1.516(12)
C(46A)-H(46A)	0.9500
F(51A)-C(57A)	1.335(13)
F(52A)-C(57A)	1.328(14)
F(53A)-C(57A)	1.293(14)
F(54A)-C(58A)	1.322(13)
F(55A)-C(58A)	1.324(13)
F(56A)-C(58A)	1.340(13)
C(51A)-C(56A)	1.406(13)
C(51A)-C(52A)	1.407(13)
C(51A)-B(31A)	1.646(12)
C(52A)-C(53A)	1.385(13)
C(52A)-H(52A)	0.9500
C(53A)-C(54A)	1.399(13)
C(53A)-C(57A)	1.489(13)
C(54A)-C(55A)	1.390(13)
C(54A)-H(54A)	0.9500
C(55A)-C(56A)	1.398(13)
C(55A)-C(58A)	1.496(12)
C(56A)-H(56A)	0.9500
F(61A)-C(67A)	1.320(13)
F(62A)-C(67A)	1.341(12)
F(63A)-C(67A)	1.275(12)
F(64A)-C(68A)	1.357(14)
F(65A)-C(68A)	1.334(15)
F(66A)-C(68A)	1.340(14)
C(61A)-C(62A)	1.392(13)
C(61A)-C(66A)	1.401(13)
C(61A)-B(31A)	1.650(12)
C(62A)-C(63A)	1.402(13)
C(62A)-H(62A)	0.9500
C(63A)-C(64A)	1.375(13)
C(63A)-C(67A)	1.494(13)
C(64A)-C(65A)	1.389(13)
C(64A)-H(64A)	0.9500
C(65A)-C(66A)	1.397(13)
C(65A)-C(68A)	1.510(13)
C(66A)-H(66A)	0.9500
Cl(71)-C(71)	1.749(5)
Cl(72)-C(71)	1.754(5)
C(71)-H(71A)	0.9900
C(71)-H(71B)	0.9900
O(1)-Fe(1)-O(2)	178.20(11)
O(1)-Fe(1)-N(4)	86.57(11)
O(2)-Fe(1)-N(4)	91.64(10)
O(1)-Fe(1)-N(3)	86.93(11)
O(2)-Fe(1)-N(3)	93.09(9)
N(4)-Fe(1)-N(3)	96.86(11)
O(1)-Fe(1)-N(1)	91.11(10)
O(2)-Fe(1)-N(1)	88.93(9)
N(4)-Fe(1)-N(1)	85.14(10)
N(3)-Fe(1)-N(1)	177.11(11)
O(1)-Fe(1)-N(2)	91.07(11)
O(2)-Fe(1)-N(2)	90.72(9)
N(4)-Fe(1)-N(2)	177.01(10)
N(3)-Fe(1)-N(2)	84.83(11)
N(1)-Fe(1)-N(2)	93.08(10)
Fe(1)-O(2)-H(2A)	125(2)
Fe(1)-O(2)-H(2B)	125(2)
H(2A)-O(2)-H(2B)	105(3)
C(14)-N(1)-C(1)	108.4(2)
C(14)-N(1)-C(13)	104.8(2)
C(1)-N(1)-C(13)	108.1(2)
C(14)-N(1)-Fe(1)	119.82(18)
C(1)-N(1)-Fe(1)	108.05(18)
C(13)-N(1)-Fe(1)	107.07(19)
C(4)-N(2)-C(5)	105.1(2)
C(4)-N(2)-C(3)	108.4(3)
C(5)-N(2)-C(3)	109.2(2)
C(4)-N(2)-Fe(1)	119.85(19)
C(5)-N(2)-Fe(1)	107.0(2)
C(3)-N(2)-Fe(1)	107.1(2)
C(8)-N(3)-C(7)	108.2(3)
C(8)-N(3)-C(6)	105.8(2)
C(7)-N(3)-C(6)	109.4(3)
C(8)-N(3)-Fe(1)	114.7(2)
C(7)-N(3)-Fe(1)	115.43(19)
C(6)-N(3)-Fe(1)	102.63(19)
C(11)-N(4)-C(12)	108.9(3)
C(11)-N(4)-C(10)	108.2(3)
C(12)-N(4)-C(10)	106.3(2)
C(11)-N(4)-Fe(1)	115.8(2)
C(12)-N(4)-Fe(1)	102.6(2)
C(10)-N(4)-Fe(1)	114.3(2)
N(1)-C(1)-C(2)	114.3(3)
N(1)-C(1)-H(1A)	108.7
C(2)-C(1)-H(1A)	108.7
N(1)-C(1)-H(1AB)	108.7

C(2)-C(1)-H(1AB)	108.7
H(1A)-C(1)-H(1AB)	107.6
C(3)-C(2)-C(1)	118.5(3)
C(3)-C(2)-H(2AA)	107.7
C(1)-C(2)-H(2AA)	107.7
C(3)-C(2)-H(2AB)	107.7
C(1)-C(2)-H(2AB)	107.7
H(2AA)-C(2)-H(2AB)	107.1
N(2)-C(3)-C(2)	114.7(3)
N(2)-C(3)-H(3A)	108.6
C(2)-C(3)-H(3A)	108.6
N(2)-C(3)-H(3AB)	108.6
C(2)-C(3)-H(3AB)	108.6
H(3A)-C(3)-H(3AB)	107.6
N(2)-C(4)-D(4A)	109.5
N(2)-C(4)-D(4B)	109.5
D(4A)-C(4)-D(4B)	109.5
N(2)-C(4)-D(4C)	109.5
D(4A)-C(4)-D(4C)	109.5
D(4B)-C(4)-D(4C)	109.5
C(6)-C(5)-N(2)	111.3(3)
C(6)-C(5)-H(5A)	109.4
N(2)-C(5)-H(5A)	109.4
C(6)-C(5)-H(5AB)	109.4
N(2)-C(5)-H(5AB)	109.4
H(5A)-C(5)-H(5AB)	108.0
C(5)-C(6)-N(3)	109.3(3)
C(5)-C(6)-H(6A)	109.8
N(3)-C(6)-H(6A)	109.8
C(5)-C(6)-H(6AB)	109.8
N(3)-C(6)-H(6AB)	109.8
H(6A)-C(6)-H(6AB)	108.3
N(3)-C(7)-D(7A)	109.5
N(3)-C(7)-D(7B)	109.5
D(7A)-C(7)-D(7B)	109.5
N(3)-C(7)-D(7C)	109.5
D(7A)-C(7)-D(7C)	109.5
D(7B)-C(7)-D(7C)	109.5
N(3)-C(8)-C(9)	114.4(3)
N(3)-C(8)-H(8A)	108.7
C(9)-C(8)-H(8A)	108.7
N(3)-C(8)-H(8AB)	108.7
C(9)-C(8)-H(8AB)	108.7
H(8A)-C(8)-H(8AB)	107.6
C(10)-C(9)-C(8)	111.9(3)
C(10)-C(9)-H(9A)	109.2
C(8)-C(9)-H(9A)	109.2
C(10)-C(9)-H(9AB)	109.2
C(8)-C(9)-H(9AB)	109.2
H(9A)-C(9)-H(9AB)	107.9
N(4)-C(10)-C(9)	114.6(3)
N(4)-C(10)-H(10A)	108.6
C(9)-C(10)-H(10A)	108.6
N(4)-C(10)-H(10B)	108.6
C(9)-C(10)-H(10B)	108.6
H(10A)-C(10)-H(10B)	107.6
N(4)-C(11)-D(11A)	109.5
N(4)-C(11)-D(11B)	109.5
D(11A)-C(11)-D(11B)	109.5
N(4)-C(11)-D(11C)	109.5
D(11A)-C(11)-D(11C)	109.5
D(11B)-C(11)-D(11C)	109.5
N(4)-C(12)-C(13)	110.2(3)
N(4)-C(12)-H(12A)	109.6
C(13)-C(12)-H(12A)	109.6
N(4)-C(12)-H(12B)	109.6
C(13)-C(12)-H(12B)	109.6
H(12A)-C(12)-H(12B)	108.1
C(12)-C(13)-N(1)	110.8(3)
C(12)-C(13)-H(13A)	109.5
N(1)-C(13)-H(13A)	109.5
C(12)-C(13)-H(13B)	109.5
N(1)-C(13)-H(13B)	109.5
H(13A)-C(13)-H(13B)	108.1
N(1)-C(14)-D(14A)	109.5
N(1)-C(14)-D(14B)	109.5
D(14A)-C(14)-D(14B)	109.5
N(1)-C(14)-D(14C)	109.5
D(14A)-C(14)-D(14C)	109.5
D(14B)-C(14)-D(14C)	109.5
O(22)-S(21)-O(21)	118.2(8)
O(22)-S(21)-N(21)	116.8(7)
O(21)-S(21)-N(21)	108.8(6)
O(22)-S(21)-C(21)	105.6(5)
O(21)-S(21)-C(21)	103.8(7)
N(21)-S(21)-C(21)	101.4(5)
O(24)-S(22)-O(23)	117.4(8)
O(24)-S(22)-N(21)	112.0(6)
O(23)-S(22)-N(21)	115.7(7)
O(24)-S(22)-C(22)	105.4(7)
O(23)-S(22)-C(22)	103.3(7)
N(21)-S(22)-C(22)	100.5(4)
S(22)-N(21)-S(21)	126.0(6)
F(23)-C(21)-F(22)	109.0(6)
F(23)-C(21)-F(21)	108.9(6)

F(22)-C(21)-F(21)	107.8(5)
F(23)-C(21)-S(21)	110.3(5)
F(22)-C(21)-S(21)	110.3(5)
F(21)-C(21)-S(21)	110.5(5)
F(25)-C(22)-F(26)	108.7(8)
F(25)-C(22)-F(24)	108.7(7)
F(26)-C(22)-F(24)	107.6(9)
F(25)-C(22)-S(22)	110.2(6)
F(26)-C(22)-S(22)	112.8(7)
F(24)-C(22)-S(22)	108.9(7)
O(22A)-S(21A)-O(21A)	118.3(18)
O(22A)-S(21A)-N(21A)	118.7(16)
O(21A)-S(21A)-N(21A)	107.4(16)
O(22A)-S(21A)-C(21A)	104.5(12)
O(21A)-S(21A)-C(21A)	102.7(15)
N(21A)-S(21A)-C(21A)	102.6(10)
O(24A)-S(22A)-O(23A)	118.5(19)
O(24A)-S(22A)-N(21A)	110.2(16)
O(23A)-S(22A)-N(21A)	115.7(16)
O(24A)-S(22A)-C(22A)	105.0(16)
O(23A)-S(22A)-C(22A)	104.9(15)
N(21A)-S(22A)-C(22A)	100.0(10)
S(21A)-N(21A)-S(22A)	126.9(14)
F(23A)-C(21A)-F(21A)	108.9(12)
F(23A)-C(21A)-F(22A)	108.8(13)
F(21A)-C(21A)-F(22A)	111.9(12)
F(23A)-C(21A)-S(21A)	110.9(11)
F(21A)-C(21A)-S(21A)	108.5(11)
F(22A)-C(21A)-S(21A)	107.8(10)
F(25A)-C(22A)-F(24A)	106.3(16)
F(25A)-C(22A)-F(26A)	108.3(18)
F(24A)-C(22A)-F(26A)	108.0(19)
F(25A)-C(22A)-S(22A)	112.8(14)
F(24A)-C(22A)-S(22A)	110.5(17)
F(26A)-C(22A)-S(22A)	110.7(15)
C(36)-C(31)-C(32)	115.3(8)
C(36)-C(31)-B(31)	121.6(8)
C(32)-C(31)-B(31)	123.0(8)
C(33)-C(32)-C(31)	122.5(9)
C(33)-C(32)-H(32)	118.8
C(31)-C(32)-H(32)	118.8
C(32)-C(33)-C(34)	120.4(8)
C(32)-C(33)-C(37)	122.7(8)
C(34)-C(33)-C(37)	116.9(8)
C(35)-C(34)-C(33)	118.5(9)
C(35)-C(34)-H(34)	120.7
C(33)-C(34)-H(34)	120.7
C(34)-C(35)-C(36)	120.3(8)
C(34)-C(35)-C(38)	120.8(8)
C(36)-C(35)-C(38)	118.8(9)
C(35)-C(36)-C(31)	122.8(9)
C(35)-C(36)-H(36)	118.6
C(31)-C(36)-H(36)	118.6
F(31)-C(37)-F(32)	107.2(10)
F(31)-C(37)-F(33)	105.7(10)
F(32)-C(37)-F(33)	105.4(9)
F(31)-C(37)-C(33)	114.1(9)
F(32)-C(37)-C(33)	112.2(10)
F(33)-C(37)-C(33)	111.6(10)
F(34)-C(38)-F(35)	98.7(9)
F(34)-C(38)-F(36)	108.9(8)
F(35)-C(38)-F(36)	104.8(9)
F(34)-C(38)-C(35)	114.5(8)
F(35)-C(38)-C(35)	114.6(10)
F(36)-C(38)-C(35)	114.0(10)
C(42)-C(41)-C(46)	115.7(8)
C(42)-C(41)-B(31)	123.0(8)
C(46)-C(41)-B(31)	121.3(8)
C(43)-C(42)-C(41)	121.5(9)
C(43)-C(42)-H(42)	119.3
C(41)-C(42)-H(42)	119.3
C(42)-C(43)-C(44)	122.1(8)
C(42)-C(43)-C(47)	119.6(8)
C(44)-C(43)-C(47)	118.3(8)
C(45)-C(44)-C(43)	116.7(8)
C(45)-C(44)-H(44)	121.6
C(43)-C(44)-H(44)	121.6
C(44)-C(45)-C(46)	121.6(8)
C(44)-C(45)-C(48)	121.1(8)
C(46)-C(45)-C(48)	117.1(8)
C(45)-C(46)-C(41)	122.3(8)
C(45)-C(46)-H(46)	118.8
C(41)-C(46)-H(46)	118.8
F(43)-C(47)-F(41)	107.4(9)
F(43)-C(47)-F(42)	106.9(9)
F(41)-C(47)-F(42)	106.4(9)
F(43)-C(47)-C(43)	112.3(9)
F(41)-C(47)-C(43)	111.8(8)
F(42)-C(47)-C(43)	111.6(10)
F(45)-C(48)-F(44)	107.8(10)
F(45)-C(48)-F(46)	106.1(9)
F(44)-C(48)-F(46)	106.6(9)
F(45)-C(48)-C(45)	111.6(10)
F(44)-C(48)-C(45)	112.1(9)
F(46)-C(48)-C(45)	112.3(9)
C(56)-C(51)-C(52)	116.0(8)
C(56)-C(51)-B(31)	125.5(8)
C(52)-C(51)-B(31)	118.3(8)
C(53)-C(52)-C(51)	122.3(9)
C(53)-C(52)-H(52)	118.9
C(51)-C(52)-H(52)	118.9
C(52)-C(53)-C(54)	121.0(8)
C(52)-C(53)-C(57)	118.8(8)
C(54)-C(53)-C(57)	120.1(8)
C(55)-C(54)-C(53)	117.5(8)
C(55)-C(54)-H(54)	121.3
C(53)-C(54)-H(54)	121.3
C(54)-C(55)-C(56)	121.5(8)
C(54)-C(55)-C(58)	119.4(8)
C(56)-C(55)-C(58)	118.9(8)
C(55)-C(56)-C(51)	121.6(9)
C(55)-C(56)-H(56)	119.2
C(51)-C(56)-H(56)	119.2
F(53)-C(57)-F(51)	106.9(9)
F(53)-C(57)-F(52)	105.1(8)
F(51)-C(57)-F(52)	103.3(9)
F(53)-C(57)-C(53)	114.9(8)
F(51)-C(57)-C(53)	113.4(10)
F(52)-C(57)-C(53)	112.3(9)
F(54)-C(58)-F(55)	108.5(9)
F(54)-C(58)-F(56)	102.9(9)
F(55)-C(58)-F(56)	105.9(7)
F(54)-C(58)-C(55)	112.4(9)
F(55)-C(58)-C(55)	114.0(8)
F(56)-C(58)-C(55)	112.3(8)
C(62)-C(61)-C(66)	115.2(8)
C(62)-C(61)-B(31)	123.8(8)
C(66)-C(61)-B(31)	121.0(8)
C(63)-C(62)-C(61)	122.2(8)
C(63)-C(62)-H(62)	118.9
C(61)-C(62)-H(62)	118.9
C(64)-C(63)-C(62)	120.7(8)
C(64)-C(63)-C(67)	119.4(8)
C(62)-C(63)-C(67)	119.5(7)
C(63)-C(64)-C(65)	118.8(8)
C(63)-C(64)-H(64)	120.6
C(65)-C(64)-H(64)	120.6
C(64)-C(65)-C(66)	119.6(8)
C(64)-C(65)-C(68)	119.1(8)
C(66)-C(65)-C(68)	120.5(8)
C(65)-C(66)-C(61)	123.1(8)
C(65)-C(66)-H(66)	118.5
C(61)-C(66)-H(66)	118.5
F(63)-C(67)-F(61)	109.3(10)
F(63)-C(67)-F(62)	104.1(8)
F(61)-C(67)-F(62)	104.1(9)
F(63)-C(67)-C(63)	114.9(7)
F(61)-C(67)-C(63)	112.3(9)
F(62)-C(67)-C(63)	111.3(8)
F(65)-C(68)-F(66)	104.5(9)
F(65)-C(68)-F(64)	112.9(10)
F(66)-C(68)-F(64)	101.4(9)
F(65)-C(68)-C(65)	113.2(11)
F(66)-C(68)-C(65)	113.9(9)
F(64)-C(68)-C(65)	110.2(10)
C(41)-B(31)-C(31)	106.6(8)
C(41)-B(31)-C(51)	113.7(8)
C(31)-B(31)-C(51)	109.6(8)
C(41)-B(31)-C(61)	111.5(7)
C(31)-B(31)-C(61)	112.5(7)
C(51)-B(31)-C(61)	103.1(8)
C(36A)-C(31A)-C(32A)	116.1(13)
C(36A)-C(31A)-B(31A)	123.0(13)
C(32A)-C(31A)-B(31A)	120.8(13)
C(33A)-C(32A)-C(31A)	122.2(14)
C(33A)-C(32A)-H(32A)	118.9
C(31A)-C(32A)-H(32A)	118.9
C(32A)-C(33A)-C(34A)	120.8(13)
C(32A)-C(33A)-C(37A)	119.0(14)
C(34A)-C(33A)-C(37A)	120.1(13)
C(35A)-C(34A)-C(33A)	117.6(14)
C(35A)-C(34A)-H(34A)	121.2
C(33A)-C(34A)-H(34A)	121.2
C(34A)-C(35A)-C(36A)	121.9(13)
C(34A)-C(35A)-C(38A)	120.1(13)
C(36A)-C(35A)-C(38A)	118.0(13)
C(35A)-C(36A)-C(31A)	121.3(14)
C(35A)-C(36A)-H(36A)	119.4
C(31A)-C(36A)-H(36A)	119.4
F(32A)-C(37A)-F(31A)	104.8(16)
F(32A)-C(37A)-F(33A)	105.8(15)
F(31A)-C(37A)-F(33A)	105.4(16)
F(32A)-C(37A)-C(33A)	113.8(16)
F(31A)-C(37A)-C(33A)	113.6(15)
F(33A)-C(37A)-C(33A)	112.6(17)
F(34A)-C(38A)-F(36A)	104.9(13)
F(34A)-C(38A)-F(35A)	120.4(15)
F(36A)-C(38A)-F(35A)	102.4(15)
F(34A)-C(38A)-C(35A)	110.8(13)
F(36A)-C(38A)-C(35A)	109.1(15)

F(35A)-C(38A)-C(35A)	108.5(15)
C(42A)-C(41A)-C(46A)	115.3(13)
C(42A)-C(41A)-B(31A)	121.9(13)
C(46A)-C(41A)-B(31A)	122.6(13)
C(41A)-C(42A)-C(43A)	123.1(15)
C(41A)-C(42A)-H(42A)	118.4
C(43A)-C(42A)-H(42A)	118.4
C(42A)-C(43A)-C(44A)	119.3(13)
C(42A)-C(43A)-C(47A)	121.1(13)
C(44A)-C(43A)-C(47A)	119.6(13)
C(45A)-C(44A)-C(43A)	119.0(14)
C(45A)-C(44A)-H(44A)	120.5
C(43A)-C(44A)-H(44A)	120.5
C(44A)-C(45A)-C(46A)	121.0(13)
C(44A)-C(45A)-C(48A)	118.5(13)
C(46A)-C(45A)-C(48A)	120.4(13)
C(45A)-C(46A)-C(41A)	121.9(14)
C(45A)-C(46A)-H(46A)	119.0
C(41A)-C(46A)-H(46A)	119.0
F(43A)-C(47A)-F(41A)	105.2(14)
F(43A)-C(47A)-F(42A)	102.9(15)
F(41A)-C(47A)-F(42A)	104.7(15)
F(43A)-C(47A)-C(43A)	115.6(15)
F(41A)-C(47A)-C(43A)	113.6(16)
F(42A)-C(47A)-C(43A)	113.7(17)
F(45A)-C(48A)-F(46A)	104.7(15)
F(45A)-C(48A)-F(44A)	109.2(16)
F(46A)-C(48A)-F(44A)	105.9(16)
F(45A)-C(48A)-C(45A)	112.7(15)
F(46A)-C(48A)-C(45A)	110.9(16)
F(44A)-C(48A)-C(45A)	112.9(16)
C(56A)-C(51A)-C(52A)	115.3(13)
C(56A)-C(51A)-B(31A)	124.5(14)
C(52A)-C(51A)-B(31A)	119.5(13)
C(53A)-C(52A)-C(51A)	122.8(15)
C(53A)-C(52A)-H(52A)	118.6
C(51A)-C(52A)-H(52A)	118.6
C(52A)-C(53A)-C(54A)	121.3(14)
C(52A)-C(53A)-C(57A)	118.4(13)
C(54A)-C(53A)-C(57A)	120.2(13)
C(55A)-C(54A)-C(53A)	116.8(14)
C(55A)-C(54A)-H(54A)	121.6
C(53A)-C(54A)-H(54A)	121.6
C(54A)-C(55A)-C(56A)	121.8(13)
C(54A)-C(55A)-C(58A)	118.9(13)
C(56A)-C(55A)-C(58A)	119.3(13)
C(55A)-C(56A)-C(51A)	121.9(14)
C(55A)-C(56A)-H(56A)	119.1
C(51A)-C(56A)-H(56A)	119.1
F(53A)-C(57A)-F(52A)	105.4(13)
F(53A)-C(57A)-F(51A)	104.3(13)
F(52A)-C(57A)-F(51A)	106.0(15)
F(53A)-C(57A)-C(53A)	114.4(14)
F(52A)-C(57A)-C(53A)	112.3(17)
F(51A)-C(57A)-C(53A)	113.7(14)
F(54A)-C(58A)-F(55A)	104.3(13)
F(54A)-C(58A)-F(56A)	105.0(15)
F(55A)-C(58A)-F(56A)	104.5(13)
F(54A)-C(58A)-C(55A)	115.3(15)
F(55A)-C(58A)-C(55A)	113.3(13)
F(56A)-C(58A)-C(55A)	113.3(14)
C(62A)-C(61A)-C(66A)	115.2(13)
C(62A)-C(61A)-B(31A)	124.8(13)
C(66A)-C(61A)-B(31A)	119.3(13)
C(61A)-C(62A)-C(63A)	120.7(14)
C(61A)-C(62A)-H(62A)	119.6
C(63A)-C(62A)-H(62A)	119.6
C(64A)-C(63A)-C(62A)	123.5(13)
C(64A)-C(63A)-C(67A)	121.0(13)
C(62A)-C(63A)-C(67A)	115.3(13)
C(63A)-C(64A)-C(65A)	115.8(14)
C(63A)-C(64A)-H(64A)	122.1
C(65A)-C(64A)-H(64A)	122.1
C(64A)-C(65A)-C(66A)	121.0(14)
C(64A)-C(65A)-C(68A)	120.7(13)
C(66A)-C(65A)-C(68A)	117.3(13)
C(65A)-C(66A)-C(61A)	122.0(14)
C(65A)-C(66A)-H(66A)	119.0
C(61A)-C(66A)-H(66A)	119.0
F(63A)-C(67A)-F(61A)	107.4(15)
F(63A)-C(67A)-F(62A)	102.1(10)
F(61A)-C(67A)-F(62A)	108.0(13)
F(63A)-C(67A)-C(63A)	111.8(12)
F(61A)-C(67A)-C(63A)	113.5(14)
F(62A)-C(67A)-C(63A)	113.3(12)
F(65A)-C(68A)-F(66A)	107.2(12)
F(65A)-C(68A)-F(64A)	102.9(14)
F(66A)-C(68A)-F(64A)	115.7(15)
F(65A)-C(68A)-C(65A)	110.3(16)
F(66A)-C(68A)-C(65A)	108.9(16)
F(64A)-C(68A)-C(65A)	111.5(14)
C(41A)-B(31A)-C(31A)	107.0(14)
C(41A)-B(31A)-C(51A)	114.3(13)
C(31A)-B(31A)-C(51A)	110.8(13)
C(41A)-B(31A)-C(61A)	109.5(12)
C(31A)-B(31A)-C(61A)	111.7(13)
C(51A)-B(31A)-C(61A)	103.6(14)
Cl(71)-C(71)-Cl(72)	112.4(3)
Cl(71)-C(71)-H(71A)	109.1
Cl(72)-C(71)-H(71A)	109.1
Cl(71)-C(71)-H(71B)	109.1
Cl(72)-C(71)-H(71B)	109.1
H(71A)-C(71)-H(71B)	107.9

Table S82. Anisotropic displacement parameters (Å^{2}x 10^3) for **[(D$_{12}$-TMC)Fe^{IV}(O)(H$_2$O)]{N(OTf)$_2$}B$_{Ar}$F**.
The anisotropic displacement factor exponent takes the form: $-2p^2[h^2 a^{*2}U^{11} + ... + 2 h k a^* b^* U^{12}]$

	U^{11}	U^{22}	U^{33}	U^{23}	U^{13}	U^{12}
Fe(1)	22(1)	20(1)	27(1)	3(1)	4(1)	1(1)
O(1)	32(1)	25(1)	35(1)	6(1)	8(1)	0(1)
O(2)	20(1)	25(1)	29(1)	4(1)	-1(1)	-1(1)
N(1)	23(1)	23(1)	34(1)	2(1)	6(1)	0(1)
N(2)	30(1)	21(1)	32(1)	1(1)	3(1)	-1(1)
N(3)	27(1)	25(1)	34(1)	5(1)	3(1)	5(1)
N(4)	28(1)	28(1)	30(1)	3(1)	6(1)	0(1)
C(1)	25(2)	26(2)	46(2)	3(1)	5(1)	-3(1)
C(2)	31(2)	29(2)	51(2)	-1(2)	2(2)	-10(1)
C(3)	37(2)	25(2)	45(2)	-4(1)	7(2)	-8(1)
C(4)	38(2)	31(2)	26(2)	0(1)	0(1)	-2(1)
C(5)	37(2)	29(2)	38(2)	-4(1)	7(1)	5(1)
C(6)	35(2)	26(2)	41(2)	2(1)	8(1)	6(1)
C(7)	24(2)	32(2)	44(2)	8(1)	7(1)	6(1)
C(8)	30(2)	36(2)	40(2)	12(1)	0(1)	7(1)
C(9)	35(2)	46(2)	35(2)	8(2)	-5(1)	5(2)
C(10)	38(2)	44(2)	30(2)	6(1)	0(1)	5(2)
C(11)	37(2)	29(2)	34(2)	-6(1)	3(1)	-6(1)
C(12)	33(2)	34(2)	31(2)	2(1)	11(1)	0(1)
C(13)	28(2)	30(2)	35(2)	-2(1)	11(1)	2(1)
C(14)	21(1)	26(1)	40(2)	4(1)	3(1)	1(1)
S(21)	21(2)	26(2)	24(2)	0(1)	0(1)	-1(1)
S(22)	16(1)	27(2)	29(2)	8(1)	-5(1)	-2(1)
F(21)	62(3)	84(4)	30(2)	10(2)	-2(2)	-18(3)
F(22)	36(2)	69(4)	43(3)	-11(2)	-8(2)	-18(2)
F(23)	53(2)	78(4)	57(3)	-32(3)	8(2)	5(2)
F(24)	32(3)	56(5)	39(3)	-1(3)	2(2)	13(2)
F(25)	53(4)	59(3)	39(4)	-11(3)	-17(3)	20(3)
F(26)	53(4)	34(2)	62(6)	-8(3)	3(4)	-6(2)
O(21)	17(3)	29(3)	36(5)	3(3)	-5(2)	1(2)
O(22)	30(3)	27(3)	42(4)	7(3)	4(4)	1(2)
O(23)	21(4)	31(3)	36(5)	8(3)	-3(3)	-6(2)
O(24)	17(3)	45(2)	31(2)	10(2)	-6(2)	2(3)
N(21)	14(2)	28(2)	37(4)	5(2)	-6(2)	-4(2)
C(21)	31(2)	53(4)	35(3)	-6(3)	0(2)	-10(2)
C(22)	29(3)	36(3)	35(3)	-4(3)	-3(2)	5(2)
S(21A)	10(3)	31(4)	25(4)	-1(3)	-3(3)	-6(3)
S(22A)	12(3)	30(3)	21(2)	-5(2)	-4(2)	-4(2)
F(21A)	54(5)	100(10)	21(4)	0(5)	2(4)	-3(6)
F(22A)	39(4)	74(9)	53(8)	-33(6)	-5(4)	-21(5)
F(23A)	61(5)	61(7)	59(8)	-34(5)	1(5)	15(5)
F(24A)	36(7)	43(7)	41(7)	3(5)	1(6)	15(5)
F(25A)	37(6)	57(6)	31(7)	-18(6)	-10(5)	17(5)
F(26A)	46(8)	25(5)	48(9)	-6(5)	-1(7)	-3(4)
O(21A)	11(5)	42(9)	30(8)	8(6)	-7(5)	-6(5)
O(22A)	26(7)	25(7)	30(7)	5(6)	5(7)	5(5)
O(23A)	12(6)	29(6)	22(6)	-2(4)	-5(5)	-6(5)
O(24A)	17(3)	45(2)	31(2)	10(2)	-6(2)	2(3)
N(21A)	13(4)	28(5)	25(6)	5(4)	-6(4)	-5(4)
C(21A)	36(5)	52(6)	32(5)	-13(5)	2(4)	-5(5)
C(22A)	26(6)	32(5)	36(6)	1(4)	-3(4)	2(5)
F(31)	24(4)	39(4)	46(3)	2(3)	-8(3)	-2(3)
F(32)	40(3)	51(4)	35(3)	-5(3)	-1(2)	-6(3)
F(33)	53(5)	39(3)	53(4)	11(3)	-6(3)	-20(3)
F(34)	68(5)	28(2)	66(3)	1(2)	-14(3)	17(2)
F(35)	47(4)	63(6)	61(4)	44(4)	2(4)	3(4)
F(36)	31(4)	48(6)	88(7)	39(5)	17(4)	14(3)
C(31)	29(3)	24(3)	22(4)	7(3)	3(3)	1(3)
C(32)	26(1)	23(2)	24(5)	8(3)	2(3)	2(2)
C(33)	26(4)	25(3)	27(4)	8(3)	2(3)	0(3)
C(34)	31(5)	24(4)	32(4)	2(3)	2(3)	0(3)
C(35)	27(4)	22(3)	33(4)	7(3)	1(3)	6(3)
C(36)	29(6)	27(3)	26(5)	2(3)	2(4)	2(4)
C(37)	30(3)	28(4)	35(4)	1(3)	-4(3)	-6(3)
C(38)	32(4)	29(3)	43(4)	7(3)	0(3)	5(3)
F(41)	31(2)	69(6)	35(4)	20(4)	-5(2)	1(3)
F(42)	62(5)	41(4)	24(3)	1(3)	10(3)	1(3)
F(43)	53(5)	23(3)	48(3)	10(2)	-14(4)	-4(3)
F(44)	29(3)	46(4)	61(6)	8(4)	-2(3)	-8(3)
F(45)	44(4)	91(5)	95(6)	-65(4)	-20(4)	0(4)
F(46)	30(3)	45(3)	45(4)	3(2)	-13(3)	3(2)
C(41)	24(3)	19(3)	24(4)	-3(3)	-2(3)	5(3)
C(42)	23(4)	20(4)	22(4)	-1(3)	-4(3)	5(3)
C(43)	26(3)	21(3)	23(4)	-2(3)	0(3)	1(3)
C(44)	28(3)	19(3)	26(5)	-2(4)	1(3)	2(3)
C(45)	24(3)	26(3)	26(5)	-7(3)	-1(3)	1(2)
C(46)	29(2)	26(2)	21(5)	-2(4)	-5(3)	6(1)
C(47)	25(3)	26(3)	25(3)	4(3)	-3(3)	-4(3)
C(48)	36(3)	30(3)	34(4)	-9(3)	-8(3)	-1(3)
F(51)	111(8)	83(6)	85(6)	-18(5)	33(5)	-49(5)
F(52)	61(4)	76(4)	49(3)	27(3)	9(3)	-8(3)
F(53)	32(3)	75(5)	210(10)	71(6)	29(5)	11(3)
F(54)	104(6)	35(4)	58(5)	-22(3)	-29(4)	4(4)
F(55)	33(2)	35(3)	96(5)	6(4)	-26(3)	6(2)
F(56)	54(3)	31(3)	47(4)	6(3)	7(2)	11(2)
C(51)	27(3)	29(3)	21(4)	0(3)	-1(3)	4(3)
C(52)	28(3)	30(4)	25(6)	2(3)	-5(3)	5(3)
C(53)	29(3)	37(3)	27(4)	6(3)	-2(3)	1(3)
C(54)	25(3)	37(3)	24(6)	-3(3)	1(3)	10(3)
C(55)	30(3)	29(3)	15(5)	-2(3)	-6(3)	6(2)

C(56)	28(2)	25(2)	22(6)	-3(2)	-3(2)	4(1)
C(57)	29(3)	50(4)	48(5)	12(3)	5(3)	4(3)
C(58)	29(3)	30(3)	23(4)	-10(3)	-8(3)	8(2)
F(61)	119(6)	54(5)	24(3)	9(3)	-8(3)	15(4)
F(62)	98(4)	54(3)	62(3)	9(2)	-14(3)	34(3)
F(63)	102(5)	51(3)	43(2)	14(2)	-8(3)	-12(3)
F(64)	165(9)	50(4)	45(3)	-21(3)	-12(4)	-1(6)
F(65)	103(5)	86(6)	189(9)	-58(6)	-29(5)	-31(4)
F(66)	151(7)	34(2)	56(3)	-4(2)	-13(5)	-18(4)
C(61)	22(5)	25(3)	27(3)	2(2)	2(3)	9(3)
C(62)	18(5)	28(3)	20(3)	2(2)	0(3)	6(3)
C(63)	33(4)	30(3)	24(3)	-1(2)	-2(3)	9(3)
C(64)	37(5)	39(4)	26(3)	-4(3)	-4(3)	11(4)
C(65)	52(5)	34(3)	32(3)	-8(3)	1(3)	4(3)
C(66)	36(6)	26(3)	32(3)	-2(2)	7(3)	8(3)
C(67)	76(5)	43(4)	26(3)	2(3)	-8(3)	8(3)
C(68)	107(5)	38(4)	46(4)	-10(3)	-11(4)	-7(4)
B(31)	27(3)	26(3)	24(3)	4(3)	-1(3)	6(3)
F(31A)	31(8)	35(4)	50(6)	8(3)	-12(6)	-13(5)
F(32A)	63(6)	64(10)	48(8)	-24(7)	-8(5)	6(6)
F(33A)	43(6)	48(8)	70(11)	23(7)	-21(6)	-22(6)
F(34A)	84(9)	62(7)	117(9)	-61(7)	-51(7)	48(6)
F(35A)	51(7)	30(4)	39(4)	-6(4)	-6(4)	7(4)
F(36A)	32(4)	38(7)	69(6)	19(5)	1(4)	8(4)
C(31A)	28(5)	25(4)	23(6)	2(4)	0(4)	2(4)
C(32A)	26(1)	23(2)	24(5)	8(3)	2(3)	2(2)
C(33A)	26(5)	26(5)	29(6)	1(4)	2(4)	-3(4)
C(34A)	27(6)	23(5)	29(6)	1(5)	8(4)	3(4)
C(35A)	26(5)	24(5)	32(6)	2(4)	3(4)	1(4)
C(36A)	23(6)	22(5)	25(7)	5(5)	0(5)	2(4)
C(37A)	37(6)	29(6)	36(6)	4(4)	1(4)	-4(4)
C(38A)	37(6)	22(5)	45(5)	-3(4)	-3(5)	11(4)
F(41A)	36(5)	69(9)	35(6)	10(5)	-6(4)	15(5)
F(42A)	79(11)	35(4)	22(6)	1(4)	-2(6)	-11(5)
F(43A)	43(6)	37(7)	60(6)	21(6)	-13(5)	-7(5)
F(44A)	33(6)	68(9)	53(7)	-8(5)	-12(4)	-16(6)
F(45A)	54(7)	36(4)	45(5)	-19(4)	-22(4)	5(4)
F(46A)	44(6)	71(6)	70(12)	7(6)	-23(7)	9(5)
C(41A)	30(4)	22(5)	23(5)	-4(4)	-8(4)	7(4)
C(42A)	23(6)	23(7)	28(6)	-5(5)	-3(4)	-1(5)
C(43A)	30(5)	23(5)	23(5)	-4(4)	-4(4)	4(4)
C(44A)	28(5)	25(6)	30(7)	-8(5)	-4(5)	-1(4)
C(45A)	29(4)	25(5)	27(6)	-5(5)	-5(4)	7(4)
C(46A)	29(2)	26(2)	21(5)	-2(4)	-5(3)	6(1)
C(47A)	29(5)	29(5)	29(5)	-5(4)	-1(4)	-1(4)
C(48A)	32(5)	37(5)	47(7)	-14(5)	-7(4)	4(4)
F(51A)	25(4)	30(4)	75(7)	10(4)	4(4)	3(3)
F(52A)	109(11)	110(10)	54(5)	-5(5)	19(6)	-52(9)
F(53A)	37(4)	59(5)	130(9)	11(6)	-16(5)	-8(3)
F(54A)	48(5)	36(5)	37(5)	-21(4)	-2(4)	-6(4)
F(55A)	33(3)	39(4)	130(11)	-7(8)	-3(6)	14(3)
F(56A)	122(12)	39(6)	47(5)	5(4)	17(7)	36(8)
C(51A)	27(4)	27(4)	23(6)	-1(5)	-5(5)	6(4)
C(52A)	31(5)	31(5)	23(7)	0(5)	-4(5)	0(4)
C(53A)	31(5)	37(4)	34(6)	-4(5)	-3(5)	3(4)
C(54A)	29(5)	40(5)	24(8)	-3(5)	-4(5)	4(4)
C(55A)	30(4)	31(4)	21(8)	-11(4)	-5(4)	9(3)
C(56A)	28(2)	25(2)	22(6)	-3(2)	-3(2)	4(1)
C(57A)	36(5)	42(5)	56(6)	0(5)	10(5)	0(4)
C(58A)	33(4)	34(4)	33(7)	-13(4)	-7(4)	9(4)
F(61A)	126(12)	49(7)	31(4)	-2(4)	-2(5)	-8(8)
F(62A)	92(7)	27(3)	61(5)	3(3)	19(5)	11(4)
F(63A)	41(3)	89(6)	67(5)	29(4)	6(3)	4(3)
F(64A)	189(14)	62(9)	33(5)	-6(4)	-19(6)	-49(10)
F(65A)	93(6)	44(5)	62(5)	-15(4)	-5(4)	-17(4)
F(66A)	103(7)	34(4)	85(6)	-21(4)	6(6)	11(5)
C(61A)	22(7)	31(5)	29(4)	-2(3)	-2(4)	11(4)
C(62A)	27(9)	36(5)	29(5)	-1(4)	0(5)	8(5)
C(63A)	42(7)	36(4)	31(5)	-1(4)	-7(5)	12(4)
C(64A)	46(8)	34(4)	29(5)	-7(4)	-7(5)	19(5)
C(65A)	59(7)	33(4)	35(5)	-6(4)	-4(5)	9(5)
C(66A)	38(8)	25(4)	34(5)	-4(4)	0(5)	19(4)
C(67A)	52(5)	34(4)	33(4)	-4(3)	-2(4)	13(4)
C(68A)	96(6)	39(5)	37(5)	-11(5)	1(5)	1(5)
B(31A)	28(4)	25(4)	28(4)	1(4)	-2(4)	7(4)
Cl(71)	112(1)	74(1)	60(1)	-18(1)	6(1)	12(1)
Cl(72)	75(1)	42(1)	64(1)	-10(1)	10(1)	7(1)
C(71)	81(4)	63(3)	66(3)	-19(2)	18(3)	-16(3)

Table S83. Hydrogen coordinates (x 10^4) and isotropic displacement parameters (Å^{2}x 10^3) for **[(D$_{12}$-TMC)Fe^{IV}(O)(H$_2$O)]{N(OTf)$_2$}B$_{Ar}$F**.

	x	y	z	U(eq)
H(2A)	2710(20)	4254(19)	5217(9)	37
H(2B)	3537(17)	4280(18)	5500(12)	37
H(1A)	-474	3287	5787	39
H(1AB)	341	2796	6026	39
H(2AA)	-261	2389	5303	44
H(2AB)	174	3035	5054	44
H(3A)	1426	2035	5472	43
H(3AB)	1187	2089	4929	43
H(5A)	2916	1948	4922	41
H(5AB)	3602	2603	4867	41
H(6A)	4353	1977	5452	41
H(6AB)	3283	1897	5709	41
H(8A)	5037	2446	6207	43
H(8AB)	3913	2249	6365	43
H(9A)	4819	3025	6875	46
H(9AB)	4684	3573	6475	46
H(10A)	2967	2918	6863	44
H(10B)	3401	3600	7074	44
H(12A)	1575	3965	6925	39
H(12B)	1405	3240	6704	39
H(13A)	955	4480	6263	37
H(13B)	141	3915	6390	37
H(32)	7785	3951	6879	29
H(34)	9015	2096	6827	35
H(36)	10199	3477	7712	33
H(42)	8724	5277	6743	26
H(44)	11514	6154	6595	29
H(46)	10981	4967	7701	31
H(52)	7184	3987	7848	33
H(54)	4974	5394	7679	34
H(56)	7939	5828	7400	30
H(62)	8992	3606	8296	26
H(64)	10009	4632	9425	41
H(66)	9785	5543	8177	37
H(32A)	7716	3868	6958	29
H(34A)	9170	2087	6853	32
H(36A)	10305	3522	7714	28
H(42A)	8778	5213	6762	30
H(44A)	11534	6125	6677	33
H(46A)	10928	4945	7768	31
H(52A)	7047	3922	7721	34
H(54A)	4938	5405	7775	38
H(56A)	7888	5818	7452	30
H(62A)	8772	3637	8353	37
H(64A)	9826	4719	9460	43
H(66A)	9385	5584	8202	39
H(71A)	5908	2859	3249	84
H(71B)	6214	2207	3539	84

Table S84. Hydrogen bonds for **$[(D_{12}\text{-TMC})Fe^{IV}(O)(H_2O)]\{N(OTf)_2\}B_{Ar}F$** [Å and °].

D-H...A	d(D-H)	d(H...A)	d(D...A)	<(DHA)
O(2)-H(2A)...O(22^a)#1	0.844(18)	2.62(4)	3.030(12)	111(3)
O(2)-H(2A)...O(23^a)#1	0.844(18)	1.95(3)	2.767(17)	162(4)
O(2)-H(2A)...O(23A^b)#1	0.844(18)	2.03(5)	2.86(4)	167(4)
O(2)-H(2B)...O(21^a)	0.839(18)	1.92(2)	2.721(9)	158(4)
O(2)-H(2B)...O(21A^b)	0.839(18)	2.00(3)	2.80(2)	160(4)

Symmetry transformations used to generate equivalent atoms: #1 -x+1,-y+1,-z+1

[(TMC)Co^{II}(OTf)][Al{OC(CF_3)$_3$}$_4$]

The crystal structure of [(TMC)Co^{II}(OTf)][Al{OC(CF_3)$_3$}$_4$] was solved in the monoclinic space group *C*2/*c* and the asymmetric unit contains one cationic complex molecule with coordinated triflate anion and two half of the Al{OC(CF_3)$_3$}$_4$ anions. The ligand, the triflate anion and all of the OC(CF_3)$_3$ groups were found to be fully disordered. The disorder was refined with the help of same distance restraints on 1,2 and 1,3 distances, similarity restraints on anisotropic displacement parameters and advanced rigid bond restraints. Some atoms with disordered positions were set to have the same anisotropic displacement factor as their equivalent or neighboring atom.

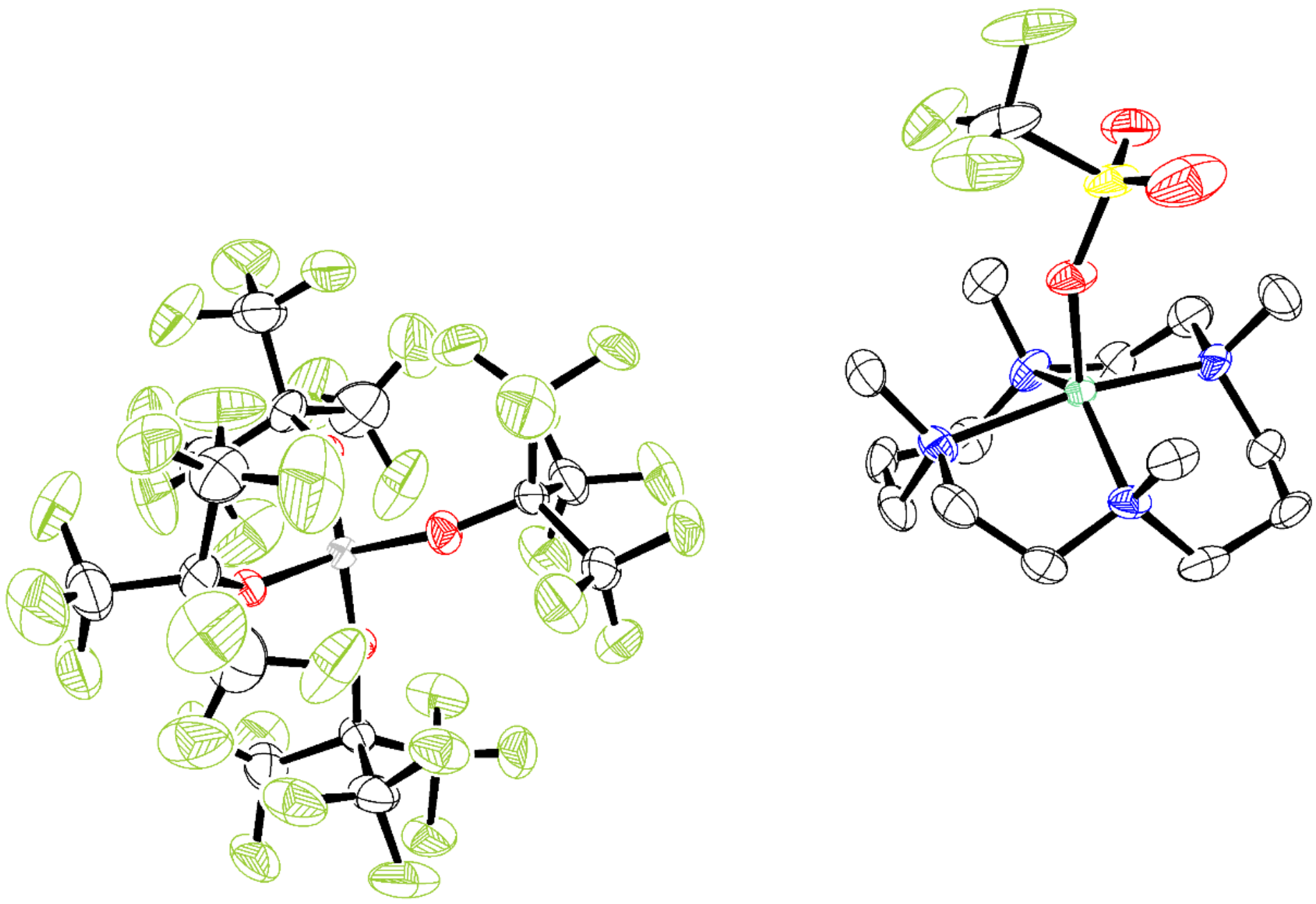

Figure S17. Molecular structure of **[(TMC)Co^{II}(OTf)][Al{OC(CF_3)$_3$}$_4$]**.

Table S85. Crystal data and structure refinement for **[(TMC)Co^{II}(OTf)][Al{OC(CF_3)$_3$}$_4$]**.

CCDC No	2062719	
Empirical formula	$C_{31}H_{32}AlCoF_{39}N_4O_7S$	
Formula weight	1431.57	
Temperature	100(2) K	
Wavelength	0.71073 Å	
Crystal system	Monoclinic	
Space group	*C*2/*c*	
Unit cell dimensions	a = 27.0120(7) Å	α = 90°
	b = 25.3757(7) Å	β = 132.6389(10)°
	c = 19.7465(5) Å	γ = 90°
Volume	9957.0(5) Å^3	
Z	8	
Density (calculated)	1.910 Mg/m^3	
Absorption coefficient	0.600 mm^{-1}	
F(000)	5672	
Crystal size	0.163 x 0.132 x 0.109 mm^3	
Theta range for data collection	2.202 to 26.732°.	
Index ranges	-34 ≤ h ≤ 34, -32 ≤ k ≤ 32, -24 ≤ l ≤ 24	
Reflections collected	251038	
Independent reflections	10582 [R(int) = 0.0561]	
Completeness to theta = 25.242°	99.9 %	
Absorption correction	Semi-empirical from equivalents	
Refinement method	Full-matrix least-squares on *F*^{2}	
Data / restraints / parameters	10582 / 5173 / 1450	
Goodness-of-fit on *F*^{2}	1.028	
Final R indices [I>2σ(I)]	R1 = 0.0548, wR2 = 0.1427	
R indices (all data)	R1 = 0.0659, wR2 = 0.1537	
Largest diff. peak and hole	0.982 and -0.740 eÅ^{-3}	

Table S86. Atomic coordinates (x 10[4]) and equivalent isotropic displacement parameters (Å^{2}x 10^3) for **[(TMC)Co^{II}(OTf)][Al{OC(CF$_3$)$_3$}$_4$]**. U(eq) is defined as one third of the trace of the orthogonalized U^{ij} tensor.

	x	y	z	U(eq)
Co(1)	8116(1)	4368(1)	7744(1)	22(1)
N(1)	7294(4)	4891(3)	6621(5)	30(2)
N(2)	8646(3)	4500(2)	7313(4)	34(1)
N(3)	8848(3)	3776(2)	8601(5)	33(1)
N(4)	7297(3)	3923(2)	7381(4)	29(1)
C(1)	7117(6)	4806(6)	5723(7)	43(2)
C(2)	7687(5)	4928(4)	5774(7)	45(2)
C(3)	8239(4)	4524(3)	6299(6)	45(2)
C(4)	9030(3)	4995(4)	7759(6)	44(2)
C(5)	9134(3)	4051(3)	7714(5)	42(1)
C(6)	9407(3)	3913(3)	8652(5)	46(2)
C(7)	9149(4)	3748(3)	9566(5)	51(2)
C(8)	8593(4)	3243(3)	8190(6)	38(2)
C(9)	7987(6)	3070(5)	8036(10)	44(3)
C(10)	7337(6)	3347(4)	7225(9)	39(2)
C(11)	7289(4)	3981(3)	8125(6)	38(1)
C(12)	6659(3)	4143(3)	6493(4)	33(1)
C(13)	6696(3)	4727(3)	6473(4)	40(1)
C(14)	7379(5)	5465(3)	6832(7)	40(2)
N(1A)	7261(6)	4715(4)	6563(8)	50(3)
N(2A)	8794(4)	4705(3)	7626(6)	34(2)
N(3A)	8790(4)	3690(3)	8264(6)	32(2)
N(4A)	7448(4)	3926(4)	7777(6)	45(2)
C(1A)	7260(8)	4734(8)	5798(10)	54(3)
C(2A)	7858(7)	4984(7)	6011(9)	55(3)
C(3A)	8501(6)	4693(4)	6665(7)	46(2)
C(4A)	9043(5)	5251(4)	8000(7)	48(2)
C(5A)	9411(4)	4365(3)	8211(6)	45(2)
C(6A)	9212(4)	3796(3)	8058(8)	42(2)
C(7A)	9228(5)	3658(4)	9282(7)	49(3)
C(8A)	8448(6)	3179(4)	7832(7)	39(2)
C(9A)	7921(8)	3031(7)	7872(14)	49(3)
C(10A)	7315(9)	3393(6)	7340(13)	51(4)
C(11A)	7614(7)	3858(6)	8666(8)	74(4)
C(12A)	6807(4)	4237(4)	7117(6)	60(2)
C(13A)	6691(4)	4418(5)	6295(6)	59(2)
C(14A)	7245(8)	5266(5)	6811(10)	74(5)
S(1)	8840(1)	4990(1)	9718(2)	43(1)
F(1)	9087(3)	5880(3)	10522(4)	87(2)
F(2)	8054(2)	5753(2)	9357(4)	97(2)
F(3)	8747(4)	5952(2)	9200(5)	86(2)
O(1)	8336(4)	4853(4)	8738(5)	38(2)
O(2)	9511(2)	4982(2)	10086(3)	57(1)
O(3)	8712(4)	4747(3)	10238(4)	86(2)
C(15)	8645(5)	5687(3)	9662(6)	59(2)
S(1A)	8517(2)	5080(2)	9453(3)	38(1)
F(1A)	8758(7)	6013(4)	10138(9)	82(3)
F(2A)	9192(4)	5862(3)	9559(6)	54(2)
F(3A)	8159(4)	6011(4)	8682(7)	97(2)
O(1A)	8477(8)	4883(8)	8723(10)	33(3)
O(2A)	7865(6)	5068(4)	9175(7)	86(2)
O(3A)	9061(5)	4912(4)	10357(5)	66(3)
C(15A)	8668(8)	5783(5)	9449(11)	48(3)
Al(21)	5000	7312(1)	2500	20(1)
F(21)	5659(2)	7014(2)	5194(2)	67(1)
F(22)	6628(3)	6724(3)	5770(4)	64(2)
F(23)	6216(2)	7432(1)	4951(2)	68(1)
F(24)	6582(3)	6025(3)	4672(5)	83(2)
F(25)	6686(1)	6811(2)	4397(2)	65(1)
F(26)	5958(2)	6296(2)	3276(2)	57(1)
F(27)	5198(2)	5793(1)	3491(2)	62(1)
F(28)	4786(2)	6276(2)	3901(3)	62(1)
F(29)	5733(5)	5894(4)	4929(6)	57(2)
O(21)	5230(5)	6933(4)	3399(6)	34(1)
C(21)	5696(3)	6634(2)	4129(4)	30(1)
C(22)	6053(3)	6951(2)	5032(4)	54(2)
C(23)	6248(3)	6438(2)	4126(3)	47(1)
C(24)	5353(2)	6136(2)	4113(3)	42(1)
F(21A)	5823(7)	7393(4)	4944(9)	75(4)
F(22A)	6458(12)	6709(11)	5740(14)	80(7)
F(23A)	6594(5)	7181(5)	4939(8)	75(4)
F(24A)	6595(6)	6047(6)	4705(10)	30(3)
F(25A)	6183(8)	6524(6)	3562(11)	62(3)
F(26A)	5625(6)	5870(4)	3408(8)	66(3)
F(27A)	4701(5)	6069(5)	3474(8)	56(3)
F(28A)	5067(7)	6596(6)	4617(9)	79(4)
F(29A)	5614(16)	5894(11)	4853(19)	51(5)
O(21A)	5200(18)	6930(15)	3360(20)	34(1)
C(21A)	5640(8)	6625(6)	4118(13)	31(3)
C(22A)	6143(9)	6988(6)	4965(13)	55(4)
C(23A)	6022(7)	6264(6)	3964(9)	44(3)
C(24A)	5244(7)	6287(6)	4258(10)	44(3)
F(31)	5042(4)	8347(5)	1590(7)	88(3)
F(32)	5524(6)	7762(4)	1310(7)	118(4)
F(33)	5863(3)	8590(2)	1753(4)	70(2)
F(34)	6331(6)	7135(3)	2574(8)	87(3)
F(35)	6825(3)	7825(2)	2595(5)	94(2)
F(36)	7123(2)	7522(2)	3826(4)	86(1)
F(37)	6935(9)	8609(6)	3580(16)	104(2)
F(38)	6761(3)	8217(2)	4359(3)	76(1)

F(39)	5991(3)	8804(1)	3231(4)	69(1)
O(31)	5729(4)	7675(4)	2972(6)	26(1)
C(31)	6071(4)	7962(3)	2833(5)	40(2)
C(32)	5613(4)	8182(3)	1841(5)	60(2)
C(33)	6605(4)	7586(3)	2966(6)	71(2)
C(34)	6446(4)	8410(3)	3542(5)	76(1)
F(31A)	6458(5)	7925(4)	2062(8)	85(3)
F(32A)	5422(4)	7734(4)	1172(6)	46(2)
F(33A)	6221(9)	7215(5)	2370(13)	76(4)
F(34A)	6869(16)	8628(10)	3560(30)	104(2)
F(35A)	7040(4)	7866(4)	4057(7)	86(1)
F(36A)	6456(5)	8392(4)	4154(6)	76(1)
F(37A)	5676(6)	8772(3)	1745(8)	64(2)
F(38A)	4865(5)	8378(5)	1463(10)	50(2)
F(39A)	5434(5)	8846(2)	2559(6)	63(2)
O(31A)	5605(8)	7701(8)	2795(12)	39(3)
C(31A)	5946(6)	8014(5)	2682(7)	41(2)
C(32A)	6034(4)	7702(3)	2077(7)	47(2)
C(33A)	6595(5)	8217(4)	3638(8)	76(3)
C(34A)	5524(5)	8504(3)	2150(7)	43(2)
Al(41)	5000	6692(1)	7500	26(1)
F(41)	5900(2)	7738(2)	8016(2)	44(1)
F(42)	5530(2)	8214(1)	8490(2)	43(1)
F(43)	6595(2)	8104(2)	9351(4)	53(1)
F(44)	6812(2)	7026(2)	9238(3)	51(1)
F(45)	6523(2)	6549(1)	9819(3)	46(1)
F(46)	7120(2)	7243(2)	10528(3)	53(1)
F(47)	6277(2)	7908(1)	10406(2)	51(1)
F(48)	5238(1)	7683(1)	9374(2)	40(1)
F(49)	5977(2)	7114(1)	10347(2)	40(1)
O(41)	5406(1)	7076(1)	8480(2)	28(1)
C(41)	5949(2)	7364(1)	9159(2)	27(1)
C(42)	6001(2)	7862(2)	8756(3)	36(1)
C(43)	6614(2)	7044(2)	9698(3)	38(1)
C(44)	5860(2)	7520(2)	9833(2)	34(1)
F(41A)	6100(20)	7685(17)	8060(30)	52(7)
F(42A)	5481(15)	8066(11)	8240(20)	47(7)
F(43A)	6570(20)	8099(19)	9310(30)	53(1)
F(44A)	7065(14)	7066(14)	9480(20)	40(6)
F(45A)	6797(14)	6569(9)	10050(20)	39(6)
F(46A)	7160(20)	7345(17)	10600(20)	30(7)
F(47A)	6037(12)	7849(9)	10049(19)	39(5)
F(48A)	5122(8)	7466(9)	8938(15)	39(5)
F(49A)	5911(18)	7007(9)	10106(17)	31(6)
O(41A)	5660(13)	6904(9)	8459(13)	34(5)
C(41A)	6054(10)	7281(8)	9102(14)	31(4)
C(42A)	6051(14)	7793(10)	8670(20)	41(5)
C(43A)	6789(11)	7064(10)	9834(17)	32(5)
C(44A)	5789(10)	7404(9)	9565(15)	32(4)
F(51)	6511(5)	6174(3)	6917(7)	60(2)
F(52)	6162(6)	6884(3)	7090(9)	70(2)
F(53)	6974(4)	6471(3)	8246(5)	67(2)
F(54)	6682(4)	5357(2)	7976(5)	59(2)
F(55)	6698(4)	5749(3)	8932(5)	65(2)
F(56)	5874(5)	5234(3)	7930(7)	76(2)
F(57)	5090(6)	6220(5)	5771(8)	88(2)
F(58)	5497(5)	5439(4)	6193(6)	76(2)
F(59)	4808(5)	5706(5)	6344(7)	99(3)
O(51)	5670(6)	6380(5)	7680(8)	44(2)
C(51)	5897(5)	6064(3)	7379(6)	46(2)
C(52)	6378(6)	6403(4)	7385(7)	57(2)
C(53)	6298(5)	5597(3)	8072(6)	53(2)
C(54)	5322(6)	5843(5)	6426(7)	88(2)
F(51A)	6288(5)	5999(4)	6770(7)	67(2)
F(52A)	6007(8)	6724(4)	6964(11)	90(3)
F(53A)	6802(5)	6215(5)	8155(6)	90(3)
F(54A)	6290(5)	5165(4)	7651(6)	79(3)
F(55A)	6325(5)	5553(3)	8654(4)	70(2)
F(56A)	5427(6)	5160(3)	7526(7)	89(2)
F(57A)	4921(7)	6275(5)	5676(9)	88(2)
F(58A)	5171(5)	5444(4)	5902(6)	89(3)
F(59A)	4520(5)	5795(5)	6040(7)	92(3)
O(51A)	5515(7)	6286(5)	7609(8)	52(3)
C(51A)	5665(5)	5965(4)	7221(6)	52(2)
C(52A)	6205(6)	6236(5)	7287(7)	64(3)
C(53A)	5937(6)	5445(4)	7770(7)	60(2)
C(54A)	5079(6)	5867(5)	6194(6)	73(3)

Table S87. Bond lengths [Å] and angles [°] for **[(TMC)Co^{II}(OTf)][Al{OC(CF$_3$)$_3$}$_4$]**.

Co(1)-O(1A)	1.961(13)	C(8A)-H(8AC)	0.9900
Co(1)-O(1)	2.036(7)	C(9A)-C(10A)	1.517(14)
Co(1)-N(1A)	2.062(12)	C(9A)-H(9AA)	0.9900
Co(1)-N(3)	2.119(6)	C(9A)-H(9AC)	0.9900
Co(1)-N(2)	2.129(5)	C(10A)-H(10C)	0.9900
Co(1)-N(4)	2.130(6)	C(10A)-H(10D)	0.9900
Co(1)-N(4A)	2.164(8)	C(11A)-H(11D)	0.9800
Co(1)-N(2A)	2.169(7)	C(11A)-H(11E)	0.9800
Co(1)-N(3A)	2.190(7)	C(11A)-H(11F)	0.9800
Co(1)-N(1)	2.223(8)	C(12A)-C(13A)	1.502(13)
N(1)-C(14)	1.488(9)	C(12A)-H(12C)	0.9900
N(1)-C(13)	1.492(8)	C(12A)-H(12D)	0.9900
N(1)-C(1)	1.508(9)	C(13A)-H(13C)	0.9900
N(2)-C(4)	1.482(9)	C(13A)-H(13D)	0.9900
N(2)-C(3)	1.495(9)	C(14A)-H(14D)	0.9800
N(2)-C(5)	1.498(8)	C(14A)-H(14E)	0.9800
N(3)-C(7)	1.484(9)	C(14A)-H(14F)	0.9800
N(3)-C(6)	1.485(8)	S(1)-O(2)	1.424(5)
N(3)-C(8)	1.486(8)	S(1)-O(3)	1.426(5)
N(4)-C(11)	1.491(8)	S(1)-O(1)	1.466(7)
N(4)-C(12)	1.503(8)	S(1)-C(15)	1.829(9)
N(4)-C(10)	1.513(9)	F(1)-C(15)	1.342(9)
C(1)-C(2)	1.504(11)	F(2)-C(15)	1.278(10)
C(1)-H(1A)	0.9900	F(3)-C(15)	1.305(10)
C(1)-H(1AB)	0.9900	S(1A)-O(3A)	1.405(8)
C(2)-C(3)	1.502(10)	S(1A)-O(2A)	1.448(10)
C(2)-H(2A)	0.9900	S(1A)-O(1A)	1.460(11)
C(2)-H(2AB)	0.9900	S(1A)-C(15A)	1.830(13)
C(3)-H(3A)	0.9900	F(1A)-C(15A)	1.342(14)
C(3)-H(3AB)	0.9900	F(2A)-C(15A)	1.295(15)
C(4)-H(4A)	0.9800	F(3A)-C(15A)	1.304(16)
C(4)-H(4B)	0.9800	Al(21)-O(31A)	1.639(14)
C(4)-H(4C)	0.9800	Al(21)-O(31A)#1	1.639(14)
C(5)-C(6)	1.498(9)	Al(21)-O(21A)	1.70(3)
C(5)-H(5A)	0.9900	Al(21)-O(21)#1	1.725(8)
C(5)-H(5AB)	0.9900	Al(21)-O(21)	1.725(8)
C(6)-H(6A)	0.9900	Al(21)-O(31)	1.763(8)
C(6)-H(6AB)	0.9900	Al(21)-O(31)#1	1.763(8)
C(7)-H(7A)	0.9800	F(21)-C(22)	1.312(7)
C(7)-H(7B)	0.9800	F(22)-C(22)	1.342(7)
C(7)-H(7C)	0.9800	F(23)-C(22)	1.343(6)
C(8)-C(9)	1.513(11)	F(24)-C(23)	1.326(7)
C(8)-H(8A)	0.9900	F(25)-C(23)	1.313(6)
C(8)-H(8AB)	0.9900	F(26)-C(23)	1.334(6)
C(9)-C(10)	1.528(11)	F(27)-C(24)	1.320(6)
C(9)-H(9A)	0.9900	F(28)-C(24)	1.332(6)
C(9)-H(9AB)	0.9900	F(29)-C(24)	1.337(7)
C(10)-H(10A)	0.9900	O(21)-C(21)	1.333(6)
C(10)-H(10B)	0.9900	C(21)-C(24)	1.556(6)
C(11)-H(11A)	0.9800	C(21)-C(22)	1.557(6)
C(11)-H(11B)	0.9800	C(21)-C(23)	1.577(7)
C(11)-H(11C)	0.9800	F(21A)-C(22A)	1.324(14)
C(12)-C(13)	1.488(9)	F(22A)-C(22A)	1.344(14)
C(12)-H(12A)	0.9900	F(23A)-C(22A)	1.344(14)
C(12)-H(12B)	0.9900	F(24A)-C(23A)	1.330(13)
C(13)-H(13A)	0.9900	F(25A)-C(23A)	1.312(13)
C(13)-H(13B)	0.9900	F(26A)-C(23A)	1.326(14)
C(14)-H(14A)	0.9800	F(27A)-C(24A)	1.333(13)
C(14)-H(14B)	0.9800	F(28A)-C(24A)	1.345(13)
C(14)-H(14C)	0.9800	F(29A)-C(24A)	1.335(14)
N(1A)-C(13A)	1.454(13)	O(21A)-C(21A)	1.353(14)
N(1A)-C(14A)	1.492(12)	C(21A)-C(24A)	1.534(13)
N(1A)-C(1A)	1.511(12)	C(21A)-C(22A)	1.554(13)
N(2A)-C(3A)	1.481(10)	C(21A)-C(23A)	1.557(13)
N(2A)-C(4A)	1.498(10)	F(31)-C(32)	1.330(10)
N(2A)-C(5A)	1.500(10)	F(32)-C(32)	1.396(10)
N(3A)-C(6A)	1.471(11)	F(33)-C(32)	1.310(8)
N(3A)-C(8A)	1.482(9)	F(34)-C(33)	1.297(11)
N(3A)-C(7A)	1.492(11)	F(35)-C(33)	1.358(8)
N(4A)-C(11A)	1.498(11)	F(36)-C(33)	1.284(9)
N(4A)-C(12A)	1.504(12)	F(37)-C(34)	1.369(12)
N(4A)-C(10A)	1.510(13)	F(38)-C(34)	1.307(8)
C(1A)-C(2A)	1.506(15)	F(39)-C(34)	1.365(9)
C(1A)-H(1AA)	0.9900	O(31)-C(31)	1.342(8)
C(1A)-H(1AC)	0.9900	C(31)-C(34)	1.536(9)
C(2A)-C(3A)	1.480(13)	C(31)-C(32)	1.548(8)
C(2A)-H(2AA)	0.9900	C(31)-C(33)	1.595(9)
C(2A)-H(2AC)	0.9900	F(31A)-C(32A)	1.294(10)
C(3A)-H(3AA)	0.9900	F(32A)-C(32A)	1.389(11)
C(3A)-H(3AC)	0.9900	F(33A)-C(32A)	1.311(12)
C(4A)-H(4AA)	0.9800	F(34A)-C(33A)	1.350(13)
C(4A)-H(4AB)	0.9800	F(35A)-C(33A)	1.256(12)
C(4A)-H(4AC)	0.9800	F(36A)-C(33A)	1.374(13)
C(5A)-C(6A)	1.498(10)	F(37A)-C(34A)	1.310(10)
C(5A)-H(5AA)	0.9900	F(38A)-C(34A)	1.356(12)
C(5A)-H(5AC)	0.9900	F(39A)-C(34A)	1.316(9)
C(6A)-H(6AA)	0.9900	O(31A)-C(31A)	1.346(12)
C(6A)-H(6AC)	0.9900	C(31A)-C(34A)	1.524(11)
C(7A)-H(7AA)	0.9800	C(31A)-C(33A)	1.555(12)
C(7A)-H(7AB)	0.9800	C(31A)-C(32A)	1.583(12)
C(7A)-H(7AC)	0.9800	Al(41)-O(41A)	1.575(19)
C(8A)-C(9A)	1.524(14)	Al(41)-O(41A)#2	1.575(19)
C(8A)-H(8AA)	0.9900	Al(41)-O(51A)	1.623(10)

Al(41)-O(51A)#2	1.623(10)
Al(41)-O(41)	1.738(2)
Al(41)-O(41)#2	1.739(2)
Al(41)-O(51)#2	1.776(10)
Al(41)-O(51)	1.776(10)
F(41)-C(42)	1.331(5)
F(42)-C(42)	1.335(4)
F(43)-C(42)	1.332(4)
F(44)-C(43)	1.331(4)
F(45)-C(43)	1.332(4)
F(46)-C(43)	1.332(5)
F(47)-C(44)	1.337(4)
F(48)-C(44)	1.325(4)
F(49)-C(44)	1.326(4)
O(41)-C(41)	1.349(4)
C(41)-C(42)	1.550(4)
C(41)-C(44)	1.555(5)
C(41)-C(43)	1.559(5)
F(41A)-C(42A)	1.319(15)
F(42A)-C(42A)	1.346(15)
F(43A)-C(42A)	1.335(15)
F(44A)-C(43A)	1.331(15)
F(45A)-C(43A)	1.320(15)
F(46A)-C(43A)	1.321(15)
F(47A)-C(44A)	1.332(15)
F(48A)-C(44A)	1.336(15)
F(49A)-C(44A)	1.337(15)
O(41A)-C(41A)	1.352(15)
C(41A)-C(44A)	1.527(14)
C(41A)-C(42A)	1.556(14)
C(41A)-C(43A)	1.564(14)
F(51)-C(52)	1.330(9)
F(52)-C(52)	1.307(10)
F(53)-C(52)	1.344(10)
F(54)-C(53)	1.322(9)
F(55)-C(53)	1.311(9)
F(56)-C(53)	1.344(10)
F(57)-C(54)	1.371(10)
F(58)-C(54)	1.336(10)
F(59)-C(54)	1.331(11)
O(51)-C(51)	1.367(9)
C(51)-C(54)	1.519(11)
C(51)-C(52)	1.553(10)
C(51)-C(53)	1.563(9)
F(51A)-C(52A)	1.329(10)
F(52A)-C(52A)	1.328(11)
F(53A)-C(52A)	1.341(10)
F(54A)-C(53A)	1.333(9)
F(55A)-C(53A)	1.320(10)
F(56A)-C(53A)	1.325(12)
F(57A)-C(54A)	1.307(11)
F(58A)-C(54A)	1.318(10)
F(59A)-C(54A)	1.335(12)
O(51A)-C(51A)	1.356(10)
C(51A)-C(54A)	1.528(10)
C(51A)-C(52A)	1.536(11)
C(51A)-C(53A)	1.541(10)

O(1A)-Co(1)-N(1A)	104.9(7)
O(1)-Co(1)-N(3)	98.8(4)
O(1)-Co(1)-N(2)	117.0(3)
N(3)-Co(1)-N(2)	85.1(2)
O(1)-Co(1)-N(4)	101.7(3)
N(3)-Co(1)-N(4)	95.1(2)
N(2)-Co(1)-N(4)	140.8(3)
O(1A)-Co(1)-N(4A)	100.6(5)
N(1A)-Co(1)-N(4A)	86.4(4)
O(1A)-Co(1)-N(2A)	87.2(5)
N(1A)-Co(1)-N(2A)	95.2(4)
N(4A)-Co(1)-N(2A)	171.4(3)
O(1A)-Co(1)-N(3A)	111.2(6)
N(1A)-Co(1)-N(3A)	143.6(4)
N(4A)-Co(1)-N(3A)	91.4(3)
N(2A)-Co(1)-N(3A)	82.3(3)
O(1)-Co(1)-N(1)	92.4(4)
N(3)-Co(1)-N(1)	168.8(3)
N(2)-Co(1)-N(1)	89.7(2)
N(4)-Co(1)-N(1)	82.6(2)
C(14)-N(1)-C(13)	105.7(6)
C(14)-N(1)-C(1)	109.5(7)
C(13)-N(1)-C(1)	106.9(7)
C(14)-N(1)-Co(1)	116.4(6)
C(13)-N(1)-Co(1)	104.3(4)
C(1)-N(1)-Co(1)	113.2(6)
C(4)-N(2)-C(3)	110.2(5)
C(4)-N(2)-C(5)	108.6(6)
C(3)-N(2)-C(5)	109.0(5)
C(4)-N(2)-Co(1)	106.5(4)
C(3)-N(2)-Co(1)	117.2(4)
C(5)-N(2)-Co(1)	104.9(4)
C(7)-N(3)-C(6)	106.1(6)
C(7)-N(3)-C(8)	107.7(6)
C(6)-N(3)-C(8)	108.9(6)
C(7)-N(3)-Co(1)	117.6(5)
C(6)-N(3)-Co(1)	104.2(4)
C(8)-N(3)-Co(1)	111.9(5)
C(11)-N(4)-C(12)	110.1(5)
C(11)-N(4)-C(10)	110.2(8)
C(12)-N(4)-C(10)	107.7(6)
C(11)-N(4)-Co(1)	108.8(4)
C(12)-N(4)-Co(1)	107.8(4)
C(10)-N(4)-Co(1)	112.3(7)
C(2)-C(1)-N(1)	113.4(9)
C(2)-C(1)-H(1A)	108.9
N(1)-C(1)-H(1A)	108.9
C(2)-C(1)-H(1AB)	108.9
N(1)-C(1)-H(1AB)	108.9
H(1A)-C(1)-H(1AB)	107.7
C(3)-C(2)-C(1)	113.5(8)
C(3)-C(2)-H(2A)	108.9
C(1)-C(2)-H(2A)	108.9
C(3)-C(2)-H(2AB)	108.9
C(1)-C(2)-H(2AB)	108.9
H(2A)-C(2)-H(2AB)	107.7
N(2)-C(3)-C(2)	116.0(6)
N(2)-C(3)-H(3A)	108.3
C(2)-C(3)-H(3A)	108.3
N(2)-C(3)-H(3AB)	108.3
C(2)-C(3)-H(3AB)	108.3
H(3A)-C(3)-H(3AB)	107.4
N(2)-C(4)-H(4A)	109.5
N(2)-C(4)-H(4B)	109.5
H(4A)-C(4)-H(4B)	109.5
N(2)-C(4)-H(4C)	109.5
H(4A)-C(4)-H(4C)	109.5
H(4B)-C(4)-H(4C)	109.5
C(6)-C(5)-N(2)	110.7(5)
C(6)-C(5)-H(5A)	109.5
N(2)-C(5)-H(5A)	109.5
C(6)-C(5)-H(5AB)	109.5
N(2)-C(5)-H(5AB)	109.5
H(5A)-C(5)-H(5AB)	108.1
N(3)-C(6)-C(5)	110.1(5)
N(3)-C(6)-H(6A)	109.6
C(5)-C(6)-H(6A)	109.6
N(3)-C(6)-H(6AB)	109.6
C(5)-C(6)-H(6AB)	109.6
H(6A)-C(6)-H(6AB)	108.2
N(3)-C(7)-H(7A)	109.5
N(3)-C(7)-H(7B)	109.5
H(7A)-C(7)-H(7B)	109.5
N(3)-C(7)-H(7C)	109.5
H(7A)-C(7)-H(7C)	109.5
H(7B)-C(7)-H(7C)	109.5
N(3)-C(8)-C(9)	114.4(8)
N(3)-C(8)-H(8A)	108.7
C(9)-C(8)-H(8A)	108.7
N(3)-C(8)-H(8AB)	108.7
C(9)-C(8)-H(8AB)	108.7
H(8A)-C(8)-H(8AB)	107.6
C(8)-C(9)-C(10)	113.3(10)
C(8)-C(9)-H(9A)	108.9
C(10)-C(9)-H(9A)	108.9
C(8)-C(9)-H(9AB)	108.9
C(10)-C(9)-H(9AB)	108.9
H(9A)-C(9)-H(9AB)	107.7
N(4)-C(10)-C(9)	115.0(10)
N(4)-C(10)-H(10A)	108.5
C(9)-C(10)-H(10A)	108.5
N(4)-C(10)-H(10B)	108.5
C(9)-C(10)-H(10B)	108.5
H(10A)-C(10)-H(10B)	107.5
N(4)-C(11)-H(11A)	109.5
N(4)-C(11)-H(11B)	109.5
H(11A)-C(11)-H(11B)	109.5
N(4)-C(11)-H(11C)	109.5
H(11A)-C(11)-H(11C)	109.5
H(11B)-C(11)-H(11C)	109.5
C(13)-C(12)-N(4)	110.8(5)
C(13)-C(12)-H(12A)	109.5
N(4)-C(12)-H(12A)	109.5
C(13)-C(12)-H(12B)	109.5
N(4)-C(12)-H(12B)	109.5
H(12A)-C(12)-H(12B)	108.1
C(12)-C(13)-N(1)	110.9(5)
C(12)-C(13)-H(13A)	109.5
N(1)-C(13)-H(13A)	109.5
C(12)-C(13)-H(13B)	109.5
N(1)-C(13)-H(13B)	109.5
H(13A)-C(13)-H(13B)	108.0
N(1)-C(14)-H(14A)	109.5
N(1)-C(14)-H(14B)	109.5
H(14A)-C(14)-H(14B)	109.5
N(1)-C(14)-H(14C)	109.5
H(14A)-C(14)-H(14C)	109.5
H(14B)-C(14)-H(14C)	109.5
C(13A)-N(1A)-C(14A)	110.3(11)
C(13A)-N(1A)-C(1A)	111.7(10)
C(14A)-N(1A)-C(1A)	108.5(11)
C(13A)-N(1A)-Co(1)	106.9(7)

C(14A)-N(1A)-Co(1)	106.7(8)
C(1A)-N(1A)-Co(1)	112.7(8)
C(3A)-N(2A)-C(4A)	108.8(7)
C(3A)-N(2A)-C(5A)	109.0(7)
C(4A)-N(2A)-C(5A)	105.0(8)
C(3A)-N(2A)-Co(1)	111.9(7)
C(4A)-N(2A)-Co(1)	116.2(5)
C(5A)-N(2A)-Co(1)	105.6(5)
C(6A)-N(3A)-C(8A)	107.4(7)
C(6A)-N(3A)-C(7A)	109.1(8)
C(8A)-N(3A)-C(7A)	110.2(7)
C(6A)-N(3A)-Co(1)	106.2(5)
C(8A)-N(3A)-Co(1)	114.9(6)
C(7A)-N(3A)-Co(1)	108.8(5)
C(11A)-N(4A)-C(12A)	109.3(9)
C(11A)-N(4A)-C(10A)	109.5(11)
C(12A)-N(4A)-C(10A)	106.6(9)
C(11A)-N(4A)-Co(1)	120.1(6)
C(12A)-N(4A)-Co(1)	101.3(6)
C(10A)-N(4A)-Co(1)	109.1(11)
C(2A)-C(1A)-N(1A)	117.3(12)
C(2A)-C(1A)-H(1AA)	108.0
N(1A)-C(1A)-H(1AA)	108.0
C(2A)-C(1A)-H(1AC)	108.0
N(1A)-C(1A)-H(1AC)	108.0
H(1AA)-C(1A)-H(1AC)	107.2
C(3A)-C(2A)-C(1A)	115.9(12)
C(3A)-C(2A)-H(2AA)	108.3
C(1A)-C(2A)-H(2AA)	108.3
C(3A)-C(2A)-H(2AC)	108.3
C(1A)-C(2A)-H(2AC)	108.3
H(2AA)-C(2A)-H(2AC)	107.4
C(2A)-C(3A)-N(2A)	113.6(8)
C(2A)-C(3A)-H(3AA)	108.8
N(2A)-C(3A)-H(3AA)	108.8
C(2A)-C(3A)-H(3AC)	108.8
N(2A)-C(3A)-H(3AC)	108.8
H(3AA)-C(3A)-H(3AC)	107.7
N(2A)-C(4A)-H(4AA)	109.5
N(2A)-C(4A)-H(4AB)	109.5
H(4AA)-C(4A)-H(4AB)	109.5
N(2A)-C(4A)-H(4AC)	109.5
H(4AA)-C(4A)-H(4AC)	109.5
H(4AB)-C(4A)-H(4AC)	109.5
C(6A)-C(5A)-N(2A)	109.8(7)
C(6A)-C(5A)-H(5AA)	109.7
N(2A)-C(5A)-H(5AA)	109.7
C(6A)-C(5A)-H(5AC)	109.7
N(2A)-C(5A)-H(5AC)	109.7
H(5AA)-C(5A)-H(5AC)	108.2
N(3A)-C(6A)-C(5A)	111.1(6)
N(3A)-C(6A)-H(6AA)	109.4
C(5A)-C(6A)-H(6AA)	109.4
N(3A)-C(6A)-H(6AC)	109.4
C(5A)-C(6A)-H(6AC)	109.4
H(6AA)-C(6A)-H(6AC)	108.0
N(3A)-C(7A)-H(7AA)	109.5
N(3A)-C(7A)-H(7AB)	109.5
H(7AA)-C(7A)-H(7AB)	109.5
N(3A)-C(7A)-H(7AC)	109.5
H(7AA)-C(7A)-H(7AC)	109.5
H(7AB)-C(7A)-H(7AC)	109.5
N(3A)-C(8A)-C(9A)	115.2(10)
N(3A)-C(8A)-H(8AA)	108.5
C(9A)-C(8A)-H(8AA)	108.5
N(3A)-C(8A)-H(8AC)	108.5
C(9A)-C(8A)-H(8AC)	108.5
H(8AA)-C(8A)-H(8AC)	107.5
C(10A)-C(9A)-C(8A)	115.0(14)
C(10A)-C(9A)-H(9AA)	108.5
C(8A)-C(9A)-H(9AA)	108.5
C(10A)-C(9A)-H(9AC)	108.5
C(8A)-C(9A)-H(9AC)	108.5
H(9AA)-C(9A)-H(9AC)	107.5
N(4A)-C(10A)-C(9A)	114.8(14)
N(4A)-C(10A)-H(10C)	108.6
C(9A)-C(10A)-H(10C)	108.6
N(4A)-C(10A)-H(10D)	108.6
C(9A)-C(10A)-H(10D)	108.6
H(10C)-C(10A)-H(10D)	107.5
N(4A)-C(11A)-H(11D)	109.5
N(4A)-C(11A)-H(11E)	109.5
H(11D)-C(11A)-H(11E)	109.5
N(4A)-C(11A)-H(11F)	109.5
H(11D)-C(11A)-H(11F)	109.5
H(11E)-C(11A)-H(11F)	109.5
C(13A)-C(12A)-N(4A)	111.8(7)
C(13A)-C(12A)-H(12C)	109.2
N(4A)-C(12A)-H(12C)	109.2
C(13A)-C(12A)-H(12D)	109.2
N(4A)-C(12A)-H(12D)	109.2
H(12C)-C(12A)-H(12D)	107.9
N(1A)-C(13A)-C(12A)	111.8(8)
N(1A)-C(13A)-H(13C)	109.3
C(12A)-C(13A)-H(13C)	109.3
N(1A)-C(13A)-H(13D)	109.3
C(12A)-C(13A)-H(13D)	109.3
H(13C)-C(13A)-H(13D)	107.9
N(1A)-C(14A)-H(14D)	109.5
N(1A)-C(14A)-H(14E)	109.5
H(14D)-C(14A)-H(14E)	109.5
N(1A)-C(14A)-H(14F)	109.5
H(14D)-C(14A)-H(14F)	109.5
H(14E)-C(14A)-H(14F)	109.5
O(2)-S(1)-O(3)	118.2(4)
O(2)-S(1)-O(1)	113.1(4)
O(3)-S(1)-O(1)	112.8(5)
O(2)-S(1)-C(15)	105.0(3)
O(3)-S(1)-C(15)	105.1(4)
O(1)-S(1)-C(15)	100.2(5)
S(1)-O(1)-Co(1)	145.1(7)
F(2)-C(15)-F(3)	113.1(10)
F(2)-C(15)-F(1)	107.8(7)
F(3)-C(15)-F(1)	105.6(8)
F(2)-C(15)-S(1)	111.8(6)
F(3)-C(15)-S(1)	110.6(6)
F(1)-C(15)-S(1)	107.6(7)
O(3A)-S(1A)-O(2A)	115.6(7)
O(3A)-S(1A)-O(1A)	117.7(10)
O(2A)-S(1A)-O(1A)	111.2(7)
O(3A)-S(1A)-C(15A)	104.9(7)
O(2A)-S(1A)-C(15A)	103.6(7)
O(1A)-S(1A)-C(15A)	101.5(9)
S(1A)-O(1A)-Co(1)	149.1(11)
F(2A)-C(15A)-F(3A)	107.3(13)
F(2A)-C(15A)-F(1A)	109.7(12)
F(3A)-C(15A)-F(1A)	108.0(11)
F(2A)-C(15A)-S(1A)	111.7(9)
F(3A)-C(15A)-S(1A)	112.0(10)
F(1A)-C(15A)-S(1A)	108.0(10)
O(31A)-Al(21)-O(21A)	115.0(15)
O(31A)#1-Al(21)-O(21A)	105.2(16)
O(21)#1-Al(21)-O(21)	112.0(9)
O(21)#1-Al(21)-O(31)	109.3(6)
O(21)-Al(21)-O(31)	104.7(5)
O(21)#1-Al(21)-O(31)#1	104.7(5)
O(21)-Al(21)-O(31)#1	109.3(6)
O(31)-Al(21)-O(31)#1	117.1(8)
C(21)-O(21)-Al(21)	148.0(9)
O(21)-C(21)-C(24)	109.0(6)
O(21)-C(21)-C(22)	110.1(6)
C(24)-C(21)-C(22)	110.0(4)
O(21)-C(21)-C(23)	112.2(6)
C(24)-C(21)-C(23)	107.2(4)
C(22)-C(21)-C(23)	108.2(4)
F(21)-C(22)-F(22)	108.8(6)
F(21)-C(22)-F(23)	107.7(6)
F(22)-C(22)-F(23)	106.7(6)
F(21)-C(22)-C(21)	111.4(5)
F(22)-C(22)-C(21)	112.7(6)
F(23)-C(22)-C(21)	109.3(5)
F(25)-C(23)-F(24)	108.0(5)
F(25)-C(23)-F(26)	107.9(5)
F(24)-C(23)-F(26)	107.8(6)
F(25)-C(23)-C(21)	111.3(4)
F(24)-C(23)-C(21)	111.7(5)
F(26)-C(23)-C(21)	109.8(4)
F(27)-C(24)-F(28)	108.1(4)
F(27)-C(24)-F(29)	108.6(6)
F(28)-C(24)-F(29)	106.5(6)
F(27)-C(24)-C(21)	111.3(4)
F(28)-C(24)-C(21)	109.3(4)
F(29)-C(24)-C(21)	112.8(6)
C(21A)-O(21A)-Al(21)	151(3)
O(21A)-C(21A)-C(24A)	108.5(19)
O(21A)-C(21A)-C(22A)	108.8(19)
C(24A)-C(21A)-C(22A)	110.3(11)
O(21A)-C(21A)-C(23A)	109.1(19)
C(24A)-C(21A)-C(23A)	109.9(11)
C(22A)-C(21A)-C(23A)	110.2(11)
F(21A)-C(22A)-F(22A)	109.8(17)
F(21A)-C(22A)-F(23A)	107.9(14)
F(22A)-C(22A)-F(23A)	110.2(16)
F(21A)-C(22A)-C(21A)	110.4(12)
F(22A)-C(22A)-C(21A)	109.3(15)
F(23A)-C(22A)-C(21A)	109.3(12)
F(25A)-C(23A)-F(26A)	106.9(13)
F(25A)-C(23A)-F(24A)	104.9(13)
F(26A)-C(23A)-F(24A)	106.5(12)
F(25A)-C(23A)-C(21A)	110.9(12)
F(26A)-C(23A)-C(21A)	109.5(11)
F(24A)-C(23A)-C(21A)	117.5(12)
F(27A)-C(24A)-F(29A)	106.9(17)
F(27A)-C(24A)-F(28A)	110.4(14)
F(29A)-C(24A)-F(28A)	107.0(18)
F(27A)-C(24A)-C(21A)	112.0(11)
F(29A)-C(24A)-C(21A)	112.3(18)
F(28A)-C(24A)-C(21A)	108.1(11)
C(31)-O(31)-Al(21)	148.4(7)
O(31)-C(31)-C(34)	109.5(8)

O(31)-C(31)-C(32)	112.6(6)
C(34)-C(31)-C(32)	110.7(6)
O(31)-C(31)-C(33)	108.3(7)
C(34)-C(31)-C(33)	109.2(6)
C(32)-C(31)-C(33)	106.4(6)
F(33)-C(32)-F(31)	106.0(7)
F(33)-C(32)-F(32)	110.3(7)
F(31)-C(32)-F(32)	113.1(9)
F(33)-C(32)-C(31)	114.3(6)
F(31)-C(32)-C(31)	108.3(7)
F(32)-C(32)-C(31)	104.9(7)
F(36)-C(33)-F(34)	110.3(8)
F(36)-C(33)-F(35)	106.7(6)
F(34)-C(33)-F(35)	110.7(8)
F(36)-C(33)-C(31)	110.3(6)
F(34)-C(33)-C(31)	110.0(7)
F(35)-C(33)-C(31)	108.8(6)
F(38)-C(34)-F(39)	117.7(8)
F(38)-C(34)-F(37)	105.9(11)
F(39)-C(34)-F(37)	107.1(10)
F(38)-C(34)-C(31)	109.0(6)
F(39)-C(34)-C(31)	107.2(6)
F(37)-C(34)-C(31)	109.9(11)
C(31A)-O(31A)-Al(21)	157.8(14)
O(31A)-C(31A)-C(34A)	108.7(11)
O(31A)-C(31A)-C(33A)	109.3(11)
C(34A)-C(31A)-C(33A)	105.6(9)
O(31A)-C(31A)-C(32A)	107.5(13)
C(34A)-C(31A)-C(32A)	108.2(8)
C(33A)-C(31A)-C(32A)	117.4(9)
F(31A)-C(32A)-F(33A)	110.1(9)
F(31A)-C(32A)-F(32A)	104.2(8)
F(33A)-C(32A)-F(32A)	112.9(12)
F(31A)-C(32A)-C(31A)	112.7(10)
F(33A)-C(32A)-C(31A)	110.3(10)
F(32A)-C(32A)-C(31A)	106.5(8)
F(35A)-C(33A)-F(34A)	105.5(15)
F(35A)-C(33A)-F(36A)	111.2(10)
F(34A)-C(33A)-F(36A)	106.7(17)
F(35A)-C(33A)-C(31A)	110.7(10)
F(34A)-C(33A)-C(31A)	111.7(17)
F(36A)-C(33A)-C(31A)	110.9(9)
F(37A)-C(34A)-F(39A)	107.2(7)
F(37A)-C(34A)-F(38A)	104.7(10)
F(39A)-C(34A)-F(38A)	96.1(9)
F(37A)-C(34A)-C(31A)	116.6(8)
F(39A)-C(34A)-C(31A)	118.5(8)
F(38A)-C(34A)-C(31A)	111.1(8)
O(41A)-Al(41)-O(51A)	83.5(12)
O(41A)#2-Al(41)-O(51A)	123.1(9)
O(41A)-Al(41)-O(51A)#2	123.1(9)
O(41A)#2-Al(41)-O(51A)#2	83.5(12)
O(51A)-Al(41)-O(51A)#2	101.3(10)
O(41)-Al(41)-O(41)#2	111.82(17)
O(41)-Al(41)-O(51)#2	105.2(4)
O(41)#2-Al(41)-O(51)#2	103.8(4)
O(41)-Al(41)-O(51)	103.8(4)
O(41)#2-Al(41)-O(51)	105.1(4)
O(51)#2-Al(41)-O(51)	127.0(8)
C(41)-O(41)-Al(41)	148.3(2)
O(41)-C(41)-C(42)	110.3(3)
O(41)-C(41)-C(44)	107.1(3)
C(42)-C(41)-C(44)	110.6(3)
O(41)-C(41)-C(43)	111.8(3)
C(42)-C(41)-C(43)	108.2(3)
C(44)-C(41)-C(43)	108.8(3)
F(41)-C(42)-F(43)	107.8(4)
F(41)-C(42)-F(42)	107.1(3)
F(43)-C(42)-F(42)	107.2(4)
F(41)-C(42)-C(41)	110.4(3)
F(43)-C(42)-C(41)	113.3(3)
F(42)-C(42)-C(41)	110.7(3)
F(44)-C(43)-F(45)	107.5(4)
F(44)-C(43)-F(46)	107.4(4)
F(45)-C(43)-F(46)	107.2(4)
F(44)-C(43)-C(41)	111.2(3)
F(45)-C(43)-C(41)	110.3(3)
F(46)-C(43)-C(41)	113.0(4)
F(48)-C(44)-F(49)	107.4(3)
F(48)-C(44)-F(47)	107.5(3)
F(49)-C(44)-F(47)	107.2(3)
F(48)-C(44)-C(41)	110.8(3)
F(49)-C(44)-C(41)	111.2(3)
F(47)-C(44)-C(41)	112.5(3)
C(41A)-O(41A)-Al(41)	154(2)
O(41A)-C(41A)-C(44A)	108.5(16)
O(41A)-C(41A)-C(42A)	111.4(16)
C(44A)-C(41A)-C(42A)	109.4(14)
O(41A)-C(41A)-C(43A)	108.5(15)
C(44A)-C(41A)-C(43A)	109.5(14)
C(42A)-C(41A)-C(43A)	109.5(14)
F(41A)-C(42A)-F(43A)	107(2)
F(41A)-C(42A)-F(42A)	108(2)
F(43A)-C(42A)-F(42A)	109(2)
F(41A)-C(42A)-C(41A)	111.4(19)
F(43A)-C(42A)-C(41A)	111(2)
F(42A)-C(42A)-C(41A)	110.4(17)
F(45A)-C(43A)-F(46A)	108(2)
F(45A)-C(43A)-F(44A)	106.4(19)
F(46A)-C(43A)-F(44A)	110(2)
F(45A)-C(43A)-C(41A)	111.6(16)
F(46A)-C(43A)-C(41A)	112(2)
F(44A)-C(43A)-C(41A)	108.9(16)
F(47A)-C(44A)-F(48A)	105.8(17)
F(47A)-C(44A)-F(49A)	109.5(18)
F(48A)-C(44A)-F(49A)	106.1(19)
F(47A)-C(44A)-C(41A)	113.0(16)
F(48A)-C(44A)-C(41A)	110.8(16)
F(49A)-C(44A)-C(41A)	111.2(17)
C(51)-O(51)-Al(41)	150.6(8)
O(51)-C(51)-C(54)	111.8(8)
O(51)-C(51)-C(52)	107.0(8)
C(54)-C(51)-C(52)	111.2(8)
O(51)-C(51)-C(53)	108.2(8)
C(54)-C(51)-C(53)	109.0(7)
C(52)-C(51)-C(53)	109.5(7)
F(52)-C(52)-F(51)	110.4(9)
F(52)-C(52)-F(53)	103.6(9)
F(51)-C(52)-F(53)	106.3(9)
F(52)-C(52)-C(51)	113.3(8)
F(51)-C(52)-C(51)	111.7(8)
F(53)-C(52)-C(51)	111.0(7)
F(55)-C(53)-F(54)	106.4(7)
F(55)-C(53)-F(56)	108.0(7)
F(54)-C(53)-F(56)	107.3(7)
F(55)-C(53)-C(51)	112.6(7)
F(54)-C(53)-C(51)	111.7(6)
F(56)-C(53)-C(51)	110.5(7)
F(59)-C(54)-F(58)	109.9(10)
F(59)-C(54)-F(57)	107.1(9)
F(58)-C(54)-F(57)	104.7(8)
F(59)-C(54)-C(51)	110.8(9)
F(58)-C(54)-C(51)	114.0(8)
F(57)-C(54)-C(51)	110.0(10)
C(51A)-O(51A)-Al(41)	149.8(10)
O(51A)-C(51A)-C(54A)	114.2(8)
O(51A)-C(51A)-C(52A)	108.2(9)
C(54A)-C(51A)-C(52A)	104.8(8)
O(51A)-C(51A)-C(53A)	107.4(8)
C(54A)-C(51A)-C(53A)	111.5(8)
C(52A)-C(51A)-C(53A)	110.6(8)
F(52A)-C(52A)-F(51A)	106.0(10)
F(52A)-C(52A)-F(53A)	113.3(12)
F(51A)-C(52A)-F(53A)	106.4(9)
F(52A)-C(52A)-C(51A)	109.1(9)
F(51A)-C(52A)-C(51A)	112.2(9)
F(53A)-C(52A)-C(51A)	109.9(8)
F(55A)-C(53A)-F(56A)	107.1(9)
F(55A)-C(53A)-F(54A)	108.7(8)
F(56A)-C(53A)-F(54A)	109.7(9)
F(55A)-C(53A)-C(51A)	109.1(8)
F(56A)-C(53A)-C(51A)	109.3(8)
F(54A)-C(53A)-C(51A)	112.8(8)
F(57A)-C(54A)-F(58A)	111.2(9)
F(57A)-C(54A)-F(59A)	102.2(8)
F(58A)-C(54A)-F(59A)	107.9(10)
F(57A)-C(54A)-C(51A)	113.9(10)
F(58A)-C(54A)-C(51A)	112.3(8)
F(59A)-C(54A)-C(51A)	108.6(8)

Symmetry transformations used to generate equivalent atoms:
#1 -x+1,y,-z+1/2 #2 -x+1,y,-z+3/2

Table S88. Anisotropic displacement parameters ($Å^2$x 10^3) for **[(TMC)Co^{II}(OTf)][Al{OC(CF_3)$_3$}$_4$]**.
The anisotropic displacement factor exponent takes the form: -2p^2[h^2 a*^{2}U^{11} + ... + 2 h k a* b* U^{12}]

	U^{11}	U^{22}	U^{33}	U^{23}	U^{13}	U^{12}
Co(1)	25(1)	20(1)	20(1)	-2(1)	16(1)	1(1)
N(1)	32(2)	38(4)	20(2)	0(3)	17(2)	4(3)
N(2)	44(3)	25(3)	48(3)	-5(2)	37(3)	0(2)
N(3)	31(3)	18(3)	37(3)	0(2)	18(3)	3(2)
N(4)	30(3)	35(2)	24(3)	-7(2)	19(2)	-8(2)
C(1)	60(5)	41(5)	21(3)	3(3)	24(4)	14(4)
C(2)	81(5)	29(4)	30(4)	5(3)	38(4)	-3(3)
C(3)	62(4)	46(4)	49(4)	-4(3)	46(3)	-7(3)
C(4)	43(3)	34(4)	62(4)	-2(3)	38(3)	-1(3)
C(5)	38(3)	33(3)	66(4)	2(3)	41(3)	7(3)
C(6)	26(3)	34(3)	57(4)	-1(3)	20(3)	4(2)
C(7)	52(4)	43(4)	31(4)	4(3)	17(3)	15(3)
C(8)	43(4)	22(3)	38(4)	-2(3)	23(3)	3(3)
C(9)	68(4)	22(3)	36(5)	-11(3)	32(4)	-16(3)
C(10)	44(3)	37(4)	44(4)	-17(3)	33(3)	-20(3)
C(11)	48(4)	43(3)	43(4)	-12(3)	38(3)	-14(3)
C(12)	20(2)	46(3)	30(3)	-2(3)	16(2)	-3(2)
C(13)	28(3)	52(4)	31(3)	3(3)	16(2)	10(2)
C(14)	48(4)	35(4)	33(4)	1(3)	26(4)	17(3)
N(1A)	45(4)	51(6)	21(3)	-12(4)	9(3)	18(4)
N(2A)	60(4)	21(4)	49(4)	-3(3)	48(3)	-1(3)
N(3A)	32(3)	12(3)	30(4)	-7(3)	13(3)	5(2)
N(4A)	42(4)	68(4)	34(4)	-32(3)	30(4)	-29(3)
C(1A)	69(6)	46(6)	18(4)	5(4)	18(4)	20(5)
C(2A)	91(6)	47(6)	29(5)	-5(4)	42(5)	1(5)
C(3A)	80(5)	39(4)	47(4)	-2(3)	53(4)	-5(4)
C(4A)	82(6)	28(4)	67(6)	-9(4)	64(5)	-14(4)
C(5A)	41(4)	44(4)	64(4)	-9(3)	41(3)	-1(3)
C(6A)	38(4)	30(4)	56(5)	0(4)	30(4)	12(3)
C(7A)	51(5)	28(4)	25(4)	-2(3)	9(4)	16(4)
C(8A)	54(5)	10(3)	27(4)	-7(3)	17(4)	-6(3)
C(9A)	77(5)	34(5)	31(6)	-9(4)	35(4)	-24(4)
C(10A)	61(5)	53(5)	40(5)	-24(4)	35(4)	-29(4)
C(11A)	89(8)	114(9)	56(5)	-45(6)	64(6)	-67(7)
C(12A)	33(3)	89(5)	55(4)	-44(4)	28(3)	-16(3)
C(13A)	32(4)	67(6)	41(4)	-24(4)	10(3)	14(4)
C(14A)	66(8)	53(7)	24(4)	-14(6)	0(5)	39(6)
S(1)	59(1)	42(1)	37(1)	-23(1)	37(1)	-22(1)
F(1)	71(3)	91(5)	98(4)	-78(4)	57(3)	-36(3)
F(2)	58(2)	100(3)	112(3)	-53(3)	49(2)	-3(2)
F(3)	115(5)	41(2)	117(4)	-20(3)	85(4)	-14(3)
O(1)	49(4)	36(3)	35(2)	-17(2)	31(2)	-13(3)
O(2)	47(2)	52(2)	44(2)	-20(2)	20(2)	-6(2)
O(3)	130(4)	91(4)	81(3)	-46(3)	89(3)	-60(3)
C(15)	50(3)	56(4)	77(4)	-42(3)	46(3)	-19(3)
S(1A)	53(2)	39(2)	36(2)	-18(1)	36(2)	-16(2)
F(1A)	143(9)	47(5)	111(7)	-40(5)	108(7)	-35(6)
F(2A)	48(4)	52(4)	66(5)	-10(3)	40(4)	-18(3)
F(3A)	58(2)	100(3)	112(3)	-53(3)	49(2)	-3(2)
O(1A)	39(5)	36(4)	29(4)	-15(3)	24(3)	-12(4)
O(2A)	130(4)	91(4)	81(3)	-46(3)	89(3)	-60(3)
O(3A)	88(7)	54(5)	20(4)	-11(3)	22(4)	-15(5)
C(15A)	43(5)	45(7)	81(6)	-32(5)	52(4)	-20(4)
Al(21)	26(1)	20(1)	20(1)	0	18(1)	0
F(21)	98(3)	73(2)	49(2)	1(2)	57(2)	5(2)
F(22)	57(3)	70(3)	26(2)	5(2)	12(2)	-12(2)
F(23)	89(3)	46(2)	42(2)	-11(1)	34(2)	-26(2)
F(24)	59(3)	88(5)	57(3)	33(3)	22(3)	32(3)
F(25)	34(1)	83(2)	59(2)	19(2)	25(1)	-5(1)
F(26)	48(2)	78(3)	42(2)	4(2)	29(2)	19(2)
F(27)	73(2)	37(2)	50(2)	-3(1)	31(2)	-16(1)
F(28)	42(2)	80(3)	61(2)	26(2)	34(2)	-4(2)
F(29)	56(3)	54(3)	45(3)	26(2)	27(2)	1(2)
O(21)	40(2)	34(1)	28(1)	8(1)	23(1)	3(1)
C(21)	34(2)	30(2)	20(2)	4(2)	16(2)	-7(2)
C(22)	69(3)	48(3)	29(2)	2(2)	27(2)	-9(2)
C(23)	38(3)	52(3)	34(2)	13(2)	18(2)	4(2)
C(24)	39(2)	42(3)	33(2)	11(2)	20(2)	-5(2)
F(21A)	101(9)	35(5)	83(8)	-28(5)	59(7)	-16(5)
F(22A)	96(14)	79(11)	24(6)	0(6)	23(7)	20(10)
F(23A)	36(5)	55(7)	74(7)	12(6)	14(5)	-16(4)
F(24A)	23(5)	33(7)	47(7)	3(5)	29(5)	3(5)
F(25A)	72(9)	76(9)	77(9)	19(7)	65(8)	22(6)
F(26A)	62(6)	54(6)	60(6)	-9(5)	33(5)	12(5)
F(27A)	44(5)	56(7)	43(5)	7(4)	19(5)	-17(5)
F(28A)	87(8)	116(9)	76(7)	19(6)	72(7)	25(7)
F(29A)	57(9)	46(8)	48(8)	12(6)	35(7)	-6(6)
O(21A)	40(2)	34(1)	28(1)	8(1)	23(1)	3(1)
C(21A)	32(5)	39(5)	26(4)	4(4)	21(4)	7(4)
C(22A)	55(6)	51(7)	37(6)	-1(5)	23(5)	-4(5)
C(23A)	42(6)	52(7)	40(6)	9(4)	28(5)	14(5)
C(24A)	44(6)	52(7)	37(6)	7(4)	27(5)	-1(5)
F(31)	81(5)	112(6)	81(4)	64(4)	59(4)	43(4)
F(32)	198(9)	110(6)	100(6)	-37(5)	123(6)	-37(6)
F(33)	96(4)	58(3)	70(3)	22(3)	62(3)	-3(3)
F(34)	106(6)	43(3)	157(7)	6(4)	107(5)	4(3)
F(35)	94(4)	92(4)	151(6)	14(4)	106(4)	-10(3)
F(36)	44(2)	88(3)	105(3)	30(3)	42(2)	9(2)
F(37)	81(4)	96(3)	100(3)	7(2)	47(3)	-53(2)
F(38)	78(3)	60(3)	44(2)	-2(2)	23(2)	-30(2)

F(39)	112(4)	31(2)	104(4)	-8(2)	89(3)	-4(2)
O(31)	27(3)	27(2)	21(3)	1(2)	16(2)	-5(2)
C(31)	45(3)	31(3)	45(3)	4(2)	32(3)	-9(2)
C(32)	72(4)	60(4)	59(3)	6(3)	49(3)	2(3)
C(33)	61(4)	73(4)	98(4)	-5(4)	62(3)	-13(3)
C(34)	78(3)	60(3)	44(2)	-2(2)	23(2)	-30(2)
F(31A)	74(5)	110(6)	117(7)	58(6)	82(5)	34(5)
F(32A)	47(3)	67(5)	38(3)	12(3)	35(3)	-10(3)
F(33A)	100(7)	65(6)	144(8)	44(5)	115(7)	40(5)
F(34A)	81(4)	96(3)	100(3)	7(2)	47(3)	-53(2)
F(35A)	44(2)	88(3)	105(3)	30(3)	42(2)	9(2)
F(36A)	78(3)	60(3)	44(2)	-2(2)	23(2)	-30(2)
F(37A)	101(6)	42(4)	102(6)	24(4)	90(5)	15(4)
F(38A)	68(5)	36(4)	72(6)	24(4)	57(4)	22(4)
F(39A)	95(5)	34(3)	85(5)	12(3)	71(5)	12(3)
O(31A)	34(5)	43(5)	45(7)	-9(5)	29(5)	-16(4)
C(31A)	42(4)	40(5)	53(4)	4(4)	37(3)	-8(3)
C(32A)	44(4)	51(4)	69(4)	25(4)	47(4)	16(3)
C(33A)	52(5)	80(6)	67(5)	15(4)	28(4)	-36(4)
C(34A)	62(4)	33(4)	60(4)	5(3)	52(4)	-3(4)
Al(41)	43(1)	18(1)	30(1)	0	31(1)	0
F(41)	54(2)	38(2)	41(1)	9(1)	33(2)	-2(1)
F(42)	48(1)	20(1)	34(1)	4(1)	17(1)	6(1)
F(43)	45(1)	39(1)	47(1)	4(1)	21(1)	-14(1)
F(44)	44(2)	60(2)	63(2)	22(2)	43(2)	17(2)
F(45)	55(2)	34(1)	64(2)	25(1)	46(2)	23(1)
F(46)	31(2)	66(3)	38(2)	19(2)	14(1)	10(2)
F(47)	53(2)	47(1)	27(1)	-13(1)	16(1)	-7(1)
F(48)	41(1)	42(1)	30(1)	-1(1)	21(1)	17(1)
F(49)	45(2)	43(2)	33(2)	12(1)	28(2)	14(1)
O(41)	32(1)	24(1)	31(1)	-5(1)	22(1)	1(1)
C(41)	28(2)	22(2)	25(1)	3(1)	15(1)	3(1)
C(42)	36(2)	26(2)	31(2)	3(1)	16(2)	-2(1)
C(43)	33(2)	41(2)	39(2)	16(2)	24(2)	10(2)
C(44)	34(2)	31(2)	23(2)	0(1)	14(2)	6(2)
F(41A)	53(16)	51(14)	40(9)	21(8)	27(11)	5(12)
F(42A)	46(8)	18(12)	52(13)	14(10)	22(8)	-1(8)
F(43A)	45(1)	39(1)	47(1)	4(1)	21(1)	-14(1)
F(44A)	40(12)	39(12)	36(11)	10(9)	23(11)	20(12)
F(45A)	45(13)	19(7)	39(11)	5(7)	23(10)	12(9)
F(46A)	26(11)	24(10)	31(9)	-8(8)	16(7)	-8(8)
F(47A)	32(10)	38(9)	35(11)	-10(8)	18(8)	-14(8)
F(48A)	26(6)	36(10)	34(9)	-2(8)	12(6)	4(6)
F(49A)	38(10)	28(9)	17(10)	-2(8)	15(9)	6(8)
O(41A)	39(7)	26(7)	27(6)	3(5)	19(5)	-9(6)
C(41A)	32(5)	23(6)	30(5)	4(4)	18(4)	1(5)
C(42A)	38(7)	27(8)	34(7)	9(6)	15(6)	-2(6)
C(43A)	34(7)	19(7)	34(7)	2(6)	19(5)	9(7)
C(44A)	29(6)	25(7)	27(7)	0(5)	13(5)	0(6)
F(51)	99(6)	57(4)	79(4)	11(3)	83(5)	17(3)
F(52)	118(6)	40(4)	94(5)	11(4)	89(5)	12(4)
F(53)	73(4)	82(5)	79(3)	-9(3)	65(3)	-1(3)
F(54)	82(4)	48(3)	59(3)	18(2)	53(4)	37(3)
F(55)	87(5)	71(4)	44(3)	24(3)	48(3)	43(3)
F(56)	115(6)	57(4)	99(6)	15(4)	89(5)	19(4)
F(57)	96(5)	118(3)	48(2)	26(2)	49(3)	73(3)
F(58)	87(5)	101(5)	52(4)	-3(4)	52(4)	30(5)
F(59)	83(5)	134(7)	74(6)	-56(5)	51(5)	-5(5)
O(51)	55(4)	47(4)	51(3)	-6(3)	44(3)	11(3)
C(51)	72(4)	49(4)	49(3)	17(3)	54(3)	33(3)
C(52)	90(5)	55(5)	64(4)	18(4)	68(4)	26(4)
C(53)	82(5)	44(4)	52(4)	18(3)	53(4)	26(4)
C(54)	96(5)	118(3)	48(2)	26(2)	49(3)	73(3)
F(51A)	87(6)	81(6)	78(4)	18(4)	73(5)	32(4)
F(52A)	159(9)	55(6)	134(7)	24(6)	131(7)	27(6)
F(53A)	76(5)	113(7)	80(4)	-5(5)	52(4)	8(5)
F(54A)	112(6)	72(5)	82(5)	30(4)	77(5)	63(5)
F(55A)	87(5)	84(5)	31(3)	28(3)	37(3)	42(4)
F(56A)	120(6)	67(4)	111(6)	-17(4)	91(6)	4(4)
F(57A)	96(5)	118(3)	48(2)	26(2)	49(3)	73(3)
F(58A)	102(7)	94(5)	53(5)	-5(4)	45(5)	51(6)
F(59A)	78(5)	115(6)	56(5)	-23(4)	34(4)	25(5)
O(51A)	85(6)	52(5)	54(4)	32(3)	61(5)	42(5)
C(51A)	68(4)	68(5)	40(3)	28(3)	45(3)	47(4)
C(52A)	87(5)	70(6)	60(4)	21(4)	61(4)	39(5)
C(53A)	89(6)	63(6)	47(4)	13(4)	53(4)	40(5)
C(54A)	90(6)	89(5)	42(4)	8(4)	46(4)	51(5)

Table S89. Hydrogen coordinates (x 10^4) and isotropic displacement parameters ($Å^2$x 10^3) for **[(TMC)Co^{II}(OTf)][Al{OC(CF$_3$)$_3$}$_4$]**.

	x	y	z	U(eq)
H(1A)	6979	4435	5531	52
H(1AB)	6728	5032	5245	52
H(2A)	7507	4952	5140	55
H(2AB)	7879	5276	6070	55
H(3A)	8036	4173	6033	54
H(3AB)	8548	4595	6203	54
H(4A)	9293	5067	7594	67
H(4B)	9334	4960	8429	67
H(4C)	8716	5287	7550	67
H(5A)	8905	3740	7306	50
H(5AB)	9509	4150	7758	50
H(6A)	9719	3610	8900	55
H(6AB)	9663	4215	9073	55
H(7A)	8791	3697	9571	77
H(7B)	9389	4077	9885	77
H(7C)	9464	3452	9880	77
H(8A)	8960	2984	8600	46
H(8AB)	8473	3237	7593	46
H(9A)	8074	3140	8601	53
H(9AB)	7927	2685	7925	53
H(10A)	7286	3318	6681	47
H(10B)	6953	3160	7080	47
H(11A)	7274	4355	8230	58
H(11B)	7697	3822	8693	58
H(11C)	6892	3802	7942	58
H(12A)	6584	3989	5969	39
H(12B)	6273	4044	6426	39
H(13A)	6725	4883	6959	48
H(13B)	6282	4861	5871	48
H(14A)	6982	5655	6300	60
H(14B)	7780	5593	6966	60
H(14C)	7431	5524	7368	60
H(1AA)	7219	4368	5590	65
H(1AC)	6852	4926	5273	65
H(2AA)	7753	5028	5427	66
H(2AC)	7922	5340	6264	66
H(3AA)	8829	4847	6648	56
H(3AC)	8425	4322	6462	56
H(4AA)	9358	5358	7936	71
H(4AB)	9272	5256	8652	71
H(4AC)	8661	5495	7657	71
H(5AA)	9683	4429	8051	54
H(5AC)	9689	4455	8869	54
H(6AA)	8960	3702	7409	51
H(6AC)	9621	3574	8456	51
H(7AA)	8946	3629	9427	73
H(7AB)	9505	3977	9570	73
H(7AC)	9521	3349	9520	73
H(8AA)	8793	2898	8138	47
H(8AC)	8228	3189	7178	47
H(9AA)	7763	2668	7630	59
H(9AC)	8139	3027	8524	59
H(10C)	7144	3446	6716	61
H(10D)	6956	3216	7269	61
H(11D)	7254	3659	8555	111
H(11E)	7658	4205	8920	111
H(11F)	8039	3665	9104	111
H(12C)	6423	4016	6908	72
H(12D)	6828	4547	7440	72
H(13C)	6610	4108	5928	71
H(13D)	6283	4642	5902	71
H(14D)	6857	5452	6260	110
H(14E)	7660	5448	7064	110
H(14F)	7209	5260	7272	110

[(TMC)Co^{II}(Br)]Br

The structure of **[(TMC)Co^{II}(Br)]Br** was solved in the orthorhombic space group *Pca*2$_1$. The asymmetric unit contains two molecules of **[(TMC)Co^{II}(Br)]Br** and an additional water molecule. One of the complex molecules was found to be disordered over two positions. The disorder was refined with the help of same distance restraints, similarity restraints on anisotropic displacement parameters and advanced rigid bond restraints. Some disordered atoms with very close positions were set to the same anisotropic displacement parameters. The disorder ratio was refined freely and converged to 0.562(6).

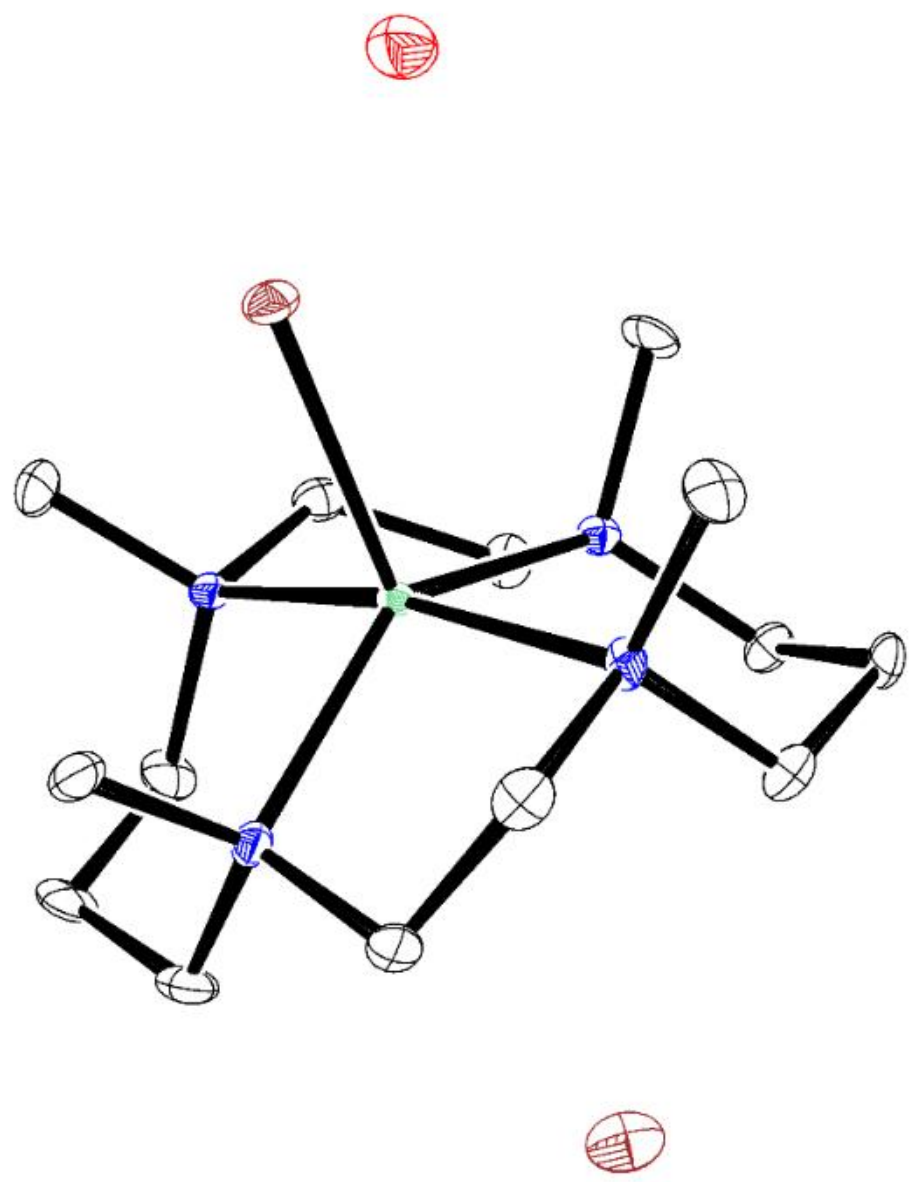

Figure S18. Molecular structure of **[(TMC)Co^{II}(Br)]Br**, (additional water molecule shown).

Table S90. Crystal data and structure refinement for **[(TMC)Co^{II}(Br)]Br**.

CCDC No	2062709	
Empirical formula	$C_{28}H_{66}Br_4Co_2N_8O$	
Formula weight	968.38	
Temperature	100(2) K	
Wavelength	0.71073 Å	
Crystal system	Orthorhombic	
Space group	*Pca*2$_1$	
Unit cell dimensions	a = 14.8850(9) Å	α = 90°
	b = 8.5718(6) Å	β = 90°
	c = 29.4703(19) Å	γ = 90°
Volume	3760.2(4) Å^3	
Z	4	
Density (calculated)	1.711 Mg/m^3	
Absorption coefficient	5.167 mm^{-1}	
F(000)	1968	
Crystal size	0.439 x 0.413 x 0.252 mm^3	
Theta range for data collection	2.376 to 25.072°.	
Index ranges	-17 ≤ h ≤ 17, -10 ≤ k ≤ 10, -35 ≤ l ≤ 34	
Reflections collected	54214	
Independent reflections	6638 [R(int) = 0.0607]	
Completeness to theta = 25.072°	99.9 %	
Absorption correction	Semi-empirical from equivalents	
Refinement method	Full-matrix least-squares on F^2	
Data / restraints / parameters	6638 / 946 / 485	
Goodness-of-fit on F^2	1.070	
Final R indices [I>2σ(I)]	R1 = 0.0233, wR2 = 0.0473	
R indices (all data)	R1 = 0.0251, wR2 = 0.0478	
Absolute structure parameter	0.053(4)	
Largest diff. peak and hole	0.475 and -0.500 eÅ^{-3}	

Table S91. Atomic coordinates (x 10^4) and equivalent isotropic displacement parameters ($Å^2$x 10^3) for **[(TMC)Co^{II}(Br)]Br**. U(eq) is defined as one third of the trace of the orthogonalized U^{ij} tensor.

	x	y	z	U(eq)
Br(1)	7055(1)	4778(1)	3909(1)	14(1)
Co(1)	6232(1)	2772(1)	3477(1)	7(1)
N(1)	4948(2)	3575(4)	3261(1)	9(1)
N(2)	5469(2)	1789(4)	4054(1)	10(1)
N(3)	7071(2)	761(5)	3431(1)	11(1)
N(4)	6836(2)	3378(5)	2812(1)	11(1)
C(1)	4275(3)	2774(6)	3551(2)	13(1)
C(2)	4594(3)	2622(6)	4032(2)	13(1)
C(3)	5834(3)	2055(6)	4514(2)	16(1)
C(4)	5296(3)	83(5)	3996(2)	16(1)
C(5)	6142(3)	-898(5)	3966(2)	18(1)
C(6)	6630(3)	-778(5)	3515(2)	16(1)
C(7)	7827(3)	953(6)	3758(2)	17(1)
C(8)	7441(3)	740(5)	2962(2)	14(1)
C(9)	7643(3)	2371(6)	2797(2)	15(1)
C(10)	7146(3)	5018(6)	2746(2)	17(1)
C(11)	6231(3)	2962(6)	2428(2)	14(1)
C(12)	5344(3)	3839(6)	2425(2)	15(1)
C(13)	4686(3)	3270(6)	2780(2)	12(1)
C(14)	4902(3)	5294(5)	3349(2)	15(1)
Br(21)	1512(1)	4904(1)	6496(1)	16(1)
Co(21)	2209(1)	2911(1)	6014(1)	8(1)
N(21)	2207(8)	4169(13)	5382(4)	15(1)
N(22)	1172(6)	1506(9)	5667(3)	16(1)
N(23)	2301(6)	1142(10)	6556(4)	14(2)
N(24)	3625(7)	3045(12)	6098(3)	13(2)
C(21)	1380(6)	3687(11)	5123(3)	15(1)
C(22)	1228(6)	1963(10)	5181(3)	15(2)
C(23)	301(7)	2037(13)	5857(4)	15(2)
C(24)	1244(8)	-224(11)	5694(4)	16(1)
C(25)	1334(9)	-891(18)	6168(4)	16(1)
C(26)	2235(8)	-500(13)	6385(4)	17(2)
C(27)	1658(7)	1304(12)	6942(3)	16(2)
C(28)	3222(6)	1369(11)	6747(3)	19(2)
C(29)	3892(7)	1688(14)	6375(4)	20(2)
C(30)	3828(7)	4492(16)	6347(4)	19(2)
C(31)	4179(9)	3008(12)	5674(4)	15(1)
C(32)	3890(10)	4200(30)	5317(7)	15(1)
C(33)	2999(10)	3770(30)	5092(7)	16(2)
C(34)	2165(12)	5903(15)	5416(6)	14(2)
N(21A)	2286(10)	3842(16)	5329(5)	15(1)
N(22A)	997(8)	1855(12)	5791(4)	16(1)
N(23A)	2619(8)	974(13)	6418(4)	16(3)
N(24A)	3629(9)	3701(15)	6144(4)	13(3)
C(21A)	1590(7)	2914(16)	5075(4)	15(1)
C(22A)	764(7)	2687(14)	5367(4)	20(3)
C(23A)	183(9)	2005(17)	6093(6)	20(3)
C(24A)	1094(11)	153(14)	5692(5)	16(1)
C(25A)	1409(11)	-800(20)	6087(5)	16(1)
C(26A)	2393(10)	-585(17)	6213(5)	17(2)
C(27A)	2195(11)	1085(15)	6869(5)	19(3)
C(28A)	3609(8)	1054(14)	6465(5)	18(3)
C(29A)	3918(8)	2721(14)	6538(4)	17(2)
C(30A)	3766(10)	5347(17)	6291(5)	17(3)
C(31A)	4236(11)	3393(16)	5749(4)	15(1)
C(32A)	3990(13)	4190(40)	5313(9)	15(1)
C(33A)	3151(12)	3570(40)	5085(10)	16(2)
C(34A)	2054(16)	5513(19)	5322(9)	14(2)
Br(11)	3470(1)	9348(1)	5047(1)	19(1)
Br(12)	25(1)	-1063(1)	7563(1)	23(1)
O(11)	4165(3)	6463(5)	4377(2)	29(1)

Table S92. Bond lengths [Å] and angles [°] for **[(TMC)Co^{II}(Br)]Br**.

Br(1)-Co(1)	2.4650(8)	C(27)-H(27B)	0.9800
Co(1)-N(1)	2.130(4)	C(27)-H(27C)	0.9800
Co(1)-N(3)	2.133(4)	C(28)-C(29)	1.509(12)
Co(1)-N(2)	2.212(4)	C(28)-H(28A)	0.9900
Co(1)-N(4)	2.217(4)	C(28)-H(28B)	0.9900
N(1)-C(1)	1.486(6)	C(29)-H(29A)	0.9900
N(1)-C(13)	1.492(6)	C(29)-H(29B)	0.9900
N(1)-C(14)	1.498(6)	C(30)-H(30A)	0.9800
N(2)-C(3)	1.477(6)	C(30)-H(30B)	0.9800
N(2)-C(2)	1.487(6)	C(30)-H(30C)	0.9800
N(2)-C(4)	1.494(6)	C(31)-C(32)	1.525(16)
N(3)-C(8)	1.490(6)	C(31)-H(31A)	0.9900
N(3)-C(7)	1.491(6)	C(31)-H(31B)	0.9900
N(3)-C(6)	1.494(6)	C(32)-C(33)	1.528(14)
N(4)-C(9)	1.479(6)	C(32)-H(32A)	0.9900
N(4)-C(11)	1.490(6)	C(32)-H(32B)	0.9900
N(4)-C(10)	1.492(6)	C(33)-H(33A)	0.9900
C(1)-C(2)	1.499(7)	C(33)-H(33B)	0.9900
C(1)-H(1A)	0.9900	C(34)-H(34A)	0.9800
C(1)-H(1AB)	0.9900	C(34)-H(34B)	0.9800
C(2)-H(2A)	0.9900	C(34)-H(34C)	0.9800
C(2)-H(2AB)	0.9900	N(21A)-C(34A)	1.474(15)
C(3)-H(3A)	0.9800	N(21A)-C(33A)	1.494(15)
C(3)-H(3B)	0.9800	N(21A)-C(21A)	1.506(14)
C(3)-H(3C)	0.9800	N(22A)-C(22A)	1.481(13)
C(4)-C(5)	1.517(7)	N(22A)-C(24A)	1.494(13)
C(4)-H(4A)	0.9900	N(22A)-C(23A)	1.508(14)
C(4)-H(4AB)	0.9900	N(23A)-C(27A)	1.476(15)
C(5)-C(6)	1.519(7)	N(23A)-C(28A)	1.482(15)
C(5)-H(5A)	0.9900	N(23A)-C(26A)	1.504(14)
C(5)-H(5AB)	0.9900	N(24A)-C(30A)	1.490(15)
C(6)-H(6A)	0.9900	N(24A)-C(29A)	1.495(14)
C(6)-H(6AB)	0.9900	N(24A)-C(31A)	1.498(15)
C(7)-H(7A)	0.9800	C(21A)-C(22A)	1.513(14)
C(7)-H(7B)	0.9800	C(21A)-H(21C)	0.9900
C(7)-H(7C)	0.9800	C(21A)-H(21D)	0.9900
C(8)-C(9)	1.511(6)	C(22A)-H(22C)	0.9900
C(8)-H(8A)	0.9900	C(22A)-H(22D)	0.9900
C(8)-H(8AB)	0.9900	C(23A)-H(23D)	0.9800
C(9)-H(9A)	0.9900	C(23A)-H(23E)	0.9800
C(9)-H(9AB)	0.9900	C(23A)-H(23F)	0.9800
C(10)-H(10A)	0.9800	C(24A)-C(25A)	1.497(15)
C(10)-H(10B)	0.9800	C(24A)-H(24C)	0.9900
C(10)-H(10C)	0.9800	C(24A)-H(24D)	0.9900
C(11)-C(12)	1.519(6)	C(25A)-C(26A)	1.522(16)
C(11)-H(11A)	0.9900	C(25A)-H(25C)	0.9900
C(11)-H(11B)	0.9900	C(25A)-H(25D)	0.9900
C(12)-C(13)	1.515(7)	C(26A)-H(26C)	0.9900
C(12)-H(12A)	0.9900	C(26A)-H(26D)	0.9900
C(12)-H(12B)	0.9900	C(27A)-H(27D)	0.9800
C(13)-H(13A)	0.9900	C(27A)-H(27E)	0.9800
C(13)-H(13B)	0.9900	C(27A)-H(27F)	0.9800
C(14)-H(14A)	0.9800	C(28A)-C(29A)	1.517(14)
C(14)-H(14B)	0.9800	C(28A)-H(28C)	0.9900
C(14)-H(14C)	0.9800	C(28A)-H(28D)	0.9900
Br(21)-Co(21)	2.4536(8)	C(29A)-H(29C)	0.9900
Co(21)-N(22A)	2.123(11)	C(29A)-H(29D)	0.9900
Co(21)-N(24)	2.125(11)	C(30A)-H(30D)	0.9800
Co(21)-N(23A)	2.133(12)	C(30A)-H(30E)	0.9800
Co(21)-N(21)	2.152(13)	C(30A)-H(30F)	0.9800
Co(21)-N(21A)	2.171(17)	C(31A)-C(32A)	1.502(16)
Co(21)-N(22)	2.209(9)	C(31A)-H(31C)	0.9900
Co(21)-N(23)	2.209(10)	C(31A)-H(31D)	0.9900
Co(21)-N(24A)	2.252(14)	C(32A)-C(33A)	1.517(17)
N(21)-C(34)	1.491(12)	C(32A)-H(32C)	0.9900
N(21)-C(33)	1.498(13)	C(32A)-H(32D)	0.9900
N(21)-C(21)	1.506(11)	C(33A)-H(33C)	0.9900
N(22)-C(23)	1.484(12)	C(33A)-H(33D)	0.9900
N(22)-C(22)	1.486(11)	C(34A)-H(34D)	0.9800
N(22)-C(24)	1.489(10)	C(34A)-H(34E)	0.9800
N(23)-C(27)	1.491(12)	C(34A)-H(34F)	0.9800
N(23)-C(28)	1.495(12)	O(11)-H(11D)	0.86(3)
N(23)-C(26)	1.497(12)	O(11)-H(11E)	0.84(3)
N(24)-C(30)	1.473(13)		
N(24)-C(29)	1.475(13)	N(1)-Co(1)-N(3)	140.14(15)
N(24)-C(31)	1.497(12)	N(1)-Co(1)-N(2)	83.83(14)
C(21)-C(22)	1.506(12)	N(3)-Co(1)-N(2)	92.37(14)
C(21)-H(21A)	0.9900	N(1)-Co(1)-N(4)	91.35(14)
C(21)-H(21B)	0.9900	N(3)-Co(1)-N(4)	84.01(14)
C(22)-H(22A)	0.9900	N(2)-Co(1)-N(4)	167.63(14)
C(22)-H(22B)	0.9900	N(1)-Co(1)-Br(1)	112.04(11)
C(23)-H(23A)	0.9800	N(3)-Co(1)-Br(1)	107.81(10)
C(23)-H(23B)	0.9800	N(2)-Co(1)-Br(1)	97.12(10)
C(23)-H(23C)	0.9800	N(4)-Co(1)-Br(1)	95.25(10)
C(24)-C(25)	1.516(13)	C(1)-N(1)-C(13)	106.9(3)
C(24)-H(24A)	0.9900	C(1)-N(1)-C(14)	108.9(4)
C(24)-H(24B)	0.9900	C(13)-N(1)-C(14)	109.0(4)
C(25)-C(26)	1.524(13)	C(1)-N(1)-Co(1)	106.4(3)
C(25)-H(25A)	0.9900	C(13)-N(1)-Co(1)	117.5(3)
C(25)-H(25B)	0.9900	C(14)-N(1)-Co(1)	107.8(3)
C(26)-H(26A)	0.9900	C(3)-N(2)-C(2)	106.8(4)
C(26)-H(26B)	0.9900	C(3)-N(2)-C(4)	108.6(4)
C(27)-H(27A)	0.9800	C(2)-N(2)-C(4)	108.3(3)

C(3)-N(2)-Co(1)	117.2(3)
C(2)-N(2)-Co(1)	103.4(3)
C(4)-N(2)-Co(1)	111.9(3)
C(8)-N(3)-C(7)	108.9(4)
C(8)-N(3)-C(6)	107.7(4)
C(7)-N(3)-C(6)	108.8(4)
C(8)-N(3)-Co(1)	106.6(3)
C(7)-N(3)-Co(1)	108.2(3)
C(6)-N(3)-Co(1)	116.5(3)
C(9)-N(4)-C(11)	109.1(4)
C(9)-N(4)-C(10)	107.2(4)
C(11)-N(4)-C(10)	108.2(4)
C(9)-N(4)-Co(1)	102.7(3)
C(11)-N(4)-Co(1)	111.7(3)
C(10)-N(4)-Co(1)	117.5(3)
N(1)-C(1)-C(2)	111.8(4)
N(1)-C(1)-H(1A)	109.3
C(2)-C(1)-H(1A)	109.3
N(1)-C(1)-H(1AB)	109.3
C(2)-C(1)-H(1AB)	109.3
H(1A)-C(1)-H(1AB)	107.9
N(2)-C(2)-C(1)	111.2(4)
N(2)-C(2)-H(2A)	109.4
C(1)-C(2)-H(2A)	109.4
N(2)-C(2)-H(2AB)	109.4
C(1)-C(2)-H(2AB)	109.4
H(2A)-C(2)-H(2AB)	108.0
N(2)-C(3)-H(3A)	109.5
N(2)-C(3)-H(3B)	109.5
H(3A)-C(3)-H(3B)	109.5
N(2)-C(3)-H(3C)	109.5
H(3A)-C(3)-H(3C)	109.5
H(3B)-C(3)-H(3C)	109.5
N(2)-C(4)-C(5)	114.0(4)
N(2)-C(4)-H(4A)	108.8
C(5)-C(4)-H(4A)	108.8
N(2)-C(4)-H(4AB)	108.8
C(5)-C(4)-H(4AB)	108.8
H(4A)-C(4)-H(4AB)	107.6
C(4)-C(5)-C(6)	114.2(4)
C(4)-C(5)-H(5A)	108.7
C(6)-C(5)-H(5A)	108.7
C(4)-C(5)-H(5AB)	108.7
C(6)-C(5)-H(5AB)	108.7
H(5A)-C(5)-H(5AB)	107.6
N(3)-C(6)-C(5)	114.5(4)
N(3)-C(6)-H(6A)	108.6
C(5)-C(6)-H(6A)	108.6
N(3)-C(6)-H(6AB)	108.6
C(5)-C(6)-H(6AB)	108.6
H(6A)-C(6)-H(6AB)	107.6
N(3)-C(7)-H(7A)	109.5
N(3)-C(7)-H(7B)	109.5
H(7A)-C(7)-H(7B)	109.5
N(3)-C(7)-H(7C)	109.5
H(7A)-C(7)-H(7C)	109.5
H(7B)-C(7)-H(7C)	109.5
N(3)-C(8)-C(9)	111.1(4)
N(3)-C(8)-H(8A)	109.4
C(9)-C(8)-H(8A)	109.4
N(3)-C(8)-H(8AB)	109.4
C(9)-C(8)-H(8AB)	109.4
H(8A)-C(8)-H(8AB)	108.0
N(4)-C(9)-C(8)	111.6(4)
N(4)-C(9)-H(9A)	109.3
C(8)-C(9)-H(9A)	109.3
N(4)-C(9)-H(9AB)	109.3
C(8)-C(9)-H(9AB)	109.3
H(9A)-C(9)-H(9AB)	108.0
N(4)-C(10)-H(10A)	109.5
N(4)-C(10)-H(10B)	109.5
H(10A)-C(10)-H(10B)	109.5
N(4)-C(10)-H(10C)	109.5
H(10A)-C(10)-H(10C)	109.5
H(10B)-C(10)-H(10C)	109.5
N(4)-C(11)-C(12)	114.4(4)
N(4)-C(11)-H(11A)	108.7
C(12)-C(11)-H(11A)	108.7
N(4)-C(11)-H(11B)	108.7
C(12)-C(11)-H(11B)	108.7
H(11A)-C(11)-H(11B)	107.6
C(13)-C(12)-C(11)	113.5(4)
C(13)-C(12)-H(12A)	108.9
C(11)-C(12)-H(12A)	108.9
C(13)-C(12)-H(12B)	108.9
C(11)-C(12)-H(12B)	108.9
H(12A)-C(12)-H(12B)	107.7
N(1)-C(13)-C(12)	115.6(4)
N(1)-C(13)-H(13A)	108.4
C(12)-C(13)-H(13A)	108.4
N(1)-C(13)-H(13B)	108.4
C(12)-C(13)-H(13B)	108.4
H(13A)-C(13)-H(13B)	107.5
N(1)-C(14)-H(14A)	109.5
N(1)-C(14)-H(14B)	109.5
H(14A)-C(14)-H(14B)	109.5
N(1)-C(14)-H(14C)	109.5
H(14A)-C(14)-H(14C)	109.5
H(14B)-C(14)-H(14C)	109.5
N(22A)-Co(21)-N(23A)	94.8(4)
N(24)-Co(21)-N(21)	94.4(4)
N(22A)-Co(21)-N(21A)	85.1(4)
N(23A)-Co(21)-N(21A)	142.2(5)
N(24)-Co(21)-N(22)	141.0(4)
N(21)-Co(21)-N(22)	82.6(3)
N(24)-Co(21)-N(23)	83.8(3)
N(21)-Co(21)-N(23)	166.2(4)
N(22)-Co(21)-N(23)	90.2(3)
N(22A)-Co(21)-N(24A)	168.2(5)
N(23A)-Co(21)-N(24A)	82.5(4)
N(21A)-Co(21)-N(24A)	90.0(5)
N(22A)-Co(21)-Br(21)	96.7(3)
N(24)-Co(21)-Br(21)	108.3(3)
N(23A)-Co(21)-Br(21)	109.8(4)
N(21)-Co(21)-Br(21)	98.7(3)
N(21A)-Co(21)-Br(21)	107.7(4)
N(22)-Co(21)-Br(21)	110.6(2)
N(23)-Co(21)-Br(21)	94.8(3)
N(24A)-Co(21)-Br(21)	95.1(3)
C(34)-N(21)-C(33)	107.6(12)
C(34)-N(21)-C(21)	105.9(9)
C(33)-N(21)-C(21)	106.9(10)
C(34)-N(21)-Co(21)	116.1(10)
C(33)-N(21)-Co(21)	112.1(11)
C(21)-N(21)-Co(21)	107.6(7)
C(23)-N(22)-C(22)	109.3(8)
C(23)-N(22)-C(24)	110.4(8)
C(22)-N(22)-C(24)	108.1(8)
C(23)-N(22)-Co(21)	105.6(6)
C(22)-N(22)-Co(21)	105.2(5)
C(24)-N(22)-Co(21)	117.9(6)
C(27)-N(23)-C(28)	106.8(8)
C(27)-N(23)-C(26)	107.6(8)
C(28)-N(23)-C(26)	108.0(8)
C(27)-N(23)-Co(21)	116.6(6)
C(28)-N(23)-Co(21)	103.9(6)
C(26)-N(23)-Co(21)	113.4(7)
C(30)-N(24)-C(29)	109.4(9)
C(30)-N(24)-C(31)	108.8(9)
C(29)-N(24)-C(31)	107.2(8)
C(30)-N(24)-Co(21)	107.9(7)
C(29)-N(24)-Co(21)	106.8(7)
C(31)-N(24)-Co(21)	116.5(7)
C(22)-C(21)-N(21)	109.5(8)
C(22)-C(21)-H(21A)	109.8
N(21)-C(21)-H(21A)	109.8
C(22)-C(21)-H(21B)	109.8
N(21)-C(21)-H(21B)	109.8
H(21A)-C(21)-H(21B)	108.2
N(22)-C(22)-C(21)	112.2(7)
N(22)-C(22)-H(22A)	109.2
C(21)-C(22)-H(22A)	109.2
N(22)-C(22)-H(22B)	109.2
C(21)-C(22)-H(22B)	109.2
H(22A)-C(22)-H(22B)	107.9
N(22)-C(23)-H(23A)	109.5
N(22)-C(23)-H(23B)	109.5
H(23A)-C(23)-H(23B)	109.5
N(22)-C(23)-H(23C)	109.5
H(23A)-C(23)-H(23C)	109.5
H(23B)-C(23)-H(23C)	109.5
N(22)-C(24)-C(25)	115.5(10)
N(22)-C(24)-H(24A)	108.4
C(25)-C(24)-H(24A)	108.4
N(22)-C(24)-H(24B)	108.4
C(25)-C(24)-H(24B)	108.4
H(24A)-C(24)-H(24B)	107.5
C(24)-C(25)-C(26)	112.4(9)
C(24)-C(25)-H(25A)	109.1
C(26)-C(25)-H(25A)	109.1
C(24)-C(25)-H(25B)	109.1
C(26)-C(25)-H(25B)	109.1
H(25A)-C(25)-H(25B)	107.9
N(23)-C(26)-C(25)	113.9(10)
N(23)-C(26)-H(26A)	108.8
C(25)-C(26)-H(26A)	108.8
N(23)-C(26)-H(26B)	108.8
C(25)-C(26)-H(26B)	108.8
H(26A)-C(26)-H(26B)	107.7
N(23)-C(27)-H(27A)	109.5
N(23)-C(27)-H(27B)	109.5
H(27A)-C(27)-H(27B)	109.5
N(23)-C(27)-H(27C)	109.5
H(27A)-C(27)-H(27C)	109.5
H(27B)-C(27)-H(27C)	109.5
N(23)-C(28)-C(29)	110.9(9)
N(23)-C(28)-H(28A)	109.5
C(29)-C(28)-H(28A)	109.5
N(23)-C(28)-H(28B)	109.5
C(29)-C(28)-H(28B)	109.5

H(28A)-C(28)-H(28B)	108.1
N(24)-C(29)-C(28)	111.5(8)
N(24)-C(29)-H(29A)	109.3
C(28)-C(29)-H(29A)	109.3
N(24)-C(29)-H(29B)	109.3
C(28)-C(29)-H(29B)	109.3
H(29A)-C(29)-H(29B)	108.0
N(24)-C(30)-H(30A)	109.5
N(24)-C(30)-H(30B)	109.5
H(30A)-C(30)-H(30B)	109.5
N(24)-C(30)-H(30C)	109.5
H(30A)-C(30)-H(30C)	109.5
H(30B)-C(30)-H(30C)	109.5
N(24)-C(31)-C(32)	114.0(13)
N(24)-C(31)-H(31A)	108.8
C(32)-C(31)-H(31A)	108.8
N(24)-C(31)-H(31B)	108.8
C(32)-C(31)-H(31B)	108.8
H(31A)-C(31)-H(31B)	107.6
C(31)-C(32)-C(33)	112.5(15)
C(31)-C(32)-H(32A)	109.1
C(33)-C(32)-H(32A)	109.1
C(31)-C(32)-H(32B)	109.1
C(33)-C(32)-H(32B)	109.1
H(32A)-C(32)-H(32B)	107.8
N(21)-C(33)-C(32)	112.4(13)
N(21)-C(33)-H(33A)	109.1
C(32)-C(33)-H(33A)	109.1
N(21)-C(33)-H(33B)	109.1
C(32)-C(33)-H(33B)	109.1
H(33A)-C(33)-H(33B)	107.9
N(21)-C(34)-H(34A)	109.5
N(21)-C(34)-H(34B)	109.5
H(34A)-C(34)-H(34B)	109.5
N(21)-C(34)-H(34C)	109.5
H(34A)-C(34)-H(34C)	109.5
H(34B)-C(34)-H(34C)	109.5
C(34A)-N(21A)-C(33A)	110.2(16)
C(34A)-N(21A)-C(21A)	110.2(12)
C(33A)-N(21A)-C(21A)	105.7(13)
C(34A)-N(21A)-Co(21)	111.0(13)
C(33A)-N(21A)-Co(21)	115.8(14)
C(21A)-N(21A)-Co(21)	103.4(9)
C(22A)-N(22A)-C(24A)	109.2(10)
C(22A)-N(22A)-C(23A)	105.6(10)
C(24A)-N(22A)-C(23A)	106.0(10)
C(22A)-N(22A)-Co(21)	104.7(7)
C(24A)-N(22A)-Co(21)	113.2(8)
C(23A)-N(22A)-Co(21)	117.7(8)
C(27A)-N(23A)-C(28A)	109.8(11)
C(27A)-N(23A)-C(26A)	108.9(10)
C(28A)-N(23A)-C(26A)	107.5(10)
C(27A)-N(23A)-Co(21)	109.3(8)
C(28A)-N(23A)-Co(21)	107.5(8)
C(26A)-N(23A)-Co(21)	113.8(9)
C(30A)-N(24A)-C(29A)	105.5(11)
C(30A)-N(24A)-C(31A)	108.1(11)
C(29A)-N(24A)-C(31A)	109.3(11)
C(30A)-N(24A)-Co(21)	117.6(9)
C(29A)-N(24A)-Co(21)	103.5(8)
C(31A)-N(24A)-Co(21)	112.3(10)
N(21A)-C(21A)-C(22A)	110.1(10)
N(21A)-C(21A)-H(21C)	109.6
C(22A)-C(21A)-H(21C)	109.6
N(21A)-C(21A)-H(21D)	109.6
C(22A)-C(21A)-H(21D)	109.6
H(21C)-C(21A)-H(21D)	108.1
N(22A)-C(22A)-C(21A)	110.6(10)
N(22A)-C(22A)-H(22C)	109.5
C(21A)-C(22A)-H(22C)	109.5
N(22A)-C(22A)-H(22D)	109.5
C(21A)-C(22A)-H(22D)	109.5
H(22C)-C(22A)-H(22D)	108.1
N(22A)-C(23A)-H(23D)	109.5
N(22A)-C(23A)-H(23E)	109.5
H(23D)-C(23A)-H(23E)	109.5
N(22A)-C(23A)-H(23F)	109.5
H(23D)-C(23A)-H(23F)	109.5
H(23E)-C(23A)-H(23F)	109.5
N(22A)-C(24A)-C(25A)	114.3(13)
N(22A)-C(24A)-H(24C)	108.7
C(25A)-C(24A)-H(24C)	108.7
N(22A)-C(24A)-H(24D)	108.7
C(25A)-C(24A)-H(24D)	108.7
H(24C)-C(24A)-H(24D)	107.6
C(24A)-C(25A)-C(26A)	115.1(12)
C(24A)-C(25A)-H(25C)	108.5
C(26A)-C(25A)-H(25C)	108.5
C(24A)-C(25A)-H(25D)	108.5
C(26A)-C(25A)-H(25D)	108.5
H(25C)-C(25A)-H(25D)	107.5
N(23A)-C(26A)-C(25A)	114.9(13)
N(23A)-C(26A)-H(26C)	108.6
C(25A)-C(26A)-H(26C)	108.6
N(23A)-C(26A)-H(26D)	108.6
C(25A)-C(26A)-H(26D)	108.6
H(26C)-C(26A)-H(26D)	107.5
N(23A)-C(27A)-H(27D)	109.5
N(23A)-C(27A)-H(27E)	109.5
H(27D)-C(27A)-H(27E)	109.5
N(23A)-C(27A)-H(27F)	109.5
H(27D)-C(27A)-H(27F)	109.5
H(27E)-C(27A)-H(27F)	109.5
N(23A)-C(28A)-C(29A)	111.0(10)
N(23A)-C(28A)-H(28C)	109.4
C(29A)-C(28A)-H(28C)	109.4
N(23A)-C(28A)-H(28D)	109.4
C(29A)-C(28A)-H(28D)	109.4
H(28C)-C(28A)-H(28D)	108.0
N(24A)-C(29A)-C(28A)	109.4(10)
N(24A)-C(29A)-H(29C)	109.8
C(28A)-C(29A)-H(29C)	109.8
N(24A)-C(29A)-H(29D)	109.8
C(28A)-C(29A)-H(29D)	109.8
H(29C)-C(29A)-H(29D)	108.2
N(24A)-C(30A)-H(30D)	109.5
N(24A)-C(30A)-H(30E)	109.5
H(30D)-C(30A)-H(30E)	109.5
N(24A)-C(30A)-H(30F)	109.5
H(30D)-C(30A)-H(30F)	109.5
H(30E)-C(30A)-H(30F)	109.5
N(24A)-C(31A)-C(32A)	116.0(17)
N(24A)-C(31A)-H(31C)	108.3
C(32A)-C(31A)-H(31C)	108.3
N(24A)-C(31A)-H(31D)	108.3
C(32A)-C(31A)-H(31D)	108.3
H(31C)-C(31A)-H(31D)	107.4
C(31A)-C(32A)-C(33A)	114.7(18)
C(31A)-C(32A)-H(32C)	108.6
C(33A)-C(32A)-H(32C)	108.6
C(31A)-C(32A)-H(32D)	108.6
C(33A)-C(32A)-H(32D)	108.6
H(32C)-C(32A)-H(32D)	107.6
N(21A)-C(33A)-C(32A)	116.2(17)
N(21A)-C(33A)-H(33C)	108.2
C(32A)-C(33A)-H(33C)	108.2
N(21A)-C(33A)-H(33D)	108.2
C(32A)-C(33A)-H(33D)	108.2
H(33C)-C(33A)-H(33D)	107.4
N(21A)-C(34A)-H(34D)	109.5
N(21A)-C(34A)-H(34E)	109.5
H(34D)-C(34A)-H(34E)	109.5
N(21A)-C(34A)-H(34F)	109.5
H(34D)-C(34A)-H(34F)	109.5
H(34E)-C(34A)-H(34F)	109.5
H(11D)-O(11)-H(11E)	105(4)

Table S93. Anisotropic displacement parameters (Å^{2}x 10^3) for **[(TMC)Co^{II}(Br)]Br**.
The anisotropic displacement factor exponent takes the form: -2p^2[h^2 a*^{2}U^{11} + ... + 2 h k a* b* U^{12}]

	U^{11}	U^{22}	U^{33}	U^{23}	U^{13}	U^{12}
Br(1)	15(1)	12(1)	15(1)	-4(1)	-2(1)	-3(1)
Co(1)	6(1)	6(1)	9(1)	-2(1)	-1(1)	0(1)
N(1)	9(2)	9(2)	10(2)	0(2)	1(2)	-1(2)
N(2)	11(2)	9(2)	9(2)	-2(2)	-1(2)	2(2)
N(3)	11(2)	13(2)	8(2)	-1(2)	-2(2)	3(2)
N(4)	8(2)	12(2)	12(2)	1(2)	-4(2)	-1(2)
C(1)	6(2)	18(3)	16(3)	0(2)	0(2)	0(2)
C(2)	14(2)	11(2)	14(3)	-1(2)	2(2)	-3(2)
C(3)	21(3)	19(3)	9(3)	1(2)	0(2)	1(2)
C(4)	19(3)	10(2)	18(3)	4(2)	3(2)	-4(2)
C(5)	26(3)	5(2)	22(3)	2(2)	2(2)	-1(2)
C(6)	17(3)	8(2)	23(3)	-5(2)	-2(2)	1(2)
C(7)	18(3)	19(3)	15(3)	-8(2)	-5(2)	7(2)
C(8)	12(2)	16(3)	13(3)	-3(2)	2(2)	7(2)
C(9)	8(2)	21(3)	15(3)	-2(2)	2(2)	1(2)
C(10)	16(3)	14(3)	20(3)	4(2)	2(2)	-6(2)
C(11)	14(2)	19(3)	10(3)	-2(2)	-1(2)	-2(2)
C(12)	19(3)	16(3)	10(3)	1(2)	-8(2)	-1(2)
C(13)	10(2)	16(2)	11(3)	-2(2)	-1(2)	-1(2)
C(14)	15(2)	9(2)	22(3)	-2(2)	-1(2)	7(2)
Br(21)	16(1)	13(1)	19(1)	-4(1)	4(1)	2(1)
Co(21)	10(1)	8(1)	8(1)	0(1)	0(1)	2(1)
N(21)	15(2)	20(4)	8(2)	-3(3)	-2(2)	-6(2)
N(22)	26(2)	6(2)	18(2)	-1(2)	3(2)	-5(2)
N(23)	14(4)	14(4)	13(5)	-1(4)	1(4)	6(4)
N(24)	9(4)	21(5)	9(4)	2(4)	4(3)	9(4)
C(21)	15(2)	20(4)	8(2)	-3(3)	-2(2)	-6(2)
C(22)	15(4)	19(4)	11(4)	-3(3)	4(3)	-3(4)
C(23)	16(5)	20(5)	8(6)	1(5)	2(4)	-4(4)
C(24)	26(2)	6(2)	18(2)	-1(2)	3(2)	-5(2)
C(25)	26(2)	6(2)	18(2)	-1(2)	3(2)	-5(2)
C(26)	27(4)	7(3)	17(6)	4(4)	5(4)	4(3)
C(27)	20(5)	19(5)	9(5)	2(4)	2(4)	5(4)
C(28)	15(4)	24(5)	19(5)	5(4)	-2(4)	5(4)
C(29)	11(5)	28(6)	20(5)	12(5)	0(4)	6(4)
C(30)	10(5)	31(7)	18(6)	1(6)	-4(4)	-2(5)
C(31)	13(2)	19(2)	13(2)	-3(2)	5(2)	0(2)
C(32)	13(2)	19(2)	13(2)	-3(2)	5(2)	0(2)
C(33)	17(4)	19(5)	13(3)	-1(3)	3(3)	-4(4)
C(34)	12(5)	18(5)	11(8)	1(5)	-8(4)	-5(4)
N(21A)	15(2)	20(4)	8(2)	-3(3)	-2(2)	-6(2)
N(22A)	26(2)	6(2)	18(2)	-1(2)	3(2)	-5(2)
N(23A)	19(5)	11(5)	17(6)	-5(4)	0(5)	7(4)
N(24A)	12(5)	17(7)	9(5)	-1(5)	0(4)	6(6)
C(21A)	15(2)	20(4)	8(2)	-3(3)	-2(2)	-6(2)
C(22A)	15(5)	22(6)	22(5)	4(5)	-6(4)	-8(5)
C(23A)	18(6)	21(7)	21(8)	-4(7)	5(6)	-12(5)
C(24A)	26(2)	6(2)	18(2)	-1(2)	3(2)	-5(2)
C(25A)	26(2)	6(2)	18(2)	-1(2)	3(2)	-5(2)
C(26A)	27(4)	7(3)	17(6)	4(4)	5(4)	4(3)
C(27A)	26(8)	13(6)	19(7)	-2(5)	0(6)	8(6)
C(28A)	18(6)	24(6)	13(6)	9(6)	-4(5)	8(5)
C(29A)	17(5)	25(6)	10(5)	2(5)	-9(4)	-2(5)
C(30A)	19(7)	15(6)	17(7)	-3(6)	-1(5)	-7(6)
C(31A)	13(2)	19(2)	13(2)	-3(2)	5(2)	0(2)
C(32A)	13(2)	19(2)	13(2)	-3(2)	5(2)	0(2)
C(33A)	17(4)	19(5)	13(3)	-1(3)	3(3)	-4(4)
C(34A)	12(5)	18(5)	11(8)	1(5)	-8(4)	-5(4)
Br(11)	19(1)	19(1)	20(1)	-4(1)	0(1)	3(1)
Br(12)	19(1)	22(1)	28(1)	-6(1)	-5(1)	8(1)
O(11)	33(2)	25(2)	28(2)	-3(2)	2(2)	3(2)

Table S94. Hydrogen coordinates (x 10^4) and isotropic displacement parameters ($Å^2$x 10^3) for **[(TMC)Co^{II}(Br)]Br**.

	x	y	z	U(eq)
H(1A)	4154	1722	3427	16
H(1AB)	3706	3370	3546	16
H(2A)	4660	3673	4167	16
H(2AB)	4141	2043	4211	16
H(3A)	6431	1578	4537	24
H(3B)	5433	1583	4739	24
H(3C)	5881	3178	4571	24
H(4A)	4939	-74	3717	19
H(4AB)	4931	-289	4255	19
H(5A)	5981	-2003	4020	21
H(5AB)	6557	-575	4211	21
H(6A)	7093	-1605	3501	19
H(6AB)	6195	-978	3268	19
H(7A)	8212	26	3749	26
H(7B)	7586	1083	4066	26
H(7C)	8180	1876	3676	26
H(8A)	7001	243	2755	16
H(8AB)	7999	111	2956	16
H(9A)	8120	2834	2988	18
H(9AB)	7869	2323	2481	18
H(10A)	7465	5102	2456	25
H(10B)	7551	5311	2994	25
H(10C)	6625	5718	2744	25
H(11A)	6105	1829	2441	17
H(11B)	6550	3172	2140	17
H(12A)	5463	4961	2476	18
H(12B)	5066	3729	2121	18
H(13A)	4097	3772	2723	15
H(13B)	4604	2132	2741	15
H(14A)	5327	5836	3150	23
H(14B)	5058	5501	3667	23
H(14C)	4292	5670	3289	23
H(21A)	852	4271	5236	17
H(21B)	1457	3933	4797	17
H(22A)	663	1665	5027	18
H(22B)	1726	1385	5035	18
H(23A)	263	3176	5837	22
H(23B)	257	1716	6175	22
H(23C)	-193	1570	5684	22
H(24A)	1772	-558	5514	20
H(24B)	704	-685	5550	20
H(25A)	1264	-2038	6155	20
H(25B)	844	-473	6361	20
H(26A)	2341	-1223	6642	20
H(26B)	2717	-675	6159	20
H(27A)	1041	1236	6827	24
H(27B)	1748	2316	7089	24
H(27C)	1763	465	7162	24
H(28A)	3404	421	6916	23
H(28B)	3216	2255	6963	23
H(29A)	4490	1886	6510	23
H(29B)	3942	758	6177	23
H(30A)	3662	5394	6161	29
H(30B)	4472	4533	6415	29
H(30C)	3486	4511	6631	29
H(31A)	4142	1950	5540	18
H(31B)	4814	3204	5754	18
H(32A)	4362	4270	5081	18
H(32B)	3831	5233	5461	18
H(33A)	2950	4323	4798	20
H(33B)	2993	2632	5028	20
H(34A)	2226	6361	5113	21
H(34B)	2655	6275	5610	21
H(34C)	1588	6213	5548	21
H(21C)	1841	1885	4990	17
H(21D)	1423	3470	4793	17
H(22C)	500	3716	5441	24
H(22D)	310	2079	5197	24
H(23D)	-341	1561	5939	30
H(23E)	73	3110	6159	30
H(23F)	289	1443	6378	30
H(24C)	1526	22	5440	20
H(24D)	507	-257	5590	20
H(25C)	1305	-1915	6018	20
H(25D)	1037	-532	6355	20
H(26C)	2563	-1412	6431	20
H(26D)	2762	-731	5937	20
H(27D)	1545	928	6840	29
H(27E)	2311	2118	6999	29
H(27F)	2446	282	7069	29
H(28C)	3894	630	6187	22
H(28D)	3801	404	6725	22
H(29C)	3654	3137	6821	21
H(29D)	4580	2751	6567	21
H(30D)	3615	6052	6041	26
H(30E)	4396	5500	6378	26
H(30F)	3378	5572	6552	26
H(31C)	4249	2254	5693	18
H(31D)	4853	3715	5834	18
H(32C)	4500	4096	5099	18
H(32D)	3904	5320	5375	18
H(33C)	3104	4049	4780	20

H(33D)	3225	2432	5041	20
H(34D)	2077	5898	5009	21
H(34E)	2485	6094	5508	21
H(34F)	1447	5658	5444	21
H(11D)	4040(40)	7170(60)	4573(18)	43
H(11E)	3670(30)	6190(70)	4270(20)	43

Table S95. Hydrogen bonds for **[(TMC)Co^{II}(Br)]Br** [Å and °].

D-H...A	d(D-H)	d(H...A)	d(D...A)	<(DHA)
O(11)-H(11D)...Br(11)	0.86(3)	2.48(3)	3.329(4)	170(6)
O(11)-H(11E)...Br(1)#1	0.84(3)	2.76(3)	3.591(4)	178(6)

Symmetry transformations used to generate equivalent atoms: #1 x-1/2,-y+1,z

[(TMC)Co^{II}(NO$_3$)]B$_{Ar}$F

The structure of **[(TMC)Co^{II}(NO$_3$)]B$_{Ar}$F** was solved in the triclinic space group $P\bar{1}$ and the asymmetric unit contains one full molecule (anion and cation). All of the CF_3 groups of the anion were found to be disordered and were modelled over three positions. The disorder was refined with the help of 1,2 and 1,3-distance restraints, similarity restraints on anisotropic displacement parameters, restraints of the anisotropic displacement parameters for the fluorine atoms towards isotropic behavior and advanced rigid bond restraints. To allow for full anisotropic refinement a few atoms were set to have the same anisotropic displacement parameters. All of the disorder ratios were allowed to refine freely and converged to 0.145(3):0.242(3):0.612(3), 0.667(3):0.261(3):0.072(3), 0.505(3):0.083(3):0.412(3), 0.323(3):0.329(3):0.348(3), 0.560(3):0.121(2):0.319(3), 0.297(3):0.284(3):0.419(3), 0.555(3):0.297(3):0.148(3) and 0.524(3):0.179(3):0.297(3).

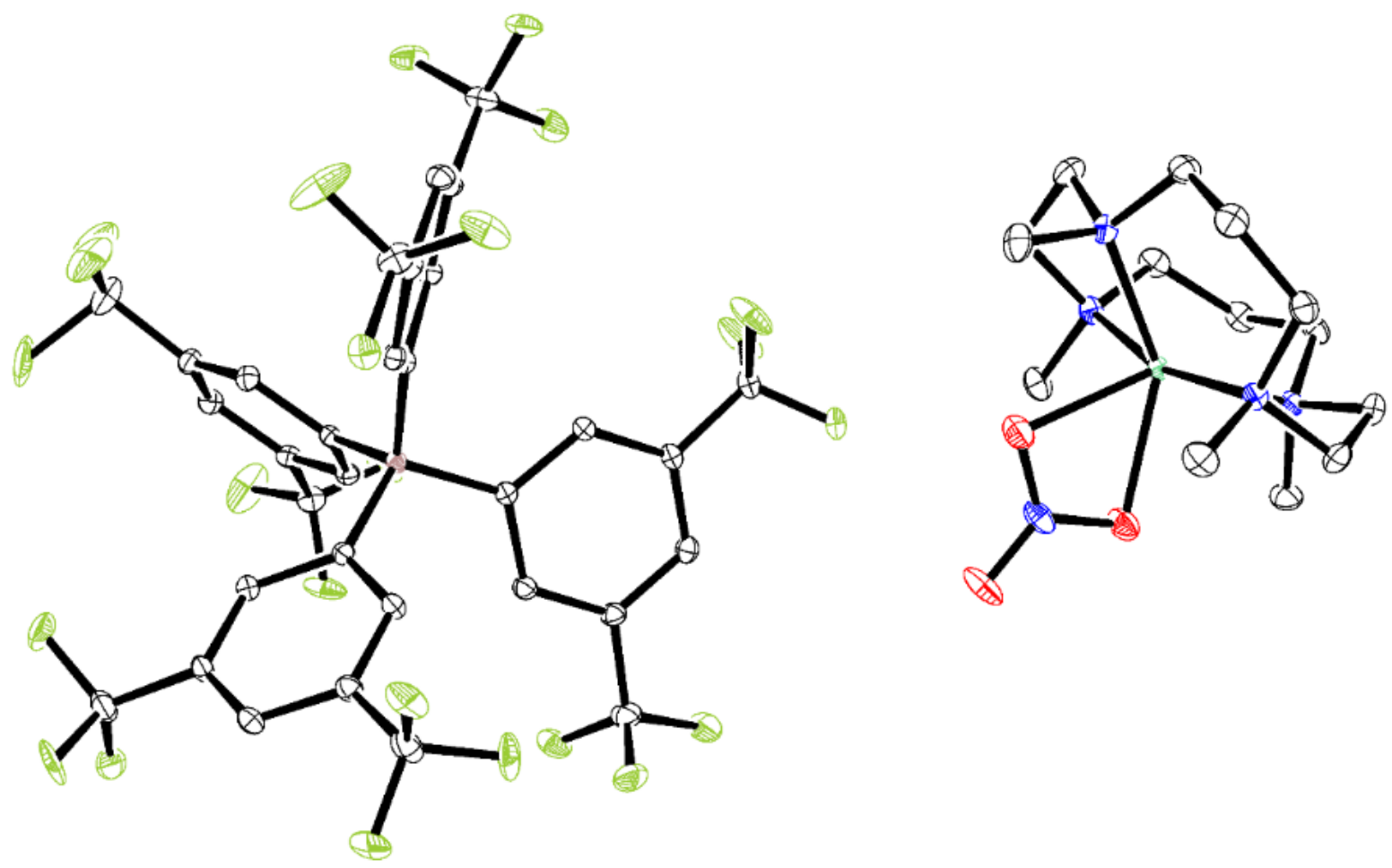

Figure S19. Molecular structure of **[(TMC)Co^{II}(NO$_3$)]B$_{Ar}$F**.

Table S96. Crystal data and structure refinement for **[(TMC)Co^{II}(NO$_3$)]B$_{Ar}$F**.

CCDC No	2062720	
Empirical formula	$C_{46}H_{44}BCoF_{24}N_5O_3$	
Formula weight	1240.60	
Temperature	100(2) K	
Wavelength	0.71073 Å	
Crystal system	Triclinic	
Space group	$P\bar{1}$	
Unit cell dimensions	a = 11.8795(4) Å	α = 84.7528(15)°
	b = 13.5264(5) Å	β = 75.8545(14)°
	c = 17.8028(6) Å	γ = 64.8499(14)°
Volume	2510.71(15) Å^3	
Z	2	
Density (calculated)	1.641 Mg/m^3	
Absorption coefficient	0.478 mm^{-1}	
F(000)	1254	
Crystal size	0.419 x 0.361 x 0.206 mm^3	
Theta range for data collection	2.360 to 32.032°.	
Index ranges	-17 ≤ h ≤ 17, -20 ≤ k ≤ 20, -26 ≤ l ≤ 26	
Reflections collected	537306	
Independent reflections	17482 [R(int) = 0.0518]	
Completeness to theta = 25.242°	99.9 %	
Absorption correction	Semi-empirical from equivalents	
Refinement method	Full-matrix least-squares on F^2	
Data / restraints / parameters	17482 / 10367 / 1163	
Goodness-of-fit on F^2	1.044	
Final R indices [I>2σ(I)]	R1 = 0.0311, wR2 = 0.0756	
R indices (all data)	R1 = 0.0385, wR2 = 0.0789	
Largest diff. peak and hole	0.500 and -0.354 eÅ^{-3}	

Table S97. Atomic coordinates (x 10^4) and equivalent isotropic displacement parameters ($Å^2$x 10^3) for **[(TMC)Co^{II}(NO$_3$)]B$_{Ar}$F**. U(eq) is defined as one third of the trace of the orthogonalized U^{ij} tensor.

	x	y	z	U(eq)
Co(1)	6658(1)	616(1)	6249(1)	9(1)
O(1)	8002(1)	-31(1)	5115(1)	20(1)
O(2)	6010(1)	918(1)	5159(1)	21(1)
O(3)	7326(1)	299(1)	4043(1)	28(1)
N(1)	8399(1)	-137(1)	6710(1)	12(1)
N(2)	6674(1)	2228(1)	6245(1)	13(1)
N(3)	4628(1)	1512(1)	6810(1)	13(1)
N(4)	6537(1)	-939(1)	6563(1)	13(1)
N(5)	7121(1)	392(1)	4744(1)	17(1)
C(1)	8422(1)	560(1)	7302(1)	15(1)
C(2)	8426(1)	1646(1)	6997(1)	17(1)
C(3)	7138(1)	2482(1)	6878(1)	17(1)
C(4)	7403(1)	2441(1)	5492(1)	18(1)
C(5)	5303(1)	2991(1)	6334(1)	18(1)
C(6)	4473(1)	2624(1)	6981(1)	18(1)
C(7)	3750(1)	1642(1)	6299(1)	21(1)
C(8)	4216(1)	1023(1)	7552(1)	17(1)
C(9)	4312(1)	-122(1)	7462(1)	18(1)
C(10)	5671(1)	-1000(1)	7315(1)	16(1)
C(11)	6205(1)	-1345(1)	5939(1)	19(1)
C(12)	7865(1)	-1686(1)	6610(1)	16(1)
C(13)	8366(1)	-1155(1)	7086(1)	16(1)
C(14)	9612(1)	-450(1)	6106(1)	19(1)
F(21)	-1247(10)	6413(9)	5129(5)	19(2)
F(22)	556(9)	6495(12)	4606(8)	22(2)
F(23)	-1173(8)	7687(5)	4312(6)	21(2)
F(21A)	-1432(16)	6597(16)	5088(9)	25(2)
F(22A)	537(15)	6350(20)	4673(15)	24(3)
F(23A)	-940(18)	7661(9)	4225(11)	26(2)
F(21B)	-1060(30)	6280(30)	5147(11)	20(3)
F(22B)	497(18)	6670(30)	4540(19)	20(3)
F(23B)	-1410(20)	7640(11)	4377(17)	23(4)
F(24)	-1539(9)	3311(7)	3923(5)	26(2)
F(25)	-2850(6)	4656(7)	3391(7)	36(2)
F(26)	-1298(9)	3336(7)	2690(5)	16(1)
F(24A)	-1920(20)	3583(19)	4007(10)	27(4)
F(25A)	-2753(16)	4660(20)	3146(16)	26(4)
F(26A)	-1140(30)	3162(18)	2796(14)	17(4)
F(24B)	-1285(12)	3153(9)	3838(6)	24(1)
F(25B)	-2859(8)	4578(10)	3587(9)	41(2)
F(26B)	-1448(12)	3511(9)	2659(6)	28(2)
F(31)	3818(18)	835(11)	948(12)	26(3)
F(32)	1899(13)	1107(14)	942(13)	22(3)
F(33)	3310(30)	856(13)	-141(7)	24(3)
F(31A)	3654(19)	818(15)	1023(11)	24(2)
F(32A)	1835(13)	1127(16)	800(9)	27(2)
F(33A)	3450(30)	848(19)	-138(8)	24(2)
F(31B)	3816(16)	853(11)	935(11)	32(3)
F(32B)	1853(11)	1190(12)	1014(10)	34(3)
F(33B)	3172(19)	847(12)	-104(6)	30(2)
F(34)	850(20)	5755(12)	-901(10)	23(4)
F(35)	1440(30)	4191(17)	-1437(11)	28(3)
F(36)	2827(14)	4820(20)	-1440(12)	24(3)
F(34A)	810(20)	5713(12)	-909(13)	22(2)
F(35A)	1620(30)	4141(18)	-1484(12)	33(3)
F(36A)	2833(16)	4910(20)	-1383(13)	29(3)
F(34B)	1004(18)	5783(10)	-931(13)	22(2)
F(35B)	1350(30)	4250(18)	-1417(12)	38(3)
F(36B)	2928(14)	4660(20)	-1437(13)	32(3)
F(41)	7006(14)	1691(9)	1026(9)	24(3)
F(42)	7135(11)	3216(9)	664(4)	21(2)
F(43)	8056(8)	2281(13)	1568(6)	26(2)
F(41A)	7110(30)	1617(17)	1110(20)	25(4)
F(42A)	7040(30)	3150(30)	622(13)	21(2)
F(43A)	8049(19)	2440(30)	1530(20)	28(4)
F(41B)	6930(20)	1779(17)	953(15)	23(2)
F(42B)	7257(19)	3238(16)	730(9)	27(2)
F(43B)	7993(14)	2134(19)	1629(11)	34(3)
F(44)	4711(5)	4681(4)	4209(3)	30(1)
F(45)	5583(3)	2939(3)	4225(2)	39(1)
F(46)	3566(4)	3798(4)	4484(3)	27(1)
F(44A)	4392(14)	4821(9)	4227(10)	43(3)
F(45A)	5774(7)	3177(7)	4170(5)	33(2)
F(46A)	3774(11)	3547(11)	4515(8)	26(2)
F(44B)	5239(14)	4362(14)	4176(11)	26(3)
F(45B)	5084(18)	2874(9)	4371(8)	27(3)
F(46B)	3395(9)	4353(15)	4448(6)	33(2)
F(51)	4676(7)	7673(6)	1287(4)	27(1)
F(52)	3950(10)	8585(5)	352(5)	36(2)
F(53)	5061(6)	6862(6)	216(4)	23(1)
F(51A)	5026(8)	7083(9)	1199(6)	32(2)
F(52A)	3912(7)	8687(5)	922(8)	43(2)
F(53A)	4771(10)	7417(12)	40(4)	43(2)
F(51B)	4582(14)	7786(14)	1284(7)	43(2)
F(52B)	4016(17)	8463(10)	223(8)	34(2)
F(53B)	5111(11)	6756(10)	320(7)	31(2)
F(54)	-718(9)	9830(6)	1348(10)	52(2)
F(55)	-1493(11)	8616(12)	1432(7)	42(3)
F(56)	-563(7)	9023(9)	348(4)	49(2)
F(54A)	-555(10)	9788(6)	876(5)	37(2)

F(55A)	-1198(9)	9219(7)	1943(5)	44(2)
F(56A)	-1265(10)	8550(10)	924(6)	45(3)
F(54B)	-1085(4)	9517(3)	1847(2)	47(1)
F(55B)	-1423(3)	8526(3)	1159(2)	26(1)
F(56B)	-574(4)	9587(3)	607(2)	50(1)
C(21)	916(1)	5076(1)	2533(1)	9(1)
C(22)	593(1)	5798(1)	3148(1)	11(1)
C(23)	-354(1)	5879(1)	3811(1)	12(1)
C(24)	-1075(1)	5282(1)	3875(1)	13(1)
C(25)	-810(1)	4599(1)	3255(1)	11(1)
C(26)	169(1)	4486(1)	2606(1)	10(1)
C(27)	-562(1)	6613(1)	4458(1)	16(1)
C(28)	-1609(1)	3975(1)	3315(1)	16(1)
C(31)	2190(1)	4262(1)	1081(1)	10(1)
C(32)	2500(1)	3137(1)	1116(1)	11(1)
C(33)	2536(1)	2552(1)	498(1)	12(1)
C(34)	2269(1)	3069(1)	-190(1)	13(1)
C(35)	1977(1)	4175(1)	-242(1)	13(1)
C(36)	1935(1)	4761(1)	378(1)	12(1)
C(37)	2868(1)	1354(1)	561(1)	20(1)
C(38)	1792(1)	4729(1)	-997(1)	19(1)
C(41)	3464(1)	4269(1)	2146(1)	10(1)
C(42)	4648(1)	3715(1)	1632(1)	12(1)
C(43)	5786(1)	3197(1)	1880(1)	13(1)
C(44)	5805(1)	3205(1)	2656(1)	13(1)
C(45)	4648(1)	3747(1)	3177(1)	11(1)
C(46)	3508(1)	4257(1)	2929(1)	11(1)
C(47)	6999(1)	2598(1)	1292(1)	19(1)
C(48)	4629(1)	3803(1)	4017(1)	13(1)
C(51)	2044(1)	6152(1)	1535(1)	10(1)
C(52)	3098(1)	6411(1)	1282(1)	11(1)
C(53)	2975(1)	7436(1)	988(1)	11(1)
C(54)	1796(1)	8255(1)	938(1)	13(1)
C(55)	732(1)	8020(1)	1195(1)	13(1)
C(56)	855(1)	6996(1)	1483(1)	12(1)
C(57)	4155(1)	7637(1)	711(1)	15(1)
C(58)	-564(1)	8900(1)	1187(1)	22(1)
B(1)	2150(1)	4932(1)	1821(1)	9(1)

Table S98. Bond lengths [Å] and angles [°] for **[(TMC)Co^{II}(NO$_3$)]B$_{Ar}$F**.

Co(1)-N(4)	2.1818(8)	F(35B)-C(38)	1.346(10)
Co(1)-N(2)	2.1887(8)	F(36B)-C(38)	1.352(10)
Co(1)-O(2)	2.2003(8)	F(41)-C(47)	1.351(7)
Co(1)-N(3)	2.2006(9)	F(42)-C(47)	1.353(6)
Co(1)-N(1)	2.2087(8)	F(43)-C(47)	1.344(7)
Co(1)-O(1)	2.2249(8)	F(41A)-C(47)	1.338(12)
O(1)-N(5)	1.2714(12)	F(42A)-C(47)	1.350(12)
O(2)-N(5)	1.2712(13)	F(43A)-C(47)	1.341(12)
O(3)-N(5)	1.2198(11)	F(41B)-C(47)	1.349(11)
N(1)-C(13)	1.4871(13)	F(42B)-C(47)	1.327(10)
N(1)-C(1)	1.4886(12)	F(43B)-C(47)	1.340(10)
N(1)-C(14)	1.4899(13)	F(44)-C(48)	1.315(4)
N(2)-C(4)	1.4845(13)	F(45)-C(48)	1.336(3)
N(2)-C(5)	1.4894(13)	F(46)-C(48)	1.334(4)
N(2)-C(3)	1.4919(13)	F(44A)-C(48)	1.353(9)
N(3)-C(6)	1.4866(13)	F(45A)-C(48)	1.343(7)
N(3)-C(7)	1.4906(13)	F(46A)-C(48)	1.319(9)
N(3)-C(8)	1.4907(13)	F(44B)-C(48)	1.336(10)
N(4)-C(11)	1.4829(13)	F(45B)-C(48)	1.309(10)
N(4)-C(12)	1.4872(13)	F(46B)-C(48)	1.386(9)
N(4)-C(10)	1.4944(13)	F(51)-C(57)	1.334(4)
C(1)-C(2)	1.5208(14)	F(52)-C(57)	1.332(5)
C(1)-H(1A)	0.9900	F(53)-C(57)	1.334(5)
C(1)-H(1AB)	0.9900	F(51A)-C(57)	1.431(6)
C(2)-C(3)	1.5197(15)	F(52A)-C(57)	1.392(6)
C(2)-H(2A)	0.9900	F(53A)-C(57)	1.226(7)
C(2)-H(2AB)	0.9900	F(51B)-C(57)	1.309(9)
C(3)-H(3A)	0.9900	F(52B)-C(57)	1.329(9)
C(3)-H(3AB)	0.9900	F(53B)-C(57)	1.346(9)
C(4)-H(4A)	0.9800	F(54)-C(58)	1.241(7)
C(4)-H(4B)	0.9800	F(55)-C(58)	1.285(9)
C(4)-H(4C)	0.9800	F(56)-C(58)	1.487(7)
C(5)-C(6)	1.5167(15)	F(54A)-C(58)	1.279(7)
C(5)-H(5A)	0.9900	F(55A)-C(58)	1.378(7)
C(5)-H(5AB)	0.9900	F(56A)-C(58)	1.305(9)
C(6)-H(6A)	0.9900	F(54B)-C(58)	1.358(3)
C(6)-H(6AB)	0.9900	F(55B)-C(58)	1.330(3)
C(7)-H(7A)	0.9800	F(56B)-C(58)	1.323(3)
C(7)-H(7B)	0.9800	C(21)-C(26)	1.4033(12)
C(7)-H(7C)	0.9800	C(21)-C(22)	1.4034(12)
C(8)-C(9)	1.5240(15)	C(21)-B(1)	1.6395(13)
C(8)-H(8A)	0.9900	C(22)-C(23)	1.3928(12)
C(8)-H(8AB)	0.9900	C(22)-H(22)	0.9500
C(9)-C(10)	1.5178(15)	C(23)-C(24)	1.3861(13)
C(9)-H(9A)	0.9900	C(23)-C(27)	1.4944(13)
C(9)-H(9AB)	0.9900	C(24)-C(25)	1.3932(13)
C(10)-H(10A)	0.9900	C(24)-H(24)	0.9500
C(10)-H(10B)	0.9900	C(25)-C(26)	1.3915(13)
C(11)-H(11A)	0.9800	C(25)-C(28)	1.4963(13)
C(11)-H(11B)	0.9800	C(26)-H(26)	0.9500
C(11)-H(11C)	0.9800	C(31)-C(36)	1.4034(12)
C(12)-C(13)	1.5179(14)	C(31)-C(32)	1.4049(13)
C(12)-H(12A)	0.9900	C(31)-B(1)	1.6456(13)
C(12)-H(12B)	0.9900	C(32)-C(33)	1.3938(12)
C(13)-H(13A)	0.9900	C(32)-H(32)	0.9500
C(13)-H(13B)	0.9900	C(33)-C(34)	1.3903(13)
C(14)-H(14A)	0.9800	C(33)-C(37)	1.4963(14)
C(14)-H(14B)	0.9800	C(34)-C(35)	1.3851(13)
C(14)-H(14C)	0.9800	C(34)-H(34)	0.9500
F(21)-C(27)	1.348(6)	C(35)-C(36)	1.3955(13)
F(22)-C(27)	1.357(6)	C(35)-C(38)	1.4964(14)
F(23)-C(27)	1.353(6)	C(36)-H(36)	0.9500
F(21A)-C(27)	1.333(10)	C(41)-C(46)	1.4053(12)
F(22A)-C(27)	1.343(11)	C(41)-C(42)	1.4064(13)
F(23A)-C(27)	1.350(10)	C(41)-B(1)	1.6540(13)
F(21B)-C(27)	1.351(12)	C(42)-C(43)	1.3951(13)
F(22B)-C(27)	1.336(12)	C(42)-H(42)	0.9500
F(23B)-C(27)	1.345(12)	C(43)-C(44)	1.3878(13)
F(24)-C(28)	1.337(7)	C(43)-C(47)	1.5010(14)
F(25)-C(28)	1.345(6)	C(44)-C(45)	1.3898(13)
F(26)-C(28)	1.351(6)	C(44)-H(44)	0.9500
F(24A)-C(28)	1.326(12)	C(45)-C(46)	1.3952(13)
F(25A)-C(28)	1.368(12)	C(45)-C(48)	1.4978(13)
F(26A)-C(28)	1.337(12)	C(46)-H(46)	0.9500
F(24B)-C(28)	1.368(8)	C(51)-C(52)	1.4008(12)
F(25B)-C(28)	1.340(9)	C(51)-C(56)	1.4072(13)
F(26B)-C(28)	1.311(9)	C(51)-B(1)	1.6431(13)
F(31)-C(37)	1.364(9)	C(52)-C(53)	1.3964(13)
F(32)-C(37)	1.342(10)	C(52)-H(52)	0.9500
F(33)-C(37)	1.351(9)	C(53)-C(54)	1.3857(13)
F(31A)-C(37)	1.329(10)	C(53)-C(57)	1.4980(13)
F(32A)-C(37)	1.350(9)	C(54)-C(55)	1.3926(13)
F(33A)-C(37)	1.352(10)	C(54)-H(54)	0.9500
F(31B)-C(37)	1.345(9)	C(55)-C(56)	1.3921(13)
F(32B)-C(37)	1.369(8)	C(55)-C(58)	1.4964(14)
F(33B)-C(37)	1.315(8)	C(56)-H(56)	0.9500
F(34)-C(38)	1.355(9)		
F(35)-C(38)	1.359(10)	N(4)-Co(1)-N(2)	165.73(3)
F(36)-C(38)	1.332(10)	N(4)-Co(1)-O(2)	99.77(3)
F(34A)-C(38)	1.336(10)	N(2)-Co(1)-O(2)	92.59(3)
F(35A)-C(38)	1.332(10)	N(4)-Co(1)-N(3)	90.70(3)
F(36A)-C(38)	1.367(10)	N(2)-Co(1)-N(3)	83.14(3)
F(34B)-C(38)	1.329(10)	O(2)-Co(1)-N(3)	85.52(3)

N(4)-Co(1)-N(1)	83.32(3)
N(2)-Co(1)-N(1)	91.31(3)
O(2)-Co(1)-N(1)	142.10(3)
N(3)-Co(1)-N(1)	132.36(3)
N(4)-Co(1)-O(1)	93.07(3)
N(2)-Co(1)-O(1)	99.55(3)
O(2)-Co(1)-O(1)	57.98(3)
N(3)-Co(1)-O(1)	143.42(3)
N(1)-Co(1)-O(1)	84.20(3)
N(5)-O(1)-Co(1)	92.91(6)
N(5)-O(2)-Co(1)	94.07(6)
C(13)-N(1)-C(1)	108.64(7)
C(13)-N(1)-C(14)	107.67(8)
C(1)-N(1)-C(14)	108.20(8)
C(13)-N(1)-Co(1)	104.81(6)
C(1)-N(1)-Co(1)	113.12(6)
C(14)-N(1)-Co(1)	114.12(6)
C(4)-N(2)-C(5)	108.47(8)
C(4)-N(2)-C(3)	108.30(8)
C(5)-N(2)-C(3)	107.75(8)
C(4)-N(2)-Co(1)	111.33(6)
C(5)-N(2)-Co(1)	103.31(6)
C(3)-N(2)-Co(1)	117.25(6)
C(6)-N(3)-C(7)	107.32(8)
C(6)-N(3)-C(8)	108.29(8)
C(7)-N(3)-C(8)	108.42(8)
C(6)-N(3)-Co(1)	105.67(6)
C(7)-N(3)-Co(1)	113.32(7)
C(8)-N(3)-Co(1)	113.52(6)
C(11)-N(4)-C(12)	108.45(8)
C(11)-N(4)-C(10)	108.14(8)
C(12)-N(4)-C(10)	107.60(8)
C(11)-N(4)-Co(1)	110.72(6)
C(12)-N(4)-Co(1)	103.18(6)
C(10)-N(4)-Co(1)	118.28(6)
O(3)-N(5)-O(2)	122.43(10)
O(3)-N(5)-O(1)	122.53(10)
O(2)-N(5)-O(1)	115.03(8)
N(1)-C(1)-C(2)	114.04(8)
N(1)-C(1)-H(1A)	108.7
C(2)-C(1)-H(1A)	108.7
N(1)-C(1)-H(1AB)	108.7
C(2)-C(1)-H(1AB)	108.7
H(1A)-C(1)-H(1AB)	107.6
C(3)-C(2)-C(1)	113.83(8)
C(3)-C(2)-H(2A)	108.8
C(1)-C(2)-H(2A)	108.8
C(3)-C(2)-H(2AB)	108.8
C(1)-C(2)-H(2AB)	108.8
H(2A)-C(2)-H(2AB)	107.7
N(2)-C(3)-C(2)	115.93(8)
N(2)-C(3)-H(3A)	108.3
C(2)-C(3)-H(3A)	108.3
N(2)-C(3)-H(3AB)	108.3
C(2)-C(3)-H(3AB)	108.3
H(3A)-C(3)-H(3AB)	107.4
N(2)-C(4)-H(4A)	109.5
N(2)-C(4)-H(4B)	109.5
H(4A)-C(4)-H(4B)	109.5
N(2)-C(4)-H(4C)	109.5
H(4A)-C(4)-H(4C)	109.5
H(4B)-C(4)-H(4C)	109.5
N(2)-C(5)-C(6)	110.54(8)
N(2)-C(5)-H(5A)	109.5
C(6)-C(5)-H(5A)	109.5
N(2)-C(5)-H(5AB)	109.5
C(6)-C(5)-H(5AB)	109.5
H(5A)-C(5)-H(5AB)	108.1
N(3)-C(6)-C(5)	110.35(8)
N(3)-C(6)-H(6A)	109.6
C(5)-C(6)-H(6A)	109.6
N(3)-C(6)-H(6AB)	109.6
C(5)-C(6)-H(6AB)	109.6
H(6A)-C(6)-H(6AB)	108.1
N(3)-C(7)-H(7A)	109.5
N(3)-C(7)-H(7B)	109.5
H(7A)-C(7)-H(7B)	109.5
N(3)-C(7)-H(7C)	109.5
H(7A)-C(7)-H(7C)	109.5
H(7B)-C(7)-H(7C)	109.5
N(3)-C(8)-C(9)	114.09(8)
N(3)-C(8)-H(8A)	108.7
C(9)-C(8)-H(8A)	108.7
N(3)-C(8)-H(8AB)	108.7
C(9)-C(8)-H(8AB)	108.7
H(8A)-C(8)-H(8AB)	107.6
C(10)-C(9)-C(8)	113.68(8)
C(10)-C(9)-H(9A)	108.8
C(8)-C(9)-H(9A)	108.8
C(10)-C(9)-H(9AB)	108.8
C(8)-C(9)-H(9AB)	108.8
H(9A)-C(9)-H(9AB)	107.7
N(4)-C(10)-C(9)	116.20(8)
N(4)-C(10)-H(10A)	108.2
C(9)-C(10)-H(10A)	108.2
N(4)-C(10)-H(10B)	108.2
C(9)-C(10)-H(10B)	108.2
H(10A)-C(10)-H(10B)	107.4
N(4)-C(11)-H(11A)	109.5
N(4)-C(11)-H(11B)	109.5
H(11A)-C(11)-H(11B)	109.5
N(4)-C(11)-H(11C)	109.5
H(11A)-C(11)-H(11C)	109.5
H(11B)-C(11)-H(11C)	109.5
N(4)-C(12)-C(13)	110.14(8)
N(4)-C(12)-H(12A)	109.6
C(13)-C(12)-H(12A)	109.6
N(4)-C(12)-H(12B)	109.6
C(13)-C(12)-H(12B)	109.6
H(12A)-C(12)-H(12B)	108.1
N(1)-C(13)-C(12)	110.23(8)
N(1)-C(13)-H(13A)	109.6
C(12)-C(13)-H(13A)	109.6
N(1)-C(13)-H(13B)	109.6
C(12)-C(13)-H(13B)	109.6
H(13A)-C(13)-H(13B)	108.1
N(1)-C(14)-H(14A)	109.5
N(1)-C(14)-H(14B)	109.5
H(14A)-C(14)-H(14B)	109.5
N(1)-C(14)-H(14C)	109.5
H(14A)-C(14)-H(14C)	109.5
H(14B)-C(14)-H(14C)	109.5
C(26)-C(21)-C(22)	115.60(8)
C(26)-C(21)-B(1)	125.04(8)
C(22)-C(21)-B(1)	119.27(8)
C(23)-C(22)-C(21)	122.58(8)
C(23)-C(22)-H(22)	118.7
C(21)-C(22)-H(22)	118.7
C(24)-C(23)-C(22)	120.80(8)
C(24)-C(23)-C(27)	120.78(8)
C(22)-C(23)-C(27)	118.41(8)
C(23)-C(24)-C(25)	117.57(8)
C(23)-C(24)-H(24)	121.2
C(25)-C(24)-H(24)	121.2
C(26)-C(25)-C(24)	121.51(8)
C(26)-C(25)-C(28)	120.91(8)
C(24)-C(25)-C(28)	117.58(8)
C(25)-C(26)-C(21)	121.80(8)
C(25)-C(26)-H(26)	119.1
C(21)-C(26)-H(26)	119.1
F(21A)-C(27)-F(22A)	107.7(12)
F(22B)-C(27)-F(23B)	107.2(15)
F(21A)-C(27)-F(23A)	106.9(9)
F(22A)-C(27)-F(23A)	106.0(12)
F(22B)-C(27)-F(21B)	106.0(16)
F(23B)-C(27)-F(21B)	105.4(15)
F(21)-C(27)-F(23)	106.8(5)
F(21)-C(27)-F(22)	106.2(7)
F(23)-C(27)-F(22)	105.4(7)
F(21A)-C(27)-C(23)	114.6(11)
F(22B)-C(27)-C(23)	114.4(13)
F(22A)-C(27)-C(23)	110.5(11)
F(23B)-C(27)-C(23)	111.9(12)
F(21)-C(27)-C(23)	112.4(6)
F(23A)-C(27)-C(23)	110.7(9)
F(21B)-C(27)-C(23)	111.4(15)
F(23)-C(27)-C(23)	113.5(5)
F(22)-C(27)-C(23)	112.0(6)
F(24A)-C(28)-F(26A)	107.8(15)
F(26B)-C(28)-F(25B)	108.8(8)
F(24)-C(28)-F(25)	106.7(6)
F(24)-C(28)-F(26)	105.7(6)
F(25)-C(28)-F(26)	105.8(6)
F(26B)-C(28)-F(24B)	106.5(8)
F(25B)-C(28)-F(24B)	104.7(7)
F(24A)-C(28)-F(25A)	104.8(13)
F(26A)-C(28)-F(25A)	103.2(15)
F(26B)-C(28)-C(25)	113.0(7)
F(24A)-C(28)-C(25)	116.3(13)
F(24)-C(28)-C(25)	113.1(6)
F(26A)-C(28)-C(25)	114.8(15)
F(25B)-C(28)-C(25)	113.3(6)
F(25)-C(28)-C(25)	111.0(4)
F(26)-C(28)-C(25)	113.9(5)
F(24B)-C(28)-C(25)	109.8(7)
F(25A)-C(28)-C(25)	108.8(14)
C(36)-C(31)-C(32)	115.64(8)
C(36)-C(31)-B(1)	122.99(8)
C(32)-C(31)-B(1)	121.37(8)
C(33)-C(32)-C(31)	122.42(8)
C(33)-C(32)-H(32)	118.8
C(31)-C(32)-H(32)	118.8
C(34)-C(33)-C(32)	120.75(8)
C(34)-C(33)-C(37)	118.60(8)
C(32)-C(33)-C(37)	120.65(9)
C(35)-C(34)-C(33)	117.93(8)
C(35)-C(34)-H(34)	121.0
C(33)-C(34)-H(34)	121.0
C(34)-C(35)-C(36)	121.27(9)
C(34)-C(35)-C(38)	118.87(8)

C(36)-C(35)-C(38)	119.73(9)	F(45B)-C(48)-F(44B)	104.4(10)
C(35)-C(36)-C(31)	121.99(8)	F(44)-C(48)-F(45)	107.2(3)
C(35)-C(36)-H(36)	119.0	F(46)-C(48)-F(45)	105.1(3)
C(31)-C(36)-H(36)	119.0	F(46A)-C(48)-F(45A)	107.5(6)
F(33B)-C(37)-F(31B)	109.2(9)	F(46A)-C(48)-F(44A)	105.2(8)
F(31A)-C(37)-F(32A)	106.7(11)	F(45A)-C(48)-F(44A)	103.9(7)
F(32)-C(37)-F(33)	107.4(13)	F(45B)-C(48)-F(46B)	104.6(8)
F(31A)-C(37)-F(33A)	105.4(13)	F(44B)-C(48)-F(46B)	103.0(9)
F(32A)-C(37)-F(33A)	104.3(13)	F(45B)-C(48)-C(45)	117.1(6)
F(32)-C(37)-F(31)	105.3(12)	F(44)-C(48)-C(45)	113.7(3)
F(33)-C(37)-F(31)	105.5(12)	F(46A)-C(48)-C(45)	116.3(7)
F(33B)-C(37)-F(32B)	107.0(10)	F(46)-C(48)-C(45)	112.6(3)
F(31B)-C(37)-F(32B)	104.7(10)	F(44B)-C(48)-C(45)	115.1(9)
F(33B)-C(37)-C(33)	114.7(7)	F(45)-C(48)-C(45)	111.77(16)
F(31A)-C(37)-C(33)	115.2(9)	F(45A)-C(48)-C(45)	111.9(4)
F(32)-C(37)-C(33)	114.4(8)	F(44A)-C(48)-C(45)	111.2(8)
F(31B)-C(37)-C(33)	110.9(7)	F(46B)-C(48)-C(45)	111.2(5)
F(32A)-C(37)-C(33)	113.2(8)	C(52)-C(51)-C(56)	115.62(8)
F(33)-C(37)-C(33)	112.0(8)	C(52)-C(51)-B(1)	123.87(8)
F(33A)-C(37)-C(33)	111.1(11)	C(56)-C(51)-B(1)	120.37(8)
F(31)-C(37)-C(33)	111.7(7)	C(53)-C(52)-C(51)	122.02(8)
F(32B)-C(37)-C(33)	109.7(6)	C(53)-C(52)-H(52)	119.0
F(35A)-C(38)-F(34A)	108.3(13)	C(51)-C(52)-H(52)	119.0
F(34B)-C(38)-F(35B)	107.3(12)	C(54)-C(53)-C(52)	121.46(8)
F(34B)-C(38)-F(36B)	106.9(12)	C(54)-C(53)-C(57)	119.90(8)
F(35B)-C(38)-F(36B)	105.9(13)	C(52)-C(53)-C(57)	118.64(8)
F(36)-C(38)-F(34)	106.4(13)	C(53)-C(54)-C(55)	117.54(8)
F(36)-C(38)-F(35)	105.9(12)	C(53)-C(54)-H(54)	121.2
F(34)-C(38)-F(35)	105.4(13)	C(55)-C(54)-H(54)	121.2
F(35A)-C(38)-F(36A)	105.5(12)	C(56)-C(55)-C(54)	121.03(9)
F(34A)-C(38)-F(36A)	105.3(13)	C(56)-C(55)-C(58)	119.86(9)
F(34B)-C(38)-C(35)	114.2(10)	C(54)-C(55)-C(58)	119.07(9)
F(35A)-C(38)-C(35)	112.9(11)	C(55)-C(56)-C(51)	122.33(8)
F(36)-C(38)-C(35)	114.6(9)	C(55)-C(56)-H(56)	118.8
F(34A)-C(38)-C(35)	112.5(9)	C(51)-C(56)-H(56)	118.8
F(35B)-C(38)-C(35)	112.4(9)	F(51B)-C(57)-F(52B)	109.5(10)
F(36B)-C(38)-C(35)	109.6(10)	F(52)-C(57)-F(53)	106.5(5)
F(34)-C(38)-C(35)	112.2(7)	F(52)-C(57)-F(51)	104.8(5)
F(35)-C(38)-C(35)	111.7(8)	F(53)-C(57)-F(51)	106.6(4)
F(36A)-C(38)-C(35)	111.8(11)	F(51B)-C(57)-F(53B)	105.2(8)
C(46)-C(41)-C(42)	115.34(8)	F(52B)-C(57)-F(53B)	105.5(9)
C(46)-C(41)-B(1)	123.91(8)	F(53A)-C(57)-F(52A)	111.2(6)
C(42)-C(41)-B(1)	120.67(8)	F(53A)-C(57)-F(51A)	106.9(7)
C(43)-C(42)-C(41)	122.29(8)	F(52A)-C(57)-F(51A)	95.8(6)
C(43)-C(42)-H(42)	118.9	F(53A)-C(57)-C(53)	120.2(4)
C(41)-C(42)-H(42)	118.9	F(51B)-C(57)-C(53)	112.4(7)
C(44)-C(43)-C(42)	121.25(9)	F(52B)-C(57)-C(53)	113.6(8)
C(44)-C(43)-C(47)	120.13(9)	F(52)-C(57)-C(53)	113.4(5)
C(42)-C(43)-C(47)	118.60(9)	F(53)-C(57)-C(53)	112.0(3)
C(43)-C(44)-C(45)	117.59(8)	F(51)-C(57)-C(53)	112.9(4)
C(43)-C(44)-H(44)	121.2	F(53B)-C(57)-C(53)	110.1(6)
C(45)-C(44)-H(44)	121.2	F(52A)-C(57)-C(53)	110.2(3)
C(44)-C(45)-C(46)	121.15(8)	F(51A)-C(57)-C(53)	109.7(3)
C(44)-C(45)-C(48)	119.36(8)	F(54)-C(58)-F(55)	119.0(8)
C(46)-C(45)-C(48)	119.48(8)	F(54A)-C(58)-F(56A)	113.4(7)
C(45)-C(46)-C(41)	122.36(8)	F(56B)-C(58)-F(55B)	106.7(2)
C(45)-C(46)-H(46)	118.8	F(56B)-C(58)-F(54B)	106.3(2)
C(41)-C(46)-H(46)	118.8	F(55B)-C(58)-F(54B)	103.5(2)
F(42B)-C(47)-F(43B)	108.6(11)	F(54A)-C(58)-F(55A)	102.7(5)
F(41A)-C(47)-F(43A)	107.5(17)	F(56A)-C(58)-F(55A)	105.2(6)
F(42B)-C(47)-F(41B)	107.0(12)	F(54)-C(58)-F(56)	100.4(7)
F(43B)-C(47)-F(41B)	106.3(13)	F(55)-C(58)-F(56)	100.0(6)
F(41A)-C(47)-F(42A)	106.3(17)	F(54)-C(58)-C(55)	114.9(4)
F(43A)-C(47)-F(42A)	106.3(17)	F(54A)-C(58)-C(55)	114.3(5)
F(43)-C(47)-F(41)	107.1(8)	F(55)-C(58)-C(55)	115.1(6)
F(43)-C(47)-F(42)	106.9(7)	F(56A)-C(58)-C(55)	112.3(6)
F(41)-C(47)-F(42)	106.1(8)	F(56B)-C(58)-C(55)	113.81(19)
F(42B)-C(47)-C(43)	112.8(9)	F(55B)-C(58)-C(55)	113.75(19)
F(41A)-C(47)-C(43)	111.8(15)	F(54B)-C(58)-C(55)	111.89(18)
F(43B)-C(47)-C(43)	110.9(8)	F(55A)-C(58)-C(55)	107.9(4)
F(43A)-C(47)-C(43)	113.0(14)	F(56)-C(58)-C(55)	103.2(3)
F(43)-C(47)-C(43)	113.5(4)	C(21)-B(1)-C(51)	108.40(7)
F(41B)-C(47)-C(43)	110.9(10)	C(21)-B(1)-C(31)	111.57(7)
F(42A)-C(47)-C(43)	111.5(15)	C(51)-B(1)-C(31)	108.82(7)
F(41)-C(47)-C(43)	111.2(6)	C(21)-B(1)-C(41)	108.42(7)
F(42)-C(47)-C(43)	111.7(5)	C(51)-B(1)-C(41)	109.58(7)
F(44)-C(48)-F(46)	105.8(3)	C(31)-B(1)-C(41)	110.02(7)

Table S99. Anisotropic displacement parameters (Å^{2}x 10^3) for **[(TMC)Co^{II}(NO$_3$)]B$_{ArF}$**.
The anisotropic displacement factor exponent takes the form: -2p^2[h^2 a*^{2}U^{11} + ... + 2 h k a* b* U^{12}]

	U^{11}	U^{22}	U^{33}	U^{23}	U^{13}	U^{12}
Co(1)	10(1)	11(1)	9(1)	1(1)	-3(1)	-6(1)
O(1)	20(1)	29(1)	15(1)	-1(1)	-6(1)	-11(1)
O(2)	20(1)	31(1)	16(1)	3(1)	-5(1)	-12(1)
O(3)	39(1)	43(1)	10(1)	0(1)	-6(1)	-25(1)
N(1)	12(1)	14(1)	11(1)	0(1)	-2(1)	-6(1)
N(2)	14(1)	14(1)	13(1)	3(1)	-4(1)	-8(1)
N(3)	12(1)	14(1)	15(1)	2(1)	-4(1)	-7(1)
N(4)	14(1)	12(1)	13(1)	0(1)	-3(1)	-7(1)
N(5)	24(1)	23(1)	12(1)	2(1)	-6(1)	-17(1)
C(1)	18(1)	21(1)	12(1)	1(1)	-6(1)	-11(1)
C(2)	20(1)	22(1)	15(1)	0(1)	-6(1)	-14(1)
C(3)	21(1)	17(1)	16(1)	-2(1)	-4(1)	-12(1)
C(4)	22(1)	21(1)	16(1)	6(1)	-5(1)	-15(1)
C(5)	17(1)	12(1)	25(1)	5(1)	-6(1)	-6(1)
C(6)	15(1)	12(1)	23(1)	0(1)	-2(1)	-4(1)
C(7)	16(1)	25(1)	25(1)	6(1)	-10(1)	-10(1)
C(8)	16(1)	18(1)	16(1)	1(1)	1(1)	-9(1)
C(9)	16(1)	20(1)	20(1)	3(1)	-1(1)	-12(1)
C(10)	19(1)	16(1)	15(1)	4(1)	-3(1)	-10(1)
C(11)	25(1)	20(1)	19(1)	-2(1)	-6(1)	-14(1)
C(12)	16(1)	10(1)	20(1)	0(1)	-5(1)	-3(1)
C(13)	16(1)	14(1)	17(1)	4(1)	-7(1)	-4(1)
C(14)	11(1)	25(1)	17(1)	-2(1)	-1(1)	-6(1)
F(21)	16(4)	25(4)	13(2)	-7(2)	4(2)	-8(3)
F(22)	21(2)	35(5)	18(3)	-5(3)	-5(2)	-18(2)
F(23)	23(4)	15(1)	21(2)	-8(1)	2(3)	-5(1)
F(21A)	22(4)	33(6)	20(3)	-15(3)	11(3)	-15(4)
F(22A)	25(3)	36(5)	17(3)	-5(3)	-3(2)	-19(2)
F(23A)	27(5)	17(2)	29(4)	-12(2)	5(3)	-8(2)
F(21B)	16(7)	30(7)	10(4)	-4(3)	3(3)	-9(6)
F(22B)	13(3)	29(7)	20(7)	-11(5)	1(4)	-10(3)
F(23B)	28(7)	18(3)	24(6)	-3(3)	-6(6)	-9(3)
F(24)	40(5)	30(4)	16(2)	1(2)	4(2)	-27(3)
F(25)	6(1)	22(2)	78(8)	-13(3)	-1(2)	-6(1)
F(26)	20(2)	16(2)	18(2)	0(2)	-7(1)	-11(2)
F(24A)	36(10)	33(9)	23(5)	3(5)	1(5)	-29(7)
F(25A)	10(5)	19(6)	46(10)	-2(6)	-5(5)	-5(4)
F(26A)	15(6)	16(6)	23(7)	3(5)	-8(5)	-8(5)
F(24B)	35(4)	20(2)	22(3)	5(2)	-1(2)	-18(2)
F(25B)	15(2)	29(2)	76(6)	-7(3)	4(2)	-12(1)
F(26B)	41(4)	39(4)	25(2)	5(2)	-12(2)	-33(4)
F(31)	39(5)	17(5)	27(6)	14(3)	-23(5)	-12(4)
F(32)	41(4)	18(3)	17(6)	-4(3)	-7(3)	-20(3)
F(33)	36(6)	18(5)	20(4)	-12(3)	-7(3)	-10(4)
F(31A)	45(4)	14(3)	16(2)	1(3)	-14(3)	-12(3)
F(32A)	38(3)	29(3)	27(6)	-1(3)	-4(3)	-26(2)
F(33A)	39(5)	14(3)	13(3)	-3(2)	-5(3)	-7(3)
F(31B)	48(4)	11(3)	29(4)	-4(3)	-20(3)	1(3)
F(32B)	63(3)	30(3)	26(4)	4(3)	-7(2)	-36(3)
F(33B)	60(6)	15(2)	19(2)	1(2)	-18(3)	-15(3)
F(34)	21(6)	14(4)	29(6)	5(3)	-12(4)	-1(4)
F(35)	56(9)	21(5)	17(5)	6(4)	-27(6)	-17(6)
F(36)	26(3)	20(6)	15(3)	6(4)	-3(2)	-1(3)
F(34A)	26(4)	23(4)	16(4)	6(3)	-12(3)	-7(3)
F(35A)	56(6)	32(4)	17(3)	2(3)	-21(3)	-16(4)
F(36A)	30(4)	37(7)	16(3)	9(3)	1(2)	-17(4)
F(34B)	29(4)	20(2)	18(3)	7(2)	-12(3)	-9(2)
F(35B)	62(7)	29(4)	35(5)	3(3)	-37(5)	-16(4)
F(36B)	36(3)	31(7)	15(3)	10(3)	2(2)	-8(3)
F(41)	23(4)	18(3)	25(4)	-8(3)	-7(3)	-1(3)
F(42)	16(3)	33(2)	14(1)	-3(2)	1(1)	-13(2)
F(43)	9(2)	45(5)	18(2)	-7(3)	-2(2)	-5(2)
F(41A)	16(5)	20(4)	30(8)	-10(4)	-3(5)	1(4)
F(42A)	16(3)	33(2)	14(1)	-3(2)	1(1)	-13(2)
F(43A)	9(4)	45(9)	26(5)	-2(5)	-6(3)	-8(4)
F(41B)	19(4)	19(3)	22(4)	-9(3)	0(3)	0(3)
F(42B)	19(4)	38(2)	25(4)	-7(2)	4(2)	-18(2)
F(43B)	8(2)	46(5)	40(5)	-11(4)	-6(3)	0(3)
F(44)	60(2)	36(2)	14(1)	1(1)	-7(2)	-41(2)
F(45)	38(2)	36(1)	14(1)	4(1)	-10(1)	13(1)
F(46)	28(1)	55(3)	10(1)	2(1)	0(1)	-32(2)
F(44A)	102(9)	18(2)	24(4)	7(2)	-29(5)	-34(4)
F(45A)	17(1)	64(4)	17(2)	18(2)	-11(1)	-14(2)
F(46A)	43(4)	38(4)	12(2)	-4(2)	-1(3)	-34(4)
F(44B)	27(6)	45(7)	19(5)	-7(6)	-2(6)	-29(6)
F(45B)	63(9)	15(4)	11(6)	3(4)	-18(6)	-19(5)
F(46B)	17(1)	64(4)	17(2)	18(2)	-11(1)	-14(2)
F(51)	24(1)	53(2)	15(1)	-6(1)	-4(1)	-28(1)
F(52)	24(1)	22(1)	64(4)	20(2)	-9(2)	-17(1)
F(53)	19(1)	36(2)	17(1)	-13(1)	9(1)	-19(1)
F(51A)	25(4)	44(5)	44(4)	28(4)	-22(3)	-27(4)
F(52A)	22(3)	18(3)	88(7)	-15(4)	8(4)	-15(2)
F(53A)	40(3)	90(6)	21(2)	-8(3)	3(2)	-51(4)
F(51B)	40(3)	90(6)	21(2)	-8(3)	3(2)	-51(4)
F(52B)	29(4)	49(5)	33(2)	28(3)	-13(2)	-28(4)
F(53B)	15(2)	26(2)	45(5)	-8(3)	13(2)	-9(2)
F(54)	39(4)	23(3)	88(6)	-18(4)	-26(5)	5(3)
F(55)	13(3)	43(5)	50(6)	21(5)	8(4)	-5(3)
F(56)	27(3)	63(5)	36(3)	24(3)	-19(2)	3(3)
F(54A)	20(2)	14(2)	60(5)	17(3)	-1(4)	2(2)

F(55A)	20(2)	43(4)	35(2)	-4(2)	10(2)	11(3)
F(56A)	30(4)	43(3)	67(6)	-6(4)	-35(4)	-5(2)
F(54B)	26(1)	36(2)	62(2)	-33(2)	-18(2)	13(1)
F(55B)	12(1)	23(1)	44(2)	1(1)	-11(1)	-5(1)
F(56B)	21(1)	43(2)	64(2)	41(2)	-6(2)	-2(2)
C(21)	9(1)	10(1)	10(1)	1(1)	-3(1)	-4(1)
C(22)	10(1)	12(1)	11(1)	0(1)	-2(1)	-5(1)
C(23)	10(1)	13(1)	11(1)	-2(1)	-2(1)	-4(1)
C(24)	10(1)	15(1)	13(1)	0(1)	0(1)	-6(1)
C(25)	10(1)	11(1)	14(1)	2(1)	-3(1)	-6(1)
C(26)	10(1)	10(1)	11(1)	1(1)	-4(1)	-4(1)
C(27)	15(1)	21(1)	14(1)	-5(1)	1(1)	-9(1)
C(28)	13(1)	16(1)	22(1)	-1(1)	-1(1)	-8(1)
C(31)	9(1)	12(1)	10(1)	0(1)	-2(1)	-4(1)
C(32)	14(1)	12(1)	9(1)	1(1)	-3(1)	-6(1)
C(33)	16(1)	11(1)	12(1)	-1(1)	-3(1)	-6(1)
C(34)	16(1)	15(1)	11(1)	-1(1)	-4(1)	-7(1)
C(35)	13(1)	15(1)	9(1)	1(1)	-4(1)	-6(1)
C(36)	12(1)	12(1)	11(1)	1(1)	-4(1)	-4(1)
C(37)	33(1)	15(1)	15(1)	0(1)	-8(1)	-11(1)
C(38)	25(1)	19(1)	12(1)	2(1)	-8(1)	-8(1)
C(41)	11(1)	9(1)	10(1)	0(1)	-3(1)	-5(1)
C(42)	11(1)	14(1)	11(1)	-1(1)	-2(1)	-5(1)
C(43)	10(1)	14(1)	13(1)	-2(1)	-1(1)	-4(1)
C(44)	12(1)	14(1)	14(1)	1(1)	-5(1)	-6(1)
C(45)	14(1)	12(1)	10(1)	2(1)	-4(1)	-7(1)
C(46)	11(1)	11(1)	10(1)	0(1)	-2(1)	-5(1)
C(47)	11(1)	24(1)	18(1)	-6(1)	-2(1)	-4(1)
C(48)	16(1)	14(1)	12(1)	3(1)	-6(1)	-8(1)
C(51)	10(1)	11(1)	9(1)	0(1)	-2(1)	-5(1)
C(52)	10(1)	11(1)	11(1)	-1(1)	-2(1)	-5(1)
C(53)	11(1)	13(1)	11(1)	-1(1)	0(1)	-7(1)
C(54)	14(1)	11(1)	13(1)	1(1)	-2(1)	-6(1)
C(55)	11(1)	11(1)	14(1)	1(1)	-3(1)	-3(1)
C(56)	10(1)	13(1)	13(1)	1(1)	-3(1)	-6(1)
C(57)	14(1)	16(1)	16(1)	1(1)	-1(1)	-9(1)
C(58)	14(1)	16(1)	35(1)	6(1)	-8(1)	-5(1)
B(1)	9(1)	10(1)	9(1)	0(1)	-3(1)	-4(1)

Table S100. Hydrogen coordinates (x 10^4) and isotropic displacement parameters ($Å^2$x 10^3) for **[(TMC)Co^{II}(NO$_3$)]B$_{ArF}$**.

	x	y	z	U(eq)
H(1A)	9193	151	7511	19
H(1AB)	7666	704	7735	19
H(2A)	8702	1953	7365	20
H(2AB)	9062	1514	6496	20
H(3A)	6495	2565	7370	20
H(3AB)	7188	3195	6770	20
H(4A)	7092	2301	5070	27
H(4B)	8312	1958	5431	27
H(4C)	7286	3204	5479	27
H(5A)	5044	3015	5842	22
H(5AB)	5180	3736	6452	22
H(6A)	4716	2619	7476	21
H(6AB)	3567	3145	7038	21
H(7A)	3889	2100	5858	31
H(7B)	2863	1988	6594	31
H(7C)	3922	923	6109	31
H(8A)	3320	1506	7791	21
H(8AB)	4749	994	7910	21
H(9A)	3804	-301	7939	22
H(9AB)	3933	-128	7027	22
H(10A)	6055	-965	7741	20
H(10B)	5636	-1721	7339	20
H(11A)	6765	-1314	5441	29
H(11B)	5314	-887	5922	29
H(11C)	6318	-2101	6043	29
H(12A)	8421	-1856	6082	20
H(12B)	7881	-2378	6853	20
H(13A)	7808	-982	7613	19
H(13B)	9236	-1669	7133	19
H(14A)	9709	-1040	5779	28
H(14B)	10335	-701	6355	28
H(14C)	9590	183	5785	28
H(22)	1037	6248	3111	13
H(24)	-1725	5338	4324	16
H(26)	337	3996	2202	12
H(32)	2692	2760	1577	14
H(34)	2287	2675	-611	16
H(36)	1727	5520	323	14
H(42)	4672	3693	1096	14
H(44)	6580	2852	2825	15
H(46)	2733	4609	3303	13
H(52)	3922	5873	1310	13
H(54)	1716	8950	737	15
H(56)	110	6862	1650	14

[(TMC)Co^{II}(I)]B$_{ArF}$

[(TMC)Co^{II}(I)]B$_{ArF}$ was solved in the orthorhombic space group *Pbca*. The asymmetric unit contains a full complex cation and a full anion. The complex was found to be fully disordered while the anion exhibits disorder at two CF_3 groups. The disorder was modelled using same distance restraints, similarity restraints on anisotropic displacement parameters and advanced rigid bond restraints. Some disordered atoms with similar positions were set to the same anisotropic displacement parameters. The disorder ratio refined to 0.793(4) and for the CF_3 groups to 0.75(1) and 0.58(4).

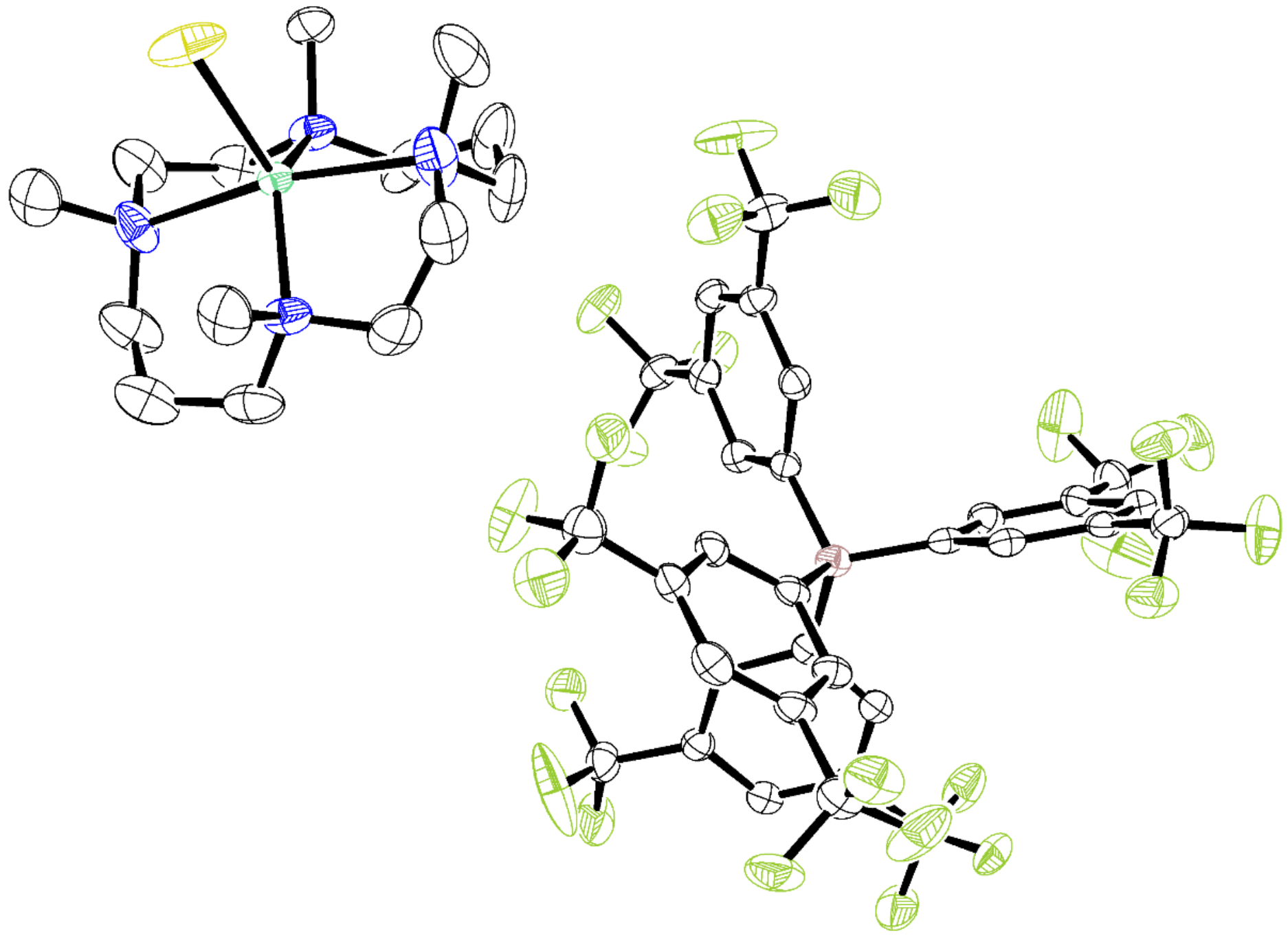

Figure S20. Molecular structure of **[(TMC)Co^{II}(I)]B$_{ArF}$**.

Table S101. Crystal data and structure refinement for **[(TMC)Co^{II}(I)]B$_{ArF}$**.

CCDC No	2062712	
Empirical formula	$C_{46}H_{44}BCoF_{24}IN_4$	
Formula weight	1305.49	
Temperature	100(2) K	
Wavelength	0.71073 Å	
Crystal system	Orthorhombic	
Space group	*Pbca*	
Unit cell dimensions	a = 18.2525(7) Å	α = 90°
	b = 19.8249(8) Å	β = 90°
	c = 27.9446(10) Å	γ = 90°
Volume	10111.9(7) Å^3	
Z	8	
Density (calculated)	1.715 Mg/m^3	
Absorption coefficient	1.077 mm^{-1}	
F(000)	5192	
Crystal size	0.160 x 0.139 x 0.108 mm^3	
Theta range for data collection	2.103 to 25.025°.	
Index ranges	-21 ≤ h ≤ 21, -23 ≤ k ≤ 23, -33 ≤ l ≤ 33	
Reflections collected	79350	
Independent reflections	8923 [R(int) = 0.0706]	
Completeness to theta = 25.025°	99.9 %	
Absorption correction	Semi-empirical from equivalents	
Refinement method	Full-matrix least-squares on F^2	
Data / restraints / parameters	8923 / 2209 / 891	
Goodness-of-fit on F^2	1.015	
Final R indices [I>2σ(I)]	R1 = 0.0566, wR2 = 0.1448	
R indices (all data)	R1 = 0.0890, wR2 = 0.1659	
Largest diff. peak and hole	0.602 and -1.364 eÅ^{-3}	

Table S102. Atomic coordinates (x 10^4) and equivalent isotropic displacement parameters (Å^{2}x 10^3) for **[(TMC)Co^{II}(I)]B$_{ArF}$**. U(eq) is defined as one third of the trace of the orthogonalized U^{ij} tensor.

	x	y	z	U(eq)
I(1)	2692(1)	3432(1)	10136(1)	59(1)
Co(1)	2904(2)	3809(2)	9273(1)	21(1)
N(1)	2133(3)	3459(3)	8762(2)	33(1)
N(2)	2342(4)	4816(4)	9308(3)	52(2)
N(3)	3861(3)	4396(4)	9253(3)	33(1)
N(4)	3510(3)	2869(3)	9044(3)	42(2)
C(1)	1901(5)	3963(4)	8401(3)	52(2)
C(2)	1559(5)	4587(4)	8597(4)	66(2)
C(3)	2081(5)	5061(4)	8842(3)	62(2)
C(4)	1758(5)	4877(4)	9670(4)	68(2)
C(5)	2966(4)	5257(4)	9465(3)	54(2)
C(6)	3640(4)	5100(4)	9193(3)	49(2)
C(7)	4270(7)	4356(8)	9714(4)	52(1)
C(8)	4383(4)	4210(5)	8864(3)	48(2)
C(9)	4657(4)	3489(5)	8873(3)	61(2)
C(10)	4094(4)	2959(5)	8713(3)	60(2)
C(11)	3804(4)	2433(4)	9464(3)	52(1)
C(12)	2915(4)	2454(4)	8835(3)	50(2)
C(13)	2457(4)	2884(4)	8503(3)	46(2)
C(14)	1487(6)	3237(4)	9036(4)	36(2)
I(1A)	2914(4)	3248(3)	10116(3)	53(1)
Co(1A)	2940(8)	3864(7)	9319(5)	21(1)
N(1A)	2388(11)	4796(9)	9463(8)	49(4)
N(2A)	4023(9)	4375(10)	9269(9)	33(1)
N(3A)	3532(10)	3125(10)	8922(7)	44(4)
N(4A)	1964(9)	3652(9)	8872(6)	33(1)
C(1A)	2681(12)	5418(11)	9240(11)	51(4)
C(2A)	3479(12)	5522(13)	9304(10)	49(4)
C(3A)	3962(14)	5040(11)	9055(10)	42(4)
C(4A)	4370(20)	4330(30)	9736(13)	52(1)
C(5A)	4472(16)	3953(12)	8930(12)	47(4)
C(6A)	4306(11)	3238(12)	9030(10)	47(4)
C(7A)	3443(18)	2414(12)	9100(10)	55(6)
C(8A)	3423(11)	3084(13)	8402(7)	44(4)
C(9A)	2637(12)	2978(13)	8256(9)	40(4)
C(10A)	2132(13)	3569(13)	8367(7)	40(4)
C(11A)	1490(20)	3061(18)	9037(17)	36(2)
C(12A)	1507(14)	4270(13)	8902(10)	60(4)
C(13A)	1599(12)	4695(13)	9342(10)	59(4)
C(14A)	2370(15)	4956(15)	9992(8)	52(1)
F(21)	458(2)	7980(2)	5753(2)	75(1)
F(22)	1123(2)	8689(3)	5392(1)	81(1)
F(23)	255(2)	9022(2)	5840(1)	54(1)
F(24)	1531(2)	10242(2)	7093(1)	56(1)
F(25)	2516(2)	9847(2)	7391(1)	43(1)
F(26)	1495(2)	9524(2)	7666(1)	46(1)
F(31)	2112(2)	4953(2)	5970(1)	57(1)
F(32)	993(2)	5246(2)	5881(1)	44(1)
F(33)	1279(2)	4522(2)	6410(1)	46(1)
F(34)	526(2)	6778(2)	7843(1)	63(1)
F(35)	1297(2)	6103(2)	8136(1)	54(1)
F(36)	347(2)	5718(2)	7790(1)	74(1)
F(41)	4795(2)	9020(2)	7198(2)	68(1)
F(42)	4880(2)	8908(2)	7958(1)	49(1)
F(43)	5609(2)	8369(2)	7503(1)	50(1)
F(44)	4647(8)	6465(10)	8514(6)	56(4)
F(45)	4071(13)	5787(5)	8078(5)	63(4)
F(46)	3492(8)	6430(7)	8555(6)	59(4)
F(44A)	4460(14)	6555(11)	8608(6)	56(5)
F(45A)	3400(6)	6219(15)	8384(11)	81(6)
F(46A)	4337(14)	5840(8)	8049(8)	67(5)
F(51)	5121(5)	5873(4)	6074(3)	94(3)
F(52)	4931(4)	5861(3)	5327(2)	60(2)
F(53)	4181(3)	5400(2)	5776(3)	68(2)
F(51A)	5345(7)	6030(7)	5634(9)	73(6)
F(52A)	4393(13)	5489(8)	5454(7)	81(6)
F(53A)	4707(10)	5568(8)	6158(5)	46(4)
F(54)	4447(2)	8122(2)	4830(1)	53(1)
F(55)	3278(2)	8188(2)	4847(1)	50(1)
F(56)	3918(2)	8808(1)	5311(1)	38(1)
C(21)	2273(2)	8006(2)	6667(2)	22(1)
C(22)	1765(2)	8031(2)	6291(2)	27(1)
C(23)	1309(3)	8579(2)	6220(2)	26(1)
C(24)	1339(2)	9131(2)	6521(2)	26(1)
C(25)	1821(2)	9114(2)	6900(2)	23(1)
C(26)	2279(2)	8560(2)	6971(2)	23(1)
C(27)	792(3)	8571(3)	5801(2)	36(1)
C(28)	1836(3)	9679(3)	7254(2)	30(1)
C(31)	2222(2)	6697(2)	6794(2)	22(1)
C(32)	2111(2)	6162(2)	6480(2)	25(1)
C(33)	1605(3)	5660(2)	6566(2)	26(1)
C(34)	1179(3)	5657(2)	6975(2)	27(1)
C(35)	1277(3)	6181(3)	7297(2)	27(1)
C(36)	1778(2)	6690(2)	7207(2)	25(1)
C(37)	1508(3)	5102(3)	6211(2)	32(1)
C(38)	856(3)	6192(3)	7757(2)	39(1)
C(41)	3374(2)	7387(2)	7165(2)	22(1)
C(42)	3869(2)	7932(3)	7184(2)	25(1)
C(43)	4404(3)	7984(2)	7532(2)	28(1)
C(44)	4479(3)	7486(3)	7884(2)	30(1)

C(45)	4008(3)	6945(2)	7867(2)	28(1)
C(46)	3468(3)	6893(2)	7517(2)	26(1)
C(47)	4912(3)	8567(3)	7544(2)	36(1)
C(48)	4055(3)	6407(3)	8242(2)	39(1)
C(51)	3309(2)	7242(2)	6246(2)	23(1)
C(52)	3745(2)	6664(2)	6189(2)	24(1)
C(53)	4203(3)	6572(2)	5799(2)	26(1)
C(54)	4259(3)	7066(2)	5446(2)	26(1)
C(55)	3856(2)	7647(2)	5500(2)	25(1)
C(56)	3390(2)	7735(2)	5892(2)	23(1)
C(57)	4627(3)	5936(3)	5754(2)	35(1)
C(58)	3876(3)	8184(3)	5127(2)	32(1)
B(21)	2791(3)	7331(3)	6718(2)	24(1)

Table S103. Bond lengths [Å] and angles [°] for **[(TMC)Co^{II}(I)]B$_{ArF}$**.

I(1)-Co(1)	2.555(3)	C(8A)-H(8AC)	0.9900
Co(1)-N(3)	2.100(6)	C(9A)-C(10A)	1.52(2)
Co(1)-N(1)	2.122(6)	C(9A)-H(9AA)	0.9900
Co(1)-N(2)	2.247(7)	C(9A)-H(9AC)	0.9900
Co(1)-N(4)	2.260(7)	C(10A)-H(10C)	0.9900
N(1)-C(14)	1.473(11)	C(10A)-H(10D)	0.9900
N(1)-C(13)	1.474(10)	C(11A)-H(11D)	0.9800
N(1)-C(1)	1.482(9)	C(11A)-H(11E)	0.9800
N(2)-C(3)	1.469(11)	C(11A)-H(11F)	0.9800
N(2)-C(4)	1.475(12)	C(12A)-C(13A)	1.50(2)
N(2)-C(5)	1.501(10)	C(12A)-H(12C)	0.9900
N(3)-C(6)	1.462(11)	C(12A)-H(12D)	0.9900
N(3)-C(7)	1.491(9)	C(13A)-H(13C)	0.9900
N(3)-C(8)	1.492(10)	C(13A)-H(13D)	0.9900
N(4)-C(10)	1.422(10)	C(14A)-H(14D)	0.9800
N(4)-C(12)	1.482(9)	C(14A)-H(14E)	0.9800
N(4)-C(11)	1.554(10)	C(14A)-H(14F)	0.9800
C(1)-C(2)	1.490(13)	F(21)-C(27)	1.328(7)
C(1)-H(1A)	0.9900	F(22)-C(27)	1.316(6)
C(1)-H(1AB)	0.9900	F(23)-C(27)	1.330(6)
C(2)-C(3)	1.502(11)	F(24)-C(28)	1.328(6)
C(2)-H(2A)	0.9900	F(25)-C(28)	1.342(6)
C(2)-H(2AB)	0.9900	F(26)-C(28)	1.344(6)
C(3)-H(3A)	0.9900	F(31)-C(37)	1.326(6)
C(3)-H(3AB)	0.9900	F(32)-C(37)	1.347(6)
C(4)-H(4A)	0.9800	F(33)-C(37)	1.343(6)
C(4)-H(4B)	0.9800	F(34)-C(38)	1.330(7)
C(4)-H(4C)	0.9800	F(35)-C(38)	1.342(6)
C(5)-C(6)	1.478(12)	F(36)-C(38)	1.324(6)
C(5)-H(5A)	0.9900	F(41)-C(47)	1.337(6)
C(5)-H(5AB)	0.9900	F(42)-C(47)	1.341(6)
C(6)-H(6A)	0.9900	F(43)-C(47)	1.336(6)
C(6)-H(6AB)	0.9900	F(44)-C(48)	1.327(9)
C(7)-H(7A)	0.9800	F(45)-C(48)	1.313(9)
C(7)-H(7B)	0.9800	F(46)-C(48)	1.352(9)
C(7)-H(7C)	0.9800	F(44A)-C(48)	1.296(11)
C(8)-C(9)	1.514(12)	F(45A)-C(48)	1.314(11)
C(8)-H(8A)	0.9900	F(46A)-C(48)	1.349(11)
C(8)-H(8AB)	0.9900	F(51)-C(57)	1.276(6)
C(9)-C(10)	1.537(12)	F(52)-C(57)	1.324(6)
C(9)-H(9A)	0.9900	F(53)-C(57)	1.340(6)
C(9)-H(9AB)	0.9900	F(51A)-C(57)	1.365(11)
C(10)-H(10A)	0.9900	F(52A)-C(57)	1.292(11)
C(10)-H(10B)	0.9900	F(53A)-C(57)	1.353(10)
C(11)-H(11A)	0.9800	F(54)-C(58)	1.338(6)
C(11)-H(11B)	0.9800	F(55)-C(58)	1.343(6)
C(11)-H(11C)	0.9800	F(56)-C(58)	1.343(6)
C(12)-C(13)	1.513(11)	C(21)-C(26)	1.387(7)
C(12)-H(12A)	0.9900	C(21)-C(22)	1.403(7)
C(12)-H(12B)	0.9900	C(21)-B(21)	1.644(7)
C(13)-H(13A)	0.9900	C(22)-C(23)	1.383(7)
C(13)-H(13B)	0.9900	C(22)-H(22)	0.9500
C(14)-H(14A)	0.9800	C(23)-C(24)	1.381(7)
C(14)-H(14B)	0.9800	C(23)-C(27)	1.503(7)
C(14)-H(14C)	0.9800	C(24)-C(25)	1.378(7)
I(1A)-Co(1A)	2.541(13)	C(24)-H(24)	0.9500
Co(1A)-N(3A)	2.133(16)	C(25)-C(26)	1.394(7)
Co(1A)-N(1A)	2.142(17)	C(25)-C(28)	1.495(7)
Co(1A)-N(4A)	2.217(16)	C(26)-H(26)	0.9500
Co(1A)-N(2A)	2.224(17)	C(31)-C(32)	1.393(7)
N(1A)-C(1A)	1.482(19)	C(31)-C(36)	1.410(7)
N(1A)-C(13A)	1.49(2)	C(31)-B(21)	1.644(7)
N(1A)-C(14A)	1.51(2)	C(32)-C(33)	1.379(7)
N(2A)-C(3A)	1.45(2)	C(32)-H(32)	0.9500
N(2A)-C(4A)	1.45(2)	C(33)-C(34)	1.383(7)
N(2A)-C(5A)	1.51(2)	C(33)-C(37)	1.497(7)
N(3A)-C(6A)	1.46(2)	C(34)-C(35)	1.386(7)
N(3A)-C(8A)	1.468(19)	C(34)-H(34)	0.9500
N(3A)-C(7A)	1.503(19)	C(35)-C(36)	1.385(7)
N(4A)-C(10A)	1.451(18)	C(35)-C(38)	1.498(7)
N(4A)-C(12A)	1.484(19)	C(36)-H(36)	0.9500
N(4A)-C(11A)	1.53(2)	C(41)-C(46)	1.396(7)
C(1A)-C(2A)	1.48(2)	C(41)-C(42)	1.410(7)
C(1A)-H(1AA)	0.9900	C(41)-B(21)	1.646(7)
C(1A)-H(1AC)	0.9900	C(42)-C(43)	1.382(7)
C(2A)-C(3A)	1.47(2)	C(42)-H(42)	0.9500
C(2A)-H(2AA)	0.9900	C(43)-C(44)	1.400(7)
C(2A)-H(2AC)	0.9900	C(43)-C(47)	1.482(7)
C(3A)-H(3AA)	0.9900	C(44)-C(45)	1.376(7)
C(3A)-H(3AC)	0.9900	C(44)-H(44)	0.9500
C(4A)-H(4AA)	0.9800	C(45)-C(46)	1.393(7)
C(4A)-H(4AB)	0.9800	C(45)-C(48)	1.497(7)
C(4A)-H(4AC)	0.9800	C(46)-H(46)	0.9500
C(5A)-C(6A)	1.48(2)	C(51)-C(56)	1.398(7)
C(5A)-H(5AA)	0.9900	C(51)-C(52)	1.405(7)
C(5A)-H(5AC)	0.9900	C(51)-B(21)	1.631(7)
C(6A)-H(6AA)	0.9900	C(52)-C(53)	1.385(7)
C(6A)-H(6AC)	0.9900	C(52)-H(52)	0.9500
C(7A)-H(7AA)	0.9800	C(53)-C(54)	1.394(7)
C(7A)-H(7AB)	0.9800	C(53)-C(57)	1.485(7)
C(7A)-H(7AC)	0.9800	C(54)-C(55)	1.376(7)
C(8A)-C(9A)	1.51(2)	C(54)-H(54)	0.9500
C(8A)-H(8AA)	0.9900	C(55)-C(56)	1.399(6)

C(55)-C(58)	1.489(7)
C(56)-H(56)	0.9500
N(3)-Co(1)-N(1)	135.6(3)
N(3)-Co(1)-N(2)	83.6(3)
N(1)-Co(1)-N(2)	91.0(3)
N(3)-Co(1)-N(4)	92.5(3)
N(1)-Co(1)-N(4)	82.2(3)
N(2)-Co(1)-N(4)	165.6(3)
N(3)-Co(1)-I(1)	108.2(2)
N(1)-Co(1)-I(1)	116.1(2)
N(2)-Co(1)-I(1)	98.6(2)
N(4)-Co(1)-I(1)	95.8(2)
C(14)-N(1)-C(13)	110.2(6)
C(14)-N(1)-C(1)	109.1(7)
C(13)-N(1)-C(1)	107.6(6)
C(14)-N(1)-Co(1)	106.1(6)
C(13)-N(1)-Co(1)	108.5(4)
C(1)-N(1)-Co(1)	115.3(5)
C(3)-N(2)-C(4)	110.3(7)
C(3)-N(2)-C(5)	108.2(7)
C(4)-N(2)-C(5)	107.4(7)
C(3)-N(2)-Co(1)	113.8(5)
C(4)-N(2)-Co(1)	115.6(6)
C(5)-N(2)-Co(1)	100.6(5)
C(6)-N(3)-C(7)	106.7(8)
C(6)-N(3)-C(8)	109.2(7)
C(7)-N(3)-C(8)	107.3(7)
C(6)-N(3)-Co(1)	107.6(4)
C(7)-N(3)-Co(1)	111.3(6)
C(8)-N(3)-Co(1)	114.4(5)
C(10)-N(4)-C(12)	111.3(6)
C(10)-N(4)-C(11)	107.6(7)
C(12)-N(4)-C(11)	104.0(6)
C(10)-N(4)-Co(1)	116.6(6)
C(12)-N(4)-Co(1)	102.2(4)
C(11)-N(4)-Co(1)	114.4(5)
N(1)-C(1)-C(2)	115.5(7)
N(1)-C(1)-H(1A)	108.4
C(2)-C(1)-H(1A)	108.4
N(1)-C(1)-H(1AB)	108.4
C(2)-C(1)-H(1AB)	108.4
H(1A)-C(1)-H(1AB)	107.5
C(1)-C(2)-C(3)	114.9(7)
C(1)-C(2)-H(2A)	108.5
C(3)-C(2)-H(2A)	108.5
C(1)-C(2)-H(2AB)	108.5
C(3)-C(2)-H(2AB)	108.5
H(2A)-C(2)-H(2AB)	107.5
N(2)-C(3)-C(2)	113.7(7)
N(2)-C(3)-H(3A)	108.8
C(2)-C(3)-H(3A)	108.8
N(2)-C(3)-H(3AB)	108.8
C(2)-C(3)-H(3AB)	108.8
H(3A)-C(3)-H(3AB)	107.7
N(2)-C(4)-H(4A)	109.5
N(2)-C(4)-H(4B)	109.5
H(4A)-C(4)-H(4B)	109.5
N(2)-C(4)-H(4C)	109.5
H(4A)-C(4)-H(4C)	109.5
H(4B)-C(4)-H(4C)	109.5
C(6)-C(5)-N(2)	111.0(7)
C(6)-C(5)-H(5A)	109.4
N(2)-C(5)-H(5A)	109.4
C(6)-C(5)-H(5AB)	109.4
N(2)-C(5)-H(5AB)	109.4
H(5A)-C(5)-H(5AB)	108.0
N(3)-C(6)-C(5)	111.9(7)
N(3)-C(6)-H(6A)	109.2
C(5)-C(6)-H(6A)	109.2
N(3)-C(6)-H(6AB)	109.2
C(5)-C(6)-H(6AB)	109.2
H(6A)-C(6)-H(6AB)	107.9
N(3)-C(7)-H(7A)	109.5
N(3)-C(7)-H(7B)	109.5
H(7A)-C(7)-H(7B)	109.5
N(3)-C(7)-H(7C)	109.5
H(7A)-C(7)-H(7C)	109.5
H(7B)-C(7)-H(7C)	109.5
N(3)-C(8)-C(9)	115.6(7)
N(3)-C(8)-H(8A)	108.4
C(9)-C(8)-H(8A)	108.4
N(3)-C(8)-H(8AB)	108.4
C(9)-C(8)-H(8AB)	108.4
H(8A)-C(8)-H(8AB)	107.4
C(8)-C(9)-C(10)	114.9(7)
C(8)-C(9)-H(9A)	108.5
C(10)-C(9)-H(9A)	108.5
C(8)-C(9)-H(9AB)	108.5
C(10)-C(9)-H(9AB)	108.5
H(9A)-C(9)-H(9AB)	107.5
N(4)-C(10)-C(9)	113.5(7)
N(4)-C(10)-H(10A)	108.9
C(9)-C(10)-H(10A)	108.9
N(4)-C(10)-H(10B)	108.9
C(9)-C(10)-H(10B)	108.9
H(10A)-C(10)-H(10B)	107.7
N(4)-C(11)-H(11A)	109.5
N(4)-C(11)-H(11B)	109.5
H(11A)-C(11)-H(11B)	109.5
N(4)-C(11)-H(11C)	109.5
H(11A)-C(11)-H(11C)	109.5
H(11B)-C(11)-H(11C)	109.5
N(4)-C(12)-C(13)	109.5(7)
N(4)-C(12)-H(12A)	109.8
C(13)-C(12)-H(12A)	109.8
N(4)-C(12)-H(12B)	109.8
C(13)-C(12)-H(12B)	109.8
H(12A)-C(12)-H(12B)	108.2
N(1)-C(13)-C(12)	110.9(6)
N(1)-C(13)-H(13A)	109.5
C(12)-C(13)-H(13A)	109.5
N(1)-C(13)-H(13B)	109.5
C(12)-C(13)-H(13B)	109.5
H(13A)-C(13)-H(13B)	108.0
N(1)-C(14)-H(14A)	109.5
N(1)-C(14)-H(14B)	109.5
H(14A)-C(14)-H(14B)	109.5
N(1)-C(14)-H(14C)	109.5
H(14A)-C(14)-H(14C)	109.5
H(14B)-C(14)-H(14C)	109.5
N(3A)-Co(1A)-N(1A)	158.3(10)
N(3A)-Co(1A)-N(4A)	89.0(8)
N(1A)-Co(1A)-N(4A)	83.8(8)
N(3A)-Co(1A)-N(2A)	80.2(8)
N(1A)-Co(1A)-N(2A)	92.1(9)
N(4A)-Co(1A)-N(2A)	139.9(10)
N(3A)-Co(1A)-I(1A)	97.8(7)
N(1A)-Co(1A)-I(1A)	103.9(8)
N(4A)-Co(1A)-I(1A)	112.9(8)
N(2A)-Co(1A)-I(1A)	106.8(8)
C(1A)-N(1A)-C(13A)	111.3(18)
C(1A)-N(1A)-C(14A)	104.2(18)
C(13A)-N(1A)-C(14A)	103.2(17)
C(1A)-N(1A)-Co(1A)	118.0(15)
C(13A)-N(1A)-Co(1A)	107.2(13)
C(14A)-N(1A)-Co(1A)	112.1(15)
C(3A)-N(2A)-C(4A)	117(2)
C(3A)-N(2A)-C(5A)	106.6(18)
C(4A)-N(2A)-C(5A)	107(2)
C(3A)-N(2A)-Co(1A)	111.8(14)
C(4A)-N(2A)-Co(1A)	108(2)
C(5A)-N(2A)-Co(1A)	105.7(14)
C(6A)-N(3A)-C(8A)	110.2(17)
C(6A)-N(3A)-C(7A)	100.3(18)
C(8A)-N(3A)-C(7A)	105.1(17)
C(6A)-N(3A)-Co(1A)	106.0(13)
C(8A)-N(3A)-Co(1A)	118.9(14)
C(7A)-N(3A)-Co(1A)	114.7(15)
C(10A)-N(4A)-C(12A)	105.6(16)
C(10A)-N(4A)-C(11A)	109(2)
C(12A)-N(4A)-C(11A)	107(2)
C(10A)-N(4A)-Co(1A)	113.5(13)
C(12A)-N(4A)-Co(1A)	105.2(12)
C(11A)-N(4A)-Co(1A)	115.5(19)
C(2A)-C(1A)-N(1A)	115(2)
C(2A)-C(1A)-H(1AA)	108.6
N(1A)-C(1A)-H(1AA)	108.6
C(2A)-C(1A)-H(1AC)	108.6
N(1A)-C(1A)-H(1AC)	108.6
H(1AA)-C(1A)-H(1AC)	107.6
C(3A)-C(2A)-C(1A)	116(2)
C(3A)-C(2A)-H(2AA)	108.2
C(1A)-C(2A)-H(2AA)	108.2
C(3A)-C(2A)-H(2AC)	108.2
C(1A)-C(2A)-H(2AC)	108.2
H(2AA)-C(2A)-H(2AC)	107.4
N(2A)-C(3A)-C(2A)	116(2)
N(2A)-C(3A)-H(3AA)	108.3
C(2A)-C(3A)-H(3AA)	108.3
N(2A)-C(3A)-H(3AC)	108.3
C(2A)-C(3A)-H(3AC)	108.3
H(3AA)-C(3A)-H(3AC)	107.4
N(2A)-C(4A)-H(4AA)	109.5
N(2A)-C(4A)-H(4AB)	109.5
H(4AA)-C(4A)-H(4AB)	109.5
N(2A)-C(4A)-H(4AC)	109.5
H(4AA)-C(4A)-H(4AC)	109.5
H(4AB)-C(4A)-H(4AC)	109.5
C(6A)-C(5A)-N(2A)	107.5(18)
C(6A)-C(5A)-H(5AA)	110.2
N(2A)-C(5A)-H(5AA)	110.2
C(6A)-C(5A)-H(5AC)	110.2
N(2A)-C(5A)-H(5AC)	110.2
H(5AA)-C(5A)-H(5AC)	108.5
N(3A)-C(6A)-C(5A)	107.9(18)
N(3A)-C(6A)-H(6AA)	110.1
C(5A)-C(6A)-H(6AA)	110.1
N(3A)-C(6A)-H(6AC)	110.1
C(5A)-C(6A)-H(6AC)	110.1

H(6AA)-C(6A)-H(6AC)	108.4
N(3A)-C(7A)-H(7AA)	109.5
N(3A)-C(7A)-H(7AB)	109.5
H(7AA)-C(7A)-H(7AB)	109.5
N(3A)-C(7A)-H(7AC)	109.5
H(7AA)-C(7A)-H(7AC)	109.5
H(7AB)-C(7A)-H(7AC)	109.5
N(3A)-C(8A)-C(9A)	113.9(17)
N(3A)-C(8A)-H(8AA)	108.8
C(9A)-C(8A)-H(8AA)	108.8
N(3A)-C(8A)-H(8AC)	108.8
C(9A)-C(8A)-H(8AC)	108.8
H(8AA)-C(8A)-H(8AC)	107.7
C(8A)-C(9A)-C(10A)	114.4(18)
C(8A)-C(9A)-H(9AA)	108.7
C(10A)-C(9A)-H(9AA)	108.7
C(8A)-C(9A)-H(9AC)	108.7
C(10A)-C(9A)-H(9AC)	108.7
H(9AA)-C(9A)-H(9AC)	107.6
N(4A)-C(10A)-C(9A)	114.4(18)
N(4A)-C(10A)-H(10C)	108.6
C(9A)-C(10A)-H(10C)	108.6
N(4A)-C(10A)-H(10D)	108.6
C(9A)-C(10A)-H(10D)	108.6
H(10C)-C(10A)-H(10D)	107.6
N(4A)-C(11A)-H(11D)	109.5
N(4A)-C(11A)-H(11E)	109.5
H(11D)-C(11A)-H(11E)	109.5
N(4A)-C(11A)-H(11F)	109.5
H(11D)-C(11A)-H(11F)	109.5
H(11E)-C(11A)-H(11F)	109.5
N(4A)-C(12A)-C(13A)	116.7(17)
N(4A)-C(12A)-H(12C)	108.1
C(13A)-C(12A)-H(12C)	108.1
N(4A)-C(12A)-H(12D)	108.1
C(13A)-C(12A)-H(12D)	108.1
H(12C)-C(12A)-H(12D)	107.3
N(1A)-C(13A)-C(12A)	111.7(18)
N(1A)-C(13A)-H(13C)	109.3
C(12A)-C(13A)-H(13C)	109.3
N(1A)-C(13A)-H(13D)	109.3
C(12A)-C(13A)-H(13D)	109.3
H(13C)-C(13A)-H(13D)	107.9
N(1A)-C(14A)-H(14D)	109.5
N(1A)-C(14A)-H(14E)	109.5
H(14D)-C(14A)-H(14E)	109.5
N(1A)-C(14A)-H(14F)	109.5
H(14D)-C(14A)-H(14F)	109.5
H(14E)-C(14A)-H(14F)	109.5
C(26)-C(21)-C(22)	115.9(4)
C(26)-C(21)-B(21)	125.9(4)
C(22)-C(21)-B(21)	118.2(4)
C(23)-C(22)-C(21)	122.1(4)
C(23)-C(22)-H(22)	118.9
C(21)-C(22)-H(22)	118.9
C(24)-C(23)-C(22)	120.8(4)
C(24)-C(23)-C(27)	120.4(5)
C(22)-C(23)-C(27)	118.8(4)
C(25)-C(24)-C(23)	118.2(4)
C(25)-C(24)-H(24)	120.9
C(23)-C(24)-H(24)	120.9
C(24)-C(25)-C(26)	120.8(4)
C(24)-C(25)-C(28)	120.1(4)
C(26)-C(25)-C(28)	119.0(4)
C(21)-C(26)-C(25)	122.1(4)
C(21)-C(26)-H(26)	118.9
C(25)-C(26)-H(26)	118.9
F(22)-C(27)-F(21)	106.2(5)
F(22)-C(27)-F(23)	106.8(5)
F(21)-C(27)-F(23)	105.2(4)
F(22)-C(27)-C(23)	112.8(4)
F(21)-C(27)-C(23)	112.1(4)
F(23)-C(27)-C(23)	113.2(4)
F(24)-C(28)-F(25)	106.0(4)
F(24)-C(28)-F(26)	106.8(4)
F(25)-C(28)-F(26)	104.0(4)
F(24)-C(28)-C(25)	113.5(4)
F(25)-C(28)-C(25)	113.0(4)
F(26)-C(28)-C(25)	112.8(4)
C(32)-C(31)-C(36)	115.2(4)
C(32)-C(31)-B(21)	126.3(4)
C(36)-C(31)-B(21)	118.5(4)
C(33)-C(32)-C(31)	122.4(4)
C(33)-C(32)-H(32)	118.8
C(31)-C(32)-H(32)	118.8
C(32)-C(33)-C(34)	121.7(4)
C(32)-C(33)-C(37)	119.8(4)
C(34)-C(33)-C(37)	118.5(4)
C(33)-C(34)-C(35)	117.4(4)
C(33)-C(34)-H(34)	121.3
C(35)-C(34)-H(34)	121.3
C(36)-C(35)-C(34)	120.9(4)
C(36)-C(35)-C(38)	119.0(5)
C(34)-C(35)-C(38)	120.1(4)
C(35)-C(36)-C(31)	122.3(4)
C(35)-C(36)-H(36)	118.8
C(31)-C(36)-H(36)	118.8
F(31)-C(37)-F(33)	106.2(4)
F(31)-C(37)-F(32)	106.2(4)
F(33)-C(37)-F(32)	104.4(4)
F(31)-C(37)-C(33)	113.8(4)
F(33)-C(37)-C(33)	113.3(4)
F(32)-C(37)-C(33)	112.3(4)
F(36)-C(38)-F(34)	106.9(5)
F(36)-C(38)-F(35)	105.9(5)
F(34)-C(38)-F(35)	104.1(4)
F(36)-C(38)-C(35)	114.1(5)
F(34)-C(38)-C(35)	113.5(5)
F(35)-C(38)-C(35)	111.5(4)
C(46)-C(41)-C(42)	115.6(4)
C(46)-C(41)-B(21)	124.5(4)
C(42)-C(41)-B(21)	119.6(4)
C(43)-C(42)-C(41)	122.4(5)
C(43)-C(42)-H(42)	118.8
C(41)-C(42)-H(42)	118.8
C(42)-C(43)-C(44)	120.7(4)
C(42)-C(43)-C(47)	121.2(5)
C(44)-C(43)-C(47)	118.1(4)
C(45)-C(44)-C(43)	117.7(4)
C(45)-C(44)-H(44)	121.2
C(43)-C(44)-H(44)	121.2
C(44)-C(45)-C(46)	121.6(5)
C(44)-C(45)-C(48)	119.7(4)
C(46)-C(45)-C(48)	118.7(4)
C(45)-C(46)-C(41)	121.9(4)
C(45)-C(46)-H(46)	119.0
C(41)-C(46)-H(46)	119.0
F(43)-C(47)-F(41)	106.7(5)
F(43)-C(47)-F(42)	105.4(4)
F(41)-C(47)-F(42)	106.2(5)
F(43)-C(47)-C(43)	111.4(4)
F(41)-C(47)-C(43)	113.9(4)
F(42)-C(47)-C(43)	112.7(4)
F(44A)-C(48)-F(45A)	110.1(12)
F(45)-C(48)-F(44)	105.2(9)
F(44A)-C(48)-F(46A)	106.6(12)
F(45A)-C(48)-F(46A)	103.3(12)
F(45)-C(48)-F(46)	105.9(8)
F(44)-C(48)-F(46)	104.1(8)
F(44A)-C(48)-C(45)	115.1(10)
F(45)-C(48)-C(45)	115.1(7)
F(45A)-C(48)-C(45)	111.2(8)
F(44)-C(48)-C(45)	112.8(9)
F(46A)-C(48)-C(45)	109.8(11)
F(46)-C(48)-C(45)	112.7(7)
C(56)-C(51)-C(52)	115.4(4)
C(56)-C(51)-B(21)	123.9(4)
C(52)-C(51)-B(21)	120.7(4)
C(53)-C(52)-C(51)	122.7(4)
C(53)-C(52)-H(52)	118.6
C(51)-C(52)-H(52)	118.6
C(52)-C(53)-C(54)	120.6(4)
C(52)-C(53)-C(57)	119.6(4)
C(54)-C(53)-C(57)	119.9(4)
C(55)-C(54)-C(53)	118.1(4)
C(55)-C(54)-H(54)	121.0
C(53)-C(54)-H(54)	121.0
C(54)-C(55)-C(56)	121.1(4)
C(54)-C(55)-C(58)	120.6(4)
C(56)-C(55)-C(58)	118.3(4)
C(51)-C(56)-C(55)	122.2(4)
C(51)-C(56)-H(56)	118.9
C(55)-C(56)-H(56)	118.9
F(51)-C(57)-F(52)	109.1(6)
F(51)-C(57)-F(53)	108.7(6)
F(52)-C(57)-F(53)	101.9(5)
F(52A)-C(57)-F(53A)	102.0(11)
F(52A)-C(57)-F(51A)	104.6(12)
F(53A)-C(57)-F(51A)	100.1(10)
F(51)-C(57)-C(53)	113.0(5)
F(52A)-C(57)-C(53)	117.7(9)
F(52)-C(57)-C(53)	112.9(4)
F(53)-C(57)-C(53)	110.6(4)
F(53A)-C(57)-C(53)	116.3(7)
F(51A)-C(57)-C(53)	113.9(7)
F(54)-C(58)-F(56)	106.1(4)
F(54)-C(58)-F(55)	105.8(4)
F(56)-C(58)-F(55)	105.4(4)
F(54)-C(58)-C(55)	112.8(4)
F(56)-C(58)-C(55)	113.1(4)
F(55)-C(58)-C(55)	113.0(4)
C(51)-B(21)-C(31)	112.8(4)
C(51)-B(21)-C(21)	110.6(4)
C(31)-B(21)-C(21)	105.7(4)
C(51)-B(21)-C(41)	104.2(4)
C(31)-B(21)-C(41)	111.1(4)
C(21)-B(21)-C(41)	112.5(4)

Table S104. Anisotropic displacement parameters ($Å^2$x 10^3) for **[(TMC)Co^{II}(I)]B$_{Ar}$F**.
The anisotropic displacement factor exponent takes the form: $-2p^2[h^2 a^{*2}U^{11} + ... + 2 h k a^* b^* U^{12}]$

	U^{11}	U^{22}	U^{33}	U^{23}	U^{13}	U^{12}
I(1)	70(1)	80(1)	27(1)	3(1)	8(1)	-39(1)
Co(1)	22(1)	25(1)	15(1)	1(1)	-2(1)	-3(1)
N(1)	28(2)	45(2)	25(2)	4(1)	-4(2)	-10(2)
N(2)	44(3)	39(3)	74(4)	-8(3)	-17(3)	7(3)
N(3)	28(2)	45(2)	25(2)	4(1)	-4(2)	-10(2)
N(4)	33(3)	33(4)	61(4)	-16(3)	-5(3)	5(3)
C(1)	62(5)	54(4)	41(4)	20(3)	-27(3)	-20(4)
C(2)	58(5)	45(4)	93(6)	24(4)	-40(4)	-8(4)
C(3)	60(4)	35(4)	91(5)	17(4)	-35(4)	2(3)
C(4)	46(5)	39(5)	119(7)	-29(5)	7(5)	4(4)
C(5)	49(4)	34(4)	77(5)	-11(4)	-21(4)	-2(3)
C(6)	45(4)	42(4)	61(5)	5(4)	-13(3)	-18(3)
C(7)	45(3)	57(3)	54(3)	-1(2)	-19(2)	-3(3)
C(8)	31(4)	73(5)	40(4)	5(4)	-2(3)	-16(4)
C(9)	36(4)	81(5)	65(5)	-12(4)	2(4)	-4(4)
C(10)	41(4)	70(5)	69(5)	-25(4)	10(3)	3(4)
C(11)	45(3)	57(3)	54(3)	-1(2)	-19(2)	-3(3)
C(12)	43(4)	41(4)	67(4)	-26(3)	-1(3)	0(3)
C(13)	44(4)	55(4)	40(4)	-24(3)	-1(3)	-15(3)
C(14)	30(3)	36(5)	41(3)	1(4)	-2(2)	-13(4)
I(1A)	73(3)	55(2)	30(2)	11(2)	6(2)	-8(2)
Co(1A)	22(1)	25(1)	15(1)	1(1)	-2(1)	-3(1)
N(1A)	49(6)	36(7)	63(6)	1(6)	-15(6)	12(6)
N(2A)	28(2)	45(2)	25(2)	4(1)	-4(2)	-10(2)
N(3A)	36(6)	48(7)	47(7)	-10(7)	0(6)	2(6)
N(4A)	28(2)	45(2)	25(2)	4(1)	-4(2)	-10(2)
C(1A)	57(7)	35(7)	61(8)	-2(7)	-10(7)	11(6)
C(2A)	58(8)	43(8)	47(9)	-8(8)	-5(8)	-5(7)
C(3A)	41(8)	43(6)	42(8)	0(6)	-2(7)	-12(6)
C(4A)	45(3)	57(3)	54(3)	-1(2)	-19(2)	-3(3)
C(5A)	33(8)	58(7)	50(7)	-6(7)	-2(7)	-1(6)
C(6A)	33(7)	59(7)	49(8)	-7(7)	2(6)	4(6)
C(7A)	67(13)	52(10)	46(12)	-9(10)	12(12)	2(9)
C(8A)	42(6)	43(8)	46(7)	-13(7)	1(6)	-2(7)
C(9A)	45(7)	44(8)	31(8)	1(7)	-2(6)	-17(6)
C(10A)	40(7)	51(8)	29(6)	5(6)	-9(6)	-15(7)
C(11A)	30(3)	36(5)	41(3)	1(4)	-2(2)	-13(4)
C(12A)	56(8)	57(7)	67(8)	6(7)	-16(7)	6(6)
C(13A)	52(7)	52(8)	74(9)	0(7)	-16(7)	14(7)
C(14A)	45(3)	57(3)	54(3)	-1(2)	-19(2)	-3(3)
F(21)	78(3)	43(2)	105(3)	-7(2)	-63(2)	-3(2)
F(22)	50(2)	160(4)	33(2)	9(2)	-9(2)	11(2)
F(23)	40(2)	54(2)	67(2)	-11(2)	-22(2)	16(2)
F(24)	90(3)	28(2)	50(2)	-7(2)	-12(2)	17(2)
F(25)	43(2)	38(2)	48(2)	-14(2)	4(2)	-15(2)
F(26)	61(2)	41(2)	37(2)	-15(1)	23(2)	-18(2)
F(31)	44(2)	48(2)	79(3)	-37(2)	15(2)	-17(2)
F(32)	55(2)	43(2)	32(2)	-2(1)	-16(1)	-11(2)
F(33)	69(2)	25(2)	44(2)	0(1)	-9(2)	-18(2)
F(34)	71(2)	67(2)	51(2)	4(2)	28(2)	16(2)
F(35)	65(2)	65(2)	31(2)	12(2)	-1(2)	-7(2)
F(36)	75(3)	95(3)	53(2)	-22(2)	31(2)	-58(2)
F(41)	71(2)	57(2)	75(3)	23(2)	-35(2)	-40(2)
F(42)	41(2)	47(2)	59(2)	-27(2)	2(2)	-14(2)
F(43)	26(2)	55(2)	70(2)	-14(2)	7(2)	-15(2)
F(44)	51(5)	71(7)	47(6)	15(5)	-19(5)	0(5)
F(45)	119(11)	32(4)	37(4)	1(3)	-14(6)	1(5)
F(46)	63(5)	53(6)	60(6)	25(5)	28(5)	20(4)
F(44A)	88(10)	47(7)	32(6)	10(5)	-16(7)	-25(8)
F(45A)	41(5)	94(13)	106(12)	72(9)	-14(6)	-10(6)
F(46A)	77(11)	40(6)	84(9)	-3(6)	-32(7)	21(6)
F(51)	98(6)	98(6)	87(5)	-50(4)	-63(5)	75(5)
F(52)	82(5)	45(3)	53(3)	7(2)	36(3)	29(3)
F(53)	58(3)	23(2)	122(6)	3(3)	35(3)	4(2)
F(51A)	55(6)	40(8)	123(15)	22(9)	51(8)	33(6)
F(52A)	112(13)	59(9)	72(10)	-33(9)	-35(10)	33(8)
F(53A)	52(10)	42(9)	44(6)	11(6)	9(6)	25(7)
F(54)	70(2)	45(2)	45(2)	11(2)	30(2)	8(2)
F(55)	62(2)	39(2)	50(2)	14(2)	-22(2)	-12(2)
F(56)	52(2)	22(2)	41(2)	0(1)	2(1)	-9(1)
C(21)	20(2)	25(2)	22(2)	-1(2)	4(2)	-7(2)
C(22)	23(2)	26(2)	31(2)	-6(2)	3(2)	-4(2)
C(23)	21(2)	28(2)	30(2)	-1(2)	2(2)	-1(2)
C(24)	24(2)	24(2)	30(2)	4(2)	6(2)	-1(2)
C(25)	21(2)	24(2)	24(2)	-3(2)	9(2)	-5(2)
C(26)	22(2)	25(2)	23(2)	-1(2)	3(2)	-7(2)
C(27)	32(3)	37(3)	40(3)	-2(2)	-6(2)	3(2)
C(28)	33(3)	25(3)	32(3)	-1(2)	6(2)	-4(2)
C(31)	20(2)	21(2)	25(2)	1(2)	-2(2)	-1(2)
C(32)	23(2)	27(2)	24(2)	0(2)	-2(2)	-2(2)
C(33)	27(2)	25(2)	26(2)	2(2)	-8(2)	-1(2)
C(34)	25(2)	25(2)	32(2)	4(2)	-4(2)	-4(2)
C(35)	24(2)	29(3)	27(2)	2(2)	-3(2)	-6(2)
C(36)	22(2)	21(2)	30(2)	-2(2)	-3(2)	0(2)
C(37)	34(3)	28(3)	33(3)	0(2)	-4(2)	-10(2)
C(38)	41(3)	40(3)	36(3)	0(2)	3(2)	-13(2)
C(41)	19(2)	24(2)	24(2)	-8(2)	5(2)	1(2)
C(42)	23(2)	30(3)	23(2)	-6(2)	3(2)	-2(2)
C(43)	18(2)	32(2)	33(2)	-7(2)	-1(2)	-5(2)
C(44)	25(2)	38(3)	29(2)	-7(2)	-7(2)	3(2)

C(45)	25(2)	27(2)	32(2)	-4(2)	0(2)	5(2)
C(46)	22(2)	22(2)	32(2)	-5(2)	2(2)	2(2)
C(47)	31(3)	40(3)	38(3)	-5(2)	-6(2)	-6(2)
C(48)	39(3)	38(3)	39(3)	0(2)	-7(2)	1(2)
C(51)	20(2)	24(2)	25(2)	-8(2)	-4(2)	-6(2)
C(52)	22(2)	24(2)	25(2)	0(2)	-2(2)	-6(2)
C(53)	25(2)	24(2)	30(2)	-2(2)	2(2)	-2(2)
C(54)	24(2)	29(2)	25(2)	-6(2)	1(2)	-2(2)
C(55)	23(2)	27(2)	26(2)	-4(2)	-2(2)	-8(2)
C(56)	22(2)	23(2)	25(2)	-6(2)	-1(2)	-2(2)
C(57)	42(3)	29(3)	34(2)	1(2)	8(2)	7(2)
C(58)	37(3)	28(3)	31(3)	0(2)	-1(2)	-3(2)
B(21)	23(3)	24(3)	25(2)	-5(2)	0(2)	-4(2)

Table S105. Hydrogen coordinates (x 10^4) and isotropic displacement parameters (Å^{2}x 10^3) for **[(TMC)Co^{II}(I)]B$_{Ar}$F**.

	x	y	z	U(eq)
H(1A)	1546	3747	8181	62
H(1AB)	2334	4094	8209	62
H(2A)	1175	4456	8829	79
H(2AB)	1317	4832	8332	79
H(3A)	2509	5136	8630	74
H(3AB)	1835	5501	8888	74
H(4A)	1569	5340	9670	102
H(4B)	1957	4769	9986	102
H(4C)	1360	4564	9593	102
H(5A)	2834	5736	9415	64
H(5AB)	3056	5189	9811	64
H(6A)	3556	5192	8850	59
H(6AB)	4041	5398	9304	59
H(7A)	4353	3882	9797	78
H(7B)	3983	4572	9968	78
H(7C)	4742	4587	9681	78
H(8A)	4811	4515	8883	58
H(8AB)	4140	4291	8552	58
H(9A)	4816	3381	9203	73
H(9AB)	5092	3456	8663	73
H(10A)	3888	3096	8400	72
H(10B)	4347	2522	8668	72
H(11A)	4002	2009	9339	78
H(11B)	3403	2335	9687	78
H(11C)	4191	2680	9632	78
H(12A)	2604	2269	9093	61
H(12B)	3129	2071	8655	61
H(13A)	2767	3053	8238	56
H(13B)	2061	2605	8362	56
H(14A)	1101	3094	8815	53
H(14B)	1308	3612	9233	53
H(14C)	1624	2859	9244	53
H(1AA)	2573	5406	8893	61
H(1AC)	2420	5811	9377	61
H(2AA)	3603	5982	9192	59
H(2AC)	3590	5502	9650	59
H(3AA)	3782	4986	8723	50
H(3AC)	4459	5239	9037	50
H(4AA)	4087	4594	9968	78
H(4AB)	4869	4512	9716	78
H(4AC)	4388	3859	9838	78
H(5AA)	4345	4065	8595	57
H(5AC)	5000	4042	8979	57
H(6AA)	4616	2942	8830	57
H(6AC)	4405	3134	9371	57
H(7AA)	3088	2176	8898	82
H(7AB)	3268	2421	9431	82
H(7AC)	3916	2181	9085	82
H(8AA)	3722	2708	8275	52
H(8AC)	3603	3506	8254	52
H(9AA)	2448	2571	8421	48
H(9AC)	2621	2889	7908	48
H(10C)	2363	3988	8248	48
H(10D)	1667	3506	8190	48
H(11D)	1041	3043	8844	53
H(11E)	1359	3122	9374	53
H(11F)	1762	2639	8998	53
H(12C)	986	4134	8881	72
H(12D)	1615	4554	8620	72
H(13C)	1366	5140	9289	71
H(13D)	1349	4475	9615	71
H(14D)	1917	4779	10132	78
H(14E)	2388	5446	10038	78
H(14F)	2792	4748	10150	78
H(22)	1734	7659	6077	32
H(24)	1036	9513	6467	31
H(26)	2607	8562	7235	28
H(32)	2395	6142	6195	30
H(34)	833	5308	7034	33
H(36)	1824	7047	7432	30
H(42)	3834	8276	6948	30
H(44)	4843	7520	8125	36
H(46)	3155	6510	7517	31
H(52)	3726	6323	6427	29
H(54)	4568	7003	5175	31
H(56)	3121	8143	5920	28

[(D_{12}-TMC)Co^{II}(**I**)]**I**

The crystal structure of **[(D_{12}-TMC)Co^{II}(I)]I** was solved in the tetragonal space group $P4_12_12$ and refined as inversion twin (twin ratio 0.5(2)). The asymmetric unit contains half of the molecule and the molecule was found to be disordered over two positions. The disorder was modelled with same distance restraints on 1,2 and 1,3-distances, strong restraints on the anisotropic displacement parameters and strong advanced rigid bond restraints. Additionally, some anisotropic displacement parameters were set to the same parameters. The disorder ratio was refined freely and converged to 0.847(4).

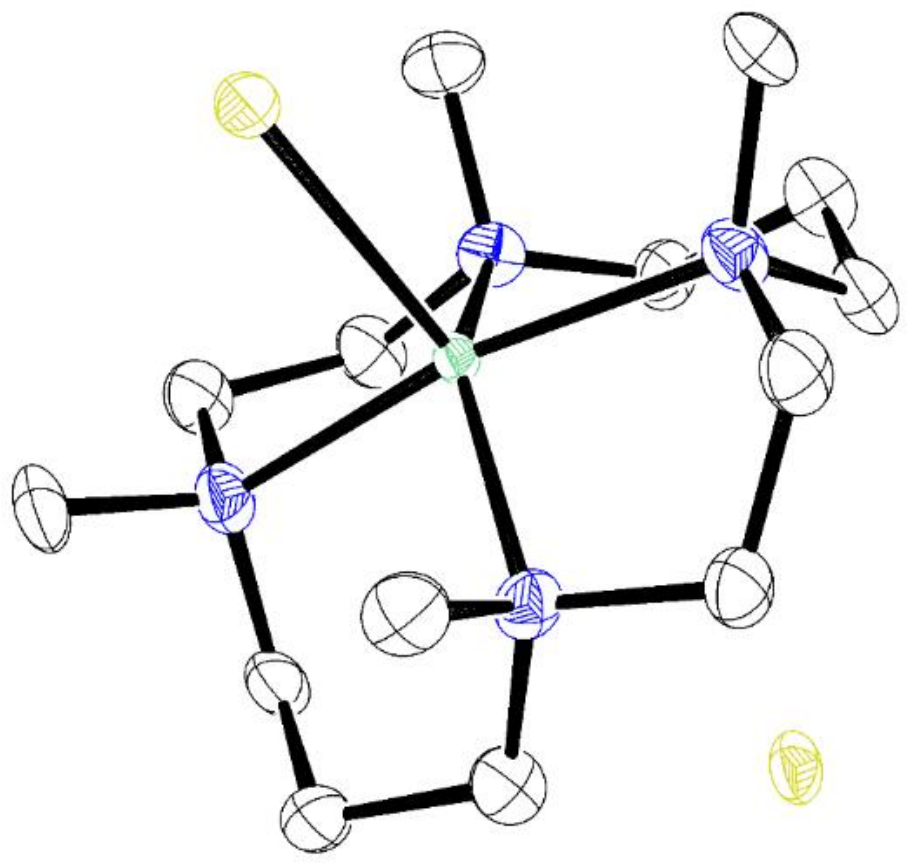

Figure S21. Molecular structure of **[(D_{12}-TMC)Co^{II}(I)]I**.

Table S106. Crystal data and structure refinement for **[(D_{12}-TMC)Co^{II}(I)]I**.

CCDC No	2062711	
Empirical formula	$C_{28}H_{40}Co_2D_{24}I_4N_8$	
Formula weight	1162.48	
Temperature	100(2) K	
Wavelength	0.71073 Å	
Crystal system	Tetragonal	
Space group	$P4_12_12$	
Unit cell dimensions	a = 8.8340(4) Å	α = 90°
	b = 8.8340(4) Å	β = 90°
	c = 25.2354(11) Å	γ = 90°
Volume	1969.4(2) Å^3	
Z	2	
Density (calculated)	1.960 Mg/m^3	
Absorption coefficient	4.007 mm^{-1}	
F(000)	1108	
Crystal size	0.161 x 0.085 x 0.057 mm^3	
Theta range for data collection	2.443 to 31.506°.	
Index ranges	-12 ≤ h ≤ 12, -12 ≤ k ≤ 12, -37 ≤ l ≤ 37	
Reflections collected	165537	
Independent reflections	3267 [R(int) = 0.0523]	
Completeness to theta = 25.242°	99.7 %	
Absorption correction	Semi-empirical from equivalents	
Refinement method	Full-matrix least-squares on F^2	
Data / restraints / parameters	3267 / 619 / 180	
Goodness-of-fit on F^2	1.175	
Final R indices [I>2σ(I)]	R1 = 0.0798, wR2 = 0.2028	
R indices (all data)	R1 = 0.0829, wR2 = 0.2041	
Absolute structure parameter	0.5(2)	
Extinction coefficient	0.0024(3)	
Largest diff. peak and hole	2.658 and -2.956 eÅ^{-3}	

Table S107. Atomic coordinates (x 10^4) and equivalent isotropic displacement parameters ($Å^2$x 10^3) for **[(D_{12}-TMC)Co^{II}(I)]I**. U(eq) is defined as one third of the trace of the orthogonalized U^{ij} tensor.

	x	y	z	U(eq)
I(1)	7498(1)	7498(1)	0	24(1)
I(2)	13568(1)	13568(1)	0	28(1)
Co(1)	9635(2)	9635(2)	0	14(1)
N(1)	10718(15)	8935(15)	-737(5)	26(3)
N(2)	8881(14)	11525(15)	-463(5)	24(2)
C(1)	10640(20)	7324(19)	-899(7)	33(4)
D(1A)	9579	6996	-904	50
D(1B)	11078	7207	-1253	50
D(1C)	11207	6704	-645	50
C(2)	9940(20)	9820(20)	-1156(5)	30(3)
C(3)	9690(20)	11459(19)	-981(6)	30(3)
C(4)	7232(17)	11390(20)	-565(8)	32(4)
D(4A)	7012	10389	-714	49
D(4B)	6678	11516	-231	49
D(4C)	6916	12174	-816	49
C(5)	9180(20)	13029(18)	-241(7)	28(3)
C(6)	8580(30)	13290(20)	317(6)	30(3)
C(7)	9380(20)	12333(18)	734(6)	28(3)
I(1A)	12564(11)	12564(11)	0	64(5)
I(2A)	6528(14)	6528(14)	0	68(5)
Co(1A)	10409(11)	10409(11)	0	14(1)
N(1A)	11230(50)	8560(40)	-477(13)	30(6)
N(2A)	9300(50)	10980(50)	-737(11)	27(5)
C(1A)	12900(50)	8590(80)	-520(30)	30(3)
D(1AA)	13228	9557	-665	45
D(1AB)	13341	8458	-162	45
D(1AC)	13241	7761	-747	45
C(2A)	10600(90)	8600(50)	-1022(14)	31(6)
C(3A)	10020(80)	10160(70)	-1182(11)	30(6)
C(4A)	9380(80)	12610(50)	-860(20)	25(8)
D(4AA)	8905	13191	-571	38
D(4AB)	10436	12921	-897	38
D(4AC)	8836	12817	-1191	38
C(5A)	7680(50)	10560(80)	-728(18)	27(6)
C(6A)	6800(60)	11410(90)	-301(19)	23(7)
C(7A)	7080(40)	10780(80)	252(18)	23(7)

Table S108. Bond lengths [Å] and angles [°] for **[(D$_{12}$-TMC)Co^{II}(I)]I**.

I(1)-Co(1)	2.670(3)	C(3)-C(2)-H(2AB)	109.4
Co(1)-N(2)	2.145(13)	H(2A)-C(2)-H(2AB)	108.0
Co(1)-N(2)#1	2.145(13)	N(2)-C(3)-C(2)	111.1(13)
Co(1)-N(1)	2.181(13)	N(2)-C(3)-H(3A)	109.4
Co(1)-N(1)#1	2.182(13)	C(2)-C(3)-H(3A)	109.4
N(1)-C(7)#1	1.479(16)	N(2)-C(3)-H(3AB)	109.4
N(1)-C(1)	1.482(16)	C(2)-C(3)-H(3AB)	109.4
N(1)-C(2)	1.482(15)	H(3A)-C(3)-H(3AB)	108.0
N(2)-C(5)	1.465(16)	N(2)-C(4)-D(4A)	109.5
N(2)-C(4)	1.485(16)	N(2)-C(4)-D(4B)	109.5
N(2)-C(3)	1.491(15)	D(4A)-C(4)-D(4B)	109.5
C(1)-D(1A)	0.9800	N(2)-C(4)-D(4C)	109.5
C(1)-D(1B)	0.9800	D(4A)-C(4)-D(4C)	109.5
C(1)-D(1C)	0.9800	D(4B)-C(4)-D(4C)	109.5
C(2)-C(3)	1.53(2)	N(2)-C(5)-C(6)	115.4(14)
C(2)-H(2A)	0.9900	N(2)-C(5)-H(5A)	108.4
C(2)-H(2AB)	0.9900	C(6)-C(5)-H(5A)	108.4
C(3)-H(3A)	0.9900	N(2)-C(5)-H(5AB)	108.4
C(3)-H(3AB)	0.9900	C(6)-C(5)-H(5AB)	108.4
C(4)-D(4A)	0.9800	H(5A)-C(5)-H(5AB)	107.5
C(4)-D(4B)	0.9800	C(5)-C(6)-C(7)	113.2(15)
C(4)-D(4C)	0.9800	C(5)-C(6)-H(6A)	108.9
C(5)-C(6)	1.524(19)	C(7)-C(6)-H(6A)	108.9
C(5)-H(5A)	0.9900	C(5)-C(6)-H(6B)	108.9
C(5)-H(5AB)	0.9900	C(7)-C(6)-H(6B)	108.9
C(6)-C(7)	1.524(19)	H(6A)-C(6)-H(6B)	107.8
C(6)-H(6A)	0.9900	N(1)#1-C(7)-C(6)	114.6(14)
C(6)-H(6B)	0.9900	N(1)#1-C(7)-H(7A)	108.6
C(7)-H(7A)	0.9900	C(6)-C(7)-H(7A)	108.6
C(7)-H(7AB)	0.9900	N(1)#1-C(7)-H(7AB)	108.6
I(1A)-Co(1A)	2.693(16)	C(6)-C(7)-H(7AB)	108.6
Co(1A)-N(1A)	2.16(2)	H(7A)-C(7)-H(7AB)	107.6
Co(1A)-N(1A)#1	2.16(2)	N(1A)-Co(1A)-N(1A)#1	145(3)
Co(1A)-N(2A)#1	2.16(2)	N(1A)-Co(1A)-N(2A)#1	93.4(12)
Co(1A)-N(2A)	2.16(2)	N(1A)#1-Co(1A)-N(2A)#1	81.3(12)
N(1A)-C(7A)#1	1.48(2)	N(1A)-Co(1A)-N(2A)	81.3(12)
N(1A)-C(1A)	1.48(2)	N(1A)#1-Co(1A)-N(2A)	93.4(12)
N(1A)-C(2A)	1.48(2)	N(2A)#1-Co(1A)-N(2A)	162(3)
N(2A)-C(3A)	1.48(2)	N(1A)-Co(1A)-I(1A)	107.3(13)
N(2A)-C(5A)	1.48(2)	N(1A)#1-Co(1A)-I(1A)	107.3(13)
N(2A)-C(4A)	1.48(2)	N(2A)#1-Co(1A)-I(1A)	99.0(13)
C(1A)-D(1AA)	0.9800	N(2A)-Co(1A)-I(1A)	99.0(13)
C(1A)-D(1AB)	0.9800	C(7A)#1-N(1A)-C(1A)	108(2)
C(1A)-D(1AC)	0.9800	C(7A)#1-N(1A)-C(2A)	106(2)
C(2A)-C(3A)	1.52(3)	C(1A)-N(1A)-C(2A)	108(2)
C(2A)-H(2AA)	0.9900	C(7A)#1-N(1A)-Co(1A)	111(2)
C(2A)-H(2AC)	0.9900	C(1A)-N(1A)-Co(1A)	111(2)
C(3A)-H(3AA)	0.9900	C(2A)-N(1A)-Co(1A)	111.8(17)
C(3A)-H(3AC)	0.9900	C(3A)-N(2A)-C(5A)	108(2)
C(4A)-D(4AA)	0.9800	C(3A)-N(2A)-C(4A)	107(2)
C(4A)-D(4AB)	0.9800	C(5A)-N(2A)-C(4A)	107(2)
C(4A)-D(4AC)	0.9800	C(3A)-N(2A)-Co(1A)	110.1(18)
C(5A)-C(6A)	1.53(3)	C(5A)-N(2A)-Co(1A)	111(2)
C(5A)-H(5AA)	0.9900	C(4A)-N(2A)-Co(1A)	113(2)
C(5A)-H(5AC)	0.9900	N(1A)-C(1A)-D(1AA)	109.5
C(6A)-C(7A)	1.52(3)	N(1A)-C(1A)-D(1AB)	109.5
C(6A)-H(6C)	0.9900	D(1AA)-C(1A)-D(1AB)	109.5
C(6A)-H(6D)	0.9900	N(1A)-C(1A)-D(1AC)	109.5
C(7A)-H(7AA)	0.9900	D(1AA)-C(1A)-D(1AC)	109.5
C(7A)-H(7AC)	0.9900	D(1AB)-C(1A)-D(1AC)	109.5
		N(1A)-C(2A)-C(3A)	113(2)
N(2)-Co(1)-N(2)#1	141.3(7)	N(1A)-C(2A)-H(2AA)	108.9
N(2)-Co(1)-N(1)	83.8(5)	C(3A)-C(2A)-H(2AA)	108.9
N(2)#1-Co(1)-N(1)	92.0(5)	N(1A)-C(2A)-H(2AC)	108.9
N(2)-Co(1)-N(1)#1	92.0(5)	C(3A)-C(2A)-H(2AC)	108.9
N(2)#1-Co(1)-N(1)#1	83.8(5)	H(2AA)-C(2A)-H(2AC)	107.7
N(1)-Co(1)-N(1)#1	167.4(8)	N(2A)-C(3A)-C(2A)	113(2)
N(2)-Co(1)-I(1)	109.3(3)	N(2A)-C(3A)-H(3AA)	109.1
N(2)#1-Co(1)-I(1)	109.3(3)	C(2A)-C(3A)-H(3AA)	109.1
N(1)-Co(1)-I(1)	96.3(4)	N(2A)-C(3A)-H(3AC)	109.1
N(1)#1-Co(1)-I(1)	96.3(4)	C(2A)-C(3A)-H(3AC)	109.1
C(7)#1-N(1)-C(1)	107.4(13)	H(3AA)-C(3A)-H(3AC)	107.8
C(7)#1-N(1)-C(2)	108.0(13)	N(2A)-C(4A)-D(4AA)	109.5
C(1)-N(1)-C(2)	106.9(13)	N(2A)-C(4A)-D(4AB)	109.5
C(7)#1-N(1)-Co(1)	110.1(9)	D(4AA)-C(4A)-D(4AB)	109.5
C(1)-N(1)-Co(1)	119.1(10)	N(2A)-C(4A)-D(4AC)	109.5
C(2)-N(1)-Co(1)	104.8(9)	D(4AA)-C(4A)-D(4AC)	109.5
C(5)-N(2)-C(4)	108.3(13)	D(4AB)-C(4A)-D(4AC)	109.5
C(5)-N(2)-C(3)	106.6(13)	N(2A)-C(5A)-C(6A)	112(3)
C(4)-N(2)-C(3)	108.5(13)	N(2A)-C(5A)-H(5AA)	109.1
C(5)-N(2)-Co(1)	116.2(9)	C(6A)-C(5A)-H(5AA)	109.1
C(4)-N(2)-Co(1)	109.6(10)	N(2A)-C(5A)-H(5AC)	109.1
C(3)-N(2)-Co(1)	107.3(9)	C(6A)-C(5A)-H(5AC)	109.1
N(1)-C(1)-D(1A)	109.5	H(5AA)-C(5A)-H(5AC)	107.9
N(1)-C(1)-D(1B)	109.5	C(7A)-C(6A)-C(5A)	112(4)
D(1A)-C(1)-D(1B)	109.5	C(7A)-C(6A)-H(6C)	109.1
N(1)-C(1)-D(1C)	109.5	C(5A)-C(6A)-H(6C)	109.1
D(1A)-C(1)-D(1C)	109.5	C(7A)-C(6A)-H(6D)	109.1
D(1B)-C(1)-D(1C)	109.5	C(5A)-C(6A)-H(6D)	109.1
N(1)-C(2)-C(3)	111.2(12)	H(6C)-C(6A)-H(6D)	107.9
N(1)-C(2)-H(2A)	109.4	N(1A)#1-C(7A)-C(6A)	114(3)
C(3)-C(2)-H(2A)	109.4	N(1A)#1-C(7A)-H(7AA)	108.8
N(1)-C(2)-H(2AB)	109.4	C(6A)-C(7A)-H(7AA)	108.8

N(1A)#1-C(7A)-H(7AC)	108.8	H(7AA)-C(7A)-H(7AC)	107.7
C(6A)-C(7A)-H(7AC)	108.8		

Symmetry transformations used to generate equivalent atoms: #1 y,x,-z

Table S109. Anisotropic displacement parameters ($Å^2$x 10^3) for **[(D$_{12}$-TMC)Co^{II}(I)]I**.
The anisotropic displacement factor exponent takes the form: $-2p^2[h^2 a^{*2}U^{11} + ... + 2 h k a^* b^* U^{12}]$

	U^{11}	U^{22}	U^{33}	U^{23}	U^{13}	U^{12}
I(1)	21(1)	21(1)	30(1)	-4(1)	4(1)	-3(1)
I(2)	31(1)	31(1)	23(1)	-9(1)	9(1)	-7(1)
Co(1)	14(1)	14(1)	14(1)	-2(1)	2(1)	-1(1)
N(1)	30(7)	27(5)	19(5)	-4(4)	1(5)	4(5)
N(2)	25(5)	26(5)	20(4)	6(4)	-1(4)	2(4)
C(1)	43(10)	29(6)	28(8)	-12(6)	9(8)	2(6)
C(2)	35(8)	37(6)	19(5)	1(4)	0(5)	5(6)
C(3)	34(7)	33(6)	22(5)	3(5)	3(5)	4(6)
C(4)	26(6)	33(9)	38(9)	6(8)	-5(5)	2(6)
C(5)	31(8)	33(6)	21(5)	-1(5)	-4(5)	3(6)
C(6)	38(7)	26(7)	26(5)	5(5)	5(5)	5(6)
C(7)	34(9)	27(9)	22(6)	-1(6)	5(6)	9(7)
I(1A)	69(6)	69(6)	55(8)	32(7)	-32(7)	-24(10)
I(2A)	77(7)	77(7)	50(7)	23(7)	-23(7)	-21(10)
Co(1A)	14(1)	14(1)	14(1)	-2(1)	2(1)	-1(1)
N(1A)	38(8)	28(13)	23(10)	1(12)	4(8)	6(9)
N(2A)	32(9)	29(9)	21(10)	3(9)	1(8)	5(8)
C(1A)	38(7)	26(7)	26(5)	5(5)	5(5)	5(6)
C(2A)	38(11)	31(10)	24(10)	1(10)	3(9)	6(10)
C(3A)	34(11)	32(10)	23(9)	1(9)	1(9)	5(9)
C(4A)	32(18)	29(10)	15(19)	2(11)	-7(17)	2(10)
C(5A)	31(9)	23(12)	25(12)	6(12)	0(9)	7(9)
C(6A)	28(12)	17(13)	23(12)	7(12)	-2(10)	3(11)
C(7A)	28(12)	17(13)	23(12)	7(12)	-2(10)	3(11)

Table S110. Hydrogen coordinates (x 10^4) and isotropic displacement parameters ($Å^2$x 10^3) for **[(D$_{12}$-TMC)Co^{II}(I)]I**.

	x	y	z	U(eq)
H(2A)	8956	9348	-1238	36
H(2AB)	10565	9809	-1483	36
H(3A)	10682	11976	-948	36
H(3AB)	9093	11998	-1253	36
H(5A)	8724	13796	-479	34
H(5AB)	10286	13198	-239	34
H(6A)	7481	13054	324	36
H(6B)	8698	14373	409	36
H(7A)	10482	12401	675	33
H(7AB)	9162	12768	1088	33
H(2AA)	9762	7866	-1048	37
H(2AC)	11399	8288	-1275	37
H(3AA)	10875	10771	-1319	35
H(3AC)	9275	10044	-1473	35
H(5AA)	7589	9454	-663	32
H(5AC)	7226	10773	-1079	32
H(6C)	7094	12492	-310	27
H(6D)	5702	11350	-381	27
H(7AA)	6267	11138	491	27
H(7AC)	7029	9664	238	27

[(D_{12}-TMC)Co^{II}(I)]$B_{Ar}F$

[(D_{12}-TMC)Co^{II}(I)]$B_{Ar}F$ was solved in the orthorhombic space group *Pbca*. The asymmetric unit contains a full complex cation and a full anion. The complex was found to be fully disordered over three positions while the anion exhibits disorder at three CF_3 groups. The disorder was modelled using same distance restraints, similarity restraints on anisotropic displacement parameters and advanced rigid bond restraints. The disordered iodine atom was set to the same anisotropic displacement parameter. The disorder ratio refined to 0.717(2):0.090(2):0.193(2) and for the CF_3 groups to 0.709(9), 0.55(2) and 0.45(5).

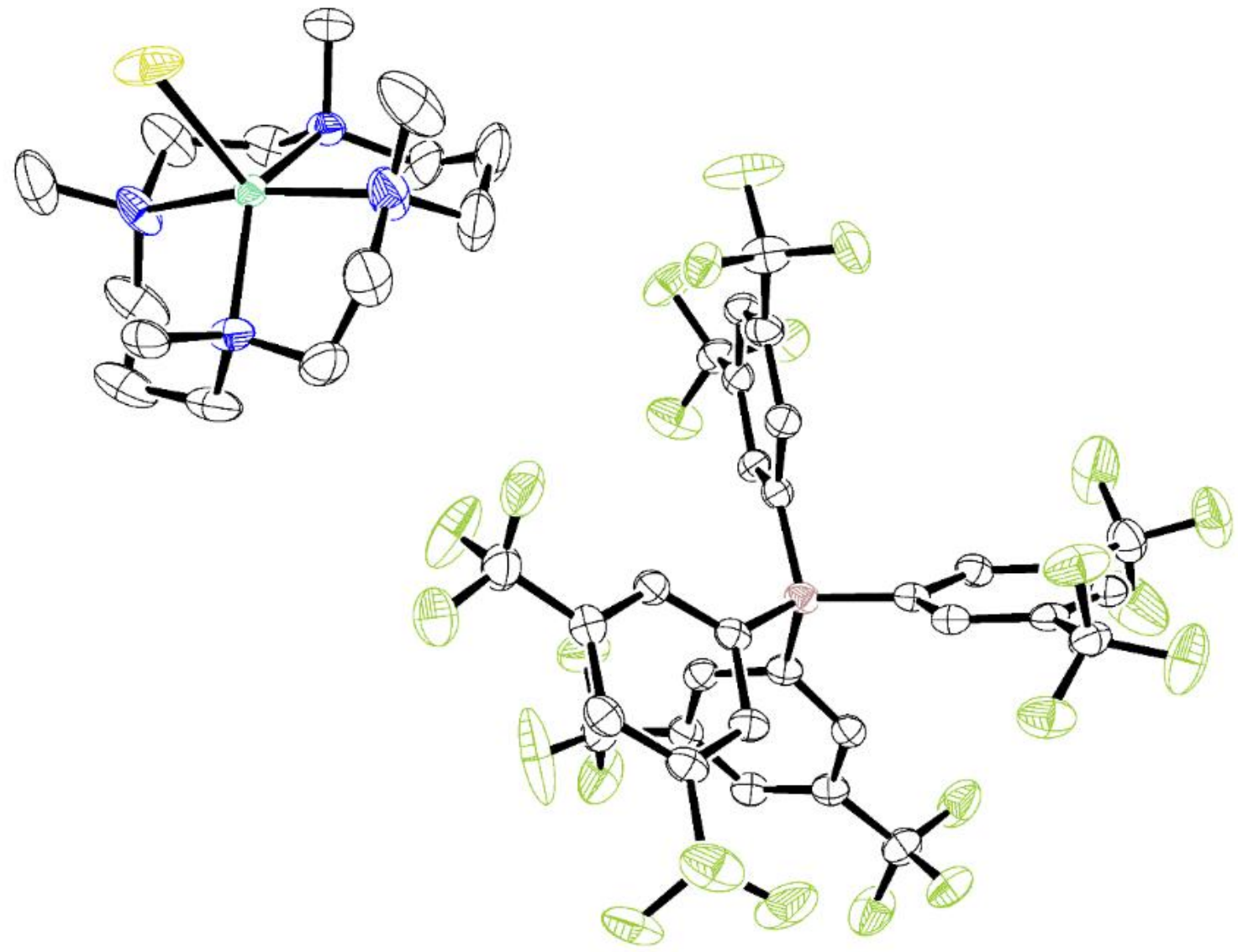

Figure S22. Molecular structure of **[(D_{12}-TMC)Co^{II}(I)]$B_{Ar}F$**.

Table S111. Crystal data and structure refinement for **[(D_{12}-TMC)Co^{II}(I)]$B_{Ar}F$**.

CCDC No	2062713	
Empirical formula	$C_{46}H_{32}BCoD_{12}F_{24}IN_4$	
Formula weight	1317.56	
Temperature	100(2) K	
Wavelength	0.71073 Å	
Crystal system	Orthorhombic	
Space group	*Pbca*	
Unit cell dimensions	a = 18.2417(7) Å	α = 90°
	b = 19.8474(8) Å	β = 90°
	c = 28.1034(10) Å	γ = 90°
Volume	10174.8(7) $Å^3$	
Z	8	
Density (calculated)	1.720 Mg/m^3	
Absorption coefficient	1.071 mm^{-1}	
F(000)	5192	
Crystal size	0.600 x 0.367 x 0.309 mm^3	
Theta range for data collection	1.680 to 26.732°.	
Index ranges	-23 ≤ h ≤ 23, -25 ≤ k ≤ 25, -35 ≤ l ≤ 35	
Reflections collected	290382	
Independent reflections	10807 [R(int) = 0.0705]	
Completeness to theta = 25.242°	100.0 %	
Absorption correction	Semi-empirical from equivalents	
Refinement method	Full-matrix least-squares on F^2	
Data / restraints / parameters	10807 / 4420 / 1130	
Goodness-of-fit on F^2	1.019	
Final R indices [I>2σ(I)]	R1 = 0.0428, wR2 = 0.1063	
R indices (all data)	R1 = 0.0650, wR2 = 0.1209	
Extinction coefficient	0.00027(5)	
Largest diff. peak and hole	0.561 and -0.823 e$Å^{-3}$	

Table S112. Atomic coordinates (x 10^4) and equivalent isotropic displacement parameters ($Å^2$x 10^3) for **[(D$_{12}$-TMC)Co^{II}(I)]B$_{ArF}$**. U(eq) is defined as one third of the trace of the orthogonalized U^{ij} tensor.

	x	y	z	U(eq)
Co(1)	2912(1)	3838(1)	9276(1)	23(1)
I(1)	2671(1)	3443(1)	10134(1)	54(1)
N(1)	3869(3)	4418(3)	9241(2)	30(1)
N(2)	3525(3)	2895(3)	9030(2)	41(1)
N(3)	2131(2)	3482(2)	8756(2)	29(1)
N(4)	2337(3)	4818(3)	9308(2)	47(1)
C(1)	4373(3)	4253(3)	8846(2)	46(1)
C(2)	4665(3)	3542(4)	8845(2)	57(2)
C(3)	4102(3)	3009(3)	8690(2)	63(2)
C(4)	3831(4)	2468(3)	9434(3)	66(2)
D(4A)	4053	2059	9302	99
D(4B)	3434	2345	9652	99
D(4C)	4204	2726	9606	99
C(5)	2924(3)	2476(3)	8814(2)	51(1)
C(6)	2464(3)	2911(3)	8493(2)	46(1)
C(7)	1488(7)	3219(4)	9016(4)	38(2)
D(7A)	1121	3059	8787	57
D(7B)	1276	3577	9212	57
D(7C)	1641	2844	9220	57
C(8)	1891(3)	3988(3)	8400(2)	52(1)
C(9)	1537(3)	4601(3)	8596(3)	66(2)
C(10)	2066(3)	5075(3)	8844(3)	65(2)
C(11)	1746(3)	4880(3)	9665(3)	74(2)
D(11A)	1564	5345	9669	112
D(11B)	1937	4763	9980	112
D(11C)	1344	4574	9582	112
C(12)	2948(3)	5264(3)	9472(3)	54(1)
C(13)	3637(3)	5133(3)	9198(2)	49(2)
C(14)	4283(8)	4348(6)	9700(3)	35(2)
D(14A)	4371	3869	9766	53
D(14B)	3995	4544	9960	53
D(14C)	4753	4584	9675	53
I(1A)	2894(14)	3181(9)	10080(5)	34(1)
N(1A)	3633(13)	3184(14)	8916(9)	42(3)
N(2A)	1962(13)	3480(14)	8853(9)	38(4)
N(3A)	2109(13)	4661(11)	9467(9)	47(3)
N(4A)	3789(15)	4533(13)	9293(11)	38(3)
C(1A)	3544(16)	3160(20)	8395(10)	43(4)
C(2A)	2795(15)	2905(19)	8262(13)	42(4)
C(3A)	2173(17)	3394(19)	8361(10)	40(4)
C(4A)	1610(20)	2878(17)	9060(14)	39(7)
D(4AA)	1219	2722	8848	59
D(4AB)	1403	2991	9371	59
D(4AC)	1977	2520	9097	59
C(5A)	1381(15)	4036(15)	8865(10)	44(4)
C(6A)	1365(13)	4387(16)	9337(11)	47(4)
C(7A)	2000(20)	4830(20)	9989(9)	44(6)
D(7AA)	1507	5001	10039	67
D(7AB)	2362	5173	10083	67
D(7AC)	2078	4422	10180	67
C(8A)	2218(16)	5290(16)	9201(13)	49(4)
C(9A)	2980(16)	5559(14)	9274(16)	47(4)
C(10A)	3570(20)	5150(20)	9042(13)	41(4)
C(11A)	4050(20)	4660(20)	9771(10)	39(6)
D(11D)	3697	4931	9944	58
D(11E)	4524	4895	9756	58
D(11F)	4121	4225	9936	58
C(12A)	4385(19)	4222(15)	8996(14)	42(4)
C(13A)	4373(15)	3469(15)	9030(15)	45(3)
C(14A)	3670(20)	2463(17)	9087(17)	44(6)
D(14D)	3243	2215	8972	66
D(14E)	3682	2455	9435	66
D(14F)	4119	2252	8963	66
I(1B)	2874(7)	3308(3)	10124(2)	34(1)
N(1B)	3482(9)	3075(9)	8923(6)	41(3)
N(2B)	1979(8)	3777(8)	8872(5)	41(3)
N(3B)	2412(8)	4861(8)	9510(5)	37(3)
N(4B)	4010(12)	4366(9)	9262(9)	32(3)
C(1B)	3358(8)	3070(11)	8402(6)	40(3)
C(2B)	2556(10)	3019(10)	8270(8)	41(3)
C(3B)	2133(10)	3668(10)	8370(6)	41(3)
C(4B)	1470(20)	3269(15)	9063(15)	42(6)
D(4BA)	1009	3283	8883	64
D(4BB)	1369	3365	9399	64
D(4BC)	1689	2820	9034	64
C(5B)	1572(10)	4457(9)	8898(7)	52(3)
C(6B)	1618(9)	4762(10)	9389(7)	52(3)
C(7B)	2416(13)	5030(11)	10036(5)	51(4)
D(7BA)	2150	5452	10089	76
D(7BB)	2923	5080	10145	76
D(7BC)	2178	4666	10214	76
C(8B)	2716(9)	5442(10)	9257(8)	48(3)
C(9B)	3525(9)	5520(9)	9310(7)	45(3)
C(10B)	3976(10)	5030(8)	9032(6)	39(3)
C(11B)	4380(30)	4420(20)	9719(10)	40(7)
D(11G)	4815	4707	9684	60
D(11H)	4529	3972	9827	60
D(11I)	4047	4622	9952	60
C(12B)	4462(11)	3928(9)	8934(8)	43(3)
C(13B)	4287(8)	3192(8)	9001(7)	43(3)

C(14B)	3352(13)	2359(9)	9088(8)	51(4)
D(14G)	2826	2287	9139	77
D(14H)	3615	2281	9387	77
D(14I)	3530	2044	8846	77
F(21)	450(2)	7993(1)	5757(1)	82(1)
F(22)	265(1)	9033(1)	5832(1)	59(1)
F(23)	1129(2)	8676(2)	5391(1)	92(1)
F(24)	1525(2)	10253(1)	7087(1)	60(1)
F(25)	1491(1)	9533(1)	7656(1)	50(1)
F(26)	2511(1)	9859(1)	7384(1)	46(1)
F(31)	460(10)	6768(8)	7810(6)	49(3)
F(32)	1268(8)	6174(9)	8148(5)	35(3)
F(33)	414(13)	5671(11)	7800(8)	68(4)
F(31A)	615(13)	6811(6)	7873(7)	61(3)
F(32A)	1335(8)	6032(13)	8109(5)	63(3)
F(33A)	290(10)	5797(11)	7766(8)	72(4)
F(34)	1267(1)	4540(1)	6412(1)	47(1)
F(35)	1006(1)	5266(1)	5880(1)	43(1)
F(36)	2119(1)	4961(1)	5979(1)	54(1)
F(41)	4795(2)	9034(1)	7208(1)	73(1)
F(42)	5610(1)	8386(1)	7509(1)	53(1)
F(43)	4878(1)	8916(1)	7963(1)	53(1)
F(44)	3489(6)	6448(4)	8558(3)	59(2)
F(45)	4051(9)	5800(4)	8082(3)	67(3)
F(46)	4639(6)	6469(8)	8509(5)	62(3)
F(44A)	3393(4)	6244(11)	8370(7)	94(5)
F(45A)	4317(10)	5850(6)	8033(6)	79(4)
F(46A)	4470(9)	6555(8)	8594(5)	63(4)
F(51)	3919(1)	8812(1)	5313(1)	42(1)
F(52)	3296(1)	8180(1)	4849(1)	56(1)
F(53)	4469(1)	8130(1)	4844(1)	57(1)
F(54)	4950(3)	5871(2)	5342(1)	64(2)
F(55)	5116(4)	5879(3)	6085(2)	103(3)
F(56)	4183(2)	5412(2)	5779(2)	76(2)
F(54A)	5360(5)	6041(5)	5650(6)	86(5)
F(55A)	4729(6)	5571(5)	6167(3)	50(3)
F(56A)	4422(9)	5507(6)	5463(5)	95(6)
C(21)	2279(2)	8016(1)	6665(1)	24(1)
C(22)	1771(2)	8040(2)	6288(1)	28(1)
C(23)	1313(2)	8589(2)	6214(1)	28(1)
C(24)	1336(2)	9143(2)	6516(1)	28(1)
C(25)	1819(2)	9126(1)	6896(1)	25(1)
C(26)	2279(2)	8573(1)	6967(1)	25(1)
C(27)	800(2)	8576(2)	5800(1)	40(1)
C(28)	1832(2)	9690(2)	7247(1)	31(1)
C(31)	2225(2)	6712(1)	6797(1)	24(1)
C(32)	1780(2)	6706(2)	7205(1)	27(1)
C(33)	1275(2)	6196(2)	7295(1)	29(1)
C(34)	1183(2)	5670(2)	6975(1)	29(1)
C(35)	1606(2)	5672(1)	6567(1)	26(1)
C(36)	2117(2)	6178(1)	6482(1)	24(1)
C(37)	860(2)	6204(2)	7755(1)	40(1)
C(38)	1507(2)	5117(2)	6213(1)	32(1)
C(41)	3376(2)	7398(1)	7164(1)	24(1)
C(42)	3867(2)	7949(2)	7186(1)	27(1)
C(43)	4398(2)	7999(2)	7535(1)	30(1)
C(44)	4476(2)	7500(2)	7882(1)	32(1)
C(45)	4006(2)	6954(2)	7866(1)	31(1)
C(46)	3469(2)	6904(1)	7511(1)	26(1)
C(47)	4915(2)	8578(2)	7550(1)	38(1)
C(48)	4047(2)	6417(2)	8237(1)	41(1)
C(51)	3318(2)	7251(1)	6249(1)	24(1)
C(52)	3397(2)	7743(1)	5896(1)	25(1)
C(53)	3865(2)	7656(2)	5507(1)	27(1)
C(54)	4267(2)	7071(2)	5453(1)	29(1)
C(55)	4210(2)	6581(1)	5806(1)	28(1)
C(56)	3752(2)	6674(1)	6193(1)	26(1)
C(57)	3884(2)	8189(2)	5133(1)	34(1)
C(58)	4640(2)	5944(2)	5762(1)	37(1)
B(21)	2795(2)	7345(2)	6719(1)	24(1)

Table S113. Bond lengths [Å] and angles [°] for **[(D$_{12}$-TMC)Co^{II}(I)]B$_{ArF}$**.

Co(1)-N(2B)	2.050(14)	C(8A)-C(9A)	1.50(2)
Co(1)-N(1B)	2.087(18)	C(8A)-H(8AA)	0.9900
Co(1)-N(1)	2.093(7)	C(8A)-H(8AC)	0.9900
Co(1)-N(1A)	2.11(2)	C(9A)-C(10A)	1.498(19)
Co(1)-N(4A)	2.11(3)	C(9A)-H(9AA)	0.9900
Co(1)-N(3)	2.160(4)	C(9A)-H(9AC)	0.9900
Co(1)-N(4)	2.213(6)	C(10A)-H(10C)	0.9900
Co(1)-N(2A)	2.22(2)	C(10A)-H(10D)	0.9900
Co(1)-N(3A)	2.26(2)	C(11A)-D(11D)	0.9800
Co(1)-N(4B)	2.26(2)	C(11A)-D(11E)	0.9800
Co(1)-N(2)	2.286(5)	C(11A)-D(11F)	0.9800
Co(1)-N(3B)	2.322(16)	C(12A)-C(13A)	1.497(19)
N(1)-C(1)	1.481(8)	C(12A)-H(12C)	0.9900
N(1)-C(13)	1.486(8)	C(12A)-H(12D)	0.9900
N(1)-C(14)	1.501(7)	C(13A)-H(13C)	0.9900
N(2)-C(3)	1.440(8)	C(13A)-H(13D)	0.9900
N(2)-C(5)	1.504(7)	C(14A)-D(14D)	0.9800
N(2)-C(4)	1.522(8)	C(14A)-D(14E)	0.9800
N(3)-C(7)	1.476(8)	C(14A)-D(14F)	0.9800
N(3)-C(8)	1.484(6)	N(1B)-C(1B)	1.481(16)
N(3)-C(6)	1.485(7)	N(1B)-C(13B)	1.504(16)
N(4)-C(11)	1.477(9)	N(1B)-C(14B)	1.516(16)
N(4)-C(10)	1.486(8)	N(2B)-C(3B)	1.455(16)
N(4)-C(12)	1.497(7)	N(2B)-C(4B)	1.474(19)
C(1)-C(2)	1.509(9)	N(2B)-C(5B)	1.541(16)
C(1)-H(1A)	0.9900	N(3B)-C(8B)	1.463(17)
C(1)-H(1AB)	0.9900	N(3B)-C(6B)	1.501(16)
C(2)-C(3)	1.537(8)	N(3B)-C(7B)	1.517(16)
C(2)-H(2A)	0.9900	N(4B)-C(11B)	1.455(19)
C(2)-H(2AB)	0.9900	N(4B)-C(10B)	1.469(18)
C(3)-H(3A)	0.9900	N(4B)-C(12B)	1.513(18)
C(3)-H(3AB)	0.9900	C(1B)-C(2B)	1.512(16)
C(4)-D(4A)	0.9800	C(1B)-H(1BA)	0.9900
C(4)-D(4B)	0.9800	C(1B)-H(1BB)	0.9900
C(4)-D(4C)	0.9800	C(2B)-C(3B)	1.528(17)
C(5)-C(6)	1.504(8)	C(2B)-H(2BA)	0.9900
C(5)-H(5A)	0.9900	C(2B)-H(2BB)	0.9900
C(5)-H(5AB)	0.9900	C(3B)-H(3BA)	0.9900
C(6)-H(6A)	0.9900	C(3B)-H(3BB)	0.9900
C(6)-H(6AB)	0.9900	C(4B)-D(4BA)	0.9800
C(7)-D(7A)	0.9800	C(4B)-D(4BB)	0.9800
C(7)-D(7B)	0.9800	C(4B)-D(4BC)	0.9800
C(7)-D(7C)	0.9800	C(5B)-C(6B)	1.510(17)
C(8)-C(9)	1.483(9)	C(5B)-H(5BA)	0.9900
C(8)-H(8A)	0.9900	C(5B)-H(5BB)	0.9900
C(8)-H(8AB)	0.9900	C(6B)-H(6BA)	0.9900
C(9)-C(10)	1.517(8)	C(6B)-H(6BB)	0.9900
C(9)-H(9A)	0.9900	C(7B)-D(7BA)	0.9800
C(9)-H(9AB)	0.9900	C(7B)-D(7BB)	0.9800
C(10)-H(10A)	0.9900	C(7B)-D(7BC)	0.9800
C(10)-H(10B)	0.9900	C(8B)-C(9B)	1.492(17)
C(11)-D(11A)	0.9800	C(8B)-H(8BA)	0.9900
C(11)-D(11B)	0.9800	C(8B)-H(8BB)	0.9900
C(11)-D(11C)	0.9800	C(9B)-C(10B)	1.496(16)
C(12)-C(13)	1.497(9)	C(9B)-H(9BA)	0.9900
C(12)-H(12A)	0.9900	C(9B)-H(9BB)	0.9900
C(12)-H(12B)	0.9900	C(10B)-H(10E)	0.9900
C(13)-H(13A)	0.9900	C(10B)-H(10F)	0.9900
C(13)-H(13B)	0.9900	C(11B)-D(11G)	0.9800
C(14)-D(14A)	0.9800	C(11B)-D(11H)	0.9800
C(14)-D(14B)	0.9800	C(11B)-D(11I)	0.9800
C(14)-D(14C)	0.9800	C(12B)-C(13B)	1.506(17)
N(1A)-C(1A)	1.474(18)	C(12B)-H(12E)	0.9900
N(1A)-C(13A)	1.499(18)	C(12B)-H(12F)	0.9900
N(1A)-C(14A)	1.510(18)	C(13B)-H(13E)	0.9900
N(2A)-C(3A)	1.446(18)	C(13B)-H(13F)	0.9900
N(2A)-C(4A)	1.48(2)	C(14B)-D(14G)	0.9800
N(2A)-C(5A)	1.530(18)	C(14B)-D(14H)	0.9800
N(3A)-C(8A)	1.469(18)	C(14B)-D(14I)	0.9800
N(3A)-C(6A)	1.506(18)	F(21)-C(27)	1.326(4)
N(3A)-C(7A)	1.516(18)	F(22)-C(27)	1.336(4)
N(4A)-C(11A)	1.45(2)	F(23)-C(27)	1.311(4)
N(4A)-C(10A)	1.47(2)	F(24)-C(28)	1.329(4)
N(4A)-C(12A)	1.504(19)	F(25)-C(28)	1.341(4)
C(1A)-C(2A)	1.505(19)	F(26)-C(28)	1.341(4)
C(1A)-H(1AA)	0.9900	F(31)-C(37)	1.344(10)
C(1A)-H(1AC)	0.9900	F(32)-C(37)	1.332(9)
C(2A)-C(3A)	1.518(19)	F(33)-C(37)	1.341(10)
C(2A)-H(2AA)	0.9900	F(31A)-C(37)	1.326(9)
C(2A)-H(2AC)	0.9900	F(32A)-C(37)	1.363(9)
C(3A)-H(3AA)	0.9900	F(33A)-C(37)	1.318(9)
C(3A)-H(3AC)	0.9900	F(34)-C(38)	1.348(4)
C(4A)-D(4AA)	0.9800	F(35)-C(38)	1.341(4)
C(4A)-D(4AB)	0.9800	F(36)-C(38)	1.333(4)
C(4A)-D(4AC)	0.9800	F(41)-C(47)	1.339(4)
C(5A)-C(6A)	1.497(19)	F(42)-C(47)	1.328(4)
C(5A)-H(5AA)	0.9900	F(43)-C(47)	1.342(4)
C(5A)-H(5AC)	0.9900	F(44)-C(48)	1.361(7)
C(6A)-H(6AA)	0.9900	F(45)-C(48)	1.301(8)
C(6A)-H(6AC)	0.9900	F(46)-C(48)	1.326(8)
C(7A)-D(7AA)	0.9800	F(44A)-C(48)	1.297(8)
C(7A)-D(7AB)	0.9800	F(45A)-C(48)	1.356(10)
C(7A)-D(7AC)	0.9800	F(46A)-C(48)	1.293(9)

F(51)-C(57)	1.339(4)
F(52)-C(57)	1.337(4)
F(53)-C(57)	1.345(4)
F(54)-C(58)	1.317(4)
F(55)-C(58)	1.263(5)
F(56)-C(58)	1.345(5)
F(54A)-C(58)	1.365(8)
F(55A)-C(58)	1.366(8)
F(56A)-C(58)	1.271(8)
C(21)-C(26)	1.393(4)
C(21)-C(22)	1.407(4)
C(21)-B(21)	1.639(4)
C(22)-C(23)	1.388(4)
C(22)-H(22)	0.9500
C(23)-C(24)	1.389(4)
C(23)-C(27)	1.494(4)
C(24)-C(25)	1.385(4)
C(24)-H(24)	0.9500
C(25)-C(26)	1.396(4)
C(25)-C(28)	1.493(4)
C(26)-H(26)	0.9500
C(31)-C(36)	1.396(4)
C(31)-C(32)	1.406(4)
C(31)-B(21)	1.644(4)
C(32)-C(33)	1.391(4)
C(32)-H(32)	0.9500
C(33)-C(34)	1.390(4)
C(33)-C(37)	1.499(4)
C(34)-C(35)	1.382(4)
C(34)-H(34)	0.9500
C(35)-C(36)	1.390(4)
C(35)-C(38)	1.496(4)
C(36)-H(36)	0.9500
C(41)-C(46)	1.392(4)
C(41)-C(42)	1.415(4)
C(41)-B(21)	1.644(4)
C(42)-C(43)	1.382(4)
C(42)-H(42)	0.9500
C(43)-C(44)	1.396(5)
C(43)-C(47)	1.488(4)
C(44)-C(45)	1.381(4)
C(44)-H(44)	0.9500
C(45)-C(46)	1.402(4)
C(45)-C(48)	1.493(5)
C(46)-H(46)	0.9500
C(51)-C(52)	1.401(4)
C(51)-C(56)	1.402(4)
C(51)-B(21)	1.639(4)
C(52)-C(53)	1.397(4)
C(52)-H(52)	0.9500
C(53)-C(54)	1.383(4)
C(53)-C(57)	1.492(4)
C(54)-C(55)	1.393(4)
C(54)-H(54)	0.9500
C(55)-C(56)	1.385(4)
C(55)-C(58)	1.492(4)
C(56)-H(56)	0.9500

N(2B)-Co(1)-N(1B)	96.2(6)
N(1A)-Co(1)-N(4A)	86.6(9)
N(1)-Co(1)-N(3)	134.3(2)
N(1)-Co(1)-N(4)	85.0(2)
N(3)-Co(1)-N(4)	90.09(17)
N(1A)-Co(1)-N(2A)	91.9(8)
N(4A)-Co(1)-N(2A)	144.3(11)
N(1A)-Co(1)-N(3A)	164.7(10)
N(4A)-Co(1)-N(3A)	90.8(8)
N(2A)-Co(1)-N(3A)	81.5(8)
N(2B)-Co(1)-N(4B)	138.8(7)
N(1B)-Co(1)-N(4B)	83.5(6)
N(1)-Co(1)-N(2)	91.6(2)
N(3)-Co(1)-N(2)	81.42(17)
N(4)-Co(1)-N(2)	164.5(2)
N(2B)-Co(1)-N(3B)	83.2(5)
N(1B)-Co(1)-N(3B)	164.7(6)
N(4B)-Co(1)-N(3B)	87.0(6)
C(1)-N(1)-C(13)	109.0(6)
C(1)-N(1)-C(14)	108.2(6)
C(13)-N(1)-C(14)	107.6(6)
C(1)-N(1)-Co(1)	115.6(4)
C(13)-N(1)-Co(1)	107.0(4)
C(14)-N(1)-Co(1)	109.2(7)
C(3)-N(2)-C(5)	110.6(5)
C(3)-N(2)-C(4)	108.3(5)
C(5)-N(2)-C(4)	105.1(5)
C(3)-N(2)-Co(1)	115.4(4)
C(5)-N(2)-Co(1)	102.7(3)
C(4)-N(2)-Co(1)	114.2(4)
C(7)-N(3)-C(8)	109.8(7)
C(7)-N(3)-C(6)	107.5(5)
C(8)-N(3)-C(6)	107.6(5)
C(7)-N(3)-Co(1)	107.8(6)
C(8)-N(3)-Co(1)	115.5(3)
C(6)-N(3)-Co(1)	108.4(3)
C(11)-N(4)-C(10)	108.9(6)
C(11)-N(4)-C(12)	106.6(5)
C(10)-N(4)-C(12)	108.4(5)
C(11)-N(4)-Co(1)	116.5(4)
C(10)-N(4)-Co(1)	115.1(4)
C(12)-N(4)-Co(1)	100.3(3)
N(1)-C(1)-C(2)	115.2(5)
N(1)-C(1)-H(1A)	108.5
C(2)-C(1)-H(1A)	108.5
N(1)-C(1)-H(1AB)	108.5
C(2)-C(1)-H(1AB)	108.5
H(1A)-C(1)-H(1AB)	107.5
C(1)-C(2)-C(3)	114.1(5)
C(1)-C(2)-H(2A)	108.7
C(3)-C(2)-H(2A)	108.7
C(1)-C(2)-H(2AB)	108.7
C(3)-C(2)-H(2AB)	108.7
H(2A)-C(2)-H(2AB)	107.6
N(2)-C(3)-C(2)	114.1(5)
N(2)-C(3)-H(3A)	108.7
C(2)-C(3)-H(3A)	108.7
N(2)-C(3)-H(3AB)	108.7
C(2)-C(3)-H(3AB)	108.7
H(3A)-C(3)-H(3AB)	107.6
N(2)-C(4)-D(4A)	109.5
N(2)-C(4)-D(4B)	109.5
D(4A)-C(4)-D(4B)	109.5
N(2)-C(4)-D(4C)	109.5
D(4A)-C(4)-D(4C)	109.5
D(4B)-C(4)-D(4C)	109.5
N(2)-C(5)-C(6)	109.3(5)
N(2)-C(5)-H(5A)	109.8
C(6)-C(5)-H(5A)	109.8
N(2)-C(5)-H(5AB)	109.8
C(6)-C(5)-H(5AB)	109.8
H(5A)-C(5)-H(5AB)	108.3
N(3)-C(6)-C(5)	111.6(4)
N(3)-C(6)-H(6A)	109.3
C(5)-C(6)-H(6A)	109.3
N(3)-C(6)-H(6AB)	109.3
C(5)-C(6)-H(6AB)	109.3
H(6A)-C(6)-H(6AB)	108.0
N(3)-C(7)-D(7A)	109.5
N(3)-C(7)-D(7B)	109.5
D(7A)-C(7)-D(7B)	109.5
N(3)-C(7)-D(7C)	109.5
D(7A)-C(7)-D(7C)	109.5
D(7B)-C(7)-D(7C)	109.5
C(9)-C(8)-N(3)	115.7(5)
C(9)-C(8)-H(8A)	108.4
N(3)-C(8)-H(8A)	108.4
C(9)-C(8)-H(8AB)	108.4
N(3)-C(8)-H(8AB)	108.4
H(8A)-C(8)-H(8AB)	107.4
C(8)-C(9)-C(10)	113.8(5)
C(8)-C(9)-H(9A)	108.8
C(10)-C(9)-H(9A)	108.8
C(8)-C(9)-H(9AB)	108.8
C(10)-C(9)-H(9AB)	108.8
H(9A)-C(9)-H(9AB)	107.7
N(4)-C(10)-C(9)	113.7(5)
N(4)-C(10)-H(10A)	108.8
C(9)-C(10)-H(10A)	108.8
N(4)-C(10)-H(10B)	108.8
C(9)-C(10)-H(10B)	108.8
H(10A)-C(10)-H(10B)	107.7
N(4)-C(11)-D(11A)	109.5
N(4)-C(11)-D(11B)	109.5
D(11A)-C(11)-D(11B)	109.5
N(4)-C(11)-D(11C)	109.5
D(11A)-C(11)-D(11C)	109.5
D(11B)-C(11)-D(11C)	109.5
N(4)-C(12)-C(13)	111.3(5)
N(4)-C(12)-H(12A)	109.4
C(13)-C(12)-H(12A)	109.4
N(4)-C(12)-H(12B)	109.4
C(13)-C(12)-H(12B)	109.4
H(12A)-C(12)-H(12B)	108.0
N(1)-C(13)-C(12)	111.2(5)
N(1)-C(13)-H(13A)	109.4
C(12)-C(13)-H(13A)	109.4
N(1)-C(13)-H(13B)	109.4
C(12)-C(13)-H(13B)	109.4
H(13A)-C(13)-H(13B)	108.0
N(1)-C(14)-D(14A)	109.5
N(1)-C(14)-D(14B)	109.5
D(14A)-C(14)-D(14B)	109.5
N(1)-C(14)-D(14C)	109.5
D(14A)-C(14)-D(14C)	109.5
D(14B)-C(14)-D(14C)	109.5
C(1A)-N(1A)-C(13A)	108.7(18)
C(1A)-N(1A)-C(14A)	106.8(19)
C(13A)-N(1A)-C(14A)	104.3(17)
C(1A)-N(1A)-Co(1)	115(2)
C(13A)-N(1A)-Co(1)	103.2(15)
C(14A)-N(1A)-Co(1)	118(2)

C(3A)-N(2A)-C(4A)	113(2)
C(3A)-N(2A)-C(5A)	107.0(17)
C(4A)-N(2A)-C(5A)	105.9(18)
C(3A)-N(2A)-Co(1)	110.0(19)
C(4A)-N(2A)-Co(1)	113(2)
C(5A)-N(2A)-Co(1)	107.4(14)
C(8A)-N(3A)-C(6A)	107.8(18)
C(8A)-N(3A)-C(7A)	108.7(19)
C(6A)-N(3A)-C(7A)	101.6(17)
C(8A)-N(3A)-Co(1)	114.0(18)
C(6A)-N(3A)-Co(1)	105.4(14)
C(7A)-N(3A)-Co(1)	118(2)
C(11A)-N(4A)-C(10A)	113(2)
C(11A)-N(4A)-C(12A)	110(2)
C(10A)-N(4A)-C(12A)	105.8(18)
C(11A)-N(4A)-Co(1)	112(2)
C(10A)-N(4A)-Co(1)	109(2)
C(12A)-N(4A)-Co(1)	105.5(16)
N(1A)-C(1A)-C(2A)	111(2)
N(1A)-C(1A)-H(1AA)	109.5
C(2A)-C(1A)-H(1AA)	109.5
N(1A)-C(1A)-H(1AC)	109.5
C(2A)-C(1A)-H(1AC)	109.5
H(1AA)-C(1A)-H(1AC)	108.0
C(1A)-C(2A)-C(3A)	115(2)
C(1A)-C(2A)-H(2AA)	108.6
C(3A)-C(2A)-H(2AA)	108.6
C(1A)-C(2A)-H(2AC)	108.6
C(3A)-C(2A)-H(2AC)	108.6
H(2AA)-C(2A)-H(2AC)	107.6
N(2A)-C(3A)-C(2A)	117(2)
N(2A)-C(3A)-H(3AA)	108.1
C(2A)-C(3A)-H(3AA)	108.1
N(2A)-C(3A)-H(3AC)	108.1
C(2A)-C(3A)-H(3AC)	108.1
H(3AA)-C(3A)-H(3AC)	107.3
N(2A)-C(4A)-D(4AA)	109.5
N(2A)-C(4A)-D(4AB)	109.5
D(4AA)-C(4A)-D(4AB)	109.5
N(2A)-C(4A)-D(4AC)	109.5
D(4AA)-C(4A)-D(4AC)	109.5
D(4AB)-C(4A)-D(4AC)	109.5
C(6A)-C(5A)-N(2A)	111.7(17)
C(6A)-C(5A)-H(5AA)	109.3
N(2A)-C(5A)-H(5AA)	109.3
C(6A)-C(5A)-H(5AC)	109.3
N(2A)-C(5A)-H(5AC)	109.3
H(5AA)-C(5A)-H(5AC)	107.9
C(5A)-C(6A)-N(3A)	111.4(18)
C(5A)-C(6A)-H(6AA)	109.3
N(3A)-C(6A)-H(6AA)	109.3
C(5A)-C(6A)-H(6AC)	109.3
N(3A)-C(6A)-H(6AC)	109.3
H(6AA)-C(6A)-H(6AC)	108.0
N(3A)-C(7A)-D(7AA)	109.5
N(3A)-C(7A)-D(7AB)	109.5
D(7AA)-C(7A)-D(7AB)	109.5
N(3A)-C(7A)-D(7AC)	109.5
D(7AA)-C(7A)-D(7AC)	109.5
D(7AB)-C(7A)-D(7AC)	109.5
N(3A)-C(8A)-C(9A)	111(2)
N(3A)-C(8A)-H(8AA)	109.4
C(9A)-C(8A)-H(8AA)	109.4
N(3A)-C(8A)-H(8AC)	109.4
C(9A)-C(8A)-H(8AC)	109.4
H(8AA)-C(8A)-H(8AC)	108.0
C(10A)-C(9A)-C(8A)	115(2)
C(10A)-C(9A)-H(9AA)	108.6
C(8A)-C(9A)-H(9AA)	108.6
C(10A)-C(9A)-H(9AC)	108.6
C(8A)-C(9A)-H(9AC)	108.6
H(9AA)-C(9A)-H(9AC)	107.6
N(4A)-C(10A)-C(9A)	116(2)
N(4A)-C(10A)-H(10C)	108.3
C(9A)-C(10A)-H(10C)	108.3
N(4A)-C(10A)-H(10D)	108.3
C(9A)-C(10A)-H(10D)	108.3
H(10C)-C(10A)-H(10D)	107.4
N(4A)-C(11A)-D(11D)	109.5
N(4A)-C(11A)-D(11E)	109.5
D(11D)-C(11A)-D(11E)	109.5
N(4A)-C(11A)-D(11F)	109.5
D(11D)-C(11A)-D(11F)	109.5
D(11E)-C(11A)-D(11F)	109.5
C(13A)-C(12A)-N(4A)	111.4(19)
C(13A)-C(12A)-H(12C)	109.4
N(4A)-C(12A)-H(12C)	109.4
C(13A)-C(12A)-H(12D)	109.4
N(4A)-C(12A)-H(12D)	109.4
H(12C)-C(12A)-H(12D)	108.0
C(12A)-C(13A)-N(1A)	112.2(19)
C(12A)-C(13A)-H(13C)	109.2
N(1A)-C(13A)-H(13C)	109.2
C(12A)-C(13A)-H(13D)	109.2
N(1A)-C(13A)-H(13D)	109.2
H(13C)-C(13A)-H(13D)	107.9
N(1A)-C(14A)-D(14D)	109.5
N(1A)-C(14A)-D(14E)	109.5
D(14D)-C(14A)-D(14E)	109.5
N(1A)-C(14A)-D(14F)	109.5
D(14D)-C(14A)-D(14F)	109.5
D(14E)-C(14A)-D(14F)	109.5
C(1B)-N(1B)-C(13B)	107.2(14)
C(1B)-N(1B)-C(14B)	105.8(13)
C(13B)-N(1B)-C(14B)	104.6(14)
C(1B)-N(1B)-Co(1)	113.6(12)
C(13B)-N(1B)-Co(1)	107.7(10)
C(14B)-N(1B)-Co(1)	117.1(13)
C(3B)-N(2B)-C(4B)	111.9(18)
C(3B)-N(2B)-C(5B)	105.5(12)
C(4B)-N(2B)-C(5B)	106.1(16)
C(3B)-N(2B)-Co(1)	112.7(11)
C(4B)-N(2B)-Co(1)	111(2)
C(5B)-N(2B)-Co(1)	108.9(10)
C(8B)-N(3B)-C(6B)	111.1(14)
C(8B)-N(3B)-C(7B)	107.4(14)
C(6B)-N(3B)-C(7B)	104.8(13)
C(8B)-N(3B)-Co(1)	113.7(11)
C(6B)-N(3B)-Co(1)	101.6(10)
C(7B)-N(3B)-Co(1)	117.8(11)
C(11B)-N(4B)-C(10B)	110(2)
C(11B)-N(4B)-C(12B)	109(2)
C(10B)-N(4B)-C(12B)	105.7(16)
C(11B)-N(4B)-Co(1)	116(3)
C(10B)-N(4B)-Co(1)	112.7(14)
C(12B)-N(4B)-Co(1)	103.1(12)
N(1B)-C(1B)-C(2B)	113.0(14)
N(1B)-C(1B)-H(1BA)	109.0
C(2B)-C(1B)-H(1BA)	109.0
N(1B)-C(1B)-H(1BB)	109.0
C(2B)-C(1B)-H(1BB)	109.0
H(1BA)-C(1B)-H(1BB)	107.8
C(1B)-C(2B)-C(3B)	112.8(14)
C(1B)-C(2B)-H(2BA)	109.0
C(3B)-C(2B)-H(2BA)	109.0
C(1B)-C(2B)-H(2BB)	109.0
C(3B)-C(2B)-H(2BB)	109.0
H(2BA)-C(2B)-H(2BB)	107.8
N(2B)-C(3B)-C(2B)	113.7(15)
N(2B)-C(3B)-H(3BA)	108.8
C(2B)-C(3B)-H(3BA)	108.8
N(2B)-C(3B)-H(3BB)	108.8
C(2B)-C(3B)-H(3BB)	108.8
H(3BA)-C(3B)-H(3BB)	107.7
N(2B)-C(4B)-D(4BA)	109.5
N(2B)-C(4B)-D(4BB)	109.5
D(4BA)-C(4B)-D(4BB)	109.5
N(2B)-C(4B)-D(4BC)	109.5
D(4BA)-C(4B)-D(4BC)	109.5
D(4BB)-C(4B)-D(4BC)	109.5
C(6B)-C(5B)-N(2B)	111.5(13)
C(6B)-C(5B)-H(5BA)	109.3
N(2B)-C(5B)-H(5BA)	109.3
C(6B)-C(5B)-H(5BB)	109.3
N(2B)-C(5B)-H(5BB)	109.3
H(5BA)-C(5B)-H(5BB)	108.0
N(3B)-C(6B)-C(5B)	108.3(13)
N(3B)-C(6B)-H(6BA)	110.0
C(5B)-C(6B)-H(6BA)	110.0
N(3B)-C(6B)-H(6BB)	110.0
C(5B)-C(6B)-H(6BB)	110.0
H(6BA)-C(6B)-H(6BB)	108.4
N(3B)-C(7B)-D(7BA)	109.5
N(3B)-C(7B)-D(7BB)	109.5
D(7BA)-C(7B)-D(7BB)	109.5
N(3B)-C(7B)-D(7BC)	109.5
D(7BA)-C(7B)-D(7BC)	109.5
D(7BB)-C(7B)-D(7BC)	109.5
N(3B)-C(8B)-C(9B)	114.2(15)
N(3B)-C(8B)-H(8BA)	108.7
C(9B)-C(8B)-H(8BA)	108.7
N(3B)-C(8B)-H(8BB)	108.7
C(9B)-C(8B)-H(8BB)	108.7
H(8BA)-C(8B)-H(8BB)	107.6
C(8B)-C(9B)-C(10B)	115.0(15)
C(8B)-C(9B)-H(9BA)	108.5
C(10B)-C(9B)-H(9BA)	108.5
C(8B)-C(9B)-H(9BB)	108.5
C(10B)-C(9B)-H(9BB)	108.5
H(9BA)-C(9B)-H(9BB)	107.5
N(4B)-C(10B)-C(9B)	112.1(14)
N(4B)-C(10B)-H(10E)	109.2
C(9B)-C(10B)-H(10E)	109.2
N(4B)-C(10B)-H(10F)	109.2
C(9B)-C(10B)-H(10F)	109.2
H(10E)-C(10B)-H(10F)	107.9
N(4B)-C(11B)-D(11G)	109.5
N(4B)-C(11B)-D(11H)	109.5
D(11G)-C(11B)-D(11H)	109.5
N(4B)-C(11B)-D(11I)	109.5

D(11G)-C(11B)-D(11I)	109.5	F(35)-C(38)-F(34)	104.8(2)
D(11H)-C(11B)-D(11I)	109.5	F(36)-C(38)-C(35)	113.4(2)
C(13B)-C(12B)-N(4B)	111.5(14)	F(35)-C(38)-C(35)	112.7(3)
C(13B)-C(12B)-H(12E)	109.3	F(34)-C(38)-C(35)	112.9(3)
N(4B)-C(12B)-H(12E)	109.3	C(46)-C(41)-C(42)	115.9(3)
C(13B)-C(12B)-H(12F)	109.3	C(46)-C(41)-B(21)	124.5(2)
N(4B)-C(12B)-H(12F)	109.3	C(42)-C(41)-B(21)	119.4(3)
H(12E)-C(12B)-H(12F)	108.0	C(43)-C(42)-C(41)	121.9(3)
N(1B)-C(13B)-C(12B)	109.7(13)	C(43)-C(42)-H(42)	119.0
N(1B)-C(13B)-H(13E)	109.7	C(41)-C(42)-H(42)	119.0
C(12B)-C(13B)-H(13E)	109.7	C(42)-C(43)-C(44)	121.1(3)
N(1B)-C(13B)-H(13F)	109.7	C(42)-C(43)-C(47)	121.3(3)
C(12B)-C(13B)-H(13F)	109.7	C(44)-C(43)-C(47)	117.6(3)
H(13E)-C(13B)-H(13F)	108.2	C(45)-C(44)-C(43)	118.1(3)
N(1B)-C(14B)-D(14G)	109.5	C(45)-C(44)-H(44)	121.0
N(1B)-C(14B)-D(14H)	109.5	C(43)-C(44)-H(44)	121.0
D(14G)-C(14B)-D(14H)	109.5	C(44)-C(45)-C(46)	120.8(3)
N(1B)-C(14B)-D(14I)	109.5	C(44)-C(45)-C(48)	120.4(3)
D(14G)-C(14B)-D(14I)	109.5	C(46)-C(45)-C(48)	118.8(3)
D(14H)-C(14B)-D(14I)	109.5	C(41)-C(46)-C(45)	122.2(3)
C(26)-C(21)-C(22)	115.6(3)	C(41)-C(46)-H(46)	118.9
C(26)-C(21)-B(21)	126.0(3)	C(45)-C(46)-H(46)	118.9
C(22)-C(21)-B(21)	118.4(2)	F(42)-C(47)-F(41)	106.7(3)
C(23)-C(22)-C(21)	122.3(3)	F(42)-C(47)-F(43)	105.5(3)
C(23)-C(22)-H(22)	118.8	F(41)-C(47)-F(43)	105.9(3)
C(21)-C(22)-H(22)	118.8	F(42)-C(47)-C(43)	112.5(3)
C(22)-C(23)-C(24)	120.7(3)	F(41)-C(47)-C(43)	113.4(3)
C(22)-C(23)-C(27)	118.6(3)	F(43)-C(47)-C(43)	112.3(3)
C(24)-C(23)-C(27)	120.6(3)	F(46A)-C(48)-F(44A)	112.5(9)
C(25)-C(24)-C(23)	118.1(3)	F(45)-C(48)-F(46)	105.2(8)
C(25)-C(24)-H(24)	120.9	F(46A)-C(48)-F(45A)	106.7(9)
C(23)-C(24)-H(24)	120.9	F(44A)-C(48)-F(45A)	103.6(9)
C(24)-C(25)-C(26)	120.7(3)	F(45)-C(48)-F(44)	105.5(6)
C(24)-C(25)-C(28)	120.1(3)	F(46)-C(48)-F(44)	103.0(7)
C(26)-C(25)-C(28)	119.1(3)	F(46A)-C(48)-C(45)	114.8(7)
C(21)-C(26)-C(25)	122.4(3)	F(44A)-C(48)-C(45)	110.1(5)
C(21)-C(26)-H(26)	118.8	F(45)-C(48)-C(45)	116.0(5)
C(25)-C(26)-H(26)	118.8	F(46)-C(48)-C(45)	112.8(7)
F(23)-C(27)-F(21)	105.8(3)	F(45A)-C(48)-C(45)	108.4(8)
F(23)-C(27)-F(22)	106.9(3)	F(44)-C(48)-C(45)	113.1(4)
F(21)-C(27)-F(22)	104.3(3)	C(52)-C(51)-C(56)	115.6(3)
F(23)-C(27)-C(23)	113.2(3)	C(52)-C(51)-B(21)	123.5(3)
F(21)-C(27)-C(23)	112.8(3)	C(56)-C(51)-B(21)	120.8(3)
F(22)-C(27)-C(23)	113.2(3)	C(53)-C(52)-C(51)	122.1(3)
F(24)-C(28)-F(26)	106.0(2)	C(53)-C(52)-H(52)	119.0
F(24)-C(28)-F(25)	106.9(3)	C(51)-C(52)-H(52)	119.0
F(26)-C(28)-F(25)	103.9(3)	C(54)-C(53)-C(52)	121.0(3)
F(24)-C(28)-C(25)	113.6(3)	C(54)-C(53)-C(57)	120.3(3)
F(26)-C(28)-C(25)	113.0(3)	C(52)-C(53)-C(57)	118.6(3)
F(25)-C(28)-C(25)	112.6(2)	C(53)-C(54)-C(55)	117.9(3)
C(36)-C(31)-C(32)	115.4(3)	C(53)-C(54)-H(54)	121.0
C(36)-C(31)-B(21)	125.8(3)	C(55)-C(54)-H(54)	121.0
C(32)-C(31)-B(21)	118.7(3)	C(56)-C(55)-C(54)	120.8(3)
C(33)-C(32)-C(31)	122.5(3)	C(56)-C(55)-C(58)	119.6(3)
C(33)-C(32)-H(32)	118.8	C(54)-C(55)-C(58)	119.6(3)
C(31)-C(32)-H(32)	118.8	C(55)-C(56)-C(51)	122.6(3)
C(34)-C(33)-C(32)	120.7(3)	C(55)-C(56)-H(56)	118.7
C(34)-C(33)-C(37)	120.4(3)	C(51)-C(56)-H(56)	118.7
C(32)-C(33)-C(37)	118.9(3)	F(52)-C(57)-F(51)	106.0(3)
C(35)-C(34)-C(33)	117.8(3)	F(52)-C(57)-F(53)	106.0(3)
C(35)-C(34)-H(34)	121.1	F(51)-C(57)-F(53)	105.7(3)
C(33)-C(34)-H(34)	121.1	F(52)-C(57)-C(53)	113.1(3)
C(34)-C(35)-C(36)	121.3(3)	F(51)-C(57)-C(53)	112.9(3)
C(34)-C(35)-C(38)	118.8(3)	F(53)-C(57)-C(53)	112.5(3)
C(36)-C(35)-C(38)	119.9(3)	F(55)-C(58)-F(54)	109.6(4)
C(35)-C(36)-C(31)	122.3(3)	F(55)-C(58)-F(56)	108.7(5)
C(35)-C(36)-H(36)	118.9	F(54)-C(58)-F(56)	102.2(4)
C(31)-C(36)-H(36)	118.9	F(56A)-C(58)-F(54A)	104.1(9)
F(33A)-C(37)-F(31A)	106.5(10)	F(56A)-C(58)-F(55A)	102.7(8)
F(32)-C(37)-F(33)	103.1(12)	F(54A)-C(58)-F(55A)	98.8(7)
F(32)-C(37)-F(31)	104.2(9)	F(55)-C(58)-C(55)	112.9(3)
F(33)-C(37)-F(31)	108.4(12)	F(56A)-C(58)-C(55)	117.9(5)
F(33A)-C(37)-F(32A)	109.3(11)	F(54)-C(58)-C(55)	113.1(3)
F(31A)-C(37)-F(32A)	105.0(8)	F(56)-C(58)-C(55)	109.6(3)
F(33A)-C(37)-C(33)	114.4(9)	F(54A)-C(58)-C(55)	113.9(4)
F(31A)-C(37)-C(33)	113.4(7)	F(55A)-C(58)-C(55)	116.9(4)
F(32)-C(37)-C(33)	115.5(8)	C(21)-B(21)-C(51)	110.6(2)
F(33)-C(37)-C(33)	112.3(10)	C(21)-B(21)-C(41)	112.9(2)
F(31)-C(37)-C(33)	112.5(9)	C(51)-B(21)-C(41)	104.1(2)
F(32A)-C(37)-C(33)	107.8(8)	C(21)-B(21)-C(31)	105.6(2)
F(36)-C(38)-F(35)	106.2(3)	C(51)-B(21)-C(31)	112.9(2)
F(36)-C(38)-F(34)	106.2(3)	C(41)-B(21)-C(31)	110.9(2)

Table S114. Anisotropic displacement parameters ($Å^2$x 10^3) for **[(D$_{12}$-TMC)Co^{II}(I)]B$_{ArF}$**.
The anisotropic displacement factor exponent takes the form: $-2p^2[h^2 a^{*2}U^{11} + ... + 2 h k a^* b^* U^{12}]$

	U^{11}	U^{22}	U^{33}	U^{23}	U^{13}	U^{12}
Co(1)	23(1)	28(1)	19(1)	4(1)	1(1)	-2(1)
I(1)	62(1)	79(1)	21(1)	4(1)	7(1)	-35(1)
N(1)	31(3)	42(2)	18(2)	2(2)	-3(2)	-15(2)
N(2)	26(2)	38(3)	58(3)	-22(2)	-2(2)	10(2)
N(3)	24(2)	40(2)	23(2)	4(2)	-4(2)	-7(2)
N(4)	37(2)	30(2)	74(3)	-5(2)	-14(2)	2(2)
C(1)	30(2)	80(3)	28(3)	5(3)	1(2)	-22(3)
C(2)	27(3)	92(4)	53(3)	-21(3)	12(2)	-6(3)
C(3)	32(2)	87(4)	68(3)	-37(3)	9(2)	2(2)
C(4)	48(4)	46(3)	105(5)	-4(3)	-25(3)	16(3)
C(5)	37(2)	46(3)	71(3)	-33(2)	-3(2)	4(2)
C(6)	41(3)	58(3)	38(3)	-22(2)	2(2)	-14(2)
C(7)	31(4)	42(4)	42(4)	-1(3)	6(3)	-12(3)
C(8)	54(3)	58(3)	44(3)	25(2)	-31(2)	-18(2)
C(9)	60(3)	47(3)	92(4)	30(3)	-46(3)	-9(2)
C(10)	61(3)	36(3)	99(4)	18(3)	-38(3)	-1(2)
C(11)	43(3)	57(4)	123(6)	-44(4)	4(4)	8(3)
C(12)	50(3)	29(3)	83(4)	-9(3)	-19(3)	-3(2)
C(13)	48(3)	40(3)	58(4)	12(3)	-12(3)	-16(2)
C(14)	27(4)	53(4)	26(3)	-1(3)	-8(2)	-13(3)
I(1A)	52(1)	31(3)	20(2)	0(2)	10(2)	-7(3)
N(1A)	32(6)	51(6)	43(6)	-15(6)	-1(6)	0(6)
N(2A)	34(6)	44(7)	36(6)	4(6)	-10(6)	-2(6)
N(3A)	40(6)	36(6)	63(7)	-2(6)	-10(6)	4(6)
N(4A)	35(6)	47(6)	32(6)	0(6)	-1(6)	-8(6)
C(1A)	36(7)	51(8)	43(6)	-15(7)	-2(6)	1(7)
C(2A)	42(7)	45(8)	40(7)	-11(7)	-5(6)	-6(6)
C(3A)	37(6)	46(7)	37(6)	1(7)	-8(6)	-10(6)
C(4A)	31(13)	47(11)	39(13)	13(12)	-14(11)	1(11)
C(5A)	35(7)	45(7)	52(7)	8(6)	-13(6)	-2(6)
C(6A)	36(7)	42(8)	62(8)	2(7)	-12(7)	6(7)
C(7A)	39(15)	30(14)	65(8)	-11(9)	3(10)	7(13)
C(8A)	45(6)	37(7)	65(7)	-2(6)	-9(7)	3(6)
C(9A)	48(6)	37(7)	55(8)	-7(7)	-10(7)	-5(6)
C(10A)	41(6)	38(6)	45(7)	-1(6)	-8(6)	-12(6)
C(11A)	35(14)	51(16)	30(8)	1(9)	2(9)	-23(12)
C(12A)	32(7)	60(6)	35(7)	-5(7)	-4(7)	-8(6)
C(13A)	30(6)	62(6)	43(7)	-12(7)	2(6)	-4(6)
C(14A)	31(13)	53(9)	49(12)	-12(10)	5(12)	5(9)
I(1B)	52(1)	31(3)	20(2)	0(2)	10(2)	-7(3)
N(1B)	33(5)	46(6)	43(5)	-12(5)	-5(5)	0(5)
N(2B)	32(5)	48(6)	42(5)	9(5)	-10(5)	-8(5)
N(3B)	38(5)	24(5)	49(6)	-5(5)	-5(5)	13(4)
N(4B)	22(6)	44(5)	32(6)	-2(5)	-5(5)	-7(5)
C(1B)	34(5)	46(6)	40(5)	-11(5)	0(5)	-2(5)
C(2B)	41(6)	46(6)	37(6)	-3(6)	-9(6)	-14(5)
C(3B)	37(6)	50(7)	37(5)	7(6)	-17(5)	-6(5)
C(4B)	22(11)	56(10)	50(12)	24(11)	-18(9)	-4(9)
C(5B)	37(6)	52(6)	66(6)	8(5)	-13(6)	-3(5)
C(6B)	44(6)	44(7)	69(6)	5(6)	-10(6)	8(6)
C(7B)	63(11)	41(10)	48(7)	-17(7)	5(7)	13(9)
C(8B)	47(5)	38(6)	59(6)	-1(5)	-3(6)	3(5)
C(9B)	49(6)	40(6)	44(7)	-5(6)	0(6)	-3(6)
C(10B)	37(6)	45(5)	35(6)	-3(5)	-1(5)	-10(5)
C(11B)	33(14)	56(14)	30(8)	-6(8)	-7(8)	-5(12)
C(12B)	30(6)	57(6)	42(6)	-13(6)	-1(5)	-3(5)
C(13B)	29(5)	55(6)	45(6)	-17(6)	3(5)	5(5)
C(14B)	67(11)	43(8)	43(9)	-18(7)	4(10)	-2(8)
F(21)	83(2)	48(1)	114(2)	-5(1)	-69(2)	-7(1)
F(22)	48(1)	55(1)	75(2)	-10(1)	-27(1)	17(1)
F(23)	54(2)	188(3)	34(1)	10(2)	-11(1)	10(2)
F(24)	95(2)	29(1)	56(1)	-8(1)	-13(1)	17(1)
F(25)	66(1)	42(1)	41(1)	-14(1)	28(1)	-18(1)
F(26)	44(1)	42(1)	51(1)	-16(1)	7(1)	-16(1)
F(31)	34(5)	69(5)	44(4)	8(4)	1(4)	21(4)
F(32)	44(5)	32(5)	29(4)	3(3)	1(3)	6(3)
F(33)	83(9)	76(6)	44(5)	-15(5)	26(6)	-53(7)
F(31A)	73(8)	62(4)	49(6)	-10(3)	34(5)	-1(4)
F(32A)	90(5)	75(9)	25(3)	7(4)	10(3)	3(4)
F(33A)	53(4)	92(7)	69(7)	-23(5)	30(4)	-43(5)
F(34)	71(1)	29(1)	41(1)	1(1)	-9(1)	-20(1)
F(35)	51(1)	47(1)	32(1)	-1(1)	-16(1)	-14(1)
F(36)	44(1)	47(1)	71(2)	-30(1)	8(1)	-12(1)
F(41)	80(2)	60(2)	80(2)	26(1)	-43(1)	-45(1)
F(42)	26(1)	61(1)	72(2)	-17(1)	7(1)	-16(1)
F(43)	42(1)	52(1)	64(1)	-29(1)	0(1)	-15(1)
F(44)	67(4)	57(4)	51(4)	25(3)	23(3)	25(3)
F(45)	139(9)	31(3)	31(3)	1(2)	-11(4)	1(4)
F(46)	53(4)	79(5)	56(5)	22(4)	-25(4)	2(3)
F(44A)	42(3)	127(10)	115(9)	94(7)	-20(4)	-17(4)
F(45A)	98(8)	40(4)	99(8)	3(4)	-38(5)	22(5)
F(46A)	97(8)	56(5)	38(4)	13(3)	-22(5)	-33(6)
F(51)	60(1)	26(1)	40(1)	-1(1)	3(1)	-10(1)
F(52)	71(2)	42(1)	55(1)	16(1)	-28(1)	-13(1)
F(53)	74(2)	52(1)	44(1)	14(1)	30(1)	11(1)
F(54)	88(4)	48(2)	56(2)	6(2)	39(2)	31(2)
F(55)	115(5)	108(5)	85(4)	-51(4)	-66(4)	86(4)
F(56)	63(2)	26(2)	139(5)	4(2)	44(3)	6(2)
F(54A)	66(5)	49(5)	143(13)	29(6)	60(7)	29(4)
F(55A)	64(6)	38(5)	47(4)	13(3)	16(4)	27(4)

F(56A)	131(11)	62(7)	92(9)	-46(7)	-67(8)	52(7)
C(21)	20(1)	26(1)	25(1)	-1(1)	3(1)	-5(1)
C(22)	24(1)	28(2)	30(2)	-5(1)	1(1)	-3(1)
C(23)	22(1)	32(2)	31(2)	1(1)	0(1)	-3(1)
C(24)	25(1)	28(2)	31(2)	4(1)	6(1)	-1(1)
C(25)	25(1)	25(1)	26(1)	-1(1)	9(1)	-6(1)
C(26)	23(1)	28(1)	23(1)	0(1)	2(1)	-7(1)
C(27)	34(2)	42(2)	44(2)	-2(2)	-10(2)	3(1)
C(28)	34(2)	25(2)	35(2)	-1(1)	6(1)	-5(1)
C(31)	21(1)	27(1)	25(1)	3(1)	-5(1)	-2(1)
C(32)	26(1)	26(2)	28(2)	-1(1)	-3(1)	-2(1)
C(33)	25(1)	35(2)	27(1)	1(1)	-2(1)	-5(1)
C(34)	26(1)	27(2)	34(2)	5(1)	-7(1)	-8(1)
C(35)	26(1)	26(1)	27(1)	2(1)	-8(1)	-2(1)
C(36)	26(1)	24(1)	22(1)	2(1)	-4(1)	-4(1)
C(37)	43(2)	42(2)	36(2)	-1(2)	5(1)	-13(1)
C(38)	35(2)	30(2)	32(2)	-1(1)	-6(1)	-10(1)
C(41)	21(1)	24(1)	26(1)	-6(1)	3(1)	-1(1)
C(42)	25(1)	29(2)	26(1)	-4(1)	2(1)	-5(1)
C(43)	24(1)	34(2)	33(2)	-6(1)	-1(1)	-4(1)
C(44)	26(1)	38(2)	31(2)	-4(1)	-6(1)	0(1)
C(45)	30(2)	32(2)	31(2)	-1(1)	-1(1)	4(1)
C(46)	25(1)	22(1)	33(2)	-4(1)	0(1)	-1(1)
C(47)	32(2)	42(2)	39(2)	-3(1)	-9(1)	-12(1)
C(48)	41(2)	38(2)	44(2)	6(2)	-10(1)	1(1)
C(51)	23(1)	24(1)	24(1)	-5(1)	-3(1)	-6(1)
C(52)	24(1)	24(1)	28(1)	-5(1)	-2(1)	-2(1)
C(53)	27(1)	29(2)	25(1)	-4(1)	-2(1)	-6(1)
C(54)	29(2)	31(2)	27(2)	-4(1)	3(1)	-3(1)
C(55)	28(1)	25(1)	31(2)	-4(1)	1(1)	-1(1)
C(56)	28(1)	24(1)	26(1)	-1(1)	-3(1)	-4(1)
C(57)	42(2)	30(2)	31(2)	-2(1)	1(1)	-4(1)
C(58)	43(2)	32(2)	36(2)	-2(1)	8(1)	7(1)
B(21)	22(2)	24(2)	27(2)	-4(1)	-2(1)	-3(1)

Table S115. Hydrogen coordinates (x 10^4) and isotropic displacement parameters ($Å^2$x 10^3) for **[(D$_{12}$-TMC)Co^{II}(I)]B$_{Ar}$F**.

	x	y	z	U(eq)
H(1A)	4794	4567	8858	55
H(1AB)	4114	4333	8541	55
H(2A)	4839	3430	9169	69
H(2AB)	5092	3519	8628	69
H(3A)	3879	3153	8385	75
H(3AB)	4360	2578	8633	75
H(5A)	2615	2281	9069	62
H(5AB)	3140	2100	8629	62
H(6A)	2773	3088	8232	55
H(6AB)	2071	2634	8349	55
H(8A)	2323	4128	8212	63
H(8AB)	1542	3768	8179	63
H(9A)	1153	4463	8825	80
H(9AB)	1294	4847	8333	80
H(10A)	2490	5156	8632	78
H(10B)	1818	5513	8896	78
H(12A)	3040	5185	9815	65
H(12B)	2803	5742	9432	65
H(13A)	3555	5243	8858	58
H(13B)	4032	5429	9318	58
H(1AA)	3922	2861	8257	52
H(1AC)	3616	3617	8262	52
H(2AA)	2700	2483	8439	51
H(2AC)	2794	2795	7919	51
H(3AA)	2314	3840	8233	48
H(3AC)	1737	3241	8181	48
H(5AA)	1485	4368	8612	53
H(5AC)	893	3837	8801	53
H(6AA)	1201	4067	9585	56
H(6AC)	1007	4762	9324	56
H(8AA)	1857	5630	9310	59
H(8AC)	2135	5208	8858	59
H(9AA)	3002	6024	9148	56
H(9AC)	3080	5581	9620	56
H(10C)	3409	5028	8718	50
H(10D)	4012	5441	9008	50
H(12C)	4321	4358	8660	51
H(12D)	4868	4391	9105	51
H(13C)	4515	3332	9356	54
H(13D)	4738	3280	8806	54
H(1BA)	3624	2684	8261	48
H(1BB)	3563	3488	8263	48
H(2BA)	2330	2646	8451	50
H(2BB)	2515	2910	7927	50
H(3BA)	2419	4055	8248	50
H(3BB)	1663	3655	8194	50
H(5BA)	1788	4772	8664	62
H(5BB)	1051	4388	8812	62
H(6BA)	1358	5200	9395	63
H(6BB)	1385	4459	9624	63
H(8BA)	2474	5856	9375	57
H(8BB)	2599	5398	8914	57
H(9BA)	3651	5474	9651	53
H(9BB)	3661	5982	9211	53
H(10E)	3763	4979	8709	47
H(10F)	4479	5209	8996	47
H(12E)	4363	4056	8600	51
H(12F)	4989	4004	8998	51
H(13E)	4575	2919	8773	51
H(13F)	4423	3051	9328	51
H(22)	1742	7668	6076	33
H(24)	1030	9522	6463	34
H(26)	2605	8576	7230	30
H(32)	1826	7063	7428	32
H(34)	839	5319	7034	35
H(36)	2403	6158	6200	29
H(42)	3832	8295	6953	32
H(44)	4842	7534	8121	38
H(46)	3158	6521	7507	32
H(52)	3125	8149	5922	30
H(54)	4573	7005	5183	35
H(56)	3732	6333	6430	31

$[(D_{12}\text{-TMC})Co^{II}(NO_3)]B_{Ar}F$

The structure of **$[(D_{12}\text{-TMC})Co^{II}(NO_3)]B_{Ar}F$** was solved in the triclinic space group $P\bar{1}$ and the asymmetric unit contains one full molecule (anion and cation). Three of the CF_3 groups of the anion were found to be disordered and were modelled over three positions each. The disorder was refined with the help of 1,2 and 1,3-distance restraints, similarity restraints on anisotropic displacement parameters and advanced rigid bond restraints. To allow for full anisotropic refinement a few atoms were set to have the same anisotropic displacement parameters. All of the disorder ratios were allowed to refine freely and converged to 0.464(2):0.371(3):0.164(2), 0.645(3):0.249(3):0.106(3) and 0.630(3):0.105(2):0.265(3).

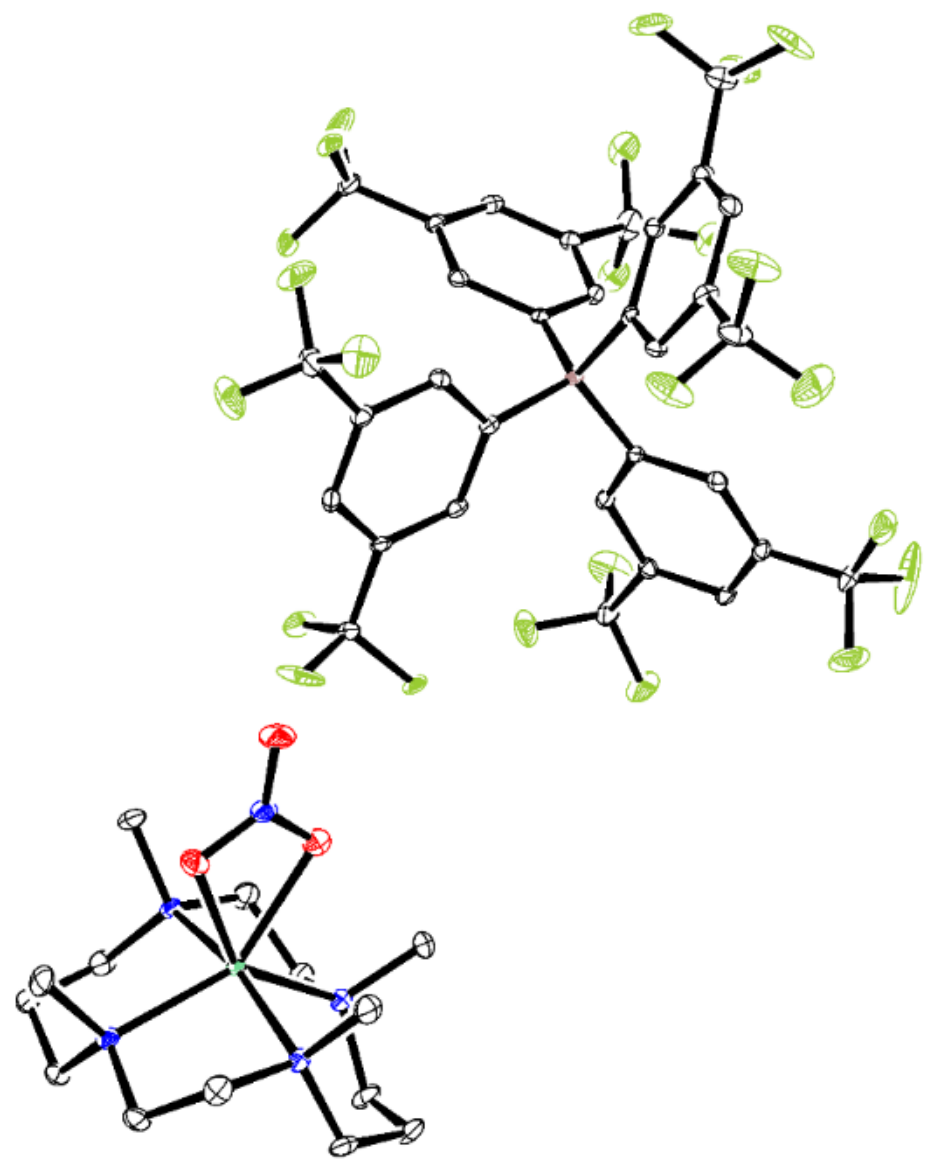

Figure S23. Molecular structure of **$[(D_{12}\text{-TMC})Co^{II}(NO_3)]B_{Ar}F$**.

Table S116. Crystal data and structure refinement for **$[(D_{12}\text{-TMC})Co^{II}(NO_3)]B_{Ar}F$**.

CCDC No	2062721	
Empirical formula	$C_{46}H_{32}BCoD_{12}F_{24}N_5O_3$	
Formula weight	1252.67	
Temperature	100(2) K	
Wavelength	0.71073 Å	
Crystal system	Triclinic	
Space group	$P\bar{1}$	
Unit cell dimensions	a = 11.8760(8) Å	α = 84.732(2)°
	b = 13.5042(8) Å	β = 75.856(2)°
	c = 17.7906(11) Å	γ = 64.880(2)°
Volume	2504.8(3) Å^3	
Z	2	
Density (calculated)	1.661 Mg/m^3	
Absorption coefficient	0.479 mm^{-1}	
F(000)	1254	
Crystal size	0.980 x 0.510 x 0.052 mm^3	
Theta range for data collection	2.361 to 29.129°.	
Index ranges	-16 ≤ h ≤ 16, -18 ≤ k ≤ 18, -24 ≤ l ≤ 24	
Reflections collected	91232	
Independent reflections	13440 [R(int) = 0.1075]	
Completeness to theta = 25.242°	99.3 %	
Absorption correction	Semi-empirical from equivalents	
Refinement method	Full-matrix least-squares on F^2	
Data / restraints / parameters	13440 / 3795 / 818	
Goodness-of-fit on F^2	1.032	
Final R indices [I>2σ(I)]	R1 = 0.0545, wR2 = 0.1137	
R indices (all data)	R1 = 0.0827, wR2 = 0.1248	
Largest diff. peak and hole	0.836 and -0.758 eÅ^{-3}	

Table S117. Atomic coordinates (x 10^4) and equivalent isotropic displacement parameters (Å^{2}x 10^3) for **[(D$_{12}$-TMC)Co^{II}(NO$_3$)]B$_{Ar}$F**. U(eq) is defined as one third of the trace of the orthogonalized U^{ij} tensor.

	x	y	z	U(eq)
Co(1)	6657(1)	616(1)	6248(1)	8(1)
O(1)	8001(2)	-25(2)	5113(1)	20(1)
O(2)	6011(2)	913(2)	5159(1)	21(1)
O(3)	7328(2)	300(2)	4042(1)	27(1)
N(1)	8395(2)	-136(2)	6710(1)	11(1)
N(2)	6673(2)	2229(2)	6245(1)	11(1)
N(3)	4631(2)	1508(2)	6808(1)	12(1)
N(4)	6536(2)	-942(2)	6562(1)	11(1)
N(5)	7120(2)	394(2)	4744(1)	16(1)
C(1)	8420(2)	565(2)	7302(1)	14(1)
C(2)	8421(2)	1650(2)	6996(1)	15(1)
C(3)	7134(2)	2486(2)	6876(1)	16(1)
C(4)	7400(2)	2441(2)	5490(1)	17(1)
D(4A)	7097	2287	5068	25
D(4B)	8311	1967	5432	25
D(4C)	7271	3208	5471	25
C(5)	5298(2)	2993(2)	6333(2)	17(1)
C(6)	4474(2)	2626(2)	6979(2)	16(1)
C(7)	3752(2)	1639(2)	6295(2)	19(1)
D(7A)	3893	2096	5853	28
D(7B)	2865	1989	6589	28
D(7C)	3921	919	6106	28
C(8)	4219(2)	1018(2)	7549(1)	16(1)
C(9)	4315(2)	-126(2)	7461(1)	17(1)
C(10)	5670(2)	-1001(2)	7312(1)	15(1)
C(11)	6210(2)	-1348(2)	5936(1)	17(1)
D(11A)	6768	-1311	5438	26
D(11B)	5317	-894	5920	26
D(11C)	6328	-2107	6038	26
C(12)	7867(2)	-1686(2)	6609(1)	16(1)
C(13)	8363(2)	-1154(2)	7087(1)	14(1)
C(14)	9609(2)	-452(2)	6105(1)	17(1)
D(14A)	9697	-1034	5774	26
D(14B)	10332	-715	6355	26
D(14C)	9595	185	5789	26
F(21)	-1268(2)	6443(1)	5123(1)	28(1)
F(22)	541(2)	6485(2)	4615(1)	29(1)
F(23)	-1142(2)	7674(1)	4296(1)	34(1)
F(24)	-1447(2)	3251(2)	3893(1)	40(1)
F(25)	-2850(2)	4623(2)	3457(2)	56(1)
F(26)	-1341(2)	3382(1)	2685(1)	28(1)
F(31)	3767(2)	832(1)	966(1)	30(1)
F(32)	1861(2)	1139(1)	933(1)	37(1)
F(33)	3300(2)	844(1)	-128(1)	32(1)
F(34)	890(2)	5751(1)	-913(1)	26(1)
F(35)	1468(2)	4192(1)	-1446(1)	37(1)
F(36)	2863(2)	4790(2)	-1421(1)	33(1)
F(41)	7000(2)	1705(1)	1018(1)	28(1)
F(42)	7149(2)	3213(1)	675(1)	28(1)
F(43)	8036(1)	2272(2)	1576(1)	36(1)
F(44)	4709(6)	4688(4)	4208(4)	24(1)
F(45)	5594(5)	2939(4)	4220(4)	32(2)
F(46)	3562(6)	3793(6)	4484(5)	20(2)
F(44A)	4307(16)	4838(10)	4242(14)	33(4)
F(45A)	5775(12)	3209(12)	4173(11)	27(3)
F(46A)	3810(15)	3498(16)	4523(13)	22(4)
F(44B)	5190(20)	4405(17)	4178(15)	24(1)
F(45B)	5150(20)	2841(13)	4351(13)	24(5)
F(46B)	3414(13)	4293(18)	4452(12)	35(5)
F(51)	4695(9)	7632(7)	1292(4)	22(1)
F(52)	3950(9)	8591(4)	351(4)	28(2)
F(53)	5053(5)	6857(4)	203(2)	19(1)
F(51A)	5017(13)	7084(13)	1171(8)	22(1)
F(52A)	3922(14)	8683(9)	900(11)	28(2)
F(53A)	4764(13)	7413(14)	14(6)	19(1)
F(51B)	4510(20)	7925(17)	1282(11)	31(4)
F(52B)	4070(20)	8394(11)	171(8)	28(3)
F(53B)	5159(13)	6729(11)	375(7)	19(1)
F(54)	-1052(4)	9566(3)	1819(3)	25(1)
F(55)	-1439(5)	8484(5)	1197(3)	25(1)
F(56)	-581(3)	9569(4)	584(3)	25(1)
F(54A)	-546(4)	9807(4)	854(3)	25(1)
F(55A)	-1203(4)	9265(4)	1971(3)	25(1)
F(56A)	-1319(6)	8572(5)	982(3)	25(1)
F(54B)	-762(9)	9843(7)	1422(7)	25(1)
F(55B)	-1538(10)	8666(10)	1398(7)	25(1)
F(56B)	-572(9)	9113(9)	373(5)	25(1)
C(21)	916(2)	5074(2)	2535(1)	8(1)
C(22)	590(2)	5799(2)	3149(1)	10(1)
C(23)	-354(2)	5880(2)	3810(1)	10(1)
C(24)	-1072(2)	5281(2)	3873(1)	12(1)
C(25)	-810(2)	4597(2)	3258(1)	11(1)
C(26)	167(2)	4482(2)	2609(1)	10(1)
C(27)	-563(2)	6616(2)	4458(1)	15(1)
C(28)	-1608(2)	3973(2)	3317(1)	15(1)
C(31)	2189(2)	4262(2)	1082(1)	9(1)
C(32)	2501(2)	3135(2)	1114(1)	10(1)
C(33)	2535(2)	2549(2)	498(1)	12(1)
C(34)	2272(2)	3065(2)	-190(1)	12(1)
C(35)	1975(2)	4172(2)	-241(1)	12(1)

C(36)	1937(2)	4757(2)	379(1)	10(1)
C(37)	2870(3)	1351(2)	560(1)	19(1)
C(38)	1791(2)	4728(2)	-996(1)	18(1)
C(41)	3461(2)	4268(2)	2149(1)	8(1)
C(42)	4643(2)	3714(2)	1633(1)	10(1)
C(43)	5779(2)	3201(2)	1883(1)	11(1)
C(44)	5801(2)	3204(2)	2657(1)	11(1)
C(45)	4645(2)	3747(2)	3177(1)	9(1)
C(46)	3509(2)	4254(2)	2931(1)	10(1)
C(47)	6994(2)	2600(2)	1294(1)	18(1)
C(48)	4626(2)	3802(2)	4014(1)	12(1)
C(51)	2041(2)	6152(2)	1534(1)	8(1)
C(52)	3097(2)	6413(2)	1280(1)	10(1)
C(53)	2977(2)	7436(2)	986(1)	10(1)
C(54)	1798(2)	8254(2)	940(1)	12(1)
C(55)	733(2)	8020(2)	1196(1)	11(1)
C(56)	860(2)	6997(2)	1482(1)	11(1)
C(57)	4157(2)	7635(2)	711(1)	14(1)
C(58)	-558(2)	8899(2)	1186(2)	22(1)
B(1)	2152(2)	4931(2)	1822(1)	8(1)

Table S118. Bond lengths [Å] and angles [°] for **[(D$_{12}$-TMC)Co^{II}(NO$_3$)]B$_{Ar}$F**.

Co(1)-N(4)	2.1814(19)	F(55)-C(58)	1.376(6)
Co(1)-N(2)	2.1852(19)	F(56)-C(58)	1.336(4)
Co(1)-N(3)	2.1962(19)	F(54A)-C(58)	1.316(5)
Co(1)-O(2)	2.1975(18)	F(55A)-C(58)	1.436(5)
Co(1)-N(1)	2.204(2)	F(56A)-C(58)	1.292(6)
Co(1)-O(1)	2.2237(17)	F(54B)-C(58)	1.283(8)
O(1)-N(5)	1.269(3)	F(55B)-C(58)	1.292(9)
O(2)-N(5)	1.267(3)	F(56B)-C(58)	1.453(8)
O(3)-N(5)	1.221(3)	C(21)-C(22)	1.404(3)
N(1)-C(13)	1.485(3)	C(21)-C(26)	1.404(3)
N(1)-C(14)	1.490(3)	C(21)-B(1)	1.642(3)
N(1)-C(1)	1.494(3)	C(22)-C(23)	1.387(3)
N(2)-C(4)	1.484(3)	C(22)-H(22)	0.9500
N(2)-C(3)	1.488(3)	C(23)-C(24)	1.384(3)
N(2)-C(5)	1.494(3)	C(23)-C(27)	1.498(3)
N(3)-C(8)	1.488(3)	C(24)-C(25)	1.388(3)
N(3)-C(6)	1.491(3)	C(24)-H(24)	0.9500
N(3)-C(7)	1.494(3)	C(25)-C(26)	1.389(3)
N(4)-C(11)	1.482(3)	C(25)-C(28)	1.494(3)
N(4)-C(12)	1.488(3)	C(26)-H(26)	0.9500
N(4)-C(10)	1.491(3)	C(31)-C(36)	1.400(3)
C(1)-C(2)	1.516(3)	C(31)-C(32)	1.404(3)
C(1)-H(1A)	0.9900	C(31)-B(1)	1.646(3)
C(1)-H(1AB)	0.9900	C(32)-C(33)	1.393(3)
C(2)-C(3)	1.519(4)	C(32)-H(32)	0.9500
C(2)-H(2A)	0.9900	C(33)-C(34)	1.387(3)
C(2)-H(2AB)	0.9900	C(33)-C(37)	1.494(3)
C(3)-H(3A)	0.9900	C(34)-C(35)	1.382(3)
C(3)-H(3AB)	0.9900	C(34)-H(34)	0.9500
C(4)-D(4A)	0.9800	C(35)-C(36)	1.396(3)
C(4)-D(4B)	0.9800	C(35)-C(38)	1.496(3)
C(4)-D(4C)	0.9800	C(36)-H(36)	0.9500
C(5)-C(6)	1.512(3)	C(41)-C(46)	1.404(3)
C(5)-H(5A)	0.9900	C(41)-C(42)	1.406(3)
C(5)-H(5AB)	0.9900	C(41)-B(1)	1.650(3)
C(6)-H(6A)	0.9900	C(42)-C(43)	1.393(3)
C(6)-H(6AB)	0.9900	C(42)-H(42)	0.9500
C(7)-D(7A)	0.9800	C(43)-C(44)	1.385(3)
C(7)-D(7B)	0.9800	C(43)-C(47)	1.504(3)
C(7)-D(7C)	0.9800	C(44)-C(45)	1.388(3)
C(8)-C(9)	1.521(3)	C(44)-H(44)	0.9500
C(8)-H(8A)	0.9900	C(45)-C(46)	1.390(3)
C(8)-H(8AB)	0.9900	C(45)-C(48)	1.492(3)
C(9)-C(10)	1.514(3)	C(46)-H(46)	0.9500
C(9)-H(9A)	0.9900	C(51)-C(56)	1.401(3)
C(9)-H(9AB)	0.9900	C(51)-C(52)	1.404(3)
C(10)-H(10A)	0.9900	C(51)-B(1)	1.640(3)
C(10)-H(10B)	0.9900	C(52)-C(53)	1.392(3)
C(11)-D(11A)	0.9800	C(52)-H(52)	0.9500
C(11)-D(11B)	0.9800	C(53)-C(54)	1.383(3)
C(11)-D(11C)	0.9800	C(53)-C(57)	1.495(3)
C(12)-C(13)	1.517(3)	C(54)-C(55)	1.392(3)
C(12)-H(12A)	0.9900	C(54)-H(54)	0.9500
C(12)-H(12B)	0.9900	C(55)-C(56)	1.387(3)
C(13)-H(13A)	0.9900	C(55)-C(58)	1.492(3)
C(13)-H(13B)	0.9900	C(56)-H(56)	0.9500
C(14)-D(14A)	0.9800		
C(14)-D(14B)	0.9800	N(4)-Co(1)-N(2)	165.77(7)
C(14)-D(14C)	0.9800	N(4)-Co(1)-N(3)	90.61(7)
F(21)-C(27)	1.335(3)	N(2)-Co(1)-N(3)	83.25(7)
F(22)-C(27)	1.342(3)	N(4)-Co(1)-O(2)	99.60(7)
F(23)-C(27)	1.336(3)	N(2)-Co(1)-O(2)	92.74(7)
F(24)-C(28)	1.338(3)	N(3)-Co(1)-O(2)	85.55(7)
F(25)-C(28)	1.331(3)	N(4)-Co(1)-N(1)	83.30(7)
F(26)-C(28)	1.333(3)	N(2)-Co(1)-N(1)	91.35(7)
F(31)-C(37)	1.340(3)	N(3)-Co(1)-N(1)	132.38(7)
F(32)-C(37)	1.351(3)	O(2)-Co(1)-N(1)	142.06(7)
F(33)-C(37)	1.333(3)	N(4)-Co(1)-O(1)	93.25(7)
F(34)-C(38)	1.334(3)	N(2)-Co(1)-O(1)	99.35(7)
F(35)-C(38)	1.342(3)	N(3)-Co(1)-O(1)	143.38(7)
F(36)-C(38)	1.345(3)	O(2)-Co(1)-O(1)	57.89(7)
F(41)-C(47)	1.343(3)	N(1)-Co(1)-O(1)	84.22(7)
F(42)-C(47)	1.340(3)	N(5)-O(1)-Co(1)	92.90(13)
F(43)-C(47)	1.332(3)	N(5)-O(2)-Co(1)	94.18(13)
F(44)-C(48)	1.323(5)	C(13)-N(1)-C(14)	107.59(18)
F(45)-C(48)	1.343(5)	C(13)-N(1)-C(1)	108.60(17)
F(46)-C(48)	1.338(5)	C(14)-N(1)-C(1)	108.10(17)
F(44A)-C(48)	1.358(10)	C(13)-N(1)-Co(1)	104.99(13)
F(45A)-C(48)	1.342(10)	C(14)-N(1)-Co(1)	114.16(14)
F(46A)-C(48)	1.330(11)	C(1)-N(1)-Co(1)	113.12(14)
F(44B)-C(48)	1.342(11)	C(4)-N(2)-C(3)	108.41(17)
F(45B)-C(48)	1.335(11)	C(4)-N(2)-C(5)	108.38(18)
F(46B)-C(48)	1.362(11)	C(3)-N(2)-C(5)	107.69(18)
F(51)-C(57)	1.340(5)	C(4)-N(2)-Co(1)	111.20(14)
F(52)-C(57)	1.338(5)	C(3)-N(2)-Co(1)	117.43(14)
F(53)-C(57)	1.344(5)	C(5)-N(2)-Co(1)	103.27(13)
F(51A)-C(57)	1.385(10)	C(8)-N(3)-C(6)	108.32(18)
F(52A)-C(57)	1.379(10)	C(8)-N(3)-C(7)	108.47(18)
F(53A)-C(57)	1.263(9)	C(6)-N(3)-C(7)	107.16(19)
F(51B)-C(57)	1.335(11)	C(8)-N(3)-Co(1)	113.59(14)
F(52B)-C(57)	1.327(11)	C(6)-N(3)-Co(1)	105.60(13)
F(53B)-C(57)	1.349(10)	C(7)-N(3)-Co(1)	113.36(15)
F(54)-C(58)	1.355(5)	C(11)-N(4)-C(12)	108.39(18)

C(11)-N(4)-C(10)	108.34(17)
C(12)-N(4)-C(10)	107.79(18)
C(11)-N(4)-Co(1)	110.66(14)
C(12)-N(4)-Co(1)	102.98(13)
C(10)-N(4)-Co(1)	118.20(14)
O(3)-N(5)-O(2)	122.6(2)
O(3)-N(5)-O(1)	122.4(2)
O(2)-N(5)-O(1)	115.03(19)
N(1)-C(1)-C(2)	114.03(18)
N(1)-C(1)-H(1A)	108.7
C(2)-C(1)-H(1A)	108.7
N(1)-C(1)-H(1AB)	108.7
C(2)-C(1)-H(1AB)	108.7
H(1A)-C(1)-H(1AB)	107.6
C(1)-C(2)-C(3)	114.05(19)
C(1)-C(2)-H(2A)	108.7
C(3)-C(2)-H(2A)	108.7
C(1)-C(2)-H(2AB)	108.7
C(3)-C(2)-H(2AB)	108.7
H(2A)-C(2)-H(2AB)	107.6
N(2)-C(3)-C(2)	115.85(19)
N(2)-C(3)-H(3A)	108.3
C(2)-C(3)-H(3A)	108.3
N(2)-C(3)-H(3AB)	108.3
C(2)-C(3)-H(3AB)	108.3
H(3A)-C(3)-H(3AB)	107.4
N(2)-C(4)-D(4A)	109.5
N(2)-C(4)-D(4B)	109.5
D(4A)-C(4)-D(4B)	109.5
N(2)-C(4)-D(4C)	109.5
D(4A)-C(4)-D(4C)	109.5
D(4B)-C(4)-D(4C)	109.5
N(2)-C(5)-C(6)	110.42(19)
N(2)-C(5)-H(5A)	109.6
C(6)-C(5)-H(5A)	109.6
N(2)-C(5)-H(5AB)	109.6
C(6)-C(5)-H(5AB)	109.6
H(5A)-C(5)-H(5AB)	108.1
N(3)-C(6)-C(5)	110.34(19)
N(3)-C(6)-H(6A)	109.6
C(5)-C(6)-H(6A)	109.6
N(3)-C(6)-H(6AB)	109.6
C(5)-C(6)-H(6AB)	109.6
H(6A)-C(6)-H(6AB)	108.1
N(3)-C(7)-D(7A)	109.5
N(3)-C(7)-D(7B)	109.5
D(7A)-C(7)-D(7B)	109.5
N(3)-C(7)-D(7C)	109.5
D(7A)-C(7)-D(7C)	109.5
D(7B)-C(7)-D(7C)	109.5
N(3)-C(8)-C(9)	114.17(19)
N(3)-C(8)-H(8A)	108.7
C(9)-C(8)-H(8A)	108.7
N(3)-C(8)-H(8AB)	108.7
C(9)-C(8)-H(8AB)	108.7
H(8A)-C(8)-H(8AB)	107.6
C(10)-C(9)-C(8)	113.64(19)
C(10)-C(9)-H(9A)	108.8
C(8)-C(9)-H(9A)	108.8
C(10)-C(9)-H(9AB)	108.8
C(8)-C(9)-H(9AB)	108.8
H(9A)-C(9)-H(9AB)	107.7
N(4)-C(10)-C(9)	116.4(2)
N(4)-C(10)-H(10A)	108.2
C(9)-C(10)-H(10A)	108.2
N(4)-C(10)-H(10B)	108.2
C(9)-C(10)-H(10B)	108.2
H(10A)-C(10)-H(10B)	107.3
N(4)-C(11)-D(11A)	109.5
N(4)-C(11)-D(11B)	109.5
D(11A)-C(11)-D(11B)	109.5
N(4)-C(11)-D(11C)	109.5
D(11A)-C(11)-D(11C)	109.5
D(11B)-C(11)-D(11C)	109.5
N(4)-C(12)-C(13)	110.13(19)
N(4)-C(12)-H(12A)	109.6
C(13)-C(12)-H(12A)	109.6
N(4)-C(12)-H(12B)	109.6
C(13)-C(12)-H(12B)	109.6
H(12A)-C(12)-H(12B)	108.1
N(1)-C(13)-C(12)	109.94(19)
N(1)-C(13)-H(13A)	109.7
C(12)-C(13)-H(13A)	109.7
N(1)-C(13)-H(13B)	109.7
C(12)-C(13)-H(13B)	109.7
H(13A)-C(13)-H(13B)	108.2
N(1)-C(14)-D(14A)	109.5
N(1)-C(14)-D(14B)	109.5
D(14A)-C(14)-D(14B)	109.5
N(1)-C(14)-D(14C)	109.5
D(14A)-C(14)-D(14C)	109.5
D(14B)-C(14)-D(14C)	109.5
C(22)-C(21)-C(26)	115.57(19)
C(22)-C(21)-B(1)	119.14(18)
C(26)-C(21)-B(1)	125.21(19)
C(23)-C(22)-C(21)	122.5(2)
C(23)-C(22)-H(22)	118.7
C(21)-C(22)-H(22)	118.7
C(24)-C(23)-C(22)	120.8(2)
C(24)-C(23)-C(27)	120.7(2)
C(22)-C(23)-C(27)	118.47(19)
C(23)-C(24)-C(25)	117.7(2)
C(23)-C(24)-H(24)	121.1
C(25)-C(24)-H(24)	121.1
C(24)-C(25)-C(26)	121.5(2)
C(24)-C(25)-C(28)	117.74(19)
C(26)-C(25)-C(28)	120.7(2)
C(25)-C(26)-C(21)	121.7(2)
C(25)-C(26)-H(26)	119.2
C(21)-C(26)-H(26)	119.2
F(21)-C(27)-F(23)	106.88(19)
F(21)-C(27)-F(22)	106.1(2)
F(23)-C(27)-F(22)	105.69(19)
F(21)-C(27)-C(23)	113.13(19)
F(23)-C(27)-C(23)	112.6(2)
F(22)-C(27)-C(23)	111.95(19)
F(25)-C(28)-F(26)	106.8(2)
F(25)-C(28)-F(24)	106.3(2)
F(26)-C(28)-F(24)	104.90(19)
F(25)-C(28)-C(25)	112.3(2)
F(26)-C(28)-C(25)	113.91(19)
F(24)-C(28)-C(25)	112.1(2)
C(36)-C(31)-C(32)	115.3(2)
C(36)-C(31)-B(1)	123.2(2)
C(32)-C(31)-B(1)	121.46(19)
C(33)-C(32)-C(31)	122.7(2)
C(33)-C(32)-H(32)	118.7
C(31)-C(32)-H(32)	118.7
C(34)-C(33)-C(32)	120.6(2)
C(34)-C(33)-C(37)	118.6(2)
C(32)-C(33)-C(37)	120.7(2)
C(35)-C(34)-C(33)	117.9(2)
C(35)-C(34)-H(34)	121.0
C(33)-C(34)-H(34)	121.0
C(34)-C(35)-C(36)	121.3(2)
C(34)-C(35)-C(38)	118.9(2)
C(36)-C(35)-C(38)	119.6(2)
C(35)-C(36)-C(31)	122.1(2)
C(35)-C(36)-H(36)	118.9
C(31)-C(36)-H(36)	118.9
F(33)-C(37)-F(31)	106.8(2)
F(33)-C(37)-F(32)	106.5(2)
F(31)-C(37)-F(32)	105.0(2)
F(33)-C(37)-C(33)	113.0(2)
F(31)-C(37)-C(33)	112.7(2)
F(32)-C(37)-C(33)	112.3(2)
F(34)-C(38)-F(35)	106.9(2)
F(34)-C(38)-F(36)	106.2(2)
F(35)-C(38)-F(36)	105.6(2)
F(34)-C(38)-C(35)	113.21(19)
F(35)-C(38)-C(35)	112.4(2)
F(36)-C(38)-C(35)	112.1(2)
C(46)-C(41)-C(42)	115.2(2)
C(46)-C(41)-B(1)	124.15(18)
C(42)-C(41)-B(1)	120.55(19)
C(43)-C(42)-C(41)	122.1(2)
C(43)-C(42)-H(42)	118.9
C(41)-C(42)-H(42)	118.9
C(44)-C(43)-C(42)	121.5(2)
C(44)-C(43)-C(47)	120.0(2)
C(42)-C(43)-C(47)	118.5(2)
C(43)-C(44)-C(45)	117.4(2)
C(43)-C(44)-H(44)	121.3
C(45)-C(44)-H(44)	121.3
C(44)-C(45)-C(46)	121.2(2)
C(44)-C(45)-C(48)	119.3(2)
C(46)-C(45)-C(48)	119.43(19)
C(45)-C(46)-C(41)	122.5(2)
C(45)-C(46)-H(46)	118.7
C(41)-C(46)-H(46)	118.7
F(43)-C(47)-F(42)	106.9(2)
F(43)-C(47)-F(41)	107.0(2)
F(42)-C(47)-F(41)	105.83(19)
F(43)-C(47)-C(43)	113.1(2)
F(42)-C(47)-C(43)	112.1(2)
F(41)-C(47)-C(43)	111.48(19)
F(44)-C(48)-F(46)	106.1(4)
F(45B)-C(48)-F(44B)	105.2(13)
F(46A)-C(48)-F(45A)	105.9(10)
F(44)-C(48)-F(45)	106.7(4)
F(46)-C(48)-F(45)	105.5(4)
F(46A)-C(48)-F(44A)	103.8(12)
F(45A)-C(48)-F(44A)	104.9(10)
F(45B)-C(48)-F(46B)	104.9(12)
F(44B)-C(48)-F(46B)	102.8(12)
F(44)-C(48)-C(45)	113.6(4)
F(46A)-C(48)-C(45)	117.1(12)
F(45B)-C(48)-C(45)	115.6(11)
F(46)-C(48)-C(45)	112.7(4)
F(44B)-C(48)-C(45)	115.1(11)

F(45A)-C(48)-C(45)	112.6(9)
F(45)-C(48)-C(45)	111.6(3)
F(44A)-C(48)-C(45)	111.6(10)
F(46B)-C(48)-C(45)	111.9(11)
C(56)-C(51)-C(52)	115.3(2)
C(56)-C(51)-B(1)	120.85(18)
C(52)-C(51)-B(1)	123.7(2)
C(53)-C(52)-C(51)	122.2(2)
C(53)-C(52)-H(52)	118.9
C(51)-C(52)-H(52)	118.9
C(54)-C(53)-C(52)	121.2(2)
C(54)-C(53)-C(57)	120.2(2)
C(52)-C(53)-C(57)	118.6(2)
C(53)-C(54)-C(55)	117.8(2)
C(53)-C(54)-H(54)	121.1
C(55)-C(54)-H(54)	121.1
C(56)-C(55)-C(54)	120.7(2)
C(56)-C(55)-C(58)	120.1(2)
C(54)-C(55)-C(58)	119.1(2)
C(55)-C(56)-C(51)	122.8(2)
C(55)-C(56)-H(56)	118.6
C(51)-C(56)-H(56)	118.6
F(52B)-C(57)-F(51B)	107.1(12)
F(52)-C(57)-F(51)	106.6(5)
F(52)-C(57)-F(53)	106.3(4)
F(51)-C(57)-F(53)	106.3(4)
F(52B)-C(57)-F(53B)	104.4(10)
F(51B)-C(57)-F(53B)	106.4(11)
F(53A)-C(57)-F(52A)	110.2(10)
F(53A)-C(57)-F(51A)	107.2(9)
F(52A)-C(57)-F(51A)	97.5(9)
F(53A)-C(57)-C(53)	118.9(7)
F(52B)-C(57)-C(53)	114.0(11)
F(51B)-C(57)-C(53)	113.0(11)
F(52)-C(57)-C(53)	113.3(5)
F(51)-C(57)-C(53)	112.3(5)
F(53)-C(57)-C(53)	111.5(3)
F(53B)-C(57)-C(53)	111.3(8)
F(52A)-C(57)-C(53)	110.7(6)
F(51A)-C(57)-C(53)	110.2(7)
F(54B)-C(58)-F(55B)	113.8(7)
F(56A)-C(58)-F(54A)	114.3(4)
F(56)-C(58)-F(54)	104.7(3)
F(56)-C(58)-F(55)	107.8(3)
F(54)-C(58)-F(55)	104.8(3)
F(56A)-C(58)-F(55A)	102.3(4)
F(54A)-C(58)-F(55A)	101.5(3)
F(54B)-C(58)-F(56B)	100.7(6)
F(55B)-C(58)-F(56B)	99.0(7)
F(54B)-C(58)-C(55)	115.2(5)
F(55B)-C(58)-C(55)	118.8(6)
F(56A)-C(58)-C(55)	114.6(3)
F(54A)-C(58)-C(55)	114.3(3)
F(56)-C(58)-C(55)	114.2(2)
F(54)-C(58)-C(55)	112.4(3)
F(55)-C(58)-C(55)	112.3(3)
F(55A)-C(58)-C(55)	108.0(3)
F(56B)-C(58)-C(55)	105.6(4)
C(51)-B(1)-C(21)	108.40(17)
C(51)-B(1)-C(31)	108.62(17)
C(21)-B(1)-C(31)	111.37(17)
C(51)-B(1)-C(41)	109.96(17)
C(21)-B(1)-C(41)	108.36(17)
C(31)-B(1)-C(41)	110.12(18)

Table S119. Anisotropic displacement parameters ($Å^2$x 10^3) for **[(D_{12}-TMC)Co^{II}(NO_3)]$B_{Ar}F$**.
The anisotropic displacement factor exponent takes the form: $-2p^2[h^2 a^{*2}U^{11} + ... + 2 h k a^* b^* U^{12}]$

	U^{11}	U^{22}	U^{33}	U^{23}	U^{13}	U^{12}
Co(1)	8(1)	11(1)	6(1)	1(1)	-1(1)	-5(1)
O(1)	19(1)	30(1)	12(1)	0(1)	-4(1)	-12(1)
O(2)	18(1)	32(1)	15(1)	3(1)	-2(1)	-12(1)
O(3)	39(1)	43(1)	7(1)	0(1)	-4(1)	-26(1)
N(1)	11(1)	14(1)	7(1)	-1(1)	1(1)	-6(1)
N(2)	12(1)	14(1)	9(1)	2(1)	-1(1)	-8(1)
N(3)	11(1)	14(1)	12(1)	2(1)	-2(1)	-6(1)
N(4)	11(1)	12(1)	12(1)	0(1)	-1(1)	-6(1)
N(5)	24(1)	24(1)	9(1)	1(1)	-4(1)	-17(1)
C(1)	16(1)	22(1)	7(1)	-1(1)	-4(1)	-10(1)
C(2)	18(1)	22(1)	10(1)	-1(1)	-4(1)	-13(1)
C(3)	22(1)	16(1)	13(1)	-2(1)	-2(1)	-12(1)
C(4)	21(1)	21(1)	12(1)	6(1)	-2(1)	-14(1)
C(5)	16(1)	12(1)	22(1)	5(1)	-5(1)	-6(1)
C(6)	12(1)	12(1)	21(1)	-1(1)	-1(1)	-3(1)
C(7)	12(1)	26(1)	20(1)	8(1)	-9(1)	-9(1)
C(8)	13(1)	19(1)	13(1)	1(1)	4(1)	-7(1)
C(9)	16(1)	20(1)	16(1)	3(1)	1(1)	-12(1)
C(10)	20(1)	15(1)	12(1)	5(1)	-2(1)	-10(1)
C(11)	23(1)	19(1)	15(1)	-2(1)	-4(1)	-14(1)
C(12)	15(1)	11(1)	20(1)	0(1)	-2(1)	-4(1)
C(13)	14(1)	13(1)	15(1)	5(1)	-6(1)	-3(1)
C(14)	9(1)	24(1)	16(1)	-3(1)	2(1)	-6(1)
F(21)	32(1)	44(1)	12(1)	-13(1)	11(1)	-25(1)
F(22)	21(1)	50(1)	21(1)	-15(1)	-2(1)	-18(1)
F(23)	50(1)	16(1)	29(1)	-9(1)	-11(1)	-5(1)
F(24)	73(1)	47(1)	24(1)	17(1)	-14(1)	-50(1)
F(25)	9(1)	26(1)	128(2)	-22(1)	-2(1)	-7(1)
F(26)	36(1)	41(1)	22(1)	-6(1)	-1(1)	-31(1)
F(31)	50(1)	13(1)	25(1)	1(1)	-22(1)	-4(1)
F(32)	48(1)	27(1)	44(1)	4(1)	-4(1)	-28(1)
F(33)	62(1)	14(1)	17(1)	-4(1)	-14(1)	-10(1)
F(34)	33(1)	20(1)	18(1)	5(1)	-12(1)	-3(1)
F(35)	70(1)	29(1)	24(1)	5(1)	-33(1)	-22(1)
F(36)	30(1)	46(1)	14(1)	11(1)	1(1)	-13(1)
F(41)	23(1)	24(1)	30(1)	-15(1)	3(1)	-4(1)
F(42)	22(1)	36(1)	18(1)	-2(1)	9(1)	-11(1)
F(43)	8(1)	61(1)	26(1)	-15(1)	-3(1)	-1(1)
F(44)	44(4)	31(3)	12(1)	-2(2)	-3(3)	-31(3)
F(45)	30(3)	31(3)	11(2)	8(2)	-6(2)	9(2)
F(46)	23(2)	39(4)	5(2)	1(3)	1(2)	-24(3)
F(44A)	72(11)	18(4)	19(5)	4(3)	-13(8)	-28(5)
F(45A)	16(3)	51(7)	15(4)	19(5)	-9(3)	-16(4)
F(46A)	34(6)	31(8)	8(4)	-1(5)	4(5)	-23(6)
F(44B)	44(4)	31(3)	12(1)	-2(2)	-3(3)	-31(3)
F(45B)	46(11)	17(6)	12(8)	7(5)	-10(8)	-16(6)
F(46B)	20(6)	61(12)	13(7)	4(10)	3(5)	-12(6)
F(51)	22(2)	43(4)	12(2)	-9(2)	1(2)	-24(3)
F(52)	22(2)	21(2)	44(4)	15(2)	-3(3)	-16(2)
F(53)	15(1)	32(2)	9(2)	-5(2)	5(1)	-13(1)
F(51A)	22(2)	43(4)	12(2)	-9(2)	1(2)	-24(3)
F(52A)	22(2)	21(2)	44(4)	15(2)	-3(3)	-16(2)
F(53A)	15(1)	32(2)	9(2)	-5(2)	5(1)	-13(1)
F(51B)	32(7)	58(10)	18(4)	-9(5)	7(4)	-38(7)
F(52B)	27(6)	40(6)	24(5)	15(5)	-5(5)	-24(6)
F(53B)	15(1)	32(2)	9(2)	-5(2)	5(1)	-13(1)
F(54)	17(1)	19(1)	34(1)	5(1)	-4(1)	-3(1)
F(55)	17(1)	19(1)	34(1)	5(1)	-4(1)	-3(1)
F(56)	17(1)	19(1)	34(1)	5(1)	-4(1)	-3(1)
F(54A)	17(1)	19(1)	34(1)	5(1)	-4(1)	-3(1)
F(55A)	17(1)	19(1)	34(1)	5(1)	-4(1)	-3(1)
F(56A)	17(1)	19(1)	34(1)	5(1)	-4(1)	-3(1)
F(54B)	17(1)	19(1)	34(1)	5(1)	-4(1)	-3(1)
F(55B)	17(1)	19(1)	34(1)	5(1)	-4(1)	-3(1)
F(56B)	17(1)	19(1)	34(1)	5(1)	-4(1)	-3(1)
C(21)	7(1)	10(1)	7(1)	2(1)	-2(1)	-4(1)
C(22)	8(1)	11(1)	11(1)	1(1)	-1(1)	-4(1)
C(23)	8(1)	11(1)	11(1)	-1(1)	-3(1)	-2(1)
C(24)	7(1)	16(1)	10(1)	2(1)	1(1)	-5(1)
C(25)	7(1)	12(1)	14(1)	3(1)	-3(1)	-4(1)
C(26)	7(1)	9(1)	10(1)	0(1)	-2(1)	-2(1)
C(27)	14(1)	21(1)	12(1)	-6(1)	3(1)	-9(1)
C(28)	11(1)	16(1)	19(1)	-1(1)	2(1)	-9(1)
C(31)	5(1)	12(1)	9(1)	-2(1)	1(1)	-4(1)
C(32)	11(1)	14(1)	6(1)	0(1)	-2(1)	-5(1)
C(33)	13(1)	12(1)	11(1)	1(1)	-1(1)	-6(1)
C(34)	13(1)	15(1)	10(1)	-2(1)	-2(1)	-6(1)
C(35)	9(1)	16(1)	8(1)	1(1)	-1(1)	-4(1)
C(36)	8(1)	12(1)	10(1)	1(1)	-2(1)	-4(1)
C(37)	31(1)	17(1)	13(1)	-1(1)	-7(1)	-11(1)
C(38)	23(1)	19(1)	12(1)	1(1)	-5(1)	-7(1)
C(41)	9(1)	7(1)	8(1)	-1(1)	-1(1)	-4(1)
C(42)	11(1)	12(1)	7(1)	-2(1)	-2(1)	-5(1)
C(43)	11(1)	13(1)	9(1)	-2(1)	0(1)	-5(1)
C(44)	9(1)	13(1)	14(1)	1(1)	-3(1)	-6(1)
C(45)	13(1)	10(1)	6(1)	2(1)	-2(1)	-7(1)
C(46)	10(1)	11(1)	8(1)	-1(1)	1(1)	-5(1)
C(47)	10(1)	25(1)	15(1)	-5(1)	-1(1)	-3(1)
C(48)	15(1)	14(1)	9(1)	3(1)	-4(1)	-9(1)
C(51)	10(1)	12(1)	4(1)	-2(1)	0(1)	-5(1)

C(52)	9(1)	12(1)	7(1)	-1(1)	-1(1)	-4(1)
C(53)	12(1)	13(1)	7(1)	-1(1)	1(1)	-7(1)
C(54)	15(1)	12(1)	10(1)	1(1)	-1(1)	-7(1)
C(55)	11(1)	11(1)	9(1)	0(1)	-2(1)	-3(1)
C(56)	10(1)	14(1)	10(1)	0(1)	-1(1)	-7(1)
C(57)	13(1)	19(1)	12(1)	0(1)	1(1)	-9(1)
C(58)	15(1)	15(1)	33(1)	6(1)	-7(1)	-5(1)
B(1)	7(1)	12(1)	5(1)	0(1)	0(1)	-4(1)

Table S120. Hydrogen coordinates (x 10^4) and isotropic displacement parameters ($Å^2$x 10^3) for **[(D$_{12}$-TMC)Co^{II}(NO$_3$)]B$_{Ar}$F**.

	x	y	z	U(eq)
H(1A)	9192	157	7510	17
H(1AB)	7666	708	7737	17
H(2A)	9057	1518	6496	18
H(2AB)	8696	1959	7364	18
H(3A)	6490	2571	7367	19
H(3AB)	7185	3200	6765	19
H(5A)	5039	3015	5841	20
H(5AB)	5175	3740	6450	20
H(6A)	4718	2621	7474	20
H(6AB)	3567	3145	7037	20
H(8A)	3323	1502	7788	19
H(8AB)	4751	991	7908	19
H(9A)	3808	-305	7939	21
H(9AB)	3933	-132	7026	21
H(10A)	6055	-967	7739	18
H(10B)	5636	-1723	7336	18
H(12A)	8424	-1853	6081	20
H(12B)	7886	-2382	6850	20
H(13A)	7802	-980	7614	17
H(13B)	9233	-1667	7136	17
H(22)	1034	6251	3112	12
H(24)	-1723	5337	4323	14
H(26)	334	3991	2205	11
H(32)	2697	2757	1575	13
H(34)	2294	2669	-612	15
H(36)	1733	5517	322	12
H(42)	4668	3690	1097	12
H(44)	6577	2848	2826	14
H(46)	2735	4606	3305	12
H(52)	3921	5874	1310	12
H(54)	1717	8952	741	15
H(56)	115	6863	1650	13

[(D_{12}-TMC)Co^{II}(NO_3)]$B_{Ar}F$

The structure of **[(D_{12}-TMC)Co^{II}(NO_3)]$B_{Ar}F$** was solved in the triclinic space group $P\bar{1}$ and the asymmetric unit contains one full molecule (anion and cation). Three of the CF_3 groups of the anion were found to be disordered and were modelled over three positions each. The disorder was refined with the help of 1,2 and 1,3-distance restraints, similarity restraints on anisotropic displacement parameters and advanced rigid bond restraints. To allow for full anisotropic refinement a few atoms were set to have the same anisotropic displacement parameters. All of the disorder ratios were allowed to refine freely and converged to 0.464(2):0.371(3):0.164(2), 0.645(3):0.249(3):0.106(3) and 0.630(3):0.105(2):0.265(3).

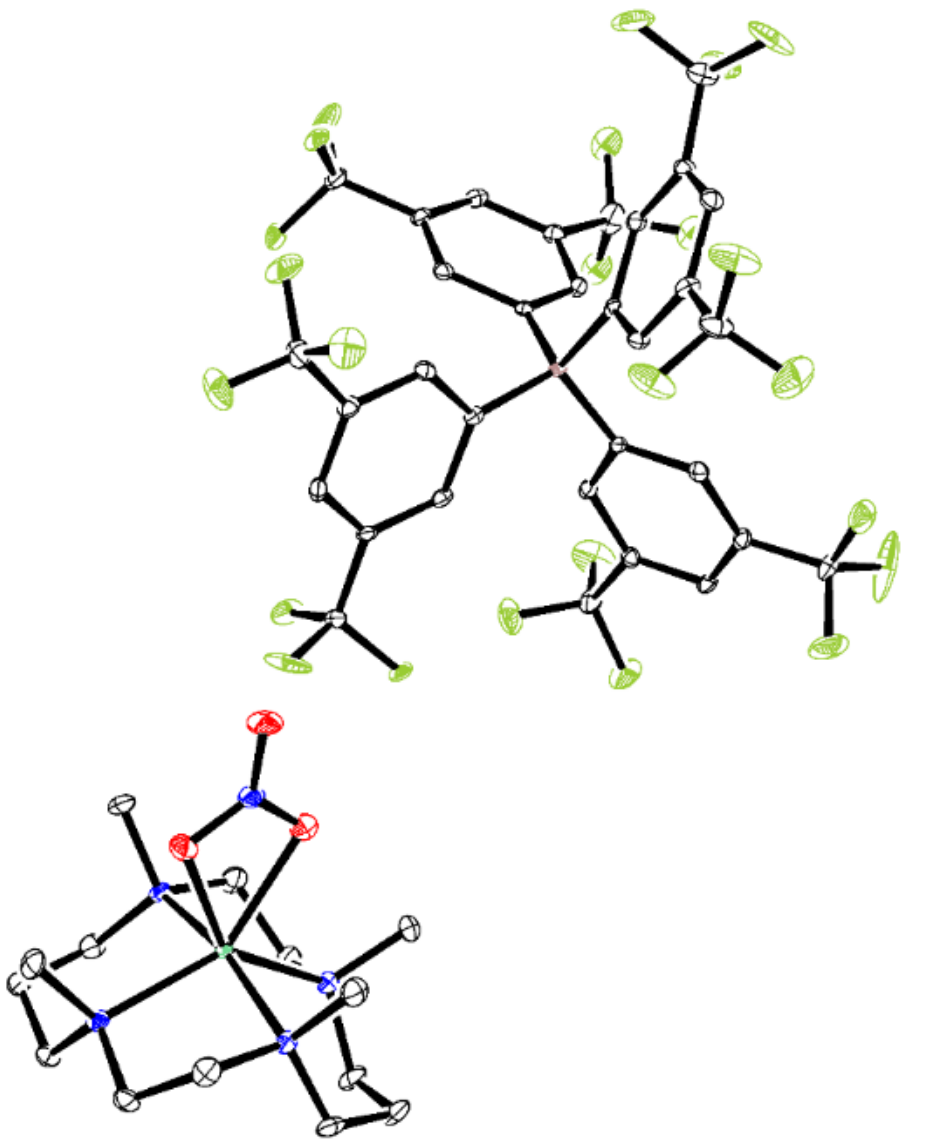

Figure S24. Molecular structure of **[(D_{12}-TMC)Co^{II}(NO_3)]$B_{Ar}F$**.

Table S121. Crystal data and structure refinement for **[(D_{12}-TMC)Co^{II}(NO_3)]$B_{Ar}F$**.

CCDC No	2062721	
Empirical formula	$C_{46}H_{32}BCoD_{12}F_{24}N_5O_3$	
Formula weight	1252.67	
Temperature	100(2) K	
Wavelength	0.71073 Å	
Crystal system	Triclinic	
Space group	$P\bar{1}$	
Unit cell dimensions	a = 11.8760(8) Å	α = 84.732(2)°
	b = 13.5042(8) Å	β = 75.856(2)°
	c = 17.7906(11) Å	γ = 64.880(2)°
Volume	2504.8(3) $Å^3$	
Z	2	
Density (calculated)	1.661 Mg/m^3	
Absorption coefficient	0.479 mm^{-1}	
F(000)	1254	
Crystal size	0.980 x 0.510 x 0.052 mm^3	
Theta range for data collection	2.361 to 29.129°.	
Index ranges	-16 ≤ h ≤ 16, -18 ≤ k ≤ 18, -24 ≤ l ≤ 24	
Reflections collected	91232	
Independent reflections	13440 [R(int) = 0.1075]	
Completeness to theta = 25.242°	99.3 %	
Absorption correction	Semi-empirical from equivalents	
Refinement method	Full-matrix least-squares on F^2	
Data / restraints / parameters	13440 / 3795 / 818	
Goodness-of-fit on F^2	1.032	
Final R indices [I>2σ(I)]	R1 = 0.0545, wR2 = 0.1137	
R indices (all data)	R1 = 0.0827, wR2 = 0.1248	
Largest diff. peak and hole	0.836 and -0.758 $eÅ^{-3}$	

Table S122. Atomic coordinates (x 10^4) and equivalent isotropic displacement parameters ($Å^2$x 10^3) for **[(D_{12}-TMC)Co^{II}(NO_3)]B_{Ar}F**. U(eq) is defined as one third of the trace of the orthogonalized U^{ij} tensor.

	x	y	z	U(eq)
Co(1)	6657(1)	616(1)	6248(1)	8(1)
O(1)	8001(2)	-25(2)	5113(1)	20(1)
O(2)	6011(2)	913(2)	5159(1)	21(1)
O(3)	7328(2)	300(2)	4042(1)	27(1)
N(1)	8395(2)	-136(2)	6710(1)	11(1)
N(2)	6673(2)	2229(2)	6245(1)	11(1)
N(3)	4631(2)	1508(2)	6808(1)	12(1)
N(4)	6536(2)	-942(2)	6562(1)	11(1)
N(5)	7120(2)	394(2)	4744(1)	16(1)
C(1)	8420(2)	565(2)	7302(1)	14(1)
C(2)	8421(2)	1650(2)	6996(1)	15(1)
C(3)	7134(2)	2486(2)	6876(1)	16(1)
C(4)	7400(2)	2441(2)	5490(1)	17(1)
D(4A)	7097	2287	5068	25
D(4B)	8311	1967	5432	25
D(4C)	7271	3208	5471	25
C(5)	5298(2)	2993(2)	6333(2)	17(1)
C(6)	4474(2)	2626(2)	6979(2)	16(1)
C(7)	3752(2)	1639(2)	6295(2)	19(1)
D(7A)	3893	2096	5853	28
D(7B)	2865	1989	6589	28
D(7C)	3921	919	6106	28
C(8)	4219(2)	1018(2)	7549(1)	16(1)
C(9)	4315(2)	-126(2)	7461(1)	17(1)
C(10)	5670(2)	-1001(2)	7312(1)	15(1)
C(11)	6210(2)	-1348(2)	5936(1)	17(1)
D(11A)	6768	-1311	5438	26
D(11B)	5317	-894	5920	26
D(11C)	6328	-2107	6038	26
C(12)	7867(2)	-1686(2)	6609(1)	16(1)
C(13)	8363(2)	-1154(2)	7087(1)	14(1)
C(14)	9609(2)	-452(2)	6105(1)	17(1)
D(14A)	9697	-1034	5774	26
D(14B)	10332	-715	6355	26
D(14C)	9595	185	5789	26
F(21)	-1268(2)	6443(1)	5123(1)	28(1)
F(22)	541(2)	6485(2)	4615(1)	29(1)
F(23)	-1142(2)	7674(1)	4296(1)	34(1)
F(24)	-1447(2)	3251(2)	3893(1)	40(1)
F(25)	-2850(2)	4623(2)	3457(2)	56(1)
F(26)	-1341(2)	3382(1)	2685(1)	28(1)
F(31)	3767(2)	832(1)	966(1)	30(1)
F(32)	1861(2)	1139(1)	933(1)	37(1)
F(33)	3300(2)	844(1)	-128(1)	32(1)
F(34)	890(2)	5751(1)	-913(1)	26(1)
F(35)	1468(2)	4192(1)	-1446(1)	37(1)
F(36)	2863(2)	4790(2)	-1421(1)	33(1)
F(41)	7000(2)	1705(1)	1018(1)	28(1)
F(42)	7149(2)	3213(1)	675(1)	28(1)
F(43)	8036(1)	2272(2)	1576(1)	36(1)
F(44)	4709(6)	4688(4)	4208(4)	24(1)
F(45)	5594(5)	2939(4)	4220(4)	32(2)
F(46)	3562(6)	3793(6)	4484(5)	20(2)
F(44A)	4307(16)	4838(10)	4242(14)	33(4)
F(45A)	5775(12)	3209(12)	4173(11)	27(3)
F(46A)	3810(15)	3498(16)	4523(13)	22(4)
F(44B)	5190(20)	4405(17)	4178(15)	24(1)
F(45B)	5150(20)	2841(13)	4351(13)	24(5)
F(46B)	3414(13)	4293(18)	4452(12)	35(5)
F(51)	4695(9)	7632(7)	1292(4)	22(1)
F(52)	3950(9)	8591(4)	351(4)	28(2)
F(53)	5053(5)	6857(4)	203(2)	19(1)
F(51A)	5017(13)	7084(13)	1171(8)	22(1)
F(52A)	3922(14)	8683(9)	900(11)	28(2)
F(53A)	4764(13)	7413(14)	14(6)	19(1)
F(51B)	4510(20)	7925(17)	1282(11)	31(4)
F(52B)	4070(20)	8394(11)	171(8)	28(3)
F(53B)	5159(13)	6729(11)	375(7)	19(1)
F(54)	-1052(4)	9566(3)	1819(3)	25(1)
F(55)	-1439(5)	8484(5)	1197(3)	25(1)
F(56)	-581(3)	9569(4)	584(3)	25(1)
F(54A)	-546(4)	9807(4)	854(3)	25(1)
F(55A)	-1203(4)	9265(4)	1971(3)	25(1)
F(56A)	-1319(6)	8572(5)	982(3)	25(1)
F(54B)	-762(9)	9843(7)	1422(7)	25(1)
F(55B)	-1538(10)	8666(10)	1398(7)	25(1)
F(56B)	-572(9)	9113(9)	373(5)	25(1)
C(21)	916(2)	5074(2)	2535(1)	8(1)
C(22)	590(2)	5799(2)	3149(1)	10(1)
C(23)	-354(2)	5880(2)	3810(1)	10(1)
C(24)	-1072(2)	5281(2)	3873(1)	12(1)
C(25)	-810(2)	4597(2)	3258(1)	11(1)
C(26)	167(2)	4482(2)	2609(1)	10(1)
C(27)	-563(2)	6616(2)	4458(1)	15(1)
C(28)	-1608(2)	3973(2)	3317(1)	15(1)
C(31)	2189(2)	4262(2)	1082(1)	9(1)
C(32)	2501(2)	3135(2)	1114(1)	10(1)
C(33)	2535(2)	2549(2)	498(1)	12(1)
C(34)	2272(2)	3065(2)	-190(1)	12(1)
C(35)	1975(2)	4172(2)	-241(1)	12(1)

C(36)	1937(2)	4757(2)	379(1)	10(1)
C(37)	2870(3)	1351(2)	560(1)	19(1)
C(38)	1791(2)	4728(2)	-996(1)	18(1)
C(41)	3461(2)	4268(2)	2149(1)	8(1)
C(42)	4643(2)	3714(2)	1633(1)	10(1)
C(43)	5779(2)	3201(2)	1883(1)	11(1)
C(44)	5801(2)	3204(2)	2657(1)	11(1)
C(45)	4645(2)	3747(2)	3177(1)	9(1)
C(46)	3509(2)	4254(2)	2931(1)	10(1)
C(47)	6994(2)	2600(2)	1294(1)	18(1)
C(48)	4626(2)	3802(2)	4014(1)	12(1)
C(51)	2041(2)	6152(2)	1534(1)	8(1)
C(52)	3097(2)	6413(2)	1280(1)	10(1)
C(53)	2977(2)	7436(2)	986(1)	10(1)
C(54)	1798(2)	8254(2)	940(1)	12(1)
C(55)	733(2)	8020(2)	1196(1)	11(1)
C(56)	860(2)	6997(2)	1482(1)	11(1)
C(57)	4157(2)	7635(2)	711(1)	14(1)
C(58)	-558(2)	8899(2)	1186(2)	22(1)
B(1)	2152(2)	4931(2)	1822(1)	8(1)

Table S123. Bond lengths [Å] and angles [°] for **[(D$_{12}$-TMC)Co^{II}(NO$_3$)]B$_{Ar}$F**.

Co(1)-N(4)	2.1814(19)	F(55)-C(58)	1.376(6)
Co(1)-N(2)	2.1852(19)	F(56)-C(58)	1.336(4)
Co(1)-N(3)	2.1962(19)	F(54A)-C(58)	1.316(5)
Co(1)-O(2)	2.1975(18)	F(55A)-C(58)	1.436(5)
Co(1)-N(1)	2.204(2)	F(56A)-C(58)	1.292(6)
Co(1)-O(1)	2.2237(17)	F(54B)-C(58)	1.283(8)
O(1)-N(5)	1.269(3)	F(55B)-C(58)	1.292(9)
O(2)-N(5)	1.267(3)	F(56B)-C(58)	1.453(8)
O(3)-N(5)	1.221(3)	C(21)-C(22)	1.404(3)
N(1)-C(13)	1.485(3)	C(21)-C(26)	1.404(3)
N(1)-C(14)	1.490(3)	C(21)-B(1)	1.642(3)
N(1)-C(1)	1.494(3)	C(22)-C(23)	1.387(3)
N(2)-C(4)	1.484(3)	C(22)-H(22)	0.9500
N(2)-C(3)	1.488(3)	C(23)-C(24)	1.384(3)
N(2)-C(5)	1.494(3)	C(23)-C(27)	1.498(3)
N(3)-C(8)	1.488(3)	C(24)-C(25)	1.388(3)
N(3)-C(6)	1.491(3)	C(24)-H(24)	0.9500
N(3)-C(7)	1.494(3)	C(25)-C(26)	1.389(3)
N(4)-C(11)	1.482(3)	C(25)-C(28)	1.494(3)
N(4)-C(12)	1.488(3)	C(26)-H(26)	0.9500
N(4)-C(10)	1.491(3)	C(31)-C(36)	1.400(3)
C(1)-C(2)	1.516(3)	C(31)-C(32)	1.404(3)
C(1)-H(1A)	0.9900	C(31)-B(1)	1.646(3)
C(1)-H(1AB)	0.9900	C(32)-C(33)	1.393(3)
C(2)-C(3)	1.519(4)	C(32)-H(32)	0.9500
C(2)-H(2A)	0.9900	C(33)-C(34)	1.387(3)
C(2)-H(2AB)	0.9900	C(33)-C(37)	1.494(3)
C(3)-H(3A)	0.9900	C(34)-C(35)	1.382(3)
C(3)-H(3AB)	0.9900	C(34)-H(34)	0.9500
C(4)-D(4A)	0.9800	C(35)-C(36)	1.396(3)
C(4)-D(4B)	0.9800	C(35)-C(38)	1.496(3)
C(4)-D(4C)	0.9800	C(36)-H(36)	0.9500
C(5)-C(6)	1.512(3)	C(41)-C(46)	1.404(3)
C(5)-H(5A)	0.9900	C(41)-C(42)	1.406(3)
C(5)-H(5AB)	0.9900	C(41)-B(1)	1.650(3)
C(6)-H(6A)	0.9900	C(42)-C(43)	1.393(3)
C(6)-H(6AB)	0.9900	C(42)-H(42)	0.9500
C(7)-D(7A)	0.9800	C(43)-C(44)	1.385(3)
C(7)-D(7B)	0.9800	C(43)-C(47)	1.504(3)
C(7)-D(7C)	0.9800	C(44)-C(45)	1.388(3)
C(8)-C(9)	1.521(3)	C(44)-H(44)	0.9500
C(8)-H(8A)	0.9900	C(45)-C(46)	1.390(3)
C(8)-H(8AB)	0.9900	C(45)-C(48)	1.492(3)
C(9)-C(10)	1.514(3)	C(46)-H(46)	0.9500
C(9)-H(9A)	0.9900	C(51)-C(56)	1.401(3)
C(9)-H(9AB)	0.9900	C(51)-C(52)	1.404(3)
C(10)-H(10A)	0.9900	C(51)-B(1)	1.640(3)
C(10)-H(10B)	0.9900	C(52)-C(53)	1.392(3)
C(11)-D(11A)	0.9800	C(52)-H(52)	0.9500
C(11)-D(11B)	0.9800	C(53)-C(54)	1.383(3)
C(11)-D(11C)	0.9800	C(53)-C(57)	1.495(3)
C(12)-C(13)	1.517(3)	C(54)-C(55)	1.392(3)
C(12)-H(12A)	0.9900	C(54)-H(54)	0.9500
C(12)-H(12B)	0.9900	C(55)-C(56)	1.387(3)
C(13)-H(13A)	0.9900	C(55)-C(58)	1.492(3)
C(13)-H(13B)	0.9900	C(56)-H(56)	0.9500
C(14)-D(14A)	0.9800		
C(14)-D(14B)	0.9800	N(4)-Co(1)-N(2)	165.77(7)
C(14)-D(14C)	0.9800	N(4)-Co(1)-N(3)	90.61(7)
F(21)-C(27)	1.335(3)	N(2)-Co(1)-N(3)	83.25(7)
F(22)-C(27)	1.342(3)	N(4)-Co(1)-O(2)	99.60(7)
F(23)-C(27)	1.336(3)	N(2)-Co(1)-O(2)	92.74(7)
F(24)-C(28)	1.338(3)	N(3)-Co(1)-O(2)	85.55(7)
F(25)-C(28)	1.331(3)	N(4)-Co(1)-N(1)	83.30(7)
F(26)-C(28)	1.333(3)	N(2)-Co(1)-N(1)	91.35(7)
F(31)-C(37)	1.340(3)	N(3)-Co(1)-N(1)	132.38(7)
F(32)-C(37)	1.351(3)	O(2)-Co(1)-N(1)	142.06(7)
F(33)-C(37)	1.333(3)	N(4)-Co(1)-O(1)	93.25(7)
F(34)-C(38)	1.334(3)	N(2)-Co(1)-O(1)	99.35(7)
F(35)-C(38)	1.342(3)	N(3)-Co(1)-O(1)	143.38(7)
F(36)-C(38)	1.345(3)	O(2)-Co(1)-O(1)	57.89(7)
F(41)-C(47)	1.343(3)	N(1)-Co(1)-O(1)	84.22(7)
F(42)-C(47)	1.340(3)	N(5)-O(1)-Co(1)	92.90(13)
F(43)-C(47)	1.332(3)	N(5)-O(2)-Co(1)	94.18(13)
F(44)-C(48)	1.323(5)	C(13)-N(1)-C(14)	107.59(18)
F(45)-C(48)	1.343(5)	C(13)-N(1)-C(1)	108.60(17)
F(46)-C(48)	1.338(5)	C(14)-N(1)-C(1)	108.10(17)
F(44A)-C(48)	1.358(10)	C(13)-N(1)-Co(1)	104.99(13)
F(45A)-C(48)	1.342(10)	C(14)-N(1)-Co(1)	114.16(14)
F(46A)-C(48)	1.330(11)	C(1)-N(1)-Co(1)	113.12(14)
F(44B)-C(48)	1.342(11)	C(4)-N(2)-C(3)	108.41(17)
F(45B)-C(48)	1.335(11)	C(4)-N(2)-C(5)	108.38(18)
F(46B)-C(48)	1.362(11)	C(3)-N(2)-C(5)	107.69(18)
F(51)-C(57)	1.340(5)	C(4)-N(2)-Co(1)	111.20(14)
F(52)-C(57)	1.338(5)	C(3)-N(2)-Co(1)	117.43(14)
F(53)-C(57)	1.344(5)	C(5)-N(2)-Co(1)	103.27(13)
F(51A)-C(57)	1.385(10)	C(8)-N(3)-C(6)	108.32(18)
F(52A)-C(57)	1.379(10)	C(8)-N(3)-C(7)	108.47(18)
F(53A)-C(57)	1.263(9)	C(6)-N(3)-C(7)	107.16(19)
F(51B)-C(57)	1.335(11)	C(8)-N(3)-Co(1)	113.59(14)
F(52B)-C(57)	1.327(11)	C(6)-N(3)-Co(1)	105.60(13)
F(53B)-C(57)	1.349(10)	C(7)-N(3)-Co(1)	113.36(15)
F(54)-C(58)	1.355(5)	C(11)-N(4)-C(12)	108.39(18)

C(11)-N(4)-C(10)	108.34(17)
C(12)-N(4)-C(10)	107.79(18)
C(11)-N(4)-Co(1)	110.66(14)
C(12)-N(4)-Co(1)	102.98(13)
C(10)-N(4)-Co(1)	118.20(14)
O(3)-N(5)-O(2)	122.6(2)
O(3)-N(5)-O(1)	122.4(2)
O(2)-N(5)-O(1)	115.03(19)
N(1)-C(1)-C(2)	114.03(18)
N(1)-C(1)-H(1A)	108.7
C(2)-C(1)-H(1A)	108.7
N(1)-C(1)-H(1AB)	108.7
C(2)-C(1)-H(1AB)	108.7
H(1A)-C(1)-H(1AB)	107.6
C(1)-C(2)-C(3)	114.05(19)
C(1)-C(2)-H(2A)	108.7
C(3)-C(2)-H(2A)	108.7
C(1)-C(2)-H(2AB)	108.7
C(3)-C(2)-H(2AB)	108.7
H(2A)-C(2)-H(2AB)	107.6
N(2)-C(3)-C(2)	115.85(19)
N(2)-C(3)-H(3A)	108.3
C(2)-C(3)-H(3A)	108.3
N(2)-C(3)-H(3AB)	108.3
C(2)-C(3)-H(3AB)	108.3
H(3A)-C(3)-H(3AB)	107.4
N(2)-C(4)-D(4A)	109.5
N(2)-C(4)-D(4B)	109.5
D(4A)-C(4)-D(4B)	109.5
N(2)-C(4)-D(4C)	109.5
D(4A)-C(4)-D(4C)	109.5
D(4B)-C(4)-D(4C)	109.5
N(2)-C(5)-C(6)	110.42(19)
N(2)-C(5)-H(5A)	109.6
C(6)-C(5)-H(5A)	109.6
N(2)-C(5)-H(5AB)	109.6
C(6)-C(5)-H(5AB)	109.6
H(5A)-C(5)-H(5AB)	108.1
N(3)-C(6)-C(5)	110.34(19)
N(3)-C(6)-H(6A)	109.6
C(5)-C(6)-H(6A)	109.6
N(3)-C(6)-H(6AB)	109.6
C(5)-C(6)-H(6AB)	109.6
H(6A)-C(6)-H(6AB)	108.1
N(3)-C(7)-D(7A)	109.5
N(3)-C(7)-D(7B)	109.5
D(7A)-C(7)-D(7B)	109.5
N(3)-C(7)-D(7C)	109.5
D(7A)-C(7)-D(7C)	109.5
D(7B)-C(7)-D(7C)	109.5
N(3)-C(8)-C(9)	114.17(19)
N(3)-C(8)-H(8A)	108.7
C(9)-C(8)-H(8A)	108.7
N(3)-C(8)-H(8AB)	108.7
C(9)-C(8)-H(8AB)	108.7
H(8A)-C(8)-H(8AB)	107.6
C(10)-C(9)-C(8)	113.64(19)
C(10)-C(9)-H(9A)	108.8
C(8)-C(9)-H(9A)	108.8
C(10)-C(9)-H(9AB)	108.8
C(8)-C(9)-H(9AB)	108.8
H(9A)-C(9)-H(9AB)	107.7
N(4)-C(10)-C(9)	116.4(2)
N(4)-C(10)-H(10A)	108.2
C(9)-C(10)-H(10A)	108.2
N(4)-C(10)-H(10B)	108.2
C(9)-C(10)-H(10B)	108.2
H(10A)-C(10)-H(10B)	107.3
N(4)-C(11)-D(11A)	109.5
N(4)-C(11)-D(11B)	109.5
D(11A)-C(11)-D(11B)	109.5
N(4)-C(11)-D(11C)	109.5
D(11A)-C(11)-D(11C)	109.5
D(11B)-C(11)-D(11C)	109.5
N(4)-C(12)-C(13)	110.13(19)
N(4)-C(12)-H(12A)	109.6
C(13)-C(12)-H(12A)	109.6
N(4)-C(12)-H(12B)	109.6
C(13)-C(12)-H(12B)	109.6
H(12A)-C(12)-H(12B)	108.1
N(1)-C(13)-C(12)	109.94(19)
N(1)-C(13)-H(13A)	109.7
C(12)-C(13)-H(13A)	109.7
N(1)-C(13)-H(13B)	109.7
C(12)-C(13)-H(13B)	109.7
H(13A)-C(13)-H(13B)	108.2
N(1)-C(14)-D(14A)	109.5
N(1)-C(14)-D(14B)	109.5
D(14A)-C(14)-D(14B)	109.5
N(1)-C(14)-D(14C)	109.5
D(14A)-C(14)-D(14C)	109.5
D(14B)-C(14)-D(14C)	109.5
C(22)-C(21)-C(26)	115.57(19)
C(22)-C(21)-B(1)	119.14(18)
C(26)-C(21)-B(1)	125.21(19)
C(23)-C(22)-C(21)	122.5(2)
C(23)-C(22)-H(22)	118.7
C(21)-C(22)-H(22)	118.7
C(24)-C(23)-C(22)	120.8(2)
C(24)-C(23)-C(27)	120.7(2)
C(22)-C(23)-C(27)	118.47(19)
C(23)-C(24)-C(25)	117.7(2)
C(23)-C(24)-H(24)	121.1
C(25)-C(24)-H(24)	121.1
C(24)-C(25)-C(26)	121.5(2)
C(24)-C(25)-C(28)	117.74(19)
C(26)-C(25)-C(28)	120.7(2)
C(25)-C(26)-C(21)	121.7(2)
C(25)-C(26)-H(26)	119.2
C(21)-C(26)-H(26)	119.2
F(21)-C(27)-F(23)	106.88(19)
F(21)-C(27)-F(22)	106.1(2)
F(23)-C(27)-F(22)	105.69(19)
F(21)-C(27)-C(23)	113.13(19)
F(23)-C(27)-C(23)	112.6(2)
F(22)-C(27)-C(23)	111.95(19)
F(25)-C(28)-F(26)	106.8(2)
F(25)-C(28)-F(24)	106.3(2)
F(26)-C(28)-F(24)	104.90(19)
F(25)-C(28)-C(25)	112.3(2)
F(26)-C(28)-C(25)	113.91(19)
F(24)-C(28)-C(25)	112.1(2)
C(36)-C(31)-C(32)	115.3(2)
C(36)-C(31)-B(1)	123.2(2)
C(32)-C(31)-B(1)	121.46(19)
C(33)-C(32)-C(31)	122.7(2)
C(33)-C(32)-H(32)	118.7
C(31)-C(32)-H(32)	118.7
C(34)-C(33)-C(32)	120.6(2)
C(34)-C(33)-C(37)	118.6(2)
C(32)-C(33)-C(37)	120.7(2)
C(35)-C(34)-C(33)	117.9(2)
C(35)-C(34)-H(34)	121.0
C(33)-C(34)-H(34)	121.0
C(34)-C(35)-C(36)	121.3(2)
C(34)-C(35)-C(38)	118.9(2)
C(36)-C(35)-C(38)	119.6(2)
C(35)-C(36)-C(31)	122.1(2)
C(35)-C(36)-H(36)	118.9
C(31)-C(36)-H(36)	118.9
F(33)-C(37)-F(31)	106.8(2)
F(33)-C(37)-F(32)	106.5(2)
F(31)-C(37)-F(32)	105.0(2)
F(33)-C(37)-C(33)	113.0(2)
F(31)-C(37)-C(33)	112.7(2)
F(32)-C(37)-C(33)	112.3(2)
F(34)-C(38)-F(35)	106.9(2)
F(34)-C(38)-F(36)	106.2(2)
F(35)-C(38)-F(36)	105.6(2)
F(34)-C(38)-C(35)	113.21(19)
F(35)-C(38)-C(35)	112.4(2)
F(36)-C(38)-C(35)	112.1(2)
C(46)-C(41)-C(42)	115.2(2)
C(46)-C(41)-B(1)	124.15(18)
C(42)-C(41)-B(1)	120.55(19)
C(43)-C(42)-C(41)	122.1(2)
C(43)-C(42)-H(42)	118.9
C(41)-C(42)-H(42)	118.9
C(44)-C(43)-C(42)	121.5(2)
C(44)-C(43)-C(47)	120.0(2)
C(42)-C(43)-C(47)	118.5(2)
C(43)-C(44)-C(45)	117.4(2)
C(43)-C(44)-H(44)	121.3
C(45)-C(44)-H(44)	121.3
C(44)-C(45)-C(46)	121.2(2)
C(44)-C(45)-C(48)	119.3(2)
C(46)-C(45)-C(48)	119.43(19)
C(45)-C(46)-C(41)	122.5(2)
C(45)-C(46)-H(46)	118.7
C(41)-C(46)-H(46)	118.7
F(43)-C(47)-F(42)	106.9(2)
F(43)-C(47)-F(41)	107.0(2)
F(42)-C(47)-F(41)	105.83(19)
F(43)-C(47)-C(43)	113.1(2)
F(42)-C(47)-C(43)	112.1(2)
F(41)-C(47)-C(43)	111.48(19)
F(44)-C(48)-F(46)	106.1(4)
F(45B)-C(48)-F(44B)	105.2(13)
F(46A)-C(48)-F(45A)	105.9(10)
F(44)-C(48)-F(45)	106.7(4)
F(46)-C(48)-F(45)	105.5(4)
F(46A)-C(48)-F(44A)	103.8(12)
F(45A)-C(48)-F(44A)	104.9(10)
F(45B)-C(48)-F(46B)	104.9(12)
F(44B)-C(48)-F(46B)	102.8(12)
F(44)-C(48)-C(45)	113.6(4)
F(46A)-C(48)-C(45)	117.1(12)
F(45B)-C(48)-C(45)	115.6(11)
F(46)-C(48)-C(45)	112.7(4)
F(44B)-C(48)-C(45)	115.1(11)

F(45A)-C(48)-C(45)	112.6(9)	F(52B)-C(57)-C(53)	114.0(11)
F(45)-C(48)-C(45)	111.6(3)	F(51B)-C(57)-C(53)	113.0(11)
F(44A)-C(48)-C(45)	111.6(10)	F(52)-C(57)-C(53)	113.3(5)
F(46B)-C(48)-C(45)	111.9(11)	F(51)-C(57)-C(53)	112.3(5)
C(56)-C(51)-C(52)	115.3(2)	F(53)-C(57)-C(53)	111.5(3)
C(56)-C(51)-B(1)	120.85(18)	F(53B)-C(57)-C(53)	111.3(8)
C(52)-C(51)-B(1)	123.7(2)	F(52A)-C(57)-C(53)	110.7(6)
C(53)-C(52)-C(51)	122.2(2)	F(51A)-C(57)-C(53)	110.2(7)
C(53)-C(52)-H(52)	118.9	F(54B)-C(58)-F(55B)	113.8(7)
C(51)-C(52)-H(52)	118.9	F(56A)-C(58)-F(54A)	114.3(4)
C(54)-C(53)-C(52)	121.2(2)	F(56)-C(58)-F(54)	104.7(3)
C(54)-C(53)-C(57)	120.2(2)	F(56)-C(58)-F(55)	107.8(3)
C(52)-C(53)-C(57)	118.6(2)	F(54)-C(58)-F(55)	104.8(3)
C(53)-C(54)-C(55)	117.8(2)	F(56A)-C(58)-F(55A)	102.3(4)
C(53)-C(54)-H(54)	121.1	F(54A)-C(58)-F(55A)	101.5(3)
C(55)-C(54)-H(54)	121.1	F(54B)-C(58)-F(56B)	100.7(6)
C(56)-C(55)-C(54)	120.7(2)	F(55B)-C(58)-F(56B)	99.0(7)
C(56)-C(55)-C(58)	120.1(2)	F(54B)-C(58)-C(55)	115.2(5)
C(54)-C(55)-C(58)	119.1(2)	F(55B)-C(58)-C(55)	118.8(6)
C(55)-C(56)-C(51)	122.8(2)	F(56A)-C(58)-C(55)	114.6(3)
C(55)-C(56)-H(56)	118.6	F(54A)-C(58)-C(55)	114.3(3)
C(51)-C(56)-H(56)	118.6	F(56)-C(58)-C(55)	114.2(2)
F(52B)-C(57)-F(51B)	107.1(12)	F(54)-C(58)-C(55)	112.4(3)
F(52)-C(57)-F(51)	106.6(5)	F(55)-C(58)-C(55)	112.3(3)
F(52)-C(57)-F(53)	106.3(4)	F(55A)-C(58)-C(55)	108.0(3)
F(51)-C(57)-F(53)	106.3(4)	F(56B)-C(58)-C(55)	105.6(4)
F(52B)-C(57)-F(53B)	104.4(10)	C(51)-B(1)-C(21)	108.40(17)
F(51B)-C(57)-F(53B)	106.4(11)	C(51)-B(1)-C(31)	108.62(17)
F(53A)-C(57)-F(52A)	110.2(10)	C(21)-B(1)-C(31)	111.37(17)
F(53A)-C(57)-F(51A)	107.2(9)	C(51)-B(1)-C(41)	109.96(17)
F(52A)-C(57)-F(51A)	97.5(9)	C(21)-B(1)-C(41)	108.36(17)
F(53A)-C(57)-C(53)	118.9(7)	C(31)-B(1)-C(41)	110.12(18)

Table S124. Anisotropic displacement parameters (Å^{2}x 10^3) for **[(D$_{12}$-TMC)Co^{II}(NO$_3$)]B$_{Ar}$F**.
The anisotropic displacement factor exponent takes the form: -2p^2[h^2 a*^{2}U^{11} + ... + 2 h k a* b* U^{12}]

	U^{11}	U^{22}	U^{33}	U^{23}	U^{13}	U^{12}
Co(1)	8(1)	11(1)	6(1)	1(1)	-1(1)	-5(1)
O(1)	19(1)	30(1)	12(1)	0(1)	-4(1)	-12(1)
O(2)	18(1)	32(1)	15(1)	3(1)	-2(1)	-12(1)
O(3)	39(1)	43(1)	7(1)	0(1)	-4(1)	-26(1)
N(1)	11(1)	14(1)	7(1)	-1(1)	1(1)	-6(1)
N(2)	12(1)	14(1)	9(1)	2(1)	-1(1)	-8(1)
N(3)	11(1)	14(1)	12(1)	2(1)	-2(1)	-6(1)
N(4)	11(1)	12(1)	12(1)	0(1)	-1(1)	-6(1)
N(5)	24(1)	24(1)	9(1)	1(1)	-4(1)	-17(1)
C(1)	16(1)	22(1)	7(1)	-1(1)	-4(1)	-10(1)
C(2)	18(1)	22(1)	10(1)	-1(1)	-4(1)	-13(1)
C(3)	22(1)	16(1)	13(1)	-2(1)	-2(1)	-12(1)
C(4)	21(1)	21(1)	12(1)	6(1)	-2(1)	-14(1)
C(5)	16(1)	12(1)	22(1)	5(1)	-5(1)	-6(1)
C(6)	12(1)	12(1)	21(1)	-1(1)	-1(1)	-3(1)
C(7)	12(1)	26(1)	20(1)	8(1)	-9(1)	-9(1)
C(8)	13(1)	19(1)	13(1)	1(1)	4(1)	-7(1)
C(9)	16(1)	20(1)	16(1)	3(1)	1(1)	-12(1)
C(10)	20(1)	15(1)	12(1)	5(1)	-2(1)	-10(1)
C(11)	23(1)	19(1)	15(1)	-2(1)	-4(1)	-14(1)
C(12)	15(1)	11(1)	20(1)	0(1)	-2(1)	-4(1)
C(13)	14(1)	13(1)	15(1)	5(1)	-6(1)	-3(1)
C(14)	9(1)	24(1)	16(1)	-3(1)	2(1)	-6(1)
F(21)	32(1)	44(1)	12(1)	-13(1)	11(1)	-25(1)
F(22)	21(1)	50(1)	21(1)	-15(1)	-2(1)	-18(1)
F(23)	50(1)	16(1)	29(1)	-9(1)	-11(1)	-5(1)
F(24)	73(1)	47(1)	24(1)	17(1)	-14(1)	-50(1)
F(25)	9(1)	26(1)	128(2)	-22(1)	-2(1)	-7(1)
F(26)	36(1)	41(1)	22(1)	-6(1)	-1(1)	-31(1)
F(31)	50(1)	13(1)	25(1)	1(1)	-22(1)	-4(1)
F(32)	48(1)	27(1)	44(1)	4(1)	-4(1)	-28(1)
F(33)	62(1)	14(1)	17(1)	-4(1)	-14(1)	-10(1)
F(34)	33(1)	20(1)	18(1)	5(1)	-12(1)	-3(1)
F(35)	70(1)	29(1)	24(1)	5(1)	-33(1)	-22(1)
F(36)	30(1)	46(1)	14(1)	11(1)	1(1)	-13(1)
F(41)	23(1)	24(1)	30(1)	-15(1)	3(1)	-4(1)
F(42)	22(1)	36(1)	18(1)	-2(1)	9(1)	-11(1)
F(43)	8(1)	61(1)	26(1)	-15(1)	-3(1)	-1(1)
F(44)	44(4)	31(3)	12(1)	-2(2)	-3(3)	-31(3)
F(45)	30(3)	31(3)	11(2)	8(2)	-6(2)	9(2)
F(46)	23(2)	39(4)	5(2)	1(3)	1(2)	-24(3)
F(44A)	72(11)	18(4)	19(5)	4(3)	-13(8)	-28(5)
F(45A)	16(3)	51(7)	15(4)	19(5)	-9(3)	-16(4)
F(46A)	34(6)	31(8)	8(4)	-1(5)	4(5)	-23(6)
F(44B)	44(4)	31(3)	12(1)	-2(2)	-3(3)	-31(3)
F(45B)	46(11)	17(6)	12(8)	7(5)	-10(8)	-16(6)
F(46B)	20(6)	61(12)	13(7)	4(10)	3(5)	-12(6)
F(51)	22(2)	43(4)	12(2)	-9(2)	1(2)	-24(3)
F(52)	22(2)	21(2)	44(4)	15(2)	-3(3)	-16(2)
F(53)	15(1)	32(2)	9(2)	-5(2)	5(1)	-13(1)
F(51A)	22(2)	43(4)	12(2)	-9(2)	1(2)	-24(3)
F(52A)	22(2)	21(2)	44(4)	15(2)	-3(3)	-16(2)
F(53A)	15(1)	32(2)	9(2)	-5(2)	5(1)	-13(1)
F(51B)	32(7)	58(10)	18(4)	-9(5)	7(4)	-38(7)
F(52B)	27(6)	40(6)	24(5)	15(5)	-5(5)	-24(6)
F(53B)	15(1)	32(2)	9(2)	-5(2)	5(1)	-13(1)
F(54)	17(1)	19(1)	34(1)	5(1)	-4(1)	-3(1)
F(55)	17(1)	19(1)	34(1)	5(1)	-4(1)	-3(1)
F(56)	17(1)	19(1)	34(1)	5(1)	-4(1)	-3(1)
F(54A)	17(1)	19(1)	34(1)	5(1)	-4(1)	-3(1)
F(55A)	17(1)	19(1)	34(1)	5(1)	-4(1)	-3(1)
F(56A)	17(1)	19(1)	34(1)	5(1)	-4(1)	-3(1)
F(54B)	17(1)	19(1)	34(1)	5(1)	-4(1)	-3(1)
F(55B)	17(1)	19(1)	34(1)	5(1)	-4(1)	-3(1)
F(56B)	17(1)	19(1)	34(1)	5(1)	-4(1)	-3(1)
C(21)	7(1)	10(1)	7(1)	2(1)	-2(1)	-4(1)
C(22)	8(1)	11(1)	11(1)	1(1)	-1(1)	-4(1)
C(23)	8(1)	11(1)	11(1)	-1(1)	-3(1)	-2(1)
C(24)	7(1)	16(1)	10(1)	2(1)	1(1)	-5(1)
C(25)	7(1)	12(1)	14(1)	3(1)	-3(1)	-4(1)
C(26)	7(1)	9(1)	10(1)	0(1)	-2(1)	-2(1)
C(27)	14(1)	21(1)	12(1)	-6(1)	3(1)	-9(1)
C(28)	11(1)	16(1)	19(1)	-1(1)	2(1)	-9(1)
C(31)	5(1)	12(1)	9(1)	-2(1)	1(1)	-4(1)
C(32)	11(1)	14(1)	6(1)	0(1)	-2(1)	-5(1)
C(33)	13(1)	12(1)	11(1)	1(1)	-1(1)	-6(1)
C(34)	13(1)	15(1)	10(1)	-2(1)	-2(1)	-6(1)
C(35)	9(1)	16(1)	8(1)	1(1)	-1(1)	-4(1)
C(36)	8(1)	12(1)	10(1)	1(1)	-2(1)	-4(1)
C(37)	31(1)	17(1)	13(1)	-1(1)	-7(1)	-11(1)
C(38)	23(1)	19(1)	12(1)	1(1)	-5(1)	-7(1)
C(41)	9(1)	7(1)	8(1)	-1(1)	-1(1)	-4(1)
C(42)	11(1)	12(1)	7(1)	-2(1)	-2(1)	-5(1)
C(43)	11(1)	13(1)	9(1)	-2(1)	0(1)	-5(1)
C(44)	9(1)	13(1)	14(1)	1(1)	-3(1)	-6(1)
C(45)	13(1)	10(1)	6(1)	2(1)	-2(1)	-7(1)
C(46)	10(1)	11(1)	8(1)	-1(1)	1(1)	-5(1)
C(47)	10(1)	25(1)	15(1)	-5(1)	-1(1)	-3(1)
C(48)	15(1)	14(1)	9(1)	3(1)	-4(1)	-9(1)
C(51)	10(1)	12(1)	4(1)	-2(1)	0(1)	-5(1)

C(52)	9(1)	12(1)	7(1)	-1(1)	-1(1)	-4(1)
C(53)	12(1)	13(1)	7(1)	-1(1)	1(1)	-7(1)
C(54)	15(1)	12(1)	10(1)	1(1)	-1(1)	-7(1)
C(55)	11(1)	11(1)	9(1)	0(1)	-2(1)	-3(1)
C(56)	10(1)	14(1)	10(1)	0(1)	-1(1)	-7(1)
C(57)	13(1)	19(1)	12(1)	0(1)	1(1)	-9(1)
C(58)	15(1)	15(1)	33(1)	6(1)	-7(1)	-5(1)
B(1)	7(1)	12(1)	5(1)	0(1)	0(1)	-4(1)

Table S125. Hydrogen coordinates (x 10^4) and isotropic displacement parameters (Å^{2}x 10^3) for **[(D$_{12}$-TMC)Co^{II}(NO$_3$)]B$_{Ar}$F**.

	x	y	z	U(eq)
H(1A)	9192	157	7510	17
H(1AB)	7666	708	7737	17
H(2A)	9057	1518	6496	18
H(2AB)	8696	1959	7364	18
H(3A)	6490	2571	7367	19
H(3AB)	7185	3200	6765	19
H(5A)	5039	3015	5841	20
H(5AB)	5175	3740	6450	20
H(6A)	4718	2621	7474	20
H(6AB)	3567	3145	7037	20
H(8A)	3323	1502	7788	19
H(8AB)	4751	991	7908	19
H(9A)	3808	-305	7939	21
H(9AB)	3933	-132	7026	21
H(10A)	6055	-967	7739	18
H(10B)	5636	-1723	7336	18
H(12A)	8424	-1853	6081	20
H(12B)	7886	-2382	6850	20
H(13A)	7802	-980	7614	17
H(13B)	9233	-1667	7136	17
H(22)	1034	6251	3112	12
H(24)	-1723	5337	4323	14
H(26)	334	3991	2205	11
H(32)	2697	2757	1575	13
H(34)	2294	2669	-612	15
H(36)	1733	5517	322	12
H(42)	4668	3690	1097	12
H(44)	6577	2848	2826	14
H(46)	2735	4606	3305	12
H(52)	3921	5874	1310	12
H(54)	1717	8952	741	15
H(56)	115	6863	1650	13

[(D$_{12}$-TMC)Co^{II}(CH$_3$CN)$_2$][Co^{II}(NO$_3$)$_4$]

The crystal structure of **[(D$_{12}$-TMC)Co^{II}(CH$_3$CN)$_2$][Co^{II}(NO$_3$)$_4$]** was solved in the monoclinic space group *P*2$_1$/*c* as non-merohedral twin with two domains. The twin ratio was refined and converged to 0.1062(7). The asymmetric unit contains the [(D$_{12}$-TMC)Co^{II}(CH$_3$CN)$_2$] cation and the [Co(NO$_3$)$_4$] anion. Two nitrate ligands in the anion were found to be disordered over two positions. The disorder was refined using same distance restraints on 1,2 and 1,3 distances, similarities on anisotropic displacement parameters and advanced rigid bond restraints. The anisotropic displacement of the two disorder positions of one nitrogen atoms were set to the same anisotropic displacement parameters to allow for anisotropic refinement. The disorder ratios were allowed to refine freely and converged to 0.63(5) and 0.57(5).

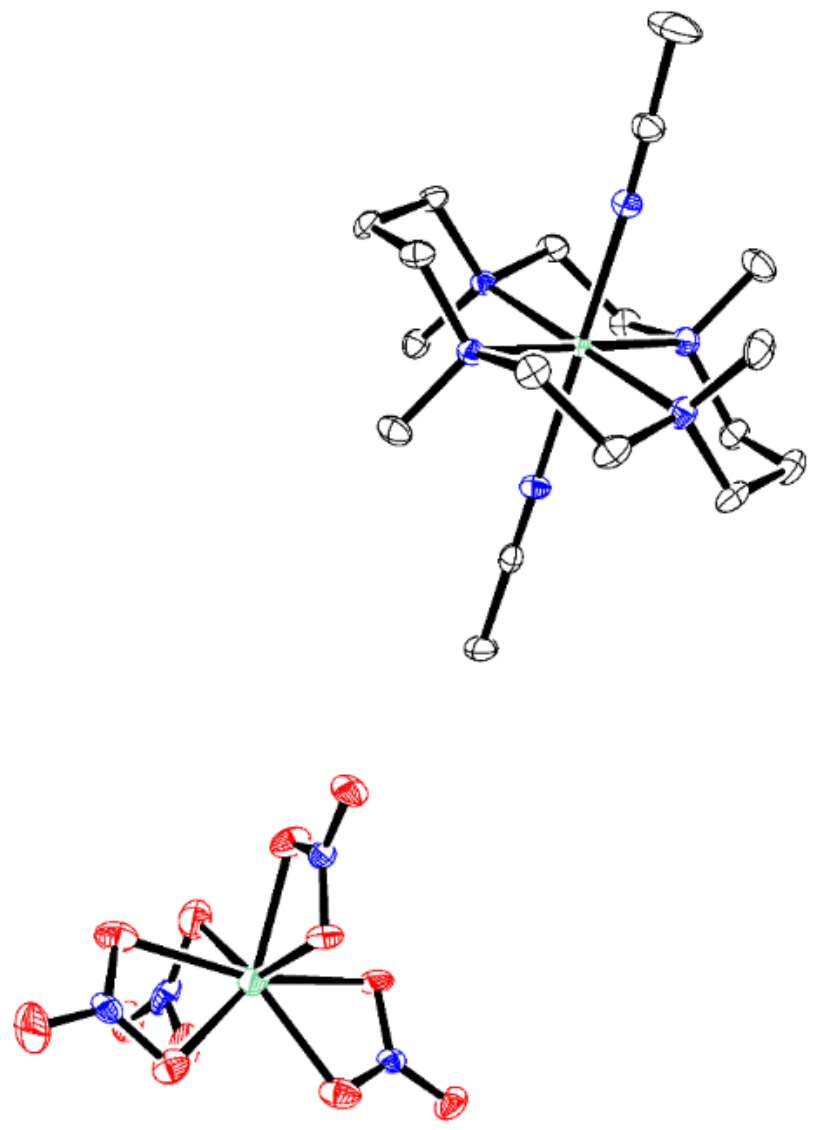

Figure S24. Molecular structure of **[(D$_{12}$-TMC)Co^{II}(CH$_3$CN)$_2$][Co(NO$_3$)$_4$].**

Table S126. Crystal data and structure refinement for **[(D$_{12}$-TMC)Co^{II}(CH$_3$CN)$_2$][Co(NO$_3$)$_4$]**.

CCDC No	2062722	
Empirical formula	C$_{18}$H$_{26}$Co$_2$D$_{12}$N$_{10}$O$_{12}$	
Formula weight	716.52	
Temperature	100(2) K	
Wavelength	0.71073 Å	
Crystal system	Monoclinic	
Space group	*P*2$_1$/*c*	
Unit cell dimensions	a = 10.1396(4) Å	α = 90°
	b = 15.3890(6) Å	β = 97.926(2)°
	c = 18.8077(8) Å	γ = 90°
Volume	2906.7(2) Å^3	
Z	4	
Density (calculated)	1.637 Mg/m^3	
Absorption coefficient	1.216 mm^{-1}	
F(000)	1464	
Crystal size	0.175 x 0.054 x 0.049 mm^3	
Theta range for data collection	2.187 to 28.280°.	
Index ranges	-13 ≤ h ≤ 13, 0 ≤ k ≤ 20, 0 ≤ l ≤ 25	
Reflections collected	6476	
Independent reflections	6476 [merged data]	
Completeness to theta = 25.242°	93.6 %	
Absorption correction	Semi-empirical from equivalents	
Refinement method	Full-matrix least-squares on *F*^{2}	
Data / restraints / parameters	6476 / 957 / 454	
Goodness-of-fit on *F*^{2}	1.045	
Final R indices [I>2σ(I)]	R1 = 0.0558, wR2 = 0.1077	
R indices (all data)	R1 = 0.0845, wR2 = 0.1187	
Largest diff. peak and hole	0.766 and -1.047 eÅ^{-3}	

Table S127. Atomic coordinates (x 10^4) and equivalent isotropic displacement parameters ($Å^2$x 10^3) for **[(D$_{12}$-TMC)Co^{II}(CH$_3$CN)$_2$][Co(NO$_3$)$_4$]**. U(eq) is defined as one third of the trace of the orthogonalized U^{ij} tensor.

	x	y	z	U(eq)
Co(1)	3006(1)	7579(1)	3555(1)	11(1)
N(1)	3580(3)	8400(2)	4484(2)	17(1)
N(2)	3860(3)	8445(2)	2841(2)	16(1)
N(3)	2416(3)	6770(2)	2622(2)	13(1)
N(4)	2140(3)	6720(2)	4268(2)	13(1)
C(1)	5015(4)	8653(3)	4516(2)	20(1)
C(2)	5328(4)	9176(3)	3869(2)	22(1)
C(3)	5240(4)	8700(3)	3146(3)	20(1)
C(4)	3110(4)	9251(3)	2604(2)	24(1)
D(4A)	3609	9585	2287	35
D(4B)	2991	9604	3025	35
D(4C)	2236	9095	2345	35
C(5)	3879(4)	7886(3)	2193(2)	16(1)
C(6)	2563(4)	7393(3)	2034(2)	17(1)
C(7)	3214(4)	5986(2)	2516(2)	16(1)
D(7A)	2841	5695	2070	24
D(7B)	3192	5588	2921	24
D(7C)	4137	6156	2487	24
C(8)	985(4)	6512(3)	2586(2)	16(1)
C(9)	673(4)	5985(2)	3235(2)	16(1)
C(10)	751(4)	6456(3)	3959(3)	18(1)
C(11)	2881(4)	5914(3)	4498(2)	19(1)
D(11A)	3042	5582	4074	28
D(11B)	2356	5562	4791	28
D(11C)	3735	6066	4782	28
C(12)	2114(4)	7284(3)	4914(2)	19(1)
C(13)	3431(4)	7784(3)	5070(2)	21(1)
C(14)	2796(4)	9195(3)	4583(3)	24(1)
D(14A)	2804	9579	4168	36
D(14B)	3190	9497	5020	36
D(14C)	1876	9033	4627	36
N(5)	4863(3)	6871(2)	3759(2)	15(1)
N(6)	1150(3)	8287(2)	3355(2)	17(1)
C(15)	5828(4)	6492(3)	3860(2)	14(1)
C(16)	7070(4)	6013(3)	3982(3)	22(1)
C(17)	150(4)	8629(3)	3274(3)	19(1)
C(18)	-1142(5)	9057(4)	3179(3)	41(1)
Co(21)	11984(1)	2957(1)	3947(1)	34(1)
O(24)	10853(3)	3891(2)	4519(2)	23(1)
O(25)	10266(3)	3588(2)	3411(2)	26(1)
O(26)	8999(3)	4423(2)	3970(2)	25(1)
O(27)	13755(3)	3538(2)	4483(2)	28(1)
O(28)	13105(3)	3960(2)	3408(2)	24(1)
O(29)	14987(3)	4434(2)	3966(2)	27(1)
O(21)	10510(20)	1864(13)	4140(6)	32(2)
O(22)	11998(11)	2214(9)	5029(10)	25(3)
O(23)	10690(30)	1120(14)	5143(13)	48(5)
N(21)	11020(20)	1745(11)	4788(7)	20(3)
O(21A)	10860(30)	2009(13)	4185(11)	31(3)
O(22A)	11963(19)	2082(13)	5257(14)	40(3)
O(23A)	10420(30)	1124(19)	5022(17)	52(5)
N(21A)	11150(30)	1680(20)	4807(12)	43(5)
N(22)	10008(3)	3983(2)	3971(2)	17(1)
N(23)	13984(3)	3994(2)	3952(2)	17(1)
O(30)	13253(17)	1991(7)	3709(6)	32(3)
O(31)	12069(14)	2231(11)	2687(13)	38(3)
O(32)	13610(20)	1242(14)	2781(10)	29(3)
N(24)	12970(60)	1790(40)	3053(12)	26(1)
O(30A)	13510(30)	1760(30)	3745(11)	68(7)
O(31A)	12080(30)	2335(15)	2920(17)	37(3)
O(32A)	13350(40)	1280(30)	2654(19)	36(6)
N(24A)	13010(100)	1800(60)	3089(19)	26(1)

Table S128. Bond lengths [Å] and angles [°] for **[(D$_{12}$-TMC)Co^{II}(CH$_3$CN)$_2$][Co(NO$_3$)$_4$]**.

Co(1)-N(4)	2.154(3)		
Co(1)-N(2)	2.157(3)	N(4)-Co(1)-N(2)	179.56(13)
Co(1)-N(6)	2.162(3)	N(4)-Co(1)-N(6)	89.88(12)
Co(1)-N(5)	2.164(3)	N(2)-Co(1)-N(6)	89.70(12)
Co(1)-N(3)	2.167(3)	N(4)-Co(1)-N(5)	90.01(12)
Co(1)-N(1)	2.170(3)	N(2)-Co(1)-N(5)	90.41(12)
N(1)-C(13)	1.477(5)	N(6)-Co(1)-N(5)	179.79(14)
N(1)-C(14)	1.485(5)	N(4)-Co(1)-N(3)	93.52(12)
N(1)-C(1)	1.499(5)	N(2)-Co(1)-N(3)	86.61(12)
N(2)-C(3)	1.489(5)	N(6)-Co(1)-N(3)	90.72(12)
N(2)-C(4)	1.491(5)	N(5)-Co(1)-N(3)	89.47(12)
N(2)-C(5)	1.493(5)	N(4)-Co(1)-N(1)	86.74(12)
N(3)-C(7)	1.481(5)	N(2)-Co(1)-N(1)	93.12(13)
N(3)-C(6)	1.487(5)	N(6)-Co(1)-N(1)	88.70(13)
N(3)-C(8)	1.497(5)	N(5)-Co(1)-N(1)	91.12(12)
N(4)-C(11)	1.484(5)	N(3)-Co(1)-N(1)	179.36(13)
N(4)-C(12)	1.495(5)	C(13)-N(1)-C(14)	108.8(3)
N(4)-C(10)	1.504(5)	C(13)-N(1)-C(1)	109.6(3)
C(1)-C(2)	1.528(6)	C(14)-N(1)-C(1)	108.3(3)
C(1)-H(1A)	0.9900	C(13)-N(1)-Co(1)	100.6(2)
C(1)-H(1AB)	0.9900	C(14)-N(1)-Co(1)	119.5(3)
C(2)-C(3)	1.536(6)	C(1)-N(1)-Co(1)	109.6(2)
C(2)-H(2A)	0.9900	C(3)-N(2)-C(4)	108.1(3)
C(2)-H(2AB)	0.9900	C(3)-N(2)-C(5)	110.4(3)
C(3)-H(3A)	0.9900	C(4)-N(2)-C(5)	107.4(3)
C(3)-H(3AB)	0.9900	C(3)-N(2)-Co(1)	111.0(3)
C(4)-D(4A)	0.9800	C(4)-N(2)-Co(1)	117.9(2)
C(4)-D(4B)	0.9800	C(5)-N(2)-Co(1)	101.7(2)
C(4)-D(4C)	0.9800	C(7)-N(3)-C(6)	108.2(3)
C(5)-C(6)	1.529(5)	C(7)-N(3)-C(8)	108.8(3)
C(5)-H(5A)	0.9900	C(6)-N(3)-C(8)	109.5(3)
C(5)-H(5AB)	0.9900	C(7)-N(3)-Co(1)	118.8(2)
C(6)-H(6A)	0.9900	C(6)-N(3)-Co(1)	100.8(2)
C(6)-H(6AB)	0.9900	C(8)-N(3)-Co(1)	110.2(2)
C(7)-D(7A)	0.9800	C(11)-N(4)-C(12)	108.2(3)
C(7)-D(7B)	0.9800	C(11)-N(4)-C(10)	107.2(3)
C(7)-D(7C)	0.9800	C(12)-N(4)-C(10)	110.5(3)
C(8)-C(9)	1.534(5)	C(11)-N(4)-Co(1)	117.5(2)
C(8)-H(8A)	0.9900	C(12)-N(4)-Co(1)	101.8(2)
C(8)-H(8AB)	0.9900	C(10)-N(4)-Co(1)	111.5(3)
C(9)-C(10)	1.536(6)	N(1)-C(1)-C(2)	114.3(3)
C(9)-H(9A)	0.9900	N(1)-C(1)-H(1A)	108.7
C(9)-H(9AB)	0.9900	C(2)-C(1)-H(1A)	108.7
C(10)-H(10A)	0.9900	N(1)-C(1)-H(1AB)	108.7
C(10)-H(10B)	0.9900	C(2)-C(1)-H(1AB)	108.7
C(11)-D(11A)	0.9800	H(1A)-C(1)-H(1AB)	107.6
C(11)-D(11B)	0.9800	C(1)-C(2)-C(3)	117.4(3)
C(11)-D(11C)	0.9800	C(1)-C(2)-H(2A)	108.0
C(12)-C(13)	1.535(6)	C(3)-C(2)-H(2A)	108.0
C(12)-H(12A)	0.9900	C(1)-C(2)-H(2AB)	108.0
C(12)-H(12B)	0.9900	C(3)-C(2)-H(2AB)	108.0
C(13)-H(13A)	0.9900	H(2A)-C(2)-H(2AB)	107.2
C(13)-H(13B)	0.9900	N(2)-C(3)-C(2)	113.8(3)
C(14)-D(14A)	0.9800	N(2)-C(3)-H(3A)	108.8
C(14)-D(14B)	0.9800	C(2)-C(3)-H(3A)	108.8
C(14)-D(14C)	0.9800	N(2)-C(3)-H(3AB)	108.8
N(5)-C(15)	1.132(5)	C(2)-C(3)-H(3AB)	108.8
N(6)-C(17)	1.133(5)	H(3A)-C(3)-H(3AB)	107.7
C(15)-C(16)	1.450(6)	N(2)-C(4)-D(4A)	109.5
C(16)-H(16A)	0.9800	N(2)-C(4)-D(4B)	109.5
C(16)-H(16B)	0.9800	D(4A)-C(4)-D(4B)	109.5
C(16)-H(16C)	0.9800	N(2)-C(4)-D(4C)	109.5
C(17)-C(18)	1.456(6)	D(4A)-C(4)-D(4C)	109.5
C(18)-H(18A)	0.9800	D(4B)-C(4)-D(4C)	109.5
C(18)-H(18B)	0.9800	N(2)-C(5)-C(6)	109.7(3)
C(18)-H(18C)	0.9800	N(2)-C(5)-H(5A)	109.7
Co(21)-O(21A)	1.94(2)	C(6)-C(5)-H(5A)	109.7
Co(21)-O(30)	2.056(15)	N(2)-C(5)-H(5AB)	109.7
Co(21)-O(25)	2.124(3)	C(6)-C(5)-H(5AB)	109.7
Co(21)-O(27)	2.131(3)	H(5A)-C(5)-H(5AB)	108.2
Co(21)-O(31A)	2.17(3)	N(3)-C(6)-C(5)	110.4(3)
Co(21)-O(24)	2.208(3)	N(3)-C(6)-H(6A)	109.6
Co(21)-O(28)	2.240(3)	C(5)-C(6)-H(6A)	109.6
Co(21)-O(21)	2.31(2)	N(3)-C(6)-H(6AB)	109.6
Co(21)-O(22)	2.33(2)	C(5)-C(6)-H(6AB)	109.6
O(24)-N(22)	1.253(4)	H(6A)-C(6)-H(6AB)	108.1
O(25)-N(22)	1.275(4)	N(3)-C(7)-D(7A)	109.5
O(26)-N(22)	1.227(4)	N(3)-C(7)-D(7B)	109.5
O(27)-N(23)	1.267(4)	D(7A)-C(7)-D(7B)	109.5
O(28)-N(23)	1.263(4)	N(3)-C(7)-D(7C)	109.5
O(29)-N(23)	1.219(4)	D(7A)-C(7)-D(7C)	109.5
O(21)-N(21)	1.268(9)	D(7B)-C(7)-D(7C)	109.5
O(22)-N(21)	1.263(10)	N(3)-C(8)-C(9)	114.1(3)
O(23)-N(21)	1.240(10)	N(3)-C(8)-H(8A)	108.7
O(21A)-N(21A)	1.273(13)	C(9)-C(8)-H(8A)	108.7
O(22A)-N(21A)	1.260(13)	N(3)-C(8)-H(8AB)	108.7
O(23A)-N(21A)	1.236(14)	C(9)-C(8)-H(8AB)	108.7
O(30)-N(24)	1.268(10)	H(8A)-C(8)-H(8AB)	107.6
O(31)-N(24)	1.262(9)	C(8)-C(9)-C(10)	117.7(3)
O(32)-N(24)	1.219(10)	C(8)-C(9)-H(9A)	107.9
O(30A)-N(24A)	1.267(13)	C(10)-C(9)-H(9A)	107.9
O(31A)-N(24A)	1.265(13)	C(8)-C(9)-H(9AB)	107.9
O(32A)-N(24A)	1.226(14)	C(10)-C(9)-H(9AB)	107.9

H(9A)-C(9)-H(9AB)	107.2	O(21A)-Co(21)-O(31A)	88.4(10)
N(4)-C(10)-C(9)	113.6(3)	O(25)-Co(21)-O(31A)	84.9(6)
N(4)-C(10)-H(10A)	108.8	O(27)-Co(21)-O(31A)	118.0(7)
C(9)-C(10)-H(10A)	108.8	O(21A)-Co(21)-O(24)	90.9(7)
N(4)-C(10)-H(10B)	108.8	O(30)-Co(21)-O(24)	163.6(4)
C(9)-C(10)-H(10B)	108.8	O(25)-Co(21)-O(24)	59.14(11)
H(10A)-C(10)-H(10B)	107.7	O(27)-Co(21)-O(24)	87.55(12)
N(4)-C(11)-D(11A)	109.5	O(31A)-Co(21)-O(24)	144.1(6)
N(4)-C(11)-D(11B)	109.5	O(21A)-Co(21)-O(28)	166.5(6)
D(11A)-C(11)-D(11B)	109.5	O(30)-Co(21)-O(28)	91.6(3)
N(4)-C(11)-D(11C)	109.5	O(25)-Co(21)-O(28)	84.52(12)
D(11A)-C(11)-D(11C)	109.5	O(27)-Co(21)-O(28)	58.56(11)
D(11B)-C(11)-D(11C)	109.5	O(31A)-Co(21)-O(28)	79.3(8)
N(4)-C(12)-C(13)	109.5(3)	O(24)-Co(21)-O(28)	95.88(11)
N(4)-C(12)-H(12A)	109.8	O(30)-Co(21)-O(21)	86.8(5)
C(13)-C(12)-H(12A)	109.8	O(25)-Co(21)-O(21)	84.3(4)
N(4)-C(12)-H(12B)	109.8	O(27)-Co(21)-O(21)	138.7(3)
C(13)-C(12)-H(12B)	109.8	O(24)-Co(21)-O(21)	90.4(4)
H(12A)-C(12)-H(12B)	108.2	O(28)-Co(21)-O(21)	162.1(3)
N(1)-C(13)-C(12)	110.8(3)	O(30)-Co(21)-O(22)	84.6(4)
N(1)-C(13)-H(13A)	109.5	O(25)-Co(21)-O(22)	123.0(3)
C(12)-C(13)-H(13A)	109.5	O(27)-Co(21)-O(22)	83.6(2)
N(1)-C(13)-H(13B)	109.5	O(24)-Co(21)-O(22)	80.5(3)
C(12)-C(13)-H(13B)	109.5	O(28)-Co(21)-O(22)	142.2(2)
H(13A)-C(13)-H(13B)	108.1	O(21)-Co(21)-O(22)	55.4(3)
N(1)-C(14)-D(14A)	109.5	N(22)-O(24)-Co(21)	90.9(2)
N(1)-C(14)-D(14B)	109.5	N(22)-O(25)-Co(21)	94.2(2)
D(14A)-C(14)-D(14B)	109.5	N(23)-O(27)-Co(21)	95.4(2)
N(1)-C(14)-D(14C)	109.5	N(23)-O(28)-Co(21)	90.4(2)
D(14A)-C(14)-D(14C)	109.5	N(21)-O(21)-Co(21)	94.2(12)
D(14B)-C(14)-D(14C)	109.5	N(21)-O(22)-Co(21)	93.3(10)
C(15)-N(5)-Co(1)	179.1(4)	O(23)-N(21)-O(22)	120.2(11)
C(17)-N(6)-Co(1)	176.4(4)	O(23)-N(21)-O(21)	121.9(11)
N(5)-C(15)-C(16)	179.3(5)	O(22)-N(21)-O(21)	117.1(9)
C(15)-C(16)-H(16A)	109.5	N(21A)-O(21A)-Co(21)	116.3(16)
C(15)-C(16)-H(16B)	109.5	O(23A)-N(21A)-O(22A)	119.2(19)
H(16A)-C(16)-H(16B)	109.5	O(23A)-N(21A)-O(21A)	120.2(19)
C(15)-C(16)-H(16C)	109.5	O(22A)-N(21A)-O(21A)	117.8(15)
H(16A)-C(16)-H(16C)	109.5	O(26)-N(22)-O(24)	122.6(4)
H(16B)-C(16)-H(16C)	109.5	O(26)-N(22)-O(25)	121.9(3)
N(6)-C(17)-C(18)	178.9(5)	O(24)-N(22)-O(25)	115.6(3)
C(17)-C(18)-H(18A)	109.5	O(29)-N(23)-O(28)	122.2(4)
C(17)-C(18)-H(18B)	109.5	O(29)-N(23)-O(27)	122.3(4)
H(18A)-C(18)-H(18B)	109.5	O(28)-N(23)-O(27)	115.5(3)
C(17)-C(18)-H(18C)	109.5	N(24)-O(30)-Co(21)	108.9(12)
H(18A)-C(18)-H(18C)	109.5	O(32)-N(24)-O(31)	122.2(12)
H(18B)-C(18)-H(18C)	109.5	O(32)-N(24)-O(30)	120.9(12)
O(21A)-Co(21)-O(25)	89.1(7)	O(31)-N(24)-O(30)	116.6(11)
O(30)-Co(21)-O(25)	136.4(4)	N(24A)-O(31A)-Co(21)	100.8(19)
O(21A)-Co(21)-O(27)	133.6(7)	O(32A)-N(24A)-O(31A)	121.6(18)
O(30)-Co(21)-O(27)	83.9(4)	O(32A)-N(24A)-O(30A)	121(2)
O(25)-Co(21)-O(27)	128.03(12)	O(31A)-N(24A)-O(30A)	117.5(17)

Table S129. Anisotropic displacement parameters (Å^{2}x 10^3) for **[(D$_{12}$-TMC)Co^{II}(CH$_3$CN)$_2$][Co(NO$_3$)$_4$]**.
The anisotropic displacement factor exponent takes the form: -2p^2[h^2 a*^{2}U^{11} + ... + 2 h k a* b* U^{12}]

	U^{11}	U^{22}	U^{33}	U^{23}	U^{13}	U^{12}
Co(1)	9(1)	13(1)	11(1)	1(1)	1(1)	1(1)
N(1)	15(2)	18(2)	18(2)	-1(1)	5(1)	-3(1)
N(2)	15(2)	17(2)	18(2)	3(1)	3(1)	-1(1)
N(3)	13(1)	15(1)	11(2)	2(1)	0(1)	1(1)
N(4)	12(1)	14(1)	14(2)	1(1)	2(1)	2(1)
C(1)	15(2)	25(2)	20(2)	-7(2)	0(2)	-6(2)
C(2)	18(2)	21(2)	27(2)	-4(2)	4(2)	-7(2)
C(3)	15(2)	19(2)	25(2)	1(2)	4(2)	-3(2)
C(4)	26(2)	20(2)	25(2)	10(2)	4(2)	5(2)
C(5)	19(2)	21(2)	10(2)	2(2)	4(2)	0(2)
C(6)	18(2)	23(2)	11(2)	6(2)	0(2)	-1(2)
C(7)	16(2)	16(2)	17(2)	-2(2)	4(2)	-1(2)
C(8)	14(2)	22(2)	12(2)	0(2)	-1(2)	-2(2)
C(9)	12(2)	17(2)	19(2)	-1(2)	1(2)	-5(2)
C(10)	14(2)	24(2)	16(2)	2(2)	2(2)	1(2)
C(11)	19(2)	21(2)	16(2)	7(2)	4(2)	4(2)
C(12)	21(2)	23(2)	14(2)	-2(2)	5(2)	0(2)
C(13)	21(2)	29(2)	12(2)	-3(2)	1(2)	-5(2)
C(14)	23(2)	19(2)	32(3)	-10(2)	8(2)	-2(2)
N(5)	14(1)	19(2)	12(2)	2(1)	1(1)	1(1)
N(6)	15(2)	17(2)	20(2)	0(1)	1(1)	2(1)
C(15)	17(2)	13(2)	13(2)	0(2)	2(2)	-4(2)
C(16)	13(2)	22(2)	27(2)	-2(2)	-2(2)	4(2)
C(17)	17(2)	20(2)	21(2)	5(2)	3(2)	1(2)
C(18)	22(2)	48(3)	51(3)	13(3)	4(2)	18(2)
Co(21)	14(1)	23(1)	64(1)	-5(1)	-2(1)	4(1)
O(24)	17(1)	31(2)	17(2)	0(1)	-4(1)	1(1)
O(25)	18(1)	36(2)	24(2)	-12(1)	1(1)	2(1)
O(26)	17(1)	26(2)	33(2)	3(1)	7(1)	9(1)
O(27)	21(2)	40(2)	23(2)	10(1)	4(1)	3(1)
O(28)	22(2)	30(2)	17(2)	-1(1)	-7(1)	0(1)
O(29)	15(1)	27(2)	38(2)	-7(2)	3(1)	-3(1)
O(21)	33(6)	41(6)	20(4)	4(3)	-5(3)	4(4)
O(22)	19(3)	33(4)	25(5)	1(4)	8(4)	-4(3)
O(23)	48(10)	39(5)	60(7)	25(5)	13(6)	-14(6)
N(21)	17(5)	19(4)	26(4)	3(3)	6(3)	7(4)
O(21A)	27(7)	28(5)	40(6)	-2(4)	6(5)	-4(4)
O(22A)	33(5)	53(7)	36(7)	31(7)	15(6)	12(4)
O(23A)	35(9)	58(9)	67(13)	15(8)	29(9)	6(6)
N(21A)	25(7)	56(10)	51(7)	18(6)	20(5)	19(6)
N(22)	13(2)	18(2)	19(2)	1(1)	3(1)	-2(1)
N(23)	13(2)	18(2)	20(2)	-4(1)	1(1)	5(1)
O(30)	49(5)	26(4)	25(3)	-8(3)	20(3)	-11(3)
O(31)	23(3)	26(5)	66(8)	-5(5)	7(5)	1(3)
O(32)	29(8)	24(4)	37(6)	-9(4)	15(5)	0(4)
N(24)	17(4)	26(2)	36(3)	-14(3)	11(3)	-7(3)
O(30A)	50(9)	115(18)	37(5)	-19(8)	-2(6)	17(11)
O(31A)	29(5)	29(6)	53(8)	-18(6)	8(6)	-2(4)
O(32A)	29(13)	34(8)	46(10)	-21(8)	12(8)	6(7)
N(24A)	17(4)	26(2)	36(3)	-14(3)	11(3)	-7(3)

Table S130. Hydrogen coordinates (x 10^4) and isotropic displacement parameters ($Å^2$x 10^3) for **[(D$_{12}$-TMC)Co^{II}(CH$_3$CN)$_2$][Co(NO$_3$)$_4$]**.

	x	y	z	U(eq)
H(1A)	5564	8119	4555	24
H(1AB)	5276	9000	4956	24
H(2A)	6241	9412	3985	26
H(2AB)	4712	9678	3806	26
H(3A)	5610	9080	2798	24
H(3AB)	5799	8171	3211	24
H(5A)	4627	7469	2278	20
H(5AB)	4012	8252	1776	20
H(6A)	1813	7810	1983	21
H(6AB)	2537	7073	1575	21
H(8A)	729	6165	2146	19
H(8AB)	431	7044	2546	19
H(9A)	1295	5486	3297	19
H(9AB)	-236	5744	3117	19
H(10A)	183	6981	3897	21
H(10B)	386	6070	4306	21
H(12A)	1985	6920	5333	23
H(12B)	1362	7698	4827	23
H(13A)	3454	8107	5527	25
H(13B)	4184	7369	5124	25
H(16A)	7453	5974	3532	32
H(16B)	6902	5427	4153	32
H(16C)	7695	6315	4343	32
H(18A)	-1776	8710	3408	61
H(18B)	-1464	9115	2665	61
H(18C)	-1057	9635	3399	61

[(cleav.-D_{12}-TMC)(Co^{II})$_2$(I)$_4$]

The structure of [(cleav.-D_{12}-TMC)(Co^{II})$_2$(I)$_4$] was solved in the monoclinic space group *P*2$_1$/*c*. The asymmetric unit contains half a molecule of [(cleav.-D_{12}-TMC)(Co^{II})$_2$(I)$_4$]. Due to the lack of further investigations, the protium to deuterium content in both N-dimethyl positions are unknown. For solving the structure, it makes no difference if protium(H) is exchanged with deuterium(D). Therefore the sum formula was simplified as if it was in full protium content, given as "C_{14} H_{34} Co_2 I_4 N_4".

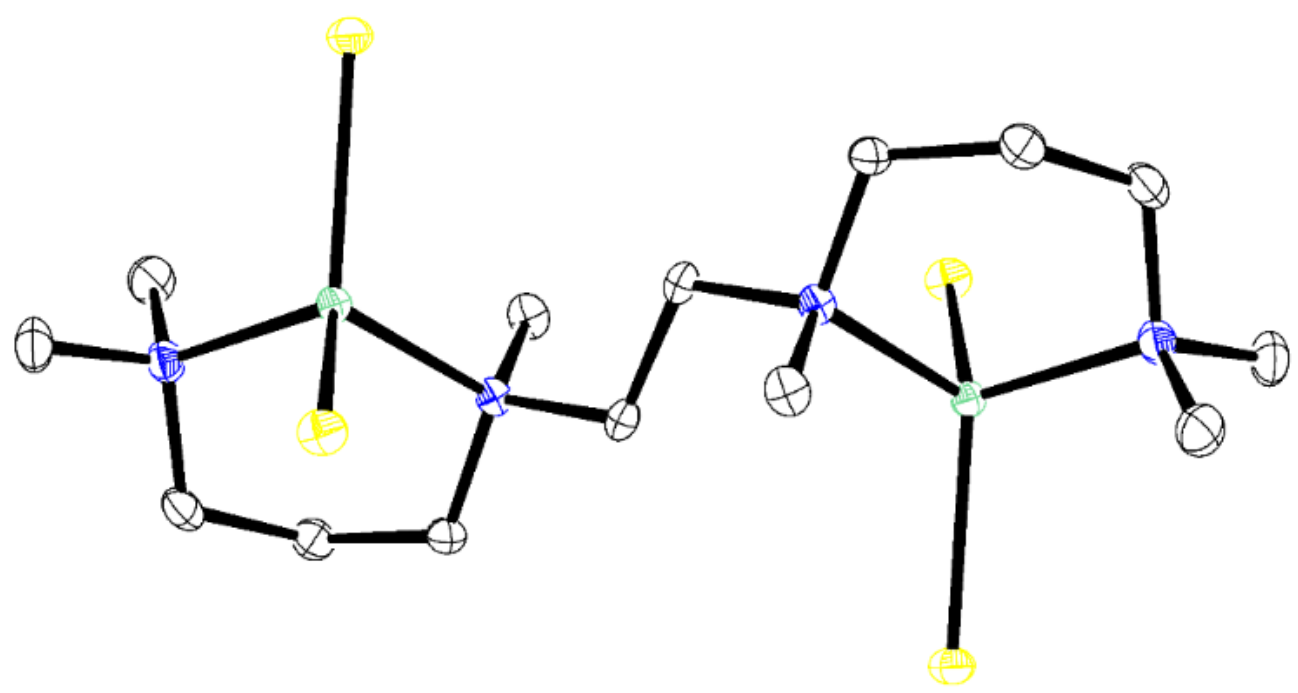

Figure S25. Molecular structure of **[(cleav.-D_{12}-TMC)(Co^{II})$_2$(I)$_4$]**.

Table S131. Crystal data and structure refinement for [(cleav.-D_{12}-TMC)(Co^{II})$_2$(I)$_4$].

CCDC No	2062723	
Empirical formula	$C_{14}H_{34}Co_2I_4N_4$	
Formula weight	883.91	
Temperature	100(2) K	
Wavelength	0.71073 Å	
Crystal system	Monoclinic	
Space group	*P*2$_1$/*c*	
Unit cell dimensions	a = 8.0217(2) Å	α = 90°
	b = 12.7337(3) Å	β = 98.9067(12)°
	c = 12.8262(3) Å	γ = 90°
Volume	1294.35(5) Å^3	
Z	2	
Density (calculated)	2.268 Mg/m^3	
Absorption coefficient	6.057 mm^{-1}	
F(000)	824	
Crystal size	0.058 x 0.050 x 0.028 mm^3	
Theta range for data collection	2.267 to 28.693°.	
Index ranges	-10 ≤ h ≤ 10, -17 ≤ k ≤ 17, -17 ≤ l ≤ 17	
Reflections collected	62947	
Independent reflections	3343 [R(int) = 0.0607]	
Completeness to theta = 25.242°	100.0 %	
Absorption correction	Semi-empirical from equivalents	
Refinement method	Full-matrix least-squares on F^2	
Data / restraints / parameters	3343 / 0 / 112	
Goodness-of-fit on F^2	1.064	
Final R indices [I>2σ(I)]	R1 = 0.0208, wR2 = 0.0373	
R indices (all data)	R1 = 0.0288, wR2 = 0.0394	
Largest diff. peak and hole	0.688 and -0.708 eÅ^{-3}	

Table S132. Atomic coordinates (x 10^4) and equivalent isotropic displacement parameters ($Å^2$x 10^3) for [(cleav.-D_{12}-TMC)(Co^{II})$_2$(I)$_4$]. U(eq) is defined as one third of the trace of the orthogonalized U^{ij} tensor.

	x	y	z	U(eq)
I(1)	6656(1)	5523(1)	8194(1)	17(1)
I(2)	2291(1)	7000(1)	6297(1)	18(1)
Co(1)	3718(1)	5302(1)	7108(1)	13(1)
N(1)	2176(3)	4594(2)	8032(2)	17(1)
N(2)	3670(3)	4248(2)	5876(2)	13(1)
C(1)	2995(4)	3730(2)	8697(2)	23(1)
C(2)	1611(4)	5389(2)	8748(2)	24(1)
C(3)	661(3)	4191(2)	7313(2)	19(1)
C(4)	1062(3)	3416(2)	6477(2)	18(1)
C(5)	1886(3)	3872(2)	5580(2)	17(1)
C(6)	4126(3)	4751(2)	4903(2)	15(1)
C(7)	4816(3)	3339(2)	6175(2)	17(1)

Table S133. Bond lengths [Å] and angles [°] for [(cleav.-D_{12}-TMC)(Co^{II})$_2$(I)$_4$].

I(1)-Co(1)	2.5605(4)	C(7)-N(2)-Co(1)	111.67(16)
I(2)-Co(1)	2.5884(4)	C(6)-N(2)-Co(1)	112.39(15)
Co(1)-N(1)	2.049(2)	C(5)-N(2)-Co(1)	107.67(15)
Co(1)-N(2)	2.070(2)	N(1)-C(1)-H(1A)	109.5
N(1)-C(1)	1.481(3)	N(1)-C(1)-H(1B)	109.5
N(1)-C(2)	1.484(3)	H(1A)-C(1)-H(1B)	109.5
N(1)-C(3)	1.499(3)	N(1)-C(1)-H(1C)	109.5
N(2)-C(7)	1.491(3)	H(1A)-C(1)-H(1C)	109.5
N(2)-C(6)	1.497(3)	H(1B)-C(1)-H(1C)	109.5
N(2)-C(5)	1.501(3)	N(1)-C(2)-H(2A)	109.5
C(1)-H(1A)	0.9800	N(1)-C(2)-H(2B)	109.5
C(1)-H(1B)	0.9800	H(2A)-C(2)-H(2B)	109.5
C(1)-H(1C)	0.9800	N(1)-C(2)-H(2C)	109.5
C(2)-H(2A)	0.9800	H(2A)-C(2)-H(2C)	109.5
C(2)-H(2B)	0.9800	H(2B)-C(2)-H(2C)	109.5
C(2)-H(2C)	0.9800	N(1)-C(3)-C(4)	114.5(2)
C(3)-C(4)	1.527(4)	N(1)-C(3)-H(3A)	108.6
C(3)-H(3A)	0.9900	C(4)-C(3)-H(3A)	108.6
C(3)-H(3AB)	0.9900	N(1)-C(3)-H(3AB)	108.6
C(4)-C(5)	1.527(4)	C(4)-C(3)-H(3AB)	108.6
C(4)-H(4A)	0.9900	H(3A)-C(3)-H(3AB)	107.6
C(4)-H(4AB)	0.9900	C(3)-C(4)-C(5)	116.5(2)
C(5)-H(5A)	0.9900	C(3)-C(4)-H(4A)	108.2
C(5)-H(5AB)	0.9900	C(5)-C(4)-H(4A)	108.2
C(6)-C(6)#1	1.524(5)	C(3)-C(4)-H(4AB)	108.2
C(6)-H(6A)	0.9900	C(5)-C(4)-H(4AB)	108.2
C(6)-H(6AB)	0.9900	H(4A)-C(4)-H(4AB)	107.3
C(7)-H(7A)	0.9800	N(2)-C(5)-C(4)	116.0(2)
C(7)-H(7B)	0.9800	N(2)-C(5)-H(5A)	108.3
C(7)-H(7C)	0.9800	C(4)-C(5)-H(5A)	108.3
		N(2)-C(5)-H(5AB)	108.3
N(1)-Co(1)-N(2)	102.44(9)	C(4)-C(5)-H(5AB)	108.3
N(1)-Co(1)-I(1)	108.72(6)	H(5A)-C(5)-H(5AB)	107.4
N(2)-Co(1)-I(1)	113.16(6)	N(2)-C(6)-C(6)#1	112.3(3)
N(1)-Co(1)-I(2)	109.34(6)	N(2)-C(6)-H(6A)	109.1
N(2)-Co(1)-I(2)	106.26(6)	C(6)#1-C(6)-H(6A)	109.1
I(1)-Co(1)-I(2)	116.031(14)	N(2)-C(6)-H(6AB)	109.1
C(1)-N(1)-C(2)	107.5(2)	C(6)#1-C(6)-H(6AB)	109.1
C(1)-N(1)-C(3)	110.2(2)	H(6A)-C(6)-H(6AB)	107.9
C(2)-N(1)-C(3)	108.5(2)	N(2)-C(7)-H(7A)	109.5
C(1)-N(1)-Co(1)	113.95(17)	N(2)-C(7)-H(7B)	109.5
C(2)-N(1)-Co(1)	109.11(17)	H(7A)-C(7)-H(7B)	109.5
C(3)-N(1)-Co(1)	107.41(16)	N(2)-C(7)-H(7C)	109.5
C(7)-N(2)-C(6)	109.09(19)	H(7A)-C(7)-H(7C)	109.5
C(7)-N(2)-C(5)	110.0(2)	H(7B)-C(7)-H(7C)	109.5
C(6)-N(2)-C(5)	105.81(19)		

Symmetry transformations used to generate equivalent atoms: #1 -x+1,-y+1,-z+1

Table S134. Anisotropic displacement parameters ($Å^2$x 10^3) for [(cleav.-D_{12}-TMC)(Co^{II})$_2$(I)$_4$]. The anisotropic displacement factor exponent takes the form: $-2p^2[h^2 a^{*2}U^{11} + ... + 2 h k a^* b^* U^{12}]$

	U^{11}	U^{22}	U^{33}	U^{23}	U^{13}	U^{12}
I(1)	15(1)	18(1)	18(1)	-3(1)	-1(1)	1(1)
I(2)	19(1)	14(1)	21(1)	0(1)	1(1)	4(1)
Co(1)	12(1)	13(1)	13(1)	-1(1)	2(1)	1(1)
N(1)	16(1)	21(1)	16(1)	2(1)	5(1)	1(1)
N(2)	13(1)	11(1)	14(1)	2(1)	3(1)	1(1)
C(1)	24(1)	26(2)	20(1)	7(1)	4(1)	2(1)
C(2)	25(2)	31(2)	19(1)	-1(1)	10(1)	3(1)
C(3)	13(1)	22(1)	22(1)	3(1)	6(1)	0(1)
C(4)	17(1)	18(1)	20(1)	0(1)	4(1)	-2(1)
C(5)	13(1)	21(1)	16(1)	-4(1)	2(1)	-2(1)
C(6)	18(1)	16(1)	10(1)	1(1)	3(1)	1(1)
C(7)	19(1)	12(1)	21(1)	2(1)	5(1)	3(1)

Table S135. Hydrogen coordinates (x 10^4) and isotropic displacement parameters ($Å^2$x 10^3) for [(cleav.-D_{12}-TMC)(Co^{II})$_2$(I)$_4$].

	x	y	z	U(eq)
H(1A)	3430	3207	8248	35
H(1B)	2168	3400	9080	35
H(1C)	3929	4013	9202	35
H(2A)	1019	5958	8329	36
H(2B)	2593	5675	9211	36
H(2C)	846	5060	9178	36
H(3A)	44	4796	6953	23
H(3AB)	-100	3844	7745	23
H(4A)	-2	3067	6165	22
H(4AB)	1817	2867	6835	22
H(5A)	1864	3328	5026	20
H(5AB)	1189	4468	5267	20
H(6A)	3280	5298	4653	17
H(6AB)	4089	4215	4341	17
H(7A)	4828	2885	5559	26
H(7B)	4412	2938	6739	26
H(7C)	5960	3595	6424	26

Powder X-ray diffraction

Powder X-ray diffraction data for **cobalt triflate** was collected on a STOE STADI P equipped with a Dectris Mythen 1K detector. Cu-Kα radiation with wavelength 1.54178 Å and a Ge-monochromator were used. Rietveld refinement in the trigonal space group $R\bar{3}$ against the experimental data was performed using TOPAS Academic.[12]

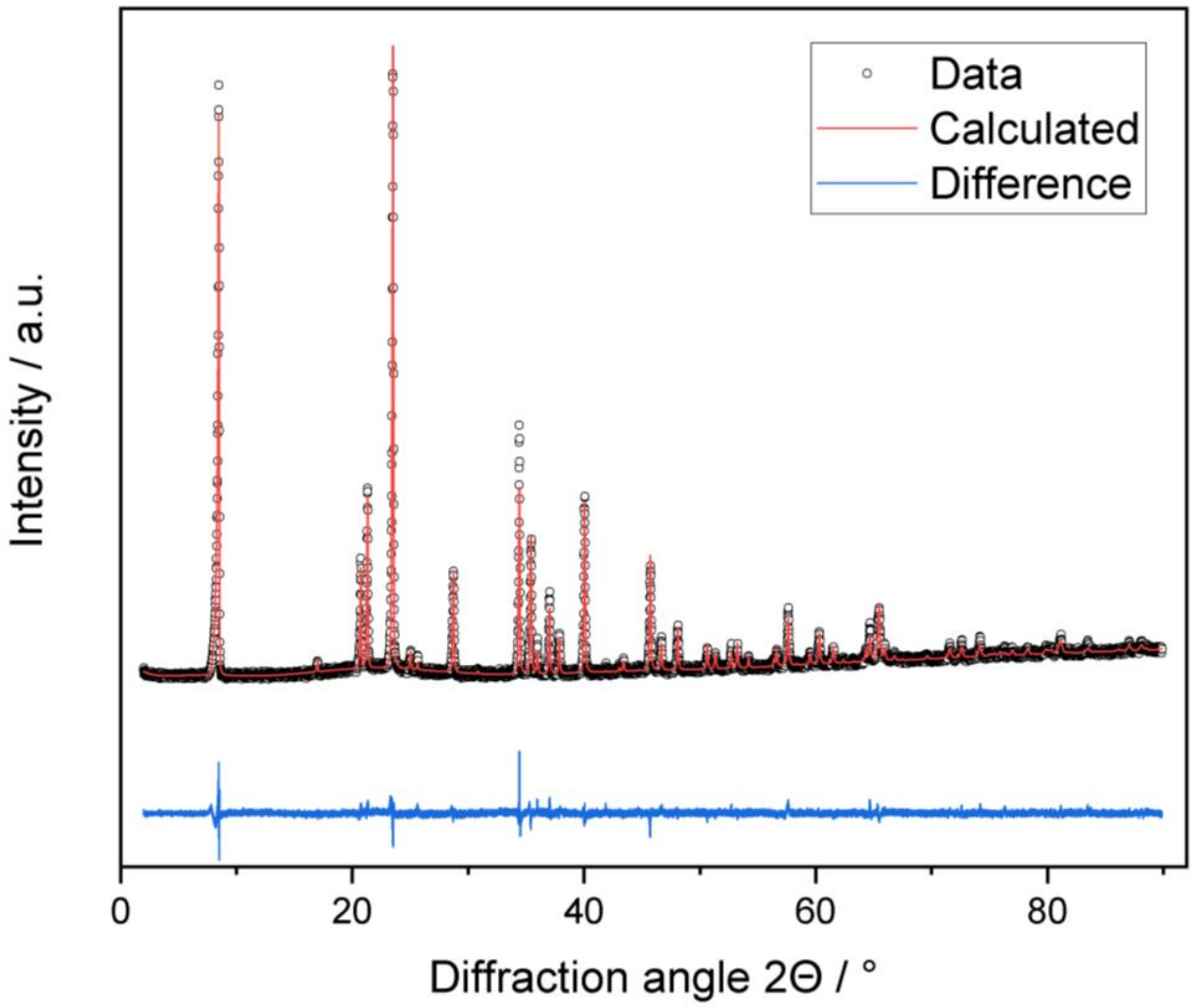

Figure S26. X-ray diffraction data for cobalt triflate with calculated diffraction data from Rietveld refinement and difference plot.

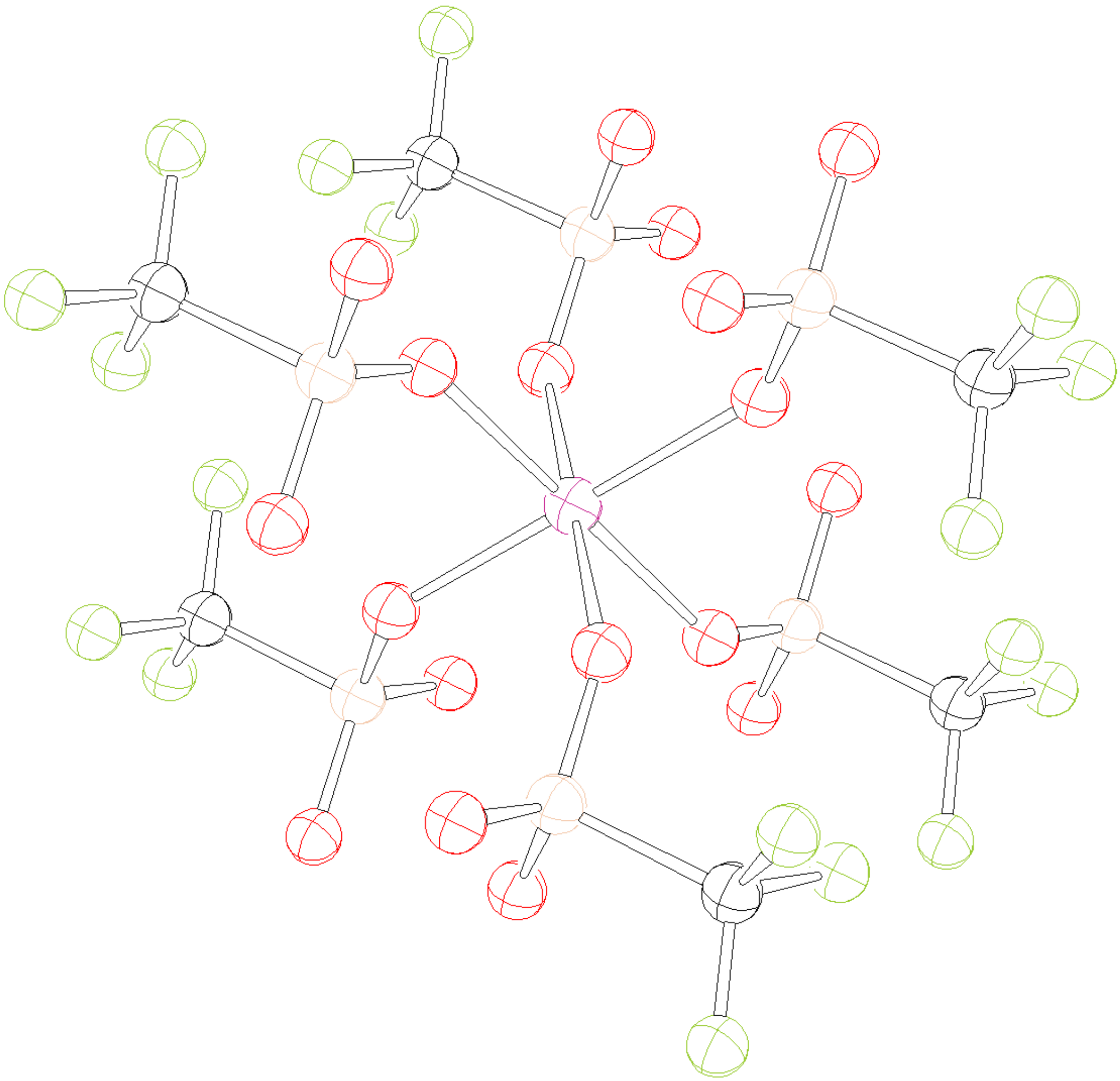

Figure S27. Excerpt of the crystal structure of cobalt triflate: Octahedral coordination of cobalt by triflate anions.

Table S136. Crystal data and refinement for **cobalt triflate**.

CSD No	2062767	
Empirical formula	$C_2CoF_6O_6S_2$	
Formula weight	357.07	
Wavelength	1.54178 Å	
Crystal system	Trigonal	
Space group	$R\overline{3}$	
Unit cell dimensions	a = 4.98728(19) Å	α= 90°
	b = 4.98728(19) Å	β= 90°
	c = 31.2328(11) Å	γ = 120°
Volume	672.77(6) Å^3	
Z	3	
Density (calculated)	2.643969 Mg/m^3	
Goodness-of-fit	2.39132213	
R indices	R_{wp} = 0.0169335708, R_{exp} = 0.00708125879	

Table S137. Atomic coordinates (x 10^4) and equivalent isotropic displacement parameters (Å^{2}x 10^2) for **cobalt triflate**.

	x	y	z	B(iso)
Co(1)	0	0	0	8(4)
S(2)	0	0	2821.3(8)	8(4)
O(3)	-514(5)	-3021(4)	2923.3(8)	8(4)
C(2)	0	0	2247(2)	8(4)
F(7)	139(5)	2544(4)	2085.7(8)	8(4)

Table S138. Bond lengths [Å] and angles [°] for **cobalt triflate**.

Co(1)-O(3)	2.090(2)	O(3)-S(2)-O(3)	115.20(10)
S(2)-O(3)	1.432(2)	C(6)-S(2)-O(3)	102.85(14)
S(2)-C(6)	1.793(8)	Co(1)-O(3)-S(2)	137.77(18)
C(6)-F(7)	1.334(4)	F(7)-C(6)-F(7)	106.6(3)
O(3)-Co(1)-O(3)	86.36(9)	S(2)-C(6)-F(7)	112.2(3)

References

[1] L. Krause, R. Herbst-Irmer, G. M. Sheldrick, D. Stalke, *J. Appl. Cryst.* **2015**, 48, 3–10

[2] G. M. Sheldrick, *Acta Cryst.* **2015**, A71, 3–8

[3] G. M. Sheldrick, *Acta Cryst.* **2015**, C71, 3–8

[4] https://www.ccdc.cam.ac.uk/structures/

[5] P. Müller, *Cryst. Rev.* **2009**, 15(1), 57

[6] A. Thorn, B. Dittrich, G. M. Sheldrick, *Acta Cryst.* **2012**, A68, 448

[7] A. L. Spek, *Acta Cryst.* **2015**, C71, 9-18.

[8] A. L. Spek, *J. Appl. Cryst.* **2003**, 36, 7-11

[9] A. L. Spek, *Acta Cryst.* **2009**, D65, 148-155

[10] A. L. Spek, *Inorg. Chim. Acta* **2018**, 470, 232-237

[11] A. L. Spek, *Acta Cryst.* **2020**, E76, 1-11

[12] A. A. Coelho , *J. Appl. Cryst.* **2018**, 51, 210-218
